# Genome-wide analysis of miRNAs and their target genes in wheat cultivars with different ploidy levels under drought stress

**DOI:** 10.1007/s00425-025-04757-3

**Published:** 2025-07-01

**Authors:** Ferhat Ulu, Necdet Mehmet Unel, Mehmet Cengiz Baloglu

**Affiliations:** 1 Plantomics Research Laboratory, Department of Genetics and Bioengineering, Faculty of Engineering and Architecture, Kastamonu University, Kastamonu, Turkey; 2 Research and Application Center, Kastamonu University, Kastamonu, Turkey; 3 SUNUM Nanotechnology Research Centre, Sabanci University, 34956 Istanbul, Turkey

**Keywords:** Aridity, Bread wheat, Durum wheat, Einkorn wheat, Micro-RNA, Mirnome, Ploidy, Stress response, Target genes, *Triticum aestivum*, *Triticum monococcum*, *Triticum turgidum*

## Abstract

**Main conclusion:**

This study provides novel insight into the role of miRNAs in the drought resistance of different wheat cultivars, revealing a correlation between ploidy level and drought tolerance.

**Abstract:**

MicroRNAs (miRNAs) are endogenous, mostly conserved, non-coding regulatory RNAs with 20–24 nt in length. Although many studies have been conducted on miRNAs that play a role in wheat drought stress response, there are no comparative studies in wheat cultivars with different ploidy levels. Here we compared miRNAs profiles of three wheat cultivars with different chromosome numbers and drought resistance levels using miRNAome and qRT-PCR analysis. Bioinformatics analysis showed that all cultivars shared 93 miRNAs in the control leaf, while 91 miRNAs were shared in stress-treated leaf groups. A total of 90 and 92 miRNAs were expressed by all cultivars in control and stress root samples, respectively. Also, 17 and 21 miRNAs were expressed species-specifically in control and stress leaf, whereas 23 and 20 were expressed in control and stress root groups, respectively. Also, tae-miR159a and tae-miR167c expressions showed drought resistance increases as the ploidy level rises, and *Triticum aestivum* and *Triticum turgidum* are more tolerant than *Triticum monococcum*. Furthermore, according to in silico analysis 729 and 771 genes were targeted in control-leaf and stress-leaf groups of all cultivars; also, 775 and 776 genes were targeted in control-root and stress-root samples by determined miRNAs, respectively. Additionally, degradome data showed 351 and 356 genes were targeted in leaf and root tissues, respectively. These findings propose that genotypic variation is responsible for the differential expression of miRNAs and the target genes in drought stress response. The results could serve as a guide for future research on the drought response mechanism.

**Supplementary Information:**

The online version contains supplementary material available at 10.1007/s00425-025-04757-3.

## Introduction

Wheat is one of the most critical cereals planted around 221 million ha farmable area in 2023 with around 800 million tons, and in second place after maize in terms of cultivation and production (FAO 2025). It contains carbohydrates, proteins, and various nutrients, besides offering lots of proteins, meets %20 of the total calories consumed by people (FAO 2025). Although the demand for wheat is parallelly increasing based on human population growth, climate changes cause instability in the distribution of wheat yield by years (Zampieri et al. 2017). It is calculated that the population will be reached 9 billion by 2050, so wheat yield should be increased, notwithstanding abiotic stress conditions to supply future demand. Studies on improving wheat varieties resistant to such stress conditions have gained importance day by day (Baloglu et al. 2014).

Abiotic stress, such as salinity, high temperature, and drought, is a significant handicap against wheat production, depending on climatic changes. Drought is one of the most common abiotic stresses that limit plant growth, development, and survival (Zhu 2016). Physiological and biochemical processes may be disturbed by dehydration and cost-unfavorable aftereffects for plants. For this reason, divers’ strategies, such as root elongation and osmotic stress regulation, have been enhanced throughout evolution by plants to protect themselves from the harsh effects of drought.

Basically, plants stand out from drought with two main strategies: evade and/or endure. In the first place, they extend the roots to a more profound level, economize the water supply, and adjust their life cycles to the precipitation regime (Nezhadahmadi et al. 2013). The other is by managing a small quantity of dehydration till eluding a dry period and then regrowing with the rainy season (Nezhadahmadi et al. 2013). Drought response is a complex mechanism including multiple genes, proteins, transcription factors (Alvarez et al. 2014), metabolites (Xiao et al. 2012), hormones (Reddy et al. 2014), and microRNAs (miRNAs) (Budak and Akpinar 2015). Thus, identifying critical regulators, like microRNAs, in drought response is vital to ensure food security and agricultural sustainability.

MicroRNAs (miRNAs) are endogenous, mostly conserved, small non-coding regulatory RNAs with 20–24 nt in length that regulate target gene expression in plants and animals (Reinhart et al. 2002; Kozomara et al. 2019; Chao et al. 2022). Even if miRNAs were first discovered in *C. elegans* in 1993 (Wightman et al. 1993), it took almost 10 years to discover them in plants (Reinhart et al. 2002). To date, 48,885 mature miRNAs’ sequences have been submitted to the miRBase database (http://www.mirbase.org/) (Kozomara et al. 2019). Our expertise about their biogenesis and mechanisms has been expanded in the following years from their discovery (Chao et al. 2022). Post-transcriptional degradation and translational repression are two different mechanisms in how they monitor their targets (Zhang 2015).

miRNAs are essential in many biological processes, such as hormone regulation, growth, differentiation, nutritional balance, and stress responses (Budak and Akpinar 2015; Ferdous et al. 2015; Liu et al. 2016). Many miRNAs associated with abiotic stress have been identified in different tissues of wheat (Budak et al. 2015b; Pandey et al. 2017; Alptekin et al. 2017; Singroha et al. 2021). Drought-related miRNAs have been identified in many plant species, such as *Arabidopsis thaliana* (Pegler et al. 2019), *Oryza sativa* (rice) (Nadarajah and Kumar 2019), *Hordeum vulgare* (barley) (Ferdous et al. 2017), *Zea mays* (maize) (Liu et al. 2019), and Triticeae (Pandey et al. 2014; Akdogan et al. 2016; Hua et al. 2019; Iquebal et al. 2019; Singroha et al. 2021; Gómez-Martín et al. 2023; Shamloo-Dashtpagerdi et al. 2023). It is shown that MYC2 is targeted by miR1119 and their expression levels were changed in wheat root under water scarcity (Shamloo-Dashtpagerdi et al. 2023). When wheat root tissues encounter water deficiency, miR1119 adjusts its expression, which also impacts its target—the MYC2 transcription factor. This is accompanied by alterations in the activity of several stress-response genes, a rise in abscisic acid (ABA) levels, and heightened activity within the cellular antioxidant system (Shamloo-Dashtpagerdi et al. 2023). During the early and middle stages of grain filling under drought conditions, Zhengmai 1860 (drought-resistant wheat variety), unlike Bainong 207 (drought-resistant wheat variety) and Zhoumai 18 (drought-susceptible wheat variety), upregulated the expression of tae-miR408. This action suppressed the translation of the allene oxide synthase (TaAOS) protein, leading to a decrease in jasmonic acid (JA) and abscisic acid (ABA) levels. As a result, it slowed chlorophyll degradation via the MAPK pathway, preserved the chloroplast structure and photosynthetic activity, and ultimately delayed leaf senescence (Zhou et al. 2024).

It is important not only to show differential expression profile of miRNAs but also to present their targets for discovering regulatory mechanisms underlying drought response. One of the procedures to establish the target mRNAs on a large scale is degradome sequencing, also named Parallel Analysis of RNA Ends (PARE) (German et al. 2009; Addo-Quaye et al. 2009). It is a hybridized method by modifying 5′-Rapid Amplification of cDNA Ends (RACE) with high-throughput sequencing to construct cleaved mRNA libraries and helps validate numerous target mRNAs instead of a few with RACE.

Polyploidy describes the presence of more than two complete sets of chromosomes in an organism. It has been reported that polyploidy causes various changes in genetic, epigenetic, transcriptional, and metabolic network levels (Lavania et al. 2012). Additionally, the studies also show that polyploidy is advantageous in tolerating stress conditions (Saleh et al. 2008; Meng et al. 2011; Wang et al. 2013). A previous study analyzed the movement of small RNA molecules (sRNAs) during the process of allopolyploidization in wheat, revealing notable shifts in sRNA expression profiles that occur with the formation of hybrid or allopolyploid genomes (Kenan-Eichler et al., 2011). In this study, we aimed to reveal previously defined and unidentified miRNAs and their target genes formed due to drought stress in three wheat varieties with different drought tolerance and ploidy levels—*Triticum aestivum*, a hexaploid cultivar (2n = 6x = 42, AABBDD), intermediate-tolerant; *Triticum turgidum* durum, a high-tolerant tetraploid cultivar (2n = 4x = 28, AABB); and *Triticum monococcum*, a low tolerant diploid cultivar (2n = 2x = 14, A^m^A^m^)—and to determine which miRNAs are lost and preserved during genome folding and their potential contribution to drought stress tolerance. In addition, both next-generation sequencing and Quantitative Real-Time PCR declared differential expression profiles of miRNAs and their target genes. Gene Ontology (GO) and MapMan pathway analyses of the target genes were performed to determine which metabolic pathways they play a role in. So, it can be understood how these genetic variations affect the expression of miRNAs, which are crucial for regulating gene expression and enabling plants to respond to drought stress.

## Materials and methods

### Plant materials, growth condition and stress application

Since wheat originated from Anatolia, wheat varieties developed in Anatolia with different ploidy levels and different drought tolerance were selected for the study. The seeds of *Triticum aestivum* (cv. Yüreğir-89), *Triticum turgidum* durum (cv. Kızıltan-91), and *Triticum monococcum* (cv. Siyez) were obtained from the Directorate of East Mediterranean Agricultural Research Institute (Adana, Turkey), the Directorate of Field Crops Central Research Institute (Ankara, Turkey), and İhsangazi Municipality (Kastamonu, Turkey), respectively.

In the study, 200 seeds (10 seeds per pot) were planted in 20 pots for each of the three different wheat varieties and grown under field conditions. The plantlets were covered to protect them from rain during cultivation and drought stress exposure. They were grown for 6 weeks and watered with 200 mL water once in 2 days and with 200 mL ½ Hoagland solution (Hoagland and Arnon 1950) twice a week.

At the end of the 6th week, the pots subjected to drought stress were carefully adjusted to an equal weight using water on the first day of the stress period. After this initial adjustment, no additional irrigation was provided until the drought stress phase came to an end. The weight measurements for all pots were recorded every 3 days following the initiation of the stress application (Fig. S1). Approximately 10 days later, the weights of the pots experiencing drought stress were found to be equalized. The samples were taken from the root and leaf tissues of the plants for RNA isolation when dehydration symptoms, like wilting leaves, were observed (Hackenberg et al. 2015; Ma et al. 2015). Upon completion of the drought treatment, leaf and root tissue samples were collected and promptly frozen in liquid nitrogen. These samples were subsequently stored at − 80 °C until they were ready for RNA isolation.

## Total RNA isolation

Total RNA from all leaf and root samples was isolated using Trizol reagent (Life Technologies Corporation) as stated in the product’s instructions. Afterward, purity and amount of isolated RNA were checked by using agarose (Thermo Fisher Scientific) gel electrophoresis, Multi-scan GO nano-spectrophotometer (Thermo Fisher Scientific), and Agilent 2100 Bioanalyzer (Agilent Technologies). The RNA samples were exposed to DNase I (Thermo) to avoid the risk of genomic DNA contamination. After it was again evidenced that the RNA samples were pure and sufficiently agreed with the evaluation, they were stabilized and protected from degradation at room temperature using RNAstable Tube Kit (Biomatrica) according to the manufacturer's instructions.

## Construction of miRNA and degradome libraries and sequencing

Degradome and small RNA sequencing were performed by BGI (Beijing Institute of Genomics, Shenzhen, China). After the RNA isolation from control and treated samples, small RNA fragments were purified by PAGE gel electrophoresis. Then, bands with a length of 18–30 nucleotides were removed from the gel and purified. The adapter regions were ligated to these purified small RNA fragments′ 3′ and 5′ ends. Following this step, a reverse transcription reaction was performed using primers suitable for adapter regions and then PCR amplification of RNA fragments was performed. PCR products were again run in PAGE electrophoresis and then gel purified. Finally, quality control tests of these libraries were made using Agilent 2100 Bioanalyzer and ABI StepOnePlus Real-Time PCR systems, and the libraries were made ready for sequencing. After the libraries were constructed, they were sequenced using Illumina HiSeq 2000 Sequencing System.

The RNAs from control samples were assembled to form six libraries following the process outlined by German et al. (2009) for the construction of degradome libraries. After extraction of degraded poly (A) RNAs, T4 RNA ligase ligated a 5′-adaptor molecule involved in specific sequence recognition by Mme I to the phosphate located on 5′ of poly (A) RNAs. Free adaptors were eliminated using an Oligotex kit, and reverse transcription was performed directly with purified ligated RNAs. Following the RT reaction, cDNAs were amplified with short PCR (5 cycles). After PCR products were exposed to Mme I and 3′-dsDNA adapters were ligated to the cleaved fragments, 20 cycles of PCR were performed on the ligated products. Finally, appropriate products collected from the gel were used for library construction and sequenced using Illumina HiSeq 2000 Sequencing System.

## Bioinformatic analysis of miRNAs

The formation of each unique sequence reads after extraction of low-quality fragments, clearance of contamination due to adapter–adapter ligation, and cleaning of adapter/acceptor sequences were considered small RNA fragments. It was then compared with non-coding RNAs (rRNA, tRNA, snRNA, snoRNA) available in Rfam (http://www.sanger.ac.uk/software/Rfam) (Griffiths-Jones et al. 2005; Nawrocki et al. 2015) and GenBank (http://www.ncbi.nlm.nih.gov) to classify the degraded fragments of non-coding RNAs.

SOAP (Short Oligonucleotide Analysis Package) analysis was used to determine miRNA expression levels and their location on the genome. Genome data of *T. aestivum* were downloaded from the"Wheat Genome Database v2.1"(https://www.wheatgenome.org/) and used in SOAP analysis. All sequences were searched using miRBase 22.1 (http://www.mirbase.org) (Griffiths-Jones et al. 2008) to identify known miRNAs. All sRNAs and identified orthologs of known miRNAs in miRBase were screened using the SOAP 2.0 program (Li et al. 2009) and Expressed Sequence Tags (ESTs).

In addition, pre-miRNA sequences were determined by allowing a maximum difference of three nucleotides (Zuker 2003) between known miRNAs and sequenced miRNA sequences. Moreover, all determined miRNA sequences in mature form were made against the wheat genome (http://www.ncbi.nlm.nih.gov/genome/11) by BLASTn (1e^−20^) and their RNA hairpin structures were estimated using the RNAfold (http://rna.tbi.univie.ac.at//cgi-bin/RNAWebSuite/RNAfold.cgi). Furthermore, the miRDeepFinder program (Xie et al. 2012) was used as an alternative to the abovementioned analyses to identify miRNAs and perform their functional analysis. miRDeepFinder has been used to classify miRNAs, identify pre-miRNA structures, target gene analyses, reveal expression profiles, and detect gene network analyses.

On the other hand, as a result of the mapping performed through the analyses mentioned above, sRNA (siRNA, piRNA, and snoRNA) sequences that did not correspond to any region in the genome were BLASTn into the wheat genome, and considering the criteria required for a sequence to be accepted as a miRNA in the literature (Allen et al. 2004), the relevant the folded secondary structure of the sequences was determined. Thus, considering the unique characters of miRNAs, the relevant sequences were predicted as novel miRNAs and identified as potential miRNA candidates. As an alternative to this analysis, all potential novel miRNAs were estimated using the default parameters of the MIREAP (MicroRNA Discovery by Deep Sequencing) program developed by BGI.

Venn diagram (https://bioinformatics.psb.ugent.be/webtools/Venn) was used to compare common and specific miRNAs among cultivars. The heatmaps and phylogenetic tree were created with Permut Matrix software using miRNA expression levels (Caraux and Pinloche 2005). Venn diagrams and heatmaps were also used to compare common and specific target genes and gene expression levels among cultivars.

## Degradome analysis of potential miRNA target genes

First, adapters and poor-quality reads were removed for analysis to identify miRNA target genes with the aid of degradome libraries. Then, sequences of 20–21 nucleotides in length were identified as potentially fragmented target gene sequences via CleaveLand (v2.0) program (Addo-Quaye et al. 2009). After that, BLASTn was performed on wheat EST and mRNA sequences from wheat genome databases (IWGSC; http://www.wheatgenome.org/) and NCBI database. Only perfectly matched gene fragments were considered candidate target genes.

## Bioinformatic analysis of potential miRNA target genes

To determine the potential target genes, the miRU (https://www.zhaolab.org/psRNATarget/) database was used based on BLASTn and was performed on the EST and cDNA sequences of wheat on the NCBI database.

## GO (Gene Ontology) and MapMan site of analysis for potential miRNA target genes

Detected miRNA target genes’ functions were annotated via BLAST2GO software (http://www.blast2go.com) (Conesa et al. 2005), and they were classified as biological processes, cellular components, and molecular functions. Also, the MapMan Site of Analysis program (https://mapman.gabipd.org/mapman) was used to find metabolic pathways containing the predicted target genes.

## cDNA synthesis and qRT-PCR verification

qRT-PCR was performed to both verify RNA-Seq results and find out the expression of the target genes. Based on differential expression data from RNA-Seq and literature, six miRNAs and one novel were selected. cDNA was synthesized using the iScript cDNA synthesis kit (BioRad). Stem–Loop primers were designed and used according to Chen et al. (2005). miRNA-specific forward primers and universal reverse primers are listed in Table S1. A total of 4 tubes were prepared for each sample (No-RT, No-Template, No-Stem Loop Primer, and null) for each miRNA. 1 µg isolated Total RNA, 1 mM Stem-Loop primer, 2 µL 5X Reaction Buffer, and 1 µL iScript Reverse Transcriptase were added to reaction tubes and the final volume was up to 10 µL with nuclease-free water. After that, the protocol was performed as follows: priming at 25 °C for 5 min, reverse transcription at 46 °C for 20 min, and RT inactivation at 95 °C for 1 min. After cDNA synthesis, serial dilution was performed and ½, ¼, 1/8, No-RT, No-Template, and No-Stem Loop Primer samples were subjected to qRT-PCR. qRT-PCR reaction was performed by using SYBR Green Mastermix (BioRad) with the Rotor-Gene Q system (Qiagen) instrument at 95 °C for 5 min, followed by 45 cycles of 10 s at 95 °C and 30 s at 60 °C followed by melting curve analysis with 1 °C increment in the range of 50–95 °C.

Also, three target genes for each miRNA were selected according to bioinformatics analysis of degradome data, psRNATarget database, GO, and MapMan software. The target genes-specific primers were designed using Primer 3 software (https://primer3.ut.ee/), then checked through the PRIMER-BLAST program (Ye et al. 2012) on the NCBI website and they are presented in the Table S1. *GAPDH* was selected as internal control instead of 18 s *rRNA* or *β-Actin* according to the results of NormFinder (Andersen et al. 2004) and BestKeeper (Pfaffl et al. 2004) algorithms (Table S2). iScript cDNA synthesis kit (BioRad) was also used for transforming the RNA samples to cDNA for mRNAs; the protocol was the same as mentioned above. The samples from three biological and three technical replicates were set up for each reaction. The results were analyzed using the 2^−ΔΔCt^ method (Schmittgen and Livak 2008). t-test analysis was performed for statistical analysis.

## Results

### Analysis of miR-Seq data and annotation of miRNAs

The raw reads between 18.140.201 and 35.073.803 from sRNA libraries constructed from control and drought-treated groups of three wheat cultivars’ leaf and root tissues were gathered. After removing the adaptor and low-quality sequence, 16.280.114 to 33.765.686 clean reads remained, ranging from 17 to 32 nt (Table S3). All libraries were mapped to miRBase, Rfam, siRNA, piRNA, and snoRNA. Every unique small RNA was mapped to only one annotation. The distribution of base quality on the clean tag, the length distribution of sRNA, the genome distribution of tags, and the distribution of sRNA were presented in Fig. S2a–d. The majority of sRNAs were 21–24 nt long. 20–24 nt long sequences represented the highest abundance (Fig. S2b). These results are similar to the literature that miRNAs are 20–24 nt in length and 21 and 24 nt miRNAs have a higher abundance (Li et al. 2011; Wang et al. 2011; Xu et al. 2013).

## Analysis of differentially expressed miRNAs

**Fig. 1:**
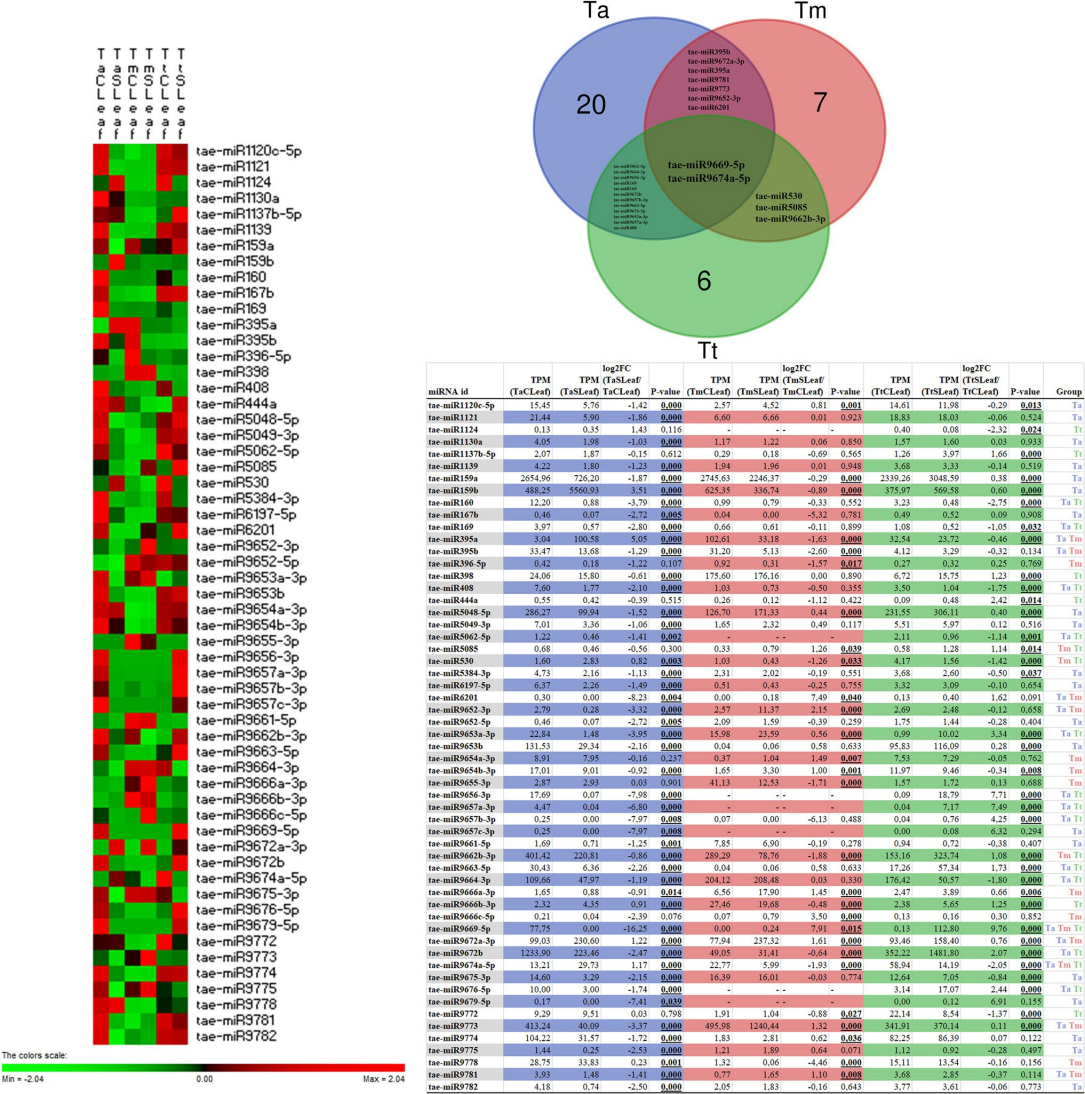
Differential expression of drought-responsive miRNAs (log2 fold change > 1 or <  − 1, *p* < 0.05) in leaf tissue. Heatmap shows z-score normalized expression profiles of differentially expressed miRNAs (log2 fold change > 1 or <  − 1, *p* < 0.05) in control (C) and stress-treated (S) leaf (L) tissues of *Triticum aestivum* (Ta), *Triticum monococcum* (Tm), and *Triticum turgidum* (Tt). Venn diagram displays the number of shared and species-specific significantly regulated miRNAs (log2 fold change > 1 or <  − 1, *p* < 0.05) among the three wheat species. The accompanying table shows TPM values, log2 fold change (log2FC), p-values, and group designation for each miRNA based on its significance in each species

In the leaf dataset, numerous miRNAs exhibited significant differential expression between stress and control conditions (Fig. 1). Significance was defined as a log2 fold change (log2FC) > 1 or <  − 1, with *p* < 0.05. The “Group” column in the table from the figure indicates which wheat variants—*Triticum aestivum* (Ta), *Triticum turgidum* (Tt), or *Triticum monococcum* (Tm)—show such significant regulation. Notably, tae‐miR9669‐5p and tae‐miR9674a‐5p appear in the group “Ta Tm Tt,” meaning they meet significance criteria across all three species. tae‐miR9669‐5p shows a dramatic negative fold change (log2FC − 16.25) in Ta leaves, yet significant positive shifts in Tm (+ 7.91) and Tt (+ 9.76). This striking contrast indicates that despite being commonly “stress‐responsive” in all three species, the regulatory outcome highly depends on each species’ genetic background or subgenome interactions. tae‐miR9674a‐5p similarly stands out by being significantly induced in Ta (log2FC + 1.17) but repressed in Tm (− 1.93) and Tt (− 2.05).

A subset of miRNAs shows significant changes in two of the three variants. tae‐miR160 is markedly down‐regulated in both Ta (− 3.79) and Tt (− 2.75). tae‐miR169 is likewise repressed in Ta (− 2.80) and more moderately down‐regulated in Tt (− 1.05). Other examples include tae‐miR408, tae‐miR5062‐5p**,** tae‐miR9653a‐3p, and tae‐miR9657 family members, each showing significant expression changes in Ta and Tt. tae‐miR395a is strongly up‐regulated in Ta (log2FC + 5.05) but suppressed in Tm (− 1.63). tae‐miR6201, tae‐miR9652‐3p, and tae‐miR9672a‐3p also show significance in Ta and Tm, often with opposite or mixed directionality (up in one species, down in the other). Fewer leaf miRNAs fall exclusively into “Tm Tt”. The examples include tae‐miR5085 and tae‐miR530, though each displays different degrees and directions of change between Tm and Tt. Many entries—such as tae‐miR1120c‐5p (Ta), tae‐miR1124 (Tt), or tae‐miR9654a‐3p (Tm)—are regulated significantly in one species only. For instance, tae‐miR1124 is up‐regulated in Tt leaves (log2FC + 1.43) but not in Ta or Tm, implying species‐specific adaptive roles. The miR159 family is well represented; tae‐miR159a is firmly down-regulated in Ta (− 1.87) but moderately up-regulated in Tt (+ 0.38), while tae‐miR159b surges in Ta leaves (+ 3.51) and modestly in Tt (+ 0.60). Several other stress‐associated families—miR395**,** miR398**,** miR408—appear significantly regulated. Overall, the leaf data show that many miRNAs are commonly stress‐responsive across multiple wheat species, but the magnitude and direction of differential expression can diverge widely.

**Fig. 2:**
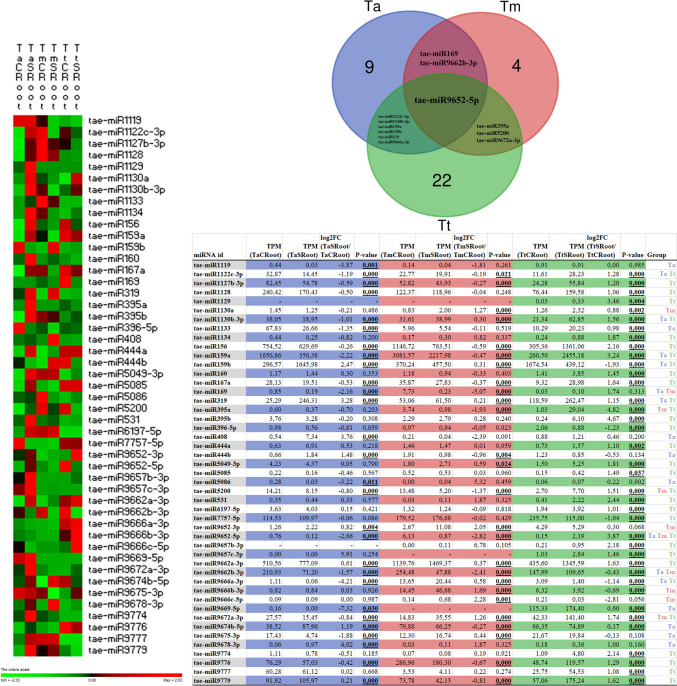
Differential expression of drought-responsive miRNAs (log2 fold change > 1 or <  − 1, *p* < 0.05) in root tissue. Heatmap presents z-score normalized expression profiles of differentially expressed miRNAs (log2 fold change > 1 or <  − 1, *p* < 0.05) in control (C) and stress-treated (S) root (R) tissues of *Triticum aestivum* (Ta), *Triticum monococcum* (Tm), and *Triticum turgidum* (Tt). Venn diagram illustrates shared and species-specific miRNAs with significant differential expression (log2 fold change > 1 or <  − 1, *p* < 0.05) across the three species. The table shows TPM values, log2 fold change (log2FC), p-values, and significant species groupings for each miRNA in root samples

The root dataset (Fig. 2) highlights multiple miRNAs exhibiting significant changes under stress (log2 fold change > 1 or <  − 1, *p* < 0.05). As with the leaf analysis, the “Group” column in the table from the figure designates which wheat variants (Ta: *Triticum aestivum*, Tm: *Triticum monococcum*, and Tt: *Triticum turgidum*) show statistically significant differential expression for each miRNA. In many cases, only one or two species meet these thresholds, while a few are shared by all three. Only tae‐miR9652‐5p is listed as “Ta Tm Tt” in the root dataset, highlighting its significance across all three species. Interestingly, the direction of regulation differs markedly. In Ta, the transition from 0.76 TPM (control) to 0.12 TPM (stress) corresponds to a log2 fold change of − 2.66, indicating a strong down‐regulation under stress. In Tm, a decrease from 6.13 TPM to 0.87 TPM yields a log2FC of − 2.82, suggesting substantial repression. In Tt, expression rises from 0.15 to 2.19 TPM (log2FC + 3.87), implying strong induction under stress.

Many miRNAs are jointly significant in exactly two of the three species, revealing overlapping yet not universal stress‐response pathways. tae‐miR159a shows a sharp decline in Ta (− 2.22 log2FC) but a robust induction in Tt (+ 3.24). This “opposite” expression profile within the same miRNA family points to potentially divergent target interactions and physiological outcomes. tae‐miR159b also follows an opposing trend: a strong increase in Ta roots (+ 2.47) but a decrease in Tt (− 1.93). tae‐miR319 is up‐regulated in both Ta (+ 3.28) and Tt (+ 1.15), suggesting a possible shared role in modulating root growth or other stress‐response pathways. Additional examples include tae‐miR1122c‐3p (down-regulated in Ta, up‐regulated in Tt) and tae‐miR1130b‐3p (down-regulated in Ta, up‐regulated in Tt). tae‐miR169 is down‐regulated in both Ta (log2FC − 2.16) and Tm (− 5.07). tae‐miR9662b‐3p is strongly repressed in both Ta (− 1.57) and Tm (− 2.41). tae‐miR395a is a prime example of shared significant expression change in Tm and Tt, which is down‐regulated in Tm (− 1.93) but sharply up‐regulated in Tt roots (+ 4.82). tae-miR5200 also shows a substantial induction (log2FC + 1.51) in Tt but was downregulated (log2FC −1.37) in Tm and did not meet significance in Ta. tae‐miR9672a‐3p is up‐regulated in Tm (+ 1.26) and Tt (+ 1.74), pointing to a consistent positive shift in root expression. tae‐miR1119 (− 3.87), tae‐miR1133 (− 1.35), tae‐miR5086 (− 3.22), and others show strong down‐regulation in Ta roots. In contrast, tae‐miR408 (log2FC + 3.76), tae‐miR444b (+ 1.48), and tae‐miR9678‐3p (+ 4.02) are up‐regulated specifically in Ta. tae‐miR1130a (log2FC + 1.27) and tae‐miR9666b‐3p (+ 1.69), tae‐miR9666c‐5p (+ 2.28) appear to be selectively induced in Tm. Many miRNAs did not meet significance in Ta and Tm, yet are strongly regulated in Tt, e.g., tae‐miR1127b‐3p (+ 1.20), tae‐miR1128 (+ 1.06), tae‐miR1134 (+ 1.87), tae‐miR156 (+ 2.16), and a host of others (e.g., tae‐miR9774, tae‐miR9776, tae‐miR9777, tae‐miR9779).

A central goal of this study is to discern how miRNA‐mediated regulation under stress might differ between leaf and root tissues across three wheat species. Overall, although numerous miRNAs appear in both datasets, only a subset reaches significance (log2 fold change > 1 or <  − 1, *p* < 0.05) in the same species and both tissues. miR159 family, which frequently appears in drought‐stress studies, shows strong regulation in the leaves and roots of *T. aestivum* and *T. turgidum*. In Ta leaves, *tae‐miR159b* is dramatically induced (log2FC + 3.51), whereas in Ta roots, *tae‐miR159b* likewise exhibits a positive fold change (+ 2.47). In Tt, however, the direction of change can differ between tissues (mildly up-regulated in leaves vs. strongly down-regulated in roots for some family members). Thus, while miR159 is consistently “stress‐responsive” in both leaves and roots, the magnitude and direction of its shifts can vary depending on species and isoform. miR9652‐5p was found to be notably significant in roots across all three species (Ta Tm Tt), and *tae‐miR9652‐5p* is also differentially expressed in leaves for at least two species (Ta and Tm). It is one of the few miRNAs that is systemically (leaf and root) stress‐responsive in multiple wheat genomes. However, it is up‐regulated in some cases and down‐regulated in others.

Many robustly up‐ or down‐regulated miRNAs in leaf tissue do not meet significance thresholds in roots, and vice versa, suggesting strong tissue specificity. As an example, *tae‐miR9669‐5p* stands out in leaves because it is significantly altered in all three wheat variants and does not appear in the root significance list. On the other hand, several miRNAs (e.g., *tae‐miR169* in Tm, *tae‐miR408* in Ta, *tae‐miR9666* family members in Tm) show significant changes in roots only. In some instances, the same miRNA may be up‐regulated in one tissue yet down‐regulated in the other for the same species. tae‐miR159a is substantially repressed in Ta roots (− 2.22 log2FC) but in leaves, different miR159 isoforms (e.g., tae‐miR159b) can be strongly induced. miR395 Family members are frequently up‐regulated in one tissue (e.g., Tt roots) and either neutral or suppressed in the other (Tt leaves). Even when specific miRNAs are significant in both tissues within a single species, the strength and direction of their regulation may differ between species. *T. aestivum* (Ta) often shows large‐magnitude fold changes (e.g., for miR159, miR9652, miR408), aligning with the more complex hexaploid genome potentially intensifying transcriptional and post‐transcriptional regulatory shifts. *T. turgidum* (Tt) consistently exhibits prominent regulation of certain well‐known stress‐responsive families (e.g., miR159, miR319, miR395) in both tissues but can display opposite directions of regulation compared to Ta. *Though diploid, T. monococcum (Tm)* sometimes mirrors Tt or Ta patterns and sometimes diverges widely, indicating that the presence or absence of particular subgenomes can drastically alter the expression outcomes of the same miRNA.

To find out the supposed roles of miRNAs, we investigated their expression profiles by comparing them on an individual basis of the cultivars. First, the heatmap was generated using TPM values of miRNAs from *T. aestivum* leaf samples libraries of control and drought-stressed groups and the log2 ratio was used to normalize the data. The results indicated that 70 miRNAs were upregulated, while 37 others were downregulated under drought stress (Fig. S3a). Among them, the expression levels of tae-miR395a, tae-miR159b, tae-miR9672a-3p, tae-miR9674a-5p, tae-miR1122c-3p, tae-miR167c-5p were significantly increased, while the expression levels of tae-miR9669-5p, tae-miR9656-3p, tae-miR9657a-3p, tae-miR9653a-3p, tae-miR160, tae-miR9672b, tae-miR159a were the most decreased. On the other hand, 52 miRNAs were upregulated, while 54 others were downregulated under drought stress in root samples (Fig. S3b). Among them, the expression levels of tae-miR408, tae-miR319, tae-miR159b, tae-miR9674b-5p, tae-miR9653b and tae-miR9662a-3p were significantly increased, while the expression levels of tae-miR159a, tae-miR9675-3p, tae-miR9662b-3p, tae-miR1133, tae-miR1122c-3p, tae-miR1125, tae-miR1118, tae-miR1123, tae-miR1136, tae-miR1128, tae-miR1135 and tae-miR156 were the most decreased.

Analyzing *T. monococcum* leaf samples showed that 30 miRNAs were upregulated, while 69 others were downregulated under drought stress (Fig. S3c). Among them, the expression levels of tae-miR9672a-3p, tae-miR9666a-3p, tae-miR9773, tae-miR5048-5p, tae-miR167c-5p were significantly increased, while the expression levels of tae-miR395b, tae-miR9674a-5p, tae-miR9662b-3p, tae-miR395a, tae-miR159b, tae-miR159a, tae-miR5200 were the most decreased. On the other hand, 39 miRNAs were upregulated, while 62 others were downregulated under drought stress in root samples (Fig. S3d). Among them, the expression levels of tae-miR9652-3p, tae-miR9666b-3p, tae-miR9672a-3p, tae-miR9662a-3p and tae-miR159b were significantly increased, while the expression levels of tae-miR395a/b, tae-miR9674a-5p, tae-miR9662b-3p, tae-miR159a/b and tae-miR5200 were the most decreased.

Under drought stress, 30 miRNAs were upregulated and 80 others were downregulated in *T. turgidum* leaf samples (Fig. S3e). Among them, the expression levels of tae-miR9669-5p, tae-miR9656-3p, tae-miR9657a-3p, tae-miR9653a-3p, tae-miR159a/a, and tae-miR156 were significantly increased while the expression levels of tae-miR9674a-5p, tae-miR9664-3p, tae-miR9772, and tae-miR7757-5p were the most decreased. On the other hand, 50 miRNAs were upregulated, while 59 others were downregulated under drought stress in root samples (Fig. S3f). Among them, the expression levels of tae-miR395a, tae-miR395b, tae-miR159a, tae-miR156, tae-miR167a and tae-miR9778 were significantly increased while the expression levels of tae-miR159b and tae-miR7757-5p were the most decreased.

A comparative analysis of differentially expressed miRNAs was performed for the cultivars'control and drought-treated miR-Seq libraries. First, common miRNAs were evaluated between control leaf samples of them. As a results, tae-miR167a, tae-miR1130b-3p, tae-miR9666a-3p, tae-miR1134, tae-miR1131, tae-miR1125, tae-miR9775, tae-miR9779, tae-miR1117, tae-miR9672a-3p, tae-miR9652-3p, tae-miR9665-3p, tae-miR1118 and tae-miR9777 in *T. monococcum*; tae-miR9780, tae-miR5049-3p, tae-miR1127a, tae-miR9781, tae-miR1137a, tae-miR1120c-5p and tae-miR1123 in *T. turgidum*; tae-miR9777a, tae-miR2275-3p, tae-miR9679-5p, tae-miR9657c-3p, tae-miR9669-5p, tae-miR9656-3p, tae-miR9657a-3p and tae-miR160 in *T. aestivum* were highly expressed (Fig. S4a). Additionally, it was clearly shown that the miRNA expression profile of *T. monococcum* was separated from the other cultivars based on the phylogenetic tree (Fig. S4a). Also, when the same analysis was performed in drought-stress leaf samples, we found that in *T. aestivum* tae-miR9777a, tae-miR159b, tae-miR9777, tae-miR5200, tae-miR9668-5p, tae-miR1127a, tae-miR1137a, tae-miR444a, tae-miR1123, tae-miR156; in *T. turgidum* tae-miR9657b-5p, tae-miR9657c-3p, tae-miR9679-5p, tae-miR9657b-3p, tae-miR9657a-3p, tae-miR9656-3p, tae-miR531; and in *T. monococcum* tae-miR1134, tae-miR1134, tae-miR1138, tae-miR1139, tae-miR9781, tae-miR1120c-5p, tae-miR5049-3p, tae-miR9674b-5p, tae-miR5048-5p, tae-miR5085, tae-miR159a, tae-miR396-5p, tae-miR9665-3p, tae-miR9670-3p, tae-miR169, tae-miR1117, tae-miR9780, tae-miR9773, tae-miR9652-3p were abundant (Fig. S4b). Again, *T. monococcum* was separated from the other cultivars based on its miRNA expression profile (Fig. S4b). Besides the shared miRNAs between all cultivars, we examined miRNAs expressed in *T. aestivum* and *T. turgidum* but not in *T. monococcum* (Fig. S4c).

Second, common miRNAs were evaluated between control root samples of them. As a results, tae-miR9662a-3p, tae-miR9665-3p, tae-miR5085, tae-miR5175-5p, tae-miR5384-3p, tae-miR9775, tae-miR397-5p, tae-miR9776, tae-miR167c-5p, tae-miR444a, tae-miR9773, tae-miR444b, tae-miR5050, tae-miR9655-3p and tae-miR9677a in *T. monococcum*; tae-miR9656-3p, tae-miR9657a-3p, tae-miR9669-5p, tae-miR9657c-3p, tae-miR9679-5p, tae-miR9657b-3p, tae-miR530, tae-miR9671-5p, tae-miR9659-3p, tae-miR171b, tae-miR1129 in *T. turgidum*; tae-miR9772, tae-miR1127a, tae-miR1137a, tae-miR6197-5p, tae-miR1134, tae-miR1133, tae-miR1124 in *T. aestivum* were highly expressed (Fig. S4d). A phylogenetic tree revealed that *T. monococcum* had a distinct miRNA expression profile from the other cultivars (Fig. S4d). Also, when the same analysis was performed in drought-stress root samples, we found that in *T. aestivum* tae-miR1133, tae-miR319, tae-miR6197-5p, tae-miR1127a, tae-miR9668-5p, tae-miR1137a, tae-miR9772, tae-miR9678-3p and tae-miR408; in *T. turgidum* tae-miR1119, tae-miR395a, tae-miR171b, tae-miR1129, tae-miR9679-5p, tae-miR9657a-3p, tae-miR9656-5p, tae-miR9669-5p; and in *T. monococcum* tae-miR5175, tae-miR530, tae-miR9661-5p, tae-miR9677a, tae-miR6201, tae-miR9673-5p, tae-miR9666b-3p, tae-miR9666a-3p, tae-miR9773, tae-miR9652-3p, tae-miR5085 were abundant (Fig. S4e). Again, *T. monococcum* was separated from the other cultivars based on its miRNA expression profile (Fig. S4e). Apart from miRNAs shared by all cultivars, we examined miRNAs expressed in *T. aestivum* and *T. turgidum* but not in *T. monococcum* (Fig. S4f).

## In silico and differential expression analysis of novel miRNAs

To determine novel miRNAs, unmapped sRNA sequences were blasted to Triticum genome and the secondary structure of the mapped ones was predicted according to the criteria in the literature (Allen et al. 2004). Subsequently, 50 sequences were established as novel miRNAs (Table S4) and their secondary structures were drawn in Fig. S5.

To find out the supposed roles of predicted novel miRNAs, we investigated their expression profiles by comparing them on an individual basis of the cultivars. The heatmap was generated using TPM values of novel miRNAs from *T. aestivum* leaf sample libraries of control and drought-stressed groups and normalized the data using the log2 ratio. According to the results, drought stress increased the expression of 10 novel miRNAs and decreased the expression of another 10 miRNAs (Fig. S6a). Among them, the expression levels of novel_sir16010, novel_sir16009, novel_sir18988, novel_sir53265 and novel_sir19059 were significantly increased, while the expression levels of novel_sir34266, novel_sir48490, and novel_sir29469 were the most decreased. On the other hand, 6 novel miRNAs were upregulated while 9 others were downregulated under drought stress in root samples (Fig. S6b). Among them, the expression levels of novel_sir47032, novel_sir16009, novel_sir29469, novel_sir24348, and novel_sir29231 were significantly increased, while the expression levels of novel_sir5591, novel_sir4616, and novel_sir18988 were the most decreased.

There were three novel miRNAs upregulated and four new miRNAs downregulated by drought stress, according to the analysis of *T. monococcum* leaf samples (Fig. S6c). In particular, novel_sir21467, novel_sir19059, and novel_sir30489 were significantly upregulated, while the expression levels of novel_sir30489, novel_sir19059, novel_sir18988, novel_sir16009, novel_sir16010, and novel_sir335 were the most decreased. On the other hand, six novel miRNAs were upregulated while seven others were downregulated under drought stress in root samples (Fig. S6d). Among them, the expression levels of novel_sir762, novel_sir29469, novel_sir15147, novel_sir5588, novel_sir51511, and novel_sir40708 were significantly increased while the expression levels of novel_sir48409, novel_sir6928, novel_sir18988, novel_sir51062, novel_sir16009, and novel_sir16010 were the most decreased.

Analysis of *T. turgidum* leaf samples showed that five novel miRNAs were upregulated while three others were downregulated under drought stress (Fig. S6e). Among them, the expression levels of novel_sir53510, novel_sir2547, novel_sir22208, and novel_sir39220 were significantly increased, while the expression level of novel_sir13297 was the most decreased. On the other hand, 23 novel miRNAs were upregulated while 37 others were downregulated under drought stress in root samples (Fig. S6f). Among them, the expression levels of novel_sir48680, novel_sir19326, novel_sir16009, novel_sir2112, novel_sir24348, novel_sir29469, and novel_sir15147 were significantly increased while the expression levels of novel_sir5588, novel_sir4616, novel_sir7746, novel_sir7708, novel_sir52778, novel_sir15474, novel_sir13120, novel_sir13117, and novel_sir52936 were the most decreased.

## Analysis of target gene prediction

**Fig. 3:**
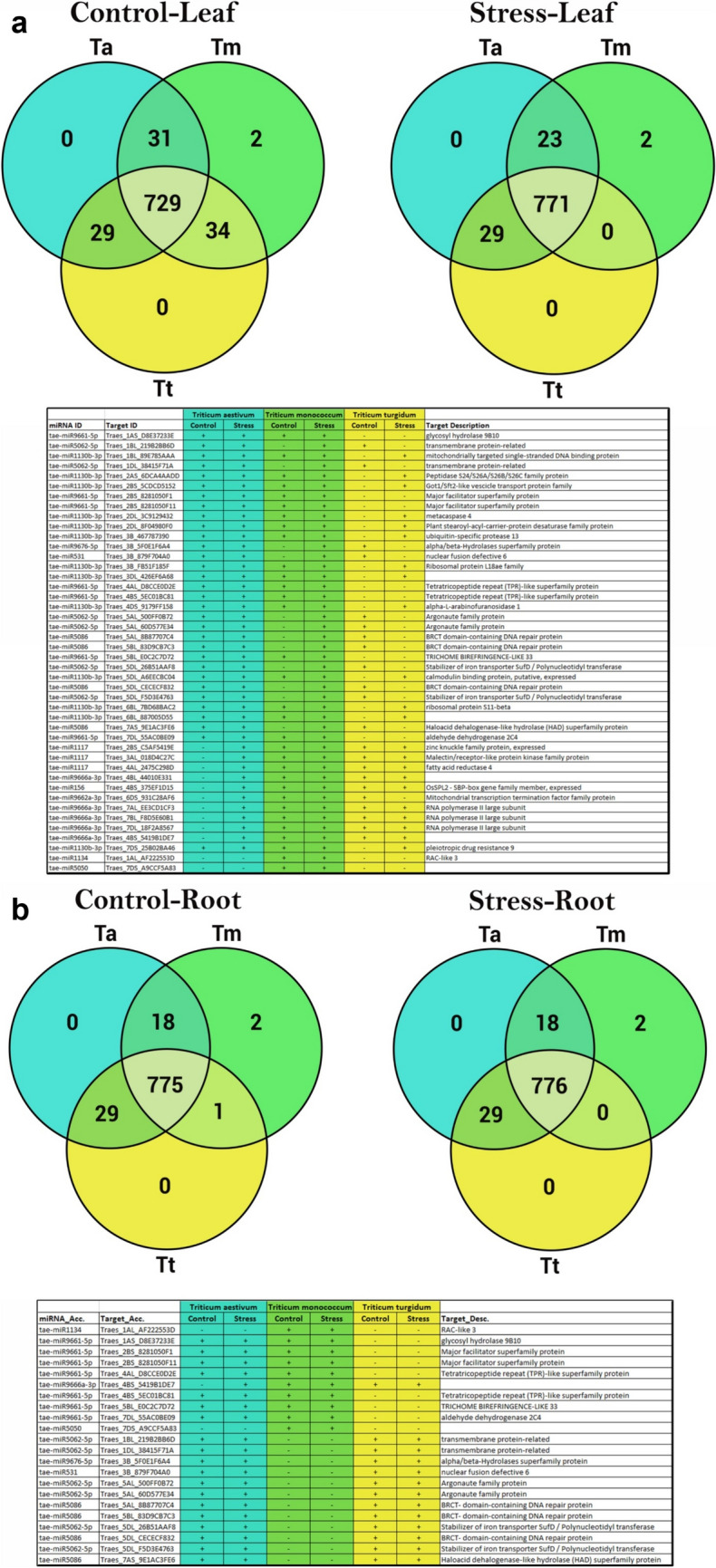
Venn diagram of all identified miRNAs'target genes. The figure shows shared and specific miRNAs'target genes in leaf tissues (**a**) and root tissues (**b**) of the three cultivars

The miRNA sequences were searched on the psRNA target database to predict the target genes (Dai and Zhao 2011). We found that 729 and 771 genes were targeted in control-leaf and stress-leaf groups of all cultivars, respectively (Fig. 3a). On the other hand, 775 genes in control and 776 genes in drought-stressed groups were targeted in root samples (Fig. 3b). Also, all target genes discovered by using the psRNA target database were also annotated (Table S5−6).

In addition to in silico analysis, we performed degradome analysis for target gene prediction. The results based on degradome data showed that 351 and 356 genes were targeted in leaf and root tissues, respectively (Fig. S7−8). Also, we detected those 23 genes not expressed in control but existed in stress groups using degradome data (Fig. S9). Moreover, all target genes discovered based on degradome data were annotated (Table S7).

When the MapMan analysis was applied to the predicted target genes, results showed that they have a role in 35 different metabolic pathways, predominately on stress, RNA, protein, and signaling (Fig. S10a). Investigation of stress pathways revealed that identified miRNAs target abiotic stress, signaling pathways, transcription factor families (ERF, WRKY, MYB), heat shock proteins, and PR proteins (Fig. S10b). Moreover, the targeted genes from all transcription factor families involved in the MapMan database were shown in Fig. S10c, and hereunder; some transcription factor families (AP2EREBP, MADS, MYB, NAC, GRAS, WRKY, and SBP) were highly targeted by miRNAs under drought stress. Furthermore, cellular functions, regulations, and RNA–Protein relationship pathways are presented in Fig. S10d-f, respectively.

## Validation of differentially expressed miRNAs and target genes by qRT-PCR

The qRT-PCR was performed for confirmation of differentially expressed miRNA data from the sequencing and in silico analysis and to find out the correlation between miRNAs and their targets. miRNAs were chosen based on significant differences in expression levels between control and drought-stressed samples and the targets were selected by considering in silico and bioinformatic analysis. The results were compatible with miR-Seq data and in harmony with target gene expression patterns.

**Fig. 4:**
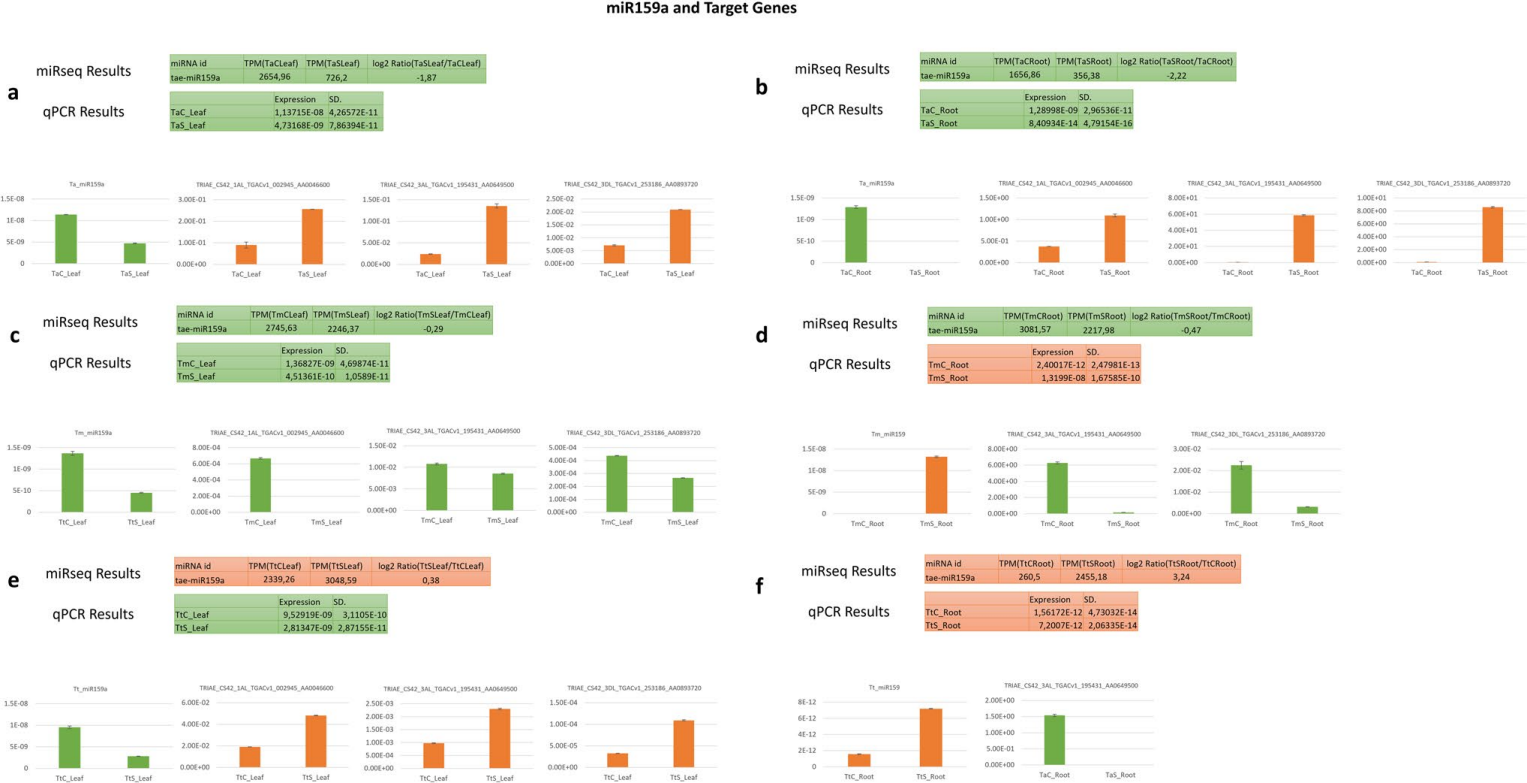
qRT-PCR analysis of differentially expressed miR159a and its target genes. The figure shows difference of expression level of miR159a and the target genes under drought stress in comparison with control group in *T. aestivum* leaf samples (**a**), *T. aestivum* root samples (**b**), *T. monococcum* leaf samples (**c**), *T. monococcum* root samples (**d**), *T. turgidum* leaf samples (**e**), and *T. turgidum* root samples (**f**). Green color indicates downregulation while red color indicates upregulation of the gene

First, we analyzed miR159a and its three targets, two of which are members of gibberellin and abscisic acid-regulated MYB (GAMYB) transcription factor family but the last one has not been annotated. In both *T. aestivum* leaf and root tissues, miR159a was downregulated while the targets were upregulated (Fig. 4a, b). miR159a and the targets’ expression level were decreased in *T. monococcum* leaf tissues (Fig. 4c), but the expression of miR159a was increased and the targets were suppressed in the root (Fig. 4d). Finally, it was decreased in the *T. turgidum* leaf but increased in root tissues, while its targets were upregulated in the leaf but downregulated in root tissues (Fig. 4e, f). Because of large data, we present only miR159a and its target genes qRT-PCR figure in text and the other figures are submitted as supplementary files (Fig. S11).

Next, miR156 and its three targets were studied, all of which are members of Squamosa Promoter Binding Protein-Like (SPL) transcription factor family. In both *T. aestivum* leaf and root tissues, miR156 was downregulated, while the targets were upregulated in the root but downregulated in leaf tissues (Fig. S11a-I, II). miR156 expression level was increased in *T. monococcum* leaf and root tissues (Fig. S11a-III, IV) but expressions of its targets were suppressed in the leaf while they were not detected in root tissues (Fig. S11a-III, IV). Finally, it was increased in *T. turgidum* in leaf and root tissues while its targets were downregulated in the leaf but were not detected in root tissues (Fig. S11a-V, VI).

miR167c and its three targets were also selected for validation experiments in which, 4-hydroxyphenylpyruvate dioxygenase and 3-hydroxyisobutyrate dehydrogenase, but the last one has not been annotated. In *T. aestivum* leaf tissues, miR167c was upregulated but downregulated in the root, while the targets were downregulated in the root but upregulated in leaf tissues (Fig. S11b-I, II). miR167c expression level was suppressed in the *T. monococcum* leaf but increased in root tissues (Fig. S11b-III, IV) and expressions of its targets were upregulated in the leaf while they were not detected in root tissues (Fig. S11b-III, IV). Finally, it was decreased in *T. turgidum* in the leaf and root tissues, while its targets were upregulated in the leaf but were not detected in root tissues (Fig. S11b-V, VI).

Next, miR5048 and its three targets which code nucleoprotein TPR, casein protein kinase 2 (CK2), and vesicle-associated membrane protein 7 (VAMP7) were analyzed. In both *T. aestivum* leaf and root tissues, miR5048 was downregulated while the target, CK2, was upregulated in root and leaf tissues (Fig. S11c-I, II). miR5048 expression level was increased in *T. monococcum* leaf and root tissues (Fig. S11c-III, IV) but expressions of its target, VAMP7, were suppressed in both leaf and root tissues (Fig. S11c-III, IV). Finally, it increased in *T. turgidum* in leaf and root tissues while its targets were not detected in the root or leaf tissues (Fig. S11c-V, VI).

In addition, miR9664-3p and its three targets which code Calcium-dependent protein kinase 2 (CPK2), a member of heat shock protein 90 family (HSP90) and Serine-glyoxylate aminotransferase (SGAT) were chosen for qRT-PCR analysis. In both *T. aestivum* leaf and root tissues, miR9664-3p was downregulated while the target, CPK2, was upregulated in leaf and root tissues; also, HSP90 was upregulated in root tissues (Fig. S11d-I, II). miR9664-3p expression level was increased in *T. monococcum* root tissues but decreased in leaves (Fig. S11d-III, IV). Expression of its targets was increased in the leaf while not detected in root tissues (Fig. S11d-III, IV). Finally, it was suppressed in *T. turgidum* in leaf and root tissues while its targets, HSP90 and SGAT, were increased in the leaf but none were detected in root tissues (Fig. S11d-V, VI).

Furthermore, we selected miR9672a and its three targets, and two were coded for alanine-glyoxylate transaminase/serine-glyoxylate transaminase and the pyruvate dehydrogenase E1. In *T. aestivum* leaf tissues, miR9672a was upregulated while the targets were downregulated (Fig. S11e-I). miR9672a expression level was increased in *T. monococcum* (Figure S11e-II) but expressions of its targets were not detected in leaf tissues (Fig. S11e-II). Finally, it was decreased in *T. turgidum* in leaf tissues while its targets were upregulated in the leaf (Fig. S11e-III).

Finally, we analyzed novel miRNA16009 and its three targets, which are Serine-glyoxylate aminotransferase (SGAT), and the other has not been annotated. In *T. aestivum* leaf tissues, novel miRNA16009 was upregulated while the targets were downregulated (Fig. S11f-I). Novel miRNA16009 expression level was decreased in *T. monococcum* (Fig. S11f-II) but expressions of its targets were upregulated in leaf tissues (Fig. S11f-II). Also, it was increased in *T. turgidum* in leaf tissues while its target, SGAT, was downregulated in the leaf (Fig. S11f-III).

## Discussion

Plants are sessile organisms, so drought stress is one of the major handicaps for them. Through millions of years of evolution, they have developed different strategies to protect themselves from its fatal effect. Crop yield is restricted by rainlessness in regions like Anatolia, where agriculture depends on rain. Wheat has been cultivated for thousands of years since day one in Anatolia. So, it gives us an open lab experiment to study drought stress response on the inheritable genetic background of ancient to modern wheat. To date, a vast number of researches have been performed for drought stress miRNAs on different cultivated plants such as Arabidopsis (Pegler et al. 2019), rice (Nadarajah and Kumar 2019), barley (Ferdous et al. 2017), maize (Liu et al. 2019), and wheat (Akdogan et al. 2016; Hua et al. 2019; Singroha et al. 2021; Yue et al. 2022; Gómez-Martín et al. 2023). In these studies, most of the results agreed with our findings. It was shown that miR156 was three times higher than the whole seedlings control group under drought in *Arabidopsis* according to Pegler et al. (2019); in rice, miR159 was increased and MYB proteins was suppressed as a result of drought (Nadarajah and Kumar 2019); miR5048 was decreased in barley (Ferdous et al. 2017); miR159, miR156 and miR167 was up- and down-regulated in maize (Liu et al. 2019); miR156 and miR167 were upregulated in soybean (Ramesh et al. 2019); miR156, miR159, miR167, and miR5048 was up- and down-regulated in wheat (Akdogan et al. 2016; Hua et al. 2019; Singroha et al. 2021; Yue et al. 2022; Gómez-Martín et al. 2023). Although many studies have been published about drought-associated miRNAs in wheat, a few of them have been compared to different genotypes and also cultivars with different ploidy levels have been rarely subjected to a study.

In this study, we constructed miRNA and their degradome libraries using NGS technology to identify expression differences under drought stress among three wheat cultivars with different ploidy levels and across leaf and root tissues. *Triticum monococcum*, *Triticum turgidum*, and *Triticum aestivum* were subjected to miR-seq for this purpose. As a result of the bioinformatics analyses, 112 wheat miRNAs that were shared by all cultivars or expressed as cultivar-specific were identified. It was revealed that miRNAs identified as shared or cultivar-specific differ in root and leaf tissues. Among unmapped sRNA sequences which do not correspond to any known miRNAs, 50 predicted miRNAs were characterized for the first time according to the criteria (Allen et al. 2004). In addition, target genes of all detected miRNAs were determined with the help of bioinformatics tools and degradome analyses, and 825 genes were identified. GO and MapMan pathway analyses of the determined target genes were performed to obtain information about the functions of target genes.

A wheat miRNA—pri-tae-miR159a—was transferred to plant tissues using the barley strip mosaic virus (BSMV) system. Silencing of pri-tae-miR159a and accumulation of vsiRNAs in wheat resulted in phenotypes resistant to Pst Chinese yellow rust disease (*Puccinia striiformis* f. sp. tritici) (Feng et al. 2013). The regulation of taMyb3 genes, one of this miRNA's target genes, played an important role in protecting wheat against disease (Feng et al. 2013). Similarly, Zhou et al. (2010) determined that the expression level of miR159a decreased as a result of drought stress in rice. Our results are similar with the previous study and showed a decrease in the expression level of tae-miR159a in the leaf samples of *T. aestivum*, *T. monococcum*, and *T. turgidum*. As a result of the decrease in the expression of tae-miR159a, an increase was observed in the expression of gibberellin and abscisic acid-regulated MYB (GAMYB) transcription factor family genes according to qRT-PCR analysis. The GAMYB transcription gene family was first identified as an activator of gibberellin acid (GA) that regulates cereal grains genes (Woodger et al. 2003). In addition, MYB proteins are known to be the largest of the plant transcription factor families. In plants, members of this family are responsible for the development, metabolism, and response to biotic and abiotic stresses (Dubos et al. 2010). Rahaie et al. (2011) examined the expression profile of ten MYB genes in salt stress-resistant and susceptible cultivars of *T. aestivum*. The *TaMYBsdu1* gene, which provides resistance to salt and drought stress, was characterized in the study. Baloglu et al. (2014) showed that the *TaMYB33* gene plays a role in the short-term abiotic stress response in *T. turgidum* and *T. aestivum* cultivars. These results are similar to our findings, and it was observed that the expression of MYB genes increased with the suppression of tae-miR159a for *T. aestivum* and *T. turgidum* in response to drought stress. In *T. monococcum*, although the suppression of tae-miR159a was confirmed by both miRNAome and qRT-PCR, a decrease in the expression of these three target genes belonging to the MYB transcription factor family was observed. It can be explained by the fact that different miRNAs can also regulate these target genes.

As a result of the literature research, it has been revealed that miR156 plays very different roles in different plants. For example, delayed flowering and biomass increase were observed in alfalfa plants as a result of overexpression of miR156 (Aung et al. 2015a, b). In another study, a high expression level of At-miR156 from *Arabidopsis* plant was shown to increase carotenoid and branching in *Brassica napus* plant (Wei et al. 2010). However, miR156 is also responsible for the regulation of different abiotic stresses in plants. For example, different studies have shown that miR156 expression changes in rice, wheat, tobacco, poplar, hybrid Paulownia corn, barley, and peach plants as a result of abiotic stresses such as drought, salt, cadmium, and ABA (Hackenberg et al. 2015; Budak et al. 2015a, b; Fan et al. 2016; Zhang et al. 2016a, b; Duan et al. 2016; He et al. 2016). Similar to the literature, the responses of tae-miR156 to drought stress in leaf and root tissues of wheat species with different ploidy levels varied in our study. It was observed that the expression of tae-miR156 increased and accordingly the expression of all target genes belonging to the Squamosa Promoter Binding Protein-Like (SPL) family decreased, especially after the application of drought stress in the leaf tissues of all wheat species. Arshad et al. (2017) showed drought tolerance was increased in plants when SPL genes were knockdown, and thus plants gained drought resistance. Similar results were obtained in our study, and it was determined that the degradation of SPL transcripts formed a resistance mechanism to drought conditions on *T. aestivum*, *T. monococcum*, and *T. turgidum*. In addition, it was observed that the expression of tae-miR156 increased in root tissues of *T. monococcum* and *T. turgidum*, but qRT-PCR could not determine target genes. On the other hand, it was found that the expression level of tae-miR156 decreased and the expression of different SPL genes increased due to drought stress in the root tissue of *T. aestivum*. In a different study, overexpression of SPLs (SPL3, SPL9 and the most dominant SPL10) was shown to inhibit lateral root development (Yu et al. 2015). Therefore, when the plant is exposed to prolonged drought stress, it tends to elongate with the development of the primary root by reducing or stopping the lateral root development. This situation can be considered among the mechanisms for increasing drought tolerance for *T. aestivum*.

It was determined that the expression of miR167c in the control and drought-stressed root tissues of *T. aestivum* decreased significantly after drought stress. This decrease in miR167 caused an increase in the expression of all the target genes studied, namely TRIAE_CS42_6AS_TGACv1_ 485975_AA1555050 encoding the 4-hydroxyphenylpyruvate dioxygenase enzyme, and the TRIAE_CS42_7BL_TGACv1 coded target gene encoding the 3-hydroxyisobutyrate dehydrogenase enzyme. According to GO annotation analysis, these enzymes are involved in phenylalanine metabolism and valine, leucine-isoleucine degradation pathways, respectively. 4-hydroxyphenylpyruvate dioxygenase (HPPD) is the first processed enzyme involved in the biosynthesis of vitamin E and is characterized by catalyzing the conversion of p-hydroxyphenyl pyruvate (HPP) to homogentisic acid (HGA). In the study conducted by Jiang et al. (2017), an HPPD gene was cloned from *Medicago sativa* L. and expressed at high levels in alfalfa leaves. Drought (with the aid of polyethylene glycol), NaCl, abscisic acid, and salicylic acid have been shown to significantly induce the expression of this gene, particularly in cotyledons and root tissues. Overexpression of HPPD was found to significantly increase the level of β-tocotrienol and total vitamin E content in *Arabidopsis* seeds. Moreover, these transgenic *Arabidopsis* seeds exhibited an accelerated germination time compared to wild-type seeds under normal conditions as well as under NaCl and ABA treatments. In our study, the increase in the expression of HPPD gene as a result of suppression of miR167c expression in root tissues exposed to drought stress of *T. aestivum* can be shown as one of the mechanisms created by *T. aestivum* for root tissue against drought stress. According to miRNAome analysis results, it was observed that the expression of tae-miR167c increased in control and drought stress leaf tissues of *T. monococcum*. It was determined that the expression of the same target gene of tae-miR167c in *T. aestivum* was also suppressed. However, the expression level of miR167c was opposite in root tissue compared to leaf tissue. It was observed that the expression of miR167c increased and the expression of any target gene was not detected. The increase in the expression of HPPD gene in the leaf tissue of *T. monococcum* can also be considered as a drought tolerance mechanism for *T. monococcum*. In addition, when the 3-hydroxyisobutyrate dehydrogenase enzyme is examined, it has been determined that it is involved in the destruction of branched-chain amino acids (BCAAs) (Schertl et al. 2017). Amino acid catabolism in plants becomes especially important in metabolic stress conditions (e.g., abiotic stress conditions in the extended dark and limited carbohydrate availability). Under these conditions, the amino acids are used as alternative substrates for respiration. The complete oxidation of the BCAAs leucine, isoleucine (Ile) and valine (Val) in the mitochondria effectively allows the formation of ATP by oxidative phosphorylation. Schertl et al., (2017) showed that 3-hydroxyisobutyrate dehydrogenase is responsible for the degradation of isoleucine and valine amino acids. Therefore, it has been observed that some of the energy required to perform metabolic activities in *T. monococcum* leaf tissues during drought stress is realized by destroying some branched-chain amino acids. Therefore, it can be considered one of the drought resistance mechanisms for *T. monococcum*.

Another miRNA that has been studied in detail is tae-mir5048. It was determined that the expression of tae-mir5048 decreased as a result of drought stress and therefore the expression of the target gene increased as a result of the decrease in the pressure on the target gene coded TRIAE_CS42_2BL_TGACv1_ 132794_AA0439840. When the annotation of the target gene was examined, it was determined that the gene encodes casein protein kinase 2 (CK2). CK2, formerly known as casein kinase II is a continuously synthesized Ser/Thr kinase found in all eukaryotes. CK2 involves multiple developmental and stress-sensitive pathways in plants, including light signaling and the circadian clock (Mulekar and Huq 2014). The CK2 substrates identified so far are mainly transcription factors or regulatory proteins. In a study, ABA 17 (Rab17), a substrate of CK2, was examined (Riera et al. 2004). A study showed that the ABA 17-responsive CK2 substrate stopped seed germination under stress conditions in maize. Phosphorylation of Rab17 is required for function as well as subcellular localization. The Rab17 mutant accumulated in the nucleolus and did not inhibit germination under high salt stress when overexpressed in *Arabidopsis* (Riera et al. 2004). The decrease in the expression of tae-mir5048 as a result of drought stress in the leaf and root tissues of *T. aestivum* may cause the increase in the expression of CK2, which means that the expression of one or more of the CK2 substrates increases, so it is thought that it can form one of the drought resistance mechanisms in *T. aestivum*. When miRNA sequencing and quantitative Real time PCR results of tae-mir5048 in the leaf tissues of *T. monococcum* were examined, it was observed that the expression of tae-mir5048 increased with drought stress as a result of both analyses. Accordingly, it was observed that the expression level of the target gene called vesicle-associated membrane protein 7 (VAMP7) decreased. Likewise, it was determined that the expression of tae-mir5048 increased and the expression of the same target gene decreased accordingly after drought stress application in the root tissues of *T. monococcum*. Triacylglycerol is transported from the ER to the cis-Golgi in a special vesicle, the pre-chylomicron transport vesicle (PCTV). VAMP7 was found to be more concentrated on PCTV membranes than on ER membranes. VAMP7 has been previously described in association with post-Golgi regions in eukaryotes (Siddiqi et al. 2006). The same researchers have also shown that VAMP7 is concentrated in the intestinal ER and plays a functional role in delivering triacylglycerol from the ER to the Golgi. It was observed that the expression of tae-mir5048 was increased and the expression of VAMP7 was decreased as a result of drought stress in the leaf and root tissues of *T. monococcum*. So, it can be concluded that *T. monococcum* tended to decrease triacylglycerol transport. Thus, it can be said that *T. monococcum* tends to slow down the processes related to lipid metabolism while struggling with drought stress.

As a result of miRNAome and qRT-PCR analyses, a decrease in the expression level of tae-miR9664 was observed. It was found that the expression of the target gene of tae-miR9664, annotated as calcium-dependent protein kinase 2, and coded TRIAE_CS42_2AL_TGACv1_ 093275_AA0276480 increased in drought stress compared to the control. In root samples of *T. aestivum*, the expression of tae-miR9664 was not adjusted as a result of miRNAome analysis, only qRT-PCR was performed and it was determined that its expression decreased under drought stress. Accordingly, the expression of calcium-dependent protein kinase increased in the leaf and the expression of target genes coded TRIAE_CS42_7DS_TGACv1_622763_AA2044980 belonging to the Hsp90 gene family in root tissue was found to increase. Previous studies have shown that Hsp90s are essential in plant growth, stress response, and disease resistance (Rizhsky et al. 2002; Sangster and Queitsch 2005; Jarosz and Lindquist 2010). The results obtained are similar to the literature. The increase in the expression of the Hsp90 in the root tissue of *T. aestivum* can be shown among the responses to drought stress. Similarly, in the leaf samples of *T. monococcum*, a decrease of approximately 3.5 times was detected as a result of the miRNAome analysis of tae-miR9664 and a decrease of approximately 2 times as a result of qRT-PCR analysis. So, it was revealed that the expression levels of all selected target genes increased. Among the target genes, TRIAE_CS42_7DS_TGACv1_622788_AA2045350 encodes Serine-glyoxylate aminotransferase enzyme and TRIAE_CS42_7DS_ TGACv1_622763_ AA2044980 encodes heat shock protein 90 (heat shock protein 90). The expression of tae-miR9664 in control and drought-stressed root tissues of *T. monococcum* was determined using only qRT-PCR. As a result of this analysis, it was determined that the expression of tae-miR9664 increased after drought stress application. However, none of the selected target genes were identified in qRT-PCR. One of the possible reasons for this is that the expression of target genes cannot be detected even using qRT-PCR. Finally, sequencing and PCR analyses of tae-miR9664 in the leaf tissues of *T. turgidum* were examined and it was observed that the expression level of this miRNA decreased in both analyses. In addition, an increase in the expression levels of the target genes of this miRNA encoding the calcium-dependent protein kinase and serine-glyoxylate aminotransferase enzymes (SGAT) were detected. This state can be explained by the decreased expression of tae-miR9664 in leaf tissues of *T. turgidum* after drought stress application. In *T. turgidum* leaf tissue, expression changes of miRNAome and target genes could not be detected but only the changes in the expression level of tae-miR9664 were determined by qRT-PCR. It was observed that the expression of tae-miR9664 decreased significantly due to drought stress in root samples of *T. turgidum*. However, after this decrease, the expression of selected target genes could not be observed.

tae-miR9664 was first detected in germinated wheat germ in a study by Han et al. (2014). This study clarified the regulatory networks of miRNAs that play a role in the development of wheat leaves and seeds, and 55 new miRNAs were identified together with tae-miR9664 (Han et al. 2014). However, tae-miR9664 has not been specifically examined in this study or in any other study from literature, and no research has been done on its target genes. Within the scope of the study, the expression of tae-miR9664 decreased as a result of drought stress. Accordingly, it was determined that the expression of the target genes encoding serine-glyoxylate aminotransferase (SGAT) enzyme and heat shock protein 90 were increased according to miRNAome and qRT-PCR analyses of *T. aestivum*, *T. monococcum*, and *T. turgidum* leaf samples. SGAT converts glyoxylate and serine to glycine and hydroxy pyruvate during photorespiration (Modde et al. 2017). When exposed to drought stress, plants increase the production of osmoprotectants such as proline and glycine betaine (Baloǧlu et al. 2012; Kavas et al. 2013). Therefore, the increase in the expression of the gene encoding the SGAT enzyme in leaf tissues as a result of drought stress in all wheat varieties can be explained in this way. In addition, different studies have shown that the expression levels of genes encoding heat shock proteins increase depending on salt and drought stresses (Yer et al. 2016). Therefore, in addition to their other functions, it has been stated that the genes encoding heat shock proteins also act as stress response genes in different abiotic stresses. The responses of the leaf samples of *T. aestivum*, *T. monococcum*, and *T. turgidum* to drought were examined at both miRNA and mRNA levels, and similar results were obtained in the literature related to abiotic stresses. In addition, results close to the leaf samples were obtained only in root samples of *T. aestivum*, which shows that *T. aestivum* is trying to respond to drought stress with all selected tissues.

Another miRNA whose expression was examined by miRNAome and qRT-PCR is tae-miR9672a. Its expression was approximately 2.3 and 1.2 times lower than the control according to miRNAome and qRT-PCR, respectively. Three target genes of tae-miR9672a were selected and their qRT-PCR analyses were performed. The annotations of the selected target genes were determined as the genes encoding the alanine-glyoxylate transaminase/serine-glyoxylate transaminase enzymes and pyruvate dehydrogenase E1 enzyme. The expression levels of all target genes increased under drought stress compared to the control. This is an expected situation, and the pressure of tae-miR9672a, whose expression was decreased as a result of drought stress, on its target genes decreased and an increase in the expression levels of target genes was observed. One of its target genes, Pyruvate dehydrogenase complex (PDC), is a multienzyme that catalyzes the complex oxidative decarboxylation of pyruvate to yield acetyl-CoA and NADH. The PDC complex found in plants also contains three essential components: pyruvate dehydrogenase (E1), dihydrolipoyl acetyltransferase (E2), and dihydrolipoyl dehydrogenase (E3) (Tovar-Méndez et al. 2003). These enzymes are associated with pyruvate metabolism and the citric acid cycle (TCA) and are directly related to metabolism. The high expression of pyruvate dehydrogenase E1, one of the target genes of tae-miR9672a in *T. aestivum* after drought stress, can be considered as an indication of an active metabolic situation which means that its metabolic activities are not affected by drought stress. In addition, the high expression of alanine-glyoxylate transaminase/serine-glyoxylate transaminase (AGXT1 and AGXT2) target genes can be shown as other evidence supporting this situation. According to GO and MapMan analyses, the fact that AGXT1 and AGXT2 genes are also involved in metabolic pathways such as glyoxylate and dicarboxylate metabolism, Glycine, serine, and threonine metabolism, and Alanine, aspartate, and glutamate metabolism is an indication that *T. aestivum* fulfills its metabolic activities during drought stress. The changes in the expression level of tae-miR9672a were also examined in the control and drought-stressed leaf tissues of *T. monococcum*, and as a result of miRNAome and qRT-PCR analyses, it was observed that its expression increased significantly after drought stress application. However, the expression level of three selected target genes of tae-miR9672a could not be determined with qRT-PCR. It is because target gene expression is likely too low to be measured by qRT-PCR. Changes in the expression level of tae-miR9672a and its target genes were also obtained in leaf tissues of *T. turgidum* exposed to drought stress, similar to the leaf samples of *T. aestivum*. It was observed that the expression of 3 selected target genes increased while the expression of tae-miR9672a decreased after drought stress application. Similar comments can be made regarding the drought stress for *T. turgidum*. The increase in the expression levels of genes closely related to metabolism in leaf tissues exposed to drought stress can indicate that *T. turgidum* can perform its metabolic activities without being affected by drought stress. In addition, the changes in the expression levels of tae-miR9672a and its target genes in root samples of *T. aestivum*, *T. monococcum*, and *T. turgidum* could not be determined by both miRNA sequencing and qRT-PCR. This may be due to the low expression of the target genes of the studied miRNA in root tissues.

Besides the known miRNAs, we also analyzed one of the novel miRNA candidates, miRNA16009. In leaf samples of *T. aestivum*, it was observed that the expression of novel miRNA16009 increased approximately 8.5-fold according to miRNAome and approximately fourfold according to qRT-PCR analysis after drought stress. miRNA sequencing and qRT-PCR analyses were found to be compatible with each other for miRNA16009. Depending on the increase in the expression of novel miRNA16009, it was observed that the expression of all three selected target genes decreased after the application of drought stress. The target genes encoded TRIAE_CS42_7AS_TGACv1_ 570299_AA1833530 and TRIAE_CS42_7DS_TGACv1_622788_AA2045350 were found to represent the Serine-glyoxylate aminotransferase (SGAT) enzyme and the TRIAE_CS42_3B_AA2045350 enzyme still represents an undetected gene as a result of the analysis. SGAT converts glyoxylate and serine to glycine and hydroxy pyruvate during photorespiration. In addition, SGAT also works with many other substrates, including asparagine (Modde et al. 2017). The study showed that SGAT activity causes surprisingly straightforward changes in metabolism and interferes with photosynthetic CO_2_ uptake and biomass accumulation in *Arabidopsis*. They also observed that faster serine turnover during photorespiration gradually decreased leaf serine content during the day, consequently stimulating the phosphoserine pathway. In our study, suppression of SGAT genes during drought stress can be considered an indicator that photosynthesis continues unaffected by drought stress. In addition, the decrease in these target genes'expression can be considered a tolerance mechanism against drought stress for *T. aestivum*. However, the situation mentioned above for *T. aestivum* is not the case for *T. monococcum*. After drought stress in leaf tissues of *T. monococcum*, it was observed that the expression level of novel miRNA16009 decreased as a result of both miRNA sequencing and quantitative PCR analysis. Accordingly, it was observed that the expression of one of the SGAT genes and the other target gene belonging to the Hsp90 family, encoded by TRIAE_CS42_7DS_TGACv1_622763_AA2044980, increased. This situation can be interpreted as an indication that photosynthesis is affected by drought stress due to the increase in the expression of SGAT genes in *T. monococcum* and the biomass decreases accordingly. In addition, the increase in the expression of one of the Hsp90 genes in *T. monococcum* as a result of drought stress can also be interpreted as a result of *T. monococcum* combating drought stress by slowing down metabolic events. It was observed that the expression level of novel miRNA16009 increased significantly after drought stress application in the leaf tissues of *T. turgidum*. As in *T. aestivum*, the decrease in the expression of one of the SGAT genes in the leaf tissues of *T. turgidum* after drought stress indicates that photosynthesis continues unaffected by drought stress. The responses of *T. aestivum* and *T. turgidum* to drought stress show similarities at miRNAome and transcriptome levels. In addition, the changes in the expression levels of novel miRNA16009 and its target genes in the root samples of *T. aestivum*, *T. monococcum*, and *T. turgidum* could not be determined by both miRNA sequencing and qRT-PCR. The low expression of the target genes of the studied miRNA in root tissues may explain it.

Polyploidy describes the presence of more than two complete sets of chromosomes in an organism. It has been reported that polyploidy causes various changes in genetic, epigenetic, transcriptional, and metabolic network levels (Lavania et al. 2012). Additionally, studies also show that polyploidy is advantageous in tolerating stress conditions. For example, tetraploid turnip (Meng et al. 2011), citrus (Saleh et al. 2008), and black locust (*Robinia pseudoacacia*) (Wang et al. 2013) have been reported to be more resistant to salt stress than their diploid ancestors. In addition, Indian chrysanthemum (Liu et al. 2011) have been shown to be more tolerant to salt and drought. However, the biological processes underlying these advantages of polyploidy in stress tolerance have not been revealed. A study using published RNASeq data from five different plant species in 2021 showed that microRNAs play a role in this mechanism (Esposito et al. 2021). In this study, differences were found between tetraploid and diploid individuals in the expression of pre-miRNAs targeting some genes that play a role in DNA metabolism, secondary metabolism, and stress tolerance (Esposito et al. 2021). In another study comparing the transcriptome data of *T. aestivum* and its ancestral genome donors and wild relatives, miRNAs and their target genes that are likely to play a role in biotic and abiotic stress resistance in *Aegilops sharonensis* were identified (Alptekin and Budak 2017). Those results provide insight into the origin of miRNAs involved in stress tolerance in bread wheat (Alptekin and Budak 2017). Further it is suggested that miR6180, and miR9653, expressed in bread wheat but not detected in ancestral species, may have arisen by selection of agricultural traits (Alptekin and Budak 2017). In this context, it can be thought that polyploidization may reveal new miRNAs. Lastly, it can be said that both the expression profiles of different transcription factor genes and biochemical analysis show that tetraploid and hexaploid wheat species have higher drought tolerance than diploid wheat according to our previous studies (Baloglu et al. 2014; Baloglu and Cetin 2020). As a result of bioinformatic analysis, differences in miRNA expression levels were detected among the species that were the subject of our study. In this respect, it can be said that polyploidy provides an advantage in drought tolerance. However, further studies are needed to confirm this hypothesis.

## Conclusions

Salt, drought, and very low–high temperatures, also known as abiotic stresses, adversely affect the production of wheat, the world's leading staple food. Recently, the role of miRNAs in plant growth and development under different abiotic stress conditions has attracted more attention from researchers. However, studies on miRNAs continue since the precise functions of most of them are not known enough. In this study, the differences in expression levels of miRNAs and target genes in leaf and root tissues after drought stress were investigated in different wheat species with A (*T. monococcum),* AB (*T. turgidum* ssp. durum), and ABD (*T. aestivum*) genomes. This study revealed that the different responses of miRNAs and their target genes to drought stress are the result of genotypic variation. Also, it was shown that modern wheat cultivars, *T. turgidum* ssp. durum and *T. aestivum*, are more resistant to drought stress than *T. monococcum*, which is known as the ancestor of wheat. The findings obtained might be a guide in future studies investigating the drought stress response.

## Supplementary Information

Below is the link to the electronic supplementary material.

Supplementary file1 (PNG 3968 KB)

Supplementary file2 (PNG 3292 KB)

Supplementary file3 (PNG 297 KB)

Supplementary file4 (PNG 1230 KB)

Supplementary file5 (JPG 1262 KB)

Supplementary file6 (JPG 1278 KB)

Supplementary file7 (PNG 332 KB)

Supplementary file8 (PNG 2772 KB)

Supplementary file9 (PNG 6046 KB)

Supplementary file10 (DOCX 17 KB)

Supplementary file11 (DOCX 16 KB)

Supplementary file12 (DOCX 16 KB)

Supplementary file13 (DOCX 15 KB)

Supplementary file14 (PDF 3230 KB)

Supplementary file15 (PDF 3178 KB)

Supplementary file16 (XLSX 223 KB)

Supplementary file17 (PNG 2759 KB)

Supplementary file18 (PNG 1916 KB)

## Data Availability

The data that support the findings will be available in NCBI SRA Database with Bioproject ID: PRJNA929049 (https://www.ncbi.nlm.nih.gov/bioproject/PRJNA929049/).
