# Supplementary material for: Genome-wide analysis of miRNAs and their target genes in wheat cultivars with different ploidy levels under drought stress
Source: Planta. 2025 Jul 1;262(2):38. doi: 10.1007/s00425-025-04757-3 (PMC12213836; doi:10.1007/s00425-025-04757-3)
Supplement: Supplementary file 1 — Supplementary file1 (PNG 3968 KB) [file 425_2025_4757_MOESM1_ESM.docx]

**Table S1:** Sequences of miRNA-specific forward primers and universal reverse primer, and sequences of forward and reverse primers of miRNA target genes.

|  | Primer Names | Primer Sequences (5’-3’) |
| --- | --- | --- |
| 1 | miR9664-3p-RT | GTCGTATCCAGTGCAGGGTCCGAGGTATTCGCACTGGATACGACCTACGA |
| 2 | miR9664-3P-F | GCGGCGTTGCAGTCCTCGATG |
| 3 | T9664_2044980-F | CGGCTACATGTCGAGCAAGA |
| 4 | T9664_2044980-R | GAAGGTGTTGGGGTCGTCAA |
| 5 | T9664_2045350-F | TTTCTGAGTACTGTCGCCCG |
| 6 | T9664_2045350-R | CTACTTGGACACTCAGGCGG |
| 7 | T9664_0276480-F | TTCCAGCTAACCAACGCAGG |
| 8 | T9664_0276480-R | GAACGGAGGGAGTACAAGCA |
| 9 | miR167c-RT | GTCGTATCCAGTGCAGGGTCCGAGGTATTCGCACTGGATACGACGCAGAT |
| 10 | miR167c-F | GCGGCGTGAAGCTGCCAGCATG |
| 11 | T167c_1530460-F | TCAAAGCAGGCACCCCATAC |
| 12 | T167c_1530460-R | TACGCAGAATGTCCAGCCAA |
| 13 | T167c_1555050-F | GAGTTCACGACGGAGGACG |
| 14 | T167c_1555050-R | GTAGCAACACCCCTTGGTCG |
| 15 | T167c_1894090-F | GCAAGGTTGGGGTCTCCTTT |
| 16 | T167c_1894090-R | TGCAAGGGTATGGACGAACT |
| 17 | miR9672a-3p-RT | GTCGTATCCAGTGCAGGGTCCGAGGTATTCGCACTGGATACGACGATGCT |
| 18 | miR9672a-3p-F | CGGCGGCCACGACTGTCATTA |
| 19 | T9672a_492770-F | CTGCTGGAGAACGTGTTTGC |
| 20 | T9672a_492770-R | ATCGACAAAACTAGGGGCGG |
| 21 | T9672a_1833530-F | ATGGTGCTCAAGGACATCGG |
| 22 | T9672a_1833530-R | TACTACGCCTGCATGCTCTG |
| 23 | T9672a_1944290-F | GAGCAAATCCAACGCACGAA |
| 24 | T9672a_1944290-R | AAAACGCCGCTCTCACTGTA |
| 25 | Novel_16009-RT | GTCGTATCCAGTGCAGGGTCCGAGGTATTCGCACTGGATACGACGGGAAG |
| 26 | Novel_16009-F | TCGCTTAATGCAACACGAGGA |
| 27 | miR5048-5p-F | GCGGCGGTTTGCAGGTTTTAGGT |
| 28 | miR5048-5p-RT | GTCGTATCCAGTGCAGGGTCCGAGGTATTCGCACTGGATACGACACTTAG |
| 29 | T5048_783320-F | GAGCGCGAGTTCATTTGTTATC |
| 30 | T5048_783320-R | CTGACGACAGTTCTCGACTATG |
| 31 | T5048_439840-F | AAGAGCCTGTGTGGTGTGTG |
| 32 | T5048_439840-R | AAAGGAGTTGGACGGCTCATC |
| 33 | T5048_1621120-F | TACTGCTGAACTTGCCGAGG |
| 34 | T5048_1621120-R | AGAGGAAGGCTGTGAAGTGC |
| 35 | miR156-RT | GTCGTATCCAGTGCAGGGTCCGAGGTATTCGCACTGGATACGACGTGCTC |
| 36 | miR156-F | GCGGCGGTGACAGAAGAGAGT |
| 37 | T156_1285110-F | CGTGACTCAGCGTGAGCAAA |
| 38 | T156_1285110-R | AAAACGCTCGTAGGGACTGG |
| 39 | T156_1396830-F | GCTGGCGTTAAAGGGTGAAC |
| 40 | T156_1396830-R | CATACTGCATAGCGGGAGGG |
| 41 | T156_1539460-F | GCCAGCCATTGCAAAAGGAC |
| 42 | T156_1539460-R | CCAGTCTGCTGTGCTCATGT |
| 43 | miR159a-RT | GTCGTATCCAGTGCAGGGTCCGAGGTATTCGCACTGGATACGACTAGAGC |
| 44 | miR159a-F | CGGCGGTTTGGATTGAAGGGA |
| 45 | T159a_AA_466-F | TCTACAATGGTGCAGGCAGG |
| 46 | T159a _AA_466-R | CTTGCAGGTTGGCTGAACAC |
| 47 | T159a _AA_6495-F | GGCGTACTCTCTCTTGCTGA |
| 48 | T159a _AA_6495-R | AGTACGAACCCAGCACACAA |
| 49 | T159a _AA_893720-F | CGTTACTGCTTTTCTGGCGT |
| 50 | T159a _AA_893720-R | CGAACCCAGCACACAAACAA |
| 51 | Uni-R | GTGCAGGGTCCGAGGT |
| 52 | 18s rRNA-F | TCAACTTTCGATGGTAGGATAGTG |
| 53 | 18s rRNA-R | CCGTGTCAGGATTGGGTAATTT |
| 54 | Β-Actin-F | TACTCCCTCACAACAACCGC |
| 55 | Β-Actin-R | CTCCTAGCCGTTTCCAGCTC |
| 56 | GAPDH-F | TTAGACTTGCGAAGCCAGCA |
| 57 | GAPDH-R | AAATGCCCTTGAGGTTTCCC |
