# Supplementary material for: Genome-wide analysis of miRNAs and their target genes in wheat cultivars with different ploidy levels under drought stress
Source: Planta. 2025 Jul 1;262(2):38. doi: 10.1007/s00425-025-04757-3 (PMC12213836; doi:10.1007/s00425-025-04757-3)
Supplement: Supplementary file 2 — Supplementary file2 (PNG 3292 KB) [file 425_2025_4757_MOESM2_ESM.docx]

**Table S2:** Internal control gene selection by using NormFinder and BestKeeper algoriths. (a) Mean Ct values of qRT-PCR analysis performed in 3 technical replicates with 8 biological samples. (b) Result of NormFinder algorithm. (c) Result of BestKeeper algorithm.

**a)**

|  | Sample 1 | Sample 2 | | Sample 3 | Sample 4 | Sample 5 | Sample 6 | Sample 7 | Sample 8 |
| --- | --- | --- | --- | --- | --- | --- | --- | --- | --- |
| *18s rRNA* | 32,78 | 33,00 | 33,90 | | 33,98 | 35,24 | 39,21 | 30,58 | 31,80 |
| *β-Actin* | 22,99 | 23,22 | 23,06 | | 20,63 | 20,18 | 19,78 | 22,06 | 22,16 |
| *GAPDH* | 20,27 | 20,42 | 20,23 | | 20,91 | 21,02 | 21,33 | 20,60 | 20,29 |

**b)**

| Gene name | Stability value | |  | | Best gene | GAPDH |
| --- | --- | --- | --- | --- | --- | --- |
| *18s rRNA* | 0,081 |  | |  | |  |
| *β-Actin* | 0,099 |  | |  | |  |
| *GAPDH* | **0,031** |  | |  | | **X** |

**c)**

|  | *18s rRNA* | *β-Actin* | *GAPDH* |
| --- | --- | --- | --- |
| n | 8 | 8 | 8 |
| geo Mean [CP] | 33,72720972 | 21,72127449 | 20,63022809 |
| ar Mean [CP] | 33,81125 | 21,76 | 20,63375 |
| min [CP] | 30,58 | 19,78 | 20,23 |
| max [CP] | 39,21 | 23,22 | 21,33 |
| std dev [± CP] | 1,77125 | 1,1725 | **0,3396875** |
| CV [% CP] | 5,238640985 | 5,388327206 | 1,646271279 |
