# Supplementary material for: Genome-wide analysis of miRNAs and their target genes in wheat cultivars with different ploidy levels under drought stress
Source: Planta. 2025 Jul 1;262(2):38. doi: 10.1007/s00425-025-04757-3 (PMC12213836; doi:10.1007/s00425-025-04757-3)
Supplement: Supplementary file 3 — Supplementary file3 (PNG 297 KB) [file 425_2025_4757_MOESM3_ESM.docx]

**Table S2:** Raw and clean reads number of 12 sRNA libraries. TaCLeaf: *Triticum aestivum* Control-Leaf, TaSLeaf: *Triticum aestivum* Stress-Leaf, TaCRoot: *Triticum aestivum* Control-Root, TaSRoot: *Triticum aestivum* Stress-Root, TmCLeaf: *Triticum monococcum* Control-Leaf, TmSLeaf: *Triticum monococcum* Stress-Leaf, TmCRoot: *Triticum monococcum* Control-Root, TmSRoot: *Triticum monococcum* Stress-Root, TtCLeaf: *Triticum turgidum* Control-Leaf*,* TtSLeaf: *Triticum turgidum* Stress-Leaf*,* TtCRoot: *Triticum turgidum* Control-Root*,* TtSRoot: *Triticum turgidum* Stress-Root.

|  | Raw Read | Clean Read | (%) Percentage | Mapped Read | (%) Percentage | miRNA Read | (%) Percentage |
| --- | --- | --- | --- | --- | --- | --- | --- |
| TaCLeaf | 26.965.687 | 23.544.441 | 87.31 | 20.680.695 | 87.84 | 390.550 | 0.019 |
| TaCRoot | 32.716.109 | 31.452.275 | 96.14 | 24.894.077 | 79.15 | 339.833 | 0.014 |
| TaSLeaf | 29.810.353 | 28.122.741 | 94.34 | 24.818.118 | 88.25 | 513.413 | 0.021 |
| TaSRoot | 34.143.091 | 31.834.021 | 93.24 | 25.264.117 | 79.36 | 344.204 | 0.014 |
| TmCLeaf | 28.979.797 | 27.102.904 | 93.52 | 24.999.153 | 92.24 | 389.047 | 0.016 |
| TmCRoot | 30.037.262 | 28.656.659 | 95.40 | 22.892.237 | 79.88 | 368.995 | 0.016 |
| TmSLeaf | 18.140.201 | 16.280.114 | 89.75 | 14.862.873 | 91.29 | 223.443 | 0.015 |
| TmSRoot | 27.983.453 | 26.359.594 | 94.20 | 19.147.336 | 72.64 | 287.079 | 0.015 |
| TtCLeaf | 23.861.857 | 22.172.587 | 92.92 | 19.717.814 | 88.93 | 278.125 | 0.014 |
| TtCRoot | 35.073.803 | 33.765.686 | 96.27 | 27.337.728 | 80.96 | 342.739 | 0.012 |
| TtSLeaf | 26.030.274 | 24.823.650 | 95.36 | 22.030.127 | 88.75 | 387.260 | 0.018 |
| TtSRoot | 32.261.077 | 30.434.530 | 94.34 | 24.349.786 | 80.01 | 486.192 | 0.020 |
