# Supplementary material for: Genome-wide analysis of miRNAs and their target genes in wheat cultivars with different ploidy levels under drought stress
Source: Planta. 2025 Jul 1;262(2):38. doi: 10.1007/s00425-025-04757-3 (PMC12213836; doi:10.1007/s00425-025-04757-3)
Supplement: Supplementary file 4 — Supplementary file4 (PNG 1230 KB) [file 425_2025_4757_MOESM4_ESM.docx]

**Table S3**: Sequences of established novel miRNAs.

| Id | Location | Maximum Free Energy | Count |
| --- | --- | --- | --- |
| Triticum_aestivum-m0094 | scaffold174714:21833:21943:- | -60.5 | 4614 |
| Triticum_aestivum-m0039 | scaffold117787:15737:15895:+ | -39.4 | 4005 |
| Triticum_aestivum-m0129 | scaffold24740:28445:28770:+ | -74.3 | 2470 |
| Triticum_aestivum-m0179 | scaffold35790:58054:58283:- | -108.1 | 2449 |
| Triticum_aestivum-m0198 | scaffold41725:26899:27004:+ | -62.1 | 2253 |
| Triticum_aestivum-m0061 | scaffold13621:86260:86345:+ | -51.3 | 1655 |
| Triticum_aestivum-m0322 | scaffold82190:27881:28034:- | -60.6 | 1261 |
| Triticum_aestivum-m0070 | scaffold145762:14177:14271:+ | -38.7 | 1238 |
| Triticum_aestivum-m0195 | scaffold40305:137896:138092:+ | -99.2 | 566 |
| Triticum_aestivum-m0135 | scaffold25946:6700:6903:+ | -89.9 | 422 |
| Triticum_aestivum-m0186 | scaffold38344:24325:24416:+ | -61.6 | 420 |
| Triticum_aestivum-m0217 | scaffold45816:118314:118430:+ | -57.1 | 400 |
| Triticum_aestivum-m0313 | scaffold8029:13219:13345:- | -60.6 | 386 |
| Triticum_aestivum-m0192 | scaffold39942:33886:33982:+ | -46.5 | 379 |
| Triticum_aestivum-m0326 | scaffold8253:58055:58191:+ | -65.7 | 365 |
| Triticum_aestivum-m0285 | scaffold65338:17034:17124:+ | -48.5 | 334 |
| Triticum_aestivum-m0218 | scaffold46052:17541:17640:- | -54.1 | 330 |
| Triticum_aestivum-m0162 | scaffold31301:80387:80730:- | -106.3 | 328 |
| Triticum_aestivum-m0287 | scaffold65642:17799:17882:+ | -49.3 | 313 |
| Triticum_aestivum-m0314 | scaffold8083:12431:12550:- | -29.63 | 265 |
