# Supplementary material for: Genome-wide analysis of miRNAs and their target genes in wheat cultivars with different ploidy levels under drought stress
Source: Planta. 2025 Jul 1;262(2):38. doi: 10.1007/s00425-025-04757-3 (PMC12213836; doi:10.1007/s00425-025-04757-3)
Supplement: Supplementary file 6 — Supplementary file6 (JPG 1278 KB) [file 425_2025_4757_MOESM6_ESM.pdf]

Table S5: Annotation of miRNA target genes that discovered by using psRNATarget database in control and stress-treated root samples of all cultivars.

							Triticum aestivum_Control_Root												
miRNA_Acc.	Target_Acc.	Pfam	KOG	KEGG/ec	KO	GO	Best-hit-arabi-name	arabi-defline	Best-hit-rice-name	rice-defline	Expectation	UPE$	miRNA_start	miRNA_end	Target_start	Target_end	miRNA_aligned_fragment	Target_aligned_fragment	Inhibition
tae-miR159a	Traes_1AL_041EBB4A6.1						AT2G32460.1	myb domain protein 101	LOC_Os01g59660.2	MYB family transcription factor, putative, expressed	2,50	-1.0	1	21	488	508	UUUGGAUUGAAGGGAGCUCUG	UGGAGCUCCCUUCAUUCCAAU	Cleavage
tae-miR159b	Traes_1AL_041EBB4A6.1						AT2G32460.1	myb domain protein 101	LOC_Os01g59660.2	MYB family transcription factor, putative, expressed	2,50	-1.0	1	21	488	508	UUUGGAUUGAAGGGAGCUCUG	UGGAGCUCCCUUCAUUCCAAU	Cleavage
tae-miR159a	Traes_1AL_041EBB4A6.2						AT2G32460.1	myb domain protein 101	LOC_Os01g59660.2	MYB family transcription factor, putative, expressed	2,50	-1.0	1	21	512	532	UUUGGAUUGAAGGGAGCUCUG	UGGAGCUCCCUUCAUUCCAAU	Cleavage
tae-miR159b	Traes_1AL_041EBB4A6.2						AT2G32460.1	myb domain protein 101	LOC_Os01g59660.2	MYB family transcription factor, putative, expressed	2,50	-1.0	1	21	512	532	UUUGGAUUGAAGGGAGCUCUG	UGGAGCUCCCUUCAUUCCAAU	Cleavage
tae-miR159a	Traes_1AL_041EBB4A6.3						AT2G32460.1	myb domain protein 101	LOC_Os01g59660.2	MYB family transcription factor, putative, expressed	2,50	-1.0	1	21	488	508	UUUGGAUUGAAGGGAGCUCUG	UGGAGCUCCCUUCAUUCCAAU	Cleavage
tae-miR159b	Traes_1AL_041EBB4A6.3						AT2G32460.1	myb domain protein 101	LOC_Os01g59660.2	MYB family transcription factor, putative, expressed	2,50	-1.0	1	21	488	508	UUUGGAUUGAAGGGAGCUCUG	UGGAGCUCCCUUCAUUCCAAU	Cleavage
tae-miR159a	Traes_1AL_041EBB4A6.4								LOC_Os01g59660.2	MYB family transcription factor, putative, expressed	2,50	-1.0	1	21	410	430	UUUGGAUUGAAGGGAGCUCUG	UGGAGCUCCCUUCAUUCCAAU	Cleavage
tae-miR159b	Traes_1AL_041EBB4A6.4								LOC_Os01g59660.2	MYB family transcription factor, putative, expressed	2,50	-1.0	1	21	410	430	UUUGGAUUGAAGGGAGCUCUG	UGGAGCUCCCUUCAUUCCAAU	Cleavage
tae-miR160	Traes_1AL_147CF243C.1	PF06507				GO:GO:0009725,GO:GO:0006355,GO:GO:0005634,GO:GO:0003677	AT4G30080.1	auxin response factor 16	LOC_Os10g33940.1	auxin response factor 18, putative, expressed	0,00	-1.0	1	21	642	662	UGCCUGGCUCCCUGUAUGCCA	AGGCAUACAGGGAGCCAGGCA	Cleavage
tae-miR160	Traes_1AL_147CF243C.2						AT4G30080.1	auxin response factor 16	LOC_Os10g33940.1	auxin response factor 18, putative, expressed	0,00	-1.0	1	21	267	287	UGCCUGGCUCCCUGUAUGCCA	AGGCAUACAGGGAGCCAGGCA	Cleavage
tae-miR1127b-3p	Traes_1AL_4A458DF14.2	PF13812,PF01535,PF13041					AT3G62470.1	Pentatricopeptide repeat (PPR) superfamily protein	LOC_Os05g28720.1	PPR repeat containing protein, expressed	1,50	-1.0	1	21	1738	1758	ACAAGUAUUUCUGGACGGAGG	CCUCCGUCCGGAAAUACUUGU	Cleavage
tae-miR1130b-3p	Traes_1AL_51B1D5DBC.6	PF07714		2.7.11.1		GO:GO:0006468,GO:GO:0004672	AT5G14640.1	shaggy-like kinase 13	LOC_Os10g37740.1	CGMC_GSK.9 - CGMC includes CDA, MAPK, GSK3, and CLKC kinases, expressed	0,00	-1.0	1	21	914	934	UCUUAUAUUAUGGGACGGAGG	CCUCCGUCCCAUAAUAUAAGA	Cleavage
tae-miR1122c-3p	Traes_1AL_51B1D5DBC.6	PF07714		2.7.11.1		GO:GO:0006468,GO:GO:0004672	AT5G14640.1	shaggy-like kinase 13	LOC_Os10g37740.1	CGMC_GSK.9 - CGMC includes CDA, MAPK, GSK3, and CLKC kinases, expressed	1,50	-1.0	1	21	914	934	UCUAAUAUUAUGGGACGGAGG	CCUCCGUCCCAUAAUAUAAGA	Cleavage
tae-miR5049-3p	Traes_1AL_7D6DC73FC.3	PF14531	KOG0583	2.7.11.1			AT3G01090.2	SNF1 kinase homolog 10	LOC_Os05g45420.2	CAMK_KIN1/SNF1/Nim1_like_AMPKh.3 - CAMK includes calcium/calmodulin depedent protein kinases, expressed	2,00	-1.0	1	21	1256	1276	AAUAUGGAUCGGAGGGAGUAC	UUUCUCCCUCCGAUCCAUAUA	Cleavage
tae-miR1128	Traes_1AL_968B97E50.2	PF06480,PF00004,PF01434			K08956	GO:GO:0016021,GO:GO:0008270,GO:GO:0005524,GO:GO:0004222,GO:GO:0006508	AT1G07510.1	FTSH protease 10	LOC_Os05g38400.3	OsFtsH8 FtsH protease, homologue of AtFtsH3/10, expressed	3,00	-1.0	1	21	100	121	UACUACUCCCUCCGUCC-GAAA	UUUCCGGACGGAGGGAGUAGUU	Cleavage
tae-miR7757-5p	Traes_1AL_B6931117F.1	PF00931				GO:GO:0043531	AT3G46530.1	NB-ARC domain-containing disease resistance protein	LOC_Os08g10440.1	NBS-LRR disease resistance protein, putative, expressed	1,50	-1.0	1	22	1301	1322	AUAAAACCUUCAGCUAUCCAUC	AAUGGAUAGCUGAAGGCUUUAU	Cleavage
tae-miR7757-5p	Traes_1AL_B6931117F.2	PF00931				GO:GO:0043531	AT3G46530.1	NB-ARC domain-containing disease resistance protein	LOC_Os08g10440.1	NBS-LRR disease resistance protein, putative, expressed	1,50	-1.0	1	22	1343	1364	AUAAAACCUUCAGCUAUCCAUC	AAUGGAUAGCUGAAGGCUUUAU	Cleavage
tae-miR7757-5p	Traes_1AL_B6931117F.3	PF00931				GO:GO:0043531	AT3G46530.1	NB-ARC domain-containing disease resistance protein	LOC_Os08g10440.1	NBS-LRR disease resistance protein, putative, expressed	1,50	-1.0	1	22	1301	1322	AUAAAACCUUCAGCUAUCCAUC	AAUGGAUAGCUGAAGGCUUUAU	Cleavage
tae-miR7757-5p	Traes_1AL_B6931117F.4	PF00931				GO:GO:0043531	AT3G46530.1	NB-ARC domain-containing disease resistance protein	LOC_Os08g10440.1	NBS-LRR disease resistance protein, putative, expressed	1,50	-1.0	1	22	1301	1322	AUAAAACCUUCAGCUAUCCAUC	AAUGGAUAGCUGAAGGCUUUAU	Cleavage
tae-miR9778	Traes_1AS_17306388F.1	PF00931				GO:GO:0043531	AT3G07040.1	NB-ARC domain-containing disease resistance protein	LOC_Os08g42670.2	resistance protein, putative, expressed	2,00	-1.0	1	21	126	146	UGCAUCAUCUCGAACUCGUCG	CAAUGAGCUCGAGAUGAUGCA	Cleavage
tae-miR9778	Traes_1AS_17306388F.3	PF00931				GO:GO:0043531	AT3G46530.1	NB-ARC domain-containing disease resistance protein	LOC_Os12g37290.1	resistance protein T10rga2-1A, putative, expressed	2,00	-1.0	1	21	126	146	UGCAUCAUCUCGAACUCGUCG	CAAUGAGCUCGAGAUGAUGCA	Cleavage
tae-miR1135	Traes_1AS_4A925522B.4	PF13920					AT5G01960.1	RING/U-box superfamily protein	LOC_Os01g07390.1	zinc finger, C3HC4 type family protein, expressed	0,00	-1.0	1	24	511	534	CUGCGACAAGUAAUUCCGAACGGA	UCCGUUCGGAAUUACUUGUCGCAG	Cleavage
tae-miR6197-5p	Traes_1AS_5DCFDC258.1	PF04578,PF13968					AT5G45540.1	Protein of unknown function (DUF594)	LOC_Os07g08270.1	expressed protein	3,00	-1.0	1	21	1353	1376	UCUGUAAACAAAUGUA---GGACG	ACUCCAACUACAUUUGUUUACAGA	Cleavage
tae-miR1137a	Traes_1AS_8912BD052.27										0,00	-1.0	1	20	136	155	UAGUACAAAGUUGAGUCAUC	GAUGACUCAACUUUGUACUA	Cleavage
tae-miR9674a-5p	Traes_1AS_92A698B19.1	PF01535,PF13041			K17964		AT1G63130.1	Tetratricopeptide repeat (TPR)-like superfamily protein	LOC_Os04g28300.1	Rf1, mitochondrial precursor, putative, expressed	2,50	-1.0	1	21	1761	1781	GCAUCAUCCAUCCUACCAUUC	AAAUGGAAGGAUCGAUGAUGC	Cleavage
tae-miR9674a-5p	Traes_1AS_92A698B19.10	PF01535,PF13041					AT1G63130.1	Tetratricopeptide repeat (TPR)-like superfamily protein	LOC_Os04g28300.1	Rf1, mitochondrial precursor, putative, expressed	2,50	-1.0	1	21	1761	1781	GCAUCAUCCAUCCUACCAUUC	AAAUGGAAGGAUCGAUGAUGC	Cleavage
tae-miR9674a-5p	Traes_1AS_92A698B19.12	PF13041					AT1G12300.1	Tetratricopeptide repeat (TPR)-like superfamily protein	LOC_Os04g28300.1	Rf1, mitochondrial precursor, putative, expressed	2,50	-1.0	1	21	96	116	GCAUCAUCCAUCCUACCAUUC	AAAUGGAAGGAUCGAUGAUGC	Cleavage
tae-miR9674a-5p	Traes_1AS_92A698B19.2	PF01535,PF13041			K17964		AT1G63130.1	Tetratricopeptide repeat (TPR)-like superfamily protein	LOC_Os04g28300.1	Rf1, mitochondrial precursor, putative, expressed	2,50	-1.0	1	21	1761	1781	GCAUCAUCCAUCCUACCAUUC	AAAUGGAAGGAUCGAUGAUGC	Cleavage
tae-miR9674a-5p	Traes_1AS_92A698B19.3	PF01535,PF13041			K17964		AT1G63130.1	Tetratricopeptide repeat (TPR)-like superfamily protein	LOC_Os04g28300.1	Rf1, mitochondrial precursor, putative, expressed	2,50	-1.0	1	21	1761	1781	GCAUCAUCCAUCCUACCAUUC	AAAUGGAAGGAUCGAUGAUGC	Cleavage
tae-miR9674a-5p	Traes_1AS_92A698B19.4	PF01535,PF13041			K17964		AT1G63130.1	Tetratricopeptide repeat (TPR)-like superfamily protein	LOC_Os04g28300.1	Rf1, mitochondrial precursor, putative, expressed	2,50	-1.0	1	21	1761	1781	GCAUCAUCCAUCCUACCAUUC	AAAUGGAAGGAUCGAUGAUGC	Cleavage
tae-miR9674a-5p	Traes_1AS_92A698B19.5	PF01535,PF13041			K17964		AT1G63130.1	Tetratricopeptide repeat (TPR)-like superfamily protein	LOC_Os04g28300.1	Rf1, mitochondrial precursor, putative, expressed	2,50	-1.0	1	21	1761	1781	GCAUCAUCCAUCCUACCAUUC	AAAUGGAAGGAUCGAUGAUGC	Cleavage
tae-miR9674a-5p	Traes_1AS_92A698B19.6	PF01535,PF13041			K17964		AT1G63130.1	Tetratricopeptide repeat (TPR)-like superfamily protein	LOC_Os04g28300.1	Rf1, mitochondrial precursor, putative, expressed	2,50	-1.0	1	21	1761	1781	GCAUCAUCCAUCCUACCAUUC	AAAUGGAAGGAUCGAUGAUGC	Cleavage
tae-miR9674a-5p	Traes_1AS_92A698B19.7	PF01535,PF13041			K17964		AT1G63130.1	Tetratricopeptide repeat (TPR)-like superfamily protein	LOC_Os04g28300.1	Rf1, mitochondrial precursor, putative, expressed	2,50	-1.0	1	21	1761	1781	GCAUCAUCCAUCCUACCAUUC	AAAUGGAAGGAUCGAUGAUGC	Cleavage
tae-miR9674a-5p	Traes_1AS_92A698B19.8	PF01535,PF13041			K17964		AT1G63130.1	Tetratricopeptide repeat (TPR)-like superfamily protein	LOC_Os04g28300.1	Rf1, mitochondrial precursor, putative, expressed	2,50	-1.0	1	21	1761	1781	GCAUCAUCCAUCCUACCAUUC	AAAUGGAAGGAUCGAUGAUGC	Cleavage
tae-miR9674a-5p	Traes_1AS_92A698B19.9	PF01535,PF13041			K17964		AT1G63130.1	Tetratricopeptide repeat (TPR)-like superfamily protein	LOC_Os04g28300.1	Rf1, mitochondrial precursor, putative, expressed	2,50	-1.0	1	21	1761	1781	GCAUCAUCCAUCCUACCAUUC	AAAUGGAAGGAUCGAUGAUGC	Cleavage
tae-miR5049-3p	Traes_1AS_A482D4FF9.1	PF04997		2.7.7.6		GO:GO:0006351,GO:GO:0003899,GO:GO:0003677	AT1G63020.1	nuclear RNA polymerase D1A	LOC_Os02g05880.1	RNA polymerase Rpb1, domain 2 family protein, expressed	0,00	-1.0	1	21	557	577	AAUAUGGAUCGGAGGGAGUAC	GUACUCCCUCCGAUCCAUAUU	Cleavage
tae-miR9778	Traes_1AS_C7A8188D1.1	PF13191,PF00931	KOG4658			GO:GO:0043531	AT1G58390.1	Disease resistance protein (CC-NBS-LRR class) family	LOC_Os08g42670.2	resistance protein, putative, expressed	1,00	-1.0	1	21	126	146	UGCAUCAUCUCGAACUCGUCG	CAACGAGCUCGAGAUGAUGCA	Cleavage
tae-miR9778	Traes_1AS_C7A8188D1.3	PF00931				GO:GO:0043531	AT3G46530.1	NB-ARC domain-containing disease resistance protein	LOC_Os02g57310.1	pib, putative, expressed	1,00	-1.0	1	21	126	146	UGCAUCAUCUCGAACUCGUCG	CAACGAGCUCGAGAUGAUGCA	Cleavage
tae-miR9661-5p	Traes_1AS_D8E37233E.1										2,00	-1.0	1	21	101	121	UGAAGUAGAGCAGGGACCUCA	CCAAGUCCCUGCUCUACUUCG	Cleavage
tae-miR1128	Traes_1BL_087CA0963.1	PF16876,PF08235	KOG2116	3.1.3.4			AT3G09560.1	Lipin family protein	LOC_Os05g38710.1	lipin, N-terminal conserved region family protein, expressed	3,00	-1.0	1	21	2048	2069	UACUACUCCCUCCG-UCCGAAA	UUUAGGAACGGAGGGAGUAGUA	Cleavage
tae-miR1128	Traes_1BL_0FDC38712.1	PF13639				GO:GO:0008270,GO:GO:0005515	AT1G17970.1	RING/U-box superfamily protein	LOC_Os05g47670.1	zinc finger, C3HC4 type domain containing protein, expressed	1,50	-1.0	1	21	225	245	UACUACUCCCUCCGUCCGAAA	UUUCGGACGGAGGGAGUACUA	Cleavage
tae-miR1127b-3p	Traes_1BL_0FDC38712.1	PF13639				GO:GO:0008270,GO:GO:0005515	AT1G17970.1	RING/U-box superfamily protein	LOC_Os05g47670.1	zinc finger, C3HC4 type domain containing protein, expressed	1,50	-1.0	1	21	121	141	ACAAGUAUUUCUGGACGGAGG	CCUCCGUCCGGAAAUACUUGU	Cleavage
tae-miR5062-5p	Traes_1BL_219B2BB6D.16						AT2G46060.1	transmembrane protein-related	LOC_Os10g30910.2	transmembrane protein-related, putative, expressed	2,50	-1.0	1	23	836	858	UGAACCUUAGGGAACAGCCGCAU	UUUUGGAUGUUCCCCAAGGUUCA	Cleavage
tae-miR5062-5p	Traes_1BL_219B2BB6D.17						AT2G46060.1	transmembrane protein-related	LOC_Os10g30910.2	transmembrane protein-related, putative, expressed	2,50	-1.0	1	23	836	858	UGAACCUUAGGGAACAGCCGCAU	UUUUGGAUGUUCCCCAAGGUUCA	Cleavage
tae-miR5062-5p	Traes_1BL_219B2BB6D.18						AT2G46060.1	transmembrane protein-related	LOC_Os10g30910.2	transmembrane protein-related, putative, expressed	2,50	-1.0	1	23	836	858	UGAACCUUAGGGAACAGCCGCAU	UUUUGGAUGUUCCCCAAGGUUCA	Cleavage
tae-miR160	Traes_1BL_54CD82AC3.1	PF06507				GO:GO:0009725,GO:GO:0006355,GO:GO:0005634,GO:GO:0003677	AT4G30080.1	auxin response factor 16	LOC_Os10g33940.1	auxin response factor 18, putative, expressed	0,00	-1.0	1	21	642	662	UGCCUGGCUCCCUGUAUGCCA	AGGCAUACAGGGAGCCAGGCA	Cleavage
tae-miR160	Traes_1BL_54CD82AC3.2						AT4G30080.1	auxin response factor 16	LOC_Os10g33940.1	auxin response factor 18, putative, expressed	0,00	-1.0	1	21	369	389	UGCCUGGCUCCCUGUAUGCCA	AGGCAUACAGGGAGCCAGGCA	Cleavage
tae-miR160	Traes_1BL_54CD82AC3.3						AT4G30080.1	auxin response factor 16	LOC_Os10g33940.1	auxin response factor 18, putative, expressed	0,00	-1.0	1	21	267	287	UGCCUGGCUCCCUGUAUGCCA	AGGCAUACAGGGAGCCAGGCA	Cleavage
tae-miR1130b-3p	Traes_1BL_89E785AAA.2	PF00436	KOG1653			GO:GO:0003697,GO:GO:0006260	AT4G11060.1	mitochondrially targeted single-stranded DNA binding protein	LOC_Os05g43440.2	single-stranded DNA-binding protein, putative, expressed	2,00	-1.0	1	21	591	611	UCUUAUAUUAUGGGACGGAGG	CCUCUGUCCCAUAAUAUAAGG	Cleavage
tae-miR159a	Traes_1BL_CF98E922B.1	PF00249					AT5G06100.2	myb domain protein 33	LOC_Os01g59660.2	MYB family transcription factor, putative, expressed	2,50	-1.0	1	21	839	859	UUUGGAUUGAAGGGAGCUCUG	UGGAGCUCCCUUCACUCCAAU	Cleavage
tae-miR159b	Traes_1BL_CF98E922B.1	PF00249					AT5G06100.2	myb domain protein 33	LOC_Os01g59660.2	MYB family transcription factor, putative, expressed	2,50	-1.0	1	21	839	859	UUUGGAUUGAAGGGAGCUCUG	UGGAGCUCCCUUCACUCCAAU	Cleavage
tae-miR159a	Traes_1BL_CF98E922B.2	PF00249					AT5G06100.3	myb domain protein 33	LOC_Os01g59660.1	MYB family transcription factor, putative, expressed	2,50	-1.0	1	21	839	859	UUUGGAUUGAAGGGAGCUCUG	UGGAGCUCCCUUCACUCCAAU	Cleavage
tae-miR159b	Traes_1BL_CF98E922B.2	PF00249					AT5G06100.3	myb domain protein 33	LOC_Os01g59660.1	MYB family transcription factor, putative, expressed	2,50	-1.0	1	21	839	859	UUUGGAUUGAAGGGAGCUCUG	UGGAGCUCCCUUCACUCCAAU	Cleavage
tae-miR159a	Traes_1BL_CF98E922B.3						AT5G06100.3	myb domain protein 33	LOC_Os01g59660.2	MYB family transcription factor, putative, expressed	2,50	-1.0	1	21	512	532	UUUGGAUUGAAGGGAGCUCUG	UGGAGCUCCCUUCACUCCAAU	Cleavage
tae-miR159b	Traes_1BL_CF98E922B.3						AT5G06100.3	myb domain protein 33	LOC_Os01g59660.2	MYB family transcription factor, putative, expressed	2,50	-1.0	1	21	512	532	UUUGGAUUGAAGGGAGCUCUG	UGGAGCUCCCUUCACUCCAAU	Cleavage
tae-miR159a	Traes_1BL_CF98E922B.4						AT5G06100.1	myb domain protein 33	LOC_Os01g59660.1	MYB family transcription factor, putative, expressed	2,50	-1.0	1	21	512	532	UUUGGAUUGAAGGGAGCUCUG	UGGAGCUCCCUUCACUCCAAU	Cleavage
tae-miR159b	Traes_1BL_CF98E922B.4						AT5G06100.1	myb domain protein 33	LOC_Os01g59660.1	MYB family transcription factor, putative, expressed	2,50	-1.0	1	21	512	532	UUUGGAUUGAAGGGAGCUCUG	UGGAGCUCCCUUCACUCCAAU	Cleavage
tae-miR1117	Traes_1BL_F01A87F44.1	PF13359					AT2G13770.1		LOC_Os01g33990.1	transposon protein, putative, CACTA, En/Spm sub-class, expressed	2,50	-1.0	1	24	37	60	UAGUACCGGUUCGUGGCACGAACC	GGUUCGUGGCACGAACCGGUGCUA	Cleavage
tae-miR1131	Traes_1BL_F01A87F44.1	PF13359					AT2G13770.1		LOC_Os01g33990.1	transposon protein, putative, CACTA, En/Spm sub-class, expressed	2,50	-1.0	1	22	63	84	UAGUACCGGUUCGUGGCUAACC	GGUUAGCCACGAACCGGUAAUG	Cleavage
tae-miR1122b-3p	Traes_1BS_34C60A54E.1	PF00651				GO:GO:0005515	AT5G19000.1	BTB-POZ and MATH domain 1	LOC_Os08g41120.1	MBTB29 - Bric-a-Brac, Tramtrack, Broad Complex BTB domain with Meprin and TRAF Homology MATH domain, expressed	1,50	-1.0	1	21	47	67	AGACUUAUAUGUAGGAACGGA	UCCGUUCCUAAAUAUAAGUCU	Translation
tae-miR9662a-3p	Traes_1BS_566CEE7F6.1	PF02536				GO:GO:0006355,GO:GO:0005739,GO:GO:0003690	AT1G21150.1	Mitochondrial transcription termination factor family protein	LOC_Os06g12100.1	mTERF family protein, expressed	2,00	-1.0	1	21	441	461	UUGAACAUCCCAGAGCCACCG	UCGUGGCGCUGGGAUGUUCAG	Cleavage
tae-miR9662b-3p	Traes_1BS_566CEE7F6.1	PF02536				GO:GO:0006355,GO:GO:0005739,GO:GO:0003690	AT1G21150.1	Mitochondrial transcription termination factor family protein	LOC_Os06g12100.1	mTERF family protein, expressed	2,50	-1.0	1	21	440	460	UGAACAUCCCAGAGCCACCGG	CUCGUGGCGCUGGGAUGUUCA	Cleavage
tae-miR9662a-3p	Traes_1BS_566CEE7F6.2	PF02536				GO:GO:0006355,GO:GO:0005739,GO:GO:0003690	AT1G21150.1	Mitochondrial transcription termination factor family protein	LOC_Os06g12100.1	mTERF family protein, expressed	2,00	-1.0	1	21	66	86	UUGAACAUCCCAGAGCCACCG	UCGUGGCGCUGGGAUGUUCAG	Cleavage
tae-miR9662b-3p	Traes_1BS_566CEE7F6.2	PF02536				GO:GO:0006355,GO:GO:0005739,GO:GO:0003690	AT1G21150.1	Mitochondrial transcription termination factor family protein	LOC_Os06g12100.1	mTERF family protein, expressed	2,50	-1.0	1	21	65	85	UGAACAUCCCAGAGCCACCGG	CUCGUGGCGCUGGGAUGUUCA	Cleavage
tae-miR9778	Traes_1BS_70B733D34.1	PF13191,PF00931				GO:GO:0043531	AT3G07040.1	NB-ARC domain-containing disease resistance protein	LOC_Os08g42670.1	resistance protein, putative, expressed	2,00	-1.0	1	21	126	146	UGCAUCAUCUCGAACUCGUCG	CAAUGAGCUCGAGAUGAUGCA	Cleavage
tae-miR9778	Traes_1BS_70B733D34.2	PF13191,PF00931				GO:GO:0043531	AT3G07040.1	NB-ARC domain-containing disease resistance protein	LOC_Os08g42670.2	resistance protein, putative, expressed	2,00	-1.0	1	21	126	146	UGCAUCAUCUCGAACUCGUCG	CAAUGAGCUCGAGAUGAUGCA	Cleavage
tae-miR5049-3p	Traes_1BS_7EC2BDE95.1	PF16760		2.4.1.21			AT1G11720.2	starch synthase 3	LOC_Os08g09230.1	starch synthase III, putative, expressed	1,50	-1.0	1	21	3430	3450	AAUAUGGAUCGGAGGGAGUAC	CUACUCCCUCCAAUCCAUAUU	Translation
tae-miR5049-3p	Traes_1BS_B0BB95B97.6	PF04997		2.7.7.6		GO:GO:0006351,GO:GO:0003899,GO:GO:0003677	AT1G63020.1	nuclear RNA polymerase D1A	LOC_Os02g05880.1	RNA polymerase Rpb1, domain 2 family protein, expressed	0,00	-1.0	1	21	623	643	AAUAUGGAUCGGAGGGAGUAC	GUACUCCCUCCGAUCCAUAUU	Cleavage
tae-miR7757-5p	Traes_1BS_CF1C4F54E.2	PF00931				GO:GO:0043531	AT1G09920.1	TRAF-type zinc finger-related	LOC_Os11g45750.1	WRKY125, expressed	2,50	-1.0	1	22	119	140	AUAAAACCUUCAGCUAUCCAUC	AAUGGUUAGCUGAAGGUUUCAU	Cleavage
tae-miR159a	Traes_1DL_25540DE53.1						AT2G32460.1	myb domain protein 101	LOC_Os01g59660.2	MYB family transcription factor, putative, expressed	2,50	-1.0	1	21	512	532	UUUGGAUUGAAGGGAGCUCUG	UGGAGCUCCCUUCAUUCCAAU	Cleavage
tae-miR159b	Traes_1DL_25540DE53.1						AT2G32460.1	myb domain protein 101	LOC_Os01g59660.2	MYB family transcription factor, putative, expressed	2,50	-1.0	1	21	512	532	UUUGGAUUGAAGGGAGCUCUG	UGGAGCUCCCUUCAUUCCAAU	Cleavage
tae-miR159a	Traes_1DL_25540DE53.2						AT2G32460.1	myb domain protein 101	LOC_Os01g59660.1	MYB family transcription factor, putative, expressed	2,50	-1.0	1	21	512	532	UUUGGAUUGAAGGGAGCUCUG	UGGAGCUCCCUUCAUUCCAAU	Cleavage
tae-miR159b	Traes_1DL_25540DE53.2						AT2G32460.1	myb domain protein 101	LOC_Os01g59660.1	MYB family transcription factor, putative, expressed	2,50	-1.0	1	21	512	532	UUUGGAUUGAAGGGAGCUCUG	UGGAGCUCCCUUCAUUCCAAU	Cleavage
tae-miR159a	Traes_1DL_25540DE53.3						AT5G06100.3	myb domain protein 33	LOC_Os01g59660.1	MYB family transcription factor, putative, expressed	2,50	-1.0	1	21	470	490	UUUGGAUUGAAGGGAGCUCUG	UGGAGCUCCCUUCAUUCCAAU	Cleavage
tae-miR159b	Traes_1DL_25540DE53.3						AT5G06100.3	myb domain protein 33	LOC_Os01g59660.1	MYB family transcription factor, putative, expressed	2,50	-1.0	1	21	470	490	UUUGGAUUGAAGGGAGCUCUG	UGGAGCUCCCUUCAUUCCAAU	Cleavage
tae-miR159a	Traes_1DL_25540DE53.4						AT5G06100.3	myb domain protein 33	LOC_Os01g59660.2	MYB family transcription factor, putative, expressed	2,50	-1.0	1	21	470	490	UUUGGAUUGAAGGGAGCUCUG	UGGAGCUCCCUUCAUUCCAAU	Cleavage
tae-miR159b	Traes_1DL_25540DE53.4						AT5G06100.3	myb domain protein 33	LOC_Os01g59660.2	MYB family transcription factor, putative, expressed	2,50	-1.0	1	21	470	490	UUUGGAUUGAAGGGAGCUCUG	UGGAGCUCCCUUCAUUCCAAU	Cleavage
tae-miR159a	Traes_1DL_25540DE53.5						AT5G06100.2	myb domain protein 33	LOC_Os01g59660.2	MYB family transcription factor, putative, expressed	2,50	-1.0	1	21	470	490	UUUGGAUUGAAGGGAGCUCUG	UGGAGCUCCCUUCAUUCCAAU	Cleavage
tae-miR159b	Traes_1DL_25540DE53.5						AT5G06100.2	myb domain protein 33	LOC_Os01g59660.2	MYB family transcription factor, putative, expressed	2,50	-1.0	1	21	470	490	UUUGGAUUGAAGGGAGCUCUG	UGGAGCUCCCUUCAUUCCAAU	Cleavage
tae-miR159a	Traes_1DL_25540DE53.6						AT5G06100.2	myb domain protein 33	LOC_Os01g59660.1	MYB family transcription factor, putative, expressed	2,50	-1.0	1	21	470	490	UUUGGAUUGAAGGGAGCUCUG	UGGAGCUCCCUUCAUUCCAAU	Cleavage
tae-miR159b	Traes_1DL_25540DE53.6						AT5G06100.2	myb domain protein 33	LOC_Os01g59660.1	MYB family transcription factor, putative, expressed	2,50	-1.0	1	21	470	490	UUUGGAUUGAAGGGAGCUCUG	UGGAGCUCCCUUCAUUCCAAU	Cleavage
tae-miR1137a	Traes_1DL_34A359364.2	PF09439	KOG0070		K07937		AT5G14670.1	ADP-ribosylation factor A1B	LOC_Os03g59740.1	ADP-ribosylation factor, putative, expressed	2,50	-1.0	1	20	516	535	UAGUACAAAGUUGAGUCAUC	GAUGACCCAAUUUUGUACUA	Translation
tae-miR5062-5p	Traes_1DL_38415F71A.19						AT2G46060.2	transmembrane protein-related	LOC_Os10g30910.2	transmembrane protein-related, putative, expressed	2,50	-1.0	1	23	836	858	UGAACCUUAGGGAACAGCCGCAU	UUUUGGAUGUUCCCCAAGGUUCA	Cleavage
tae-miR5062-5p	Traes_1DL_38415F71A.20						AT2G46060.2	transmembrane protein-related	LOC_Os10g30910.2	transmembrane protein-related, putative, expressed	2,50	-1.0	1	23	836	858	UGAACCUUAGGGAACAGCCGCAU	UUUUGGAUGUUCCCCAAGGUUCA	Cleavage
tae-miR5062-5p	Traes_1DL_38415F71A.22						AT2G46060.1	transmembrane protein-related	LOC_Os10g30910.2	transmembrane protein-related, putative, expressed	2,50	-1.0	1	23	836	858	UGAACCUUAGGGAACAGCCGCAU	UUUUGGAUGUUCCCCAAGGUUCA	Cleavage
tae-miR1122a	Traes_1DL_38B88249C.2	PF00069	KOG0580	2.7.11.1		GO:GO:0006468,GO:GO:0005524,GO:GO:0004672	AT2G42960.1	Protein kinase superfamily protein	LOC_Os10g35450.1	serine/threonine-protein kinase, putative, expressed	0,00	-1.0	1	20	976	995	UAGAUACAUCCGUAUCUAGA	UCUAGAUACGGAUGUAUCUA	Cleavage
tae-miR1120a	Traes_1DL_6A91BC5A5.1	PF08652			K14845		AT4G17620.1	glycine-rich protein	LOC_Os05g32580.1	glycine-rich protein, putative, expressed	2,50	-1.0	1	24	25	48	ACAUUCUUAUAUUAUGAGACGGAG	CUCCGUCCCAUAAUAUAAGAACGU	Cleavage
tae-miR1120b-3p	Traes_1DL_6A91BC5A5.1	PF08652			K14845		AT4G17620.1	glycine-rich protein	LOC_Os05g32580.1	glycine-rich protein, putative, expressed	2,50	-1.0	1	21	25	45	UUCUUAUAUUGUGGGACAGAG	CUCCGUCCCAUAAUAUAAGAA	Translation
tae-miR1130b-3p	Traes_1DL_6A91BC5A5.1	PF08652			K14845		AT4G17620.1	glycine-rich protein	LOC_Os05g32580.1	glycine-rich protein, putative, expressed	0,00	-1.0	1	21	24	44	UCUUAUAUUAUGGGACGGAGG	CCUCCGUCCCAUAAUAUAAGA	Cleavage
tae-miR1122c-3p	Traes_1DL_6A91BC5A5.1	PF08652			K14845		AT4G17620.1	glycine-rich protein	LOC_Os05g32580.1	glycine-rich protein, putative, expressed	1,50	-1.0	1	21	24	44	UCUAAUAUUAUGGGACGGAGG	CCUCCGUCCCAUAAUAUAAGA	Cleavage
tae-miR1120c-5p	Traes_1DL_6A91BC5A5.1	PF08652			K14845		AT4G17620.1	glycine-rich protein	LOC_Os05g32580.1	glycine-rich protein, putative, expressed	2,50	-1.0	1	21	67	87	UAAUAUAAGAACGUUUUUGAC	GUCAAACACGCUCUUAUAUUA	Translation
tae-miR160	Traes_1DL_AEC2F3FB3.1	PF06507				GO:GO:0009725,GO:GO:0006355,GO:GO:0005634,GO:GO:0003677	AT4G30080.1	auxin response factor 16	LOC_Os10g33940.1	auxin response factor 18, putative, expressed	0,00	-1.0	1	21	642	662	UGCCUGGCUCCCUGUAUGCCA	AGGCAUACAGGGAGCCAGGCA	Cleavage
tae-miR1122b-3p	Traes_1DL_B5E6E273B.1										1,50	-1.0	1	21	35	55	AGACUUAUAUGUAGGAACGGA	UCCGUUCCUAAAUAUAAGUCU	Translation
tae-miR5175-5p	Traes_1DL_C1BE1742F.2	PF03641					AT3G53450.1	Putative lysine decarboxylase family protein	LOC_Os10g33900.1	possible lysine decarboxylase domain containing protein, expressed	2,00	-1.0	1	21	109	130	UUCCAAAUUACUCGUCG-UGGU	UUCAGCGACGAGUAAUUUGGAA	Cleavage
tae-miR1133	Traes_1DL_D391AE3DC.1	PF16212		3.6.3.1		GO:GO:0016021,GO:GO:0015914,GO:GO:0005524,GO:GO:0004012,GO:GO:0000287	AT1G59820.1	aminophospholipid ATPase 3	LOC_Os10g27220.1	phospholipid-transporting ATPase 3, putative, expressed	2,00	-1.0	1	22	858	879	CAUAUACUCCCUCCGUCCGAAA	AAAUGAACGGAGGGAGUAUAUG	Cleavage
tae-miR1133	Traes_1DL_D391AE3DC.2	PF16212		3.6.3.1		GO:GO:0016021,GO:GO:0015914,GO:GO:0005524,GO:GO:0004012,GO:GO:0000287	AT1G59820.1	aminophospholipid ATPase 3	LOC_Os10g27220.1	phospholipid-transporting ATPase 3, putative, expressed	2,00	-1.0	1	22	858	879	CAUAUACUCCCUCCGUCCGAAA	AAAUGAACGGAGGGAGUAUAUG	Cleavage
tae-miR1133	Traes_1DL_D391AE3DC.3	PF16212		3.6.3.1		GO:GO:0016021,GO:GO:0015914,GO:GO:0005524,GO:GO:0004012,GO:GO:0000287	AT1G59820.1	aminophospholipid ATPase 3	LOC_Os10g27220.1	phospholipid-transporting ATPase 3, putative, expressed	2,00	-1.0	1	22	822	843	CAUAUACUCCCUCCGUCCGAAA	AAAUGAACGGAGGGAGUAUAUG	Cleavage
tae-miR1128	Traes_1DL_E30609261.7						AT1G04300.3	TRAF-like superfamily protein	LOC_Os05g43280.1	MATH domain containing protein, expressed	3,00	-1.0	1	21	76	96	UACUACUCCCUCCGUCCGAAA	UUCCAGACGGAGGGAGUAGUU	Cleavage
tae-miR1122a	Traes_1DS_06B8348E0.1	PF03283				GO:GO:0016787	AT3G09410.1	Pectinacetylesterase family protein	LOC_Os05g02120.1	pectinacetylesterase domain containing protein, expressed	0,00	-1.0	1	20	794	813	UAGAUACAUCCGUAUCUAGA	UCUAGAUACGGAUGUAUCUA	Cleavage
tae-miR1122a	Traes_1DS_06B8348E0.2	PF03283				GO:GO:0016787	AT3G09410.1	Pectinacetylesterase family protein	LOC_Os05g02120.1	pectinacetylesterase domain containing protein, expressed	0,00	-1.0	1	20	620	639	UAGAUACAUCCGUAUCUAGA	UCUAGAUACGGAUGUAUCUA	Cleavage
tae-miR5048-5p	Traes_1DS_26F1546F6.1	PF00168,PF07714,PF00069	KOG1187	2.7.11.1		GO:GO:0005515,GO:GO:0006468,GO:GO:0004672,GO:GO:0005524	AT1G63430.1	Leucine-rich repeat protein kinase family protein	LOC_Os04g58700.1	expressed protein	2,50	-1.0	1	22	917	938	UUUGCAGGUUUUAGGUCUAAGU	ACUUGGACCUAAAACCUGCCAA	Cleavage
tae-miR5048-5p	Traes_1DS_26F1546F6.2	PF00168,PF07714,PF00069	KOG1187	2.7.11.1		GO:GO:0005515,GO:GO:0006468,GO:GO:0004672,GO:GO:0005524	AT1G63430.1	Leucine-rich repeat protein kinase family protein	LOC_Os04g58700.1	expressed protein	2,50	-1.0	1	22	917	938	UUUGCAGGUUUUAGGUCUAAGU	ACUUGGACCUAAAACCUGCCAA	Cleavage
tae-miR7757-5p	Traes_1DS_35EF41CCB.2	PF00931				GO:GO:0043531	AT3G50950.1	HOPZ-ACTIVATED RESISTANCE 1	LOC_Os05g40150.1	RGH2B, putative, expressed	1,50	-1.0	1	22	587	608	AUAAAACCUUCAGCUAUCCAUC	AGUGGAUAGCUGAAGGUUUCAU	Cleavage
tae-miR9674a-5p	Traes_1DS_F3DE3946F.1	PF13041					AT1G62930.1	Tetratricopeptide repeat (TPR)-like superfamily protein	LOC_Os04g28300.1	Rf1, mitochondrial precursor, putative, expressed	2,50	-1.0	1	21	522	542	GCAUCAUCCAUCCUACCAUUC	AAAUGGAAGGAUCGAUGAUGC	Cleavage
tae-miR5049-3p	Traes_1DS_FA7FC5C3C.1	PF04997		2.7.7.6		GO:GO:0006351,GO:GO:0003899,GO:GO:0003677	AT1G63020.1	nuclear RNA polymerase D1A	LOC_Os02g05880.1	RNA polymerase Rpb1, domain 2 family protein, expressed	0,00	-1.0	1	21	623	643	AAUAUGGAUCGGAGGGAGUAC	GUACUCCCUCCGAUCCAUAUU	Cleavage
tae-miR1128	Traes_2AL_24A1BF00C.1						AT5G06210.1	RNA binding (RRM/RBD/RNP motifs) family protein	LOC_Os04g33810.1	RNA recognition motif containing protein, putative, expressed	0,00	-1.0	1	21	37	57	UACUACUCCCUCCGUCCGAAA	UUUCGGACGGAGGGAGUAGUA	Cleavage
tae-miR5049-3p	Traes_2AL_28B13CC56.1					GO:GO:0005681,GO:GO:0000398	AT1G80070.1	Pre-mRNA-processing-splicing factor	LOC_Os06g07080.1	pre-mRNA-processing-splicing factor 8, putative, expressed	1,50	-1.0	1	21	346	366	AAUAUGGAUCGGAGGGAGUAC	CUACUCCCUCCGUUCCAUAUU	Cleavage
tae-miR1127a	Traes_2AL_4197D219B.1						AT3G16200.1		LOC_Os01g31220.1	expressed protein	2,50	-1.0	1	19	991	1009	UCCUUCCGUUCGGAAUUAC	GUAAUUCCGAACGGAGGGU	Cleavage
tae-miR164	Traes_2AL_57BF37A86.1	PF02365				GO:GO:0006355,GO:GO:0003677	AT5G61430.1	NAC domain containing protein 100	LOC_Os04g38720.1	no apical meristem protein, putative, expressed	1,50	-1.0	1	21	680	700	UGGAGAAGCAGGGCACGUGCA	CGCACGUGACCUGCUUCUCCA	Cleavage
tae-miR164	Traes_2AL_57BF37A86.2	PF02365				GO:GO:0006355,GO:GO:0003677	AT5G61430.1	NAC domain containing protein 100	LOC_Os04g38720.1	no apical meristem protein, putative, expressed	1,50	-1.0	1	21	606	626	UGGAGAAGCAGGGCACGUGCA	CGCACGUGACCUGCUUCUCCA	Cleavage
tae-miR1122a	Traes_2AL_64B73E3ED.1			2.8.1.8		GO:GO:0051539,GO:GO:0016992,GO:GO:0009107	AT2G20860.1	lipoic acid synthase 1	LOC_Os04g38330.1	radical SAM enzyme, putative, expressed	1,00	-1.0	1	20	121	140	UAGAUACAUCCGUAUCUAGA	UCUAAAUACGGAUGUAUCUA	Cleavage
tae-miR1133	Traes_2AL_673CA8FB2.1	PF04603	KOG3329				AT1G69680.1	Mog1/PsbP/DUF1795-like photosystem II reaction center PsbP family protein	LOC_Os04g38610.2	RAN guanine nucleotide release factor, putative, expressed	2,50	-1.0	1	22	553	575	CAUAUACUCCCUCCGUCCGA-AA	UUGUGGGACGGAGGGAGUAUUUG	Cleavage
tae-miR1120b-3p	Traes_2AL_673CA8FB2.1	PF04603	KOG3329				AT1G69680.1	Mog1/PsbP/DUF1795-like photosystem II reaction center PsbP family protein	LOC_Os04g38610.2	RAN guanine nucleotide release factor, putative, expressed	2,50	-1.0	1	21	493	513	UUCUUAUAUUGUGGGACAGAG	CUCUGUCCCACAAUGUAAGAC	Cleavage
tae-miR1120a	Traes_2AL_979E96822.1	PF00443		3.4.19.12		GO:GO:0036459,GO:GO:0016579	AT4G39370.3	ubiquitin-specific protease 27	LOC_Os04g34984.1	ubiquitin carboxyl-terminal hydrolase domain containing protein, expressed	2,50	-1.0	1	24	753	776	ACAUUCUUAUAUUAUGAGACGGAG	CUCCAUCCCAUAAUAUAAGAGUGU	Cleavage
tae-miR1130b-3p	Traes_2AL_979E96822.1	PF00443		3.4.19.12		GO:GO:0036459,GO:GO:0016579	AT4G39370.3	ubiquitin-specific protease 27	LOC_Os04g34984.1	ubiquitin carboxyl-terminal hydrolase domain containing protein, expressed	1,00	-1.0	1	21	752	772	UCUUAUAUUAUGGGACGGAGG	CCUCCAUCCCAUAAUAUAAGA	Cleavage
tae-miR1122c-3p	Traes_2AL_979E96822.1	PF00443		3.4.19.12		GO:GO:0036459,GO:GO:0016579	AT4G39370.3	ubiquitin-specific protease 27	LOC_Os04g34984.1	ubiquitin carboxyl-terminal hydrolase domain containing protein, expressed	2,50	-1.0	1	21	752	772	UCUAAUAUUAUGGGACGGAGG	CCUCCAUCCCAUAAUAUAAGA	Cleavage
tae-miR1120c-5p	Traes_2AL_979E96822.1	PF00443		3.4.19.12		GO:GO:0036459,GO:GO:0016579	AT4G39370.3	ubiquitin-specific protease 27	LOC_Os04g34984.1	ubiquitin carboxyl-terminal hydrolase domain containing protein, expressed	2,50	-1.0	1	21	795	815	UAAUAUAAGAACGUUUUUGAC	GUAAAAAACGCUCUUAUAUUA	Translation
tae-miR160	Traes_2AL_C70217249.1	PF06507,PF02362				GO:GO:0009725,GO:GO:0006355,GO:GO:0005634,GO:GO:0003677	AT1G77850.1	auxin response factor 17	LOC_Os04g59430.1	auxin response factor, putative, expressed	1,00	-1.0	1	21	1164	1184	UGCCUGGCUCCCUGUAUGCCA	UGGCAUGCAGGGAGCCAGGCA	Cleavage
tae-miR1134	Traes_2AL_D2CC35E59.1										3,00	-1.0	1	24	512	535	CAACAACAACAAGAAGAAGAAGAU	AUCUUCAUGGUCUUGUUGUUGUUG	Cleavage
tae-miR1134	Traes_2AL_D2CC35E59.2										3,00	-1.0	1	24	620	643	CAACAACAACAAGAAGAAGAAGAU	AUCUUCAUGGUCUUGUUGUUGUUG	Cleavage
tae-miR1134	Traes_2AL_D2CC35E59.3										3,00	-1.0	1	24	509	532	CAACAACAACAAGAAGAAGAAGAU	AUCUUCAUGGUCUUGUUGUUGUUG	Cleavage
tae-miR1134	Traes_2AL_D2CC35E59.4										3,00	-1.0	1	24	611	634	CAACAACAACAAGAAGAAGAAGAU	AUCUUCAUGGUCUUGUUGUUGUUG	Cleavage
tae-miR1134	Traes_2AL_D2CC35E59.5										3,00	-1.0	1	24	509	532	CAACAACAACAAGAAGAAGAAGAU	AUCUUCAUGGUCUUGUUGUUGUUG	Cleavage
tae-miR1134	Traes_2AL_D2CC35E59.6										3,00	-1.0	1	24	509	532	CAACAACAACAAGAAGAAGAAGAU	AUCUUCAUGGUCUUGUUGUUGUUG	Cleavage
tae-miR5384-3p	Traes_2AL_D79C9535C.1	PF00067	KOG0157	1.14.13.109		GO:GO:0055114,GO:GO:0020037,GO:GO:0016705,GO:GO:0005506	AT5G36110.1	cytochrome P450, family 716, subfamily A, polypeptide 1	LOC_Os09g21260.1	cytochrome P450 domain containing protein, expressed	2,50	-1.0	1	21	1250	1270	UGAGCGCGCCGCCGUCGAAUG	CGUUCGGCGGCGGCGCGCGCA	Cleavage
tae-miR9778	Traes_2AS_530CF4042.10	PF00931				GO:GO:0043531	AT3G50950.1	HOPZ-ACTIVATED RESISTANCE 1	LOC_Os04g12830.1	expressed protein	2,50	-1.0	1	21	126	146	UGCAUCAUCUCGAACUCGUCG	CGGCGAGUUCCAGAUGAUGCA	Translation
tae-miR9778	Traes_2AS_530CF4042.2	PF00931				GO:GO:0043531	AT3G50950.1	HOPZ-ACTIVATED RESISTANCE 1	LOC_Os04g12830.1	expressed protein	2,50	-1.0	1	21	126	146	UGCAUCAUCUCGAACUCGUCG	CGGCGAGUUCCAGAUGAUGCA	Translation
tae-miR9778	Traes_2AS_530CF4042.3	PF00931				GO:GO:0043531	AT3G50950.1	HOPZ-ACTIVATED RESISTANCE 1	LOC_Os04g12830.1	expressed protein	2,50	-1.0	1	21	126	146	UGCAUCAUCUCGAACUCGUCG	CGGCGAGUUCCAGAUGAUGCA	Translation
tae-miR1130b-3p	Traes_2AS_6DCA4AADD.1					GO:GO:0016020,GO:GO:0008236,GO:GO:0006508	AT2G31140.1	Peptidase S24/S26A/S26B/S26C family protein	LOC_Os04g01240.1	serine-type peptidase, putative, expressed	1,50	-1.0	1	21	288	308	UCUUAUAUUAUGGGACGGAGG	CCUCCGUCCCAUAAUAUAGGA	Cleavage
tae-miR9664-3p	Traes_2AS_86633353C.1	PF00931	KOG4658		K13457	GO:GO:0043531	AT3G07040.1	NB-ARC domain-containing disease resistance protein	LOC_Os11g46210.1	NB-ARC domain containing protein, expressed	2,50	-1.0	1	21	240	260	UUGCAGUCCUCGAUGUCGUAG	CUACGACAUCGAGGAUUGCAU	Cleavage
tae-miR1130b-3p	Traes_2BL_171777733.1	PF00443		3.4.19.12		GO:GO:0036459,GO:GO:0016579	AT4G39370.3	ubiquitin-specific protease 27	LOC_Os04g34984.1	ubiquitin carboxyl-terminal hydrolase domain containing protein, expressed	0,00	-1.0	1	21	752	772	UCUUAUAUUAUGGGACGGAGG	CCUCCGUCCCAUAAUAUAAGA	Cleavage
tae-miR1122c-3p	Traes_2BL_171777733.1	PF00443		3.4.19.12		GO:GO:0036459,GO:GO:0016579	AT4G39370.3	ubiquitin-specific protease 27	LOC_Os04g34984.1	ubiquitin carboxyl-terminal hydrolase domain containing protein, expressed	1,50	-1.0	1	21	752	772	UCUAAUAUUAUGGGACGGAGG	CCUCCGUCCCAUAAUAUAAGA	Cleavage
tae-miR1120c-5p	Traes_2BL_171777733.1	PF00443		3.4.19.12		GO:GO:0036459,GO:GO:0016579	AT4G39370.3	ubiquitin-specific protease 27	LOC_Os04g34984.1	ubiquitin carboxyl-terminal hydrolase domain containing protein, expressed	2,50	-1.0	1	21	795	815	UAAUAUAAGAACGUUUUUGAC	GUAAAAAACGCUCUUAUAUUA	Translation
tae-miR5384-3p	Traes_2BL_1BE5C9C71.1	PF00067		1.14.13.109		GO:GO:0055114,GO:GO:0020037,GO:GO:0016705,GO:GO:0005506	AT2G42850.1	cytochrome P450, family 718	LOC_Os09g21260.1	cytochrome P450 domain containing protein, expressed	2,50	-1.0	1	21	455	475	UGAGCGCGCCGCCGUCGAAUG	CGUUCGGCGGCGGCGCGCGCA	Cleavage
tae-miR5384-3p	Traes_2BL_1BE5C9C71.2	PF00067				GO:GO:0055114,GO:GO:0020037,GO:GO:0016705,GO:GO:0005506	AT5G36110.1	cytochrome P450, family 716, subfamily A, polypeptide 1	LOC_Os09g21260.1	cytochrome P450 domain containing protein, expressed	2,50	-1.0	1	21	455	475	UGAGCGCGCCGCCGUCGAAUG	CGUUCGGCGGCGGCGCGCGCA	Cleavage
tae-miR1122a	Traes_2BL_40442CE82.2	PF14392,PF00098				GO:GO:0008270,GO:GO:0003676	AT5G52380.1	VASCULAR-RELATED NAC-DOMAIN 6	LOC_Os04g46920.1	zinc knuckle domain containing protein, expressed	1,50	-1.0	1	20	703	722	UAGAUACAUCCGUAUCUAGA	UCUAGAUACAGAUGUAUCUA	Translation
tae-miR164	Traes_2BL_6AEE8AC28.1	PF02365				GO:GO:0006355,GO:GO:0003677	AT5G61430.1	NAC domain containing protein 100	LOC_Os04g38720.1	no apical meristem protein, putative, expressed	1,50	-1.0	1	21	680	700	UGGAGAAGCAGGGCACGUGCA	CGCACGUGACCUGCUUCUCCA	Cleavage
tae-miR164	Traes_2BL_6AEE8AC28.2	PF02365				GO:GO:0006355,GO:GO:0003677	AT5G61430.1	NAC domain containing protein 100	LOC_Os04g38720.1	no apical meristem protein, putative, expressed	1,50	-1.0	1	21	672	692	UGGAGAAGCAGGGCACGUGCA	CGCACGUGACCUGCUUCUCCA	Cleavage
tae-miR164	Traes_2BL_6AEE8AC28.3	PF02365				GO:GO:0006355,GO:GO:0003677	AT5G61430.1	NAC domain containing protein 100	LOC_Os04g38720.1	no apical meristem protein, putative, expressed	1,50	-1.0	1	21	633	653	UGGAGAAGCAGGGCACGUGCA	CGCACGUGACCUGCUUCUCCA	Cleavage
tae-miR1122b-3p	Traes_2BL_7141904F2.2	PF03097					AT1G73390.1	Endosomal targeting BRO1-like domain-containing protein	LOC_Os04g44530.3	expressed protein	2,50	-1.0	1	21	1058	1078	AGACUUAUAUGUAGGAACGGA	UCCGUUCUUAAAUAUAAGUCU	Translation
tae-miR1122a	Traes_2BL_7141904F2.2	PF03097					AT1G73390.1	Endosomal targeting BRO1-like domain-containing protein	LOC_Os04g44530.3	expressed protein	2,50	-1.0	1	20	1098	1117	UAGAUACAUCCGUAUCUAGA	ACUACAUACGGAUGUAUAUA	Cleavage
tae-miR1134	Traes_2BL_8339D35AC.1										3,00	-1.0	1	24	509	532	CAACAACAACAAGAAGAAGAAGAU	AUCUUCAUGGUCUUGUUGUUGUUG	Cleavage
tae-miR1134	Traes_2BL_8339D35AC.2										3,00	-1.0	1	24	512	535	CAACAACAACAAGAAGAAGAAGAU	AUCUUCAUGGUCUUGUUGUUGUUG	Cleavage
tae-miR1134	Traes_2BL_8339D35AC.3										3,00	-1.0	1	24	509	532	CAACAACAACAAGAAGAAGAAGAU	AUCUUCAUGGUCUUGUUGUUGUUG	Cleavage
tae-miR1134	Traes_2BL_8339D35AC.4										3,00	-1.0	1	24	302	325	CAACAACAACAAGAAGAAGAAGAU	AUCUUCAUGGUCUUGUUGUUGUUG	Cleavage
tae-miR1134	Traes_2BL_8339D35AC.5										3,00	-1.0	1	24	509	532	CAACAACAACAAGAAGAAGAAGAU	AUCUUCAUGGUCUUGUUGUUGUUG	Cleavage
tae-miR1135	Traes_2BL_A4AE931B0.1						AT2G02090.1	SNF2 domain-containing protein / helicase domain-containing protein	LOC_Os04g47830.1	SNF2 family N-terminal domain containing protein, expressed	2,00	-1.0	1	24	1154	1177	CUGCGACAAGUAAUUCCGAACGGA	UCUGUCCGGAAUUACUUGUCGCAA	Cleavage
tae-miR1122b-3p	Traes_2BL_FF8002798.2	PF13414					AT1G56090.1	Tetratricopeptide repeat (TPR)-like superfamily protein	LOC_Os04g52580.1	tetratricopeptide repeat domain containing protein, expressed	2,50	-1.0	1	21	889	909	AGACUUAUAUGUAGGAACGGA	UCGGUUCCUAAAUAUAAGUCU	Translation
tae-miR9778	Traes_2BS_0EFFAAE28.1	PF00931				GO:GO:0043531	AT3G07040.1	NB-ARC domain-containing disease resistance protein	LOC_Os08g14850.1	resistance protein, putative, expressed	2,50	-1.0	1	21	126	146	UGCAUCAUCUCGAACUCGUCG	CGGCGAGUUCCAGAUGAUGCA	Translation
tae-miR9778	Traes_2BS_0EFFAAE28.2	PF00931				GO:GO:0043531	AT3G07040.1	NB-ARC domain-containing disease resistance protein	LOC_Os08g14850.1	resistance protein, putative, expressed	2,50	-1.0	1	21	126	146	UGCAUCAUCUCGAACUCGUCG	CGGCGAGUUCCAGAUGAUGCA	Translation
tae-miR9662a-3p	Traes_2BS_257C5B099.1	PF02536				GO:GO:0006355,GO:GO:0005739,GO:GO:0003690	AT5G07900.1	Mitochondrial transcription termination factor family protein	LOC_Os06g12100.1	mTERF family protein, expressed	2,00	-1.0	1	21	66	86	UUGAACAUCCCAGAGCCACCG	CCGUGGUUCUGGGAUGUUCAG	Cleavage
tae-miR9662b-3p	Traes_2BS_257C5B099.1	PF02536				GO:GO:0006355,GO:GO:0005739,GO:GO:0003690	AT5G07900.1	Mitochondrial transcription termination factor family protein	LOC_Os06g12100.1	mTERF family protein, expressed	2,00	-1.0	1	21	65	85	UGAACAUCCCAGAGCCACCGG	CCCGUGGUUCUGGGAUGUUCA	Cleavage
tae-miR9662a-3p	Traes_2BS_257C5B0991.1	PF02536				GO:GO:0006355,GO:GO:0005739,GO:GO:0003690	AT5G07900.1	Mitochondrial transcription termination factor family protein	LOC_Os06g12100.1	mTERF family protein, expressed	2,00	-1.0	1	21	66	86	UUGAACAUCCCAGAGCCACCG	CCGUGGUUCUGGGAUGUUCAG	Cleavage
tae-miR9662b-3p	Traes_2BS_257C5B0991.1	PF02536				GO:GO:0006355,GO:GO:0005739,GO:GO:0003690	AT5G07900.1	Mitochondrial transcription termination factor family protein	LOC_Os06g12100.1	mTERF family protein, expressed	2,00	-1.0	1	21	65	85	UGAACAUCCCAGAGCCACCGG	CCCGUGGUUCUGGGAUGUUCA	Cleavage
tae-miR5048-5p	Traes_2BS_47E2D6444.1	PF07714		2.7.11.1		GO:GO:0006468,GO:GO:0004672	AT4G04570.2	cysteine-rich RLK (RECEPTOR-like protein kinase) 40	LOC_Os04g30030.1	cysteine-rich receptor-like protein kinase 12 precursor, putative, expressed	2,50	-1.0	1	22	221	242	UUUGCAGGUUUUAGGUCUAAGU	ACCUGGACCUAAAACCUGCGAA	Cleavage
tae-miR5048-5p	Traes_2BS_47E2D6444.2	PF00069	KOG1187	2.7.11.1		GO:GO:0006468,GO:GO:0005524,GO:GO:0004672	AT4G38830.1	cysteine-rich RLK (RECEPTOR-like protein kinase) 26	LOC_Os04g30030.1	cysteine-rich receptor-like protein kinase 12 precursor, putative, expressed	2,50	-1.0	1	22	959	980	UUUGCAGGUUUUAGGUCUAAGU	ACCUGGACCUAAAACCUGCGAA	Cleavage
tae-miR5048-5p	Traes_2BS_47E2D6444.3	PF00069	KOG1187	2.7.11.1		GO:GO:0006468,GO:GO:0005524,GO:GO:0004672	AT4G38830.1	cysteine-rich RLK (RECEPTOR-like protein kinase) 26	LOC_Os04g30030.1	cysteine-rich receptor-like protein kinase 12 precursor, putative, expressed	2,50	-1.0	1	22	947	968	UUUGCAGGUUUUAGGUCUAAGU	ACCUGGACCUAAAACCUGCGAA	Cleavage
tae-miR5048-5p	Traes_2BS_47E2D6444.4	PF07714		2.7.11.1		GO:GO:0006468,GO:GO:0004672	AT4G23160.1	cysteine-rich RLK (RECEPTOR-like protein kinase) 8	LOC_Os04g30030.1	cysteine-rich receptor-like protein kinase 12 precursor, putative, expressed	2,50	-1.0	1	22	221	242	UUUGCAGGUUUUAGGUCUAAGU	ACCUGGACCUAAAACCUGCGAA	Cleavage
tae-miR9664-3p	Traes_2BS_5045C640C.1	PF00931	KOG4658		K13457	GO:GO:0043531	AT3G07040.1	NB-ARC domain-containing disease resistance protein	LOC_Os11g46210.1	NB-ARC domain containing protein, expressed	2,50	-1.0	1	21	240	260	UUGCAGUCCUCGAUGUCGUAG	CUACGACAUCGAGGAUUGCAU	Cleavage
tae-miR9664-3p	Traes_2BS_5045C640C.2	PF00931	KOG4658		K13457	GO:GO:0043531	AT3G07040.1	NB-ARC domain-containing disease resistance protein	LOC_Os11g46210.1	NB-ARC domain containing protein, expressed	2,50	-1.0	1	21	240	260	UUGCAGUCCUCGAUGUCGUAG	CUACGACAUCGAGGAUUGCAU	Cleavage
tae-miR9664-3p	Traes_2BS_5045C640C.5	PF00931	KOG4658		K13457	GO:GO:0043531	AT3G07040.1	NB-ARC domain-containing disease resistance protein	LOC_Os11g46210.1	NB-ARC domain containing protein, expressed	2,50	-1.0	1	21	240	260	UUGCAGUCCUCGAUGUCGUAG	CUACGACAUCGAGGAUUGCAU	Cleavage
tae-miR1120c-5p	Traes_2BS_5C64FC44A.1	PF07714	KOG1187	2.7.11.1		GO:GO:0006468,GO:GO:0004672	AT4G23140.1	cysteine-rich RLK (RECEPTOR-like protein kinase) 6	LOC_Os07g35580.1	TKL_IRAK_DUF26-lc.20 - DUF26 kinases have homology to DUF26 containing loci, expressed	0,00	-1.0	1	21	21	41	UAAUAUAAGAACGUUUUUGAC	GUCAAAAACGUUCUUAUAUUA	Cleavage
tae-miR1120c-5p	Traes_2BS_5C64FC44A.2	PF07714		2.7.11.1		GO:GO:0006468,GO:GO:0004672	AT4G23180.1	cysteine-rich RLK (RECEPTOR-like protein kinase) 10	LOC_Os07g35580.1	TKL_IRAK_DUF26-lc.20 - DUF26 kinases have homology to DUF26 containing loci, expressed	0,00	-1.0	1	21	2	22	UAAUAUAAGAACGUUUUUGAC	GUCAAAAACGUUCUUAUAUUA	Cleavage
tae-miR1130b-3p	Traes_2BS_5CDCD5152.1	PF04178	KOG1743			GO:GO:0016192	AT5G01430.1	Got1/Sft2-like vescicle transport protein family	LOC_Os03g11100.1	vesicle transport protein GOT1B, putative, expressed	2,00	-1.0	1	20	1	21	UCUUAUAUUAUGGGA-CGGAG	GUCCGAUCCCAUAAUAUAAGA	Cleavage
tae-miR1121	Traes_2BS_6249E432F.1	PF01426				GO:GO:0003682	AT4G22140.1	PHD finger family protein / bromo-adjacent homology (BAH) domain-containing protein	LOC_Os07g08880.1	ES43 protein, putative, expressed	0,00	-1.0	1	22	538	559	AGUAGUGAUCUAAACGCUCUUA	UAAGAGCGUUUAGAUCACUACU	Cleavage
tae-miR1121	Traes_2BS_6249E432F.2	PF01426				GO:GO:0003682	AT4G22140.1	PHD finger family protein / bromo-adjacent homology (BAH) domain-containing protein	LOC_Os07g08880.1	ES43 protein, putative, expressed	0,00	-1.0	1	22	538	559	AGUAGUGAUCUAAACGCUCUUA	UAAGAGCGUUUAGAUCACUACU	Cleavage
tae-miR1121	Traes_2BS_6249E432F.3	PF01426				GO:GO:0003682	AT4G39100.1	PHD finger family protein / bromo-adjacent homology (BAH) domain-containing protein	LOC_Os07g08880.1	ES43 protein, putative, expressed	0,00	-1.0	1	22	448	469	AGUAGUGAUCUAAACGCUCUUA	UAAGAGCGUUUAGAUCACUACU	Cleavage
tae-miR1133	Traes_2BS_67D862F4A.1	PF01428	KOG3183			GO:GO:0008270	AT2G41835.1	zinc finger (C2H2 type, AN1-like) family protein	LOC_Os07g38240.1	ZOS7-05 - C2H2 zinc finger protein, expressed	2,50	-1.0	1	22	30	51	CAUAUACUCCCUCCGUCCGAAA	UUUGGGACGGAGGGAGUAUAAG	Cleavage
tae-miR9662a-3p	Traes_2BS_74269A4C5.1	PF02536				GO:GO:0006355,GO:GO:0005739,GO:GO:0003690	AT5G07900.1	Mitochondrial transcription termination factor family protein	LOC_Os06g12100.1	mTERF family protein, expressed	2,00	-1.0	1	21	189	209	UUGAACAUCCCAGAGCCACCG	CCGUGGUUCUGGGAUGUUCAG	Cleavage
tae-miR9662b-3p	Traes_2BS_74269A4C5.1	PF02536				GO:GO:0006355,GO:GO:0005739,GO:GO:0003690	AT5G07900.1	Mitochondrial transcription termination factor family protein	LOC_Os06g12100.1	mTERF family protein, expressed	2,00	-1.0	1	21	188	208	UGAACAUCCCAGAGCCACCGG	CCCGUGGUUCUGGGAUGUUCA	Cleavage
tae-miR1128	Traes_2BS_7AEB138EB.1	PF01397,PF03936		4.2.3.104		GO:GO:0016829,GO:GO:0010333,GO:GO:0008152,GO:GO:0000287	AT5G23960.2	terpene synthase 21	LOC_Os07g11790.1	terpene synthase, putative, expressed	1,50	-1.0	1	21	84	104	UACUACUCCCUCCGUCCGAAA	UUUCGGACGGAGGGAGUACUA	Cleavage
tae-miR9661-5p	Traes_2BS_8281050F1.1										3,00	-1.0	1	21	845	865	UGAAGUAGAGCAGGGACCUCA	GACCGUUCCUGCUCUACUUCA	Cleavage
tae-miR9661-5p	Traes_2BS_8281050F11.1										3,00	-1.0	1	21	845	865	UGAAGUAGAGCAGGGACCUCA	GACCGUUCCUGCUCUACUUCA	Cleavage
tae-miR1122b-3p	Traes_2BS_A20CCBEC3.1	PF05368		1.23.1.1,1.23.1.3			AT1G32100.1	pinoresinol reductase 1	LOC_Os12g16220.2	nmrA-like family domain containing protein, expressed	2,50	-1.0	1	21	389	409	AGACUUAUAUGUAGGAACGGA	UCUGUUCCUAAAUAUAAGUCU	Translation

tae-miR1128	Traes_2BS_B3A88F254.2	PF00479,PF02781		1.1.1.49	K00036	GO:GO:0055114,GO:GO:0050661,GO:GO:0006006,GO:GO:0004345	AT5G13110.1	glucose-6-phosphate dehydrogenase 2	LOC_Os07g22350.1	glucose-6-phosphate 1-dehydrogenase, chloroplast precursor, putative, expressed	2,00	-1.0	1	21	88	109	UACUACUCCCUCCGUCC-GAAA	UUUCCGGACGGAGGGAGUAGUA	Cleavage
tae-miR1128	Traes_2BS_B3A88F254.3	PF00479,PF02781		1.1.1.49	K00036	GO:GO:0055114,GO:GO:0050661,GO:GO:0006006,GO:GO:0004345	AT5G13110.1	glucose-6-phosphate dehydrogenase 2	LOC_Os07g22350.1	glucose-6-phosphate 1-dehydrogenase, chloroplast precursor, putative, expressed	2,00	-1.0	1	21	88	109	UACUACUCCCUCCGUCC-GAAA	UUUCCGGACGGAGGGAGUAGUA	Cleavage
tae-miR1127a	Traes_2BS_C00FC92D4.2						AT2G18910.1	hydroxyproline-rich glycoprotein family protein	LOC_Os02g26370.1	wiskott-Aldrich syndrome protein family member 2, putative, expressed	1,50	-1.0	1	19	497	515	UCCUUCCGUUCGGAAUUAC	GUAAUUCCGAACGGAGGGA	Cleavage
tae-miR1117	Traes_2BS_C5AF5419E.1			3.4.23.47,3.1.26.13,3.1.13.2,2.7.7.49,2.7.7.7					LOC_Os02g49670.1	zinc knuckle family protein, expressed	0,00	-1.0	1	24	926	949	UAGUACCGGUUCGUGGCACGAACC	GGUUCGUGCCACGAACCGGUACUA	Cleavage
tae-miR9662a-3p	Traes_2BS_DDF26C784.1	PF02536				GO:GO:0006355,GO:GO:0005739,GO:GO:0003690	AT5G07900.1	Mitochondrial transcription termination factor family protein	LOC_Os06g12100.1	mTERF family protein, expressed	2,00	-1.0	1	21	459	479	UUGAACAUCCCAGAGCCACCG	CCGUGGUUCUGGGAUGUUCAG	Cleavage
tae-miR9662b-3p	Traes_2BS_DDF26C784.1	PF02536				GO:GO:0006355,GO:GO:0005739,GO:GO:0003690	AT5G07900.1	Mitochondrial transcription termination factor family protein	LOC_Os06g12100.1	mTERF family protein, expressed	2,00	-1.0	1	21	458	478	UGAACAUCCCAGAGCCACCGG	CCCGUGGUUCUGGGAUGUUCA	Cleavage
tae-miR5384-3p	Traes_2BS_EBCD10293.1	PF02225,PF00082		3.4.21.25		GO:GO:0006508,GO:GO:0004252	AT1G04110.1	Subtilase family protein	LOC_Os04g10360.1	OsSub40 - Putative Subtilisin homologue, expressed	2,50	-1.0	1	21	116	136	UGAGCGCGCCGCCGUCGAAUG	UGUUCGCCGACGGCGCGCUCA	Cleavage
tae-miR5384-3p	Traes_2BS_EBCD10293.2	PF02225,PF00082		3.4.21.25		GO:GO:0006508,GO:GO:0004252	AT1G04110.1	Subtilase family protein	LOC_Os04g10360.1	OsSub40 - Putative Subtilisin homologue, expressed	2,50	-1.0	1	21	116	136	UGAGCGCGCCGCCGUCGAAUG	UGUUCGCCGACGGCGCGCUCA	Cleavage
tae-miR1130b-3p	Traes_2DL_10B2965EF.8	PF00443		3.4.19.12		GO:GO:0036459,GO:GO:0016579	AT4G39370.2	ubiquitin-specific protease 27	LOC_Os04g34984.3	ubiquitin carboxyl-terminal hydrolase domain containing protein, expressed	0,00	-1.0	1	21	752	772	UCUUAUAUUAUGGGACGGAGG	CCUCCGUCCCAUAAUAUAAGA	Cleavage
tae-miR1122c-3p	Traes_2DL_10B2965EF.8	PF00443		3.4.19.12		GO:GO:0036459,GO:GO:0016579	AT4G39370.2	ubiquitin-specific protease 27	LOC_Os04g34984.3	ubiquitin carboxyl-terminal hydrolase domain containing protein, expressed	1,50	-1.0	1	21	752	772	UCUAAUAUUAUGGGACGGAGG	CCUCCGUCCCAUAAUAUAAGA	Cleavage
tae-miR1120c-5p	Traes_2DL_10B2965EF.8	PF00443		3.4.19.12		GO:GO:0036459,GO:GO:0016579	AT4G39370.2	ubiquitin-specific protease 27	LOC_Os04g34984.3	ubiquitin carboxyl-terminal hydrolase domain containing protein, expressed	1,50	-1.0	1	21	795	815	UAAUAUAAGAACGUUUUUGAC	GUCAAAAACGCUCUUAUAUUA	Translation
tae-miR1130b-3p	Traes_2DL_3C9129432.1	PF00656				GO:GO:0006508,GO:GO:0004197	AT1G79340.1	metacaspase 4	LOC_Os01g58580.1	ICE-like protease p20 domain containing protein, putative, expressed	1,50	-1.0	1	21	164	184	UCUUAUAUUAUGGGACGGAGG	CUUCCGUCCCAUAAUGUAAGA	Cleavage
tae-miR1133	Traes_2DL_4481C0380.4										2,50	-1.0	1	22	1	22	CAUAUACUCCCUCCGUCCGAAA	UUUGGGACGGAGGGAGUACAUG	Cleavage
tae-miR1137a	Traes_2DL_4F8C87C42.5	PF04499	KOG2073				AT1G30470.1	SIT4 phosphatase-associated family protein	LOC_Os04g52940.1	SIT4 phosphatase-associated protein domain containing protein, expressed	1,00	-1.0	1	20	1791	1810	UAGUACAAAGUUGAGUCAUC	GAUGACCCAACUUUGUACUA	Cleavage
tae-miR1127b-3p	Traes_2DL_4F8C87C42.5	PF04499	KOG2073				AT1G30470.1	SIT4 phosphatase-associated family protein	LOC_Os04g52940.1	SIT4 phosphatase-associated protein domain containing protein, expressed	1,50	-1.0	1	21	1822	1842	ACAAGUAUUUCUGGACGGAGG	CCUCCGUCCGGAAAUACUUGU	Cleavage
tae-miR1137b-5p	Traes_2DL_4F8C87C42.5	PF04499	KOG2073				AT1G30470.1	SIT4 phosphatase-associated family protein	LOC_Os04g52940.1	SIT4 phosphatase-associated protein domain containing protein, expressed	1,50	-1.0	1	21	1944	1964	UCCGUUCCAGAAUAGAUGACC	GGUCAUCUAUUUUGGAACGGA	Translation
tae-miR1122a	Traes_2DL_4F8C87C42.6	PF04499					AT1G30470.1	SIT4 phosphatase-associated family protein	LOC_Os04g52940.1	SIT4 phosphatase-associated protein domain containing protein, expressed	2,50	-1.0	1	20	1750	1769	UAGAUACAUCCGUAUCUAGA	ACUACAUACGGAUGUAUAUA	Cleavage
tae-miR1121	Traes_2DL_74E241841.2	PF01426				GO:GO:0003682	AT4G39100.1	PHD finger family protein / bromo-adjacent homology (BAH) domain-containing protein	LOC_Os07g08880.1	ES43 protein, putative, expressed	2,50	-1.0	1	22	448	469	AGUAGUGAUCUAAACGCUCUUA	UAAGAGUGUUUGGAUCACUACU	Translation
tae-miR1130b-3p	Traes_2DL_8F04980F0.5	PF03405		1.14.19.2		GO:GO:0055114,GO:GO:0045300,GO:GO:0006631	AT3G02630.1	Plant stearoyl-acyl-carrier-protein desaturase family protein	LOC_Os04g31070.1	acyl-desaturase, chloroplast precursor, putative, expressed	2,50	-1.0	1	21	468	488	UCUUAUAUUAUGGGACGGAGG	CCUCCGUCCCCUAAUAUAAGG	Translation
tae-miR1128	Traes_2DL_9AD532712.8	PF15613,PF15614					AT5G08630.1	DDT domain-containing protein	LOC_Os04g35864.1	DDT domain-containing protein, putative, expressed	2,00	-1.0	1	21	305	325	UACUACUCCCUCCGUCCGAAA	GUCCGGACGGAGGGAGUAGUU	Cleavage
tae-miR1122a	Traes_2DL_B3CD23EBC.1	PF05496,PF00004				GO:GO:0009378,GO:GO:0006310,GO:GO:0006281,GO:GO:0005524	AT1G03000.1	peroxin 6	LOC_Os04g52690.1	AAA family ATPase, putative, expressed	2,50	-1.0	1	20	9	28	UAGAUACAUCCGUAUCUAGA	ACUACAUACGGAUGUAUAUA	Cleavage
tae-miR9662a-3p	Traes_2DL_BE754F0C1.1	PF02536	KOG1267		K15032	GO:GO:0006355,GO:GO:0005739,GO:GO:0003690	AT5G07900.1	Mitochondrial transcription termination factor family protein	LOC_Os06g12100.1	mTERF family protein, expressed	1,00	-1.0	1	21	687	707	UUGAACAUCCCAGAGCCACCG	CCGUGGCUCUGGGAUGUUCAG	Cleavage
tae-miR9662b-3p	Traes_2DL_BE754F0C1.1	PF02536	KOG1267		K15032	GO:GO:0006355,GO:GO:0005739,GO:GO:0003690	AT5G07900.1	Mitochondrial transcription termination factor family protein	LOC_Os06g12100.1	mTERF family protein, expressed	1,00	-1.0	1	21	686	706	UGAACAUCCCAGAGCCACCGG	CCCGUGGCUCUGGGAUGUUCA	Cleavage
tae-miR1134	Traes_2DL_E8A7ABB5D.1										3,00	-1.0	1	24	509	532	CAACAACAACAAGAAGAAGAAGAU	AUCUUCAUGGUCUUGUUGUUGUUG	Cleavage
tae-miR1134	Traes_2DL_E8A7ABB5D.2										3,00	-1.0	1	24	629	652	CAACAACAACAAGAAGAAGAAGAU	AUCUUCAUGGUCUUGUUGUUGUUG	Cleavage
tae-miR1134	Traes_2DL_E8A7ABB5D.3										3,00	-1.0	1	24	614	637	CAACAACAACAAGAAGAAGAAGAU	AUCUUCAUGGUCUUGUUGUUGUUG	Cleavage
tae-miR1134	Traes_2DL_E8A7ABB5D.4										3,00	-1.0	1	24	512	535	CAACAACAACAAGAAGAAGAAGAU	AUCUUCAUGGUCUUGUUGUUGUUG	Cleavage
tae-miR1134	Traes_2DL_E8A7ABB5D.5										3,00	-1.0	1	24	509	532	CAACAACAACAAGAAGAAGAAGAU	AUCUUCAUGGUCUUGUUGUUGUUG	Cleavage
tae-miR1134	Traes_2DL_E8A7ABB5D.6										3,00	-1.0	1	24	509	532	CAACAACAACAAGAAGAAGAAGAU	AUCUUCAUGGUCUUGUUGUUGUUG	Cleavage
tae-miR1134	Traes_2DL_E8A7ABB5D.7										3,00	-1.0	1	24	91	114	CAACAACAACAAGAAGAAGAAGAU	AUCUUCAUGGUCUUGUUGUUGUUG	Cleavage
tae-miR1134	Traes_2DL_E8A7ABB5D.8										3,00	-1.0	1	24	77	100	CAACAACAACAAGAAGAAGAAGAU	AUCUUCAUGGUCUUGUUGUUGUUG	Cleavage
tae-miR1134	Traes_2DL_E8A7ABB5D.9										3,00	-1.0	1	24	28	51	CAACAACAACAAGAAGAAGAAGAU	AUCUUCAUGGUCUUGUUGUUGUUG	Cleavage
tae-miR160	Traes_2DL_EF12C0B89.2						AT1G77850.1	auxin response factor 17	LOC_Os04g59430.1	auxin response factor, putative, expressed	1,00	-1.0	1	21	225	245	UGCCUGGCUCCCUGUAUGCCA	UGGCAUGCAGGGAGCCAGGCA	Cleavage
tae-miR9664-3p	Traes_2DS_480CFDFE4.1	PF01637	KOG4658		K13457	GO:GO:0005524	AT3G07040.1	NB-ARC domain-containing disease resistance protein	LOC_Os11g46210.1	NB-ARC domain containing protein, expressed	2,50	-1.0	1	21	243	263	UUGCAGUCCUCGAUGUCGUAG	CUACGACAUCGAGGAUUGCAU	Cleavage
tae-miR9664-3p	Traes_2DS_480CFDFE4.2	PF01637				GO:GO:0005524	AT3G07040.1	NB-ARC domain-containing disease resistance protein	LOC_Os11g46210.1	NB-ARC domain containing protein, expressed	2,50	-1.0	1	21	259	279	UUGCAGUCCUCGAUGUCGUAG	CUACGACAUCGAGGAUUGCAU	Cleavage
tae-miR5049-3p	Traes_2DS_851973269.7										1,50	-1.0	1	21	178	198	AAUAUGGAUCGGAGGGAGUAC	GUACUCCCUCCGAUUCAUAUU	Cleavage
tae-miR1122b-3p	Traes_2DS_9B301D4F8.2	PF00069	KOG0580,KOG2345	2.7.11.1		GO:GO:0006468,GO:GO:0005524,GO:GO:0004672	AT5G18500.2	Protein kinase superfamily protein	LOC_Os11g39450.1	cysteine-rich receptor-like protein kinase 7 precursor, putative, expressed	1,50	-1.0	1	21	843	863	AGACUUAUAUGUAGGAACGGA	UCCGUUCCUAAAUAUAAGUCU	Translation
tae-miR1123	Traes_2DS_C866A54F1.2						AT4G36730.1	G-box binding factor 1	LOC_Os07g10890.1	transcription factor, putative, expressed	1,00	-1.0	1	23	792	814	UCCGUGAGACCUGGUCUCAUAGA	UCUAUGAGACCAGGUCUCACGGG	Cleavage
tae-miR1123	Traes_2DS_C866A54F1.4						AT4G36730.1	G-box binding factor 1	LOC_Os07g10890.1	transcription factor, putative, expressed	1,00	-1.0	1	23	786	808	UCCGUGAGACCUGGUCUCAUAGA	UCUAUGAGACCAGGUCUCACGGG	Cleavage
tae-miR9778	Traes_2DS_C9635AFF7.1	PF00931				GO:GO:0043531	AT3G07040.1	NB-ARC domain-containing disease resistance protein	LOC_Os08g14850.1	resistance protein, putative, expressed	2,50	-1.0	1	21	126	146	UGCAUCAUCUCGAACUCGUCG	CGGCGAGUUCCAGAUGAUGCA	Translation
tae-miR9778	Traes_2DS_C9635AFF7.2	PF00931				GO:GO:0043531	AT3G07040.1	NB-ARC domain-containing disease resistance protein	LOC_Os08g14850.1	resistance protein, putative, expressed	2,50	-1.0	1	21	105	125	UGCAUCAUCUCGAACUCGUCG	CGGCGAGUUCCAGAUGAUGCA	Translation
tae-miR1117	Traes_3AL_018D4C27C.1	PF07714,PF12819		2.7.11.1		GO:GO:0006468,GO:GO:0004672	AT3G51550.1	Malectin/receptor-like protein kinase family protein	LOC_Os05g25430.2	receptor-like protein kinase At3g46290 precursor, putative, expressed	1,50	-1.0	1	24	1609	1632	UAGUACCGGUUCGUGGCACGAACC	GGUUCGUGCCACGAACCGGUGCUA	Cleavage
tae-miR1133	Traes_3AL_30294D0A5.1			1.1.1.8		GO:GO:0055114,GO:GO:0009331,GO:GO:0006072,GO:GO:0004367	AT2G41540.3	6-phosphogluconate dehydrogenase family protein	LOC_Os01g71280.1	glycerol-3-phosphate dehydrogenase, putative, expressed	2,50	-1.0	1	22	392	413	CAUAUACUCCCUCCGUCCGAAA	UUUAGGACGGAGGGAGUAUUUG	Cleavage
tae-miR1133	Traes_3AL_386795528.2	PF00249					AT5G02320.2	myb domain protein 3r-5	LOC_Os01g62410.1	MYB family transcription factor, putative, expressed	0,00	-1.0	1	22	39	60	CAUAUACUCCCUCCGUCCGAAA	UUUCGGACGGAGGGAGUAUAUG	Cleavage
tae-miR1122a	Traes_3AL_5E5C8D264.2								LOC_Os05g46470.1	expressed protein	1,00	-1.0	1	20	18	37	UAGAUACAUCCGUAUCUAGA	UCUAGACACGGAUGUAUCUA	Cleavage
tae-miR1137b-5p	Traes_3AL_7A4D34425.2						AT1G29820.2	Magnesium transporter CorA-like family protein	LOC_Os01g47460.1	expressed protein	2,50	-1.0	1	21	1006	1026	UCCGUUCCAGAAUAGAUGACC	UAUCAUUUAUUCAGGAACGGA	Cleavage
tae-miR1122a	Traes_3AL_B9EDE61D1.1	PF06017				GO:GO:0016459,GO:GO:0003774	AT5G53310.1	myosin heavy chain-related	LOC_Os01g43360.1	myosin-Ie, putative, expressed	2,50	-1.0	1	20	4	23	UAGAUACAUCCGUAUCUAGA	ACUACAUACGGAUGUAUAUA	Cleavage
tae-miR1122a	Traes_3AL_B9EDE61D1.2	PF06017				GO:GO:0016459,GO:GO:0003774	AT5G53310.1	myosin heavy chain-related	LOC_Os01g43360.1	myosin-Ie, putative, expressed	2,50	-1.0	1	20	4	23	UAGAUACAUCCGUAUCUAGA	ACUACAUACGGAUGUAUAUA	Cleavage
tae-miR5049-3p	Traes_3AL_CF1A4ACDE.1	PF03759				GO:GO:0005089	AT1G52240.1	RHO guanyl-nucleotide exchange factor 11	LOC_Os01g55520.1	ATROPGEF7/ROPGEF7, putative, expressed	1,00	-1.0	1	21	917	937	AAUAUGGAUCGGAGGGAGUAC	UCCCUCCCUCCGAUCCAUAUU	Cleavage
tae-miR159a	Traes_3AL_F09C7CA19.1	PF00249					AT5G06100.1	myb domain protein 33	LOC_Os01g59660.2	MYB family transcription factor, putative, expressed	2,50	-1.0	1	21	620	640	UUUGGAUUGAAGGGAGCUCUG	UGGAGCUCCCUUCACUCCAAG	Cleavage
tae-miR159b	Traes_3AL_F09C7CA19.1	PF00249					AT5G06100.1	myb domain protein 33	LOC_Os01g59660.2	MYB family transcription factor, putative, expressed	2,50	-1.0	1	21	620	640	UUUGGAUUGAAGGGAGCUCUG	UGGAGCUCCCUUCACUCCAAG	Cleavage
tae-miR1121	Traes_3AL_F2EDB3661.7										2,00	-1.0	1	22	224	245	AGUAGUGAUCUAAACGCUCUUA	CCUCCGCGUUUAGAUCACUACU	Cleavage
tae-miR1133	Traes_3AL_FA6F160DF.7										3,00	-1.0	1	22	142	164	CAUAUACUCCCUCCG-UCCGAAA	UUUCGGAACGGAGGGAGUAUAUU	Cleavage
tae-miR5200	Traes_3AS_2A0765E10.1	PF14531		2.7.11.1			AT1G62400.1	Protein kinase superfamily protein	LOC_Os01g01740.1	protein kinase domain containing protein, expressed	2,50	-1.0	1	21	177	197	UGUAGAUACUCCCUAAGGCUU	UUCCCUUAGGAAGUAUCUACA	Translation
tae-miR5200	Traes_3AS_2A0765E10.2	PF14531		2.7.11.1			AT1G62400.1	Protein kinase superfamily protein	LOC_Os01g01740.1	protein kinase domain containing protein, expressed	2,50	-1.0	1	21	66	86	UGUAGAUACUCCCUAAGGCUU	UUCCCUUAGGAAGUAUCUACA	Translation
tae-miR5200	Traes_3AS_2A0765E10.3	PF14531		2.7.11.1			AT1G62400.1	Protein kinase superfamily protein	LOC_Os01g01740.1	protein kinase domain containing protein, expressed	2,50	-1.0	1	21	12	32	UGUAGAUACUCCCUAAGGCUU	UUCCCUUAGGAAGUAUCUACA	Translation
tae-miR9674b-5p	Traes_3AS_6CB1DBD28.1	PF01535,PF13041					AT3G09040.1	Pentatricopeptide repeat (PPR) superfamily protein	LOC_Os04g43430.3	pentatricopeptide, putative, expressed	2,00	-1.0	1	21	429	449	AUAGCAUCAUCCAUCCUACCC	GGGUAGGCUGGAUGAUGCUAG	Cleavage
tae-miR9674b-5p	Traes_3AS_6CB1DBD28.1	PF01535,PF13041					AT3G09040.1	Pentatricopeptide repeat (PPR) superfamily protein	LOC_Os04g43430.3	pentatricopeptide, putative, expressed	2,00	-1.0	1	21	429	449	AUAGCAUCAUCCAUCCUACCC	GGGUAGGCUGGAUGAUGCUAG	Cleavage
tae-miR1121	Traes_3AS_A466B9ECC.1	PF13424					AT3G54360.1	zinc ion binding	LOC_Os01g01420.2	expressed protein	0,00	-1.0	1	22	725	746	AGUAGUGAUCUAAACGCUCUUA	UAAGAGCGUUUAGAUCACUACU	Cleavage
tae-miR6197-5p	Traes_3AS_A466B9ECC.1	PF13424					AT3G54360.1	zinc ion binding	LOC_Os01g01420.2	expressed protein	2,50	-1.0	1	21	763	783	UCUGUAAACAAAUGUAGGACG	GCUCUUACAUUUCUUUACAGA	Cleavage
tae-miR1121	Traes_3AS_A466B9ECC.2	PF13424					AT3G54360.1	zinc ion binding	LOC_Os01g01420.2	expressed protein	0,00	-1.0	1	22	725	746	AGUAGUGAUCUAAACGCUCUUA	UAAGAGCGUUUAGAUCACUACU	Cleavage
tae-miR6197-5p	Traes_3AS_A466B9ECC.2	PF13424					AT3G54360.1	zinc ion binding	LOC_Os01g01420.2	expressed protein	2,50	-1.0	1	21	763	783	UCUGUAAACAAAUGUAGGACG	GCUCUUACAUUUCUUUACAGA	Cleavage
tae-miR1133	Traes_3AS_A6D3DD27E.2						AT4G32920.2	glycine-rich protein	LOC_Os01g09246.1	glycine-rich protein, putative, expressed	2,50	-1.0	1	22	16	37	CAUAUACUCCCUCCGUCCGAAA	UUUGGGACGGAGGGAGUAUGUG	Cleavage
tae-miR1133	Traes_3AS_A6D3DD27E.4						AT4G32920.1	glycine-rich protein	LOC_Os01g09246.1	glycine-rich protein, putative, expressed	2,50	-1.0	1	22	9	30	CAUAUACUCCCUCCGUCCGAAA	UUUGGGACGGAGGGAGUAUGUG	Cleavage
tae-miR1130b-3p	Traes_3AS_AA474B823.1	PF00725,PF16113,PF02737	KOG2304	5.1.2.3,4.2.1.17,1.1.1.35,5.3.3.8		GO:GO:0055114,GO:GO:0016491,GO:GO:0006631,GO:GO:0003857	AT4G29010.1	Enoyl-CoA hydratase/isomerase family	LOC_Os02g17390.1	3-hydroxyacyl-CoA dehydrogenase, putative, expressed	1,50	-1.0	1	21	1652	1672	UCUUAUAUUAUGGGACGGAGG	CCUCCGUCCCAUAAUAUGAGA	Cleavage
tae-miR1122c-3p	Traes_3AS_AA474B823.1	PF00725,PF16113,PF02737	KOG2304	5.1.2.3,4.2.1.17,1.1.1.35,5.3.3.8		GO:GO:0055114,GO:GO:0016491,GO:GO:0006631,GO:GO:0003857	AT4G29010.1	Enoyl-CoA hydratase/isomerase family	LOC_Os02g17390.1	3-hydroxyacyl-CoA dehydrogenase, putative, expressed	1,50	-1.0	1	21	1652	1672	UCUAAUAUUAUGGGACGGAGG	CCUCCGUCCCAUAAUAUGAGA	Cleavage
tae-miR1130b-3p	Traes_3AS_AA474B823.3	PF00725,PF00378,PF02737	KOG2304	5.1.2.3,4.2.1.17,1.1.1.35,5.3.3.8		GO:GO:0055114,GO:GO:0016491,GO:GO:0006631,GO:GO:0003857,GO:GO:0008152,GO:GO:0003824	AT4G29010.1	Enoyl-CoA hydratase/isomerase family	LOC_Os02g17390.1	3-hydroxyacyl-CoA dehydrogenase, putative, expressed	1,50	-1.0	1	21	1397	1417	UCUUAUAUUAUGGGACGGAGG	CCUCCGUCCCAUAAUAUGAGA	Cleavage
tae-miR1122c-3p	Traes_3AS_AA474B823.3	PF00725,PF00378,PF02737	KOG2304	5.1.2.3,4.2.1.17,1.1.1.35,5.3.3.8		GO:GO:0055114,GO:GO:0016491,GO:GO:0006631,GO:GO:0003857,GO:GO:0008152,GO:GO:0003824	AT4G29010.1	Enoyl-CoA hydratase/isomerase family	LOC_Os02g17390.1	3-hydroxyacyl-CoA dehydrogenase, putative, expressed	1,50	-1.0	1	21	1397	1417	UCUAAUAUUAUGGGACGGAGG	CCUCCGUCCCAUAAUAUGAGA	Cleavage
tae-miR159a	Traes_3AS_D1D1A1994.1								LOC_Os01g11430.1	expressed protein	2,50	-1.0	1	21	101	121	UUUGGAUUGAAGGGAGCUCUG	CAGAGCCCCCUUCAAACCAAA	Cleavage
tae-miR159b	Traes_3AS_D1D1A1994.1								LOC_Os01g11430.1	expressed protein	2,50	-1.0	1	21	101	121	UUUGGAUUGAAGGGAGCUCUG	CAGAGCCCCCUUCAAACCAAA	Cleavage
tae-miR1122b-3p	Traes_3AS_D7F2F36BB.2						AT3G49720.2		LOC_Os01g05070.1	expressed protein	1,50	-1.0	1	21	564	584	AGACUUAUAUGUAGGAACGGA	UCCGUUCUAAAUAUAAGUCU	Translation
tae-miR1122b-3p	Traes_3AS_D7F2F36BB.3						AT3G49720.1		LOC_Os01g05070.1	expressed protein	1,50	-1.0	1	21	540	560	AGACUUAUAUGUAGGAACGGA	UCCGUUCCUAAAUAUAAGUCU	Translation
tae-miR5200	Traes_3B_0E2173F88.1	PF14531	KOG0580	2.7.11.1			AT1G62400.1	Protein kinase superfamily protein	LOC_Os01g01740.1	protein kinase domain containing protein, expressed	2,50	-1.0	1	21	171	191	UGUAGAUACUCCCUAAGGCUU	UUCCCUUAGGAAGUAUCUACA	Translation
tae-miR5200	Traes_3B_0E2173F88.2	PF14531	KOG1164	2.7.11.1			AT1G62400.1	Protein kinase superfamily protein	LOC_Os01g01740.1	protein kinase domain containing protein, expressed	2,50	-1.0	1	21	177	197	UGUAGAUACUCCCUAAGGCUU	UUCCCUUAGGAAGUAUCUACA	Translation
tae-miR5200	Traes_3B_0E2173F88.3	PF14531		2.7.11.1			AT1G62400.1	Protein kinase superfamily protein	LOC_Os01g01740.1	protein kinase domain containing protein, expressed	2,50	-1.0	1	21	171	191	UGUAGAUACUCCCUAAGGCUU	UUCCCUUAGGAAGUAUCUACA	Translation
tae-miR9652-5p	Traes_3B_119F6A405.1	PF16487,PF16488,PF08699,PF02170				GO:GO:0005515	AT1G48410.1	Stabilizer of iron transporter SufD / Polynucleotidyl transferase	LOC_Os03g58600.1	PAZ domain containing protein, putative, expressed	2,50	-1.0	1	22	589	610	CCUGUUUGUCAUUAAGUUUCUU	AAGAAACUGAAUGACAAGCAGG	Cleavage
tae-miR9662a-3p	Traes_3B_224A6B7E5.1	PF02536				GO:GO:0006355,GO:GO:0005739,GO:GO:0003690	AT5G07900.1	Mitochondrial transcription termination factor family protein	LOC_Os06g12100.1	mTERF family protein, expressed	2,00	-1.0	1	21	189	209	UUGAACAUCCCAGAGCCACCG	CCGUGGUUCUGGGAUGUUCAG	Cleavage
tae-miR9662b-3p	Traes_3B_224A6B7E5.1	PF02536				GO:GO:0006355,GO:GO:0005739,GO:GO:0003690	AT5G07900.1	Mitochondrial transcription termination factor family protein	LOC_Os06g12100.1	mTERF family protein, expressed	2,00	-1.0	1	21	188	208	UGAACAUCCCAGAGCCACCGG	CCCGUGGUUCUGGGAUGUUCA	Cleavage
tae-miR159a	Traes_3B_25320ED72.1	PF00249	KOG0048		K09422		AT5G06100.1	myb domain protein 33	LOC_Os01g59660.2	MYB family transcription factor, putative, expressed	2,50	-1.0	1	21	956	976	UUUGGAUUGAAGGGAGCUCUG	UGGAGCUCCCUUCACUCCAAG	Cleavage
tae-miR159b	Traes_3B_25320ED72.1	PF00249	KOG0048		K09422		AT5G06100.1	myb domain protein 33	LOC_Os01g59660.2	MYB family transcription factor, putative, expressed	2,50	-1.0	1	21	956	976	UUUGGAUUGAAGGGAGCUCUG	UGGAGCUCCCUUCACUCCAAG	Cleavage
tae-miR159a	Traes_3B_25320ED72.2	PF00249	KOG0048		K09422		AT5G06100.1	myb domain protein 33	LOC_Os01g59660.1	MYB family transcription factor, putative, expressed	2,50	-1.0	1	21	956	976	UUUGGAUUGAAGGGAGCUCUG	UGGAGCUCCCUUCACUCCAAG	Cleavage
tae-miR159b	Traes_3B_25320ED72.2	PF00249	KOG0048		K09422		AT5G06100.1	myb domain protein 33	LOC_Os01g59660.1	MYB family transcription factor, putative, expressed	2,50	-1.0	1	21	956	976	UUUGGAUUGAAGGGAGCUCUG	UGGAGCUCCCUUCACUCCAAG	Cleavage
tae-miR1130b-3p	Traes_3B_467787390.8	PF00917,PF13423,PF14533,PF12436		3.4.19.12		GO:GO:0005515	AT3G11910.1	ubiquitin-specific protease 13	LOC_Os01g56490.1	ubiquitin carboxyl-terminal hydrolase, putative, expressed	1,50	-1.0	1	21	3080	3100	UCUUAUAUUAUGGGACGGAGG	CCUCCGUCCCAAAAUAUAAGA	Translation
tae-miR9776	Traes_3B_470061369.1	PF00270		3.6.4.13		GO:GO:0005524,GO:GO:0003676	AT5G51280.1	DEAD-box protein abstrakt, putative	LOC_Os02g05660.1	DEAD-box ATP-dependent RNA helicase 35A, putative, expressed	2,50	-1.0	1	21	34	54	UUGGACGAGGAUGUGCAACUG	AAGUGGCACAUCCUCGUCGAA	Cleavage
tae-miR1122a	Traes_3B_472072804.1	PF04874,PF01778	KOG3064		K14831		AT1G23280.1	MAK16 protein-related	LOC_Os07g16950.1	Mak16 protein domain containing protein, expressed	1,50	-1.0	1	20	789	808	UAGAUACAUCCGUAUCUAGA	UCUAGAUACGGAGGUAUCUA	Cleavage
tae-miR9778	Traes_3B_4C839468C.10						AT3G46530.1	NB-ARC domain-containing disease resistance protein	LOC_Os12g37290.1	resistance protein T10rga2-1A, putative, expressed	1,00	-1.0	1	21	126	146	UGCAUCAUCUCGAACUCGUCG	GGACGAGCUCGAGAUGAUGCA	Cleavage
tae-miR9778	Traes_3B_4C839468C.11						AT3G46530.1	NB-ARC domain-containing disease resistance protein	LOC_Os12g37290.1	resistance protein T10rga2-1A, putative, expressed	1,00	-1.0	1	21	126	146	UGCAUCAUCUCGAACUCGUCG	GGACGAGCUCGAGAUGAUGCA	Cleavage
tae-miR9778	Traes_3B_4C839468C.12						AT3G46530.1	NB-ARC domain-containing disease resistance protein	LOC_Os12g37290.1	resistance protein T10rga2-1A, putative, expressed	1,00	-1.0	1	21	126	146	UGCAUCAUCUCGAACUCGUCG	GGACGAGCUCGAGAUGAUGCA	Cleavage
tae-miR9778	Traes_3B_4C839468C.13						AT1G50180.1	NB-ARC domain-containing disease resistance protein	LOC_Os02g57310.1	pib, putative, expressed	1,00	-1.0	1	21	102	122	UGCAUCAUCUCGAACUCGUCG	GGACGAGCUCGAGAUGAUGCA	Cleavage
tae-miR9778	Traes_3B_4C839468C.2						AT3G46530.1	NB-ARC domain-containing disease resistance protein	LOC_Os12g37290.1	resistance protein T10rga2-1A, putative, expressed	1,00	-1.0	1	21	126	146	UGCAUCAUCUCGAACUCGUCG	GGACGAGCUCGAGAUGAUGCA	Cleavage
tae-miR9778	Traes_3B_4C839468C.3						AT3G46530.1	NB-ARC domain-containing disease resistance protein	LOC_Os12g37290.1	resistance protein T10rga2-1A, putative, expressed	1,00	-1.0	1	21	126	146	UGCAUCAUCUCGAACUCGUCG	GGACGAGCUCGAGAUGAUGCA	Cleavage
tae-miR9778	Traes_3B_4C839468C.4						AT3G46530.1	NB-ARC domain-containing disease resistance protein	LOC_Os12g37290.1	resistance protein T10rga2-1A, putative, expressed	1,00	-1.0	1	21	126	146	UGCAUCAUCUCGAACUCGUCG	GGACGAGCUCGAGAUGAUGCA	Cleavage
tae-miR9778	Traes_3B_4C839468C.5						AT3G46530.1	NB-ARC domain-containing disease resistance protein	LOC_Os12g37290.1	resistance protein T10rga2-1A, putative, expressed	1,00	-1.0	1	21	126	146	UGCAUCAUCUCGAACUCGUCG	GGACGAGCUCGAGAUGAUGCA	Cleavage
tae-miR9778	Traes_3B_4C839468C.6						AT3G46530.1	NB-ARC domain-containing disease resistance protein	LOC_Os12g37290.1	resistance protein T10rga2-1A, putative, expressed	1,00	-1.0	1	21	126	146	UGCAUCAUCUCGAACUCGUCG	GGACGAGCUCGAGAUGAUGCA	Cleavage
tae-miR9778	Traes_3B_4C839468C.7						AT3G46530.1	NB-ARC domain-containing disease resistance protein	LOC_Os12g37290.1	resistance protein T10rga2-1A, putative, expressed	1,00	-1.0	1	21	126	146	UGCAUCAUCUCGAACUCGUCG	GGACGAGCUCGAGAUGAUGCA	Cleavage
tae-miR9778	Traes_3B_4C839468C.8						AT3G46530.1	NB-ARC domain-containing disease resistance protein	LOC_Os12g37290.1	resistance protein T10rga2-1A, putative, expressed	1,00	-1.0	1	21	126	146	UGCAUCAUCUCGAACUCGUCG	GGACGAGCUCGAGAUGAUGCA	Cleavage
tae-miR9778	Traes_3B_4C839468C.9						AT3G46530.1	NB-ARC domain-containing disease resistance protein	LOC_Os12g37290.1	resistance protein T10rga2-1A, putative, expressed	1,00	-1.0	1	21	126	146	UGCAUCAUCUCGAACUCGUCG	GGACGAGCUCGAGAUGAUGCA	Cleavage
tae-miR1137a	Traes_3B_59B827C5C.2	PF01912	KOG3185			GO:GO:0043022,GO:GO:0042256	AT3G55620.1	Translation initiation factor IF6	LOC_Os07g44620.2	eukaryotic translation initiation factor 6, putative, expressed	0,00	-1.0	1	20	748	767	UAGUACAAAGUUGAGUCAUC	GAUGACUCAACUUUGUACUA	Cleavage
tae-miR1122b-3p	Traes_3B_5DD9C1BE5.1								LOC_Os01g40830.1	transposon protein, putative, Mutator sub-class, expressed	2,50	-1.0	1	21	71	91	AGACUUAUAUGUAGGAACGGA	UCCGUUCUUAAAUAUAAGUCU	Translation
tae-miR7757-5p	Traes_3B_5EFB9A61F.1								LOC_Os12g17490.1	stripe rust resistance protein Yr10, putative, expressed	1,50	-1.0	1	22	113	134	AUAAAACCUUCAGCUAUCCAUC	GAUGGAUAGCUGAAGGUUUUGU	Cleavage
tae-miR7757-5p	Traes_3B_5EFB9A61F.3								LOC_Os12g17490.1	stripe rust resistance protein Yr10, putative, expressed	1,50	-1.0	1	22	89	110	AUAAAACCUUCAGCUAUCCAUC	GAUGGAUAGCUGAAGGUUUUGU	Cleavage
tae-miR9676-5p	Traes_3B_5F0E1F6A4.1						AT1G52700.1	alpha/beta-Hydrolases superfamily protein	LOC_Os05g51050.1	acyl-protein thioesterase, putative, expressed	2,50	-1.0	1	22	38	59	UGGAUGUCAUCGUGGCCGUACA	UGUACGGACAUGAUGACAUCCA	Cleavage
tae-miR1122b-3p	Traes_3B_6D2DCE280.3	PF00025	KOG0084			GO:GO:0005525	AT4G17530.1	RAB GTPase homolog 1C	LOC_Os01g37800.1	ras-related protein, putative, expressed	2,50	-1.0	1	21	576	596	AGACUUAUAUGUAGGAACGGA	UCUGUUCCUAAAUAUAAGUCU	Translation
tae-miR7757-5p	Traes_3B_7FFFF7BCF.1								LOC_Os02g18510.1	stripe rust resistance protein Yr10, putative, expressed	0,00	-1.0	1	22	89	110	AUAAAACCUUCAGCUAUCCAUC	GAUGGAUAGCUGAAGGUUUUAU	Cleavage
tae-miR531	Traes_3B_879F704A0.1										2,50	-1.0	1	21	155	175	CGCUCGCCGGAGCAGCGUGCA	UCCGCGCUGCUCCGGCGAGGG	Cleavage
tae-miR531	Traes_3B_879F704A0.2										2,50	-1.0	1	21	155	175	CGCUCGCCGGAGCAGCGUGCA	UCCGCGCUGCUCCGGCGAGGG	Cleavage
tae-miR531	Traes_3B_879F704A0.3										2,50	-1.0	1	21	50	70	CGCUCGCCGGAGCAGCGUGCA	UCCGCGCUGCUCCGGCGAGGG	Cleavage
tae-miR531	Traes_3B_879F704A0.4										2,50	-1.0	1	21	50	70	CGCUCGCCGGAGCAGCGUGCA	UCCGCGCUGCUCCGGCGAGGG	Cleavage
tae-miR5049-3p	Traes_3B_9D776A8C4.1	PF00804				GO:GO:0016020	AT3G24350.2	syntaxin of plants 32	LOC_Os01g08400.1	syntaxin, putative, expressed	0,00	-1.0	1	21	575	595	AAUAUGGAUCGGAGGGAGUAC	GUACUCCCUCCGAUCCAUAUU	Cleavage
tae-miR1121	Traes_3B_9F321609E.8										1,50	-1.0	1	22	6	28	AGUAGUGAUCUAAACGCUCU-UA	UAUAGAGCGUUUAGAUCACUAUU	Cleavage
tae-miR9778	Traes_3B_F07C33521.1						AT3G46710.1	NB-ARC domain-containing disease resistance protein	LOC_Os02g57310.1	pib, putative, expressed	1,00	-1.0	1	21	126	146	UGCAUCAUCUCGAACUCGUCG	GGACGAGCUCGAGAUGAUGCA	Cleavage
tae-miR9778	Traes_3B_F07C33521.10						AT3G46710.1	NB-ARC domain-containing disease resistance protein	LOC_Os02g57310.1	pib, putative, expressed	1,00	-1.0	1	21	126	146	UGCAUCAUCUCGAACUCGUCG	GGACGAGCUCGAGAUGAUGCA	Cleavage
tae-miR9778	Traes_3B_F07C33521.2	PF00931				GO:GO:0043531	AT3G46530.1	NB-ARC domain-containing disease resistance protein	LOC_Os08g42670.2	resistance protein, putative, expressed	1,00	-1.0	1	21	126	146	UGCAUCAUCUCGAACUCGUCG	GGACGAGCUCGAGAUGAUGCA	Cleavage
tae-miR9778	Traes_3B_F07C33521.3	PF00931				GO:GO:0043531	AT3G46530.1	NB-ARC domain-containing disease resistance protein	LOC_Os08g42670.2	resistance protein, putative, expressed	1,00	-1.0	1	21	126	146	UGCAUCAUCUCGAACUCGUCG	GGACGAGCUCGAGAUGAUGCA	Cleavage
tae-miR9778	Traes_3B_F07C33521.4	PF00931				GO:GO:0043531	AT3G46530.1	NB-ARC domain-containing disease resistance protein	LOC_Os08g42670.2	resistance protein, putative, expressed	1,00	-1.0	1	21	126	146	UGCAUCAUCUCGAACUCGUCG	GGACGAGCUCGAGAUGAUGCA	Cleavage
tae-miR9778	Traes_3B_F07C33521.5	PF00931				GO:GO:0043531	AT3G46530.1	NB-ARC domain-containing disease resistance protein	LOC_Os08g42670.1	resistance protein, putative, expressed	1,00	-1.0	1	21	126	146	UGCAUCAUCUCGAACUCGUCG	GGACGAGCUCGAGAUGAUGCA	Cleavage
tae-miR9778	Traes_3B_F07C33521.6	PF00931				GO:GO:0043531	AT3G46530.1	NB-ARC domain-containing disease resistance protein	LOC_Os08g42670.1	resistance protein, putative, expressed	1,00	-1.0	1	21	126	146	UGCAUCAUCUCGAACUCGUCG	GGACGAGCUCGAGAUGAUGCA	Cleavage
tae-miR9778	Traes_3B_F07C33521.7	PF00931				GO:GO:0043531	AT3G46530.1	NB-ARC domain-containing disease resistance protein	LOC_Os08g42670.2	resistance protein, putative, expressed	1,00	-1.0	1	21	126	146	UGCAUCAUCUCGAACUCGUCG	GGACGAGCUCGAGAUGAUGCA	Cleavage
tae-miR9778	Traes_3B_F07C33521.8						AT3G46710.1	NB-ARC domain-containing disease resistance protein	LOC_Os02g57310.1	pib, putative, expressed	1,00	-1.0	1	21	126	146	UGCAUCAUCUCGAACUCGUCG	GGACGAGCUCGAGAUGAUGCA	Cleavage
tae-miR9778	Traes_3B_F07C33521.9						AT3G46710.1	NB-ARC domain-containing disease resistance protein	LOC_Os02g57310.1	pib, putative, expressed	1,00	-1.0	1	21	126	146	UGCAUCAUCUCGAACUCGUCG	GGACGAGCUCGAGAUGAUGCA	Cleavage
tae-miR1128	Traes_3B_F61EDC1AE.3	PF02212,PF00350,PF01031				GO:GO:0005525,GO:GO:0003924	AT4G33650.1	dynamin-related protein 3A	LOC_Os01g69130.1	dynamin family protein, putative, expressed	2,00	-1.0	1	21	1581	1601	UACUACUCCCUCCGUCCGAAA	UUCCGAACGGAGGGAGUAGUA	Cleavage
tae-miR1127a	Traes_3B_F61EDC1AE.3	PF02212,PF00350,PF01031				GO:GO:0005525,GO:GO:0003924	AT4G33650.1	dynamin-related protein 3A	LOC_Os01g69130.1	dynamin family protein, putative, expressed	1,50	-1.0	1	19	1577	1595	UCCUUCCGUUCGGAAUUAC	GUAAUUCCGAACGGAGGGA	Cleavage
tae-miR1130b-3p	Traes_3B_FB51F185F.2						AT1G53560.1	Ribosomal protein L18ae family	LOC_Os01g14070.1	60S ribosomal protein L18a-1, putative, expressed	1,50	-1.0	1	21	43	63	UCUUAUAUUAUGGGACGGAGG	CCUCCGUCCCAUAAUGUAAGA	Cleavage
tae-miR164	Traes_3B_FD8ACC50C.1			6.3.2.19		GO:GO:0007275,GO:GO:0006511,GO:GO:0005634	AT1G66650.1	Protein with RING/U-box and TRAF-like domains	LOC_Os01g03190.1	expressed protein	2,50	-1.0	1	21	1544	1564	UGGAGAAGCAGGGCACGUGCA	CGCACGGGCCCUGCUUCUGCA	Cleavage
tae-miR164	Traes_3B_FD8ACC50C.2			6.3.2.19		GO:GO:0007275,GO:GO:0006511,GO:GO:0005634	AT1G66650.1	Protein with RING/U-box and TRAF-like domains	LOC_Os01g03190.1	expressed protein	2,50	-1.0	1	21	1499	1519	UGGAGAAGCAGGGCACGUGCA	CGCACGGGCCCUGCUUCUGCA	Cleavage
tae-miR164	Traes_3B_FD8ACC50C.3			6.3.2.19		GO:GO:0007275,GO:GO:0006511,GO:GO:0005634	AT1G66650.1	Protein with RING/U-box and TRAF-like domains	LOC_Os01g03190.1	expressed protein	2,50	-1.0	1	21	1433	1453	UGGAGAAGCAGGGCACGUGCA	CGCACGGGCCCUGCUUCUGCA	Cleavage
tae-miR164	Traes_3B_FD8ACC50C.4			6.3.2.19		GO:GO:0007275,GO:GO:0006511,GO:GO:0005634	AT1G66650.1	Protein with RING/U-box and TRAF-like domains	LOC_Os01g03190.1	expressed protein	2,50	-1.0	1	21	764	784	UGGAGAAGCAGGGCACGUGCA	CGCACGGGCCCUGCUUCUGCA	Cleavage
tae-miR164	Traes_3B_FD8ACC50C.5			6.3.2.19		GO:GO:0007275,GO:GO:0006511,GO:GO:0005634	AT1G66650.1	Protein with RING/U-box and TRAF-like domains	LOC_Os01g03190.1	expressed protein	2,50	-1.0	1	21	752	772	UGGAGAAGCAGGGCACGUGCA	CGCACGGGCCCUGCUUCUGCA	Cleavage
tae-miR164	Traes_3B_FD8ACC50C.6			6.3.2.19		GO:GO:0007275,GO:GO:0006511,GO:GO:0005634	AT1G66650.1	Protein with RING/U-box and TRAF-like domains	LOC_Os01g03190.1	expressed protein	2,50	-1.0	1	21	731	751	UGGAGAAGCAGGGCACGUGCA	CGCACGGGCCCUGCUUCUGCA	Cleavage
tae-miR164	Traes_3B_FD8ACC50C.7			6.3.2.19		GO:GO:0007275,GO:GO:0006511,GO:GO:0005634	AT1G66650.1	Protein with RING/U-box and TRAF-like domains	LOC_Os01g03190.1	expressed protein	2,50	-1.0	1	21	710	730	UGGAGAAGCAGGGCACGUGCA	CGCACGGGCCCUGCUUCUGCA	Cleavage
tae-miR164	Traes_3B_FD8ACC50C.8			6.3.2.19		GO:GO:0007275,GO:GO:0006511,GO:GO:0005634	AT1G66650.1	Protein with RING/U-box and TRAF-like domains	LOC_Os01g03190.1	expressed protein	2,50	-1.0	1	21	653	673	UGGAGAAGCAGGGCACGUGCA	CGCACGGGCCCUGCUUCUGCA	Cleavage
tae-miR164	Traes_3B_FD8ACC50C.9			6.3.2.19		GO:GO:0007275,GO:GO:0006511,GO:GO:0005634	AT1G66650.1	Protein with RING/U-box and TRAF-like domains	LOC_Os01g03190.1	expressed protein	2,50	-1.0	1	21	356	376	UGGAGAAGCAGGGCACGUGCA	CGCACGGGCCCUGCUUCUGCA	Cleavage
tae-miR5049-3p	Traes_3DL_2B3694DC3.2	PF01217					AT3G09800.1	SNARE-like superfamily protein	LOC_Os05g38610.1	clathrin adaptor complex small chain domain containing protein, expressed	1,50	-1.0	1	21	228	248	AAUAUGGAUCGGAGGGAGUAC	CUACUCCCUCCGAUCCAAAUU	Cleavage
tae-miR1130b-3p	Traes_3DL_426EF6A68.2										2,50	-1.0	1	21	214	234	UCUUAUAUUAUGGGACGGAGG	CCUCCAUCCCGUAAUAUAAGA	Translation
tae-miR7757-5p	Traes_3DL_5C0BC5611.4								LOC_Os12g17490.1	stripe rust resistance protein Yr10, putative, expressed	1,50	-1.0	1	22	89	110	AUAAAACCUUCAGCUAUCCAUC	GAUGGAUAGCUGAAGGUUUUGU	Cleavage
tae-miR1137a	Traes_3DL_96847DCCF.1	PF00069	KOG1187	2.7.11.1		GO:GO:0006468,GO:GO:0005524,GO:GO:0004672	AT1G34300.1	lectin protein kinase family protein	LOC_Os01g47840.1	S-locus-like receptor protein kinase, putative, expressed	1,00	-1.0	1	20	88	107	UAGUACAAAGUUGAGUCAUC	GAUGACCCAACUUUGUACUA	Cleavage
tae-miR5049-3p	Traes_3DL_D1CF4784D.2	PF13307,PF06733		3.6.4.12		GO:GO:0016818,GO:GO:0008026,GO:GO:0006139,GO:GO:0005524,GO:GO:0003676,GO:GO:0004003,GO:GO:0003677	AT1G79950.1	RAD3-like DNA-binding helicase protein	LOC_Os01g40980.1	helicase, putative, expressed	2,50	-1.0	1	21	1646	1666	AAUAUGGAUCGGAGGGAGUAC	AUAAUCCCUCCGUUCCAUAUU	Cleavage
tae-miR159a	Traes_3DL_D9A29948B.1	PF00249					AT5G06100.1	myb domain protein 33	LOC_Os01g59660.1	MYB family transcription factor, putative, expressed	2,50	-1.0	1	21	620	640	UUUGGAUUGAAGGGAGCUCUG	UGGAGCUCCCUUCACUCCAAG	Cleavage
tae-miR159b	Traes_3DL_D9A29948B.1	PF00249					AT5G06100.1	myb domain protein 33	LOC_Os01g59660.1	MYB family transcription factor, putative, expressed	2,50	-1.0	1	21	620	640	UUUGGAUUGAAGGGAGCUCUG	UGGAGCUCCCUUCACUCCAAG	Cleavage
tae-miR159a	Traes_3DL_D9A29948B.2	PF00249					AT5G06100.1	myb domain protein 33	LOC_Os01g59660.1	MYB family transcription factor, putative, expressed	2,50	-1.0	1	21	620	640	UUUGGAUUGAAGGGAGCUCUG	UGGAGCUCCCUUCACUCCAAG	Cleavage
tae-miR159b	Traes_3DL_D9A29948B.2	PF00249					AT5G06100.1	myb domain protein 33	LOC_Os01g59660.1	MYB family transcription factor, putative, expressed	2,50	-1.0	1	21	620	640	UUUGGAUUGAAGGGAGCUCUG	UGGAGCUCCCUUCACUCCAAG	Cleavage
tae-miR1128	Traes_3DS_1DE20CFF3.2	PF01121	KOG3220	2.7.1.24	K00859	GO:GO:0015937,GO:GO:0005524,GO:GO:0004140	AT2G27490.1	dephospho-CoA kinase family	LOC_Os01g25880.1	dephospho-CoA kinase, putative, expressed	2,50	-1.0	1	21	71	91	UACUACUCCCUCCGUCCGAAA	UUUGGGACGGAGGGAGUAGUA	Cleavage
tae-miR1122a	Traes_3DS_1DE20CFF3.2	PF01121	KOG3220	2.7.1.24	K00859	GO:GO:0015937,GO:GO:0005524,GO:GO:0004140	AT2G27490.1	dephospho-CoA kinase family	LOC_Os01g25880.1	dephospho-CoA kinase, putative, expressed	1,50	-1.0	1	20	2	21	UAGAUACAUCCGUAUCUAGA	UCUAGAUACAGAUGUAUCUA	Translation
tae-miR1128	Traes_3DS_1DE20CFF3.3	PF01121	KOG3220	2.7.1.24	K00859	GO:GO:0015937,GO:GO:0005524,GO:GO:0004140	AT2G27490.1	dephospho-CoA kinase family	LOC_Os01g25880.1	dephospho-CoA kinase, putative, expressed	2,50	-1.0	1	21	71	91	UACUACUCCCUCCGUCCGAAA	UUUGGGACGGAGGGAGUAGUA	Cleavage
tae-miR1122a	Traes_3DS_1DE20CFF3.3	PF01121	KOG3220	2.7.1.24	K00859	GO:GO:0015937,GO:GO:0005524,GO:GO:0004140	AT2G27490.1	dephospho-CoA kinase family	LOC_Os01g25880.1	dephospho-CoA kinase, putative, expressed	1,50	-1.0	1	20	2	21	UAGAUACAUCCGUAUCUAGA	UCUAGAUACAGAUGUAUCUA	Translation
tae-miR1122b-3p	Traes_3DS_D42FCD586.1	PF00274		4.1.2.13		GO:GO:0006096,GO:GO:0004332	AT2G01140.1	Aldolase superfamily protein	LOC_Os01g02880.1	fructose-bisphospate aldolase isozyme, putative, expressed	1,50	-1.0	1	21	786	806	AGACUUAUAUGUAGGAACGGA	UCCGUUCCUAAAUAUAAGUCU	Translation
tae-miR1122b-3p	Traes_3DS_D42FCD586.2	PF00274		4.1.2.13		GO:GO:0006096,GO:GO:0004332	AT2G01140.1	Aldolase superfamily protein	LOC_Os01g02880.1	fructose-bisphospate aldolase isozyme, putative, expressed	1,50	-1.0	1	21	795	815	AGACUUAUAUGUAGGAACGGA	UCCGUUCUAAAUAUAAGUCU	Translation
tae-miR7757-5p	Traes_4AL_13A3A40D3.1	PF13401	KOG4658				AT3G50950.2	HOPZ-ACTIVATED RESISTANCE 1	LOC_Os11g11810.1	NBS-LRR disease resistance protein, putative, expressed	1,50	-1.0	1	22	1337	1358	AUAAAACCUUCAGCUAUCCAUC	AGUGGAUAGCUGAAGGUUUCAU	Cleavage
tae-miR1135	Traes_4AL_158B970F4.1						AT1G50020.1		LOC_Os11g34870.1	expressed protein	2,50	-1.0	1	24	464	487	CUGCGACAAGUAAUUCCGAACGGA	UCCGUUCCGAAUUACUCGUCGCAG	Cleavage
tae-miR5175-5p	Traes_4AL_158B970F4.1						AT1G50020.1		LOC_Os11g34870.1	expressed protein	3,00	-1.0	1	21	532	552	UUCCAAAUUACUCGUCGUGGU	UCUGAGACGAGUAAUUUGGAA	Cleavage
tae-miR1117	Traes_4AL_2475C298D.1	PF07993		1.2.1.84		GO:GO:0080019	AT3G44540.1	fatty acid reductase 4	LOC_Os04g28600.1	male sterility protein, putative, expressed	2,50	-1.0	1	24	684	707	UAGUACCGGUUCGUGGCACGAACC	GGUUCAUGCCAUGAACCGGUACUA	Cleavage
tae-miR9778	Traes_4AL_2FC39B934.1	PF13191,PF00931				GO:GO:0043531	AT1G53350.1	Disease resistance protein (CC-NBS-LRR class) family	LOC_Os08g14850.1	resistance protein, putative, expressed	2,50	-1.0	1	21	126	146	UGCAUCAUCUCGAACUCGUCG	UGGCGAGUUCCAGAUGAUGCA	Translation
tae-miR408	Traes_4AL_364F56EDB.1										0,00	-1.0	1	21	8	28	CUGCACUGCCUCUUCCCUGGC	CCCAGGGAAGAGGCAGUGCAG	Cleavage
tae-miR9662a-3p	Traes_4AL_3A009739E.1	PF02536				GO:GO:0006355,GO:GO:0005739,GO:GO:0003690	AT1G21150.1	Mitochondrial transcription termination factor family protein	LOC_Os06g12100.1	mTERF family protein, expressed	2,50	-1.0	1	21	66	86	UUGAACAUCCCAGAGCCACCG	CCGUGGCUCUGGAAUGUUCAG	Cleavage
tae-miR9662b-3p	Traes_4AL_3A009739E.1	PF02536				GO:GO:0006355,GO:GO:0005739,GO:GO:0003690	AT1G21150.1	Mitochondrial transcription termination factor family protein	LOC_Os06g12100.1	mTERF family protein, expressed	2,50	-1.0	1	21	65	85	UGAACAUCCCAGAGCCACCGG	CCCGUGGCUCUGGAAUGUUCA	Cleavage
tae-miR1121	Traes_4AL_400EB3C17.10	PF14259,PF00076	KOG0120			GO:GO:0003676	AT4G36690.4	U2 snRNP auxilliary factor, large subunit, splicing factor	LOC_Os11g41820.2	RNA recognition motif containing protein, expressed	2,50	-1.0	1	22	10	31	AGUAGUGAUCUAAACGCUCUUA	UAAGAGCGUUUAGAUCACUAAA	Cleavage
tae-miR1121	Traes_4AL_400EB3C17.3	PF14259,PF00076	KOG0120			GO:GO:0003676	AT4G36690.1	U2 snRNP auxilliary factor, large subunit, splicing factor	LOC_Os11g41820.2	RNA recognition motif containing protein, expressed	2,50	-1.0	1	22	37	58	AGUAGUGAUCUAAACGCUCUUA	UAAGAGCGUUUAGAUCACUAAA	Cleavage
tae-miR1121	Traes_4AL_400EB3C17.7	PF14259,PF00076	KOG0120			GO:GO:0003676	AT4G36690.4	U2 snRNP auxilliary factor, large subunit, splicing factor	LOC_Os11g41820.2	RNA recognition motif containing protein, expressed	2,50	-1.0	1	22	10	31	AGUAGUGAUCUAAACGCUCUUA	UAAGAGCGUUUAGAUCACUAAA	Cleavage
tae-miR1137a	Traes_4AL_40790E994.6	PF05185		2.1.1.125,2.1.1.126		GO:GO:0008168,GO:GO:0006479	AT4G16570.1	protein arginine methyltransferase 7	LOC_Os06g01640.1	arginine N-methyltransferase 7, putative, expressed	1,00	-1.0	1	20	1324	1343	UAGUACAAAGUUGAGUCAUC	GAUGACCCAACUUUGUACUA	Cleavage
tae-miR1137a	Traes_4AL_40790E994.7			2.1.1.125,2.1.1.126		GO:GO:0008168,GO:GO:0006479	AT4G16570.1	protein arginine methyltransferase 7	LOC_Os06g01640.1	arginine N-methyltransferase 7, putative, expressed	1,00	-1.0	1	20	835	854	UAGUACAAAGUUGAGUCAUC	GAUGACCCAACUUUGUACUA	Cleavage
tae-miR1127b-3p	Traes_4AL_44E99BA01.2	PF07714		2.7.11.1		GO:GO:0006468,GO:GO:0004672	AT4G33950.1	Protein kinase superfamily protein	LOC_Os03g41460.1	CAMK_CAMK_like.20 - CAMK includes calcium/calmodulin depedent protein kinases, expressed	1,50	-1.0	1	21	394	414	ACAAGUAUUUCUGGACGGAGG	CCUCCGUCCGGAAAUACUUGU	Cleavage
tae-miR1121	Traes_4AL_541C86EB9.2	PF00076				GO:GO:0003676,GO:GO:0017069,GO:GO:0000398	AT1G21320.1	nucleotide binding;nucleic acid binding	LOC_Os06g03690.2	RNA recognition motif containing protein, putative, expressed	1,00	-1.0	1	22	558	579	AGUAGUGAUCUAAACGCUCUUA	UGAGAUCGUUUAGAUCACUACU	Cleavage
tae-miR1121	Traes_4AL_541C86EB9.3	PF00076				GO:GO:0003676,GO:GO:0017069,GO:GO:0000398	AT1G21320.1	nucleotide binding;nucleic acid binding	LOC_Os06g03690.2	RNA recognition motif containing protein, putative, expressed	1,00	-1.0	1	22	558	579	AGUAGUGAUCUAAACGCUCUUA	UGAGAUCGUUUAGAUCACUACU	Cleavage
tae-miR1122b-3p	Traes_4AL_6252B0BA0.1	PF05562					AT1G29395.1	COLD REGULATED 314 INNER MEMBRANE 1	LOC_Os05g49170.1	cold acclimation protein WCOR413, putative, expressed	1,50	-1.0	1	21	46	66	AGACUUAUAUGUAGGAACGGA	UCCGUUCCUAAAUAUAAGUCU	Translation
tae-miR1137b-5p	Traes_4AL_748BB4675.1	PF00657		3.1.1.1		GO:GO:0016788	AT5G45910.1	GDSL-like Lipase/Acylhydrolase superfamily protein	LOC_Os01g12320.1	GDSL-like lipase/acylhydrolase, putative, expressed	2,50	-1.0	1	21	844	864	UCCGUUCCAGAAUAGAUGACC	ACUCAUCUAUUCUGGGACGGU	Cleavage
tae-miR9778	Traes_4AL_79B47AF61.1	PF13191,PF00931				GO:GO:0043531	AT3G07040.1	NB-ARC domain-containing disease resistance protein	LOC_Os08g42670.1	resistance protein, putative, expressed	2,50	-1.0	1	21	126	146	UGCAUCAUCUCGAACUCGUCG	GGACGAGCUCGACAUGAUGCA	Cleavage
tae-miR9778	Traes_4AL_79B47AF61.3	PF00931				GO:GO:0043531	AT3G46530.1	NB-ARC domain-containing disease resistance protein	LOC_Os08g42670.2	resistance protein, putative, expressed	2,50	-1.0	1	21	126	146	UGCAUCAUCUCGAACUCGUCG	GGACGAGCUCGACAUGAUGCA	Cleavage
tae-miR9778	Traes_4AL_79B47AF61.4	PF00931				GO:GO:0043531	AT3G46530.1	NB-ARC domain-containing disease resistance protein	LOC_Os08g42670.2	resistance protein, putative, expressed	2,50	-1.0	1	21	126	146	UGCAUCAUCUCGAACUCGUCG	GGACGAGCUCGACAUGAUGCA	Cleavage
tae-miR5048-5p	Traes_4AL_85A7C60F9.1	PF00069		2.7.11.1		GO:GO:0006468,GO:GO:0005524,GO:GO:0004672	AT4G21230.1	cysteine-rich RLK (RECEPTOR-like protein kinase) 27	LOC_Os11g10640.1	protein kinase domain containing protein, expressed	2,50	-1.0	1	22	170	191	UUUGCAGGUUUUAGGUCUAAGU	ACUUGGACCUAAAACCUGCGAA	Cleavage
tae-miR5048-5p	Traes_4AL_85A7C60F9.7	PF00069		2.7.11.1		GO:GO:0006468,GO:GO:0005524,GO:GO:0004672	AT4G21230.1	cysteine-rich RLK (RECEPTOR-like protein kinase) 27	LOC_Os11g10640.1	protein kinase domain containing protein, expressed	2,50	-1.0	1	22	170	191	UUUGCAGGUUUUAGGUCUAAGU	ACUUGGACCUAAAACCUGCGAA	Cleavage
tae-miR5048-5p	Traes_4AL_85A7C60F9.8	PF00069		2.7.11.1		GO:GO:0006468,GO:GO:0005524,GO:GO:0004672	AT4G21230.1	cysteine-rich RLK (RECEPTOR-like protein kinase) 27	LOC_Os11g10640.1	protein kinase domain containing protein, expressed	2,50	-1.0	1	22	170	191	UUUGCAGGUUUUAGGUCUAAGU	ACUUGGACCUAAAACCUGCGAA	Cleavage
tae-miR1122b-3p	Traes_4AL_954AE4933.1	PF12171					AT2G02080.1	indeterminate(ID)-domain 4	LOC_Os04g47860.4	ZOS4-11 - C2H2 zinc finger protein, expressed	1,50	-1.0	1	21	583	603	AGACUUAUAUGUAGGAACGGA	UCCGUUCCUAAAUAUAAGUCU	Translation
tae-miR1122a	Traes_4AL_9726307FF.1	PF01926,PF06421,PF00679		3.6.5.3		GO:GO:0005525	AT5G39900.1	Small GTP-binding protein	LOC_Os06g05250.2	GTP-binding protein GUF1, putative, expressed	0,00	-1.0	1	20	1903	1922	UAGAUACAUCCGUAUCUAGA	UCUAGAUACGGAUGUAUCUA	Cleavage
tae-miR1128	Traes_4AL_99942CBEA.1	PF02365				GO:GO:0006355,GO:GO:0003677	AT2G46770.1	NAC (No Apical Meristem) domain transcriptional regulator superfamily protein	LOC_Os06g04090.1	no apical meristem protein, putative, expressed	3,00	-1.0	1	21	693	714	UACUACUCCCUCCG-UCCGAAA	UUUAGGAACGGAGGGAGUAGUA	Cleavage
tae-miR1122b-3p	Traes_4AL_99942CBEA.1	PF02365				GO:GO:0006355,GO:GO:0003677	AT2G46770.1	NAC (No Apical Meristem) domain transcriptional regulator superfamily protein	LOC_Os06g04090.1	no apical meristem protein, putative, expressed	2,50	-1.0	1	21	557	577	AGACUUAUAUGUAGGAACGGA	UCCGUUCUUAAAUAUAAGUCU	Translation
tae-miR9662a-3p	Traes_4AL_A4B73CC6A1.1	PF02536	KOG1267		K15032	GO:GO:0006355,GO:GO:0005739,GO:GO:0003690	AT5G07900.1	Mitochondrial transcription termination factor family protein	LOC_Os06g12100.1	mTERF family protein, expressed	2,50	-1.0	1	21	693	713	UUGAACAUCCCAGAGCCACCG	CCGUGGCUCUGGAAUGUUCAG	Cleavage

tae-miR9662b-3p	Traes_4AL_A4B73CC6A1.1	PF02536	KOG1267		K15032	GO:GO:0006355,GO:GO:0005739,GO:GO:0003690	AT5G07900.1	Mitochondrial transcription termination factor family protein	LOC_Os06g12100.1	mTERF family protein, expressed	2,50	-1.0	1	21	692	712	UGAACAUCCCAGAGCCACCGG	CCCGUGGCUCUGGAAUGUUCA	Cleavage
tae-miR9662a-3p	Traes_4AL_A4B73CC6A1.2	PF02536	KOG1267		K15032	GO:GO:0006355,GO:GO:0005739,GO:GO:0003690	AT5G07900.1	Mitochondrial transcription termination factor family protein	LOC_Os06g12100.1	mTERF family protein, expressed	2,50	-1.0	1	21	693	713	UUGAACAUCCCAGAGCCACCG	CCGUGGCUCUGGAAUGUUCAG	Cleavage
tae-miR9662b-3p	Traes_4AL_A4B73CC6A1.2	PF02536	KOG1267		K15032	GO:GO:0006355,GO:GO:0005739,GO:GO:0003690	AT5G07900.1	Mitochondrial transcription termination factor family protein	LOC_Os06g12100.1	mTERF family protein, expressed	2,50	-1.0	1	21	692	712	UGAACAUCCCAGAGCCACCGG	CCCGUGGCUCUGGAAUGUUCA	Cleavage
tae-miR1130b-3p	Traes_4AL_C55D89913.1	PF03219				GO:GO:0016021,GO:GO:0006810,GO:GO:0005524,GO:GO:0005471	AT5G05310.2	TLC ATP/ADP transporter	LOC_Os11g02159.1	expressed protein	0,00	-1.0	1	21	10	30	UCUUAUAUUAUGGGACGGAGG	CCUCCGUCCCAUAAUAUAAGA	Cleavage
tae-miR1122c-3p	Traes_4AL_C55D89913.1	PF03219				GO:GO:0016021,GO:GO:0006810,GO:GO:0005524,GO:GO:0005471	AT5G05310.2	TLC ATP/ADP transporter	LOC_Os11g02159.1	expressed protein	1,50	-1.0	1	21	10	30	UCUAAUAUUAUGGGACGGAGG	CCUCCGUCCCAUAAUAUAAGA	Cleavage
tae-miR1130b-3p	Traes_4AL_C55D89913.5	PF03219				GO:GO:0016021,GO:GO:0006810,GO:GO:0005524,GO:GO:0005471	AT5G05310.2	TLC ATP/ADP transporter	LOC_Os11g02159.2	expressed protein	0,00	-1.0	1	21	10	30	UCUUAUAUUAUGGGACGGAGG	CCUCCGUCCCAUAAUAUAAGA	Cleavage
tae-miR1122c-3p	Traes_4AL_C55D89913.5	PF03219				GO:GO:0016021,GO:GO:0006810,GO:GO:0005524,GO:GO:0005471	AT5G05310.2	TLC ATP/ADP transporter	LOC_Os11g02159.2	expressed protein	1,50	-1.0	1	21	10	30	UCUAAUAUUAUGGGACGGAGG	CCUCCGUCCCAUAAUAUAAGA	Cleavage
tae-miR1130b-3p	Traes_4AL_C55D89913.6	PF03219				GO:GO:0016021,GO:GO:0006810,GO:GO:0005524,GO:GO:0005471	AT5G05310.2	TLC ATP/ADP transporter	LOC_Os11g02159.1	expressed protein	0,00	-1.0	1	21	10	30	UCUUAUAUUAUGGGACGGAGG	CCUCCGUCCCAUAAUAUAAGA	Cleavage
tae-miR1122c-3p	Traes_4AL_C55D89913.6	PF03219				GO:GO:0016021,GO:GO:0006810,GO:GO:0005524,GO:GO:0005471	AT5G05310.2	TLC ATP/ADP transporter	LOC_Os11g02159.1	expressed protein	1,50	-1.0	1	21	10	30	UCUAAUAUUAUGGGACGGAGG	CCUCCGUCCCAUAAUAUAAGA	Cleavage
tae-miR1130b-3p	Traes_4AL_C55D89913.7	PF03219				GO:GO:0016021,GO:GO:0006810,GO:GO:0005524,GO:GO:0005471	AT5G05310.2	TLC ATP/ADP transporter	LOC_Os11g02159.2	expressed protein	0,00	-1.0	1	21	10	30	UCUUAUAUUAUGGGACGGAGG	CCUCCGUCCCAUAAUAUAAGA	Cleavage
tae-miR1122c-3p	Traes_4AL_C55D89913.7	PF03219				GO:GO:0016021,GO:GO:0006810,GO:GO:0005524,GO:GO:0005471	AT5G05310.2	TLC ATP/ADP transporter	LOC_Os11g02159.2	expressed protein	1,50	-1.0	1	21	10	30	UCUAAUAUUAUGGGACGGAGG	CCUCCGUCCCAUAAUAUAAGA	Cleavage
tae-miR1130b-3p	Traes_4AL_C55D89913.8					GO:GO:0016021,GO:GO:0006810,GO:GO:0005524,GO:GO:0005471	AT5G05310.2	TLC ATP/ADP transporter	LOC_Os11g02159.2	expressed protein	0,00	-1.0	1	21	10	30	UCUUAUAUUAUGGGACGGAGG	CCUCCGUCCCAUAAUAUAAGA	Cleavage
tae-miR1122c-3p	Traes_4AL_C55D89913.8					GO:GO:0016021,GO:GO:0006810,GO:GO:0005524,GO:GO:0005471	AT5G05310.2	TLC ATP/ADP transporter	LOC_Os11g02159.2	expressed protein	1,50	-1.0	1	21	10	30	UCUAAUAUUAUGGGACGGAGG	CCUCCGUCCCAUAAUAUAAGA	Cleavage
tae-miR5049-3p	Traes_4AL_C9E08C423.5										0,00	-1.0	1	21	69	89	AAUAUGGAUCGGAGGGAGUAC	CUACUCCCUCCGAUCCAUAUU	Cleavage
tae-miR9782	Traes_4AL_D576D98B4.1	PF00657		3.1.1.3		GO:GO:0016788	AT5G03810.1	GDSL-like Lipase/Acylhydrolase family protein	LOC_Os03g47940.1	GDSL-like lipase/acylhydrolase, putative, expressed	1,00	-1.0	1	24	2	25	GUAUUAGGUUGGUCAAAUUGACGA	UCGUCAAUUUGACCAACCUAAUAU	Cleavage
tae-miR9782	Traes_4AL_D576D98B4.2			3.1.1.1			AT5G03810.1	GDSL-like Lipase/Acylhydrolase family protein	LOC_Os03g47940.1	GDSL-like lipase/acylhydrolase, putative, expressed	1,00	-1.0	1	24	2	25	GUAUUAGGUUGGUCAAAUUGACGA	UCGUCAAUUUGACCAACCUAAUAU	Cleavage
tae-miR9661-5p	Traes_4AL_D8CCE0D2E.1										2,00	-1.0	1	21	647	667	UGAAGUAGAGCAGGGACCUCA	GGAGGUCUCUGCUCUACUUCC	Cleavage
tae-miR9661-5p	Traes_4AL_D8CCE0D2E.2										2,00	-1.0	1	21	644	664	UGAAGUAGAGCAGGGACCUCA	GGAGGUCUCUGCUCUACUUCC	Cleavage
tae-miR9661-5p	Traes_4AL_D8CCE0D2E.3										2,00	-1.0	1	21	620	640	UGAAGUAGAGCAGGGACCUCA	GGAGGUCUCUGCUCUACUUCC	Cleavage
tae-miR9661-5p	Traes_4AL_D8CCE0D2E.4										2,00	-1.0	1	21	587	607	UGAAGUAGAGCAGGGACCUCA	GGAGGUCUCUGCUCUACUUCC	Cleavage
tae-miR5384-3p	Traes_4AL_E60E5B7BA.1	PF02861	KOG1051		K03695	GO:GO:0019538	AT3G52490.1	Double Clp-N motif-containing P-loop nucleoside triphosphate hydrolases superfamily protein	LOC_Os11g05820.1	transposon protein, putative, Mutator sub-class, expressed	2,50	-1.0	1	21	269	289	UGAGCGCGCCGCCGUCGAAUG	UGUUCCACGGCGGCGCGCACA	Cleavage
tae-miR5384-3p	Traes_4AL_E60E5B7BA.2		KOG1051				AT3G52490.1	Double Clp-N motif-containing P-loop nucleoside triphosphate hydrolases superfamily protein	LOC_Os11g05820.1	transposon protein, putative, Mutator sub-class, expressed	2,50	-1.0	1	21	140	160	UGAGCGCGCCGCCGUCGAAUG	UGUUCCACGGCGGCGCGCACA	Cleavage
tae-miR1130b-3p	Traes_4AS_0E2735576.2			3.4.21.108					LOC_Os05g05330.1	expressed protein	1,50	-1.0	1	21	977	997	UCUUAUAUUAUGGGACGGAGG	CCUCCGUCUCAUAAUAUAAGA	Cleavage
tae-miR1120c-5p	Traes_4AS_0E2735576.2			3.4.21.108					LOC_Os05g05330.1	expressed protein	0,00	-1.0	1	21	1020	1040	UAAUAUAAGAACGUUUUUGAC	GACAAAAACGUUCUUAUAUUA	Cleavage
tae-miR9674a-5p	Traes_4AS_AB9088E9B.2	PF11955					AT5G21970.1	Ubiquitin carboxyl-terminal hydrolase family protein	LOC_Os11g34160.1	ubiquitin carboxyl-terminal hydrolase, family 1, putative, expressed	2,50	-1.0	1	21	1383	1403	GCAUCAUCCAUCCUACCAUUC	UGAUGGUCAGAUGGAUGAUGC	Cleavage
tae-miR9674a-5p	Traes_4AS_AB9088E9B.3	PF11955					AT5G21970.1	Ubiquitin carboxyl-terminal hydrolase family protein	LOC_Os11g34160.1	ubiquitin carboxyl-terminal hydrolase, family 1, putative, expressed	2,50	-1.0	1	21	1383	1403	GCAUCAUCCAUCCUACCAUUC	UGAUGGUCAGAUGGAUGAUGC	Cleavage
tae-miR5384-3p	Traes_4AS_C7CBD4A47.1	PF03703					AT1G14345.1	NAD(P)-linked oxidoreductase superfamily protein	LOC_Os03g07440.1	putativ eribonuclease P, putative, expressed	2,50	-1.0	1	21	194	214	UGAGCGCGCCGCCGUCGAAUG	UCUUCGACGGCGGCGCGCACU	Cleavage
tae-miR1135	Traes_4AS_D533B306B.3	PF01485	KOG1815	6.3.2.19		GO:GO:0016567,GO:GO:0004842	AT2G16090.1	RING/U-box superfamily protein	LOC_Os03g13100.1	ariadne-1, putative, expressed	2,00	-1.0	1	24	1698	1721	CUGCGACAAGUAAUUCCGAACGGA	CGAACCAGGAAUUACUUGUCGCAG	Cleavage
tae-miR1131	Traes_4BL_178D6FFF1.1	PF04749					AT5G05350.1	PLAC8 family protein	LOC_Os03g18790.1	SAG20, putative, expressed	1,50	-1.0	1	22	167	188	UAGUACCGGUUCGUGGCUAACC	GGUUAGCCACGAACCGAUACUA	Cleavage
tae-miR1131	Traes_4BL_178D6FFF1.2	PF04749					AT5G05350.1	PLAC8 family protein	LOC_Os03g18790.1	SAG20, putative, expressed	1,50	-1.0	1	22	167	188	UAGUACCGGUUCGUGGCUAACC	GGUUAGCCACGAACCGAUACUA	Cleavage
tae-miR9662a-3p	Traes_4BL_29D8171E0.1								LOC_Os06g12100.1	mTERF family protein, expressed	2,00	-1.0	1	21	132	152	UUGAACAUCCCAGAGCCACCG	UCGUGGCGCUGGGAUGUUCAG	Cleavage
tae-miR9662b-3p	Traes_4BL_29D8171E0.1								LOC_Os06g12100.1	mTERF family protein, expressed	2,50	-1.0	1	21	131	151	UGAACAUCCCAGAGCCACCGG	CUCGUGGCGCUGGGAUGUUCA	Cleavage
tae-miR1122b-3p	Traes_4BL_2D34597CF.2	PF04068,PF04034					AT5G10070.2	RNase L inhibitor protein-related	LOC_Os03g09920.1	RNase L inhibitor protein-related, putative, expressed	2,50	-1.0	1	21	454	474	AGACUUAUAUGUAGGAACGGA	UCUGUUCCUAAAUAUAAGUCU	Translation
tae-miR1122b-3p	Traes_4BL_2D34597CF.3	PF04068,PF04034					AT5G10070.2	RNase L inhibitor protein-related	LOC_Os03g09920.1	RNase L inhibitor protein-related, putative, expressed	2,50	-1.0	1	21	454	474	AGACUUAUAUGUAGGAACGGA	UCUGUUCCUAAAUAUAAGUCU	Translation
tae-miR1128	Traes_4BL_37FDD905B.1	PF10250					AT1G04910.1	O-fucosyltransferase family protein	LOC_Os11g29120.2	growth regulator related protein, putative, expressed	2,50	-1.0	1	21	125	145	UACUACUCCCUCCGUCCGAAA	UUUGGGACGGAGGGAGUAGGA	Cleavage
tae-miR5384-3p	Traes_4BL_3B7A98FD6.1	PF03703					AT1G14345.1	NAD(P)-linked oxidoreductase superfamily protein	LOC_Os03g07440.1	putativ eribonuclease P, putative, expressed	2,50	-1.0	1	21	191	211	UGAGCGCGCCGCCGUCGAAUG	UCUUCGACGGCGGCGCGCACU	Cleavage
tae-miR9666a-3p	Traes_4BL_44010E331.1										3,00	-1.0	1	22	176	197	CGGUAGGGCUGUAUGAUGGCGA	UCGGCAUGAUACAGCCCUACCU	Cleavage
tae-miR1122b-3p	Traes_4BL_62355968B.1	PF00013				GO:GO:0003723	AT5G15270.2	RNA-binding KH domain-containing protein	LOC_Os03g25970.1	KH domain-containing protein, putative, expressed	2,50	-1.0	1	21	706	726	AGACUUAUAUGUAGGAACGGA	UCUGUUCCUAAAUAUAAGUCU	Translation
tae-miR1122a	Traes_4BL_62355968B.1	PF00013				GO:GO:0003723	AT5G15270.2	RNA-binding KH domain-containing protein	LOC_Os03g25970.1	KH domain-containing protein, putative, expressed	2,50	-1.0	1	20	747	766	UAGAUACAUCCGUAUCUAGA	ACUACAUACGGAUGUAUAUA	Cleavage
tae-miR1137a	Traes_4BL_D08D5FFB5.2	PF08282	KOG3189	5.4.2.8		GO:GO:0009298,GO:GO:0005737,GO:GO:0004615	AT2G45790.1	phosphomannomutase	LOC_Os04g58580.1	phosphomannomutase, putative, expressed	2,00	-1.0	1	20	722	741	UAGUACAAAGUUGAGUCAUC	AGUGUCUCAACUUUGUACUA	Cleavage
tae-miR1137a	Traes_4BL_F3D5D7034.2						AT3G16990.1	Haem oxygenase-like, multi-helical	LOC_Os03g19390.1	TENA/THI-4 family protein, putative, expressed	1,00	-1.0	1	19	1	19	UAGUACAAAGUUGAGUCAU	AUGACCCAACUUUGUACUA	Cleavage
tae-miR1137b-5p	Traes_4BL_F3D5D7034.2						AT3G16990.1	Haem oxygenase-like, multi-helical	LOC_Os03g19390.1	TENA/THI-4 family protein, putative, expressed	1,50	-1.0	1	21	37	57	UCCGUUCCAGAAUAGAUGACC	GGUCAUCUAUUUUGGAACGGA	Translation
tae-miR9662a-3p	Traes_4BS_257C5B099.1	PF02536				GO:GO:0006355,GO:GO:0005739,GO:GO:0003690	AT5G07900.1	Mitochondrial transcription termination factor family protein	LOC_Os06g12100.1	mTERF family protein, expressed	2,00	-1.0	1	21	66	86	UUGAACAUCCCAGAGCCACCG	CCGUGGUUCUGGGAUGUUCAG	Cleavage
tae-miR9662b-3p	Traes_4BS_257C5B099.1	PF02536				GO:GO:0006355,GO:GO:0005739,GO:GO:0003690	AT5G07900.1	Mitochondrial transcription termination factor family protein	LOC_Os06g12100.1	mTERF family protein, expressed	2,00	-1.0	1	21	65	85	UGAACAUCCCAGAGCCACCGG	CCCGUGGUUCUGGGAUGUUCA	Cleavage
tae-miR156	Traes_4BS_375EF1D15.1								LOC_Os01g69830.1	OsSPL2 - SBP-box gene family member, expressed	2,50	-1.0	1	21	235	255	UGACAGAAGAGAGUGAGCACA	UGUGCUCUCUCUUUUCUGUCA	Cleavage
tae-miR1118	Traes_4BS_4BC02509F.1						AT3G43790.3	zinc induced facilitator-like 2	LOC_Os12g03830.1	major facilitator superfamily antiporter, putative, expressed	2,50	-1.0	1	23	525	547	CACUACAUUAUGGAAUGGAGGGA	UCCCUCCGUUCCAUAAUGUAAUG	Cleavage
tae-miR9661-5p	Traes_4BS_5EC01BC81.1										2,00	-1.0	1	21	653	673	UGAAGUAGAGCAGGGACCUCA	GGAGGUCUCUGCUCUACUUCC	Cleavage
tae-miR9661-5p	Traes_4BS_5EC01BC81.2										2,00	-1.0	1	21	653	673	UGAAGUAGAGCAGGGACCUCA	GGAGGUCUCUGCUCUACUUCC	Cleavage
tae-miR9661-5p	Traes_4BS_5EC01BC81.3										2,00	-1.0	1	21	653	673	UGAAGUAGAGCAGGGACCUCA	GGAGGUCUCUGCUCUACUUCC	Cleavage
tae-miR1122b-3p	Traes_4BS_6252B0BA0.1	PF05562					AT1G29395.1	COLD REGULATED 314 INNER MEMBRANE 1	LOC_Os05g49170.1	cold acclimation protein WCOR413, putative, expressed	1,50	-1.0	1	21	46	66	AGACUUAUAUGUAGGAACGGA	UCCGUUCCUAAAUAUAAGUCU	Translation
tae-miR7757-5p	Traes_4BS_B45729F4D.4	PF00931				GO:GO:0043531	AT3G07040.1	NB-ARC domain-containing disease resistance protein	LOC_Os11g11810.1	NBS-LRR disease resistance protein, putative, expressed	2,50	-1.0	1	22	653	674	AUAAAACCUUCAGCUAUCCAUC	AAUGGAUAUCUGAAGGUUUCAU	Cleavage
tae-miR5384-3p	Traes_4BS_F1D7488B8.1	PF02861	KOG1051		K03695	GO:GO:0019538	AT3G52490.1	Double Clp-N motif-containing P-loop nucleoside triphosphate hydrolases superfamily protein	LOC_Os11g05820.1	transposon protein, putative, Mutator sub-class, expressed	2,50	-1.0	1	21	269	289	UGAGCGCGCCGCCGUCGAAUG	UGUUCCACGGCGGCGCGCACA	Cleavage
tae-miR1122a	Traes_4BS_F395A220A.2	PF01595	KOG2118				AT2G14520.1	CBS domain-containing protein with a domain of unknown function (DUF21)	LOC_Os03g47120.1	CBS domain containing protein, expressed	0,00	-1.0	1	20	1336	1355	UAGAUACAUCCGUAUCUAGA	UCUAGAUACGGAUGUAUCUA	Cleavage
tae-miR7757-5p	Traes_4DL_113F6DD2B.1	PF00931				GO:GO:0043531	AT1G50180.1	NB-ARC domain-containing disease resistance protein	LOC_Os12g13550.1	NBS-LRR disease resistance protein, putative, expressed	2,50	-1.0	1	22	215	236	AUAAAACCUUCAGCUAUCCAUC	AAUGGAUGGCUGAAGGCUUUAU	Cleavage
tae-miR7757-5p	Traes_4DL_113F6DD2B.2	PF00931				GO:GO:0043531	AT1G50180.1	NB-ARC domain-containing disease resistance protein	LOC_Os10g22484.2	RGH2B, putative, expressed	2,50	-1.0	1	22	215	236	AUAAAACCUUCAGCUAUCCAUC	AAUGGAUGGCUGAAGGCUUUAU	Cleavage
tae-miR5085	Traes_4DL_341830EFF.1										2,50	-1.0	1	21	583	603	AAGGACAUUUUUUGUGGCCUG	GAGGACACAAAAAAUGUCGUU	Cleavage
tae-miR1133	Traes_4DL_529524815.5						AT1G67250.1	Proteasome maturation factor UMP1	LOC_Os02g55640.1	proteasome maturation factor UMP1 family protein, expressed	2,00	-1.0	1	22	103	127	CAUAUACUCCCUCCG-UCCGA--AA	UUAGUCGGAUCGGAGGGAGUAUAUG	Cleavage
tae-miR1130b-3p	Traes_4DL_84745919A.2			2.7.7.19			AT3G51620.2	PAP/OAS1 substrate-binding domain superfamily	LOC_Os03g21850.1	nucleotidyltransferase, putative, expressed	2,50	-1.0	1	21	2964	2984	UCUUAUAUUAUGGGACGGAGG	CCUCUGUCCCAUAAUAUAGGA	Cleavage
tae-miR1130a	Traes_4DL_84745919A.2			2.7.7.19			AT3G51620.2	PAP/OAS1 substrate-binding domain superfamily	LOC_Os03g21850.1	nucleotidyltransferase, putative, expressed	1,50	-1.0	1	23	3016	3038	CCUCCGUCUCGUAAUGUAAGACG	UGUCUUACAUUAUGAGACGGAGG	Translation
tae-miR1128	Traes_4DL_93D4DED90.3	PF00009		3.6.5.3	K03231		AT5G60390.3	GTP binding Elongation factor Tu family protein	LOC_Os03g08010.1	elongation factor Tu, putative, expressed	3,00	-1.0	1	21	565	586	UACUACUCCCUCCG-UCCGAAA	UUUUGGAACGGAGGGAGUAGUA	Cleavage
tae-miR1137a	Traes_4DL_93D4DED90.3	PF00009		3.6.5.3	K03231		AT5G60390.3	GTP binding Elongation factor Tu family protein	LOC_Os03g08010.1	elongation factor Tu, putative, expressed	2,50	-1.0	1	20	508	527	UAGUACAAAGUUGAGUCAUC	GAUGACCCAACUUUGUAUUA	Cleavage
tae-miR1137b-5p	Traes_4DL_93D4DED90.3	PF00009		3.6.5.3	K03231		AT5G60390.3	GTP binding Elongation factor Tu family protein	LOC_Os03g08010.1	elongation factor Tu, putative, expressed	1,50	-1.0	1	21	556	576	UCCGUUCCAGAAUAGAUGACC	GGUCAUCUAUUUUGGAACGGA	Translation
tae-miR1137b-5p	Traes_4DS_28E6C4F21.2										1,50	-1.0	1	21	61	81	UCCGUUCCAGAAUAGAUGACC	AGUCAUCUAUUUUGGAACGGA	Translation
tae-miR1137b-5p	Traes_4DS_28E6C4F21.3										1,50	-1.0	1	21	61	81	UCCGUUCCAGAAUAGAUGACC	AGUCAUCUAUUUUGGAACGGA	Translation
tae-miR1137b-5p	Traes_4DS_28E6C4F21.4										1,50	-1.0	1	21	61	81	UCCGUUCCAGAAUAGAUGACC	AGUCAUCUAUUUUGGAACGGA	Translation
tae-miR7757-5p	Traes_4DS_5B29EE3DD.5	PF00931				GO:GO:0043531	AT3G50950.1	HOPZ-ACTIVATED RESISTANCE 1	LOC_Os11g11810.1	NBS-LRR disease resistance protein, putative, expressed	2,50	-1.0	1	22	1379	1400	AUAAAACCUUCAGCUAUCCAUC	AAUGGAUAUCUGAAGGUUUCAU	Cleavage
tae-miR7757-5p	Traes_4DS_5B29EE3DD.6	PF00931				GO:GO:0043531	AT3G50950.1	HOPZ-ACTIVATED RESISTANCE 1	LOC_Os11g11810.1	NBS-LRR disease resistance protein, putative, expressed	2,50	-1.0	1	22	1379	1400	AUAAAACCUUCAGCUAUCCAUC	AAUGGAUAUCUGAAGGUUUCAU	Cleavage
tae-miR7757-5p	Traes_4DS_5B29EE3DD.7	PF00931				GO:GO:0043531	AT1G58390.1	Disease resistance protein (CC-NBS-LRR class) family	LOC_Os11g11810.1	NBS-LRR disease resistance protein, putative, expressed	2,50	-1.0	1	22	824	845	AUAAAACCUUCAGCUAUCCAUC	AAUGGAUAUCUGAAGGUUUCAU	Cleavage
tae-miR1130b-3p	Traes_4DS_9179FF158.4	PF06964		3.2.1.55		GO:GO:0046556,GO:GO:0046373	AT3G10740.1	alpha-L-arabinofuranosidase 1	LOC_Os12g03470.1	alpha-N-arabinofuranosidase A, putative, expressed	1,50	-1.0	1	21	41	61	UCUUAUAUUAUGGGACGGAGG	CCUCCGUCCCAUAAUGUAAGA	Cleavage
tae-miR9778	Traes_4DS_B08D83A2F.1	PF00931				GO:GO:0043531	AT3G46530.1	NB-ARC domain-containing disease resistance protein	LOC_Os11g39550.1	Leucine Rich Repeat family protein, expressed	2,50	-1.0	1	21	126	146	UGCAUCAUCUCGAACUCGUCG	UGGCGAGUUCCAGAUGAUGCA	Translation
tae-miR164	Traes_4DS_C08BF9DB2.1			2.4.1.14			AT1G04920.1	sucrose phosphate synthase 3F	LOC_Os11g12810.1	sucrose-phosphate synthase, putative, expressed	2,50	-1.0	1	21	387	407	UGGAGAAGCAGGGCACGUGCA	UUCACGUGCCCUGCUUUUCCU	Cleavage
tae-miR1128	Traes_4DS_C22A101FE.2						AT1G07910.1	RNAligase	LOC_Os07g09400.1	RNA ligase isoform 1, putative, expressed	2,50	-1.0	1	21	670	690	UACUACUCCCUCCGUCCGAAA	UUUGGGACGAAGGGAGUAGUA	Cleavage
tae-miR1128	Traes_4DS_C22A101FE.9						AT1G07910.1	RNAligase	LOC_Os07g09400.1	RNA ligase isoform 1, putative, expressed	2,50	-1.0	1	21	358	378	UACUACUCCCUCCGUCCGAAA	UUUGGGACGAAGGGAGUAGUA	Cleavage
tae-miR1122a	Traes_4DS_E8239194E.2	PF01394					AT3G08530.1	Clathrin, heavy chain	LOC_Os12g01390.1	clathrin heavy chain, putative, expressed	2,50	-1.0	1	20	692	711	UAGAUACAUCCGUAUCUAGA	UCUAAAUACGGAUGUAUCAA	Cleavage
tae-miR5384-3p	Traes_4DS_ECFDD82BA.1	PF02861	KOG1051		K03695	GO:GO:0019538	AT3G52490.1	Double Clp-N motif-containing P-loop nucleoside triphosphate hydrolases superfamily protein	LOC_Os11g05820.1	transposon protein, putative, Mutator sub-class, expressed	2,50	-1.0	1	21	269	289	UGAGCGCGCCGCCGUCGAAUG	UGUUCCACGGCGGCGCGCACA	Cleavage
tae-miR1137b-5p	Traes_4DS_EFE0451D7.3	PF00270		3.6.4.12		GO:GO:0005524,GO:GO:0003676	AT1G31360.1	RECQ helicase L2	LOC_Os11g48090.5	helicase conserved C-terminal domain containing protein, expressed	1,50	-1.0	1	21	950	970	UCCGUUCCAGAAUAGAUGACC	AGUCAUCUAUUUUGGAACGGA	Translation
tae-miR1128	Traes_5AL_3AB81DF9B.1	PF00056,PF02866	KOG1496	1.1.1.82		GO:GO:0055114,GO:GO:0016491,GO:GO:0016616,GO:GO:0016615,GO:GO:0006108	AT5G58330.1	lactate/malate dehydrogenase family protein	LOC_Os08g44810.1	lactate/malate dehydrogenase, putative, expressed	3,00	-1.0	1	21	151	172	UACUACUCCCUCCG-UCCGAAA	UUUAGGAACGGAGGGAGUAGUA	Cleavage
tae-miR9652-5p	Traes_5AL_481F73E59.1	PF16487,PF16486,PF16488,PF08699,PF02170,PF02171	KOG1041			GO:GO:0005515,GO:GO:0003676	AT5G43810.1	Stabilizer of iron transporter SufD / Polynucleotidyl transferase	LOC_Os03g58600.1	PAZ domain containing protein, putative, expressed	2,50	-1.0	1	22	883	904	CCUGUUUGUCAUUAAGUUUCUU	AAGAAACUGAAUGACAAGCAGG	Cleavage
tae-miR9652-5p	Traes_5AL_481F73E59.2	PF00240,PF16487,PF16486,PF16488,PF08699,PF02170,PF02171	KOG1041			GO:GO:0005515,GO:GO:0003676	AT5G43810.1	Stabilizer of iron transporter SufD / Polynucleotidyl transferase	LOC_Os03g58600.1	PAZ domain containing protein, putative, expressed	2,50	-1.0	1	22	883	904	CCUGUUUGUCAUUAAGUUUCUU	AAGAAACUGAAUGACAAGCAGG	Cleavage
tae-miR9652-5p	Traes_5AL_481F73E59.3	PF00240,PF16487,PF16486,PF16488,PF08699,PF02170,PF02171	KOG1041			GO:GO:0005515,GO:GO:0003676	AT5G43810.2	Stabilizer of iron transporter SufD / Polynucleotidyl transferase	LOC_Os03g58600.1	PAZ domain containing protein, putative, expressed	2,50	-1.0	1	22	883	904	CCUGUUUGUCAUUAAGUUUCUU	AAGAAACUGAAUGACAAGCAGG	Cleavage
tae-miR9652-5p	Traes_5AL_481F73E59.4	PF00240,PF16486,PF16488,PF08699,PF02170,PF02171	KOG1041			GO:GO:0005515,GO:GO:0003676	AT1G48410.1	Stabilizer of iron transporter SufD / Polynucleotidyl transferase	LOC_Os03g58600.1	PAZ domain containing protein, putative, expressed	2,50	-1.0	1	22	883	904	CCUGUUUGUCAUUAAGUUUCUU	AAGAAACUGAAUGACAAGCAGG	Cleavage
tae-miR9652-5p	Traes_5AL_481F73E59.5	PF00240,PF16488,PF08699,PF02170,PF02171				GO:GO:0005515,GO:GO:0003676	AT1G48410.1	Stabilizer of iron transporter SufD / Polynucleotidyl transferase	LOC_Os03g58600.1	PAZ domain containing protein, putative, expressed	2,50	-1.0	1	22	583	604	CCUGUUUGUCAUUAAGUUUCUU	AAGAAACUGAAUGACAAGCAGG	Cleavage
tae-miR9652-5p	Traes_5AL_481F73E59.6	PF02171,PF16486,PF16488,PF08699,PF02170				GO:GO:0003676,GO:GO:0005515	AT1G48410.1	Stabilizer of iron transporter SufD / Polynucleotidyl transferase	LOC_Os03g58600.1	PAZ domain containing protein, putative, expressed	2,50	-1.0	1	22	883	904	CCUGUUUGUCAUUAAGUUUCUU	AAGAAACUGAAUGACAAGCAGG	Cleavage
tae-miR5062-5p	Traes_5AL_500FF0B72.1	PF02171				GO:GO:0003676	AT2G27880.1	Argonaute family protein	LOC_Os03g58600.1	PAZ domain containing protein, putative, expressed	2,50	-1.0	1	23	43	65	UGAACCUUAGGGAACAGCCGCAU	ACCAGGCUAUUCCCUGAGGUUCA	Cleavage
tae-miR5062-5p	Traes_5AL_60D577E34.1	PF02171				GO:GO:0003676	AT2G27880.1	Argonaute family protein	LOC_Os03g58600.1	PAZ domain containing protein, putative, expressed	1,50	-1.0	1	23	43	65	UGAACCUUAGGGAACAGCCGCAU	ACCAGGCUGUUCCCUGAGGUUCA	Cleavage
tae-miR1127b-3p	Traes_5AL_7256A9F19.2	PF00400	KOG0279			GO:GO:0005515	AT3G18860.2	transducin family protein / WD-40 repeat family protein	LOC_Os07g03160.1	WD domain, G-beta repeat domain containing protein, expressed	1,50	-1.0	1	21	991	1011	ACAAGUAUUUCUGGACGGAGG	CCUCCGUCCGGAAAUACUUGU	Cleavage
tae-miR1122b-3p	Traes_5AL_7FF780810.1						AT5G41940.1	Ypt/Rab-GAP domain of gyp1p superfamily protein	LOC_Os09g35960.1	TBC domain containing protein, expressed	1,50	-1.0	1	21	22	42	AGACUUAUAUGUAGGAACGGA	UCCGUUCCUAAAUAUAAGUCU	Translation
tae-miR5086	Traes_5AL_8B87707C4.1										3,00	-1.0	1	21	350	370	ACAUUGGUGGAAGGCGUGGUA	AGUCAUGCCUUCCACCAAUGA	Cleavage
tae-miR5086	Traes_5AL_8B87707C4.2										3,00	-1.0	1	21	350	370	ACAUUGGUGGAAGGCGUGGUA	AGUCAUGCCUUCCACCAAUGA	Cleavage
tae-miR5086	Traes_5AL_8B87707C4.3										3,00	-1.0	1	21	326	346	ACAUUGGUGGAAGGCGUGGUA	AGUCAUGCCUUCCACCAAUGA	Cleavage
tae-miR7757-5p	Traes_5AL_B4E8A3115.1	PF00931				GO:GO:0043531	AT3G46710.1	NB-ARC domain-containing disease resistance protein	LOC_Os05g40150.1	RGH2B, putative, expressed	1,50	-1.0	1	22	368	389	AUAAAACCUUCAGCUAUCCAUC	AAUGGAUAGCUGAAGGUCUUAU	Cleavage
tae-miR7757-5p	Traes_5AL_B4E8A3115.2	PF03106,PF12796,PF00931	KOG4658			GO:GO:0043565,GO:GO:0006355,GO:GO:0003700,GO:GO:0043531	AT2G31820.1	Ankyrin repeat family protein	LOC_Os11g45750.2	WRKY125, expressed	1,50	-1.0	1	22	2324	2345	AUAAAACCUUCAGCUAUCCAUC	AAUGGAUAGCUGAAGGUCUUAU	Cleavage
tae-miR7757-5p	Traes_5AL_B4E8A3115.3	PF03106,PF12796,PF00931	KOG4658			GO:GO:0043565,GO:GO:0006355,GO:GO:0003700,GO:GO:0043531	AT2G31820.1	Ankyrin repeat family protein	LOC_Os11g45750.2	WRKY125, expressed	1,50	-1.0	1	22	2324	2345	AUAAAACCUUCAGCUAUCCAUC	AAUGGAUAGCUGAAGGUCUUAU	Cleavage
tae-miR7757-5p	Traes_5AL_B4E8A3115.4	PF03106,PF12796,PF00931	KOG4658			GO:GO:0043565,GO:GO:0006355,GO:GO:0003700,GO:GO:0043531	AT2G31820.1	Ankyrin repeat family protein	LOC_Os11g45750.2	WRKY125, expressed	1,50	-1.0	1	22	2324	2345	AUAAAACCUUCAGCUAUCCAUC	AAUGGAUAGCUGAAGGUCUUAU	Cleavage
tae-miR7757-5p	Traes_5AL_B4E8A3115.5	PF03106,PF12796,PF00931	KOG4658			GO:GO:0043565,GO:GO:0006355,GO:GO:0003700,GO:GO:0043531	AT2G31820.1	Ankyrin repeat family protein	LOC_Os11g45750.1	WRKY125, expressed	1,50	-1.0	1	22	2324	2345	AUAAAACCUUCAGCUAUCCAUC	AAUGGAUAGCUGAAGGUCUUAU	Cleavage
tae-miR7757-5p	Traes_5AL_B4E8A3115.6	PF00931,PF03106				GO:GO:0043531,GO:GO:0043565,GO:GO:0006355,GO:GO:0003700	AT3G46710.1	NB-ARC domain-containing disease resistance protein	LOC_Os05g40150.1	RGH2B, putative, expressed	1,50	-1.0	1	22	368	389	AUAAAACCUUCAGCUAUCCAUC	AAUGGAUAGCUGAAGGUCUUAU	Cleavage
tae-miR9652-5p	Traes_5AL_C9236F111.1	PF16487,PF16488,PF08699,PF02170				GO:GO:0005515	AT1G48410.2	Stabilizer of iron transporter SufD / Polynucleotidyl transferase	LOC_Os03g58600.1	PAZ domain containing protein, putative, expressed	2,50	-1.0	1	22	778	799	CCUGUUUGUCAUUAAGUUUCUU	AAGAAACUGAAUGACAAGCAGG	Cleavage
tae-miR5384-3p	Traes_5AS_A928BA201.1	PF04535					AT5G44550.1	Uncharacterised protein family (UPF0497)	LOC_Os12g41690.1	membrane associated DUF588 domain containing protein, putative, expressed	2,50	-1.0	1	21	506	526	UGAGCGCGCCGCCGUCGAAUG	CAUUCGUCGGCGUCGCGCUCA	Cleavage
tae-miR7757-5p	Traes_5AS_BDFB08E23.1	PF00931	KOG4658			GO:GO:0043531	AT3G07040.1	NB-ARC domain-containing disease resistance protein	LOC_Os12g37740.1	resistance protein LR10, putative, expressed	2,50	-1.0	1	22	1349	1370	AUAAAACCUUCAGCUAUCCAUC	GAUGGAUUGCUGAAGGUUUCAU	Cleavage
tae-miR7757-5p	Traes_5AS_BDFB08E23.2	PF00931	KOG4658			GO:GO:0043531	AT3G07040.1	NB-ARC domain-containing disease resistance protein	LOC_Os12g37740.1	resistance protein LR10, putative, expressed	2,50	-1.0	1	22	1349	1370	AUAAAACCUUCAGCUAUCCAUC	GAUGGAUUGCUGAAGGUUUCAU	Cleavage
tae-miR7757-5p	Traes_5AS_BDFB08E23.3	PF00931				GO:GO:0043531	AT3G07040.1	NB-ARC domain-containing disease resistance protein	LOC_Os12g37740.1	resistance protein LR10, putative, expressed	2,50	-1.0	1	22	524	545	AUAAAACCUUCAGCUAUCCAUC	GAUGGAUUGCUGAAGGUUUCAU	Cleavage
tae-miR7757-5p	Traes_5AS_BDFB08E23.4	PF00931				GO:GO:0043531	AT3G46530.1	NB-ARC domain-containing disease resistance protein	LOC_Os12g37740.1	resistance protein LR10, putative, expressed	2,50	-1.0	1	22	1349	1370	AUAAAACCUUCAGCUAUCCAUC	GAUGGAUUGCUGAAGGUUUCAU	Cleavage
tae-miR7757-5p	Traes_5AS_BDFB08E23.5	PF00931				GO:GO:0043531	AT3G46530.1	NB-ARC domain-containing disease resistance protein	LOC_Os12g37740.1	resistance protein LR10, putative, expressed	2,50	-1.0	1	22	1349	1370	AUAAAACCUUCAGCUAUCCAUC	GAUGGAUUGCUGAAGGUUUCAU	Cleavage
tae-miR1128	Traes_5AS_E1F692526.2										3,00	-1.0	1	21	111	132	UACUACUCCCUCCGUCCGA-AA	UUAUGGGACGGAGGGAGUAGUA	Cleavage
tae-miR1130b-3p	Traes_5AS_E1F692526.2										3,00	-1.0	1	21	40	61	UCUU-AUAUUAUGGGACGGAGG	CCUCCGUCCCAUAAUAUUAAGA	Cleavage
tae-miR1122c-3p	Traes_5AS_E1F692526.2										3,00	-1.0	1	21	40	61	UC-UAAUAUUAUGGGACGGAGG	CCUCCGUCCCAUAAUAUUAAGA	Cleavage
tae-miR164	Traes_5AS_E5FD82E36.1	PF02365				GO:GO:0006355,GO:GO:0003677	AT1G56010.2	NAC domain containing protein 1	LOC_Os12g41680.1	No apical meristem protein, putative, expressed	1,00	-1.0	1	21	653	673	UGGAGAAGCAGGGCACGUGCA	AGCAAGUGCCCUGCUUCUCCA	Cleavage
tae-miR164	Traes_5AS_E5FD82E36.2	PF02365				GO:GO:0006355,GO:GO:0003677	AT1G56010.2	NAC domain containing protein 1	LOC_Os12g41680.1	No apical meristem protein, putative, expressed	1,00	-1.0	1	21	656	676	UGGAGAAGCAGGGCACGUGCA	AGCAAGUGCCCUGCUUCUCCA	Cleavage
tae-miR1118	Traes_5AS_EC41EF7CA.4	PF00013	KOG2190			GO:GO:0003723	AT3G04610.1	RNA-binding KH domain-containing protein	LOC_Os12g40560.1	KH domain containing protein, putative, expressed	1,50	-1.0	1	23	1333	1355	CACUACAUUAUGGAAUGGAGGGA	UCCCUCCAUUCCACAAUGUAGUG	Translation
tae-miR1125	Traes_5AS_EC41EF7CA.4	PF00013	KOG2190			GO:GO:0003723	AT3G04610.1	RNA-binding KH domain-containing protein	LOC_Os12g40560.1	KH domain containing protein, putative, expressed	1,00	-1.0	1	24	1450	1473	AACCAACGAGACCAACUGCGGCGG	CCGCCGCAGUCGGUCUCGUUGGUU	Cleavage
tae-miR408	Traes_5BL_0212ACBD9.1										2,50	-1.0	1	21	2512	2532	CUGCACUGCCUCUUCCCUGGC	GCCAAGGAAGAGGCUGUGCAG	Cleavage
tae-miR1137a	Traes_5BL_0EC4788EF.2	PF08244,PF00251	KOG0228	3.2.1.26			AT1G55120.1	beta-fructofuranosidase 5	LOC_Os01g73580.1	glycosyl hydrolases, putative, expressed	1,00	-1.0	1	20	1543	1562	UAGUACAAAGUUGAGUCAUC	GAUGACCCAACUUUGUACUA	Cleavage
tae-miR9652-5p	Traes_5BL_0F64FDA44.1	PF08699,PF02170				GO:GO:0005515	AT1G48410.1	Stabilizer of iron transporter SufD / Polynucleotidyl transferase	LOC_Os03g58600.1	PAZ domain containing protein, putative, expressed	2,50	-1.0	1	22	583	604	CCUGUUUGUCAUUAAGUUUCUU	AAGAAACUGAAUGACAAGCAGG	Cleavage
tae-miR1122a	Traes_5BL_1B9186ED8.1	PF07762							LOC_Os11g07360.1	expressed protein	2,50	-1.0	1	20	986	1005	UAGAUACAUCCGUAUCUAGA	UCGAGAUACAGAUGUAUCUA	Translation
tae-miR7757-5p	Traes_5BL_1E751EF1F.1	PF00931,PF03514	KOG4658			GO:GO:0043531	AT1G50600.1	scarecrow-like 5	LOC_Os10g22484.1	RGH2B, putative, expressed	2,00	-1.0	1	22	1799	1820	AUAAAACCUUCAGCUAUCCAUC	AAUGGGUUGCUGAAGGUUUUAU	Cleavage
tae-miR7757-5p	Traes_5BL_1E751EF1F.2	PF00931				GO:GO:0043531	AT3G46730.1	NB-ARC domain-containing disease resistance protein	LOC_Os10g22484.1	RGH2B, putative, expressed	2,00	-1.0	1	22	1799	1820	AUAAAACCUUCAGCUAUCCAUC	AAUGGGUUGCUGAAGGUUUUAU	Cleavage
tae-miR7757-5p	Traes_5BL_1E751EF1F.5	PF00931,PF03514				GO:GO:0043531	AT1G50600.1	scarecrow-like 5	LOC_Os10g22484.1	RGH2B, putative, expressed	2,00	-1.0	1	22	512	533	AUAAAACCUUCAGCUAUCCAUC	AAUGGGUUGCUGAAGGUUUUAU	Cleavage
tae-miR5049-3p	Traes_5BL_4BFE76791.5								LOC_Os08g04590.1	csAtPR5, putative, expressed	2,50	-1.0	1	21	416	436	AAUAUGGAUCGGAGGGAGUAC	AUGCUCCCUCUGAUCCAUAUU	Translation
tae-miR1127b-3p	Traes_5BL_5E94C243F.1						AT3G54630.1		LOC_Os08g36490.1	kinetochore protein, putative, expressed	2,50	-1.0	1	21	914	934	ACAAGUAUUUCUGGACGGAGG	CCUCUGUCCUGAAAUACUUGU	Cleavage
tae-miR1127b-3p	Traes_5BL_5E94C243F.2						AT3G54630.1		LOC_Os08g36490.1	kinetochore protein, putative, expressed	2,50	-1.0	1	21	898	918	ACAAGUAUUUCUGGACGGAGG	CCUCUGUCCUGAAAUACUUGU	Cleavage
tae-miR9674b-5p	Traes_5BL_6216A6108.1	PF13041					AT3G22470.1	Pentatricopeptide repeat (PPR) superfamily protein	LOC_Os10g35240.2	Rf1, mitochondrial precursor, putative, expressed	3,00	-1.0	1	21	411	431	AUAGCAUCAUCCAUCCUACCC	GGGCCGAAUGGAUGAUGCUAU	Cleavage
tae-miR9674b-5p	Traes_5BL_6216A6108.1	PF13041					AT3G22470.1	Pentatricopeptide repeat (PPR) superfamily protein	LOC_Os10g35240.2	Rf1, mitochondrial precursor, putative, expressed	3,00	-1.0	1	21	411	431	AUAGCAUCAUCCAUCCUACCC	GGGCCGAAUGGAUGAUGCUAU	Cleavage
tae-miR5384-3p	Traes_5BL_6240DE6BA.1	PF00067		1.14.13.89		GO:GO:0055114,GO:GO:0020037,GO:GO:0016705,GO:GO:0005506	AT5G67310.1	cytochrome P450, family 81, subfamily G, polypeptide 1	LOC_Os03g55240.1	cytochrome P450, putative, expressed	2,50	-1.0	1	21	338	358	UGAGCGCGCCGCCGUCGAAUG	CCUUCGACGGCGCCGCGCUCG	Cleavage
tae-miR5384-3p	Traes_5BL_6240DE6BA.2	PF00067		1.14.13.89		GO:GO:0055114,GO:GO:0020037,GO:GO:0016705,GO:GO:0005506	AT5G67310.1	cytochrome P450, family 81, subfamily G, polypeptide 1	LOC_Os03g55240.1	cytochrome P450, putative, expressed	2,50	-1.0	1	21	338	358	UGAGCGCGCCGCCGUCGAAUG	CCUUCGACGGCGCCGCGCUCG	Cleavage
tae-miR5384-3p	Traes_5BL_6240DE6BA.3	PF00067		1.14.13.89		GO:GO:0055114,GO:GO:0020037,GO:GO:0016705,GO:GO:0005506	AT5G67310.1	cytochrome P450, family 81, subfamily G, polypeptide 1	LOC_Os03g55240.1	cytochrome P450, putative, expressed	2,50	-1.0	1	21	338	358	UGAGCGCGCCGCCGUCGAAUG	CCUUCGACGGCGCCGCGCUCG	Cleavage
tae-miR5384-3p	Traes_5BL_6240DE6BA.4	PF00067		1.14.13.89		GO:GO:0055114,GO:GO:0020037,GO:GO:0016705,GO:GO:0005506	AT5G67310.1	cytochrome P450, family 81, subfamily G, polypeptide 1	LOC_Os03g55240.1	cytochrome P450, putative, expressed	2,50	-1.0	1	21	338	358	UGAGCGCGCCGCCGUCGAAUG	CCUUCGACGGCGCCGCGCUCG	Cleavage
tae-miR1122a	Traes_5BL_637A3C970.8	PF01501		2.4.1.43		GO:GO:0016757,GO:GO:0047262	AT5G47780.1	galacturonosyltransferase 4	LOC_Os09g30280.1	glycosyl transferase 8 domain containing protein, putative, expressed	1,50	-1.0	1	20	49	68	UAGAUACAUCCGUAUCUAGA	UCUAGAUACGAAUGUAUCUA	Translation
tae-miR1121	Traes_5BL_72EFF0AF0.1	PF07690				GO:GO:0055085,GO:GO:0016021	AT5G59250.1	Major facilitator superfamily protein	LOC_Os10g42830.1	transporter family protein, putative, expressed	0,00	-1.0	1	22	1237	1258	AGUAGUGAUCUAAACGCUCUUA	UAAGAGCGUUUAGAUCACUACU	Cleavage
tae-miR408	Traes_5BL_7486582EB.1										2,50	-1.0	1	21	8	28	CUGCACUGCCUCUUCCCUGGC	CGCAGGGAAGAGGCAGUGCCA	Cleavage
tae-miR1122a	Traes_5BL_7821274AB.1	PF07762							LOC_Os11g07360.1	expressed protein	2,50	-1.0	1	20	986	1005	UAGAUACAUCCGUAUCUAGA	UCGAGAUACAGAUGUAUCUA	Translation
tae-miR7757-5p	Traes_5BL_7F0FD1538.2	PF00847,PF00931	KOG4658			GO:GO:0006355,GO:GO:0003700,GO:GO:0043531	AT3G46710.1	NB-ARC domain-containing disease resistance protein	LOC_Os05g40150.1	RGH2B, putative, expressed	1,50	-1.0	1	22	2096	2117	AUAAAACCUUCAGCUAUCCAUC	AAUGGAUAGCUGAAAGUUUUAU	Cleavage
tae-miR7757-5p	Traes_5BL_7F0FD1538.3	PF00847,PF00931	KOG4658			GO:GO:0006355,GO:GO:0003700,GO:GO:0043531	AT3G46710.1	NB-ARC domain-containing disease resistance protein	LOC_Os05g40150.1	RGH2B, putative, expressed	1,50	-1.0	1	22	1379	1400	AUAAAACCUUCAGCUAUCCAUC	AAUGGAUAGCUGAAAGUUUUAU	Cleavage
tae-miR7757-5p	Traes_5BL_7F0FD1538.4	PF00931	KOG4658			GO:GO:0043531	AT3G46710.1	NB-ARC domain-containing disease resistance protein	LOC_Os05g40150.1	RGH2B, putative, expressed	1,50	-1.0	1	22	1379	1400	AUAAAACCUUCAGCUAUCCAUC	AAUGGAUAGCUGAAAGUUUUAU	Cleavage
tae-miR5086	Traes_5BL_83D9CB7C3.1										3,00	-1.0	1	21	497	517	ACAUUGGUGGAAGGCGUGGUA	AGUCAUGCCUUCCACCAAUGA	Cleavage
tae-miR5086	Traes_5BL_83D9CB7C3.2										3,00	-1.0	1	21	608	628	ACAUUGGUGGAAGGCGUGGUA	AGUCAUGCCUUCCACCAAUGA	Cleavage
tae-miR5086	Traes_5BL_83D9CB7C3.3										3,00	-1.0	1	21	473	493	ACAUUGGUGGAAGGCGUGGUA	AGUCAUGCCUUCCACCAAUGA	Cleavage
tae-miR5086	Traes_5BL_83D9CB7C3.4										3,00	-1.0	1	21	608	628	ACAUUGGUGGAAGGCGUGGUA	AGUCAUGCCUUCCACCAAUGA	Cleavage
tae-miR5086	Traes_5BL_83D9CB7C3.5										3,00	-1.0	1	21	497	517	ACAUUGGUGGAAGGCGUGGUA	AGUCAUGCCUUCCACCAAUGA	Cleavage
tae-miR156	Traes_5BL_96D5EA4EE.1	PF03110				GO:GO:0005634,GO:GO:0003677	AT2G42200.1	squamosa promoter binding protein-like 9	LOC_Os09g31438.1	OsSPL17 - SBP-box gene family member, expressed	1,00	-1.0	1	21	805	825	UGACAGAAGAGAGUGAGCACA	UGUGCUCUCUCUCUUCUGUCA	Cleavage
tae-miR156	Traes_5BL_96D5EA4EE.2	PF03110				GO:GO:0005634,GO:GO:0003677	AT2G42200.1	squamosa promoter binding protein-like 9	LOC_Os09g31438.1	OsSPL17 - SBP-box gene family member, expressed	1,00	-1.0	1	21	802	822	UGACAGAAGAGAGUGAGCACA	UGUGCUCUCUCUCUUCUGUCA	Cleavage
tae-miR156	Traes_5BL_96D5EA4EE.3								LOC_Os09g31438.1	OsSPL17 - SBP-box gene family member, expressed	1,00	-1.0	1	21	67	87	UGACAGAAGAGAGUGAGCACA	UGUGCUCUCUCUCUUCUGUCA	Cleavage
tae-miR156	Traes_5BL_96FDFC152.1	PF03110				GO:GO:0005634,GO:GO:0003677	AT1G76580.1	Squamosa promoter-binding protein-like (SBP domain) transcription factor family protein	LOC_Os09g32944.1	OsSPL18 - SBP-box gene family member, expressed	1,00	-1.0	1	21	508	528	UGACAGAAGAGAGUGAGCACA	UGUGCUCUCUCUCUUCUGUCA	Cleavage
tae-miR156	Traes_5BL_96FDFC152.2	PF03110				GO:GO:0005634,GO:GO:0003677	AT5G50570.1	Squamosa promoter-binding protein-like (SBP domain) transcription factor family protein	LOC_Os09g32944.1	OsSPL18 - SBP-box gene family member, expressed	1,00	-1.0	1	21	490	510	UGACAGAAGAGAGUGAGCACA	UGUGCUCUCUCUCUUCUGUCA	Cleavage
tae-miR5384-3p	Traes_5BL_AD734CCEF.1	PF12695	KOG1515	3.1.1.1			AT3G48690.1	alpha/beta-Hydrolases superfamily protein	LOC_Os09g28730.1	gibberellin receptor GID1L2, putative, expressed	2,50	-1.0	1	21	242	262	UGAGCGCGCCGCCGUCGAAUG	UCUUCCACGGCGGCGCGUUCA	Cleavage
tae-miR9664-3p	Traes_5BL_D27214492.1	PF00931				GO:GO:0043531	AT3G46530.1	NB-ARC domain-containing disease resistance protein	LOC_Os11g11550.1	NBS-LRR disease resistance protein, putative, expressed	2,50	-1.0	1	21	237	257	UUGCAGUCCUCGAUGUCGUAG	GCACGACAUCGAGGAUUGCAU	Cleavage
tae-miR9664-3p	Traes_5BL_D27214492.2	PF00931				GO:GO:0043531	AT3G46530.1	NB-ARC domain-containing disease resistance protein	LOC_Os11g11550.2	NBS-LRR disease resistance protein, putative, expressed	2,50	-1.0	1	21	237	257	UUGCAGUCCUCGAUGUCGUAG	GCACGACAUCGAGGAUUGCAU	Cleavage
tae-miR9664-3p	Traes_5BL_D27214492.3	PF00931				GO:GO:0043531	AT3G46530.1	NB-ARC domain-containing disease resistance protein	LOC_Os11g11550.2	NBS-LRR disease resistance protein, putative, expressed	2,50	-1.0	1	21	237	257	UUGCAGUCCUCGAUGUCGUAG	GCACGACAUCGAGGAUUGCAU	Cleavage
tae-miR9664-3p	Traes_5BL_D27214492.4	PF00931				GO:GO:0043531	AT3G46530.1	NB-ARC domain-containing disease resistance protein	LOC_Os11g11550.2	NBS-LRR disease resistance protein, putative, expressed	2,50	-1.0	1	21	237	257	UUGCAGUCCUCGAUGUCGUAG	GCACGACAUCGAGGAUUGCAU	Cleavage
tae-miR9664-3p	Traes_5BL_D27214492.5	PF00931				GO:GO:0043531	AT3G46530.1	NB-ARC domain-containing disease resistance protein	LOC_Os11g11550.1	NBS-LRR disease resistance protein, putative, expressed	2,50	-1.0	1	21	237	257	UUGCAGUCCUCGAUGUCGUAG	GCACGACAUCGAGGAUUGCAU	Cleavage
tae-miR7757-5p	Traes_5BL_DD4C9F446.1	PF00069	KOG1187	2.7.11.1		GO:GO:0006468,GO:GO:0005524,GO:GO:0004672	AT3G55950.1	CRINKLY4 related 3	LOC_Os05g40150.1	RGH2B, putative, expressed	1,50	-1.0	1	22	62	83	AUAAAACCUUCAGCUAUCCAUC	AAUGGAUAGCGGAAGGUUUUAU	Cleavage
tae-miR7757-5p	Traes_5BL_DD4C9F446.2	PF00069	KOG1187	2.7.11.1		GO:GO:0006468,GO:GO:0005524,GO:GO:0004672	AT3G55950.1	CRINKLY4 related 3	LOC_Os05g40150.1	RGH2B, putative, expressed	1,50	-1.0	1	22	62	83	AUAAAACCUUCAGCUAUCCAUC	AAUGGAUAGCGGAAGGUUUUAU	Cleavage
tae-miR7757-5p	Traes_5BL_DD4C9F446.4						AT3G46710.1	NB-ARC domain-containing disease resistance protein	LOC_Os05g40150.1	RGH2B, putative, expressed	1,50	-1.0	1	22	62	83	AUAAAACCUUCAGCUAUCCAUC	AAUGGAUAGCGGAAGGUUUUAU	Cleavage
tae-miR7757-5p	Traes_5BL_DD4C9F446.5						AT3G46710.1	NB-ARC domain-containing disease resistance protein	LOC_Os05g40150.1	RGH2B, putative, expressed	1,50	-1.0	1	22	62	83	AUAAAACCUUCAGCUAUCCAUC	AAUGGAUAGCGGAAGGUUUUAU	Cleavage
tae-miR7757-5p	Traes_5BL_DD4C9F446.6						AT3G46710.1	NB-ARC domain-containing disease resistance protein	LOC_Os05g40150.1	RGH2B, putative, expressed	1,50	-1.0	1	22	62	83	AUAAAACCUUCAGCUAUCCAUC	AAUGGAUAGCGGAAGGUUUUAU	Cleavage
tae-miR9661-5p	Traes_5BL_E0C2C7D72.1										3,00	-1.0	1	21	98	118	UGAAGUAGAGCAGGGACCUCA	UCGUCUCCCUGCUCUACUUCA	Cleavage
tae-miR9661-5p	Traes_5BL_E0C2C7D72.2										3,00	-1.0	1	21	98	118	UGAAGUAGAGCAGGGACCUCA	UCGUCUCCCUGCUCUACUUCA	Cleavage
tae-miR9661-5p	Traes_5BL_E0C2C7D72.3										3,00	-1.0	1	21	95	115	UGAAGUAGAGCAGGGACCUCA	UCGUCUCCCUGCUCUACUUCA	Cleavage
tae-miR9652-5p	Traes_5BL_FC199BC2D.1	PF16488,PF02170				GO:GO:0005515	AT1G48410.2	Stabilizer of iron transporter SufD / Polynucleotidyl transferase	LOC_Os03g58600.1	PAZ domain containing protein, putative, expressed	2,50	-1.0	1	22	412	433	CCUGUUUGUCAUUAAGUUUCUU	AAGAAACUGAAUGACAAGCAGG	Cleavage
tae-miR1122b-3p	Traes_5BS_321AF7B69.2	PF14416,PF13839					AT5G20680.3	TRICHOME BIREFRINGENCE-LIKE 16	LOC_Os12g33194.1	expressed protein	2,50	-1.0	1	21	23	43	AGACUUAUAUGUAGGAACGGA	UCCAUUCCUAAAUAUAAGUCU	Translation
tae-miR1122a	Traes_5BS_321AF7B69.2	PF14416,PF13839					AT5G20680.3	TRICHOME BIREFRINGENCE-LIKE 16	LOC_Os12g33194.1	expressed protein	2,50	-1.0	1	20	63	82	UAGAUACAUCCGUAUCUAGA	ACUACAUACGGAUGUAUAUA	Cleavage
tae-miR7757-5p	Traes_5BS_3FFCC89AD.1	PF00931	KOG4658			GO:GO:0043531	AT3G07040.1	NB-ARC domain-containing disease resistance protein	LOC_Os12g37770.1	RGH1A, putative, expressed	2,50	-1.0	1	22	1337	1358	AUAAAACCUUCAGCUAUCCAUC	GGUGGAUUGCUGAAGGAUUUAU	Cleavage
tae-miR7757-5p	Traes_5BS_3FFCC89AD.2	PF00931	KOG4658			GO:GO:0043531	AT3G46730.1	NB-ARC domain-containing disease resistance protein	LOC_Os12g37770.1	RGH1A, putative, expressed	2,50	-1.0	1	22	1298	1319	AUAAAACCUUCAGCUAUCCAUC	GGUGGAUUGCUGAAGGAUUUAU	Cleavage
tae-miR1118	Traes_5BS_550BDEE86.2	PF00013	KOG2190			GO:GO:0003723	AT3G04610.1	RNA-binding KH domain-containing protein	LOC_Os12g40560.1	KH domain containing protein, putative, expressed	1,50	-1.0	1	23	1135	1157	CACUACAUUAUGGAAUGGAGGGA	UCCCUCCAUUCCACAAUGUAGUG	Translation
tae-miR5384-3p	Traes_5BS_90796C6CA.1	PF07762							LOC_Os01g56900.1	expressed protein	2,00	-1.0	1	21	461	481	UGAGCGCGCCGCCGUCGAAUG	UGUACGAUGGCGGCGCGCUCA	Cleavage
tae-miR5384-3p	Traes_5BS_90796C6CA.2	PF07762							LOC_Os01g56900.1	expressed protein	2,00	-1.0	1	21	34	54	UGAGCGCGCCGCCGUCGAAUG	UGUACGAUGGCGGCGCGCUCA	Cleavage
tae-miR5384-3p	Traes_5BS_90796C6CA.3	PF07762							LOC_Os01g56900.1	expressed protein	2,00	-1.0	1	21	392	412	UGAGCGCGCCGCCGUCGAAUG	UGUACGAUGGCGGCGCGCUCA	Cleavage
tae-miR164	Traes_5BS_9F043AE17.1	PF02365				GO:GO:0006355,GO:GO:0003677	AT1G56010.2	NAC domain containing protein 1	LOC_Os12g41680.1	No apical meristem protein, putative, expressed	1,00	-1.0	1	21	656	676	UGGAGAAGCAGGGCACGUGCA	AGCAAGUGCCCUGCUUCUCCA	Cleavage
tae-miR5384-3p	Traes_5BS_BA0400E10.1	PF14570,PF03552		2.4.2.24	K00770	GO:GO:0030244,GO:GO:0016760,GO:GO:0016020	AT1G02730.1	cellulose synthase-like D5	LOC_Os12g36890.1	CSLD4 - cellulose synthase-like family D, expressed	3,00	-1.0	1	21	1358	1378	UGAGCGCGCCGCCGUCGAAUG	CCGACGACGGCGGCGCGCUCC	Cleavage
tae-miR7757-5p	Traes_5BS_BCEA0112C.1	PF00931	KOG4658			GO:GO:0043531	AT3G07040.1	NB-ARC domain-containing disease resistance protein	LOC_Os12g37740.1	resistance protein LR10, putative, expressed	2,50	-1.0	1	22	1340	1361	AUAAAACCUUCAGCUAUCCAUC	GAUGGAUCGCGGAAGGUUUUAU	Cleavage
tae-miR7757-5p	Traes_5BS_BCEA0112C.2	PF00931	KOG4658			GO:GO:0043531	AT3G07040.1	NB-ARC domain-containing disease resistance protein	LOC_Os12g37740.1	resistance protein LR10, putative, expressed	2,50	-1.0	1	22	1340	1361	AUAAAACCUUCAGCUAUCCAUC	GAUGGAUCGCGGAAGGUUUUAU	Cleavage
tae-miR7757-5p	Traes_5BS_BCEA0112C.3	PF00931	KOG4658			GO:GO:0043531	AT3G07040.1	NB-ARC domain-containing disease resistance protein	LOC_Os12g37740.1	resistance protein LR10, putative, expressed	2,50	-1.0	1	22	1307	1328	AUAAAACCUUCAGCUAUCCAUC	GAUGGAUCGCGGAAGGUUUUAU	Cleavage
tae-miR7757-5p	Traes_5BS_BCEA0112C.4	PF00931				GO:GO:0043531	AT3G07040.1	NB-ARC domain-containing disease resistance protein	LOC_Os12g37740.1	resistance protein LR10, putative, expressed	2,50	-1.0	1	22	128	149	AUAAAACCUUCAGCUAUCCAUC	GAUGGAUCGCGGAAGGUUUUAU	Cleavage
tae-miR408	Traes_5BS_E10756355.1										2,50	-1.0	1	21	2512	2532	CUGCACUGCCUCUUCCCUGGC	GCCAAGGAAGAGGCUGUGCAG	Cleavage
tae-miR7757-5p	Traes_5BS_F5AFB24CD.2	PF00931				GO:GO:0043531	AT3G46530.1	NB-ARC domain-containing disease resistance protein	LOC_Os10g03570.1	RGH1A, putative, expressed	1,50	-1.0	1	22	779	800	AUAAAACCUUCAGCUAUCCAUC	GGUGGAUAGCUGAAGGUCUUAU	Cleavage
tae-miR7757-5p	Traes_5BS_F5AFB24CD.3	PF00931				GO:GO:0043531	AT3G46530.1	NB-ARC domain-containing disease resistance protein	LOC_Os10g03570.1	RGH1A, putative, expressed	1,50	-1.0	1	22	779	800	AUAAAACCUUCAGCUAUCCAUC	GGUGGAUAGCUGAAGGUCUUAU	Cleavage
tae-miR7757-5p	Traes_5BS_F5AFB24CD.4	PF00931				GO:GO:0043531	AT5G43470.2	Disease resistance protein (CC-NBS-LRR class) family	LOC_Os10g03570.1	RGH1A, putative, expressed	1,50	-1.0	1	22	371	392	AUAAAACCUUCAGCUAUCCAUC	GGUGGAUAGCUGAAGGUCUUAU	Cleavage

tae-miR156	Traes_5DL_1272B4BAC.1	PF03110				GO:GO:0005634,GO:GO:0003677	AT5G50670.1	Squamosa promoter-binding protein-like (SBP domain) transcription factor family protein	LOC_Os09g32944.1	OsSPL18 - SBP-box gene family member, expressed	1,00	-1.0	1	21	505	525	UGACAGAAGAGAGUGAGCACA	UGUGCUCUCUCUCUUCUGUCA	Cleavage
tae-miR156	Traes_5DL_1272B4BAC.2	PF03110				GO:GO:0005634,GO:GO:0003677	AT5G50670.1	Squamosa promoter-binding protein-like (SBP domain) transcription factor family protein	LOC_Os09g32944.1	OsSPL18 - SBP-box gene family member, expressed	1,00	-1.0	1	21	487	507	UGACAGAAGAGAGUGAGCACA	UGUGCUCUCUCUCUUCUGUCA	Cleavage
tae-miR156	Traes_5DL_1272B4BAC.3								LOC_Os09g32944.1	OsSPL18 - SBP-box gene family member, expressed	1,00	-1.0	1	21	382	402	UGACAGAAGAGAGUGAGCACA	UGUGCUCUCUCUCUUCUGUCA	Cleavage
tae-miR7757-5p	Traes_5DL_1733FB4DA.1	PF00931,PF03106				GO:GO:0043531,GO:GO:0043565,GO:GO:0006355,GO:GO:0003700	AT3G46530.1	NB-ARC domain-containing disease resistance protein	LOC_Os05g40150.1	RGH2B, putative, expressed	1,50	-1.0	1	22	617	638	AUAAAACCUUCAGCUAUCCAUC	AAUGGAUAGCUGAAGGAUUUAU	Cleavage
tae-miR7757-5p	Traes_5DL_1733FB4DA.2	PF03106,PF00931				GO:GO:0043565,GO:GO:0006355,GO:GO:0003700,GO:GO:0043531	AT3G46530.1	NB-ARC domain-containing disease resistance protein	LOC_Os05g40150.1	RGH2B, putative, expressed	1,50	-1.0	1	22	617	638	AUAAAACCUUCAGCUAUCCAUC	AAUGGAUAGCUGAAGGAUUUAU	Cleavage
tae-miR7757-5p	Traes_5DL_1733FB4DA.3	PF03106,PF00931				GO:GO:0043565,GO:GO:0006355,GO:GO:0003700,GO:GO:0043531	AT3G46530.1	NB-ARC domain-containing disease resistance protein	LOC_Os05g40150.1	RGH2B, putative, expressed	1,50	-1.0	1	22	617	638	AUAAAACCUUCAGCUAUCCAUC	AAUGGAUAGCUGAAGGAUUUAU	Cleavage
tae-miR7757-5p	Traes_5DL_1733FB4DA.4	PF00931				GO:GO:0043531	AT3G46530.1	NB-ARC domain-containing disease resistance protein	LOC_Os05g40150.1	RGH2B, putative, expressed	1,50	-1.0	1	22	617	638	AUAAAACCUUCAGCUAUCCAUC	AAUGGAUAGCUGAAGGAUUUAU	Cleavage
tae-miR7757-5p	Traes_5DL_1733FB4DA.5	PF00931				GO:GO:0043531	AT3G46530.1	NB-ARC domain-containing disease resistance protein	LOC_Os05g40150.1	RGH2B, putative, expressed	1,50	-1.0	1	22	617	638	AUAAAACCUUCAGCUAUCCAUC	AAUGGAUAGCUGAAGGAUUUAU	Cleavage
tae-miR7757-5p	Traes_5DL_1733FB4DA.6	PF00931				GO:GO:0043531	AT3G46530.1	NB-ARC domain-containing disease resistance protein	LOC_Os05g40150.1	RGH2B, putative, expressed	1,50	-1.0	1	22	617	638	AUAAAACCUUCAGCUAUCCAUC	AAUGGAUAGCUGAAGGAUUUAU	Cleavage
tae-miR7757-5p	Traes_5DL_1733FB4DA.7	PF00931				GO:GO:0043531	AT3G46530.1	NB-ARC domain-containing disease resistance protein	LOC_Os11g45750.1	WRKY125, expressed	1,50	-1.0	1	22	617	638	AUAAAACCUUCAGCUAUCCAUC	AAUGGAUAGCUGAAGGAUUUAU	Cleavage
tae-miR7757-5p	Traes_5DL_1733FB4DA.8	PF00931				GO:GO:0043531	AT3G46530.1	NB-ARC domain-containing disease resistance protein	LOC_Os11g45750.2	WRKY125, expressed	1,50	-1.0	1	22	617	638	AUAAAACCUUCAGCUAUCCAUC	AAUGGAUAGCUGAAGGAUUUAU	Cleavage
tae-miR5062-5p	Traes_5DL_26B51AAF8.1	PF02171				GO:GO:0003676	AT1G48410.2	Stabilizer of iron transporter SufD / Polynucleotidyl transferase	LOC_Os03g58600.1	PAZ domain containing protein, putative, expressed	1,50	-1.0	1	23	43	65	UGAACCUUAGGGAACAGCCGCAU	ACCAGGCUGUUCCCUGAGGUUCA	Cleavage
tae-miR1127b-3p	Traes_5DL_4E67E0243.9	PF03828		2.7.7.19			AT3G51620.2	PAP/OAS1 substrate-binding domain superfamily	LOC_Os12g02260.1	nucleotidyltransferase, putative, expressed	1,50	-1.0	1	21	41	61	ACAAGUAUUUCUGGACGGAGG	CCUCCGUCCGGAAAUACUUGU	Cleavage
tae-miR9652-5p	Traes_5DL_714CCB5BC.1	PF16488,PF02170				GO:GO:0005515	AT1G48410.3	Stabilizer of iron transporter SufD / Polynucleotidyl transferase	LOC_Os03g58600.1	PAZ domain containing protein, putative, expressed	2,50	-1.0	1	22	412	433	CCUGUUUGUCAUUAAGUUUCUU	AAGAAACUGAAUGACAAGCAGG	Cleavage
tae-miR9662a-3p	Traes_5DL_7643B2ADB.1	PF02536				GO:GO:0006355,GO:GO:0005739,GO:GO:0003690	AT5G64950.1	Mitochondrial transcription termination factor family protein	LOC_Os06g12100.1	mTERF family protein, expressed	2,50	-1.0	1	21	411	431	UUGAACAUCCCAGAGCCACCG	CCGUGGCUCUGUGAUGUUCAG	Translation
tae-miR9662b-3p	Traes_5DL_7643B2ADB.1	PF02536				GO:GO:0006355,GO:GO:0005739,GO:GO:0003690	AT5G64950.1	Mitochondrial transcription termination factor family protein	LOC_Os06g12100.1	mTERF family protein, expressed	2,50	-1.0	1	21	410	430	UGAACAUCCCAGAGCCACCGG	CCCGUGGCUCUGUGAUGUUCA	Cleavage
tae-miR5384-3p	Traes_5DL_78767F75C.1	PF00135	KOG1515	3.1.1.1			AT3G48690.1	alpha/beta-Hydrolases superfamily protein	LOC_Os09g28730.1	gibberellin receptor GID1L2, putative, expressed	2,50	-1.0	1	21	242	262	UGAGCGCGCCGCCGUCGAAUG	UCUUCCACGGCGGCGCGUUCA	Cleavage
tae-miR5049-3p	Traes_5DL_799647EA7.4										1,50	-1.0	1	21	65	85	AAUAUGGAUCGGAGGGAGUAC	GUACUCCCUCCGUUCCAUAUU	Cleavage
tae-miR9652-5p	Traes_5DL_8DE6C67FA.1	PF02171,PF16487,PF16488,PF02170				GO:GO:0003676,GO:GO:0005515	AT1G48410.2	Stabilizer of iron transporter SufD / Polynucleotidyl transferase	LOC_Os03g58600.1	PAZ domain containing protein, putative, expressed	2,50	-1.0	1	22	412	433	CCUGUUUGUCAUUAAGUUUCUU	AAGAAACUGAAUGACAAGCAGG	Cleavage
tae-miR9652-5p	Traes_5DL_8DE6C67FA.2	PF16487,PF16488,PF02170				GO:GO:0005515	AT1G48410.3	Stabilizer of iron transporter SufD / Polynucleotidyl transferase	LOC_Os03g58600.1	PAZ domain containing protein, putative, expressed	2,50	-1.0	1	22	412	433	CCUGUUUGUCAUUAAGUUUCUU	AAGAAACUGAAUGACAAGCAGG	Cleavage
tae-miR9652-5p	Traes_5DL_8DE6C67FA.3	PF16487,PF16488,PF02170				GO:GO:0005515	AT1G48410.3	Stabilizer of iron transporter SufD / Polynucleotidyl transferase	LOC_Os03g58600.1	PAZ domain containing protein, putative, expressed	2,50	-1.0	1	22	415	436	CCUGUUUGUCAUUAAGUUUCUU	AAGAAACUGAAUGACAAGCAGG	Cleavage
tae-miR9652-5p	Traes_5DL_8DE6C67FA.4	PF02170				GO:GO:0005515	AT1G48410.3	Stabilizer of iron transporter SufD / Polynucleotidyl transferase	LOC_Os03g58600.1	PAZ domain containing protein, putative, expressed	2,50	-1.0	1	22	400	421	CCUGUUUGUCAUUAAGUUUCUU	AAGAAACUGAAUGACAAGCAGG	Cleavage
tae-miR9652-5p	Traes_5DL_8DE6C67FA.5	PF02170				GO:GO:0005515	AT1G48410.2	Stabilizer of iron transporter SufD / Polynucleotidyl transferase	LOC_Os03g58600.1	PAZ domain containing protein, putative, expressed	2,50	-1.0	1	22	400	421	CCUGUUUGUCAUUAAGUUUCUU	AAGAAACUGAAUGACAAGCAGG	Cleavage
tae-miR408	Traes_5DL_90BD9F306.1										0,00	-1.0	1	21	8	28	CUGCACUGCCUCUUCCCUGGC	CCCAGGGAAGAGGCAGUGCAG	Cleavage
tae-miR9666b-3p	Traes_5DL_9101D5AEC.1	PF05001		2.7.7.6		GO:GO:0006366,GO:GO:0005665,GO:GO:0003677	AT4G35800.1	RNA polymerase II large subunit	LOC_Os05g05860.1	retrotransposon protein, putative, unclassified, expressed	2,50	-1.0	1	22	1183	1204	CGGUUGGGCUGUAUGAUGGCGA	AGUCCAUCAUACAGCCCAACAA	Cleavage
tae-miR1130b-3p	Traes_5DL_A6EECBC04.1								LOC_Os03g32160.1	calmodulin binding protein, putative, expressed	1,50	-1.0	1	21	376	396	UCUUAUAUUAUGGGACGGAGG	AUUCCGUCCCAUAAUGUAAGA	Cleavage
tae-miR9652-5p	Traes_5DL_B3713A4D1.1	PF16488,PF08699,PF16486,PF02170				GO:GO:0005515	AT1G48410.1	Stabilizer of iron transporter SufD / Polynucleotidyl transferase	LOC_Os03g58600.1	PAZ domain containing protein, putative, expressed	2,50	-1.0	1	22	940	961	CCUGUUUGUCAUUAAGUUUCUU	AAGAAACUGAAUGACAAGCAGG	Cleavage
tae-miR5086	Traes_5DL_CECECF832.1										3,00	-1.0	1	21	461	481	ACAUUGGUGGAAGGCGUGGUA	AGUCAUGCCUUCCACCAAUGA	Cleavage
tae-miR5086	Traes_5DL_CECECF832.2										3,00	-1.0	1	21	485	505	ACAUUGGUGGAAGGCGUGGUA	AGUCAUGCCUUCCACCAAUGA	Cleavage
tae-miR5086	Traes_5DL_CECECF832.3										3,00	-1.0	1	21	101	121	ACAUUGGUGGAAGGCGUGGUA	AGUCAUGCCUUCCACCAAUGA	Cleavage
tae-miR5086	Traes_5DL_CECECF832.4										3,00	-1.0	1	21	485	505	ACAUUGGUGGAAGGCGUGGUA	AGUCAUGCCUUCCACCAAUGA	Cleavage
tae-miR5086	Traes_5DL_CECECF832.5										3,00	-1.0	1	21	101	121	ACAUUGGUGGAAGGCGUGGUA	AGUCAUGCCUUCCACCAAUGA	Cleavage
tae-miR5062-5p	Traes_5DL_F5D3E4763.1	PF02171				GO:GO:0003676	AT1G48410.3	Stabilizer of iron transporter SufD / Polynucleotidyl transferase	LOC_Os03g58600.1	PAZ domain containing protein, putative, expressed	2,50	-1.0	1	23	43	65	UGAACCUUAGGGAACAGCCGCAU	ACCAGGUUGUUCCCUGAGGUUCA	Cleavage
tae-miR7757-5p	Traes_5DS_0479C6E0F.1	PF00931				GO:GO:0043531	AT3G07040.1	NB-ARC domain-containing disease resistance protein	LOC_Os12g06920.1	NBS-LRR disease resistance protein, putative, expressed	2,50	-1.0	1	22	383	404	AUAAAACCUUCAGCUAUCCAUC	GAUGGAUCGCUGAAGGUUUGAU	Cleavage
tae-miR9662a-3p	Traes_5DS_094A03C13.1	PF02536				GO:GO:0006355,GO:GO:0005739,GO:GO:0003690	AT5G07900.1	Mitochondrial transcription termination factor family protein	LOC_Os06g12100.1	mTERF family protein, expressed	1,00	-1.0	1	21	549	569	UUGAACAUCCCAGAGCCACCG	CCGUGGCUCUGGGAUGUUCAG	Cleavage
tae-miR9662b-3p	Traes_5DS_094A03C13.1	PF02536				GO:GO:0006355,GO:GO:0005739,GO:GO:0003690	AT5G07900.1	Mitochondrial transcription termination factor family protein	LOC_Os06g12100.1	mTERF family protein, expressed	1,00	-1.0	1	21	548	568	UGAACAUCCCAGAGCCACCGG	CCCGUGGCUCUGGGAUGUUCA	Cleavage
tae-miR9662a-3p	Traes_5DS_094A03C13.2	PF02536				GO:GO:0006355,GO:GO:0005739,GO:GO:0003690	AT5G07900.1	Mitochondrial transcription termination factor family protein	LOC_Os06g12100.1	mTERF family protein, expressed	1,00	-1.0	1	21	243	263	UUGAACAUCCCAGAGCCACCG	CCGUGGCUCUGGGAUGUUCAG	Cleavage
tae-miR9662b-3p	Traes_5DS_094A03C13.2	PF02536				GO:GO:0006355,GO:GO:0005739,GO:GO:0003690	AT5G07900.1	Mitochondrial transcription termination factor family protein	LOC_Os06g12100.1	mTERF family protein, expressed	1,00	-1.0	1	21	242	262	UGAACAUCCCAGAGCCACCGG	CCCGUGGCUCUGGGAUGUUCA	Cleavage
tae-miR5384-3p	Traes_5DS_149714893.1	PF04535					AT5G44550.1	Uncharacterised protein family (UPF0497)	LOC_Os12g41690.1	membrane associated DUF588 domain containing protein, putative, expressed	2,50	-1.0	1	21	158	178	UGAGCGCGCCGCCGUCGAAUG	CGUUCGUCGGCGUCGCGCUCA	Cleavage
tae-miR5384-3p	Traes_5DS_149714893.2	PF04535					AT5G44550.1	Uncharacterised protein family (UPF0497)	LOC_Os12g41690.1	membrane associated DUF588 domain containing protein, putative, expressed	2,50	-1.0	1	21	152	172	UGAGCGCGCCGCCGUCGAAUG	CGUUCGUCGGCGUCGCGCUCA	Cleavage
tae-miR164	Traes_5DS_511F35A45.1						AT1G56010.2	NAC domain containing protein 1	LOC_Os12g41680.1	No apical meristem protein, putative, expressed	1,00	-1.0	1	21	209	229	UGGAGAAGCAGGGCACGUGCA	AGCAAGUGCCCUGCUUCUCCA	Cleavage
tae-miR7757-5p	Traes_5DS_8DB03B7DA.1	PF00931	KOG4658			GO:GO:0043531	AT3G07040.1	NB-ARC domain-containing disease resistance protein	LOC_Os12g37740.1	resistance protein LR10, putative, expressed	2,50	-1.0	1	22	1349	1370	AUAAAACCUUCAGCUAUCCAUC	GAUGGAUCGCGGAAGGUUUUAU	Cleavage
tae-miR7757-5p	Traes_5DS_8DB03B7DA.2	PF00931				GO:GO:0043531	AT3G07040.1	NB-ARC domain-containing disease resistance protein	LOC_Os12g37740.1	resistance protein LR10, putative, expressed	2,50	-1.0	1	22	128	149	AUAAAACCUUCAGCUAUCCAUC	GAUGGAUCGCGGAAGGUUUUAU	Cleavage
tae-miR7757-5p	Traes_5DS_9DFE4D217.1	PF00931				GO:GO:0043531	AT1G53350.1	Disease resistance protein (CC-NBS-LRR class) family	LOC_Os10g22484.1	RGH2B, putative, expressed	2,50	-1.0	1	22	185	206	AUAAAACCUUCAGCUAUCCAUC	AAUGGGUAGCUGAAGGUUGUAU	Cleavage
tae-miR7757-5p	Traes_5DS_9DFE4D217.2	PF00931				GO:GO:0043531	AT1G53350.1	Disease resistance protein (CC-NBS-LRR class) family	LOC_Os10g22484.1	RGH2B, putative, expressed	2,50	-1.0	1	22	185	206	AUAAAACCUUCAGCUAUCCAUC	AAUGGGUAGCUGAAGGUUGUAU	Cleavage
tae-miR7757-5p	Traes_5DS_9DFE4D217.3	PF00931				GO:GO:0043531	AT1G53350.1	Disease resistance protein (CC-NBS-LRR class) family	LOC_Os10g22484.1	RGH2B, putative, expressed	2,50	-1.0	1	22	185	206	AUAAAACCUUCAGCUAUCCAUC	AAUGGGUAGCUGAAGGUUGUAU	Cleavage
tae-miR9662a-3p	Traes_5DS_9F4A5AE9F.1	PF02536				GO:GO:0006355,GO:GO:0005739,GO:GO:0003690	AT5G07900.1	Mitochondrial transcription termination factor family protein	LOC_Os06g12100.1	mTERF family protein, expressed	2,00	-1.0	1	21	441	461	UUGAACAUCCCAGAGCCACCG	UCGUGGCGCUGGGAUGUUCAG	Cleavage
tae-miR9662b-3p	Traes_5DS_9F4A5AE9F.1	PF02536				GO:GO:0006355,GO:GO:0005739,GO:GO:0003690	AT5G07900.1	Mitochondrial transcription termination factor family protein	LOC_Os06g12100.1	mTERF family protein, expressed	2,50	-1.0	1	21	440	460	UGAACAUCCCAGAGCCACCGG	CUCGUGGCGCUGGGAUGUUCA	Cleavage
tae-miR9662a-3p	Traes_5DS_9F4A5AE9F.2	PF02536				GO:GO:0006355,GO:GO:0005739,GO:GO:0003690	AT5G07900.1	Mitochondrial transcription termination factor family protein	LOC_Os06g12100.1	mTERF family protein, expressed	2,00	-1.0	1	21	66	86	UUGAACAUCCCAGAGCCACCG	UCGUGGCGCUGGGAUGUUCAG	Cleavage
tae-miR9662b-3p	Traes_5DS_9F4A5AE9F.2	PF02536				GO:GO:0006355,GO:GO:0005739,GO:GO:0003690	AT5G07900.1	Mitochondrial transcription termination factor family protein	LOC_Os06g12100.1	mTERF family protein, expressed	2,50	-1.0	1	21	65	85	UGAACAUCCCAGAGCCACCGG	CUCGUGGCGCUGGGAUGUUCA	Cleavage
tae-miR1122b-3p	Traes_5DS_9F8FFD461.1	PF00792,PF00613		2.7.1.137		GO:GO:0048015,GO:GO:0046854	AT1G60490.1	vacuolar protein sorting 34	LOC_Os05g08810.2	phosphatidylinositol 3-kinase, root isoform, putative, expressed	1,50	-1.0	1	21	1777	1797	AGACUUAUAUGUAGGAACGGA	UCCGUUCCUAAAUAUAAGUCU	Translation
tae-miR1122b-3p	Traes_5DS_9F8FFD461.2	PF00792,PF00613		2.7.1.137		GO:GO:0048015,GO:GO:0046854	AT1G60490.1	vacuolar protein sorting 34	LOC_Os05g08810.2	phosphatidylinositol 3-kinase, root isoform, putative, expressed	1,50	-1.0	1	21	1882	1902	AGACUUAUAUGUAGGAACGGA	UCCGUUCCUAAAUAUAAGUCU	Translation
tae-miR1122b-3p	Traes_5DS_9F8FFD461.3	PF00792,PF00613		2.7.1.137		GO:GO:0048015,GO:GO:0046854	AT1G60490.1	vacuolar protein sorting 34	LOC_Os05g08810.2	phosphatidylinositol 3-kinase, root isoform, putative, expressed	1,50	-1.0	1	21	1801	1821	AGACUUAUAUGUAGGAACGGA	UCCGUUCCUAAAUAUAAGUCU	Translation
tae-miR1122b-3p	Traes_5DS_A6AE32DCD.1	PF03483	KOG2472	6.1.1.20		GO:GO:0004826,GO:GO:0003723	AT1G72550.1	tRNA synthetase beta subunit family protein	LOC_Os05g48510.1	phenylalanyl-tRNA synthetase beta chain, putative, expressed	1,50	-1.0	1	21	1065	1085	AGACUUAUAUGUAGGAACGGA	UCCGUUCCUAAAUAUAAGUCU	Translation
tae-miR1122b-3p	Traes_5DS_A6AE32DCD.2	PF03484,PF03483		6.1.1.20		GO:GO:0006432,GO:GO:0005524,GO:GO:0003723,GO:GO:0000287,GO:GO:0004826	AT1G72550.2	tRNA synthetase beta subunit family protein	LOC_Os05g48510.1	phenylalanyl-tRNA synthetase beta chain, putative, expressed	1,50	-1.0	1	21	1146	1166	AGACUUAUAUGUAGGAACGGA	UCCGUUCCUAAAUAUAAGUCU	Translation
tae-miR1122b-3p	Traes_5DS_A6AE32DCD.3	PF03484,PF03483		6.1.1.20		GO:GO:0006432,GO:GO:0005524,GO:GO:0003723,GO:GO:0000287,GO:GO:0004826	AT1G72550.2	tRNA synthetase beta subunit family protein	LOC_Os05g48510.1	phenylalanyl-tRNA synthetase beta chain, putative, expressed	1,50	-1.0	1	21	1146	1166	AGACUUAUAUGUAGGAACGGA	UCCGUUCCUAAAUAUAAGUCU	Translation
tae-miR9662a-3p	Traes_5DS_C87840EBA.2						AT1G79220.1	Mitochondrial transcription termination factor family protein	LOC_Os06g12100.1	mTERF family protein, expressed	2,00	-1.0	1	21	429	449	UUGAACAUCCCAGAGCCACCG	CCGUGGUUCUGGGAUGUUCAG	Cleavage
tae-miR9662b-3p	Traes_5DS_C87840EBA.2						AT1G79220.1	Mitochondrial transcription termination factor family protein	LOC_Os06g12100.1	mTERF family protein, expressed	2,00	-1.0	1	21	428	448	UGAACAUCCCAGAGCCACCGG	CCCGUGGUUCUGGGAUGUUCA	Cleavage
tae-miR1128	Traes_5DS_D165422B3.1	PF00503	KOG0082			GO:GO:0031683,GO:GO:0019001,GO:GO:0007186,GO:GO:0004871,GO:GO:0003924	AT2G23460.1	extra-large G-protein 1	LOC_Os12g40190.1	G-protein alpha subunit, putative, expressed	3,00	-1.0	1	21	59	80	UACUACUCCCUCCG-UCCGAAA	UUUAGGAACGGAGGGAGUAGUA	Cleavage
tae-miR1118	Traes_5DS_F8D323D4D.2	PF00013	KOG2190			GO:GO:0003723	AT3G04610.1	RNA-binding KH domain-containing protein	LOC_Os12g40560.1	KH domain containing protein, putative, expressed	1,50	-1.0	1	23	1329	1351	CACUACAUUAUGGAAUGGAGGGA	CUCCUCCAUUCCACAAUGUAGUG	Translation
tae-miR160	Traes_6AL_232AF0B76.1						AT4G30080.1	auxin response factor 16	LOC_Os02g41800.1	auxin response factor, putative, expressed	0,00	-1.0	1	21	195	215	UGCCUGGCUCCCUGUAUGCCA	AGGCAUACAGGGAGCCAGGCA	Cleavage
tae-miR1121	Traes_6AL_23B31796F.2	PF00230	KOG0223			GO:GO:0016020,GO:GO:0006810,GO:GO:0005215	AT3G53420.1	plasma membrane intrinsic protein 2A	LOC_Os02g41860.3	aquaporin protein, putative, expressed	2,00	-1.0	1	22	846	867	AGUAGUGAUCUAAACGCUCUUA	UAAAAGUGUUUAGAUCACUACU	Cleavage
tae-miR1121	Traes_6AL_23B31796F.3	PF00230	KOG0223			GO:GO:0016020,GO:GO:0006810,GO:GO:0005215	AT3G53420.1	plasma membrane intrinsic protein 2A	LOC_Os02g41860.3	aquaporin protein, putative, expressed	2,00	-1.0	1	22	760	781	AGUAGUGAUCUAAACGCUCUUA	UAAAAGUGUUUAGAUCACUACU	Cleavage
tae-miR1121	Traes_6AL_23B31796F.5	PF00230	KOG0223			GO:GO:0016020,GO:GO:0006810,GO:GO:0005215	AT3G53420.1	plasma membrane intrinsic protein 2A	LOC_Os02g41860.3	aquaporin protein, putative, expressed	2,00	-1.0	1	22	742	763	AGUAGUGAUCUAAACGCUCUUA	UAAAAGUGUUUAGAUCACUACU	Cleavage
tae-miR5049-3p	Traes_6AL_36AE1B4CF.4	PF03031		3.1.3.16			AT4G21670.1	C-terminal domain phosphatase-like 1	LOC_Os02g42600.1	double-stranded RNA binding motif containing protein, expressed	1,50	-1.0	1	21	1787	1807	AAUAUGGAUCGGAGGGAGUAC	AUACUCCCUCCGACCCAUAUU	Cleavage
tae-miR5049-3p	Traes_6AL_36AE1B4CF1.4	PF03031		3.1.3.16			AT4G21670.1	C-terminal domain phosphatase-like 1	LOC_Os02g42600.1	double-stranded RNA binding motif containing protein, expressed	1,50	-1.0	1	21	1787	1807	AAUAUGGAUCGGAGGGAGUAC	AUACUCCCUCCGACCCAUAUU	Cleavage
tae-miR5049-3p	Traes_6AL_6AD463C67.3			3.6.4.12			AT2G01440.1	DEAD/DEAH box RNA helicase family protein			2,50	-1.0	1	21	15	35	AAUAUGGAUCGGAGGGAGUAC	GUACUCCUUCCGAUCCAUAAU	Cleavage
tae-miR1121	Traes_6AL_7E70268F1.3	PF00719		3.6.1.1		GO:GO:0006796,GO:GO:0005737,GO:GO:0004427,GO:GO:0000287	AT2G46860.1	pyrophosphorylase 3	LOC_Os02g47600.1	soluble inorganic pyrophosphatase, putative, expressed	0,00	-1.0	1	22	497	518	AGUAGUGAUCUAAACGCUCUUA	UAAGAGCGUUUAGAUCACUACU	Cleavage
tae-miR1122b-3p	Traes_6AL_94CA6F0D8.5	PF00067				GO:GO:0055114,GO:GO:0020037,GO:GO:0016705,GO:GO:0005506	AT1G31800.1	cytochrome P450, family 97, subfamily A, polypeptide 3	LOC_Os02g57290.2	cytochrome P450, putative, expressed	2,50	-1.0	1	21	1030	1050	AGACUUAUAUGUAGGAACGGA	UCCAUUCCUAAAUAUAAGUCU	Translation
tae-miR1122a	Traes_6AL_94CA6F0D8.5	PF00067				GO:GO:0055114,GO:GO:0020037,GO:GO:0016705,GO:GO:0005506	AT1G31800.1	cytochrome P450, family 97, subfamily A, polypeptide 3	LOC_Os02g57290.2	cytochrome P450, putative, expressed	2,50	-1.0	1	20	1071	1090	UAGAUACAUCCGUAUCUAGA	ACUACAUACGGAUGUAUAUA	Cleavage
tae-miR9662a-3p	Traes_6AS_0A204AEC9.1	PF02536	KOG1267		K15032	GO:GO:0006355,GO:GO:0005739,GO:GO:0003690	AT5G07900.1	Mitochondrial transcription termination factor family protein	LOC_Os06g12100.1	mTERF family protein, expressed	2,00	-1.0	1	21	681	701	UUGAACAUCCCAGAGCCACCG	CCGUGCCUCUGGGAUGUUCAG	Cleavage
tae-miR9662b-3p	Traes_6AS_0A204AEC9.1	PF02536	KOG1267		K15032	GO:GO:0006355,GO:GO:0005739,GO:GO:0003690	AT5G07900.1	Mitochondrial transcription termination factor family protein	LOC_Os06g12100.1	mTERF family protein, expressed	2,00	-1.0	1	21	680	700	UGAACAUCCCAGAGCCACCGG	CCCGUGCCUCUGGGAUGUUCA	Cleavage
tae-miR9662a-3p	Traes_6AS_0A204AEC9.2	PF02536	KOG1267		K15032	GO:GO:0006355,GO:GO:0005739,GO:GO:0003690	AT5G07900.1	Mitochondrial transcription termination factor family protein	LOC_Os06g12100.1	mTERF family protein, expressed	2,00	-1.0	1	21	681	701	UUGAACAUCCCAGAGCCACCG	CCGUGCCUCUGGGAUGUUCAG	Cleavage
tae-miR9662b-3p	Traes_6AS_0A204AEC9.2	PF02536	KOG1267		K15032	GO:GO:0006355,GO:GO:0005739,GO:GO:0003690	AT5G07900.1	Mitochondrial transcription termination factor family protein	LOC_Os06g12100.1	mTERF family protein, expressed	2,00	-1.0	1	21	680	700	UGAACAUCCCAGAGCCACCGG	CCCGUGCCUCUGGGAUGUUCA	Cleavage
tae-miR9662a-3p	Traes_6AS_1496A7825.1	PF02536				GO:GO:0006355,GO:GO:0005739,GO:GO:0003690	AT5G07900.1	Mitochondrial transcription termination factor family protein	LOC_Os06g12100.1	mTERF family protein, expressed	2,50	-1.0	1	21	549	569	UUGAACAUCCCAGAGCCACCG	CCGUGGCUCGGGGAUGUUCAA	Cleavage
tae-miR9662b-3p	Traes_6AS_1496A7825.1	PF02536				GO:GO:0006355,GO:GO:0005739,GO:GO:0003690	AT5G07900.1	Mitochondrial transcription termination factor family protein	LOC_Os06g12100.1	mTERF family protein, expressed	2,50	-1.0	1	21	548	568	UGAACAUCCCAGAGCCACCGG	CCCGUGGCUCGGGGAUGUUCA	Translation
tae-miR9662a-3p	Traes_6AS_1496A7825.2	PF02536				GO:GO:0006355,GO:GO:0005739,GO:GO:0003690	AT5G07900.1	Mitochondrial transcription termination factor family protein	LOC_Os06g12100.1	mTERF family protein, expressed	2,50	-1.0	1	21	132	152	UUGAACAUCCCAGAGCCACCG	CCGUGGCUCGGGGAUGUUCAA	Cleavage
tae-miR9662b-3p	Traes_6AS_1496A7825.2	PF02536				GO:GO:0006355,GO:GO:0005739,GO:GO:0003690	AT5G07900.1	Mitochondrial transcription termination factor family protein	LOC_Os06g12100.1	mTERF family protein, expressed	2,50	-1.0	1	21	131	151	UGAACAUCCCAGAGCCACCGG	CCCGUGGCUCGGGGAUGUUCA	Translation
tae-miR9662a-3p	Traes_6AS_15C1E6F18.1	PF02536				GO:GO:0006355,GO:GO:0005739,GO:GO:0003690	AT1G21150.1	Mitochondrial transcription termination factor family protein	LOC_Os06g12100.1	mTERF family protein, expressed	2,50	-1.0	1	21	33	53	UUGAACAUCCCAGAGCCACCG	CUGUGGCUCUGGAAUGUUCAG	Cleavage
tae-miR9662b-3p	Traes_6AS_15C1E6F18.1	PF02536				GO:GO:0006355,GO:GO:0005739,GO:GO:0003690	AT1G21150.1	Mitochondrial transcription termination factor family protein	LOC_Os06g12100.1	mTERF family protein, expressed	2,50	-1.0	1	21	32	52	UGAACAUCCCAGAGCCACCGG	CCUGUGGCUCUGGAAUGUUCA	Cleavage
tae-miR9662a-3p	Traes_6AS_15C1E6F18.2	PF02536				GO:GO:0006355,GO:GO:0005739,GO:GO:0003690	AT1G21150.1	Mitochondrial transcription termination factor family protein	LOC_Os06g12100.1	mTERF family protein, expressed	2,50	-1.0	1	21	33	53	UUGAACAUCCCAGAGCCACCG	CUGUGGCUCUGGAAUGUUCAG	Cleavage
tae-miR9662b-3p	Traes_6AS_15C1E6F18.2	PF02536				GO:GO:0006355,GO:GO:0005739,GO:GO:0003690	AT1G21150.1	Mitochondrial transcription termination factor family protein	LOC_Os06g12100.1	mTERF family protein, expressed	2,50	-1.0	1	21	32	52	UGAACAUCCCAGAGCCACCGG	CCUGUGGCUCUGGAAUGUUCA	Cleavage
tae-miR9662a-3p	Traes_6AS_2BDC1663A.1	PF02536	KOG1267		K15032	GO:GO:0006355,GO:GO:0005739,GO:GO:0003690	AT5G07900.1	Mitochondrial transcription termination factor family protein	LOC_Os06g12100.1	mTERF family protein, expressed	1,00	-1.0	1	21	681	701	UUGAACAUCCCAGAGCCACCG	CCGUGGCUCUGGGAUGUUCAG	Cleavage
tae-miR9662b-3p	Traes_6AS_2BDC1663A.1	PF02536	KOG1267		K15032	GO:GO:0006355,GO:GO:0005739,GO:GO:0003690	AT5G07900.1	Mitochondrial transcription termination factor family protein	LOC_Os06g12100.1	mTERF family protein, expressed	1,00	-1.0	1	21	680	700	UGAACAUCCCAGAGCCACCGG	CCCGUGGCUCUGGGAUGUUCA	Cleavage
tae-miR156	Traes_6AS_32A35158A.1	PF03110				GO:GO:0005634,GO:GO:0003677	AT5G43270.2	squamosa promoter binding protein-like 2	LOC_Os06g45310.1	OsSPL11 - SBP-box gene family member, expressed	1,00	-1.0	1	21	736	756	UGACAGAAGAGAGUGAGCACA	CGUGCUCUCUCUCUUCUGUCA	Cleavage
tae-miR156	Traes_6AS_32A35158A.2								LOC_Os06g45310.1	OsSPL11 - SBP-box gene family member, expressed	1,00	-1.0	1	21	292	312	UGACAGAAGAGAGUGAGCACA	CGUGCUCUCUCUCUUCUGUCA	Cleavage
tae-miR156	Traes_6AS_32A35158A.3								LOC_Os06g45310.1	OsSPL11 - SBP-box gene family member, expressed	1,00	-1.0	1	21	64	84	UGACAGAAGAGAGUGAGCACA	CGUGCUCUCUCUCUUCUGUCA	Cleavage
tae-miR9662a-3p	Traes_6AS_3641B9C871.1	PF02536	KOG1267		K15032	GO:GO:0006355,GO:GO:0005739,GO:GO:0003690	AT5G07900.1	Mitochondrial transcription termination factor family protein	LOC_Os06g12100.1	mTERF family protein, expressed	1,00	-1.0	1	21	687	707	UUGAACAUCCCAGAGCCACCG	CCGUGGCUCUGGGAUGUUCAG	Cleavage
tae-miR9662b-3p	Traes_6AS_3641B9C871.1	PF02536	KOG1267		K15032	GO:GO:0006355,GO:GO:0005739,GO:GO:0003690	AT5G07900.1	Mitochondrial transcription termination factor family protein	LOC_Os06g12100.1	mTERF family protein, expressed	1,00	-1.0	1	21	686	706	UGAACAUCCCAGAGCCACCGG	CCCGUGGCUCUGGGAUGUUCA	Cleavage
tae-miR9662a-3p	Traes_6AS_3641B9C871.2	PF02536	KOG1267		K15032	GO:GO:0006355,GO:GO:0005739,GO:GO:0003690	AT5G07900.1	Mitochondrial transcription termination factor family protein	LOC_Os06g12100.1	mTERF family protein, expressed	1,00	-1.0	1	21	687	707	UUGAACAUCCCAGAGCCACCG	CCGUGGCUCUGGGAUGUUCAG	Cleavage
tae-miR9662b-3p	Traes_6AS_3641B9C871.2	PF02536	KOG1267		K15032	GO:GO:0006355,GO:GO:0005739,GO:GO:0003690	AT5G07900.1	Mitochondrial transcription termination factor family protein	LOC_Os06g12100.1	mTERF family protein, expressed	1,00	-1.0	1	21	686	706	UGAACAUCCCAGAGCCACCGG	CCCGUGGCUCUGGGAUGUUCA	Cleavage
tae-miR9662a-3p	Traes_6AS_36EAEADDD.1	PF02536				GO:GO:0006355,GO:GO:0005739,GO:GO:0003690	AT1G21150.1	Mitochondrial transcription termination factor family protein	LOC_Os06g12100.1	mTERF family protein, expressed	2,00	-1.0	1	21	33	53	UUGAACAUCCCAGAGCCACCG	CUGUGGUUCUGGGAUGUUCAG	Cleavage
tae-miR9662b-3p	Traes_6AS_36EAEADDD.1	PF02536				GO:GO:0006355,GO:GO:0005739,GO:GO:0003690	AT1G21150.1	Mitochondrial transcription termination factor family protein	LOC_Os06g12100.1	mTERF family protein, expressed	2,00	-1.0	1	21	32	52	UGAACAUCCCAGAGCCACCGG	CCUGUGGUUCUGGGAUGUUCA	Cleavage
tae-miR9662a-3p	Traes_6AS_36EAEADDD.2								LOC_Os06g12100.1	mTERF family protein, expressed	2,00	-1.0	1	21	33	53	UUGAACAUCCCAGAGCCACCG	CUGUGGUUCUGGGAUGUUCAG	Cleavage
tae-miR9662b-3p	Traes_6AS_36EAEADDD.2								LOC_Os06g12100.1	mTERF family protein, expressed	2,00	-1.0	1	21	32	52	UGAACAUCCCAGAGCCACCGG	CCUGUGGUUCUGGGAUGUUCA	Cleavage
tae-miR9662a-3p	Traes_6AS_36EAEADDD.3	PF02536				GO:GO:0006355,GO:GO:0005739,GO:GO:0003690	AT1G21150.1	Mitochondrial transcription termination factor family protein	LOC_Os06g12100.1	mTERF family protein, expressed	2,00	-1.0	1	21	33	53	UUGAACAUCCCAGAGCCACCG	CUGUGGUUCUGGGAUGUUCAG	Cleavage
tae-miR9662b-3p	Traes_6AS_36EAEADDD.3	PF02536				GO:GO:0006355,GO:GO:0005739,GO:GO:0003690	AT1G21150.1	Mitochondrial transcription termination factor family protein	LOC_Os06g12100.1	mTERF family protein, expressed	2,00	-1.0	1	21	32	52	UGAACAUCCCAGAGCCACCGG	CCUGUGGUUCUGGGAUGUUCA	Cleavage
tae-miR9662a-3p	Traes_6AS_48B701BB41.1	PF02536				GO:GO:0006355,GO:GO:0005739,GO:GO:0003690	AT1G21150.1	Mitochondrial transcription termination factor family protein	LOC_Os06g12100.1	mTERF family protein, expressed	1,00	-1.0	1	21	33	53	UUGAACAUCCCAGAGCCACCG	ACGUGGCUCUGGGAUGUUCAG	Cleavage
tae-miR9662b-3p	Traes_6AS_48B701BB41.1	PF02536				GO:GO:0006355,GO:GO:0005739,GO:GO:0003690	AT1G21150.1	Mitochondrial transcription termination factor family protein	LOC_Os06g12100.1	mTERF family protein, expressed	1,00	-1.0	1	21	32	52	UGAACAUCCCAGAGCCACCGG	CACGUGGCUCUGGGAUGUUCA	Cleavage
tae-miR9662a-3p	Traes_6AS_48B701BB41.2	PF02536				GO:GO:0006355,GO:GO:0005739,GO:GO:0003690	AT1G21150.1	Mitochondrial transcription termination factor family protein	LOC_Os06g12100.1	mTERF family protein, expressed	1,00	-1.0	1	21	33	53	UUGAACAUCCCAGAGCCACCG	ACGUGGCUCUGGGAUGUUCAG	Cleavage
tae-miR9662b-3p	Traes_6AS_48B701BB41.2	PF02536				GO:GO:0006355,GO:GO:0005739,GO:GO:0003690	AT1G21150.1	Mitochondrial transcription termination factor family protein	LOC_Os06g12100.1	mTERF family protein, expressed	1,00	-1.0	1	21	32	52	UGAACAUCCCAGAGCCACCGG	CACGUGGCUCUGGGAUGUUCA	Cleavage
tae-miR9662a-3p	Traes_6AS_48B701BB41.3	PF02536				GO:GO:0006355,GO:GO:0005739,GO:GO:0003690	AT1G21150.1	Mitochondrial transcription termination factor family protein	LOC_Os06g12100.1	mTERF family protein, expressed	1,00	-1.0	1	21	33	53	UUGAACAUCCCAGAGCCACCG	ACGUGGCUCUGGGAUGUUCAG	Cleavage
tae-miR9662b-3p	Traes_6AS_48B701BB41.3	PF02536				GO:GO:0006355,GO:GO:0005739,GO:GO:0003690	AT1G21150.1	Mitochondrial transcription termination factor family protein	LOC_Os06g12100.1	mTERF family protein, expressed	1,00	-1.0	1	21	32	52	UGAACAUCCCAGAGCCACCGG	CACGUGGCUCUGGGAUGUUCA	Cleavage
tae-miR9662a-3p	Traes_6AS_48B701BB41.4	PF02536				GO:GO:0006355,GO:GO:0005739,GO:GO:0003690	AT1G21150.1	Mitochondrial transcription termination factor family protein	LOC_Os06g12100.1	mTERF family protein, expressed	1,00	-1.0	1	21	33	53	UUGAACAUCCCAGAGCCACCG	ACGUGGCUCUGGGAUGUUCAG	Cleavage
tae-miR9662b-3p	Traes_6AS_48B701BB41.4	PF02536				GO:GO:0006355,GO:GO:0005739,GO:GO:0003690	AT1G21150.1	Mitochondrial transcription termination factor family protein	LOC_Os06g12100.1	mTERF family protein, expressed	1,00	-1.0	1	21	32	52	UGAACAUCCCAGAGCCACCGG	CACGUGGCUCUGGGAUGUUCA	Cleavage
tae-miR9662a-3p	Traes_6AS_48B701BB41.5	PF02536				GO:GO:0006355,GO:GO:0005739,GO:GO:0003690	AT1G21150.1	Mitochondrial transcription termination factor family protein	LOC_Os06g12100.1	mTERF family protein, expressed	1,00	-1.0	1	21	33	53	UUGAACAUCCCAGAGCCACCG	ACGUGGCUCUGGGAUGUUCAG	Cleavage
tae-miR9662b-3p	Traes_6AS_48B701BB41.5	PF02536				GO:GO:0006355,GO:GO:0005739,GO:GO:0003690	AT1G21150.1	Mitochondrial transcription termination factor family protein	LOC_Os06g12100.1	mTERF family protein, expressed	1,00	-1.0	1	21	32	52	UGAACAUCCCAGAGCCACCGG	CACGUGGCUCUGGGAUGUUCA	Cleavage
tae-miR1138	Traes_6AS_5F8725CDA.1	PF02705			K03549	GO:GO:0071805,GO:GO:0016020,GO:GO:0015079	AT4G13420.1	high affinity K+ transporter 5	LOC_Os02g31910.2	potassium transporter, putative, expressed	3,00	-1.0	1	23	2223	2245	GCUUAGAUGUGACAUCCUUAAAA	AGUUAGUUAUGUCACAUCUAAGC	Cleavage
tae-miR1138	Traes_6AS_5F8725CDA.2	PF02705				GO:GO:0071805,GO:GO:0016020,GO:GO:0015079	AT4G13420.1	high affinity K+ transporter 5	LOC_Os02g31910.2	potassium transporter, putative, expressed	3,00	-1.0	1	23	1969	1991	GCUUAGAUGUGACAUCCUUAAAA	AGUUAGUUAUGUCACAUCUAAGC	Cleavage
tae-miR9662a-3p	Traes_6AS_6612DF8D7.1	PF02536				GO:GO:0006355,GO:GO:0005739,GO:GO:0003690	AT5G07900.1	Mitochondrial transcription termination factor family protein	LOC_Os06g12100.1	mTERF family protein, expressed	1,00	-1.0	1	21	81	101	UUGAACAUCCCAGAGCCACCG	CCGUGGCUCUGGGAUGUUCAG	Cleavage
tae-miR9662b-3p	Traes_6AS_6612DF8D7.1	PF02536				GO:GO:0006355,GO:GO:0005739,GO:GO:0003690	AT5G07900.1	Mitochondrial transcription termination factor family protein	LOC_Os06g12100.1	mTERF family protein, expressed	1,00	-1.0	1	21	80	100	UGAACAUCCCAGAGCCACCGG	CCCGUGGCUCUGGGAUGUUCA	Cleavage
tae-miR9662a-3p	Traes_6AS_6612DF8D7.2	PF02536				GO:GO:0006355,GO:GO:0005739,GO:GO:0003690	AT5G07900.1	Mitochondrial transcription termination factor family protein	LOC_Os06g12100.1	mTERF family protein, expressed	1,00	-1.0	1	21	81	101	UUGAACAUCCCAGAGCCACCG	CCGUGGCUCUGGGAUGUUCAG	Cleavage
tae-miR9662b-3p	Traes_6AS_6612DF8D7.2	PF02536				GO:GO:0006355,GO:GO:0005739,GO:GO:0003690	AT5G07900.1	Mitochondrial transcription termination factor family protein	LOC_Os06g12100.1	mTERF family protein, expressed	1,00	-1.0	1	21	80	100	UGAACAUCCCAGAGCCACCGG	CCCGUGGCUCUGGGAUGUUCA	Cleavage
tae-miR9662a-3p	Traes_6AS_6612DF8D7.3	PF02536				GO:GO:0006355,GO:GO:0005739,GO:GO:0003690	AT5G07900.1	Mitochondrial transcription termination factor family protein	LOC_Os06g12100.1	mTERF family protein, expressed	1,00	-1.0	1	21	81	101	UUGAACAUCCCAGAGCCACCG	CCGUGGCUCUGGGAUGUUCAG	Cleavage
tae-miR9662b-3p	Traes_6AS_6612DF8D7.3	PF02536				GO:GO:0006355,GO:GO:0005739,GO:GO:0003690	AT5G07900.1	Mitochondrial transcription termination factor family protein	LOC_Os06g12100.1	mTERF family protein, expressed	1,00	-1.0	1	21	80	100	UGAACAUCCCAGAGCCACCGG	CCCGUGGCUCUGGGAUGUUCA	Cleavage
tae-miR9662a-3p	Traes_6AS_8D2111A83.1	PF02536				GO:GO:0006355,GO:GO:0005739,GO:GO:0003690	AT1G21150.1	Mitochondrial transcription termination factor family protein	LOC_Os06g12100.1	mTERF family protein, expressed	2,50	-1.0	1	21	33	53	UUGAACAUCCCAGAGCCACCG	UCGUGGCUCGGGGAUGUUCAG	Cleavage
tae-miR9662b-3p	Traes_6AS_8D2111A83.1	PF02536				GO:GO:0006355,GO:GO:0005739,GO:GO:0003690	AT1G21150.1	Mitochondrial transcription termination factor family protein	LOC_Os06g12100.1	mTERF family protein, expressed	2,50	-1.0	1	21	32	52	UGAACAUCCCAGAGCCACCGG	CUCGUGGCUCGGGGAUGUUCA	Translation
tae-miR9662a-3p	Traes_6AS_8D2111A83.2	PF02536				GO:GO:0006355,GO:GO:0005739,GO:GO:0003690	AT1G21150.1	Mitochondrial transcription termination factor family protein	LOC_Os06g12100.1	mTERF family protein, expressed	2,50	-1.0	1	21	246	266	UUGAACAUCCCAGAGCCACCG	UCGUGGCUCGGGGAUGUUCAG	Cleavage
tae-miR9662b-3p	Traes_6AS_8D2111A83.2	PF02536				GO:GO:0006355,GO:GO:0005739,GO:GO:0003690	AT1G21150.1	Mitochondrial transcription termination factor family protein	LOC_Os06g12100.1	mTERF family protein, expressed	2,50	-1.0	1	21	245	265	UGAACAUCCCAGAGCCACCGG	CUCGUGGCUCGGGGAUGUUCA	Translation
tae-miR9662a-3p	Traes_6AS_8D2111A83.3	PF02536				GO:GO:0006355,GO:GO:0005739,GO:GO:0003690	AT1G21150.1	Mitochondrial transcription termination factor family protein	LOC_Os06g12100.1	mTERF family protein, expressed	2,50	-1.0	1	21	246	266	UUGAACAUCCCAGAGCCACCG	UCGUGGCUCGGGGAUGUUCAG	Cleavage
tae-miR9662b-3p	Traes_6AS_8D2111A83.3	PF02536				GO:GO:0006355,GO:GO:0005739,GO:GO:0003690	AT1G21150.1	Mitochondrial transcription termination factor family protein	LOC_Os06g12100.1	mTERF family protein, expressed	2,50	-1.0	1	21	245	265	UGAACAUCCCAGAGCCACCGG	CUCGUGGCUCGGGGAUGUUCA	Translation
tae-miR9662a-3p	Traes_6AS_8D2111A83.4	PF02536				GO:GO:0006355,GO:GO:0005739,GO:GO:0003690	AT1G21150.1	Mitochondrial transcription termination factor family protein	LOC_Os06g12100.1	mTERF family protein, expressed	2,50	-1.0	1	21	246	266	UUGAACAUCCCAGAGCCACCG	UCGUGGCUCGGGGAUGUUCAG	Cleavage
tae-miR9662b-3p	Traes_6AS_8D2111A83.4	PF02536				GO:GO:0006355,GO:GO:0005739,GO:GO:0003690	AT1G21150.1	Mitochondrial transcription termination factor family protein	LOC_Os06g12100.1	mTERF family protein, expressed	2,50	-1.0	1	21	245	265	UGAACAUCCCAGAGCCACCGG	CUCGUGGCUCGGGGAUGUUCA	Translation
tae-miR9662a-3p	Traes_6AS_8D2111A83.5	PF02536				GO:GO:0006355,GO:GO:0005739,GO:GO:0003690	AT1G21150.1	Mitochondrial transcription termination factor family protein	LOC_Os06g12100.1	mTERF family protein, expressed	2,50	-1.0	1	21	246	266	UUGAACAUCCCAGAGCCACCG	UCGUGGCUCGGGGAUGUUCAG	Cleavage
tae-miR9662b-3p	Traes_6AS_8D2111A83.5	PF02536				GO:GO:0006355,GO:GO:0005739,GO:GO:0003690	AT1G21150.1	Mitochondrial transcription termination factor family protein	LOC_Os06g12100.1	mTERF family protein, expressed	2,50	-1.0	1	21	245	265	UGAACAUCCCAGAGCCACCGG	CUCGUGGCUCGGGGAUGUUCA	Translation
tae-miR9662a-3p	Traes_6AS_8D2111A83.6						AT1G21150.1	Mitochondrial transcription termination factor family protein	LOC_Os06g12100.1	mTERF family protein, expressed	2,50	-1.0	1	21	246	266	UUGAACAUCCCAGAGCCACCG	UCGUGGCUCGGGGAUGUUCAG	Cleavage
tae-miR9662b-3p	Traes_6AS_8D2111A83.6						AT1G21150.1	Mitochondrial transcription termination factor family protein	LOC_Os06g12100.1	mTERF family protein, expressed	2,50	-1.0	1	21	245	265	UGAACAUCCCAGAGCCACCGG	CUCGUGGCUCGGGGAUGUUCA	Translation
tae-miR9662a-3p	Traes_6AS_8D2111A83.7	PF02536				GO:GO:0006355,GO:GO:0005739,GO:GO:0003690	AT1G21150.1	Mitochondrial transcription termination factor family protein	LOC_Os06g12100.1	mTERF family protein, expressed	2,50	-1.0	1	21	33	53	UUGAACAUCCCAGAGCCACCG	UCGUGGCUCGGGGAUGUUCAG	Cleavage
tae-miR9662b-3p	Traes_6AS_8D2111A83.7	PF02536				GO:GO:0006355,GO:GO:0005739,GO:GO:0003690	AT1G21150.1	Mitochondrial transcription termination factor family protein	LOC_Os06g12100.1	mTERF family protein, expressed	2,50	-1.0	1	21	32	52	UGAACAUCCCAGAGCCACCGG	CUCGUGGCUCGGGGAUGUUCA	Translation
tae-miR1122b-3p	Traes_6AS_8E6E52967.2	PF08263					AT1G17250.1	receptor like protein 3	LOC_Os02g06120.1	Leucine Rich Repeat family protein, expressed	1,50	-1.0	1	21	120	140	AGACUUAUAUGUAGGAACGGA	UCCGUUCCUAAAUAUAAGUCU	Translation
tae-miR156	Traes_6AS_99C6A3B0B.1	PF03110				GO:GO:0005634,GO:GO:0003677	AT5G43270.1	squamosa promoter binding protein-like 2	LOC_Os02g04680.2	OsSPL3 - SBP-box gene family member, expressed	1,00	-1.0	1	21	1477	1497	UGACAGAAGAGAGUGAGCACA	CAUGCUCUCUCUCUUCUGUCA	Cleavage
tae-miR156	Traes_6AS_99C6A3B0B.2	PF03110				GO:GO:0005634,GO:GO:0003677	AT5G43270.3	squamosa promoter binding protein-like 2	LOC_Os02g04680.2	OsSPL3 - SBP-box gene family member, expressed	1,00	-1.0	1	21	1477	1497	UGACAGAAGAGAGUGAGCACA	CAUGCUCUCUCUCUUCUGUCA	Cleavage
tae-miR156	Traes_6AS_99C6A3B0B.3	PF03110				GO:GO:0005634,GO:GO:0003677	AT5G43270.2	squamosa promoter binding protein-like 2	LOC_Os02g04680.2	OsSPL3 - SBP-box gene family member, expressed	1,00	-1.0	1	21	1477	1497	UGACAGAAGAGAGUGAGCACA	CAUGCUCUCUCUCUUCUGUCA	Cleavage
tae-miR156	Traes_6AS_99C6A3B0B.4	PF03110				GO:GO:0005634,GO:GO:0003677	AT5G43270.2	squamosa promoter binding protein-like 2	LOC_Os02g04680.2	OsSPL3 - SBP-box gene family member, expressed	1,00	-1.0	1	21	1156	1176	UGACAGAAGAGAGUGAGCACA	CAUGCUCUCUCUCUUCUGUCA	Cleavage
tae-miR156	Traes_6AS_99C6A3B0B.5	PF03110				GO:GO:0005634,GO:GO:0003677	AT5G43270.3	squamosa promoter binding protein-like 2	LOC_Os02g04680.2	OsSPL3 - SBP-box gene family member, expressed	1,00	-1.0	1	21	1156	1176	UGACAGAAGAGAGUGAGCACA	CAUGCUCUCUCUCUUCUGUCA	Cleavage
tae-miR156	Traes_6AS_99C6A3B0B.6	PF03110				GO:GO:0005634,GO:GO:0003677	AT5G43270.1	squamosa promoter binding protein-like 2	LOC_Os02g04680.2	OsSPL3 - SBP-box gene family member, expressed	1,00	-1.0	1	21	1156	1176	UGACAGAAGAGAGUGAGCACA	CAUGCUCUCUCUCUUCUGUCA	Cleavage
tae-miR156	Traes_6AS_99C6A3B0B.7	PF03110				GO:GO:0005634,GO:GO:0003677	AT5G43270.3	squamosa promoter binding protein-like 2	LOC_Os02g04680.2	OsSPL3 - SBP-box gene family member, expressed	1,00	-1.0	1	21	1156	1176	UGACAGAAGAGAGUGAGCACA	CAUGCUCUCUCUCUUCUGUCA	Cleavage
tae-miR156	Traes_6AS_99C6A3B0B.8	PF03110				GO:GO:0005634,GO:GO:0003677	AT5G43270.1	squamosa promoter binding protein-like 2	LOC_Os02g04680.2	OsSPL3 - SBP-box gene family member, expressed	1,00	-1.0	1	21	1156	1176	UGACAGAAGAGAGUGAGCACA	CAUGCUCUCUCUCUUCUGUCA	Cleavage
tae-miR9662a-3p	Traes_6AS_A2EAE01C1.1	PF02536				GO:GO:0006355,GO:GO:0005739,GO:GO:0003690	AT5G07900.1	Mitochondrial transcription termination factor family protein	LOC_Os06g12100.1	mTERF family protein, expressed	2,50	-1.0	1	21	465	485	UUGAACAUCCCAGAGCCACCG	CCGUGGCUCUGGAAUGUUCAG	Cleavage
tae-miR9662b-3p	Traes_6AS_A2EAE01C1.1	PF02536				GO:GO:0006355,GO:GO:0005739,GO:GO:0003690	AT5G07900.1	Mitochondrial transcription termination factor family protein	LOC_Os06g12100.1	mTERF family protein, expressed	2,50	-1.0	1	21	464	484	UGAACAUCCCAGAGCCACCGG	CCCGUGGCUCUGGAAUGUUCA	Cleavage
tae-miR9662a-3p	Traes_6AS_A2EAE01C1.2	PF02536				GO:GO:0006355,GO:GO:0005739,GO:GO:0003690	AT5G07900.1	Mitochondrial transcription termination factor family protein	LOC_Os06g12100.1	mTERF family protein, expressed	2,50	-1.0	1	21	66	86	UUGAACAUCCCAGAGCCACCG	CCGUGGCUCUGGAAUGUUCAG	Cleavage
tae-miR9662b-3p	Traes_6AS_A2EAE01C1.2	PF02536				GO:GO:0006355,GO:GO:0005739,GO:GO:0003690	AT5G07900.1	Mitochondrial transcription termination factor family protein	LOC_Os06g12100.1	mTERF family protein, expressed	2,50	-1.0	1	21	65	85	UGAACAUCCCAGAGCCACCGG	CCCGUGGCUCUGGAAUGUUCA	Cleavage
tae-miR9662a-3p	Traes_6AS_B24138E62.1	PF02536				GO:GO:0006355,GO:GO:0005739,GO:GO:0003690	AT5G07900.1	Mitochondrial transcription termination factor family protein	LOC_Os06g12100.1	mTERF family protein, expressed	2,50	-1.0	1	21	375	395	UUGAACAUCCCAGAGCCACCG	CCGUGGCUCUGGAAUGUUCAG	Cleavage
tae-miR9662b-3p	Traes_6AS_B24138E62.1	PF02536				GO:GO:0006355,GO:GO:0005739,GO:GO:0003690	AT5G07900.1	Mitochondrial transcription termination factor family protein	LOC_Os06g12100.1	mTERF family protein, expressed	2,50	-1.0	1	21	374	394	UGAACAUCCCAGAGCCACCGG	CCCGUGGCUCUGGAAUGUUCA	Cleavage
tae-miR9662a-3p	Traes_6AS_EBADF37C7.1	PF02536				GO:GO:0006355,GO:GO:0005739,GO:GO:0003690	AT5G07900.1	Mitochondrial transcription termination factor family protein	LOC_Os06g12100.1	mTERF family protein, expressed	2,50	-1.0	1	21	681	701	UUGAACAUCCCAGAGCCACCG	CCGUGGCUCUGGGAUGCUCAG	Cleavage
tae-miR9662b-3p	Traes_6AS_EBADF37C7.1	PF02536				GO:GO:0006355,GO:GO:0005739,GO:GO:0003690	AT5G07900.1	Mitochondrial transcription termination factor family protein	LOC_Os06g12100.1	mTERF family protein, expressed	2,50	-1.0	1	21	680	700	UGAACAUCCCAGAGCCACCGG	CCCGUGGCUCUGGGAUGCUCA	Cleavage
tae-miR9662a-3p	Traes_6AS_EBADF37C7.2	PF02536				GO:GO:0006355,GO:GO:0005739,GO:GO:0003690	AT5G07900.1	Mitochondrial transcription termination factor family protein	LOC_Os06g12100.1	mTERF family protein, expressed	2,50	-1.0	1	21	378	398	UUGAACAUCCCAGAGCCACCG	CCGUGGCUCUGGGAUGCUCAG	Cleavage
tae-miR9662b-3p	Traes_6AS_EBADF37C7.2	PF02536				GO:GO:0006355,GO:GO:0005739,GO:GO:0003690	AT5G07900.1	Mitochondrial transcription termination factor family protein	LOC_Os06g12100.1	mTERF family protein, expressed	2,50	-1.0	1	21	377	397	UGAACAUCCCAGAGCCACCGG	CCCGUGGCUCUGGGAUGCUCA	Cleavage
tae-miR9662a-3p	Traes_6AS_EE73C76541.2	PF02536				GO:GO:0006355,GO:GO:0005739,GO:GO:0003690	AT5G07900.1	Mitochondrial transcription termination factor family protein	LOC_Os06g12100.1	mTERF family protein, expressed	1,00	-1.0	1	21	420	440	UUGAACAUCCCAGAGCCACCG	CCGUGGCUCUGGGAUGUUCAG	Cleavage
tae-miR9662b-3p	Traes_6AS_EE73C76541.2	PF02536				GO:GO:0006355,GO:GO:0005739,GO:GO:0003690	AT5G07900.1	Mitochondrial transcription termination factor family protein	LOC_Os06g12100.1	mTERF family protein, expressed	1,00	-1.0	1	21	419	439	UGAACAUCCCAGAGCCACCGG	CCCGUGGCUCUGGGAUGUUCA	Cleavage
tae-miR9662a-3p	Traes_6AS_EE73C76541.6	PF02536				GO:GO:0006355,GO:GO:0005739,GO:GO:0003690	AT5G07900.1	Mitochondrial transcription termination factor family protein	LOC_Os06g12100.1	mTERF family protein, expressed	1,00	-1.0	1	21	66	86	UUGAACAUCCCAGAGCCACCG	CCGUGGCUCUGGGAUGUUCAG	Cleavage
tae-miR9662b-3p	Traes_6AS_EE73C76541.6	PF02536				GO:GO:0006355,GO:GO:0005739,GO:GO:0003690	AT5G07900.1	Mitochondrial transcription termination factor family protein	LOC_Os06g12100.1	mTERF family protein, expressed	1,00	-1.0	1	21	65	85	UGAACAUCCCAGAGCCACCGG	CCCGUGGCUCUGGGAUGUUCA	Cleavage
tae-miR9662a-3p	Traes_6AS_EE8BA0A57.1	PF02536				GO:GO:0006355,GO:GO:0005739,GO:GO:0003690	AT1G21150.1	Mitochondrial transcription termination factor family protein	LOC_Os06g12100.1	mTERF family protein, expressed	2,00	-1.0	1	21	462	482	UUGAACAUCCCAGAGCCACCG	CCGUGCCUCUGGGAUGUUCAG	Cleavage
tae-miR9662b-3p	Traes_6AS_EE8BA0A57.1	PF02536				GO:GO:0006355,GO:GO:0005739,GO:GO:0003690	AT1G21150.1	Mitochondrial transcription termination factor family protein	LOC_Os06g12100.1	mTERF family protein, expressed	2,00	-1.0	1	21	461	481	UGAACAUCCCAGAGCCACCGG	CCCGUGCCUCUGGGAUGUUCA	Cleavage
tae-miR9662a-3p	Traes_6AS_EE8BA0A57.2	PF02536				GO:GO:0006355,GO:GO:0005739,GO:GO:0003690	AT5G07900.1	Mitochondrial transcription termination factor family protein	LOC_Os06g12100.1	mTERF family protein, expressed	2,00	-1.0	1	21	510	530	UUGAACAUCCCAGAGCCACCG	CCGUGCCUCUGGGAUGUUCAG	Cleavage
tae-miR9662b-3p	Traes_6AS_EE8BA0A57.2	PF02536				GO:GO:0006355,GO:GO:0005739,GO:GO:0003690	AT5G07900.1	Mitochondrial transcription termination factor family protein	LOC_Os06g12100.1	mTERF family protein, expressed	2,00	-1.0	1	21	509	529	UGAACAUCCCAGAGCCACCGG	CCCGUGCCUCUGGGAUGUUCA	Cleavage
tae-miR5049-3p	Traes_6AS_F5DBB6DBD.3	PF13855	KOG0472			GO:GO:0005515	AT1G72300.1	Leucine-rich receptor-like protein kinase family protein	LOC_Os02g05970.1	phytosulfokine receptor precursor, putative, expressed	1,50	-1.0	1	21	1927	1947	AAUAUGGAUCGGAGGGAGUAC	UUACUCCCUCCAAUCCAUAUU	Translation
tae-miR9662a-3p	Traes_6AS_FBBCFBCB0.1	PF02536	KOG1267			GO:GO:0006355,GO:GO:0005739,GO:GO:0003690	AT5G07900.1	Mitochondrial transcription termination factor family protein	LOC_Os06g12100.1	mTERF family protein, expressed	2,50	-1.0	1	21	681	701	UUGAACAUCCCAGAGCCACCG	CCGUGGCUCUGGGAUGUUUAG	Cleavage
tae-miR9662b-3p	Traes_6AS_FBBCFBCB0.1	PF02536	KOG1267			GO:GO:0006355,GO:GO:0005739,GO:GO:0003690	AT5G07900.1	Mitochondrial transcription termination factor family protein	LOC_Os06g12100.1	mTERF family protein, expressed	2,50	-1.0	1	21	680	700	UGAACAUCCCAGAGCCACCGG	CCCGUGGCUCUGGGAUGUUUA	Cleavage
tae-miR1127b-3p	Traes_6BL_028068EFA.2		KOG1877				AT5G21080.1	Uncharacterized protein	LOC_Os02g53990.1	cyclin-related protein, putative, expressed	1,50	-1.0	1	21	2801	2821	ACAAGUAUUUCUGGACGGAGG	CCUCCGUCCGGAAAUACUUGU	Cleavage
tae-miR6197-5p	Traes_6BL_67725F06B.2	PF02358		3.1.3.12		GO:GO:0005992,GO:GO:0003824	AT5G65140.2	Haloacid dehalogenase-like hydrolase (HAD) superfamily protein	LOC_Os02g51680.1	uncharacterized glycosyl hydrolase Rv2006/MT2062, putative, expressed	3,00	-1.0	1	21	278	298	UCUGUAAACAAAUGUAGGACG	GAACCUCCAUUUGUUUACAGG	Cleavage
tae-miR1127b-3p	Traes_6BL_731ED1C4C.1	PF00646				GO:GO:0005515	AT5G56820.1	F-box/RNI-like/FBD-like domains-containing protein	LOC_Os02g57910.1	OsFBX73 - F-box domain containing protein, expressed	1,50	-1.0	1	21	1020	1040	ACAAGUAUUUCUGGACGGAGG	UCUCCGUCCAAAAAUACUUGU	Translation
tae-miR1130b-3p	Traes_6BL_7BD68BAC2.3	PF00366				GO:GO:0006412,GO:GO:0005840,GO:GO:0005622,GO:GO:0003735	AT5G23740.1	ribosomal protein S11-beta	LOC_Os04g52361.1	ribosomal protein S17, putative, expressed	1,50	-1.0	1	21	215	235	UCUUAUAUUAUGGGACGGAGG	CCUCCGUCCCAUAAUGUAAGA	Cleavage
tae-miR1122a	Traes_6BL_7E9C131F4.3	PF00249	KOG0724				AT5G52660.1	Homeodomain-like superfamily protein	LOC_Os02g45670.1	myb-like DNA-binding domain containing protein, expressed	0,00	-1.0	1	20	761	780	UAGAUACAUCCGUAUCUAGA	UCUAGAUACGGAUGUAUCUA	Cleavage
tae-miR1130b-3p	Traes_6BL_887005D55.2										1,50	-1.0	1	21	79	99	UCUUAUAUUAUGGGACGGAGG	CCUCCGUCCCAUAAUAUAAUA	Cleavage
tae-miR1118	Traes_6BL_E14604962.2	PF00076				GO:GO:0003676	AT5G51120.2	polyadenylate-binding protein 1	LOC_Os02g52140.2	RNA recognition motif containing protein, putative, expressed	2,00	-1.0	1	23	35	57	CACUACAUUAUGGAAUGGAGGGA	UCCCUCUGUUCCAUAAUGUAGUG	Cleavage
tae-miR1118	Traes_6BL_E14604962.3	PF00076				GO:GO:0003676	AT5G51120.2	polyadenylate-binding protein 1	LOC_Os02g52140.2	RNA recognition motif containing protein, putative, expressed	2,00	-1.0	1	23	35	57	CACUACAUUAUGGAAUGGAGGGA	UCCCUCUGUUCCAUAAUGUAGUG	Cleavage
tae-miR1127b-3p	Traes_6BS_1AFDF35E5.3										2,50	-1.0	1	21	171	191	ACAAGUAUUUCUGGACGGAGG	CUUUCGUCCGGAAAUACUUGU	Cleavage
tae-miR9662a-3p	Traes_6BS_28F0D2955.1	PF02536				GO:GO:0006355,GO:GO:0005739,GO:GO:0003690	AT5G64950.1	Mitochondrial transcription termination factor family protein	LOC_Os06g12100.1	mTERF family protein, expressed	2,50	-1.0	1	21	396	416	UUGAACAUCCCAGAGCCACCG	CUGUGGCUCUCGGAUGUUCAG	Translation
tae-miR9662b-3p	Traes_6BS_28F0D2955.1	PF02536				GO:GO:0006355,GO:GO:0005739,GO:GO:0003690	AT5G64950.1	Mitochondrial transcription termination factor family protein	LOC_Os06g12100.1	mTERF family protein, expressed	2,50	-1.0	1	21	395	415	UGAACAUCCCAGAGCCACCGG	CCUGUGGCUCUCGGAUGUUCA	Translation
tae-miR9662a-3p	Traes_6BS_2AC6A2287.1	PF02536				GO:GO:0006355,GO:GO:0005739,GO:GO:0003690	AT5G07900.1	Mitochondrial transcription termination factor family protein	LOC_Os06g12100.1	mTERF family protein, expressed	2,50	-1.0	1	21	66	86	UUGAACAUCCCAGAGCCACCG	CCGUGGCUCUGGGAUGCUCAG	Cleavage
tae-miR9662b-3p	Traes_6BS_2AC6A2287.1	PF02536				GO:GO:0006355,GO:GO:0005739,GO:GO:0003690	AT5G07900.1	Mitochondrial transcription termination factor family protein	LOC_Os06g12100.1	mTERF family protein, expressed	2,50	-1.0	1	21	65	85	UGAACAUCCCAGAGCCACCGG	CCCGUGGCUCUGGGAUGCUCA	Cleavage
tae-miR9662a-3p	Traes_6BS_2F108C5BB.1	PF02536				GO:GO:0006355,GO:GO:0005739,GO:GO:0003690	AT5G07900.1	Mitochondrial transcription termination factor family protein	LOC_Os06g12100.1	mTERF family protein, expressed	2,00	-1.0	1	21	435	455	UUGAACAUCCCAGAGCCACCG	UCGUGGCGCUGGGAUGUUCAG	Cleavage
tae-miR9662b-3p	Traes_6BS_2F108C5BB.1	PF02536				GO:GO:0006355,GO:GO:0005739,GO:GO:0003690	AT5G07900.1	Mitochondrial transcription termination factor family protein	LOC_Os06g12100.1	mTERF family protein, expressed	2,50	-1.0	1	21	434	454	UGAACAUCCCAGAGCCACCGG	CUCGUGGCGCUGGGAUGUUCA	Cleavage
tae-miR408	Traes_6BS_348308070.13										2,50	-1.0	1	21	2	22	CUGCACUGCCUCUUCCCUGGC	CCCAGGGCCGAGGCAGUGCAG	Cleavage
tae-miR408	Traes_6BS_348308070.15										2,50	-1.0	1	21	11	31	CUGCACUGCCUCUUCCCUGGC	CCCAGGGCCGAGGCAGUGCAG	Cleavage

tae-miR408	Traes_6BS_348308070.17										2,50	-1.0	1	21	14	34	CUGCACUGCCUCUUCCCUGGC	CCCAGGGCCGAGGCAGUGCAG	Cleavage
tae-miR408	Traes_6BS_348308070.19										2,50	-1.0	1	21	11	31	CUGCACUGCCUCUUCCCUGGC	CCCAGGGCCGAGGCAGUGCAG	Cleavage
tae-miR408	Traes_6BS_348308070.2										2,50	-1.0	1	21	14	34	CUGCACUGCCUCUUCCCUGGC	CCCAGGGCCGAGGCAGUGCAG	Cleavage
tae-miR408	Traes_6BS_348308070.3										2,50	-1.0	1	21	17	37	CUGCACUGCCUCUUCCCUGGC	CCCAGGGCCGAGGCAGUGCAG	Cleavage
tae-miR408	Traes_6BS_348308070.5										2,50	-1.0	1	21	17	37	CUGCACUGCCUCUUCCCUGGC	CCCAGGGCCGAGGCAGUGCAG	Cleavage
tae-miR408	Traes_6BS_348308070.6										2,50	-1.0	1	21	14	34	CUGCACUGCCUCUUCCCUGGC	CCCAGGGCCGAGGCAGUGCAG	Cleavage
tae-miR408	Traes_6BS_348308070.8										2,50	-1.0	1	21	17	37	CUGCACUGCCUCUUCCCUGGC	CCCAGGGCCGAGGCAGUGCAG	Cleavage
tae-miR408	Traes_6BS_348308070.9										2,50	-1.0	1	21	17	37	CUGCACUGCCUCUUCCCUGGC	CCCAGGGCCGAGGCAGUGCAG	Cleavage
tae-miR9662a-3p	Traes_6BS_3A00751B5.1	PF02536				GO:GO:0006355,GO:GO:0005739,GO:GO:0003690	AT5G07900.1	Mitochondrial transcription termination factor family protein	LOC_Os06g12100.1	mTERF family protein, expressed	2,50	-1.0	1	21	69	89	UUGAACAUCCCAGAGCCACCG	CCGUGGCUCUGGGAUGUUUAG	Cleavage
tae-miR9662b-3p	Traes_6BS_3A00751B5.1	PF02536				GO:GO:0006355,GO:GO:0005739,GO:GO:0003690	AT5G07900.1	Mitochondrial transcription termination factor family protein	LOC_Os06g12100.1	mTERF family protein, expressed	2,50	-1.0	1	21	68	88	UGAACAUCCCAGAGCCACCGG	CCCGUGGCUCUGGGAUGUUUA	Cleavage
tae-miR9662a-3p	Traes_6BS_3A00751B5.2	PF02536				GO:GO:0006355,GO:GO:0005739,GO:GO:0003690	AT5G07900.1	Mitochondrial transcription termination factor family protein	LOC_Os06g12100.1	mTERF family protein, expressed	2,50	-1.0	1	21	69	89	UUGAACAUCCCAGAGCCACCG	CCGUGGCUCUGGGAUGUUUAG	Cleavage
tae-miR9662b-3p	Traes_6BS_3A00751B5.2	PF02536				GO:GO:0006355,GO:GO:0005739,GO:GO:0003690	AT5G07900.1	Mitochondrial transcription termination factor family protein	LOC_Os06g12100.1	mTERF family protein, expressed	2,50	-1.0	1	21	68	88	UGAACAUCCCAGAGCCACCGG	CCCGUGGCUCUGGGAUGUUUA	Cleavage
tae-miR9662a-3p	Traes_6BS_46641470F.1	PF02536				GO:GO:0006355,GO:GO:0005739,GO:GO:0003690	AT1G21150.1	Mitochondrial transcription termination factor family protein	LOC_Os06g12100.1	mTERF family protein, expressed	2,50	-1.0	1	21	33	53	UUGAACAUCCCAGAGCCACCG	CCGUGGCUCUGGGAUGUUUAG	Cleavage
tae-miR9662b-3p	Traes_6BS_46641470F.1	PF02536				GO:GO:0006355,GO:GO:0005739,GO:GO:0003690	AT1G21150.1	Mitochondrial transcription termination factor family protein	LOC_Os06g12100.1	mTERF family protein, expressed	2,50	-1.0	1	21	32	52	UGAACAUCCCAGAGCCACCGG	CCCGUGGCUCUGGGAUGUUUA	Cleavage
tae-miR9662a-3p	Traes_6BS_4E6E57FEB.1	PF02536				GO:GO:0006355,GO:GO:0005739,GO:GO:0003690	AT5G07900.1	Mitochondrial transcription termination factor family protein	LOC_Os06g12100.1	mTERF family protein, expressed	3,00	-1.0	1	21	65	86	UUGAACAUCCCAGAGCCAC-CG	CGCGUGGCUCUGGGAUGUUCAG	Cleavage
tae-miR9662b-3p	Traes_6BS_4E6E57FEB.1	PF02536				GO:GO:0006355,GO:GO:0005739,GO:GO:0003690	AT5G07900.1	Mitochondrial transcription termination factor family protein	LOC_Os06g12100.1	mTERF family protein, expressed	2,00	-1.0	1	21	64	85	UGAACAUCCCAGAGCCAC-CGG	CCGCGUGGCUCUGGGAUGUUCA	Cleavage
tae-miR9662a-3p	Traes_6BS_4E6E57FEB.2	PF02536				GO:GO:0006355,GO:GO:0005739,GO:GO:0003690	AT5G07900.1	Mitochondrial transcription termination factor family protein	LOC_Os06g12100.1	mTERF family protein, expressed	3,00	-1.0	1	21	65	86	UUGAACAUCCCAGAGCCAC-CG	CGCGUGGCUCUGGGAUGUUCAG	Cleavage
tae-miR9662b-3p	Traes_6BS_4E6E57FEB.2	PF02536				GO:GO:0006355,GO:GO:0005739,GO:GO:0003690	AT5G07900.1	Mitochondrial transcription termination factor family protein	LOC_Os06g12100.1	mTERF family protein, expressed	2,00	-1.0	1	21	64	85	UGAACAUCCCAGAGCCAC-CGG	CCGCGUGGCUCUGGGAUGUUCA	Cleavage
tae-miR9662a-3p	Traes_6BS_4E6E57FEB.3	PF02536				GO:GO:0006355,GO:GO:0005739,GO:GO:0003690	AT5G07900.1	Mitochondrial transcription termination factor family protein	LOC_Os06g12100.1	mTERF family protein, expressed	3,00	-1.0	1	21	65	86	UUGAACAUCCCAGAGCCAC-CG	CGCGUGGCUCUGGGAUGUUCAG	Cleavage
tae-miR9662b-3p	Traes_6BS_4E6E57FEB.3	PF02536				GO:GO:0006355,GO:GO:0005739,GO:GO:0003690	AT5G07900.1	Mitochondrial transcription termination factor family protein	LOC_Os06g12100.1	mTERF family protein, expressed	2,00	-1.0	1	21	64	85	UGAACAUCCCAGAGCCAC-CGG	CCGCGUGGCUCUGGGAUGUUCA	Cleavage
tae-miR9662b-3p	Traes_6BS_508C2BEA2.1	PF02536				GO:GO:0006355,GO:GO:0005739,GO:GO:0003690	AT1G21150.1	Mitochondrial transcription termination factor family protein	LOC_Os06g12100.1	mTERF family protein, expressed	3,00	-1.0	1	21	32	52	UGAACAUCCCAGAGCCACCGG	CCUGUGGUUCUGGGAUGUUCG	Cleavage
tae-miR156	Traes_6BS_542961EA4.1								LOC_Os06g45310.1	OsSPL11 - SBP-box gene family member, expressed	1,00	-1.0	1	21	283	303	UGACAGAAGAGAGUGAGCACA	CGUGCUCUCUCUCUUCUGUCA	Cleavage
tae-miR156	Traes_6BS_542961EA4.2								LOC_Os06g45310.1	OsSPL11 - SBP-box gene family member, expressed	1,00	-1.0	1	21	349	369	UGACAGAAGAGAGUGAGCACA	CGUGCUCUCUCUCUUCUGUCA	Cleavage
tae-miR1122b-3p	Traes_6BS_7965FEB75.1	PF00300	KOG0235	3.1.3.3	K15634		AT3G50520.1	Phosphoglycerate mutase family protein	LOC_Os08g37140.1	phosphoglycerate mutase, putative, expressed	2,50	-1.0	1	21	87	107	AGACUUAUAUGUAGGAACGGA	UCCGUUCGUAUAUAUAAGUCU	Translation
tae-miR9662a-3p	Traes_6BS_7F1846171.1	PF02536				GO:GO:0006355,GO:GO:0005739,GO:GO:0003690	AT5G07900.1	Mitochondrial transcription termination factor family protein	LOC_Os06g12100.1	mTERF family protein, expressed	2,50	-1.0	1	21	33	53	UUGAACAUCCCAGAGCCACCG	CUGUGGCUCUCGGAUGUUCAG	Translation
tae-miR9662b-3p	Traes_6BS_7F1846171.1	PF02536				GO:GO:0006355,GO:GO:0005739,GO:GO:0003690	AT5G07900.1	Mitochondrial transcription termination factor family protein	LOC_Os06g12100.1	mTERF family protein, expressed	2,50	-1.0	1	21	32	52	UGAACAUCCCAGAGCCACCGG	CCUGUGGCUCUCGGAUGUUCA	Translation
tae-miR9662a-3p	Traes_6BS_82F5BF11F.1	PF02536				GO:GO:0006355,GO:GO:0005739,GO:GO:0003690	AT1G79220.1	Mitochondrial transcription termination factor family protein	LOC_Os06g12100.1	mTERF family protein, expressed	2,00	-1.0	1	21	267	287	UUGAACAUCCCAGAGCCACCG	CCGUGCCUCUGGGAUGUUCAG	Cleavage
tae-miR9662b-3p	Traes_6BS_82F5BF11F.1	PF02536				GO:GO:0006355,GO:GO:0005739,GO:GO:0003690	AT1G79220.1	Mitochondrial transcription termination factor family protein	LOC_Os06g12100.1	mTERF family protein, expressed	2,00	-1.0	1	21	266	286	UGAACAUCCCAGAGCCACCGG	CCCGUGCCUCUGGGAUGUUCA	Cleavage
tae-miR9662a-3p	Traes_6BS_843505CB3.1	PF02536				GO:GO:0006355,GO:GO:0005739,GO:GO:0003690	AT1G21150.1	Mitochondrial transcription termination factor family protein	LOC_Os06g12100.1	mTERF family protein, expressed	2,50	-1.0	1	21	69	89	UUGAACAUCCCAGAGCCACCG	CCGUGGCUCUGGAAUGUUCAG	Cleavage
tae-miR9662b-3p	Traes_6BS_843505CB3.1	PF02536				GO:GO:0006355,GO:GO:0005739,GO:GO:0003690	AT1G21150.1	Mitochondrial transcription termination factor family protein	LOC_Os06g12100.1	mTERF family protein, expressed	2,50	-1.0	1	21	68	88	UGAACAUCCCAGAGCCACCGG	CCCGUGGCUCUGGAAUGUUCA	Cleavage
tae-miR9662a-3p	Traes_6BS_843505CB3.2	PF02536				GO:GO:0006355,GO:GO:0005739,GO:GO:0003690	AT1G21150.1	Mitochondrial transcription termination factor family protein	LOC_Os06g12100.1	mTERF family protein, expressed	2,50	-1.0	1	21	69	89	UUGAACAUCCCAGAGCCACCG	CCGUGGCUCUGGAAUGUUCAG	Cleavage
tae-miR9662b-3p	Traes_6BS_843505CB3.2	PF02536				GO:GO:0006355,GO:GO:0005739,GO:GO:0003690	AT1G21150.1	Mitochondrial transcription termination factor family protein	LOC_Os06g12100.1	mTERF family protein, expressed	2,50	-1.0	1	21	68	88	UGAACAUCCCAGAGCCACCGG	CCCGUGGCUCUGGAAUGUUCA	Cleavage
tae-miR9662a-3p	Traes_6BS_843505CB3.3	PF02536				GO:GO:0006355,GO:GO:0005739,GO:GO:0003690	AT1G21150.1	Mitochondrial transcription termination factor family protein	LOC_Os06g12100.1	mTERF family protein, expressed	2,50	-1.0	1	21	69	89	UUGAACAUCCCAGAGCCACCG	CCGUGGCUCUGGAAUGUUCAG	Cleavage
tae-miR9662b-3p	Traes_6BS_843505CB3.3	PF02536				GO:GO:0006355,GO:GO:0005739,GO:GO:0003690	AT1G21150.1	Mitochondrial transcription termination factor family protein	LOC_Os06g12100.1	mTERF family protein, expressed	2,50	-1.0	1	21	68	88	UGAACAUCCCAGAGCCACCGG	CCCGUGGCUCUGGAAUGUUCA	Cleavage
tae-miR9662a-3p	Traes_6BS_843505CB3.4	PF02536				GO:GO:0006355,GO:GO:0005739,GO:GO:0003690	AT1G21150.1	Mitochondrial transcription termination factor family protein	LOC_Os06g12100.1	mTERF family protein, expressed	2,50	-1.0	1	21	69	89	UUGAACAUCCCAGAGCCACCG	CCGUGGCUCUGGAAUGUUCAG	Cleavage
tae-miR9662b-3p	Traes_6BS_843505CB3.4	PF02536				GO:GO:0006355,GO:GO:0005739,GO:GO:0003690	AT1G21150.1	Mitochondrial transcription termination factor family protein	LOC_Os06g12100.1	mTERF family protein, expressed	2,50	-1.0	1	21	68	88	UGAACAUCCCAGAGCCACCGG	CCCGUGGCUCUGGAAUGUUCA	Cleavage
tae-miR9662a-3p	Traes_6BS_843505CB3.5	PF02536				GO:GO:0006355,GO:GO:0005739,GO:GO:0003690	AT1G21150.1	Mitochondrial transcription termination factor family protein	LOC_Os06g12100.1	mTERF family protein, expressed	2,50	-1.0	1	21	69	89	UUGAACAUCCCAGAGCCACCG	CCGUGGCUCUGGAAUGUUCAG	Cleavage
tae-miR9662b-3p	Traes_6BS_843505CB3.5	PF02536				GO:GO:0006355,GO:GO:0005739,GO:GO:0003690	AT1G21150.1	Mitochondrial transcription termination factor family protein	LOC_Os06g12100.1	mTERF family protein, expressed	2,50	-1.0	1	21	68	88	UGAACAUCCCAGAGCCACCGG	CCCGUGGCUCUGGAAUGUUCA	Cleavage
tae-miR9662a-3p	Traes_6BS_843505CB3.6	PF02536				GO:GO:0006355,GO:GO:0005739,GO:GO:0003690	AT1G21150.1	Mitochondrial transcription termination factor family protein	LOC_Os06g12100.1	mTERF family protein, expressed	2,50	-1.0	1	21	69	89	UUGAACAUCCCAGAGCCACCG	CCGUGGCUCUGGAAUGUUCAG	Cleavage
tae-miR9662b-3p	Traes_6BS_843505CB3.6	PF02536				GO:GO:0006355,GO:GO:0005739,GO:GO:0003690	AT1G21150.1	Mitochondrial transcription termination factor family protein	LOC_Os06g12100.1	mTERF family protein, expressed	2,50	-1.0	1	21	68	88	UGAACAUCCCAGAGCCACCGG	CCCGUGGCUCUGGAAUGUUCA	Cleavage
tae-miR9662a-3p	Traes_6BS_843505CB3.7	PF02536				GO:GO:0006355,GO:GO:0005739,GO:GO:0003690	AT1G21150.1	Mitochondrial transcription termination factor family protein	LOC_Os06g12100.1	mTERF family protein, expressed	2,50	-1.0	1	21	69	89	UUGAACAUCCCAGAGCCACCG	CCGUGGCUCUGGAAUGUUCAG	Cleavage
tae-miR9662b-3p	Traes_6BS_843505CB3.7	PF02536				GO:GO:0006355,GO:GO:0005739,GO:GO:0003690	AT1G21150.1	Mitochondrial transcription termination factor family protein	LOC_Os06g12100.1	mTERF family protein, expressed	2,50	-1.0	1	21	68	88	UGAACAUCCCAGAGCCACCGG	CCCGUGGCUCUGGAAUGUUCA	Cleavage
tae-miR1127b-3p	Traes_6BS_94CE86A01.2	PF00069	KOG0984	2.7.11.1		GO:GO:0006468,GO:GO:0005524,GO:GO:0004672	AT4G23270.1	cysteine-rich RLK (RECEPTOR-like protein kinase) 19	LOC_Os10g12620.1	protein kinase domain containing protein, expressed	2,50	-1.0	1	21	763	783	ACAAGUAUUUCUGGACGGAGG	AAUCCGUUCAGAAUUACUUGU	Cleavage
tae-miR1127a	Traes_6BS_94CE86A01.2	PF00069	KOG0984	2.7.11.1		GO:GO:0006468,GO:GO:0005524,GO:GO:0004672	AT4G23270.1	cysteine-rich RLK (RECEPTOR-like protein kinase) 19	LOC_Os10g12620.1	protein kinase domain containing protein, expressed	1,50	-1.0	1	19	842	860	UCCUUCCGUUCGGAAUUAC	GUAAUUCCGAACGGAGGGA	Cleavage
tae-miR9662a-3p	Traes_6BS_97770F04E.1	PF02536				GO:GO:0006355,GO:GO:0005739,GO:GO:0003690	AT5G07900.1	Mitochondrial transcription termination factor family protein	LOC_Os06g12100.1	mTERF family protein, expressed	2,50	-1.0	1	21	33	53	UUGAACAUCCCAGAGCCACCG	UCGUGGCUCUGGAAUGUUCAG	Cleavage
tae-miR9662b-3p	Traes_6BS_97770F04E.1	PF02536				GO:GO:0006355,GO:GO:0005739,GO:GO:0003690	AT5G07900.1	Mitochondrial transcription termination factor family protein	LOC_Os06g12100.1	mTERF family protein, expressed	2,50	-1.0	1	21	32	52	UGAACAUCCCAGAGCCACCGG	CUCGUGGCUCUGGAAUGUUCA	Cleavage
tae-miR9662a-3p	Traes_6BS_97770F04E.3	PF02536				GO:GO:0006355,GO:GO:0005739,GO:GO:0003690	AT5G07900.1	Mitochondrial transcription termination factor family protein	LOC_Os06g12100.1	mTERF family protein, expressed	2,50	-1.0	1	21	33	53	UUGAACAUCCCAGAGCCACCG	UCGUGGCUCUGGAAUGUUCAG	Cleavage
tae-miR9662b-3p	Traes_6BS_97770F04E.3	PF02536				GO:GO:0006355,GO:GO:0005739,GO:GO:0003690	AT5G07900.1	Mitochondrial transcription termination factor family protein	LOC_Os06g12100.1	mTERF family protein, expressed	2,50	-1.0	1	21	32	52	UGAACAUCCCAGAGCCACCGG	CUCGUGGCUCUGGAAUGUUCA	Cleavage
tae-miR9662a-3p	Traes_6BS_97770F04E.4	PF02536				GO:GO:0006355,GO:GO:0005739,GO:GO:0003690	AT5G07900.1	Mitochondrial transcription termination factor family protein	LOC_Os06g12100.1	mTERF family protein, expressed	2,50	-1.0	1	21	33	53	UUGAACAUCCCAGAGCCACCG	UCGUGGCUCUGGAAUGUUCAG	Cleavage
tae-miR9662b-3p	Traes_6BS_97770F04E.4	PF02536				GO:GO:0006355,GO:GO:0005739,GO:GO:0003690	AT5G07900.1	Mitochondrial transcription termination factor family protein	LOC_Os06g12100.1	mTERF family protein, expressed	2,50	-1.0	1	21	32	52	UGAACAUCCCAGAGCCACCGG	CUCGUGGCUCUGGAAUGUUCA	Cleavage
tae-miR9662a-3p	Traes_6BS_97770F04E.5	PF02536				GO:GO:0006355,GO:GO:0005739,GO:GO:0003690	AT5G07900.1	Mitochondrial transcription termination factor family protein	LOC_Os06g12100.1	mTERF family protein, expressed	2,50	-1.0	1	21	33	53	UUGAACAUCCCAGAGCCACCG	UCGUGGCUCUGGAAUGUUCAG	Cleavage
tae-miR9662b-3p	Traes_6BS_97770F04E.5	PF02536				GO:GO:0006355,GO:GO:0005739,GO:GO:0003690	AT5G07900.1	Mitochondrial transcription termination factor family protein	LOC_Os06g12100.1	mTERF family protein, expressed	2,50	-1.0	1	21	32	52	UGAACAUCCCAGAGCCACCGG	CUCGUGGCUCUGGAAUGUUCA	Cleavage
tae-miR9662a-3p	Traes_6BS_97770F04E.6	PF02536				GO:GO:0006355,GO:GO:0005739,GO:GO:0003690	AT5G07900.1	Mitochondrial transcription termination factor family protein	LOC_Os06g12100.1	mTERF family protein, expressed	2,50	-1.0	1	21	33	53	UUGAACAUCCCAGAGCCACCG	UCGUGGCUCUGGAAUGUUCAG	Cleavage
tae-miR9662b-3p	Traes_6BS_97770F04E.6	PF02536				GO:GO:0006355,GO:GO:0005739,GO:GO:0003690	AT5G07900.1	Mitochondrial transcription termination factor family protein	LOC_Os06g12100.1	mTERF family protein, expressed	2,50	-1.0	1	21	32	52	UGAACAUCCCAGAGCCACCGG	CUCGUGGCUCUGGAAUGUUCA	Cleavage
tae-miR1137a	Traes_6BS_A4F1E63CE.2	PF05971		2.1.1.181		GO:GO:0008168	AT2G21070.3	methyltransferases	LOC_Os02g02880.1	expressed protein	2,00	-1.0	1	20	884	903	UAGUACAAAGUUGAGUCAUC	AGUGUCUCAACUUUGUACUA	Cleavage
tae-miR1137a	Traes_6BS_A4F1E63CE.3	PF05971		2.1.1.181		GO:GO:0008168	AT2G21070.3	methyltransferases	LOC_Os02g02880.1	expressed protein	2,00	-1.0	1	20	871	890	UAGUACAAAGUUGAGUCAUC	AGUGUCUCAACUUUGUACUA	Cleavage
tae-miR1128	Traes_6BS_BF5C480A9.12										2,50	-1.0	1	21	60	80	UACUACUCCCUCCGUCCGAAA	UUUGGGACGGAGGGAGUAUUA	Cleavage
tae-miR9662a-3p	Traes_6BS_C3247654A.1	PF02536				GO:GO:0006355,GO:GO:0005739,GO:GO:0003690	AT5G07900.1	Mitochondrial transcription termination factor family protein	LOC_Os06g12100.1	mTERF family protein, expressed	2,50	-1.0	1	21	57	77	UUGAACAUCCCAGAGCCACCG	CUGUGGCUCUGCGAUGUUCAG	Translation
tae-miR9662b-3p	Traes_6BS_C3247654A.1	PF02536				GO:GO:0006355,GO:GO:0005739,GO:GO:0003690	AT5G07900.1	Mitochondrial transcription termination factor family protein	LOC_Os06g12100.1	mTERF family protein, expressed	2,50	-1.0	1	21	56	76	UGAACAUCCCAGAGCCACCGG	CCUGUGGCUCUGCGAUGUUCA	Cleavage
tae-miR9662a-3p	Traes_6BS_CBD931158.1	PF02536				GO:GO:0006355,GO:GO:0005739,GO:GO:0003690	AT1G21150.1	Mitochondrial transcription termination factor family protein	LOC_Os06g12100.1	mTERF family protein, expressed	2,00	-1.0	1	21	33	53	UUGAACAUCCCAGAGCCACCG	CCGUGCCUCUGGGAUGUUCAG	Cleavage
tae-miR9662b-3p	Traes_6BS_CBD931158.1	PF02536				GO:GO:0006355,GO:GO:0005739,GO:GO:0003690	AT1G21150.1	Mitochondrial transcription termination factor family protein	LOC_Os06g12100.1	mTERF family protein, expressed	2,00	-1.0	1	21	32	52	UGAACAUCCCAGAGCCACCGG	CCCGUGCCUCUGGGAUGUUCA	Cleavage
tae-miR9662a-3p	Traes_6BS_CBD931158.2	PF02536				GO:GO:0006355,GO:GO:0005739,GO:GO:0003690	AT1G21150.1	Mitochondrial transcription termination factor family protein	LOC_Os06g12100.1	mTERF family protein, expressed	2,00	-1.0	1	21	33	53	UUGAACAUCCCAGAGCCACCG	CCGUGCCUCUGGGAUGUUCAG	Cleavage
tae-miR9662b-3p	Traes_6BS_CBD931158.2	PF02536				GO:GO:0006355,GO:GO:0005739,GO:GO:0003690	AT1G21150.1	Mitochondrial transcription termination factor family protein	LOC_Os06g12100.1	mTERF family protein, expressed	2,00	-1.0	1	21	32	52	UGAACAUCCCAGAGCCACCGG	CCCGUGCCUCUGGGAUGUUCA	Cleavage
tae-miR9662a-3p	Traes_6BS_CBD931158.3	PF02536				GO:GO:0006355,GO:GO:0005739,GO:GO:0003690	AT1G21150.1	Mitochondrial transcription termination factor family protein	LOC_Os06g12100.1	mTERF family protein, expressed	2,00	-1.0	1	21	33	53	UUGAACAUCCCAGAGCCACCG	CCGUGCCUCUGGGAUGUUCAG	Cleavage
tae-miR9662b-3p	Traes_6BS_CBD931158.3	PF02536				GO:GO:0006355,GO:GO:0005739,GO:GO:0003690	AT1G21150.1	Mitochondrial transcription termination factor family protein	LOC_Os06g12100.1	mTERF family protein, expressed	2,00	-1.0	1	21	32	52	UGAACAUCCCAGAGCCACCGG	CCCGUGCCUCUGGGAUGUUCA	Cleavage
tae-miR9662a-3p	Traes_6BS_CBD931158.4	PF02536				GO:GO:0006355,GO:GO:0005739,GO:GO:0003690	AT1G21150.1	Mitochondrial transcription termination factor family protein	LOC_Os06g12100.1	mTERF family protein, expressed	2,00	-1.0	1	21	33	53	UUGAACAUCCCAGAGCCACCG	CCGUGCCUCUGGGAUGUUCAG	Cleavage
tae-miR9662b-3p	Traes_6BS_CBD931158.4	PF02536				GO:GO:0006355,GO:GO:0005739,GO:GO:0003690	AT1G21150.1	Mitochondrial transcription termination factor family protein	LOC_Os06g12100.1	mTERF family protein, expressed	2,00	-1.0	1	21	32	52	UGAACAUCCCAGAGCCACCGG	CCCGUGCCUCUGGGAUGUUCA	Cleavage
tae-miR9662a-3p	Traes_6BS_CBD931158.5	PF02536				GO:GO:0006355,GO:GO:0005739,GO:GO:0003690	AT1G21150.1	Mitochondrial transcription termination factor family protein	LOC_Os06g12100.1	mTERF family protein, expressed	2,00	-1.0	1	21	33	53	UUGAACAUCCCAGAGCCACCG	CCGUGCCUCUGGGAUGUUCAG	Cleavage
tae-miR9662b-3p	Traes_6BS_CBD931158.5	PF02536				GO:GO:0006355,GO:GO:0005739,GO:GO:0003690	AT1G21150.1	Mitochondrial transcription termination factor family protein	LOC_Os06g12100.1	mTERF family protein, expressed	2,00	-1.0	1	21	32	52	UGAACAUCCCAGAGCCACCGG	CCCGUGCCUCUGGGAUGUUCA	Cleavage
tae-miR9662a-3p	Traes_6BS_CBD931158.6	PF02536				GO:GO:0006355,GO:GO:0005739,GO:GO:0003690	AT1G21150.1	Mitochondrial transcription termination factor family protein	LOC_Os06g12100.1	mTERF family protein, expressed	2,00	-1.0	1	21	33	53	UUGAACAUCCCAGAGCCACCG	CCGUGCCUCUGGGAUGUUCAG	Cleavage
tae-miR9662b-3p	Traes_6BS_CBD931158.6	PF02536				GO:GO:0006355,GO:GO:0005739,GO:GO:0003690	AT1G21150.1	Mitochondrial transcription termination factor family protein	LOC_Os06g12100.1	mTERF family protein, expressed	2,00	-1.0	1	21	32	52	UGAACAUCCCAGAGCCACCGG	CCCGUGCCUCUGGGAUGUUCA	Cleavage
tae-miR156	Traes_6BS_F50663DF7.1	PF03110				GO:GO:0005634,GO:GO:0003677	AT5G43270.1	squamosa promoter binding protein-like 2	LOC_Os02g04680.2	OsSPL3 - SBP-box gene family member, expressed	1,00	-1.0	1	21	1387	1407	UGACAGAAGAGAGUGAGCACA	CAUGCUCUCUCUCUUCUGUCA	Cleavage
tae-miR9662a-3p	Traes_6BS_F8176B2AA.1	PF02536				GO:GO:0006355,GO:GO:0005739,GO:GO:0003690	AT5G64950.1	Mitochondrial transcription termination factor family protein	LOC_Os06g12100.1	mTERF family protein, expressed	2,50	-1.0	1	21	204	224	UUGAACAUCCCAGAGCCACCG	CCGUGGCUCUGCGAUGUUCAG	Translation
tae-miR9662b-3p	Traes_6BS_F8176B2AA.1	PF02536				GO:GO:0006355,GO:GO:0005739,GO:GO:0003690	AT5G64950.1	Mitochondrial transcription termination factor family protein	LOC_Os06g12100.1	mTERF family protein, expressed	2,50	-1.0	1	21	203	223	UGAACAUCCCAGAGCCACCGG	CCCGUGGCUCUGCGAUGUUCA	Cleavage
tae-miR9662a-3p	Traes_6BS_F8176B2AA.2	PF02536				GO:GO:0006355,GO:GO:0005739,GO:GO:0003690	AT5G64950.1	Mitochondrial transcription termination factor family protein	LOC_Os06g12100.1	mTERF family protein, expressed	2,50	-1.0	1	21	204	224	UUGAACAUCCCAGAGCCACCG	CCGUGGCUCUGCGAUGUUCAG	Translation
tae-miR9662b-3p	Traes_6BS_F8176B2AA.2	PF02536				GO:GO:0006355,GO:GO:0005739,GO:GO:0003690	AT5G64950.1	Mitochondrial transcription termination factor family protein	LOC_Os06g12100.1	mTERF family protein, expressed	2,50	-1.0	1	21	203	223	UGAACAUCCCAGAGCCACCGG	CCCGUGGCUCUGCGAUGUUCA	Cleavage
tae-miR9662a-3p	Traes_6BS_F8176B2AA.3	PF02536				GO:GO:0006355,GO:GO:0005739,GO:GO:0003690	AT5G64950.1	Mitochondrial transcription termination factor family protein	LOC_Os06g12100.1	mTERF family protein, expressed	2,50	-1.0	1	21	198	218	UUGAACAUCCCAGAGCCACCG	CCGUGGCUCUGCGAUGUUCAG	Translation
tae-miR9662b-3p	Traes_6BS_F8176B2AA.3	PF02536				GO:GO:0006355,GO:GO:0005739,GO:GO:0003690	AT5G64950.1	Mitochondrial transcription termination factor family protein	LOC_Os06g12100.1	mTERF family protein, expressed	2,50	-1.0	1	21	197	217	UGAACAUCCCAGAGCCACCGG	CCCGUGGCUCUGCGAUGUUCA	Cleavage
tae-miR5049-3p	Traes_6DL_1E6A51BD3.10	PF00063		3.6.4.1		GO:GO:0016459,GO:GO:0005524,GO:GO:0003774	AT1G04600.1	myosin XI A	LOC_Os03g64290.1	myosin, putative, expressed	1,50	-1.0	1	21	1321	1341	AAUAUGGAUCGGAGGGAGUAC	AUACUCCCUCCGAUCCAUAAU	Cleavage
tae-miR1122b-3p	Traes_6DL_244C908B5.14	PF12171					AT2G02070.1	indeterminate(ID)-domain 5	LOC_Os04g47860.4	ZOS4-11 - C2H2 zinc finger protein, expressed	1,50	-1.0	1	21	584	604	AGACUUAUAUGUAGGAACGGA	UCCGUUCCUAAAUAUAAGUCU	Translation
tae-miR5384-3p	Traes_6DL_5282E0007.1	PF14541	KOG1339	3.4.23.12		GO:GO:0006508,GO:GO:0004190	AT5G10770.1	Eukaryotic aspartyl protease family protein	LOC_Os02g48870.1	aspartic proteinase nepenthesin-2 precursor, putative, expressed	2,50	-1.0	1	21	1178	1198	UGAGCGCGCCGCCGUCGAAUG	UGUUCGACGGCGGCGCGGUCG	Cleavage
tae-miR5384-3p	Traes_6DL_5282E0007.2	PF14541,PF14543	KOG1339	3.4.23.12		GO:GO:0006508,GO:GO:0004190	AT5G10770.1	Eukaryotic aspartyl protease family protein	LOC_Os02g48870.1	aspartic proteinase nepenthesin-2 precursor, putative, expressed	2,50	-1.0	1	21	1016	1036	UGAGCGCGCCGCCGUCGAAUG	UGUUCGACGGCGGCGCGGUCG	Cleavage
tae-miR1122b-3p	Traes_6DL_84E14AD8D.4	PF00067				GO:GO:0055114,GO:GO:0020037,GO:GO:0016705,GO:GO:0005506	AT1G31800.1	cytochrome P450, family 97, subfamily A, polypeptide 3	LOC_Os02g57290.4	cytochrome P450, putative, expressed	1,50	-1.0	1	21	1030	1050	AGACUUAUAUGUAGGAACGGA	UCCGUUCCUAAAUAUAAGUCU	Translation
tae-miR1122a	Traes_6DL_84E14AD8D.4	PF00067				GO:GO:0055114,GO:GO:0020037,GO:GO:0016705,GO:GO:0005506	AT1G31800.1	cytochrome P450, family 97, subfamily A, polypeptide 3	LOC_Os02g57290.4	cytochrome P450, putative, expressed	2,50	-1.0	1	20	1071	1090	UAGAUACAUCCGUAUCUAGA	ACUACAUACGGAUGUAUAUA	Cleavage
tae-miR1118	Traes_6DL_C9216369C.2	PF00891				GO:GO:0008171	AT5G54160.1	O-methyltransferase 1	LOC_Os11g20090.1	O-methyltransferase, putative, expressed	2,50	-1.0	1	23	421	443	CACUACAUUAUGGAAUGGAGGGA	UUGCUCCGUUUCAUAAUGUAGUG	Cleavage
tae-miR444b	Traes_6DL_D1C1DBD34.1										0,00	-1.0	1	21	274	294	UUGCUGCCUCAAGCUUGCUGC	GCAGCAAGCUUGAGGCAGCAA	Cleavage
tae-miR444a	Traes_6DL_D1C1DBD34.1										0,00	-1.0	1	21	274	294	UUGCUGCCUCAAGCUUGCUGC	GCAGCAAGCUUGAGGCAGCAA	Cleavage
tae-miR1117	Traes_6DL_E940A7985.1	PF00854		3.6.3.43		GO:GO:0016020,GO:GO:0006810,GO:GO:0005215	AT1G62200.1	Major facilitator superfamily protein	LOC_Os04g50940.1	peptide transporter PTR2, putative, expressed	2,50	-1.0	1	24	155	178	UAGUACCGGUUCGUGGCACGAACC	GGUUCGUGGCACAAACCGGUACUA	Cleavage
tae-miR1131	Traes_6DL_E940A7985.1	PF00854		3.6.3.43		GO:GO:0016020,GO:GO:0006810,GO:GO:0005215	AT1G62200.1	Major facilitator superfamily protein	LOC_Os04g50940.1	peptide transporter PTR2, putative, expressed	1,00	-1.0	1	22	181	202	UAGUACCGGUUCGUGGCUAACC	GAUUCGCCACGAACCGGUACUA	Cleavage
tae-miR1122a	Traes_6DS_0D9BB546D.1	PF00300		3.1.3.3	K15634		AT3G50520.1	Phosphoglycerate mutase family protein	LOC_Os08g37140.1	phosphoglycerate mutase, putative, expressed	2,50	-1.0	1	20	111	130	UAGAUACAUCCGUAUCUAGA	ACUACAUACGGAUGUAUAUA	Cleavage
tae-miR9662a-3p	Traes_6DS_0E84AAF77.1	PF02536				GO:GO:0006355,GO:GO:0005739,GO:GO:0003690	AT5G07900.1	Mitochondrial transcription termination factor family protein	LOC_Os06g12100.1	mTERF family protein, expressed	2,50	-1.0	1	21	480	500	UUGAACAUCCCAGAGCCACCG	CCGUGGCUCUGGGAUGCUCAG	Cleavage
tae-miR9662b-3p	Traes_6DS_0E84AAF77.1	PF02536				GO:GO:0006355,GO:GO:0005739,GO:GO:0003690	AT5G07900.1	Mitochondrial transcription termination factor family protein	LOC_Os06g12100.1	mTERF family protein, expressed	2,50	-1.0	1	21	479	499	UGAACAUCCCAGAGCCACCGG	CCCGUGGCUCUGGGAUGCUCA	Cleavage
tae-miR9674b-5p	Traes_6DS_1BEF46735.1	PF13041					AT1G63130.1	Tetratricopeptide repeat (TPR)-like superfamily protein	LOC_Os10g35240.1	Rf1, mitochondrial precursor, putative, expressed	3,00	-1.0	1	21	537	557	AUAGCAUCAUCCAUCCUACCC	GGGCCGAAUGGAUGAUGCUAU	Cleavage
tae-miR9674b-5p	Traes_6DS_1BEF46735.1	PF13041					AT1G63130.1	Tetratricopeptide repeat (TPR)-like superfamily protein	LOC_Os10g35240.1	Rf1, mitochondrial precursor, putative, expressed	3,00	-1.0	1	21	537	557	AUAGCAUCAUCCAUCCUACCC	GGGCCGAAUGGAUGAUGCUAU	Cleavage
tae-miR1127b-3p	Traes_6DS_2207A3BC4.7	PF14438,PF06741	KOG2375				AT3G14010.4	CTC-interacting domain 4	LOC_Os02g27950.2	ataxin-2 related protein, putative, expressed	1,50	-1.0	1	21	1729	1749	ACAAGUAUUUCUGGACGGAGG	CCUCCGUCCGGAAAUACUUGU	Cleavage
tae-miR9662a-3p	Traes_6DS_2D4EFFF3E.1	PF02536				GO:GO:0006355,GO:GO:0005739,GO:GO:0003690	AT5G07900.1	Mitochondrial transcription termination factor family protein	LOC_Os06g12100.1	mTERF family protein, expressed	1,00	-1.0	1	21	369	389	UUGAACAUCCCAGAGCCACCG	CCGUGGCUCUGGGAUGUUCAG	Cleavage
tae-miR9662b-3p	Traes_6DS_2D4EFFF3E.1	PF02536				GO:GO:0006355,GO:GO:0005739,GO:GO:0003690	AT5G07900.1	Mitochondrial transcription termination factor family protein	LOC_Os06g12100.1	mTERF family protein, expressed	1,00	-1.0	1	21	368	388	UGAACAUCCCAGAGCCACCGG	CCCGUGGCUCUGGGAUGUUCA	Cleavage
tae-miR9662a-3p	Traes_6DS_3F194116E.1	PF02536				GO:GO:0006355,GO:GO:0005739,GO:GO:0003690	AT5G07900.1	Mitochondrial transcription termination factor family protein	LOC_Os06g12100.1	mTERF family protein, expressed	2,50	-1.0	1	21	525	545	UUGAACAUCCCAGAGCCACCG	CCGUGGCUCGGGGAUGUUCAA	Cleavage
tae-miR9662b-3p	Traes_6DS_3F194116E.1	PF02536				GO:GO:0006355,GO:GO:0005739,GO:GO:0003690	AT5G07900.1	Mitochondrial transcription termination factor family protein	LOC_Os06g12100.1	mTERF family protein, expressed	2,50	-1.0	1	21	524	544	UGAACAUCCCAGAGCCACCGG	CCCGUGGCUCGGGGAUGUUCA	Translation
tae-miR9662a-3p	Traes_6DS_3FC2ADA87.1	PF02536				GO:GO:0006355,GO:GO:0005739,GO:GO:0003690	AT5G07900.1	Mitochondrial transcription termination factor family protein	LOC_Os06g12100.1	mTERF family protein, expressed	3,00	-1.0	1	21	242	263	UUGAACAUCCCAGAGCCAC-CG	CGCGUGGCUCUGGGAUGUUCAG	Cleavage
tae-miR9662b-3p	Traes_6DS_3FC2ADA87.1	PF02536				GO:GO:0006355,GO:GO:0005739,GO:GO:0003690	AT5G07900.1	Mitochondrial transcription termination factor family protein	LOC_Os06g12100.1	mTERF family protein, expressed	2,00	-1.0	1	21	241	262	UGAACAUCCCAGAGCCAC-CGG	CCGCGUGGCUCUGGGAUGUUCA	Cleavage
tae-miR9662a-3p	Traes_6DS_3FC2ADA87.2	PF02536				GO:GO:0006355,GO:GO:0005739,GO:GO:0003690	AT1G21150.1	Mitochondrial transcription termination factor family protein	LOC_Os06g12100.1	mTERF family protein, expressed	3,00	-1.0	1	21	65	86	UUGAACAUCCCAGAGCCAC-CG	CGCGUGGCUCUGGGAUGUUCAG	Cleavage
tae-miR9662b-3p	Traes_6DS_3FC2ADA87.2	PF02536				GO:GO:0006355,GO:GO:0005739,GO:GO:0003690	AT1G21150.1	Mitochondrial transcription termination factor family protein	LOC_Os06g12100.1	mTERF family protein, expressed	2,00	-1.0	1	21	64	85	UGAACAUCCCAGAGCCAC-CGG	CCGCGUGGCUCUGGGAUGUUCA	Cleavage
tae-miR9662a-3p	Traes_6DS_422644406.1	PF02536				GO:GO:0006355,GO:GO:0005739,GO:GO:0003690	AT5G07900.1	Mitochondrial transcription termination factor family protein	LOC_Os06g12100.1	mTERF family protein, expressed	2,50	-1.0	1	21	468	488	UUGAACAUCCCAGAGCCACCG	CCGUGGCUCUGGAAUGUUCAG	Cleavage
tae-miR9662b-3p	Traes_6DS_422644406.1	PF02536				GO:GO:0006355,GO:GO:0005739,GO:GO:0003690	AT5G07900.1	Mitochondrial transcription termination factor family protein	LOC_Os06g12100.1	mTERF family protein, expressed	2,50	-1.0	1	21	467	487	UGAACAUCCCAGAGCCACCGG	CCCGUGGCUCUGGAAUGUUCA	Cleavage
tae-miR9662a-3p	Traes_6DS_422644406.2	PF02536				GO:GO:0006355,GO:GO:0005739,GO:GO:0003690	AT5G07900.1	Mitochondrial transcription termination factor family protein	LOC_Os06g12100.1	mTERF family protein, expressed	2,50	-1.0	1	21	66	86	UUGAACAUCCCAGAGCCACCG	CCGUGGCUCUGGAAUGUUCAG	Cleavage
tae-miR9662b-3p	Traes_6DS_422644406.2	PF02536				GO:GO:0006355,GO:GO:0005739,GO:GO:0003690	AT5G07900.1	Mitochondrial transcription termination factor family protein	LOC_Os06g12100.1	mTERF family protein, expressed	2,50	-1.0	1	21	65	85	UGAACAUCCCAGAGCCACCGG	CCCGUGGCUCUGGAAUGUUCA	Cleavage
tae-miR9662a-3p	Traes_6DS_422644406.3	PF02536				GO:GO:0006355,GO:GO:0005739,GO:GO:0003690	AT5G07900.1	Mitochondrial transcription termination factor family protein	LOC_Os06g12100.1	mTERF family protein, expressed	2,50	-1.0	1	21	66	86	UUGAACAUCCCAGAGCCACCG	CCGUGGCUCUGGAAUGUUCAG	Cleavage
tae-miR9662b-3p	Traes_6DS_422644406.3	PF02536				GO:GO:0006355,GO:GO:0005739,GO:GO:0003690	AT5G07900.1	Mitochondrial transcription termination factor family protein	LOC_Os06g12100.1	mTERF family protein, expressed	2,50	-1.0	1	21	65	85	UGAACAUCCCAGAGCCACCGG	CCCGUGGCUCUGGAAUGUUCA	Cleavage
tae-miR9662a-3p	Traes_6DS_422644406.4	PF02536				GO:GO:0006355,GO:GO:0005739,GO:GO:0003690	AT5G07900.1	Mitochondrial transcription termination factor family protein	LOC_Os06g12100.1	mTERF family protein, expressed	2,50	-1.0	1	21	66	86	UUGAACAUCCCAGAGCCACCG	CCGUGGCUCUGGAAUGUUCAG	Cleavage
tae-miR9662b-3p	Traes_6DS_422644406.4	PF02536				GO:GO:0006355,GO:GO:0005739,GO:GO:0003690	AT5G07900.1	Mitochondrial transcription termination factor family protein	LOC_Os06g12100.1	mTERF family protein, expressed	2,50	-1.0	1	21	65	85	UGAACAUCCCAGAGCCACCGG	CCCGUGGCUCUGGAAUGUUCA	Cleavage
tae-miR156	Traes_6DS_7B1596298.1	PF03110				GO:GO:0005634,GO:GO:0003677	AT5G43270.2	squamosa promoter binding protein-like 2	LOC_Os02g04680.2	OsSPL3 - SBP-box gene family member, expressed	1,00	-1.0	1	21	1150	1170	UGACAGAAGAGAGUGAGCACA	CAUGCUCUCUCUCUUCUGUCA	Cleavage
tae-miR156	Traes_6DS_7B1596298.2	PF03110				GO:GO:0005634,GO:GO:0003677	AT5G43270.1	squamosa promoter binding protein-like 2	LOC_Os02g04680.2	OsSPL3 - SBP-box gene family member, expressed	1,00	-1.0	1	21	1150	1170	UGACAGAAGAGAGUGAGCACA	CAUGCUCUCUCUCUUCUGUCA	Cleavage
tae-miR156	Traes_6DS_7B1596298.4								LOC_Os02g04680.1	OsSPL3 - SBP-box gene family member, expressed	1,00	-1.0	1	21	271	291	UGACAGAAGAGAGUGAGCACA	CAUGCUCUCUCUCUUCUGUCA	Cleavage
tae-miR9674b-5p	Traes_6DS_7C389C178.1	PF12854,PF13041					AT1G62670.1	rna processing factor 2	LOC_Os10g35240.2	Rf1, mitochondrial precursor, putative, expressed	3,00	-1.0	1	21	537	557	AUAGCAUCAUCCAUCCUACCC	GGGCCGAAUGGAUGAUGCUAU	Cleavage
tae-miR9674a-5p	Traes_6DS_7C389C178.1	PF12854,PF13041					AT1G62670.1	rna processing factor 2	LOC_Os10g35240.2	Rf1, mitochondrial precursor, putative, expressed	2,50	-1.0	1	21	954	974	GCAUCAUCCAUCCUACCAUUC	GAAUGGAAGGAUAGAUGAUGC	Cleavage
tae-miR9674b-5p	Traes_6DS_7C389C178.1	PF12854,PF13041					AT1G62670.1	rna processing factor 2	LOC_Os10g35240.2	Rf1, mitochondrial precursor, putative, expressed	3,00	-1.0	1	21	537	557	AUAGCAUCAUCCAUCCUACCC	GGGCCGAAUGGAUGAUGCUAU	Cleavage
tae-miR9674a-5p	Traes_6DS_7C389C178.2	PF12854					AT1G62670.1	rna processing factor 2	LOC_Os10g35436.1	Rf1, mitochondrial precursor, putative, expressed	2,50	-1.0	1	21	51	71	GCAUCAUCCAUCCUACCAUUC	GAAUGGAAGGAUAGAUGAUGC	Cleavage
tae-miR9674b-5p	Traes_6DS_7C389C178.3	PF12854,PF13041					AT1G62670.1	rna processing factor 2	LOC_Os10g35240.1	Rf1, mitochondrial precursor, putative, expressed	3,00	-1.0	1	21	537	557	AUAGCAUCAUCCAUCCUACCC	GGGCCGAAUGGAUGAUGCUAU	Cleavage
tae-miR9674a-5p	Traes_6DS_7C389C178.3	PF12854,PF13041					AT1G62670.1	rna processing factor 2	LOC_Os10g35240.1	Rf1, mitochondrial precursor, putative, expressed	2,50	-1.0	1	21	954	974	GCAUCAUCCAUCCUACCAUUC	GAAUGGAAGGAUAGAUGAUGC	Cleavage
tae-miR9674b-5p	Traes_6DS_7C389C178.3	PF12854,PF13041					AT1G62670.1	rna processing factor 2	LOC_Os10g35240.1	Rf1, mitochondrial precursor, putative, expressed	3,00	-1.0	1	21	537	557	AUAGCAUCAUCCAUCCUACCC	GGGCCGAAUGGAUGAUGCUAU	Cleavage
tae-miR9662a-3p	Traes_6DS_7D3BEF6A9.1	PF02536				GO:GO:0006355,GO:GO:0005739,GO:GO:0003690	AT1G21150.1	Mitochondrial transcription termination factor family protein	LOC_Os06g12100.1	mTERF family protein, expressed	2,50	-1.0	1	21	33	53	UUGAACAUCCCAGAGCCACCG	UCGUGGCUCGGGGAUGUUCAG	Cleavage
tae-miR9662b-3p	Traes_6DS_7D3BEF6A9.1	PF02536				GO:GO:0006355,GO:GO:0005739,GO:GO:0003690	AT1G21150.1	Mitochondrial transcription termination factor family protein	LOC_Os06g12100.1	mTERF family protein, expressed	2,50	-1.0	1	21	32	52	UGAACAUCCCAGAGCCACCGG	CUCGUGGCUCGGGGAUGUUCA	Translation
tae-miR9662a-3p	Traes_6DS_7D3BEF6A9.2	PF02536				GO:GO:0006355,GO:GO:0005739,GO:GO:0003690	AT1G21150.1	Mitochondrial transcription termination factor family protein	LOC_Os06g12100.1	mTERF family protein, expressed	2,50	-1.0	1	21	246	266	UUGAACAUCCCAGAGCCACCG	UCGUGGCUCGGGGAUGUUCAG	Cleavage
tae-miR9662b-3p	Traes_6DS_7D3BEF6A9.2	PF02536				GO:GO:0006355,GO:GO:0005739,GO:GO:0003690	AT1G21150.1	Mitochondrial transcription termination factor family protein	LOC_Os06g12100.1	mTERF family protein, expressed	2,50	-1.0	1	21	245	265	UGAACAUCCCAGAGCCACCGG	CUCGUGGCUCGGGGAUGUUCA	Translation
tae-miR9662a-3p	Traes_6DS_7D3BEF6A9.3	PF02536				GO:GO:0006355,GO:GO:0005739,GO:GO:0003690	AT1G21150.1	Mitochondrial transcription termination factor family protein	LOC_Os06g12100.1	mTERF family protein, expressed	2,50	-1.0	1	21	246	266	UUGAACAUCCCAGAGCCACCG	UCGUGGCUCGGGGAUGUUCAG	Cleavage
tae-miR9662b-3p	Traes_6DS_7D3BEF6A9.3	PF02536				GO:GO:0006355,GO:GO:0005739,GO:GO:0003690	AT1G21150.1	Mitochondrial transcription termination factor family protein	LOC_Os06g12100.1	mTERF family protein, expressed	2,50	-1.0	1	21	245	265	UGAACAUCCCAGAGCCACCGG	CUCGUGGCUCGGGGAUGUUCA	Translation
tae-miR9662a-3p	Traes_6DS_893F318A9.1	PF02536				GO:GO:0006355,GO:GO:0005739,GO:GO:0003690	AT5G07900.1	Mitochondrial transcription termination factor family protein	LOC_Os06g12100.1	mTERF family protein, expressed	2,50	-1.0	1	21	549	569	UUGAACAUCCCAGAGCCACCG	CCGUGGCUCGGGGAUGUUCAA	Cleavage
tae-miR9662b-3p	Traes_6DS_893F318A9.1	PF02536				GO:GO:0006355,GO:GO:0005739,GO:GO:0003690	AT5G07900.1	Mitochondrial transcription termination factor family protein	LOC_Os06g12100.1	mTERF family protein, expressed	2,50	-1.0	1	21	548	568	UGAACAUCCCAGAGCCACCGG	CCCGUGGCUCGGGGAUGUUCA	Translation
tae-miR9662a-3p	Traes_6DS_893F318A9.2	PF02536				GO:GO:0006355,GO:GO:0005739,GO:GO:0003690	AT5G07900.1	Mitochondrial transcription termination factor family protein	LOC_Os06g12100.1	mTERF family protein, expressed	2,50	-1.0	1	21	132	152	UUGAACAUCCCAGAGCCACCG	CCGUGGCUCGGGGAUGUUCAA	Cleavage
tae-miR9662b-3p	Traes_6DS_893F318A9.2	PF02536				GO:GO:0006355,GO:GO:0005739,GO:GO:0003690	AT5G07900.1	Mitochondrial transcription termination factor family protein	LOC_Os06g12100.1	mTERF family protein, expressed	2,50	-1.0	1	21	131	151	UGAACAUCCCAGAGCCACCGG	CCCGUGGCUCGGGGAUGUUCA	Translation
tae-miR9662a-3p	Traes_6DS_931C28AF6.1	PF02536				GO:GO:0006355,GO:GO:0005739,GO:GO:0003690	AT1G79220.1	Mitochondrial transcription termination factor family protein	LOC_Os06g12100.1	mTERF family protein, expressed	2,50	-1.0	1	21	288	308	UUGAACAUCCCAGAGCCACCG	AUGUGGCUCUCGGAUGUUCAG	Translation
tae-miR9662a-3p	Traes_6DS_931C28AF6.2	PF02536				GO:GO:0006355,GO:GO:0005739,GO:GO:0003690	AT1G79220.1	Mitochondrial transcription termination factor family protein	LOC_Os06g12100.1	mTERF family protein, expressed	2,50	-1.0	1	21	276	296	UUGAACAUCCCAGAGCCACCG	AUGUGGCUCUCGGAUGUUCAG	Translation
tae-miR9662a-3p	Traes_6DS_931C28AF6.3	PF02536				GO:GO:0006355,GO:GO:0005739,GO:GO:0003690	AT1G79220.1	Mitochondrial transcription termination factor family protein	LOC_Os06g12100.1	mTERF family protein, expressed	2,50	-1.0	1	21	273	293	UUGAACAUCCCAGAGCCACCG	AUGUGGCUCUCGGAUGUUCAG	Translation
tae-miR9662a-3p	Traes_6DS_931C28AF6.4	PF02536				GO:GO:0006355,GO:GO:0005739,GO:GO:0003690	AT1G79220.1	Mitochondrial transcription termination factor family protein	LOC_Os06g12100.1	mTERF family protein, expressed	2,50	-1.0	1	21	273	293	UUGAACAUCCCAGAGCCACCG	AUGUGGCUCUCGGAUGUUCAG	Translation
tae-miR9662a-3p	Traes_6DS_931C28AF6.5	PF02536				GO:GO:0006355,GO:GO:0005739,GO:GO:0003690	AT1G79220.1	Mitochondrial transcription termination factor family protein	LOC_Os06g12100.1	mTERF family protein, expressed	2,50	-1.0	1	21	276	296	UUGAACAUCCCAGAGCCACCG	AUGUGGCUCUCGGAUGUUCAG	Translation
tae-miR9662b-3p	Traes_6DS_93C7DA226.1	PF02536				GO:GO:0006355,GO:GO:0005739,GO:GO:0003690	AT5G07900.1	Mitochondrial transcription termination factor family protein	LOC_Os06g12100.1	mTERF family protein, expressed	3,00	-1.0	1	21	503	523	UGAACAUCCCAGAGCCACCGG	CCCGUGCUUCUGGGAUGUUCA	Cleavage
tae-miR9662b-3p	Traes_6DS_93C7DA2261.1	PF02536				GO:GO:0006355,GO:GO:0005739,GO:GO:0003690	AT5G07900.1	Mitochondrial transcription termination factor family protein	LOC_Os06g12100.1	mTERF family protein, expressed	3,00	-1.0	1	21	503	523	UGAACAUCCCAGAGCCACCGG	CCCGUGCUUCUGGGAUGUUCA	Cleavage
tae-miR9662a-3p	Traes_6DS_A490DF33E.1	PF02536				GO:GO:0006355,GO:GO:0005739,GO:GO:0003690	AT5G07900.1	Mitochondrial transcription termination factor family protein	LOC_Os06g12100.1	mTERF family protein, expressed	2,50	-1.0	1	21	471	491	UUGAACAUCCCAGAGCCACCG	CCGUGGCUCUGGAAUGUUCAG	Cleavage
tae-miR9662b-3p	Traes_6DS_A490DF33E.1	PF02536				GO:GO:0006355,GO:GO:0005739,GO:GO:0003690	AT5G07900.1	Mitochondrial transcription termination factor family protein	LOC_Os06g12100.1	mTERF family protein, expressed	2,50	-1.0	1	21	470	490	UGAACAUCCCAGAGCCACCGG	CCCGUGGCUCUGGAAUGUUCA	Cleavage
tae-miR9662a-3p	Traes_6DS_A490DF33E.2	PF02536				GO:GO:0006355,GO:GO:0005739,GO:GO:0003690	AT1G21150.1	Mitochondrial transcription termination factor family protein	LOC_Os06g12100.1	mTERF family protein, expressed	2,50	-1.0	1	21	66	86	UUGAACAUCCCAGAGCCACCG	CCGUGGCUCUGGAAUGUUCAG	Cleavage
tae-miR9662b-3p	Traes_6DS_A490DF33E.2	PF02536				GO:GO:0006355,GO:GO:0005739,GO:GO:0003690	AT1G21150.1	Mitochondrial transcription termination factor family protein	LOC_Os06g12100.1	mTERF family protein, expressed	2,50	-1.0	1	21	65	85	UGAACAUCCCAGAGCCACCGG	CCCGUGGCUCUGGAAUGUUCA	Cleavage
tae-miR1122b-3p	Traes_6DS_C1B7F14A41.2	PF08263,PF07714,PF13855	KOG1187	2.7.11.1		GO:GO:0006468,GO:GO:0004672,GO:GO:0005515	AT1G72300.1	Leucine-rich receptor-like protein kinase family protein	LOC_Os02g05930.1	phytosulfokine receptor precursor, putative, expressed	1,50	-1.0	1	21	3044	3064	AGACUUAUAUGUAGGAACGGA	UCCGUUCCUAAAUAUAAGUCU	Translation
tae-miR156	Traes_6DS_C4D93239C.1	PF03110				GO:GO:0005634,GO:GO:0003677	AT5G43270.1	squamosa promoter binding protein-like 2	LOC_Os06g45310.1	OsSPL11 - SBP-box gene family member, expressed	1,00	-1.0	1	21	577	597	UGACAGAAGAGAGUGAGCACA	CAUGCUCUCUCUCUUCUGUCA	Cleavage
tae-miR156	Traes_6DS_C4D93239C.2								LOC_Os06g45310.1	OsSPL11 - SBP-box gene family member, expressed	1,00	-1.0	1	21	88	108	UGACAGAAGAGAGUGAGCACA	CAUGCUCUCUCUCUUCUGUCA	Cleavage
tae-miR156	Traes_6DS_C4D93239C.3								LOC_Os06g45310.1	OsSPL11 - SBP-box gene family member, expressed	1,00	-1.0	1	21	64	84	UGACAGAAGAGAGUGAGCACA	CAUGCUCUCUCUCUUCUGUCA	Cleavage
tae-miR9662a-3p	Traes_6DS_CF627A1BC.1								LOC_Os06g12100.1	mTERF family protein, expressed	2,50	-1.0	1	21	75	95	UUGAACAUCCCAGAGCCACCG	CCGUGGCUCGGGGAUGUUCAG	Cleavage
tae-miR9662b-3p	Traes_6DS_CF627A1BC.1								LOC_Os06g12100.1	mTERF family protein, expressed	2,50	-1.0	1	21	74	94	UGAACAUCCCAGAGCCACCGG	CCCGUGGCUCGGGGAUGUUCA	Translation
tae-miR9662a-3p	Traes_6DS_DAD8BEF41.1	PF02536				GO:GO:0006355,GO:GO:0005739,GO:GO:0003690	AT5G07900.1	Mitochondrial transcription termination factor family protein	LOC_Os06g12100.1	mTERF family protein, expressed	2,50	-1.0	1	21	477	497	UUGAACAUCCCAGAGCCACCG	CCGUGGCUCUGGAAUGUUCAG	Cleavage
tae-miR9662b-3p	Traes_6DS_DAD8BEF41.1	PF02536				GO:GO:0006355,GO:GO:0005739,GO:GO:0003690	AT5G07900.1	Mitochondrial transcription termination factor family protein	LOC_Os06g12100.1	mTERF family protein, expressed	2,50	-1.0	1	21	476	496	UGAACAUCCCAGAGCCACCGG	CCCGUGGCUCUGGAAUGUUCA	Cleavage
tae-miR9662a-3p	Traes_6DS_E352D66A4.1	PF02536				GO:GO:0006355,GO:GO:0005739,GO:GO:0003690	AT5G64950.1	Mitochondrial transcription termination factor family protein	LOC_Os06g12100.1	mTERF family protein, expressed	2,50	-1.0	1	21	450	470	UUGAACAUCCCAGAGCCACCG	CCGUGGCUCUGGGAUGUUUAG	Cleavage
tae-miR9662b-3p	Traes_6DS_E352D66A4.1	PF02536				GO:GO:0006355,GO:GO:0005739,GO:GO:0003690	AT5G64950.1	Mitochondrial transcription termination factor family protein	LOC_Os06g12100.1	mTERF family protein, expressed	2,50	-1.0	1	21	449	469	UGAACAUCCCAGAGCCACCGG	CCCGUGGCUCUGGGAUGUUUA	Cleavage
tae-miR9662a-3p	Traes_6DS_E95930B7D.1	PF02536				GO:GO:0006355,GO:GO:0005739,GO:GO:0003690	AT5G64950.1	Mitochondrial transcription termination factor family protein	LOC_Os06g12100.1	mTERF family protein, expressed	2,50	-1.0	1	21	450	470	UUGAACAUCCCAGAGCCACCG	CCGUGGCUCUGGGAUGUUUAG	Cleavage
tae-miR9662b-3p	Traes_6DS_E95930B7D.1	PF02536				GO:GO:0006355,GO:GO:0005739,GO:GO:0003690	AT5G64950.1	Mitochondrial transcription termination factor family protein	LOC_Os06g12100.1	mTERF family protein, expressed	2,50	-1.0	1	21	449	469	UGAACAUCCCAGAGCCACCGG	CCCGUGGCUCUGGGAUGUUUA	Cleavage
tae-miR9662a-3p	Traes_6DS_EF75D5C67.1	PF02536				GO:GO:0006355,GO:GO:0005739,GO:GO:0003690	AT5G07900.1	Mitochondrial transcription termination factor family protein	LOC_Os06g12100.1	mTERF family protein, expressed	2,00	-1.0	1	21	66	86	UUGAACAUCCCAGAGCCACCG	UCGUGGCGCUGGGAUGUUCAG	Cleavage
tae-miR9662b-3p	Traes_6DS_EF75D5C67.1	PF02536				GO:GO:0006355,GO:GO:0005739,GO:GO:0003690	AT5G07900.1	Mitochondrial transcription termination factor family protein	LOC_Os06g12100.1	mTERF family protein, expressed	2,50	-1.0	1	21	65	85	UGAACAUCCCAGAGCCACCGG	CUCGUGGCGCUGGGAUGUUCA	Cleavage
tae-miR9662a-3p	Traes_6DS_F6C7E588F.1	PF02536	KOG1267			GO:GO:0006355,GO:GO:0005739,GO:GO:0003690	AT5G07900.1	Mitochondrial transcription termination factor family protein	LOC_Os06g12100.1	mTERF family protein, expressed	2,00	-1.0	1	21	657	677	UUGAACAUCCCAGAGCCACCG	CCGUGGUUCUGGGAUGUUCAG	Cleavage
tae-miR9662b-3p	Traes_6DS_F6C7E588F.1	PF02536	KOG1267			GO:GO:0006355,GO:GO:0005739,GO:GO:0003690	AT5G07900.1	Mitochondrial transcription termination factor family protein	LOC_Os06g12100.1	mTERF family protein, expressed	2,00	-1.0	1	21	656	676	UGAACAUCCCAGAGCCACCGG	CCCGUGGUUCUGGGAUGUUCA	Cleavage
tae-miR9662a-3p	Traes_6DS_FB434BB3C.1	PF02536				GO:GO:0006355,GO:GO:0005739,GO:GO:0003690	AT5G64950.1	Mitochondrial transcription termination factor family protein	LOC_Os06g12100.1	mTERF family protein, expressed	2,50	-1.0	1	21	246	266	UUGAACAUCCCAGAGCCACCG	CCGUGGCUCUCGGAUGUUCAG	Translation
tae-miR9662b-3p	Traes_6DS_FB434BB3C.1	PF02536				GO:GO:0006355,GO:GO:0005739,GO:GO:0003690	AT5G64950.1	Mitochondrial transcription termination factor family protein	LOC_Os06g12100.1	mTERF family protein, expressed	2,50	-1.0	1	21	245	265	UGAACAUCCCAGAGCCACCGG	CCCGUGGCUCUCGGAUGUUCA	Translation
tae-miR9662a-3p	Traes_6DS_FB434BB3C.2	PF02536				GO:GO:0006355,GO:GO:0005739,GO:GO:0003690	AT5G64950.1	Mitochondrial transcription termination factor family protein	LOC_Os06g12100.1	mTERF family protein, expressed	2,50	-1.0	1	21	246	266	UUGAACAUCCCAGAGCCACCG	CCGUGGCUCUCGGAUGUUCAG	Translation
tae-miR9662b-3p	Traes_6DS_FB434BB3C.2	PF02536				GO:GO:0006355,GO:GO:0005739,GO:GO:0003690	AT5G64950.1	Mitochondrial transcription termination factor family protein	LOC_Os06g12100.1	mTERF family protein, expressed	2,50	-1.0	1	21	245	265	UGAACAUCCCAGAGCCACCGG	CCCGUGGCUCUCGGAUGUUCA	Translation
tae-miR1122a	Traes_7AL_135129885.3	PF01138	KOG1612				AT3G07750.1	3\'-5\'-exoribonuclease family protein	LOC_Os12g21500.1	exosome complex exonuclease, putative, expressed	2,50	-1.0	1	20	721	740	UAGAUACAUCCGUAUCUAGA	ACUACAUACGGAUGUAUAUA	Cleavage
tae-miR164	Traes_7AL_628A69311.1	PF02365				GO:GO:0006355,GO:GO:0003677	AT1G56010.2	NAC domain containing protein 1	LOC_Os06g46270.1	no apical meristem protein, putative, expressed	1,00	-1.0	1	21	650	670	UGGAGAAGCAGGGCACGUGCA	AGCAAGUGCCCUGCUUCUCCA	Cleavage
tae-miR1130b-3p	Traes_7AL_955F54182.1	PF02705				GO:GO:0071805,GO:GO:0016020,GO:GO:0015079	AT2G30070.1	potassium transporter 1	LOC_Os06g45940.2	potassium transporter, putative, expressed	0,00	-1.0	1	21	1141	1161	UCUUAUAUUAUGGGACGGAGG	CCUCCGUCCCAUAAUAUAAGA	Cleavage
tae-miR1122c-3p	Traes_7AL_955F54182.1	PF02705				GO:GO:0071805,GO:GO:0016020,GO:GO:0015079	AT2G30070.1	potassium transporter 1	LOC_Os06g45940.2	potassium transporter, putative, expressed	1,50	-1.0	1	21	1141	1161	UCUAAUAUUAUGGGACGGAGG	CCUCCGUCCCAUAAUAUAAGA	Cleavage
tae-miR1133	Traes_7AL_ADBB1224B.1	PF08315					AT3G05070.1		LOC_Os06g51420.1	coiled-coil domain-containing protein 12, putative, expressed	2,50	-1.0	1	22	52	73	CAUAUACUCCCUCCGUCCGAAA	UUCCGGACGGAGGGAGUAUAAU	Cleavage
tae-miR164	Traes_7AL_BE3253C8F.1	PF02365				GO:GO:0006355,GO:GO:0003677	AT1G56010.2	NAC domain containing protein 1	LOC_Os06g46270.1	no apical meristem protein, putative, expressed	1,00	-1.0	1	21	653	673	UGGAGAAGCAGGGCACGUGCA	AGCAAGUGCCCUGCUUCUCCA	Cleavage
tae-miR164	Traes_7AL_BE3253C8F.2						AT1G56010.2	NAC domain containing protein 1	LOC_Os06g46270.1	no apical meristem protein, putative, expressed	1,00	-1.0	1	21	215	235	UGGAGAAGCAGGGCACGUGCA	AGCAAGUGCCCUGCUUCUCCA	Cleavage
tae-miR160	Traes_7AL_E3ADC8C38.1	PF06507				GO:GO:0009725,GO:GO:0006355,GO:GO:0005634,GO:GO:0003677	AT2G28350.1	auxin response factor 10	LOC_Os06g47150.2	auxin response factor 18, putative, expressed	0,00	-1.0	1	21	399	419	UGCCUGGCUCCCUGUAUGCCA	AGGCAUACAGGGAGCCAGGCA	Cleavage
tae-miR9666a-3p	Traes_7AL_EE3CD1CF3.10	PF05001		2.7.7.6,2.7.7.48		GO:GO:0006366,GO:GO:0005665,GO:GO:0003677	AT4G35800.1	RNA polymerase II large subunit	LOC_Os05g05860.1	retrotransposon protein, putative, unclassified, expressed	2,00	-1.0	1	22	229	250	CGGUAGGGCUGUAUGAUGGCGA	UCCCCGUCAUACAGCCCUACCU	Cleavage
tae-miR9666a-3p	Traes_7AL_EE3CD1CF3.2	PF05001,PF05000,PF04983,PF04990,PF04992,PF00623,PF04997,PF04998		2.7.7.6	K03006	GO:GO:0006366,GO:GO:0005665,GO:GO:0003677,GO:GO:0006351,GO:GO:0003899	AT4G35800.1	RNA polymerase II large subunit	LOC_Os05g05860.1	retrotransposon protein, putative, unclassified, expressed	2,00	-1.0	1	22	5032	5053	CGGUAGGGCUGUAUGAUGGCGA	UCCCCGUCAUACAGCCCUACCU	Cleavage
tae-miR9666a-3p	Traes_7AL_EE3CD1CF3.3	PF05001,PF05000,PF04983,PF04990,PF04992,PF00623,PF04997,PF04998		2.7.7.6	K03006	GO:GO:0006366,GO:GO:0005665,GO:GO:0003677,GO:GO:0006351,GO:GO:0003899	AT4G35800.1	RNA polymerase II large subunit	LOC_Os05g05860.1	retrotransposon protein, putative, unclassified, expressed	2,00	-1.0	1	22	4948	4969	CGGUAGGGCUGUAUGAUGGCGA	UCCCCGUCAUACAGCCCUACCU	Cleavage
tae-miR9666a-3p	Traes_7AL_EE3CD1CF3.4	PF05001,PF05000,PF04983,PF04990,PF04997,PF04992,PF00623,PF04998		2.7.7.6	K03006	GO:GO:0006366,GO:GO:0005665,GO:GO:0003677,GO:GO:0006351,GO:GO:0003899	AT4G35800.1	RNA polymerase II large subunit	LOC_Os05g05860.1	retrotransposon protein, putative, unclassified, expressed	2,00	-1.0	1	22	4801	4822	CGGUAGGGCUGUAUGAUGGCGA	UCCCCGUCAUACAGCCCUACCU	Cleavage
tae-miR9666a-3p	Traes_7AL_EE3CD1CF3.5	PF05001,PF05000,PF04997,PF04983,PF04990,PF04992,PF00623,PF04998		2.7.7.6		GO:GO:0006366,GO:GO:0005665,GO:GO:0003677,GO:GO:0006351,GO:GO:0003899	AT4G35800.1	RNA polymerase II large subunit	LOC_Os05g05860.1	retrotransposon protein, putative, unclassified, expressed	2,00	-1.0	1	22	4393	4414	CGGUAGGGCUGUAUGAUGGCGA	UCCCCGUCAUACAGCCCUACCU	Cleavage
tae-miR9666a-3p	Traes_7AL_EE3CD1CF3.6	PF05001,PF05000,PF04983,PF04990,PF04992,PF04998		2.7.7.6		GO:GO:0006366,GO:GO:0005665,GO:GO:0003677,GO:GO:0006351,GO:GO:0003899	AT4G35800.1	RNA polymerase II large subunit	LOC_Os05g05860.1	retrotransposon protein, putative, unclassified, expressed	2,00	-1.0	1	22	3409	3430	CGGUAGGGCUGUAUGAUGGCGA	UCCCCGUCAUACAGCCCUACCU	Cleavage

tae-miR9666a-3p	Traes_7AL_EE3CD1CF3.8	PF05001,PF05000,PF04990,PF04992,PF04998		2.7.7.6		GO:GO:0006366,GO:GO:0005665,GO:GO:0003677,GO:GO:0006351,GO:GO:0003899	AT4G35800.1	RNA polymerase II large subunit	LOC_Os05g05860.1	retrotransposon protein, putative, unclassified, expressed	2,00	-1.0	1	22	2941	2962	CGGUAGGGCUGUAUGAUGGCGA	UCCCCGUCAUACAGCCCUACCU	Cleavage
tae-miR1137b-5p	Traes_7AS_0F0291904.1	PF00657		3.1.1.1		GO:GO:0016788	AT5G45910.1	GDSL-like Lipase/Acylhydrolase superfamily protein	LOC_Os01g12320.1	GDSL-like lipase/acylhydrolase, putative, expressed	2,50	-1.0	1	21	844	864	UCCGUUCCAGAAUAGAUGACC	ACUCAUCUAUUCUGGGACGGU	Cleavage
tae-miR1137b-5p	Traes_7AS_0F0291904.2	PF00657		3.1.1.1		GO:GO:0016788	AT2G27360.1	GDSL-like Lipase/Acylhydrolase superfamily protein	LOC_Os01g12320.2	GDSL-like lipase/acylhydrolase, putative, expressed	2,50	-1.0	1	21	451	471	UCCGUUCCAGAAUAGAUGACC	ACUCAUCUAUUCUGGGACGGU	Cleavage
tae-miR1137b-5p	Traes_7AS_23DFA827E.1	PF00400	KOG1188			GO:GO:0005515	AT2G47790.1	Transducin/WD40 repeat-like superfamily protein	LOC_Os12g19590.3	WD domain, G-beta repeat domain containing protein, expressed	1,50	-1.0	1	21	11	31	UCCGUUCCAGAAUAGAUGACC	AGUCAUCUAUUUUGGAACGGA	Translation
tae-miR1122a	Traes_7AS_9674F6981.1	PF01031		3.6.5.5		GO:GO:0005525	AT1G10290.1	dynamin-like protein 6	LOC_Os06g13820.1	dynamin, putative, expressed	1,50	-1.0	1	20	1467	1486	UAGAUACAUCCGUAUCUAGA	UCUAGAUACAGAUGUAUCUA	Translation
tae-miR1122a	Traes_7AS_9674F6981.2	PF01031		3.6.5.5		GO:GO:0005525	AT1G10290.1	dynamin-like protein 6	LOC_Os06g13820.1	dynamin, putative, expressed	1,50	-1.0	1	20	1473	1492	UAGAUACAUCCGUAUCUAGA	UCUAGAUACAGAUGUAUCUA	Translation
tae-miR1122a	Traes_7AS_9674F6981.3	PF01031		3.6.5.5		GO:GO:0005525	AT1G10290.1	dynamin-like protein 6	LOC_Os06g13820.1	dynamin, putative, expressed	1,50	-1.0	1	20	1467	1486	UAGAUACAUCCGUAUCUAGA	UCUAGAUACAGAUGUAUCUA	Translation
tae-miR1130b-3p	Traes_7AS_9819F7C48.1	PF01154,PF08540		2.3.3.10		GO:GO:0008299,GO:GO:0004421	AT4G11820.2	hydroxymethylglutaryl-CoA synthase / HMG-CoA synthase / 3-hydroxy-3-methylglutaryl coenzyme A synthase	LOC_Os08g43170.1	hydroxymethylglutaryl-CoA synthase, putative, expressed	0,00	-1.0	1	21	1268	1288	UCUUAUAUUAUGGGACGGAGG	AAUCCGUCCCAUAAUAUAAGA	Cleavage
tae-miR1122c-3p	Traes_7AS_9819F7C48.1	PF01154,PF08540		2.3.3.10		GO:GO:0008299,GO:GO:0004421	AT4G11820.2	hydroxymethylglutaryl-CoA synthase / HMG-CoA synthase / 3-hydroxy-3-methylglutaryl coenzyme A synthase	LOC_Os08g43170.1	hydroxymethylglutaryl-CoA synthase, putative, expressed	1,50	-1.0	1	21	1268	1288	UCUAAUAUUAUGGGACGGAGG	AAUCCGUCCCAUAAUAUAAGA	Cleavage
tae-miR1120c-5p	Traes_7AS_9819F7C48.1	PF01154,PF08540		2.3.3.10		GO:GO:0008299,GO:GO:0004421	AT4G11820.2	hydroxymethylglutaryl-CoA synthase / HMG-CoA synthase / 3-hydroxy-3-methylglutaryl coenzyme A synthase	LOC_Os08g43170.1	hydroxymethylglutaryl-CoA synthase, putative, expressed	1,50	-1.0	1	21	1311	1331	UAAUAUAAGAACGUUUUUGAC	GUCAAAAACGCUCUUAUAUUA	Translation
tae-miR5086	Traes_7AS_9E1AC3FE6.1										3,00	-1.0	1	21	324	344	ACAUUGGUGGAAGGCGUGGUA	CGCCAUGGCUUCCACCAAUGG	Cleavage
tae-miR7757-5p	Traes_7AS_E43D64A80.1	PF00931	KOG4658			GO:GO:0043531	AT3G46530.1	NB-ARC domain-containing disease resistance protein	LOC_Os07g26430.1	resistance protein, putative, expressed	2,50	-1.0	1	22	377	398	AUAAAACCUUCAGCUAUCCAUC	AAUGGAUAACUGAAGAUUUUAU	Cleavage
tae-miR7757-5p	Traes_7AS_E43D64A80.2	PF00931	KOG4658			GO:GO:0043531	AT3G46530.1	NB-ARC domain-containing disease resistance protein	LOC_Os07g26430.1	resistance protein, putative, expressed	2,50	-1.0	1	22	1424	1445	AUAAAACCUUCAGCUAUCCAUC	AAUGGAUAACUGAAGAUUUUAU	Cleavage
tae-miR156	Traes_7AS_FE67F9E86.1								LOC_Os08g41940.1	OsSPL16 - SBP-box gene family member, expressed	1,00	-1.0	1	21	358	378	UGACAGAAGAGAGUGAGCACA	UGUGCUCUCUCUCUUCUGUCA	Cleavage
tae-miR156	Traes_7AS_FE67F9E86.2								LOC_Os08g41940.1	OsSPL16 - SBP-box gene family member, expressed	1,00	-1.0	1	21	358	378	UGACAGAAGAGAGUGAGCACA	UGUGCUCUCUCUCUUCUGUCA	Cleavage
tae-miR156	Traes_7AS_FE67F9E86.3								LOC_Os08g41940.1	OsSPL16 - SBP-box gene family member, expressed	1,00	-1.0	1	21	331	351	UGACAGAAGAGAGUGAGCACA	UGUGCUCUCUCUCUUCUGUCA	Cleavage
tae-miR9654a-3p	Traes_7BL_03C3797A2.1	PF00635,PF00400,PF00069	KOG1187			GO:GO:0005515,GO:GO:0006468,GO:GO:0005524,GO:GO:0004672	AT1G79990.3	structural molecules	LOC_Os11g10640.1	protein kinase domain containing protein, expressed	2,00	-1.0	1	22	1813	1834	UUCUGAAAGGCUUGAAGCGAAU	GAGGGCCUCAAGCCUUUCAGAA	Cleavage
tae-miR9654a-3p	Traes_7BL_03C3797A2.2	PF00635,PF00400,PF00069	KOG1187			GO:GO:0005515,GO:GO:0006468,GO:GO:0005524,GO:GO:0004672	AT1G79990.3	structural molecules	LOC_Os11g10640.1	protein kinase domain containing protein, expressed	2,00	-1.0	1	22	1813	1834	UUCUGAAAGGCUUGAAGCGAAU	GAGGGCCUCAAGCCUUUCAGAA	Cleavage
tae-miR9654a-3p	Traes_7BL_03C3797A2.3	PF00635,PF00400,PF00069	KOG1187			GO:GO:0005515,GO:GO:0006468,GO:GO:0005524,GO:GO:0004672	AT1G79990.3	structural molecules	LOC_Os11g10640.1	protein kinase domain containing protein, expressed	2,00	-1.0	1	22	1513	1534	UUCUGAAAGGCUUGAAGCGAAU	GAGGGCCUCAAGCCUUUCAGAA	Cleavage
tae-miR9654a-3p	Traes_7BL_03C3797A2.4	PF00635,PF00400,PF00069	KOG1187			GO:GO:0005515,GO:GO:0006468,GO:GO:0005524,GO:GO:0004672	AT1G79990.3	structural molecules	LOC_Os11g10640.1	protein kinase domain containing protein, expressed	2,00	-1.0	1	22	1513	1534	UUCUGAAAGGCUUGAAGCGAAU	GAGGGCCUCAAGCCUUUCAGAA	Cleavage
tae-miR9654a-3p	Traes_7BL_03C3797A2.5	PF00635,PF00400,PF00069	KOG0294			GO:GO:0005515,GO:GO:0006468,GO:GO:0005524,GO:GO:0004672	AT1G79990.3	structural molecules	LOC_Os11g10640.1	protein kinase domain containing protein, expressed	2,00	-1.0	1	22	1399	1420	UUCUGAAAGGCUUGAAGCGAAU	GAGGGCCUCAAGCCUUUCAGAA	Cleavage
tae-miR9654a-3p	Traes_7BL_03C3797A2.6	PF00635,PF00400	KOG0294			GO:GO:0005515	AT1G79990.3	structural molecules	LOC_Os02g11830.2	coatomer subunit beta, putative, expressed	2,00	-1.0	1	22	973	994	UUCUGAAAGGCUUGAAGCGAAU	GAGGGCCUCAAGCCUUUCAGAA	Cleavage
tae-miR9654a-3p	Traes_7BL_03C3797A2.7	PF00635,PF00069	KOG1187	2.7.11.1		GO:GO:0006468,GO:GO:0005524,GO:GO:0004672	AT4G21410.1	cysteine-rich RLK (RECEPTOR-like protein kinase) 29	LOC_Os04g30030.1	cysteine-rich receptor-like protein kinase 12 precursor, putative, expressed	2,00	-1.0	1	22	1813	1834	UUCUGAAAGGCUUGAAGCGAAU	GAGGGCCUCAAGCCUUUCAGAA	Cleavage
tae-miR1130b-3p	Traes_7BL_0665E8707.6										1,00	-1.0	1	21	46	66	UCUUAUAUUAUGGGACGGAGG	CCUCCGUCCCAUAAUAUAAGU	Cleavage
tae-miR1122c-3p	Traes_7BL_0665E8707.6										2,50	-1.0	1	21	46	66	UCUAAUAUUAUGGGACGGAGG	CCUCCGUCCCAUAAUAUAAGU	Cleavage
tae-miR1133	Traes_7BL_07C4E8081.43										0,00	-1.0	1	22	46	67	CAUAUACUCCCUCCGUCCGAAA	UUUCGGACGGAGGGAGUAUAUG	Cleavage
tae-miR1133	Traes_7BL_07C4E8081.44										0,00	-1.0	1	22	46	67	CAUAUACUCCCUCCGUCCGAAA	UUUCGGACGGAGGGAGUAUAUG	Cleavage
tae-miR1133	Traes_7BL_07C4E8081.45										0,00	-1.0	1	22	46	67	CAUAUACUCCCUCCGUCCGAAA	UUUCGGACGGAGGGAGUAUAUG	Cleavage
tae-miR160	Traes_7BL_18D335F08.1						AT4G30080.1	auxin response factor 16	LOC_Os06g47150.2	auxin response factor 18, putative, expressed	0,00	-1.0	1	21	171	191	UGCCUGGCUCCCUGUAUGCCA	AGGCAUACAGGGAGCCAGGCA	Cleavage
tae-miR160	Traes_7BL_18D335F08.2						AT4G30080.1	auxin response factor 16	LOC_Os06g47150.1	auxin response factor 18, putative, expressed	0,00	-1.0	1	21	171	191	UGCCUGGCUCCCUGUAUGCCA	AGGCAUACAGGGAGCCAGGCA	Cleavage
tae-miR1137a	Traes_7BL_C184D0525.2	PF00249					AT5G61620.1	myb-like transcription factor family protein	LOC_Os08g05510.1	MYB family transcription factor, putative, expressed	0,00	-1.0	1	20	913	932	UAGUACAAAGUUGAGUCAUC	GAUGACUCAACUUUGUACUA	Cleavage
tae-miR395b	Traes_7BL_DC0D8773C.1	PF01909,PF04926,PF04928		2.7.7.19		GO:GO:0016779,GO:GO:0043631,GO:GO:0003723,GO:GO:0004652,GO:GO:0005634	AT4G32850.5	nuclear poly(a) polymerase	LOC_Os06g36360.4	expressed protein	2,50	-1.0	1	20	1385	1404	UGAAGUGUUUGGGGGAACUC	CAGUUCACCCAAAAACUUCA	Cleavage
tae-miR395b	Traes_7BL_DC0D8773C.10	PF04926,PF04928		2.7.7.19		GO:GO:0043631,GO:GO:0003723,GO:GO:0004652,GO:GO:0005634	AT4G32850.10	nuclear poly(a) polymerase	LOC_Os06g36360.1	expressed protein	2,50	-1.0	1	20	1148	1167	UGAAGUGUUUGGGGGAACUC	CAGUUCACCCAAAAACUUCA	Cleavage
tae-miR395b	Traes_7BL_DC0D8773C.11	PF04926,PF04928		2.7.7.19		GO:GO:0043631,GO:GO:0003723,GO:GO:0004652,GO:GO:0005634	AT4G32850.5	nuclear poly(a) polymerase	LOC_Os06g36360.3	expressed protein	2,50	-1.0	1	20	848	867	UGAAGUGUUUGGGGGAACUC	CAGUUCACCCAAAAACUUCA	Cleavage
tae-miR395b	Traes_7BL_DC0D8773C.12	PF04926,PF04928		2.7.7.19		GO:GO:0043631,GO:GO:0003723,GO:GO:0004652,GO:GO:0005634	AT4G32850.5	nuclear poly(a) polymerase	LOC_Os06g36360.1	expressed protein	2,50	-1.0	1	20	848	867	UGAAGUGUUUGGGGGAACUC	CAGUUCACCCAAAAACUUCA	Cleavage
tae-miR395b	Traes_7BL_DC0D8773C.13	PF04926,PF04928		2.7.7.19		GO:GO:0043631,GO:GO:0003723,GO:GO:0004652,GO:GO:0005634	AT4G32850.9	nuclear poly(a) polymerase	LOC_Os06g36360.1	expressed protein	2,50	-1.0	1	20	848	867	UGAAGUGUUUGGGGGAACUC	CAGUUCACCCAAAAACUUCA	Cleavage
tae-miR395b	Traes_7BL_DC0D8773C.14	PF04926,PF04928		2.7.7.19		GO:GO:0043631,GO:GO:0003723,GO:GO:0004652,GO:GO:0005634	AT4G32850.5	nuclear poly(a) polymerase	LOC_Os06g36360.3	expressed protein	2,50	-1.0	1	20	848	867	UGAAGUGUUUGGGGGAACUC	CAGUUCACCCAAAAACUUCA	Cleavage
tae-miR395b	Traes_7BL_DC0D8773C.2	PF01909,PF04926,PF04928		2.7.7.19		GO:GO:0016779,GO:GO:0043631,GO:GO:0003723,GO:GO:0004652,GO:GO:0005634	AT4G32850.5	nuclear poly(a) polymerase	LOC_Os06g36360.4	expressed protein	2,50	-1.0	1	20	1385	1404	UGAAGUGUUUGGGGGAACUC	CAGUUCACCCAAAAACUUCA	Cleavage
tae-miR395b	Traes_7BL_DC0D8773C.3	PF01909,PF04926,PF04928		2.7.7.19		GO:GO:0016779,GO:GO:0043631,GO:GO:0003723,GO:GO:0004652,GO:GO:0005634	AT4G32850.9	nuclear poly(a) polymerase	LOC_Os06g36360.1	expressed protein	2,50	-1.0	1	20	1385	1404	UGAAGUGUUUGGGGGAACUC	CAGUUCACCCAAAAACUUCA	Cleavage
tae-miR395b	Traes_7BL_DC0D8773C.4	PF01909,PF04926,PF04928		2.7.7.19		GO:GO:0016779,GO:GO:0043631,GO:GO:0003723,GO:GO:0004652,GO:GO:0005634	AT4G32850.5	nuclear poly(a) polymerase	LOC_Os06g36360.2	expressed protein	2,50	-1.0	1	20	1385	1404	UGAAGUGUUUGGGGGAACUC	CAGUUCACCCAAAAACUUCA	Cleavage
tae-miR395b	Traes_7BL_DC0D8773C.5	PF01909,PF04926,PF04928		2.7.7.19		GO:GO:0016779,GO:GO:0043631,GO:GO:0003723,GO:GO:0004652,GO:GO:0005634	AT4G32850.5	nuclear poly(a) polymerase	LOC_Os06g36360.2	expressed protein	2,50	-1.0	1	20	1385	1404	UGAAGUGUUUGGGGGAACUC	CAGUUCACCCAAAAACUUCA	Cleavage
tae-miR395b	Traes_7BL_DC0D8773C.6	PF04926,PF04928		2.7.7.19		GO:GO:0043631,GO:GO:0003723,GO:GO:0004652,GO:GO:0005634	AT4G32850.10	nuclear poly(a) polymerase	LOC_Os06g36360.1	expressed protein	2,50	-1.0	1	20	1148	1167	UGAAGUGUUUGGGGGAACUC	CAGUUCACCCAAAAACUUCA	Cleavage
tae-miR395b	Traes_7BL_DC0D8773C.7	PF04926,PF04928		2.7.7.19		GO:GO:0043631,GO:GO:0003723,GO:GO:0004652,GO:GO:0005634	AT4G32850.10	nuclear poly(a) polymerase	LOC_Os06g36360.2	expressed protein	2,50	-1.0	1	20	1148	1167	UGAAGUGUUUGGGGGAACUC	CAGUUCACCCAAAAACUUCA	Cleavage
tae-miR395b	Traes_7BL_DC0D8773C.8	PF04926,PF04928		2.7.7.19		GO:GO:0043631,GO:GO:0003723,GO:GO:0004652,GO:GO:0005634	AT4G32850.10	nuclear poly(a) polymerase	LOC_Os06g36360.1	expressed protein	2,50	-1.0	1	20	1148	1167	UGAAGUGUUUGGGGGAACUC	CAGUUCACCCAAAAACUUCA	Cleavage
tae-miR395b	Traes_7BL_DC0D8773C.9	PF04926,PF04928		2.7.7.19		GO:GO:0043631,GO:GO:0003723,GO:GO:0004652,GO:GO:0005634	AT4G32850.10	nuclear poly(a) polymerase	LOC_Os06g36360.1	expressed protein	2,50	-1.0	1	20	1148	1167	UGAAGUGUUUGGGGGAACUC	CAGUUCACCCAAAAACUUCA	Cleavage
tae-miR164	Traes_7BL_F1A0270C6.1								LOC_Os06g46270.1	no apical meristem protein, putative, expressed	1,00	-1.0	1	21	32	52	UGGAGAAGCAGGGCACGUGCA	AGCAAGUGCCCUGCUUCUCCA	Cleavage
tae-miR9666a-3p	Traes_7BL_F8D5E60B1.1	PF05001,PF05000,PF04983,PF04990,PF04992,PF00623,PF04997,PF04998		2.7.7.6	K03006	GO:GO:0006366,GO:GO:0005665,GO:GO:0003677,GO:GO:0006351,GO:GO:0003899	AT4G35800.1	RNA polymerase II large subunit	LOC_Os05g05860.1	retrotransposon protein, putative, unclassified, expressed	2,00	-1.0	1	22	5050	5071	CGGUAGGGCUGUAUGAUGGCGA	UCCCCCUCAUACAGCCCUACCU	Cleavage
tae-miR9666a-3p	Traes_7BL_F8D5E60B1.1	PF05001,PF05000,PF04983,PF04990,PF04992,PF00623,PF04997,PF04998		2.7.7.6	K03006	GO:GO:0006366,GO:GO:0005665,GO:GO:0003677,GO:GO:0006351,GO:GO:0003899	AT4G35800.1	RNA polymerase II large subunit	LOC_Os05g05860.1	retrotransposon protein, putative, unclassified, expressed	2,50	-1.0	1	22	4966	4987	CGGUAGGGCUGUAUGAUGGCGA	UCGCCAUCAUACAGCCCUACUU	Cleavage
tae-miR9666a-3p	Traes_7BL_F8D5E60B1.2	PF05001,PF05000,PF04983,PF04990,PF04992,PF00623,PF04997,PF04998		2.7.7.6	K03006	GO:GO:0006366,GO:GO:0005665,GO:GO:0003677,GO:GO:0006351,GO:GO:0003899	AT4G35800.1	RNA polymerase II large subunit	LOC_Os05g05860.1	retrotransposon protein, putative, unclassified, expressed	2,00	-1.0	1	22	5011	5032	CGGUAGGGCUGUAUGAUGGCGA	UCCCCCUCAUACAGCCCUACCU	Cleavage
tae-miR9666a-3p	Traes_7BL_F8D5E60B1.2	PF05001,PF05000,PF04983,PF04990,PF04992,PF00623,PF04997,PF04998		2.7.7.6	K03006	GO:GO:0006366,GO:GO:0005665,GO:GO:0003677,GO:GO:0006351,GO:GO:0003899	AT4G35800.1	RNA polymerase II large subunit	LOC_Os05g05860.1	retrotransposon protein, putative, unclassified, expressed	2,50	-1.0	1	22	4927	4948	CGGUAGGGCUGUAUGAUGGCGA	UCGCCAUCAUACAGCCCUACUU	Cleavage
tae-miR9666a-3p	Traes_7BL_F8D5E60B1.3	PF05001,PF05000,PF04983,PF04990,PF04997,PF04992,PF00623,PF04998		2.7.7.6		GO:GO:0006366,GO:GO:0005665,GO:GO:0003677,GO:GO:0006351,GO:GO:0003899	AT4G35800.1	RNA polymerase II large subunit	LOC_Os05g05860.1	retrotransposon protein, putative, unclassified, expressed	2,00	-1.0	1	22	4780	4801	CGGUAGGGCUGUAUGAUGGCGA	UCCCCCUCAUACAGCCCUACCU	Cleavage
tae-miR9666a-3p	Traes_7BL_F8D5E60B1.3	PF05001,PF05000,PF04983,PF04990,PF04997,PF04992,PF00623,PF04998		2.7.7.6		GO:GO:0006366,GO:GO:0005665,GO:GO:0003677,GO:GO:0006351,GO:GO:0003899	AT4G35800.1	RNA polymerase II large subunit	LOC_Os05g05860.1	retrotransposon protein, putative, unclassified, expressed	2,50	-1.0	1	22	4696	4717	CGGUAGGGCUGUAUGAUGGCGA	UCGCCAUCAUACAGCCCUACUU	Cleavage
tae-miR9666a-3p	Traes_7BL_F8D5E60B1.4	PF05001,PF05000,PF04983,PF04990,PF04992,PF04998		2.7.7.6		GO:GO:0006366,GO:GO:0005665,GO:GO:0003677,GO:GO:0006351,GO:GO:0003899	AT4G35800.1	RNA polymerase II large subunit	LOC_Os05g05860.1	retrotransposon protein, putative, unclassified, expressed	2,00	-1.0	1	22	3409	3430	CGGUAGGGCUGUAUGAUGGCGA	UCCCCCUCAUACAGCCCUACCU	Cleavage
tae-miR9666a-3p	Traes_7BL_F8D5E60B1.4	PF05001,PF05000,PF04983,PF04990,PF04992,PF04998		2.7.7.6		GO:GO:0006366,GO:GO:0005665,GO:GO:0003677,GO:GO:0006351,GO:GO:0003899	AT4G35800.1	RNA polymerase II large subunit	LOC_Os05g05860.1	retrotransposon protein, putative, unclassified, expressed	2,50	-1.0	1	22	3325	3346	CGGUAGGGCUGUAUGAUGGCGA	UCGCCAUCAUACAGCCCUACUU	Cleavage
tae-miR1137a	Traes_7BS_2A6C29F49.3	PF00566					AT3G49350.1	Ypt/Rab-GAP domain of gyp1p superfamily protein	LOC_Os09g35960.1	TBC domain containing protein, expressed	2,00	-1.0	1	20	75	94	UAGUACAAAGUUGAGUCAUC	AGUGUCUCAACUUUGUACUA	Cleavage
tae-miR1137a	Traes_7BS_2A6C29F49.4	PF00566					AT3G49350.1	Ypt/Rab-GAP domain of gyp1p superfamily protein	LOC_Os09g35960.1	TBC domain containing protein, expressed	2,00	-1.0	1	20	81	100	UAGUACAAAGUUGAGUCAUC	AGUGUCUCAACUUUGUACUA	Cleavage
tae-miR1130b-3p	Traes_7BS_47B8FC9CB.2	PF01154,PF08540		2.3.3.10		GO:GO:0008299,GO:GO:0004421	AT4G11820.2	hydroxymethylglutaryl-CoA synthase / HMG-CoA synthase / 3-hydroxy-3-methylglutaryl coenzyme A synthase	LOC_Os08g43170.1	hydroxymethylglutaryl-CoA synthase, putative, expressed	0,00	-1.0	1	21	1268	1288	UCUUAUAUUAUGGGACGGAGG	CCUCCGUCCCAUAAUAUAAGA	Cleavage
tae-miR1122c-3p	Traes_7BS_47B8FC9CB.2	PF01154,PF08540		2.3.3.10		GO:GO:0008299,GO:GO:0004421	AT4G11820.2	hydroxymethylglutaryl-CoA synthase / HMG-CoA synthase / 3-hydroxy-3-methylglutaryl coenzyme A synthase	LOC_Os08g43170.1	hydroxymethylglutaryl-CoA synthase, putative, expressed	1,50	-1.0	1	21	1268	1288	UCUAAUAUUAUGGGACGGAGG	CCUCCGUCCCAUAAUAUAAGA	Cleavage
tae-miR1120c-5p	Traes_7BS_47B8FC9CB.2	PF01154,PF08540		2.3.3.10		GO:GO:0008299,GO:GO:0004421	AT4G11820.2	hydroxymethylglutaryl-CoA synthase / HMG-CoA synthase / 3-hydroxy-3-methylglutaryl coenzyme A synthase	LOC_Os08g43170.1	hydroxymethylglutaryl-CoA synthase, putative, expressed	1,50	-1.0	1	21	1311	1331	UAAUAUAAGAACGUUUUUGAC	GUCAAAAACGCUCUUAUAUUA	Translation
tae-miR1130b-3p	Traes_7BS_47B8FC9CB.3	PF01154,PF08540		2.3.3.10	K01641	GO:GO:0008299,GO:GO:0004421	AT4G11820.2	hydroxymethylglutaryl-CoA synthase / HMG-CoA synthase / 3-hydroxy-3-methylglutaryl coenzyme A synthase	LOC_Os08g43170.1	hydroxymethylglutaryl-CoA synthase, putative, expressed	0,00	-1.0	1	21	1265	1285	UCUUAUAUUAUGGGACGGAGG	CCUCCGUCCCAUAAUAUAAGA	Cleavage
tae-miR1122c-3p	Traes_7BS_47B8FC9CB.3	PF01154,PF08540		2.3.3.10	K01641	GO:GO:0008299,GO:GO:0004421	AT4G11820.2	hydroxymethylglutaryl-CoA synthase / HMG-CoA synthase / 3-hydroxy-3-methylglutaryl coenzyme A synthase	LOC_Os08g43170.1	hydroxymethylglutaryl-CoA synthase, putative, expressed	1,50	-1.0	1	21	1265	1285	UCUAAUAUUAUGGGACGGAGG	CCUCCGUCCCAUAAUAUAAGA	Cleavage
tae-miR1120c-5p	Traes_7BS_47B8FC9CB.3	PF01154,PF08540		2.3.3.10	K01641	GO:GO:0008299,GO:GO:0004421	AT4G11820.2	hydroxymethylglutaryl-CoA synthase / HMG-CoA synthase / 3-hydroxy-3-methylglutaryl coenzyme A synthase	LOC_Os08g43170.1	hydroxymethylglutaryl-CoA synthase, putative, expressed	1,50	-1.0	1	21	1308	1328	UAAUAUAAGAACGUUUUUGAC	GUCAAAAACGCUCUUAUAUUA	Translation
tae-miR9662a-3p	Traes_7BS_598310C32.1	PF02536				GO:GO:0006355,GO:GO:0005739,GO:GO:0003690	AT1G21150.1	Mitochondrial transcription termination factor family protein	LOC_Os06g12100.1	mTERF family protein, expressed	2,50	-1.0	1	21	33	53	UUGAACAUCCCAGAGCCACCG	CCGUGGCUCUGCGAUGUUCAG	Translation
tae-miR9662b-3p	Traes_7BS_598310C32.1	PF02536				GO:GO:0006355,GO:GO:0005739,GO:GO:0003690	AT1G21150.1	Mitochondrial transcription termination factor family protein	LOC_Os06g12100.1	mTERF family protein, expressed	2,50	-1.0	1	21	32	52	UGAACAUCCCAGAGCCACCGG	CCCGUGGCUCUGCGAUGUUCA	Cleavage
tae-miR1121	Traes_7BS_5A0E77FB8.2	PF00664,PF00005		3.6.3.44		GO:GO:0055085,GO:GO:0042626,GO:GO:0016021,GO:GO:0006810,GO:GO:0005524,GO:GO:0016887	AT3G59140.1	multidrug resistance-associated protein 14	LOC_Os06g06440.1	ABC transporter, ATP-binding protein, putative, expressed	1,00	-1.0	1	22	3478	3499	AGUAGUGAUCUAAACGCUCUUA	UAAGAGUGUUUAGAUCACUACU	Cleavage
tae-miR1137a	Traes_7BS_6716D1D07.2	PF01105				GO:GO:0016021,GO:GO:0006810	AT1G09580.1	emp24/gp25L/p24 family/GOLD family protein	LOC_Os06g10760.1	emp24/gp25L/p24 family protein, putative, expressed	0,00	-1.0	1	20	347	366	UAGUACAAAGUUGAGUCAUC	GAUGACUCAACUUUGUACUA	Cleavage
tae-miR9781	Traes_7BS_A42F893FC.1	PF10150		3.1.26.12		GO:GO:0003723	AT2G04270.2	RNAse E/G-like	LOC_Os08g23430.1	starch binding domain containing protein, putative, expressed	1,50	-1.0	1	21	117	137	UUUUGUCACAUAUAAUACAUA	UAUGUAUUUUAUGUGACAAAA	Cleavage
tae-miR9664-3p	Traes_7DL_101A9F756.1	PF00931	KOG4658			GO:GO:0043531	AT1G58807.1	Disease resistance protein (CC-NBS-LRR class) family	LOC_Os11g11550.1	NBS-LRR disease resistance protein, putative, expressed	2,50	-1.0	1	21	102	122	UUGCAGUCCUCGAUGUCGUAG	GCACGACAUCGAGGAUUGCAU	Cleavage
tae-miR9664-3p	Traes_7DL_101A9F756.2	PF00931	KOG4658			GO:GO:0043531	AT1G58807.1	Disease resistance protein (CC-NBS-LRR class) family	LOC_Os11g11550.2	NBS-LRR disease resistance protein, putative, expressed	2,50	-1.0	1	21	102	122	UUGCAGUCCUCGAUGUCGUAG	GCACGACAUCGAGGAUUGCAU	Cleavage
tae-miR9664-3p	Traes_7DL_101A9F756.3	PF00931	KOG4658			GO:GO:0043531	AT1G58807.1	Disease resistance protein (CC-NBS-LRR class) family	LOC_Os11g11550.1	NBS-LRR disease resistance protein, putative, expressed	2,50	-1.0	1	21	231	251	UUGCAGUCCUCGAUGUCGUAG	GCACGACAUCGAGGAUUGCAU	Cleavage
tae-miR9666a-3p	Traes_7DL_18F2A8567.1	PF05001,PF05000,PF04983,PF04990,PF04992,PF00623,PF04997,PF04998		2.7.7.6	K03006	GO:GO:0006366,GO:GO:0005665,GO:GO:0003677,GO:GO:0006351,GO:GO:0003899	AT4G35800.1	RNA polymerase II large subunit	LOC_Os05g05860.1	retrotransposon protein, putative, unclassified, expressed	2,50	-1.0	1	22	4927	4948	CGGUAGGGCUGUAUGAUGGCGA	UCGCCAUCAUACAGCCCUACUU	Cleavage
tae-miR9666a-3p	Traes_7DL_18F2A8567.11	PF05001		2.7.7.6,2.7.7.48		GO:GO:0006366,GO:GO:0005665,GO:GO:0003677					2,00	-1.0	1	22	244	265	CGGUAGGGCUGUAUGAUGGCGA	UCGCCCUCAUACAGCCCUACCU	Cleavage
tae-miR9666a-3p	Traes_7DL_18F2A8567.11	PF05001		2.7.7.6,2.7.7.48		GO:GO:0006366,GO:GO:0005665,GO:GO:0003677					2,50	-1.0	1	22	139	160	CGGUAGGGCUGUAUGAUGGCGA	UCGCCAUCAUACAGCCCUACUU	Cleavage
tae-miR9666a-3p	Traes_7DL_18F2A8567.12	PF05001		2.7.7.6,2.7.7.48		GO:GO:0006366,GO:GO:0005665,GO:GO:0003677					2,00	-1.0	1	22	250	271	CGGUAGGGCUGUAUGAUGGCGA	UCGCCCUCAUACAGCCCUACCU	Cleavage
tae-miR9666a-3p	Traes_7DL_18F2A8567.12	PF05001		2.7.7.6,2.7.7.48		GO:GO:0006366,GO:GO:0005665,GO:GO:0003677					2,50	-1.0	1	22	145	166	CGGUAGGGCUGUAUGAUGGCGA	UCGCCAUCAUACAGCCCUACUU	Cleavage
tae-miR9666a-3p	Traes_7DL_18F2A8567.14										2,00	-1.0	1	22	201	222	CGGUAGGGCUGUAUGAUGGCGA	UCGCCCUCAUACAGCCCUACCU	Cleavage
tae-miR9666a-3p	Traes_7DL_18F2A8567.14										2,50	-1.0	1	22	96	117	CGGUAGGGCUGUAUGAUGGCGA	UCGCCAUCAUACAGCCCUACUU	Cleavage
tae-miR9666a-3p	Traes_7DL_18F2A8567.2	PF05001,PF05000,PF04983,PF04990,PF04992,PF00623,PF04997,PF04998		2.7.7.6	K03006	GO:GO:0006366,GO:GO:0005665,GO:GO:0003677,GO:GO:0006351,GO:GO:0003899	AT4G35800.1	RNA polymerase II large subunit	LOC_Os05g05860.1	retrotransposon protein, putative, unclassified, expressed	2,00	-1.0	1	22	5032	5053	CGGUAGGGCUGUAUGAUGGCGA	UCGCCCUCAUACAGCCCUACCU	Cleavage
tae-miR9666a-3p	Traes_7DL_18F2A8567.2	PF05001,PF05000,PF04983,PF04990,PF04992,PF00623,PF04997,PF04998		2.7.7.6	K03006	GO:GO:0006366,GO:GO:0005665,GO:GO:0003677,GO:GO:0006351,GO:GO:0003899	AT4G35800.1	RNA polymerase II large subunit	LOC_Os05g05860.1	retrotransposon protein, putative, unclassified, expressed	2,50	-1.0	1	22	4927	4948	CGGUAGGGCUGUAUGAUGGCGA	UCGCCAUCAUACAGCCCUACUU	Cleavage
tae-miR9666a-3p	Traes_7DL_18F2A8567.3	PF05001,PF05000,PF04983,PF04990,PF04992,PF00623,PF04997,PF04998		2.7.7.6	K03006	GO:GO:0006366,GO:GO:0005665,GO:GO:0003677,GO:GO:0006351,GO:GO:0003899	AT4G35800.1	RNA polymerase II large subunit	LOC_Os05g05860.1	retrotransposon protein, putative, unclassified, expressed	2,00	-1.0	1	22	4969	4990	CGGUAGGGCUGUAUGAUGGCGA	UCGCCCUCAUACAGCCCUACCU	Cleavage
tae-miR9666a-3p	Traes_7DL_18F2A8567.3	PF05001,PF05000,PF04983,PF04990,PF04992,PF00623,PF04997,PF04998		2.7.7.6	K03006	GO:GO:0006366,GO:GO:0005665,GO:GO:0003677,GO:GO:0006351,GO:GO:0003899	AT4G35800.1	RNA polymerase II large subunit	LOC_Os05g05860.1	retrotransposon protein, putative, unclassified, expressed	2,50	-1.0	1	22	4927	4948	CGGUAGGGCUGUAUGAUGGCGA	UCGCCAUCAUACAGCCCUACUU	Cleavage
tae-miR9666a-3p	Traes_7DL_18F2A8567.4	PF05001,PF05000,PF04983,PF04990,PF04992,PF00623,PF04997,PF04998		2.7.7.6	K03006	GO:GO:0006366,GO:GO:0005665,GO:GO:0003677,GO:GO:0006351,GO:GO:0003899	AT4G35800.1	RNA polymerase II large subunit	LOC_Os05g05860.1	retrotransposon protein, putative, unclassified, expressed	2,50	-1.0	1	22	4927	4948	CGGUAGGGCUGUAUGAUGGCGA	UCGCCAUCAUACAGCCCUACUU	Cleavage
tae-miR9666a-3p	Traes_7DL_18F2A8567.5	PF05001,PF05000,PF04983,PF04990,PF04992,PF00623,PF04997,PF04998		2.7.7.6	K03006	GO:GO:0006366,GO:GO:0005665,GO:GO:0003677,GO:GO:0006351,GO:GO:0003899	AT4G35800.1	RNA polymerase II large subunit	LOC_Os05g05860.1	retrotransposon protein, putative, unclassified, expressed	2,50	-1.0	1	22	4927	4948	CGGUAGGGCUGUAUGAUGGCGA	UCGCCAUCAUACAGCCCUACUU	Cleavage
tae-miR9666a-3p	Traes_7DL_18F2A8567.6	PF05001,PF05000,PF04983,PF04990,PF04992,PF00623,PF04997,PF04998		2.7.7.6	K03006	GO:GO:0006366,GO:GO:0005665,GO:GO:0003677,GO:GO:0006351,GO:GO:0003899	AT4G35800.1	RNA polymerase II large subunit	LOC_Os05g05860.1	retrotransposon protein, putative, unclassified, expressed	2,00	-1.0	1	22	5032	5053	CGGUAGGGCUGUAUGAUGGCGA	UCGCCCUCAUACAGCCCUACCU	Cleavage
tae-miR9666a-3p	Traes_7DL_18F2A8567.6	PF05001,PF05000,PF04983,PF04990,PF04992,PF00623,PF04997,PF04998		2.7.7.6	K03006	GO:GO:0006366,GO:GO:0005665,GO:GO:0003677,GO:GO:0006351,GO:GO:0003899	AT4G35800.1	RNA polymerase II large subunit	LOC_Os05g05860.1	retrotransposon protein, putative, unclassified, expressed	2,50	-1.0	1	22	4927	4948	CGGUAGGGCUGUAUGAUGGCGA	UCGCCAUCAUACAGCCCUACUU	Cleavage
tae-miR9666a-3p	Traes_7DL_18F2A8567.7	PF05001,PF05000,PF04983,PF04990,PF04997,PF04992,PF00623,PF04998		2.7.7.6	K03006	GO:GO:0006366,GO:GO:0005665,GO:GO:0003677,GO:GO:0006351,GO:GO:0003899	AT4G35800.1	RNA polymerase II large subunit	LOC_Os05g05860.1	retrotransposon protein, putative, unclassified, expressed	2,00	-1.0	1	22	4801	4822	CGGUAGGGCUGUAUGAUGGCGA	UCGCCCUCAUACAGCCCUACCU	Cleavage
tae-miR9666a-3p	Traes_7DL_18F2A8567.7	PF05001,PF05000,PF04983,PF04990,PF04997,PF04992,PF00623,PF04998		2.7.7.6	K03006	GO:GO:0006366,GO:GO:0005665,GO:GO:0003677,GO:GO:0006351,GO:GO:0003899	AT4G35800.1	RNA polymerase II large subunit	LOC_Os05g05860.1	retrotransposon protein, putative, unclassified, expressed	2,50	-1.0	1	22	4696	4717	CGGUAGGGCUGUAUGAUGGCGA	UCGCCAUCAUACAGCCCUACUU	Cleavage
tae-miR9666a-3p	Traes_7DL_18F2A8567.8	PF05001,PF05000,PF04983,PF04990,PF04992,PF04998		2.7.7.6		GO:GO:0006366,GO:GO:0005665,GO:GO:0003677,GO:GO:0006351,GO:GO:0003899	AT4G35800.1	RNA polymerase II large subunit	LOC_Os05g05860.1	retrotransposon protein, putative, unclassified, expressed	2,00	-1.0	1	22	3409	3430	CGGUAGGGCUGUAUGAUGGCGA	UCGCCCUCAUACAGCCCUACCU	Cleavage
tae-miR9666a-3p	Traes_7DL_18F2A8567.8	PF05001,PF05000,PF04983,PF04990,PF04992,PF04998		2.7.7.6		GO:GO:0006366,GO:GO:0005665,GO:GO:0003677,GO:GO:0006351,GO:GO:0003899	AT4G35800.1	RNA polymerase II large subunit	LOC_Os05g05860.1	retrotransposon protein, putative, unclassified, expressed	2,50	-1.0	1	22	3304	3325	CGGUAGGGCUGUAUGAUGGCGA	UCGCCAUCAUACAGCCCUACUU	Cleavage
tae-miR9666a-3p	Traes_7DL_18F2A8567.9	PF05001,PF05000,PF04990,PF04992,PF04998		2.7.7.6		GO:GO:0006366,GO:GO:0005665,GO:GO:0003677,GO:GO:0006351,GO:GO:0003899	AT4G35800.1	RNA polymerase II large subunit	LOC_Os05g05860.1	retrotransposon protein, putative, unclassified, expressed	2,00	-1.0	1	22	2941	2962	CGGUAGGGCUGUAUGAUGGCGA	UCGCCCUCAUACAGCCCUACCU	Cleavage
tae-miR9666a-3p	Traes_7DL_18F2A8567.9	PF05001,PF05000,PF04990,PF04992,PF04998		2.7.7.6		GO:GO:0006366,GO:GO:0005665,GO:GO:0003677,GO:GO:0006351,GO:GO:0003899	AT4G35800.1	RNA polymerase II large subunit	LOC_Os05g05860.1	retrotransposon protein, putative, unclassified, expressed	2,50	-1.0	1	22	2836	2857	CGGUAGGGCUGUAUGAUGGCGA	UCGCCAUCAUACAGCCCUACUU	Cleavage
tae-miR164	Traes_7DL_1E343B6E6.1						AT1G56010.2	NAC domain containing protein 1	LOC_Os06g46270.1	no apical meristem protein, putative, expressed	1,00	-1.0	1	21	236	256	UGGAGAAGCAGGGCACGUGCA	AGCAAGUGCCCUGCUUCUCCA	Cleavage
tae-miR164	Traes_7DL_1E343B6E6.2								LOC_Os06g46270.1	no apical meristem protein, putative, expressed	1,00	-1.0	1	21	32	52	UGGAGAAGCAGGGCACGUGCA	AGCAAGUGCCCUGCUUCUCCA	Cleavage
tae-miR1135	Traes_7DL_20BFD8A19.4	PF00145		2.1.1.37			AT4G19020.1	chromomethylase 2	LOC_Os10g01570.1	C-5 cytosine-specific DNA methylase, putative, expressed	1,50	-1.0	1	24	954	977	CUGCGACAAGUAAUUCCGAACGGA	UCUGUUCGGAAUUACUUGUCGUAG	Cleavage
tae-miR1127a	Traes_7DL_20BFD8A19.4	PF00145		2.1.1.37			AT4G19020.1	chromomethylase 2	LOC_Os10g01570.1	C-5 cytosine-specific DNA methylase, putative, expressed	1,50	-1.0	1	19	1032	1050	UCCUUCCGUUCGGAAUUAC	GUAAUUCCGAACGGAGGGA	Cleavage
tae-miR9778	Traes_7DL_31A23053A.1								LOC_Os11g39600.1	resistance protein, putative, expressed	1,00	-1.0	1	21	132	152	UGCAUCAUCUCGAACUCGUCG	CCUCGAGUUCGAGAUGAUGCA	Cleavage
tae-miR1122a	Traes_7DL_37135CE6E.2	PF00076	KOG0128			GO:GO:0003676	AT4G24270.2	EMBRYO DEFECTIVE 140	LOC_Os08g02130.2	RNA recognition motif containing protein, putative, expressed	2,50	-1.0	1	20	2105	2124	UAGAUACAUCCGUAUCUAGA	UCUAAAUACGGAUGUAUCAA	Cleavage
tae-miR1122a	Traes_7DL_37135CE6E.3	PF00076	KOG0128			GO:GO:0003676	AT4G24270.2	EMBRYO DEFECTIVE 140	LOC_Os08g02130.2	RNA recognition motif containing protein, putative, expressed	2,50	-1.0	1	20	2090	2109	UAGAUACAUCCGUAUCUAGA	UCUAAAUACGGAUGUAUCAA	Cleavage
tae-miR1122a	Traes_7DL_37135CE6E.6	PF00076	KOG0130			GO:GO:0003676	AT4G24270.2	EMBRYO DEFECTIVE 140	LOC_Os08g02130.1	RNA recognition motif containing protein, putative, expressed	2,50	-1.0	1	20	1241	1260	UAGAUACAUCCGUAUCUAGA	UCUAAAUACGGAUGUAUCAA	Cleavage
tae-miR9661-5p	Traes_7DL_55AC0BE09.1										2,50	-1.0	1	21	143	163	UGAAGUAGAGCAGGGACCUCA	UCAGGUCCCUGCGCUACUUCG	Cleavage
tae-miR9661-5p	Traes_7DL_55AC0BE09.2										2,50	-1.0	1	21	143	163	UGAAGUAGAGCAGGGACCUCA	UCAGGUCCCUGCGCUACUUCG	Cleavage
tae-miR9661-5p	Traes_7DL_55AC0BE09.3										2,50	-1.0	1	21	116	136	UGAAGUAGAGCAGGGACCUCA	UCAGGUCCCUGCGCUACUUCG	Cleavage
tae-miR9661-5p	Traes_7DL_55AC0BE09.4										2,50	-1.0	1	21	116	136	UGAAGUAGAGCAGGGACCUCA	UCAGGUCCCUGCGCUACUUCG	Cleavage
tae-miR9661-5p	Traes_7DL_55AC0BE09.5										2,50	-1.0	1	21	116	136	UGAAGUAGAGCAGGGACCUCA	UCAGGUCCCUGCGCUACUUCG	Cleavage
tae-miR160	Traes_7DL_55ADB3528.1	PF06507				GO:GO:0009725,GO:GO:0006355,GO:GO:0005634,GO:GO:0003677	AT4G30080.1	auxin response factor 16	LOC_Os06g47150.1	auxin response factor 18, putative, expressed	0,00	-1.0	1	21	714	734	UGCCUGGCUCCCUGUAUGCCA	AGGCAUACAGGGAGCCAGGCA	Cleavage
tae-miR160	Traes_7DL_55ADB3528.2	PF06507				GO:GO:0009725,GO:GO:0006355,GO:GO:0005634,GO:GO:0003677	AT4G30080.1	auxin response factor 16	LOC_Os06g47150.3	auxin response factor 18, putative, expressed	0,00	-1.0	1	21	714	734	UGCCUGGCUCCCUGUAUGCCA	AGGCAUACAGGGAGCCAGGCA	Cleavage
tae-miR1134	Traes_7DL_5E2559796.1										2,00	-1.0	1	24	2562	2585	CAACAACAACAAGAAGAAGAAGAU	UUGUUGUUGUUCUUGUUGUUGUUG	Cleavage
tae-miR1134	Traes_7DL_5E2559796.2										2,00	-1.0	1	24	2556	2579	CAACAACAACAAGAAGAAGAAGAU	UUGUUGUUGUUCUUGUUGUUGUUG	Cleavage
tae-miR1122b-3p	Traes_7DL_613007B24.2	PF00400	KOG0279,KOG1332			GO:GO:0005515	AT1G15750.3	Transducin family protein / WD-40 repeat family protein	LOC_Os08g06480.1	lissencephaly type-1-like homology motif, putative, expressed	1,50	-1.0	1	21	1492	1512	AGACUUAUAUGUAGGAACGGA	UCCGUUCCUAAAUAUAAGUCU	Translation
tae-miR164	Traes_7DL_91CFE828F.1	PF02365				GO:GO:0006355,GO:GO:0003677	AT1G56010.2	NAC domain containing protein 1	LOC_Os06g46270.1	no apical meristem protein, putative, expressed	1,00	-1.0	1	21	497	517	UGGAGAAGCAGGGCACGUGCA	AGCAAGUGCCCUGCUUCUCCA	Cleavage
tae-miR164	Traes_7DL_91CFE828F.2						AT1G56010.2	NAC domain containing protein 1	LOC_Os06g46270.1	no apical meristem protein, putative, expressed	1,00	-1.0	1	21	296	316	UGGAGAAGCAGGGCACGUGCA	AGCAAGUGCCCUGCUUCUCCA	Cleavage
tae-miR9778	Traes_7DL_9F0AD190A.2	PF00931	KOG4658			GO:GO:0043531	AT1G58400.1	Disease resistance protein (CC-NBS-LRR class) family	LOC_Os08g14850.1	resistance protein, putative, expressed	2,50	-1.0	1	21	126	146	UGCAUCAUCUCGAACUCGUCG	AGGCGAGUUUGAGAUGAUGCA	Cleavage
tae-miR9778	Traes_7DL_9F0AD190A.3	PF00931	KOG4658			GO:GO:0043531	AT1G58400.1	Disease resistance protein (CC-NBS-LRR class) family	LOC_Os08g14850.1	resistance protein, putative, expressed	2,50	-1.0	1	21	126	146	UGCAUCAUCUCGAACUCGUCG	AGGCGAGUUUGAGAUGAUGCA	Cleavage
tae-miR5048-5p	Traes_7DL_AB7C59D49.1	PF00069		2.7.11.1		GO:GO:0006468,GO:GO:0005524,GO:GO:0004672	AT4G21230.1	cysteine-rich RLK (RECEPTOR-like protein kinase) 27	LOC_Os11g12270.1	expressed protein	0,00	-1.0	1	22	53	74	UUUGCAGGUUUUAGGUCUAAGU	ACUUAGACCUAAAACCUGCAAA	Cleavage
tae-miR5048-5p	Traes_7DL_AB7C59D49.2	PF00069		2.7.11.1		GO:GO:0006468,GO:GO:0005524,GO:GO:0004672	AT1G11300.1	protein serine/threonine kinases;protein kinases;ATP binding;sugar binding;kinases;carbohydrate binding	LOC_Os11g17380.1	protein kinase domain containing protein, expressed	0,00	-1.0	1	22	53	74	UUUGCAGGUUUUAGGUCUAAGU	ACUUAGACCUAAAACCUGCAAA	Cleavage
tae-miR5048-5p	Traes_7DL_AB7C59D49.3	PF00069		2.7.11.1		GO:GO:0006468,GO:GO:0005524,GO:GO:0004672	AT1G11300.1	protein serine/threonine kinases;protein kinases;ATP binding;sugar binding;kinases;carbohydrate binding	LOC_Os11g17380.1	protein kinase domain containing protein, expressed	0,00	-1.0	1	22	53	74	UUUGCAGGUUUUAGGUCUAAGU	ACUUAGACCUAAAACCUGCAAA	Cleavage
tae-miR5048-5p	Traes_7DL_AB7C59D49.4	PF00069		2.7.11.1		GO:GO:0006468,GO:GO:0005524,GO:GO:0004672	AT1G11300.1	protein serine/threonine kinases;protein kinases;ATP binding;sugar binding;kinases;carbohydrate binding	LOC_Os11g17380.1	protein kinase domain containing protein, expressed	0,00	-1.0	1	22	53	74	UUUGCAGGUUUUAGGUCUAAGU	ACUUAGACCUAAAACCUGCAAA	Cleavage
tae-miR5048-5p	Traes_7DL_AB7C59D49.5	PF00069		2.7.11.1		GO:GO:0006468,GO:GO:0005524,GO:GO:0004672	AT4G21230.1	cysteine-rich RLK (RECEPTOR-like protein kinase) 27	LOC_Os11g12270.2	expressed protein	0,00	-1.0	1	22	53	74	UUUGCAGGUUUUAGGUCUAAGU	ACUUAGACCUAAAACCUGCAAA	Cleavage
tae-miR5048-5p	Traes_7DL_AB7C59D49.6	PF00069		2.7.11.1		GO:GO:0006468,GO:GO:0005524,GO:GO:0004672	AT4G21230.1	cysteine-rich RLK (RECEPTOR-like protein kinase) 27	LOC_Os11g12270.2	expressed protein	0,00	-1.0	1	22	53	74	UUUGCAGGUUUUAGGUCUAAGU	ACUUAGACCUAAAACCUGCAAA	Cleavage
tae-miR1122a	Traes_7DL_C074DC1F6.3	PF00141		1.11.1.7		GO:GO:0055114,GO:GO:0020037,GO:GO:0006979,GO:GO:0004601	AT5G64120.1	Peroxidase superfamily protein	LOC_Os07g44550.1	peroxidase precursor, putative, expressed	2,50	-1.0	1	20	109	128	UAGAUACAUCCGUAUCUAGA	ACUACAUACGGAUGUAUAUA	Cleavage
tae-miR395b	Traes_7DL_CB073ADC6.1	PF01909,PF04926,PF04928		2.7.7.19	K14376	GO:GO:0016779,GO:GO:0043631,GO:GO:0003723,GO:GO:0004652,GO:GO:0005634	AT4G32850.5	nuclear poly(a) polymerase	LOC_Os06g36360.3	expressed protein	2,50	-1.0	1	20	1598	1617	UGAAGUGUUUGGGGGAACUC	CAGUUCACCCAAAAACUUCA	Cleavage
tae-miR395b	Traes_7DL_CB073ADC6.10	PF04926,PF04928		2.7.7.19		GO:GO:0043631,GO:GO:0003723,GO:GO:0004652,GO:GO:0005634	AT4G32850.9	nuclear poly(a) polymerase	LOC_Os06g36360.1	expressed protein	2,50	-1.0	1	20	851	870	UGAAGUGUUUGGGGGAACUC	CAGUUCACCCAAAAACUUCA	Cleavage
tae-miR395b	Traes_7DL_CB073ADC6.11	PF04926,PF04928		2.7.7.19		GO:GO:0043631,GO:GO:0003723,GO:GO:0004652,GO:GO:0005634	AT4G32850.5	nuclear poly(a) polymerase	LOC_Os06g36360.2	expressed protein	2,50	-1.0	1	20	851	870	UGAAGUGUUUGGGGGAACUC	CAGUUCACCCAAAAACUUCA	Cleavage
tae-miR395b	Traes_7DL_CB073ADC6.12	PF04926,PF04928		2.7.7.19		GO:GO:0043631,GO:GO:0003723,GO:GO:0004652,GO:GO:0005634	AT4G32850.9	nuclear poly(a) polymerase	LOC_Os06g36360.2	expressed protein	2,50	-1.0	1	20	851	870	UGAAGUGUUUGGGGGAACUC	CAGUUCACCCAAAAACUUCA	Cleavage
tae-miR395b	Traes_7DL_CB073ADC6.2	PF01909,PF04926,PF04928		2.7.7.19	K14376	GO:GO:0016779,GO:GO:0043631,GO:GO:0003723,GO:GO:0004652,GO:GO:0005634	AT4G32850.5	nuclear poly(a) polymerase	LOC_Os06g36360.2	expressed protein	2,50	-1.0	1	20	1598	1617	UGAAGUGUUUGGGGGAACUC	CAGUUCACCCAAAAACUUCA	Cleavage
tae-miR395b	Traes_7DL_CB073ADC6.3	PF01909,PF04926,PF04928		2.7.7.19	K14376	GO:GO:0016779,GO:GO:0043631,GO:GO:0003723,GO:GO:0004652,GO:GO:0005634	AT4G32850.9	nuclear poly(a) polymerase	LOC_Os06g36360.1	expressed protein	2,50	-1.0	1	20	1598	1617	UGAAGUGUUUGGGGGAACUC	CAGUUCACCCAAAAACUUCA	Cleavage
tae-miR395b	Traes_7DL_CB073ADC6.4	PF04926,PF04928		2.7.7.19		GO:GO:0043631,GO:GO:0003723,GO:GO:0004652,GO:GO:0005634	AT4G32850.10	nuclear poly(a) polymerase	LOC_Os06g36360.3	expressed protein	2,50	-1.0	1	20	1157	1176	UGAAGUGUUUGGGGGAACUC	CAGUUCACCCAAAAACUUCA	Cleavage
tae-miR395b	Traes_7DL_CB073ADC6.5	PF04926,PF04928		2.7.7.19		GO:GO:0043631,GO:GO:0003723,GO:GO:0004652,GO:GO:0005634	AT4G32850.10	nuclear poly(a) polymerase	LOC_Os06g36360.1	expressed protein	2,50	-1.0	1	20	1157	1176	UGAAGUGUUUGGGGGAACUC	CAGUUCACCCAAAAACUUCA	Cleavage
tae-miR395b	Traes_7DL_CB073ADC6.6	PF04926,PF04928		2.7.7.19		GO:GO:0043631,GO:GO:0003723,GO:GO:0004652,GO:GO:0005634	AT4G32850.10	nuclear poly(a) polymerase	LOC_Os06g36360.3	expressed protein	2,50	-1.0	1	20	1157	1176	UGAAGUGUUUGGGGGAACUC	CAGUUCACCCAAAAACUUCA	Cleavage
tae-miR395b	Traes_7DL_CB073ADC6.7	PF04926,PF04928		2.7.7.19		GO:GO:0043631,GO:GO:0003723,GO:GO:0004652,GO:GO:0005634	AT4G32850.10	nuclear poly(a) polymerase	LOC_Os06g36360.2	expressed protein	2,50	-1.0	1	20	1157	1176	UGAAGUGUUUGGGGGAACUC	CAGUUCACCCAAAAACUUCA	Cleavage
tae-miR395b	Traes_7DL_CB073ADC6.8	PF04926,PF04928		2.7.7.19		GO:GO:0043631,GO:GO:0003723,GO:GO:0004652,GO:GO:0005634	AT4G32850.10	nuclear poly(a) polymerase	LOC_Os06g36360.2	expressed protein	2,50	-1.0	1	20	1079	1098	UGAAGUGUUUGGGGGAACUC	CAGUUCACCCAAAAACUUCA	Cleavage
tae-miR395b	Traes_7DL_CB073ADC6.9	PF04926,PF04928		2.7.7.19		GO:GO:0043631,GO:GO:0003723,GO:GO:0004652,GO:GO:0005634	AT4G32850.9	nuclear poly(a) polymerase	LOC_Os06g36360.4	expressed protein	2,50	-1.0	1	20	851	870	UGAAGUGUUUGGGGGAACUC	CAGUUCACCCAAAAACUUCA	Cleavage
tae-miR1130b-3p	Traes_7DL_CF6AABA1F.2						AT4G31430.3		LOC_Os06g40460.1	expressed protein	0,00	-1.0	1	21	1271	1291	UCUUAUAUUAUGGGACGGAGG	CCUCCGUCCCAUAAUAUAAGA	Cleavage
tae-miR1122c-3p	Traes_7DL_CF6AABA1F.2						AT4G31430.3		LOC_Os06g40460.1	expressed protein	1,50	-1.0	1	21	1271	1291	UCUAAUAUUAUGGGACGGAGG	CCUCCGUCCCAUAAUAUAAGA	Cleavage
tae-miR1128	Traes_7DL_D5FCF9F33.4								LOC_Os06g46480.1	expressed protein	2,50	-1.0	1	21	96	116	UACUACUCCCUCCGUCCGAAA	UUUGGGACGGAGGGAGUAUUA	Cleavage
tae-miR1122a	Traes_7DL_D5FCF9F33.4								LOC_Os06g46480.1	expressed protein	0,00	-1.0	1	20	23	42	UAGAUACAUCCGUAUCUAGA	UCUAGAUACGGAUGUAUCUA	Cleavage
tae-miR5049-3p	Traes_7DS_06CD5BBFB.4						AT3G21350.2	RNA polymerase transcriptional regulation mediator-related	LOC_Os06g11370.1	mediator of RNA polymerase II transcription subunit 6, putative, expressed	0,00	-1.0	1	21	99	119	AAUAUGGAUCGGAGGGAGUAC	UUACUCCCUCCGAUCCAUAUU	Cleavage
tae-miR1134	Traes_7DS_205267E87.1										2,50	-1.0	1	24	92	115	CAACAACAACAAGAAGAAGAAGAU	CUGUUCUUCUUCUUGUUGUUGUCU	Cleavage
tae-miR1130b-3p	Traes_7DS_25B02BA46.5	PF00005		3.6.3.44		GO:GO:0016887,GO:GO:0005524	AT3G53480.1	pleiotropic drug resistance 9	LOC_Os02g32690.1	pleiotropic drug resistance protein 15, putative, expressed	1,50	-1.0	1	21	730	750	UCUUAUAUUAUGGGACGGAGG	CCUCCGUCCCAUAAUGUAAGA	Cleavage
tae-miR1133	Traes_7DS_25F082B1E.8										3,00	-1.0	1	22	19	41	CAUAUACUCCCUCCGUCC-GAAA	UUUCCGGACGGAGGGAGUAUAUA	Cleavage
tae-miR9662a-3p	Traes_7DS_2B1FE04A6.1	PF02536				GO:GO:0006355,GO:GO:0005739,GO:GO:0003690	AT5G07900.1	Mitochondrial transcription termination factor family protein	LOC_Os06g12100.1	mTERF family protein, expressed	2,50	-1.0	1	21	561	581	UUGAACAUCCCAGAGCCACCG	CCGUGGCUCUGCGAUGUUCAG	Translation
tae-miR9662b-3p	Traes_7DS_2B1FE04A6.1	PF02536				GO:GO:0006355,GO:GO:0005739,GO:GO:0003690	AT5G07900.1	Mitochondrial transcription termination factor family protein	LOC_Os06g12100.1	mTERF family protein, expressed	2,50	-1.0	1	21	560	580	UGAACAUCCCAGAGCCACCGG	CCCGUGGCUCUGCGAUGUUCA	Cleavage
tae-miR1130b-3p	Traes_7DS_3C83C8EF9.1						AT4G31340.1	myosin heavy chain-related	LOC_Os08g40790.1	DNA repair ATPase-related, putative, expressed	1,00	-1.0	1	21	37	57	UCUUAUAUUAUGGGACGGAGG	CCUCCGUUCCAUAAUAUAAGA	Cleavage
tae-miR1122c-3p	Traes_7DS_3C83C8EF9.1						AT4G31340.1	myosin heavy chain-related	LOC_Os08g40790.1	DNA repair ATPase-related, putative, expressed	2,50	-1.0	1	21	37	57	UCUAAUAUUAUGGGACGGAGG	CCUCCGUUCCAUAAUAUAAGA	Cleavage
tae-miR1120c-5p	Traes_7DS_3C83C8EF9.1						AT4G31340.1	myosin heavy chain-related	LOC_Os08g40790.1	DNA repair ATPase-related, putative, expressed	1,50	-1.0	1	21	80	100	UAAUAUAAGAACGUUUUUGAC	GUCAAAAACGCUCUUAUAUUA	Translation
tae-miR1130b-3p	Traes_7DS_3C83C8EF9.3						AT4G31340.1	myosin heavy chain-related	LOC_Os08g40790.3	DNA repair ATPase-related, putative, expressed	1,00	-1.0	1	21	37	57	UCUUAUAUUAUGGGACGGAGG	CCUCCGUUCCAUAAUAUAAGA	Cleavage
tae-miR1122c-3p	Traes_7DS_3C83C8EF9.3						AT4G31340.1	myosin heavy chain-related	LOC_Os08g40790.3	DNA repair ATPase-related, putative, expressed	2,50	-1.0	1	21	37	57	UCUAAUAUUAUGGGACGGAGG	CCUCCGUUCCAUAAUAUAAGA	Cleavage
tae-miR1120c-5p	Traes_7DS_3C83C8EF9.3						AT4G31340.1	myosin heavy chain-related	LOC_Os08g40790.3	DNA repair ATPase-related, putative, expressed	1,50	-1.0	1	21	80	100	UAAUAUAAGAACGUUUUUGAC	GUCAAAAACGCUCUUAUAUUA	Translation
tae-miR9662a-3p	Traes_7DS_5B1210681.1	PF02536			K15032	GO:GO:0006355,GO:GO:0005739,GO:GO:0003690	AT1G21150.1	Mitochondrial transcription termination factor family protein	LOC_Os06g12100.1	mTERF family protein, expressed	2,50	-1.0	1	21	285	305	UUGAACAUCCCAGAGCCACCG	CCGUGGCUCUGCGAUGUUCAG	Translation
tae-miR9662b-3p	Traes_7DS_5B1210681.1	PF02536			K15032	GO:GO:0006355,GO:GO:0005739,GO:GO:0003690	AT1G21150.1	Mitochondrial transcription termination factor family protein	LOC_Os06g12100.1	mTERF family protein, expressed	2,50	-1.0	1	21	284	304	UGAACAUCCCAGAGCCACCGG	CCCGUGGCUCUGCGAUGUUCA	Cleavage
tae-miR9778	Traes_7DS_74534FCD3.1	PF00931				GO:GO:0043531	AT1G50180.1	NB-ARC domain-containing disease resistance protein	LOC_Os10g03570.1	RGH1A, putative, expressed	2,50	-1.0	1	21	111	131	UGCAUCAUCUCGAACUCGUCG	AGACGAGCUGGAGAUGAUGCA	Cleavage
tae-miR9778	Traes_7DS_74534FCD3.2	PF00931				GO:GO:0043531	AT3G46530.1	NB-ARC domain-containing disease resistance protein	LOC_Os10g03570.1	RGH1A, putative, expressed	2,50	-1.0	1	21	111	131	UGCAUCAUCUCGAACUCGUCG	AGACGAGCUGGAGAUGAUGCA	Cleavage
tae-miR9778	Traes_7DS_74534FCD3.3	PF00931				GO:GO:0043531	AT3G46530.1	NB-ARC domain-containing disease resistance protein	LOC_Os10g03570.1	RGH1A, putative, expressed	2,50	-1.0	1	21	111	131	UGCAUCAUCUCGAACUCGUCG	AGACGAGCUGGAGAUGAUGCA	Cleavage
tae-miR9778	Traes_7DS_74534FCD3.4	PF00931				GO:GO:0043531	AT3G46530.1	NB-ARC domain-containing disease resistance protein	LOC_Os10g03570.1	RGH1A, putative, expressed	2,50	-1.0	1	21	111	131	UGCAUCAUCUCGAACUCGUCG	AGACGAGCUGGAGAUGAUGCA	Cleavage
tae-miR9778	Traes_7DS_74534FCD3.5	PF00931				GO:GO:0043531	AT3G46530.1	NB-ARC domain-containing disease resistance protein	LOC_Os10g03570.1	RGH1A, putative, expressed	2,50	-1.0	1	21	111	131	UGCAUCAUCUCGAACUCGUCG	AGACGAGCUGGAGAUGAUGCA	Cleavage
tae-miR9778	Traes_7DS_74534FCD3.6	PF00931				GO:GO:0043531	AT3G46530.1	NB-ARC domain-containing disease resistance protein	LOC_Os10g03570.1	RGH1A, putative, expressed	2,50	-1.0	1	21	111	131	UGCAUCAUCUCGAACUCGUCG	AGACGAGCUGGAGAUGAUGCA	Cleavage
tae-miR1133	Traes_7DS_98F9E4E55.2	PF16211,PF00125	KOG1757			GO:GO:0003677	AT3G54560.1	histone H2A 11	LOC_Os03g53190.2	Core histone H2A/H2B/H3/H4 domain containing protein, putative, expressed	2,00	-1.0	1	22	2	24	CAUAUACUCCCUCCGUCCGA-AA	UUAUGGGACGGAGGGAGUAUAUA	Cleavage
tae-miR1122a	Traes_7DS_CA61ECF35.1						AT1G05060.1		LOC_Os06g13220.1	expressed protein	0,00	-1.0	1	20	33	52	UAGAUACAUCCGUAUCUAGA	UCUAGAUACGGAUGUAUCUA	Cleavage
tae-miR9775	Traes_7DS_D4DFDF981.1										2,00	-1.0	1	22	1609	1630	UGUGCGCAAUAAGAUUUUGCUA	GUGCAUGAUCUUAUUGCGCACA	Cleavage
tae-miR9775	Traes_7DS_D4DFDF981.2										2,00	-1.0	1	22	1702	1723	UGUGCGCAAUAAGAUUUUGCUA	GUGCAUGAUCUUAUUGCGCACA	Cleavage
tae-miR156	Traes_7DS_DBB29C7CD.1								LOC_Os08g41940.1	OsSPL16 - SBP-box gene family member, expressed	1,00	-1.0	1	21	359	379	UGACAGAAGAGAGUGAGCACA	UGUGCUCUCUCUCUUCUGUCA	Cleavage
tae-miR5049-3p	Traes_7DS_EFB144315.5	PF00076,PF04059	KOG4660			GO:GO:0003676	AT1G29400.2	MEI2-like protein 5	LOC_Os02g48790.5	AML1, putative, expressed	1,50	-1.0	1	21	2298	2318	AAUAUGGAUCGGAGGGAGUAC	UUACUCCCUCUGAUCCAUAUU	Translation

Triticum aestivum Stress Root

miRNA_Acc.	Target_Acc.	Pfam	KOG	KEGG/ec	KO	GO	Best-hit-arabi-name	arabi-defline	Best-hit-rice-name	rice-defline	Expectation	UPE$	miRNA_start	miRNA_end	Target_start	Target_end	miRNA_aligned_fragment	Target_aligned_fragment	Inhibition
tae-miR159b	Traes_1AL_041EBB4A6.1						AT2G32460.1	myb domain protein 101	LOC_Os01g59660.2	MYB family transcription factor, putative, expressed	2,50	-1.0	1	21	488	508	UUUGGAUUGAAGGGAGCUCUG	UGGAGCUCCCUUCAUUCCAAU	Cleavage
tae-miR159a	Traes_1AL_041EBB4A6.1						AT2G32460.1	myb domain protein 101	LOC_Os01g59660.2	MYB family transcription factor, putative, expressed	2,50	-1.0	1	21	488	508	UUUGGAUUGAAGGGAGCUCUG	UGGAGCUCCCUUCAUUCCAAU	Cleavage
tae-miR159b	Traes_1AL_041EBB4A6.2						AT2G32460.1	myb domain protein 101	LOC_Os01g59660.2	MYB family transcription factor, putative, expressed	2,50	-1.0	1	21	512	532	UUUGGAUUGAAGGGAGCUCUG	UGGAGCUCCCUUCAUUCCAAU	Cleavage
tae-miR159a	Traes_1AL_041EBB4A6.2						AT2G32460.1	myb domain protein 101	LOC_Os01g59660.2	MYB family transcription factor, putative, expressed	2,50	-1.0	1	21	512	532	UUUGGAUUGAAGGGAGCUCUG	UGGAGCUCCCUUCAUUCCAAU	Cleavage
tae-miR159b	Traes_1AL_041EBB4A6.3						AT2G32460.1	myb domain protein 101	LOC_Os01g59660.2	MYB family transcription factor, putative, expressed	2,50	-1.0	1	21	488	508	UUUGGAUUGAAGGGAGCUCUG	UGGAGCUCCCUUCAUUCCAAU	Cleavage
tae-miR159a	Traes_1AL_041EBB4A6.3						AT2G32460.1	myb domain protein 101	LOC_Os01g59660.2	MYB family transcription factor, putative, expressed	2,50	-1.0	1	21	488	508	UUUGGAUUGAAGGGAGCUCUG	UGGAGCUCCCUUCAUUCCAAU	Cleavage
tae-miR159b	Traes_1AL_041EBB4A6.4								LOC_Os01g59660.2	MYB family transcription factor, putative, expressed	2,50	-1.0	1	21	410	430	UUUGGAUUGAAGGGAGCUCUG	UGGAGCUCCCUUCAUUCCAAU	Cleavage
tae-miR159a	Traes_1AL_041EBB4A6.4								LOC_Os01g59660.2	MYB family transcription factor, putative, expressed	2,50	-1.0	1	21	410	430	UUUGGAUUGAAGGGAGCUCUG	UGGAGCUCCCUUCAUUCCAAU	Cleavage
tae-miR160	Traes_1AL_147CF243C.1	PF06507				GO:GO:0009725,GO:GO:0006355,GO:GO:0005634,GO:GO:0003677	AT4G30080.1	auxin response factor 16	LOC_Os10g33940.1	auxin response factor 18, putative, expressed	0,00	-1.0	1	21	642	662	UGCCUGGCUCCCUGUAUGCCA	AGGCAUACAGGGAGCCAGGCA	Cleavage
tae-miR160	Traes_1AL_147CF243C.2						AT4G30080.1	auxin response factor 16	LOC_Os10g33940.1	auxin response factor 18, putative, expressed	0,00	-1.0	1	21	267	287	UGCCUGGCUCCCUGUAUGCCA	AGGCAUACAGGGAGCCAGGCA	Cleavage
tae-miR1127b-3p	Traes_1AL_4A458DF14.2	PF13812,PF01535,PF13041					AT3G62470.1	Pentatricopeptide repeat (PPR) superfamily protein	LOC_Os05g28720.1	PPR repeat containing protein, expressed	1,50	-1.0	1	21	1738	1758	ACAAGUAUUUCUGGACGGAGG	CCUCCGUCCGGAAAUACUUGU	Cleavage
tae-miR1130b-3p	Traes_1AL_51B1D5DBC.6	PF07714		2.7.11.1		GO:GO:0006468,GO:GO:0004672	AT5G14640.1	shaggy-like kinase 13	LOC_Os10g37740.1	CGMC_GSK.9 - CGMC includes CDA, MAPK, GSK3, and CLKC kinases, expressed	0,00	-1.0	1	21	914	934	UCUUAUAUUAUGGGACGGAGG	CCUCCGUCCCAUAAUAUAAGA	Cleavage
tae-miR1122c-3p	Traes_1AL_51B1D5DBC.6	PF07714		2.7.11.1		GO:GO:0006468,GO:GO:0004672	AT5G14640.1	shaggy-like kinase 13	LOC_Os10g37740.1	CGMC_GSK.9 - CGMC includes CDA, MAPK, GSK3, and CLKC kinases, expressed	1,50	-1.0	1	21	914	934	UCUAAUAUUAUGGGACGGAGG	CCUCCGUCCCAUAAUAUAAGA	Cleavage
tae-miR5049-3p	Traes_1AL_7D6DC73FC.3	PF14531	KOG0583	2.7.11.1			AT3G01090.2	SNF1 kinase homolog 10	LOC_Os05g45420.2	CAMK_KIN1/SNF1/Nim1_like_AMPKh.3 - CAMK includes calcium/calmodulin depedent protein kinases, expressed	2,00	-1.0	1	21	1256	1276	AAUAUGGAUCGGAGGGAGUAC	UUUCUCCCUCCGAUCCAUAUA	Cleavage
tae-miR1128	Traes_1AL_968B97E50.2	PF06480,PF00004,PF01434			K08956	GO:GO:0016021,GO:GO:0008270,GO:GO:0005524,GO:GO:0004222,GO:GO:0006508	AT1G07510.1	FTSH protease 10	LOC_Os05g38400.3	OsFtsH8 FtsH protease, homologue of AtFtsH3/10, expressed	3,00	-1.0	1	21	100	121	UACUACUCCCUCCGUCC-GAAA	UUUCCGGACGGAGGGAGUAGUU	Cleavage
tae-miR7757-5p	Traes_1AL_B6931117F.1	PF00931				GO:GO:0043531	AT3G46530.1	NB-ARC domain-containing disease resistance protein	LOC_Os08g10440.1	NBS-LRR disease resistance protein, putative, expressed	1,50	-1.0	1	22	1301	1322	AUAAAACCUUCAGCUAUCCAUC	AAUGGAUAGCUGAAGGCUUUAU	Cleavage
tae-miR7757-5p	Traes_1AL_B6931117F.2	PF00931				GO:GO:0043531	AT3G46530.1	NB-ARC domain-containing disease resistance protein	LOC_Os08g10440.1	NBS-LRR disease resistance protein, putative, expressed	1,50	-1.0	1	22	1343	1364	AUAAAACCUUCAGCUAUCCAUC	AAUGGAUAGCUGAAGGCUUUAU	Cleavage
tae-miR7757-5p	Traes_1AL_B6931117F.3	PF00931				GO:GO:0043531	AT3G46530.1	NB-ARC domain-containing disease resistance protein	LOC_Os08g10440.1	NBS-LRR disease resistance protein, putative, expressed	1,50	-1.0	1	22	1301	1322	AUAAAACCUUCAGCUAUCCAUC	AAUGGAUAGCUGAAGGCUUUAU	Cleavage
tae-miR7757-5p	Traes_1AL_B6931117F.4	PF00931				GO:GO:0043531	AT3G46530.1	NB-ARC domain-containing disease resistance protein	LOC_Os08g10440.1	NBS-LRR disease resistance protein, putative, expressed	1,50	-1.0	1	22	1301	1322	AUAAAACCUUCAGCUAUCCAUC	AAUGGAUAGCUGAAGGCUUUAU	Cleavage
tae-miR9778	Traes_1AS_17306388F.1	PF00931				GO:GO:0043531	AT3G07040.1	NB-ARC domain-containing disease resistance protein	LOC_Os08g42670.2	resistance protein, putative, expressed	2,00	-1.0	1	21	126	146	UGCAUCAUCUCGAACUCGUCG	CAAUGAGCUCGAGAUGAUGCA	Cleavage
tae-miR9778	Traes_1AS_17306388F.3	PF00931				GO:GO:0043531	AT3G46530.1	NB-ARC domain-containing disease resistance protein	LOC_Os12g37290.1	resistance protein T10rga2-1A, putative, expressed	2,00	-1.0	1	21	126	146	UGCAUCAUCUCGAACUCGUCG	CAAUGAGCUCGAGAUGAUGCA	Cleavage
tae-miR1135	Traes_1AS_4A925522B.4	PF13920					AT5G01960.1	RING/U-box superfamily protein	LOC_Os01g07390.1	zinc finger, C3HC4 type family protein, expressed	0,00	-1.0	1	24	511	534	CUGCGACAAGUAAUUCCGAACGGA	UCCGUUCGGAAUUACUUGUCGCAG	Cleavage
tae-miR6197-5p	Traes_1AS_5DCFDC258.1	PF04578,PF13968					AT5G45540.1	Protein of unknown function (DUF594)	LOC_Os07g08270.1	expressed protein	3,00	-1.0	1	21	1353	1376	UCUGUAAACAAAUGUA---GGACG	ACUCCAACUACAUUUGUUUACAGA	Cleavage
tae-miR1137a	Traes_1AS_8912BD052.27										0,00	-1.0	1	20	136	155	UAGUACAAAGUUGAGUCAUC	GAUGACUCAACUUUGUACUA	Cleavage
tae-miR9674a-5p	Traes_1AS_92A698B19.1	PF01535,PF13041			K17964		AT1G63130.1	Tetratricopeptide repeat (TPR)-like superfamily protein	LOC_Os04g28300.1	Rf1, mitochondrial precursor, putative, expressed	2,50	-1.0	1	21	1761	1781	GCAUCAUCCAUCCUACCAUUC	AAAUGGAAGGAUCGAUGAUGC	Cleavage
tae-miR9674a-5p	Traes_1AS_92A698B19.10	PF01535,PF13041					AT1G63130.1	Tetratricopeptide repeat (TPR)-like superfamily protein	LOC_Os04g28300.1	Rf1, mitochondrial precursor, putative, expressed	2,50	-1.0	1	21	1761	1781	GCAUCAUCCAUCCUACCAUUC	AAAUGGAAGGAUCGAUGAUGC	Cleavage
tae-miR9674a-5p	Traes_1AS_92A698B19.12	PF13041					AT1G12300.1	Tetratricopeptide repeat (TPR)-like superfamily protein	LOC_Os04g28300.1	Rf1, mitochondrial precursor, putative, expressed	2,50	-1.0	1	21	96	116	GCAUCAUCCAUCCUACCAUUC	AAAUGGAAGGAUCGAUGAUGC	Cleavage
tae-miR9674a-5p	Traes_1AS_92A698B19.2	PF01535,PF13041			K17964		AT1G63130.1	Tetratricopeptide repeat (TPR)-like superfamily protein	LOC_Os04g28300.1	Rf1, mitochondrial precursor, putative, expressed	2,50	-1.0	1	21	1761	1781	GCAUCAUCCAUCCUACCAUUC	AAAUGGAAGGAUCGAUGAUGC	Cleavage
tae-miR9674a-5p	Traes_1AS_92A698B19.3	PF01535,PF13041			K17964		AT1G63130.1	Tetratricopeptide repeat (TPR)-like superfamily protein	LOC_Os04g28300.1	Rf1, mitochondrial precursor, putative, expressed	2,50	-1.0	1	21	1761	1781	GCAUCAUCCAUCCUACCAUUC	AAAUGGAAGGAUCGAUGAUGC	Cleavage
tae-miR9674a-5p	Traes_1AS_92A698B19.4	PF01535,PF13041			K17964		AT1G63130.1	Tetratricopeptide repeat (TPR)-like superfamily protein	LOC_Os04g28300.1	Rf1, mitochondrial precursor, putative, expressed	2,50	-1.0	1	21	1761	1781	GCAUCAUCCAUCCUACCAUUC	AAAUGGAAGGAUCGAUGAUGC	Cleavage
tae-miR9674a-5p	Traes_1AS_92A698B19.5	PF01535,PF13041			K17964		AT1G63130.1	Tetratricopeptide repeat (TPR)-like superfamily protein	LOC_Os04g28300.1	Rf1, mitochondrial precursor, putative, expressed	2,50	-1.0	1	21	1761	1781	GCAUCAUCCAUCCUACCAUUC	AAAUGGAAGGAUCGAUGAUGC	Cleavage
tae-miR9674a-5p	Traes_1AS_92A698B19.6	PF01535,PF13041			K17964		AT1G63130.1	Tetratricopeptide repeat (TPR)-like superfamily protein	LOC_Os04g28300.1	Rf1, mitochondrial precursor, putative, expressed	2,50	-1.0	1	21	1761	1781	GCAUCAUCCAUCCUACCAUUC	AAAUGGAAGGAUCGAUGAUGC	Cleavage
tae-miR9674a-5p	Traes_1AS_92A698B19.7	PF01535,PF13041			K17964		AT1G63130.1	Tetratricopeptide repeat (TPR)-like superfamily protein	LOC_Os04g28300.1	Rf1, mitochondrial precursor, putative, expressed	2,50	-1.0	1	21	1761	1781	GCAUCAUCCAUCCUACCAUUC	AAAUGGAAGGAUCGAUGAUGC	Cleavage
tae-miR9674a-5p	Traes_1AS_92A698B19.8	PF01535,PF13041			K17964		AT1G63130.1	Tetratricopeptide repeat (TPR)-like superfamily protein	LOC_Os04g28300.1	Rf1, mitochondrial precursor, putative, expressed	2,50	-1.0	1	21	1761	1781	GCAUCAUCCAUCCUACCAUUC	AAAUGGAAGGAUCGAUGAUGC	Cleavage
tae-miR9674a-5p	Traes_1AS_92A698B19.9	PF01535,PF13041			K17964		AT1G63130.1	Tetratricopeptide repeat (TPR)-like superfamily protein	LOC_Os04g28300.1	Rf1, mitochondrial precursor, putative, expressed	2,50	-1.0	1	21	1761	1781	GCAUCAUCCAUCCUACCAUUC	AAAUGGAAGGAUCGAUGAUGC	Cleavage
tae-miR5049-3p	Traes_1AS_A482D4FF9.1	PF04997		2.7.7.6		GO:GO:0006351,GO:GO:0003899,GO:GO:0003677	AT1G63020.1	nuclear RNA polymerase D1A	LOC_Os02g05880.1	RNA polymerase Rpb1, domain 2 family protein, expressed	0,00	-1.0	1	21	557	577	AAUAUGGAUCGGAGGGAGUAC	GUACUCCCUCCGAUCCAUAUU	Cleavage
tae-miR9778	Traes_1AS_C7A8188D1.1	PF13191,PF00931	KOG4658			GO:GO:0043531	AT1G58390.1	Disease resistance protein (CC-NBS-LRR class) family	LOC_Os08g42670.2	resistance protein, putative, expressed	1,00	-1.0	1	21	126	146	UGCAUCAUCUCGAACUCGUCG	CAACGAGCUCGAGAUGAUGCA	Cleavage
tae-miR9778	Traes_1AS_C7A8188D1.3	PF00931				GO:GO:0043531	AT3G46530.1	NB-ARC domain-containing disease resistance protein	LOC_Os02g57310.1	pib, putative, expressed	1,00	-1.0	1	21	126	146	UGCAUCAUCUCGAACUCGUCG	CAACGAGCUCGAGAUGAUGCA	Cleavage
tae-miR9661-5p	Traes_1AS_D8E37233E.1										2,00	-1.0	1	21	101	121	UGAAGUAGAGCAGGACCUCA	CCAAGUCCCUGCUCUACUUCG	Cleavage
tae-miR1128	Traes_1BL_087CA0963.1	PF16876,PF08235	KOG2116	3.1.3.4			AT3G09560.1	Lipin family protein	LOC_Os05g38710.1	lipin, N-terminal conserved region family protein, expressed	3,00	-1.0	1	21	2048	2069	UACUACUCCCUCCG-UCCGAAA	UUUAGGAACGGAGGGAGUAGUA	Cleavage
tae-miR1128	Traes_1BL_0FDC38712.1	PF13639				GO:GO:0008270,GO:GO:0005515	AT1G17970.1	RING/U-box superfamily protein	LOC_Os05g47670.1	zinc finger, C3HC4 type domain containing protein, expressed	1,50	-1.0	1	21	225	245	UACUACUCCCUCCGUCCGAAA	UUUCGGACGGAGGGAGUACUA	Cleavage
tae-miR1127b-3p	Traes_1BL_0FDC38712.1	PF13639				GO:GO:0008270,GO:GO:0005515	AT1G17970.1	RING/U-box superfamily protein	LOC_Os05g47670.1	zinc finger, C3HC4 type domain containing protein, expressed	1,50	-1.0	1	21	121	141	ACAAGUAUUUCUGGACGGAGG	CCUCCGUCCGGAAAUACUUGU	Cleavage
tae-miR5062-5p	Traes_1BL_219B2BB6D.16						AT2G46060.1	transmembrane protein-related	LOC_Os10g30910.2	transmembrane protein-related, putative, expressed	2,50	-1.0	1	23	836	858	UGAACCUUAGGGAACAGCCGCAU	UUUUGGAUGUUCCCCAAGGUUCA	Cleavage
tae-miR5062-5p	Traes_1BL_219B2BB6D.17						AT2G46060.1	transmembrane protein-related	LOC_Os10g30910.2	transmembrane protein-related, putative, expressed	2,50	-1.0	1	23	836	858	UGAACCUUAGGGAACAGCCGCAU	UUUUGGAUGUUCCCCAAGGUUCA	Cleavage
tae-miR5062-5p	Traes_1BL_219B2BB6D.18						AT2G46060.1	transmembrane protein-related	LOC_Os10g30910.2	transmembrane protein-related, putative, expressed	2,50	-1.0	1	23	836	858	UGAACCUUAGGGAACAGCCGCAU	UUUUGGAUGUUCCCCAAGGUUCA	Cleavage
tae-miR160	Traes_1BL_54CD82AC3.1	PF06507				GO:GO:0009725,GO:GO:0006355,GO:GO:0005634,GO:GO:0003677	AT4G30080.1	auxin response factor 16	LOC_Os10g33940.1	auxin response factor 18, putative, expressed	0,00	-1.0	1	21	642	662	UGCCUGGCUCCCUGUAUGCCA	AGGCAUACAGGGAGCCAGGCA	Cleavage
tae-miR160	Traes_1BL_54CD82AC3.2						AT4G30080.1	auxin response factor 16	LOC_Os10g33940.1	auxin response factor 18, putative, expressed	0,00	-1.0	1	21	369	389	UGCCUGGCUCCCUGUAUGCCA	AGGCAUACAGGGAGCCAGGCA	Cleavage
tae-miR160	Traes_1BL_54CD82AC3.3						AT4G30080.1	auxin response factor 16	LOC_Os10g33940.1	auxin response factor 18, putative, expressed	0,00	-1.0	1	21	267	287	UGCCUGGCUCCCUGUAUGCCA	AGGCAUACAGGGAGCCAGGCA	Cleavage
tae-miR1130b-3p	Traes_1BL_89E785AAA.2	PF00436	KOG1653			GO:GO:0003697,GO:GO:0006260	AT4G11060.1	mitochondrially targeted single-stranded DNA binding protein	LOC_Os05g43440.2	single-stranded DNA-binding protein, putative, expressed	2,00	-1.0	1	21	591	611	UCUUAUAUUAUGGGACGGAGG	CCUCUGUCCCAUAAUAUAAGG	Cleavage
tae-miR159b	Traes_1BL_CF98E922B.1	PF00249					AT5G06100.2	myb domain protein 33	LOC_Os01g59660.2	MYB family transcription factor, putative, expressed	2,50	-1.0	1	21	839	859	UUUGGAUUGAAGGGAGCUCUG	UGGAGCUCCCUUCACUCCAAU	Cleavage
tae-miR159a	Traes_1BL_CF98E922B.1	PF00249					AT5G06100.2	myb domain protein 33	LOC_Os01g59660.2	MYB family transcription factor, putative, expressed	2,50	-1.0	1	21	839	859	UUUGGAUUGAAGGGAGCUCUG	UGGAGCUCCCUUCACUCCAAU	Cleavage
tae-miR159b	Traes_1BL_CF98E922B.2	PF00249					AT5G06100.3	myb domain protein 33	LOC_Os01g59660.1	MYB family transcription factor, putative, expressed	2,50	-1.0	1	21	839	859	UUUGGAUUGAAGGGAGCUCUG	UGGAGCUCCCUUCACUCCAAU	Cleavage
tae-miR159a	Traes_1BL_CF98E922B.2	PF00249					AT5G06100.3	myb domain protein 33	LOC_Os01g59660.1	MYB family transcription factor, putative, expressed	2,50	-1.0	1	21	839	859	UUUGGAUUGAAGGGAGCUCUG	UGGAGCUCCCUUCACUCCAAU	Cleavage
tae-miR159b	Traes_1BL_CF98E922B.3						AT5G06100.3	myb domain protein 33	LOC_Os01g59660.2	MYB family transcription factor, putative, expressed	2,50	-1.0	1	21	512	532	UUUGGAUUGAAGGGAGCUCUG	UGGAGCUCCCUUCACUCCAAU	Cleavage
tae-miR159a	Traes_1BL_CF98E922B.3						AT5G06100.3	myb domain protein 33	LOC_Os01g59660.2	MYB family transcription factor, putative, expressed	2,50	-1.0	1	21	512	532	UUUGGAUUGAAGGGAGCUCUG	UGGAGCUCCCUUCACUCCAAU	Cleavage
tae-miR159b	Traes_1BL_CF98E922B.4						AT5G06100.1	myb domain protein 33	LOC_Os01g59660.1	MYB family transcription factor, putative, expressed	2,50	-1.0	1	21	512	532	UUUGGAUUGAAGGGAGCUCUG	UGGAGCUCCCUUCACUCCAAU	Cleavage
tae-miR159a	Traes_1BL_CF98E922B.4						AT5G06100.1	myb domain protein 33	LOC_Os01g59660.1	MYB family transcription factor, putative, expressed	2,50	-1.0	1	21	512	532	UUUGGAUUGAAGGGAGCUCUG	UGGAGCUCCCUUCACUCCAAU	Cleavage
tae-miR1117	Traes_1BL_F01A87F44.1	PF13359					AT2G13770.1		LOC_Os01g33990.1	transposon protein, putative, CACTA, En/Spm sub-class, expressed	2,50	-1.0	1	24	37	60	UAGUACCGGUUCGUGGCACGAACC	GGUUCGUGGCACGAACCGGUGCUA	Cleavage
tae-miR1131	Traes_1BL_F01A87F44.1	PF13359					AT2G13770.1		LOC_Os01g33990.1	transposon protein, putative, CACTA, En/Spm sub-class, expressed	2,50	-1.0	1	22	63	84	UAGUACCGGUUCGUGGCUAACC	GGUUAGCCACGAACCGGUAAUG	Cleavage
tae-miR1122b-3p	Traes_1BS_34C60A54E.1	PF00651				GO:GO:0005515	AT5G19000.1	BTB-POZ and MATH domain 1	LOC_Os08g41120.1	MBTB29 - Bric-a-Brac, Tramtrack, Broad Complex BTB domain with Meprin and TRAF Homology MATH domain, expressed	1,50	-1.0	1	21	47	67	AGACUUAUAUGUAGGAACGGA	UCCGUUCCUAAAUAUAAGUCU	Translation
tae-miR9662a-3p	Traes_1BS_566CEE7F6.1	PF02536				GO:GO:0006355,GO:GO:0005739,GO:GO:0003690	AT1G21150.1	Mitochondrial transcription termination factor family protein	LOC_Os06g12100.1	mTERF family protein, expressed	2,00	-1.0	1	21	441	461	UUGAACAUCCCAGAGCCACCG	UCGUGGCGCUGGGAUGUUCAG	Cleavage
tae-miR9662b-3p	Traes_1BS_566CEE7F6.1	PF02536				GO:GO:0006355,GO:GO:0005739,GO:GO:0003690	AT1G21150.1	Mitochondrial transcription termination factor family protein	LOC_Os06g12100.1	mTERF family protein, expressed	2,50	-1.0	1	21	440	460	UGAACAUCCCAGAGCCACCGG	CUCGUGGCGCUGGGAUGUUCA	Cleavage
tae-miR9662a-3p	Traes_1BS_566CEE7F6.2	PF02536				GO:GO:0006355,GO:GO:0005739,GO:GO:0003690	AT1G21150.1	Mitochondrial transcription termination factor family protein	LOC_Os06g12100.1	mTERF family protein, expressed	2,00	-1.0	1	21	66	86	UUGAACAUCCCAGAGCCACCG	UCGUGGCGCUGGGAUGUUCAG	Cleavage
tae-miR9662b-3p	Traes_1BS_566CEE7F6.2	PF02536				GO:GO:0006355,GO:GO:0005739,GO:GO:0003690	AT1G21150.1	Mitochondrial transcription termination factor family protein	LOC_Os06g12100.1	mTERF family protein, expressed	2,50	-1.0	1	21	65	85	UGAACAUCCCAGAGCCACCGG	CUCGUGGCGCUGGGAUGUUCA	Cleavage
tae-miR9778	Traes_1BS_70B733D34.1	PF13191,PF00931				GO:GO:0043531	AT3G07040.1	NB-ARC domain-containing disease resistance protein	LOC_Os08g42670.1	resistance protein, putative, expressed	2,00	-1.0	1	21	126	146	UGCAUCAUCUCGAACUCGUCG	CAAUGAGCUCGAGAUGAUGCA	Cleavage
tae-miR9778	Traes_1BS_70B733D34.2	PF13191,PF00931				GO:GO:0043531	AT3G07040.1	NB-ARC domain-containing disease resistance protein	LOC_Os08g42670.2	resistance protein, putative, expressed	2,00	-1.0	1	21	126	146	UGCAUCAUCUCGAACUCGUCG	CAAUGAGCUCGAGAUGAUGCA	Cleavage
tae-miR5049-3p	Traes_1BS_7EC2BDE95.1	PF16760		2.4.1.21			AT1G11720.2	starch synthase 3	LOC_Os08g09230.1	starch synthase III, putative, expressed	1,50	-1.0	1	21	3430	3450	AAUAUGGAUCGGAGGGAGUAC	CUACUCCCUCCAAUCCAUAUU	Translation
tae-miR5049-3p	Traes_1BS_B0BB95B97.6	PF04997		2.7.7.6		GO:GO:0006351,GO:GO:0003899,GO:GO:0003677	AT1G63020.2	nuclear RNA polymerase D1A	LOC_Os02g05880.1	RNA polymerase Rpb1, domain 2 family protein, expressed	0,00	-1.0	1	21	623	643	AAUAUGGAUCGGAGGGAGUAC	GUACUCCCUCCGAUCCAUAUU	Cleavage
tae-miR7757-5p	Traes_1BS_CF1C4F54E.2	PF00931				GO:GO:0043531	AT1G09920.1	TRAF-type zinc finger-related	LOC_Os11g45750.1	WRKY125, expressed	2,50	-1.0	1	22	119	140	AUAAAACCUUCAGCUAUCCAUC	AAUGGUUAGCUGAAGGUUUCAU	Cleavage
tae-miR159b	Traes_1DL_25540DE53.1						AT2G32460.1	myb domain protein 101	LOC_Os01g59660.2	MYB family transcription factor, putative, expressed	2,50	-1.0	1	21	512	532	UUUGGAUUGAAGGGAGCUCUG	UGGAGCUCCCUUCAUUCCAAU	Cleavage
tae-miR159a	Traes_1DL_25540DE53.1						AT2G32460.1	myb domain protein 101	LOC_Os01g59660.2	MYB family transcription factor, putative, expressed	2,50	-1.0	1	21	512	532	UUUGGAUUGAAGGGAGCUCUG	UGGAGCUCCCUUCAUUCCAAU	Cleavage
tae-miR159b	Traes_1DL_25540DE53.2						AT2G32460.1	myb domain protein 101	LOC_Os01g59660.1	MYB family transcription factor, putative, expressed	2,50	-1.0	1	21	512	532	UUUGGAUUGAAGGGAGCUCUG	UGGAGCUCCCUUCAUUCCAAU	Cleavage
tae-miR159a	Traes_1DL_25540DE53.2						AT2G32460.1	myb domain protein 101	LOC_Os01g59660.1	MYB family transcription factor, putative, expressed	2,50	-1.0	1	21	512	532	UUUGGAUUGAAGGGAGCUCUG	UGGAGCUCCCUUCAUUCCAAU	Cleavage
tae-miR159b	Traes_1DL_25540DE53.3						AT5G06100.3	myb domain protein 33	LOC_Os01g59660.1	MYB family transcription factor, putative, expressed	2,50	-1.0	1	21	470	490	UUUGGAUUGAAGGGAGCUCUG	UGGAGCUCCCUUCAUUCCAAU	Cleavage
tae-miR159a	Traes_1DL_25540DE53.3						AT5G06100.3	myb domain protein 33	LOC_Os01g59660.1	MYB family transcription factor, putative, expressed	2,50	-1.0	1	21	470	490	UUUGGAUUGAAGGGAGCUCUG	UGGAGCUCCCUUCAUUCCAAU	Cleavage
tae-miR159b	Traes_1DL_25540DE53.4						AT5G06100.3	myb domain protein 33	LOC_Os01g59660.2	MYB family transcription factor, putative, expressed	2,50	-1.0	1	21	470	490	UUUGGAUUGAAGGGAGCUCUG	UGGAGCUCCCUUCAUUCCAAU	Cleavage
tae-miR159a	Traes_1DL_25540DE53.4						AT5G06100.3	myb domain protein 33	LOC_Os01g59660.2	MYB family transcription factor, putative, expressed	2,50	-1.0	1	21	470	490	UUUGGAUUGAAGGGAGCUCUG	UGGAGCUCCCUUCAUUCCAAU	Cleavage
tae-miR159b	Traes_1DL_25540DE53.5						AT5G06100.2	myb domain protein 33	LOC_Os01g59660.2	MYB family transcription factor, putative, expressed	2,50	-1.0	1	21	470	490	UUUGGAUUGAAGGGAGCUCUG	UGGAGCUCCCUUCAUUCCAAU	Cleavage
tae-miR159a	Traes_1DL_25540DE53.5						AT5G06100.2	myb domain protein 33	LOC_Os01g59660.2	MYB family transcription factor, putative, expressed	2,50	-1.0	1	21	470	490	UUUGGAUUGAAGGGAGCUCUG	UGGAGCUCCCUUCAUUCCAAU	Cleavage
tae-miR159b	Traes_1DL_25540DE53.6						AT5G06100.2	myb domain protein 33	LOC_Os01g59660.1	MYB family transcription factor, putative, expressed	2,50	-1.0	1	21	470	490	UUUGGAUUGAAGGGAGCUCUG	UGGAGCUCCCUUCAUUCCAAU	Cleavage
tae-miR159a	Traes_1DL_25540DE53.6						AT5G06100.2	myb domain protein 33	LOC_Os01g59660.1	MYB family transcription factor, putative, expressed	2,50	-1.0	1	21	470	490	UUUGGAUUGAAGGGAGCUCUG	UGGAGCUCCCUUCAUUCCAAU	Cleavage
tae-miR1137a	Traes_1DL_34A359364.2	PF09439	KOG0070		K07937		AT5G14670.1	ADP-ribosylation factor A1B	LOC_Os03g59740.1	ADP-ribosylation factor, putative, expressed	2,50	-1.0	1	20	516	535	UAGUACAAAGUUGAGUCAUC	GAUGACCCAAUUUUGUACUA	Translation
tae-miR5062-5p	Traes_1DL_38415F71A.19						AT2G46060.2	transmembrane protein-related	LOC_Os10g30910.2	transmembrane protein-related, putative, expressed	2,50	-1.0	1	23	836	858	UGAACCUUAGGGAACAGCCGCAU	UUUUGGAUGUUCCCCAAGGUUCA	Cleavage
tae-miR5062-5p	Traes_1DL_38415F71A.20						AT2G46060.2	transmembrane protein-related	LOC_Os10g30910.2	transmembrane protein-related, putative, expressed	2,50	-1.0	1	23	836	858	UGAACCUUAGGGAACAGCCGCAU	UUUUGGAUGUUCCCCAAGGUUCA	Cleavage
tae-miR5062-5p	Traes_1DL_38415F71A.22						AT2G46060.1	transmembrane protein-related	LOC_Os10g30910.2	transmembrane protein-related, putative, expressed	2,50	-1.0	1	23	836	858	UGAACCUUAGGGAACAGCCGCAU	UUUUGGAUGUUCCCCAAGGUUCA	Cleavage
tae-miR1122a	Traes_1DL_38B88249C.2	PF00069	KOG0580	2.7.11.1		GO:GO:0006468,GO:GO:0005524,GO:GO:0004672	AT2G42960.1	Protein kinase superfamily protein	LOC_Os10g35450.1	serine/threonine-protein kinase, putative, expressed	0,00	-1.0	1	20	976	995	UAGAUACAUCCGUAUCUAGA	UCUAGAUACGGAUGUAUCUA	Cleavage
tae-miR1120a	Traes_1DL_6A91BC5A5.1	PF08652			K14845		AT4G17620.1	glycine-rich protein	LOC_Os05g32580.1	glycine-rich protein, putative, expressed	2,50	-1.0	1	24	25	48	ACAUUCUUAUAUUAUGAGACGGAG	CUCCGUCCCAUAAUAUAAGAACGU	Cleavage
tae-miR1120b-3p	Traes_1DL_6A91BC5A5.1	PF08652			K14845		AT4G17620.1	glycine-rich protein	LOC_Os05g32580.1	glycine-rich protein, putative, expressed	2,50	-1.0	1	21	25	45	UUCUUAUAUUGUGGGACAGAG	CUCCGUCCCAUAAUAUAAGAA	Translation
tae-miR1130b-3p	Traes_1DL_6A91BC5A5.1	PF08652			K14845		AT4G17620.1	glycine-rich protein	LOC_Os05g32580.1	glycine-rich protein, putative, expressed	0,00	-1.0	1	21	24	44	UCUUAUAUUAUGGGACGGAGG	CCUCCGUCCCAUAAUAUAAGA	Cleavage
tae-miR1122c-3p	Traes_1DL_6A91BC5A5.1	PF08652			K14845		AT4G17620.1	glycine-rich protein	LOC_Os05g32580.1	glycine-rich protein, putative, expressed	1,50	-1.0	1	21	24	44	UCUAAUAUUAUGGGACGGAGG	CCUCCGUCCCAUAAUAUAAGA	Cleavage
tae-miR1120c-5p	Traes_1DL_6A91BC5A5.1	PF08652			K14845		AT4G17620.1	glycine-rich protein	LOC_Os05g32580.1	glycine-rich protein, putative, expressed	2,50	-1.0	1	21	67	87	UAAUAUAAGAACGUUUUUGAC	GUCAAACACGCUCUUAUAUUA	Translation
tae-miR160	Traes_1DL_AEC2F3FB3.1	PF06507				GO:GO:0009725,GO:GO:0006355,GO:GO:0005634,GO:GO:0003677	AT4G30080.1	auxin response factor 16	LOC_Os10g33940.1	auxin response factor 18, putative, expressed	0,00	-1.0	1	21	642	662	UGCCUGGCUCCCUGUAUGCCA	AGGCAUACAGGGAGCCAGGCA	Cleavage
tae-miR1122b-3p	Traes_1DL_B5E6E273B.1										1,50	-1.0	1	21	35	55	AGACUUAUAUGUAGGAACGGA	UCCGUUCCUAAAUAUAAGUCU	Translation
tae-miR5175-5p	Traes_1DL_C1BE1742F.2	PF03641					AT3G53450.1	Putative lysine decarboxylase family protein	LOC_Os10g33900.1	possible lysine decarboxylase domain containing protein, expressed	2,00	-1.0	1	21	109	130	UUCCAAAUUACUCGUCG-UGGU	UUCAGCGACGAGUAAUUUGGAA	Cleavage
tae-miR1133	Traes_1DL_D391AE3DC.1	PF16212		3.6.3.1		GO:GO:0016021,GO:GO:0015914,GO:GO:0005524,GO:GO:0004012,GO:GO:0000287	AT1G59820.1	aminophospholipid ATPase 3	LOC_Os10g27220.1	phospholipid-transporting ATPase 3, putative, expressed	2,00	-1.0	1	22	858	879	CAUAUACUCCCUCCGUCCGAAA	AAAUGAACGGAGGGAGUAUAUG	Cleavage
tae-miR1133	Traes_1DL_D391AE3DC.2	PF16212		3.6.3.1		GO:GO:0016021,GO:GO:0015914,GO:GO:0005524,GO:GO:0004012,GO:GO:0000287	AT1G59820.1	aminophospholipid ATPase 3	LOC_Os10g27220.1	phospholipid-transporting ATPase 3, putative, expressed	2,00	-1.0	1	22	858	879	CAUAUACUCCCUCCGUCCGAAA	AAAUGAACGGAGGGAGUAUAUG	Cleavage
tae-miR1133	Traes_1DL_D391AE3DC.3	PF16212		3.6.3.1		GO:GO:0016021,GO:GO:0015914,GO:GO:0005524,GO:GO:0004012,GO:GO:0000287	AT1G59820.1	aminophospholipid ATPase 3	LOC_Os10g27220.1	phospholipid-transporting ATPase 3, putative, expressed	2,00	-1.0	1	22	822	843	CAUAUACUCCCUCCGUCCGAAA	AAAUGAACGGAGGGAGUAUAUG	Cleavage
tae-miR1128	Traes_1DL_E30609261.7						AT1G04300.3	TRAF-like superfamily protein	LOC_Os05g43280.1	MATH domain containing protein, expressed	3,00	-1.0	1	21	76	96	UACUACUCCCUCCGUCCGAAA	UUCCAGACGGAGGGAGUAGUU	Cleavage
tae-miR1122a	Traes_1DS_06B8348E0.1	PF03283				GO:GO:0016787	AT3G09410.1	Pectinacetylesterase family protein	LOC_Os05g02120.1	pectinacetylesterase domain containing protein, expressed	0,00	-1.0	1	20	794	813	UAGAUACAUCCGUAUCUAGA	UCUAGAUACGGAUGUAUCUA	Cleavage
tae-miR1122a	Traes_1DS_06B8348E0.2	PF03283				GO:GO:0016787	AT3G09410.1	Pectinacetylesterase family protein	LOC_Os05g02120.1	pectinacetylesterase domain containing protein, expressed	0,00	-1.0	1	20	620	639	UAGAUACAUCCGUAUCUAGA	UCUAGAUACGGAUGUAUCUA	Cleavage
tae-miR5048-5p	Traes_1DS_26F1546F6.1	PF00168,PF07714,PF00069	KOG1187	2.7.11.1		GO:GO:0005515,GO:GO:0006468,GO:GO:0004672,GO:GO:0005524	AT1G63430.1	Leucine-rich repeat protein kinase family protein	LOC_Os04g58700.1	expressed protein	2,50	-1.0	1	22	917	938	UUUGCAGGUUUUAGGUCUAAGU	ACUUGGACCUAAAACCUGCCAA	Cleavage
tae-miR5048-5p	Traes_1DS_26F1546F6.2	PF00168,PF07714,PF00069	KOG1187	2.7.11.1		GO:GO:0005515,GO:GO:0006468,GO:GO:0004672,GO:GO:0005524	AT1G63430.1	Leucine-rich repeat protein kinase family protein	LOC_Os04g58700.1	expressed protein	2,50	-1.0	1	22	917	938	UUUGCAGGUUUUAGGUCUAAGU	ACUUGGACCUAAAACCUGCCAA	Cleavage
tae-miR7757-5p	Traes_1DS_35EF41CCB.2	PF00931				GO:GO:0043531	AT3G50950.1	HOPZ-ACTIVATED RESISTANCE 1	LOC_Os05g40150.1	RGH2B, putative, expressed	1,50	-1.0	1	22	587	608	AUAAAACCUUCAGCUAUCCAUC	AGUGGAUAGCUGAAGGUUUCAU	Cleavage
tae-miR9674a-5p	Traes_1DS_F3DE3946F.1	PF13041					AT1G62930.1	Tetratricopeptide repeat (TPR)-like superfamily protein	LOC_Os04g28300.1	Rf1, mitochondrial precursor, putative, expressed	2,50	-1.0	1	21	522	542	GCAUCAUCCAUCCUACCAUUC	AAAUGGAAGGAUCGAUGAUGC	Cleavage
tae-miR5049-3p	Traes_1DS_FA7FC5C3C.1	PF04997		2.7.7.6		GO:GO:0006351,GO:GO:0003899,GO:GO:0003677	AT1G63020.1	nuclear RNA polymerase D1A	LOC_Os02g05880.1	RNA polymerase Rpb1, domain 2 family protein, expressed	0,00	-1.0	1	21	623	643	AAUAUGGAUCGGAGGGAGUAC	GUACUCCCUCCGAUCCAUAUU	Cleavage
tae-miR1128	Traes_2AL_24A1BF00C.1						AT5G06210.1	RNA binding (RRM/RBD/RNP motifs) family protein	LOC_Os04g33810.1	RNA recognition motif containing protein, putative, expressed	0,00	-1.0	1	21	37	57	UACUACUCCCUCCGUCCGAAA	UUUCGGACGGAGGGAGUAGUA	Cleavage
tae-miR5049-3p	Traes_2AL_28B13CC56.1					GO:GO:0005681,GO:GO:0000398	AT1G80070.1	Pre-mRNA-processing-splicing factor	LOC_Os06g07080.1	pre-mRNA-processing-splicing factor 8, putative, expressed	1,50	-1.0	1	21	346	366	AAUAUGGAUCGGAGGGAGUAC	CUACUCCCUCCGUUCCAUAUU	Cleavage
tae-miR1127a	Traes_2AL_4197D219B.1						AT3G16200.1		LOC_Os01g31220.1	expressed protein	2,50	-1.0	1	19	991	1009	UCCUUCCGUUCGGAAUUAC	GUAAUUCCGAACGGAGGGU	Cleavage
tae-miR164	Traes_2AL_57BF37A86.1	PF02365				GO:GO:0006355,GO:GO:0003677	AT5G61430.1	NAC domain containing protein 100	LOC_Os04g38720.1	no apical meristem protein, putative, expressed	1,50	-1.0	1	21	680	700	UGGAGAAGCAGGGCACGUGCA	CGCACGUGACCUGCUUCUCCA	Cleavage
tae-miR164	Traes_2AL_57BF37A86.2	PF02365				GO:GO:0006355,GO:GO:0003677	AT5G61430.1	NAC domain containing protein 100	LOC_Os04g38720.1	no apical meristem protein, putative, expressed	1,50	-1.0	1	21	606	626	UGGAGAAGCAGGGCACGUGCA	CGCACGUGACCUGCUUCUCCA	Cleavage
tae-miR1122a	Traes_2AL_64B73E3ED.1			2.8.1.8		GO:GO:0051539,GO:GO:0016992,GO:GO:0009107	AT2G20860.1	lipoic acid synthase 1	LOC_Os04g38330.1	radical SAM enzyme, putative, expressed	1,00	-1.0	1	20	121	140	UAGAUACAUCCGUAUCUAGA	UCUAAAUACGGAUGUAUCUA	Cleavage
tae-miR1120b-3p	Traes_2AL_673CA8FB2.1	PF04603	KOG3329				AT1G69680.1	Mog1/PsbP/DUF1795-like photosystem II reaction center PsbP family protein	LOC_Os04g38610.2	RAN guanine nucleotide release factor, putative, expressed	2,50	-1.0	1	21	493	513	UUCUUAUAUUGUGGGACAGAG	CUCUGUCCCACAAUGUAAGAC	Cleavage
tae-miR1133	Traes_2AL_673CA8FB2.1	PF04603	KOG3329				AT1G69680.1	Mog1/PsbP/DUF1795-like photosystem II reaction center PsbP family protein	LOC_Os04g38610.2	RAN guanine nucleotide release factor, putative, expressed	2,50	-1.0	1	22	553	575	CAUAUACUCCCUCCGUCCGA-AA	UUGUGGGACGGAGGGAGUAUUUG	Cleavage
tae-miR1120a	Traes_2AL_979E96822.1	PF00443		3.4.19.12		GO:GO:0036459,GO:GO:0016579	AT4G39370.3	ubiquitin-specific protease 27	LOC_Os04g34984.1	ubiquitin carboxyl-terminal hydrolase domain containing protein, expressed	2,50	-1.0	1	24	753	776	ACAUUCUUAUAUUAUGAGACGGAG	CUCCAUCCCAUAAUAUAAGAGUGU	Cleavage
tae-miR1130b-3p	Traes_2AL_979E96822.1	PF00443		3.4.19.12		GO:GO:0036459,GO:GO:0016579	AT4G39370.3	ubiquitin-specific protease 27	LOC_Os04g34984.1	ubiquitin carboxyl-terminal hydrolase domain containing protein, expressed	1,00	-1.0	1	21	752	772	UCUUAUAUUAUGGGACGGAGG	CCUCCAUCCCAUAAUAUAAGA	Cleavage
tae-miR1122c-3p	Traes_2AL_979E96822.1	PF00443		3.4.19.12		GO:GO:0036459,GO:GO:0016579	AT4G39370.3	ubiquitin-specific protease 27	LOC_Os04g34984.1	ubiquitin carboxyl-terminal hydrolase domain containing protein, expressed	2,50	-1.0	1	21	752	772	UCUAAUAUUAUGGGACGGAGG	CCUCCAUCCCAUAAUAUAAGA	Cleavage
tae-miR1120c-5p	Traes_2AL_979E96822.1	PF00443		3.4.19.12		GO:GO:0036459,GO:GO:0016579	AT4G39370.3	ubiquitin-specific protease 27	LOC_Os04g34984.1	ubiquitin carboxyl-terminal hydrolase domain containing protein, expressed	2,50	-1.0	1	21	795	815	UAAUAUAAGAACGUUUUUGAC	GUAAAAAACGCUCUUAUAUUA	Translation
tae-miR160	Traes_2AL_C70217249.1	PF06507,PF02362				GO:GO:0009725,GO:GO:0006355,GO:GO:0005634,GO:GO:0003677	AT1G77850.1	auxin response factor 17	LOC_Os04g59430.1	auxin response factor, putative, expressed	1,00	-1.0	1	21	1164	1184	UGCCUGGCUCCCUGUAUGCCA	UGGCAUGCAGGGAGCCAGGCA	Cleavage
tae-miR1134	Traes_2AL_D2CC35E59.1										3,00	-1.0	1	24	512	535	CAACAACAACAAGAAGAAGAAGAU	AUCUUCAUGGUCUUGUUGUUGUUG	Cleavage
tae-miR1134	Traes_2AL_D2CC35E59.2										3,00	-1.0	1	24	620	643	CAACAACAACAAGAAGAAGAAGAU	AUCUUCAUGGUCUUGUUGUUGUUG	Cleavage
tae-miR1134	Traes_2AL_D2CC35E59.3										3,00	-1.0	1	24	509	532	CAACAACAACAAGAAGAAGAAGAU	AUCUUCAUGGUCUUGUUGUUGUUG	Cleavage
tae-miR1134	Traes_2AL_D2CC35E59.4										3,00	-1.0	1	24	611	634	CAACAACAACAAGAAGAAGAAGAU	AUCUUCAUGGUCUUGUUGUUGUUG	Cleavage
tae-miR1134	Traes_2AL_D2CC35E59.5										3,00	-1.0	1	24	509	532	CAACAACAACAAGAAGAAGAAGAU	AUCUUCAUGGUCUUGUUGUUGUUG	Cleavage
tae-miR1134	Traes_2AL_D2CC35E59.6										3,00	-1.0	1	24	509	532	CAACAACAACAAGAAGAAGAAGAU	AUCUUCAUGGUCUUGUUGUUGUUG	Cleavage
tae-miR5384-3p	Traes_2AL_D79C9535C.1	PF00067	KOG0157	1.14.13.109		GO:GO:0055114,GO:GO:0020037,GO:GO:0016705,GO:GO:0005506	AT5G36110.1	cytochrome P450, family 716, subfamily A, polypeptide 1	LOC_Os09g21260.1	cytochrome P450 domain containing protein, expressed	2,50	-1.0	1	21	1250	1270	UGAGCGCGCCGCCGUCGAAUG	CGUUCGGCGGCGGCGCGCGCA	Cleavage
tae-miR9778	Traes_2AS_530CF4042.10	PF00931				GO:GO:0043531	AT3G50950.1	HOPZ-ACTIVATED RESISTANCE 1	LOC_Os04g12830.1	expressed protein	2,50	-1.0	1	21	126	146	UGCAUCAUCUCGAACUCGUCG	CGGCGAGUUCCAGAUGAUGCA	Translation
tae-miR9778	Traes_2AS_530CF4042.2	PF00931				GO:GO:0043531	AT3G50950.1	HOPZ-ACTIVATED RESISTANCE 1	LOC_Os04g12830.1	expressed protein	2,50	-1.0	1	21	126	146	UGCAUCAUCUCGAACUCGUCG	CGGCGAGUUCCAGAUGAUGCA	Translation
tae-miR9778	Traes_2AS_530CF4042.3	PF00931				GO:GO:0043531	AT3G50950.1	HOPZ-ACTIVATED RESISTANCE 1	LOC_Os04g12830.1	expressed protein	2,50	-1.0	1	21	126	146	UGCAUCAUCUCGAACUCGUCG	CGGCGAGUUCCAGAUGAUGCA	Translation
tae-miR1130b-3p	Traes_2AS_6DCA4AADD.1					GO:GO:0016020,GO:GO:0008236,GO:GO:0006508	AT2G31140.1	Peptidase S24/S26A/S26B/S26C family protein	LOC_Os04g01240.1	serine-type peptidase, putative, expressed	1,50	-1.0	1	21	288	308	UCUUAUAUUAUGGGACGGAGG	CCUCCGUCCCAUAAUAUAGGA	Cleavage
tae-miR9664-3p	Traes_2AS_86633353C.1	PF00931	KOG4658		K13457	GO:GO:0043531	AT3G07040.1	NB-ARC domain-containing disease resistance protein	LOC_Os11g46210.1	NB-ARC domain containing protein, expressed	2,50	-1.0	1	21	240	260	UUGCAGUCCUCGAUGUCGUAG	CUACGACAUCGAGGAUUGCAU	Cleavage
tae-miR1130b-3p	Traes_2BL_171777733.1	PF00443		3.4.19.12		GO:GO:0036459,GO:GO:0016579	AT4G39370.3	ubiquitin-specific protease 27	LOC_Os04g34984.1	ubiquitin carboxyl-terminal hydrolase domain containing protein, expressed	0,00	-1.0	1	21	752	772	UCUUAUAUUAUGGGACGGAGG	CCUCCGUCCCAUAAUAUAAGA	Cleavage
tae-miR1122c-3p	Traes_2BL_171777733.1	PF00443		3.4.19.12		GO:GO:0036459,GO:GO:0016579	AT4G39370.3	ubiquitin-specific protease 27	LOC_Os04g34984.1	ubiquitin carboxyl-terminal hydrolase domain containing protein, expressed	1,50	-1.0	1	21	752	772	UCUAAUAUUAUGGGACGGAGG	CCUCCGUCCCAUAAUAUAAGA	Cleavage
tae-miR1120c-5p	Traes_2BL_171777733.1	PF00443		3.4.19.12		GO:GO:0036459,GO:GO:0016579	AT4G39370.3	ubiquitin-specific protease 27	LOC_Os04g34984.1	ubiquitin carboxyl-terminal hydrolase domain containing protein, expressed	2,50	-1.0	1	21	795	815	UAAUAUAAGAACGUUUUUGAC	GUAAAAAACGCUCUUAUAUUA	Translation
tae-miR5384-3p	Traes_2BL_1BE5C9C71.1	PF00067		1.14.13.109		GO:GO:0055114,GO:GO:0020037,GO:GO:0016705,GO:GO:0005506	AT2G42850.1	cytochrome P450, family 718	LOC_Os09g21260.1	cytochrome P450 domain containing protein, expressed	2,50	-1.0	1	21	455	475	UGAGCGCGCCGCCGUCGAAUG	CGUUCGGCGGCGGCGCGCGCA	Cleavage
tae-miR5384-3p	Traes_2BL_1BE5C9C71.2	PF00067				GO:GO:0055114,GO:GO:0020037,GO:GO:0016705,GO:GO:0005506	AT5G36110.1	cytochrome P450, family 716, subfamily A, polypeptide 1	LOC_Os09g21260.1	cytochrome P450 domain containing protein, expressed	2,50	-1.0	1	21	455	475	UGAGCGCGCCGCCGUCGAAUG	CGUUCGGCGGCGGCGCGCGCA	Cleavage
tae-miR1122a	Traes_2BL_40442CE82.2	PF14392,PF00098				GO:GO:0008270,GO:GO:0003676	AT5G52380.1	VASCULAR-RELATED NAC-DOMAIN 6	LOC_Os04g46920.1	zinc knuckle domain containing protein, expressed	1,50	-1.0	1	20	703	722	UAGAUACAUCCGUAUCUAGA	UCUAGAUACAGAUGUAUCUA	Translation
tae-miR164	Traes_2BL_6AEE8AC28.1	PF02365				GO:GO:0006355,GO:GO:0003677	AT5G61430.1	NAC domain containing protein 100	LOC_Os04g38720.1	no apical meristem protein, putative, expressed	1,50	-1.0	1	21	680	700	UGGAGAAGCAGGGCACGUGCA	CGCACGUGACCUGCUUCUCCA	Cleavage
tae-miR164	Traes_2BL_6AEE8AC28.2	PF02365				GO:GO:0006355,GO:GO:0003677	AT5G61430.1	NAC domain containing protein 100	LOC_Os04g38720.1	no apical meristem protein, putative, expressed	1,50	-1.0	1	21	672	692	UGGAGAAGCAGGGCACGUGCA	CGCACGUGACCUGCUUCUCCA	Cleavage
tae-miR164	Traes_2BL_6AEE8AC28.3	PF02365				GO:GO:0006355,GO:GO:0003677	AT5G61430.1	NAC domain containing protein 100	LOC_Os04g38720.1	no apical meristem protein, putative, expressed	1,50	-1.0	1	21	633	653	UGGAGAAGCAGGGCACGUGCA	CGCACGUGACCUGCUUCUCCA	Cleavage
tae-miR1122b-3p	Traes_2BL_7141904F2.2	PF03097					AT1G73390.1	Endosomal targeting BRO1-like domain-containing protein	LOC_Os04g44530.3	expressed protein	2,50	-1.0	1	21	1058	1078	AGACUUAUAUGUAGGAACGGA	UCCGUUCUUAAAUAUAAGUCU	Translation
tae-miR1122a	Traes_2BL_7141904F2.2	PF03097					AT1G73390.1	Endosomal targeting BRO1-like domain-containing protein	LOC_Os04g44530.3	expressed protein	2,50	-1.0	1	20	1098	1117	UAGAUACAUCCGUAUCUAGA	ACUACAUACGGAUGUAUAUA	Cleavage
tae-miR1134	Traes_2BL_8339D35AC.1										3,00	-1.0	1	24	509	532	CAACAACAACAAGAAGAAGAAGAU	AUCUUCAUGGUCUUGUUGUUGUUG	Cleavage
tae-miR1134	Traes_2BL_8339D35AC.2										3,00	-1.0	1	24	512	535	CAACAACAACAAGAAGAAGAAGAU	AUCUUCAUGGUCUUGUUGUUGUUG	Cleavage
tae-miR1134	Traes_2BL_8339D35AC.3										3,00	-1.0	1	24	509	532	CAACAACAACAAGAAGAAGAAGAU	AUCUUCAUGGUCUUGUUGUUGUUG	Cleavage
tae-miR1134	Traes_2BL_8339D35AC.4										3,00	-1.0	1	24	302	325	CAACAACAACAAGAAGAAGAAGAU	AUCUUCAUGGUCUUGUUGUUGUUG	Cleavage
tae-miR1134	Traes_2BL_8339D35AC.5										3,00	-1.0	1	24	509	532	CAACAACAACAAGAAGAAGAAGAU	AUCUUCAUGGUCUUGUUGUUGUUG	Cleavage
tae-miR1135	Traes_2BL_A4AE931B0.1						AT2G02090.1	SNF2 domain-containing protein / helicase domain-containing protein	LOC_Os04g47830.1	SNF2 family N-terminal domain containing protein, expressed	2,00	-1.0	1	24	1154	1177	CUGCGACAAGUAAUUCCGAACGGA	UCUGUCCGGAAUUACUUGUCGCAA	Cleavage
tae-miR1122b-3p	Traes_2BL_FF8002798.2	PF13414					AT1G56090.1	Tetratricopeptide repeat (TPR)-like superfamily protein	LOC_Os04g52580.1	tetratricopeptide repeat domain containing protein, expressed	2,50	-1.0	1	21	889	909	AGACUUAUAUGUAGGAACGGA	UCGGUUCCUAAAUAUAAGUCU	Translation
tae-miR9778	Traes_2BS_0EFFAAE28.1	PF00931				GO:GO:0043531	AT3G07040.1	NB-ARC domain-containing disease resistance protein	LOC_Os08g14850.1	resistance protein, putative, expressed	2,50	-1.0	1	21	126	146	UGCAUCAUCUCGAACUCGUCG	CGGCGAGUUCCAGAUGAUGCA	Translation
tae-miR9778	Traes_2BS_0EFFAAE28.2	PF00931				GO:GO:0043531	AT3G07040.1	NB-ARC domain-containing disease resistance protein	LOC_Os08g14850.1	resistance protein, putative, expressed	2,50	-1.0	1	21	126	146	UGCAUCAUCUCGAACUCGUCG	CGGCGAGUUCCAGAUGAUGCA	Translation
tae-miR9662a-3p	Traes_2BS_257C5B099.1	PF02536				GO:GO:0006355,GO:GO:0005739,GO:GO:0003690	AT5G07900.1	Mitochondrial transcription termination factor family protein	LOC_Os06g12100.1	mTERF family protein, expressed	2,00	-1.0	1	21	66	86	UUGAACAUCCCAGAGCCACCG	CCGUGGUUCUGGGAUGUUCAG	Cleavage
tae-miR9662b-3p	Traes_2BS_257C5B099.1	PF02536				GO:GO:0006355,GO:GO:0005739,GO:GO:0003690	AT5G07900.1	Mitochondrial transcription termination factor family protein	LOC_Os06g12100.1	mTERF family protein, expressed	2,00	-1.0	1	21	65	85	UGAACAUCCCAGAGCCACCGG	CCCGUGGUUCUGGGAUGUUCA	Cleavage
tae-miR9662a-3p	Traes_2BS_257C5B0991.1	PF02536				GO:GO:0006355,GO:GO:0005739,GO:GO:0003690	AT5G07900.1	Mitochondrial transcription termination factor family protein	LOC_Os06g12100.1	mTERF family protein, expressed	2,00	-1.0	1	21	66	86	UUGAACAUCCCAGAGCCACCG	CCGUGGUUCUGGGAUGUUCAG	Cleavage
tae-miR9662b-3p	Traes_2BS_257C5B0991.1	PF02536				GO:GO:0006355,GO:GO:0005739,GO:GO:0003690	AT5G07900.1	Mitochondrial transcription termination factor family protein	LOC_Os06g12100.1	mTERF family protein, expressed	2,00	-1.0	1	21	65	85	UGAACAUCCCAGAGCCACCGG	CCCGUGGUUCUGGGAUGUUCA	Cleavage
tae-miR5048-5p	Traes_2BS_47E2D6444.1	PF07714		2.7.11.1		GO:GO:0006468,GO:GO:0004672	AT4G04570.2	cysteine-rich RLK (RECEPTOR-like protein kinase) 40	LOC_Os04g30030.1	cysteine-rich receptor-like protein kinase 12 precursor, putative, expressed	2,50	-1.0	1	22	221	242	UUUGCAGGUUUUAGGUCUAAGU	ACCUGGACCUAAAACCUGCGAA	Cleavage
tae-miR5048-5p	Traes_2BS_47E2D6444.2	PF00069	KOG1187	2.7.11.1		GO:GO:0006468,GO:GO:0005524,GO:GO:0004672	AT4G38830.1	cysteine-rich RLK (RECEPTOR-like protein kinase) 26	LOC_Os04g30030.1	cysteine-rich receptor-like protein kinase 12 precursor, putative, expressed	2,50	-1.0	1	22	959	980	UUUGCAGGUUUUAGGUCUAAGU	ACCUGGACCUAAAACCUGCGAA	Cleavage
tae-miR5048-5p	Traes_2BS_47E2D6444.3	PF00069	KOG1187	2.7.11.1		GO:GO:0006468,GO:GO:0005524,GO:GO:0004672	AT4G38830.1	cysteine-rich RLK (RECEPTOR-like protein kinase) 26	LOC_Os04g30030.1	cysteine-rich receptor-like protein kinase 12 precursor, putative, expressed	2,50	-1.0	1	22	947	968	UUUGCAGGUUUUAGGUCUAAGU	ACCUGGACCUAAAACCUGCGAA	Cleavage
tae-miR5048-5p	Traes_2BS_47E2D6444.4	PF07714		2.7.11.1		GO:GO:0006468,GO:GO:0004672	AT4G23160.1	cysteine-rich RLK (RECEPTOR-like protein kinase) 8	LOC_Os04g30030.1	cysteine-rich receptor-like protein kinase 12 precursor, putative, expressed	2,50	-1.0	1	22	221	242	UUUGCAGGUUUUAGGUCUAAGU	ACCUGGACCUAAAACCUGCGAA	Cleavage
tae-miR9664-3p	Traes_2BS_5045C640C.1	PF00931	KOG4658		K13457	GO:GO:0043531	AT3G07040.1	NB-ARC domain-containing disease resistance protein	LOC_Os11g46210.1	NB-ARC domain containing protein, expressed	2,50	-1.0	1	21	240	260	UUGCAGUCCUCGAUGUCGUAG	CUACGACAUCGAGGAUUGCAU	Cleavage
tae-miR9664-3p	Traes_2BS_5045C640C.2	PF00931	KOG4658		K13457	GO:GO:0043531	AT3G07040.1	NB-ARC domain-containing disease resistance protein	LOC_Os11g46210.1	NB-ARC domain containing protein, expressed	2,50	-1.0	1	21	240	260	UUGCAGUCCUCGAUGUCGUAG	CUACGACAUCGAGGAUUGCAU	Cleavage
tae-miR9664-3p	Traes_2BS_5045C640C.5	PF00931	KOG4658		K13457	GO:GO:0043531	AT3G07040.1	NB-ARC domain-containing disease resistance protein	LOC_Os11g46210.1	NB-ARC domain containing protein, expressed	2,50	-1.0	1	21	240	260	UUGCAGUCCUCGAUGUCGUAG	CUACGACAUCGAGGAUUGCAU	Cleavage
tae-miR1120c-5p	Traes_2BS_5C64FC44A.1	PF07714	KOG1187	2.7.11.1		GO:GO:0006468,GO:GO:0004672	AT4G23140.1	cysteine-rich RLK (RECEPTOR-like protein kinase) 6	LOC_Os07g35580.1	TKL_IRAK_DUF26-lc.20 - DUF26 kinases have homology to DUF26 containing loci, expressed	0,00	-1.0	1	21	21	41	UAAUAUAAGAACGUUUUUGAC	GUCAAAAACGUUCUUAUAUUA	Cleavage
tae-miR1120c-5p	Traes_2BS_5C64FC44A.2	PF07714		2.7.11.1		GO:GO:0006468,GO:GO:0004672	AT4G23180.1	cysteine-rich RLK (RECEPTOR-like protein kinase) 10	LOC_Os07g35580.1	TKL_IRAK_DUF26-lc.20 - DUF26 kinases have homology to DUF26 containing loci, expressed	0,00	-1.0	1	21	2	22	UAAUAUAAGAACGUUUUUGAC	GUCAAAAACGUUCUUAUAUUA	Cleavage
tae-miR1130b-3p	Traes_2BS_5CDCD5152.1	PF04178	KOG1743			GO:GO:0016192	AT5G01430.1	Got1/Sft2-like vescicle transport protein family	LOC_Os03g11100.1	vesicle transport protein GOT1B, putative, expressed	2,00	-1.0	1	20	1	21	UCUUAUAUUAUGGGA-CGGAG	GUCCGAUCCCAUAAUAUAAGA	Cleavage
tae-miR1121	Traes_2BS_6249E432F.1	PF01426				GO:GO:0003682	AT4G22140.1	PHD finger family protein / bromo-adjacent homology (BAH) domain-containing protein	LOC_Os07g08880.1	ES43 protein, putative, expressed	0,00	-1.0	1	22	538	559	AGUAGUGAUCUAAACGCUCUUA	UAAGAGCGUUUAGAUCACUACU	Cleavage
tae-miR1121	Traes_2BS_6249E432F.2	PF01426				GO:GO:0003682	AT4G22140.1	PHD finger family protein / bromo-adjacent homology (BAH) domain-containing protein	LOC_Os07g08880.1	ES43 protein, putative, expressed	0,00	-1.0	1	22	538	559	AGUAGUGAUCUAAACGCUCUUA	UAAGAGCGUUUAGAUCACUACU	Cleavage

tae-miR1121	Traes_2BS_6249E432F.3	PF01426				GO:GO:0003682	AT4G39100.1	PHD finger family protein / bromo-adjacent homology (BAH) domain-containing protein	LOC_Os07g08880.1	ES43 protein, putative, expressed	0,00	-1.0	1	22	448	469	AGUAGUGAUCUAAACGCUCUUA	UAAGAGCGUUUAGAUCACUACU	Cleavage
tae-miR1133	Traes_2BS_67D862F4A.1	PF01428	KOG3183			GO:GO:0008270	AT2G41835.1	zinc finger (C2H2 type, AN1-like) family protein	LOC_Os07g38240.1	ZOS7-05 - C2H2 zinc finger protein, expressed	2,50	-1.0	1	22	30	51	CAUAUACUCCCUCCGUCCGAAA	UUUGGGACGGAGGGAGUAUAAG	Cleavage
tae-miR9662a-3p	Traes_2BS_74269A4C5.1	PF02536				GO:GO:0006355,GO:GO:0005739,GO:GO:0003690	AT5G07900.1	Mitochondrial transcription termination factor family protein	LOC_Os06g12100.1	mTERF family protein, expressed	2,00	-1.0	1	21	189	209	UUGAACAUCCCAGAGCCACCG	CCGUGGUUCUGGGAUGUUCAG	Cleavage
tae-miR9662b-3p	Traes_2BS_74269A4C5.1	PF02536				GO:GO:0006355,GO:GO:0005739,GO:GO:0003690	AT5G07900.1	Mitochondrial transcription termination factor family protein	LOC_Os06g12100.1	mTERF family protein, expressed	2,00	-1.0	1	21	188	208	UGAACAUCCCAGAGCCACCGG	CCCGUGGUUCUGGGAUGUUCA	Cleavage
tae-miR1128	Traes_2BS_7AEB138EB.1	PF01397,PF03936		4.2.3.104		GO:GO:0016829,GO:GO:0010333,GO:GO:0008152,GO:GO:0000287	AT5G23960.2	terpene synthase 21	LOC_Os07g11790.1	terpene synthase, putative, expressed	1,50	-1.0	1	21	84	104	UACUACUCCCUCCGUCCGAAA	UUUCGGACGGAGGGAGUACUA	Cleavage
tae-miR9661-5p	Traes_2BS_8281050F1.1										3,00	-1.0	1	21	845	865	UGAAGUAGAGCAGGGACCUCA	GACCGUUCCUGCUCUACUUCA	Cleavage
tae-miR9661-5p	Traes_2BS_8281050F11.1										3,00	-1.0	1	21	845	865	UGAAGUAGAGCAGGGACCUCA	GACCGUUCCUGCUCUACUUCA	Cleavage
tae-miR1122b-3p	Traes_2BS_A20CCBEC3.1	PF05368		1.23.1.1,1.23.1.3			AT1G32100.1	pinoresinol reductase 1	LOC_Os12g16220.2	nmrA-like family domain containing protein, expressed	2,50	-1.0	1	21	389	409	AGACUUAUAUGUAGGAACGGA	UCUGUUCCUAAAUAUAAGUCU	Translation
tae-miR1128	Traes_2BS_B3A88F254.2	PF00479,PF02781		1.1.1.49	K00036	GO:GO:0055114,GO:GO:0050661,GO:GO:0006006,GO:GO:0004345	AT5G13110.1	glucose-6-phosphate dehydrogenase 2	LOC_Os07g22350.1	glucose-6-phosphate 1-dehydrogenase, chloroplast precursor, putative, expressed	2,00	-1.0	1	21	88	109	UACUACUCCCUCCGUCC-GAAA	UUUCCGGACGGAGGGAGUAGUA	Cleavage
tae-miR1128	Traes_2BS_B3A88F254.3	PF00479,PF02781		1.1.1.49	K00036	GO:GO:0055114,GO:GO:0050661,GO:GO:0006006,GO:GO:0004345	AT5G13110.1	glucose-6-phosphate dehydrogenase 2	LOC_Os07g22350.1	glucose-6-phosphate 1-dehydrogenase, chloroplast precursor, putative, expressed	2,00	-1.0	1	21	88	109	UACUACUCCCUCCGUCC-GAAA	UUUCCGGACGGAGGGAGUAGUA	Cleavage
tae-miR1127a	Traes_2BS_C00FC92D4.2						AT2G18910.1	hydroxyproline-rich glycoprotein family protein	LOC_Os02g26370.1	wiskott-Aldrich syndrome protein family member 2, putative, expressed	1,50	-1.0	1	19	497	515	UCCUUCCGUUCGGAAUUAC	GUAAUUCCGAACGGAGGGA	Cleavage
tae-miR1117	Traes_2BS_C5AF5419E.1			3.4.23.47,3.1.26.13,3.1.13.2,2.7.7.49,2.7.7.7					LOC_Os02g49670.1	zinc knuckle family protein, expressed	0,00	-1.0	1	24	926	949	UAGUACCGGUUCGUGGCACGAACC	GGUUCGUGCCACGAACCGGUACUA	Cleavage
tae-miR9662a-3p	Traes_2BS_DDF26C784.1	PF02536				GO:GO:0006355,GO:GO:0005739,GO:GO:0003690	AT5G07900.1	Mitochondrial transcription termination factor family protein	LOC_Os06g12100.1	mTERF family protein, expressed	2,00	-1.0	1	21	459	479	UUGAACAUCCCAGAGCCACCG	CCGUGGUUCUGGGAUGUUCAG	Cleavage
tae-miR9662b-3p	Traes_2BS_DDF26C784.1	PF02536				GO:GO:0006355,GO:GO:0005739,GO:GO:0003690	AT5G07900.1	Mitochondrial transcription termination factor family protein	LOC_Os06g12100.1	mTERF family protein, expressed	2,00	-1.0	1	21	458	478	UGAACAUCCCAGAGCCACCGG	CCCGUGGUUCUGGGAUGUUCA	Cleavage
tae-miR5384-3p	Traes_2BS_EBCD10293.1	PF02225,PF00082		3.4.21.25		GO:GO:0006508,GO:GO:0004252	AT1G04110.1	Subtilase family protein	LOC_Os04g10360.1	OsSub40 - Putative Subtilisin homologue, expressed	2,50	-1.0	1	21	116	136	UGAGCGCGCCGCCGUCGAAUG	UGUUCGCCGACGGCGCGCUCA	Cleavage
tae-miR5384-3p	Traes_2BS_EBCD10293.2	PF02225,PF00082		3.4.21.25		GO:GO:0006508,GO:GO:0004252	AT1G04110.1	Subtilase family protein	LOC_Os04g10360.1	OsSub40 - Putative Subtilisin homologue, expressed	2,50	-1.0	1	21	116	136	UGAGCGCGCCGCCGUCGAAUG	UGUUCGCCGACGGCGCGCUCA	Cleavage
tae-miR1130b-3p	Traes_2DL_10B2965EF.8	PF00443		3.4.19.12		GO:GO:0036459,GO:GO:0016579	AT4G39370.2	ubiquitin-specific protease 27	LOC_Os04g34984.3	ubiquitin carboxyl-terminal hydrolase domain containing protein, expressed	0,00	-1.0	1	21	752	772	UCUUAUAUUAUGGGACGGAGG	CCUCCGUCCCAUAAUAUAAGA	Cleavage
tae-miR1122c-3p	Traes_2DL_10B2965EF.8	PF00443		3.4.19.12		GO:GO:0036459,GO:GO:0016579	AT4G39370.2	ubiquitin-specific protease 27	LOC_Os04g34984.3	ubiquitin carboxyl-terminal hydrolase domain containing protein, expressed	1,50	-1.0	1	21	752	772	UCUAAUAUUAUGGGACGGAGG	CCUCCGUCCCAUAAUAUAAGA	Cleavage
tae-miR1120c-5p	Traes_2DL_10B2965EF.8	PF00443		3.4.19.12		GO:GO:0036459,GO:GO:0016579	AT4G39370.2	ubiquitin-specific protease 27	LOC_Os04g34984.3	ubiquitin carboxyl-terminal hydrolase domain containing protein, expressed	1,50	-1.0	1	21	795	815	UAAUAUAAGAACGUUUUUGAC	GUCAAAAACGCUCUUAUAUUA	Translation
tae-miR1130b-3p	Traes_2DL_3C9129432.1	PF00656				GO:GO:0006508,GO:GO:0004197	AT1G79340.1	metacaspase 4	LOC_Os01g58580.1	ICE-like protease p20 domain containing protein, putative, expressed	1,50	-1.0	1	21	164	184	UCUUAUAUUAUGGGACGGAGG	CUUCCGUCCCAUAAUGUAAGA	Cleavage
tae-miR1133	Traes_2DL_4481C0380.4										2,50	-1.0	1	22	1	22	CAUAUACUCCCUCCGUCCGAAA	UUUGGGACGGAGGGAGUACAUG	Cleavage
tae-miR1137a	Traes_2DL_4F8C87C42,5	PF04499	KOG2073				AT1G30470.1	SIT4 phosphatase-associated family protein	LOC_Os04g52940.1	SIT4 phosphatase-associated protein domain containing protein, expressed	1,00	-1.0	1	20	1791	1810	UAGUACAAAGUUGAGUCAUC	GAUGACCCAACUUUGAGUCAUC	Cleavage
tae-miR1127b-3p	Traes_2DL_4F8C87C42,5	PF04499	KOG2073				AT1G30470.1	SIT4 phosphatase-associated family protein	LOC_Os04g52940.1	SIT4 phosphatase-associated protein domain containing protein, expressed	1,50	-1.0	1	21	1822	1842	ACAAGUAUUUCUGGACGGAGG	CCUCCGUCCGGAAAUACUUGU	Cleavage
tae-miR1137b-5p	Traes_2DL_4F8C87C42,5	PF04499	KOG2073				AT1G30470.1	SIT4 phosphatase-associated family protein	LOC_Os04g52940.1	SIT4 phosphatase-associated protein domain containing protein, expressed	1,50	-1.0	1	21	1944	1964	UCCGUUCCAGAAUAGAUGACC	GGUCAUCUAUUUUGGAACGGA	Translation
tae-miR1122a	Traes_2DL_4F8C87C42.6	PF04499					AT1G30470.1	SIT4 phosphatase-associated family protein	LOC_Os04g52940.1	SIT4 phosphatase-associated protein domain containing protein, expressed	2,50	-1.0	1	20	1750	1769	UAGAUACAUCCGUAUCUAGA	ACUACAUACGGAUGUAUAUA	Cleavage
tae-miR1121	Traes_2DL_74E241841.2	PF01426				GO:GO:0003682	AT4G39100.1	PHD finger family protein / bromo-adjacent homology (BAH) domain-containing protein	LOC_Os07g08880.1	ES43 protein, putative, expressed	2,50	-1.0	1	22	448	469	AGUAGUGAUCUAAACGCUCUUA	UAAGAGUGUUUGGAUCACUACU	Translation
tae-miR1130b-3p	Traes_2DL_8F04980F0.5	PF03405		1.14.19.2		GO:GO:0055114,GO:GO:0045300,GO:GO:0006631	AT3G02630.1	Plant stearoyl-acyl-carrier-protein desaturase family protein	LOC_Os04g31070.1	acyl-desaturase, chloroplast precursor, putative, expressed	2,50	-1.0	1	21	468	488	UCUUAUAUUAUGGGACGGAGG	CCUCCGUCCCCUAAUAUAAGG	Translation
tae-miR1128	Traes_2DL_9AD532712.8	PF15613,PF15614					AT5G08630.1	DDT domain-containing protein	LOC_Os04g35864.1	DDT domain-containing protein, putative, expressed	2,00	-1.0	1	21	305	325	UACUACUCCCUCCGUCCGAAA	GUCCGGACGGAGGGAGUAGUU	Cleavage
tae-miR1122a	Traes_2DL_B3CD23EBC.1	PF05496,PF00004				GO:GO:0009378,GO:GO:0006310,GO:GO:0006281,GO:GO:0005524	AT1G03000.1	peroxin 6	LOC_Os04g52690.1	AAA family ATPase, putative, expressed	2,50	-1.0	1	20	9	28	UAGAUACAUCCGUAUCUAGA	ACUACAUACGGAUGUAUAUA	Cleavage
tae-miR9662a-3p	Traes_2DL_BE754F0C1.1	PF02536	KOG1267		K15032	GO:GO:0006355,GO:GO:0005739,GO:GO:0003690	AT5G07900.1	Mitochondrial transcription termination factor family protein	LOC_Os06g12100.1	mTERF family protein, expressed	1,00	-1.0	1	21	687	707	UUGAACAUCCCAGAGCCACCG	CCGUGGCUCUGGGAUGUUCAG	Cleavage
tae-miR9662b-3p	Traes_2DL_BE754F0C1.1	PF02536	KOG1267		K15032	GO:GO:0006355,GO:GO:0005739,GO:GO:0003690	AT5G07900.1	Mitochondrial transcription termination factor family protein	LOC_Os06g12100.1	mTERF family protein, expressed	1,00	-1.0	1	21	686	706	UGAACAUCCCAGAGCCACCGG	CCCGUGGCUCUGGGAUGUUCA	Cleavage
tae-miR1134	Traes_2DL_E8A7ABB5D.1										3,00	-1.0	1	24	509	532	CAACAACAACAAGAAGAAGAAGAU	AUCUUCAUGGUCUUGUUGUUGUUG	Cleavage
tae-miR1134	Traes_2DL_E8A7ABB5D.2										3,00	-1.0	1	24	629	652	CAACAACAACAAGAAGAAGAAGAU	AUCUUCAUGGUCUUGUUGUUGUUG	Cleavage
tae-miR1134	Traes_2DL_E8A7ABB5D.3										3,00	-1.0	1	24	614	637	CAACAACAACAAGAAGAAGAAGAU	AUCUUCAUGGUCUUGUUGUUGUUG	Cleavage
tae-miR1134	Traes_2DL_E8A7ABB5D.4										3,00	-1.0	1	24	512	535	CAACAACAACAAGAAGAAGAAGAU	AUCUUCAUGGUCUUGUUGUUGUUG	Cleavage
tae-miR1134	Traes_2DL_E8A7ABB5D.5										3,00	-1.0	1	24	509	532	CAACAACAACAAGAAGAAGAAGAU	AUCUUCAUGGUCUUGUUGUUGUUG	Cleavage
tae-miR1134	Traes_2DL_E8A7ABB5D.6										3,00	-1.0	1	24	509	532	CAACAACAACAAGAAGAAGAAGAU	AUCUUCAUGGUCUUGUUGUUGUUG	Cleavage
tae-miR1134	Traes_2DL_E8A7ABB5D.7										3,00	-1.0	1	24	91	114	CAACAACAACAAGAAGAAGAAGAU	AUCUUCAUGGUCUUGUUGUUGUUG	Cleavage
tae-miR1134	Traes_2DL_E8A7ABB5D.8										3,00	-1.0	1	24	77	100	CAACAACAACAAGAAGAAGAAGAU	AUCUUCAUGGUCUUGUUGUUGUUG	Cleavage
tae-miR1134	Traes_2DL_E8A7ABB5D.9										3,00	-1.0	1	24	28	51	CAACAACAACAAGAAGAAGAAGAU	AUCUUCAUGGUCUUGUUGUUGUUG	Cleavage
tae-miR160	Traes_2DL_EF12C0B89.2						AT1G77850.1	auxin response factor 17	LOC_Os04g59430.1	auxin response factor, putative, expressed	1,00	-1.0	1	21	225	245	UGCCUGGCUCCCUGUAUGCCA	UGGCAUGCAGGGAGCCAGGCA	Cleavage
tae-miR9664-3p	Traes_2DS_480CFDFE4.1	PF01637	KOG4658		K13457	GO:GO:0005524	AT3G07040.1	NB-ARC domain-containing disease resistance protein	LOC_Os11g46210.1	NB-ARC domain containing protein, expressed	2,50	-1.0	1	21	243	263	UUGCAGUCCUCGAUGUCGUAG	CUACGACAUCGAGGAUUGCAU	Cleavage
tae-miR9664-3p	Traes_2DS_480CFDFE4.2	PF01637				GO:GO:0005524	AT3G07040.1	NB-ARC domain-containing disease resistance protein	LOC_Os11g46210.1	NB-ARC domain containing protein, expressed	2,50	-1.0	1	21	259	279	UUGCAGUCCUCGAUGUCGUAG	CUACGACAUCGAGGAUUGCAU	Cleavage
tae-miR5049-3p	Traes_2DS_851973269.7										1,50	-1.0	1	21	178	198	AAUAUGGAUCGGAGGGAGUAC	GUACUCCCUCCGAUUCAUAUU	Cleavage
tae-miR1122b-3p	Traes_2DS_9B301D4F8.2	PF00069	KOG0580,KOG2345	2.7.11.1		GO:GO:0006468,GO:GO:0005524,GO:GO:0004672	AT5G18500.2	Protein kinase superfamily protein	LOC_Os11g39450.1	cysteine-rich receptor-like protein kinase 7 precursor, putative, expressed	1,50	-1.0	1	21	843	863	AGACUUAUAUGUAGGAACGGA	UCCGUUCCUAAAUAUAAGUCU	Translation
tae-miR1123	Traes_2DS_C866A54F1.2						AT4G36730.1	G-box binding factor 1	LOC_Os07g10890.1	transcription factor, putative, expressed	1,00	-1.0	1	23	792	814	UCCGUGAGACCUGGUCUCAUAGA	UCUAUGAGACCAGGUCUCACGGG	Cleavage
tae-miR1123	Traes_2DS_C866A54F1.4						AT4G36730.1	G-box binding factor 1	LOC_Os07g10890.1	transcription factor, putative, expressed	1,00	-1.0	1	23	786	808	UCCGUGAGACCUGGUCUCAUAGA	UCUAUGAGACCAGGUCUCACGGG	Cleavage
tae-miR9778	Traes_2DS_C9635AFF7.1	PF00931				GO:GO:0043531	AT3G07040.1	NB-ARC domain-containing disease resistance protein	LOC_Os08g14850.1	resistance protein, putative, expressed	2,50	-1.0	1	21	126	146	UGCAUCAUCUCGAACUCGUCG	CGGCGAGUUCCAGAUGAUGCA	Translation
tae-miR9778	Traes_2DS_C9635AFF7.2	PF00931				GO:GO:0043531	AT3G07040.1	NB-ARC domain-containing disease resistance protein	LOC_Os08g14850.1	resistance protein, putative, expressed	2,50	-1.0	1	21	105	125	UGCAUCAUCUCGAACUCGUCG	CGGCGAGUUCCAGAUGAUGCA	Translation
tae-miR1117	Traes_3AL_018D4C27C.1	PF07714,PF12819		2.7.11.1		GO:GO:0006468,GO:GO:0004672	AT3G51550.1	Malectin/receptor-like protein kinase family protein	LOC_Os05g25430.2	receptor-like protein kinase At3g46290 precursor, putative, expressed	1,50	-1.0	1	24	1609	1632	UAGUACCGGUUCGUGGCACGAACC	GGUUCGUGCCACGAACCGGUGCUA	Cleavage
tae-miR1133	Traes_3AL_30294D0A5.1			1.1.1.8		GO:GO:0055114,GO:GO:0009331,GO:GO:0006072,GO:GO:0004367	AT2G41540.3	6-phosphogluconate dehydrogenase family protein	LOC_Os01g71280.1	glycerol-3-phosphate dehydrogenase, putative, expressed	2,50	-1.0	1	22	392	413	CAUAUACUCCCUCCGUCCGAAA	UUUAGGACGGAGGGAGUAUUUG	Cleavage
tae-miR1133	Traes_3AL_386795528.2	PF00249					AT5G02320.2	myb domain protein 3r-5	LOC_Os01g62410.1	MYB family transcription factor, putative, expressed	0,00	-1.0	1	22	39	60	CAUAUACUCCCUCCGUCCGAAA	UUUCGGACGGAGGGAGUAUAUG	Cleavage
tae-miR1122a	Traes_3AL_5E5C8D264.2								LOC_Os05g46470.1	expressed protein	1,00	-1.0	1	20	18	37	UAGAUACAUCCGUAUCUAGA	UCUAGACACGGAUGUAUCUA	Cleavage
tae-miR1137b-5p	Traes_3AL_7A4D34425.2						AT1G29820.2	Magnesium transporter CorA-like family protein	LOC_Os01g47460.1	expressed protein	2,50	-1.0	1	21	1006	1026	UCCGUUCCAGAAUAGAUGACC	UAUCAUUUAUUCAGGAACGGA	Cleavage
tae-miR1122a	Traes_3AL_B9EDE61D1.1	PF06017				GO:GO:0016459,GO:GO:0003774	AT5G53310.1	myosin heavy chain-related	LOC_Os01g43360.1	myosin-Ie, putative, expressed	2,50	-1.0	1	20	4	23	UAGAUACAUCCGUAUCUAGA	ACUACAUACGGAUGUAUAUA	Cleavage
tae-miR1122a	Traes_3AL_B9EDE61D1.2	PF06017				GO:GO:0016459,GO:GO:0003774	AT5G53310.1	myosin heavy chain-related	LOC_Os01g43360.1	myosin-Ie, putative, expressed	2,50	-1.0	1	20	4	23	UAGAUACAUCCGUAUCUAGA	ACUACAUACGGAUGUAUAUA	Cleavage
tae-miR5049-3p	Traes_3AL_CF1A4ACDE.1	PF03759				GO:GO:0005089	AT1G52240.1	RHO guanyl-nucleotide exchange factor 11	LOC_Os01g55520.1	ATROPGEF7/ROPGEF7, putative, expressed	1,00	-1.0	1	21	917	937	AAUAUGGAUCGGAGGGAGUAC	UCCCUCCCUCCGAUCCAUAUU	Cleavage
tae-miR159b	Traes_3AL_F09C7CA19.1	PF00249					AT5G06100.1	myb domain protein 33	LOC_Os01g59660.2	MYB family transcription factor, putative, expressed	2,50	-1.0	1	21	620	640	UUUGGAUUGAAGGGAGCUCUG	UGGAGCUCCCUUCACUCCAAG	Cleavage
tae-miR159a	Traes_3AL_F09C7CA19.1	PF00249					AT5G06100.1	myb domain protein 33	LOC_Os01g59660.2	MYB family transcription factor, putative, expressed	2,50	-1.0	1	21	620	640	UUUGGAUUGAAGGGAGCUCUG	UGGAGCUCCCUUCACUCCAAG	Cleavage
tae-miR1121	Traes_3AL_F2EDB3661.7										2,00	-1.0	1	22	224	245	AGUAGUGAUCUAAACGCUCUUA	CCUCCGCGUUUAGAUCACUACU	Cleavage
tae-miR1133	Traes_3AL_FA6F160DF.7										3,00	-1.0	1	22	142	164	CAUAUACUCCCUCCG-UCCGAAA	UUUCGGAACGGAGGGAGUAUAUU	Cleavage
tae-miR5200	Traes_3AS_2A0765E10.1	PF14531		2.7.11.1			AT1G62400.1	Protein kinase superfamily protein	LOC_Os01g01740.1	protein kinase domain containing protein, expressed	2,50	-1.0	1	21	177	197	UGUAGAUACUCCCUAAGGCUU	UUCCCUUAGGAAGUAUCUACA	Translation
tae-miR5200	Traes_3AS_2A0765E10.2	PF14531		2.7.11.1			AT1G62400.1	Protein kinase superfamily protein	LOC_Os01g01740.1	protein kinase domain containing protein, expressed	2,50	-1.0	1	21	66	86	UGUAGAUACUCCCUAAGGCUU	UUCCCUUAGGAAGUAUCUACA	Translation
tae-miR5200	Traes_3AS_2A0765E10.3	PF14531		2.7.11.1			AT1G62400.1	Protein kinase superfamily protein	LOC_Os01g01740.1	protein kinase domain containing protein, expressed	2,50	-1.0	1	21	12	32	UGUAGAUACUCCCUAAGGCUU	UUCCCUUAGGAAGUAUCUACA	Translation
tae-miR9674b-5p	Traes_3AS_6CB1DBD28.1	PF01535,PF13041					AT3G09040.1	Pentatricopeptide repeat (PPR) superfamily protein	LOC_Os04g43430.3	pentatricopeptide, putative, expressed	2,00	-1.0	1	21	429	449	AUAGCAUCAUCCAUCCUACCC	GGGUAGGCUGGAUGAUGCUAG	Cleavage
tae-miR1121	Traes_3AS_A466B9ECC.1	PF13424					AT3G54360.1	zinc ion binding	LOC_Os01g01420.2	expressed protein	0,00	-1.0	1	22	725	746	AGUAGUGAUCUAAACGCUCUUA	UAAGAGCGUUUAGAUCACUACU	Cleavage
tae-miR6197-5p	Traes_3AS_A466B9ECC.1	PF13424					AT3G54360.1	zinc ion binding	LOC_Os01g01420.2	expressed protein	2,50	-1.0	1	21	763	783	UCUGUAAACAAAUGUAGGACG	GCUCUUACAUUUCUUUACAGA	Cleavage
tae-miR1121	Traes_3AS_A466B9ECC.2	PF13424					AT3G54360.1	zinc ion binding	LOC_Os01g01420.2	expressed protein	0,00	-1.0	1	22	725	746	AGUAGUGAUCUAAACGCUCUUA	UAAGAGCGUUUAGAUCACUACU	Cleavage
tae-miR6197-5p	Traes_3AS_A466B9ECC.2	PF13424					AT3G54360.1	zinc ion binding	LOC_Os01g01420.2	expressed protein	2,50	-1.0	1	21	763	783	UCUGUAAACAAAUGUAGGACG	GCUCUUACAUUUCUUUACAGA	Cleavage
tae-miR1133	Traes_3AS_A6D3DD27E.2						AT4G32920.2	glycine-rich protein	LOC_Os01g09246.1	glycine-rich protein, putative, expressed	2,50	-1.0	1	22	16	37	CAUAUACUCCCUCCGUCCGAAA	UUUGGGACGGAGGGAGUAUGUG	Cleavage
tae-miR1133	Traes_3AS_A6D3DD27E.4						AT4G32920.1	glycine-rich protein	LOC_Os01g09246.1	glycine-rich protein, putative, expressed	2,50	-1.0	1	22	9	30	CAUAUACUCCCUCCGUCCGAAA	UUUGGGACGGAGGGAGUAUGUG	Cleavage
tae-miR1130b-3p	Traes_3AS_AA474B823.1	PF00725,PF16113,PF02737	KOG2304	5.1.2.3,4.2.1.17,1.1.1.35,5.3.3.8		GO:GO:0055114,GO:GO:0016491,GO:GO:0006631,GO:GO:0003857	AT4G29010.1	Enoyl-CoA hydratase/isomerase family	LOC_Os02g17390.1	3-hydroxyacyl-CoA dehydrogenase, putative, expressed	1,50	-1.0	1	21	1652	1672	UCUUAUAUUAUGGGACGGAGG	CCUCCGUCCCAUAAUAUGAGA	Cleavage
tae-miR1122c-3p	Traes_3AS_AA474B823.1	PF00725,PF16113,PF02737	KOG2304	5.1.2.3,4.2.1.17,1.1.1.35,5.3.3.8		GO:GO:0055114,GO:GO:0016491,GO:GO:0006631,GO:GO:0003857	AT4G29010.1	Enoyl-CoA hydratase/isomerase family	LOC_Os02g17390.1	3-hydroxyacyl-CoA dehydrogenase, putative, expressed	1,50	-1.0	1	21	1652	1672	UCUAAUAUUAUGGGACGGAGG	CCUCCGUCCCAUAAUAUGAGA	Cleavage
tae-miR1130b-3p	Traes_3AS_AA474B823.3	PF00725,PF00378,PF02737	KOG2304	5.1.2.3,4.2.1.17,1.1.1.35,5.3.3.8		GO:GO:0055114,GO:GO:0016491,GO:GO:0006631,GO:GO:0003857,GO:GO:0008152,GO:GO:0003824	AT4G29010.1	Enoyl-CoA hydratase/isomerase family	LOC_Os02g17390.1	3-hydroxyacyl-CoA dehydrogenase, putative, expressed	1,50	-1.0	1	21	1397	1417	UCUUAUAUUAUGGGACGGAGG	CCUCCGUCCCAUAAUAUGAGA	Cleavage
tae-miR1122c-3p	Traes_3AS_AA474B823.3	PF00725,PF00378,PF02737	KOG2304	5.1.2.3,4.2.1.17,1.1.1.35,5.3.3.8		GO:GO:0055114,GO:GO:0016491,GO:GO:0006631,GO:GO:0003857,GO:GO:0008152,GO:GO:0003824	AT4G29010.1	Enoyl-CoA hydratase/isomerase family	LOC_Os02g17390.1	3-hydroxyacyl-CoA dehydrogenase, putative, expressed	1,50	-1.0	1	21	1397	1417	UCUAAUAUUAUGGGACGGAGG	CCUCCGUCCCAUAAUAUGAGA	Cleavage
tae-miR159b	Traes_3AS_D1D1A1994.1								LOC_Os01g11430.1	expressed protein	2,50	-1.0	1	21	101	121	UUUGGAUUGAAGGGAGCUCUG	CAGAGCCCCCUUCAAACCAAA	Cleavage
tae-miR159a	Traes_3AS_D1D1A1994.1								LOC_Os01g11430.1	expressed protein	2,50	-1.0	1	21	101	121	UUUGGAUUGAAGGGAGCUCUG	CAGAGCCCCCUUCAAACCAAA	Cleavage
tae-miR1122b-3p	Traes_3AS_D7F2F36BB.2						AT3G49720.2		LOC_Os01g05070.1	expressed protein	1,50	-1.0	1	21	564	584	AGACUUAUAUGUAGGAACGGA	UCCGUUCCUAAAUAUAAGUCU	Translation
tae-miR1122b-3p	Traes_3AS_D7F2F36BB.3						AT3G49720.1		LOC_Os01g05070.1	expressed protein	1,50	-1.0	1	21	540	560	AGACUUAUAUGUAGGAACGGA	UCCGUUCCUAAAUAUAAGUCU	Translation
tae-miR5200	Traes_3B_0E2173F88.1	PF14531	KOG0580	2.7.11.1			AT1G62400.1	Protein kinase superfamily protein	LOC_Os01g01740.1	protein kinase domain containing protein, expressed	2,50	-1.0	1	21	171	191	UGUAGAUACUCCCUAAGGCUU	UUCCCUUAGGAAGUAUCUACA	Translation
tae-miR5200	Traes_3B_0E2173F88.2	PF14531	KOG1164	2.7.11.1			AT1G62400.1	Protein kinase superfamily protein	LOC_Os01g01740.1	protein kinase domain containing protein, expressed	2,50	-1.0	1	21	177	197	UGUAGAUACUCCCUAAGGCUU	UUCCCUUAGGAAGUAUCUACA	Translation
tae-miR5200	Traes_3B_0E2173F88.3	PF14531		2.7.11.1			AT1G62400.1	Protein kinase superfamily protein	LOC_Os01g01740.1	protein kinase domain containing protein, expressed	2,50	-1.0	1	21	171	191	UGUAGAUACUCCCUAAGGCUU	UUCCCUUAGGAAGUAUCUACA	Translation
tae-miR9652-5p	Traes_3B_119F6A405.1										2,50	-1.0	1	22	589	610	CCUGUUUGUCAUUAAGUUUCUU	AAGAAACUGAAUGACAAGCAGG	Cleavage
tae-miR9662a-3p	Traes_3B_224A6B7E5.1	PF02536				GO:GO:0006355,GO:GO:0005739,GO:GO:0003690	AT5G07900.1	Mitochondrial transcription termination factor family protein	LOC_Os06g12100.1	mTERF family protein, expressed	2,00	-1.0	1	21	189	209	UUGAACAUCCCAGAGCCACCG	CCGUGGUUCUGGGAUGUUCAG	Cleavage
tae-miR9662b-3p	Traes_3B_224A6B7E5.1	PF02536				GO:GO:0006355,GO:GO:0005739,GO:GO:0003690	AT5G07900.1	Mitochondrial transcription termination factor family protein	LOC_Os06g12100.1	mTERF family protein, expressed	2,00	-1.0	1	21	188	208	UGAACAUCCCAGAGCCACCGG	CCCGUGGUUCUGGGAUGUUCA	Cleavage
tae-miR159b	Traes_3B_25320ED72.1	PF00249	KOG0048		K09422		AT5G06100.1	myb domain protein 33	LOC_Os01g59660.2	MYB family transcription factor, putative, expressed	2,50	-1.0	1	21	956	976	UUUGGAUUGAAGGGAGCUCUG	UGGAGCUCCCUUCACUCCAAG	Cleavage
tae-miR159a	Traes_3B_25320ED72.1	PF00249	KOG0048		K09422		AT5G06100.1	myb domain protein 33	LOC_Os01g59660.2	MYB family transcription factor, putative, expressed	2,50	-1.0	1	21	956	976	UUUGGAUUGAAGGGAGCUCUG	UGGAGCUCCCUUCACUCCAAG	Cleavage
tae-miR159b	Traes_3B_25320ED72.2	PF00249	KOG0048		K09422		AT5G06100.1	myb domain protein 33	LOC_Os01g59660.1	MYB family transcription factor, putative, expressed	2,50	-1.0	1	21	956	976	UUUGGAUUGAAGGGAGCUCUG	UGGAGCUCCCUUCACUCCAAG	Cleavage
tae-miR159a	Traes_3B_25320ED72.2	PF00249	KOG0048		K09422		AT5G06100.1	myb domain protein 33	LOC_Os01g59660.1	MYB family transcription factor, putative, expressed	2,50	-1.0	1	21	956	976	UUUGGAUUGAAGGGAGCUCUG	UGGAGCUCCCUUCACUCCAAG	Cleavage
tae-miR1130b-3p	Traes_3B_467787390.8	PF00917,PF13423,PF14533,PF12436		3.4.19.12		GO:GO:0005515	AT3G11910.1	ubiquitin-specific protease 13	LOC_Os01g56490.1	ubiquitin carboxyl-terminal hydrolase, putative, expressed	1,50	-1.0	1	21	3080	3100	UCUUAUAUUAUGGGACGGAGG	CCUCCGUCCCAAAAUAUAAGA	Translation
tae-miR9776	Traes_3B_470061369.1	PF00270		3.6.4.13		GO:GO:0005524,GO:GO:0003676	AT5G51280.1	DEAD-box protein abstrakt, putative	LOC_Os02g05660.1	DEAD-box ATP-dependent RNA helicase 35A, putative, expressed	2,50	-1.0	1	21	34	54	UUGGACGAGGAUGUGCAACUG	AAGUGGCACAUCCUCGUCGAA	Cleavage
tae-miR1122a	Traes_3B_472072804.1	PF04874,PF01778	KOG3064		K14831		AT1G23280.1	MAK16 protein-related	LOC_Os07g16950.1	Mak16 protein domain containing protein, expressed	1,50	-1.0	1	20	789	808	UAGAUACAUCCGUAUCUAGA	UCUAGAUACGGAGGUAUCUA	Cleavage
tae-miR9778	Traes_3B_4C839468C.10						AT3G46530.1	NB-ARC domain-containing disease resistance protein	LOC_Os12g37290.1	resistance protein T10rga2-1A, putative, expressed	1,00	-1.0	1	21	126	146	UGCAUCAUCUCGAACUCGUCG	GGACGAGCUCGAGAUGAUGCA	Cleavage
tae-miR9778	Traes_3B_4C839468C.11						AT3G46530.1	NB-ARC domain-containing disease resistance protein	LOC_Os12g37290.1	resistance protein T10rga2-1A, putative, expressed	1,00	-1.0	1	21	126	146	UGCAUCAUCUCGAACUCGUCG	GGACGAGCUCGAGAUGAUGCA	Cleavage
tae-miR9778	Traes_3B_4C839468C.12						AT3G46530.1	NB-ARC domain-containing disease resistance protein	LOC_Os12g37290.1	resistance protein T10rga2-1A, putative, expressed	1,00	-1.0	1	21	126	146	UGCAUCAUCUCGAACUCGUCG	GGACGAGCUCGAGAUGAUGCA	Cleavage
tae-miR9778	Traes_3B_4C839468C.13						AT1G50180.1	NB-ARC domain-containing disease resistance protein	LOC_Os02g57310.1	pib, putative, expressed	1,00	-1.0	1	21	102	122	UGCAUCAUCUCGAACUCGUCG	GGACGAGCUCGAGAUGAUGCA	Cleavage
tae-miR9778	Traes_3B_4C839468C.2						AT3G46530.1	NB-ARC domain-containing disease resistance protein	LOC_Os12g37290.1	resistance protein T10rga2-1A, putative, expressed	1,00	-1.0	1	21	126	146	UGCAUCAUCUCGAACUCGUCG	GGACGAGCUCGAGAUGAUGCA	Cleavage
tae-miR9778	Traes_3B_4C839468C.3						AT3G46530.1	NB-ARC domain-containing disease resistance protein	LOC_Os12g37290.1	resistance protein T10rga2-1A, putative, expressed	1,00	-1.0	1	21	126	146	UGCAUCAUCUCGAACUCGUCG	GGACGAGCUCGAGAUGAUGCA	Cleavage
tae-miR9778	Traes_3B_4C839468C.4						AT3G46530.1	NB-ARC domain-containing disease resistance protein	LOC_Os12g37290.1	resistance protein T10rga2-1A, putative, expressed	1,00	-1.0	1	21	126	146	UGCAUCAUCUCGAACUCGUCG	GGACGAGCUCGAGAUGAUGCA	Cleavage
tae-miR9778	Traes_3B_4C839468C.5						AT3G46530.1	NB-ARC domain-containing disease resistance protein	LOC_Os12g37290.1	resistance protein T10rga2-1A, putative, expressed	1,00	-1.0	1	21	126	146	UGCAUCAUCUCGAACUCGUCG	GGACGAGCUCGAGAUGAUGCA	Cleavage
tae-miR9778	Traes_3B_4C839468C.6						AT3G46530.1	NB-ARC domain-containing disease resistance protein	LOC_Os12g37290.1	resistance protein T10rga2-1A, putative, expressed	1,00	-1.0	1	21	126	146	UGCAUCAUCUCGAACUCGUCG	GGACGAGCUCGAGAUGAUGCA	Cleavage
tae-miR9778	Traes_3B_4C839468C.7						AT3G46530.1	NB-ARC domain-containing disease resistance protein	LOC_Os12g37290.1	resistance protein T10rga2-1A, putative, expressed	1,00	-1.0	1	21	126	146	UGCAUCAUCUCGAACUCGUCG	GGACGAGCUCGAGAUGAUGCA	Cleavage
tae-miR9778	Traes_3B_4C839468C.8						AT3G46530.1	NB-ARC domain-containing disease resistance protein	LOC_Os12g37290.1	resistance protein T10rga2-1A, putative, expressed	1,00	-1.0	1	21	126	146	UGCAUCAUCUCGAACUCGUCG	GGACGAGCUCGAGAUGAUGCA	Cleavage
tae-miR9778	Traes_3B_4C839468C.9						AT3G46530.1	NB-ARC domain-containing disease resistance protein	LOC_Os12g37290.1	resistance protein T10rga2-1A, putative, expressed	1,00	-1.0	1	21	126	146	UGCAUCAUCUCGAACUCGUCG	GGACGAGCUCGAGAUGAUGCA	Cleavage
tae-miR1137a	Traes_3B_59B827C5C.2	PF01912	KOG3185			GO:GO:0043022,GO:GO:0042256	AT3G55620.1	Translation initiation factor IF6	LOC_Os07g44620.2	eukaryotic translation initiation factor 6, putative, expressed	0,00	-1.0	1	20	748	767	UAGUACAAAGUUGAGUCAUC	GAUGACUCAACUUUGUACUA	Cleavage
tae-miR1122b-3p	Traes_3B_5DD9C1BE5.1								LOC_Os01g40830.1	transposon protein, putative, Mutator sub-class, expressed	2,50	-1.0	1	21	71	91	AGACUUAUAUGUAGGAACGGA	UCCGUUCUUAAAUAUAAGUCU	Translation
tae-miR7757-5p	Traes_3B_5EFB9A61F.1								LOC_Os12g17490.1	stripe rust resistance protein Yr10, putative, expressed	1,50	-1.0	1	22	113	134	AUAAAACCUUCAGCUAUCCAUC	GAUGGAUAGCUGAAGGUUUUGU	Cleavage
tae-miR7757-5p	Traes_3B_5EFB9A61F.3								LOC_Os12g17490.1	stripe rust resistance protein Yr10, putative, expressed	1,50	-1.0	1	22	89	110	AUAAAACCUUCAGCUAUCCAUC	GAUGGAUAGCUGAAGGUUUUGU	Cleavage
tae-miR9676-5p	Traes_3B_5F0E1F6A4.1						AT1G52700.1	alpha/beta-Hydrolases superfamily protein	LOC_Os05g51050.1	acyl-protein thioesterase, putative, expressed	2,50	-1.0	1	22	38	59	UGGAUGUCAUCGUGGCCGUACA	UGUACGGACAUGAUGACAUCCA	Cleavage
tae-miR1122b-3p	Traes_3B_6D2DCE280.3	PF00025	KOG0084			GO:GO:0005525	AT4G17530.1	RAB GTPase homolog 1C	LOC_Os01g37800.1	ras-related protein, putative, expressed	2,50	-1.0	1	21	576	596	AGACUUAUAUGUAGGAACGGA	UCUGUUCCUAAAUAUAAGUCU	Translation
tae-miR7757-5p	Traes_3B_7FFFF7BCF.1								LOC_Os02g18510.1	stripe rust resistance protein Yr10, putative, expressed	0,00	-1.0	1	22	89	110	AUAAAACCUUCAGCUAUCCAUC	GAUGGAUAGCUGAAGGUUUUAU	Cleavage
tae-miR531	Traes_3B_879F704A0.1										2,50	-1.0	1	21	155	175	CGCUCGCCGGAGCAGCGUGCA	UCCGCGCUGCUCCGGCGAGGG	Cleavage
tae-miR531	Traes_3B_879F704A0.2										2,50	-1.0	1	21	155	175	CGCUCGCCGGAGCAGCGUGCA	UCCGCGCUGCUCCGGCGAGGG	Cleavage
tae-miR531	Traes_3B_879F704A0.3										2,50	-1.0	1	21	50	70	CGCUCGCCGGAGCAGCGUGCA	UCCGCGCUGCUCCGGCGAGGG	Cleavage
tae-miR531	Traes_3B_879F704A0.4										2,50	-1.0	1	21	50	70	CGCUCGCCGGAGCAGCGUGCA	UCCGCGCUGCUCCGGCGAGGG	Cleavage
tae-miR5049-3p	Traes_3B_9D776A8C4.1	PF00804				GO:GO:0016020	AT3G24350.2	syntaxin of plants 32	LOC_Os01g08400.1	syntaxin, putative, expressed	0,00	-1.0	1	21	575	595	AAUAUGGAUCGGAGGGAGUAC	GUACUCCCUCCGAUCCAUAUU	Cleavage
tae-miR1121	Traes_3B_9F321609E.8										1,50	-1.0	1	22	6	28	AGUAGUGAUCUAAACGCUCU-UA	UAUAGAGCGUUUAGAUCACUAUU	Cleavage
tae-miR9778	Traes_3B_F07C33521.1						AT3G46710.1	NB-ARC domain-containing disease resistance protein	LOC_Os02g57310.1	pib, putative, expressed	1,00	-1.0	1	21	126	146	UGCAUCAUCUCGAACUCGUCG	GGACGAGCUCGAGAUGAUGCA	Cleavage
tae-miR9778	Traes_3B_F07C33521.10						AT3G46710.1	NB-ARC domain-containing disease resistance protein	LOC_Os02g57310.1	pib, putative, expressed	1,00	-1.0	1	21	126	146	UGCAUCAUCUCGAACUCGUCG	GGACGAGCUCGAGAUGAUGCA	Cleavage
tae-miR9778	Traes_3B_F07C33521.2	PF00931				GO:GO:0043531	AT3G46530.1	NB-ARC domain-containing disease resistance protein	LOC_Os08g42670.2	resistance protein, putative, expressed	1,00	-1.0	1	21	126	146	UGCAUCAUCUCGAACUCGUCG	GGACGAGCUCGAGAUGAUGCA	Cleavage
tae-miR9778	Traes_3B_F07C33521.3	PF00931				GO:GO:0043531	AT3G46530.1	NB-ARC domain-containing disease resistance protein	LOC_Os08g42670.2	resistance protein, putative, expressed	1,00	-1.0	1	21	126	146	UGCAUCAUCUCGAACUCGUCG	GGACGAGCUCGAGAUGAUGCA	Cleavage
tae-miR9778	Traes_3B_F07C33521.4	PF00931				GO:GO:0043531	AT3G46530.1	NB-ARC domain-containing disease resistance protein	LOC_Os08g42670.2	resistance protein, putative, expressed	1,00	-1.0	1	21	126	146	UGCAUCAUCUCGAACUCGUCG	GGACGAGCUCGAGAUGAUGCA	Cleavage
tae-miR9778	Traes_3B_F07C33521.5	PF00931				GO:GO:0043531	AT3G46530.1	NB-ARC domain-containing disease resistance protein	LOC_Os08g42670.1	resistance protein, putative, expressed	1,00	-1.0	1	21	126	146	UGCAUCAUCUCGAACUCGUCG	GGACGAGCUCGAGAUGAUGCA	Cleavage
tae-miR9778	Traes_3B_F07C33521.6	PF00931				GO:GO:0043531	AT3G46530.1	NB-ARC domain-containing disease resistance protein	LOC_Os08g42670.1	resistance protein, putative, expressed	1,00	-1.0	1	21	126	146	UGCAUCAUCUCGAACUCGUCG	GGACGAGCUCGAGAUGAUGCA	Cleavage
tae-miR9778	Traes_3B_F07C33521.7	PF00931				GO:GO:0043531	AT3G46530.1	NB-ARC domain-containing disease resistance protein	LOC_Os08g42670.2	resistance protein, putative, expressed	1,00	-1.0	1	21	126	146	UGCAUCAUCUCGAACUCGUCG	GGACGAGCUCGAGAUGAUGCA	Cleavage
tae-miR9778	Traes_3B_F07C33521.8						AT3G46710.1	NB-ARC domain-containing disease resistance protein	LOC_Os02g57310.1	pib, putative, expressed	1,00	-1.0	1	21	126	146	UGCAUCAUCUCGAACUCGUCG	GGACGAGCUCGAGAUGAUGCA	Cleavage
tae-miR9778	Traes_3B_F07C33521.9						AT3G46710.1	NB-ARC domain-containing disease resistance protein	LOC_Os02g57310.1	pib, putative, expressed	1,00	-1.0	1	21	126	146	UGCAUCAUCUCGAACUCGUCG	GGACGAGCUCGAGAUGAUGCA	Cleavage
tae-miR1128	Traes_3B_F61EDC1AE.3	PF02212,PF00350,PF01031				GO:GO:0005525,GO:GO:0003924	AT4G33650.1	dynamin-related protein 3A	LOC_Os01g69130.1	dynamin family protein, putative, expressed	2,00	-1.0	1	21	1581	1601	UACUACUCCCUCCGUCCGAAA	UUCCGAACGGAGGGAGUAGUA	Cleavage
tae-miR1127a	Traes_3B_F61EDC1AE.3	PF02212,PF00350,PF01031				GO:GO:0005525,GO:GO:0003924	AT4G33650.1	dynamin-related protein 3A	LOC_Os01g69130.1	dynamin family protein, putative, expressed	1,50	-1.0	1	19	1577	1595	UCCUUCCGUUCGGAAUUAC	GUAAUUCCGAACGGAGGGA	Cleavage
tae-miR1130b-3p	Traes_3B_FB51F185F.2						AT1G53560.1	Ribosomal protein L18ae family	LOC_Os01g14070.1	60S ribosomal protein L18a-1, putative, expressed	1,50	-1.0	1	21	43	63	UCUUAUAUUAUGGGACGGAGG	CCUCCGUCCCAUAAUGUAAGA	Cleavage
tae-miR164	Traes_3B_FD8ACC50C.1			6.3.2.19		GO:GO:0007275,GO:GO:0006511,GO:GO:0005634	AT1G66650.1	Protein with RING/U-box and TRAF-like domains	LOC_Os01g03190.1	expressed protein	2,50	-1.0	1	21	1544	1564	UGGAGAAGCAGGGCACGUGCA	CGCACGGGCCCUGCUUCUGCA	Cleavage
tae-miR164	Traes_3B_FD8ACC50C.2			6.3.2.19		GO:GO:0007275,GO:GO:0006511,GO:GO:0005634	AT1G66650.1	Protein with RING/U-box and TRAF-like domains	LOC_Os01g03190.1	expressed protein	2,50	-1.0	1	21	1499	1519	UGGAGAAGCAGGGCACGUGCA	CGCACGGGCCCUGCUUCUGCA	Cleavage
tae-miR164	Traes_3B_FD8ACC50C.3			6.3.2.19		GO:GO:0007275,GO:GO:0006511,GO:GO:0005634	AT1G66650.1	Protein with RING/U-box and TRAF-like domains	LOC_Os01g03190.1	expressed protein	2,50	-1.0	1	21	1433	1453	UGGAGAAGCAGGGCACGUGCA	CGCACGGGCCCUGCUUCUGCA	Cleavage
tae-miR164	Traes_3B_FD8ACC50C.4			6.3.2.19		GO:GO:0007275,GO:GO:0006511,GO:GO:0005634	AT1G66650.1	Protein with RING/U-box and TRAF-like domains	LOC_Os01g03190.1	expressed protein	2,50	-1.0	1	21	764	784	UGGAGAAGCAGGGCACGUGCA	CGCACGGGCCCUGCUUCUGCA	Cleavage
tae-miR164	Traes_3B_FD8ACC50C.5			6.3.2.19		GO:GO:0007275,GO:GO:0006511,GO:GO:0005634	AT1G66650.1	Protein with RING/U-box and TRAF-like domains	LOC_Os01g03190.1	expressed protein	2,50	-1.0	1	21	752	772	UGGAGAAGCAGGGCACGUGCA	CGCACGGGCCCUGCUUCUGCA	Cleavage
tae-miR164	Traes_3B_FD8ACC50C.6			6.3.2.19		GO:GO:0007275,GO:GO:0006511,GO:GO:0005634	AT1G66650.1	Protein with RING/U-box and TRAF-like domains	LOC_Os01g03190.1	expressed protein	2,50	-1.0	1	21	731	751	UGGAGAAGCAGGGCACGUGCA	CGCACGGGCCCUGCUUCUGCA	Cleavage
tae-miR164	Traes_3B_FD8ACC50C.7			6.3.2.19		GO:GO:0007275,GO:GO:0006511,GO:GO:0005634	AT1G66650.1	Protein with RING/U-box and TRAF-like domains	LOC_Os01g03190.1	expressed protein	2,50	-1.0	1	21	710	730	UGGAGAAGCAGGGCACGUGCA	CGCACGGGCCCUGCUUCUGCA	Cleavage
tae-miR164	Traes_3B_FD8ACC50C.8			6.3.2.19		GO:GO:0007275,GO:GO:0006511,GO:GO:0005634	AT1G66650.1	Protein with RING/U-box and TRAF-like domains	LOC_Os01g03190.1	expressed protein	2,50	-1.0	1	21	653	673	UGGAGAAGCAGGGCACGUGCA	CGCACGGGCCCUGCUUCUGCA	Cleavage
tae-miR164	Traes_3B_FD8ACC50C.9			6.3.2.19		GO:GO:0007275,GO:GO:0006511,GO:GO:0005634	AT1G66650.1	Protein with RING/U-box and TRAF-like domains	LOC_Os01g03190.1	expressed protein	2,50	-1.0	1	21	356	376	UGGAGAAGCAGGGCACGUGCA	CGCACGGGCCCUGCUUCUGCA	Cleavage
tae-miR5049-3p	Traes_3DL_2B3694DC3.2	PF01217					AT3G09800.1	SNARE-like superfamily protein	LOC_Os05g38610.1	clathrin adaptor complex small chain domain containing protein, expressed	1,50	-1.0	1	21	228	248	AAUAUGGAUCGGAGGGAGUAC	CUACUCCCUCCGAUCCAAAUU	Cleavage
tae-miR1130b-3p	Traes_3DL_426EF6A68.2										2,50	-1.0	1	21	214	234	UCUUAUAUUAUGGGACGGAGG	CCUCCAUCCCGUAAUAUAAGA	Translation
tae-miR7757-5p	Traes_3DL_5C0BC5611.4								LOC_Os12g17490.1	stripe rust resistance protein Yr10, putative, expressed	1,50	-1.0	1	22	89	110	AUAAAACCUUCAGCUAUCCAUC	GAUGGAUAGCUGAAGGUUUUGU	Cleavage
tae-miR1137a	Traes_3DL_96847DCCF.1	PF00069	KOG1187	2.7.11.1		GO:GO:0006468,GO:GO:0005524,GO:GO:0004672	AT1G34300.1	lectin protein kinase family protein	LOC_Os01g47840.1	S-locus-like receptor protein kinase, putative, expressed	1,00	-1.0	1	20	88	107	UAGUACAAAGUUGAGUCAUC	GAUGACCCAACUUUGUACUA	Cleavage
tae-miR5049-3p	Traes_3DL_D1CF4784D.2	PF13307,PF06733		3.6.4.12		GO:GO:0016818,GO:GO:0008026,GO:GO:0006139,GO:GO:0005524,GO:GO:0003676,GO:GO:0004003,GO:GO:0003677	AT1G79950.1	RAD3-like DNA-binding helicase protein	LOC_Os01g40980.1	helicase, putative, expressed	2,50	-1.0	1	21	1646	1666	AAUAUGGAUCGGAGGGAGUAC	AUAAUCCCUCCGUUCCAUAUU	Cleavage
tae-miR159b	Traes_3DL_D9A29948B.1	PF00249					AT5G06100.1	myb domain protein 33	LOC_Os01g59660.1	MYB family transcription factor, putative, expressed	2,50	-1.0	1	21	620	640	UUUGGAUUGAAGGGAGCUCUG	UGGAGCUCCCUUCACUCCAAG	Cleavage
tae-miR159a	Traes_3DL_D9A29948B.1	PF00249					AT5G06100.1	myb domain protein 33	LOC_Os01g59660.1	MYB family transcription factor, putative, expressed	2,50	-1.0	1	21	620	640	UUUGGAUUGAAGGGAGCUCUG	UGGAGCUCCCUUCACUCCAAG	Cleavage
tae-miR159b	Traes_3DL_D9A29948B.2	PF00249					AT5G06100.1	myb domain protein 33	LOC_Os01g59660.1	MYB family transcription factor, putative, expressed	2,50	-1.0	1	21	620	640	UUUGGAUUGAAGGGAGCUCUG	UGGAGCUCCCUUCACUCCAAG	Cleavage
tae-miR159a	Traes_3DL_D9A29948B.2	PF00249					AT5G06100.1	myb domain protein 33	LOC_Os01g59660.1	MYB family transcription factor, putative, expressed	2,50	-1.0	1	21	620	640	UUUGGAUUGAAGGGAGCUCUG	UGGAGCUCCCUUCACUCCAAG	Cleavage
tae-miR1128	Traes_3DS_1DE20CFF3.2	PF01121	KOG3220	2.7.1.24	K00859	GO:GO:0015937,GO:GO:0005524,GO:GO:0004140	AT2G27490.1	dephospho-CoA kinase family	LOC_Os01g25880.1	dephospho-CoA kinase, putative, expressed	2,50	-1.0	1	21	71	91	UACUACUCCCUCCGUCCGAAA	UUUGGGACGGAGGGAGUACUA	Cleavage
tae-miR1122a	Traes_3DS_1DE20CFF3.2	PF01121	KOG3220	2.7.1.24	K00859	GO:GO:0015937,GO:GO:0005524,GO:GO:0004140	AT2G27490.1	dephospho-CoA kinase family	LOC_Os01g25880.1	dephospho-CoA kinase, putative, expressed	1,50	-1.0	1	20	2	21	UAGAUACAUCCGUAUCUAGA	UCUAGAUACAGAUGUAUCUA	Translation
tae-miR1128	Traes_3DS_1DE20CFF3.3	PF01121	KOG3220	2.7.1.24	K00859	GO:GO:0015937,GO:GO:0005524,GO:GO:0004140	AT2G27490.1	dephospho-CoA kinase family	LOC_Os01g25880.1	dephospho-CoA kinase, putative, expressed	2,50	-1.0	1	21	71	91	UACUACUCCCUCCGUCCGAAA	UUUGGGACGGAGGGAGUACUA	Cleavage
tae-miR1122a	Traes_3DS_1DE20CFF3.3	PF01121	KOG3220	2.7.1.24	K00859	GO:GO:0015937,GO:GO:0005524,GO:GO:0004140	AT2G27490.1	dephospho-CoA kinase family	LOC_Os01g25880.1	dephospho-CoA kinase, putative, expressed	1,50	-1.0	1	20	2	21	UAGAUACAUCCGUAUCUAGA	UCUAGAUACAGAUGUAUCUA	Translation
tae-miR1122b-3p	Traes_3DS_D42FCD586.1	PF00274		4.1.2.13		GO:GO:0006096,GO:GO:0004332	AT2G01140.1	Aldolase superfamily protein	LOC_Os01g02880.1	fructose-bisphospate aldolase isozyme, putative, expressed	1,50	-1.0	1	21	786	806	AGACUUAUAUGUAGGAACGGA	UCCGUUCCUAAAUAUAAGUCU	Translation
tae-miR1122b-3p	Traes_3DS_D42FCD586.2	PF00274		4.1.2.13		GO:GO:0006096,GO:GO:0004332	AT2G01140.1	Aldolase superfamily protein	LOC_Os01g02880.1	fructose-bisphospate aldolase isozyme, putative, expressed	1,50	-1.0	1	21	795	815	AGACUUAUAUGUAGGAACGGA	UCCGUUCCUAAAUAUAAGUCU	Translation
tae-miR7757-5p	Traes_4AL_13A3A40D3.1	PF13401	KOG4658				AT3G50950.2	HOPZ-ACTIVATED RESISTANCE 1	LOC_Os11g11810.1	NBS-LRR disease resistance protein, putative, expressed	1,50	-1.0	1	22	1337	1358	AUAAAACCUUCAGCUAUCCAUC	AGUGGAUAGCUGAAGGUUUCAU	Cleavage
tae-miR1135	Traes_4AL_158B970F4.1						AT1G50020.1		LOC_Os11g34870.1	expressed protein	2,50	-1.0	1	24	464	487	CUGCGACAAGUAAUUCCGAACGGA	UCCGUUCCGAAUUACUCGUCGCAG	Cleavage
tae-miR5175-5p	Traes_4AL_158B970F4.1						AT1G50020.1		LOC_Os11g34870.1	expressed protein	3,00	-1.0	1	21	532	552	UUCCAAAUUACUCGUCGUGGU	UCUGAGACGAGUAAUUUGGAA	Cleavage
tae-miR1117	Traes_4AL_2475C298D.1	PF07993		1.2.1.84		GO:GO:0080019	AT3G44540.1	fatty acid reductase 4	LOC_Os04g28600.1	male sterility protein, putative, expressed	2,50	-1.0	1	24	684	707	UAGUACCGGUUCGUGGCACGAACC	GGUUCAUGCCAUGAACCGGUACUA	Cleavage
tae-miR9778	Traes_4AL_2FC39B934.1	PF13191,PF00931				GO:GO:0043531	AT1G53350.1	Disease resistance protein (CC-NBS-LRR class) family	LOC_Os08g14850.1	resistance protein, putative, expressed	2,50	-1.0	1	21	126	146	UGCAUCAUCUCGAACUCGUCG	UGGCGAGUUCCAGAUGAUGCA	Translation
tae-miR408	Traes_4AL_364F56EDB.1	PF02298				GO:GO:0009055	AT2G02850.1	plantacyanin	LOC_Os03g50160.1	plastocyanin-like domain containing protein, putative, expressed	0,00	-1.0	1	21	8	28	CUGCACUGCCUCUUCCCUGGC	CCCAGGGAAGAGGCAGUGCAG	Cleavage
tae-miR9662a-3p	Traes_4AL_3A009739E.1	PF02536				GO:GO:0006355,GO:GO:0005739,GO:GO:0003690	AT1G21150.1	Mitochondrial transcription termination factor family protein	LOC_Os06g12100.1	mTERF family protein, expressed	2,50	-1.0	1	21	66	86	UUGAACAUCCCAGAGCCACCG	CCGUGGCUCUGGAAUGUUCAG	Cleavage
tae-miR9662b-3p	Traes_4AL_3A009739E.1	PF02536				GO:GO:0006355,GO:GO:0005739,GO:GO:0003690	AT1G21150.1	Mitochondrial transcription termination factor family protein	LOC_Os06g12100.1	mTERF family protein, expressed	2,50	-1.0	1	21	65	85	UGAACAUCCCAGAGCCACCGG	CCCGUGGCUCUGGAAUGUUCA	Cleavage
tae-miR1121	Traes_4AL_400EB3C17.10	PF14259,PF00076	KOG0120			GO:GO:0003676	AT4G36690.4	U2 snRNP auxilliary factor, large subunit, splicing factor	LOC_Os11g41820.2	RNA recognition motif containing protein, expressed	2,50	-1.0	1	22	10	31	AGUAGUGAUCUAAACGCUCUUA	UAAGAGCGUUUAGAUCACUAAA	Cleavage
tae-miR1121	Traes_4AL_400EB3C17.3	PF14259,PF00076	KOG0120			GO:GO:0003676	AT4G36690.1	U2 snRNP auxilliary factor, large subunit, splicing factor	LOC_Os11g41820.1	RNA recognition motif containing protein, expressed	2,50	-1.0	1	22	37	58	AGUAGUGAUCUAAACGCUCUUA	UAAGAGCGUUUAGAUCACUAAA	Cleavage
tae-miR1121	Traes_4AL_400EB3C17.7	PF14259,PF00076	KOG0120			GO:GO:0003676	AT4G36690.4	U2 snRNP auxilliary factor, large subunit, splicing factor	LOC_Os11g41820.1	RNA recognition motif containing protein, expressed	2,50	-1.0	1	22	10	31	AGUAGUGAUCUAAACGCUCUUA	UAAGAGCGUUUAGAUCACUAAA	Cleavage
tae-miR1137a	Traes_4AL_40790E994.6	PF05185		2.1.1.125,2.1.1.126		GO:GO:0008168,GO:GO:0006479	AT4G16570.1	protein arginine methyltransferase 7	LOC_Os06g01640.1	arginine N-methyltransferase 7, putative, expressed	1,00	-1.0	1	20	1324	1343	UAGUACAAAGUUGAGUCAUC	GAUGACCCAACUUUGUACUA	Cleavage
tae-miR1137a	Traes_4AL_40790E994.7			2.1.1.125,2.1.1.126		GO:GO:0008168,GO:GO:0006479	AT4G16570.1	protein arginine methyltransferase 7	LOC_Os06g01640.1	arginine N-methyltransferase 7, putative, expressed	1,00	-1.0	1	20	835	854	UAGUACAAAGUUGAGUCAUC	GAUGACCCAACUUUGUACUA	Cleavage
tae-miR1127b-3p	Traes_4AL_44E99BA01.2	PF07714		2.7.11.1		GO:GO:0006468,GO:GO:0004672	AT4G33950.1	Protein kinase superfamily protein	LOC_Os03g41460.1	CAMK_CAMK_like.20 - CAMK includes calcium/calmodulin depedent protein kinases, expressed	1,50	-1.0	1	21	394	414	ACAAGUAUUUCUGGACGGAGG	CCUCCGUCCGGAAAUACUUGU	Cleavage

tae-miR1121	Traes_4AL_541C86EB9.2	PF00076				GO:GO:0003676,GO:GO:0017069,GO:GO:0000398	AT1G21320.1	nucleotide binding;nucleic acid binding	LOC_Os06g03690.2	RNA recognition motif containing protein, putative, expressed	1,00	-1.0	1	22	558	579	AGUAGUGAUCUAAACGCUCUUA	UGAGAUCGUUUAGAUCACUACU	Cleavage
tae-miR1121	Traes_4AL_541C86EB9.3	PF00076				GO:GO:0003676,GO:GO:0017069,GO:GO:0000398	AT1G21320.1	nucleotide binding;nucleic acid binding	LOC_Os06g03690.2	RNA recognition motif containing protein, putative, expressed	1,00	-1.0	1	22	558	579	AGUAGUGAUCUAAACGCUCUUA	UGAGAUCGUUUAGAUCACUACU	Cleavage
tae-miR1122b-3p	Traes_4AL_6252B0BA0.1	PF05562					AT1G29395.1	COLD REGULATED 314 INNER MEMBRANE 1	LOC_Os05g49170.1	cold acclimation protein WCOR413, putative, expressed	1,50	-1.0	1	21	46	66	AGACUUAUAUGUAGGAACGGA	UCCGUUCCUAAAUAUAAGUCU	Translation
tae-miR1137b-5p	Traes_4AL_748BB4675.1	PF00657		3.1.1.1		GO:GO:0016788	AT5G45910.1	GDSL-like Lipase/Acylhydrolase superfamily protein	LOC_Os01g12320.1	GDSL-like lipase/acylhydrolase, putative, expressed	2,50	-1.0	1	21	844	864	UCCGUUCCAGAAUAGAUGACC	ACUCAUCUAUUCUGGGACGGU	Cleavage
tae-miR9778	Traes_4AL_79B47AF61.1	PF13191,PF00931				GO:GO:0043531	AT3G07040.1	NB-ARC domain-containing disease resistance protein	LOC_Os08g42670.1	resistance protein, putative, expressed	2,50	-1.0	1	21	126	146	UGCAUCAUCUCGAACUCGUCG	GGACGAGCUCGACAUGAUGCA	Cleavage
tae-miR9778	Traes_4AL_79B47AF61.3	PF00931				GO:GO:0043531	AT3G46530.1	NB-ARC domain-containing disease resistance protein	LOC_Os08g42670.2	resistance protein, putative, expressed	2,50	-1.0	1	21	126	146	UGCAUCAUCUCGAACUCGUCG	GGACGAGCUCGACAUGAUGCA	Cleavage
tae-miR9778	Traes_4AL_79B47AF61.4	PF00931				GO:GO:0043531	AT3G46530.1	NB-ARC domain-containing disease resistance protein	LOC_Os08g42670.2	resistance protein, putative, expressed	2,50	-1.0	1	21	126	146	UGCAUCAUCUCGAACUCGUCG	GGACGAGCUCGACAUGAUGCA	Cleavage
tae-miR5048-5p	Traes_4AL_85A7C60F9.1	PF00069		2.7.11.1		GO:GO:0006468,GO:GO:0005524,GO:GO:0004672	AT4G21230.1	cysteine-rich RLK (RECEPTOR-like protein kinase) 27	LOC_Os11g10640.1	protein kinase domain containing protein, expressed	2,50	-1.0	1	22	170	191	UUUGCAGGUUUUAGGUCUAAGU	ACUUGGACCUAAAACCUGCGAA	Cleavage
tae-miR5048-5p	Traes_4AL_85A7C60F9.7	PF00069		2.7.11.1		GO:GO:0006468,GO:GO:0005524,GO:GO:0004672	AT4G21230.1	cysteine-rich RLK (RECEPTOR-like protein kinase) 27	LOC_Os11g10640.1	protein kinase domain containing protein, expressed	2,50	-1.0	1	22	170	191	UUUGCAGGUUUUAGGUCUAAGU	ACUUGGACCUAAAACCUGCGAA	Cleavage
tae-miR5048-5p	Traes_4AL_85A7C60F9.8	PF00069		2.7.11.1		GO:GO:0006468,GO:GO:0005524,GO:GO:0004672	AT4G21230.1	cysteine-rich RLK (RECEPTOR-like protein kinase) 27	LOC_Os11g10640.1	protein kinase domain containing protein, expressed	2,50	-1.0	1	22	170	191	UUUGCAGGUUUUAGGUCUAAGU	ACUUGGACCUAAAACCUGCGAA	Cleavage
tae-miR1122b-3p	Traes_4AL_954AE4933.1	PF12171					AT2G02080.1	indeterminate(ID)-domain 4	LOC_Os04g47860.4	ZOS4-11 - C2H2 zinc finger protein, expressed	1,50	-1.0	1	21	583	603	AGACUUAUAUGUAGGAACGGA	UCCGUUCCUAAAUAUAAGUCU	Translation
tae-miR1122a	Traes_4AL_9726307FF.1	PF01926,PF06421,PF00679		3.6.5.3		GO:GO:0005525	AT5G39900.1	Small GTP-binding protein	LOC_Os06g05250.2	GTP-binding protein GUF1, putative, expressed	0,00	-1.0	1	20	1903	1922	UAGAUACAUCCGUAUCUAGA	UCUAGAUACGGAUGUAUCUA	Cleavage
tae-miR1128	Traes_4AL_99942CBEA.1	PF02365				GO:GO:0006355,GO:GO:0003677	AT2G46770.1	NAC (No Apical Meristem) domain transcriptional regulator superfamily protein	LOC_Os06g04090.1	no apical meristem protein, putative, expressed	3,00	-1.0	1	21	693	714	UACUACUCCCUCCG-UCCGAAA	UUUAGGAACGGAGGGAGUAGUA	Cleavage
tae-miR1122b-3p	Traes_4AL_99942CBEA.1	PF02365				GO:GO:0006355,GO:GO:0003677	AT2G46770.1	NAC (No Apical Meristem) domain transcriptional regulator superfamily protein	LOC_Os06g04090.1	no apical meristem protein, putative, expressed	2,50	-1.0	1	21	557	577	AGACUUAUAUGUAGGAACGGA	UCCGUUCUAAAUAUAAGUCU	Translation
tae-miR9662a-3p	Traes_4AL_A4B73CC6A1.1	PF02536	KOG1267		K15032	GO:GO:0006355,GO:GO:0005739,GO:GO:0003690	AT5G07900.1	Mitochondrial transcription termination factor family protein	LOC_Os06g12100.1	mTERF family protein, expressed	2,50	-1.0	1	21	693	713	UUGAACAUCCCAGAGCCACCG	CCGUGGCUCUGGAAUGUUCAG	Cleavage
tae-miR9662b-3p	Traes_4AL_A4B73CC6A1.1	PF02536	KOG1267		K15032	GO:GO:0006355,GO:GO:0005739,GO:GO:0003690	AT5G07900.1	Mitochondrial transcription termination factor family protein	LOC_Os06g12100.1	mTERF family protein, expressed	2,50	-1.0	1	21	692	712	UGAACAUCCCAGAGCCACCGG	CCCGUGGCUCUGGAAUGUUCA	Cleavage
tae-miR9662a-3p	Traes_4AL_A4B73CC6A1.2	PF02536	KOG1267		K15032	GO:GO:0006355,GO:GO:0005739,GO:GO:0003690	AT5G07900.1	Mitochondrial transcription termination factor family protein	LOC_Os06g12100.1	mTERF family protein, expressed	2,50	-1.0	1	21	693	713	UUGAACAUCCCAGAGCCACCG	CCGUGGCUCUGGAAUGUUCAG	Cleavage
tae-miR9662b-3p	Traes_4AL_A4B73CC6A1.2	PF02536	KOG1267		K15032	GO:GO:0006355,GO:GO:0005739,GO:GO:0003690	AT5G07900.1	Mitochondrial transcription termination factor family protein	LOC_Os06g12100.1	mTERF family protein, expressed	2,50	-1.0	1	21	692	712	UGAACAUCCCAGAGCCACCGG	CCCGUGGCUCUGGAAUGUUCA	Cleavage
tae-miR1130b-3p	Traes_4AL_C55D89913.1	PF03219				GO:GO:0016021,GO:GO:0006810,GO:GO:0005524,GO:GO:0005471	AT5G05310.2	TLC ATP/ADP transporter	LOC_Os11g02159.1	expressed protein	0,00	-1.0	1	21	10	30	UCUUAUAUUAUGGGACGGAGG	CCUCCGUCCCAUAAUAUAAGA	Cleavage
tae-miR1122c-3p	Traes_4AL_C55D89913.1	PF03219				GO:GO:0016021,GO:GO:0006810,GO:GO:0005524,GO:GO:0005471	AT5G05310.2	TLC ATP/ADP transporter	LOC_Os11g02159.1	expressed protein	1,50	-1.0	1	21	10	30	UCUAAUAUUAUGGGACGGAGG	CCUCCGUCCCAUAAUAUAAGA	Cleavage
tae-miR1130b-3p	Traes_4AL_C55D89913.5	PF03219				GO:GO:0016021,GO:GO:0006810,GO:GO:0005524,GO:GO:0005471	AT5G05310.2	TLC ATP/ADP transporter	LOC_Os11g02159.2	expressed protein	0,00	-1.0	1	21	10	30	UCUUAUAUUAUGGGACGGAGG	CCUCCGUCCCAUAAUAUAAGA	Cleavage
tae-miR1122c-3p	Traes_4AL_C55D89913.5	PF03219				GO:GO:0016021,GO:GO:0006810,GO:GO:0005524,GO:GO:0005471	AT5G05310.2	TLC ATP/ADP transporter	LOC_Os11g02159.2	expressed protein	1,50	-1.0	1	21	10	30	UCUAAUAUUAUGGGACGGAGG	CCUCCGUCCCAUAAUAUAAGA	Cleavage
tae-miR1130b-3p	Traes_4AL_C55D89913.6	PF03219				GO:GO:0016021,GO:GO:0006810,GO:GO:0005524,GO:GO:0005471	AT5G05310.2	TLC ATP/ADP transporter	LOC_Os11g02159.1	expressed protein	0,00	-1.0	1	21	10	30	UCUUAUAUUAUGGGACGGAGG	CCUCCGUCCCAUAAUAUAAGA	Cleavage
tae-miR1122c-3p	Traes_4AL_C55D89913.6	PF03219				GO:GO:0016021,GO:GO:0006810,GO:GO:0005524,GO:GO:0005471	AT5G05310.2	TLC ATP/ADP transporter	LOC_Os11g02159.1	expressed protein	1,50	-1.0	1	21	10	30	UCUAAUAUUAUGGGACGGAGG	CCUCCGUCCCAUAAUAUAAGA	Cleavage
tae-miR1130b-3p	Traes_4AL_C55D89913.7	PF03219				GO:GO:0016021,GO:GO:0006810,GO:GO:0005524,GO:GO:0005471	AT5G05310.2	TLC ATP/ADP transporter	LOC_Os11g02159.2	expressed protein	0,00	-1.0	1	21	10	30	UCUUAUAUUAUGGGACGGAGG	CCUCCGUCCCAUAAUAUAAGA	Cleavage
tae-miR1122c-3p	Traes_4AL_C55D89913.7	PF03219				GO:GO:0016021,GO:GO:0006810,GO:GO:0005524,GO:GO:0005471	AT5G05310.2	TLC ATP/ADP transporter	LOC_Os11g02159.2	expressed protein	1,50	-1.0	1	21	10	30	UCUAAUAUUAUGGGACGGAGG	CCUCCGUCCCAUAAUAUAAGA	Cleavage
tae-miR1130b-3p	Traes_4AL_C55D89913.8					GO:GO:0016021,GO:GO:0006810,GO:GO:0005524,GO:GO:0005471	AT5G05310.2	TLC ATP/ADP transporter	LOC_Os11g02159.2	expressed protein	0,00	-1.0	1	21	10	30	UCUUAUAUUAUGGGACGGAGG	CCUCCGUCCCAUAAUAUAAGA	Cleavage
tae-miR1122c-3p	Traes_4AL_C55D89913.8					GO:GO:0016021,GO:GO:0006810,GO:GO:0005524,GO:GO:0005471	AT5G05310.2	TLC ATP/ADP transporter	LOC_Os11g02159.2	expressed protein	1,50	-1.0	1	21	10	30	UCUAAUAUUAUGGGACGGAGG	CCUCCGUCCCAUAAUAUAAGA	Cleavage
tae-miR5049-3p	Traes_4AL_C9E08C423.5										0,00	-1.0	1	21	69	89	AAUAUGGAUCGGAGGGAGUAC	CUACUCCCUCCGAUCCAUAUU	Cleavage
tae-miR9782	Traes_4AL_D576D98B4.1	PF00657		3.1.1.3		GO:GO:0016788	AT5G03810.1	GDSL-like Lipase/Acylhydrolase family protein	LOC_Os03g47940.1	GDSL-like lipase/acylhydrolase, putative, expressed	1,00	-1.0	1	24	2	25	GUAUUAGGUUGGUCAAAUUGACGA	UCGUCAAUUUGACCAACCUAAUAU	Cleavage
tae-miR9782	Traes_4AL_D576D98B4.2			3.1.1.1			AT5G03810.1	GDSL-like Lipase/Acylhydrolase family protein	LOC_Os03g47940.1	GDSL-like lipase/acylhydrolase, putative, expressed	1,00	-1.0	1	24	2	25	GUAUUAGGUUGGUCAAAUUGACGA	UCGUCAAUUUGACCAACCUAAUAU	Cleavage
tae-miR9661-5p	Traes_4AL_D8CCE0D2E.1										2,00	-1.0	1	21	647	667	UGAAGUAGAGCAGGGACCUCA	GGAGGUCUCUGCUCUACUUCC	Cleavage
tae-miR9661-5p	Traes_4AL_D8CCE0D2E.2										2,00	-1.0	1	21	644	664	UGAAGUAGAGCAGGGACCUCA	GGAGGUCUCUGCUCUACUUCC	Cleavage
tae-miR9661-5p	Traes_4AL_D8CCE0D2E.3										2,00	-1.0	1	21	620	640	UGAAGUAGAGCAGGGACCUCA	GGAGGUCUCUGCUCUACUUCC	Cleavage
tae-miR9661-5p	Traes_4AL_D8CCE0D2E.4										2,00	-1.0	1	21	587	607	UGAAGUAGAGCAGGGACCUCA	GGAGGUCUCUGCUCUACUUCC	Cleavage
tae-miR5384-3p	Traes_4AL_E60E5B7BA.1	PF02861	KOG1051		K03695	GO:GO:0019538	AT3G52490.1	Double Clp-N motif-containing P-loop nucleoside triphosphate hydrolases superfamily protein	LOC_Os11g05820.1	transposon protein, putative, Mutator sub-class, expressed	2,50	-1.0	1	21	269	289	UGAGCGCGCCGCCGUCGAAUG	UGUUCCACGGCGGCGCGCACA	Cleavage
tae-miR5384-3p	Traes_4AL_E60E5B7BA.2		KOG1051				AT3G52490.1	Double Clp-N motif-containing P-loop nucleoside triphosphate hydrolases superfamily protein	LOC_Os11g05820.1	transposon protein, putative, Mutator sub-class, expressed	2,50	-1.0	1	21	140	160	UGAGCGCGCCGCCGUCGAAUG	UGUUCCACGGCGGCGCGCACA	Cleavage
tae-miR1130b-3p	Traes_4AS_0E2735576.2			3.4.21.108					LOC_Os05g05330.1	expressed protein	1,50	-1.0	1	21	977	997	UCUUAUAUUAUGGGACGGAGG	CCUCCGUCUCAUAAUAUAAGA	Cleavage
tae-miR1120c-5p	Traes_4AS_0E2735576.2			3.4.21.108					LOC_Os05g05330.1	expressed protein	0,00	-1.0	1	21	1020	1040	UAAUAUAAGAACGUUUUUGAC	GACAAAAACGUUCUUAUAUUA	Cleavage
tae-miR9674a-5p	Traes_4AS_AB9088E9B.2	PF11955					AT5G21970.1	Ubiquitin carboxyl-terminal hydrolase family protein	LOC_Os11g34160.1	ubiquitin carboxyl-terminal hydrolase, family 1, putative, expressed	2,50	-1.0	1	21	1383	1403	GCAUCAUCCAUCCUACCAUUC	UGAUGGUCAGAUGGAUGAUGC	Cleavage
tae-miR9674a-5p	Traes_4AS_AB9088E9B.3	PF11955					AT5G21970.1	Ubiquitin carboxyl-terminal hydrolase family protein	LOC_Os11g34160.1	ubiquitin carboxyl-terminal hydrolase, family 1, putative, expressed	2,50	-1.0	1	21	1383	1403	GCAUCAUCCAUCCUACCAUUC	UGAUGGUCAGAUGGAUGAUGC	Cleavage
tae-miR5384-3p	Traes_4AS_C7CBD4A47.1	PF03703					AT1G14345.1	NAD(P)-linked oxidoreductase superfamily protein	LOC_Os03g07440.1	putativ eribonuclease P, putative, expressed	2,50	-1.0	1	21	194	214	UGAGCGCGCCGCCGUCGAAUG	UCUUCGACGGCGGCGCGCACU	Cleavage
tae-miR1135	Traes_4AS_D533B306B.3	PF01485	KOG1815	6.3.2.19		GO:GO:0016567,GO:GO:0004842	AT2G16090.1	RING/U-box superfamily protein	LOC_Os03g13100.1	ariadne-1, putative, expressed	2,00	-1.0	1	24	1698	1721	CUGCGACAAGUAAUUCCGAACGGA	CGAACCAGGAAUUACUUGUCGCAG	Cleavage
tae-miR1131	Traes_4BL_178D6FFF1.1										1,50	-1.0	1	22	167	188	UAGUACCGGUUCGUGGCUAACC	GGUUAGCCACGAACCGAUACUA	Cleavage
tae-miR1131	Traes_4BL_178D6FFF1.2										1,50	-1.0	1	22	167	188	UAGUACCGGUUCGUGGCUAACC	GGUUAGCCACGAACCGAUACUA	Cleavage
tae-miR9662a-3p	Traes_4BL_29D8171E0.1								LOC_Os06g12100.1	mTERF family protein, expressed	2,00	-1.0	1	21	132	152	UUGAACAUCCCAGAGCCACCG	UCGUGGCGCUGGGAUGUUCAG	Cleavage
tae-miR9662b-3p	Traes_4BL_29D8171E0.1								LOC_Os06g12100.1	mTERF family protein, expressed	2,50	-1.0	1	21	131	151	UGAACAUCCCAGAGCCACCGG	CUCGUGGCGCUGGGAUGUUCA	Cleavage
tae-miR1122b-3p	Traes_4BL_2D34597CF.2	PF04068,PF04034					AT5G10070.2	RNase L inhibitor protein-related	LOC_Os03g09920.1	RNase L inhibitor protein-related, putative, expressed	2,50	-1.0	1	21	454	474	AGACUUAUAUGUAGGAACGGA	UCUGUUCCUAAAUAUAAGUCU	Translation
tae-miR1122b-3p	Traes_4BL_2D34597CF.3	PF04068,PF04034					AT5G10070.2	RNase L inhibitor protein-related	LOC_Os03g09920.1	RNase L inhibitor protein-related, putative, expressed	2,50	-1.0	1	21	454	474	AGACUUAUAUGUAGGAACGGA	UCUGUUCCUAAAUAUAAGUCU	Translation
tae-miR1128	Traes_4BL_37FDD905B.1	PF10250					AT1G04910.1	O-fucosyltransferase family protein	LOC_Os11g29120.2	growth regulator related protein, putative, expressed	2,50	-1.0	1	21	125	145	UACUACUCCCUCCGUCCGAAA	UUUGGGACGGAGGGAGUAGGA	Cleavage
tae-miR5384-3p	Traes_4BL_3B7A98FD6.1	PF03703					AT1G14345.1	NAD(P)-linked oxidoreductase superfamily protein	LOC_Os03g07440.1	putativ eribonuclease P, putative, expressed	2,50	-1.0	1	21	191	211	UGAGCGCGCCGCCGUCGAAUG	UCUUCGACGGCGGCGCGCACU	Cleavage
tae-miR9666a-3p	Traes_4BL_44010E331.1										3,00	-1.0	1	22	176	197	CGGUAGGGCUGUAUGAUGGCGA	UCGGCAUGAUACAGCCCUACCU	Cleavage
tae-miR1122b-3p	Traes_4BL_62355968B.1	PF00013				GO:GO:0003723	AT5G15270.2	RNA-binding KH domain-containing protein	LOC_Os03g25970.1	KH domain-containing protein, putative, expressed	2,50	-1.0	1	21	706	726	AGACUUAUAUGUAGGAACGGA	UCUGUUCCUAAAUAUAAGUCU	Translation
tae-miR1122a	Traes_4BL_62355968B.1	PF00013				GO:GO:0003723	AT5G15270.2	RNA-binding KH domain-containing protein	LOC_Os03g25970.1	KH domain-containing protein, putative, expressed	2,50	-1.0	1	20	747	766	UAGAUACAUCCGUAUCUAGA	ACUACAUACGGAUGUAUAUA	Cleavage
tae-miR1137a	Traes_4BL_D08D5FFB5.2	PF08282	KOG3189	5.4.2.8		GO:GO:0009298,GO:GO:0005737,GO:GO:0004615	AT2G45790.1	phosphomannomutase	LOC_Os04g58580.1	phosphomannomutase, putative, expressed	2,00	-1.0	1	20	722	741	UAGUACAAAGUUGAGUCAUC	AGUGUCUCAACUUUGUACUA	Cleavage
tae-miR1137a	Traes_4BL_F3D5D7034.2						AT3G16990.1	Haem oxygenase-like, multi-helical	LOC_Os03g19390.1	TENA/THI-4 family protein, putative, expressed	1,00	-1.0	1	19	1	19	UAGUACAAAGUUGAGUCAU	AUGACCCAACUUUGUACUA	Cleavage
tae-miR1137b-5p	Traes_4BL_F3D5D7034.2						AT3G16990.1	Haem oxygenase-like, multi-helical	LOC_Os03g19390.1	TENA/THI-4 family protein, putative, expressed	1,50	-1.0	1	21	37	57	UCCGUUCCAGAAUAGAUGACC	GGUCAUCUAUUUUGGAACGGA	Translation
tae-miR9662a-3p	Traes_4BS_257C5B099.1	PF02536				GO:GO:0006355,GO:GO:0005739,GO:GO:0003690	AT5G07900.1	Mitochondrial transcription termination factor family protein	LOC_Os06g12100.1	mTERF family protein, expressed	2,00	-1.0	1	21	66	86	UUGAACAUCCCAGAGCCACCG	CCGUGGUUCUGGGAUGUUCAG	Cleavage
tae-miR9662b-3p	Traes_4BS_257C5B099.1	PF02536				GO:GO:0006355,GO:GO:0005739,GO:GO:0003690	AT5G07900.1	Mitochondrial transcription termination factor family protein	LOC_Os06g12100.1	mTERF family protein, expressed	2,00	-1.0	1	21	65	85	UGAACAUCCCAGAGCCACCGG	CCCGUGGUUCUGGGAUGUUCA	Cleavage
tae-miR156	Traes_4BS_375EF1D15.1								LOC_Os01g69830.1	OsSPL2 - SBP-box gene family member, expressed	2,50	-1.0	1	21	235	255	UGACAGAAGAGAGUGAGCACA	UGUGCUCUCUCUUUUCUGUCA	Cleavage
tae-miR1118	Traes_4BS_4BC02509F.1						AT3G43790.3	zinc induced facilitator-like 2	LOC_Os12g03830.1	major facilitator superfamily antiporter, putative, expressed	2,50	-1.0	1	23	525	547	CACUACAUUAUGGAAUGGAGGGA	UCCCUCCGUUCCAUAAUGUAAUG	Cleavage
tae-miR9666a-3p	Traes_4BS_5419B1DE7.1										3,00	-1.0	1	22	176	197	CGGUAGGGCUGUAUGAUGGCGA	UCGGCAUGAUACAGCCCUACCU	Cleavage
tae-miR9661-5p	Traes_4BS_5EC01BC81.1										2,00	-1.0	1	21	653	673	UGAAGUAGAGCAGGGACCUCA	GGAGGUCUCUGCUCUACUUCC	Cleavage
tae-miR9661-5p	Traes_4BS_5EC01BC81.2										2,00	-1.0	1	21	653	673	UGAAGUAGAGCAGGGACCUCA	GGAGGUCUCUGCUCUACUUCC	Cleavage
tae-miR9661-5p	Traes_4BS_5EC01BC81.3										2,00	-1.0	1	21	653	673	UGAAGUAGAGCAGGGACCUCA	GGAGGUCUCUGCUCUACUUCC	Cleavage
tae-miR1122b-3p	Traes_4BS_6252B0BA0.1	PF05562					AT1G29395.1	COLD REGULATED 314 INNER MEMBRANE 1	LOC_Os05g49170.1	cold acclimation protein WCOR413, putative, expressed	1,50	-1.0	1	21	46	66	AGACUUAUAUGUAGGAACGGA	UCCGUUCCUAAAUAUAAGUCU	Translation
tae-miR7757-5p	Traes_4BS_B45729F4D.4	PF00931				GO:GO:0043531	AT3G07040.1	NB-ARC domain-containing disease resistance protein	LOC_Os11g11810.1	NBS-LRR disease resistance protein, putative, expressed	2,50	-1.0	1	22	653	674	AUAAAACCUUCAGCUAUCCAUC	AAUGGAUAUCUGAAGGUUUCAU	Cleavage
tae-miR5384-3p	Traes_4BS_F1D7488B8.1	PF02861	KOG1051		K03695	GO:GO:0019538	AT3G52490.1	Double Clp-N motif-containing P-loop nucleoside triphosphate hydrolases superfamily protein	LOC_Os11g05820.1	transposon protein, putative, Mutator sub-class, expressed	2,50	-1.0	1	21	269	289	UGAGCGCGCCGCCGUCGAAUG	UGUUCCACGGCGGCGCGCACA	Cleavage
tae-miR1122a	Traes_4BS_F395A220A.2	PF01595	KOG2118				AT2G14520.1	CBS domain-containing protein with a domain of unknown function (DUF21)	LOC_Os03g47120.1	CBS domain containing protein, expressed	0,00	-1.0	1	20	1336	1355	UAGAUACAUCCGUAUCUAGA	UCUAGAUACGGAUGUAUCUA	Cleavage
tae-miR7757-5p	Traes_4DL_113F6DD2B.1	PF00931				GO:GO:0043531	AT1G50180.1	NB-ARC domain-containing disease resistance protein	LOC_Os12g13550.1	NBS-LRR disease resistance protein, putative, expressed	2,50	-1.0	1	22	215	236	AUAAAACCUUCAGCUAUCCAUC	AAUGGAUGGCUGAAGGCUUUAU	Cleavage
tae-miR7757-5p	Traes_4DL_113F6DD2B.2	PF00931				GO:GO:0043531	AT1G50180.1	NB-ARC domain-containing disease resistance protein	LOC_Os10g22484.2	RGH2B, putative, expressed	2,50	-1.0	1	22	215	236	AUAAAACCUUCAGCUAUCCAUC	AAUGGAUGGCUGAAGGCUUUAU	Cleavage
tae-miR5085	Traes_4DL_341830EFF.1										2,50	-1.0	1	21	583	603	AAGGACAUUUUUUGUGGCCUG	GAGGACACAAAAAAUGUCGUU	Cleavage
tae-miR1133	Traes_4DL_529524815.5						AT1G67250.1	Proteasome maturation factor UMP1	LOC_Os02g55640.1	proteasome maturation factor UMP1 family protein, expressed	2,00	-1.0	1	22	103	127	CAUAUACUCCCUCCG-UCCGA--AA	UUAGUCGGAUCGGAGGGAGUAUAUG	Cleavage
tae-miR1130b-3p	Traes_4DL_84745919A.2			2.7.7.19			AT3G51620.2	PAP/OAS1 substrate-binding domain superfamily	LOC_Os03g21850.1	nucleotidyltransferase, putative, expressed	2,50	-1.0	1	21	2964	2984	UCUUAUAUUAUGGGACGGAGG	CCUCUGUCCCAUAAUAUAGGA	Cleavage
tae-miR1130a	Traes_4DL_84745919A.2			2.7.7.19			AT3G51620.2	PAP/OAS1 substrate-binding domain superfamily	LOC_Os03g21850.1	nucleotidyltransferase, putative, expressed	1,50	-1.0	1	23	3016	3038	CCUCCGUCUCGUAAUGUAAGACG	UGUCUUACAUUAUGAGACGGAGG	Translation
tae-miR1128	Traes_4DL_93D4DED90.3	PF00009		3.6.5.3	K03231		AT5G60390.3	GTP binding Elongation factor Tu family protein	LOC_Os03g08010.1	elongation factor Tu, putative, expressed	3,00	-1.0	1	21	565	586	UACUACUCCCUCCG-UCCGAAA	UUUUGGAACGGAGGGAGUAGUA	Cleavage
tae-miR1137a	Traes_4DL_93D4DED90.3	PF00009		3.6.5.3	K03231		AT5G60390.3	GTP binding Elongation factor Tu family protein	LOC_Os03g08010.1	elongation factor Tu, putative, expressed	2,50	-1.0	1	20	508	527	UAGUACAAAGUUGAGUCAUC	GAUGACCCAACUUUGUAUUA	Cleavage
tae-miR1137b-5p	Traes_4DL_93D4DED90.3	PF00009		3.6.5.3	K03231		AT5G60390.3	GTP binding Elongation factor Tu family protein	LOC_Os03g08010.1	elongation factor Tu, putative, expressed	1,50	-1.0	1	21	556	576	UCCGUUCCAGAAUAGAUGACC	GGUCAUCUAUUUUGGAACGGA	Translation
tae-miR1137b-5p	Traes_4DS_28E6C4F21.2										1,50	-1.0	1	21	61	81	UCCGUUCCAGAAUAGAUGACC	AGUCAUCUAUUUUGGAACGGA	Translation
tae-miR1137b-5p	Traes_4DS_28E6C4F21.3										1,50	-1.0	1	21	61	81	UCCGUUCCAGAAUAGAUGACC	AGUCAUCUAUUUUGGAACGGA	Translation
tae-miR1137b-5p	Traes_4DS_28E6C4F21.4										1,50	-1.0	1	21	61	81	UCCGUUCCAGAAUAGAUGACC	AGUCAUCUAUUUUGGAACGGA	Translation
tae-miR7757-5p	Traes_4DS_5B29EE3DD.5	PF00931				GO:GO:0043531	AT3G50950.1	HOPZ-ACTIVATED RESISTANCE 1	LOC_Os11g11810.1	NBS-LRR disease resistance protein, putative, expressed	2,50	-1.0	1	22	1379	1400	AUAAAACCUUCAGCUAUCCAUC	AAUGGAUAUCUGAAGGUUUCAU	Cleavage
tae-miR7757-5p	Traes_4DS_5B29EE3DD.6	PF00931				GO:GO:0043531	AT3G50950.1	HOPZ-ACTIVATED RESISTANCE 1	LOC_Os11g11810.1	NBS-LRR disease resistance protein, putative, expressed	2,50	-1.0	1	22	1379	1400	AUAAAACCUUCAGCUAUCCAUC	AAUGGAUAUCUGAAGGUUUCAU	Cleavage
tae-miR7757-5p	Traes_4DS_5B29EE3DD.7	PF00931				GO:GO:0043531	AT1G58390.1	Disease resistance protein (CC-NBS-LRR class) family	LOC_Os11g11810.1	NBS-LRR disease resistance protein, putative, expressed	2,50	-1.0	1	22	824	845	AUAAAACCUUCAGCUAUCCAUC	AAUGGAUAUCUGAAGGUUUCAU	Cleavage
tae-miR1130b-3p	Traes_4DS_9179FF158.4	PF06964		3.2.1.55		GO:GO:0046556,GO:GO:0046373	AT3G10740.1	alpha-L-arabinofuranosidase 1	LOC_Os12g03470.1	alpha-N-arabinofuranosidase A, putative, expressed	1,50	-1.0	1	21	41	61	UCUUAUAUUAUGGGACGGAGG	CCUCCGUCCCAUAAUGUAAGA	Cleavage
tae-miR9778	Traes_4DS_B08D83A2F.1	PF00931				GO:GO:0043531	AT3G46530.1	NB-ARC domain-containing disease resistance protein	LOC_Os11g39550.1	Leucine Rich Repeat family protein, expressed	2,50	-1.0	1	21	126	146	UGCAUCAUCUCGAACUCGUCG	UGGCGAGUUCCAGAUGAUGCA	Translation
tae-miR164	Traes_4DS_C08BF9DB2.1			2.4.1.14			AT1G04920.1	sucrose phosphate synthase 3F	LOC_Os11g12810.1	sucrose-phosphate synthase, putative, expressed	2,50	-1.0	1	21	387	407	UGGAGAAGCAGGGCACGUGCA	UUCACGUGCCCUGCUUUUCCU	Cleavage
tae-miR1128	Traes_4DS_C22A101FE.2						AT1G07910.1	RNAligase	LOC_Os07g09400.1	RNA ligase isoform 1, putative, expressed	2,50	-1.0	1	21	670	690	UACUACUCCCUCCGUCCGAAA	UUUGGGACGAAGGGAGUAGUA	Cleavage
tae-miR1128	Traes_4DS_C22A101FE.9						AT1G07910.1	RNAligase	LOC_Os07g09400.1	RNA ligase isoform 1, putative, expressed	2,50	-1.0	1	21	358	378	UACUACUCCCUCCGUCCGAAA	UUUGGGACGAAGGGAGUAGUA	Cleavage
tae-miR1122a	Traes_4DS_E8239194E.2	PF01394					AT3G08530.1	Clathrin, heavy chain	LOC_Os12g01390.1	clathrin heavy chain, putative, expressed	2,50	-1.0	1	20	692	711	UAGAUACAUCCGUAUCUAGA	UCUAAAUACGGAUGUAUCAA	Cleavage
tae-miR5384-3p	Traes_4DS_ECFDD82BA.1	PF02861	KOG1051		K03695	GO:GO:0019538	AT3G52490.1	Double Clp-N motif-containing P-loop nucleoside triphosphate hydrolases superfamily protein	LOC_Os11g05820.1	transposon protein, putative, Mutator sub-class, expressed	2,50	-1.0	1	21	269	289	UGAGCGCGCCGCCGUCGAAUG	UGUUCCACGGCGGCGCGCACA	Cleavage
tae-miR1137b-5p	Traes_4DS_EFE0451D7.3	PF00270		3.6.4.12		GO:GO:0005524,GO:GO:0003676	AT1G31360.1	RECQ helicase L2	LOC_Os11g48090.5	helicase conserved C-terminal domain containing protein, expressed	1,50	-1.0	1	21	950	970	UCCGUUCCAGAAUAGAUGACC	AGUCAUCUAUUUUGGAACGGA	Translation
tae-miR1128	Traes_5AL_3AB81DF9B.1	PF00056,PF02866	KOG1496	1.1.1.82		GO:GO:0055114,GO:GO:0016491,GO:GO:0016616,GO:GO:0016615,GO:GO:0006108	AT5G58330.1	lactate/malate dehydrogenase family protein	LOC_Os08g44810.1	lactate/malate dehydrogenase, putative, expressed	3,00	-1.0	1	21	151	172	UACUACUCCCUCCG-UCCGAAA	UUUAGGAACGGAGGGAGUAGUA	Cleavage
tae-miR9652-5p	Traes_5AL_481F73E59.1										2,50	-1.0	1	22	883	904	CCUGUUUGUCAUUAAGUUUCUU	AAGAAACUGAAUGACAAGCAGG	Cleavage
tae-miR9652-5p	Traes_5AL_481F73E59.2										2,50	-1.0	1	22	883	904	CCUGUUUGUCAUUAAGUUUCUU	AAGAAACUGAAUGACAAGCAGG	Cleavage
tae-miR9652-5p	Traes_5AL_481F73E59.3										2,50	-1.0	1	22	883	904	CCUGUUUGUCAUUAAGUUUCUU	AAGAAACUGAAUGACAAGCAGG	Cleavage
tae-miR9652-5p	Traes_5AL_481F73E59.4										2,50	-1.0	1	22	883	904	CCUGUUUGUCAUUAAGUUUCUU	AAGAAACUGAAUGACAAGCAGG	Cleavage
tae-miR9652-5p	Traes_5AL_481F73E59.5										2,50	-1.0	1	22	583	604	CCUGUUUGUCAUUAAGUUUCUU	AAGAAACUGAAUGACAAGCAGG	Cleavage
tae-miR9652-5p	Traes_5AL_481F73E59.6										2,50	-1.0	1	22	883	904	CCUGUUUGUCAUUAAGUUUCUU	AAGAAACUGAAUGACAAGCAGG	Cleavage
tae-miR5062-5p	Traes_5AL_500FF0B72.1	PF02171				GO:GO:0003676	AT2G27880.1	Argonaute family protein	LOC_Os03g58600.1	PAZ domain containing protein, putative, expressed	2,50	-1.0	1	23	43	65	UGAACCUUAGGGAACAGCCGCAU	ACCAGGCUAUUCCCUGAGGUUCA	Cleavage
tae-miR5062-5p	Traes_5AL_60D577E34.1	PF02171				GO:GO:0003676	AT2G27880.1	Argonaute family protein	LOC_Os03g58600.1	PAZ domain containing protein, putative, expressed	1,50	-1.0	1	23	43	65	UGAACCUUAGGGAACAGCCGCAU	ACCAGGCUGUUCCCUGAGGUUCA	Cleavage
tae-miR1127b-3p	Traes_5AL_7256A9F19.2	PF00400	KOG0279			GO:GO:0005515	AT3G18860.2	transducin family protein / WD-40 repeat family protein	LOC_Os07g03160.1	WD domain, G-beta repeat domain containing protein, expressed	1,50	-1.0	1	21	991	1011	ACAAGUAUUUCUGGACGGAGG	CCUCCGUCCGGAAAUACUUGU	Cleavage
tae-miR1122b-3p	Traes_5AL_7FF780810.1						AT5G41940.1	Ypt/Rab-GAP domain of gyp1p superfamily protein	LOC_Os09g35960.1	TBC domain containing protein, expressed	1,50	-1.0	1	21	22	42	AGACUUAUAUGUAGGAACGGA	UCCGUUCCUAAAUAUAAGUCU	Translation
tae-miR5086	Traes_5AL_8B87707C4.1										3,00	-1.0	1	21	350	370	ACAUUGGUGGAAGGCGUGGUA	AGUCAUGCCUUCCACCAAUGA	Cleavage
tae-miR5086	Traes_5AL_8B87707C4.2										3,00	-1.0	1	21	350	370	ACAUUGGUGGAAGGCGUGGUA	AGUCAUGCCUUCCACCAAUGA	Cleavage
tae-miR5086	Traes_5AL_8B87707C4.3										3,00	-1.0	1	21	326	346	ACAUUGGUGGAAGGCGUGGUA	AGUCAUGCCUUCCACCAAUGA	Cleavage
tae-miR7757-5p	Traes_5AL_B4E8A3115.1	PF00931				GO:GO:0043531	AT3G46710.1	NB-ARC domain-containing disease resistance protein	LOC_Os05g40150.1	RGH2B, putative, expressed	1,50	-1.0	1	22	368	389	AUAAAACCUUCAGCUAUCCAUC	AAUGGAUAGCUGAAGGUCUUAU	Cleavage
tae-miR7757-5p	Traes_5AL_B4E8A3115.2	PF03106,PF12796,PF00931	KOG4658			GO:GO:0043565,GO:GO:0006355,GO:GO:0003700,GO:GO:0043531	AT2G31820.1	Ankyrin repeat family protein	LOC_Os11g45750.2	WRKY125, expressed	1,50	-1.0	1	22	2324	2345	AUAAAACCUUCAGCUAUCCAUC	AAUGGAUAGCUGAAGGUCUUAU	Cleavage
tae-miR7757-5p	Traes_5AL_B4E8A3115.3	PF03106,PF12796,PF00931	KOG4658			GO:GO:0043565,GO:GO:0006355,GO:GO:0003700,GO:GO:0043531	AT2G31820.1	Ankyrin repeat family protein	LOC_Os11g45750.2	WRKY125, expressed	1,50	-1.0	1	22	2324	2345	AUAAAACCUUCAGCUAUCCAUC	AAUGGAUAGCUGAAGGUCUUAU	Cleavage
tae-miR7757-5p	Traes_5AL_B4E8A3115.4	PF03106,PF12796,PF00931	KOG4658			GO:GO:0043565,GO:GO:0006355,GO:GO:0003700,GO:GO:0043531	AT2G31820.1	Ankyrin repeat family protein	LOC_Os11g45750.2	WRKY125, expressed	1,50	-1.0	1	22	2324	2345	AUAAAACCUUCAGCUAUCCAUC	AAUGGAUAGCUGAAGGUCUUAU	Cleavage
tae-miR7757-5p	Traes_5AL_B4E8A3115.5	PF03106,PF12796,PF00931	KOG4658			GO:GO:0043565,GO:GO:0006355,GO:GO:0003700,GO:GO:0043531	AT2G31820.1	Ankyrin repeat family protein	LOC_Os11g45750.1	WRKY125, expressed	1,50	-1.0	1	22	2324	2345	AUAAAACCUUCAGCUAUCCAUC	AAUGGAUAGCUGAAGGUCUUAU	Cleavage
tae-miR7757-5p	Traes_5AL_B4E8A3115.6	PF00931,PF03106				GO:GO:0043531,GO:GO:0043565,GO:GO:0006355,GO:GO:0003700	AT3G46710.1	NB-ARC domain-containing disease resistance protein	LOC_Os05g40150.1	RGH2B, putative, expressed	1,50	-1.0	1	22	368	389	AUAAAACCUUCAGCUAUCCAUC	AAUGGAUAGCUGAAGGUCUUAU	Cleavage
tae-miR9652-5p	Traes_5AL_C9236F111.1										2,50	-1.0	1	22	778	799	CCUGUUUGUCAUUAAGUUUCUU	AAGAAACUGAAUGACAAGCAGG	Cleavage
tae-miR5384-3p	Traes_5AS_A928BA201.1	PF04535					AT5G44550.1	Uncharacterised protein family (UPF0497)	LOC_Os12g41690.1	membrane associated DUF588 domain containing protein, putative, expressed	2,50	-1.0	1	21	506	526	UGAGCGCGCCGCCGUCGAAUG	CAUUCGUCGGCGUCGCGCUCA	Cleavage
tae-miR7757-5p	Traes_5AS_BDFB08E23.1	PF00931	KOG4658			GO:GO:0043531	AT3G07040.1	NB-ARC domain-containing disease resistance protein	LOC_Os12g37740.1	resistance protein LR10, putative, expressed	2,50	-1.0	1	22	1349	1370	AUAAAACCUUCAGCUAUCCAUC	GAUGGAUUGCUGAAGGUUUCAU	Cleavage
tae-miR7757-5p	Traes_5AS_BDFB08E23.2	PF00931	KOG4658			GO:GO:0043531	AT3G07040.1	NB-ARC domain-containing disease resistance protein	LOC_Os12g37740.1	resistance protein LR10, putative, expressed	2,50	-1.0	1	22	1349	1370	AUAAAACCUUCAGCUAUCCAUC	GAUGGAUUGCUGAAGGUUUCAU	Cleavage
tae-miR7757-5p	Traes_5AS_BDFB08E23.3	PF00931				GO:GO:0043531	AT3G07040.1	NB-ARC domain-containing disease resistance protein	LOC_Os12g37740.1	resistance protein LR10, putative, expressed	2,50	-1.0	1	22	524	545	AUAAAACCUUCAGCUAUCCAUC	GAUGGAUUGCUGAAGGUUUCAU	Cleavage
tae-miR7757-5p	Traes_5AS_BDFB08E23.4	PF00931				GO:GO:0043531	AT3G46530.1	NB-ARC domain-containing disease resistance protein	LOC_Os12g37740.1	resistance protein LR10, putative, expressed	2,50	-1.0	1	22	1349	1370	AUAAAACCUUCAGCUAUCCAUC	GAUGGAUUGCUGAAGGUUUCAU	Cleavage
tae-miR7757-5p	Traes_5AS_BDFB08E23.5	PF00931				GO:GO:0043531	AT3G46530.1	NB-ARC domain-containing disease resistance protein	LOC_Os12g37740.1	resistance protein LR10, putative, expressed	2,50	-1.0	1	22	1349	1370	AUAAAACCUUCAGCUAUCCAUC	GAUGGAUUGCUGAAGGUUUCAU	Cleavage
tae-miR1128	Traes_5AS_E1F692526.2										3,00	-1.0	1	21	111	132	UACUACUCCCUCCGUCCGA-AA	UUAUGGGACGGAGGGAGUAGUA	Cleavage
tae-miR1130b-3p	Traes_5AS_E1F692526.2										3,00	-1.0	1	21	40	61	UCUU-AUAUUAUGGGACGGAGG	CCUCCGUCCCAUAAUAUUAAGA	Cleavage
tae-miR1122c-3p	Traes_5AS_E1F692526.2										3,00	-1.0	1	21	40	61	UC-UAAUAUUAUGGGACGGAGG	CCUCCGUCCCAUAAUAUUAAGA	Cleavage
tae-miR164	Traes_5AS_E5FD82E36.1	PF02365				GO:GO:0006355,GO:GO:0003677	AT1G56010.2	NAC domain containing protein 1	LOC_Os12g41680.1	No apical meristem protein, putative, expressed	1,00	-1.0	1	21	653	673	UGGAGAAGCAGGGCACGUGCA	AGCAAGUGCCCUGCUUCUCCA	Cleavage
tae-miR164	Traes_5AS_E5FD82E36.2	PF02365				GO:GO:0006355,GO:GO:0003677	AT1G56010.2	NAC domain containing protein 1	LOC_Os12g41680.1	No apical meristem protein, putative, expressed	1,00	-1.0	1	21	656	676	UGGAGAAGCAGGGCACGUGCA	AGCAAGUGCCCUGCUUCUCCA	Cleavage
tae-miR1118	Traes_5AS_EC41EF7CA.4	PF00013	KOG2190			GO:GO:0003723	AT3G04610.1	RNA-binding KH domain-containing protein	LOC_Os12g40560.1	KH domain containing protein, putative, expressed	1,50	-1.0	1	23	1333	1355	CACUACAUUAUGGAAUGGAGGGA	UCCCUCCAUUCCACAAUGUAGUG	Translation
tae-miR1125	Traes_5AS_EC41EF7CA.4	PF00013	KOG2190			GO:GO:0003723	AT3G04610.1	RNA-binding KH domain-containing protein	LOC_Os12g40560.1	KH domain containing protein, putative, expressed	1,00	-1.0	1	24	1450	1473	AACCAACGAGACCAACUGCGGCGG	CCGCCGCAGUCGGUCUCGUUGGUU	Cleavage
tae-miR408	Traes_5BL_0212ACBD9.1	PF05911	KOG0999				AT1G77580.2	Plant protein of unknown function (DUF869)	LOC_Os12g07160.2	plant protein of unknown function DUF869 domain containing protein, expressed	2,50	-1.0	1	21	2512	2532	CUGCACUGCCUCUUCCCUGGC	GCCAAGGAAGAGGCUGUGCAG	Cleavage
tae-miR1137a	Traes_5BL_0EC4788EF.2	PF08244,PF00251	KOG0228	3.2.1.26			AT1G55120.1	beta-fructofuranosidase 5	LOC_Os01g73580.1	glycosyl hydrolases, putative, expressed	1,00	-1.0	1	20	1543	1562	UAGUACAAAGUUGAGUCAUC	GAUGACCCAACUUUGUACUA	Cleavage
tae-miR9652-5p	Traes_5BL_0F64FDA44.1										2,50	-1.0	1	22	583	604	CCUGUUUGUCAUUAAGUUUCUU	AAGAAACUGAAUGACAAGCAGG	Cleavage
tae-miR1122a	Traes_5BL_1B9186ED8.1	PF07762							LOC_Os11g07360.1	expressed protein	2,50	-1.0	1	20	986	1005	UAGAUACAUCCGUAUCUAGA	UCGAGAUACAGAUGUAUCUA	Translation
tae-miR7757-5p	Traes_5BL_1E751EF1F.1	PF00931,PF03514	KOG4658			GO:GO:0043531	AT1G50600.1	scarecrow-like 5	LOC_Os10g22484.1	RGH2B, putative, expressed	2,00	-1.0	1	22	1799	1820	AUAAAACCUUCAGCUAUCCAUC	AAUGGGUUGCUGAAGGUUUUAU	Cleavage
tae-miR7757-5p	Traes_5BL_1E751EF1F.2	PF00931				GO:GO:0043531	AT3G46730.1	NB-ARC domain-containing disease resistance protein	LOC_Os10g22484.1	RGH2B, putative, expressed	2,00	-1.0	1	22	1799	1820	AUAAAACCUUCAGCUAUCCAUC	AAUGGGUUGCUGAAGGUUUUAU	Cleavage
tae-miR7757-5p	Traes_5BL_1E751EF1F.5	PF00931,PF03514				GO:GO:0043531	AT1G50600.1	scarecrow-like 5	LOC_Os10g22484.1	RGH2B, putative, expressed	2,00	-1.0	1	22	512	533	AUAAAACCUUCAGCUAUCCAUC	AAUGGGUUGCUGAAGGUUUUAU	Cleavage
tae-miR5049-3p	Traes_5BL_4BFE76791.5								LOC_Os08g04590.1	csAtPR5, putative, expressed	2,50	-1.0	1	21	416	436	AAUAUGGAUCGGAGGGAGUAC	AUGCUCCCUCUGAUCCAUAUU	Translation
tae-miR1127b-3p	Traes_5BL_5E94C243F.1						AT3G54630.1		LOC_Os08g36490.1	kinetochore protein, putative, expressed	2,50	-1.0	1	21	914	934	ACAAGUAUUUCUGGACGGAGG	CCUCUGUCCUGAAAUACUUGU	Cleavage
tae-miR1127b-3p	Traes_5BL_5E94C243F.2						AT3G54630.1		LOC_Os08g36490.1	kinetochore protein, putative, expressed	2,50	-1.0	1	21	898	918	ACAAGUAUUUCUGGACGGAGG	CCUCUGUCCUGAAAUACUUGU	Cleavage
tae-miR9674b-5p	Traes_5BL_6216A6108.1	PF13041					AT3G22470.1	Pentatricopeptide repeat (PPR) superfamily protein	LOC_Os10g35240.2	Rf1, mitochondrial precursor, putative, expressed	3,00	-1.0	1	21	411	431	AUAGCAUCAUCCAUCCUACCC	GGGCCGAAUGGAUGAUGCUAU	Cleavage
tae-miR5384-3p	Traes_5BL_6240DE6BA.1	PF00067		1.14.13.89		GO:GO:0055114,GO:GO:0020037,GO:GO:0016705,GO:GO:0005506	AT5G67310.1	cytochrome P450, family 81, subfamily G, polypeptide 1	LOC_Os03g55240.1	cytochrome P450, putative, expressed	2,50	-1.0	1	21	338	358	UGAGCGCGCCGCCGUCGAAUG	CCUUCGACGGCGCCGCGCUCG	Cleavage
tae-miR5384-3p	Traes_5BL_6240DE6BA.2	PF00067		1.14.13.89		GO:GO:0055114,GO:GO:0020037,GO:GO:0016705,GO:GO:0005506	AT5G67310.1	cytochrome P450, family 81, subfamily G, polypeptide 1	LOC_Os03g55240.1	cytochrome P450, putative, expressed	2,50	-1.0	1	21	338	358	UGAGCGCGCCGCCGUCGAAUG	CCUUCGACGGCGCCGCGCUCG	Cleavage
tae-miR5384-3p	Traes_5BL_6240DE6BA.3	PF00067		1.14.13.89		GO:GO:0055114,GO:GO:0020037,GO:GO:0016705,GO:GO:0005506	AT5G67310.1	cytochrome P450, family 81, subfamily G, polypeptide 1	LOC_Os03g55240.1	cytochrome P450, putative, expressed	2,50	-1.0	1	21	338	358	UGAGCGCGCCGCCGUCGAAUG	CCUUCGACGGCGCCGCGCUCG	Cleavage
tae-miR5384-3p	Traes_5BL_6240DE6BA.4	PF00067		1.14.13.89		GO:GO:0055114,GO:GO:0020037,GO:GO:0016705,GO:GO:0005506	AT5G67310.1	cytochrome P450, family 81, subfamily G, polypeptide 1	LOC_Os03g55240.1	cytochrome P450, putative, expressed	2,50	-1.0	1	21	338	358	UGAGCGCGCCGCCGUCGAAUG	CCUUCGACGGCGCCGCGCUCG	Cleavage
tae-miR1122a	Traes_5BL_637A3C970.8	PF01501		2.4.1.43		GO:GO:0016757,GO:GO:0047262	AT5G47780.1	galacturonosyltransferase 4	LOC_Os09g30280.1	glycosyl transferase 8 domain containing protein, putative, expressed	1,50	-1.0	1	20	49	68	UAGAUACAUCCGUAUCUAGA	UCUAGAUACGAAUGUAUCUA	Translation
tae-miR1121	Traes_5BL_72EFF0AF0.1	PF07690				GO:GO:0055085,GO:GO:0016021	AT5G59250.1	Major facilitator superfamily protein	LOC_Os10g42830.1	transporter family protein, putative, expressed	0,00	-1.0	1	22	1237	1258	AGUAGUGAUCUAAACGCUCUUA	UAAGAGCGUUUAGAUCACUACU	Cleavage
tae-miR408	Traes_5BL_7486582EB.1	PF02298				GO:GO:0009055	AT2G02850.1	plantacyanin	LOC_Os03g50160.1	plastocyanin-like domain containing protein, putative, expressed	2,50	-1.0	1	21	8	28	CUGCACUGCCUCUUCCCUGGC	CGCAGGGAAGAGGCAGUGCCA	Cleavage
tae-miR1122a	Traes_5BL_7821274AB.1	PF07762							LOC_Os11g07360.1	expressed protein	2,50	-1.0	1	20	986	1005	UAGAUACAUCCGUAUCUAGA	UCGAGAUACAGAUGUAUCUA	Translation
tae-miR7757-5p	Traes_5BL_7F0FD1538.2	PF00847,PF00931	KOG4658			GO:GO:0006355,GO:GO:0003700,GO:GO:0043531	AT3G46710.1	NB-ARC domain-containing disease resistance protein	LOC_Os05g40150.1	RGH2B, putative, expressed	1,50	-1.0	1	22	2096	2117	AUAAAACCUUCAGCUAUCCAUC	AAUGGAUAGCUGAAAGUUUUAU	Cleavage
tae-miR7757-5p	Traes_5BL_7F0FD1538.3	PF00847,PF00931	KOG4658			GO:GO:0006355,GO:GO:0003700,GO:GO:0043531	AT3G46710.1	NB-ARC domain-containing disease resistance protein	LOC_Os05g40150.1	RGH2B, putative, expressed	1,50	-1.0	1	22	1379	1400	AUAAAACCUUCAGCUAUCCAUC	AAUGGAUAGCUGAAAGUUUUAU	Cleavage
tae-miR7757-5p	Traes_5BL_7F0FD1538.4	PF00931	KOG4658			GO:GO:0043531	AT3G46710.1	NB-ARC domain-containing disease resistance protein	LOC_Os05g40150.1	RGH2B, putative, expressed	1,50	-1.0	1	22	1379	1400	AUAAAACCUUCAGCUAUCCAUC	AAUGGAUAGCUGAAAGUUUUAU	Cleavage
tae-miR5086	Traes_5BL_83D9CB7C3.1										3,00	-1.0	1	21	497	517	ACAUUGGUGGAAGGCGUGGUA	AGUCAUGCCUUCCACCAAUGA	Cleavage
tae-miR5086	Traes_5BL_83D9CB7C3.2										3,00	-1.0	1	21	608	628	ACAUUGGUGGAAGGCGUGGUA	AGUCAUGCCUUCCACCAAUGA	Cleavage
tae-miR5086	Traes_5BL_83D9CB7C3.3										3,00	-1.0	1	21	473	493	ACAUUGGUGGAAGGCGUGGUA	AGUCAUGCCUUCCACCAAUGA	Cleavage
tae-miR5086	Traes_5BL_83D9CB7C3.4										3,00	-1.0	1	21	608	628	ACAUUGGUGGAAGGCGUGGUA	AGUCAUGCCUUCCACCAAUGA	Cleavage
tae-miR5086	Traes_5BL_83D9CB7C3.5										3,00	-1.0	1	21	497	517	ACAUUGGUGGAAGGCGUGGUA	AGUCAUGCCUUCCACCAAUGA	Cleavage
tae-miR156	Traes_5BL_96D5EA4EE.1	PF03110				GO:GO:0005634,GO:GO:0003677	AT2G42200.1	squamosa promoter binding protein-like 9	LOC_Os09g31438.1	OsSPL17 - SBP-box gene family member, expressed	1,00	-1.0	1	21	805	825	UGACAGAAGAGAGUGAGCACA	UGUGCUCUCUCUCUUCUGUCA	Cleavage
tae-miR156	Traes_5BL_96D5EA4EE.2	PF03110				GO:GO:0005634,GO:GO:0003677	AT2G42200.1	squamosa promoter binding protein-like 9	LOC_Os09g31438.1	OsSPL17 - SBP-box gene family member, expressed	1,00	-1.0	1	21	802	822	UGACAGAAGAGAGUGAGCACA	UGUGCUCUCUCUCUUCUGUCA	Cleavage
tae-miR156	Traes_5BL_96D5EA4EE.3								LOC_Os09g31438.1	OsSPL17 - SBP-box gene family member, expressed	1,00	-1.0	1	21	67	87	UGACAGAAGAGAGUGAGCACA	UGUGCUCUCUCUCUUCUGUCA	Cleavage
tae-miR156	Traes_5BL_96FDFC152.1	PF03110				GO:GO:0005634,GO:GO:0003677	AT1G76580.1	Squamosa promoter-binding protein-like (SBP domain) transcription factor family protein	LOC_Os09g32944.1	OsSPL18 - SBP-box gene family member, expressed	1,00	-1.0	1	21	508	528	UGACAGAAGAGAGUGAGCACA	UGUGCUCUCUCUCUUCUGUCA	Cleavage
tae-miR156	Traes_5BL_96FDFC152.2	PF03110				GO:GO:0005634,GO:GO:0003677	AT5G50570.1	Squamosa promoter-binding protein-like (SBP domain) transcription factor family protein	LOC_Os09g32944.1	OsSPL18 - SBP-box gene family member, expressed	1,00	-1.0	1	21	490	510	UGACAGAAGAGAGUGAGCACA	UGUGCUCUCUCUCUUCUGUCA	Cleavage
tae-miR5384-3p	Traes_5BL_AD734CCEF.1	PF12695	KOG1515	3.1.1.1			AT3G48690.1	alpha/beta-Hydrolases superfamily protein	LOC_Os09g28730.1	gibberellin receptor GID1L2, putative, expressed	2,50	-1.0	1	21	242	262	UGAGCGCGCCGCCGUCGAAUG	UCUUCCACGGCGGCGCGUUCA	Cleavage
tae-miR9664-3p	Traes_5BL_D27214492.1	PF00931				GO:GO:0043531	AT3G46530.1	NB-ARC domain-containing disease resistance protein	LOC_Os11g11550.1	NBS-LRR disease resistance protein, putative, expressed	2,50	-1.0	1	21	237	257	UUGCAGUCCUCGAUGUCGUAG	GCACGACAUCGAGGAUUGCAU	Cleavage
tae-miR9664-3p	Traes_5BL_D27214492.2	PF00931				GO:GO:0043531	AT3G46530.1	NB-ARC domain-containing disease resistance protein	LOC_Os11g11550.2	NBS-LRR disease resistance protein, putative, expressed	2,50	-1.0	1	21	237	257	UUGCAGUCCUCGAUGUCGUAG	GCACGACAUCGAGGAUUGCAU	Cleavage
tae-miR9664-3p	Traes_5BL_D27214492.3	PF00931				GO:GO:0043531	AT3G46530.1	NB-ARC domain-containing disease resistance protein	LOC_Os11g11550.2	NBS-LRR disease resistance protein, putative, expressed	2,50	-1.0	1	21	237	257	UUGCAGUCCUCGAUGUCGUAG	GCACGACAUCGAGGAUUGCAU	Cleavage
tae-miR9664-3p	Traes_5BL_D27214492.4	PF00931				GO:GO:0043531	AT3G46530.1	NB-ARC domain-containing disease resistance protein	LOC_Os11g11550.2	NBS-LRR disease resistance protein, putative, expressed	2,50	-1.0	1	21	237	257	UUGCAGUCCUCGAUGUCGUAG	GCACGACAUCGAGGAUUGCAU	Cleavage
tae-miR9664-3p	Traes_5BL_D27214492.5	PF00931				GO:GO:0043531	AT3G46530.1	NB-ARC domain-containing disease resistance protein	LOC_Os11g11550.1	NBS-LRR disease resistance protein, putative, expressed	2,50	-1.0	1	21	237	257	UUGCAGUCCUCGAUGUCGUAG	GCACGACAUCGAGGAUUGCAU	Cleavage
tae-miR7757-5p	Traes_5BL_DD4C9F446.1	PF00069	KOG1187	2.7.11.1		GO:GO:0006468,GO:GO:0005524,GO:GO:0004672	AT3G55950.1	CRINKLY4 related 3	LOC_Os05g40150.1	RGH2B, putative, expressed	1,50	-1.0	1	22	62	83	AUAAAACCUUCAGCUAUCCAUC	AAUGGAUAGCGGAAGGUUUUAU	Cleavage
tae-miR7757-5p	Traes_5BL_DD4C9F446.2	PF00069	KOG1187	2.7.11.1		GO:GO:0006468,GO:GO:0005524,GO:GO:0004672	AT3G55950.1	CRINKLY4 related 3	LOC_Os05g40150.1	RGH2B, putative, expressed	1,50	-1.0	1	22	62	83	AUAAAACCUUCAGCUAUCCAUC	AAUGGAUAGCGGAAGGUUUUAU	Cleavage
tae-miR7757-5p	Traes_5BL_DD4C9F446.4						AT3G46710.1	NB-ARC domain-containing disease resistance protein	LOC_Os05g40150.1	RGH2B, putative, expressed	1,50	-1.0	1	22	62	83	AUAAAACCUUCAGCUAUCCAUC	AAUGGAUAGCGGAAGGUUUUAU	Cleavage
tae-miR7757-5p	Traes_5BL_DD4C9F446.5						AT3G46710.1	NB-ARC domain-containing disease resistance protein	LOC_Os05g40150.1	RGH2B, putative, expressed	1,50	-1.0	1	22	62	83	AUAAAACCUUCAGCUAUCCAUC	AAUGGAUAGCGGAAGGUUUUAU	Cleavage

tae-miR7757-5p	Traes_5BL_DD4C9F446.6						AT3G46710.1	NB-ARC domain-containing disease resistance protein	LOC_Os05g40150.1	RGH2B, putative, expressed	1,50	-1.0	1	22	62	83	AUAAAACCUUCAGCUAUCCAUC	AAUGGAUAGCGGAAGGUUUUAU	Cleavage
tae-miR9661-5p	Traes_5BL_E0C2C7D72.1										3,00	-1.0	1	21	98	118	UGAAGUAGAGCAGGGACCUCA	UCGUCUCCCUGCUCUACUUCA	Cleavage
tae-miR9661-5p	Traes_5BL_E0C2C7D72.2										3,00	-1.0	1	21	98	118	UGAAGUAGAGCAGGGACCUCA	UCGUCUCCCUGCUCUACUUCA	Cleavage
tae-miR9661-5p	Traes_5BL_E0C2C7D72.3										3,00	-1.0	1	21	95	115	UGAAGUAGAGCAGGGACCUCA	UCGUCUCCCUGCUCUACUUCA	Cleavage
tae-miR9652-5p	Traes_5BL_FC199BC2D.1										2,50	-1.0	1	22	412	433	CCUGUUUGUCAUUAAGUUUCUU	AAGAAACUGAAUGACAAGCAGG	Cleavage
tae-miR1122b-3p	Traes_5BS_321AF7B69.2	PF14416,PF13839					AT5G20680.3	TRICHOME BIREFRINGENCE-LIKE 16	LOC_Os12g33194.1	expressed protein	2,50	-1.0	1	21	23	43	AGACUUAUAUGUAGGAACGGA	UCCAUUCCUAAAUAUAAGUCU	Translation
tae-miR1122a	Traes_5BS_321AF7B69.2	PF14416,PF13839					AT5G20680.3	TRICHOME BIREFRINGENCE-LIKE 16	LOC_Os12g33194.1	expressed protein	2,50	-1.0	1	20	63	82	UAGAUACAUCCGUAUCUAGA	ACUACAUACGGAUGUAUAUA	Cleavage
tae-miR7757-5p	Traes_5BS_3FFCC89AD.1	PF00931	KOG4658			GO:GO:0043531	AT3G07040.1	NB-ARC domain-containing disease resistance protein	LOC_Os12g37770.1	RGH1A, putative, expressed	2,50	-1.0	1	22	1337	1358	AUAAAACCUUCAGCUAUCCAUC	GGUGGAUUGCUGAAGGAUUUAU	Cleavage
tae-miR7757-5p	Traes_5BS_3FFCC89AD.2	PF00931	KOG4658			GO:GO:0043531	AT3G46730.1	NB-ARC domain-containing disease resistance protein	LOC_Os12g37770.1	RGH1A, putative, expressed	2,50	-1.0	1	22	1298	1319	AUAAAACCUUCAGCUAUCCAUC	GGUGGAUUGCUGAAGGAUUUAU	Cleavage
tae-miR1118	Traes_5BS_550BDEE86.2	PF00013	KOG2190			GO:GO:0003723	AT3G04610.1	RNA-binding KH domain-containing protein	LOC_Os12g40560.1	KH domain containing protein, putative, expressed	1,50	-1.0	1	23	1135	1157	CACUACAUUAUGGAAUGGAGGGA	UCCCUCCAUUCCACAAUGUAGUG	Translation
tae-miR5384-3p	Traes_5BS_90796C6CA.1	PF07762							LOC_Os01g56900.1	expressed protein	2,00	-1.0	1	21	461	481	UGAGCGCGCCGCCGUCGAAUG	UGUACGAUGGCGGCGCGCUCA	Cleavage
tae-miR5384-3p	Traes_5BS_90796C6CA.2	PF07762							LOC_Os01g56900.1	expressed protein	2,00	-1.0	1	21	34	54	UGAGCGCGCCGCCGUCGAAUG	UGUACGAUGGCGGCGCGCUCA	Cleavage
tae-miR5384-3p	Traes_5BS_90796C6CA.3	PF07762							LOC_Os01g56900.1	expressed protein	2,00	-1.0	1	21	392	412	UGAGCGCGCCGCCGUCGAAUG	UGUACGAUGGCGGCGCGCUCA	Cleavage
tae-miR164	Traes_5BS_9F043AE17.1	PF02365				GO:GO:0006355,GO:GO:0003677	AT1G56010.2	NAC domain containing protein 1	LOC_Os12g41680.1	No apical meristem protein, putative, expressed	1,00	-1.0	1	21	656	676	UGGAGAAGCAGGGCACGUGCA	AGCAAGUGCCCUGCUUCUCCA	Cleavage
tae-miR5384-3p	Traes_5BS_BA0400E10.1	PF14570,PF03552		2.4.2.24	K00770	GO:GO:0030244,GO:GO:0016760,GO:GO:0016020	AT1G02730.1	cellulose synthase-like D5	LOC_Os12g36890.1	CSLD4 - cellulose synthase-like family D, expressed	3,00	-1.0	1	21	1358	1378	UGAGCGCGCCGCCGUCGAAUG	CCGACGACGGCGGCGCGCUCC	Cleavage
tae-miR7757-5p	Traes_5BS_BCEA0112C.1	PF00931	KOG4658			GO:GO:0043531	AT3G07040.1	NB-ARC domain-containing disease resistance protein	LOC_Os12g37740.1	resistance protein LR10, putative, expressed	2,50	-1.0	1	22	1340	1361	AUAAAACCUUCAGCUAUCCAUC	GAUGGAUCGCGGAAGGUUUUAU	Cleavage
tae-miR7757-5p	Traes_5BS_BCEA0112C.2	PF00931	KOG4658			GO:GO:0043531	AT3G07040.1	NB-ARC domain-containing disease resistance protein	LOC_Os12g37740.1	resistance protein LR10, putative, expressed	2,50	-1.0	1	22	1340	1361	AUAAAACCUUCAGCUAUCCAUC	GAUGGAUCGCGGAAGGUUUUAU	Cleavage
tae-miR7757-5p	Traes_5BS_BCEA0112C.3	PF00931	KOG4658			GO:GO:0043531	AT3G07040.1	NB-ARC domain-containing disease resistance protein	LOC_Os12g37740.1	resistance protein LR10, putative, expressed	2,50	-1.0	1	22	1307	1328	AUAAAACCUUCAGCUAUCCAUC	GAUGGAUCGCGGAAGGUUUUAU	Cleavage
tae-miR7757-5p	Traes_5BS_BCEA0112C.4	PF00931				GO:GO:0043531	AT3G07040.1	NB-ARC domain-containing disease resistance protein	LOC_Os12g37740.1	resistance protein LR10, putative, expressed	2,50	-1.0	1	22	128	149	AUAAAACCUUCAGCUAUCCAUC	GAUGGAUCGCGGAAGGUUUUAU	Cleavage
tae-miR408	Traes_5BS_E10756355.1	PF05911	KOG0999				AT1G77580.2	Plant protein of unknown function (DUF869)	LOC_Os12g07160.2	plant protein of unknown function DUF869 domain containing protein, expressed	2,50	-1.0	1	21	2512	2532	CUGCACUGCCUCUUCCCUGGC	GCCAAGGAAGAGGCUGUGCAG	Cleavage
tae-miR7757-5p	Traes_5BS_F5AFB24CD.2	PF00931				GO:GO:0043531	AT3G46530.1	NB-ARC domain-containing disease resistance protein	LOC_Os10g03570.1	RGH1A, putative, expressed	1,50	-1.0	1	22	779	800	AUAAAACCUUCAGCUAUCCAUC	GGUGGAUAGCUGAAGGUCUUAU	Cleavage
tae-miR7757-5p	Traes_5BS_F5AFB24CD.3	PF00931				GO:GO:0043531	AT3G46530.1	NB-ARC domain-containing disease resistance protein	LOC_Os10g03570.1	RGH1A, putative, expressed	1,50	-1.0	1	22	779	800	AUAAAACCUUCAGCUAUCCAUC	GGUGGAUAGCUGAAGGUCUUAU	Cleavage
tae-miR7757-5p	Traes_5BS_F5AFB24CD.4	PF00931				GO:GO:0043531	AT5G43470.2	Disease resistance protein (CC-NBS-LRR class) family	LOC_Os10g03570.1	RGH1A, putative, expressed	1,50	-1.0	1	22	371	392	AUAAAACCUUCAGCUAUCCAUC	GGUGGAUAGCUGAAGGUCUUAU	Cleavage
tae-miR156	Traes_5DL_1272B4BAC.1	PF03110				GO:GO:0005634,GO:GO:0003677	AT5G50670.1	Squamosa promoter-binding protein-like (SBP domain) transcription factor family protein	LOC_Os09g32944.1	OsSPL18 - SBP-box gene family member, expressed	1,00	-1.0	1	21	505	525	UGACAGAAGAGAGUGAGCACA	UGUGCUCUCUCUCUUCUGUCA	Cleavage
tae-miR156	Traes_5DL_1272B4BAC.2	PF03110				GO:GO:0005634,GO:GO:0003677	AT5G50670.1	Squamosa promoter-binding protein-like (SBP domain) transcription factor family protein	LOC_Os09g32944.1	OsSPL18 - SBP-box gene family member, expressed	1,00	-1.0	1	21	487	507	UGACAGAAGAGAGUGAGCACA	UGUGCUCUCUCUCUUCUGUCA	Cleavage
tae-miR156	Traes_5DL_1272B4BAC.3								LOC_Os09g32944.1	OsSPL18 - SBP-box gene family member, expressed	1,00	-1.0	1	21	382	402	UGACAGAAGAGAGUGAGCACA	UGUGCUCUCUCUCUUCUGUCA	Cleavage
tae-miR7757-5p	Traes_5DL_1733FB4DA.1	PF00931,PF03106				GO:GO:0043531,GO:GO:0043565,GO:GO:0006355,GO:GO:0003700	AT3G46530.1	NB-ARC domain-containing disease resistance protein	LOC_Os05g40150.1	RGH2B, putative, expressed	1,50	-1.0	1	22	617	638	AUAAAACCUUCAGCUAUCCAUC	AAUGGAUAGCUGAAGGAUUUAU	Cleavage
tae-miR7757-5p	Traes_5DL_1733FB4DA.2	PF03106,PF00931				GO:GO:0043565,GO:GO:0006355,GO:GO:0003700,GO:GO:0043531	AT3G46530.1	NB-ARC domain-containing disease resistance protein	LOC_Os05g40150.1	RGH2B, putative, expressed	1,50	-1.0	1	22	617	638	AUAAAACCUUCAGCUAUCCAUC	AAUGGAUAGCUGAAGGAUUUAU	Cleavage
tae-miR7757-5p	Traes_5DL_1733FB4DA.3	PF03106,PF00931				GO:GO:0043565,GO:GO:0006355,GO:GO:0003700,GO:GO:0043531	AT3G46530.1	NB-ARC domain-containing disease resistance protein	LOC_Os05g40150.1	RGH2B, putative, expressed	1,50	-1.0	1	22	617	638	AUAAAACCUUCAGCUAUCCAUC	AAUGGAUAGCUGAAGGAUUUAU	Cleavage
tae-miR7757-5p	Traes_5DL_1733FB4DA.4	PF00931				GO:GO:0043531	AT3G46530.1	NB-ARC domain-containing disease resistance protein	LOC_Os05g40150.1	RGH2B, putative, expressed	1,50	-1.0	1	22	617	638	AUAAAACCUUCAGCUAUCCAUC	AAUGGAUAGCUGAAGGAUUUAU	Cleavage
tae-miR7757-5p	Traes_5DL_1733FB4DA.5	PF00931				GO:GO:0043531	AT3G46530.1	NB-ARC domain-containing disease resistance protein	LOC_Os05g40150.1	RGH2B, putative, expressed	1,50	-1.0	1	22	617	638	AUAAAACCUUCAGCUAUCCAUC	AAUGGAUAGCUGAAGGAUUUAU	Cleavage
tae-miR7757-5p	Traes_5DL_1733FB4DA.6	PF00931				GO:GO:0043531	AT3G46530.1	NB-ARC domain-containing disease resistance protein	LOC_Os05g40150.1	RGH2B, putative, expressed	1,50	-1.0	1	22	617	638	AUAAAACCUUCAGCUAUCCAUC	AAUGGAUAGCUGAAGGAUUUAU	Cleavage
tae-miR7757-5p	Traes_5DL_1733FB4DA.7	PF00931				GO:GO:0043531	AT3G46530.1	NB-ARC domain-containing disease resistance protein	LOC_Os11g45750.1	WRKY125, expressed	1,50	-1.0	1	22	617	638	AUAAAACCUUCAGCUAUCCAUC	AAUGGAUAGCUGAAGGAUUUAU	Cleavage
tae-miR7757-5p	Traes_5DL_1733FB4DA.8	PF00931				GO:GO:0043531	AT3G46530.1	NB-ARC domain-containing disease resistance protein	LOC_Os11g45750.2	WRKY125, expressed	1,50	-1.0	1	22	617	638	AUAAAACCUUCAGCUAUCCAUC	AAUGGAUAGCUGAAGGAUUUAU	Cleavage
tae-miR5062-5p	Traes_5DL_26B51AAF8.1	PF02171				GO:GO:0003676	AT1G48410.2	Stabilizer of iron transporter SufD / Polynucleotidyl transferase	LOC_Os03g58600.1	PAZ domain containing protein, putative, expressed	1,50	-1.0	1	23	43	65	UGAACCUUAGGGAACAGCCGCAU	ACCAGGCUGUUCCCUGAGGUUCA	Cleavage
tae-miR1127b-3p	Traes_5DL_4E67E0243.9	PF03828		2.7.7.19			AT3G51620.2	PAP/OAS1 substrate-binding domain superfamily	LOC_Os12g02260.1	nucleotidyltransferase, putative, expressed	1,50	-1.0	1	21	41	61	ACAAGUAUUUCUGGACGGAGG	CCUCCGUCCGGAAAUACUUGU	Cleavage
tae-miR9652-5p	Traes_5DL_714CCB5BC.1										2,50	-1.0	1	22	412	433	CCUGUUUGUCAUUAAGUUUCUU	AAGAAACUGAAUGACAAGCAGG	Cleavage
tae-miR9662a-3p	Traes_5DL_7643B2ADB.1	PF02536				GO:GO:0006355,GO:GO:0005739,GO:GO:0003690	AT5G64950.1	Mitochondrial transcription termination factor family protein	LOC_Os06g12100.1	mTERF family protein, expressed	2,50	-1.0	1	21	411	431	UUGAACAUCCCAGAGCCACCG	CCGUGGCUCUGUGAUGUUCAG	Translation
tae-miR9662b-3p	Traes_5DL_7643B2ADB.1	PF02536				GO:GO:0006355,GO:GO:0005739,GO:GO:0003690	AT5G64950.1	Mitochondrial transcription termination factor family protein	LOC_Os06g12100.1	mTERF family protein, expressed	2,50	-1.0	1	21	410	430	UGAACAUCCCAGAGCCACCGG	CCCGUGGCUCUGUGAUGUUCA	Cleavage
tae-miR5384-3p	Traes_5DL_78767F75C.1	PF00135	KOG1515	3.1.1.1			AT3G48690.1	alpha/beta-Hydrolases superfamily protein	LOC_Os09g28730.1	gibberellin receptor GID1L2, putative, expressed	2,50	-1.0	1	21	242	262	UGAGCGCGCCGCCGUCGAAUG	UCUUCCACGGCGGCGCGUUCA	Cleavage
tae-miR5049-3p	Traes_5DL_799647EA7.4										1,50	-1.0	1	21	65	85	AAUAUGGAUCGGAGGGAGUAC	GUACUCCCUCCGUUCCAUAUU	Cleavage
tae-miR9652-5p	Traes_5DL_8DE6C67FA.1										2,50	-1.0	1	22	412	433	CCUGUUUGUCAUUAAGUUUCUU	AAGAAACUGAAUGACAAGCAGG	Cleavage
tae-miR9652-5p	Traes_5DL_8DE6C67FA.2										2,50	-1.0	1	22	412	433	CCUGUUUGUCAUUAAGUUUCUU	AAGAAACUGAAUGACAAGCAGG	Cleavage
tae-miR9652-5p	Traes_5DL_8DE6C67FA.3										2,50	-1.0	1	22	415	436	CCUGUUUGUCAUUAAGUUUCUU	AAGAAACUGAAUGACAAGCAGG	Cleavage
tae-miR9652-5p	Traes_5DL_8DE6C67FA.4										2,50	-1.0	1	22	400	421	CCUGUUUGUCAUUAAGUUUCUU	AAGAAACUGAAUGACAAGCAGG	Cleavage
tae-miR9652-5p	Traes_5DL_8DE6C67FA.5										2,50	-1.0	1	22	400	421	CCUGUUUGUCAUUAAGUUUCUU	AAGAAACUGAAUGACAAGCAGG	Cleavage
tae-miR408	Traes_5DL_90BD9F306.1	PF02298				GO:GO:0009055	AT2G02850.1	plantacyanin	LOC_Os03g50160.1	plastocyanin-like domain containing protein, putative, expressed	0,00	-1.0	1	21	8	28	CUGCACUGCCUCUUCCCUGGC	CCCAGGGAAGAGGCAGUGCAG	Cleavage
tae-miR9666b-3p	Traes_5DL_9101D5AEC.1	PF05001		2.7.7.6		GO:GO:0006366,GO:GO:0005665,GO:GO:0003677	AT4G35800.1	RNA polymerase II large subunit	LOC_Os05g05860.1	retrotransposon protein, putative, unclassified, expressed	2,50	-1.0	1	22	1183	1204	CGGUUGGGCUGUAUGAUGGCGA	AGUCCAUCAUACAGCCCAACAA	Cleavage
tae-miR1130b-3p	Traes_5DL_A6EECBC04.1								LOC_Os03g32160.1	calmodulin binding protein, putative, expressed	1,50	-1.0	1	21	376	396	UCUUAUAUUAUGGGACGGAGG	AUUCCGUCCCAUAAUGUAAGA	Cleavage
tae-miR9652-5p	Traes_5DL_B3713A4D1.1										2,50	-1.0	1	22	940	961	CCUGUUUGUCAUUAAGUUUCUU	AAGAAACUGAAUGACAAGCAGG	Cleavage
tae-miR5086	Traes_5DL_CECECF832.1										3,00	-1.0	1	21	461	481	ACAUUGGUGGAAGGCGUGGUA	AGUCAUGCCUUCCACCAAUGA	Cleavage
tae-miR5086	Traes_5DL_CECECF832.2										3,00	-1.0	1	21	485	505	ACAUUGGUGGAAGGCGUGGUA	AGUCAUGCCUUCCACCAAUGA	Cleavage
tae-miR5086	Traes_5DL_CECECF832.3										3,00	-1.0	1	21	101	121	ACAUUGGUGGAAGGCGUGGUA	AGUCAUGCCUUCCACCAAUGA	Cleavage
tae-miR5086	Traes_5DL_CECECF832.4										3,00	-1.0	1	21	485	505	ACAUUGGUGGAAGGCGUGGUA	AGUCAUGCCUUCCACCAAUGA	Cleavage
tae-miR5086	Traes_5DL_CECECF832.5										3,00	-1.0	1	21	101	121	ACAUUGGUGGAAGGCGUGGUA	AGUCAUGCCUUCCACCAAUGA	Cleavage
tae-miR5062-5p	Traes_5DL_F5D3E4763.1	PF02171				GO:GO:0003676	AT1G48410.3	Stabilizer of iron transporter SufD / Polynucleotidyl transferase	LOC_Os03g58600.1	PAZ domain containing protein, putative, expressed	2,50	-1.0	1	23	43	65	UGAACCUUAGGGAACAGCCGCAU	ACCAGGUUGUUCCCUGAGGUUCA	Cleavage
tae-miR7757-5p	Traes_5DS_0479C6E0F.1	PF00931				GO:GO:0043531	AT3G07040.1	NB-ARC domain-containing disease resistance protein	LOC_Os12g06920.1	NBS-LRR disease resistance protein, putative, expressed	2,50	-1.0	1	22	383	404	AUAAAACCUUCAGCUAUCCAUC	GAUGGAUCGCUGAAGGUUUGAU	Cleavage
tae-miR9662a-3p	Traes_5DS_094A03C13.1	PF02536				GO:GO:0006355,GO:GO:0005739,GO:GO:0003690	AT5G07900.1	Mitochondrial transcription termination factor family protein	LOC_Os06g12100.1	mTERF family protein, expressed	1,00	-1.0	1	21	549	569	UUGAACAUCCCAGAGCCACCG	CCGUGGCUCUGGGAUGUUCAG	Cleavage
tae-miR9662b-3p	Traes_5DS_094A03C13.1	PF02536				GO:GO:0006355,GO:GO:0005739,GO:GO:0003690	AT5G07900.1	Mitochondrial transcription termination factor family protein	LOC_Os06g12100.1	mTERF family protein, expressed	1,00	-1.0	1	21	548	568	UGAACAUCCCAGAGCCACCGG	CCCGUGGCUCUGGGAUGUUCA	Cleavage
tae-miR9662a-3p	Traes_5DS_094A03C13.2	PF02536				GO:GO:0006355,GO:GO:0005739,GO:GO:0003690	AT5G07900.1	Mitochondrial transcription termination factor family protein	LOC_Os06g12100.1	mTERF family protein, expressed	1,00	-1.0	1	21	243	263	UUGAACAUCCCAGAGCCACCG	CCGUGGCUCUGGGAUGUUCAG	Cleavage
tae-miR9662b-3p	Traes_5DS_094A03C13.2	PF02536				GO:GO:0006355,GO:GO:0005739,GO:GO:0003690	AT5G07900.1	Mitochondrial transcription termination factor family protein	LOC_Os06g12100.1	mTERF family protein, expressed	1,00	-1.0	1	21	242	262	UGAACAUCCCAGAGCCACCGG	CCCGUGGCUCUGGGAUGUUCA	Cleavage
tae-miR5384-3p	Traes_5DS_149714893.1	PF04535					AT5G44550.1	Uncharacterised protein family (UPF0497)	LOC_Os12g41690.1	membrane associated DUF588 domain containing protein, putative, expressed	2,50	-1.0	1	21	158	178	UGAGCGCGCCGCCGUCGAAUG	CGUUCGUCGGCGUCGCGCUCA	Cleavage
tae-miR5384-3p	Traes_5DS_149714893.2	PF04535					AT5G44550.1	Uncharacterised protein family (UPF0497)	LOC_Os12g41690.1	membrane associated DUF588 domain containing protein, putative, expressed	2,50	-1.0	1	21	152	172	UGAGCGCGCCGCCGUCGAAUG	CGUUCGUCGGCGUCGCGCUCA	Cleavage
tae-miR164	Traes_5DS_511F35A45.1						AT1G56010.2	NAC domain containing protein 1	LOC_Os12g41680.1	No apical meristem protein, putative, expressed	1,00	-1.0	1	21	209	229	UGGAGAAGCAGGGCACGUGCA	AGCAAGUGCCCUGCUUCUCCA	Cleavage
tae-miR7757-5p	Traes_5DS_8DB03B7DA.1	PF00931	KOG4658			GO:GO:0043531	AT3G07040.1	NB-ARC domain-containing disease resistance protein	LOC_Os12g37740.1	resistance protein LR10, putative, expressed	2,50	-1.0	1	22	1349	1370	AUAAAACCUUCAGCUAUCCAUC	GAUGGAUCGCGGAAGGUUUUAU	Cleavage
tae-miR7757-5p	Traes_5DS_8DB03B7DA.2	PF00931				GO:GO:0043531	AT3G07040.1	NB-ARC domain-containing disease resistance protein	LOC_Os12g37740.1	resistance protein LR10, putative, expressed	2,50	-1.0	1	22	128	149	AUAAAACCUUCAGCUAUCCAUC	GAUGGAUCGCGGAAGGUUUUAU	Cleavage
tae-miR7757-5p	Traes_5DS_9DFE4D217.1	PF00931				GO:GO:0043531	AT1G53350.1	Disease resistance protein (CC-NBS-LRR class) family	LOC_Os10g22484.1	RGH2B, putative, expressed	2,50	-1.0	1	22	185	206	AUAAAACCUUCAGCUAUCCAUC	AAUGGGUAGCUGAAGGUUGUAU	Cleavage
tae-miR7757-5p	Traes_5DS_9DFE4D217.2	PF00931				GO:GO:0043531	AT1G53350.1	Disease resistance protein (CC-NBS-LRR class) family	LOC_Os10g22484.1	RGH2B, putative, expressed	2,50	-1.0	1	22	185	206	AUAAAACCUUCAGCUAUCCAUC	AAUGGGUAGCUGAAGGUUGUAU	Cleavage
tae-miR7757-5p	Traes_5DS_9DFE4D217.3	PF00931				GO:GO:0043531	AT1G53350.1	Disease resistance protein (CC-NBS-LRR class) family	LOC_Os10g22484.1	RGH2B, putative, expressed	2,50	-1.0	1	22	185	206	AUAAAACCUUCAGCUAUCCAUC	AAUGGGUAGCUGAAGGUUGUAU	Cleavage
tae-miR9662a-3p	Traes_5DS_9F4A5AE9F.1	PF02536				GO:GO:0006355,GO:GO:0005739,GO:GO:0003690	AT5G07900.1	Mitochondrial transcription termination factor family protein	LOC_Os06g12100.1	mTERF family protein, expressed	2,00	-1.0	1	21	441	461	UUGAACAUCCCAGAGCCACCG	UCGUGGCGCUGGGAUGUUCAG	Cleavage
tae-miR9662b-3p	Traes_5DS_9F4A5AE9F.1	PF02536				GO:GO:0006355,GO:GO:0005739,GO:GO:0003690	AT5G07900.1	Mitochondrial transcription termination factor family protein	LOC_Os06g12100.1	mTERF family protein, expressed	2,50	-1.0	1	21	440	460	UGAACAUCCCAGAGCCACCGG	CUCGUGGCGCUGGGAUGUUCA	Cleavage
tae-miR9662a-3p	Traes_5DS_9F4A5AE9F.2	PF02536				GO:GO:0006355,GO:GO:0005739,GO:GO:0003690	AT5G07900.1	Mitochondrial transcription termination factor family protein	LOC_Os06g12100.1	mTERF family protein, expressed	2,00	-1.0	1	21	66	86	UUGAACAUCCCAGAGCCACCG	UCGUGGCGCUGGGAUGUUCAG	Cleavage
tae-miR9662b-3p	Traes_5DS_9F4A5AE9F.2	PF02536				GO:GO:0006355,GO:GO:0005739,GO:GO:0003690	AT5G07900.1	Mitochondrial transcription termination factor family protein	LOC_Os06g12100.1	mTERF family protein, expressed	2,50	-1.0	1	21	65	85	UGAACAUCCCAGAGCCACCGG	CUCGUGGCGCUGGGAUGUUCA	Cleavage
tae-miR1122b-3p	Traes_5DS_9F8FFD461.1	PF00792,PF00613		2.7.1.137		GO:GO:0048015,GO:GO:0046854	AT1G60490.1	vacuolar protein sorting 34	LOC_Os05g08810.2	phosphatidylinositol 3-kinase, root isoform, putative, expressed	1,50	-1.0	1	21	1777	1797	AGACUUAUAUGUAGGAACGGA	UCCGUUCCUAAAUAUAAGUCU	Translation
tae-miR1122b-3p	Traes_5DS_9F8FFD461.2	PF00792,PF00613		2.7.1.137		GO:GO:0048015,GO:GO:0046854	AT1G60490.1	vacuolar protein sorting 34	LOC_Os05g08810.2	phosphatidylinositol 3-kinase, root isoform, putative, expressed	1,50	-1.0	1	21	1882	1902	AGACUUAUAUGUAGGAACGGA	UCCGUUCCUAAAUAUAAGUCU	Translation
tae-miR1122b-3p	Traes_5DS_9F8FFD461.3	PF00792,PF00613		2.7.1.137		GO:GO:0048015,GO:GO:0046854	AT1G60490.1	vacuolar protein sorting 34	LOC_Os05g08810.2	phosphatidylinositol 3-kinase, root isoform, putative, expressed	1,50	-1.0	1	21	1801	1821	AGACUUAUAUGUAGGAACGGA	UCCGUUCCUAAAUAUAAGUCU	Translation
tae-miR1122b-3p	Traes_5DS_A6AE32DCD.1	PF03483	KOG2472	6.1.1.20		GO:GO:0004826,GO:GO:0003723	AT1G72550.1	tRNA synthetase beta subunit family protein	LOC_Os05g48510.1	phenylalanyl-tRNA synthetase beta chain, putative, expressed	1,50	-1.0	1	21	1065	1085	AGACUUAUAUGUAGGAACGGA	UCCGUUCCUAAAUAUAAGUCU	Translation
tae-miR1122b-3p	Traes_5DS_A6AE32DCD.2	PF03484,PF03483		6.1.1.20		GO:GO:0006432,GO:GO:0005524,GO:GO:0003723,GO:GO:0000287,GO:GO:0004826	AT1G72550.2	tRNA synthetase beta subunit family protein	LOC_Os05g48510.1	phenylalanyl-tRNA synthetase beta chain, putative, expressed	1,50	-1.0	1	21	1146	1166	AGACUUAUAUGUAGGAACGGA	UCCGUUCCUAAAUAUAAGUCU	Translation
tae-miR1122b-3p	Traes_5DS_A6AE32DCD.3	PF03484,PF03483		6.1.1.20		GO:GO:0006432,GO:GO:0005524,GO:GO:0003723,GO:GO:0000287,GO:GO:0004826	AT1G72550.2	tRNA synthetase beta subunit family protein	LOC_Os05g48510.1	phenylalanyl-tRNA synthetase beta chain, putative, expressed	1,50	-1.0	1	21	1146	1166	AGACUUAUAUGUAGGAACGGA	UCCGUUCCUAAAUAUAAGUCU	Translation
tae-miR9662a-3p	Traes_5DS_C87840EBA.2						AT1G79220.1	Mitochondrial transcription termination factor family protein	LOC_Os06g12100.1	mTERF family protein, expressed	2,00	-1.0	1	21	429	449	UUGAACAUCCCAGAGCCACCG	CCGUGGUUCUGGGAUGUUCAG	Cleavage
tae-miR9662b-3p	Traes_5DS_C87840EBA.2						AT1G79220.1	Mitochondrial transcription termination factor family protein	LOC_Os06g12100.1	mTERF family protein, expressed	2,00	-1.0	1	21	428	448	UGAACAUCCCAGAGCCACCGG	CCCGUGGUUCUGGGAUGUUCA	Cleavage
tae-miR1128	Traes_5DS_D165422B3.1	PF00503	KOG0082			GO:GO:0031683,GO:GO:0019001,GO:GO:0007186,GO:GO:0004871,GO:GO:0003924	AT2G23460.1	extra-large G-protein 1	LOC_Os12g40190.1	G-protein alpha subunit, putative, expressed	3,00	-1.0	1	21	59	80	UACUACUCCCUCCG-UCCGAAA	UUUAGGAACGGAGGGAGUAGUA	Cleavage
tae-miR1118	Traes_5DS_F8D323D4D.2	PF00013	KOG2190			GO:GO:0003723	AT3G04610.1	RNA-binding KH domain-containing protein	LOC_Os12g40560.1	KH domain containing protein, putative, expressed	1,50	-1.0	1	23	1329	1351	CACUACAUUAUGGAAUGGAGGGA	CUCCUCCAUUCCACAAUGUAGUG	Translation
tae-miR160	Traes_6AL_232AF0B76.1						AT4G30080.1	auxin response factor 16	LOC_Os02g41800.1	auxin response factor, putative, expressed	0,00	-1.0	1	21	195	215	UGCCUGGCUCCCUGUAUGCCA	AGGCAUACAGGGAGCCAGGCA	Cleavage
tae-miR1121	Traes_6AL_23B31796F.2	PF00230	KOG0223			GO:GO:0016020,GO:GO:0006810,GO:GO:0005215	AT3G53420.1	plasma membrane intrinsic protein 2A	LOC_Os02g41860.3	aquaporin protein, putative, expressed	2,00	-1.0	1	22	846	867	AGUAGUGAUCUAAACGCUCUUA	UAAAAGUGUUUAGAUCACUACU	Cleavage
tae-miR1121	Traes_6AL_23B31796F.3	PF00230	KOG0223			GO:GO:0016020,GO:GO:0006810,GO:GO:0005215	AT3G53420.1	plasma membrane intrinsic protein 2A	LOC_Os02g41860.3	aquaporin protein, putative, expressed	2,00	-1.0	1	22	760	781	AGUAGUGAUCUAAACGCUCUUA	UAAAAGUGUUUAGAUCACUACU	Cleavage
tae-miR1121	Traes_6AL_23B31796F.5	PF00230	KOG0223			GO:GO:0016020,GO:GO:0006810,GO:GO:0005215	AT3G53420.1	plasma membrane intrinsic protein 2A	LOC_Os02g41860.3	aquaporin protein, putative, expressed	2,00	-1.0	1	22	742	763	AGUAGUGAUCUAAACGCUCUUA	UAAAAGUGUUUAGAUCACUACU	Cleavage
tae-miR5049-3p	Traes_6AL_36AE1B4CF.4	PF03031		3.1.3.16			AT4G21670.1	C-terminal domain phosphatase-like 1	LOC_Os02g42600.1	double-stranded RNA binding motif containing protein, expressed	1,50	-1.0	1	21	1787	1807	AAUAUGGAUCGGAGGGAGUAC	AUACUCCCUCCGACCCAUAUU	Cleavage
tae-miR5049-3p	Traes_6AL_36AE1B4CF1.4	PF03031		3.1.3.16			AT4G21670.1	C-terminal domain phosphatase-like 1	LOC_Os02g42600.1	double-stranded RNA binding motif containing protein, expressed	1,50	-1.0	1	21	1787	1807	AAUAUGGAUCGGAGGGAGUAC	AUACUCCCUCCGACCCAUAUU	Cleavage
tae-miR5049-3p	Traes_6AL_6AD463C67.3			3.6.4.12			AT2G01440.1	DEAD/DEAH box RNA helicase family protein			2,50	-1.0	1	21	15	35	AAUAUGGAUCGGAGGGAGUAC	GUACUCCCUUCCGAUCCAUAAU	Cleavage
tae-miR1121	Traes_6AL_7E70268F1.3	PF00719		3.6.1.1		GO:GO:0006796,GO:GO:0005737,GO:GO:0004427,GO:GO:0000287	AT2G46860.1	pyrophosphorylase 3	LOC_Os02g47600.1	soluble inorganic pyrophosphatase, putative, expressed	0,00	-1.0	1	22	497	518	AGUAGUGAUCUAAACGCUCUUA	UAAGAGCGUUUAGAUCACUACU	Cleavage
tae-miR1122b-3p	Traes_6AL_94CA6F0D8.5	PF00067				GO:GO:0055114,GO:GO:0020037,GO:GO:0016705,GO:GO:0005506	AT1G31800.1	cytochrome P450, family 97, subfamily A, polypeptide 3	LOC_Os02g57290.2	cytochrome P450, putative, expressed	2,50	-1.0	1	21	1030	1050	AGACUUAUAUGUAGGAACGGA	UCCAUUCCUAAAUAUAAGUCU	Translation
tae-miR1122a	Traes_6AL_94CA6F0D8.5	PF00067				GO:GO:0055114,GO:GO:0020037,GO:GO:0016705,GO:GO:0005506	AT1G31800.1	cytochrome P450, family 97, subfamily A, polypeptide 3	LOC_Os02g57290.2	cytochrome P450, putative, expressed	2,50	-1.0	1	20	1071	1090	UAGAUACAUCCGUAUCUAGA	ACUACAUACGGAUGUAUAUA	Cleavage
tae-miR9662a-3p	Traes_6AS_0A204AEC9.1	PF02536	KOG1267		K15032	GO:GO:0006355,GO:GO:0005739,GO:GO:0003690	AT5G07900.1	Mitochondrial transcription termination factor family protein	LOC_Os06g12100.1	mTERF family protein, expressed	2,00	-1.0	1	21	681	701	UUGAACAUCCCAGAGCCACCG	CCGUGCCUCUGGGAUGUUCAG	Cleavage
tae-miR9662b-3p	Traes_6AS_0A204AEC9.1	PF02536	KOG1267		K15032	GO:GO:0006355,GO:GO:0005739,GO:GO:0003690	AT5G07900.1	Mitochondrial transcription termination factor family protein	LOC_Os06g12100.1	mTERF family protein, expressed	2,00	-1.0	1	21	680	700	UGAACAUCCCAGAGCCACCGG	CCCGUGCCUCUGGGAUGUUCA	Cleavage
tae-miR9662a-3p	Traes_6AS_0A204AEC9.2	PF02536	KOG1267		K15032	GO:GO:0006355,GO:GO:0005739,GO:GO:0003690	AT5G07900.1	Mitochondrial transcription termination factor family protein	LOC_Os06g12100.1	mTERF family protein, expressed	2,00	-1.0	1	21	681	701	UUGAACAUCCCAGAGCCACCG	CCGUGCCUCUGGGAUGUUCAG	Cleavage
tae-miR9662b-3p	Traes_6AS_0A204AEC9.2	PF02536	KOG1267		K15032	GO:GO:0006355,GO:GO:0005739,GO:GO:0003690	AT5G07900.1	Mitochondrial transcription termination factor family protein	LOC_Os06g12100.1	mTERF family protein, expressed	2,00	-1.0	1	21	680	700	UGAACAUCCCAGAGCCACCGG	CCCGUGCCUCUGGGAUGUUCA	Cleavage
tae-miR9662a-3p	Traes_6AS_1496A7825.1	PF02536				GO:GO:0006355,GO:GO:0005739,GO:GO:0003690	AT5G07900.1	Mitochondrial transcription termination factor family protein	LOC_Os06g12100.1	mTERF family protein, expressed	1,50	-1.0	1	21	549	569	UUGAACAUCCCAGAGCCACCG	CCGUGGCUCGGGGAUGUUCAA	Cleavage
tae-miR9662b-3p	Traes_6AS_1496A7825.1	PF02536				GO:GO:0006355,GO:GO:0005739,GO:GO:0003690	AT5G07900.1	Mitochondrial transcription termination factor family protein	LOC_Os06g12100.1	mTERF family protein, expressed	2,50	-1.0	1	21	548	568	UGAACAUCCCAGAGCCACCGG	CCCGUGGCUCGGGGAUGUUCA	Translation
tae-miR9662a-3p	Traes_6AS_1496A7825.2	PF02536				GO:GO:0006355,GO:GO:0005739,GO:GO:0003690	AT5G07900.1	Mitochondrial transcription termination factor family protein	LOC_Os06g12100.1	mTERF family protein, expressed	1,50	-1.0	1	21	132	152	UUGAACAUCCCAGAGCCACCG	CCGUGGCUCGGGGAUGUUCAA	Cleavage
tae-miR9662b-3p	Traes_6AS_1496A7825.2	PF02536				GO:GO:0006355,GO:GO:0005739,GO:GO:0003690	AT5G07900.1	Mitochondrial transcription termination factor family protein	LOC_Os06g12100.1	mTERF family protein, expressed	2,50	-1.0	1	21	131	151	UGAACAUCCCAGAGCCACCGG	CCCGUGGCUCGGGGAUGUUCA	Translation
tae-miR9662a-3p	Traes_6AS_15C1E6F18.1	PF02536				GO:GO:0006355,GO:GO:0005739,GO:GO:0003690	AT1G21150.1	Mitochondrial transcription termination factor family protein	LOC_Os06g12100.1	mTERF family protein, expressed	2,50	-1.0	1	21	33	53	UUGAACAUCCCAGAGCCACCG	CUGUGGCUCUGGAAUGUUCAG	Cleavage
tae-miR9662b-3p	Traes_6AS_15C1E6F18.1	PF02536				GO:GO:0006355,GO:GO:0005739,GO:GO:0003690	AT1G21150.1	Mitochondrial transcription termination factor family protein	LOC_Os06g12100.1	mTERF family protein, expressed	2,50	-1.0	1	21	32	52	UGAACAUCCCAGAGCCACCGG	CCUGUGGCUCUGGAAUGUUCA	Cleavage
tae-miR9662a-3p	Traes_6AS_15C1E6F18.2	PF02536				GO:GO:0006355,GO:GO:0005739,GO:GO:0003690	AT1G21150.1	Mitochondrial transcription termination factor family protein	LOC_Os06g12100.1	mTERF family protein, expressed	2,50	-1.0	1	21	33	53	UUGAACAUCCCAGAGCCACCG	CUGUGGCUCUGGAAUGUUCAG	Cleavage
tae-miR9662b-3p	Traes_6AS_15C1E6F18.2	PF02536				GO:GO:0006355,GO:GO:0005739,GO:GO:0003690	AT1G21150.1	Mitochondrial transcription termination factor family protein	LOC_Os06g12100.1	mTERF family protein, expressed	2,50	-1.0	1	21	32	52	UGAACAUCCCAGAGCCACCGG	CCUGUGGCUCUGGAAUGUUCA	Cleavage
tae-miR9662a-3p	Traes_6AS_2BDC1663A.1	PF02536	KOG1267		K15032	GO:GO:0006355,GO:GO:0005739,GO:GO:0003690	AT5G07900.1	Mitochondrial transcription termination factor family protein	LOC_Os06g12100.1	mTERF family protein, expressed	1,00	-1.0	1	21	681	701	UUGAACAUCCCAGAGCCACCG	CCGUGGCUCUGGGAUGUUCAG	Cleavage
tae-miR9662b-3p	Traes_6AS_2BDC1663A.1	PF02536	KOG1267		K15032	GO:GO:0006355,GO:GO:0005739,GO:GO:0003690	AT5G07900.1	Mitochondrial transcription termination factor family protein	LOC_Os06g12100.1	mTERF family protein, expressed	1,00	-1.0	1	21	680	700	UGAACAUCCCAGAGCCACCGG	CCCGUGGCUCUGGGAUGUUCA	Cleavage
tae-miR156	Traes_6AS_32A35158A.1	PF03110				GO:GO:0005634,GO:GO:0003677	AT5G43270.2	squamosa promoter binding protein-like 2	LOC_Os06g45310.1	OsSPL11 - SBP-box gene family member, expressed	1,00	-1.0	1	21	736	756	UGACAGAAGAGAGUGAGCACA	CGUGCUCUCUCUCUUCUGUCA	Cleavage
tae-miR156	Traes_6AS_32A35158A.2								LOC_Os06g45310.1	OsSPL11 - SBP-box gene family member, expressed	1,00	-1.0	1	21	292	312	UGACAGAAGAGAGUGAGCACA	CGUGCUCUCUCUCUUCUGUCA	Cleavage
tae-miR156	Traes_6AS_32A35158A.3								LOC_Os06g45310.1	OsSPL11 - SBP-box gene family member, expressed	1,00	-1.0	1	21	64	84	UGACAGAAGAGAGUGAGCACA	CGUGCUCUCUCUCUUCUGUCA	Cleavage
tae-miR9662a-3p	Traes_6AS_3641B9C871.1	PF02536	KOG1267		K15032	GO:GO:0006355,GO:GO:0005739,GO:GO:0003690	AT5G07900.1	Mitochondrial transcription termination factor family protein	LOC_Os06g12100.1	mTERF family protein, expressed	1,00	-1.0	1	21	687	707	UUGAACAUCCCAGAGCCACCG	CCGUGGCUCUGGGAUGUUCAG	Cleavage
tae-miR9662b-3p	Traes_6AS_3641B9C871.1	PF02536	KOG1267		K15032	GO:GO:0006355,GO:GO:0005739,GO:GO:0003690	AT5G07900.1	Mitochondrial transcription termination factor family protein	LOC_Os06g12100.1	mTERF family protein, expressed	1,00	-1.0	1	21	686	706	UGAACAUCCCAGAGCCACCGG	CCCGUGGCUCUGGGAUGUUCA	Cleavage
tae-miR9662a-3p	Traes_6AS_3641B9C871.2	PF02536	KOG1267		K15032	GO:GO:0006355,GO:GO:0005739,GO:GO:0003690	AT5G07900.1	Mitochondrial transcription termination factor family protein	LOC_Os06g12100.1	mTERF family protein, expressed	1,00	-1.0	1	21	687	707	UUGAACAUCCCAGAGCCACCG	CCGUGGCUCUGGGAUGUUCAG	Cleavage
tae-miR9662b-3p	Traes_6AS_3641B9C871.2	PF02536	KOG1267		K15032	GO:GO:0006355,GO:GO:0005739,GO:GO:0003690	AT5G07900.1	Mitochondrial transcription termination factor family protein	LOC_Os06g12100.1	mTERF family protein, expressed	1,00	-1.0	1	21	686	706	UGAACAUCCCAGAGCCACCGG	CCCGUGGCUCUGGGAUGUUCA	Cleavage
tae-miR9662a-3p	Traes_6AS_36EAEADDD.1	PF02536				GO:GO:0006355,GO:GO:0005739,GO:GO:0003690	AT1G21150.1	Mitochondrial transcription termination factor family protein	LOC_Os06g12100.1	mTERF family protein, expressed	2,00	-1.0	1	21	33	53	UUGAACAUCCCAGAGCCACCG	CUGUGGUUCUGGGAUGUUCAG	Cleavage
tae-miR9662b-3p	Traes_6AS_36EAEADDD.1	PF02536				GO:GO:0006355,GO:GO:0005739,GO:GO:0003690	AT1G21150.1	Mitochondrial transcription termination factor family protein	LOC_Os06g12100.1	mTERF family protein, expressed	2,00	-1.0	1	21	32	52	UGAACAUCCCAGAGCCACCGG	CCUGUGGUUCUGGGAUGUUCA	Cleavage
tae-miR9662a-3p	Traes_6AS_36EAEADDD.2								LOC_Os06g12100.1	mTERF family protein, expressed	2,00	-1.0	1	21	33	53	UUGAACAUCCCAGAGCCACCG	CUGUGGUUCUGGGAUGUUCAG	Cleavage
tae-miR9662b-3p	Traes_6AS_36EAEADDD.2								LOC_Os06g12100.1	mTERF family protein, expressed	2,00	-1.0	1	21	32	52	UGAACAUCCCAGAGCCACCGG	CCUGUGGUUCUGGGAUGUUCA	Cleavage
tae-miR9662a-3p	Traes_6AS_36EAEADDD.3	PF02536				GO:GO:0006355,GO:GO:0005739,GO:GO:0003690	AT1G21150.1	Mitochondrial transcription termination factor family protein	LOC_Os06g12100.1	mTERF family protein, expressed	2,00	-1.0	1	21	33	53	UUGAACAUCCCAGAGCCACCG	CUGUGGUUCUGGGAUGUUCAG	Cleavage
tae-miR9662b-3p	Traes_6AS_36EAEADDD.3	PF02536				GO:GO:0006355,GO:GO:0005739,GO:GO:0003690	AT1G21150.1	Mitochondrial transcription termination factor family protein	LOC_Os06g12100.1	mTERF family protein, expressed	2,00	-1.0	1	21	32	52	UGAACAUCCCAGAGCCACCGG	CCUGUGGUUCUGGGAUGUUCA	Cleavage
tae-miR9662a-3p	Traes_6AS_48B701BB41.1	PF02536				GO:GO:0006355,GO:GO:0005739,GO:GO:0003690	AT1G21150.1	Mitochondrial transcription termination factor family protein	LOC_Os06g12100.1	mTERF family protein, expressed	1,00	-1.0	1	21	33	53	UUGAACAUCCCAGAGCCACCG	ACGUGGCUCUGGGAUGUUCAG	Cleavage
tae-miR9662b-3p	Traes_6AS_48B701BB41.1	PF02536				GO:GO:0006355,GO:GO:0005739,GO:GO:0003690	AT1G21150.1	Mitochondrial transcription termination factor family protein	LOC_Os06g12100.1	mTERF family protein, expressed	1,00	-1.0	1	21	32	52	UGAACAUCCCAGAGCCACCGG	CACGUGGCUCUGGGAUGUUCA	Cleavage
tae-miR9662a-3p	Traes_6AS_48B701BB41.2	PF02536				GO:GO:0006355,GO:GO:0005739,GO:GO:0003690	AT1G21150.1	Mitochondrial transcription termination factor family protein	LOC_Os06g12100.1	mTERF family protein, expressed	1,00	-1.0	1	21	33	53	UUGAACAUCCCAGAGCCACCG	ACGUGGCUCUGGGAUGUUCAG	Cleavage
tae-miR9662b-3p	Traes_6AS_48B701BB41.2	PF02536				GO:GO:0006355,GO:GO:0005739,GO:GO:0003690	AT1G21150.1	Mitochondrial transcription termination factor family protein	LOC_Os06g12100.1	mTERF family protein, expressed	1,00	-1.0	1	21	32	52	UGAACAUCCCAGAGCCACCGG	CACGUGGCUCUGGGAUGUUCA	Cleavage
tae-miR9662a-3p	Traes_6AS_48B701BB41.3	PF02536				GO:GO:0006355,GO:GO:0005739,GO:GO:0003690	AT1G21150.1	Mitochondrial transcription termination factor family protein	LOC_Os06g12100.1	mTERF family protein, expressed	1,00	-1.0	1	21	33	53	UUGAACAUCCCAGAGCCACCG	ACGUGGCUCUGGGAUGUUCAG	Cleavage
tae-miR9662b-3p	Traes_6AS_48B701BB41.3	PF02536				GO:GO:0006355,GO:GO:0005739,GO:GO:0003690	AT1G21150.1	Mitochondrial transcription termination factor family protein	LOC_Os06g12100.1	mTERF family protein, expressed	1,00	-1.0	1	21	32	52	UGAACAUCCCAGAGCCACCGG	CACGUGGCUCUGGGAUGUUCA	Cleavage
tae-miR9662a-3p	Traes_6AS_48B701BB41.4	PF02536				GO:GO:0006355,GO:GO:0005739,GO:GO:0003690	AT1G21150.1	Mitochondrial transcription termination factor family protein	LOC_Os06g12100.1	mTERF family protein, expressed	1,00	-1.0	1	21	33	53	UUGAACAUCCCAGAGCCACCG	ACGUGGCUCUGGGAUGUUCAG	Cleavage
tae-miR9662b-3p	Traes_6AS_48B701BB41.4	PF02536				GO:GO:0006355,GO:GO:0005739,GO:GO:0003690	AT1G21150.1	Mitochondrial transcription termination factor family protein	LOC_Os06g12100.1	mTERF family protein, expressed	1,00	-1.0	1	21	32	52	UGAACAUCCCAGAGCCACCGG	CACGUGGCUCUGGGAUGUUCA	Cleavage
tae-miR9662a-3p	Traes_6AS_48B701BB41.5	PF02536				GO:GO:0006355,GO:GO:0005739,GO:GO:0003690	AT1G21150.1	Mitochondrial transcription termination factor family protein	LOC_Os06g12100.1	mTERF family protein, expressed	1,00	-1.0	1	21	33	53	UUGAACAUCCCAGAGCCACCG	ACGUGGCUCUGGGAUGUUCAG	Cleavage
tae-miR9662b-3p	Traes_6AS_48B701BB41.5	PF02536				GO:GO:0006355,GO:GO:0005739,GO:GO:0003690	AT1G21150.1	Mitochondrial transcription termination factor family protein	LOC_Os06g12100.1	mTERF family protein, expressed	1,00	-1.0	1	21	32	52	UGAACAUCCCAGAGCCACCGG	CACGUGGCUCUGGGAUGUUCA	Cleavage
tae-miR1138	Traes_6AS_5F8725CDA.1	PF02705			K03549	GO:GO:0071805,GO:GO:0016020,GO:GO:0015079	AT4G13420.1	high affinity K+ transporter 5	LOC_Os02g31910.2	potassium transporter, putative, expressed	3,00	-1.0	1	23	2223	2245	GCUUAGAUGUGACAUCCUUAAAA	AGUUAGUUAUGUCACAUCUAAGC	Cleavage
tae-miR1138	Traes_6AS_5F8725CDA.2	PF02705				GO:GO:0071805,GO:GO:0016020,GO:GO:0015079	AT4G13420.1	high affinity K+ transporter 5	LOC_Os02g31910.1	potassium transporter, putative, expressed	3,00	-1.0	1	23	1969	1991	GCUUAGAUGUGACAUCCUUAAAA	AGUUAGUUAUGUCACAUCUAAGC	Cleavage
tae-miR9662a-3p	Traes_6AS_6612DF8D7.1	PF02536				GO:GO:0006355,GO:GO:0005739,GO:GO:0003690	AT5G07900.1	Mitochondrial transcription termination factor family protein	LOC_Os06g12100.1	mTERF family protein, expressed	1,00	-1.0	1	21	81	101	UUGAACAUCCCAGAGCCACCG	CCGUGGCUCUGGGAUGUUCAG	Cleavage
tae-miR9662b-3p	Traes_6AS_6612DF8D7.1	PF02536				GO:GO:0006355,GO:GO:0005739,GO:GO:0003690	AT5G07900.1	Mitochondrial transcription termination factor family protein	LOC_Os06g12100.1	mTERF family protein, expressed	1,00	-1.0	1	21	80	100	UGAACAUCCCAGAGCCACCGG	CCCGUGGCUCUGGGAUGUUCA	Cleavage
tae-miR9662a-3p	Traes_6AS_6612DF8D7.2	PF02536				GO:GO:0006355,GO:GO:0005739,GO:GO:0003690	AT5G07900.1	Mitochondrial transcription termination factor family protein	LOC_Os06g12100.1	mTERF family protein, expressed	1,00	-1.0	1	21	81	101	UUGAACAUCCCAGAGCCACCG	CCGUGGCUCUGGGAUGUUCAG	Cleavage
tae-miR9662b-3p	Traes_6AS_6612DF8D7.2	PF02536				GO:GO:0006355,GO:GO:0005739,GO:GO:0003690	AT5G07900.1	Mitochondrial transcription termination factor family protein	LOC_Os06g12100.1	mTERF family protein, expressed	1,00	-1.0	1	21	80	100	UGAACAUCCCAGAGCCACCGG	CCCGUGGCUCUGGGAUGUUCA	Cleavage
tae-miR9662a-3p	Traes_6AS_6612DF8D7.3	PF02536				GO:GO:0006355,GO:GO:0005739,GO:GO:0003690	AT5G07900.1	Mitochondrial transcription termination factor family protein	LOC_Os06g12100.1	mTERF family protein, expressed	1,00	-1.0	1	21	81	101	UUGAACAUCCCAGAGCCACCG	CCGUGGCUCUGGGAUGUUCAG	Cleavage
tae-miR9662b-3p	Traes_6AS_6612DF8D7.3	PF02536				GO:GO:0006355,GO:GO:0005739,GO:GO:0003690	AT5G07900.1	Mitochondrial transcription termination factor family protein	LOC_Os06g12100.1	mTERF family protein, expressed	1,00	-1.0	1	21	80	100	UGAACAUCCCAGAGCCACCGG	CCCGUGGCUCUGGGAUGUUCA	Cleavage
tae-miR9662a-3p	Traes_6AS_8D2111A83.1	PF02536				GO:GO:0006355,GO:GO:0005739,GO:GO:0003690	AT1G21150.1	Mitochondrial transcription termination factor family protein	LOC_Os06g12100.1	mTERF family protein, expressed	2,50	-1.0	1	21	33	53	UUGAACAUCCCAGAGCCACCG	UCGUGGCUCGGGGAUGUUCAG	Cleavage
tae-miR9662b-3p	Traes_6AS_8D2111A83.1	PF02536				GO:GO:0006355,GO:GO:0005739,GO:GO:0003690	AT1G21150.1	Mitochondrial transcription termination factor family protein	LOC_Os06g12100.1		2,50	-1.0	1	21	32	52	UGAACAUCCCAGAGCCACCGG	CUCGUGGCUCGGGGAUGUUCA	Translation
tae-miR9662a-3p	Traes_6AS_8D2111A83.2	PF02536				GO:GO:0006355,GO:GO:0005739,GO:GO:0003690	AT1G21150.1	Mitochondrial transcription termination factor family protein	LOC_Os06g12100.1	mTERF family protein, expressed	2,50	-1.0	1	21	246	266	UUGAACAUCCCAGAGCCACCG	UCGUGGCUCGGGGAUGUUCAG	Cleavage
tae-miR9662b-3p	Traes_6AS_8D2111A83.2	PF02536				GO:GO:0006355,GO:GO:0005739,GO:GO:0003690	AT1G21150.1	Mitochondrial transcription termination factor family protein	LOC_Os06g12100.1	mTERF family protein, expressed	2,50	-1.0	1	21	245	265	UGAACAUCCCAGAGCCACCGG	CUCGUGGCUCGGGGAUGUUCA	Translation
tae-miR9662a-3p	Traes_6AS_8D2111A83.3	PF02536				GO:GO:0006355,GO:GO:0005739,GO:GO:0003690	AT1G21150.1	Mitochondrial transcription termination factor family protein	LOC_Os06g12100.1	mTERF family protein, expressed	2,50	-1.0	1	21	246	266	UUGAACAUCCCAGAGCCACCG	UCGUGGCUCGGGGAUGUUCAG	Cleavage
tae-miR9662b-3p	Traes_6AS_8D2111A83.3	PF02536				GO:GO:0006355,GO:GO:0005739,GO:GO:0003690	AT1G21150.1	Mitochondrial transcription termination factor family protein	LOC_Os06g12100.1	mTERF family protein, expressed	2,50	-1.0	1	21	245	265	UGAACAUCCCAGAGCCACCGG	CUCGUGGCUCGGGGAUGUUCA	Translation
tae-miR9662a-3p	Traes_6AS_8D2111A83.4	PF02536				GO:GO:0006355,GO:GO:0005739,GO:GO:0003690	AT1G21150.1	Mitochondrial transcription termination factor family protein	LOC_Os06g12100.1	mTERF family protein, expressed	2,50	-1.0	1	21	246	266	UUGAACAUCCCAGAGCCACCG	UCGUGGCUCGGGGAUGUUCAG	Cleavage
tae-miR9662b-3p	Traes_6AS_8D2111A83.4	PF02536				GO:GO:0006355,GO:GO:0005739,GO:GO:0003690	AT1G21150.1	Mitochondrial transcription termination factor family protein	LOC_Os06g12100.1	mTERF family protein, expressed	2,50	-1.0	1	21	245	265	UGAACAUCCCAGAGCCACCGG	CUCGUGGCUCGGGGAUGUUCA	Translation
tae-miR9662a-3p	Traes_6AS_8D2111A83.5	PF02536				GO:GO:0006355,GO:GO:0005739,GO:GO:0003690	AT1G21150.1	Mitochondrial transcription termination factor family protein	LOC_Os06g12100.1	mTERF family protein, expressed	2,50	-1.0	1	21	246	266	UUGAACAUCCCAGAGCCACCG	UCGUGGCUCGGGGAUGUUCAG	Cleavage
tae-miR9662b-3p	Traes_6AS_8D2111A83.5	PF02536				GO:GO:0006355,GO:GO:0005739,GO:GO:0003690	AT1G21150.1	Mitochondrial transcription termination factor family protein	LOC_Os06g12100.1	mTERF family protein, expressed	2,50	-1.0	1	21	245	265	UGAACAUCCCAGAGCCACCGG	CUCGUGGCUCGGGGAUGUUCA	Translation
tae-miR9662a-3p	Traes_6AS_8D2111A83.6						AT1G21150.1	Mitochondrial transcription termination factor family protein	LOC_Os06g12100.1	mTERF family protein, expressed	2,50	-1.0	1	21	246	266	UUGAACAUCCCAGAGCCACCG	UCGUGGCUCGGGGAUGUUCAG	Cleavage
tae-miR9662b-3p	Traes_6AS_8D2111A83.6						AT1G21150.1	Mitochondrial transcription termination factor family protein	LOC_Os06g12100.1	mTERF family protein, expressed	2,50	-1.0	1	21	245	265	UGAACAUCCCAGAGCCACCGG	CUCGUGGCUCGGGGAUGUUCA	Translation
tae-miR9662a-3p	Traes_6AS_8D2111A83.7	PF02536				GO:GO:0006355,GO:GO:0005739,GO:GO:0003690	AT1G21150.1	Mitochondrial transcription termination factor family protein	LOC_Os06g12100.1	mTERF family protein, expressed	2,50	-1.0	1	21	33	53	UUGAACAUCCCAGAGCCACCG	UCGUGGCUCGGGGAUGUUCAG	Cleavage
tae-miR9662b-3p	Traes_6AS_8D2111A83.7	PF02536				GO:GO:0006355,GO:GO:0005739,GO:GO:0003690	AT1G21150.1	Mitochondrial transcription termination factor family protein	LOC_Os06g12100.1	mTERF family protein, expressed	2,50	-1.0	1	21	32	52	UGAACAUCCCAGAGCCACCGG	CUCGUGGCUCGGGGAUGUUCA	Translation
tae-miR1122b-3p	Traes_6AS_8E6E52967.2	PF08263					AT1G17250.1	receptor like protein 3	LOC_Os02g06120.1	Leucine Rich Repeat family protein, expressed	1,50	-1.0	1	21	120	140	AGACUUAUAUGUAGGAACGGA	UCCGUUCCUAAAUAUAAGUCU	Translation
tae-miR156	Traes_6AS_99C6A3B0B.1	PF03110				GO:GO:0005634,GO:GO:0003677	AT5G43270.1	squamosa promoter binding protein-like 2	LOC_Os02g04680.2	OsSPL3 - SBP-box gene family member, expressed	1,00	-1.0	1	21	1477	1497	UGACAGAAGAGAGUGAGCACA	CAUGCUCUCUCUCUUCUGUCA	Cleavage
tae-miR156	Traes_6AS_99C6A3B0B.2	PF03110				GO:GO:0005634,GO:GO:0003677	AT5G43270.3	squamosa promoter binding protein-like 2	LOC_Os02g04680.2	OsSPL3 - SBP-box gene family member, expressed	1,00	-1.0	1	21	1477	1497	UGACAGAAGAGAGUGAGCACA	CAUGCUCUCUCUCUUCUGUCA	Cleavage
tae-miR156	Traes_6AS_99C6A3B0B.3	PF03110				GO:GO:0005634,GO:GO:0003677	AT5G43270.2	squamosa promoter binding protein-like 2	LOC_Os02g04680.2	OsSPL3 - SBP-box gene family member, expressed	1,00	-1.0	1	21	1477	1497	UGACAGAAGAGAGUGAGCACA	CAUGCUCUCUCUCUUCUGUCA	Cleavage
tae-miR156	Traes_6AS_99C6A3B0B.4	PF03110				GO:GO:0005634,GO:GO:0003677	AT5G43270.2	squamosa promoter binding protein-like 2	LOC_Os02g04680.2	OsSPL3 - SBP-box gene family member, expressed	1,00	-1.0	1	21	1156	1176	UGACAGAAGAGAGUGAGCACA	CAUGCUCUCUCUCUUCUGUCA	Cleavage
tae-miR156	Traes_6AS_99C6A3B0B.5	PF03110				GO:GO:0005634,GO:GO:0003677	AT5G43270.3	squamosa promoter binding protein-like 2	LOC_Os02g04680.2	OsSPL3 - SBP-box gene family member, expressed	1,00	-1.0	1	21	1156	1176	UGACAGAAGAGAGUGAGCACA	CAUGCUCUCUCUCUUCUGUCA	Cleavage
tae-miR156	Traes_6AS_99C6A3B0B.6	PF03110				GO:GO:0005634,GO:GO:0003677	AT5G43270.1	squamosa promoter binding protein-like 2	LOC_Os02g04680.2	OsSPL3 - SBP-box gene family member, expressed	1,00	-1.0	1	21	1156	1176	UGACAGAAGAGAGUGAGCACA	CAUGCUCUCUCUCUUCUGUCA	Cleavage
tae-miR156	Traes_6AS_99C6A3B0B.7	PF03110				GO:GO:0005634,GO:GO:0003677	AT5G43270.3	squamosa promoter binding protein-like 2	LOC_Os02g04680.2	OsSPL3 - SBP-box gene family member, expressed	1,00	-1.0	1	21	1156	1176	UGACAGAAGAGAGUGAGCACA	CAUGCUCUCUCUCUUCUGUCA	Cleavage
tae-miR156	Traes_6AS_99C6A3B0B.8	PF03110				GO:GO:0005634,GO:GO:0003677	AT5G43270.1	squamosa promoter binding protein-like 2	LOC_Os02g04680.2	OsSPL3 - SBP-box gene family member, expressed	1,00	-1.0	1	21	1156	1176	UGACAGAAGAGAGUGAGCACA	CAUGCUCUCUCUCUUCUGUCA	Cleavage
tae-miR9662a-3p	Traes_6AS_A2EAE01C1.1	PF02536				GO:GO:0006355,GO:GO:0005739,GO:GO:0003690	AT5G07900.1	Mitochondrial transcription termination factor family protein	LOC_Os06g12100.1	mTERF family protein, expressed	2,50	-1.0	1	21	465	485	UUGAACAUCCCAGAGCCACCG	CCGUGGCUCUGGAAUGUUCAG	Cleavage
tae-miR9662b-3p	Traes_6AS_A2EAE01C1.1	PF02536				GO:GO:0006355,GO:GO:0005739,GO:GO:0003690	AT5G07900.1	Mitochondrial transcription termination factor family protein	LOC_Os06g12100.1	mTERF family protein, expressed	2,50	-1.0	1	21	464	484	UGAACAUCCCAGAGCCACCGG	CCCGUGGCUCUGGAAUGUUCA	Cleavage
tae-miR9662a-3p	Traes_6AS_A2EAE01C1.2	PF02536				GO:GO:0006355,GO:GO:0005739,GO:GO:0003690	AT5G07900.1	Mitochondrial transcription termination factor family protein	LOC_Os06g12100.1	mTERF family protein, expressed	2,50	-1.0	1	21	66	86	UUGAACAUCCCAGAGCCACCG	CCGUGGCUCUGGAAUGUUCAG	Cleavage
tae-miR9662b-3p	Traes_6AS_A2EAE01C1.2	PF02536				GO:GO:0006355,GO:GO:0005739,GO:GO:0003690	AT5G07900.1	Mitochondrial transcription termination factor family protein	LOC_Os06g12100.1	mTERF family protein, expressed	2,50	-1.0	1	21	65	85	UGAACAUCCCAGAGCCACCGG	CCCGUGGCUCUGGAAUGUUCA	Cleavage
tae-miR9662a-3p	Traes_6AS_B24138E62.1	PF02536				GO:GO:0006355,GO:GO:0005739,GO:GO:0003690	AT5G07900.1	Mitochondrial transcription termination factor family protein	LOC_Os06g12100.1	mTERF family protein, expressed	2,50	-1.0	1	21	375	395	UUGAACAUCCCAGAGCCACCG	CCGUGGCUCUGGAAUGUUCAG	Cleavage
tae-miR9662b-3p	Traes_6AS_B24138E62.1	PF02536				GO:GO:0006355,GO:GO:0005739,GO:GO:0003690	AT5G07900.1	Mitochondrial transcription termination factor family protein	LOC_Os06g12100.1	mTERF family protein, expressed	2,50	-1.0	1	21	374	394	UGAACAUCCCAGAGCCACCGG	CCCGUGGCUCUGGAAUGUUCA	Cleavage
tae-miR9662a-3p	Traes_6AS_EBADF37C7.1	PF02536				GO:GO:0006355,GO:GO:0005739,GO:GO:0003690	AT5G07900.1	Mitochondrial transcription termination factor family protein	LOC_Os06g12100.1	mTERF family protein, expressed	2,50	-1.0	1	21	681	701	UUGAACAUCCCAGAGCCACCG	CCGUGGCUCUGGAUGCUCAG	Cleavage

tae-miR9662b-3p	Traes_6AS_EBADF37C7.1	PF02536				GO:GO:0006355,GO:GO:0005739,GO:GO:0003690	AT5G07900.1	Mitochondrial transcription termination factor family protein	LOC_Os06g12100.1	mTERF family protein, expressed	2,50	-1.0	1	21	680	700	UGAACAUCCCAGAGCCACCGG	CCCGUGGCUCUGGGAUGCUCA	Cleavage
tae-miR9662a-3p	Traes_6AS_EBADF37C7.2	PF02536				GO:GO:0006355,GO:GO:0005739,GO:GO:0003690	AT5G07900.1	Mitochondrial transcription termination factor family protein	LOC_Os06g12100.1	mTERF family protein, expressed	2,50	-1.0	1	21	378	398	UUGAACAUCCCAGAGCCACCG	CCGUGGCUCUGGGAUGCUCAG	Cleavage
tae-miR9662b-3p	Traes_6AS_EBADF37C7.2	PF02536				GO:GO:0006355,GO:GO:0005739,GO:GO:0003690	AT5G07900.1	Mitochondrial transcription termination factor family protein	LOC_Os06g12100.1	mTERF family protein, expressed	2,50	-1.0	1	21	377	397	UGAACAUCCCAGAGCCACCGG	CCCGUGGCUCUGGGAUGCUCA	Cleavage
tae-miR9662a-3p	Traes_6AS_EE73C76541.2	PF02536				GO:GO:0006355,GO:GO:0005739,GO:GO:0003690	AT5G07900.1	Mitochondrial transcription termination factor family protein	LOC_Os06g12100.1	mTERF family protein, expressed	1,00	-1.0	1	21	420	440	UUGAACAUCCCAGAGCCACCG	CCGUGGCUCUGGGAUGUUCAG	Cleavage
tae-miR9662b-3p	Traes_6AS_EE73C76541.2	PF02536				GO:GO:0006355,GO:GO:0005739,GO:GO:0003690	AT5G07900.1	Mitochondrial transcription termination factor family protein	LOC_Os06g12100.1	mTERF family protein, expressed	1,00	-1.0	1	21	419	439	UGAACAUCCCAGAGCCACCGG	CCCGUGGCUCUGGGAUGUUCA	Cleavage
tae-miR9662a-3p	Traes_6AS_EE73C76541.6	PF02536				GO:GO:0006355,GO:GO:0005739,GO:GO:0003690	AT5G07900.1	Mitochondrial transcription termination factor family protein	LOC_Os06g12100.1	mTERF family protein, expressed	1,00	-1.0	1	21	66	86	UUGAACAUCCCAGAGCCACCG	CCGUGGCUCUGGGAUGUUCAG	Cleavage
tae-miR9662b-3p	Traes_6AS_EE73C76541.6	PF02536				GO:GO:0006355,GO:GO:0005739,GO:GO:0003690	AT5G07900.1	Mitochondrial transcription termination factor family protein	LOC_Os06g12100.1	mTERF family protein, expressed	1,00	-1.0	1	21	65	85	UGAACAUCCCAGAGCCACCGG	CCCGUGGCUCUGGGAUGUUCA	Cleavage
tae-miR9662a-3p	Traes_6AS_EE8BA0A57.1	PF02536				GO:GO:0006355,GO:GO:0005739,GO:GO:0003690	AT1G21150.1	Mitochondrial transcription termination factor family protein	LOC_Os06g12100.1	mTERF family protein, expressed	2,00	-1.0	1	21	462	482	UUGAACAUCCCAGAGCCACCG	CCGUGCCUCUGGGAUGUUCAG	Cleavage
tae-miR9662b-3p	Traes_6AS_EE8BA0A57.1	PF02536				GO:GO:0006355,GO:GO:0005739,GO:GO:0003690	AT1G21150.1	Mitochondrial transcription termination factor family protein	LOC_Os06g12100.1	mTERF family protein, expressed	2,00	-1.0	1	21	461	481	UGAACAUCCCAGAGCCACCGG	CCCGUGCCUCUGGGAUGUUCA	Cleavage
tae-miR9662a-3p	Traes_6AS_EE8BA0A57.2	PF02536				GO:GO:0006355,GO:GO:0005739,GO:GO:0003690	AT5G07900.1	Mitochondrial transcription termination factor family protein	LOC_Os06g12100.1	mTERF family protein, expressed	2,00	-1.0	1	21	510	530	UUGAACAUCCCAGAGCCACCG	CCGUGCCUCUGGGAUGUUCAG	Cleavage
tae-miR9662b-3p	Traes_6AS_EE8BA0A57.2	PF02536				GO:GO:0006355,GO:GO:0005739,GO:GO:0003690	AT5G07900.1	Mitochondrial transcription termination factor family protein	LOC_Os06g12100.1	mTERF family protein, expressed	2,00	-1.0	1	21	509	529	UGAACAUCCCAGAGCCACCGG	CCCGUGCCUCUGGGAUGUUCA	Cleavage
tae-miR5049-3p	Traes_6AS_F5DBB6DBD.3	PF13855	KOG0472			GO:GO:0005515	AT1G72300.1	Leucine-rich receptor-like protein kinase family protein	LOC_Os02g05970.1	phytosulfokine receptor precursor, putative, expressed	1,50	-1.0	1	21	1927	1947	AAUAUGGAUCGGAGGGAGUAC	UUACUCCCUCCAAUCCAUAUU	Translation
tae-miR9662a-3p	Traes_6AS_FBBCFBCB0.1	PF02536	KOG1267			GO:GO:0006355,GO:GO:0005739,GO:GO:0003690	AT5G07900.1	Mitochondrial transcription termination factor family protein	LOC_Os06g12100.1	mTERF family protein, expressed	2,50	-1.0	1	21	681	701	UUGAACAUCCCAGAGCCACCG	CCGUGGCUCUGGGAUGUUUAG	Cleavage
tae-miR9662b-3p	Traes_6AS_FBBCFBCB0.1	PF02536	KOG1267			GO:GO:0006355,GO:GO:0005739,GO:GO:0003690	AT5G07900.1	Mitochondrial transcription termination factor family protein	LOC_Os06g12100.1	mTERF family protein, expressed	2,50	-1.0	1	21	680	700	UGAACAUCCCAGAGCCACCGG	CCCGUGGCUCUGGGAUGUUUA	Cleavage
tae-miR1127b-3p	Traes_6BL_028068EFA.2		KOG1877				AT5G21080.1	Uncharacterized protein	LOC_Os02g53990.1	cyclin-related protein, putative, expressed	1,50	-1.0	1	21	2801	2821	ACAAGUAUUUCUGGACGGAGG	CCUCCGUCCGGAAAUACUUGU	Cleavage
tae-miR6197-5p	Traes_6BL_67725F06B.2	PF02358		3.1.3.12		GO:GO:0005992,GO:GO:0003824	AT5G65140.2	Haloacid dehalogenase-like hydrolase (HAD) superfamily protein	LOC_Os02g51680.1	uncharacterized glycosyl hydrolase Rv2006/MT2062, putative, expressed	3,00	-1.0	1	21	278	298	UCUGUAAACAAAUGUAGGACG	GAACCUCCAUUUGUUUACAGG	Cleavage
tae-miR1127b-3p	Traes_6BL_731ED1C4C.1	PF00646				GO:GO:0005515	AT5G56820.1	F-box/RNI-like/FBD-like domains-containing protein	LOC_Os02g57910.1	OsFBX73 - F-box domain containing protein, expressed	1,50	-1.0	1	21	1020	1040	ACAAGUAUUUCUGGACGGAGG	UCUCCGUCCAAAAAUACUUGU	Translation
tae-miR1130b-3p	Traes_6BL_7BD68BAC2.3	PF00366				GO:GO:0006412,GO:GO:0005840,GO:GO:0005622,GO:GO:0003735	AT5G23740.1	ribosomal protein S11-beta	LOC_Os04g52361.1	ribosomal protein S17, putative, expressed	1,50	-1.0	1	21	215	235	UCUUAUAUUAUGGGACGGAGG	CCUCCGUCCCAUAAUGUAAGA	Cleavage
tae-miR1122a	Traes_6BL_7E9C131F4.3	PF00249	KOG0724				AT5G52660.1	Homeodomain-like superfamily protein	LOC_Os02g45670.1	myb-like DNA-binding domain containing protein, expressed	0,00	-1.0	1	20	761	780	UAGAUACAUCCGUAUCUAGA	UCUAGAUACGGAUGUAUCUA	Cleavage
tae-miR1130b-3p	Traes_6BL_887005D55.2										1,50	-1.0	1	21	79	99	UCUUAUAUUAUGGGACGGAGG	CCUCCGUCCCAUAAUAUAAUA	Cleavage
tae-miR1118	Traes_6BL_E14604962.2	PF00076				GO:GO:0003676	AT5G51120.2	polyadenylate-binding protein 1	LOC_Os02g52140.2	RNA recognition motif containing protein, putative, expressed	2,00	-1.0	1	23	35	57	CACUACAUUAUGGAAUGGAGGGA	UCCCUCUGUUCCAUAAUGUAGUG	Cleavage
tae-miR1118	Traes_6BL_E14604962.3	PF00076				GO:GO:0003676	AT5G51120.2	polyadenylate-binding protein 1	LOC_Os02g52140.2	RNA recognition motif containing protein, putative, expressed	2,00	-1.0	1	23	35	57	CACUACAUUAUGGAAUGGAGGGA	UCCCUCUGUUCCAUAAUGUAGUG	Cleavage
tae-miR1127b-3p	Traes_6BS_1AFDF35E5.3										2,50	-1.0	1	21	171	191	ACAAGUAUUUCUGGACGGAGG	CUUUCGUCCGGAAAUACUUGU	Cleavage
tae-miR9662a-3p	Traes_6BS_28F0D2955.1	PF02536				GO:GO:0006355,GO:GO:0005739,GO:GO:0003690	AT5G64950.1	Mitochondrial transcription termination factor family protein	LOC_Os06g12100.1	mTERF family protein, expressed	2,50	-1.0	1	21	396	416	UUGAACAUCCCAGAGCCACCG	CUGUGGCUCUCGGAUGUUCAG	Translation
tae-miR9662b-3p	Traes_6BS_28F0D2955.1	PF02536				GO:GO:0006355,GO:GO:0005739,GO:GO:0003690	AT5G64950.1	Mitochondrial transcription termination factor family protein	LOC_Os06g12100.1	mTERF family protein, expressed	2,50	-1.0	1	21	395	415	UGAACAUCCCAGAGCCACCGG	CCUGUGGCUCUCGGAUGUUCA	Translation
tae-miR9662a-3p	Traes_6BS_2AC6A2287.1	PF02536				GO:GO:0006355,GO:GO:0005739,GO:GO:0003690	AT5G07900.1	Mitochondrial transcription termination factor family protein	LOC_Os06g12100.1	mTERF family protein, expressed	2,50	-1.0	1	21	66	86	UUGAACAUCCCAGAGCCACCG	CCGUGGCUCUGGGAUGCUCAG	Cleavage
tae-miR9662b-3p	Traes_6BS_2AC6A2287.1	PF02536				GO:GO:0006355,GO:GO:0005739,GO:GO:0003690	AT5G07900.1	Mitochondrial transcription termination factor family protein	LOC_Os06g12100.1	mTERF family protein, expressed	2,50	-1.0	1	21	65	85	UGAACAUCCCAGAGCCACCGG	CCCGUGGCUCUGGGAUGCUCA	Cleavage
tae-miR9662a-3p	Traes_6BS_2F108C5BB.1	PF02536				GO:GO:0006355,GO:GO:0005739,GO:GO:0003690	AT5G07900.1	Mitochondrial transcription termination factor family protein	LOC_Os06g12100.1	mTERF family protein, expressed	2,00	-1.0	1	21	435	455	UUGAACAUCCCAGAGCCACCG	UCGUGGCGCUGGGAUGUUCAG	Cleavage
tae-miR9662b-3p	Traes_6BS_2F108C5BB.1	PF02536				GO:GO:0006355,GO:GO:0005739,GO:GO:0003690	AT5G07900.1	Mitochondrial transcription termination factor family protein	LOC_Os06g12100.1	mTERF family protein, expressed	2,50	-1.0	1	21	434	454	UGAACAUCCCAGAGCCACCGG	CUCGUGGCGCUGGGAUGUUCA	Cleavage
tae-miR408	Traes_6BS_348308070.13	PF12937				GO:GO:0005515	AT3G17540.1	F-box and associated interaction domains-containing protein	LOC_Os10g04700.1	OsFBX361 - F-box domain containing protein, expressed	2,50	-1.0	1	21	2	22	CUGCACUGCCUCUUCCCUGGC	CCCAGGGCCGAGGCAGUGCAG	Cleavage
tae-miR408	Traes_6BS_348308070.15	PF12937				GO:GO:0005515	AT3G17540.1	F-box and associated interaction domains-containing protein	LOC_Os10g04700.1	OsFBX361 - F-box domain containing protein, expressed	2,50	-1.0	1	21	11	31	CUGCACUGCCUCUUCCCUGGC	CCCAGGGCCGAGGCAGUGCAG	Cleavage
tae-miR408	Traes_6BS_348308070.17	PF12937				GO:GO:0005515	AT3G17540.1	F-box and associated interaction domains-containing protein	LOC_Os10g04700.1	OsFBX361 - F-box domain containing protein, expressed	2,50	-1.0	1	21	14	34	CUGCACUGCCUCUUCCCUGGC	CCCAGGGCCGAGGCAGUGCAG	Cleavage
tae-miR408	Traes_6BS_348308070.19	PF12937				GO:GO:0005515	AT3G17540.1	F-box and associated interaction domains-containing protein	LOC_Os10g04700.1	OsFBX361 - F-box domain containing protein, expressed	2,50	-1.0	1	21	11	31	CUGCACUGCCUCUUCCCUGGC	CCCAGGGCCGAGGCAGUGCAG	Cleavage
tae-miR408	Traes_6BS_348308070.2	PF12937				GO:GO:0005515	AT5G03970.2	F-box associated ubiquitination effector family protein	LOC_Os10g04700.2	OsFBX361 - F-box domain containing protein, expressed	2,50	-1.0	1	21	14	34	CUGCACUGCCUCUUCCCUGGC	CCCAGGGCCGAGGCAGUGCAG	Cleavage
tae-miR408	Traes_6BS_348308070.3	PF12937				GO:GO:0005515	AT3G17540.1	F-box and associated interaction domains-containing protein	LOC_Os10g04700.1	OsFBX361 - F-box domain containing protein, expressed	2,50	-1.0	1	21	17	37	CUGCACUGCCUCUUCCCUGGC	CCCAGGGCCGAGGCAGUGCAG	Cleavage
tae-miR408	Traes_6BS_348308070.5	PF12937				GO:GO:0005515	AT3G17540.1	F-box and associated interaction domains-containing protein	LOC_Os10g04700.2	OsFBX361 - F-box domain containing protein, expressed	2,50	-1.0	1	21	17	37	CUGCACUGCCUCUUCCCUGGC	CCCAGGGCCGAGGCAGUGCAG	Cleavage
tae-miR408	Traes_6BS_348308070.6	PF12937				GO:GO:0005515	AT5G03970.2	F-box associated ubiquitination effector family protein	LOC_Os10g04700.2	OsFBX361 - F-box domain containing protein, expressed	2,50	-1.0	1	21	14	34	CUGCACUGCCUCUUCCCUGGC	CCCAGGGCCGAGGCAGUGCAG	Cleavage
tae-miR408	Traes_6BS_348308070.8	PF12937				GO:GO:0005515	AT3G17540.1	F-box and associated interaction domains-containing protein	LOC_Os10g04700.1	OsFBX361 - F-box domain containing protein, expressed	2,50	-1.0	1	21	17	37	CUGCACUGCCUCUUCCCUGGC	CCCAGGGCCGAGGCAGUGCAG	Cleavage
tae-miR408	Traes_6BS_348308070.9	PF12937				GO:GO:0005515	AT3G17540.1	F-box and associated interaction domains-containing protein	LOC_Os10g04700.1	OsFBX361 - F-box domain containing protein, expressed	2,50	-1.0	1	21	17	37	CUGCACUGCCUCUUCCCUGGC	CCCAGGGCCGAGGCAGUGCAG	Cleavage
tae-miR9662a-3p	Traes_6BS_3A00751B5.1	PF02536				GO:GO:0006355,GO:GO:0005739,GO:GO:0003690	AT5G07900.1	Mitochondrial transcription termination factor family protein	LOC_Os06g12100.1	mTERF family protein, expressed	2,50	-1.0	1	21	69	89	UUGAACAUCCCAGAGCCACCG	CCGUGGCUCUGGGAUGUUUAG	Cleavage
tae-miR9662b-3p	Traes_6BS_3A00751B5.1	PF02536				GO:GO:0006355,GO:GO:0005739,GO:GO:0003690	AT5G07900.1	Mitochondrial transcription termination factor family protein	LOC_Os06g12100.1	mTERF family protein, expressed	2,50	-1.0	1	21	68	88	UGAACAUCCCAGAGCCACCGG	CCCGUGGCUCUGGGAUGUUUA	Cleavage
tae-miR9662a-3p	Traes_6BS_3A00751B5.2	PF02536				GO:GO:0006355,GO:GO:0005739,GO:GO:0003690	AT5G07900.1	Mitochondrial transcription termination factor family protein	LOC_Os06g12100.1	mTERF family protein, expressed	2,50	-1.0	1	21	69	89	UUGAACAUCCCAGAGCCACCG	CCGUGGCUCUGGGAUGUUUAG	Cleavage
tae-miR9662b-3p	Traes_6BS_3A00751B5.2	PF02536				GO:GO:0006355,GO:GO:0005739,GO:GO:0003690	AT5G07900.1	Mitochondrial transcription termination factor family protein	LOC_Os06g12100.1	mTERF family protein, expressed	2,50	-1.0	1	21	68	88	UGAACAUCCCAGAGCCACCGG	CCCGUGGCUCUGGGAUGUUUA	Cleavage
tae-miR9662a-3p	Traes_6BS_46641470F.1	PF02536				GO:GO:0006355,GO:GO:0005739,GO:GO:0003690	AT1G21150.1	Mitochondrial transcription termination factor family protein	LOC_Os06g12100.1	mTERF family protein, expressed	2,50	-1.0	1	21	33	53	UUGAACAUCCCAGAGCCACCG	CCGUGGCUCUGGGAUGUUUAG	Cleavage
tae-miR9662b-3p	Traes_6BS_46641470F.1	PF02536				GO:GO:0006355,GO:GO:0005739,GO:GO:0003690	AT1G21150.1	Mitochondrial transcription termination factor family protein	LOC_Os06g12100.1	mTERF family protein, expressed	2,50	-1.0	1	21	32	52	UGAACAUCCCAGAGCCACCGG	CCCGUGGCUCUGGGAUGUUUA	Cleavage
tae-miR9662a-3p	Traes_6BS_4E6E57FEB.1	PF02536				GO:GO:0006355,GO:GO:0005739,GO:GO:0003690	AT5G07900.1	Mitochondrial transcription termination factor family protein	LOC_Os06g12100.1	mTERF family protein, expressed	3,00	-1.0	1	21	65	86	UUGAACAUCCCAGAGCCAC-CG	CGCGUGGCUCUGGGAUGUUCAG	Cleavage
tae-miR9662b-3p	Traes_6BS_4E6E57FEB.1	PF02536				GO:GO:0006355,GO:GO:0005739,GO:GO:0003690	AT5G07900.1	Mitochondrial transcription termination factor family protein	LOC_Os06g12100.1	mTERF family protein, expressed	2,00	-1.0	1	21	64	85	UGAACAUCCCAGAGCCAC-CGG	CCGCGUGGCUCUGGGAUGUUCA	Cleavage
tae-miR9662a-3p	Traes_6BS_4E6E57FEB.2	PF02536				GO:GO:0006355,GO:GO:0005739,GO:GO:0003690	AT5G07900.1	Mitochondrial transcription termination factor family protein	LOC_Os06g12100.1	mTERF family protein, expressed	3,00	-1.0	1	21	65	86	UUGAACAUCCCAGAGCCAC-CG	CGCGUGGCUCUGGGAUGUUCAG	Cleavage
tae-miR9662b-3p	Traes_6BS_4E6E57FEB.2	PF02536				GO:GO:0006355,GO:GO:0005739,GO:GO:0003690	AT5G07900.1	Mitochondrial transcription termination factor family protein	LOC_Os06g12100.1	mTERF family protein, expressed	2,00	-1.0	1	21	64	85	UGAACAUCCCAGAGCCAC-CGG	CCGCGUGGCUCUGGGAUGUUCA	Cleavage
tae-miR9662a-3p	Traes_6BS_4E6E57FEB.3	PF02536				GO:GO:0006355,GO:GO:0005739,GO:GO:0003690	AT5G07900.1	Mitochondrial transcription termination factor family protein	LOC_Os06g12100.1	mTERF family protein, expressed	3,00	-1.0	1	21	65	86	UUGAACAUCCCAGAGCCAC-CG	CGCGUGGCUCUGGGAUGUUCAG	Cleavage
tae-miR9662b-3p	Traes_6BS_4E6E57FEB.3	PF02536				GO:GO:0006355,GO:GO:0005739,GO:GO:0003690	AT5G07900.1	Mitochondrial transcription termination factor family protein	LOC_Os06g12100.1	mTERF family protein, expressed	2,00	-1.0	1	21	64	85	UGAACAUCCCAGAGCCAC-CGG	CCGCGUGGCUCUGGGAUGUUCA	Cleavage
tae-miR9662b-3p	Traes_6BS_508C2BEA2.1	PF02536				GO:GO:0006355,GO:GO:0005739,GO:GO:0003690	AT1G21150.1	Mitochondrial transcription termination factor family protein	LOC_Os06g12100.1	mTERF family protein, expressed	3,00	-1.0	1	21	32	52	UGAACAUCCCAGAGCCACCGG	CCUGUGGUUCUGGGAUGUUCG	Cleavage
tae-miR156	Traes_6BS_542961EA4.1								LOC_Os06g45310.1	OsSPL11 - SBP-box gene family member, expressed	1,00	-1.0	1	21	283	303	UGACAGAAGAGAGUGAGCACA	CGUGCUCUCUCUCUUCUGUCA	Cleavage
tae-miR156	Traes_6BS_542961EA4.2								LOC_Os06g45310.1	OsSPL11 - SBP-box gene family member, expressed	1,00	-1.0	1	21	349	369	UGACAGAAGAGAGUGAGCACA	CGUGCUCUCUCUCUUCUGUCA	Cleavage
tae-miR1122b-3p	Traes_6BS_7965FEB75.1	PF00300	KOG0235	3.1.3.3	K15634		AT3G50520.1	Phosphoglycerate mutase family protein	LOC_Os08g37140.1	phosphoglycerate mutase, putative, expressed	2,50	-1.0	1	21	87	107	AGACUUAUAUGUAGGAACGGA	UCCGUUCGUAUAUAUAAGUCU	Translation
tae-miR9662a-3p	Traes_6BS_7F1846171.1	PF02536				GO:GO:0006355,GO:GO:0005739,GO:GO:0003690	AT5G07900.1	Mitochondrial transcription termination factor family protein	LOC_Os06g12100.1	mTERF family protein, expressed	2,50	-1.0	1	21	33	53	UUGAACAUCCCAGAGCCACCG	CUGUGGCUCUCGGAUGUUCAG	Translation
tae-miR9662b-3p	Traes_6BS_7F1846171.1	PF02536				GO:GO:0006355,GO:GO:0005739,GO:GO:0003690	AT5G07900.1	Mitochondrial transcription termination factor family protein	LOC_Os06g12100.1	mTERF family protein, expressed	2,50	-1.0	1	21	32	52	UGAACAUCCCAGAGCCACCGG	CCUGUGGCUCUCGGAUGUUCA	Translation
tae-miR9662a-3p	Traes_6BS_82F5BF11F.1	PF02536				GO:GO:0006355,GO:GO:0005739,GO:GO:0003690	AT1G79220.1	Mitochondrial transcription termination factor family protein	LOC_Os06g12100.1	mTERF family protein, expressed	2,00	-1.0	1	21	267	287	UUGAACAUCCCAGAGCCACCG	CCGUGCCUCUGGGAUGUUCAG	Cleavage
tae-miR9662b-3p	Traes_6BS_82F5BF11F.1	PF02536				GO:GO:0006355,GO:GO:0005739,GO:GO:0003690	AT1G79220.1	Mitochondrial transcription termination factor family protein	LOC_Os06g12100.1	mTERF family protein, expressed	2,00	-1.0	1	21	266	286	UGAACAUCCCAGAGCCACCGG	CCCGUGCCUCUGGGAUGUUCA	Cleavage
tae-miR9662a-3p	Traes_6BS_843505CB3.1	PF02536				GO:GO:0006355,GO:GO:0005739,GO:GO:0003690	AT1G21150.1	Mitochondrial transcription termination factor family protein	LOC_Os06g12100.1	mTERF family protein, expressed	2,50	-1.0	1	21	69	89	UUGAACAUCCCAGAGCCACCG	CCGUGGCUCUGGAAUGUUCAG	Cleavage
tae-miR9662b-3p	Traes_6BS_843505CB3.1	PF02536				GO:GO:0006355,GO:GO:0005739,GO:GO:0003690	AT1G21150.1	Mitochondrial transcription termination factor family protein	LOC_Os06g12100.1	mTERF family protein, expressed	2,50	-1.0	1	21	68	88	UGAACAUCCCAGAGCCACCGG	CCCGUGGCUCUGGAAUGUUCA	Cleavage
tae-miR9662a-3p	Traes_6BS_843505CB3.2	PF02536				GO:GO:0006355,GO:GO:0005739,GO:GO:0003690	AT1G21150.1	Mitochondrial transcription termination factor family protein	LOC_Os06g12100.1	mTERF family protein, expressed	2,50	-1.0	1	21	69	89	UUGAACAUCCCAGAGCCACCG	CCGUGGCUCUGGAAUGUUCAG	Cleavage
tae-miR9662b-3p	Traes_6BS_843505CB3.2	PF02536				GO:GO:0006355,GO:GO:0005739,GO:GO:0003690	AT1G21150.1	Mitochondrial transcription termination factor family protein	LOC_Os06g12100.1	mTERF family protein, expressed	2,50	-1.0	1	21	68	88	UGAACAUCCCAGAGCCACCGG	CCCGUGGCUCUGGAAUGUUCA	Cleavage
tae-miR9662a-3p	Traes_6BS_843505CB3.3	PF02536				GO:GO:0006355,GO:GO:0005739,GO:GO:0003690	AT1G21150.1	Mitochondrial transcription termination factor family protein	LOC_Os06g12100.1	mTERF family protein, expressed	2,50	-1.0	1	21	69	89	UUGAACAUCCCAGAGCCACCG	CCGUGGCUCUGGAAUGUUCAG	Cleavage
tae-miR9662b-3p	Traes_6BS_843505CB3.3	PF02536				GO:GO:0006355,GO:GO:0005739,GO:GO:0003690	AT1G21150.1	Mitochondrial transcription termination factor family protein	LOC_Os06g12100.1	mTERF family protein, expressed	2,50	-1.0	1	21	68	88	UGAACAUCCCAGAGCCACCGG	CCCGUGGCUCUGGAAUGUUCA	Cleavage
tae-miR9662a-3p	Traes_6BS_843505CB3.4	PF02536				GO:GO:0006355,GO:GO:0005739,GO:GO:0003690	AT1G21150.1	Mitochondrial transcription termination factor family protein	LOC_Os06g12100.1	mTERF family protein, expressed	2,50	-1.0	1	21	69	89	UUGAACAUCCCAGAGCCACCG	CCGUGGCUCUGGAAUGUUCAG	Cleavage
tae-miR9662b-3p	Traes_6BS_843505CB3.4	PF02536				GO:GO:0006355,GO:GO:0005739,GO:GO:0003690	AT1G21150.1	Mitochondrial transcription termination factor family protein	LOC_Os06g12100.1	mTERF family protein, expressed	2,50	-1.0	1	21	68	88	UGAACAUCCCAGAGCCACCGG	CCCGUGGCUCUGGAAUGUUCA	Cleavage
tae-miR9662a-3p	Traes_6BS_843505CB3.5	PF02536				GO:GO:0006355,GO:GO:0005739,GO:GO:0003690	AT1G21150.1	Mitochondrial transcription termination factor family protein	LOC_Os06g12100.1	mTERF family protein, expressed	2,50	-1.0	1	21	69	89	UUGAACAUCCCAGAGCCACCG	CCGUGGCUCUGGAAUGUUCAG	Cleavage
tae-miR9662b-3p	Traes_6BS_843505CB3.5	PF02536				GO:GO:0006355,GO:GO:0005739,GO:GO:0003690	AT1G21150.1	Mitochondrial transcription termination factor family protein	LOC_Os06g12100.1	mTERF family protein, expressed	2,50	-1.0	1	21	68	88	UGAACAUCCCAGAGCCACCGG	CCCGUGGCUCUGGAAUGUUCA	Cleavage
tae-miR9662a-3p	Traes_6BS_843505CB3.6	PF02536				GO:GO:0006355,GO:GO:0005739,GO:GO:0003690	AT1G21150.1	Mitochondrial transcription termination factor family protein	LOC_Os06g12100.1	mTERF family protein, expressed	2,50	-1.0	1	21	69	89	UUGAACAUCCCAGAGCCACCG	CCGUGGCUCUGGAAUGUUCAG	Cleavage
tae-miR9662b-3p	Traes_6BS_843505CB3.6	PF02536				GO:GO:0006355,GO:GO:0005739,GO:GO:0003690	AT1G21150.1	Mitochondrial transcription termination factor family protein	LOC_Os06g12100.1	mTERF family protein, expressed	2,50	-1.0	1	21	68	88	UGAACAUCCCAGAGCCACCGG	CCCGUGGCUCUGGAAUGUUCA	Cleavage
tae-miR9662a-3p	Traes_6BS_843505CB3.7	PF02536				GO:GO:0006355,GO:GO:0005739,GO:GO:0003690	AT1G21150.1	Mitochondrial transcription termination factor family protein	LOC_Os06g12100.1	mTERF family protein, expressed	2,50	-1.0	1	21	69	89	UUGAACAUCCCAGAGCCACCG	CCGUGGCUCUGGAAUGUUCAG	Cleavage
tae-miR9662b-3p	Traes_6BS_843505CB3.7	PF02536				GO:GO:0006355,GO:GO:0005739,GO:GO:0003690	AT1G21150.1	Mitochondrial transcription termination factor family protein	LOC_Os06g12100.1	mTERF family protein, expressed	2,50	-1.0	1	21	68	88	UGAACAUCCCAGAGCCACCGG	CCCGUGGCUCUGGAAUGUUCA	Cleavage
tae-miR1127b-3p	Traes_6BS_94CE86A01.2	PF00069	KOG0984	2.7.11.1		GO:GO:0006468,GO:GO:0005524,GO:GO:0004672	AT4G23270.1	cysteine-rich RLK (RECEPTOR-like protein kinase) 19	LOC_Os10g12620.1	protein kinase domain containing protein, expressed	2,50	-1.0	1	21	763	783	ACAAGUAUUUCUGGACGGAGG	AAUCCGUUCAGAAUUACUUGU	Cleavage
tae-miR1127a	Traes_6BS_94CE86A01.2	PF00069	KOG0984	2.7.11.1		GO:GO:0006468,GO:GO:0005524,GO:GO:0004672	AT4G23270.1	cysteine-rich RLK (RECEPTOR-like protein kinase) 19	LOC_Os10g12620.1	protein kinase domain containing protein, expressed	1,50	-1.0	1	19	842	860	UCCUUCCGUUCGGAAUUAC	GUAAUUCCGAACGGAGGA	Cleavage
tae-miR9662a-3p	Traes_6BS_97770F04E.1	PF02536				GO:GO:0006355,GO:GO:0005739,GO:GO:0003690	AT5G07900.1	Mitochondrial transcription termination factor family protein	LOC_Os06g12100.1	mTERF family protein, expressed	2,50	-1.0	1	21	33	53	UUGAACAUCCCAGAGCCACCG	UCGUGGCUCUGGAAUGUUCAG	Cleavage
tae-miR9662b-3p	Traes_6BS_97770F04E.1	PF02536				GO:GO:0006355,GO:GO:0005739,GO:GO:0003690	AT5G07900.1	Mitochondrial transcription termination factor family protein	LOC_Os06g12100.1	mTERF family protein, expressed	2,50	-1.0	1	21	32	52	UGAACAUCCCAGAGCCACCGG	CUCGUGGCUCUGGAAUGUUCA	Cleavage
tae-miR9662a-3p	Traes_6BS_97770F04E.3	PF02536				GO:GO:0006355,GO:GO:0005739,GO:GO:0003690	AT5G07900.1	Mitochondrial transcription termination factor family protein	LOC_Os06g12100.1	mTERF family protein, expressed	2,50	-1.0	1	21	33	53	UUGAACAUCCCAGAGCCACCG	UCGUGGCUCUGGAAUGUUCAG	Cleavage
tae-miR9662b-3p	Traes_6BS_97770F04E.3	PF02536				GO:GO:0006355,GO:GO:0005739,GO:GO:0003690	AT5G07900.1	Mitochondrial transcription termination factor family protein	LOC_Os06g12100.1	mTERF family protein, expressed	2,50	-1.0	1	21	32	52	UGAACAUCCCAGAGCCACCGG	CUCGUGGCUCUGGAAUGUUCA	Cleavage
tae-miR9662a-3p	Traes_6BS_97770F04E.4	PF02536				GO:GO:0006355,GO:GO:0005739,GO:GO:0003690	AT5G07900.1	Mitochondrial transcription termination factor family protein	LOC_Os06g12100.1	mTERF family protein, expressed	2,50	-1.0	1	21	33	53	UUGAACAUCCCAGAGCCACCG	UCGUGGCUCUGGAAUGUUCAG	Cleavage
tae-miR9662b-3p	Traes_6BS_97770F04E.4	PF02536				GO:GO:0006355,GO:GO:0005739,GO:GO:0003690	AT5G07900.1	Mitochondrial transcription termination factor family protein	LOC_Os06g12100.1	mTERF family protein, expressed	2,50	-1.0	1	21	32	52	UGAACAUCCCAGAGCCACCGG	CUCGUGGCUCUGGAAUGUUCA	Cleavage
tae-miR9662a-3p	Traes_6BS_97770F04E.5	PF02536				GO:GO:0006355,GO:GO:0005739,GO:GO:0003690	AT5G07900.1	Mitochondrial transcription termination factor family protein	LOC_Os06g12100.1	mTERF family protein, expressed	2,50	-1.0	1	21	33	53	UUGAACAUCCCAGAGCCACCG	UCGUGGCUCUGGAAUGUUCAG	Cleavage
tae-miR9662b-3p	Traes_6BS_97770F04E.5	PF02536				GO:GO:0006355,GO:GO:0005739,GO:GO:0003690	AT5G07900.1	Mitochondrial transcription termination factor family protein	LOC_Os06g12100.1	mTERF family protein, expressed	2,50	-1.0	1	21	32	52	UGAACAUCCCAGAGCCACCGG	CUCGUGGCUCUGGAAUGUUCA	Cleavage
tae-miR9662a-3p	Traes_6BS_97770F04E.6	PF02536				GO:GO:0006355,GO:GO:0005739,GO:GO:0003690	AT5G07900.1	Mitochondrial transcription termination factor family protein	LOC_Os06g12100.1	mTERF family protein, expressed	2,50	-1.0	1	21	33	53	UUGAACAUCCCAGAGCCACCG	UCGUGGCUCUGGAAUGUUCAG	Cleavage
tae-miR9662b-3p	Traes_6BS_97770F04E.6	PF02536				GO:GO:0006355,GO:GO:0005739,GO:GO:0003690	AT5G07900.1	Mitochondrial transcription termination factor family protein	LOC_Os06g12100.1	mTERF family protein, expressed	2,50	-1.0	1	21	32	52	UGAACAUCCCAGAGCCACCGG	CUCGUGGCUCUGGAAUGUUCA	Cleavage
tae-miR1137a	Traes_6BS_A4F1E63CE.2	PF05971		2.1.1.181		GO:GO:0008168	AT2G21070.3	methyltransferases	LOC_Os02g02880.1	expressed protein	2,00	-1.0	1	20	884	903	UAGUACAAAGUUGAGUCAUC	AGUGUCUCAACUUUGUACUA	Cleavage
tae-miR1137a	Traes_6BS_A4F1E63CE.3	PF05971		2.1.1.181		GO:GO:0008168	AT2G21070.3	methyltransferases	LOC_Os02g02880.1	expressed protein	2,00	-1.0	1	20	871	890	UAGUACAAAGUUGAGUCAUC	AGUGUCUCAACUUUGUACUA	Cleavage
tae-miR1128	Traes_6BS_BF5C480A9.12										2,50	-1.0	1	21	60	80	UACUACUCCCUCCGUCCGAAA	UUUGGGACGGAGGGAGUAUUA	Cleavage
tae-miR9662a-3p	Traes_6BS_C3247654A.1	PF02536				GO:GO:0006355,GO:GO:0005739,GO:GO:0003690	AT5G07900.1	Mitochondrial transcription termination factor family protein	LOC_Os06g12100.1	mTERF family protein, expressed	2,50	-1.0	1	21	57	77	UUGAACAUCCCAGAGCCACCG	CUGUGGCUCUGCGAUGUUCAG	Translation
tae-miR9662b-3p	Traes_6BS_C3247654A.1	PF02536				GO:GO:0006355,GO:GO:0005739,GO:GO:0003690	AT5G07900.1	Mitochondrial transcription termination factor family protein	LOC_Os06g12100.1	mTERF family protein, expressed	2,50	-1.0	1	21	56	76	UGAACAUCCCAGAGCCACCGG	CCUGUGGCUCUGCGAUGUUCA	Cleavage
tae-miR9662a-3p	Traes_6BS_CBD931158.1	PF02536				GO:GO:0006355,GO:GO:0005739,GO:GO:0003690	AT1G21150.1	Mitochondrial transcription termination factor family protein	LOC_Os06g12100.1	mTERF family protein, expressed	2,00	-1.0	1	21	33	53	UUGAACAUCCCAGAGCCACCG	CCGUGCCUCUGGGAUGUUCAG	Cleavage
tae-miR9662b-3p	Traes_6BS_CBD931158.1	PF02536				GO:GO:0006355,GO:GO:0005739,GO:GO:0003690	AT1G21150.1	Mitochondrial transcription termination factor family protein	LOC_Os06g12100.1	mTERF family protein, expressed	2,00	-1.0	1	21	32	52	UGAACAUCCCAGAGCCACCGG	CCCGUGCCUCUGGGAUGUUCA	Cleavage
tae-miR9662a-3p	Traes_6BS_CBD931158.2	PF02536				GO:GO:0006355,GO:GO:0005739,GO:GO:0003690	AT1G21150.1	Mitochondrial transcription termination factor family protein	LOC_Os06g12100.1	mTERF family protein, expressed	2,00	-1.0	1	21	33	53	UUGAACAUCCCAGAGCCACCG	CCGUGCCUCUGGGAUGUUCAG	Cleavage
tae-miR9662b-3p	Traes_6BS_CBD931158.2	PF02536				GO:GO:0006355,GO:GO:0005739,GO:GO:0003690	AT1G21150.1	Mitochondrial transcription termination factor family protein	LOC_Os06g12100.1	mTERF family protein, expressed	2,00	-1.0	1	21	32	52	UGAACAUCCCAGAGCCACCGG	CCCGUGCCUCUGGGAUGUUCA	Cleavage
tae-miR9662a-3p	Traes_6BS_CBD931158.3	PF02536				GO:GO:0006355,GO:GO:0005739,GO:GO:0003690	AT1G21150.1	Mitochondrial transcription termination factor family protein	LOC_Os06g12100.1	mTERF family protein, expressed	2,00	-1.0	1	21	33	53	UUGAACAUCCCAGAGCCACCG	CCGUGCCUCUGGGAUGUUCAG	Cleavage
tae-miR9662b-3p	Traes_6BS_CBD931158.3	PF02536				GO:GO:0006355,GO:GO:0005739,GO:GO:0003690	AT1G21150.1	Mitochondrial transcription termination factor family protein	LOC_Os06g12100.1	mTERF family protein, expressed	2,00	-1.0	1	21	32	52	UGAACAUCCCAGAGCCACCGG	CCCGUGCCUCUGGGAUGUUCA	Cleavage
tae-miR9662a-3p	Traes_6BS_CBD931158.4	PF02536				GO:GO:0006355,GO:GO:0005739,GO:GO:0003690	AT1G21150.1	Mitochondrial transcription termination factor family protein	LOC_Os06g12100.1	mTERF family protein, expressed	2,00	-1.0	1	21	33	53	UUGAACAUCCCAGAGCCACCG	CCGUGCCUCUGGGAUGUUCAG	Cleavage
tae-miR9662b-3p	Traes_6BS_CBD931158.4	PF02536				GO:GO:0006355,GO:GO:0005739,GO:GO:0003690	AT1G21150.1	Mitochondrial transcription termination factor family protein	LOC_Os06g12100.1	mTERF family protein, expressed	2,00	-1.0	1	21	32	52	UGAACAUCCCAGAGCCACCGG	CCCGUGCCUCUGGGAUGUUCA	Cleavage
tae-miR9662a-3p	Traes_6BS_CBD931158.5	PF02536				GO:GO:0006355,GO:GO:0005739,GO:GO:0003690	AT1G21150.1	Mitochondrial transcription termination factor family protein	LOC_Os06g12100.1	mTERF family protein, expressed	2,00	-1.0	1	21	33	53	UUGAACAUCCCAGAGCCACCG	CCGUGCCUCUGGGAUGUUCAG	Cleavage
tae-miR9662b-3p	Traes_6BS_CBD931158.5	PF02536				GO:GO:0006355,GO:GO:0005739,GO:GO:0003690	AT1G21150.1	Mitochondrial transcription termination factor family protein	LOC_Os06g12100.1	mTERF family protein, expressed	2,00	-1.0	1	21	32	52	UGAACAUCCCAGAGCCACCGG	CCCGUGCCUCUGGGAUGUUCA	Cleavage
tae-miR9662a-3p	Traes_6BS_CBD931158.6	PF02536				GO:GO:0006355,GO:GO:0005739,GO:GO:0003690	AT1G21150.1	Mitochondrial transcription termination factor family protein	LOC_Os06g12100.1	mTERF family protein, expressed	2,00	-1.0	1	21	33	53	UUGAACAUCCCAGAGCCACCG	CCGUGCCUCUGGGAUGUUCAG	Cleavage
tae-miR9662b-3p	Traes_6BS_CBD931158.6	PF02536				GO:GO:0006355,GO:GO:0005739,GO:GO:0003690	AT1G21150.1	Mitochondrial transcription termination factor family protein	LOC_Os06g12100.1	mTERF family protein, expressed	2,00	-1.0	1	21	32	52	UGAACAUCCCAGAGCCACCGG	CCCGUGCCUCUGGGAUGUUCA	Cleavage
tae-miR156	Traes_6BS_F50663DF7.1	PF03110				GO:GO:0005634,GO:GO:0003677	AT5G43270.1	squamosa promoter binding protein-like 2	LOC_Os02g04680.2	OsSPL3 - SBP-box gene family member, expressed	1,00	-1.0	1	21	1387	1407	UGACAGAAGAGAGUGAGCACA	CAUGCUCUCUCUCUUCUGUCA	Cleavage
tae-miR9662a-3p	Traes_6BS_F8176B2AA.1	PF02536				GO:GO:0006355,GO:GO:0005739,GO:GO:0003690	AT5G64950.1	Mitochondrial transcription termination factor family protein	LOC_Os06g12100.1	mTERF family protein, expressed	2,50	-1.0	1	21	204	224	UUGAACAUCCCAGAGCCACCG	CCGUGGCUCUGCGAUGUUCAG	Translation
tae-miR9662b-3p	Traes_6BS_F8176B2AA.1	PF02536				GO:GO:0006355,GO:GO:0005739,GO:GO:0003690	AT5G64950.1	Mitochondrial transcription termination factor family protein	LOC_Os06g12100.1	mTERF family protein, expressed	2,50	-1.0	1	21	203	223	UGAACAUCCCAGAGCCACCGG	CCCGUGGCUCUGCGAUGUUCA	Cleavage
tae-miR9662a-3p	Traes_6BS_F8176B2AA.2	PF02536				GO:GO:0006355,GO:GO:0005739,GO:GO:0003690	AT5G64950.1	Mitochondrial transcription termination factor family protein	LOC_Os06g12100.1	mTERF family protein, expressed	2,50	-1.0	1	21	204	224	UUGAACAUCCCAGAGCCACCG	CCGUGGCUCUGCGAUGUUCAG	Translation
tae-miR9662b-3p	Traes_6BS_F8176B2AA.2	PF02536				GO:GO:0006355,GO:GO:0005739,GO:GO:0003690	AT5G64950.1	Mitochondrial transcription termination factor family protein	LOC_Os06g12100.1	mTERF family protein, expressed	2,50	-1.0	1	21	203	223	UGAACAUCCCAGAGCCACCGG	CCCGUGGCUCUGCGAUGUUCA	Cleavage
tae-miR9662a-3p	Traes_6BS_F8176B2AA.3	PF02536				GO:GO:0006355,GO:GO:0005739,GO:GO:0003690	AT5G64950.1	Mitochondrial transcription termination factor family protein	LOC_Os06g12100.1	mTERF family protein, expressed	2,50	-1.0	1	21	198	218	UUGAACAUCCCAGAGCCACCG	CCGUGGCUCUGCGAUGUUCAG	Translation
tae-miR9662b-3p	Traes_6BS_F8176B2AA.3	PF02536				GO:GO:0006355,GO:GO:0005739,GO:GO:0003690	AT5G64950.1	Mitochondrial transcription termination factor family protein	LOC_Os06g12100.1	mTERF family protein, expressed	2,50	-1.0	1	21	197	217	UGAACAUCCCAGAGCCACCGG	CCCGUGGCUCUGCGAUGUUCA	Cleavage
tae-miR5049-3p	Traes_6DL_1E6A51BD3.10	PF00063		3.6.4.1		GO:GO:0016459,GO:GO:0005524,GO:GO:0003774	AT1G04600.1	myosin XI A	LOC_Os03g64290.1	myosin, putative, expressed	1,50	-1.0	1	21	1321	1341	AAUAUGGAUCGGAGGGAGUAC	AUACUCCCUCCGAUCCAUAAU	Cleavage
tae-miR1122b-3p	Traes_6DL_244C908B5.14	PF12171					AT2G02070.1	indeterminate(ID)-domain 5	LOC_Os04g47860.4	ZOS4-11 - C2H2 zinc finger protein, expressed	1,50	-1.0	1	21	584	604	AGACUUAUAUGUAGGAACGGA	UCCGUUCCUAAAUAUAAGUCU	Translation
tae-miR5384-3p	Traes_6DL_5282E0007.1	PF14541	KOG1339	3.4.23.12		GO:GO:0006508,GO:GO:0004190	AT5G10770.1	Eukaryotic aspartyl protease family protein	LOC_Os02g48870.1	aspartic proteinase nepenthesin-2 precursor, putative, expressed	2,50	-1.0	1	21	1178	1198	UGAGCGCGCCGCCGUCGAAUG	UGUUCGACGGCGGCGCGGUCG	Cleavage
tae-miR5384-3p	Traes_6DL_5282E0007.2	PF14541,PF14543	KOG1339	3.4.23.12		GO:GO:0006508,GO:GO:0004190	AT5G10770.1	Eukaryotic aspartyl protease family protein	LOC_Os02g48870.1	aspartic proteinase nepenthesin-2 precursor, putative, expressed	2,50	-1.0	1	21	1016	1036	UGAGCGCGCCGCCGUCGAAUG	UGUUCGACGGCGGCGCGGUCG	Cleavage
tae-miR1122b-3p	Traes_6DL_84E14AD8D.4	PF00067				GO:GO:0055114,GO:GO:0020037,GO:GO:0016705,GO:GO:0005506	AT1G31800.1	cytochrome P450, family 97, subfamily A, polypeptide 3	LOC_Os02g57290.4	cytochrome P450, putative, expressed	1,50	-1.0	1	21	1030	1050	AGACUUAUAUGUAGGAACGGA	UCCGUUCCUAAAUAUAAGUCU	Translation
tae-miR1122a	Traes_6DL_84E14AD8D.4	PF00067				GO:GO:0055114,GO:GO:0020037,GO:GO:0016705,GO:GO:0005506	AT1G31800.1	cytochrome P450, family 97, subfamily A, polypeptide 3	LOC_Os02g57290.4	cytochrome P450, putative, expressed	2,50	-1.0	1	20	1071	1090	UAGAUACAUCCGUAUCUAGA	ACUACAUACGGAUGUAUAUA	Cleavage
tae-miR1118	Traes_6DL_C9216369C.2	PF00891				GO:GO:0008171	AT5G54160.1	O-methyltransferase 1	LOC_Os11g20090.1	O-methyltransferase, putative, expressed	2,50	-1.0	1	23	421	443	CACUACAUUAUGGAAUGGAGGGA	UUGCUCCGUUUCAUAAUGUAGUG	Cleavage
tae-miR444b	Traes_6DL_D1C1DBD34.1	PF00319,PF01486	KOG0014			GO:GO:0046983,GO:GO:0003677,GO:GO:0006355,GO:GO:0005634,GO:GO:0003700	AT3G57230.1	AGAMOUS-like 16	LOC_Os02g49840.1	OsMADS57 - MADS-box family gene with MIKCc type-box, expressed	0,00	-1.0	1	21	274	294	UUGCUGCCUCAAGCUUGCUGC	GCAGCAAGCUUGAGGCAGCAA	Cleavage
tae-miR444a	Traes_6DL_D1C1DBD34.1	PF00319,PF01486	KOG0014			GO:GO:0046983,GO:GO:0003677,GO:GO:0006355,GO:GO:0005634,GO:GO:0003700	AT3G57230.1	AGAMOUS-like 16	LOC_Os02g49840.1	OsMADS57 - MADS-box family gene with MIKCc type-box, expressed	0,00	-1.0	1	21	274	294	UUGCUGCCUCAAGCUUGCUGC	GCAGCAAGCUUGAGGCAGCAA	Cleavage
tae-miR1117	Traes_6DL_E940A7985.1	PF00854		3.6.3.43		GO:GO:0016020,GO:GO:0006810,GO:GO:0005215	AT1G62200.1	Major facilitator superfamily protein	LOC_Os04g50940.1	peptide transporter PTR2, putative, expressed	2,50	-1.0	1	24	155	178	UAGUACCGGUUCGUGGCACGAACC	GGUUCGUGGCACAAACCGGUACUA	Cleavage
tae-miR1131	Traes_6DL_E940A7985.1	PF00854		3.6.3.43		GO:GO:0016020,GO:GO:0006810,GO:GO:0005215	AT1G62200.1	Major facilitator superfamily protein	LOC_Os04g50940.1	peptide transporter PTR2, putative, expressed	1,00	-1.0	1	22	181	202	UAGUACCGGUUCGUGGCUAACC	GAUUCGCCACGAACCGGCUACUA	Cleavage
tae-miR1122a	Traes_6DS_0D9BB546D.1	PF00300		3.1.3.3	K15634		AT3G50520.1	Phosphoglycerate mutase family protein	LOC_Os08g37140.1	phosphoglycerate mutase, putative, expressed	2,50	-1.0	1	20	111	130	UAGAUACAUCCGUAUCUAGA	ACUACAUACGGAUGUAUAUA	Cleavage
tae-miR9662a-3p	Traes_6DS_0E84AAF77.1	PF02536				GO:GO:0006355,GO:GO:0005739,GO:GO:0003690	AT5G07900.1	Mitochondrial transcription termination factor family protein	LOC_Os06g12100.1	mTERF family protein, expressed	2,50	-1.0	1	21	480	500	UUGAACAUCCCAGAGCCACCG	CCGUGGCUCUGGGAUGCUCAG	Cleavage
tae-miR9662b-3p	Traes_6DS_0E84AAF77.1	PF02536				GO:GO:0006355,GO:GO:0005739,GO:GO:0003690	AT5G07900.1	Mitochondrial transcription termination factor family protein	LOC_Os06g12100.1	mTERF family protein, expressed	2,50	-1.0	1	21	479	499	UGAACAUCCCAGAGCCACCGG	CCCGUGGCUCUGGGAUGCUCA	Cleavage
tae-miR9674b-5p	Traes_6DS_1BEF46735.1	PF13041					AT1G63130.1	Tetratricopeptide repeat (TPR)-like superfamily protein	LOC_Os10g35240.1	Rf1, mitochondrial precursor, putative, expressed	3,00	-1.0	1	21	537	557	AUAGCAUCAUCCAUCCUACCC	GGGCCGAAUGGAUGAUGCUAU	Cleavage
tae-miR1127b-3p	Traes_6DS_2207A3BC4.7	PF14438,PF06741	KOG2375				AT3G14010.4	CTC-interacting domain 4	LOC_Os02g27950.2	ataxin-2 related protein, putative, expressed	1,50	-1.0	1	21	1729	1749	ACAAGUAUUUCUGGACGGAGG	CCUCCGUCCGGAAAUACUUGU	Cleavage
tae-miR9662a-3p	Traes_6DS_2D4EFFF3E.1	PF02536				GO:GO:0006355,GO:GO:0005739,GO:GO:0003690	AT5G07900.1	Mitochondrial transcription termination factor family protein	LOC_Os06g12100.1	mTERF family protein, expressed	1,00	-1.0	1	21	369	389	UUGAACAUCCCAGAGCCACCG	CCGUGGCUCUGGGAUGUUCAG	Cleavage
tae-miR9662b-3p	Traes_6DS_2D4EFFF3E.1	PF02536				GO:GO:0006355,GO:GO:0005739,GO:GO:0003690	AT5G07900.1	Mitochondrial transcription termination factor family protein	LOC_Os06g12100.1	mTERF family protein, expressed	1,00	-1.0	1	21	368	388	UGAACAUCCCAGAGCCACCGG	CCCGUGGCUCUGGGAUGUUCA	Cleavage
tae-miR9662a-3p	Traes_6DS_3F194116E.1	PF02536				GO:GO:0006355,GO:GO:0005739,GO:GO:0003690	AT5G07900.1	Mitochondrial transcription termination factor family protein	LOC_Os06g12100.1	mTERF family protein, expressed	1,50	-1.0	1	21	525	545	UUGAACAUCCCAGAGCCACCG	CCGUGGCUCGGGGAUGUUCAA	Cleavage
tae-miR9662b-3p	Traes_6DS_3F194116E.1	PF02536				GO:GO:0006355,GO:GO:0005739,GO:GO:0003690	AT5G07900.1	Mitochondrial transcription termination factor family protein	LOC_Os06g12100.1	mTERF family protein, expressed	2,50	-1.0	1	21	524	544	UGAACAUCCCAGAGCCACCGG	CCCGUGGCUCGGGGAUGUUCA	Translation
tae-miR9662a-3p	Traes_6DS_3FC2ADA87.1	PF02536				GO:GO:0006355,GO:GO:0005739,GO:GO:0003690	AT5G07900.1	Mitochondrial transcription termination factor family protein	LOC_Os06g12100.1	mTERF family protein, expressed	3,00	-1.0	1	21	242	263	UUGAACAUCCCAGAGCCAC-CG	CGCGUGGCUCUGGGAUGUUCAG	Cleavage
tae-miR9662b-3p	Traes_6DS_3FC2ADA87.1	PF02536				GO:GO:0006355,GO:GO:0005739,GO:GO:0003690	AT5G07900.1	Mitochondrial transcription termination factor family protein	LOC_Os06g12100.1	mTERF family protein, expressed	2,00	-1.0	1	21	241	262	UGAACAUCCCAGAGCCAC-CGG	CCGCGUGGCUCUGGGAUGUUCA	Cleavage
tae-miR9662a-3p	Traes_6DS_3FC2ADA87.2	PF02536				GO:GO:0006355,GO:GO:0005739,GO:GO:0003690	AT1G21150.1	Mitochondrial transcription termination factor family protein	LOC_Os06g12100.1	mTERF family protein, expressed	3,00	-1.0	1	21	65	86	UUGAACAUCCCAGAGCCAC-CG	CGCGUGGCUCUGGGAUGUUCAG	Cleavage
tae-miR9662b-3p	Traes_6DS_3FC2ADA87.2	PF02536				GO:GO:0006355,GO:GO:0005739,GO:GO:0003690	AT1G21150.1	Mitochondrial transcription termination factor family protein	LOC_Os06g12100.1	mTERF family protein, expressed	2,00	-1.0	1	21	64	85	UGAACAUCCCAGAGCCAC-CGG	CCGCGUGGCUCUGGGAUGUUCA	Cleavage
tae-miR9662a-3p	Traes_6DS_422644406.1	PF02536				GO:GO:0006355,GO:GO:0005739,GO:GO:0003690	AT5G07900.1	Mitochondrial transcription termination factor family protein	LOC_Os06g12100.1	mTERF family protein, expressed	2,50	-1.0	1	21	468	488	UUGAACAUCCCAGAGCCACCG	CCGUGGCUCUGGAAUGUUCAG	Cleavage
tae-miR9662b-3p	Traes_6DS_422644406.1	PF02536				GO:GO:0006355,GO:GO:0005739,GO:GO:0003690	AT5G07900.1	Mitochondrial transcription termination factor family protein	LOC_Os06g12100.1	mTERF family protein, expressed	2,50	-1.0	1	21	467	487	UGAACAUCCCAGAGCCACCGG	CCCGUGGCUCUGGAAUGUUCA	Cleavage
tae-miR9662a-3p	Traes_6DS_422644406.2	PF02536				GO:GO:0006355,GO:GO:0005739,GO:GO:0003690	AT5G07900.1	Mitochondrial transcription termination factor family protein	LOC_Os06g12100.1	mTERF family protein, expressed	2,50	-1.0	1	21	66	86	UUGAACAUCCCAGAGCCACCG	CCGUGGCUCUGGAAUGUUCAG	Cleavage
tae-miR9662b-3p	Traes_6DS_422644406.2	PF02536				GO:GO:0006355,GO:GO:0005739,GO:GO:0003690	AT5G07900.1	Mitochondrial transcription termination factor family protein	LOC_Os06g12100.1	mTERF family protein, expressed	2,50	-1.0	1	21	65	85	UGAACAUCCCAGAGCCACCGG	CCCGUGGCUCUGGAAUGUUCA	Cleavage
tae-miR9662a-3p	Traes_6DS_422644406.3	PF02536				GO:GO:0006355,GO:GO:0005739,GO:GO:0003690	AT5G07900.1	Mitochondrial transcription termination factor family protein	LOC_Os06g12100.1	mTERF family protein, expressed	2,50	-1.0	1	21	66	86	UUGAACAUCCCAGAGCCACCG	CCGUGGCUCUGGAAUGUUCAG	Cleavage
tae-miR9662b-3p	Traes_6DS_422644406.3	PF02536				GO:GO:0006355,GO:GO:0005739,GO:GO:0003690	AT5G07900.1	Mitochondrial transcription termination factor family protein	LOC_Os06g12100.1	mTERF family protein, expressed	2,50	-1.0	1	21	65	85	UGAACAUCCCAGAGCCACCGG	CCCGUGGCUCUGGAAUGUUCA	Cleavage
tae-miR9662a-3p	Traes_6DS_422644406.4	PF02536				GO:GO:0006355,GO:GO:0005739,GO:GO:0003690	AT5G07900.1	Mitochondrial transcription termination factor family protein	LOC_Os06g12100.1	mTERF family protein, expressed	2,50	-1.0	1	21	66	86	UUGAACAUCCCAGAGCCACCG	CCGUGGCUCUGGAAUGUUCAG	Cleavage
tae-miR9662b-3p	Traes_6DS_422644406.4	PF02536				GO:GO:0006355,GO:GO:0005739,GO:GO:0003690	AT5G07900.1	Mitochondrial transcription termination factor family protein	LOC_Os06g12100.1	mTERF family protein, expressed	2,50	-1.0	1	21	65	85	UGAACAUCCCAGAGCCACCGG	CCCGUGGCUCUGGAAUGUUCA	Cleavage
tae-miR156	Traes_6DS_7B1596298.1	PF03110				GO:GO:0005634,GO:GO:0003677	AT5G43270.2	squamosa promoter binding protein-like 2	LOC_Os02g04680.2	OsSPL3 - SBP-box gene family member, expressed	1,00	-1.0	1	21	1150	1170	UGACAGAAGAGAGUGAGCACA	CAUGCUCUCUCUCUUCUGUCA	Cleavage
tae-miR156	Traes_6DS_7B1596298.2	PF03110				GO:GO:0005634,GO:GO:0003677	AT5G43270.1	squamosa promoter binding protein-like 2	LOC_Os02g04680.2	OsSPL3 - SBP-box gene family member, expressed	1,00	-1.0	1	21	1150	1170	UGACAGAAGAGAGUGAGCACA	CAUGCUCUCUCUCUUCUGUCA	Cleavage
tae-miR156	Traes_6DS_7B1596298.4								LOC_Os02g04680.1	OsSPL3 - SBP-box gene family member, expressed	1,00	-1.0	1	21	271	291	UGACAGAAGAGAGUGAGCACA	CAUGCUCUCUCUCUUCUGUCA	Cleavage
tae-miR9674b-5p	Traes_6DS_7C389C178.1	PF12854,PF13041					AT1G62670.1	rna processing factor 2	LOC_Os10g35240.2	Rf1, mitochondrial precursor, putative, expressed	3,00	-1.0	1	21	537	557	AUAGCAUCAUCCAUCCUACCC	GGGCCGAAUGGAUGAUGCUAU	Cleavage
tae-miR9674a-5p	Traes_6DS_7C389C178.1	PF12854,PF13041					AT1G62670.1	rna processing factor 2	LOC_Os10g35240.2	Rf1, mitochondrial precursor, putative, expressed	2,50	-1.0	1	21	954	974	GCAUCAUCCAUCCUACCAUUC	GAAUGGAAGGAUAGAUGAUGC	Cleavage
tae-miR9674a-5p	Traes_6DS_7C389C178.2	PF12854					AT1G62670.1	rna processing factor 2	LOC_Os10g35436.1	Rf1, mitochondrial precursor, putative, expressed	2,50	-1.0	1	21	51	71	GCAUCAUCCAUCCUACCAUUC	GAAUGGAAGGAUAGAUGAUGC	Cleavage
tae-miR9674b-5p	Traes_6DS_7C389C178.3	PF12854,PF13041					AT1G62670.1	rna processing factor 2	LOC_Os10g35240.1	Rf1, mitochondrial precursor, putative, expressed	3,00	-1.0	1	21	537	557	AUAGCAUCAUCCAUCCUACCC	GGGCCGAAUGGAUGAUGCUAU	Cleavage
tae-miR9674a-5p	Traes_6DS_7C389C178.3	PF12854,PF13041					AT1G62670.1	rna processing factor 2	LOC_Os10g35240.1	Rf1, mitochondrial precursor, putative, expressed	2,50	-1.0	1	21	954	974	GCAUCAUCCAUCCUACCAUUC	GAAUGGAAGGAUAGAUGAUGC	Cleavage
tae-miR9662a-3p	Traes_6DS_7D3BEF6A9.1	PF02536				GO:GO:0006355,GO:GO:0005739,GO:GO:0003690	AT1G21150.1	Mitochondrial transcription termination factor family protein	LOC_Os06g12100.1	mTERF family protein, expressed	2,50	-1.0	1	21	33	53	UUGAACAUCCCAGAGCCACCG	UCGUGGCUCGGGGAUGUUCAG	Cleavage
tae-miR9662b-3p	Traes_6DS_7D3BEF6A9.1	PF02536				GO:GO:0006355,GO:GO:0005739,GO:GO:0003690	AT1G21150.1	Mitochondrial transcription termination factor family protein	LOC_Os06g12100.1	mTERF family protein, expressed	2,50	-1.0	1	21	32	52	UGAACAUCCCAGAGCCACCGG	CUCGUGGCUCGGGGAUGUUCA	Translation
tae-miR9662a-3p	Traes_6DS_7D3BEF6A9.2	PF02536				GO:GO:0006355,GO:GO:0005739,GO:GO:0003690	AT1G21150.1	Mitochondrial transcription termination factor family protein	LOC_Os06g12100.1	mTERF family protein, expressed	2,50	-1.0	1	21	246	266	UUGAACAUCCCAGAGCCACCG	UCGUGGCUCGGGGAUGUUCAG	Cleavage
tae-miR9662b-3p	Traes_6DS_7D3BEF6A9.2	PF02536				GO:GO:0006355,GO:GO:0005739,GO:GO:0003690	AT1G21150.1	Mitochondrial transcription termination factor family protein	LOC_Os06g12100.1	mTERF family protein, expressed	2,50	-1.0	1	21	245	265	UGAACAUCCCAGAGCCACCGG	CUCGUGGCUCGGGGAUGUUCA	Translation
tae-miR9662a-3p	Traes_6DS_7D3BEF6A9.3	PF02536				GO:GO:0006355,GO:GO:0005739,GO:GO:0003690	AT1G21150.1	Mitochondrial transcription termination factor family protein	LOC_Os06g12100.1	mTERF family protein, expressed	2,50	-1.0	1	21	246	266	UUGAACAUCCCAGAGCCACCG	UCGUGGCUCGGGGAUGUUCAG	Cleavage
tae-miR9662b-3p	Traes_6DS_7D3BEF6A9.3	PF02536				GO:GO:0006355,GO:GO:0005739,GO:GO:0003690	AT1G21150.1	Mitochondrial transcription termination factor family protein	LOC_Os06g12100.1	mTERF family protein, expressed	2,50	-1.0	1	21	245	265	UGAACAUCCCAGAGCCACCGG	CUCGUGGCUCGGGGAUGUUCA	Translation
tae-miR9662a-3p	Traes_6DS_893F318A9.1	PF02536				GO:GO:0006355,GO:GO:0005739,GO:GO:0003690	AT5G07900.1	Mitochondrial transcription termination factor family protein	LOC_Os06g12100.1	mTERF family protein, expressed	1,50	-1.0	1	21	549	569	UUGAACAUCCCAGAGCCACCG	CCGUGGCUCGGGGAUGUUCAA	Cleavage
tae-miR9662b-3p	Traes_6DS_893F318A9.1	PF02536				GO:GO:0006355,GO:GO:0005739,GO:GO:0003690	AT5G07900.1	Mitochondrial transcription termination factor family protein	LOC_Os06g12100.1	mTERF family protein, expressed	2,50	-1.0	1	21	548	568	UGAACAUCCCAGAGCCACCGG	CCCGUGGCUCGGGGAUGUUCA	Translation
tae-miR9662a-3p	Traes_6DS_893F318A9.2	PF02536				GO:GO:0006355,GO:GO:0005739,GO:GO:0003690	AT5G07900.1	Mitochondrial transcription termination factor family protein	LOC_Os06g12100.1	mTERF family protein, expressed	1,50	-1.0	1	21	132	152	UUGAACAUCCCAGAGCCACCG	CCGUGGCUCGGGGAUGUUCAA	Cleavage
tae-miR9662b-3p	Traes_6DS_893F318A9.2	PF02536				GO:GO:0006355,GO:GO:0005739,GO:GO:0003690	AT5G07900.1	Mitochondrial transcription termination factor family protein	LOC_Os06g12100.1	mTERF family protein, expressed	2,50	-1.0	1	21	131	151	UGAACAUCCCAGAGCCACCGG	CCCGUGGCUCGGGGAUGUUCA	Translation
tae-miR9662a-3p	Traes_6DS_931C28AF6.1	PF02536				GO:GO:0006355,GO:GO:0005739,GO:GO:0003690	AT1G79220.1	Mitochondrial transcription termination factor family protein	LOC_Os06g12100.1	mTERF family protein, expressed	2,50	-1.0	1	21	288	308	UUGAACAUCCCAGAGCCACCG	AUGUGGCUCUCGGAUGUUCAG	Translation
tae-miR9662a-3p	Traes_6DS_931C28AF6.2	PF02536				GO:GO:0006355,GO:GO:0005739,GO:GO:0003690	AT1G79220.1	Mitochondrial transcription termination factor family protein	LOC_Os06g12100.1	mTERF family protein, expressed	2,50	-1.0	1	21	276	296	UUGAACAUCCCAGAGCCACCG	AUGUGGCUCUCGGAUGUUCAG	Translation
tae-miR9662a-3p	Traes_6DS_931C28AF6.3	PF02536				GO:GO:0006355,GO:GO:0005739,GO:GO:0003690	AT1G79220.1	Mitochondrial transcription termination factor family protein	LOC_Os06g12100.1	mTERF family protein, expressed	2,50	-1.0	1	21	273	293	UUGAACAUCCCAGAGCCACCG	AUGUGGCUCUCGGAUGUUCAG	Translation
tae-miR9662a-3p	Traes_6DS_931C28AF6.4	PF02536				GO:GO:0006355,GO:GO:0005739,GO:GO:0003690	AT1G79220.1	Mitochondrial transcription termination factor family protein	LOC_Os06g12100.1	mTERF family protein, expressed	2,50	-1.0	1	21	273	293	UUGAACAUCCCAGAGCCACCG	AUGUGGCUCUCGGAUGUUCAG	Translation
tae-miR9662a-3p	Traes_6DS_931C28AF6.5	PF02536				GO:GO:0006355,GO:GO:0005739,GO:GO:0003690	AT1G79220.1	Mitochondrial transcription termination factor family protein	LOC_Os06g12100.1	mTERF family protein, expressed	2,50	-1.0	1	21	276	296	UUGAACAUCCCAGAGCCACCG	AUGUGGCUCUCGGAUGUUCAG	Translation
tae-miR9662b-3p	Traes_6DS_93C7DA226.1	PF02536				GO:GO:0006355,GO:GO:0005739,GO:GO:0003690	AT5G07900.1	Mitochondrial transcription termination factor family protein	LOC_Os06g12100.1	mTERF family protein, expressed	3,00	-1.0	1	21	503	523	UGAACAUCCCAGAGCCACCGG	CCCGUGCUUCUGGGAUGUUCA	Cleavage
tae-miR9662b-3p	Traes_6DS_93C7DA2261.1	PF02536				GO:GO:0006355,GO:GO:0005739,GO:GO:0003690	AT5G07900.1	Mitochondrial transcription termination factor family protein	LOC_Os06g12100.1	mTERF family protein, expressed	3,00	-1.0	1	21	503	523	UGAACAUCCCAGAGCCACCGG	CCCGUGCUUCUGGGAUGUUCA	Cleavage
tae-miR9662a-3p	Traes_6DS_A490DF33E.1	PF02536				GO:GO:0006355,GO:GO:0005739,GO:GO:0003690	AT5G07900.1	Mitochondrial transcription termination factor family protein	LOC_Os06g12100.1	mTERF family protein, expressed	2,50	-1.0	1	21	471	491	UUGAACAUCCCAGAGCCACCG	CCGUGGCUCUGGAAUGUUCAG	Cleavage
tae-miR9662b-3p	Traes_6DS_A490DF33E.1	PF02536				GO:GO:0006355,GO:GO:0005739,GO:GO:0003690	AT5G07900.1	Mitochondrial transcription termination factor family protein	LOC_Os06g12100.1	mTERF family protein, expressed	2,50	-1.0	1	21	470	490	UGAACAUCCCAGAGCCACCGG	CCCGUGGCUCUGGAAUGUUCA	Cleavage

tae-miR9662a-3p	Traes_6DS_A490DF33E.2	PF02536				GO:GO:0006355,GO:GO:0005739,GO:GO:0003690	AT1G21150.1	Mitochondrial transcription termination factor family protein	LOC_Os06g12100.1	mTERF family protein, expressed	2,50	-1.0	1	21	66	86	UUGAACAUCCCAGAGCCACCG	CCGUGGCUCUGGAAUGUUCAG	Cleavage
tae-miR9662b-3p	Traes_6DS_A490DF33E.2	PF02536				GO:GO:0006355,GO:GO:0005739,GO:GO:0003690	AT1G21150.1	Mitochondrial transcription termination factor family protein	LOC_Os06g12100.1	mTERF family protein, expressed	2,50	-1.0	1	21	65	85	UGAACAUCCCAGAGCCACCGG	CCCGUGGCUCUGGAAUGUUCA	Cleavage
tae-miR1122b-3p	Traes_6DS_C1B7F14A41.2	PF08263,PF07714,PF13855	KOG1187	2.7.11.1		GO:GO:0006468,GO:GO:0004672,GO:GO:0005515	AT1G72300.1	Leucine-rich receptor-like protein kinase family protein	LOC_Os02g05930.1	phytosulfokine receptor precursor, putative, expressed	1,50	-1.0	1	21	3044	3064	AGACUUAUAUGUAGGAACGGA	UCCGUUCCUAAAUAUAAGUCU	Translation
tae-miR156	Traes_6DS_C4D93239C.1	PF03110				GO:GO:0005634,GO:GO:0003677	AT5G43270.1	squamosa promoter binding protein-like 2	LOC_Os06g45310.1	OsSPL11 - SBP-box gene family member, expressed	1,00	-1.0	1	21	577	597	UGACAGAAGAGAGUGAGCACA	CAUGCUCUCUCUCUUCUGUCA	Cleavage
tae-miR156	Traes_6DS_C4D93239C.2								LOC_Os06g45310.1	OsSPL11 - SBP-box gene family member, expressed	1,00	-1.0	1	21	88	108	UGACAGAAGAGAGUGAGCACA	CAUGCUCUCUCUCUUCUGUCA	Cleavage
tae-miR156	Traes_6DS_C4D93239C.3								LOC_Os06g45310.1	OsSPL11 - SBP-box gene family member, expressed	1,00	-1.0	1	21	64	84	UGACAGAAGAGAGUGAGCACA	CAUGCUCUCUCUCUUCUGUCA	Cleavage
tae-miR9662a-3p	Traes_6DS_CF627A1BC.1								LOC_Os06g12100.1	mTERF family protein, expressed	2,50	-1.0	1	21	75	95	UUGAACAUCCCAGAGCCACCG	CCGUGGCUCGGGGAUGUUCAG	Cleavage
tae-miR9662b-3p	Traes_6DS_CF627A1BC.1								LOC_Os06g12100.1	mTERF family protein, expressed	2,50	-1.0	1	21	74	94	UGAACAUCCCAGAGCCACCGG	CCCGUGGCUCGGGGAUGUUCA	Translation
tae-miR9662a-3p	Traes_6DS_DAD8BEF41.1	PF02536				GO:GO:0006355,GO:GO:0005739,GO:GO:0003690	AT5G07900.1	Mitochondrial transcription termination factor family protein	LOC_Os06g12100.1	mTERF family protein, expressed	2,50	-1.0	1	21	477	497	UUGAACAUCCCAGAGCCACCG	CCGUGGCUCUGGAAUGUUCAG	Cleavage
tae-miR9662b-3p	Traes_6DS_DAD8BEF41.1	PF02536				GO:GO:0006355,GO:GO:0005739,GO:GO:0003690	AT5G07900.1	Mitochondrial transcription termination factor family protein	LOC_Os06g12100.1	mTERF family protein, expressed	2,50	-1.0	1	21	476	496	UGAACAUCCCAGAGCCACCGG	CCCGUGGCUCUGGAAUGUUCA	Cleavage
tae-miR9662a-3p	Traes_6DS_E352D66A4.1	PF02536				GO:GO:0006355,GO:GO:0005739,GO:GO:0003690	AT5G64950.1	Mitochondrial transcription termination factor family protein	LOC_Os06g12100.1	mTERF family protein, expressed	2,50	-1.0	1	21	450	470	UUGAACAUCCCAGAGCCACCG	CCGUGGCUCUGGGAUGUUUAG	Cleavage
tae-miR9662b-3p	Traes_6DS_E352D66A4.1	PF02536				GO:GO:0006355,GO:GO:0005739,GO:GO:0003690	AT5G64950.1	Mitochondrial transcription termination factor family protein	LOC_Os06g12100.1	mTERF family protein, expressed	2,50	-1.0	1	21	449	469	UGAACAUCCCAGAGCCACCGG	CCCGUGGCUCUGGGAUGUUUA	Cleavage
tae-miR9662a-3p	Traes_6DS_E95930B7D.1	PF02536				GO:GO:0006355,GO:GO:0005739,GO:GO:0003690	AT5G64950.1	Mitochondrial transcription termination factor family protein	LOC_Os06g12100.1	mTERF family protein, expressed	2,50	-1.0	1	21	450	470	UUGAACAUCCCAGAGCCACCG	CCGUGGCUCUGGGAUGUUUAG	Cleavage
tae-miR9662b-3p	Traes_6DS_E95930B7D.1	PF02536				GO:GO:0006355,GO:GO:0005739,GO:GO:0003690	AT5G64950.1	Mitochondrial transcription termination factor family protein	LOC_Os06g12100.1	mTERF family protein, expressed	2,50	-1.0	1	21	449	469	UGAACAUCCCAGAGCCACCGG	CCCGUGGCUCUGGGAUGUUUA	Cleavage
tae-miR9662a-3p	Traes_6DS_EF75D5C67.1	PF02536				GO:GO:0006355,GO:GO:0005739,GO:GO:0003690	AT5G07900.1	Mitochondrial transcription termination factor family protein	LOC_Os06g12100.1	mTERF family protein, expressed	2,00	-1.0	1	21	66	86	UUGAACAUCCCAGAGCCACCG	UCGUGGCGCUGGGAUGUUCAG	Cleavage
tae-miR9662b-3p	Traes_6DS_EF75D5C67.1	PF02536				GO:GO:0006355,GO:GO:0005739,GO:GO:0003690	AT5G07900.1	Mitochondrial transcription termination factor family protein	LOC_Os06g12100.1	mTERF family protein, expressed	2,50	-1.0	1	21	65	85	UGAACAUCCCAGAGCCACCGG	CUCGUGGCGCUGGGAUGUUCA	Cleavage
tae-miR9662a-3p	Traes_6DS_F6C7E588F.1	PF02536	KOG1267			GO:GO:0006355,GO:GO:0005739,GO:GO:0003690	AT5G07900.1	Mitochondrial transcription termination factor family protein	LOC_Os06g12100.1	mTERF family protein, expressed	2,00	-1.0	1	21	657	677	UUGAACAUCCCAGAGCCACCG	CCGUGGUUCUGGAUGUUCAG	Cleavage
tae-miR9662b-3p	Traes_6DS_F6C7E588F.1	PF02536	KOG1267			GO:GO:0006355,GO:GO:0005739,GO:GO:0003690	AT5G07900.1	Mitochondrial transcription termination factor family protein	LOC_Os06g12100.1	mTERF family protein, expressed	2,00	-1.0	1	21	656	676	UGAACAUCCCAGAGCCACCGG	CCCGUGGUUCUGGGAUGUUCA	Cleavage
tae-miR9662a-3p	Traes_6DS_FB434BB3C.1	PF02536				GO:GO:0006355,GO:GO:0005739,GO:GO:0003690	AT5G64950.1	Mitochondrial transcription termination factor family protein	LOC_Os06g12100.1	mTERF family protein, expressed	2,50	-1.0	1	21	246	266	UUGAACAUCCCAGAGCCACCG	CCGUGGCUCUCGGAUGUUCAG	Translation
tae-miR9662b-3p	Traes_6DS_FB434BB3C.1	PF02536				GO:GO:0006355,GO:GO:0005739,GO:GO:0003690	AT5G64950.1	Mitochondrial transcription termination factor family protein	LOC_Os06g12100.1	mTERF family protein, expressed	2,50	-1.0	1	21	245	265	UGAACAUCCCAGAGCCACCGG	CCCGUGGCUCUCGGAUGUUCA	Translation
tae-miR9662a-3p	Traes_6DS_FB434BB3C.2	PF02536				GO:GO:0006355,GO:GO:0005739,GO:GO:0003690	AT5G64950.1	Mitochondrial transcription termination factor family protein	LOC_Os06g12100.1	mTERF family protein, expressed	2,50	-1.0	1	21	246	266	UUGAACAUCCCAGAGCCACCG	CCGUGGCUCUCGGAUGUUCAG	Translation
tae-miR9662b-3p	Traes_6DS_FB434BB3C.2	PF02536				GO:GO:0006355,GO:GO:0005739,GO:GO:0003690	AT5G64950.1	Mitochondrial transcription termination factor family protein	LOC_Os06g12100.1	mTERF family protein, expressed	2,50	-1.0	1	21	245	265	UGAACAUCCCAGAGCCACCGG	CCCGUGGCUCUCGGAUGUUCA	Translation
tae-miR1122a	Traes_7AL_135129885.3	PF01138	KOG1612				AT3G07750.1	3\'-5\'-exoribonuclease family protein	LOC_Os12g21500.1	exosome complex exonuclease, putative, expressed	2,50	-1.0	1	20	721	740	UAGAUACAUCCGUAUCUAGA	ACUACAUACGGAUGUAUAUA	Cleavage
tae-miR164	Traes_7AL_628A69311.1	PF02365				GO:GO:0006355,GO:GO:0003677	AT1G56010.2	NAC domain containing protein 1	LOC_Os06g46270.1	no apical meristem protein, putative, expressed	1,00	-1.0	1	21	650	670	UGGAGAAGCAGGGCACGUGCA	AGCAAGUGCCCUGCUUCUCCA	Cleavage
tae-miR1130b-3p	Traes_7AL_955F54182.1	PF02705				GO:GO:0071805,GO:GO:0016020,GO:GO:0015079	AT2G30070.1	potassium transporter 1	LOC_Os06g45940.2	potassium transporter, putative, expressed	0,00	-1.0	1	21	1141	1161	UCUUAUAUUAUGGGACGGAGG	CCUCCGUCCCAUAAUAUAAGA	Cleavage
tae-miR1122c-3p	Traes_7AL_955F54182.1	PF02705				GO:GO:0071805,GO:GO:0016020,GO:GO:0015079	AT2G30070.1	potassium transporter 1	LOC_Os06g45940.2	potassium transporter, putative, expressed	1,50	-1.0	1	21	1141	1161	UCUAAUAUUAUGGGACGGAGG	CCUCCGUCCCAUAAUAUAAGA	Cleavage
tae-miR1133	Traes_7AL_ADBB1224B.1	PF08315					AT3G05070.1		LOC_Os06g51420.1	coiled-coil domain-containing protein 12, putative, expressed	2,50	-1.0	1	22	52	73	CAUAUACUCCCUCCGUCCGAAA	UUCGGACGGAGGGAGUAUAAU	Cleavage
tae-miR164	Traes_7AL_BE3253C8F.1	PF02365				GO:GO:0006355,GO:GO:0003677	AT1G56010.2	NAC domain containing protein 1	LOC_Os06g46270.1	no apical meristem protein, putative, expressed	1,00	-1.0	1	21	653	673	UGGAGAAGCAGGGCACGUGCA	AGCAAGUGCCCUGCUUCUCCA	Cleavage
tae-miR164	Traes_7AL_BE3253C8F.2						AT1G56010.2	NAC domain containing protein 1	LOC_Os06g46270.1	no apical meristem protein, putative, expressed	1,00	-1.0	1	21	215	235	UGGAGAAGCAGGGCACGUGCA	AGCAAGUGCCCUGCUUCUCCA	Cleavage
tae-miR160	Traes_7AL_E3ADC8C38.1	PF06507				GO:GO:0009725,GO:GO:0006355,GO:GO:0005634,GO:GO:0003677	AT2G28350.1	auxin response factor 10	LOC_Os06g47150.2	auxin response factor 18, putative, expressed	0,00	-1.0	1	21	399	419	UGCCUGGCUCCCUGUAUGCCA	AGGCAUACAGGGAGCCAGGCA	Cleavage
tae-miR9666a-3p	Traes_7AL_EE3CD1CF3.10										2,00	-1.0	1	22	229	250	CGGUAGGGCUGUAUGAUGGCGA	UCCCCGUCAUACAGCCCUACCU	Cleavage
tae-miR9666a-3p	Traes_7AL_EE3CD1CF3.2										2,00	-1.0	1	22	5032	5053	CGGUAGGGCUGUAUGAUGGCGA	UCCCCGUCAUACAGCCCUACCU	Cleavage
tae-miR9666a-3p	Traes_7AL_EE3CD1CF3.3										2,00	-1.0	1	22	4948	4969	CGGUAGGGCUGUAUGAUGGCGA	UCCCCGUCAUACAGCCCUACCU	Cleavage
tae-miR9666a-3p	Traes_7AL_EE3CD1CF3.4										2,00	-1.0	1	22	4801	4822	CGGUAGGGCUGUAUGAUGGCGA	UCCCCGUCAUACAGCCCUACCU	Cleavage
tae-miR9666a-3p	Traes_7AL_EE3CD1CF3.5										2,00	-1.0	1	22	4393	4414	CGGUAGGGCUGUAUGAUGGCGA	UCCCCGUCAUACAGCCCUACCU	Cleavage
tae-miR9666a-3p	Traes_7AL_EE3CD1CF3.6										2,00	-1.0	1	22	3409	3430	CGGUAGGGCUGUAUGAUGGCGA	UCCCCGUCAUACAGCCCUACCU	Cleavage
tae-miR9666a-3p	Traes_7AL_EE3CD1CF3.8										2,00	-1.0	1	22	2941	2962	CGGUAGGGCUGUAUGAUGGCGA	UCCCCGUCAUACAGCCCUACCU	Cleavage
tae-miR1137b-5p	Traes_7AS_0F0291904.1	PF00657		3.1.1.1		GO:GO:0016788	AT5G45910.1	GDSL-like Lipase/Acylhydrolase superfamily protein	LOC_Os01g12320.1	GDSL-like lipase/acylhydrolase, putative, expressed	2,50	-1.0	1	21	844	864	UCCGUUCCAGAAUAGAUGACC	ACUCAUCUAUUCUGGGACGGU	Cleavage
tae-miR1137b-5p	Traes_7AS_0F0291904.2	PF00657		3.1.1.1		GO:GO:0016788	AT2G27360.1	GDSL-like Lipase/Acylhydrolase superfamily protein	LOC_Os01g12320.2	GDSL-like lipase/acylhydrolase, putative, expressed	2,50	-1.0	1	21	451	471	UCCGUUCCAGAAUAGAUGACC	ACUCAUCUAUUCUGGGACGGU	Cleavage
tae-miR1137b-5p	Traes_7AS_23DFA827E.1	PF00400	KOG1188			GO:GO:0005515	AT2G47790.1	Transducin/WD40 repeat-like superfamily protein	LOC_Os12g19590.3	WD domain, G-beta repeat domain containing protein, expressed	1,50	-1.0	1	21	11	31	UCCGUUCCAGAAUAGAUGACC	AGUCAUCUAUUUUGGAACGGA	Translation
tae-miR1122a	Traes_7AS_9674F6981.1	PF01031		3.6.5.5		GO:GO:0005525	AT1G10290.1	dynamin-like protein 6	LOC_Os06g13820.1	dynamin, putative, expressed	1,50	-1.0	1	20	1467	1486	UAGAUACAUCCGUAUCUAGA	UCUAGAUACAGAUGUAUCUA	Translation
tae-miR1122a	Traes_7AS_9674F6981.2	PF01031		3.6.5.5		GO:GO:0005525	AT1G10290.1	dynamin-like protein 6	LOC_Os06g13820.1	dynamin, putative, expressed	1,50	-1.0	1	20	1473	1492	UAGAUACAUCCGUAUCUAGA	UCUAGAUACAGAUGUAUCUA	Translation
tae-miR1122a	Traes_7AS_9674F6981.3	PF01031		3.6.5.5		GO:GO:0005525	AT1G10290.1	dynamin-like protein 6	LOC_Os06g13820.1	dynamin, putative, expressed	1,50	-1.0	1	20	1467	1486	UAGAUACAUCCGUAUCUAGA	UCUAGAUACAGAUGUAUCUA	Translation
tae-miR1130b-3p	Traes_7AS_9819F7C48.1	PF01154,PF08540		2.3.3.10		GO:GO:0008299,GO:GO:0004421	AT4G11820.2	hydroxymethylglutaryl-CoA synthase / HMG-CoA synthase / 3-hydroxy-3-methylglutaryl coenzyme A synthase	LOC_Os08g43170.1	hydroxymethylglutaryl-CoA synthase, putative, expressed	0,00	-1.0	1	21	1268	1288	UCUUAUAUUAUGGGACGGAGG	AAUCCGUCCCAUAAUAUAAGA	Cleavage
tae-miR1122c-3p	Traes_7AS_9819F7C48.1	PF01154,PF08540		2.3.3.10		GO:GO:0008299,GO:GO:0004421	AT4G11820.2	hydroxymethylglutaryl-CoA synthase / HMG-CoA synthase / 3-hydroxy-3-methylglutaryl coenzyme A synthase	LOC_Os08g43170.1	hydroxymethylglutaryl-CoA synthase, putative, expressed	1,50	-1.0	1	21	1268	1288	UCUAAUAUUAUGGGACGGAGG	AAUCCGUCCCAUAAUAUAAGA	Cleavage
tae-miR1120c-5p	Traes_7AS_9819F7C48.1	PF01154,PF08540		2.3.3.10		GO:GO:0008299,GO:GO:0004421	AT4G11820.2	hydroxymethylglutaryl-CoA synthase / HMG-CoA synthase / 3-hydroxy-3-methylglutaryl coenzyme A synthase	LOC_Os08g43170.1	hydroxymethylglutaryl-CoA synthase, putative, expressed	1,50	-1.0	1	21	1311	1331	UAAUAUAAGAACGUUUUUGAC	GUCAAAAACGCUCUUAUAUUA	Translation
tae-miR5086	Traes_7AS_9E1AC3FE6.1										3,00	-1.0	1	21	324	344	ACAUUGGUGGAAGGCGUGGUA	CGCCAUGGCUUCCACCAAUGG	Cleavage
tae-miR7757-5p	Traes_7AS_E43D64A80.1	PF00931	KOG4658			GO:GO:0043531	AT3G46530.1	NB-ARC domain-containing disease resistance protein	LOC_Os07g26430.1	resistance protein, putative, expressed	2,50	-1.0	1	22	377	398	AUAAAACCUUCAGCUAUCCAUC	AAUGGAUAACUGAAGAUUUUAU	Cleavage
tae-miR7757-5p	Traes_7AS_E43D64A80.2	PF00931	KOG4658			GO:GO:0043531	AT3G46530.1	NB-ARC domain-containing disease resistance protein	LOC_Os07g26430.1	resistance protein, putative, expressed	2,50	-1.0	1	22	1424	1445	AUAAAACCUUCAGCUAUCCAUC	AAUGGAUAACUGAAGAUUUUAU	Cleavage
tae-miR156	Traes_7AS_FE67F9E86.1								LOC_Os08g41940.1	OsSPL16 - SBP-box gene family member, expressed	1,00	-1.0	1	21	358	378	UGACAGAAGAGAGUGAGCACA	UGUGCUCUCUCUCUUCUGUCA	Cleavage
tae-miR156	Traes_7AS_FE67F9E86.2								LOC_Os08g41940.1	OsSPL16 - SBP-box gene family member, expressed	1,00	-1.0	1	21	358	378	UGACAGAAGAGAGUGAGCACA	UGUGCUCUCUCUCUUCUGUCA	Cleavage
tae-miR156	Traes_7AS_FE67F9E86.3								LOC_Os08g41940.1	OsSPL16 - SBP-box gene family member, expressed	1,00	-1.0	1	21	331	351	UGACAGAAGAGAGUGAGCACA	UGUGCUCUCUCUCUUCUGUCA	Cleavage
tae-miR9654a-3p	Traes_7BL_03C3797A2.1	PF00635,PF00400,PF00069	KOG1187			GO:GO:0005515,GO:GO:0006468,GO:GO:0005524,GO:GO:0004672	AT1G79990.3	structural molecules	LOC_Os11g10640.1	protein kinase domain containing protein, expressed	2,00	-1.0	1	22	1813	1834	UUCUGAAAGGCUUGAAGCGAAU	GAGGGCCUCAAGCCUUUCAGAA	Cleavage
tae-miR9654a-3p	Traes_7BL_03C3797A2.2	PF00635,PF00400,PF00069	KOG1187			GO:GO:0005515,GO:GO:0006468,GO:GO:0005524,GO:GO:0004672	AT1G79990.3	structural molecules	LOC_Os11g10640.1	protein kinase domain containing protein, expressed	2,00	-1.0	1	22	1813	1834	UUCUGAAAGGCUUGAAGCGAAU	GAGGGCCUCAAGCCUUUCAGAA	Cleavage
tae-miR9654a-3p	Traes_7BL_03C3797A2.3	PF00635,PF00400,PF00069	KOG1187			GO:GO:0005515,GO:GO:0006468,GO:GO:0005524,GO:GO:0004672	AT1G79990.3	structural molecules	LOC_Os11g10640.1	protein kinase domain containing protein, expressed	2,00	-1.0	1	22	1513	1534	UUCUGAAAGGCUUGAAGCGAAU	GAGGGCCUCAAGCCUUUCAGAA	Cleavage
tae-miR9654a-3p	Traes_7BL_03C3797A2.4	PF00635,PF00400,PF00069	KOG1187			GO:GO:0005515,GO:GO:0006468,GO:GO:0005524,GO:GO:0004672	AT1G79990.3	structural molecules	LOC_Os11g10640.1	protein kinase domain containing protein, expressed	2,00	-1.0	1	22	1513	1534	UUCUGAAAGGCUUGAAGCGAAU	GAGGGCCUCAAGCCUUUCAGAA	Cleavage
tae-miR9654a-3p	Traes_7BL_03C3797A2.5	PF00635,PF00400,PF00069	KOG0294			GO:GO:0005515,GO:GO:0006468,GO:GO:0005524,GO:GO:0004672	AT1G79990.3	structural molecules	LOC_Os11g10640.1	protein kinase domain containing protein, expressed	2,00	-1.0	1	22	1399	1420	UUCUGAAAGGCUUGAAGCGAAU	GAGGGCCUCAAGCCUUUCAGAA	Cleavage
tae-miR9654a-3p	Traes_7BL_03C3797A2.6	PF00635,PF00400	KOG0294			GO:GO:0005515	AT1G79990.3	structural molecules	LOC_Os02g11830.2	coatomer subunit beta, putative, expressed	2,00	-1.0	1	22	973	994	UUCUGAAAGGCUUGAAGCGAAU	GAGGGCCUCAAGCCUUUCAGAA	Cleavage
tae-miR9654a-3p	Traes_7BL_03C3797A2.7	PF00635,PF00069	KOG1187	2.7.11.1		GO:GO:0006468,GO:GO:0005524,GO:GO:0004672	AT4G21410.1	cysteine-rich RLK (RECEPTOR-like protein kinase) 29	LOC_Os04g30030.1	cysteine-rich receptor-like protein kinase 12 precursor, putative, expressed	2,00	-1.0	1	22	1813	1834	UUCUGAAAGGCUUGAAGCGAAU	GAGGGCCUCAAGCCUUUCAGAA	Cleavage
tae-miR1130b-3p	Traes_7BL_0665E8707.6										1,00	-1.0	1	21	46	66	UCUUAUAUUAUGGGACGGAGG	CCUCCGUCCCAUAAUAUAAGU	Cleavage
tae-miR1122c-3p	Traes_7BL_0665E8707.6										2,50	-1.0	1	21	46	66	UCUAAUAUUAUGGGACGGAGG	CCUCCGUCCCAUAAUAUAAGU	Cleavage
tae-miR1133	Traes_7BL_07C4E8081.43										0,00	-1.0	1	22	46	67	CAUAUACUCCCUCCGUCCGAAA	UUUCGGACGGAGGGAGUAUAUG	Cleavage
tae-miR1133	Traes_7BL_07C4E8081.44										0,00	-1.0	1	22	46	67	CAUAUACUCCCUCCGUCCGAAA	UUUCGGACGGAGGGAGUAUAUG	Cleavage
tae-miR1133	Traes_7BL_07C4E8081.45										0,00	-1.0	1	22	46	67	CAUAUACUCCCUCCGUCCGAAA	UUUCGGACGGAGGGAGUAUAUG	Cleavage
tae-miR160	Traes_7BL_18D335F08.1						AT4G30080.1	auxin response factor 16	LOC_Os06g47150.2	auxin response factor 18, putative, expressed	0,00	-1.0	1	21	171	191	UGCCUGGCUCCCUGUAUGCCA	AGGCAUACAGGGAGCCAGGCA	Cleavage
tae-miR160	Traes_7BL_18D335F08.2						AT4G30080.1	auxin response factor 16	LOC_Os06g47150.1	auxin response factor 18, putative, expressed	0,00	-1.0	1	21	171	191	UGCCUGGCUCCCUGUAUGCCA	AGGCAUACAGGGAGCCAGGCA	Cleavage
tae-miR1137a	Traes_7BL_C184D0525.2	PF00249					AT5G61620.1	myb-like transcription factor family protein	LOC_Os08g05510.1	MYB family transcription factor, putative, expressed	0,00	-1.0	1	20	913	932	UAGUACAAAGUUGAGUCAUC	GAUGACUCAACUUUGUACUA	Cleavage
tae-miR395b	Traes_7BL_DC0D8773C.1	PF01909,PF04926,PF04928		2.7.7.19		GO:GO:0016779,GO:GO:0043631,GO:GO:0003723,GO:GO:0004652,GO:GO:0005634	AT4G32850.5	nuclear poly(a) polymerase	LOC_Os06g36360.4	expressed protein	2,50	-1.0	1	20	1385	1404	UGAAGUGUUUGGGGGAACUC	CAGUUCACCCAAAAACUUCA	Cleavage
tae-miR395b	Traes_7BL_DC0D8773C.10	PF04926,PF04928		2.7.7.19		GO:GO:0043631,GO:GO:0003723,GO:GO:0004652,GO:GO:0005634	AT4G32850.10	nuclear poly(a) polymerase	LOC_Os06g36360.1	expressed protein	2,50	-1.0	1	20	1148	1167	UGAAGUGUUUGGGGGAACUC	CAGUUCACCCAAAAACUUCA	Cleavage
tae-miR395b	Traes_7BL_DC0D8773C.11	PF04926,PF04928		2.7.7.19		GO:GO:0043631,GO:GO:0003723,GO:GO:0004652,GO:GO:0005634	AT4G32850.5	nuclear poly(a) polymerase	LOC_Os06g36360.3	expressed protein	2,50	-1.0	1	20	848	867	UGAAGUGUUUGGGGGAACUC	CAGUUCACCCAAAAACUUCA	Cleavage
tae-miR395b	Traes_7BL_DC0D8773C.12	PF04926,PF04928		2.7.7.19		GO:GO:0043631,GO:GO:0003723,GO:GO:0004652,GO:GO:0005634	AT4G32850.5	nuclear poly(a) polymerase	LOC_Os06g36360.1	expressed protein	2,50	-1.0	1	20	848	867	UGAAGUGUUUGGGGGAACUC	CAGUUCACCCAAAAACUUCA	Cleavage
tae-miR395b	Traes_7BL_DC0D8773C.13	PF04926,PF04928		2.7.7.19		GO:GO:0043631,GO:GO:0003723,GO:GO:0004652,GO:GO:0005634	AT4G32850.9	nuclear poly(a) polymerase	LOC_Os06g36360.1	expressed protein	2,50	-1.0	1	20	848	867	UGAAGUGUUUGGGGGAACUC	CAGUUCACCCAAAAACUUCA	Cleavage
tae-miR395b	Traes_7BL_DC0D8773C.14	PF04926,PF04928		2.7.7.19		GO:GO:0043631,GO:GO:0003723,GO:GO:0004652,GO:GO:0005634	AT4G32850.5	nuclear poly(a) polymerase	LOC_Os06g36360.3	expressed protein	2,50	-1.0	1	20	848	867	UGAAGUGUUUGGGGGAACUC	CAGUUCACCCAAAAACUUCA	Cleavage
tae-miR395b	Traes_7BL_DC0D8773C.2	PF01909,PF04926,PF04928		2.7.7.19		GO:GO:0016779,GO:GO:0043631,GO:GO:0003723,GO:GO:0004652,GO:GO:0005634	AT4G32850.5	nuclear poly(a) polymerase	LOC_Os06g36360.4	expressed protein	2,50	-1.0	1	20	1385	1404	UGAAGUGUUUGGGGGAACUC	CAGUUCACCCAAAAACUUCA	Cleavage
tae-miR395b	Traes_7BL_DC0D8773C.3	PF01909,PF04926,PF04928		2.7.7.19		GO:GO:0016779,GO:GO:0043631,GO:GO:0003723,GO:GO:0004652,GO:GO:0005634	AT4G32850.9	nuclear poly(a) polymerase	LOC_Os06g36360.1	expressed protein	2,50	-1.0	1	20	1385	1404	UGAAGUGUUUGGGGGAACUC	CAGUUCACCCAAAAACUUCA	Cleavage
tae-miR395b	Traes_7BL_DC0D8773C.4	PF01909,PF04926,PF04928		2.7.7.19		GO:GO:0016779,GO:GO:0043631,GO:GO:0003723,GO:GO:0004652,GO:GO:0005634	AT4G32850.5	nuclear poly(a) polymerase	LOC_Os06g36360.2	expressed protein	2,50	-1.0	1	20	1385	1404	UGAAGUGUUUGGGGGAACUC	CAGUUCACCCAAAAACUUCA	Cleavage
tae-miR395b	Traes_7BL_DC0D8773C.5	PF01909,PF04926,PF04928		2.7.7.19		GO:GO:0016779,GO:GO:0043631,GO:GO:0003723,GO:GO:0004652,GO:GO:0005634	AT4G32850.5	nuclear poly(a) polymerase	LOC_Os06g36360.2	expressed protein	2,50	-1.0	1	20	1385	1404	UGAAGUGUUUGGGGGAACUC	CAGUUCACCCAAAAACUUCA	Cleavage
tae-miR395b	Traes_7BL_DC0D8773C.6	PF04926,PF04928		2.7.7.19		GO:GO:0043631,GO:GO:0003723,GO:GO:0004652,GO:GO:0005634	AT4G32850.10	nuclear poly(a) polymerase	LOC_Os06g36360.1	expressed protein	2,50	-1.0	1	20	1148	1167	UGAAGUGUUUGGGGGAACUC	CAGUUCACCCAAAAACUUCA	Cleavage
tae-miR395b	Traes_7BL_DC0D8773C.7	PF04926,PF04928		2.7.7.19		GO:GO:0043631,GO:GO:0003723,GO:GO:0004652,GO:GO:0005634	AT4G32850.10	nuclear poly(a) polymerase	LOC_Os06g36360.2	expressed protein	2,50	-1.0	1	20	1148	1167	UGAAGUGUUUGGGGGAACUC	CAGUUCACCCAAAAACUUCA	Cleavage
tae-miR395b	Traes_7BL_DC0D8773C.8	PF04926,PF04928		2.7.7.19		GO:GO:0043631,GO:GO:0003723,GO:GO:0004652,GO:GO:0005634	AT4G32850.10	nuclear poly(a) polymerase	LOC_Os06g36360.1	expressed protein	2,50	-1.0	1	20	1148	1167	UGAAGUGUUUGGGGGAACUC	CAGUUCACCCAAAAACUUCA	Cleavage
tae-miR395b	Traes_7BL_DC0D8773C.9	PF04926,PF04928		2.7.7.19		GO:GO:0043631,GO:GO:0003723,GO:GO:0004652,GO:GO:0005634	AT4G32850.10	nuclear poly(a) polymerase	LOC_Os06g36360.1	expressed protein	2,50	-1.0	1	20	1148	1167	UGAAGUGUUUGGGGGAACUC	CAGUUCACCCAAAAACUUCA	Cleavage
tae-miR164	Traes_7BL_F1A0270C6.1								LOC_Os06g46270.1	no apical meristem protein, putative, expressed	1,00	-1.0	1	21	32	52	UGGAGAAGCAGGGCACGUGCA	AGCAAGUGCCCUGCUUCUCCA	Cleavage
tae-miR9666a-3p	Traes_7BL_F8D5E60B1.1										2,00	-1.0	1	22	5050	5071	CGGUAGGGCUGUAUGAUGGCGA	UCCCCCUCAUACAGCCCUACCU	Cleavage
tae-miR9666a-3p	Traes_7BL_F8D5E60B1.1										2,50	-1.0	1	22	4966	4987	CGGUAGGGCUGUAUGAUGGCGA	UCGCCAUCAUACAGCCCUACUU	Cleavage
tae-miR9666a-3p	Traes_7BL_F8D5E60B1.2										2,00	-1.0	1	22	5011	5032	CGGUAGGGCUGUAUGAUGGCGA	UCCCCCUCAUACAGCCCUACCU	Cleavage
tae-miR9666a-3p	Traes_7BL_F8D5E60B1.2										2,50	-1.0	1	22	4927	4948	CGGUAGGGCUGUAUGAUGGCGA	UCGCCAUCAUACAGCCCUACUU	Cleavage
tae-miR9666a-3p	Traes_7BL_F8D5E60B1.3										2,00	-1.0	1	22	4780	4801	CGGUAGGGCUGUAUGAUGGCGA	UCCCCCUCAUACAGCCCUACCU	Cleavage
tae-miR9666a-3p	Traes_7BL_F8D5E60B1.3										2,50	-1.0	1	22	4696	4717	CGGUAGGGCUGUAUGAUGGCGA	UCGCCAUCAUACAGCCCUACUU	Cleavage
tae-miR9666a-3p	Traes_7BL_F8D5E60B1.4										2,00	-1.0	1	22	3409	3430	CGGUAGGGCUGUAUGAUGGCGA	UCCCCCUCAUACAGCCCUACCU	Cleavage
tae-miR9666a-3p	Traes_7BL_F8D5E60B1.4										2,50	-1.0	1	22	3325	3346	CGGUAGGGCUGUAUGAUGGCGA	UCGCCAUCAUACAGCCCUACUU	Cleavage
tae-miR1137a	Traes_7BS_2A6C29F49.3	PF00566					AT3G49350.1	Ypt/Rab-GAP domain of gyp1p superfamily protein	LOC_Os09g35960.1	TBC domain containing protein, expressed	2,00	-1.0	1	20	75	94	UAGUACAAAGUUGAGUCAUC	AGUGUCUCAACUUUGUACUA	Cleavage
tae-miR1137a	Traes_7BS_2A6C29F49.4	PF00566					AT3G49350.1	Ypt/Rab-GAP domain of gyp1p superfamily protein	LOC_Os09g35960.1	TBC domain containing protein, expressed	2,00	-1.0	1	20	81	100	UAGUACAAAGUUGAGUCAUC	AGUGUCUCAACUUUGUACUA	Cleavage
tae-miR1130b-3p	Traes_7BS_47B8FC9CB.2	PF01154,PF08540		2.3.3.10		GO:GO:0008299,GO:GO:0004421	AT4G11820.2	hydroxymethylglutaryl-CoA synthase / HMG-CoA synthase / 3-hydroxy-3-methylglutaryl coenzyme A synthase	LOC_Os08g43170.1	hydroxymethylglutaryl-CoA synthase, putative, expressed	0,00	-1.0	1	21	1268	1288	UCUUAUAUUAUGGGACGGAGG	CCUCCGUCCCAUAAUAUAAGA	Cleavage
tae-miR1122c-3p	Traes_7BS_47B8FC9CB.2	PF01154,PF08540		2.3.3.10		GO:GO:0008299,GO:GO:0004421	AT4G11820.2	hydroxymethylglutaryl-CoA synthase / HMG-CoA synthase / 3-hydroxy-3-methylglutaryl coenzyme A synthase	LOC_Os08g43170.1	hydroxymethylglutaryl-CoA synthase, putative, expressed	1,50	-1.0	1	21	1268	1288	UCUAAUAUUAUGGGACGGAGG	CCUCCGUCCCAUAAUAUAAGA	Cleavage
tae-miR1120c-5p	Traes_7BS_47B8FC9CB.2	PF01154,PF08540		2.3.3.10		GO:GO:0008299,GO:GO:0004421	AT4G11820.2	hydroxymethylglutaryl-CoA synthase / HMG-CoA synthase / 3-hydroxy-3-methylglutaryl coenzyme A synthase	LOC_Os08g43170.1	hydroxymethylglutaryl-CoA synthase, putative, expressed	1,50	-1.0	1	21	1311	1331	UAAUAUAAGAACGUUUUUGAC	GUCAAAAACGCUCUUAUAUUA	Translation
tae-miR1130b-3p	Traes_7BS_47B8FC9CB.3	PF01154,PF08540		2.3.3.10	K01641	GO:GO:0008299,GO:GO:0004421	AT4G11820.2	hydroxymethylglutaryl-CoA synthase / HMG-CoA synthase / 3-hydroxy-3-methylglutaryl coenzyme A synthase	LOC_Os08g43170.1	hydroxymethylglutaryl-CoA synthase, putative, expressed	0,00	-1.0	1	21	1265	1285	UCUUAUAUUAUGGGACGGAGG	CCUCCGUCCCAUAAUAUAAGA	Cleavage
tae-miR1122c-3p	Traes_7BS_47B8FC9CB.3	PF01154,PF08540		2.3.3.10	K01641	GO:GO:0008299,GO:GO:0004421	AT4G11820.2	hydroxymethylglutaryl-CoA synthase / HMG-CoA synthase / 3-hydroxy-3-methylglutaryl coenzyme A synthase	LOC_Os08g43170.1	hydroxymethylglutaryl-CoA synthase, putative, expressed	1,50	-1.0	1	21	1265	1285	UCUAAUAUUAUGGGACGGAGG	CCUCCGUCCCAUAAUAUAAGA	Cleavage
tae-miR1120c-5p	Traes_7BS_47B8FC9CB.3	PF01154,PF08540		2.3.3.10	K01641	GO:GO:0008299,GO:GO:0004421	AT4G11820.2	hydroxymethylglutaryl-CoA synthase / HMG-CoA synthase / 3-hydroxy-3-methylglutaryl coenzyme A synthase	LOC_Os08g43170.1	hydroxymethylglutaryl-CoA synthase, putative, expressed	1,50	-1.0	1	21	1308	1328	UAAUAUAAGAACGUUUUUGAC	GUCAAAAACGCUCUUAUAUUA	Translation
tae-miR9662a-3p	Traes_7BS_598310C32.1	PF02536				GO:GO:0006355,GO:GO:0005739,GO:GO:0003690	AT1G21150.1	Mitochondrial transcription termination factor family protein	LOC_Os06g12100.1	mTERF family protein, expressed	2,50	-1.0	1	21	33	53	UUGAACAUCCCAGAGCCACCG	CCGUGGCUCUGCGAUGUUCAG	Translation
tae-miR9662b-3p	Traes_7BS_598310C32.1	PF02536				GO:GO:0006355,GO:GO:0005739,GO:GO:0003690	AT1G21150.1	Mitochondrial transcription termination factor family protein	LOC_Os06g12100.1	mTERF family protein, expressed	2,50	-1.0	1	21	32	52	UGAACAUCCCAGAGCCACCGG	CCCGUGGCUCUGCGAUGUUCA	Cleavage
tae-miR1121	Traes_7BS_5A0E77FB8.2	PF00664,PF00005		3.6.3.44		GO:GO:0055085,GO:GO:0042626,GO:GO:0016021,GO:GO:0006810,GO:GO:0005524,GO:GO:0016887	AT3G59140.1	multidrug resistance-associated protein 14	LOC_Os06g06440.1	ABC transporter, ATP-binding protein, putative, expressed	1,00	-1.0	1	22	3478	3499	AGUAGUGAUCUAAACGCUCUUA	UAAGAGUGUUUAGAUCACUACU	Cleavage
tae-miR1137a	Traes_7BS_6716D1D07.2	PF01105				GO:GO:0016021,GO:GO:0006810	AT1G09580.1	emp24/gp25L/p24 family/GOLD family protein	LOC_Os06g10760.1	emp24/gp25L/p24 family protein, putative, expressed	0,00	-1.0	1	20	347	366	UAGUACAAAGUUGAGUCAUC	GAUGACUCAACUUUGUACUA	Cleavage
tae-miR9781	Traes_7BS_A42F893FC.1	PF10150		3.1.26.12		GO:GO:0003723	AT2G04270.2	RNAse E/G-like	LOC_Os08g23430.1	starch binding domain containing protein, putative, expressed	1,50	-1.0	1	21	117	137	UUUUGUCACAUAUAAUACAUA	UAUGUAUUUUAUGUGACAAAA	Cleavage
tae-miR9664-3p	Traes_7DL_101A9F756.1	PF00931	KOG4658			GO:GO:0043531	AT1G58807.1	Disease resistance protein (CC-NBS-LRR class) family	LOC_Os11g11550.1	NBS-LRR disease resistance protein, putative, expressed	2,50	-1.0	1	21	102	122	UUGCAGUCCUCGAUGUCGUAG	GCACGACAUCGAGGAUUGCAU	Cleavage
tae-miR9664-3p	Traes_7DL_101A9F756.2	PF00931	KOG4658			GO:GO:0043531	AT1G58807.1	Disease resistance protein (CC-NBS-LRR class) family	LOC_Os11g11550.2	NBS-LRR disease resistance protein, putative, expressed	2,50	-1.0	1	21	102	122	UUGCAGUCCUCGAUGUCGUAG	GCACGACAUCGAGGAUUGCAU	Cleavage
tae-miR9664-3p	Traes_7DL_101A9F756.3	PF00931	KOG4658			GO:GO:0043531	AT1G58807.1	Disease resistance protein (CC-NBS-LRR class) family	LOC_Os11g11550.1	NBS-LRR disease resistance protein, putative, expressed	2,50	-1.0	1	21	231	251	UUGCAGUCCUCGAUGUCGUAG	GCACGACAUCGAGGAUUGCAU	Cleavage
tae-miR9666a-3p	Traes_7DL_18F2A8567.1										2,50	-1.0	1	22	4927	4948	CGGUAGGGCUGUAUGAUGGCGA	UCGCCAUCAUACAGCCCUACUU	Cleavage
tae-miR9666a-3p	Traes_7DL_18F2A8567.11										2,00	-1.0	1	22	244	265	CGGUAGGGCUGUAUGAUGGCGA	UCGCCCUCAUACAGCCCUACCU	Cleavage
tae-miR9666a-3p	Traes_7DL_18F2A8567.11										2,50	-1.0	1	22	139	160	CGGUAGGGCUGUAUGAUGGCGA	UCGCCAUCAUACAGCCCUACUU	Cleavage
tae-miR9666a-3p	Traes_7DL_18F2A8567.12										2,00	-1.0	1	22	250	271	CGGUAGGGCUGUAUGAUGGCGA	UCGCCCUCAUACAGCCCUACCU	Cleavage
tae-miR9666a-3p	Traes_7DL_18F2A8567.12										2,50	-1.0	1	22	145	166	CGGUAGGGCUGUAUGAUGGCGA	UCGCCAUCAUACAGCCCUACUU	Cleavage
tae-miR9666a-3p	Traes_7DL_18F2A8567.14										2,00	-1.0	1	22	201	222	CGGUAGGGCUGUAUGAUGGCGA	UCGCCCUCAUACAGCCCUACCU	Cleavage
tae-miR9666a-3p	Traes_7DL_18F2A8567.14										2,50	-1.0	1	22	96	117	CGGUAGGGCUGUAUGAUGGCGA	UCGCCAUCAUACAGCCCUACUU	Cleavage
tae-miR9666a-3p	Traes_7DL_18F2A8567.2										2,00	-1.0	1	22	5032	5053	CGGUAGGGCUGUAUGAUGGCGA	UCGCCCUCAUACAGCCCUACCU	Cleavage
tae-miR9666a-3p	Traes_7DL_18F2A8567.2										2,50	-1.0	1	22	4927	4948	CGGUAGGGCUGUAUGAUGGCGA	UCGCCAUCAUACAGCCCUACUU	Cleavage
tae-miR9666a-3p	Traes_7DL_18F2A8567.3										2,00	-1.0	1	22	4969	4990	CGGUAGGGCUGUAUGAUGGCGA	UCGCCCUCAUACAGCCCUACCU	Cleavage
tae-miR9666a-3p	Traes_7DL_18F2A8567.3										2,50	-1.0	1	22	4927	4948	CGGUAGGGCUGUAUGAUGGCGA	UCGCCAUCAUACAGCCCUACUU	Cleavage
tae-miR9666a-3p	Traes_7DL_18F2A8567.4										2,50	-1.0	1	22	4927	4948	CGGUAGGGCUGUAUGAUGGCGA	UCGCCAUCAUACAGCCCUACUU	Cleavage
tae-miR9666a-3p	Traes_7DL_18F2A8567.5										2,50	-1.0	1	22	4927	4948	CGGUAGGGCUGUAUGAUGGCGA	UCGCCAUCAUACAGCCCUACUU	Cleavage
tae-miR9666a-3p	Traes_7DL_18F2A8567.6										2,00	-1.0	1	22	5032	5053	CGGUAGGGCUGUAUGAUGGCGA	UCGCCCUCAUACAGCCCUACCU	Cleavage
tae-miR9666a-3p	Traes_7DL_18F2A8567.6										2,50	-1.0	1	22	4927	4948	CGGUAGGGCUGUAUGAUGGCGA	UCGCCAUCAUACAGCCCUACUU	Cleavage
tae-miR9666a-3p	Traes_7DL_18F2A8567.7										2,00	-1.0	1	22	4801	4822	CGGUAGGGCUGUAUGAUGGCGA	UCGCCCUCAUACAGCCCUACCU	Cleavage
tae-miR9666a-3p	Traes_7DL_18F2A8567.7										2,50	-1.0	1	22	4696	4717	CGGUAGGGCUGUAUGAUGGCGA	UCGCCAUCAUACAGCCCUACUU	Cleavage
tae-miR9666a-3p	Traes_7DL_18F2A8567.8										2,00	-1.0	1	22	3409	3430	CGGUAGGGCUGUAUGAUGGCGA	UCGCCCUCAUACAGCCCUACCU	Cleavage
tae-miR9666a-3p	Traes_7DL_18F2A8567.8										2,50	-1.0	1	22	3304	3325	CGGUAGGGCUGUAUGAUGGCGA	UCGCCAUCAUACAGCCCUACUU	Cleavage
tae-miR9666a-3p	Traes_7DL_18F2A8567.9										2,00	-1.0	1	22	2941	2962	CGGUAGGGCUGUAUGAUGGCGA	UCGCCCUCAUACAGCCCUACCU	Cleavage
tae-miR9666a-3p	Traes_7DL_18F2A8567.9										2,50	-1.0	1	22	2836	2857	CGGUAGGGCUGUAUGAUGGCGA	UCGCCAUCAUACAGCCCUACUU	Cleavage
tae-miR164	Traes_7DL_1E343B6E6.1						AT1G56010.2	NAC domain containing protein 1	LOC_Os06g46270.1	no apical meristem protein, putative, expressed	1,00	-1.0	1	21	236	256	UGGAGAAGCAGGGCACGUGCA	AGCAAGUGCCCUGCUUCUCCA	Cleavage
tae-miR164	Traes_7DL_1E343B6E6.2								LOC_Os06g46270.1	no apical meristem protein, putative, expressed	1,00	-1.0	1	21	32	52	UGGAGAAGCAGGGCACGUGCA	AGCAAGUGCCCUGCUUCUCCA	Cleavage
tae-miR1135	Traes_7DL_20BFD8A19.4	PF00145		2.1.1.37			AT4G19020.1	chromomethylase 2	LOC_Os10g01570.1	C-5 cytosine-specific DNA methylase, putative, expressed	1,50	-1.0	1	24	954	977	CUGCGACAAGUAAUUCCGAACGGA	UCUGUUCGGAAUUACUUGUCGUAG	Cleavage
tae-miR1127a	Traes_7DL_20BFD8A19.4	PF00145		2.1.1.37			AT4G19020.1	chromomethylase 2	LOC_Os10g01570.1	C-5 cytosine-specific DNA methylase, putative, expressed	1,50	-1.0	1	19	1032	1050	UCCUUCCGUUCGGAAUUAC	GUAAUUCCGAACGGAGGGA	Cleavage
tae-miR9778	Traes_7DL_31A23053A.1								LOC_Os11g39600.1	resistance protein, putative, expressed	1,00	-1.0	1	21	132	152	UGCAUCAUCUCGAACUCGUCG	CCUCGAGUUCGAGAUGAUGCA	Cleavage
tae-miR1122a	Traes_7DL_37135CE6E.2	PF00076	KOG0128			GO:GO:0003676	AT4G24270.2	EMBRYO DEFECTIVE 140	LOC_Os08g02130.2	RNA recognition motif containing protein, putative, expressed	2,50	-1.0	1	20	2105	2124	UAGAUACAUCCGUAUCUAGA	UCUAAAUACGGAUGUAUCAA	Cleavage
tae-miR1122a	Traes_7DL_37135CE6E.3	PF00076	KOG0128			GO:GO:0003676	AT4G24270.2	EMBRYO DEFECTIVE 140	LOC_Os08g02130.2	RNA recognition motif containing protein, putative, expressed	2,50	-1.0	1	20	2090	2109	UAGAUACAUCCGUAUCUAGA	UCUAAAUACGGAUGUAUCAA	Cleavage
tae-miR1122a	Traes_7DL_37135CE6E.6	PF00076	KOG0130			GO:GO:0003676	AT4G24270.2	EMBRYO DEFECTIVE 140	LOC_Os08g02130.1	RNA recognition motif containing protein, putative, expressed	2,50	-1.0	1	20	1241	1260	UAGAUACAUCCGUAUCUAGA	UCUAAAUACGGAUGUAUCAA	Cleavage
tae-miR9661-5p	Traes_7DL_55AC0BE09.1										2,50	-1.0	1	21	143	163	UGAAGUAGAGCAGGGACCUCA	UCAGGUCCCUGCGCUACUUCG	Cleavage
tae-miR9661-5p	Traes_7DL_55AC0BE09.2										2,50	-1.0	1	21	143	163	UGAAGUAGAGCAGGGACCUCA	UCAGGUCCCUGCGCUACUUCG	Cleavage
tae-miR9661-5p	Traes_7DL_55AC0BE09.3										2,50	-1.0	1	21	116	136	UGAAGUAGAGCAGGGACCUCA	UCAGGUCCCUGCGCUACUUCG	Cleavage
tae-miR9661-5p	Traes_7DL_55AC0BE09.4										2,50	-1.0	1	21	116	136	UGAAGUAGAGCAGGGACCUCA	UCAGGUCCCUGCGCUACUUCG	Cleavage
tae-miR9661-5p	Traes_7DL_55AC0BE09.5										2,50	-1.0	1	21	116	136	UGAAGUAGAGCAGGGACCUCA	UCAGGUCCCUGCGCUACUUCG	Cleavage
tae-miR160	Traes_7DL_55ADB3528.1	PF06507				GO:GO:0009725,GO:GO:0006355,GO:GO:0005634,GO:GO:0003677	AT4G30080.1	auxin response factor 16	LOC_Os06g47150.1	auxin response factor 18, putative, expressed	0,00	-1.0	1	21	714	734	UGCCUGGCUCCCUGUAUGCCA	AGGCAUACAGGGAGCCAGGCA	Cleavage
tae-miR160	Traes_7DL_55ADB3528.2	PF06507				GO:GO:0009725,GO:GO:0006355,GO:GO:0005634,GO:GO:0003677	AT4G30080.1	auxin response factor 16	LOC_Os06g47150.3	auxin response factor 18, putative, expressed	0,00	-1.0	1	21	714	734	UGCCUGGCUCCCUGUAUGCCA	AGGCAUACAGGGAGCCAGGCA	Cleavage
tae-miR1134	Traes_7DL_5E2559796.1										2,00	-1.0	1	24	2562	2585	CAACAACAACAAGAAGAAGAAGAU	UUGUUGUUGUUCUUGUUGUUGUUG	Cleavage
tae-miR1134	Traes_7DL_5E2559796.2										2,00	-1.0	1	24	2556	2579	CAACAACAACAAGAAGAAGAAGAU	UUGUUGUUGUUCUUGUUGUUGUUG	Cleavage
tae-miR1122b-3p	Traes_7DL_613007B24.2	PF00400	KOG0279,KOG1332			GO:GO:0005515	AT1G15750.3	Transducin family protein / WD-40 repeat family protein	LOC_Os08g06480.1	lissencephaly type-1-like homology motif, putative, expressed	1,50	-1.0	1	21	1492	1512	AGACUUAUAUGUAGGAACGGA	UCCGUUCCUAAAUAUAAGUCU	Translation
tae-miR164	Traes_7DL_91CFE828F.1	PF02365				GO:GO:0006355,GO:GO:0003677	AT1G56010.2	NAC domain containing protein 1	LOC_Os06g46270.1	no apical meristem protein, putative, expressed	1,00	-1.0	1	21	497	517	UGGAGAAGCAGGGCACGUGCA	AGCAAGUGCCCUGCUUCUCCA	Cleavage
tae-miR164	Traes_7DL_91CFE828F.2						AT1G56010.2	NAC domain containing protein 1	LOC_Os06g46270.1	no apical meristem protein, putative, expressed	1,00	-1.0	1	21	296	316	UGGAGAAGCAGGGCACGUGCA	AGCAAGUGCCCUGCUUCUCCA	Cleavage
tae-miR9778	Traes_7DL_9F0AD190A.2	PF00931	KOG4658			GO:GO:0043531	AT1G58400.1	Disease resistance protein (CC-NBS-LRR class) family	LOC_Os08g14850.1	resistance protein, putative, expressed	2,50	-1.0	1	21	126	146	UGCAUCAUCUCGAACUCGUCG	AGGCGAGUUUGAGAUGAUGCA	Cleavage
tae-miR9778	Traes_7DL_9F0AD190A.3	PF00931	KOG4658			GO:GO:0043531	AT1G58400.1	Disease resistance protein (CC-NBS-LRR class) family	LOC_Os08g14850.1	resistance protein, putative, expressed	2,50	-1.0	1	21	126	146	UGCAUCAUCUCGAACUCGUCG	AGGCGAGUUUGAGAUGAUGCA	Cleavage
tae-miR5048-5p	Traes_7DL_AB7C59D49.1	PF00069		2.7.11.1		GO:GO:0006468,GO:GO:0005524,GO:GO:0004672	AT4G21230.1	cysteine-rich RLK (RECEPTOR-like protein kinase) 27	LOC_Os11g12270.2	expressed protein	0,00	-1.0	1	22	53	74	UUUGCAGGUUUUAGGUCUAAGU	ACUUAGACCUAAAACCUGCAAA	Cleavage
tae-miR5048-5p	Traes_7DL_AB7C59D49.2	PF00069		2.7.11.1		GO:GO:0006468,GO:GO:0005524,GO:GO:0004672	AT1G11300.1	protein serine/threonine kinases;protein kinases;ATP binding;sugar binding;kinases;carbohydrate binding	LOC_Os11g17380.1	protein kinase domain containing protein, expressed	0,00	-1.0	1	22	53	74	UUUGCAGGUUUUAGGUCUAAGU	ACUUAGACCUAAAACCUGCAAA	Cleavage
tae-miR5048-5p	Traes_7DL_AB7C59D49.3	PF00069		2.7.11.1		GO:GO:0006468,GO:GO:0005524,GO:GO:0004672	AT1G11300.1	protein serine/threonine kinases;protein kinases;ATP binding;sugar binding;kinases;carbohydrate binding	LOC_Os11g17380.1	protein kinase domain containing protein, expressed	0,00	-1.0	1	22	53	74	UUUGCAGGUUUUAGGUCUAAGU	ACUUAGACCUAAAACCUGCAAA	Cleavage
tae-miR5048-5p	Traes_7DL_AB7C59D49.4	PF00069		2.7.11.1		GO:GO:0006468,GO:GO:0005524,GO:GO:0004672	AT1G11300.1	protein serine/threonine kinases;protein kinases;ATP binding;sugar binding;kinases;carbohydrate binding	LOC_Os11g17380.1	protein kinase domain containing protein, expressed	0,00	-1.0	1	22	53	74	UUUGCAGGUUUUAGGUCUAAGU	ACUUAGACCUAAAACCUGCAAA	Cleavage
tae-miR5048-5p	Traes_7DL_AB7C59D49.5	PF00069		2.7.11.1		GO:GO:0006468,GO:GO:0005524,GO:GO:0004672	AT4G21230.1	cysteine-rich RLK (RECEPTOR-like protein kinase) 27	LOC_Os11g12270.2	expressed protein	0,00	-1.0	1	22	53	74	UUUGCAGGUUUUAGGUCUAAGU	ACUUAGACCUAAAACCUGCAAA	Cleavage
tae-miR5048-5p	Traes_7DL_AB7C59D49.6	PF00069		2.7.11.1		GO:GO:0006468,GO:GO:0005524,GO:GO:0004672	AT4G21230.1	cysteine-rich RLK (RECEPTOR-like protein kinase) 27	LOC_Os11g12270.2	expressed protein	0,00	-1.0	1	22	53	74	UUUGCAGGUUUUAGGUCUAAGU	ACUUAGACCUAAAACCUGCAAA	Cleavage
tae-miR1122a	Traes_7DL_C074DC1F6.3	PF00141		1.11.1.7		GO:GO:0055114,GO:GO:0020037,GO:GO:0006979,GO:GO:0004601	AT5G64120.1	Peroxidase superfamily protein	LOC_Os07g44550.1	peroxidase precursor, putative, expressed	2,50	-1.0	1	20	109	128	UAGAUACAUCCGUAUCUAGA	ACUACAUACGGAUGUAUAUA	Cleavage
tae-miR395b	Traes_7DL_CB073ADC6.1	PF01909,PF04926,PF04928		2.7.7.19	K14376	GO:GO:0016779,GO:GO:0043631,GO:GO:0003723,GO:GO:0004652,GO:GO:0005634	AT4G32850.5	nuclear poly(a) polymerase	LOC_Os06g36360.3	expressed protein	2,50	-1.0	1	20	1598	1617	UGAAGUGUUUGGGGGAACUC	CAGUUCACCCAAAAACUUCA	Cleavage
tae-miR395b	Traes_7DL_CB073ADC6.10	PF04926,PF04928		2.7.7.19		GO:GO:0043631,GO:GO:0003723,GO:GO:0004652,GO:GO:0005634	AT4G32850.9	nuclear poly(a) polymerase	LOC_Os06g36360.1	expressed protein	2,50	-1.0	1	20	851	870	UGAAGUGUUUGGGGGAACUC	CAGUUCACCCAAAAACUUCA	Cleavage
tae-miR395b	Traes_7DL_CB073ADC6.11	PF04926,PF04928		2.7.7.19		GO:GO:0043631,GO:GO:0003723,GO:GO:0004652,GO:GO:0005634	AT4G32850.5	nuclear poly(a) polymerase	LOC_Os06g36360.2	expressed protein	2,50	-1.0	1	20	851	870	UGAAGUGUUUGGGGGAACUC	CAGUUCACCCAAAAACUUCA	Cleavage
tae-miR395b	Traes_7DL_CB073ADC6.12	PF04926,PF04928		2.7.7.19		GO:GO:0043631,GO:GO:0003723,GO:GO:0004652,GO:GO:0005634	AT4G32850.9	nuclear poly(a) polymerase	LOC_Os06g36360.2	expressed protein	2,50	-1.0	1	20	851	870	UGAAGUGUUUGGGGGAACUC	CAGUUCACCCAAAAACUUCA	Cleavage
tae-miR395b	Traes_7DL_CB073ADC6.2	PF01909,PF04926,PF04928		2.7.7.19	K14376	GO:GO:0016779,GO:GO:0043631,GO:GO:0003723,GO:GO:0004652,GO:GO:0005634	AT4G32850.5	nuclear poly(a) polymerase	LOC_Os06g36360.2	expressed protein	2,50	-1.0	1	20	1598	1617	UGAAGUGUUUGGGGGAACUC	CAGUUCACCCAAAAACUUCA	Cleavage
tae-miR395b	Traes_7DL_CB073ADC6.3	PF01909,PF04926,PF04928		2.7.7.19	K14376	GO:GO:0016779,GO:GO:0043631,GO:GO:0003723,GO:GO:0004652,GO:GO:0005634	AT4G32850.9	nuclear poly(a) polymerase	LOC_Os06g36360.1	expressed protein	2,50	-1.0	1	20	1598	1617	UGAAGUGUUUGGGGGAACUC	CAGUUCACCCAAAAACUUCA	Cleavage
tae-miR395b	Traes_7DL_CB073ADC6.4	PF04926,PF04928		2.7.7.19		GO:GO:0043631,GO:GO:0003723,GO:GO:0004652,GO:GO:0005634	AT4G32850.10	nuclear poly(a) polymerase	LOC_Os06g36360.3	expressed protein	2,50	-1.0	1	20	1157	1176	UGAAGUGUUUGGGGGAACUC	CAGUUCACCCAAAAACUUCA	Cleavage
tae-miR395b	Traes_7DL_CB073ADC6.5	PF04926,PF04928		2.7.7.19		GO:GO:0043631,GO:GO:0003723,GO:GO:0004652,GO:GO:0005634	AT4G32850.10	nuclear poly(a) polymerase	LOC_Os06g36360.1	expressed protein	2,50	-1.0	1	20	1157	1176	UGAAGUGUUUGGGGGAACUC	CAGUUCACCCAAAAACUUCA	Cleavage
tae-miR395b	Traes_7DL_CB073ADC6.6	PF04926,PF04928		2.7.7.19		GO:GO:0043631,GO:GO:0003723,GO:GO:0004652,GO:GO:0005634	AT4G32850.10	nuclear poly(a) polymerase	LOC_Os06g36360.3	expressed protein	2,50	-1.0	1	20	1157	1176	UGAAGUGUUUGGGGGAACUC	CAGUUCACCCAAAAACUUCA	Cleavage
tae-miR395b	Traes_7DL_CB073ADC6.7	PF04926,PF04928		2.7.7.19		GO:GO:0043631,GO:GO:0003723,GO:GO:0004652,GO:GO:0005634	AT4G32850.10	nuclear poly(a) polymerase	LOC_Os06g36360.2	expressed protein	2,50	-1.0	1	20	1157	1176	UGAAGUGUUUGGGGGAACUC	CAGUUCACCCAAAAACUUCA	Cleavage
tae-miR395b	Traes_7DL_CB073ADC6.8	PF04926,PF04928		2.7.7.19		GO:GO:0043631,GO:GO:0003723,GO:GO:0004652,GO:GO:0005634	AT4G32850.10	nuclear poly(a) polymerase	LOC_Os06g36360.2	expressed protein	2,50	-1.0	1	20	1079	1098	UGAAGUGUUUGGGGGAACUC	CAGUUCACCCAAAAACUUCA	Cleavage
tae-miR395b	Traes_7DL_CB073ADC6.9	PF04926,PF04928		2.7.7.19		GO:GO:0043631,GO:GO:0003723,GO:GO:0004652,GO:GO:0005634	AT4G32850.9	nuclear poly(a) polymerase	LOC_Os06g36360.4	expressed protein	2,50	-1.0	1	20	851	870	UGAAGUGUUUGGGGGAACUC	CAGUUCACCCAAAAACUUCA	Cleavage

tae-miR1130b-3p	Traes_7DL_CF6AABA1F.2						AT4G31430.3		LOC_Os06g40460.1	expressed protein	0,00	-1.0	1	21	1271	1291	UCUUAUAUUAUGGGACGGAGG	CCUCCGUCCCAUAAUAUAAGA	Cleavage
tae-miR1122c-3p	Traes_7DL_CF6AABA1F.2						AT4G31430.3		LOC_Os06g40460.1	expressed protein	1,50	-1.0	1	21	1271	1291	UCUAAUAUUAUGGGACGGAGG	CCUCCGUCCCAUAAUAUAAGA	Cleavage
tae-miR1128	Traes_7DL_D5FCF9F33.4								LOC_Os06g46480.1	expressed protein	2,50	-1.0	1	21	96	116	UACUACUCCCUCCGUCCGAAA	UUUGGGACGGAGGGAGUAUUA	Cleavage
tae-miR1122a	Traes_7DL_D5FCF9F33.4								LOC_Os06g46480.1	expressed protein	0,00	-1.0	1	20	23	42	UAGAUACAUCCGUAUCUAGA	UCUAGAUACGGAUGUAUCUA	Cleavage
tae-miR5049-3p	Traes_7DS_06CD5BBFB.4						AT3G21350.2	RNA polymerase transcriptional regulation mediator-related	LOC_Os06g11370.1	mediator of RNA polymerase II transcription subunit 6, putative, expressed	0,00	-1.0	1	21	99	119	AAUAUGGAUCGGAGGGAGUAC	UUACUCCCUCCGAUCCAUAUU	Cleavage
tae-miR1134	Traes_7DS_205267E87.1										2,50	-1.0	1	24	92	115	CAACAACAACAAGAAGAAGAAGAU	CUGUUCUUCUUCUUGUUGUUGUCU	Cleavage
tae-miR1130b-3p	Traes_7DS_25B02BA46.5	PF00005		3.6.3.44		GO:GO:0016887,GO:GO:0005524	AT3G53480.1	pleiotropic drug resistance 9	LOC_Os02g32690.1	pleiotropic drug resistance protein 15, putative, expressed	1,50	-1.0	1	21	730	750	UCUUAUAUUAUGGGACGGAGG	CCUCCGUCCCAUAAUGUAAGA	Cleavage
tae-miR1133	Traes_7DS_25F082B1E.8										3,00	-1.0	1	22	19	41	CAUAUACUCCCUCCGUCC-GAAA	UUUCCGGACGGAGGGAGUAUAUA	Cleavage
tae-miR9662a-3p	Traes_7DS_2B1FE04A6.1	PF02536				GO:GO:0006355,GO:GO:0005739,GO:GO:0003690	AT5G07900.1	Mitochondrial transcription termination factor family protein	LOC_Os06g12100.1	mTERF family protein, expressed	2,50	-1.0	1	21	561	581	UUGAACAUCCCAGAGCCACCG	CCGUGGCUCUGCGAUGUUCAG	Translation
tae-miR9662b-3p	Traes_7DS_2B1FE04A6.1	PF02536				GO:GO:0006355,GO:GO:0005739,GO:GO:0003690	AT5G07900.1	Mitochondrial transcription termination factor family protein	LOC_Os06g12100.1	mTERF family protein, expressed	2,50	-1.0	1	21	560	580	UGAACAUCCCAGAGCCACCGG	CCCGUGGCUCUGCGAUGUUCA	Cleavage
tae-miR1130b-3p	Traes_7DS_3C83C8EF9.1						AT4G31340.1	myosin heavy chain-related	LOC_Os08g40790.1	DNA repair ATPase-related, putative, expressed	1,00	-1.0	1	21	37	57	UCUUAUAUUAUGGGACGGAGG	CCUCCGUUCCAUAAUAUAAGA	Cleavage
tae-miR1122c-3p	Traes_7DS_3C83C8EF9.1						AT4G31340.1	myosin heavy chain-related	LOC_Os08g40790.1	DNA repair ATPase-related, putative, expressed	2,50	-1.0	1	21	37	57	UCUAAUAUUAUGGGACGGAGG	CCUCCGUUCCAUAAUAUAAGA	Cleavage
tae-miR1120c-5p	Traes_7DS_3C83C8EF9.1						AT4G31340.1	myosin heavy chain-related	LOC_Os08g40790.1	DNA repair ATPase-related, putative, expressed	1,50	-1.0	1	21	80	100	UAAUAUAAGAACGUUUUUGAC	GUCAAAAACGCUCUUAUAUUA	Translation
tae-miR1130b-3p	Traes_7DS_3C83C8EF9.3						AT4G31340.1	myosin heavy chain-related	LOC_Os08g40790.3	DNA repair ATPase-related, putative, expressed	1,00	-1.0	1	21	37	57	UCUUAUAUUAUGGGACGGAGG	CCUCCGUUCCAUAAUAUAAGA	Cleavage
tae-miR1122c-3p	Traes_7DS_3C83C8EF9.3						AT4G31340.1	myosin heavy chain-related	LOC_Os08g40790.3	DNA repair ATPase-related, putative, expressed	2,50	-1.0	1	21	37	57	UCUAAUAUUAUGGGACGGAGG	CCUCCGUUCCAUAAUAUAAGA	Cleavage
tae-miR1120c-5p	Traes_7DS_3C83C8EF9.3						AT4G31340.1	myosin heavy chain-related	LOC_Os08g40790.3	DNA repair ATPase-related, putative, expressed	1,50	-1.0	1	21	80	100	UAAUAUAAGAACGUUUUUGAC	GUCAAAAACGCUCUUAUAUUA	Translation
tae-miR9662a-3p	Traes_7DS_5B1210681.1	PF02536			K15032	GO:GO:0006355,GO:GO:0005739,GO:GO:0003690	AT1G21150.1	Mitochondrial transcription termination factor family protein	LOC_Os06g12100.1	mTERF family protein, expressed	2,50	-1.0	1	21	285	305	UUGAACAUCCCAGAGCCACCG	CCGUGGCUCUGCGAUGUUCAG	Translation
tae-miR9662b-3p	Traes_7DS_5B1210681.1	PF02536			K15032	GO:GO:0006355,GO:GO:0005739,GO:GO:0003690	AT1G21150.1	Mitochondrial transcription termination factor family protein	LOC_Os06g12100.1	mTERF family protein, expressed	2,50	-1.0	1	21	284	304	UGAACAUCCCAGAGCCACCGG	CCCGUGGCUCUGCGAUGUUCA	Cleavage
tae-miR9778	Traes_7DS_74534FCD3.1	PF00931				GO:GO:0043531	AT1G50180.1	NB-ARC domain-containing disease resistance protein	LOC_Os10g03570.1	RGH1A, putative, expressed	2,50	-1.0	1	21	111	131	UGCAUCAUCUCGAACUCGUCG	AGACGAGCUGGAGAUGAUGCA	Cleavage
tae-miR9778	Traes_7DS_74534FCD3.2	PF00931				GO:GO:0043531	AT3G46530.1	NB-ARC domain-containing disease resistance protein	LOC_Os10g03570.1	RGH1A, putative, expressed	2,50	-1.0	1	21	111	131	UGCAUCAUCUCGAACUCGUCG	AGACGAGCUGGAGAUGAUGCA	Cleavage
tae-miR9778	Traes_7DS_74534FCD3.3	PF00931				GO:GO:0043531	AT3G46530.1	NB-ARC domain-containing disease resistance protein	LOC_Os10g03570.1	RGH1A, putative, expressed	2,50	-1.0	1	21	111	131	UGCAUCAUCUCGAACUCGUCG	AGACGAGCUGGAGAUGAUGCA	Cleavage
tae-miR9778	Traes_7DS_74534FCD3.4	PF00931				GO:GO:0043531	AT3G46530.1	NB-ARC domain-containing disease resistance protein	LOC_Os10g03570.1	RGH1A, putative, expressed	2,50	-1.0	1	21	111	131	UGCAUCAUCUCGAACUCGUCG	AGACGAGCUGGAGAUGAUGCA	Cleavage
tae-miR9778	Traes_7DS_74534FCD3.5	PF00931				GO:GO:0043531	AT3G46530.1	NB-ARC domain-containing disease resistance protein	LOC_Os10g03570.1	RGH1A, putative, expressed	2,50	-1.0	1	21	111	131	UGCAUCAUCUCGAACUCGUCG	AGACGAGCUGGAGAUGAUGCA	Cleavage
tae-miR9778	Traes_7DS_74534FCD3.6	PF00931				GO:GO:0043531	AT3G46530.1	NB-ARC domain-containing disease resistance protein	LOC_Os10g03570.1	RGH1A, putative, expressed	2,50	-1.0	1	21	111	131	UGCAUCAUCUCGAACUCGUCG	AGACGAGCUGGAGAUGAUGCA	Cleavage
tae-miR1133	Traes_7DS_98F9E4E55.2	PF16211,PF00125	KOG1757			GO:GO:0003677	AT3G54560.1	histone H2A 11	LOC_Os03g53190.2	Core histone H2A/H2B/H3/H4 domain containing protein, putative, expressed	2,00	-1.0	1	22	2	24	CAUAUACUCCCUCCGUCCGA-AA	UUAUGGGACGGAGGGAGUAUAUA	Cleavage
tae-miR1122a	Traes_7DS_CA61ECF35.1						AT1G05060.1		LOC_Os06g13220.1	expressed protein	0,00	-1.0	1	20	33	52	UAGAUACAUCCGUAUCUAGA	UCUAGAUACGGAUGUAUCUA	Cleavage
tae-miR9775	Traes_7DS_D4DFDF981.1										2,00	-1.0	1	22	1609	1630	UGUGCGCAAUAAGAUUUUGCUA	GUGCAUGAUCUUAUUGCGCACA	Cleavage
tae-miR9775	Traes_7DS_D4DFDF981.2										2,00	-1.0	1	22	1702	1723	UGUGCGCAAUAAGAUUUUGCUA	GUGCAUGAUCUUAUUGCGCACA	Cleavage
tae-miR156	Traes_7DS_DBB29C7CD.1								LOC_Os08g41940.1	OsSPL16 - SBP-box gene family member, expressed	1,00	-1.0	1	21	359	379	UGACAGAAGAGAGUGAGCACA	UGUGCUCUCUCUCUUCUGUCA	Cleavage
tae-miR5049-3p	Traes_7DS_EFB144315.5	PF00076,PF04059	KOG4660			GO:GO:0003676	AT1G29400.2	MEI2-like protein 5	LOC_Os02g48790.5	AML1, putative, expressed	1,50	-1.0	1	21	2298	2318	AAUAUGGAUCGGAGGGAGUAC	UUACUCCCUCUGAUCCAUAUU	Translation

Triticum turgidum_Control_Root

miRNA_Acc.	Target_Acc.	Pfam	KOG	KEGG/ec	KO	GO	Best-hit-arabi-name	arabi-define	Best-hit-rice-name	rice-define	Expectation	UPE$	miRNA_start	miRNA_end	Target_start	Target_end	miRNA_aligned_fragment	Target_aligned_fragment	Inhibition
tae-miR159b	Traes_1AL_041EBB4A6.1						AT2G32460.1	myb domain protein 101	LOC_Os01g59660.2	MYB family transcription factor, putative, expressed	2,05	-1.0	1	21	488	508	UUUGGAUUGAAGGGAGCUCUG	UGGAGCUCCCUUCAUUCCAAU	Cleavage
tae-miR159a	Traes_1AL_041EBB4A6.1						AT2G32460.1	myb domain protein 101	LOC_Os01g59660.2	MYB family transcription factor, putative, expressed	2,05	-1.0	1	21	488	508	UUUGGAUUGAAGGGAGCUCUG	UGGAGCUCCCUUCAUUCCAAU	Cleavage
tae-miR159b	Traes_1AL_041EBB4A6.2						AT2G32460.1	myb domain protein 101	LOC_Os01g59660.2	MYB family transcription factor, putative, expressed	2,05	-1.0	1	21	512	532	UUUGGAUUGAAGGGAGCUCUG	UGGAGCUCCCUUCAUUCCAAU	Cleavage
tae-miR159a	Traes_1AL_041EBB4A6.2						AT2G32460.1	myb domain protein 101	LOC_Os01g59660.2	MYB family transcription factor, putative, expressed	2,05	-1.0	1	21	512	532	UUUGGAUUGAAGGGAGCUCUG	UGGAGCUCCCUUCAUUCCAAU	Cleavage
tae-miR159b	Traes_1AL_041EBB4A6.3						AT2G32460.1	myb domain protein 101	LOC_Os01g59660.2	MYB family transcription factor, putative, expressed	2,05	-1.0	1	21	488	508	UUUGGAUUGAAGGGAGCUCUG	UGGAGCUCCCUUCAUUCCAAU	Cleavage
tae-miR159a	Traes_1AL_041EBB4A6.3						AT2G32460.1	myb domain protein 101	LOC_Os01g59660.2	MYB family transcription factor, putative, expressed	2,05	-1.0	1	21	488	508	UUUGGAUUGAAGGGAGCUCUG	UGGAGCUCCCUUCAUUCCAAU	Cleavage
tae-miR159b	Traes_1AL_041EBB4A6.4								LOC_Os01g59660.2	MYB family transcription factor, putative, expressed	2,05	-1.0	1	21	410	430	UUUGGAUUGAAGGGAGCUCUG	UGGAGCUCCCUUCAUUCCAAU	Cleavage
tae-miR159a	Traes_1AL_041EBB4A6.4								LOC_Os01g59660.2	MYB family transcription factor, putative, expressed	2,05	-1.0	1	21	410	430	UUUGGAUUGAAGGGAGCUCUG	UGGAGCUCCCUUCAUUCCAAU	Cleavage
tae-miR160	Traes_1AL_147CF243C.1	PF06507				GO:GO:0009725,GO:GO:0006355,GO:GO:0005634,GO:GO:0003677	AT4G30080.1	auxin response factor 16	LOC_Os10g33940.1	auxin response factor 18, putative, expressed	0,00	-1.0	1	21	642	662	UGCCUGGCUCCCUGUAUGCCA	AGGCAUACAGGGAGCCAGGCA	Cleavage
tae-miR160	Traes_1AL_147CF243C.2						AT4G30080.1	auxin response factor 16	LOC_Os10g33940.1	auxin response factor 18, putative, expressed	0,00	-1.0	1	21	267	287	UGCCUGGCUCCCUGUAUGCCA	AGGCAUACAGGGAGCCAGGCA	Cleavage
tae-miR1127b-3p	Traes_1AL_4A458DF14.2	PF13812,PF01535,PF13041					AT3G62470.1	Pentatricopeptide repeat (PPR) superfamily protein	LOC_Os05g28720.1	PPR repeat containing protein, expressed	1,05	-1.0	1	21	1738	1758	ACAAGUAUUUCUGGACGGAGG	CCUCCGUCCGGAAAUACUUGU	Cleavage
tae-miR1130b-3p	Traes_1AL_51B1D5DBC.6	PF07714		2.7.11.1		GO:GO:0006468,GO:GO:0004672	AT5G14640.1	shaggy-like kinase 13	LOC_Os10g37740.1	CGMC_GSK.9 - CGMC includes CDA, MAPK, GSK3, and CLKC kinases, expressed	0,00	-1.0	1	21	914	934	UCUUAUAUUAUGGGACGGAGG	CCUCCGUCCCAUAAUAUAAGA	Cleavage
tae-miR1122c-3p	Traes_1AL_51B1D5DBC.6	PF07714		2.7.11.1		GO:GO:0006468,GO:GO:0004672	AT5G14640.1	shaggy-like kinase 13	LOC_Os10g37740.1	CGMC_GSK.9 - CGMC includes CDA, MAPK, GSK3, and CLKC kinases, expressed	1,05	-1.0	1	21	914	934	UCUAAUAUUAUGGGACGGAGG	CCUCCGUCCCAUAAUAUAAGA	Cleavage
tae-miR5049-3p	Traes_1AL_7D6DC73FC.3	PF14531	KOG0583	2.7.11.1			AT3G01090.2	SNF1 kinase homolog 10	LOC_Os05g45420.2	CAMK_KIN1/SNF1/Nim1_like_AMPKh.3 - CAMK includes calcium/calmodulin depedent protein kinases, expressed	2,00	-1.0	1	21	1256	1276	AAUAUGGAUCGGAGGGAGUAC	UUUCUCCCUCCGAUCCAUAUA	Cleavage
tae-miR1128	Traes_1AL_968B97E50.2	PF06480,PF00004,PF01434			K08956	GO:GO:0016021,GO:GO:0008270,GO:GO:0005524,GO:GO:0004222,GO:GO:0006508	AT1G07510.1	FTSH protease 10	LOC_Os05g38400.3	OsFtsH8 FtsH protease, homologue of AtFtsH3/10, expressed	3,00	-1.0	1	21	100	121	UACUACUCCCUCCGUCC-GAAA	UUUCCGGACGGAGGGAGUAGUU	Cleavage
tae-miR7757-5p	Traes_1AL_B6931117F.1	PF00931				GO:GO:0043531	AT3G46530.1	NB-ARC domain-containing disease resistance protein	LOC_Os08g10440.1	NBS-LRR disease resistance protein, putative, expressed	1,05	-1.0	1	22	1301	1322	AUAAAACCUUCAGCUAUCCAUC	AAUGGAUAGCUGAAGGCUUUAU	Cleavage
tae-miR7757-5p	Traes_1AL_B6931117F.2	PF00931				GO:GO:0043531	AT3G46530.1	NB-ARC domain-containing disease resistance protein	LOC_Os08g10440.1	NBS-LRR disease resistance protein, putative, expressed	1,05	-1.0	1	22	1343	1364	AUAAAACCUUCAGCUAUCCAUC	AAUGGAUAGCUGAAGGCUUUAU	Cleavage
tae-miR7757-5p	Traes_1AL_B6931117F.3	PF00931				GO:GO:0043531	AT3G46530.1	NB-ARC domain-containing disease resistance protein	LOC_Os08g10440.1	NBS-LRR disease resistance protein, putative, expressed	1,05	-1.0	1	22	1301	1322	AUAAAACCUUCAGCUAUCCAUC	AAUGGAUAGCUGAAGGCUUUAU	Cleavage
tae-miR7757-5p	Traes_1AL_B6931117F.4	PF00931				GO:GO:0043531	AT3G46530.1	NB-ARC domain-containing disease resistance protein	LOC_Os08g10440.1	NBS-LRR disease resistance protein, putative, expressed	1,05	-1.0	1	22	1301	1322	AUAAAACCUUCAGCUAUCCAUC	AAUGGAUAGCUGAAGGCUUUAU	Cleavage
tae-miR9778	Traes_1AS_17306388F.1	PF00931				GO:GO:0043531	AT3G07040.1	NB-ARC domain-containing disease resistance protein	LOC_Os08g42670.2	resistance protein, putative, expressed	2,00	-1.0	1	21	126	146	UGCAUCAUCUCGAACUCGUCG	CAAUGAGCUCGAGAUGAUGCA	Cleavage
tae-miR9778	Traes_1AS_17306388F.3	PF00931				GO:GO:0043531	AT3G46530.1	NB-ARC domain-containing disease resistance protein	LOC_Os12g37290.1	resistance protein T10rga2-1A, putative, expressed	2,00	-1.0	1	21	126	146	UGCAUCAUCUCGAACUCGUCG	CAAUGAGCUCGAGAUGAUGCA	Cleavage
tae-miR1135	Traes_1AS_4A925522B.4	PF13920					AT5G01960.1	RING/U-box superfamily protein	LOC_Os01g07390.1	zinc finger, C3HC4 type family protein, expressed	0,00	-1.0	1	24	511	534	CUGCGACAAGUAAUUCCGAACGGA	UCCGUUCGGAAUUACUUGUCGCAG	Cleavage
tae-miR6197-5p	Traes_1AS_5DCFDC258.1	PF04578,PF13968					AT5G45540.1	Protein of unknown function (DUF594)	LOC_Os07g08270.1	expressed protein	3,00	-1.0	1	21	1353	1376	UCUGUAAACAAAUGUA---GGACG	ACUCCAACUACAUUUGUUUACAGA	Cleavage
tae-miR1137a	Traes_1AS_8912BD052.27										0,00	-1.0	1	20	136	155	UAGUACAAAGUUGAGUCAUC	GAUGACUCAACUUUGUACUA	Cleavage
tae-miR9674a-5p	Traes_1AS_92A698B19.1	PF01535,PF13041			K17964		AT1G63130.1	Tetratricopeptide repeat (TPR)-like superfamily protein	LOC_Os04g28300.1	Rf1, mitochondrial precursor, putative, expressed	2,05	-1.0	1	21	1761	1781	GCAUCAUCCAUCCUACCAUUC	AAAUGGAAGGAUCGAUGAUGC	Cleavage
tae-miR9674a-5p	Traes_1AS_92A698B19.10	PF01535,PF13041					AT1G63130.1	Tetratricopeptide repeat (TPR)-like superfamily protein	LOC_Os04g28300.1	Rf1, mitochondrial precursor, putative, expressed	2,05	-1.0	1	21	1761	1781	GCAUCAUCCAUCCUACCAUUC	AAAUGGAAGGAUCGAUGAUGC	Cleavage
tae-miR9674a-5p	Traes_1AS_92A698B19.12	PF13041					AT1G12300.1	Tetratricopeptide repeat (TPR)-like superfamily protein	LOC_Os04g28300.1	Rf1, mitochondrial precursor, putative, expressed	2,05	-1.0	1	21	96	116	GCAUCAUCCAUCCUACCAUUC	AAAUGGAAGGAUCGAUGAUGC	Cleavage
tae-miR9674a-5p	Traes_1AS_92A698B19.2	PF01535,PF13041			K17964		AT1G63130.1	Tetratricopeptide repeat (TPR)-like superfamily protein	LOC_Os04g28300.1	Rf1, mitochondrial precursor, putative, expressed	2,05	-1.0	1	21	1761	1781	GCAUCAUCCAUCCUACCAUUC	AAAUGGAAGGAUCGAUGAUGC	Cleavage
tae-miR9674a-5p	Traes_1AS_92A698B19.3	PF01535,PF13041			K17964		AT1G63130.1	Tetratricopeptide repeat (TPR)-like superfamily protein	LOC_Os04g28300.1	Rf1, mitochondrial precursor, putative, expressed	2,05	-1.0	1	21	1761	1781	GCAUCAUCCAUCCUACCAUUC	AAAUGGAAGGAUCGAUGAUGC	Cleavage
tae-miR9674a-5p	Traes_1AS_92A698B19.4	PF01535,PF13041			K17964		AT1G63130.1	Tetratricopeptide repeat (TPR)-like superfamily protein	LOC_Os04g28300.1	Rf1, mitochondrial precursor, putative, expressed	2,05	-1.0	1	21	1761	1781	GCAUCAUCCAUCCUACCAUUC	AAAUGGAAGGAUCGAUGAUGC	Cleavage
tae-miR9674a-5p	Traes_1AS_92A698B19.5	PF01535,PF13041			K17964		AT1G63130.1	Tetratricopeptide repeat (TPR)-like superfamily protein	LOC_Os04g28300.1	Rf1, mitochondrial precursor, putative, expressed	2,05	-1.0	1	21	1761	1781	GCAUCAUCCAUCCUACCAUUC	AAAUGGAAGGAUCGAUGAUGC	Cleavage
tae-miR9674a-5p	Traes_1AS_92A698B19.6	PF01535,PF13041			K17964		AT1G63130.1	Tetratricopeptide repeat (TPR)-like superfamily protein	LOC_Os04g28300.1	Rf1, mitochondrial precursor, putative, expressed	2,05	-1.0	1	21	1761	1781	GCAUCAUCCAUCCUACCAUUC	AAAUGGAAGGAUCGAUGAUGC	Cleavage
tae-miR9674a-5p	Traes_1AS_92A698B19.7	PF01535,PF13041			K17964		AT1G63130.1	Tetratricopeptide repeat (TPR)-like superfamily protein	LOC_Os04g28300.1	Rf1, mitochondrial precursor, putative, expressed	2,05	-1.0	1	21	1761	1781	GCAUCAUCCAUCCUACCAUUC	AAAUGGAAGGAUCGAUGAUGC	Cleavage
tae-miR9674a-5p	Traes_1AS_92A698B19.8	PF01535,PF13041			K17964		AT1G63130.1	Tetratricopeptide repeat (TPR)-like superfamily protein	LOC_Os04g28300.1	Rf1, mitochondrial precursor, putative, expressed	2,05	-1.0	1	21	1761	1781	GCAUCAUCCAUCCUACCAUUC	AAAUGGAAGGAUCGAUGAUGC	Cleavage
tae-miR9674a-5p	Traes_1AS_92A698B19.9	PF01535,PF13041			K17964		AT1G63130.1	Tetratricopeptide repeat (TPR)-like superfamily protein	LOC_Os04g28300.1	Rf1, mitochondrial precursor, putative, expressed	2,05	-1.0	1	21	1761	1781	GCAUCAUCCAUCCUACCAUUC	AAAUGGAAGGAUCGAUGAUGC	Cleavage
tae-miR5049-3p	Traes_1AS_A482D4FF9.1	PF04997		2.7.7.6		GO:GO:0006351,GO:GO:0003899,GO:GO:0003677	AT1G63020.1	nuclear RNA polymerase D1A	LOC_Os02g05880.1	RNA polymerase Rpb1, domain 2 family protein, expressed	0,00	-1.0	1	21	557	577	AAUAUGGAUCGGAGGGAGUAC	GUACUCCCUCCGAUCCAUAUU	Cleavage
tae-miR9778	Traes_1AS_C7A8188D1.1	PF13191,PF00931	KOG4658			GO:GO:0043531	AT1G58390.1	Disease resistance protein (CC-NBS-LRR class) family	LOC_Os08g42670.2	resistance protein, putative, expressed	1,00	-1.0	1	21	126	146	UGCAUCAUCUCGAACUCGUCG	CAACGAGCUCGAGAUGAUGCA	Cleavage
tae-miR9778	Traes_1AS_C7A8188D1.3	PF00931				GO:GO:0043531	AT3G46530.1	NB-ARC domain-containing disease resistance protein	LOC_Os02g57310.1	pib, putative, expressed	1,00	-1.0	1	21	126	146	UGCAUCAUCUCGAACUCGUCG	CAACGAGCUCGAGAUGAUGCA	Cleavage
tae-miR1128	Traes_1BL_087CA0963.1	PF16876,PF08235	KOG2116	3.1.3.4			AT3G09560.1	Lipin family protein	LOC_Os05g38710.1	lipin, N-terminal conserved region family protein, expressed	3,00	-1.0	1	21	2048	2069	UACUACUCCCUCCG-UCCGAAA	UUUAGGAACGGAGGGAGUAGUA	Cleavage
tae-miR1128	Traes_1BL_0FDC38712.1	PF13639				GO:GO:0008270,GO:GO:0005515	AT1G17970.1	RING/U-box superfamily protein	LOC_Os05g47670.1	zinc finger, C3HC4 type domain containing protein, expressed	1,05	-1.0	1	21	225	245	UACUACUCCCUCCGUCCGAAA	UUUCGGACGGAGGGAGUACUA	Cleavage
tae-miR1127b-3p	Traes_1BL_0FDC38712.1	PF13639				GO:GO:0008270,GO:GO:0005515	AT1G17970.1	RING/U-box superfamily protein	LOC_Os05g47670.1	zinc finger, C3HC4 type domain containing protein, expressed	1,05	-1.0	1	21	121	141	ACAAGUAUUUCUGGACGGAGG	CCUCCGUCCGGAAAUACUUGU	Cleavage
tae-miR5062-5p	Traes_1BL_219B2BB6D.16						AT2G46060.1	transmembrane protein-related	LOC_Os10g30910.2	transmembrane protein-related, putative, expressed	2,05	-1.0	1	23	836	858	UGAACCUUAGGGAACAGCCGCAU	UUUUGGAUGUUCCCCAAGGUUCA	Cleavage
tae-miR5062-5p	Traes_1BL_219B2BB6D.17						AT2G46060.1	transmembrane protein-related	LOC_Os10g30910.2	transmembrane protein-related, putative, expressed	2,05	-1.0	1	23	836	858	UGAACCUUAGGGAACAGCCGCAU	UUUUGGAUGUUCCCCAAGGUUCA	Cleavage
tae-miR5062-5p	Traes_1BL_219B2BB6D.18						AT2G46060.1	transmembrane protein-related	LOC_Os10g30910.2	transmembrane protein-related, putative, expressed	2,05	-1.0	1	23	836	858	UGAACCUUAGGGAACAGCCGCAU	UUUUGGAUGUUCCCCAAGGUUCA	Cleavage
tae-miR160	Traes_1BL_54CD82AC3.1	PF06507				GO:GO:0009725,GO:GO:0006355,GO:GO:0005634,GO:GO:0003677	AT4G30080.1	auxin response factor 16	LOC_Os10g33940.1	auxin response factor 18, putative, expressed	0,00	-1.0	1	21	642	662	UGCCUGGCUCCCUGUAUGCCA	AGGCAUACAGGGAGCCAGGCA	Cleavage
tae-miR160	Traes_1BL_54CD82AC3.2						AT4G30080.1	auxin response factor 16	LOC_Os10g33940.1	auxin response factor 18, putative, expressed	0,00	-1.0	1	21	369	389	UGCCUGGCUCCCUGUAUGCCA	AGGCAUACAGGGAGCCAGGCA	Cleavage
tae-miR160	Traes_1BL_54CD82AC3.3						AT4G30080.1	auxin response factor 16	LOC_Os10g33940.1	auxin response factor 18, putative, expressed	0,00	-1.0	1	21	267	287	UGCCUGGCUCCCUGUAUGCCA	AGGCAUACAGGGAGCCAGGCA	Cleavage
tae-miR1130b-3p	Traes_1BL_89E785AAA.2	PF00436	KOG1653			GO:GO:0003697,GO:GO:0006260	AT4G11060.1	mitochondrially targeted single-stranded DNA binding protein	LOC_Os05g43440.2	single-stranded DNA-binding protein, putative, expressed	2,00	-1.0	1	21	591	611	UCUUAUAUUAUGGGACGGAGG	CCUCUGUCCCAUAAUAUAAGG	Cleavage
tae-miR159b	Traes_1BL_CF98E922B.1	PF00249					AT5G06100.2	myb domain protein 33	LOC_Os01g59660.2	MYB family transcription factor, putative, expressed	2,05	-1.0	1	21	839	859	UUUGGAUUGAAGGGAGCUCUG	UGGAGCUCCCUUCACUCCAAU	Cleavage
tae-miR159a	Traes_1BL_CF98E922B.1	PF00249					AT5G06100.2	myb domain protein 33	LOC_Os01g59660.2	MYB family transcription factor, putative, expressed	2,05	-1.0	1	21	839	859	UUUGGAUUGAAGGGAGCUCUG	UGGAGCUCCCUUCACUCCAAU	Cleavage
tae-miR159b	Traes_1BL_CF98E922B.2	PF00249					AT5G06100.3	myb domain protein 33	LOC_Os01g59660.1	MYB family transcription factor, putative, expressed	2,05	-1.0	1	21	839	859	UUUGGAUUGAAGGGAGCUCUG	UGGAGCUCCCUUCACUCCAAU	Cleavage
tae-miR159a	Traes_1BL_CF98E922B.2	PF00249					AT5G06100.3	myb domain protein 33	LOC_Os01g59660.1	MYB family transcription factor, putative, expressed	2,05	-1.0	1	21	839	859	UUUGGAUUGAAGGGAGCUCUG	UGGAGCUCCCUUCACUCCAAU	Cleavage
tae-miR159b	Traes_1BL_CF98E922B.3						AT5G06100.3	myb domain protein 33	LOC_Os01g59660.2	MYB family transcription factor, putative, expressed	2,05	-1.0	1	21	512	532	UUUGGAUUGAAGGGAGCUCUG	UGGAGCUCCCUUCACUCCAAU	Cleavage
tae-miR159a	Traes_1BL_CF98E922B.3						AT5G06100.3	myb domain protein 33	LOC_Os01g59660.2	MYB family transcription factor, putative, expressed	2,05	-1.0	1	21	512	532	UUUGGAUUGAAGGGAGCUCUG	UGGAGCUCCCUUCACUCCAAU	Cleavage
tae-miR159b	Traes_1BL_CF98E922B.4						AT5G06100.1	myb domain protein 33	LOC_Os01g59660.1	MYB family transcription factor, putative, expressed	2,05	-1.0	1	21	512	532	UUUGGAUUGAAGGGAGCUCUG	UGGAGCUCCCUUCACUCCAAU	Cleavage
tae-miR159a	Traes_1BL_CF98E922B.4						AT5G06100.1	myb domain protein 33	LOC_Os01g59660.1	MYB family transcription factor, putative, expressed	2,05	-1.0	1	21	512	532	UUUGGAUUGAAGGGAGCUCUG	UGGAGCUCCCUUCACUCCAAU	Cleavage
tae-miR1117	Traes_1BL_F01A87F44.1	PF13359					AT2G13770.1		LOC_Os01g33990.1	transposon protein, putative, CACTA, En/Spm sub-class, expressed	2,05	-1.0	1	24	37	60	UAGUACCGGUUCGUGGCACGAACC	GGUUCGUGGCACGAACCGGUGCUA	Cleavage
tae-miR1131	Traes_1BL_F01A87F44.1	PF13359					AT2G13770.1		LOC_Os01g33990.1	transposon protein, putative, CACTA, En/Spm sub-class, expressed	2,05	-1.0	1	22	63	84	UAGUACCGGUUCGUGGCUAACC	GGUUAGCCACGAACCGGUAAUG	Cleavage
tae-miR1122b-3p	Traes_1BS_34C60A54E.1	PF00651				GO:GO:0005515	AT5G19000.1	BTB-POZ and MATH domain 1	LOC_Os08g41120.1	MBTB29 - Bric-a-Brac, Tramtrack, Broad Complex BTB domain with Meprin and TRAF Homology MATH domain, expressed	1,05	-1.0	1	21	47	67	AGACUUAUAUGUAGGAACGGA	UCCGUUCCUAAAUAUAAGUCU	Translation
tae-miR9662a-3p	Traes_1BS_566CEE7F6.1	PF02536				GO:GO:0006355,GO:GO:0005739,GO:GO:0003690	AT1G21150.1	Mitochondrial transcription termination factor family protein	LOC_Os06g12100.1	mTERF family protein, expressed	2,00	-1.0	1	21	441	461	UUGAACAUCCCAGAGCCACCG	UCGUGGCGCUGGGAUGUUCAG	Cleavage
tae-miR9662b-3p	Traes_1BS_566CEE7F6.1	PF02536				GO:GO:0006355,GO:GO:0005739,GO:GO:0003690	AT1G21150.1	Mitochondrial transcription termination factor family protein	LOC_Os06g12100.1	mTERF family protein, expressed	2,05	-1.0	1	21	440	460	UGAACAUCCCAGAGCCACCGG	CUCGUGGCGCUGGGAUGUUCA	Cleavage
tae-miR9662a-3p	Traes_1BS_566CEE7F6.2	PF02536				GO:GO:0006355,GO:GO:0005739,GO:GO:0003690	AT1G21150.1	Mitochondrial transcription termination factor family protein	LOC_Os06g12100.1	mTERF family protein, expressed	2,00	-1.0	1	21	66	86	UUGAACAUCCCAGAGCCACCG	UCGUGGCGCUGGGAUGUUCAG	Cleavage
tae-miR9662b-3p	Traes_1BS_566CEE7F6.2	PF02536				GO:GO:0006355,GO:GO:0005739,GO:GO:0003690	AT1G21150.1	Mitochondrial transcription termination factor family protein	LOC_Os06g12100.1	mTERF family protein, expressed	2,05	-1.0	1	21	65	85	UGAACAUCCCAGAGCCACCGG	CUCGUGGCGCUGGGAUGUUCA	Cleavage
tae-miR9778	Traes_1BS_70B733D34.1	PF13191,PF00931				GO:GO:0043531	AT3G07040.1	NB-ARC domain-containing disease resistance protein	LOC_Os08g42670.1	resistance protein, putative, expressed	2,00	-1.0	1	21	126	146	UGCAUCAUCUCGAACUCGUCG	CAAUGAGCUCGAGAUGAUGCA	Cleavage
tae-miR9778	Traes_1BS_70B733D34.2	PF13191,PF00931				GO:GO:0043531	AT3G07040.1	NB-ARC domain-containing disease resistance protein	LOC_Os08g42670.2	resistance protein, putative, expressed	2,00	-1.0	1	21	126	146	UGCAUCAUCUCGAACUCGUCG	CAAUGAGCUCGAGAUGAUGCA	Cleavage
tae-miR5049-3p	Traes_1BS_7EC2BDE95.1	PF16760		2.4.1.21			AT1G11720.2	starch synthase 3	LOC_Os08g09230.1	starch synthase III, putative, expressed	1,05	-1.0	1	21	3430	3450	AAUAUGGAUCGGAGGGAGUAC	CUACUCCCUCCAAUCCAUAUU	Translation
tae-miR5049-3p	Traes_1BS_B0BB95B97.6	PF04997		2.7.7.6		GO:GO:0006351,GO:GO:0003899,GO:GO:0003677	AT1G63020.2	nuclear RNA polymerase D1A	LOC_Os02g05880.1	RNA polymerase Rpb1, domain 2 family protein, expressed	0,00	-1.0	1	21	623	643	AAUAUGGAUCGGAGGGAGUAC	GUACUCCCUCCGAUCCAUAUU	Cleavage
tae-miR7757-5p	Traes_1BS_CF1C4F54E.2	PF00931				GO:GO:0043531	AT1G09920.1	TRAF-type zinc finger-related	LOC_Os11g45750.1	WRKY125, expressed	2,05	-1.0	1	22	119	140	AUAAAACCUUCAGCUAUCCAUC	AAUGGUUAGCUGAAGGUUUCAU	Cleavage
tae-miR159b	Traes_1DL_25540DE53.1						AT2G32460.1	myb domain protein 101	LOC_Os01g59660.2	MYB family transcription factor, putative, expressed	2,05	-1.0	1	21	512	532	UUUGGAUUGAAGGGAGCUCUG	UGGAGCUCCCUUCAUUCCAAU	Cleavage
tae-miR159a	Traes_1DL_25540DE53.1						AT2G32460.1	myb domain protein 101	LOC_Os01g59660.2	MYB family transcription factor, putative, expressed	2,05	-1.0	1	21	512	532	UUUGGAUUGAAGGGAGCUCUG	UGGAGCUCCCUUCAUUCCAAU	Cleavage
tae-miR159b	Traes_1DL_25540DE53.2						AT2G32460.1	myb domain protein 101	LOC_Os01g59660.1	MYB family transcription factor, putative, expressed	2,05	-1.0	1	21	512	532	UUUGGAUUGAAGGGAGCUCUG	UGGAGCUCCCUUCAUUCCAAU	Cleavage
tae-miR159a	Traes_1DL_25540DE53.2						AT2G32460.1	myb domain protein 101	LOC_Os01g59660.1	MYB family transcription factor, putative, expressed	2,05	-1.0	1	21	512	532	UUUGGAUUGAAGGGAGCUCUG	UGGAGCUCCCUUCAUUCCAAU	Cleavage
tae-miR159b	Traes_1DL_25540DE53.3						AT5G06100.3	myb domain protein 33	LOC_Os01g59660.1	MYB family transcription factor, putative, expressed	2,05	-1.0	1	21	470	490	UUUGGAUUGAAGGGAGCUCUG	UGGAGCUCCCUUCAUUCCAAU	Cleavage
tae-miR159a	Traes_1DL_25540DE53.3						AT5G06100.3	myb domain protein 33	LOC_Os01g59660.1	MYB family transcription factor, putative, expressed	2,05	-1.0	1	21	470	490	UUUGGAUUGAAGGGAGCUCUG	UGGAGCUCCCUUCAUUCCAAU	Cleavage
tae-miR159b	Traes_1DL_25540DE53.4						AT5G06100.3	myb domain protein 33	LOC_Os01g59660.2	MYB family transcription factor, putative, expressed	2,05	-1.0	1	21	470	490	UUUGGAUUGAAGGGAGCUCUG	UGGAGCUCCCUUCAUUCCAAU	Cleavage
tae-miR159a	Traes_1DL_25540DE53.4						AT5G06100.3	myb domain protein 33	LOC_Os01g59660.2	MYB family transcription factor, putative, expressed	2,05	-1.0	1	21	470	490	UUUGGAUUGAAGGGAGCUCUG	UGGAGCUCCCUUCAUUCCAAU	Cleavage
tae-miR159b	Traes_1DL_25540DE53.5						AT5G06100.2	myb domain protein 33	LOC_Os01g59660.2	MYB family transcription factor, putative, expressed	2,05	-1.0	1	21	470	490	UUUGGAUUGAAGGGAGCUCUG	UGGAGCUCCCUUCAUUCCAAU	Cleavage
tae-miR159a	Traes_1DL_25540DE53.5						AT5G06100.2	myb domain protein 33	LOC_Os01g59660.2	MYB family transcription factor, putative, expressed	2,05	-1.0	1	21	470	490	UUUGGAUUGAAGGGAGCUCUG	UGGAGCUCCCUUCAUUCCAAU	Cleavage
tae-miR159b	Traes_1DL_25540DE53.6						AT5G06100.2	myb domain protein 33	LOC_Os01g59660.1	MYB family transcription factor, putative, expressed	2,05	-1.0	1	21	470	490	UUUGGAUUGAAGGGAGCUCUG	UGGAGCUCCCUUCAUUCCAAU	Cleavage
tae-miR159a	Traes_1DL_25540DE53.6						AT5G06100.2	myb domain protein 33	LOC_Os01g59660.1	MYB family transcription factor, putative, expressed	2,05	-1.0	1	21	470	490	UUUGGAUUGAAGGGAGCUCUG	UGGAGCUCCCUUCAUUCCAAU	Cleavage
tae-miR1137a	Traes_1DL_34A359364.2	PF09439	KOG0070		K07937		AT5G14670.1	ADP-ribosylation factor A1B	LOC_Os03g59740.1	ADP-ribosylation factor, putative, expressed	2,05	-1.0	1	20	516	535	UAGUACAAAGUUGAGUCAUC	GAUGACCCAAUUUUGUACUA	Translation
tae-miR5062-5p	Traes_1DL_38415F71A.19						AT2G46060.2	transmembrane protein-related	LOC_Os10g30910.2	transmembrane protein-related, putative, expressed	2,05	-1.0	1	23	836	858	UGAACCUUAGGGAACAGCCGCAU	UUUUGGAUGUUCCCCAAGGUUCA	Cleavage
tae-miR5062-5p	Traes_1DL_38415F71A.20						AT2G46060.2	transmembrane protein-related	LOC_Os10g30910.2	transmembrane protein-related, putative, expressed	2,05	-1.0	1	23	836	858	UGAACCUUAGGGAACAGCCGCAU	UUUUGGAUGUUCCCCAAGGUUCA	Cleavage
tae-miR5062-5p	Traes_1DL_38415F71A.22						AT2G46060.1	transmembrane protein-related	LOC_Os10g30910.2	transmembrane protein-related, putative, expressed	2,05	-1.0	1	23	836	858	UGAACCUUAGGGAACAGCCGCAU	UUUUGGAUGUUCCCCAAGGUUCA	Cleavage
tae-miR1122a	Traes_1DL_38B88249C.2	PF00069	KOG0580	2.7.11.1		GO:GO:0006468,GO:GO:0005524,GO:GO:0004672	AT2G42960.1	Protein kinase superfamily protein	LOC_Os10g35450.1	serine/threonine-protein kinase, putative, expressed	0,00	-1.0	1	20	976	995	UAGAUACAUCCGUAUCUAGA	UCUAGAUACGGAUGUAUCUA	Cleavage
tae-miR1120a	Traes_1DL_6A91BC5A5.1	PF08652			K14845		AT4G17620.1	glycine-rich protein	LOC_Os05g32580.1	glycine-rich protein, putative, expressed	2,05	-1.0	1	24	25	48	ACAUUCUUAUAUUAUGAGACGGAG	CUCCGUCCCAUAAUAUAAGAACGU	Cleavage
tae-miR1130b-3p	Traes_1DL_6A91BC5A5.1	PF08652			K14845		AT4G17620.1	glycine-rich protein	LOC_Os05g32580.1	glycine-rich protein, putative, expressed	0,00	-1.0	1	21	24	44	UCUUAUAUUAUGGGACGGAGG	CCUCCGUCCCAUAAUAUAAGA	Cleavage
tae-miR1120b-3p	Traes_1DL_6A91BC5A5.1	PF08652			K14845		AT4G17620.1	glycine-rich protein	LOC_Os05g32580.1	glycine-rich protein, putative, expressed	2,05	-1.0	1	21	25	45	UUCUUAUAUUGUGGGACAGAG	CUCCGUCCCAUAAUAUAAGAA	Translation
tae-miR1122c-3p	Traes_1DL_6A91BC5A5.1	PF08652			K14845		AT4G17620.1	glycine-rich protein	LOC_Os05g32580.1	glycine-rich protein, putative, expressed	1,05	-1.0	1	21	24	44	UCUAAUAUUAUGGGACGGAGG	CCUCCGUCCCAUAAUAUAAGA	Cleavage
tae-miR1120c-5p	Traes_1DL_6A91BC5A5.1	PF08652			K14845		AT4G17620.1	glycine-rich protein	LOC_Os05g32580.1	glycine-rich protein, putative, expressed	2,05	-1.0	1	21	67	87	UAAUAUAAGAACGUUUUUGAC	GUCAAACACGCUCUUAUAUUA	Translation
tae-miR160	Traes_1DL_AEC2F3FB3.1	PF06507				GO:GO:0009725,GO:GO:0006355,GO:GO:0005634,GO:GO:0003677	AT4G30080.1	auxin response factor 16	LOC_Os10g33940.1	auxin response factor 18, putative, expressed	0,00	-1.0	1	21	642	662	UGCCUGGCUCCCUGUAUGCCA	AGGCAUACAGGGAGCCAGGCA	Cleavage
tae-miR1122b-3p	Traes_1DL_B5E6E273B.1										1,05	-1.0	1	21	35	55	AGACUUAUAUGUAGGAACGGA	UCCGUUCCUAAAUAUAAGUCU	Translation
tae-miR5175-5p	Traes_1DL_C1BE1742F.2	PF03641					AT3G53450.1	Putative lysine decarboxylase family protein	LOC_Os10g33900.1	possible lysine decarboxylase domain containing protein, expressed	2,00	-1.0	1	21	109	130	UUCCAAAUUACUCGUCG-UGGU	UUCAGCGACGAGUAAUUUGGAA	Cleavage
tae-miR1133	Traes_1DL_D391AE3DC.1	PF16212		3.6.3.1		GO:GO:0016021,GO:GO:0015914,GO:GO:0005524,GO:GO:0004012,GO:GO:0000287	AT1G59820.1	aminophospholipid ATPase 3	LOC_Os10g27220.1	phospholipid-transporting ATPase 3, putative, expressed	2,00	-1.0	1	22	858	879	CAUAUACUCCCUCCGUCCGAAA	AAAUGAACGGAGGGAGUAUAUG	Cleavage
tae-miR1133	Traes_1DL_D391AE3DC.2	PF16212		3.6.3.1		GO:GO:0016021,GO:GO:0015914,GO:GO:0005524,GO:GO:0004012,GO:GO:0000287	AT1G59820.1	aminophospholipid ATPase 3	LOC_Os10g27220.1	phospholipid-transporting ATPase 3, putative, expressed	2,00	-1.0	1	22	858	879	CAUAUACUCCCUCCGUCCGAAA	AAAUGAACGGAGGGAGUAUAUG	Cleavage
tae-miR1133	Traes_1DL_D391AE3DC.3	PF16212		3.6.3.1		GO:GO:0016021,GO:GO:0015914,GO:GO:0005524,GO:GO:0004012,GO:GO:0000287	AT1G59820.1	aminophospholipid ATPase 3	LOC_Os10g27220.1	phospholipid-transporting ATPase 3, putative, expressed	2,00	-1.0	1	22	822	843	CAUAUACUCCCUCCGUCCGAAA	AAAUGAACGGAGGGAGUAUAUG	Cleavage
tae-miR1128	Traes_1DL_E30609261.7						AT1G04300.3	TRAF-like superfamily protein	LOC_Os05g43280.1	MATH domain containing protein, expressed	3,00	-1.0	1	21	76	96	UACUACUCCCUCCGUCCGAAA	UUCCAGACGGAGGGAGUAGUU	Cleavage
tae-miR1122a	Traes_1DS_06B8348E0.1	PF03283				GO:GO:0016787	AT3G09410.1	Pectinacetylesterase family protein	LOC_Os05g02120.1	pectinacetylesterase domain containing protein, expressed	0,00	-1.0	1	20	794	813	UAGAUACAUCCGUAUCUAGA	UCUAGAUACGGAUGUAUCUA	Cleavage
tae-miR1122a	Traes_1DS_06B8348E0.2	PF03283				GO:GO:0016787	AT3G09410.1	Pectinacetylesterase family protein	LOC_Os05g02120.1	pectinacetylesterase domain containing protein, expressed	0,00	-1.0	1	20	620	639	UAGAUACAUCCGUAUCUAGA	UCUAGAUACGGAUGUAUCUA	Cleavage
tae-miR5048-5p	Traes_1DS_26F1546F6.1	PF00168,PF07714,PF00069	KOG1187	2.7.11.1		GO:GO:0005515,GO:GO:0006468,GO:GO:0004672,GO:GO:0005524	AT1G63430.1	Leucine-rich repeat protein kinase family protein	LOC_Os04g58700.1	expressed protein	2,05	-1.0	1	22	917	938	UUUGCAGGUUUUAGGUCUAAGU	ACUUGGACCUAAAACCUGCCAA	Cleavage
tae-miR5048-5p	Traes_1DS_26F1546F6.2	PF00168,PF07714,PF00069	KOG1187	2.7.11.1		GO:GO:0005515,GO:GO:0006468,GO:GO:0004672,GO:GO:0005524	AT1G63430.1	Leucine-rich repeat protein kinase family protein	LOC_Os04g58700.1	expressed protein	2,05	-1.0	1	22	917	938	UUUGCAGGUUUUAGGUCUAAGU	ACUUGGACCUAAAACCUGCCAA	Cleavage
tae-miR7757-5p	Traes_1DS_35EF41CCB.2	PF00931				GO:GO:0043531	AT3G50950.1	HOPZ-ACTIVATED RESISTANCE 1	LOC_Os05g40150.1	RGH2B, putative, expressed	1,05	-1.0	1	22	587	608	AUAAAACCUUCAGCUAUCCAUC	AGUGGAUAGCUGAAGGUUUCAU	Cleavage
tae-miR9674a-5p	Traes_1DS_F3DE3946F.1	PF13041					AT1G62930.1	Tetratricopeptide repeat (TPR)-like superfamily protein	LOC_Os04g28300.1	Rf1, mitochondrial precursor, putative, expressed	2,05	-1.0	1	21	522	542	GCAUCAUCCAUCCUACCAUUC	AAAUGGAAGGAUCGAUGAUGC	Cleavage
tae-miR5049-3p	Traes_1DS_FA7FC5C3C.1	PF04997		2.7.7.6		GO:GO:0006351,GO:GO:0003899,GO:GO:0003677	AT1G63020.1	nuclear RNA polymerase D1A	LOC_Os02g05880.1	RNA polymerase Rpb1, domain 2 family protein, expressed	0,00	-1.0	1	21	623	643	AAUAUGGAUCGGAGGGAGUAC	GUACUCCCUCCGAUCCAUAUU	Cleavage
tae-miR1128	Traes_2AL_24A1BF00C.1						AT5G06210.1	RNA binding (RRM/RBD/RNP motifs) family protein	LOC_Os04g33810.1	RNA recognition motif containing protein, putative, expressed	0,00	-1.0	1	21	37	57	UACUACUCCCUCCGUCCGAAA	UUUCGGACGGAGGGAGUAGUA	Cleavage
tae-miR5049-3p	Traes_2AL_28B13CC56.1					GO:GO:0005681,GO:GO:0000398	AT1G80070.1	Pre-mRNA-processing-splicing factor	LOC_Os06g07080.1	pre-mRNA-processing-splicing factor 8, putative, expressed	1,05	-1.0	1	21	346	366	AAUAUGGAUCGGAGGGAGUAC	CUACUCCCUCCGUUCCAUAUU	Cleavage
tae-miR1127a	Traes_2AL_4197D219B.1						AT3G16200.1		LOC_Os01g31220.1	expressed protein	2,05	-1.0	1	19	991	1009	UCCUUCCGUUCGGAAUUAC	GUAAUUCCGAACGGAGGGU	Cleavage
tae-miR164	Traes_2AL_57BF37A86.1	PF02365				GO:GO:0006355,GO:GO:0003677	AT5G61430.1	NAC domain containing protein 100	LOC_Os04g38720.1	no apical meristem protein, putative, expressed	1,05	-1.0	1	21	680	700	UGGAGAAGCAGGGCACGUGCA	CGCACGUGACCUGCUUCUCCA	Cleavage
tae-miR164	Traes_2AL_57BF37A86.2	PF02365				GO:GO:0006355,GO:GO:0003677	AT5G61430.1	NAC domain containing protein 100	LOC_Os04g38720.1	no apical meristem protein, putative, expressed	1,05	-1.0	1	21	606	626	UGGAGAAGCAGGGCACGUGCA	CGCACGUGACCUGCUUCUCCA	Cleavage
tae-miR1122a	Traes_2AL_64B73E3ED.1			2.8.1.8		GO:GO:0051539,GO:GO:0016992,GO:GO:0009107	AT2G20860.1	lipoic acid synthase 1	LOC_Os04g38330.1	radical SAM enzyme, putative, expressed	1,00	-1.0	1	20	121	140	UAGAUACAUCCGUAUCUAGA	UCUAAAUACGGAUGUAUCUA	Cleavage
tae-miR1120b-3p	Traes_2AL_673CA8FB2.1	PF04603	KOG3329				AT1G69680.1	Mog1/PsbP/DUF1795-like photosystem II reaction center PsbP family protein	LOC_Os04g38610.2	RAN guanine nucleotide release factor, putative, expressed	2,05	-1.0	1	21	493	513	UUCUUAUAUUGUGGGACAGAG	CUCUGUCCCACAAUGUAAGAC	Cleavage
tae-miR1133	Traes_2AL_673CA8FB2.1	PF04603	KOG3329				AT1G69680.1	Mog1/PsbP/DUF1795-like photosystem II reaction center PsbP family protein	LOC_Os04g38610.2	RAN guanine nucleotide release factor, putative, expressed	2,05	-1.0	1	22	553	575	CAUAUACUCCCUCCGUCCGA-AA	UUGUGGGACGGAGGGAGUAUUUG	Cleavage
tae-miR1120a	Traes_2AL_979E96822.1	PF00443		3.4.19.12		GO:GO:0036459,GO:GO:0016579	AT4G39370.3	ubiquitin-specific protease 27	LOC_Os04g34984.1	ubiquitin carboxyl-terminal hydrolase domain containing protein, expressed	2,05	-1.0	1	24	753	776	ACAUUCUUAUAUUAUGAGACGGAG	CUCCAUCCCAUAAUAUAAGAGUGU	Cleavage
tae-miR1130b-3p	Traes_2AL_979E96822.1	PF00443		3.4.19.12		GO:GO:0036459,GO:GO:0016579	AT4G39370.3	ubiquitin-specific protease 27	LOC_Os04g34984.1	ubiquitin carboxyl-terminal hydrolase domain containing protein, expressed	1,00	-1.0	1	21	752	772	UCUUAUAUUAUGGGACGGAGG	CCUCCAUCCCAUAAUAUAAGA	Cleavage
tae-miR1122c-3p	Traes_2AL_979E96822.1	PF00443		3.4.19.12		GO:GO:0036459,GO:GO:0016579	AT4G39370.3	ubiquitin-specific protease 27	LOC_Os04g34984.1	ubiquitin carboxyl-terminal hydrolase domain containing protein, expressed	2,05	-1.0	1	21	752	772	UCUAAUAUUAUGGGACGGAGG	CCUCCAUCCCAUAAUAUAAGA	Cleavage
tae-miR1120c-5p	Traes_2AL_979E96822.1	PF00443		3.4.19.12		GO:GO:0036459,GO:GO:0016579	AT4G39370.3	ubiquitin-specific protease 27	LOC_Os04g34984.1	ubiquitin carboxyl-terminal hydrolase domain containing protein, expressed	2,05	-1.0	1	21	795	815	UAAUAUAAGAACGUUUUUGAC	GUAAAAAACGCUCUUAUAUUA	Translation
tae-miR160	Traes_2AL_C70217249.1	PF06507,PF02362				GO:GO:0009725,GO:GO:0006355,GO:GO:0005634,GO:GO:0003677	AT1G77850.1	auxin response factor 17	LOC_Os04g59430.1	auxin response factor, putative, expressed	1,00	-1.0	1	21	1164	1184	UGCCUGGCUCCCUGUAUGCCA	UGGCAUGCAGGGAGCCAGGCA	Cleavage
tae-miR1134	Traes_2AL_D2CC35E59.1										3,00	-1.0	1	24	512	535	CAACAACAACAAGAAGAAGAAGAU	AUCUUCAUGGUCUUGUUGUUGUUG	Cleavage
tae-miR1134	Traes_2AL_D2CC35E59.2										3,00	-1.0	1	24	620	643	CAACAACAACAAGAAGAAGAAGAU	AUCUUCAUGGUCUUGUUGUUGUUG	Cleavage
tae-miR1134	Traes_2AL_D2CC35E59.3										3,00	-1.0	1	24	509	532	CAACAACAACAAGAAGAAGAAGAU	AUCUUCAUGGUCUUGUUGUUGUUG	Cleavage
tae-miR1134	Traes_2AL_D2CC35E59.4										3,00	-1.0	1	24	611	634	CAACAACAACAAGAAGAAGAAGAU	AUCUUCAUGGUCUUGUUGUUGUUG	Cleavage
tae-miR1134	Traes_2AL_D2CC35E59.5										3,00	-1.0	1	24	509	532	CAACAACAACAAGAAGAAGAAGAU	AUCUUCAUGGUCUUGUUGUUGUUG	Cleavage
tae-miR1134	Traes_2AL_D2CC35E59.6										3,00	-1.0	1	24	509	532	CAACAACAACAAGAAGAAGAAGAU	AUCUUCAUGGUCUUGUUGUUGUUG	Cleavage
tae-miR5384-3p	Traes_2AL_D79C9535C.1	PF00067	KOG0157	1.14.13.109		GO:GO:0055114,GO:GO:0020037,GO:GO:0016705,GO:GO:0005506	AT5G36110.1	cytochrome P450, family 716, subfamily A, polypeptide 1	LOC_Os09g21260.1	cytochrome P450 domain containing protein, expressed	2,05	-1.0	1	21	1250	1270	UGAGCGCGCCGCCGUCGAAUG	CGUUCGGCGGCGGCGCGCGCA	Cleavage
tae-miR9778	Traes_2AS_530CF4042.10	PF00931				GO:GO:0043531	AT3G50950.1	HOPZ-ACTIVATED RESISTANCE 1	LOC_Os04g12830.1	expressed protein	2,05	-1.0	1	21	126	146	UGCAUCAUCUCGAACUCGUCG	CGGCGAGUUCCAGAUGAUGCA	Translation
tae-miR9778	Traes_2AS_530CF4042.2	PF00931				GO:GO:0043531	AT3G50950.1	HOPZ-ACTIVATED RESISTANCE 1	LOC_Os04g12830.1	expressed protein	2,05	-1.0	1	21	126	146	UGCAUCAUCUCGAACUCGUCG	CGGCGAGUUCCAGAUGAUGCA	Translation
tae-miR9778	Traes_2AS_530CF4042.3	PF00931				GO:GO:0043531	AT3G50950.1	HOPZ-ACTIVATED RESISTANCE 1	LOC_Os04g12830.1	expressed protein	2,05	-1.0	1	21	126	146	UGCAUCAUCUCGAACUCGUCG	CGGCGAGUUCCAGAUGAUGCA	Translation
tae-miR1130b-3p	Traes_2AS_6DCA4AADD.1					GO:GO:0016020,GO:GO:0008236,GO:GO:0006508	AT2G31140.1	Peptidase S24/S26A/S26B/S26C family protein	LOC_Os04g01240.1	serine-type peptidase, putative, expressed	1,05	-1.0	1	21	288	308	UCUUAUAUUAUGGGACGGAGG	CCUCCGUCCCAUAAUAUAGGA	Cleavage
tae-miR9664-3p	Traes_2AS_86633353C.1	PF00931	KOG4658		K13457	GO:GO:0043531	AT3G07040.1	NB-ARC domain-containing disease resistance protein	LOC_Os11g46210.1	NB-ARC domain containing protein, expressed	2,05	-1.0	1	21	240	260	UUGCAGUCCUCGAUGUCGUAG	CUACGACAUCGAGGAUUGCAU	Cleavage
tae-miR1130b-3p	Traes_2BL_171777733.1	PF00443		3.4.19.12		GO:GO:0036459,GO:GO:0016579	AT4G39370.3	ubiquitin-specific protease 27	LOC_Os04g34984.1	ubiquitin carboxyl-terminal hydrolase domain containing protein, expressed	0,00	-1.0	1	21	752	772	UCUUAUAUUAUGGGACGGAGG	CCUCCGUCCCAUAAUAUAAGA	Cleavage
tae-miR1122c-3p	Traes_2BL_171777733.1	PF00443		3.4.19.12		GO:GO:0036459,GO:GO:0016579	AT4G39370.3	ubiquitin-specific protease 27	LOC_Os04g34984.1	ubiquitin carboxyl-terminal hydrolase domain containing protein, expressed	1,05	-1.0	1	21	752	772	UCUAAUAUUAUGGGACGGAGG	CCUCCGUCCCAUAAUAUAAGA	Cleavage
tae-miR1120c-5p	Traes_2BL_171777733.1	PF00443		3.4.19.12		GO:GO:0036459,GO:GO:0016579	AT4G39370.3	ubiquitin-specific protease 27	LOC_Os04g34984.1	ubiquitin carboxyl-terminal hydrolase domain containing protein, expressed	2,05	-1.0	1	21	795	815	UAAUAUAAGAACGUUUUUGAC	GUAAAAAACGCUCUUAUAUUA	Translation
tae-miR5384-3p	Traes_2BL_1BE5C9C71.1	PF00067		1.14.13.109		GO:GO:0055114,GO:GO:0020037,GO:GO:0016705,GO:GO:0005506	AT2G42850.1	cytochrome P450, family 718	LOC_Os09g21260.1	cytochrome P450 domain containing protein, expressed	2,05	-1.0	1	21	455	475	UGAGCGCGCCGCCGUCGAAUG	CGUUCGGCGGCGGCGCGCGCA	Cleavage
tae-miR5384-3p	Traes_2BL_1BE5C9C71.2	PF00067				GO:GO:0055114,GO:GO:0020037,GO:GO:0016705,GO:GO:0005506	AT5G36110.1	cytochrome P450, family 716, subfamily A, polypeptide 1	LOC_Os09g21260.1	cytochrome P450 domain containing protein, expressed	2,05	-1.0	1	21	455	475	UGAGCGCGCCGCCGUCGAAUG	CGUUCGGCGGCGGCGCGCGCA	Cleavage
tae-miR1122a	Traes_2BL_40442CE82.2	PF14392,PF00098				GO:GO:0008270,GO:GO:0003676	AT5G52380.1	VASCULAR-RELATED NAC-DOMAIN 6	LOC_Os04g46920.1	zinc knuckle domain containing protein, expressed	1,05	-1.0	1	20	703	722	UAGAUACAUCCGUAUCUAGA	UCUAGAUACAGAUGUAUCUA	Translation
tae-miR164	Traes_2BL_6AEE8AC28.1	PF02365				GO:GO:0006355,GO:GO:0003677	AT5G61430.1	NAC domain containing protein 100	LOC_Os04g38720.1	no apical meristem protein, putative, expressed	1,05	-1.0	1	21	680	700	UGGAGAAGCAGGGCACGUGCA	CGCACGUGACCUGCUUCUCCA	Cleavage
tae-miR164	Traes_2BL_6AEE8AC28.2	PF02365				GO:GO:0006355,GO:GO:0003677	AT5G61430.1	NAC domain containing protein 100	LOC_Os04g38720.1	no apical meristem protein, putative, expressed	1,05	-1.0	1	21	672	692	UGGAGAAGCAGGGCACGUGCA	CGCACGUGACCUGCUUCUCCA	Cleavage
tae-miR164	Traes_2BL_6AEE8AC28.3	PF02365				GO:GO:0006355,GO:GO:0003677	AT5G61430.1	NAC domain containing protein 100	LOC_Os04g38720.1	no apical meristem protein, putative, expressed	1,05	-1.0	1	21	633	653	UGGAGAAGCAGGGCACGUGCA	CGCACGUGACCUGCUUCUCCA	Cleavage
tae-miR1122b-3p	Traes_2BL_7141904F2.2	PF03097					AT1G73390.1	Endosomal targeting BRO1-like domain-containing protein	LOC_Os04g44530.3	expressed protein	2,05	-1.0	1	21	1058	1078	AGACUUAUAUGUAGGAACGGA	UCCGUUCUUAAAUAUAAGUCU	Translation
tae-miR1122a	Traes_2BL_7141904F2.2	PF03097					AT1G73390.1	Endosomal targeting BRO1-like domain-containing protein	LOC_Os04g44530.3	expressed protein	2,05	-1.0	1	20	1098	1117	UAGAUACAUCCGUAUCUAGA	ACUACAUACGGAUGUAUAUA	Cleavage
tae-miR1134	Traes_2BL_8339D35AC.1										3,00	-1.0	1	24	509	532	CAACAACAACAAGAAGAAGAAGAU	AUCUUCAUGGUCUUGUUGUUGUUG	Cleavage
tae-miR1134	Traes_2BL_8339D35AC.2										3,00	-1.0	1	24	512	535	CAACAACAACAAGAAGAAGAAGAU	AUCUUCAUGGUCUUGUUGUUGUUG	Cleavage
tae-miR1134	Traes_2BL_8339D35AC.3										3,00	-1.0	1	24	509	532	CAACAACAACAAGAAGAAGAAGAU	AUCUUCAUGGUCUUGUUGUUGUUG	Cleavage
tae-miR1134	Traes_2BL_8339D35AC.4										3,00	-1.0	1	24	302	325	CAACAACAACAAGAAGAAGAAGAU	AUCUUCAUGGUCUUGUUGUUGUUG	Cleavage
tae-miR1134	Traes_2BL_8339D35AC.5										3,00	-1.0	1	24	509	532	CAACAACAACAAGAAGAAGAAGAU	AUCUUCAUGGUCUUGUUGUUGUUG	Cleavage
tae-miR1135	Traes_2BL_A4AE931B0.1						AT2G02090.1	SNF2 domain-containing protein / helicase domain-containing protein	LOC_Os04g47830.1	SNF2 family N-terminal domain containing protein, expressed	2,00	-1.0	1	24	1154	1177	CUGCGACAAGUAAUUCCGAACGGA	UCUGUCCGGAAUUACUUGUCGCAA	Cleavage
tae-miR1122b-3p	Traes_2BL_FF8002798.2	PF13414					AT1G56090.1	Tetratricopeptide repeat (TPR)-like superfamily protein	LOC_Os04g52580.1	tetratricopeptide repeat domain containing protein, expressed	2,05	-1.0	1	21	889	909	AGACUUAUAUGUAGGAACGGA	UCGGUUCCUAAAUAUAAGUCU	Translation
tae-miR9778	Traes_2BS_0EFFAAE28.1	PF00931				GO:GO:0043531	AT3G07040.1	NB-ARC domain-containing disease resistance protein	LOC_Os08g14850.1	resistance protein, putative, expressed	2,05	-1.0	1	21	126	146	UGCAUCAUCUCGAACUCGUCG	CGGCGAGUUCCAGAUGAUGCA	Translation
tae-miR9778	Traes_2BS_0EFFAAE28.2	PF00931				GO:GO:0043531	AT3G07040.1	NB-ARC domain-containing disease resistance protein	LOC_Os08g14850.1	resistance protein, putative, expressed	2,05	-1.0	1	21	126	146	UGCAUCAUCUCGAACUCGUCG	CGGCGAGUUCCAGAUGAUGCA	Translation
tae-miR9662a-3p	Traes_2BS_257C5B099.1	PF02536				GO:GO:0006355,GO:GO:0005739,GO:GO:0003690	AT5G07900.1	Mitochondrial transcription termination factor family protein	LOC_Os06g12100.1	mTERF family protein, expressed	2,00	-1.0	1	21	66	86	UUGAACAUCCCAGAGCCACCG	CCGUGGUUCUGGGAUGUUCAG	Cleavage
tae-miR9662b-3p	Traes_2BS_257C5B099.1	PF02536				GO:GO:0006355,GO:GO:0005739,GO:GO:0003690	AT5G07900.1	Mitochondrial transcription termination factor family protein	LOC_Os06g12100.1	mTERF family protein, expressed	2,00	-1.0	1	21	65	85	UGAACAUCCCAGAGCCACCGG	CCCGUGGUUCUGGGAUGUUCA	Cleavage
tae-miR9662a-3p	Traes_2BS_257C5B0991.1	PF02536				GO:GO:0006355,GO:GO:0005739,GO:GO:0003690	AT5G07900.1	Mitochondrial transcription termination factor family protein	LOC_Os06g12100.1	mTERF family protein, expressed	2,00	-1.0	1	21	66	86	UUGAACAUCCCAGAGCCACCG	CCGUGGUUCUGGGAUGUUCAG	Cleavage
tae-miR9662b-3p	Traes_2BS_257C5B0991.1	PF02536				GO:GO:0006355,GO:GO:0005739,GO:GO:0003690	AT5G07900.1	Mitochondrial transcription termination factor family protein	LOC_Os06g12100.1	mTERF family protein, expressed	2,00	-1.0	1	21	65	85	UGAACAUCCCAGAGCCACCGG	CCCGUGGUUCUGGGAUGUUCA	Cleavage
tae-miR5048-5p	Traes_2BS_47E2D6444.1	PF07714		2.7.11.1		GO:GO:0006468,GO:GO:0004672	AT4G04570.2	cysteine-rich RLK (RECEPTOR-like protein kinase) 40	LOC_Os04g30030.1	cysteine-rich receptor-like protein kinase 12 precursor, putative, expressed	2,05	-1.0	1	22	221	242	UUUGCAGGUUUUAGGUCUAAGU	ACCUGGACCUAAAACCUGCGAA	Cleavage
tae-miR5048-5p	Traes_2BS_47E2D6444.2	PF00069	KOG1187	2.7.11.1		GO:GO:0006468,GO:GO:0005524,GO:GO:0004672	AT4G38830.1	cysteine-rich RLK (RECEPTOR-like protein kinase) 26	LOC_Os04g30030.1	cysteine-rich receptor-like protein kinase 12 precursor, putative, expressed	2,05	-1.0	1	22	959	980	UUUGCAGGUUUUAGGUCUAAGU	ACCUGGACCUAAAACCUGCGAA	Cleavage
tae-miR5048-5p	Traes_2BS_47E2D6444.3	PF00069	KOG1187	2.7.11.1		GO:GO:0006468,GO:GO:0005524,GO:GO:0004672	AT4G38830.1	cysteine-rich RLK (RECEPTOR-like protein kinase) 26	LOC_Os04g30030.1	cysteine-rich receptor-like protein kinase 12 precursor, putative, expressed	2,05	-1.0	1	22	947	968	UUUGCAGGUUUUAGGUCUAAGU	ACCUGGACCUAAAACCUGCGAA	Cleavage
tae-miR5048-5p	Traes_2BS_47E2D6444.4	PF07714		2.7.11.1		GO:GO:0006468,GO:GO:0004672	AT4G23160.1	cysteine-rich RLK (RECEPTOR-like protein kinase) 8	LOC_Os04g30030.1	cysteine-rich receptor-like protein kinase 12 precursor, putative, expressed	2,05	-1.0	1	22	221	242	UUUGCAGGUUUUAGGUCUAAGU	ACCUGGACCUAAAACCUGCGAA	Cleavage
tae-miR9664-3p	Traes_2BS_5045C640C.1	PF00931	KOG4658		K13457	GO:GO:0043531	AT3G07040.1	NB-ARC domain-containing disease resistance protein	LOC_Os11g46210.1	NB-ARC domain containing protein, expressed	2,05	-1.0	1	21	240	260	UUGCAGUCCUCGAUGUCGUAG	CUACGACAUCGAGGAUUGCAU	Cleavage
tae-miR9664-3p	Traes_2BS_5045C640C.2	PF00931	KOG4658		K13457	GO:GO:0043531	AT3G07040.1	NB-ARC domain-containing disease resistance protein	LOC_Os11g46210.1	NB-ARC domain containing protein, expressed	2,05	-1.0	1	21	240	260	UUGCAGUCCUCGAUGUCGUAG	CUACGACAUCGAGGAUUGCAU	Cleavage
tae-miR9664-3p	Traes_2BS_5045C640C.5	PF00931	KOG4658		K13457	GO:GO:0043531	AT3G07040.1	NB-ARC domain-containing disease resistance protein	LOC_Os11g46210.1	NB-ARC domain containing protein, expressed	2,05	-1.0	1	21	240	260	UUGCAGUCCUCGAUGUCGUAG	CUACGACAUCGAGGAUUGCAU	Cleavage
tae-miR1120c-5p	Traes_2BS_5C64FC44A.1	PF07714	KOG1187	2.7.11.1		GO:GO:0006468,GO:GO:0004672	AT4G23140.1	cysteine-rich RLK (RECEPTOR-like protein kinase) 6	LOC_Os07g35580.1	TKL_IRAK_DUF26-lc.20 - DUF26 kinases have homology to DUF26 containing loci, expressed	0,00	-1.0	1	21	21	41	UAAUAUAAGAACGUUUUUGAC	GUCAAAAACGUUCUUAUAUUA	Cleavage
tae-miR1120c-5p	Traes_2BS_5C64FC44A.2	PF07714		2.7.11.1		GO:GO:0006468,GO:GO:0004672	AT4G23180.1	cysteine-rich RLK (RECEPTOR-like protein kinase) 10	LOC_Os07g35580.1	TKL_IRAK_DUF26-lc.20 - DUF26 kinases have homology to DUF26 containing loci, expressed	0,00	-1.0	1	21	2	22	UAAUAUAAGAACGUUUUUGAC	GUCAAAAACGUUCUUAUAUUA	Cleavage
tae-miR1130b-3p	Traes_2BS_5CDCD5152.1	PF04178	KOG1743			GO:GO:0016192	AT5G01430.1	Got1/Sft2-like vescicle transport protein family	LOC_Os03g11100.1	vesicle transport protein GOT1B, putative, expressed	2,00	-1.0	1	20	1	21	UCUUAUAUUAUGGGA-CGGAG	GUCCGAUCCCAUAAUAUAAGA	Cleavage
tae-miR1121	Traes_2BS_6249E432F.1	PF01426				GO:GO:0003682	AT4G22140.1	PHD finger family protein / bromo-adjacent homology (BAH) domain-containing protein	LOC_Os07g08880.1	ES43 protein, putative, expressed	0,00	-1.0	1	22	538	559	AGUAGUGAUCUAAACGCUCUUA	UAAGAGCGUUUAGAUCACUACU	Cleavage
tae-miR1121	Traes_2BS_6249E432F.2	PF01426				GO:GO:0003682	AT4G22140.1	PHD finger family protein / bromo-adjacent homology (BAH) domain-containing protein	LOC_Os07g08880.1	ES43 protein, putative, expressed	0,00	-1.0	1	22	538	559	AGUAGUGAUCUAAACGCUCUUA	UAAGAGCGUUUAGAUCACUACU	Cleavage
tae-miR1121	Traes_2BS_6249E432F.3	PF01426				GO:GO:0003682	AT4G39100.1	PHD finger family protein / bromo-adjacent homology (BAH) domain-containing protein	LOC_Os07g08880.1	ES43 protein, putative, expressed	0,00	-1.0	1	22	448	469	AGUAGUGAUCUAAACGCUCUUA	UAAGAGCGUUUAGAUCACUACU	Cleavage
tae-miR1133	Traes_2BS_67D862F4A.1	PF01428	KOG3183			GO:GO:0008270	AT2G41835.1	zinc finger (C2H2 type, AN1-like) family protein	LOC_Os07g38240.1	ZOS7-05 - C2H2 zinc finger protein, expressed	2,05	-1.0	1	22	30	51	CAUAUACUCCCUCCGUCCGAAA	UUUGGGACGGAGGGAGUAUAAG	Cleavage
tae-miR9662a-3p	Traes_2BS_74269A4C5.1	PF02536				GO:GO:0006355,GO:GO:0005739,GO:GO:0003690	AT5G07900.1	Mitochondrial transcription termination factor family protein	LOC_Os06g12100.1	mTERF family protein, expressed	2,00	-1.0	1	21	189	209	UUGAACAUCCCAGAGCCACCG	CCGUGGUUCUGGGAUGUUCAG	Cleavage
tae-miR9662b-3p	Traes_2BS_74269A4C5.1	PF02536				GO:GO:0006355,GO:GO:0005739,GO:GO:0003690	AT5G07900.1	Mitochondrial transcription termination factor family protein	LOC_Os06g12100.1	mTERF family protein, expressed	2,00	-1.0	1	21	188	208	UGAACAUCCCAGAGCCACCGG	CCCGUGGUUCUGGGAUGUUCA	Cleavage
tae-miR1128	Traes_2BS_7AEB138EB.1	PF01397,PF03936		4.2.3.104		GO:GO:0016829,GO:GO:0010333,GO:GO:0008152,GO:GO:0000287	AT5G23960.2	terpene synthase 21	LOC_Os07g11790.1	terpene synthase, putative, expressed	1,05	-1.0	1	21	84	104	UACUACUCCCUCCGUCCGAAA	UUUCGGACGGAGGGAGUACUA	Cleavage
tae-miR1122b-3p	Traes_2BS_A20CCBEC3.1	PF05368		1.23.1.1,1.23.1.3			AT1G32100.1	pinoresinol reductase 1	LOC_Os12g16220.2	nmrA-like family domain containing protein, expressed	2,05	-1.0	1	21	389	409	AGACUUAUAUGUAGGAACGGA	UCUGUUCCUAAAUAUAAGUCU	Translation
tae-miR1128	Traes_2BS_B3A88F254.2	PF00479,PF02781		1.1.1.49	K00036	GO:GO:0055114,GO:GO:0050661,GO:GO:0006006,GO:GO:0004345	AT5G13110.1	glucose-6-phosphate dehydrogenase 2	LOC_Os07g22350.1	glucose-6-phosphate 1-dehydrogenase, chloroplast precursor, putative, expressed	2,00	-1.0	1	21	88	109	UACUACUCCCUCCGUCC-GAAA	UUUCCGGACGGAGGGAGUAGUA	Cleavage
tae-miR1128	Traes_2BS_B3A88F254.3	PF00479,PF02781		1.1.1.49	K00036	GO:GO:0055114,GO:GO:0050661,GO:GO:0006006,GO:GO:0004345	AT5G13110.1	glucose-6-phosphate dehydrogenase 2	LOC_Os07g22350.1	glucose-6-phosphate 1-dehydrogenase, chloroplast precursor, putative, expressed	2,00	-1.0	1	21	88	109	UACUACUCCCUCCGUCC-GAAA	UUUCCGGACGGAGGGAGUAGUA	Cleavage
tae-miR1127a	Traes_2BS_C00FC92D4.2						AT2G18910.1	hydroxyproline-rich glycoprotein family protein	LOC_Os02g26370.1	wiskott-Aldrich syndrome protein family member 2, putative, expressed	1,05	-1.0	1	19	497	515	UCCUUCCGUUCGGAAUUAC	GUAAUUCCGAACGGAGGGA	Cleavage

tae-miR1117	Traes_2BS_C5AF5419E.1			3.4.23.47,3.1.26.13,3.1.13.2,2.7.7.49,2.7.7.7					LOC_Os02g49670.1	zinc knuckle family protein, expressed	0,00	-1.0	1	24	926	949	UAGUACCGGUUCGUGGCACGAACC	GGUUCGUGCCACGAACCGGUACUA	Cleavage
tae-miR9662a-3p	Traes_2BS_DDF26C784.1	PF02536				GO:GO:0006355,GO:GO:0005739,GO:GO:0003690	AT5G07900.1	Mitochondrial transcription termination factor family protein	LOC_Os06g12100.1	mTERF family protein, expressed	2,00	-1.0	1	21	459	479	UUGAACAUCCCAGAGCCACCG	CCGUGGUUCUGGGAUGUUCAG	Cleavage
tae-miR9662b-3p	Traes_2BS_DDF26C784.1	PF02536				GO:GO:0006355,GO:GO:0005739,GO:GO:0003690	AT5G07900.1	Mitochondrial transcription termination factor family protein	LOC_Os06g12100.1	mTERF family protein, expressed	2,00	-1.0	1	21	458	478	UGAACAUCCCAGAGCCACCGG	CCCGUGGUUCUGGGAUGUUCA	Cleavage
tae-miR5384-3p	Traes_2BS_EBCD10293.1	PF02225,PF00082		3.4.21.25		GO:GO:0006508,GO:GO:0004252	AT1G04110.1	Subtilase family protein	LOC_Os04g10360.1	OsSub40 - Putative Subtilisin homologue, expressed	2,05	-1.0	1	21	116	136	UGAGCGCGCCGCCGUCGAAUG	UGUUCGCCGACGGCGCGCUCA	Cleavage
tae-miR5384-3p	Traes_2BS_EBCD10293.2	PF02225,PF00082		3.4.21.25		GO:GO:0006508,GO:GO:0004252	AT1G04110.1	Subtilase family protein	LOC_Os04g10360.1	OsSub40 - Putative Subtilisin homologue, expressed	2,05	-1.0	1	21	116	136	UGAGCGCGCCGCCGUCGAAUG	UGUUCGCCGACGGCGCGCUCA	Cleavage
tae-miR1130b-3p	Traes_2DL_10B2965EF.8	PF00443		3.4.19.12		GO:GO:0036459,GO:GO:0016579	AT4G39370.2	ubiquitin-specific protease 27	LOC_Os04g34984.3	ubiquitin carboxyl-terminal hydrolase domain containing protein, expressed	0,00	-1.0	1	21	752	772	UCUUAUAUUAUGGGACGGAGG	CCUCCGUCCCAUAAUAUAAGA	Cleavage
tae-miR1122c-3p	Traes_2DL_10B2965EF.8	PF00443		3.4.19.12		GO:GO:0036459,GO:GO:0016579	AT4G39370.2	ubiquitin-specific protease 27	LOC_Os04g34984.3	ubiquitin carboxyl-terminal hydrolase domain containing protein, expressed	1,05	-1.0	1	21	752	772	UCUAAUAUUAUGGGACGGAGG	CCUCCGUCCCAUAAUAUAAGA	Cleavage
tae-miR1120c-5p	Traes_2DL_10B2965EF.8	PF00443		3.4.19.12		GO:GO:0036459,GO:GO:0016579	AT4G39370.2	ubiquitin-specific protease 27	LOC_Os04g34984.3	ubiquitin carboxyl-terminal hydrolase domain containing protein, expressed	1,05	-1.0	1	21	795	815	UAAUAUAAGAACGUUUUUGAC	GUCAAAAACGCUCUUAUAUUA	Translation
tae-miR1130b-3p	Traes_2DL_3C9129432.1	PF00656				GO:GO:0006508,GO:GO:0004197	AT1G79340.1	metacaspase 4	LOC_Os01g58580.1	ICE-like protease p20 domain containing protein, putative, expressed	1,05	-1.0	1	21	164	184	UCUUAUAUUAUGGGACGGAGG	CUUCCGUCCCAUAAUGUAAGA	Cleavage
tae-miR1133	Traes_2DL_4481C0380.4										2,05	-1.0	1	22	1	22	CAUAUACUCCCUCCGUCCGAAA	UUUGGGACGGAGGGAGUACAUG	Cleavage
tae-miR1137a	Traes_2DL_4F8C87C42.5	PF04499	KOG2073				AT1G30470.1	SIT4 phosphatase-associated family protein	LOC_Os04g52940.1	SIT4 phosphatase-associated protein domain containing protein, expressed	1,00	-1.0	1	20	1791	1810	UAGUACAAAGUUGAGUCAUC	GAUGACCCAACUUUGUACUA	Cleavage
tae-miR1127b-3p	Traes_2DL_4F8C87C42.5	PF04499	KOG2073				AT1G30470.1	SIT4 phosphatase-associated family protein	LOC_Os04g52940.1	SIT4 phosphatase-associated protein domain containing protein, expressed	1,05	-1.0	1	21	1822	1842	ACAAGUAUUUCUGGACGGAGG	CCUCCGUCCGGAAAUACUUGU	Cleavage
tae-miR1137b-5p	Traes_2DL_4F8C87C42.5	PF04499	KOG2073				AT1G30470.1	SIT4 phosphatase-associated family protein	LOC_Os04g52940.1	SIT4 phosphatase-associated protein domain containing protein, expressed	1,05	-1.0	1	21	1944	1964	UCCGUUCCAGAAUAGAUGACC	GGUCAUCUAUUUUGGAACGGA	Translation
tae-miR1122a	Traes_2DL_4F8C87C42.6	PF04499					AT1G30470.1	SIT4 phosphatase-associated family protein	LOC_Os04g52940.1	SIT4 phosphatase-associated protein domain containing protein, expressed	2,05	-1.0	1	20	1750	1769	UAGAUACAUCCGUAUCUAGA	ACUACAUACGGAUGUAUAUA	Cleavage
tae-miR1121	Traes_2DL_74E241841.2	PF01426				GO:GO:0003682	AT4G39100.1	PHD finger family protein / bromo-adjacent homology (BAH) domain-containing protein	LOC_Os07g08880.1	ES43 protein, putative, expressed	2,05	-1.0	1	22	448	469	AGUAGUGAUCUAAACGCUCUUA	UAAGAGUGUUUGGAUCACUACU	Translation
tae-miR1130b-3p	Traes_2DL_8F04980F0.5	PF03405		1.14.19.2		GO:GO:0055114,GO:GO:0045300,GO:GO:0006631	AT3G02630.1	Plant stearoyl-acyl-carrier-protein desaturase family protein	LOC_Os04g31070.1	acyl-desaturase, chloroplast precursor, putative, expressed	2,05	-1.0	1	21	468	488	UCUUAUAUUAUGGGACGGAGG	CCUCCGUCCCCUAAUAUAAGG	Translation
tae-miR1128	Traes_2DL_9AD532712.8	PF15613,PF15614					AT5G08630.1	DDT domain-containing protein	LOC_Os04g35864.1	DDT domain-containing protein, putative, expressed	2,00	-1.0	1	21	305	325	UACUACUCCCUCCGUCCGAAA	GUCCGGACGGAGGGAGUAGUU	Cleavage
tae-miR1122a	Traes_2DL_B3CD23EBC.1	PF05496,PF00004				GO:GO:0009378,GO:GO:0006310,GO:GO:0006281,GO:GO:0005524	AT1G03000.1	peroxin 6	LOC_Os04g52690.1	AAA family ATPase, putative, expressed	2,05	-1.0	1	20	9	28	UAGAUACAUCCGUAUCUAGA	ACUACAUACGGAUGUAUAUA	Cleavage
tae-miR9662a-3p	Traes_2DL_BE754F0C1.1	PF02536	KOG1267		K15032	GO:GO:0006355,GO:GO:0005739,GO:GO:0003690	AT5G07900.1	Mitochondrial transcription termination factor family protein	LOC_Os06g12100.1	mTERF family protein, expressed	1,00	-1.0	1	21	687	707	UUGAACAUCCCAGAGCCACCG	CCGUGGCUCUGGGAUGUUCAG	Cleavage
tae-miR9662b-3p	Traes_2DL_BE754F0C1.1	PF02536	KOG1267		K15032	GO:GO:0006355,GO:GO:0005739,GO:GO:0003690	AT5G07900.1	Mitochondrial transcription termination factor family protein	LOC_Os06g12100.1	mTERF family protein, expressed	1,00	-1.0	1	21	686	706	UGAACAUCCCAGAGCCACCGG	CCCGUGGCUCUGGGAUGUUCA	Cleavage
tae-miR1134	Traes_2DL_E8A7ABB5D.1										3,00	-1.0	1	24	509	532	CAACAACAACAAGAAGAAGAAGAU	AUCUUCAUGGUCUUGUUGUUGUUG	Cleavage
tae-miR1134	Traes_2DL_E8A7ABB5D.2										3,00	-1.0	1	24	629	652	CAACAACAACAAGAAGAAGAAGAU	AUCUUCAUGGUCUUGUUGUUGUUG	Cleavage
tae-miR1134	Traes_2DL_E8A7ABB5D.3										3,00	-1.0	1	24	614	637	CAACAACAACAAGAAGAAGAAGAU	AUCUUCAUGGUCUUGUUGUUGUUG	Cleavage
tae-miR1134	Traes_2DL_E8A7ABB5D.4										3,00	-1.0	1	24	512	535	CAACAACAACAAGAAGAAGAAGAU	AUCUUCAUGGUCUUGUUGUUGUUG	Cleavage
tae-miR1134	Traes_2DL_E8A7ABB5D.5										3,00	-1.0	1	24	509	532	CAACAACAACAAGAAGAAGAAGAU	AUCUUCAUGGUCUUGUUGUUGUUG	Cleavage
tae-miR1134	Traes_2DL_E8A7ABB5D.6										3,00	-1.0	1	24	509	532	CAACAACAACAAGAAGAAGAAGAU	AUCUUCAUGGUCUUGUUGUUGUUG	Cleavage
tae-miR1134	Traes_2DL_E8A7ABB5D.7										3,00	-1.0	1	24	91	114	CAACAACAACAAGAAGAAGAAGAU	AUCUUCAUGGUCUUGUUGUUGUUG	Cleavage
tae-miR1134	Traes_2DL_E8A7ABB5D.8										3,00	-1.0	1	24	77	100	CAACAACAACAAGAAGAAGAAGAU	AUCUUCAUGGUCUUGUUGUUGUUG	Cleavage
tae-miR1134	Traes_2DL_E8A7ABB5D.9										3,00	-1.0	1	24	28	51	CAACAACAACAAGAAGAAGAAGAU	AUCUUCAUGGUCUUGUUGUUGUUG	Cleavage
tae-miR160	Traes_2DL_EF12C0B89.2						AT1G77850.1	auxin response factor 17	LOC_Os04g59430.1	auxin response factor, putative, expressed	1,00	-1.0	1	21	225	245	UGCCUGGCUCCCUGUAUGCCA	UGGCAUGCAGGGAGCCAGGCA	Cleavage
tae-miR9664-3p	Traes_2DS_480CFDFE4.1	PF01637	KOG4658		K13457	GO:GO:0005524	AT3G07040.1	NB-ARC domain-containing disease resistance protein	LOC_Os11g46210.1	NB-ARC domain containing protein, expressed	2,05	-1.0	1	21	243	263	UUGCAGUCCUCGAUGUCGUAG	CUACGACAUCGAGGAUUGCAU	Cleavage
tae-miR9664-3p	Traes_2DS_480CFDFE4.2	PF01637				GO:GO:0005524	AT3G07040.1	NB-ARC domain-containing disease resistance protein	LOC_Os11g46210.1	NB-ARC domain containing protein, expressed	2,05	-1.0	1	21	259	279	UUGCAGUCCUCGAUGUCGUAG	CUACGACAUCGAGGAUUGCAU	Cleavage
tae-miR5049-3p	Traes_2DS_851973269.7										1,05	-1.0	1	21	178	198	AAUAUGGAUCGGAGGGAGUAC	GUACUCCCUCCGAUUCAUAUU	Cleavage
tae-miR1122b-3p	Traes_2DS_9B301D4F8.2	PF00069	KOG0580,KOG2345	2.7.11.1		GO:GO:0006468,GO:GO:0005524,GO:GO:0004672	AT5G18500.2	Protein kinase superfamily protein	LOC_Os11g39450.1	cysteine-rich receptor-like protein kinase 7 precursor, putative, expressed	1,05	-1.0	1	21	843	863	AGACUUAUAUGUAGGAACGGA	UCCGUUCCUAAAUAUAAGUCU	Translation
tae-miR1123	Traes_2DS_C866A54F1.2						AT4G36730.1	G-box binding factor 1	LOC_Os07g10890.1	transcription factor, putative, expressed	1,00	-1.0	1	23	792	814	UCCGUGAGACCUGGUCUCAUAGA	UCUAUGAGACCAGGUCUCACGGG	Cleavage
tae-miR1123	Traes_2DS_C866A54F1.4						AT4G36730.1	G-box binding factor 1	LOC_Os07g10890.1	transcription factor, putative, expressed	1,00	-1.0	1	23	786	808	UCCGUGAGACCUGGUCUCAUAGA	UCUAUGAGACCAGGUCUCACGGG	Cleavage
tae-miR9778	Traes_2DS_C9635AFF7.1	PF00931				GO:GO:0043531	AT3G07040.1	NB-ARC domain-containing disease resistance protein	LOC_Os08g14850.1	resistance protein, putative, expressed	2,05	-1.0	1	21	126	146	UGCAUCAUCUCGAACUCGUCG	CGGCGAGUUCCAGAUGAUGCA	Translation
tae-miR9778	Traes_2DS_C9635AFF7.2	PF00931				GO:GO:0043531	AT3G07040.1	NB-ARC domain-containing disease resistance protein	LOC_Os08g14850.1	resistance protein, putative, expressed	2,05	-1.0	1	21	105	125	UGCAUCAUCUCGAACUCGUCG	CGGCGAGUUCCAGAUGAUGCA	Translation
tae-miR1117	Traes_3AL_018D4C27C.1	PF07714,PF12819		2.7.11.1		GO:GO:0006468,GO:GO:0004672	AT3G51550.1	Malectin/receptor-like protein kinase family protein	LOC_Os05g25430.2	receptor-like protein kinase At3g46290 precursor, putative, expressed	1,05	-1.0	1	24	1609	1632	UAGUACCGGUUCGUGGCACGAACC	GGUUCGUGCCACGAACCGGUGCUA	Cleavage
tae-miR1133	Traes_3AL_30294D0A5.1			1.1.1.8		GO:GO:0055114,GO:GO:0009331,GO:GO:0006072,GO:GO:0004367	AT2G41540.3	6-phosphogluconate dehydrogenase family protein	LOC_Os01g71280.1	glycerol-3-phosphate dehydrogenase, putative, expressed	2,05	-1.0	1	22	392	413	CAUAUACUCCCUCCGUCCGAAA	UUUAGGACGGAGGGAGUAUUUG	Cleavage
tae-miR1133	Traes_3AL_386795528.2	PF00249					AT5G02320.2	myb domain protein 3r-5	LOC_Os01g62410.1	MYB family transcription factor, putative, expressed	0,00	-1.0	1	22	39	60	CAUAUACUCCCUCCGUCCGAAA	UUUCGGACGGAGGGAGUAUAUG	Cleavage
tae-miR1122a	Traes_3AL_5E5C8D264.2								LOC_Os05g46470.1	expressed protein	1,00	-1.0	1	20	18	37	UAGAUACAUCCGUAUCUAGA	UCUAGACACGGAUGUAUCUA	Cleavage
tae-miR1137b-5p	Traes_3AL_7A4D34425.2						AT1G29820.2	Magnesium transporter CorA-like family protein	LOC_Os01g47460.1	expressed protein	2,05	-1.0	1	21	1006	1026	UCCGUUCCAGAAUAGAUGACC	UAUCAUUUAUUCAGGAACGGA	Cleavage
tae-miR1122a	Traes_3AL_B9EDE61D1.1	PF06017				GO:GO:0016459,GO:GO:0003774	AT5G53310.1	myosin heavy chain-related	LOC_Os01g43360.1	myosin-Ie, putative, expressed	2,05	-1.0	1	20	4	23	UAGAUACAUCCGUAUCUAGA	ACUACAUACGGAUGUAUAUA	Cleavage
tae-miR1122a	Traes_3AL_B9EDE61D1.2	PF06017				GO:GO:0016459,GO:GO:0003774	AT5G53310.1	myosin heavy chain-related	LOC_Os01g43360.1	myosin-Ie, putative, expressed	2,05	-1.0	1	20	4	23	UAGAUACAUCCGUAUCUAGA	ACUACAUACGGAUGUAUAUA	Cleavage
tae-miR5049-3p	Traes_3AL_CF1A4ACDE.1	PF03759				GO:GO:0005089	AT1G52240.1	RHO guanyl-nucleotide exchange factor 11	LOC_Os01g55520.1	ATROPGEF7/ROPGEF7, putative, expressed	1,00	-1.0	1	21	917	937	AAUAUGGAUCGGAGGGAGUAC	UCCCUCCCUCCGAUCCAUAUU	Cleavage
tae-miR159b	Traes_3AL_F09C7CA19.1	PF00249					AT5G06100.1	myb domain protein 33	LOC_Os01g59660.2	MYB family transcription factor, putative, expressed	2,05	-1.0	1	21	620	640	UUUGGAUUGAAGGGAGCUCUG	UGGAGCUCCCUUCACUCCAAG	Cleavage
tae-miR159a	Traes_3AL_F09C7CA19.1	PF00249					AT5G06100.1	myb domain protein 33	LOC_Os01g59660.2	MYB family transcription factor, putative, expressed	2,05	-1.0	1	21	620	640	UUUGGAUUGAAGGGAGCUCUG	UGGAGCUCCCUUCACUCCAAG	Cleavage
tae-miR1121	Traes_3AL_F2EDB3661.7										2,00	-1.0	1	22	224	245	AGUAGUGAUCUAAACGCUCUUA	CCUCGCGUUUAGAUCACUACU	Cleavage
tae-miR1133	Traes_3AL_FA6F160DF.7										3,00	-1.0	1	22	142	164	CAUAUACUCCCUCCG-UCCGAAA	UUUCGGAACGGAGGGAGUAUAUU	Cleavage
tae-miR5200	Traes_3AS_2A0765E10.1	PF14531		2.7.11.1			AT1G62400.1	Protein kinase superfamily protein	LOC_Os01g01740.1	protein kinase domain containing protein, expressed	2,05	-1.0	1	21	177	197	UGUAGAUACUCCCUAAGGCUU	UUCCCUUAGGAAGUAUCUACA	Translation
tae-miR5200	Traes_3AS_2A0765E10.2	PF14531		2.7.11.1			AT1G62400.1	Protein kinase superfamily protein	LOC_Os01g01740.1	protein kinase domain containing protein, expressed	2,05	-1.0	1	21	66	86	UGUAGAUACUCCCUAAGGCUU	UUCCCUUAGGAAGUAUCUACA	Translation
tae-miR5200	Traes_3AS_2A0765E10.3	PF14531		2.7.11.1			AT1G62400.1	Protein kinase superfamily protein	LOC_Os01g01740.1	protein kinase domain containing protein, expressed	2,05	-1.0	1	21	12	32	UGUAGAUACUCCCUAAGGCUU	UUCCCUUAGGAAGUAUCUACA	Translation
tae-miR9674b-5p	Traes_3AS_6CB1DBD28.1	PF01535,PF13041					AT3G09040.1	Pentatricopeptide repeat (PPR) superfamily protein	LOC_Os04g43430.3	pentatricopeptide, putative, expressed	2,00	-1.0	1	21	429	449	AUAGCAUCAUCCAUCCUACCC	GGGUAGGCUGGAUGAUGCUAG	Cleavage
tae-miR1121	Traes_3AS_A466B9ECC.1	PF13424					AT3G54360.1	zinc ion binding	LOC_Os01g01420.2	expressed protein	0,00	-1.0	1	22	725	746	AGUAGUGAUCUAAACGCUCUUA	UAAGAGCGUUUAGAUCACUACU	Cleavage
tae-miR6197-5p	Traes_3AS_A466B9ECC.1	PF13424					AT3G54360.1	zinc ion binding	LOC_Os01g01420.2	expressed protein	2,05	-1.0	1	21	763	783	UCUGUAAACAAAUGUAGGACG	GCUCUUACAUUUCUUUACAGA	Cleavage
tae-miR1121	Traes_3AS_A466B9ECC.2	PF13424					AT3G54360.1	zinc ion binding	LOC_Os01g01420.2	expressed protein	0,00	-1.0	1	22	725	746	AGUAGUGAUCUAAACGCUCUUA	UAAGAGCGUUUAGAUCACUACU	Cleavage
tae-miR6197-5p	Traes_3AS_A466B9ECC.2	PF13424					AT3G54360.1	zinc ion binding	LOC_Os01g01420.2	expressed protein	2,05	-1.0	1	21	763	783	UCUGUAAACAAAUGUAGGACG	GCUCUUACAUUUCUUUACAGA	Cleavage
tae-miR1133	Traes_3AS_A6D3DD27E.2						AT4G32920.2	glycine-rich protein	LOC_Os01g09246.1	glycine-rich protein, putative, expressed	2,05	-1.0	1	22	16	37	CAUAUACUCCCUCCGUCCGAAA	UUUGGGACGGAGGGAGUAUGUG	Cleavage
tae-miR1133	Traes_3AS_A6D3DD27E.4						AT4G32920.1	glycine-rich protein	LOC_Os01g09246.1	glycine-rich protein, putative, expressed	2,05	-1.0	1	22	9	30	CAUAUACUCCCUCCGUCCGAAA	UUUGGGACGGAGGGAGUAUGUG	Cleavage
tae-miR1130b-3p	Traes_3AS_AA474B823.1	PF00725,PF16113,PF02737	KOG2304	5.1.2.3,4.2.1.17,1.1.1.35,5.3.3.8		GO:GO:0055114,GO:GO:0016491,GO:GO:0006631,GO:GO:0003857	AT4G29010.1	Enoyl-CoA hydratase/isomerase family	LOC_Os02g17390.1	3-hydroxyacyl-CoA dehydrogenase, putative, expressed	1,05	-1.0	1	21	1652	1672	UCUUAUAUUAUGGGACGGAGG	CCUCCGUCCCAUAAUAUGAGA	Cleavage
tae-miR1122c-3p	Traes_3AS_AA474B823.1	PF00725,PF16113,PF02737	KOG2304	5.1.2.3,4.2.1.17,1.1.1.35,5.3.3.8		GO:GO:0055114,GO:GO:0016491,GO:GO:0006631,GO:GO:0003857	AT4G29010.1	Enoyl-CoA hydratase/isomerase family	LOC_Os02g17390.1	3-hydroxyacyl-CoA dehydrogenase, putative, expressed	1,05	-1.0	1	21	1652	1672	UCUAAUAUUAUGGGACGGAGG	CCUCCGUCCCAUAAUAUGAGA	Cleavage
tae-miR1130b-3p	Traes_3AS_AA474B823.3	PF00725,PF00378,PF02737	KOG2304	5.1.2.3,4.2.1.17,1.1.1.35,5.3.3.8		GO:GO:0055114,GO:GO:0016491,GO:GO:0006631,GO:GO:0003857,GO:GO:0008152,GO:GO:0003824	AT4G29010.1	Enoyl-CoA hydratase/isomerase family	LOC_Os02g17390.1	3-hydroxyacyl-CoA dehydrogenase, putative, expressed	1,05	-1.0	1	21	1397	1417	UCUUAUAUUAUGGGACGGAGG	CCUCCGUCCCAUAAUAUGAGA	Cleavage
tae-miR1122c-3p	Traes_3AS_AA474B823.3	PF00725,PF00378,PF02737	KOG2304	5.1.2.3,4.2.1.17,1.1.1.35,5.3.3.8		GO:GO:0055114,GO:GO:0016491,GO:GO:0006631,GO:GO:0003857,GO:GO:0008152,GO:GO:0003824	AT4G29010.1	Enoyl-CoA hydratase/isomerase family	LOC_Os02g17390.1	3-hydroxyacyl-CoA dehydrogenase, putative, expressed	1,05	-1.0	1	21	1397	1417	UCUAAUAUUAUGGGACGGAGG	CCUCCGUCCCAUAAUAUGAGA	Cleavage
tae-miR159b	Traes_3AS_D1D1A1994.1								LOC_Os01g11430.1	expressed protein	2,05	-1.0	1	21	101	121	UUUGGAUUGAAGGGAGCUCUG	CAGAGCCCCCUUCAAACCAAA	Cleavage
tae-miR159a	Traes_3AS_D1D1A1994.1								LOC_Os01g11430.1	expressed protein	2,05	-1.0	1	21	101	121	UUUGGAUUGAAGGGAGCUCUG	CAGAGCCCCCUUCAAACCAAA	Cleavage
tae-miR1122b-3p	Traes_3AS_D7F2F36BB.2						AT3G49720.2		LOC_Os01g05070.1	expressed protein	1,05	-1.0	1	21	564	584	AGACUUAUAUGUAGGAACGGA	UCCGUUCCUAAAUAUAAGUCU	Translation
tae-miR1122b-3p	Traes_3AS_D7F2F36BB.3						AT3G49720.1		LOC_Os01g05070.1	expressed protein	1,05	-1.0	1	21	540	560	AGACUUAUAUGUAGGAACGGA	UCCGUUCCUAAAUAUAAGUCU	Translation
tae-miR5200	Traes_3B_0E2173F88.1	PF14531	KOG0580	2.7.11.1			AT1G62400.1	Protein kinase superfamily protein	LOC_Os01g01740.1	protein kinase domain containing protein, expressed	2,05	-1.0	1	21	171	191	UGUAGAUACUCCCUAAGGCUU	UUCCCUUAGGAAGUAUCUACA	Translation
tae-miR5200	Traes_3B_0E2173F88.2	PF14531	KOG1164	2.7.11.1			AT1G62400.1	Protein kinase superfamily protein	LOC_Os01g01740.1	protein kinase domain containing protein, expressed	2,05	-1.0	1	21	177	197	UGUAGAUACUCCCUAAGGCUU	UUCCCUUAGGAAGUAUCUACA	Translation
tae-miR5200	Traes_3B_0E2173F88.3	PF14531		2.7.11.1			AT1G62400.1	Protein kinase superfamily protein	LOC_Os01g01740.1	protein kinase domain containing protein, expressed	2,05	-1.0	1	21	171	191	UGUAGAUACUCCCUAAGGCUU	UUCCCUUAGGAAGUAUCUACA	Translation
tae-miR9652-5p	Traes_3B_119F6A405.1										2,05	-1.0	1	22	589	610	CCUGUUUGUCAUUAAGUUUCUU	AAGAAACUGAAUGACAAGCAGG	Cleavage
tae-miR9662a-3p	Traes_3B_224A6B7E5.1	PF02536				GO:GO:0006355,GO:GO:0005739,GO:GO:0003690	AT5G07900.1	Mitochondrial transcription termination factor family protein	LOC_Os06g12100.1	mTERF family protein, expressed	2,00	-1.0	1	21	189	209	UUGAACAUCCCAGAGCCACCG	CCGUGGUUCUGGGAUGUUCAG	Cleavage
tae-miR9662b-3p	Traes_3B_224A6B7E5.1	PF02536				GO:GO:0006355,GO:GO:0005739,GO:GO:0003690	AT5G07900.1	Mitochondrial transcription termination factor family protein	LOC_Os06g12100.1	mTERF family protein, expressed	2,00	-1.0	1	21	188	208	UGAACAUCCCAGAGCCACCGG	CCCGUGGUUCUGGGAUGUUCA	Cleavage
tae-miR159b	Traes_3B_25320ED72.1	PF00249	KOG0048		K09422		AT5G06100.1	myb domain protein 33	LOC_Os01g59660.2	MYB family transcription factor, putative, expressed	2,05	-1.0	1	21	956	976	UUUGGAUUGAAGGGAGCUCUG	UGGAGCUCCCUUCACUCCAAG	Cleavage
tae-miR159a	Traes_3B_25320ED72.1	PF00249	KOG0048		K09422		AT5G06100.1	myb domain protein 33	LOC_Os01g59660.2	MYB family transcription factor, putative, expressed	2,05	-1.0	1	21	956	976	UUUGGAUUGAAGGGAGCUCUG	UGGAGCUCCCUUCACUCCAAG	Cleavage
tae-miR159b	Traes_3B_25320ED72.2	PF00249	KOG0048		K09422		AT5G06100.1	myb domain protein 33	LOC_Os01g59660.1	MYB family transcription factor, putative, expressed	2,05	-1.0	1	21	956	976	UUUGGAUUGAAGGGAGCUCUG	UGGAGCUCCCUUCACUCCAAG	Cleavage
tae-miR159a	Traes_3B_25320ED72.2	PF00249	KOG0048		K09422		AT5G06100.1	myb domain protein 33	LOC_Os01g59660.1	MYB family transcription factor, putative, expressed	2,05	-1.0	1	21	956	976	UUUGGAUUGAAGGGAGCUCUG	UGGAGCUCCCUUCACUCCAAG	Cleavage
tae-miR1130b-3p	Traes_3B_467787390.8	PF00917,PF13423,PF14533,PF12436		3.4.19.12		GO:GO:0005515	AT3G11910.1	ubiquitin-specific protease 13	LOC_Os01g56490.1	ubiquitin carboxyl-terminal hydrolase, putative, expressed	1,05	-1.0	1	21	3080	3100	UCUUAUAUUAUGGGACGGAGG	CCUCCGUCCCAAAAUAUAAGA	Translation
tae-miR9776	Traes_3B_470061369.1	PF00270		3.6.4.13		GO:GO:0005524,GO:GO:0003676	AT5G51280.1	DEAD-box protein abstrakt, putative	LOC_Os02g05660.1	DEAD-box ATP-dependent RNA helicase 35A, putative, expressed	2,05	-1.0	1	21	34	54	UUGGACGAGGAUGUGCAACUG	AAGUGGCACAUCCUCGUCGAA	Cleavage
tae-miR1122a	Traes_3B_472072804.1	PF04874,PF01778	KOG3064		K14831		AT1G23280.1	MAK16 protein-related	LOC_Os07g16950.1	Mak16 protein domain containing protein, expressed	1,05	-1.0	1	20	789	808	UAGAUACAUCCGUAUCUAGA	UCUAGAUACGGAGGUAUCUA	Cleavage
tae-miR9778	Traes_3B_4C839468C.10						AT3G46530.1	NB-ARC domain-containing disease resistance protein	LOC_Os12g37290.1	resistance protein T10rga2-1A, putative, expressed	1,00	-1.0	1	21	126	146	UGCAUCAUCUCGAACUCGUCG	GGACGAGCUCGAGAUGAUGCA	Cleavage
tae-miR9778	Traes_3B_4C839468C.11						AT3G46530.1	NB-ARC domain-containing disease resistance protein	LOC_Os12g37290.1	resistance protein T10rga2-1A, putative, expressed	1,00	-1.0	1	21	126	146	UGCAUCAUCUCGAACUCGUCG	GGACGAGCUCGAGAUGAUGCA	Cleavage
tae-miR9778	Traes_3B_4C839468C.12						AT3G46530.1	NB-ARC domain-containing disease resistance protein	LOC_Os12g37290.1	resistance protein T10rga2-1A, putative, expressed	1,00	-1.0	1	21	126	146	UGCAUCAUCUCGAACUCGUCG	GGACGAGCUCGAGAUGAUGCA	Cleavage
tae-miR9778	Traes_3B_4C839468C.13						AT1G50180.1	NB-ARC domain-containing disease resistance protein	LOC_Os02g57310.1	pib, putative, expressed	1,00	-1.0	1	21	102	122	UGCAUCAUCUCGAACUCGUCG	GGACGAGCUCGAGAUGAUGCA	Cleavage
tae-miR9778	Traes_3B_4C839468C.2						AT3G46530.1	NB-ARC domain-containing disease resistance protein	LOC_Os12g37290.1	resistance protein T10rga2-1A, putative, expressed	1,00	-1.0	1	21	126	146	UGCAUCAUCUCGAACUCGUCG	GGACGAGCUCGAGAUGAUGCA	Cleavage
tae-miR9778	Traes_3B_4C839468C.3						AT3G46530.1	NB-ARC domain-containing disease resistance protein	LOC_Os12g37290.1	resistance protein T10rga2-1A, putative, expressed	1,00	-1.0	1	21	126	146	UGCAUCAUCUCGAACUCGUCG	GGACGAGCUCGAGAUGAUGCA	Cleavage
tae-miR9778	Traes_3B_4C839468C.4						AT3G46530.1	NB-ARC domain-containing disease resistance protein	LOC_Os12g37290.1	resistance protein T10rga2-1A, putative, expressed	1,00	-1.0	1	21	126	146	UGCAUCAUCUCGAACUCGUCG	GGACGAGCUCGAGAUGAUGCA	Cleavage
tae-miR9778	Traes_3B_4C839468C.5						AT3G46530.1	NB-ARC domain-containing disease resistance protein	LOC_Os12g37290.1	resistance protein T10rga2-1A, putative, expressed	1,00	-1.0	1	21	126	146	UGCAUCAUCUCGAACUCGUCG	GGACGAGCUCGAGAUGAUGCA	Cleavage
tae-miR9778	Traes_3B_4C839468C.6						AT3G46530.1	NB-ARC domain-containing disease resistance protein	LOC_Os12g37290.1	resistance protein T10rga2-1A, putative, expressed	1,00	-1.0	1	21	126	146	UGCAUCAUCUCGAACUCGUCG	GGACGAGCUCGAGAUGAUGCA	Cleavage
tae-miR9778	Traes_3B_4C839468C.7						AT3G46530.1	NB-ARC domain-containing disease resistance protein	LOC_Os12g37290.1	resistance protein T10rga2-1A, putative, expressed	1,00	-1.0	1	21	126	146	UGCAUCAUCUCGAACUCGUCG	GGACGAGCUCGAGAUGAUGCA	Cleavage
tae-miR9778	Traes_3B_4C839468C.8						AT3G46530.1	NB-ARC domain-containing disease resistance protein	LOC_Os12g37290.1	resistance protein T10rga2-1A, putative, expressed	1,00	-1.0	1	21	126	146	UGCAUCAUCUCGAACUCGUCG	GGACGAGCUCGAGAUGAUGCA	Cleavage
tae-miR9778	Traes_3B_4C839468C.9						AT3G46530.1	NB-ARC domain-containing disease resistance protein	LOC_Os12g37290.1	resistance protein T10rga2-1A, putative, expressed	1,00	-1.0	1	21	126	146	UGCAUCAUCUCGAACUCGUCG	GGACGAGCUCGAGAUGAUGCA	Cleavage
tae-miR1137a	Traes_3B_59B827C5C.2	PF01912	KOG3185			GO:GO:0043022,GO:GO:0042256	AT3G55620.1	Translation initiation factor IF6	LOC_Os07g44620.2	eukaryotic translation initiation factor 6, putative, expressed	0,00	-1.0	1	20	748	767	UAGUACAAAGUUGAGUCAUC	GAUGACUCAACUUUGUACUA	Cleavage
tae-miR1122b-3p	Traes_3B_5DD9C1BE5.1								LOC_Os01g40830.1	transposon protein, putative, Mutator sub-class, expressed	2,05	-1.0	1	21	71	91	AGACUUAUAUGUAGGAACGGA	UCCGUUCUUAAAUAUAAGUCU	Translation
tae-miR7757-5p	Traes_3B_5EFB9A61F.1								LOC_Os12g17490.1	stripe rust resistance protein Yr10, putative, expressed	1,05	-1.0	1	22	113	134	AUAAAACCUUCAGCUAUCCAUC	GAUGGAUAGCUGAAGGUUUUGU	Cleavage
tae-miR7757-5p	Traes_3B_5EFB9A61F.3								LOC_Os12g17490.1	stripe rust resistance protein Yr10, putative, expressed	1,05	-1.0	1	22	89	110	AUAAAACCUUCAGCUAUCCAUC	GAUGGAUAGCUGAAGGUUUUGU	Cleavage
tae-miR9676-5p	Traes_3B_5F0E1F6A4.1						AT1G52700.1	alpha/beta-Hydrolases superfamily protein	LOC_Os05g51050.1	acyl-protein thioesterase, putative, expressed	2,05	-1.0	1	22	38	59	UGGAUGUCAUCGUGGCCGUACA	UGUACGGACAUGAUGACAUCCA	Cleavage
tae-miR1122b-3p	Traes_3B_6D2DCE280.3	PF00025	KOG0084			GO:GO:0005525	AT4G17530.1	RAB GTPase homolog 1C	LOC_Os01g37800.1	ras-related protein, putative, expressed	2,05	-1.0	1	21	576	596	AGACUUAUAUGUAGGAACGGA	UCUGUUCCUAAAUAUAAGUCU	Translation
tae-miR7757-5p	Traes_3B_7FFFF7BCF.1								LOC_Os02g18510.1	stripe rust resistance protein Yr10, putative, expressed	0,00	-1.0	1	22	89	110	AUAAAACCUUCAGCUAUCCAUC	GAUGGAUAGCUGAAGGUUUUAU	Cleavage
tae-miR531	Traes_3B_879F704A0.1										2,05	-1.0	1	21	155	175	CGCUCGCCGGAGCAGCGUGCA	UCCGCGCUGCUCCGGCGAGGG	Cleavage
tae-miR531	Traes_3B_879F704A0.2										2,05	-1.0	1	21	155	175	CGCUCGCCGGAGCAGCGUGCA	UCCGCGCUGCUCCGGCGAGGG	Cleavage
tae-miR531	Traes_3B_879F704A0.3										2,05	-1.0	1	21	50	70	CGCUCGCCGGAGCAGCGUGCA	UCCGCGCUGCUCCGGCGAGGG	Cleavage
tae-miR531	Traes_3B_879F704A0.4										2,05	-1.0	1	21	50	70	CGCUCGCCGGAGCAGCGUGCA	UCCGCGCUGCUCCGGCGAGGG	Cleavage
tae-miR5049-3p	Traes_3B_9D776A8C4.1	PF00804				GO:GO:0016020	AT3G24350.2	syntaxin of plants 32	LOC_Os01g08400.1	syntaxin, putative, expressed	0,00	-1.0	1	21	575	595	AAUAUGGAUCGGAGGGAGUAC	GUACUCCCUCCGAUCCAUAUU	Cleavage
tae-miR1121	Traes_3B_9F321609E.8										1,05	-1.0	1	22	6	28	AGUAGUGAUCUAAACGCUCU-UA	UAUAGAGCGUUUAGAUCACUAUU	Cleavage
tae-miR9778	Traes_3B_F07C33521.1						AT3G46710.1	NB-ARC domain-containing disease resistance protein	LOC_Os02g57310.1	pib, putative, expressed	1,00	-1.0	1	21	126	146	UGCAUCAUCUCGAACUCGUCG	GGACGAGCUCGAGAUGAUGCA	Cleavage
tae-miR9778	Traes_3B_F07C33521.10						AT3G46710.1	NB-ARC domain-containing disease resistance protein	LOC_Os02g57310.1	pib, putative, expressed	1,00	-1.0	1	21	126	146	UGCAUCAUCUCGAACUCGUCG	GGACGAGCUCGAGAUGAUGCA	Cleavage
tae-miR9778	Traes_3B_F07C33521.2	PF00931				GO:GO:0043531	AT3G46530.1	NB-ARC domain-containing disease resistance protein	LOC_Os08g42670.2	resistance protein, putative, expressed	1,00	-1.0	1	21	126	146	UGCAUCAUCUCGAACUCGUCG	GGACGAGCUCGAGAUGAUGCA	Cleavage
tae-miR9778	Traes_3B_F07C33521.3	PF00931				GO:GO:0043531	AT3G46530.1	NB-ARC domain-containing disease resistance protein	LOC_Os08g42670.2	resistance protein, putative, expressed	1,00	-1.0	1	21	126	146	UGCAUCAUCUCGAACUCGUCG	GGACGAGCUCGAGAUGAUGCA	Cleavage
tae-miR9778	Traes_3B_F07C33521.4	PF00931				GO:GO:0043531	AT3G46530.1	NB-ARC domain-containing disease resistance protein	LOC_Os08g42670.2	resistance protein, putative, expressed	1,00	-1.0	1	21	126	146	UGCAUCAUCUCGAACUCGUCG	GGACGAGCUCGAGAUGAUGCA	Cleavage
tae-miR9778	Traes_3B_F07C33521.5	PF00931				GO:GO:0043531	AT3G46530.1	NB-ARC domain-containing disease resistance protein	LOC_Os08g42670.1	resistance protein, putative, expressed	1,00	-1.0	1	21	126	146	UGCAUCAUCUCGAACUCGUCG	GGACGAGCUCGAGAUGAUGCA	Cleavage
tae-miR9778	Traes_3B_F07C33521.6	PF00931				GO:GO:0043531	AT3G46530.1	NB-ARC domain-containing disease resistance protein	LOC_Os08g42670.1	resistance protein, putative, expressed	1,00	-1.0	1	21	126	146	UGCAUCAUCUCGAACUCGUCG	GGACGAGCUCGAGAUGAUGCA	Cleavage
tae-miR9778	Traes_3B_F07C33521.7	PF00931				GO:GO:0043531	AT3G46530.1	NB-ARC domain-containing disease resistance protein	LOC_Os08g42670.2	resistance protein, putative, expressed	1,00	-1.0	1	21	126	146	UGCAUCAUCUCGAACUCGUCG	GGACGAGCUCGAGAUGAUGCA	Cleavage
tae-miR9778	Traes_3B_F07C33521.8						AT3G46710.1	NB-ARC domain-containing disease resistance protein	LOC_Os02g57310.1	pib, putative, expressed	1,00	-1.0	1	21	126	146	UGCAUCAUCUCGAACUCGUCG	GGACGAGCUCGAGAUGAUGCA	Cleavage
tae-miR9778	Traes_3B_F07C33521.9						AT3G46710.1	NB-ARC domain-containing disease resistance protein	LOC_Os02g57310.1	pib, putative, expressed	1,00	-1.0	1	21	126	146	UGCAUCAUCUCGAACUCGUCG	GGACGAGCUCGAGAUGAUGCA	Cleavage
tae-miR1128	Traes_3B_F61EDC1AE.3	PF02212,PF00350,PF01031				GO:GO:0005525,GO:GO:0003924	AT4G33650.1	dynamin-related protein 3A	LOC_Os01g69130.1	dynamin family protein, putative, expressed	2,00	-1.0	1	21	1581	1601	UACUACUCCCUCCGUCCGAAA	UUCCGAACGGAGGGAGUAGUA	Cleavage
tae-miR1127a	Traes_3B_F61EDC1AE.3	PF02212,PF00350,PF01031				GO:GO:0005525,GO:GO:0003924	AT4G33650.1	dynamin-related protein 3A	LOC_Os01g69130.1	dynamin family protein, putative, expressed	1,05	-1.0	1	19	1577	1595	UCCUUCCGUUCGGAAUUAC	GUAAUUCCGAACGGAGGGA	Cleavage
tae-miR1130b-3p	Traes_3B_FB51F185F.2						AT1G53560.1	Ribosomal protein L18ae family	LOC_Os01g14070.1	60S ribosomal protein L18a-1, putative, expressed	1,05	-1.0	1	21	43	63	UCUUAUAUUAUGGGACGGAGG	CCUCCGUCCCAUAAUGUAAGA	Cleavage
tae-miR164	Traes_3B_FD8ACC50C.1			6.3.2.19		GO:GO:0007275,GO:GO:0006511,GO:GO:0005634	AT1G66650.1	Protein with RING/U-box and TRAF-like domains	LOC_Os01g03190.1	expressed protein	2,05	-1.0	1	21	1544	1564	UGGAGAAGCAGGGCACGUGCA	CGCACGGGCCCUGCUUCUGCA	Cleavage
tae-miR164	Traes_3B_FD8ACC50C.2			6.3.2.19		GO:GO:0007275,GO:GO:0006511,GO:GO:0005634	AT1G66650.1	Protein with RING/U-box and TRAF-like domains	LOC_Os01g03190.1	expressed protein	2,05	-1.0	1	21	1499	1519	UGGAGAAGCAGGGCACGUGCA	CGCACGGGCCCUGCUUCUGCA	Cleavage
tae-miR164	Traes_3B_FD8ACC50C.3			6.3.2.19		GO:GO:0007275,GO:GO:0006511,GO:GO:0005634	AT1G66650.1	Protein with RING/U-box and TRAF-like domains	LOC_Os01g03190.1	expressed protein	2,05	-1.0	1	21	1433	1453	UGGAGAAGCAGGGCACGUGCA	CGCACGGGCCCUGCUUCUGCA	Cleavage
tae-miR164	Traes_3B_FD8ACC50C.4			6.3.2.19		GO:GO:0007275,GO:GO:0006511,GO:GO:0005634	AT1G66650.1	Protein with RING/U-box and TRAF-like domains	LOC_Os01g03190.1	expressed protein	2,05	-1.0	1	21	764	784	UGGAGAAGCAGGGCACGUGCA	CGCACGGGCCCUGCUUCUGCA	Cleavage
tae-miR164	Traes_3B_FD8ACC50C.5			6.3.2.19		GO:GO:0007275,GO:GO:0006511,GO:GO:0005634	AT1G66650.1	Protein with RING/U-box and TRAF-like domains	LOC_Os01g03190.1	expressed protein	2,05	-1.0	1	21	752	772	UGGAGAAGCAGGGCACGUGCA	CGCACGGGCCCUGCUUCUGCA	Cleavage
tae-miR164	Traes_3B_FD8ACC50C.6			6.3.2.19		GO:GO:0007275,GO:GO:0006511,GO:GO:0005634	AT1G66650.1	Protein with RING/U-box and TRAF-like domains	LOC_Os01g03190.1	expressed protein	2,05	-1.0	1	21	731	751	UGGAGAAGCAGGGCACGUGCA	CGCACGGGCCCUGCUUCUGCA	Cleavage
tae-miR164	Traes_3B_FD8ACC50C.7			6.3.2.19		GO:GO:0007275,GO:GO:0006511,GO:GO:0005634	AT1G66650.1	Protein with RING/U-box and TRAF-like domains	LOC_Os01g03190.1	expressed protein	2,05	-1.0	1	21	710	730	UGGAGAAGCAGGGCACGUGCA	CGCACGGGCCCUGCUUCUGCA	Cleavage
tae-miR164	Traes_3B_FD8ACC50C.8			6.3.2.19		GO:GO:0007275,GO:GO:0006511,GO:GO:0005634	AT1G66650.1	Protein with RING/U-box and TRAF-like domains	LOC_Os01g03190.1	expressed protein	2,05	-1.0	1	21	653	673	UGGAGAAGCAGGGCACGUGCA	CGCACGGGCCCUGCUUCUGCA	Cleavage
tae-miR164	Traes_3B_FD8ACC50C.9			6.3.2.19		GO:GO:0007275,GO:GO:0006511,GO:GO:0005634	AT1G66650.1	Protein with RING/U-box and TRAF-like domains	LOC_Os01g03190.1	expressed protein	2,05	-1.0	1	21	356	376	UGGAGAAGCAGGGCACGUGCA	CGCACGGGCCCUGCUUCUGCA	Cleavage
tae-miR5049-3p	Traes_3DL_2B3694DC3.2	PF01217					AT3G09800.1	SNARE-like superfamily protein	LOC_Os05g38610.1	clathrin adaptor complex small chain domain containing protein, expressed	1,05	-1.0	1	21	228	248	AAUAUGGAUCGGAGGGAGUAC	CUACUCCCUCCGAUCCAAAUU	Cleavage
tae-miR1130b-3p	Traes_3DL_426EF6A68.2										2,05	-1.0	1	21	214	234	UCUUAUAUUAUGGGACGGAGG	CCUCCAUCCCGUAAUAUAAGA	Translation
tae-miR7757-5p	Traes_3DL_5C0BC5611.4								LOC_Os12g17490.1	stripe rust resistance protein Yr10, putative, expressed	1,05	-1.0	1	22	89	110	AUAAAACCUUCAGCUAUCCAUC	GAUGGAUAGCUGAAGGUUUUGU	Cleavage
tae-miR1137a	Traes_3DL_96847DCCF.1	PF00069	KOG1187	2.7.11.1		GO:GO:0006468,GO:GO:0005524,GO:GO:0004672	AT1G34300.1	lectin protein kinase family protein	LOC_Os01g47840.1	S-locus-like receptor protein kinase, putative, expressed	1,00	-1.0	1	20	88	107	UAGUACAAAGUUGAGUCAUC	GAUGACCCAACUUUGUACUA	Cleavage
tae-miR5049-3p	Traes_3DL_D1CF4784D.2	PF13307,PF06733		3.6.4.12		GO:GO:0016818,GO:GO:0008026,GO:GO:0006139,GO:GO:0005524,GO:GO:0003676,GO:GO:0004003,GO:GO:0003677	AT1G79950.1	RAD3-like DNA-binding helicase protein	LOC_Os01g40980.1	helicase, putative, expressed	2,05	-1.0	1	21	1646	1666	AAUAUGGAUCGGAGGGAGUAC	AUAAUCCCUCCGUUCCAUAUU	Cleavage
tae-miR159b	Traes_3DL_D9A29948B.1	PF00249					AT5G06100.1	myb domain protein 33	LOC_Os01g59660.1	MYB family transcription factor, putative, expressed	2,05	-1.0	1	21	620	640	UUUGGAUUGAAGGGAGCUCUG	UGGAGCUCCCUUCACUCCAAG	Cleavage
tae-miR159a	Traes_3DL_D9A29948B.1	PF00249					AT5G06100.1	myb domain protein 33	LOC_Os01g59660.1	MYB family transcription factor, putative, expressed	2,05	-1.0	1	21	620	640	UUUGGAUUGAAGGGAGCUCUG	UGGAGCUCCCUUCACUCCAAG	Cleavage
tae-miR159b	Traes_3DL_D9A29948B.2	PF00249					AT5G06100.1	myb domain protein 33	LOC_Os01g59660.1	MYB family transcription factor, putative, expressed	2,05	-1.0	1	21	620	640	UUUGGAUUGAAGGGAGCUCUG	UGGAGCUCCCUUCACUCCAAG	Cleavage
tae-miR159a	Traes_3DL_D9A29948B.2	PF00249					AT5G06100.1	myb domain protein 33	LOC_Os01g59660.1	MYB family transcription factor, putative, expressed	2,05	-1.0	1	21	620	640	UUUGGAUUGAAGGGAGCUCUG	UGGAGCUCCCUUCACUCCAAG	Cleavage
tae-miR1128	Traes_3DS_1DE20CFF3.2	PF01121	KOG3220	2.7.1.24	K00859	GO:GO:0015937,GO:GO:0005524,GO:GO:0004140	AT2G27490.1	dephospho-CoA kinase family	LOC_Os01g25880.1	dephospho-CoA kinase, putative, expressed	2,05	-1.0	1	21	71	91	UACUACUCCCUCCGUCCGAAA	UUUGGGACGGAGGGAGUACUA	Cleavage
tae-miR1122a	Traes_3DS_1DE20CFF3.2	PF01121	KOG3220	2.7.1.24	K00859	GO:GO:0015937,GO:GO:0005524,GO:GO:0004140	AT2G27490.1	dephospho-CoA kinase family	LOC_Os01g25880.1	dephospho-CoA kinase, putative, expressed	1,05	-1.0	1	20	2	21	UAGAUACAUCCGUAUCUAGA	UCUAGAUACAGAUGUAUCUA	Translation
tae-miR1128	Traes_3DS_1DE20CFF3.3	PF01121	KOG3220	2.7.1.24	K00859	GO:GO:0015937,GO:GO:0005524,GO:GO:0004140	AT2G27490.1	dephospho-CoA kinase family	LOC_Os01g25880.1	dephospho-CoA kinase, putative, expressed	2,05	-1.0	1	21	71	91	UACUACUCCCUCCGUCCGAAA	UUUGGGACGGAGGGAGUACUA	Cleavage
tae-miR1122a	Traes_3DS_1DE20CFF3.3	PF01121	KOG3220	2.7.1.24	K00859	GO:GO:0015937,GO:GO:0005524,GO:GO:0004140	AT2G27490.1	dephospho-CoA kinase family	LOC_Os01g25880.1	dephospho-CoA kinase, putative, expressed	1,05	-1.0	1	20	2	21	UAGAUACAUCCGUAUCUAGA	UCUAGAUACAGAUGUAUCUA	Translation
tae-miR1122b-3p	Traes_3DS_D42FCD586.1	PF00274		4.1.2.13		GO:GO:0006096,GO:GO:0004332	AT2G01140.1	Aldolase superfamily protein	LOC_Os01g02880.1	fructose-bisphospate aldolase isozyme, putative, expressed	1,05	-1.0	1	21	786	806	AGACUUAUAUGUAGGAACGGA	UCCGUUCCUAAAUAUAAGUCU	Translation
tae-miR1122b-3p	Traes_3DS_D42FCD586.2	PF00274		4.1.2.13		GO:GO:0006096,GO:GO:0004332	AT2G01140.1	Aldolase superfamily protein	LOC_Os01g02880.1	fructose-bisphospate aldolase isozyme, putative, expressed	1,05	-1.0	1	21	795	815	AGACUUAUAUGUAGGAACGGA	UCCGUUCCUAAAUAUAAGUCU	Translation
tae-miR7757-5p	Traes_4AL_13A3A40D3.1	PF13401	KOG4658				AT3G50950.2	HOPZ-ACTIVATED RESISTANCE 1	LOC_Os11g11810.1	NBS-LRR disease resistance protein, putative, expressed	1,05	-1.0	1	22	1337	1358	AUAAAACCUUCAGCUAUCCAUC	AGUGGAUAGCUGAAGGUUUCAU	Cleavage
tae-miR1135	Traes_4AL_158B970F4.1						AT1G50020.1		LOC_Os11g34870.1	expressed protein	2,05	-1.0	1	24	464	487	CUGCGACAAGUAAUUCCGAACGGA	UCCGUUCGGAAUUACUCGUCGCAG	Cleavage
tae-miR5175-5p	Traes_4AL_158B970F4.1						AT1G50020.1		LOC_Os11g34870.1	expressed protein	3,00	-1.0	1	21	532	552	UUCCAAAUUACUCGUCGUGGU	UCUGAGACGAGUAAUUUGGAA	Cleavage
tae-miR1117	Traes_4AL_2475C298D.1	PF07993		1.2.1.84		GO:GO:0080019	AT3G44540.1	fatty acid reductase 4	LOC_Os04g28600.1	male sterility protein, putative, expressed	2,05	-1.0	1	24	684	707	UAGUACCGGUUCGUGGCACGAACC	GGUUCAUGCCACGAACCGGUACUA	Cleavage
tae-miR9778	Traes_4AL_2FC39B934.1	PF13191,PF00931				GO:GO:0043531	AT1G53350.1	Disease resistance protein (CC-NBS-LRR class) family	LOC_Os08g14850.1	resistance protein, putative, expressed	2,05	-1.0	1	21	126	146	UGCAUCAUCUCGAACUCGUCG	UGGCGAGUUCCAGAUGAUGCA	Translation
tae-miR408	Traes_4AL_364F56EDB.1	PF02298				GO:GO:0009055	AT2G02850.1	plantacyanin	LOC_Os03g50160.1	plastocyanin-like domain containing protein, putative, expressed	0,00	-1.0	1	21	8	28	CUGCACUGCCUCUUCCCUGGC	CCCAGGGAAGAGGCAGUGCAG	Cleavage
tae-miR9662a-3p	Traes_4AL_3A009739E.1	PF02536				GO:GO:0006355,GO:GO:0005739,GO:GO:0003690	AT1G21150.1	Mitochondrial transcription termination factor family protein	LOC_Os06g12100.1	mTERF family protein, expressed	2,05	-1.0	1	21	66	86	UUGAACAUCCCAGAGCCACCG	CCGUGGCUCUGGAAUGUUCAG	Cleavage
tae-miR9662b-3p	Traes_4AL_3A009739E.1	PF02536				GO:GO:0006355,GO:GO:0005739,GO:GO:0003690	AT1G21150.1	Mitochondrial transcription termination factor family protein	LOC_Os06g12100.1	mTERF family protein, expressed	2,05	-1.0	1	21	65	85	UGAACAUCCCAGAGCCACCGG	CCCGUGGCUCUGGAAUGUUCA	Cleavage
tae-miR1121	Traes_4AL_400EB3C17.10	PF14259,PF00076	KOG0120			GO:GO:0003676	AT4G36690.4	U2 snRNP auxilliary factor, large subunit, splicing factor	LOC_Os11g41820.2	RNA recognition motif containing protein, expressed	2,05	-1.0	1	22	10	31	AGUAGUGAUCUAAACGCUCUUA	UAAGAGCGUUUAGAUCACUAAA	Cleavage
tae-miR1121	Traes_4AL_400EB3C17.3	PF14259,PF00076	KOG0120			GO:GO:0003676	AT4G36690.4	U2 snRNP auxilliary factor, large subunit, splicing factor	LOC_Os11g41820.1	RNA recognition motif containing protein, expressed	2,05	-1.0	1	22	37	58	AGUAGUGAUCUAAACGCUCUUA	UAAGAGCGUUUAGAUCACUAAA	Cleavage
tae-miR1121	Traes_4AL_400EB3C17.7	PF14259,PF00076	KOG0120			GO:GO:0003676	AT4G36690.4	U2 snRNP auxilliary factor, large subunit, splicing factor	LOC_Os11g41820.1	RNA recognition motif containing protein, expressed	2,05	-1.0	1	22	10	31	AGUAGUGAUCUAAACGCUCUUA	UAAGAGCGUUUAGAUCACUAAA	Cleavage
tae-miR1137a	Traes_4AL_40790E994.6	PF05185		2.1.1.125,2.1.1.126		GO:GO:0008168,GO:GO:0006479	AT4G16570.1	protein arginine methyltransferase 7	LOC_Os06g01640.1	arginine N-methyltransferase 7, putative, expressed	1,00	-1.0	1	20	1324	1343	UAGUACAAAGUUGAGUCAUC	GAUGACCCAACUUUGUACUA	Cleavage
tae-miR1137a	Traes_4AL_40790E994.7			2.1.1.125,2.1.1.126		GO:GO:0008168,GO:GO:0006479	AT4G16570.1	protein arginine methyltransferase 7	LOC_Os06g01640.1	arginine N-methyltransferase 7, putative, expressed	1,00	-1.0	1	20	835	854	UAGUACAAAGUUGAGUCAUC	GAUGACCCAACUUUGUACUA	Cleavage
tae-miR1127b-3p	Traes_4AL_44E99BA01.2	PF07714		2.7.11.1		GO:GO:0006468,GO:GO:0004672	AT4G33950.1	Protein kinase superfamily protein	LOC_Os03g41460.1	CAMK_CAMK_like.20 - CAMK includes calcium/calmodulin depedent protein kinases, expressed	1,05	-1.0	1	21	394	414	ACAAGUAUUUCUGGACGGAGG	CCUCCGUCCGGAAAUACUUGU	Cleavage
tae-miR1121	Traes_4AL_541C86EB9.2	PF00076				GO:GO:0003676,GO:GO:0017069,GO:GO:0000398	AT1G21320.1	nucleotide binding;nucleic acid binding	LOC_Os06g03690.2	RNA recognition motif containing protein, putative, expressed	1,00	-1.0	1	22	558	579	AGUAGUGAUCUAAACGCUCUUA	UGAGAUCGUUUAGAUCACUACU	Cleavage
tae-miR1121	Traes_4AL_541C86EB9.3	PF00076				GO:GO:0003676,GO:GO:0017069,GO:GO:0000398	AT1G21320.1	nucleotide binding;nucleic acid binding	LOC_Os06g03690.2	RNA recognition motif containing protein, putative, expressed	1,00	-1.0	1	22	558	579	AGUAGUGAUCUAAACGCUCUUA	UGAGAUCGUUUAGAUCACUACU	Cleavage
tae-miR1122b-3p	Traes_4AL_6252B0BA0.1	PF05562					AT1G29395.1	COLD REGULATED 314 INNER MEMBRANE 1	LOC_Os05g49170.1	cold acclimation protein WCOR413, putative, expressed	1,05	-1.0	1	21	46	66	AGACUUAUAUGUAGGAACGGA	UCCGUUCCUAAAUAUAAGUCU	Translation
tae-miR1137b-5p	Traes_4AL_748BB4675.1	PF00657		3.1.1.1		GO:GO:0016788	AT5G45910.1	GDSL-like Lipase/Acylhydrolase superfamily protein	LOC_Os01g12320.1	GDSL-like lipase/acylhydrolase, putative, expressed	2,05	-1.0	1	21	844	864	UCCGUUCCAGAAUAGAUGACC	ACUCAUCUAUUCUGGGACGGU	Cleavage
tae-miR9778	Traes_4AL_79B47AF61.1	PF13191,PF00931				GO:GO:0043531	AT3G07040.1	NB-ARC domain-containing disease resistance protein	LOC_Os08g42670.1	resistance protein, putative, expressed	2,05	-1.0	1	21	126	146	UGCAUCAUCUCGAACUCGUCG	GGACGAGCUCGACAUGAUGCA	Cleavage
tae-miR9778	Traes_4AL_79B47AF61.3	PF00931				GO:GO:0043531	AT3G46530.1	NB-ARC domain-containing disease resistance protein	LOC_Os08g42670.2	resistance protein, putative, expressed	2,05	-1.0	1	21	126	146	UGCAUCAUCUCGAACUCGUCG	GGACGAGCUCGACAUGAUGCA	Cleavage
tae-miR9778	Traes_4AL_79B47AF61.4	PF00931				GO:GO:0043531	AT3G46530.1	NB-ARC domain-containing disease resistance protein	LOC_Os08g42670.2	resistance protein, putative, expressed	2,05	-1.0	1	21	126	146	UGCAUCAUCUCGAACUCGUCG	GGACGAGCUCGACAUGAUGCA	Cleavage
tae-miR5048-5p	Traes_4AL_85A7C60F9.1	PF00069		2.7.11.1		GO:GO:0006468,GO:GO:0005524,GO:GO:0004672	AT4G21230.1	cysteine-rich RLK (RECEPTOR-like protein kinase) 27	LOC_Os11g10640.1	protein kinase domain containing protein, expressed	2,05	-1.0	1	22	170	191	UUUGCAGGUUUUAGGUCUAAGU	ACUUGGACCUAAAACCUGCGAA	Cleavage
tae-miR5048-5p	Traes_4AL_85A7C60F9.7	PF00069		2.7.11.1		GO:GO:0006468,GO:GO:0005524,GO:GO:0004672	AT4G21230.1	cysteine-rich RLK (RECEPTOR-like protein kinase) 27	LOC_Os11g10640.1	protein kinase domain containing protein, expressed	2,05	-1.0	1	22	170	191	UUUGCAGGUUUUAGGUCUAAGU	ACUUGGACCUAAAACCUGCGAA	Cleavage
tae-miR5048-5p	Traes_4AL_85A7C60F9.8	PF00069		2.7.11.1		GO:GO:0006468,GO:GO:0005524,GO:GO:0004672	AT4G21230.1	cysteine-rich RLK (RECEPTOR-like protein kinase) 27	LOC_Os11g10640.1	protein kinase domain containing protein, expressed	2,05	-1.0	1	22	170	191	UUUGCAGGUUUUAGGUCUAAGU	ACUUGGACCUAAAACCUGCGAA	Cleavage
tae-miR1122b-3p	Traes_4AL_954AE4933.1	PF12171					AT2G02080.1	indeterminate(ID)-domain 4	LOC_Os04g47860.4	ZOS4-11 - C2H2 zinc finger protein, expressed	1,05	-1.0	1	21	583	603	AGACUUAUAUGUAGGAACGGA	UCCGUUCCUAAAUAUAAGUCU	Translation
tae-miR1122a	Traes_4AL_9726307FF.1	PF01926,PF06421,PF00679		3.6.5.3		GO:GO:0005525	AT5G39900.1	Small GTP-binding protein	LOC_Os06g05250.2	GTP-binding protein GUF1, putative, expressed	0,00	-1.0	1	20	1903	1922	UAGAUACAUCCGUAUCUAGA	UCUAGAUACGGAUGUAUCUA	Cleavage
tae-miR1128	Traes_4AL_99942CBEA.1	PF02365				GO:GO:0006355,GO:GO:0003677	AT2G46770.1	NAC (No Apical Meristem) domain transcriptional regulator superfamily protein	LOC_Os06g04090.1	no apical meristem protein, putative, expressed	3,00	-1.0	1	21	693	714	UACUACUCCCUCCG-UCCGAAA	UUUAGGAACGGAGGGAGUAGUA	Cleavage
tae-miR1122b-3p	Traes_4AL_99942CBEA.1	PF02365				GO:GO:0006355,GO:GO:0003677	AT2G46770.1	NAC (No Apical Meristem) domain transcriptional regulator superfamily protein	LOC_Os06g04090.1	no apical meristem protein, putative, expressed	2,05	-1.0	1	21	557	577	AGACUUAUAUGUAGGAACGGA	UCCGUUCUUAAAUAUAAGUCU	Translation
tae-miR9662a-3p	Traes_4AL_A4B73CC6A1.1	PF02536	KOG1267		K15032	GO:GO:0006355,GO:GO:0005739,GO:GO:0003690	AT5G07900.1	Mitochondrial transcription termination factor family protein	LOC_Os06g12100.1	mTERF family protein, expressed	2,05	-1.0	1	21	693	713	UUGAACAUCCCAGAGCCACCG	CCGUGGCUCUGGAAUGUUCAG	Cleavage
tae-miR9662b-3p	Traes_4AL_A4B73CC6A1.1	PF02536	KOG1267		K15032	GO:GO:0006355,GO:GO:0005739,GO:GO:0003690	AT5G07900.1	Mitochondrial transcription termination factor family protein	LOC_Os06g12100.1	mTERF family protein, expressed	2,05	-1.0	1	21	692	712	UGAACAUCCCAGAGCCACCGG	CCCGUGGCUCUGGAAUGUUCA	Cleavage
tae-miR9662a-3p	Traes_4AL_A4B73CC6A1.2	PF02536	KOG1267		K15032	GO:GO:0006355,GO:GO:0005739,GO:GO:0003690	AT5G07900.1	Mitochondrial transcription termination factor family protein	LOC_Os06g12100.1	mTERF family protein, expressed	2,05	-1.0	1	21	693	713	UUGAACAUCCCAGAGCCACCG	CCGUGGCUCUGGAAUGUUCAG	Cleavage
tae-miR9662b-3p	Traes_4AL_A4B73CC6A1.2	PF02536	KOG1267		K15032	GO:GO:0006355,GO:GO:0005739,GO:GO:0003690	AT5G07900.1	Mitochondrial transcription termination factor family protein	LOC_Os06g12100.1	mTERF family protein, expressed	2,05	-1.0	1	21	692	712	UGAACAUCCCAGAGCCACCGG	CCCGUGGCUCUGGAAUGUUCA	Cleavage
tae-miR1130b-3p	Traes_4AL_C55D89913.1	PF03219				GO:GO:0016021,GO:GO:0006810,GO:GO:0005524,GO:GO:0005471	AT5G05310.2	TLC ATP/ADP transporter	LOC_Os11g02159.1	expressed protein	0,00	-1.0	1	21	10	30	UCUUAUAUUAUGGGACGGAGG	CCUCCGUCCCAUAAUAUAAGA	Cleavage

tae-miR1122c-3p	Traes_4AL_C55D89913.1	PF03219				GO:GO:0016021,GO:GO:0006810,GO:GO:0005524,GO:GO:0005471	AT5G05310.2	TLC ATP/ADP transporter	LOC_Os11g02159.1	expressed protein	1,05	-1.0	1	21	10	30	UCUAAUAUUAUGGGACGGAGG	CCUCCGUCCCAUAAUAUAAGA	Cleavage
tae-miR1130b-3p	Traes_4AL_C55D89913.5	PF03219				GO:GO:0016021,GO:GO:0006810,GO:GO:0005524,GO:GO:0005471	AT5G05310.2	TLC ATP/ADP transporter	LOC_Os11g02159.2	expressed protein	0,00	-1.0	1	21	10	30	UCUUAUAUUAUGGGACGGAGG	CCUCCGUCCCAUAAUAUAAGA	Cleavage
tae-miR1122c-3p	Traes_4AL_C55D89913.5	PF03219				GO:GO:0016021,GO:GO:0006810,GO:GO:0005524,GO:GO:0005471	AT5G05310.2	TLC ATP/ADP transporter	LOC_Os11g02159.2	expressed protein	1,05	-1.0	1	21	10	30	UCUAAUAUUAUGGGACGGAGG	CCUCCGUCCCAUAAUAUAAGA	Cleavage
tae-miR1130b-3p	Traes_4AL_C55D89913.6	PF03219				GO:GO:0016021,GO:GO:0006810,GO:GO:0005524,GO:GO:0005471	AT5G05310.2	TLC ATP/ADP transporter	LOC_Os11g02159.1	expressed protein	0,00	-1.0	1	21	10	30	UCUUAUAUUAUGGGACGGAGG	CCUCCGUCCCAUAAUAUAAGA	Cleavage
tae-miR1122c-3p	Traes_4AL_C55D89913.6	PF03219				GO:GO:0016021,GO:GO:0006810,GO:GO:0005524,GO:GO:0005471	AT5G05310.2	TLC ATP/ADP transporter	LOC_Os11g02159.1	expressed protein	1,05	-1.0	1	21	10	30	UCUAAUAUUAUGGGACGGAGG	CCUCCGUCCCAUAAUAUAAGA	Cleavage
tae-miR1130b-3p	Traes_4AL_C55D89913.7	PF03219				GO:GO:0016021,GO:GO:0006810,GO:GO:0005524,GO:GO:0005471	AT5G05310.2	TLC ATP/ADP transporter	LOC_Os11g02159.2	expressed protein	0,00	-1.0	1	21	10	30	UCUUAUAUUAUGGGACGGAGG	CCUCCGUCCCAUAAUAUAAGA	Cleavage
tae-miR1122c-3p	Traes_4AL_C55D89913.7	PF03219				GO:GO:0016021,GO:GO:0006810,GO:GO:0005524,GO:GO:0005471	AT5G05310.2	TLC ATP/ADP transporter	LOC_Os11g02159.2	expressed protein	1,05	-1.0	1	21	10	30	UCUAAUAUUAUGGGACGGAGG	CCUCCGUCCCAUAAUAUAAGA	Cleavage
tae-miR1130b-3p	Traes_4AL_C55D89913.8					GO:GO:0016021,GO:GO:0006810,GO:GO:0005524,GO:GO:0005471	AT5G05310.2	TLC ATP/ADP transporter	LOC_Os11g02159.2	expressed protein	0,00	-1.0	1	21	10	30	UCUUAUAUUAUGGGACGGAGG	CCUCCGUCCCAUAAUAUAAGA	Cleavage
tae-miR1122c-3p	Traes_4AL_C55D89913.8					GO:GO:0016021,GO:GO:0006810,GO:GO:0005524,GO:GO:0005471	AT5G05310.2	TLC ATP/ADP transporter	LOC_Os11g02159.2	expressed protein	1,05	-1.0	1	21	10	30	UCUAAUAUUAUGGGACGGAGG	CCUCCGUCCCAUAAUAUAAGA	Cleavage
tae-miR5049-3p	Traes_4AL_C9E08C423.5										0,00	-1.0	1	21	69	89	AAUAUGGAUCGGAGGGAGUAC	CUACUCCCUCCGAUCCAUAUU	Cleavage
tae-miR9782	Traes_4AL_D576D98B4.1	PF00657		3.1.1.3		GO:GO:0016788	AT5G03810.1	GDSL-like Lipase/Acylhydrolase family protein	LOC_Os03g47940.1	GDSL-like lipase/acylhydrolase, putative, expressed	1,00	-1.0	1	24	2	25	GUAUUAGGUUGGUCAAAUUGACGA	UCGUCAAUUUGACCAACCUAAUAU	Cleavage
tae-miR9782	Traes_4AL_D576D98B4.2			3.1.1.1			AT5G03810.1	GDSL-like Lipase/Acylhydrolase family protein	LOC_Os03g47940.1	GDSL-like lipase/acylhydrolase, putative, expressed	1,00	-1.0	1	24	2	25	GUAUUAGGUUGGUCAAAUUGACGA	UCGUCAAUUUGACCAACCUAAUAU	Cleavage
tae-miR5384-3p	Traes_4AL_E60E5B7BA.1	PF02861	KOG1051		K03695	GO:GO:0019538	AT3G52490.1	Double Clp-N motif-containing P-loop nucleoside triphosphate hydrolases superfamily protein	LOC_Os11g05820.1	transposon protein, putative, Mutator sub-class, expressed	2,05	-1.0	1	21	269	289	UGAGCGCGCCGCCGUCGAAUG	UGUUCCACGGCGGCGCGCACA	Cleavage
tae-miR5384-3p	Traes_4AL_E60E5B7BA.2		KOG1051				AT3G52490.1	Double Clp-N motif-containing P-loop nucleoside triphosphate hydrolases superfamily protein	LOC_Os11g05820.1	transposon protein, putative, Mutator sub-class, expressed	2,05	-1.0	1	21	140	160	UGAGCGCGCCGCCGUCGAAUG	UGUUCCACGGCGGCGCGCACA	Cleavage
tae-miR1130b-3p	Traes_4AS_0E2735576.2			3.4.21.108					LOC_Os05g05330.1	expressed protein	1,05	-1.0	1	21	977	997	UCUUAUAUUAUGGGACGGAGG	CCUCCGUCUCAUAAUAUAAGA	Cleavage
tae-miR1120c-5p	Traes_4AS_0E2735576.2			3.4.21.108					LOC_Os05g05330.1	expressed protein	0,00	-1.0	1	21	1020	1040	UAAUAUAAGAACGUUUUUGAC	GACAAAAACGUUCUUAUAUUA	Cleavage
tae-miR9674a-5p	Traes_4AS_AB9088E9B.2	PF11955					AT5G21970.1	Ubiquitin carboxyl-terminal hydrolase family protein	LOC_Os11g34160.1	ubiquitin carboxyl-terminal hydrolase, family 1, putative, expressed	2,05	-1.0	1	21	1383	1403	GCAUCAUCCAUCCUACCAUUC	UGAUGGUCAGAUGGAUGAUGC	Cleavage
tae-miR9674a-5p	Traes_4AS_AB9088E9B.3	PF11955					AT5G21970.1	Ubiquitin carboxyl-terminal hydrolase family protein	LOC_Os11g34160.1	ubiquitin carboxyl-terminal hydrolase, family 1, putative, expressed	2,05	-1.0	1	21	1383	1403	GCAUCAUCCAUCCUACCAUUC	UGAUGGUCAGAUGGAUGAUGC	Cleavage
tae-miR5384-3p	Traes_4AS_C7CBD4A47.1	PF03703					AT1G14345.1	NAD(P)-linked oxidoreductase superfamily protein	LOC_Os03g07440.1	putativ eribonuclease P, putative, expressed	2,05	-1.0	1	21	194	214	UGAGCGCGCCGCCGUCGAAUG	UCUUCGACGGCGGCGCGCACU	Cleavage
tae-miR1135	Traes_4AS_D533B306B.3	PF01485	KOG1815	6.3.2.19		GO:GO:0016567,GO:GO:0004842	AT2G16090.1	RING/U-box superfamily protein	LOC_Os03g13100.1	ariadne-1, putative, expressed	2,00	-1.0	1	24	1698	1721	CUGCGACAAGUAAUUCCGAACGGA	CGAACCAGGAAUUACUUGUCGCAG	Cleavage
tae-miR1131	Traes_4BL_178D6FFF1.1										1,05	-1.0	1	22	167	188	UAGUACCGGUUCGUGGCUAACC	GGUUAGCCACGAACCGAUACUA	Cleavage
tae-miR1131	Traes_4BL_178D6FFF1.2										1,05	-1.0	1	22	167	188	UAGUACCGGUUCGUGGCUAACC	GGUUAGCCACGAACCGAUACUA	Cleavage
tae-miR9662a-3p	Traes_4BL_29D8171E0.1								LOC_Os06g12100.1	mTERF family protein, expressed	2,00	-1.0	1	21	132	152	UUGAACAUCCCAGAGCCACCG	UCGUGGCGCUGGGAUGUUCAG	Cleavage
tae-miR9662b-3p	Traes_4BL_29D8171E0.1								LOC_Os06g12100.1	mTERF family protein, expressed	2,05	-1.0	1	21	131	151	UGAACAUCCCAGAGCCACCGG	CUCGUGGCGCUGGGAUGUUCA	Cleavage
tae-miR1122b-3p	Traes_4BL_2D34597CF.2	PF04068,PF04034					AT5G10070.2	RNase L inhibitor protein-related	LOC_Os03g09920.1	RNase L inhibitor protein-related, putative, expressed	2,05	-1.0	1	21	454	474	AGACUUAUAUGUAGGAACGGA	UCUGUUCCUAAAUAUAAGUCU	Translation
tae-miR1122b-3p	Traes_4BL_2D34597CF.3	PF04068,PF04034					AT5G10070.2	RNase L inhibitor protein-related	LOC_Os03g09920.1	RNase L inhibitor protein-related, putative, expressed	2,05	-1.0	1	21	454	474	AGACUUAUAUGUAGGAACGGA	UCUGUUCCUAAAUAUAAGUCU	Translation
tae-miR1128	Traes_4BL_37FDD905B.1	PF10250					AT1G04910.1	O-fucosyltransferase family protein	LOC_Os11g29120.2	growth regulator related protein, putative, expressed	2,05	-1.0	1	21	125	145	UACUACUCCCUCCGUCCGAAA	UUUGGGACGGAGGGAGUAGGA	Cleavage
tae-miR5384-3p	Traes_4BL_3B7A98FD6.1	PF03703					AT1G14345.1	NAD(P)-linked oxidoreductase superfamily protein	LOC_Os03g07440.1	putativ eribonuclease P, putative, expressed	2,05	-1.0	1	21	191	211	UGAGCGCGCCGCCGUCGAAUG	UCUUCGACGGCGGCGCGCACU	Cleavage
tae-miR9666a-3p	Traes_4BL_44010E331.1										3,00	-1.0	1	22	176	197	CGGUAGGGCUGUAUGAUGGCGA	UCGGCAUGAUACAGCCCUACCU	Cleavage
tae-miR1122b-3p	Traes_4BL_623559688.1	PF00013				GO:GO:0003723	AT5G15270.2	RNA-binding KH domain-containing protein	LOC_Os03g25970.1	KH domain-containing protein, putative, expressed	2,05	-1.0	1	21	706	726	AGACUUAUAUGUAGGAACGGA	UCUGUUCCUAAAUAUAAGUCU	Translation
tae-miR1122a	Traes_4BL_623559688.1	PF00013				GO:GO:0003723	AT5G15270.2	RNA-binding KH domain-containing protein	LOC_Os03g25970.1	KH domain-containing protein, putative, expressed	2,05	-1.0	1	20	747	766	UAGAUACAUCCGUAUCUAGA	ACUACAUACGGAUGUAUAUA	Cleavage
tae-miR1137a	Traes_4BL_D08D5FFB5.2	PF08282	KOG3189	5.4.2.8		GO:GO:0009298,GO:GO:0005737,GO:GO:0004615	AT2G45790.1	phosphomannomutase	LOC_Os04g58580.1	phosphomannomutase, putative, expressed	2,00	-1.0	1	20	722	741	UAGUACAAAGUUGAGUCAUC	AGUGUCUCAACUUUGUACUA	Cleavage
tae-miR1137a	Traes_4BL_F3D5D7034.2						AT3G16990.1	Haem oxygenase-like, multi-helical	LOC_Os03g19390.1	TENA/THI-4 family protein, putative, expressed	1,00	-1.0	1	19	1	19	UAGUACAAAGUUGAGUCAU	AUGACCCAACUUUGUACUA	Cleavage
tae-miR1137b-5p	Traes_4BL_F3D5D7034.2						AT3G16990.1	Haem oxygenase-like, multi-helical	LOC_Os03g19390.1	TENA/THI-4 family protein, putative, expressed	1,05	-1.0	1	21	37	57	UCCGUUCCAGAAUAGAUGACC	GGUCAUCUAUUUUGGAACGGA	Translation
tae-miR9662a-3p	Traes_4BS_257C5B099.1	PF02536				GO:GO:0006355,GO:GO:0005739,GO:GO:0003690	AT5G07900.1	Mitochondrial transcription termination factor family protein	LOC_Os06g12100.1	mTERF family protein, expressed	2,00	-1.0	1	21	66	86	UUGAACAUCCCAGAGCCACCG	CCGUGGUUCUGGGAUGUUCAG	Cleavage
tae-miR9662b-3p	Traes_4BS_257C5B099.1	PF02536				GO:GO:0006355,GO:GO:0005739,GO:GO:0003690	AT5G07900.1	Mitochondrial transcription termination factor family protein	LOC_Os06g12100.1	mTERF family protein, expressed	2,00	-1.0	1	21	65	85	UGAACAUCCCAGAGCCACCGG	CCCGUGGUUCUGGGAUGUUCA	Cleavage
tae-miR156	Traes_4BS_375EF1D15.1								LOC_Os01g69830.1	OsSPL2 - SBP-box gene family member, expressed	2,05	-1.0	1	21	235	255	UGACAGAAGAGAGUGAGCACA	UGUGCUCUCUCUUUUCUGUCA	Cleavage
tae-miR1118	Traes_4BS_4BC02509F.1						AT3G43790.3	zinc induced facilitator-like 2	LOC_Os12g03830.1	major facilitator superfamily antiporter, putative, expressed	2,05	-1.0	1	23	525	547	CACUACAUUAUGGAAUGGAGGGA	UCCCUCCGUUCCAUAAUGUAAUG	Cleavage
tae-miR9666a-3p	Traes_4BS_5419B1DE7.1										3,00	-1.0	1	22	176	197	CGGUAGGGCUGUAUGAUGGCGA	UCGGCAUGAUACAGCCCUACCU	Cleavage
tae-miR1122b-3p	Traes_4BS_6252B0BA0.1	PF05562					AT1G29395.1	COLD REGULATED 314 INNER MEMBRANE 1	LOC_Os05g49170.1	cold acclimation protein WCOR413, putative, expressed	1,05	-1.0	1	21	46	66	AGACUUAUAUGUAGGAACGGA	UCCGUUCCUAAAUAUAAGUCU	Translation
tae-miR7757-5p	Traes_4BS_B45729F4D.4	PF00931				GO:GO:0043531	AT3G07040.1	NB-ARC domain-containing disease resistance protein	LOC_Os11g11810.1	NBS-LRR disease resistance protein, putative, expressed	2,05	-1.0	1	22	653	674	AUAAAACCUUCAGCUAUCCAUC	AAUGGAUAUCUGAAGGUUUCAU	Cleavage
tae-miR5384-3p	Traes_4BS_F1D7488B8.1	PF02861	KOG1051		K03695	GO:GO:0019538	AT3G52490.1	Double Clp-N motif-containing P-loop nucleoside triphosphate hydrolases superfamily protein	LOC_Os11g05820.1	transposon protein, putative, Mutator sub-class, expressed	2,05	-1.0	1	21	269	289	UGAGCGCGCCGCCGUCGAAUG	UGUUCCACGGCGGCGCGCACA	Cleavage
tae-miR1122a	Traes_4BS_F395A220A.2	PF01595	KOG2118				AT2G14520.1	CBS domain-containing protein with a domain of unknown function (DUF21)	LOC_Os03g47120.1	CBS domain containing protein, expressed	0,00	-1.0	1	20	1336	1355	UAGAUACAUCCGUAUCUAGA	UCUAGAUACGGAUGUAUCUA	Cleavage
tae-miR7757-5p	Traes_4DL_113F6DD2B.1	PF00931				GO:GO:0043531	AT1G50180.1	NB-ARC domain-containing disease resistance protein	LOC_Os12g13550.1	NBS-LRR disease resistance protein, putative, expressed	2,05	-1.0	1	22	215	236	AUAAAACCUUCAGCUAUCCAUC	AAUGGAUGGCUGAAGGCUUUAU	Cleavage
tae-miR7757-5p	Traes_4DL_113F6DD2B.2	PF00931				GO:GO:0043531	AT1G50180.1	NB-ARC domain-containing disease resistance protein	LOC_Os10g22484.2	RGH2B, putative, expressed	2,05	-1.0	1	22	215	236	AUAAAACCUUCAGCUAUCCAUC	AAUGGAUGGCUGAAGGCUUUAU	Cleavage
tae-miR5085	Traes_4DL_341830EFF.1										2,05	-1.0	1	21	583	603	AAGGACAUUUUUUGUGGCCUG	GAGGACACAAAAAAUGUCGUU	Cleavage
tae-miR1133	Traes_4DL_529524815.5						AT1G67250.1	Proteasome maturation factor UMP1	LOC_Os02g55640.1	proteasome maturation factor UMP1 family protein, expressed	2,00	-1.0	1	22	103	127	CAUAUACUCCCUCCG-UCCGA--AA	UUAGUCGGAUCGGAGGGAGUAUAUG	Cleavage
tae-miR1130b-3p	Traes_4DL_84745919A.2			2.7.7.19			AT3G51620.2	PAP/OAS1 substrate-binding domain superfamily	LOC_Os03g21850.1	nucleotidyltransferase, putative, expressed	2,05	-1.0	1	21	2964	2984	UCUUAUAUUAUGGGACGGAGG	CCUCUGUCCCAUAAUAUAGGA	Cleavage
tae-miR1130a	Traes_4DL_84745919A.2			2.7.7.19			AT3G51620.2	PAP/OAS1 substrate-binding domain superfamily	LOC_Os03g21850.1	nucleotidyltransferase, putative, expressed	1,05	-1.0	1	23	3016	3038	CCUCCGUCUCGUAAUGUAAGACG	UGUCUUACAUUAUGAGACGGAGG	Translation
tae-miR1128	Traes_4DL_93D4DED90.3	PF00009		3.6.5.3	K03231		AT5G60390.3	GTP binding Elongation factor Tu family protein	LOC_Os03g08010.1	elongation factor Tu, putative, expressed	3,00	-1.0	1	21	565	586	UACUACUCCCUCCG-UCCGAAA	UUUUGGAACGGAGGGAGUAGUA	Cleavage
tae-miR1137a	Traes_4DL_93D4DED90.3	PF00009		3.6.5.3	K03231		AT5G60390.3	GTP binding Elongation factor Tu family protein	LOC_Os03g08010.1	elongation factor Tu, putative, expressed	2,05	-1.0	1	20	508	527	UAGUACAAAGUUGAGUCAUC	GAUGACCCAACUUUGUAUUA	Cleavage
tae-miR1137b-5p	Traes_4DL_93D4DED90.3	PF00009		3.6.5.3	K03231		AT5G60390.3	GTP binding Elongation factor Tu family protein	LOC_Os03g08010.1	elongation factor Tu, putative, expressed	1,05	-1.0	1	21	556	576	UCCGUUCCAGAAUAGAUGACC	GGUCAUCUAUUUUGGAACGGA	Translation
tae-miR1137b-5p	Traes_4DS_28E6C4F21.2										1,05	-1.0	1	21	61	81	UCCGUUCCAGAAUAGAUGACC	AGUCAUCUAUUUUGGAACGGA	Translation
tae-miR1137b-5p	Traes_4DS_28E6C4F21.3										1,05	-1.0	1	21	61	81	UCCGUUCCAGAAUAGAUGACC	AGUCAUCUAUUUUGGAACGGA	Translation
tae-miR1137b-5p	Traes_4DS_28E6C4F21.4										1,05	-1.0	1	21	61	81	UCCGUUCCAGAAUAGAUGACC	AGUCAUCUAUUUUGGAACGGA	Translation
tae-miR7757-5p	Traes_4DS_5B29EE3DD.5	PF00931				GO:GO:0043531	AT3G50950.1	HOPZ-ACTIVATED RESISTANCE 1	LOC_Os11g11810.1	NBS-LRR disease resistance protein, putative, expressed	2,05	-1.0	1	22	1379	1400	AUAAAACCUUCAGCUAUCCAUC	AAUGGAUAUCUGAAGGUUUCAU	Cleavage
tae-miR7757-5p	Traes_4DS_5B29EE3DD.6	PF00931				GO:GO:0043531	AT3G50950.1	HOPZ-ACTIVATED RESISTANCE 1	LOC_Os11g11810.1	NBS-LRR disease resistance protein, putative, expressed	2,05	-1.0	1	22	1379	1400	AUAAAACCUUCAGCUAUCCAUC	AAUGGAUAUCUGAAGGUUUCAU	Cleavage
tae-miR7757-5p	Traes_4DS_5B29EE3DD.7	PF00931				GO:GO:0043531	AT1G58390.1	Disease resistance protein (CC-NBS-LRR class) family	LOC_Os11g11810.1	NBS-LRR disease resistance protein, putative, expressed	2,05	-1.0	1	22	824	845	AUAAAACCUUCAGCUAUCCAUC	AAUGGAUAUCUGAAGGUUUCAU	Cleavage
tae-miR1130b-3p	Traes_4DS_9179FF158.4	PF06964		3.2.1.55		GO:GO:0046556,GO:GO:0046373	AT3G10740.1	alpha-L-arabinofuranosidase 1	LOC_Os12g03470.1	alpha-N-arabinofuranosidase A, putative, expressed	1,05	-1.0	1	21	41	61	UCUUAUAUUAUGGGACGGAGG	CCUCCGUCCCAUAAUGUAAGA	Cleavage
tae-miR9778	Traes_4DS_B08D83A2F.1	PF00931				GO:GO:0043531	AT3G46530.1	NB-ARC domain-containing disease resistance protein	LOC_Os11g39550.1	Leucine Rich Repeat family protein, expressed	2,05	-1.0	1	21	126	146	UGCAUCAUCUCGAACUCGUCG	UGGCGAGUUCCAGAUGAUGCA	Translation
tae-miR164	Traes_4DS_C08BF9DB2.1			2.4.1.14			AT1G04920.1	sucrose phosphate synthase 3F	LOC_Os11g12810.1	sucrose-phosphate synthase, putative, expressed	2,05	-1.0	1	21	387	407	UGGAGAAGCAGGGCACGUGCA	UUCACGUGCCCUGCUUUUCCU	Cleavage
tae-miR1128	Traes_4DS_C22A101FE.2						AT1G07910.1	RNAligase	LOC_Os07g09400.1	RNA ligase isoform 1, putative, expressed	2,05	-1.0	1	21	670	690	UACUACUCCCUCCGUCCGAAA	UUUGGGACGAAGGGAGUAGUA	Cleavage
tae-miR1128	Traes_4DS_C22A101FE.9						AT1G07910.1	RNAligase	LOC_Os07g09400.1	RNA ligase isoform 1, putative, expressed	2,05	-1.0	1	21	358	378	UACUACUCCCUCCGUCCGAAA	UUUGGGACGAAGGGAGUAGUA	Cleavage
tae-miR1122a	Traes_4DS_E8239194E.2	PF01394					AT3G08530.1	Clathrin, heavy chain	LOC_Os12g01390.1	clathrin heavy chain, putative, expressed	2,05	-1.0	1	20	692	711	UAGAUACAUCCGUAUCUAGA	UCUAAAUACGGAUGUAUCAA	Cleavage
tae-miR5384-3p	Traes_4DS_ECFDD82BA.1	PF02861	KOG1051		K03695	GO:GO:0019538	AT3G52490.1	Double Clp-N motif-containing P-loop nucleoside triphosphate hydrolases superfamily protein	LOC_Os11g05820.1	transposon protein, putative, Mutator sub-class, expressed	2,05	-1.0	1	21	269	289	UGAGCGCGCCGCCGUCGAAUG	UGUUCCACGGCGGCGCGCACA	Cleavage
tae-miR1137b-5p	Traes_4DS_EFE0451D7.3	PF00270		3.6.4.12		GO:GO:0005524,GO:GO:0003676	AT1G31360.1	RECQ helicase L2	LOC_Os11g48090.5	helicase conserved C-terminal domain containing protein, expressed	1,05	-1.0	1	21	950	970	UCCGUUCCAGAAUAGAUGACC	AGUCAUCUAUUUUGGAACGGA	Translation
tae-miR1128	Traes_5AL_3AB81DF9B.1	PF00056,PF02866	KOG1496	1.1.1.82		GO:GO:0055114,GO:GO:0016491,GO:GO:0016616,GO:GO:0016615,GO:GO:0006108	AT5G58330.1	lactate/malate dehydrogenase family protein	LOC_Os08g44810.1	lactate/malate dehydrogenase, putative, expressed	3,00	-1.0	1	21	151	172	UACUACUCCCUCCG-UCCGAAA	UUUAGGAACGGAGGGAGUAGUA	Cleavage
tae-miR9652-5p	Traes_5AL_481F73E59.1										2,05	-1.0	1	22	883	904	CCUGUUUGUCAUUAAGUUUCUU	AAGAAACUGAAUGACAAGCAGG	Cleavage
tae-miR9652-5p	Traes_5AL_481F73E59.2										2,05	-1.0	1	22	883	904	CCUGUUUGUCAUUAAGUUUCUU	AAGAAACUGAAUGACAAGCAGG	Cleavage
tae-miR9652-5p	Traes_5AL_481F73E59.3										2,05	-1.0	1	22	883	904	CCUGUUUGUCAUUAAGUUUCUU	AAGAAACUGAAUGACAAGCAGG	Cleavage
tae-miR9652-5p	Traes_5AL_481F73E59.4										2,05	-1.0	1	22	883	904	CCUGUUUGUCAUUAAGUUUCUU	AAGAAACUGAAUGACAAGCAGG	Cleavage
tae-miR9652-5p	Traes_5AL_481F73E59.5										2,05	-1.0	1	22	583	604	CCUGUUUGUCAUUAAGUUUCUU	AAGAAACUGAAUGACAAGCAGG	Cleavage
tae-miR9652-5p	Traes_5AL_481F73E59.6										2,05	-1.0	1	22	883	904	CCUGUUUGUCAUUAAGUUUCUU	AAGAAACUGAAUGACAAGCAGG	Cleavage
tae-miR5062-5p	Traes_5AL_500FF0B72.1	PF02171				GO:GO:0003676	AT2G27880.1	Argonaute family protein	LOC_Os03g58600.1	PAZ domain containing protein, putative, expressed	2,05	-1.0	1	23	43	65	UGAACCUUAGGGAACAGCCGCAU	ACCAGGCUAUUCCCUGAGGUUCA	Cleavage
tae-miR5062-5p	Traes_5AL_60D577E34.1	PF02171				GO:GO:0003676	AT2G27880.1	Argonaute family protein	LOC_Os03g58600.1	PAZ domain containing protein, putative, expressed	1,05	-1.0	1	23	43	65	UGAACCUUAGGGAACAGCCGCAU	ACCAGGCUGUUCCCUGAGGUUCA	Cleavage
tae-miR1127b-3p	Traes_5AL_7256A9F19.2	PF00400	KOG0279			GO:GO:0005515	AT3G18860.2	transducin family protein / WD-40 repeat family protein	LOC_Os07g03160.1	WD domain, G-beta repeat domain containing protein, expressed	1,05	-1.0	1	21	991	1011	ACAAGUAUUUCUGGACGGAGG	CCUCCGUCCGGAAAUACUUGU	Cleavage
tae-miR1122b-3p	Traes_5AL_7FF780810.1						AT5G41940.1	Ypt/Rab-GAP domain of gyp1p superfamily protein	LOC_Os09g35960.1	TBC domain containing protein, expressed	1,05	-1.0	1	21	22	42	AGACUUAUAUGUAGGAACGGA	UCCGUUCCUAAAUAUAAGUCU	Translation
tae-miR5086	Traes_5AL_8B87707C4.1										3,00	-1.0	1	21	350	370	ACAUUGGUGGAAGGCGUGGUA	AGUCAUGCCUUCCACCAAUGA	Cleavage
tae-miR5086	Traes_5AL_8B87707C4.2										3,00	-1.0	1	21	350	370	ACAUUGGUGGAAGGCGUGGUA	AGUCAUGCCUUCCACCAAUGA	Cleavage
tae-miR5086	Traes_5AL_8B87707C4.3										3,00	-1.0	1	21	326	346	ACAUUGGUGGAAGGCGUGGUA	AGUCAUGCCUUCCACCAAUGA	Cleavage
tae-miR7757-5p	Traes_5AL_B4E8A3115.1	PF00931				GO:GO:0043531	AT3G46710.1	NB-ARC domain-containing disease resistance protein	LOC_Os05g40150.1	RGH2B, putative, expressed	1,05	-1.0	1	22	368	389	AUAAAACCUUCAGCUAUCCAUC	AAUGGAUAGCUGAAGGUCUUAU	Cleavage
tae-miR7757-5p	Traes_5AL_B4E8A3115.2	PF03106,PF12796,PF00931	KOG4658			GO:GO:0043565,GO:GO:0006355,GO:GO:0003700,GO:GO:0043531	AT2G31820.1	Ankyrin repeat family protein	LOC_Os11g45750.2	WRKY125, expressed	1,05	-1.0	1	22	2324	2345	AUAAAACCUUCAGCUAUCCAUC	AAUGGAUAGCUGAAGGUCUUAU	Cleavage
tae-miR7757-5p	Traes_5AL_B4E8A3115.3	PF03106,PF12796,PF00931	KOG4658			GO:GO:0043565,GO:GO:0006355,GO:GO:0003700,GO:GO:0043531	AT2G31820.1	Ankyrin repeat family protein	LOC_Os11g45750.2	WRKY125, expressed	1,05	-1.0	1	22	2324	2345	AUAAAACCUUCAGCUAUCCAUC	AAUGGAUAGCUGAAGGUCUUAU	Cleavage
tae-miR7757-5p	Traes_5AL_B4E8A3115.4	PF03106,PF12796,PF00931	KOG4658			GO:GO:0043565,GO:GO:0006355,GO:GO:0003700,GO:GO:0043531	AT2G31820.1	Ankyrin repeat family protein	LOC_Os11g45750.2	WRKY125, expressed	1,05	-1.0	1	22	2324	2345	AUAAAACCUUCAGCUAUCCAUC	AAUGGAUAGCUGAAGGUCUUAU	Cleavage
tae-miR7757-5p	Traes_5AL_B4E8A3115.5	PF03106,PF12796,PF00931	KOG4658			GO:GO:0043565,GO:GO:0006355,GO:GO:0003700,GO:GO:0043531	AT2G31820.1	Ankyrin repeat family protein	LOC_Os11g45750.1	WRKY125, expressed	1,05	-1.0	1	22	2324	2345	AUAAAACCUUCAGCUAUCCAUC	AAUGGAUAGCUGAAGGUCUUAU	Cleavage
tae-miR7757-5p	Traes_5AL_B4E8A3115.6	PF00931,PF03106				GO:GO:0043531,GO:GO:0043565,GO:GO:0006355,GO:GO:0003700	AT3G46710.1	NB-ARC domain-containing disease resistance protein	LOC_Os05g40150.1	RGH2B, putative, expressed	1,05	-1.0	1	22	368	389	AUAAAACCUUCAGCUAUCCAUC	AAUGGAUAGCUGAAGGUCUUAU	Cleavage
tae-miR9652-5p	Traes_5AL_C9236F111.1										2,05	-1.0	1	22	778	799	CCUGUUUGUCAUUAAGUUUCUU	AAGAAACUGAAUGACAAGCAGG	Cleavage
tae-miR5384-3p	Traes_5AS_A928BA201.1	PF04535					AT5G44550.1	Uncharacterised protein family (UPF0497)	LOC_Os12g41690.1	membrane associated DUF588 domain containing protein, putative, expressed	2,05	-1.0	1	21	506	526	UGAGCGCGCCGCCGUCGAAUG	CAUUCGUCGGCGUCGCGCUCA	Cleavage
tae-miR7757-5p	Traes_5AS_BDFB08E23.1	PF00931	KOG4658			GO:GO:0043531	AT3G07040.1	NB-ARC domain-containing disease resistance protein	LOC_Os12g37740.1	resistance protein LR10, putative, expressed	2,05	-1.0	1	22	1349	1370	AUAAAACCUUCAGCUAUCCAUC	GAUGGAUUGCUGAAGGUUUCAU	Cleavage
tae-miR7757-5p	Traes_5AS_BDFB08E23.2	PF00931	KOG4658			GO:GO:0043531	AT3G07040.1	NB-ARC domain-containing disease resistance protein	LOC_Os12g37740.1	resistance protein LR10, putative, expressed	2,05	-1.0	1	22	1349	1370	AUAAAACCUUCAGCUAUCCAUC	GAUGGAUUGCUGAAGGUUUCAU	Cleavage
tae-miR7757-5p	Traes_5AS_BDFB08E23.3	PF00931				GO:GO:0043531	AT3G07040.1	NB-ARC domain-containing disease resistance protein	LOC_Os12g37740.1	resistance protein LR10, putative, expressed	2,05	-1.0	1	22	524	545	AUAAAACCUUCAGCUAUCCAUC	GAUGGAUUGCUGAAGGUUUCAU	Cleavage
tae-miR7757-5p	Traes_5AS_BDFB08E23.4	PF00931				GO:GO:0043531	AT3G46530.1	NB-ARC domain-containing disease resistance protein	LOC_Os12g37740.1	resistance protein LR10, putative, expressed	2,05	-1.0	1	22	1349	1370	AUAAAACCUUCAGCUAUCCAUC	GAUGGAUUGCUGAAGGUUUCAU	Cleavage
tae-miR7757-5p	Traes_5AS_BDFB08E23.5	PF00931				GO:GO:0043531	AT3G46530.1	NB-ARC domain-containing disease resistance protein	LOC_Os12g37740.1	resistance protein LR10, putative, expressed	2,05	-1.0	1	22	1349	1370	AUAAAACCUUCAGCUAUCCAUC	GAUGGAUUGCUGAAGGUUUCAU	Cleavage
tae-miR1128	Traes_5AS_E1F692526.2										3,00	-1.0	1	21	111	132	UACUACUCCCUCCGUCCGA-AA	UUAUGGGACGGAGGGAGUAGUA	Cleavage
tae-miR1130b-3p	Traes_5AS_E1F692526.2										3,00	-1.0	1	21	40	61	UCUU-AUAUUAUGGGACGGAGG	CCUCCGUCCCAUAAUAUUAAGA	Cleavage
tae-miR1122c-3p	Traes_5AS_E1F692526.2										3,00	-1.0	1	21	40	61	UC-UAAUAUUAUGGGACGGAGG	CCUCCGUCCCAUAAUAUUAAGA	Cleavage
tae-miR164	Traes_5AS_E5FD82E36.1	PF02365				GO:GO:0006355,GO:GO:0003677	AT1G56010.2	NAC domain containing protein 1	LOC_Os12g41680.1	No apical meristem protein, putative, expressed	1,00	-1.0	1	21	653	673	UGGAGAAGCAGGGCACGUGCA	AGCAAGUGCCCUGCUUCUCCA	Cleavage
tae-miR164	Traes_5AS_E5FD82E36.2	PF02365				GO:GO:0006355,GO:GO:0003677	AT1G56010.2	NAC domain containing protein 1	LOC_Os12g41680.1	No apical meristem protein, putative, expressed	1,00	-1.0	1	21	656	676	UGGAGAAGCAGGGCACGUGCA	AGCAAGUGCCCUGCUUCUCCA	Cleavage
tae-miR1118	Traes_5AS_EC41EF7CA.4	PF00013	KOG2190			GO:GO:0003723	AT3G04610.1	RNA-binding KH domain-containing protein	LOC_Os12g40560.1	KH domain containing protein, putative, expressed	1,05	-1.0	1	23	1333	1355	CACUACAUUAUGGAAUGGAGGGA	UCCCUCCAUUCCACAAUGUAGUG	Translation
tae-miR1125	Traes_5AS_EC41EF7CA.4	PF00013	KOG2190			GO:GO:0003723	AT3G04610.1	RNA-binding KH domain-containing protein	LOC_Os12g40560.1	KH domain containing protein, putative, expressed	1,00	-1.0	1	24	1450	1473	AACCAACGAGACCAACUGCGGCGG	CCGCCGCAGUCGGUCUCGUUGGUU	Cleavage
tae-miR408	Traes_5BL_0212ACBD9.1	PF05911	KOG0999				AT1G77580.2	Plant protein of unknown function (DUF869)	LOC_Os12g07160.2	plant protein of unknown function DUF869 domain containing protein, expressed	2,05	-1.0	1	21	2512	2532	CUGCACUGCCUCUUCCCUGGC	GCCAAGGAAGAGGCUGUGCAG	Cleavage
tae-miR1137a	Traes_5BL_0EC4788EF.2	PF08244,PF00251	KOG0228	3.2.1.26			AT1G55120.1	beta-fructofuranosidase 5	LOC_Os01g73580.1	glycosyl hydrolases, putative, expressed	1,00	-1.0	1	20	1543	1562	UAGUACAAAGUUGAGUCAUC	GAUGACCCAACUUUGUACUA	Cleavage
tae-miR9652-5p	Traes_5BL_0F64FDA44.1										2,05	-1.0	1	22	583	604	CCUGUUUGUCAUUAAGUUUCUU	AAGAAACUGAAUGACAAGCAGG	Cleavage
tae-miR1122a	Traes_5BL_1B9186ED8.1	PF07762							LOC_Os11g07360.1	expressed protein	2,05	-1.0	1	20	986	1005	UAGAUACAUCCGUAUCUAGA	UCGAGAUACAGAUGUAUCUA	Translation
tae-miR7757-5p	Traes_5BL_1E751EF1F.1	PF00931,PF03514	KOG4658			GO:GO:0043531	AT1G50600.1	scarecrow-like 5	LOC_Os10g22484.1	RGH2B, putative, expressed	2,00	-1.0	1	22	1799	1820	AUAAAACCUUCAGCUAUCCAUC	AAUGGGUUGCUGAAGGUUUUAU	Cleavage
tae-miR7757-5p	Traes_5BL_1E751EF1F.2	PF00931				GO:GO:0043531	AT3G46730.1	NB-ARC domain-containing disease resistance protein	LOC_Os10g22484.1	RGH2B, putative, expressed	2,00	-1.0	1	22	1799	1820	AUAAAACCUUCAGCUAUCCAUC	AAUGGGUUGCUGAAGGUUUUAU	Cleavage
tae-miR7757-5p	Traes_5BL_1E751EF1F.5	PF00931,PF03514				GO:GO:0043531	AT1G50600.1	scarecrow-like 5	LOC_Os10g22484.1	RGH2B, putative, expressed	2,00	-1.0	1	22	512	533	AUAAAACCUUCAGCUAUCCAUC	AAUGGGUUGCUGAAGGUUUUAU	Cleavage
tae-miR5049-3p	Traes_5BL_4BFE76791.5								LOC_Os08g04590.1	csAtPR5, putative, expressed	2,05	-1.0	1	21	416	436	AAUAUGGAUCGGAGGGAGUAC	AUGCUCCCUCUGAUCCAUAUU	Translation
tae-miR1127b-3p	Traes_5BL_5E94C243F.1						AT3G54630.1		LOC_Os08g36490.1	kinetochore protein, putative, expressed	2,05	-1.0	1	21	914	934	ACAAGUAUUUCUGGACGGAGG	CCUCUGUCCUGAAAUACUUGU	Cleavage
tae-miR1127b-3p	Traes_5BL_5E94C243F.2						AT3G54630.1		LOC_Os08g36490.1	kinetochore protein, putative, expressed	2,05	-1.0	1	21	898	918	ACAAGUAUUUCUGGACGGAGG	CCUCUGUCCUGAAAUACUUGU	Cleavage
tae-miR9674b-5p	Traes_5BL_6216A6108.1	PF13041					AT3G22470.1	Pentatricopeptide repeat (PPR) superfamily protein	LOC_Os10g35240.2	Rf1, mitochondrial precursor, putative, expressed	3,00	-1.0	1	21	411	431	AUAGCAUCAUCCAUCCUACCC	GGGCCGAAUGGAUGAUGCUAU	Cleavage
tae-miR5384-3p	Traes_5BL_6240DE6BA.1	PF00067		1.14.13.89		GO:GO:0055114,GO:GO:0020037,GO:GO:0016705,GO:GO:0005506	AT5G67310.1	cytochrome P450, family 81, subfamily G, polypeptide 1	LOC_Os03g55240.1	cytochrome P450, putative, expressed	2,05	-1.0	1	21	338	358	UGAGCGCGCCGCCGUCGAAUG	CCUUCGACGGCGCCGCGCUCG	Cleavage
tae-miR5384-3p	Traes_5BL_6240DE6BA.2	PF00067		1.14.13.89		GO:GO:0055114,GO:GO:0020037,GO:GO:0016705,GO:GO:0005506	AT5G67310.1	cytochrome P450, family 81, subfamily G, polypeptide 1	LOC_Os03g55240.1	cytochrome P450, putative, expressed	2,05	-1.0	1	21	338	358	UGAGCGCGCCGCCGUCGAAUG	CCUUCGACGGCGCCGCGCUCG	Cleavage
tae-miR5384-3p	Traes_5BL_6240DE6BA.3	PF00067		1.14.13.89		GO:GO:0055114,GO:GO:0020037,GO:GO:0016705,GO:GO:0005506	AT5G67310.1	cytochrome P450, family 81, subfamily G, polypeptide 1	LOC_Os03g55240.1	cytochrome P450, putative, expressed	2,05	-1.0	1	21	338	358	UGAGCGCGCCGCCGUCGAAUG	CCUUCGACGGCGCCGCGCUCG	Cleavage
tae-miR5384-3p	Traes_5BL_6240DE6BA.4	PF00067		1.14.13.89		GO:GO:0055114,GO:GO:0020037,GO:GO:0016705,GO:GO:0005506	AT5G67310.1	cytochrome P450, family 81, subfamily G, polypeptide 1	LOC_Os03g55240.1	cytochrome P450, putative, expressed	2,05	-1.0	1	21	338	358	UGAGCGCGCCGCCGUCGAAUG	CCUUCGACGGCGCCGCGCUCG	Cleavage
tae-miR1122a	Traes_5BL_637A3C970.8	PF01501		2.4.1.43		GO:GO:0016757,GO:GO:0047262	AT5G47780.1	galacturonosyltransferase 4	LOC_Os09g30280.1	glycosyl transferase 8 domain containing protein, putative, expressed	1,05	-1.0	1	20	49	68	UAGAUACAUCCGUAUCUAGA	UCUAGAUACGAAUGUAUCUA	Translation
tae-miR1121	Traes_5BL_72EFF0AF0.1	PF07690				GO:GO:0055085,GO:GO:0016021	AT5G59250.1	Major facilitator superfamily protein	LOC_Os10g42830.1	transporter family protein, putative, expressed	0,00	-1.0	1	22	1237	1258	AGUAGUGAUCUAAACGCUCUUA	UAAGAGCGUUUAGAUCACUACU	Cleavage
tae-miR408	Traes_5BL_7486582EB.1	PF02298				GO:GO:0009055	AT2G02850.1	plantacyanin	LOC_Os03g50160.1	plastocyanin-like domain containing protein, putative, expressed	2,05	-1.0	1	21	8	28	CUGCACUGCCUCUUCCCUGGC	CGCAGGGAAGAGGCAGUGCCA	Cleavage
tae-miR1122a	Traes_5BL_7821274AB.1	PF07762							LOC_Os11g07360.1	expressed protein	2,05	-1.0	1	20	986	1005	UAGAUACAUCCGUAUCUAGA	UCGAGAUACAGAUGUAUCUA	Translation
tae-miR7757-5p	Traes_5BL_7F0FD1538.2	PF00847,PF00931	KOG4658			GO:GO:0006355,GO:GO:0003700,GO:GO:0043531	AT3G46710.1	NB-ARC domain-containing disease resistance protein	LOC_Os05g40150.1	RGH2B, putative, expressed	1,05	-1.0	1	22	2096	2117	AUAAAACCUUCAGCUAUCCAUC	AAUGGAUAGCUGAAAGUUUUAU	Cleavage
tae-miR7757-5p	Traes_5BL_7F0FD1538.3	PF00847,PF00931	KOG4658			GO:GO:0006355,GO:GO:0003700,GO:GO:0043531	AT3G46710.1	NB-ARC domain-containing disease resistance protein	LOC_Os05g40150.1	RGH2B, putative, expressed	1,05	-1.0	1	22	1379	1400	AUAAAACCUUCAGCUAUCCAUC	AAUGGAUAGCUGAAAGUUUUAU	Cleavage
tae-miR7757-5p	Traes_5BL_7F0FD1538.4	PF00931	KOG4658			GO:GO:0043531	AT3G46710.1	NB-ARC domain-containing disease resistance protein	LOC_Os05g40150.1	RGH2B, putative, expressed	1,05	-1.0	1	22	1379	1400	AUAAAACCUUCAGCUAUCCAUC	AAUGGAUAGCUGAAAGUUUUAU	Cleavage
tae-miR5086	Traes_5BL_83D9CB7C3.1										3,00	-1.0	1	21	497	517	ACAUUGGUGGAAGGCGUGGUA	AGUCAUGCCUUCCACCAAUGA	Cleavage
tae-miR5086	Traes_5BL_83D9CB7C3.2										3,00	-1.0	1	21	608	628	ACAUUGGUGGAAGGCGUGGUA	AGUCAUGCCUUCCACCAAUGA	Cleavage
tae-miR5086	Traes_5BL_83D9CB7C3.3										3,00	-1.0	1	21	473	493	ACAUUGGUGGAAGGCGUGGUA	AGUCAUGCCUUCCACCAAUGA	Cleavage
tae-miR5086	Traes_5BL_83D9CB7C3.4										3,00	-1.0	1	21	608	628	ACAUUGGUGGAAGGCGUGGUA	AGUCAUGCCUUCCACCAAUGA	Cleavage
tae-miR5086	Traes_5BL_83D9CB7C3.5										3,00	-1.0	1	21	497	517	ACAUUGGUGGAAGGCGUGGUA	AGUCAUGCCUUCCACCAAUGA	Cleavage
tae-miR156	Traes_5BL_96D5EA4EE.1	PF03110				GO:GO:0005634,GO:GO:0003677	AT2G42200.1	squamosa promoter binding protein-like 9	LOC_Os09g31438.1	OsSPL17 - SBP-box gene family member, expressed	1,00	-1.0	1	21	805	825	UGACAGAAGAGAGUGAGCACA	UGUGCUCUCUCUCUUCUGUCA	Cleavage
tae-miR156	Traes_5BL_96D5EA4EE.2	PF03110				GO:GO:0005634,GO:GO:0003677	AT2G42200.1	squamosa promoter binding protein-like 9	LOC_Os09g31438.1	OsSPL17 - SBP-box gene family member, expressed	1,00	-1.0	1	21	802	822	UGACAGAAGAGAGUGAGCACA	UGUGCUCUCUCUCUUCUGUCA	Cleavage
tae-miR156	Traes_5BL_96D5EA4EE.3								LOC_Os09g31438.1	OsSPL17 - SBP-box gene family member, expressed	1,00	-1.0	1	21	67	87	UGACAGAAGAGAGUGAGCACA	UGUGCUCUCUCUCUUCUGUCA	Cleavage
tae-miR156	Traes_5BL_96FDFC152.1	PF03110				GO:GO:0005634,GO:GO:0003677	AT1G76580.1	Squamosa promoter-binding protein-like (SBP domain) transcription factor family protein	LOC_Os09g32944.1	OsSPL18 - SBP-box gene family member, expressed	1,00	-1.0	1	21	508	528	UGACAGAAGAGAGUGAGCACA	UGUGCUCUCUCUCUUCUGUCA	Cleavage
tae-miR156	Traes_5BL_96FDFC152.2	PF03110				GO:GO:0005634,GO:GO:0003677	AT5G50570.1	Squamosa promoter-binding protein-like (SBP domain) transcription factor family protein	LOC_Os09g32944.1	OsSPL18 - SBP-box gene family member, expressed	1,00	-1.0	1	21	490	510	UGACAGAAGAGAGUGAGCACA	UGUGCUCUCUCUCUUCUGUCA	Cleavage
tae-miR5384-3p	Traes_5BL_AD734CCEF.1	PF12695	KOG1515	3.1.1.1			AT3G48690.1	alpha/beta-Hydrolases superfamily protein	LOC_Os09g28730.1	gibberellin receptor GID1L2, putative, expressed	2,05	-1.0	1	21	242	262	UGAGCGCGCCGCCGUCGAAUG	UCUUCCACGGCGGCGCGCUCA	Cleavage
tae-miR9664-3p	Traes_5BL_D27214492.1	PF00931				GO:GO:0043531	AT3G46530.1	NB-ARC domain-containing disease resistance protein	LOC_Os11g11550.1	NBS-LRR disease resistance protein, putative, expressed	2,05	-1.0	1	21	237	257	UUGCAGUCCUCGAUGUCGUAG	GCACGACAUCGAGGAUUGCAU	Cleavage
tae-miR9664-3p	Traes_5BL_D27214492.2	PF00931				GO:GO:0043531	AT3G46530.1	NB-ARC domain-containing disease resistance protein	LOC_Os11g11550.2	NBS-LRR disease resistance protein, putative, expressed	2,05	-1.0	1	21	237	257	UUGCAGUCCUCGAUGUCGUAG	GCACGACAUCGAGGAUUGCAU	Cleavage
tae-miR9664-3p	Traes_5BL_D27214492.3	PF00931				GO:GO:0043531	AT3G46530.1	NB-ARC domain-containing disease resistance protein	LOC_Os11g11550.2	NBS-LRR disease resistance protein, putative, expressed	2,05	-1.0	1	21	237	257	UUGCAGUCCUCGAUGUCGUAG	GCACGACAUCGAGGAUUGCAU	Cleavage
tae-miR9664-3p	Traes_5BL_D27214492.4	PF00931				GO:GO:0043531	AT3G46530.1	NB-ARC domain-containing disease resistance protein	LOC_Os11g11550.2	NBS-LRR disease resistance protein, putative, expressed	2,05	-1.0	1	21	237	257	UUGCAGUCCUCGAUGUCGUAG	GCACGACAUCGAGGAUUGCAU	Cleavage
tae-miR9664-3p	Traes_5BL_D27214492.5	PF00931				GO:GO:0043531	AT3G46530.1	NB-ARC domain-containing disease resistance protein	LOC_Os11g11550.1	NBS-LRR disease resistance protein, putative, expressed	2,05	-1.0	1	21	237	257	UUGCAGUCCUCGAUGUCGUAG	GCACGACAUCGAGGAUUGCAU	Cleavage
tae-miR7757-5p	Traes_5BL_DD4C9F446.1	PF00069	KOG1187	2.7.11.1		GO:GO:0006468,GO:GO:0005524,GO:GO:0004672	AT3G55950.1	CRINKLY4 related 3	LOC_Os05g40150.1	RGH2B, putative, expressed	1,05	-1.0	1	22	62	83	AUAAAACCUUCAGCUAUCCAUC	AAUGGAUAGCGGAAGGUUUUAU	Cleavage
tae-miR7757-5p	Traes_5BL_DD4C9F446.2	PF00069	KOG1187	2.7.11.1		GO:GO:0006468,GO:GO:0005524,GO:GO:0004672	AT3G55950.1	CRINKLY4 related 3	LOC_Os05g40150.1	RGH2B, putative, expressed	1,05	-1.0	1	22	62	83	AUAAAACCUUCAGCUAUCCAUC	AAUGGAUAGCGGAAGGUUUUAU	Cleavage
tae-miR7757-5p	Traes_5BL_DD4C9F446.4						AT3G46710.1	NB-ARC domain-containing disease resistance protein	LOC_Os05g40150.1	RGH2B, putative, expressed	1,05	-1.0	1	22	62	83	AUAAAACCUUCAGCUAUCCAUC	AAUGGAUAGCGGAAGGUUUUAU	Cleavage
tae-miR7757-5p	Traes_5BL_DD4C9F446.5						AT3G46710.1	NB-ARC domain-containing disease resistance protein	LOC_Os05g40150.1	RGH2B, putative, expressed	1,05	-1.0	1	22	62	83	AUAAAACCUUCAGCUAUCCAUC	AAUGGAUAGCGGAAGGUUUUAU	Cleavage
tae-miR7757-5p	Traes_5BL_DD4C9F446.6						AT3G46710.1	NB-ARC domain-containing disease resistance protein	LOC_Os05g40150.1	RGH2B, putative, expressed	1,05	-1.0	1	22	62	83	AUAAAACCUUCAGCUAUCCAUC	AAUGGAUAGCGGAAGGUUUUAU	Cleavage
tae-miR9652-5p	Traes_5BL_FC199BC2D.1										2,05	-1.0	1	22	412	433	CCUGUUUGUCAUUAAGUUUCUU	AAGAAACUGAAUGACAAGCAGG	Cleavage
tae-miR1122b-3p	Traes_5BS_321AF7B69.2	PF14416,PF13839					AT5G20680.3	TRICHOME BIREFRINGENCE-LIKE 16	LOC_Os12g33194.1	expressed protein	2,05	-1.0	1	21	23	43	AGACUUAUAUGUAGGAACGGA	UCCAUUCCUAAAUAUAAGUCU	Translation
tae-miR1122a	Traes_5BS_321AF7B69.2	PF14416,PF13839					AT5G20680.3	TRICHOME BIREFRINGENCE-LIKE 16	LOC_Os12g33194.1	expressed protein	2,05	-1.0	1	20	63	82	UAGAUACAUCCGUAUCUAGA	ACUACAUACGGAUGUAUAUA	Cleavage
tae-miR7757-5p	Traes_5BS_3FFCC89AD.1	PF00931	KOG4658			GO:GO:0043531	AT3G07040.1	NB-ARC domain-containing disease resistance protein	LOC_Os12g37770.1	RGH1A, putative, expressed	2,05	-1.0	1	22	1337	1358	AUAAAACCUUCAGCUAUCCAUC	GGUGGAUUGCUGAAGGAUUUAU	Cleavage
tae-miR7757-5p	Traes_5BS_3FFCC89AD.2	PF00931	KOG4658			GO:GO:0043531	AT3G46730.1	NB-ARC domain-containing disease resistance protein	LOC_Os12g37770.1	RGH1A, putative, expressed	2,05	-1.0	1	22	1298	1319	AUAAAACCUUCAGCUAUCCAUC	GGUGGAUUGCUGAAGGAUUUAU	Cleavage
tae-miR1118	Traes_5BS_550BDEE86.2	PF00013	KOG2190			GO:GO:0003723	AT3G04610.1	RNA-binding KH domain-containing protein	LOC_Os12g40560.1	KH domain containing protein, putative, expressed	1,05	-1.0	1	23	1135	1157	CACUACAUUAUGGAAUGGAGGGA	UCCCUCCAUUCCACAAUGUAGUG	Translation
tae-miR5384-3p	Traes_5BS_90796C6CA.1	PF07762							LOC_Os01g56900.1	expressed protein	2,00	-1.0	1	21	461	481	UGAGCGCGCCGCCGUCGAAUG	UGUACGAUGGCGGCGCGCUCA	Cleavage
tae-miR5384-3p	Traes_5BS_90796C6CA.2	PF07762							LOC_Os01g56900.1	expressed protein	2,00	-1.0	1	21	34	54	UGAGCGCGCCGCCGUCGAAUG	UGUACGAUGGCGGCGCGCUCA	Cleavage
tae-miR5384-3p	Traes_5BS_90796C6CA.3	PF07762							LOC_Os01g56900.1	expressed protein	2,00	-1.0	1	21	392	412	UGAGCGCGCCGCCGUCGAAUG	UGUACGAUGGCGGCGCGCUCA	Cleavage
tae-miR164	Traes_5BS_9F043AE17.1	PF02365				GO:GO:0006355,GO:GO:0003677	AT1G56010.2	NAC domain containing protein 1	LOC_Os12g41680.1	No apical meristem protein, putative, expressed	1,00	-1.0	1	21	656	676	UGGAGAAGCAGGGCACGUGCA	AGCAAGUGCCCUGCUUCUCCA	Cleavage
tae-miR5384-3p	Traes_5BS_BA0400E10.1	PF14570,PF03552		2.4.2.24	K00770	GO:GO:0030244,GO:GO:0016760,GO:GO:0016020	AT1G02730.1	cellulose synthase-like D5	LOC_Os12g36890.1	CSLD4 - cellulose synthase-like family D, expressed	3,00	-1.0	1	21	1358	1378	UGAGCGCGCCGCCGUCGAAUG	CCGACGACGGCGGCGCGCUCC	Cleavage
tae-miR7757-5p	Traes_5BS_BCEA0112C.1	PF00931	KOG4658			GO:GO:0043531	AT3G07040.1	NB-ARC domain-containing disease resistance protein	LOC_Os12g37740.1	resistance protein LR10, putative, expressed	2,05	-1.0	1	22	1340	1361	AUAAAACCUUCAGCUAUCCAUC	GAUGGAUCGCGGAAGGUUUUAU	Cleavage
tae-miR7757-5p	Traes_5BS_BCEA0112C.2	PF00931	KOG4658			GO:GO:0043531	AT3G07040.1	NB-ARC domain-containing disease resistance protein	LOC_Os12g37740.1	resistance protein LR10, putative, expressed	2,05	-1.0	1	22	1340	1361	AUAAAACCUUCAGCUAUCCAUC	GAUGGAUCGCGGAAGGUUUUAU	Cleavage
tae-miR7757-5p	Traes_5BS_BCEA0112C.3	PF00931	KOG4658			GO:GO:0043531	AT3G07040.1	NB-ARC domain-containing disease resistance protein	LOC_Os12g37740.1	resistance protein LR10, putative, expressed	2,05	-1.0	1	22	1307	1328	AUAAAACCUUCAGCUAUCCAUC	GAUGGAUCGCGGAAGGUUUUAU	Cleavage
tae-miR7757-5p	Traes_5BS_BCEA0112C.4	PF00931				GO:GO:0043531	AT3G07040.1	NB-ARC domain-containing disease resistance protein	LOC_Os12g37740.1	resistance protein LR10, putative, expressed	2,05	-1.0	1	22	128	149	AUAAAACCUUCAGCUAUCCAUC	GAUGGAUCGCGGAAGGUUUUAU	Cleavage
tae-miR408	Traes_5BS_E10756355.1	PF05911	KOG0999				AT1G77580.2	Plant protein of unknown function (DUF869)	LOC_Os12g07160.2	plant protein of unknown function DUF869 domain containing protein, expressed	2,05	-1.0	1	21	2512	2532	CUGCACUGCCUCUUCCCUGGC	GCCAAGGAAGAGGCUGUGCAG	Cleavage
tae-miR7757-5p	Traes_5BS_F5AFB24CD.2	PF00931				GO:GO:0043531	AT3G46530.1	NB-ARC domain-containing disease resistance protein	LOC_Os10g03570.1	RGH1A, putative, expressed	1,05	-1.0	1	22	779	800	AUAAAACCUUCAGCUAUCCAUC	GGUGGAUAGCUGAAGGUCUUAU	Cleavage
tae-miR7757-5p	Traes_5BS_F5AFB24CD.3	PF00931				GO:GO:0043531	AT3G46530.1	NB-ARC domain-containing disease resistance protein	LOC_Os10g03570.1	RGH1A, putative, expressed	1,05	-1.0	1	22	779	800	AUAAAACCUUCAGCUAUCCAUC	GGUGGAUAGCUGAAGGUCUUAU	Cleavage
tae-miR7757-5p	Traes_5BS_F5AFB24CD.4	PF00931				GO:GO:0043531	AT5G43470.2	Disease resistance protein (CC-NBS-LRR class) family	LOC_Os10g03570.1	RGH1A, putative, expressed	1,05	-1.0	1	22	371	392	AUAAAACCUUCAGCUAUCCAUC	GGUGGAUAGCUGAAGGUCUUAU	Cleavage
tae-miR156	Traes_5DL_1272B4BAC.1	PF03110				GO:GO:0005634,GO:GO:0003677	AT5G50670.1	Squamosa promoter-binding protein-like (SBP domain) transcription factor family protein	LOC_Os09g32944.1	OsSPL18 - SBP-box gene family member, expressed	1,00	-1.0	1	21	505	525	UGACAGAAGAGAGUGAGCACA	UGUGCUCUCUCUCUUCUGUCA	Cleavage
tae-miR156	Traes_5DL_1272B4BAC.2	PF03110				GO:GO:0005634,GO:GO:0003677	AT5G50670.1	Squamosa promoter-binding protein-like (SBP domain) transcription factor family protein	LOC_Os09g32944.1	OsSPL18 - SBP-box gene family member, expressed	1,00	-1.0	1	21	487	507	UGACAGAAGAGAGUGAGCACA	UGUGCUCUCUCUCUUCUGUCA	Cleavage
tae-miR156	Traes_5DL_1272B4BAC.3								LOC_Os09g32944.1	OsSPL18 - SBP-box gene family member, expressed	1,00	-1.0	1	21	382	402	UGACAGAAGAGAGUGAGCACA	UGUGCUCUCUCUCUUCUGUCA	Cleavage
tae-miR7757-5p	Traes_5DL_1733FB4DA.1	PF00931,PF03106				GO:GO:0043531,GO:GO:0043565,GO:GO:0006355,GO:GO:0003700	AT3G46530.1	NB-ARC domain-containing disease resistance protein	LOC_Os05g40150.1	RGH2B, putative, expressed	1,05	-1.0	1	22	617	638	AUAAAACCUUCAGCUAUCCAUC	AAUGGAUAGCUGAAGGAUUUAU	Cleavage
tae-miR7757-5p	Traes_5DL_1733FB4DA.2	PF03106,PF00931				GO:GO:0043565,GO:GO:0006355,GO:GO:0003700,GO:GO:0043531	AT3G46530.1	NB-ARC domain-containing disease resistance protein	LOC_Os05g40150.1	RGH2B, putative, expressed	1,05	-1.0	1	22	617	638	AUAAAACCUUCAGCUAUCCAUC	AAUGGAUAGCUGAAGGAUUUAU	Cleavage
tae-miR7757-5p	Traes_5DL_1733FB4DA.3	PF03106,PF00931				GO:GO:0043565,GO:GO:0006355,GO:GO:0003700,GO:GO:0043531	AT3G46530.1	NB-ARC domain-containing disease resistance protein	LOC_Os05g40150.1	RGH2B, putative, expressed	1,05	-1.0	1	22	617	638	AUAAAACCUUCAGCUAUCCAUC	AAUGGAUAGCUGAAGGAUUUAU	Cleavage
tae-miR7757-5p	Traes_5DL_1733FB4DA.4	PF00931				GO:GO:0043531	AT3G46530.1	NB-ARC domain-containing disease resistance protein	LOC_Os05g40150.1	RGH2B, putative, expressed	1,05	-1.0	1	22	617	638	AUAAAACCUUCAGCUAUCCAUC	AAUGGAUAGCUGAAGGAUUUAU	Cleavage
tae-miR7757-5p	Traes_5DL_1733FB4DA.5	PF00931				GO:GO:0043531	AT3G46530.1	NB-ARC domain-containing disease resistance protein	LOC_Os05g40150.1	RGH2B, putative, expressed	1,05	-1.0	1	22	617	638	AUAAAACCUUCAGCUAUCCAUC	AAUGGAUAGCUGAAGGAUUUAU	Cleavage
tae-miR7757-5p	Traes_5DL_1733FB4DA.6	PF00931				GO:GO:0043531	AT3G46530.1	NB-ARC domain-containing disease resistance protein	LOC_Os05g40150.1	RGH2B, putative, expressed	1,05	-1.0	1	22	617	638	AUAAAACCUUCAGCUAUCCAUC	AAUGGAUAGCUGAAGGAUUUAU	Cleavage
tae-miR7757-5p	Traes_5DL_1733FB4DA.7	PF00931				GO:GO:0043531	AT3G46530.1	NB-ARC domain-containing disease resistance protein	LOC_Os11g45750.1	WRKY125, expressed	1,05	-1.0	1	22	617	638	AUAAAACCUUCAGCUAUCCAUC	AAUGGAUAGCUGAAGGAUUUAU	Cleavage
tae-miR7757-5p	Traes_5DL_1733FB4DA.8	PF00931				GO:GO:0043531	AT3G46530.1	NB-ARC domain-containing disease resistance protein	LOC_Os11g45750.2	WRKY125, expressed	1,05	-1.0	1	22	617	638	AUAAAACCUUCAGCUAUCCAUC	AAUGGAUAGCUGAAGGAUUUAU	Cleavage
tae-miR5062-5p	Traes_5DL_26B51AAF8.1	PF02171				GO:GO:0003676	AT1G48410.2	Stabilizer of iron transporter SufD / Polynucleotidyl transferase	LOC_Os03g58600.1	PAZ domain containing protein, putative, expressed	1,05	-1.0	1	23	43	65	UGAACCUUAGGGAACAGCCGCAU	ACCAGGCUGUUCCCUGAGGUUCA	Cleavage
tae-miR1127b-3p	Traes_5DL_4E67E0243.9	PF03828		2.7.7.19			AT3G51620.2	PAP/OAS1 substrate-binding domain superfamily	LOC_Os12g02260.1	nucleotidyltransferase, putative, expressed	1,05	-1.0	1	21	41	61	ACAAGUAUUUCUGGACGGAGG	CCUCCGUCCGGAAAUACUUGU	Cleavage
tae-miR9652-5p	Traes_5DL_714CCB5BC.1										2,05	-1.0	1	22	412	433	CCUGUUUGUCAUUAAGUUUCUU	AAGAAACUGAAUGACAAGCAGG	Cleavage

tae-miR9662a-3p	Traes_5DL_7643B2ADB.1	PF02536				GO:GO:0006355,GO:GO:0005739,GO:GO:0003690	AT5G64950.1	Mitochondrial transcription termination factor family protein	LOC_Os06g12100.1	mTERF family protein, expressed	2,05	-1.0	1	21	411	431	UUGAACAUCCCAGAGCCACCG	CCGUGGCUCUGUGAUGUUCAG	Translation
tae-miR9662b-3p	Traes_5DL_7643B2ADB.1	PF02536				GO:GO:0006355,GO:GO:0005739,GO:GO:0003690	AT5G64950.1	Mitochondrial transcription termination factor family protein	LOC_Os06g12100.1	mTERF family protein, expressed	2,05	-1.0	1	21	410	430	UGAACAUCCCAGAGCCACCGG	CCCGUGGCUCUGUGAUGUUCA	Cleavage
tae-miR5384-3p	Traes_5DL_78767F75C.1	PF00135	KOG1515	3.1.1.1			AT3G48690.1	alpha/beta-Hydrolases superfamily protein	LOC_Os09g28730.1	gibberellin receptor GID1L2, putative, expressed	2,05	-1.0	1	21	242	262	UGAGCGCGCCGCCGUCGAAUG	UCUUCCACGGCGGCGCGUUCA	Cleavage
tae-miR5049-3p	Traes_5DL_799647EA7.4										1,05	-1.0	1	21	65	85	AAUAUGGAUCGGAGGGAGUAC	GUACUCCCUCCGUUCCAUAUU	Cleavage
tae-miR9652-5p	Traes_5DL_8DE6C67FA.1										2,05	-1.0	1	22	412	433	CCUGUUUGUCAUUAAGUUUCUU	AAGAAACUGAAUGACAAGCAGG	Cleavage
tae-miR9652-5p	Traes_5DL_8DE6C67FA.2										2,05	-1.0	1	22	412	433	CCUGUUUGUCAUUAAGUUUCUU	AAGAAACUGAAUGACAAGCAGG	Cleavage
tae-miR9652-5p	Traes_5DL_8DE6C67FA.3										2,05	-1.0	1	22	415	436	CCUGUUUGUCAUUAAGUUUCUU	AAGAAACUGAAUGACAAGCAGG	Cleavage
tae-miR9652-5p	Traes_5DL_8DE6C67FA.4										2,05	-1.0	1	22	400	421	CCUGUUUGUCAUUAAGUUUCUU	AAGAAACUGAAUGACAAGCAGG	Cleavage
tae-miR9652-5p	Traes_5DL_8DE6C67FA.5										2,05	-1.0	1	22	400	421	CCUGUUUGUCAUUAAGUUUCUU	AAGAAACUGAAUGACAAGCAGG	Cleavage
tae-miR408	Traes_5DL_90BD9F306.1	PF02298				GO:GO:0009055	AT2G02850.1	plantacyanin	LOC_Os03g50160.1	plastocyanin-like domain containing protein, putative, expressed	0,00	-1.0	1	21	8	28	CUGCACUGCCUCUUCCCUGGC	CCCAGGGAAGAGGCAGUGCAG	Cleavage
tae-miR9666b-3p	Traes_5DL_9101D5AEC.1	PF05001		2.7.7.6		GO:GO:0006366,GO:GO:0005665,GO:GO:0003677	AT4G35800.1	RNA polymerase II large subunit	LOC_Os05g05860.1	retrotransposon protein, putative, unclassified, expressed	2,05	-1.0	1	22	1183	1204	CGGUUGGGCUGUAUGAUGGCGA	AGUCCAUCAUACAGCCCAACAA	Cleavage
tae-miR1130b-3p	Traes_5DL_A6EECBC04.1								LOC_Os03g32160.1	calmodulin binding protein, putative, expressed	1,05	-1.0	1	21	376	396	UCUUAUAUUAUGGGACGGAGG	AUUCCGUCCCAUAAUGUAAGA	Cleavage
tae-miR9652-5p	Traes_5DL_B3713A4D1.1										2,05	-1.0	1	22	940	961	CCUGUUUGUCAUUAAGUUUCUU	AAGAAACUGAAUGACAAGCAGG	Cleavage
tae-miR5086	Traes_5DL_CECECF832.1										3,00	-1.0	1	21	461	481	ACAUUGGUGGAAGGCGUGGUA	AGUCAUGCCUUCCACCAAUGA	Cleavage
tae-miR5086	Traes_5DL_CECECF832.2										3,00	-1.0	1	21	485	505	ACAUUGGUGGAAGGCGUGGUA	AGUCAUGCCUUCCACCAAUGA	Cleavage
tae-miR5086	Traes_5DL_CECECF832.3										3,00	-1.0	1	21	101	121	ACAUUGGUGGAAGGCGUGGUA	AGUCAUGCCUUCCACCAAUGA	Cleavage
tae-miR5086	Traes_5DL_CECECF832.4										3,00	-1.0	1	21	485	505	ACAUUGGUGGAAGGCGUGGUA	AGUCAUGCCUUCCACCAAUGA	Cleavage
tae-miR5086	Traes_5DL_CECECF832.5										3,00	-1.0	1	21	101	121	ACAUUGGUGGAAGGCGUGGUA	AGUCAUGCCUUCCACCAAUGA	Cleavage
tae-miR5062-5p	Traes_5DL_F5D3E4763.1	PF02171				GO:GO:0003676	AT1G48410.3	Stabilizer of iron transporter SufD / Polynucleotidyl transferase	LOC_Os03g58600.1	PAZ domain containing protein, putative, expressed	2,05	-1.0	1	23	43	65	UGAACCUUAGGGAACAGCCGCAU	ACCAGGUUGUUCCCUGAGGUUCA	Cleavage
tae-miR7757-5p	Traes_5DS_0479C6E0F.1	PF00931				GO:GO:0043531	AT3G07040.1	NB-ARC domain-containing disease resistance protein	LOC_Os12g06920.1	NBS-LRR disease resistance protein, putative, expressed	2,05	-1.0	1	22	383	404	AUAAAACCUUCAGCUAUCCAUC	GAUGGAUCGCUGAAGGUUUGAU	Cleavage
tae-miR9662a-3p	Traes_5DS_094A03C13.1	PF02536				GO:GO:0006355,GO:GO:0005739,GO:GO:0003690	AT5G07900.1	Mitochondrial transcription termination factor family protein	LOC_Os06g12100.1	mTERF family protein, expressed	1,00	-1.0	1	21	549	569	UUGAACAUCCCAGAGCCACCG	CCGUGGCUCUGGGAUGUUCAG	Cleavage
tae-miR9662b-3p	Traes_5DS_094A03C13.1	PF02536				GO:GO:0006355,GO:GO:0005739,GO:GO:0003690	AT5G07900.1	Mitochondrial transcription termination factor family protein	LOC_Os06g12100.1	mTERF family protein, expressed	1,00	-1.0	1	21	548	568	UGAACAUCCCAGAGCCACCGG	CCCGUGGCUCUGGGAUGUUCA	Cleavage
tae-miR9662a-3p	Traes_5DS_094A03C13.2	PF02536				GO:GO:0006355,GO:GO:0005739,GO:GO:0003690	AT5G07900.1	Mitochondrial transcription termination factor family protein	LOC_Os06g12100.1	mTERF family protein, expressed	1,00	-1.0	1	21	243	263	UUGAACAUCCCAGAGCCACCG	CCGUGGCUCUGGGAUGUUCAG	Cleavage
tae-miR9662b-3p	Traes_5DS_094A03C13.2	PF02536				GO:GO:0006355,GO:GO:0005739,GO:GO:0003690	AT5G07900.1	Mitochondrial transcription termination factor family protein	LOC_Os06g12100.1	mTERF family protein, expressed	1,00	-1.0	1	21	242	262	UGAACAUCCCAGAGCCACCGG	CCCGUGGCUCUGGGAUGUUCA	Cleavage
tae-miR5384-3p	Traes_5DS_149714893.1	PF04535					AT5G44550.1	Uncharacterised protein family (UPF0497)	LOC_Os12g41690.1	membrane associated DUF588 domain containing protein, putative, expressed	2,05	-1.0	1	21	158	178	UGAGCGCGCCGCCGUCGAAUG	CGUUCGUCGGCGUCGCGCUCA	Cleavage
tae-miR5384-3p	Traes_5DS_149714893.2	PF04535					AT5G44550.1	Uncharacterised protein family (UPF0497)	LOC_Os12g41690.1	membrane associated DUF588 domain containing protein, putative, expressed	2,05	-1.0	1	21	152	172	UGAGCGCGCCGCCGUCGAAUG	CGUUCGUCGGCGUCGCGCUCA	Cleavage
tae-miR164	Traes_5DS_511F35A45.1						AT1G56010.2	NAC domain containing protein 1	LOC_Os12g41680.1	No apical meristem protein, putative, expressed	1,00	-1.0	1	21	209	229	UGGAGAAGCAGGGCACGUGCA	AGCAAGUGCCCUGCUUCUCCA	Cleavage
tae-miR7757-5p	Traes_5DS_8DB03B7DA.1	PF00931	KOG4658			GO:GO:0043531	AT3G07040.1	NB-ARC domain-containing disease resistance protein	LOC_Os12g37740.1	resistance protein LR10, putative, expressed	2,05	-1.0	1	22	1349	1370	AUAAAACCUUCAGCUAUCCAUC	GAUGGAUCGCGGAAGGUUUUAU	Cleavage
tae-miR7757-5p	Traes_5DS_8DB03B7DA.2	PF00931				GO:GO:0043531	AT3G07040.1	NB-ARC domain-containing disease resistance protein	LOC_Os12g37740.1	resistance protein LR10, putative, expressed	2,05	-1.0	1	22	128	149	AUAAAACCUUCAGCUAUCCAUC	GAUGGAUCGCGGAAGGUUUUAU	Cleavage
tae-miR7757-5p	Traes_5DS_9DFE4D217.1	PF00931				GO:GO:0043531	AT1G53350.1	Disease resistance protein (CC-NBS-LRR class) family	LOC_Os10g22484.1	RGH2B, putative, expressed	2,05	-1.0	1	22	185	206	AUAAAACCUUCAGCUAUCCAUC	AAUGGGUAGCUGAAGGUUGUAU	Cleavage
tae-miR7757-5p	Traes_5DS_9DFE4D217.2	PF00931				GO:GO:0043531	AT1G53350.1	Disease resistance protein (CC-NBS-LRR class) family	LOC_Os10g22484.1	RGH2B, putative, expressed	2,05	-1.0	1	22	185	206	AUAAAACCUUCAGCUAUCCAUC	AAUGGGUAGCUGAAGGUUGUAU	Cleavage
tae-miR7757-5p	Traes_5DS_9DFE4D217.3	PF00931				GO:GO:0043531	AT1G53350.1	Disease resistance protein (CC-NBS-LRR class) family	LOC_Os10g22484.1	RGH2B, putative, expressed	2,05	-1.0	1	22	185	206	AUAAAACCUUCAGCUAUCCAUC	AAUGGGUAGCUGAAGGUUGUAU	Cleavage
tae-miR9662a-3p	Traes_5DS_9F4A5AE9F.1	PF02536				GO:GO:0006355,GO:GO:0005739,GO:GO:0003690	AT5G07900.1	Mitochondrial transcription termination factor family protein	LOC_Os06g12100.1	mTERF family protein, expressed	2,00	-1.0	1	21	441	461	UUGAACAUCCCAGAGCCACCG	UCGUGGCGCUGGGAUGUUCAG	Cleavage
tae-miR9662b-3p	Traes_5DS_9F4A5AE9F.1	PF02536				GO:GO:0006355,GO:GO:0005739,GO:GO:0003690	AT5G07900.1	Mitochondrial transcription termination factor family protein	LOC_Os06g12100.1	mTERF family protein, expressed	2,05	-1.0	1	21	440	460	UGAACAUCCCAGAGCCACCGG	CUCGUGGCGCUGGGAUGUUCA	Cleavage
tae-miR9662a-3p	Traes_5DS_9F4A5AE9F.2	PF02536				GO:GO:0006355,GO:GO:0005739,GO:GO:0003690	AT5G07900.1	Mitochondrial transcription termination factor family protein	LOC_Os06g12100.1	mTERF family protein, expressed	2,00	-1.0	1	21	66	86	UUGAACAUCCCAGAGCCACCG	UCGUGGCGCUGGGAUGUUCAG	Cleavage
tae-miR9662b-3p	Traes_5DS_9F4A5AE9F.2	PF02536				GO:GO:0006355,GO:GO:0005739,GO:GO:0003690	AT5G07900.1	Mitochondrial transcription termination factor family protein	LOC_Os06g12100.1	mTERF family protein, expressed	2,05	-1.0	1	21	65	85	UGAACAUCCCAGAGCCACCGG	CUCGUGGCGCUGGGAUGUUCA	Cleavage
tae-miR1122b-3p	Traes_5DS_9F8FFD461.1	PF00792,PF00613		2.7.1.137		GO:GO:0048015,GO:GO:0046854	AT1G60490.1	vacuolar protein sorting 34	LOC_Os05g08810.2	phosphatidylinositol 3-kinase, root isoform, putative, expressed	1,05	-1.0	1	21	1777	1797	AGACUUAUAUGUAGGAACGGA	UCCGUUCCUAAAUAUAAGUCU	Translation
tae-miR1122b-3p	Traes_5DS_9F8FFD461.2	PF00792,PF00613		2.7.1.137		GO:GO:0048015,GO:GO:0046854	AT1G60490.1	vacuolar protein sorting 34	LOC_Os05g08810.2	phosphatidylinositol 3-kinase, root isoform, putative, expressed	1,05	-1.0	1	21	1882	1902	AGACUUAUAUGUAGGAACGGA	UCCGUUCCUAAAUAUAAGUCU	Translation
tae-miR1122b-3p	Traes_5DS_9F8FFD461.3	PF00792,PF00613		2.7.1.137		GO:GO:0048015,GO:GO:0046854	AT1G60490.1	vacuolar protein sorting 34	LOC_Os05g08810.2	phosphatidylinositol 3-kinase, root isoform, putative, expressed	1,05	-1.0	1	21	1801	1821	AGACUUAUAUGUAGGAACGGA	UCCGUUCCUAAAUAUAAGUCU	Translation
tae-miR1122b-3p	Traes_5DS_A6AE32DCD.1	PF03483	KOG2472	6.1.1.20		GO:GO:0004826,GO:GO:0003723	AT1G72550.1	tRNA synthetase beta subunit family protein	LOC_Os05g48510.1	phenylalanyl-tRNA synthetase beta chain, putative, expressed	1,05	-1.0	1	21	1065	1085	AGACUUAUAUGUAGGAACGGA	UCCGUUCCUAAAUAUAAGUCU	Translation
tae-miR1122b-3p	Traes_5DS_A6AE32DCD.2	PF03484,PF03483		6.1.1.20		GO:GO:0006432,GO:GO:0005524,GO:GO:0003723,GO:GO:0000287,GO:GO:0004826	AT1G72550.2	tRNA synthetase beta subunit family protein	LOC_Os05g48510.1	phenylalanyl-tRNA synthetase beta chain, putative, expressed	1,05	-1.0	1	21	1146	1166	AGACUUAUAUGUAGGAACGGA	UCCGUUCCUAAAUAUAAGUCU	Translation
tae-miR1122b-3p	Traes_5DS_A6AE32DCD.3	PF03484,PF03483		6.1.1.20		GO:GO:0006432,GO:GO:0005524,GO:GO:0003723,GO:GO:0000287,GO:GO:0004826	AT1G72550.2	tRNA synthetase beta subunit family protein	LOC_Os05g48510.1	phenylalanyl-tRNA synthetase beta chain, putative, expressed	1,05	-1.0	1	21	1146	1166	AGACUUAUAUGUAGGAACGGA	UCCGUUCCUAAAUAUAAGUCU	Translation
tae-miR9662a-3p	Traes_5DS_C87840EBA.2						AT1G79220.1	Mitochondrial transcription termination factor family protein	LOC_Os06g12100.1	mTERF family protein, expressed	2,00	-1.0	1	21	429	449	UUGAACAUCCCAGAGCCACCG	CCGUGGUUCUGGGAUGUUCAG	Cleavage
tae-miR9662b-3p	Traes_5DS_C87840EBA.2						AT1G79220.1	Mitochondrial transcription termination factor family protein	LOC_Os06g12100.1	mTERF family protein, expressed	2,00	-1.0	1	21	428	448	UGAACAUCCCAGAGCCACCGG	CCCGUGGUUCUGGGAUGUUCA	Cleavage
tae-miR1128	Traes_5DS_D165422B3.1	PF00503	KOG0082			GO:GO:0031683,GO:GO:0019001,GO:GO:0007186,GO:GO:0004871,GO:GO:0003924	AT2G23460.1	extra-large G-protein 1	LOC_Os12g40190.1	G-protein alpha subunit, putative, expressed	3,00	-1.0	1	21	59	80	UACUACUCCCUCCG-UCCGAAA	UUUAGGAACGGAGGGAGUAGUA	Cleavage
tae-miR1118	Traes_5DS_F8D323D4D.2	PF00013	KOG2190			GO:GO:0003723	AT3G04610.1	RNA-binding KH domain-containing protein	LOC_Os12g40560.1	KH domain containing protein, putative, expressed	1,05	-1.0	1	23	1329	1351	CACUACAUUAUGGAAUGGAGGGA	CUCCUCCAUUCCACAAUGUAGUG	Translation
tae-miR160	Traes_6AL_232AF0B76.1						AT4G30080.1	auxin response factor 16	LOC_Os02g41800.1	auxin response factor, putative, expressed	0,00	-1.0	1	21	195	215	UGCCUGGCUCCCUGUAUGCCA	AGGCAUACAGGGAGCCAGGCA	Cleavage
tae-miR1121	Traes_6AL_23B31796F.2	PF00230	KOG0223			GO:GO:0016020,GO:GO:0006810,GO:GO:0005215	AT3G53420.1	plasma membrane intrinsic protein 2A	LOC_Os02g41860.3	aquaporin protein, putative, expressed	2,00	-1.0	1	22	846	867	AGUAGUGAUCUAAACGCUCUUA	UAAAAGUGUUUAGAUCACUACU	Cleavage
tae-miR1121	Traes_6AL_23B31796F.3	PF00230	KOG0223			GO:GO:0016020,GO:GO:0006810,GO:GO:0005215	AT3G53420.1	plasma membrane intrinsic protein 2A	LOC_Os02g41860.3	aquaporin protein, putative, expressed	2,00	-1.0	1	22	760	781	AGUAGUGAUCUAAACGCUCUUA	UAAAAGUGUUUAGAUCACUACU	Cleavage
tae-miR1121	Traes_6AL_23B31796F.5	PF00230	KOG0223			GO:GO:0016020,GO:GO:0006810,GO:GO:0005215	AT3G53420.1	plasma membrane intrinsic protein 2A	LOC_Os02g41860.3	aquaporin protein, putative, expressed	2,00	-1.0	1	22	742	763	AGUAGUGAUCUAAACGCUCUUA	UAAAAGUGUUUAGAUCACUACU	Cleavage
tae-miR5049-3p	Traes_6AL_36AE1B4CF.4	PF03031		3.1.3.16			AT4G21670.1	C-terminal domain phosphatase-like 1	LOC_Os02g42600.1	double-stranded RNA binding motif containing protein, expressed	1,05	-1.0	1	21	1787	1807	AAUAUGGAUCGGAGGGAGUAC	AUACUCCCUCCGACCCAUAUU	Cleavage
tae-miR5049-3p	Traes_6AL_36AE1B4CF1.4	PF03031		3.1.3.16			AT4G21670.1	C-terminal domain phosphatase-like 1	LOC_Os02g42600.1	double-stranded RNA binding motif containing protein, expressed	1,05	-1.0	1	21	1787	1807	AAUAUGGAUCGGAGGGAGUAC	AUACUCCCUCCGACCCAUAUU	Cleavage
tae-miR5049-3p	Traes_6AL_6AD463C67.3			3.6.4.12			AT2G01440.1	DEAD/DEAH box RNA helicase family protein			2,05	-1.0	1	21	15	35	AAUAUGGAUCGGAGGGAGUAC	GUACUCCUUCCGAUCCAUAAU	Cleavage
tae-miR1121	Traes_6AL_7E70268F1.3	PF00719		3.6.1.1		GO:GO:0006796,GO:GO:0005737,GO:GO:0004427,GO:GO:0000287	AT2G46860.1	pyrophosphorylase 3	LOC_Os02g47600.1	soluble inorganic pyrophosphatase, putative, expressed	0,00	-1.0	1	22	497	518	AGUAGUGAUCUAAACGCUCUUA	UAAGAGCGUUUAGAUCACUACU	Cleavage
tae-miR1122b-3p	Traes_6AL_94CA6F0D8.5	PF00067				GO:GO:0055114,GO:GO:0020037,GO:GO:0016705,GO:GO:0005506	AT1G31800.1	cytochrome P450, family 97, subfamily A, polypeptide 3	LOC_Os02g57290.2	cytochrome P450, putative, expressed	2,05	-1.0	1	21	1030	1050	AGACUUAUAUGUAGGAACGGA	UCCAUUCCUAAAUAUAAGUCU	Translation
tae-miR1122a	Traes_6AL_94CA6F0D8.5	PF00067				GO:GO:0055114,GO:GO:0020037,GO:GO:0016705,GO:GO:0005506	AT1G31800.1	cytochrome P450, family 97, subfamily A, polypeptide 3	LOC_Os02g57290.2	cytochrome P450, putative, expressed	2,05	-1.0	1	20	1071	1090	UAGAUACAUCCGUAUCUAGA	ACUACAUACGGAUGUAUAUA	Cleavage
tae-miR9662a-3p	Traes_6AS_0A204AEC9.1	PF02536	KOG1267		K15032	GO:GO:0006355,GO:GO:0005739,GO:GO:0003690	AT5G07900.1	Mitochondrial transcription termination factor family protein	LOC_Os06g12100.1	mTERF family protein, expressed	2,00	-1.0	1	21	681	701	UUGAACAUCCCAGAGCCACCG	CCGUGCCUCUGGGAUGUUCAG	Cleavage
tae-miR9662b-3p	Traes_6AS_0A204AEC9.1	PF02536	KOG1267		K15032	GO:GO:0006355,GO:GO:0005739,GO:GO:0003690	AT5G07900.1	Mitochondrial transcription termination factor family protein	LOC_Os06g12100.1	mTERF family protein, expressed	2,00	-1.0	1	21	680	700	UGAACAUCCCAGAGCCACCGG	CCCGUGCCUCUGGGAUGUUCA	Cleavage
tae-miR9662a-3p	Traes_6AS_0A204AEC9.2	PF02536	KOG1267		K15032	GO:GO:0006355,GO:GO:0005739,GO:GO:0003690	AT5G07900.1	Mitochondrial transcription termination factor family protein	LOC_Os06g12100.1	mTERF family protein, expressed	2,00	-1.0	1	21	681	701	UUGAACAUCCCAGAGCCACCG	CCGUGCCUCUGGGAUGUUCAG	Cleavage
tae-miR9662b-3p	Traes_6AS_0A204AEC9.2	PF02536	KOG1267		K15032	GO:GO:0006355,GO:GO:0005739,GO:GO:0003690	AT5G07900.1	Mitochondrial transcription termination factor family protein	LOC_Os06g12100.1	mTERF family protein, expressed	2,00	-1.0	1	21	680	700	UGAACAUCCCAGAGCCACCGG	CCCGUGCCUCUGGGAUGUUCA	Cleavage
tae-miR9662a-3p	Traes_6AS_1496A7825.1	PF02536				GO:GO:0006355,GO:GO:0005739,GO:GO:0003690	AT5G07900.1	Mitochondrial transcription termination factor family protein	LOC_Os06g12100.1	mTERF family protein, expressed	1,05	-1.0	1	21	549	569	UUGAACAUCCCAGAGCCACCG	CCGUGGCUCGGGGAUGUUCAA	Cleavage
tae-miR9662b-3p	Traes_6AS_1496A7825.1	PF02536				GO:GO:0006355,GO:GO:0005739,GO:GO:0003690	AT5G07900.1	Mitochondrial transcription termination factor family protein	LOC_Os06g12100.1	mTERF family protein, expressed	2,05	-1.0	1	21	548	568	UGAACAUCCCAGAGCCACCGG	CCCGUGGCUCGGGGAUGUUCA	Translation
tae-miR9662a-3p	Traes_6AS_1496A7825.2	PF02536				GO:GO:0006355,GO:GO:0005739,GO:GO:0003690	AT5G07900.1	Mitochondrial transcription termination factor family protein	LOC_Os06g12100.1	mTERF family protein, expressed	1,05	-1.0	1	21	132	152	UUGAACAUCCCAGAGCCACCG	CCGUGGCUCGGGGAUGUUCAA	Cleavage
tae-miR9662b-3p	Traes_6AS_1496A7825.2	PF02536				GO:GO:0006355,GO:GO:0005739,GO:GO:0003690	AT5G07900.1	Mitochondrial transcription termination factor family protein	LOC_Os06g12100.1	mTERF family protein, expressed	2,05	-1.0	1	21	131	151	UGAACAUCCCAGAGCCACCGG	CCCGUGGCUCGGGGAUGUUCA	Translation
tae-miR9662a-3p	Traes_6AS_15C1E6F18.1	PF02536				GO:GO:0006355,GO:GO:0005739,GO:GO:0003690	AT1G21150.1	Mitochondrial transcription termination factor family protein	LOC_Os06g12100.1	mTERF family protein, expressed	2,05	-1.0	1	21	33	53	UUGAACAUCCCAGAGCCACCG	CUGUGGCUCUGGAAUGUUCAG	Cleavage
tae-miR9662b-3p	Traes_6AS_15C1E6F18.1	PF02536				GO:GO:0006355,GO:GO:0005739,GO:GO:0003690	AT1G21150.1	Mitochondrial transcription termination factor family protein	LOC_Os06g12100.1	mTERF family protein, expressed	2,05	-1.0	1	21	32	52	UGAACAUCCCAGAGCCACCGG	CCUGUGGCUCUGGAAUGUUCA	Cleavage
tae-miR9662a-3p	Traes_6AS_15C1E6F18.2	PF02536				GO:GO:0006355,GO:GO:0005739,GO:GO:0003690	AT1G21150.1	Mitochondrial transcription termination factor family protein	LOC_Os06g12100.1	mTERF family protein, expressed	2,05	-1.0	1	21	33	53	UUGAACAUCCCAGAGCCACCG	CUGUGGCUCUGGAAUGUUCAG	Cleavage
tae-miR9662b-3p	Traes_6AS_15C1E6F18.2	PF02536				GO:GO:0006355,GO:GO:0005739,GO:GO:0003690	AT1G21150.1	Mitochondrial transcription termination factor family protein	LOC_Os06g12100.1	mTERF family protein, expressed	2,05	-1.0	1	21	32	52	UGAACAUCCCAGAGCCACCGG	CCUGUGGCUCUGGAAUGUUCA	Cleavage
tae-miR9662a-3p	Traes_6AS_2BDC1663A.1	PF02536	KOG1267		K15032	GO:GO:0006355,GO:GO:0005739,GO:GO:0003690	AT5G07900.1	Mitochondrial transcription termination factor family protein	LOC_Os06g12100.1	mTERF family protein, expressed	1,00	-1.0	1	21	681	701	UUGAACAUCCCAGAGCCACCG	CCGUGGCUCUGGGAUGUUCAG	Cleavage
tae-miR9662b-3p	Traes_6AS_2BDC1663A.1	PF02536	KOG1267		K15032	GO:GO:0006355,GO:GO:0005739,GO:GO:0003690	AT5G07900.1	Mitochondrial transcription termination factor family protein	LOC_Os06g12100.1	mTERF family protein, expressed	1,00	-1.0	1	21	680	700	UGAACAUCCCAGAGCCACCGG	CCCGUGGCUCUGGGAUGUUCA	Cleavage
tae-miR156	Traes_6AS_32A35158A.1	PF03110				GO:GO:0005634,GO:GO:0003677	AT5G43270.2	squamosa promoter binding protein-like 2	LOC_Os06g45310.1	OsSPL11 - SBP-box gene family member, expressed	1,00	-1.0	1	21	736	756	UGACAGAAGAGAGUGAGCACA	CGUGCUCUCUCUCUUCUGUCA	Cleavage
tae-miR156	Traes_6AS_32A35158A.2								LOC_Os06g45310.1	OsSPL11 - SBP-box gene family member, expressed	1,00	-1.0	1	21	292	312	UGACAGAAGAGAGUGAGCACA	CGUGCUCUCUCUCUUCUGUCA	Cleavage
tae-miR156	Traes_6AS_32A35158A.3								LOC_Os06g45310.1	OsSPL11 - SBP-box gene family member, expressed	1,00	-1.0	1	21	64	84	UGACAGAAGAGAGUGAGCACA	CGUGCUCUCUCUCUUCUGUCA	Cleavage
tae-miR9662a-3p	Traes_6AS_3641B9C871.1	PF02536	KOG1267		K15032	GO:GO:0006355,GO:GO:0005739,GO:GO:0003690	AT5G07900.1	Mitochondrial transcription termination factor family protein	LOC_Os06g12100.1	mTERF family protein, expressed	1,00	-1.0	1	21	687	707	UUGAACAUCCCAGAGCCACCG	CCGUGGCUCUGGGAUGUUCAG	Cleavage
tae-miR9662b-3p	Traes_6AS_3641B9C871.1	PF02536	KOG1267		K15032	GO:GO:0006355,GO:GO:0005739,GO:GO:0003690	AT5G07900.1	Mitochondrial transcription termination factor family protein	LOC_Os06g12100.1	mTERF family protein, expressed	1,00	-1.0	1	21	686	706	UGAACAUCCCAGAGCCACCGG	CCCGUGGCUCUGGGAUGUUCA	Cleavage
tae-miR9662a-3p	Traes_6AS_3641B9C871.2	PF02536	KOG1267		K15032	GO:GO:0006355,GO:GO:0005739,GO:GO:0003690	AT5G07900.1	Mitochondrial transcription termination factor family protein	LOC_Os06g12100.1	mTERF family protein, expressed	1,00	-1.0	1	21	687	707	UUGAACAUCCCAGAGCCACCG	CCGUGGCUCUGGGAUGUUCAG	Cleavage
tae-miR9662b-3p	Traes_6AS_3641B9C871.2	PF02536	KOG1267		K15032	GO:GO:0006355,GO:GO:0005739,GO:GO:0003690	AT5G07900.1	Mitochondrial transcription termination factor family protein	LOC_Os06g12100.1	mTERF family protein, expressed	1,00	-1.0	1	21	686	706	UGAACAUCCCAGAGCCACCGG	CCCGUGGCUCUGGGAUGUUCA	Cleavage
tae-miR9662a-3p	Traes_6AS_36EAEADDD.1	PF02536				GO:GO:0006355,GO:GO:0005739,GO:GO:0003690	AT1G21150.1	Mitochondrial transcription termination factor family protein	LOC_Os06g12100.1	mTERF family protein, expressed	2,00	-1.0	1	21	33	53	UUGAACAUCCCAGAGCCACCG	CUGUGGUUCUGGGAUGUUCAG	Cleavage
tae-miR9662b-3p	Traes_6AS_36EAEADDD.1	PF02536				GO:GO:0006355,GO:GO:0005739,GO:GO:0003690	AT1G21150.1	Mitochondrial transcription termination factor family protein	LOC_Os06g12100.1	mTERF family protein, expressed	2,00	-1.0	1	21	32	52	UGAACAUCCCAGAGCCACCGG	CCUGUGGUUCUGGGAUGUUCA	Cleavage
tae-miR9662a-3p	Traes_6AS_36EAEADDD.2								LOC_Os06g12100.1	mTERF family protein, expressed	2,00	-1.0	1	21	33	53	UUGAACAUCCCAGAGCCACCG	CUGUGGUUCUGGGAUGUUCAG	Cleavage
tae-miR9662b-3p	Traes_6AS_36EAEADDD.2								LOC_Os06g12100.1	mTERF family protein, expressed	2,00	-1.0	1	21	32	52	UGAACAUCCCAGAGCCACCGG	CCUGUGGUUCUGGGAUGUUCA	Cleavage
tae-miR9662a-3p	Traes_6AS_36EAEADDD.3	PF02536				GO:GO:0006355,GO:GO:0005739,GO:GO:0003690	AT1G21150.1	Mitochondrial transcription termination factor family protein	LOC_Os06g12100.1	mTERF family protein, expressed	2,00	-1.0	1	21	33	53	UUGAACAUCCCAGAGCCACCG	CUGUGGUUCUGGGAUGUUCAG	Cleavage
tae-miR9662b-3p	Traes_6AS_36EAEADDD.3	PF02536				GO:GO:0006355,GO:GO:0005739,GO:GO:0003690	AT1G21150.1	Mitochondrial transcription termination factor family protein	LOC_Os06g12100.1	mTERF family protein, expressed	2,00	-1.0	1	21	32	52	UGAACAUCCCAGAGCCACCGG	CCUGUGGUUCUGGGAUGUUCA	Cleavage
tae-miR9662a-3p	Traes_6AS_48B701BB41.1	PF02536				GO:GO:0006355,GO:GO:0005739,GO:GO:0003690	AT1G21150.1	Mitochondrial transcription termination factor family protein	LOC_Os06g12100.1	mTERF family protein, expressed	1,00	-1.0	1	21	33	53	UUGAACAUCCCAGAGCCACCG	ACGUGGCUCUGGGAUGUUCAG	Cleavage
tae-miR9662b-3p	Traes_6AS_48B701BB41.1	PF02536				GO:GO:0006355,GO:GO:0005739,GO:GO:0003690	AT1G21150.1	Mitochondrial transcription termination factor family protein	LOC_Os06g12100.1	mTERF family protein, expressed	1,00	-1.0	1	21	32	52	UGAACAUCCCAGAGCCACCGG	CACGUGGCUCUGGGAUGUUCA	Cleavage
tae-miR9662a-3p	Traes_6AS_48B701BB41.2	PF02536				GO:GO:0006355,GO:GO:0005739,GO:GO:0003690	AT1G21150.1	Mitochondrial transcription termination factor family protein	LOC_Os06g12100.1	mTERF family protein, expressed	1,00	-1.0	1	21	33	53	UUGAACAUCCCAGAGCCACCG	ACGUGGCUCUGGGAUGUUCAG	Cleavage
tae-miR9662b-3p	Traes_6AS_48B701BB41.2	PF02536				GO:GO:0006355,GO:GO:0005739,GO:GO:0003690	AT1G21150.1	Mitochondrial transcription termination factor family protein	LOC_Os06g12100.1	mTERF family protein, expressed	1,00	-1.0	1	21	32	52	UGAACAUCCCAGAGCCACCGG	CACGUGGCUCUGGGAUGUUCA	Cleavage
tae-miR9662a-3p	Traes_6AS_48B701BB41.3	PF02536				GO:GO:0006355,GO:GO:0005739,GO:GO:0003690	AT1G21150.1	Mitochondrial transcription termination factor family protein	LOC_Os06g12100.1	mTERF family protein, expressed	1,00	-1.0	1	21	33	53	UUGAACAUCCCAGAGCCACCG	ACGUGGCUCUGGGAUGUUCAG	Cleavage
tae-miR9662b-3p	Traes_6AS_48B701BB41.3	PF02536				GO:GO:0006355,GO:GO:0005739,GO:GO:0003690	AT1G21150.1	Mitochondrial transcription termination factor family protein	LOC_Os06g12100.1	mTERF family protein, expressed	1,00	-1.0	1	21	32	52	UGAACAUCCCAGAGCCACCGG	CACGUGGCUCUGGGAUGUUCA	Cleavage
tae-miR9662a-3p	Traes_6AS_48B701BB41.4	PF02536				GO:GO:0006355,GO:GO:0005739,GO:GO:0003690	AT1G21150.1	Mitochondrial transcription termination factor family protein	LOC_Os06g12100.1	mTERF family protein, expressed	1,00	-1.0	1	21	33	53	UUGAACAUCCCAGAGCCACCG	ACGUGGCUCUGGGAUGUUCAG	Cleavage
tae-miR9662b-3p	Traes_6AS_48B701BB41.4	PF02536				GO:GO:0006355,GO:GO:0005739,GO:GO:0003690	AT1G21150.1	Mitochondrial transcription termination factor family protein	LOC_Os06g12100.1	mTERF family protein, expressed	1,00	-1.0	1	21	32	52	UGAACAUCCCAGAGCCACCGG	CACGUGGCUCUGGGAUGUUCA	Cleavage
tae-miR9662a-3p	Traes_6AS_48B701BB41.5	PF02536				GO:GO:0006355,GO:GO:0005739,GO:GO:0003690	AT1G21150.1	Mitochondrial transcription termination factor family protein	LOC_Os06g12100.1	mTERF family protein, expressed	1,00	-1.0	1	21	33	53	UUGAACAUCCCAGAGCCACCG	ACGUGGCUCUGGGAUGUUCAG	Cleavage
tae-miR9662b-3p	Traes_6AS_48B701BB41.5	PF02536				GO:GO:0006355,GO:GO:0005739,GO:GO:0003690	AT1G21150.1	Mitochondrial transcription termination factor family protein	LOC_Os06g12100.1	mTERF family protein, expressed	1,00	-1.0	1	21	32	52	UGAACAUCCCAGAGCCACCGG	CACGUGGCUCUGGGAUGUUCA	Cleavage
tae-miR1138	Traes_6AS_5F8725CDA.1	PF02705			K03549	GO:GO:0071805,GO:GO:0016020,GO:GO:0015079	AT4G13420.1	high affinity K+ transporter 5	LOC_Os02g31910.2	potassium transporter, putative, expressed	3,00	-1.0	1	23	2223	2245	GCUUAGAUGUGACAUCCUUAAAA	AGUUAGUUAUGUCACAUCUAAGC	Cleavage
tae-miR1138	Traes_6AS_5F8725CDA.2	PF02705				GO:GO:0071805,GO:GO:0016020,GO:GO:0015079	AT4G13420.1	high affinity K+ transporter 5	LOC_Os02g31910.1	potassium transporter, putative, expressed	3,00	-1.0	1	23	1969	1991	GCUUAGAUGUGACAUCCUUAAAA	AGUUAGUUAUGUCACAUCUAAGC	Cleavage
tae-miR9662a-3p	Traes_6AS_6612DF8D7.1	PF02536				GO:GO:0006355,GO:GO:0005739,GO:GO:0003690	AT5G07900.1	Mitochondrial transcription termination factor family protein	LOC_Os06g12100.1	mTERF family protein, expressed	1,00	-1.0	1	21	81	101	UUGAACAUCCCAGAGCCACCG	CCGUGGCUCUGGGAUGUUCAG	Cleavage
tae-miR9662b-3p	Traes_6AS_6612DF8D7.1	PF02536				GO:GO:0006355,GO:GO:0005739,GO:GO:0003690	AT5G07900.1	Mitochondrial transcription termination factor family protein	LOC_Os06g12100.1	mTERF family protein, expressed	1,00	-1.0	1	21	80	100	UGAACAUCCCAGAGCCACCGG	CCCGUGGCUCUGGGAUGUUCA	Cleavage
tae-miR9662a-3p	Traes_6AS_6612DF8D7.2	PF02536				GO:GO:0006355,GO:GO:0005739,GO:GO:0003690	AT5G07900.1	Mitochondrial transcription termination factor family protein	LOC_Os06g12100.1	mTERF family protein, expressed	1,00	-1.0	1	21	81	101	UUGAACAUCCCAGAGCCACCG	CCGUGGCUCUGGGAUGUUCAG	Cleavage
tae-miR9662b-3p	Traes_6AS_6612DF8D7.2	PF02536				GO:GO:0006355,GO:GO:0005739,GO:GO:0003690	AT5G07900.1	Mitochondrial transcription termination factor family protein	LOC_Os06g12100.1	mTERF family protein, expressed	1,00	-1.0	1	21	80	100	UGAACAUCCCAGAGCCACCGG	CCCGUGGCUCUGGGAUGUUCA	Cleavage
tae-miR9662a-3p	Traes_6AS_6612DF8D7.3	PF02536				GO:GO:0006355,GO:GO:0005739,GO:GO:0003690	AT5G07900.1	Mitochondrial transcription termination factor family protein	LOC_Os06g12100.1	mTERF family protein, expressed	1,00	-1.0	1	21	81	101	UUGAACAUCCCAGAGCCACCG	CCGUGGCUCUGGGAUGUUCAG	Cleavage
tae-miR9662b-3p	Traes_6AS_6612DF8D7.3	PF02536				GO:GO:0006355,GO:GO:0005739,GO:GO:0003690	AT5G07900.1	Mitochondrial transcription termination factor family protein	LOC_Os06g12100.1	mTERF family protein, expressed	1,00	-1.0	1	21	80	100	UGAACAUCCCAGAGCCACCGG	CCCGUGGCUCUGGGAUGUUCA	Cleavage
tae-miR9662a-3p	Traes_6AS_8D2111A83.1	PF02536				GO:GO:0006355,GO:GO:0005739,GO:GO:0003690	AT1G21150.1	Mitochondrial transcription termination factor family protein	LOC_Os06g12100.1	mTERF family protein, expressed	2,05	-1.0	1	21	33	53	UUGAACAUCCCAGAGCCACCG	UCGUGGCUCGGGGAUGUUCAG	Cleavage
tae-miR9662b-3p	Traes_6AS_8D2111A83.1	PF02536				GO:GO:0006355,GO:GO:0005739,GO:GO:0003690	AT1G21150.1	Mitochondrial transcription termination factor family protein	LOC_Os06g12100.1	mTERF family protein, expressed	2,05	-1.0	1	21	32	52	UGAACAUCCCAGAGCCACCGG	CUCGUGGCUCGGGGAUGUUCA	Translation
tae-miR9662a-3p	Traes_6AS_8D2111A83.2	PF02536				GO:GO:0006355,GO:GO:0005739,GO:GO:0003690	AT1G21150.1	Mitochondrial transcription termination factor family protein	LOC_Os06g12100.1	mTERF family protein, expressed	2,05	-1.0	1	21	246	266	UUGAACAUCCCAGAGCCACCG	UCGUGGCUCGGGGAUGUUCAG	Cleavage
tae-miR9662b-3p	Traes_6AS_8D2111A83.2	PF02536				GO:GO:0006355,GO:GO:0005739,GO:GO:0003690	AT1G21150.1	Mitochondrial transcription termination factor family protein	LOC_Os06g12100.1	mTERF family protein, expressed	2,05	-1.0	1	21	245	265	UGAACAUCCCAGAGCCACCGG	CUCGUGGCUCGGGGAUGUUCA	Translation
tae-miR9662a-3p	Traes_6AS_8D2111A83.3	PF02536				GO:GO:0006355,GO:GO:0005739,GO:GO:0003690	AT1G21150.1	Mitochondrial transcription termination factor family protein	LOC_Os06g12100.1	mTERF family protein, expressed	2,05	-1.0	1	21	246	266	UUGAACAUCCCAGAGCCACCG	UCGUGGCUCGGGGAUGUUCAG	Cleavage
tae-miR9662b-3p	Traes_6AS_8D2111A83.3	PF02536				GO:GO:0006355,GO:GO:0005739,GO:GO:0003690	AT1G21150.1	Mitochondrial transcription termination factor family protein	LOC_Os06g12100.1	mTERF family protein, expressed	2,05	-1.0	1	21	245	265	UGAACAUCCCAGAGCCACCGG	CUCGUGGCUCGGGGAUGUUCA	Translation
tae-miR9662a-3p	Traes_6AS_8D2111A83.4	PF02536				GO:GO:0006355,GO:GO:0005739,GO:GO:0003690	AT1G21150.1	Mitochondrial transcription termination factor family protein	LOC_Os06g12100.1	mTERF family protein, expressed	2,05	-1.0	1	21	246	266	UUGAACAUCCCAGAGCCACCG	UCGUGGCUCGGGGAUGUUCAG	Cleavage
tae-miR9662b-3p	Traes_6AS_8D2111A83.4	PF02536				GO:GO:0006355,GO:GO:0005739,GO:GO:0003690	AT1G21150.1	Mitochondrial transcription termination factor family protein	LOC_Os06g12100.1	mTERF family protein, expressed	2,05	-1.0	1	21	245	265	UGAACAUCCCAGAGCCACCGG	CUCGUGGCUCGGGGAUGUUCA	Translation
tae-miR9662a-3p	Traes_6AS_8D2111A83.5	PF02536				GO:GO:0006355,GO:GO:0005739,GO:GO:0003690	AT1G21150.1	Mitochondrial transcription termination factor family protein	LOC_Os06g12100.1	mTERF family protein, expressed	2,05	-1.0	1	21	246	266	UUGAACAUCCCAGAGCCACCG	UCGUGGCUCGGGGAUGUUCAG	Cleavage
tae-miR9662b-3p	Traes_6AS_8D2111A83.5	PF02536				GO:GO:0006355,GO:GO:0005739,GO:GO:0003690	AT1G21150.1	Mitochondrial transcription termination factor family protein	LOC_Os06g12100.1	mTERF family protein, expressed	2,05	-1.0	1	21	245	265	UGAACAUCCCAGAGCCACCGG	CUCGUGGCUCGGGGAUGUUCA	Translation
tae-miR9662a-3p	Traes_6AS_8D2111A83.6						AT1G21150.1	Mitochondrial transcription termination factor family protein	LOC_Os06g12100.1	mTERF family protein, expressed	2,05	-1.0	1	21	246	266	UUGAACAUCCCAGAGCCACCG	UCGUGGCUCGGGGAUGUUCAG	Cleavage
tae-miR9662b-3p	Traes_6AS_8D2111A83.6						AT1G21150.1	Mitochondrial transcription termination factor family protein	LOC_Os06g12100.1	mTERF family protein, expressed	2,05	-1.0	1	21	245	265	UGAACAUCCCAGAGCCACCGG	CUCGUGGCUCGGGGAUGUUCA	Translation
tae-miR9662a-3p	Traes_6AS_8D2111A83.7	PF02536				GO:GO:0006355,GO:GO:0005739,GO:GO:0003690	AT1G21150.1	Mitochondrial transcription termination factor family protein	LOC_Os06g12100.1	mTERF family protein, expressed	2,05	-1.0	1	21	33	53	UUGAACAUCCCAGAGCCACCG	UCGUGGCUCGGGGAUGUUCAG	Cleavage
tae-miR9662b-3p	Traes_6AS_8D2111A83.7	PF02536				GO:GO:0006355,GO:GO:0005739,GO:GO:0003690	AT1G21150.1	Mitochondrial transcription termination factor family protein	LOC_Os06g12100.1	mTERF family protein, expressed	2,05	-1.0	1	21	32	52	UGAACAUCCCAGAGCCACCGG	CUCGUGGCUCGGGGAUGUUCA	Translation
tae-miR1122b-3p	Traes_6AS_8E6E52967.2	PF08263					AT1G17250.1	receptor like protein 3	LOC_Os02g06120.1	Leucine Rich Repeat family protein, expressed	1,05	-1.0	1	21	120	140	AGACUUAUAUGUAGGAACGGA	UCCGUUCCUAAAUAUAAGUCU	Translation
tae-miR156	Traes_6AS_99C6A3B0B.1	PF03110				GO:GO:0005634,GO:GO:0003677	AT5G43270.1	squamosa promoter binding protein-like 2	LOC_Os02g04680.2	OsSPL3 - SBP-box gene family member, expressed	1,00	-1.0	1	21	1477	1497	UGACAGAAGAGAGUGAGCACA	CAUGCUCUCUCUCUUCUGUCA	Cleavage
tae-miR156	Traes_6AS_99C6A3B0B.2	PF03110				GO:GO:0005634,GO:GO:0003677	AT5G43270.3	squamosa promoter binding protein-like 2	LOC_Os02g04680.2	OsSPL3 - SBP-box gene family member, expressed	1,00	-1.0	1	21	1477	1497	UGACAGAAGAGAGUGAGCACA	CAUGCUCUCUCUCUUCUGUCA	Cleavage
tae-miR156	Traes_6AS_99C6A3B0B.3	PF03110				GO:GO:0005634,GO:GO:0003677	AT5G43270.2	squamosa promoter binding protein-like 2	LOC_Os02g04680.2	OsSPL3 - SBP-box gene family member, expressed	1,00	-1.0	1	21	1477	1497	UGACAGAAGAGAGUGAGCACA	CAUGCUCUCUCUCUUCUGUCA	Cleavage
tae-miR156	Traes_6AS_99C6A3B0B.4	PF03110				GO:GO:0005634,GO:GO:0003677	AT5G43270.2	squamosa promoter binding protein-like 2	LOC_Os02g04680.2	OsSPL3 - SBP-box gene family member, expressed	1,00	-1.0	1	21	1156	1176	UGACAGAAGAGAGUGAGCACA	CAUGCUCUCUCUCUUCUGUCA	Cleavage
tae-miR156	Traes_6AS_99C6A3B0B.5	PF03110				GO:GO:0005634,GO:GO:0003677	AT5G43270.3	squamosa promoter binding protein-like 2	LOC_Os02g04680.2	OsSPL3 - SBP-box gene family member, expressed	1,00	-1.0	1	21	1156	1176	UGACAGAAGAGAGUGAGCACA	CAUGCUCUCUCUCUUCUGUCA	Cleavage
tae-miR156	Traes_6AS_99C6A3B0B.6	PF03110				GO:GO:0005634,GO:GO:0003677	AT5G43270.1	squamosa promoter binding protein-like 2	LOC_Os02g04680.2	OsSPL3 - SBP-box gene family member, expressed	1,00	-1.0	1	21	1156	1176	UGACAGAAGAGAGUGAGCACA	CAUGCUCUCUCUCUUCUGUCA	Cleavage
tae-miR156	Traes_6AS_99C6A3B0B.7	PF03110				GO:GO:0005634,GO:GO:0003677	AT5G43270.3	squamosa promoter binding protein-like 2	LOC_Os02g04680.2	OsSPL3 - SBP-box gene family member, expressed	1,00	-1.0	1	21	1156	1176	UGACAGAAGAGAGUGAGCACA	CAUGCUCUCUCUCUUCUGUCA	Cleavage
tae-miR156	Traes_6AS_99C6A3B0B.8	PF03110				GO:GO:0005634,GO:GO:0003677	AT5G43270.1	squamosa promoter binding protein-like 2	LOC_Os02g04680.2	OsSPL3 - SBP-box gene family member, expressed	1,00	-1.0	1	21	1156	1176	UGACAGAAGAGAGUGAGCACA	CAUGCUCUCUCUCUUCUGUCA	Cleavage
tae-miR9662a-3p	Traes_6AS_A2EAE01C1.1	PF02536				GO:GO:0006355,GO:GO:0005739,GO:GO:0003690	AT5G07900.1	Mitochondrial transcription termination factor family protein	LOC_Os06g12100.1	mTERF family protein, expressed	2,05	-1.0	1	21	465	485	UUGAACAUCCCAGAGCCACCG	CCGUGGCUCUGGAAUGUUCAG	Cleavage
tae-miR9662b-3p	Traes_6AS_A2EAE01C1.1	PF02536				GO:GO:0006355,GO:GO:0005739,GO:GO:0003690	AT5G07900.1	Mitochondrial transcription termination factor family protein	LOC_Os06g12100.1	mTERF family protein, expressed	2,05	-1.0	1	21	464	484	UGAACAUCCCAGAGCCACCGG	CCCGUGGCUCUGGAAUGUUCA	Cleavage
tae-miR9662a-3p	Traes_6AS_A2EAE01C1.2	PF02536				GO:GO:0006355,GO:GO:0005739,GO:GO:0003690	AT5G07900.1	Mitochondrial transcription termination factor family protein	LOC_Os06g12100.1	mTERF family protein, expressed	2,05	-1.0	1	21	66	86	UUGAACAUCCCAGAGCCACCG	CCGUGGCUCUGGAAUGUUCAG	Cleavage
tae-miR9662b-3p	Traes_6AS_A2EAE01C1.2	PF02536				GO:GO:0006355,GO:GO:0005739,GO:GO:0003690	AT5G07900.1	Mitochondrial transcription termination factor family protein	LOC_Os06g12100.1	mTERF family protein, expressed	2,05	-1.0	1	21	65	85	UGAACAUCCCAGAGCCACCGG	CCCGUGGCUCUGGAAUGUUCA	Cleavage
tae-miR9662a-3p	Traes_6AS_B24138E62.1	PF02536				GO:GO:0006355,GO:GO:0005739,GO:GO:0003690	AT5G07900.1	Mitochondrial transcription termination factor family protein	LOC_Os06g12100.1	mTERF family protein, expressed	2,05	-1.0	1	21	375	395	UUGAACAUCCCAGAGCCACCG	CCGUGGCUCUGGAAUGUUCAG	Cleavage
tae-miR9662b-3p	Traes_6AS_B24138E62.1	PF02536				GO:GO:0006355,GO:GO:0005739,GO:GO:0003690	AT5G07900.1	Mitochondrial transcription termination factor family protein	LOC_Os06g12100.1	mTERF family protein, expressed	2,05	-1.0	1	21	374	394	UGAACAUCCCAGAGCCACCGG	CCCGUGGCUCUGGAAUGUUCA	Cleavage
tae-miR9662a-3p	Traes_6AS_EBADF37C7.1	PF02536				GO:GO:0006355,GO:GO:0005739,GO:GO:0003690	AT5G07900.1	Mitochondrial transcription termination factor family protein	LOC_Os06g12100.1	mTERF family protein, expressed	2,05	-1.0	1	21	681	701	UUGAACAUCCCAGAGCCACCG	CCGUGGCUCUGGGAUGCUCAG	Cleavage
tae-miR9662b-3p	Traes_6AS_EBADF37C7.1	PF02536				GO:GO:0006355,GO:GO:0005739,GO:GO:0003690	AT5G07900.1	Mitochondrial transcription termination factor family protein	LOC_Os06g12100.1	mTERF family protein, expressed	2,05	-1.0	1	21	680	700	UGAACAUCCCAGAGCCACCGG	CCCGUGGCUCUGGGAUGCUCA	Cleavage
tae-miR9662a-3p	Traes_6AS_EBADF37C7.2	PF02536				GO:GO:0006355,GO:GO:0005739,GO:GO:0003690	AT5G07900.1	Mitochondrial transcription termination factor family protein	LOC_Os06g12100.1	mTERF family protein, expressed	2,05	-1.0	1	21	378	398	UUGAACAUCCCAGAGCCACCG	CCGUGGCUCUGGGAUGCUCAG	Cleavage
tae-miR9662b-3p	Traes_6AS_EBADF37C7.2	PF02536				GO:GO:0006355,GO:GO:0005739,GO:GO:0003690	AT5G07900.1	Mitochondrial transcription termination factor family protein	LOC_Os06g12100.1	mTERF family protein, expressed	2,05	-1.0	1	21	377	397	UGAACAUCCCAGAGCCACCGG	CCCGUGGCUCUGGGAUGCUCA	Cleavage
tae-miR9662a-3p	Traes_6AS_EE73C76541.2	PF02536				GO:GO:0006355,GO:GO:0005739,GO:GO:0003690	AT5G07900.1	Mitochondrial transcription termination factor family protein	LOC_Os06g12100.1	mTERF family protein, expressed	1,00	-1.0	1	21	420	440	UUGAACAUCCCAGAGCCACCG	CCGUGGCUCUGGGAUGUUCAG	Cleavage
tae-miR9662b-3p	Traes_6AS_EE73C76541.2	PF02536				GO:GO:0006355,GO:GO:0005739,GO:GO:0003690	AT5G07900.1	Mitochondrial transcription termination factor family protein	LOC_Os06g12100.1	mTERF family protein, expressed	1,00	-1.0	1	21	419	439	UGAACAUCCCAGAGCCACCGG	CCCGUGGCUCUGGGAUGUUCA	Cleavage
tae-miR9662a-3p	Traes_6AS_EE73C76541.6	PF02536				GO:GO:0006355,GO:GO:0005739,GO:GO:0003690	AT5G07900.1	Mitochondrial transcription termination factor family protein	LOC_Os06g12100.1	mTERF family protein, expressed	1,00	-1.0	1	21	66	86	UUGAACAUCCCAGAGCCACCG	CCGUGGCUCUGGGAUGUUCAG	Cleavage
tae-miR9662b-3p	Traes_6AS_EE73C76541.6	PF02536				GO:GO:0006355,GO:GO:0005739,GO:GO:0003690	AT5G07900.1	Mitochondrial transcription termination factor family protein	LOC_Os06g12100.1	mTERF family protein, expressed	1,00	-1.0	1	21	65	85	UGAACAUCCCAGAGCCACCGG	CCCGUGGCUCUGGGAUGUUCA	Cleavage
tae-miR9662a-3p	Traes_6AS_EE8BA0A57.1	PF02536				GO:GO:0006355,GO:GO:0005739,GO:GO:0003690	AT1G21150.1	Mitochondrial transcription termination factor family protein	LOC_Os06g12100.1	mTERF family protein, expressed	2,00	-1.0	1	21	462	482	UUGAACAUCCCAGAGCCACCG	CCGUGCCUCUGGGAUGUUCAG	Cleavage
tae-miR9662b-3p	Traes_6AS_EE8BA0A57.1	PF02536				GO:GO:0006355,GO:GO:0005739,GO:GO:0003690	AT1G21150.1	Mitochondrial transcription termination factor family protein	LOC_Os06g12100.1	mTERF family protein, expressed	2,00	-1.0	1	21	461	481	UGAACAUCCCAGAGCCACCGG	CCCGUGCCUCUGGGAUGUUCA	Cleavage
tae-miR9662a-3p	Traes_6AS_EE8BA0A57.2	PF02536				GO:GO:0006355,GO:GO:0005739,GO:GO:0003690	AT5G07900.1	Mitochondrial transcription termination factor family protein	LOC_Os06g12100.1	mTERF family protein, expressed	2,00	-1.0	1	21	510	530	UUGAACAUCCCAGAGCCACCG	CCGUGCCUCUGGGAUGUUCAG	Cleavage
tae-miR9662b-3p	Traes_6AS_EE8BA0A57.2	PF02536				GO:GO:0006355,GO:GO:0005739,GO:GO:0003690	AT5G07900.1	Mitochondrial transcription termination factor family protein	LOC_Os06g12100.1	mTERF family protein, expressed	2,00	-1.0	1	21	509	529	UGAACAUCCCAGAGCCACCGG	CCCGUGCCUCUGGGAUGUUCA	Cleavage
tae-miR5049-3p	Traes_6AS_F5DBB6DBD.3	PF13855	KOG0472			GO:GO:0005515	AT1G72300.1	Leucine-rich receptor-like protein kinase family protein	LOC_Os02g05970.1	phytosulfokine receptor precursor, putative, expressed	1,05	-1.0	1	21	1927	1947	AAUAUGGAUCGGAGGGAGUAC	UUACUCCCUCCAAUCCAUAUU	Translation
tae-miR9662a-3p	Traes_6AS_FBBCFBCB0.1	PF02536	KOG1267			GO:GO:0006355,GO:GO:0005739,GO:GO:0003690	AT5G07900.1	Mitochondrial transcription termination factor family protein	LOC_Os06g12100.1	mTERF family protein, expressed	2,05	-1.0	1	21	681	701	UUGAACAUCCCAGAGCCACCG	CCGUGGCUCUGGGAUGUUUAG	Cleavage
tae-miR9662b-3p	Traes_6AS_FBBCFBCB0.1	PF02536	KOG1267			GO:GO:0006355,GO:GO:0005739,GO:GO:0003690	AT5G07900.1	Mitochondrial transcription termination factor family protein	LOC_Os06g12100.1	mTERF family protein, expressed	2,05	-1.0	1	21	680	700	UGAACAUCCCAGAGCCACCGG	CCCGUGGCUCUGGGAUGUUUA	Cleavage
tae-miR1127b-3p	Traes_6BL_028068EFA.2		KOG1877				AT5G21080.1	Uncharacterized protein	LOC_Os02g53990.1	cyclin-related protein, putative, expressed	1,05	-1.0	1	21	2801	2821	ACAAGUAUUUCUGGACGGAGG	CCUCCGUCCGGAAAUACUUGU	Cleavage
tae-miR6197-5p	Traes_6BL_67725F06B.2	PF02358		3.1.3.12		GO:GO:0005992,GO:GO:0003824	AT5G65140.2	Haloacid dehalogenase-like hydrolase (HAD) superfamily protein	LOC_Os02g51680.1	uncharacterized glycosyl hydrolase Rv2006/MT2062, putative, expressed	3,00	-1.0	1	21	278	298	UCUGUAAACAAAUGUAGGACG	GAACCUCCAUUUGUUUACAGG	Cleavage
tae-miR1127b-3p	Traes_6BL_731ED1C4C.1	PF00646				GO:GO:0005515	AT5G56820.1	F-box/RNI-like/FBD-like domains-containing protein	LOC_Os02g57910.1	OsFBX73 - F-box domain containing protein, expressed	1,05	-1.0	1	21	1020	1040	ACAAGUAUUUCUGGACGGAGG	UCUCCGUCCAAAAAUACUUGU	Translation
tae-miR1130b-3p	Traes_6BL_7BD68BAC2.3	PF00366				GO:GO:0006412,GO:GO:0005840,GO:GO:0005622,GO:GO:0003735	AT5G23740.1	ribosomal protein S11-beta	LOC_Os04g52361.1	ribosomal protein S17, putative, expressed	1,05	-1.0	1	21	215	235	UCUUAUAUUAUGGGACGGAGG	CCUCCGUCCCAUAAUGUAAGA	Cleavage
tae-miR1122a	Traes_6BL_7E9C131F4.3	PF00249	KOG0724				AT5G52660.1	Homeodomain-like superfamily protein	LOC_Os02g45670.1	myb-like DNA-binding domain containing protein, expressed	0,00	-1.0	1	20	761	780	UAGAUACAUCCGUAUCUAGA	UCUAGAUACGGAUGUAUCUA	Cleavage
tae-miR1130b-3p	Traes_6BL_887005D55.2										1,05	-1.0	1	21	79	99	UCUUAUAUUAUGGGACGGAGG	CCUCCGUCCCAUAAUAUAAUA	Cleavage
tae-miR1118	Traes_6BL_E14604962.2	PF00076				GO:GO:0003676	AT5G51120.2	polyadenylate-binding protein 1	LOC_Os02g52140.2	RNA recognition motif containing protein, putative, expressed	2,00	-1.0	1	23	35	57	CACUACAUUAUGGAAUGGAGGGA	UCCCUCUGUUCCAUAAUGUAGUG	Cleavage
tae-miR1118	Traes_6BL_E14604962.3	PF00076				GO:GO:0003676	AT5G51120.2	polyadenylate-binding protein 1	LOC_Os02g52140.2	RNA recognition motif containing protein, putative, expressed	2,00	-1.0	1	23	35	57	CACUACAUUAUGGAAUGGAGGGA	UCCCUCUGUUCCAUAAUGUAGUG	Cleavage
tae-miR1127b-3p	Traes_6BS_1AFDF35E5.3										2,05	-1.0	1	21	171	191	ACAAGUAUUUCUGGACGGAGG	CUUUCGUCCGGAAAUACUUGU	Cleavage
tae-miR9662a-3p	Traes_6BS_28F0D2955.1	PF02536				GO:GO:0006355,GO:GO:0005739,GO:GO:0003690	AT5G64950.1	Mitochondrial transcription termination factor family protein	LOC_Os06g12100.1	mTERF family protein, expressed	2,05	-1.0	1	21	396	416	UUGAACAUCCCAGAGCCACCG	CUGUGGCUCUCGGAUGUUCAG	Translation
tae-miR9662b-3p	Traes_6BS_28F0D2955.1	PF02536				GO:GO:0006355,GO:GO:0005739,GO:GO:0003690	AT5G64950.1	Mitochondrial transcription termination factor family protein	LOC_Os06g12100.1	mTERF family protein, expressed	2,05	-1.0	1	21	395	415	UGAACAUCCCAGAGCCACCGG	CCUGUGGCUCUCGGAUGUUCA	Translation
tae-miR9662a-3p	Traes_6BS_2AC6A2287.1	PF02536				GO:GO:0006355,GO:GO:0005739,GO:GO:0003690	AT5G07900.1	Mitochondrial transcription termination factor family protein	LOC_Os06g12100.1	mTERF family protein, expressed	2,05	-1.0	1	21	66	86	UUGAACAUCCCAGAGCCACCG	CCGUGGCUCUGGGAUGCUCAG	Cleavage
tae-miR9662b-3p	Traes_6BS_2AC6A2287.1	PF02536				GO:GO:0006355,GO:GO:0005739,GO:GO:0003690	AT5G07900.1	Mitochondrial transcription termination factor family protein	LOC_Os06g12100.1	mTERF family protein, expressed	2,05	-1.0	1	21	65	85	UGAACAUCCCAGAGCCACCGG	CCCGUGGCUCUGGGAUGCUCA	Cleavage
tae-miR9662a-3p	Traes_6BS_2F108C5BB.1	PF02536				GO:GO:0006355,GO:GO:0005739,GO:GO:0003690	AT5G07900.1	Mitochondrial transcription termination factor family protein	LOC_Os06g12100.1	mTERF family protein, expressed	2,00	-1.0	1	21	435	455	UUGAACAUCCCAGAGCCACCG	UCGUGGCGCUGGGAUGUUCAG	Cleavage
tae-miR9662b-3p	Traes_6BS_2F108C5BB.1	PF02536				GO:GO:0006355,GO:GO:0005739,GO:GO:0003690	AT5G07900.1	Mitochondrial transcription termination factor family protein	LOC_Os06g12100.1	mTERF family protein, expressed	2,05	-1.0	1	21	434	454	UGAACAUCCCAGAGCCACCGG	CUCGUGGCGCUGGGAUGUUCA	Cleavage
tae-miR408	Traes_6BS_348308070.13	PF12937				GO:GO:0005515	AT3G17540.1	F-box and associated interaction domains-containing protein	LOC_Os10g04700.1	OsFBX361 - F-box domain containing protein, expressed	2,05	-1.0	1	21	2	22	CUGCACUGCCUCUUCCCUGGC	CCCAGGGCCGAGGCAGUGCAG	Cleavage
tae-miR408	Traes_6BS_348308070.15	PF12937				GO:GO:0005515	AT3G17540.1	F-box and associated interaction domains-containing protein	LOC_Os10g04700.1	OsFBX361 - F-box domain containing protein, expressed	2,05	-1.0	1	21	11	31	CUGCACUGCCUCUUCCCUGGC	CCCAGGGCCGAGGCAGUGCAG	Cleavage
tae-miR408	Traes_6BS_348308070.17	PF12937				GO:GO:0005515	AT3G17540.1	F-box and associated interaction domains-containing protein	LOC_Os10g04700.1	OsFBX361 - F-box domain containing protein, expressed	2,05	-1.0	1	21	14	34	CUGCACUGCCUCUUCCCUGGC	CCCAGGGCCGAGGCAGUGCAG	Cleavage
tae-miR408	Traes_6BS_348308070.19	PF12937				GO:GO:0005515	AT3G17540.1	F-box and associated interaction domains-containing protein	LOC_Os10g04700.1	OsFBX361 - F-box domain containing protein, expressed	2,05	-1.0	1	21	11	31	CUGCACUGCCUCUUCCCUGGC	CCCAGGGCCGAGGCAGUGCAG	Cleavage
tae-miR408	Traes_6BS_348308070.2	PF12937				GO:GO:0005515	AT5G03970.2	F-box associated ubiquitination effector family protein	LOC_Os10g04700.2	OsFBX361 - F-box domain containing protein, expressed	2,05	-1.0	1	21	14	34	CUGCACUGCCUCUUCCCUGGC	CCCAGGGCCGAGGCAGUGCAG	Cleavage
tae-miR408	Traes_6BS_348308070.3	PF12937				GO:GO:0005515	AT3G17540.1	F-box and associated interaction domains-containing protein	LOC_Os10g04700.1	OsFBX361 - F-box domain containing protein, expressed	2,05	-1.0	1	21	17	37	CUGCACUGCCUCUUCCCUGGC	CCCAGGGCCGAGGCAGUGCAG	Cleavage
tae-miR408	Traes_6BS_348308070.5	PF12937				GO:GO:0005515	AT3G17540.1	F-box and associated interaction domains-containing protein	LOC_Os10g04700.2	OsFBX361 - F-box domain containing protein, expressed	2,05	-1.0	1	21	17	37	CUGCACUGCCUCUUCCCUGGC	CCCAGGGCCGAGGCAGUGCAG	Cleavage
tae-miR408	Traes_6BS_348308070.6	PF12937				GO:GO:0005515	AT5G03970.2	F-box associated ubiquitination effector family protein	LOC_Os10g04700.2	OsFBX361 - F-box domain containing protein, expressed	2,05	-1.0	1	21	14	34	CUGCACUGCCUCUUCCCUGGC	CCCAGGGCCGAGGCAGUGCAG	Cleavage
tae-miR408	Traes_6BS_348308070.8	PF12937				GO:GO:0005515	AT3G17540.1	F-box and associated interaction domains-containing protein	LOC_Os10g04700.1	OsFBX361 - F-box domain containing protein, expressed	2,05	-1.0	1	21	17	37	CUGCACUGCCUCUUCCCUGGC	CCCAGGGCCGAGGCAGUGCAG	Cleavage
tae-miR408	Traes_6BS_348308070.9	PF12937				GO:GO:0005515	AT3G17540.1	F-box and associated interaction domains-containing protein	LOC_Os10g04700.1	OsFBX361 - F-box domain containing protein, expressed	2,05	-1.0	1	21	17	37	CUGCACUGCCUCUUCCCUGGC	CCCAGGGCCGAGGCAGUGCAG	Cleavage
tae-miR9662a-3p	Traes_6BS_3A00751B5.1	PF02536				GO:GO:0006355,GO:GO:0005739,GO:GO:0003690	AT5G07900.1	Mitochondrial transcription termination factor family protein	LOC_Os06g12100.1	mTERF family protein, expressed	2,05	-1.0	1	21	69	89	UUGAACAUCCCAGAGCCACCG	CCGUGGCUCUGGGAUGUUUAG	Cleavage
tae-miR9662b-3p	Traes_6BS_3A00751B5.1	PF02536				GO:GO:0006355,GO:GO:0005739,GO:GO:0003690	AT5G07900.1	Mitochondrial transcription termination factor family protein	LOC_Os06g12100.1	mTERF family protein, expressed	2,05	-1.0	1	21	68	88	UGAACAUCCCAGAGCCACCGG	CCCGUGGCUCUGGGAUGUUUA	Cleavage
tae-miR9662a-3p	Traes_6BS_3A00751B5.2	PF02536				GO:GO:0006355,GO:GO:0005739,GO:GO:0003690	AT5G07900.1	Mitochondrial transcription termination factor family protein	LOC_Os06g12100.1	mTERF family protein, expressed	2,05	-1.0	1	21	69	89	UUGAACAUCCCAGAGCCACCG	CCGUGGCUCUGGGAUGUUUAG	Cleavage
tae-miR9662b-3p	Traes_6BS_3A00751B5.2	PF02536				GO:GO:0006355,GO:GO:0005739,GO:GO:0003690	AT5G07900.1	Mitochondrial transcription termination factor family protein	LOC_Os06g12100.1	mTERF family protein, expressed	2,05	-1.0	1	21	68	88	UGAACAUCCCAGAGCCACCGG	CCCGUGGCUCUGGGAUGUUUA	Cleavage
tae-miR9662a-3p	Traes_6BS_46641470F.1	PF02536				GO:GO:0006355,GO:GO:0005739,GO:GO:0003690	AT1G21150.1	Mitochondrial transcription termination factor family protein	LOC_Os06g12100.1	mTERF family protein, expressed	2,05	-1.0	1	21	33	53	UUGAACAUCCCAGAGCCACCG	CCGUGGCUCUGGGAUGUUUAG	Cleavage
tae-miR9662b-3p	Traes_6BS_46641470F.1	PF02536				GO:GO:0006355,GO:GO:0005739,GO:GO:0003690	AT1G21150.1	Mitochondrial transcription termination factor family protein	LOC_Os06g12100.1	mTERF family protein, expressed	2,05	-1.0	1	21	32	52	UGAACAUCCCAGAGCCACCGG	CCCGUGGCUCUGGGAUGUUUA	Cleavage

tae-miR9662a-3p	Traes_6BS_4E6E57FEB.1	PF02536				GO:GO:0006355,GO:GO:0005739,GO:GO:0003690	AT5G07900.1	Mitochondrial transcription termination factor family protein	LOC_Os06g12100.1	mTERF family protein, expressed	3,00	-1.0	1	21	65	86	UUGAACAUCCCAGAGCCAC-CG	CGCGUGGCUCUGGGAUGUUCAG	Cleavage
tae-miR9662b-3p	Traes_6BS_4E6E57FEB.1	PF02536				GO:GO:0006355,GO:GO:0005739,GO:GO:0003690	AT5G07900.1	Mitochondrial transcription termination factor family protein	LOC_Os06g12100.1	mTERF family protein, expressed	2,00	-1.0	1	21	64	85	UGAACAUCCCAGAGCCAC-CGG	CCGCGUGGCUCUGGGAUGUUCA	Cleavage
tae-miR9662a-3p	Traes_6BS_4E6E57FEB.2	PF02536				GO:GO:0006355,GO:GO:0005739,GO:GO:0003690	AT5G07900.1	Mitochondrial transcription termination factor family protein	LOC_Os06g12100.1	mTERF family protein, expressed	3,00	-1.0	1	21	65	86	UUGAACAUCCCAGAGCCAC-CG	CGCGUGGCUCUGGGAUGUUCAG	Cleavage
tae-miR9662b-3p	Traes_6BS_4E6E57FEB.2	PF02536				GO:GO:0006355,GO:GO:0005739,GO:GO:0003690	AT5G07900.1	Mitochondrial transcription termination factor family protein	LOC_Os06g12100.1	mTERF family protein, expressed	2,00	-1.0	1	21	64	85	UGAACAUCCCAGAGCCAC-CGG	CCGCGUGGCUCUGGGAUGUUCA	Cleavage
tae-miR9662a-3p	Traes_6BS_4E6E57FEB.3	PF02536				GO:GO:0006355,GO:GO:0005739,GO:GO:0003690	AT5G07900.1	Mitochondrial transcription termination factor family protein	LOC_Os06g12100.1	mTERF family protein, expressed	3,00	-1.0	1	21	65	86	UUGAACAUCCCAGAGCCAC-CG	CGCGUGGCUCUGGGAUGUUCAG	Cleavage
tae-miR9662b-3p	Traes_6BS_4E6E57FEB.3	PF02536				GO:GO:0006355,GO:GO:0005739,GO:GO:0003690	AT5G07900.1	Mitochondrial transcription termination factor family protein	LOC_Os06g12100.1	mTERF family protein, expressed	2,00	-1.0	1	21	64	85	UGAACAUCCCAGAGCCAC-CGG	CCGCGUGGCUCUGGGAUGUUCA	Cleavage
tae-miR9662b-3p	Traes_6BS_508C2BEA2.1	PF02536				GO:GO:0006355,GO:GO:0005739,GO:GO:0003690	AT1G21150.1	Mitochondrial transcription termination factor family protein	LOC_Os06g12100.1	mTERF family protein, expressed	3,00	-1.0	1	21	32	52	UGAACAUCCCAGAGCCACCGG	CCUGUGGUUCUGGGAUGUUCG	Cleavage
tae-miR156	Traes_6BS_542961EA4.1								LOC_Os06g45310.1	OsSPL11 - SBP-box gene family member, expressed	1,00	-1.0	1	21	283	303	UGACAGAAGAGAGUGAGCACA	CGUGCUCUCUCUCUUCUGUCA	Cleavage
tae-miR156	Traes_6BS_542961EA4.2								LOC_Os06g45310.1	OsSPL11 - SBP-box gene family member, expressed	1,00	-1.0	1	21	349	369	UGACAGAAGAGAGUGAGCACA	CGUGCUCUCUCUCUUCUGUCA	Cleavage
tae-miR1122b-3p	Traes_6BS_7965FEB75.1	PF00300	KOG0235	3.1.3.3	K15634		AT3G50520.1	Phosphoglycerate mutase family protein	LOC_Os08g37140.1	phosphoglycerate mutase, putative, expressed	2,05	-1.0	1	21	87	107	AGACUUAUAUGUAGGAACGGA	UCCGUUCGUAUAUAUAAGUCU	Translation
tae-miR9662a-3p	Traes_6BS_7F1846171.1	PF02536				GO:GO:0006355,GO:GO:0005739,GO:GO:0003690	AT5G07900.1	Mitochondrial transcription termination factor family protein	LOC_Os06g12100.1	mTERF family protein, expressed	2,05	-1.0	1	21	33	53	UUGAACAUCCCAGAGCCACCG	CUGUGGCUCUCGGAUGUUCAG	Translation
tae-miR9662b-3p	Traes_6BS_7F1846171.1	PF02536				GO:GO:0006355,GO:GO:0005739,GO:GO:0003690	AT5G07900.1	Mitochondrial transcription termination factor family protein	LOC_Os06g12100.1	mTERF family protein, expressed	2,05	-1.0	1	21	32	52	UGAACAUCCCAGAGCCACCGG	CCUGUGGCUCUCGGAUGUUCA	Translation
tae-miR9662a-3p	Traes_6BS_82F5BF11F.1	PF02536				GO:GO:0006355,GO:GO:0005739,GO:GO:0003690	AT1G79220.1	Mitochondrial transcription termination factor family protein	LOC_Os06g12100.1	mTERF family protein, expressed	2,00	-1.0	1	21	267	287	UUGAACAUCCCAGAGCCACCG	CCGUGCCUCUGGGAUGUUCAG	Cleavage
tae-miR9662b-3p	Traes_6BS_82F5BF11F.1	PF02536				GO:GO:0006355,GO:GO:0005739,GO:GO:0003690	AT1G79220.1	Mitochondrial transcription termination factor family protein	LOC_Os06g12100.1	mTERF family protein, expressed	2,00	-1.0	1	21	266	286	UGAACAUCCCAGAGCCACCGG	CCCGUGCCUCUGGGAUGUUCA	Cleavage
tae-miR9662a-3p	Traes_6BS_843505CB3.1	PF02536				GO:GO:0006355,GO:GO:0005739,GO:GO:0003690	AT1G21150.1	Mitochondrial transcription termination factor family protein	LOC_Os06g12100.1	mTERF family protein, expressed	2,05	-1.0	1	21	69	89	UUGAACAUCCCAGAGCCACCG	CCGUGGCUCUGGAAUGUUCAG	Cleavage
tae-miR9662b-3p	Traes_6BS_843505CB3.1	PF02536				GO:GO:0006355,GO:GO:0005739,GO:GO:0003690	AT1G21150.1	Mitochondrial transcription termination factor family protein	LOC_Os06g12100.1	mTERF family protein, expressed	2,05	-1.0	1	21	68	88	UGAACAUCCCAGAGCCACCGG	CCCGUGGCUCUGGAAUGUUCA	Cleavage
tae-miR9662a-3p	Traes_6BS_843505CB3.2	PF02536				GO:GO:0006355,GO:GO:0005739,GO:GO:0003690	AT1G21150.1	Mitochondrial transcription termination factor family protein	LOC_Os06g12100.1	mTERF family protein, expressed	2,05	-1.0	1	21	69	89	UUGAACAUCCCAGAGCCACCG	CCGUGGCUCUGGAAUGUUCAG	Cleavage
tae-miR9662b-3p	Traes_6BS_843505CB3.2	PF02536				GO:GO:0006355,GO:GO:0005739,GO:GO:0003690	AT1G21150.1	Mitochondrial transcription termination factor family protein	LOC_Os06g12100.1	mTERF family protein, expressed	2,05	-1.0	1	21	68	88	UGAACAUCCCAGAGCCACCGG	CCCGUGGCUCUGGAAUGUUCA	Cleavage
tae-miR9662a-3p	Traes_6BS_843505CB3.3	PF02536				GO:GO:0006355,GO:GO:0005739,GO:GO:0003690	AT1G21150.1	Mitochondrial transcription termination factor family protein	LOC_Os06g12100.1	mTERF family protein, expressed	2,05	-1.0	1	21	69	89	UUGAACAUCCCAGAGCCACCG	CCGUGGCUCUGGAAUGUUCAG	Cleavage
tae-miR9662b-3p	Traes_6BS_843505CB3.3	PF02536				GO:GO:0006355,GO:GO:0005739,GO:GO:0003690	AT1G21150.1	Mitochondrial transcription termination factor family protein	LOC_Os06g12100.1	mTERF family protein, expressed	2,05	-1.0	1	21	68	88	UGAACAUCCCAGAGCCACCGG	CCCGUGGCUCUGGAAUGUUCA	Cleavage
tae-miR9662a-3p	Traes_6BS_843505CB3.4	PF02536				GO:GO:0006355,GO:GO:0005739,GO:GO:0003690	AT1G21150.1	Mitochondrial transcription termination factor family protein	LOC_Os06g12100.1	mTERF family protein, expressed	2,05	-1.0	1	21	69	89	UUGAACAUCCCAGAGCCACCG	CCGUGGCUCUGGAAUGUUCAG	Cleavage
tae-miR9662b-3p	Traes_6BS_843505CB3.4	PF02536				GO:GO:0006355,GO:GO:0005739,GO:GO:0003690	AT1G21150.1	Mitochondrial transcription termination factor family protein	LOC_Os06g12100.1	mTERF family protein, expressed	2,05	-1.0	1	21	68	88	UGAACAUCCCAGAGCCACCGG	CCCGUGGCUCUGGAAUGUUCA	Cleavage
tae-miR9662a-3p	Traes_6BS_843505CB3.5	PF02536				GO:GO:0006355,GO:GO:0005739,GO:GO:0003690	AT1G21150.1	Mitochondrial transcription termination factor family protein	LOC_Os06g12100.1	mTERF family protein, expressed	2,05	-1.0	1	21	69	89	UUGAACAUCCCAGAGCCACCG	CCGUGGCUCUGGAAUGUUCAG	Cleavage
tae-miR9662b-3p	Traes_6BS_843505CB3.5	PF02536				GO:GO:0006355,GO:GO:0005739,GO:GO:0003690	AT1G21150.1	Mitochondrial transcription termination factor family protein	LOC_Os06g12100.1	mTERF family protein, expressed	2,05	-1.0	1	21	68	88	UGAACAUCCCAGAGCCACCGG	CCCGUGGCUCUGGAAUGUUCA	Cleavage
tae-miR9662a-3p	Traes_6BS_843505CB3.6	PF02536				GO:GO:0006355,GO:GO:0005739,GO:GO:0003690	AT1G21150.1	Mitochondrial transcription termination factor family protein	LOC_Os06g12100.1	mTERF family protein, expressed	2,05	-1.0	1	21	69	89	UUGAACAUCCCAGAGCCACCG	CCGUGGCUCUGGAAUGUUCAG	Cleavage
tae-miR9662b-3p	Traes_6BS_843505CB3.6	PF02536				GO:GO:0006355,GO:GO:0005739,GO:GO:0003690	AT1G21150.1	Mitochondrial transcription termination factor family protein	LOC_Os06g12100.1	mTERF family protein, expressed	2,05	-1.0	1	21	68	88	UGAACAUCCCAGAGCCACCGG	CCCGUGGCUCUGGAAUGUUCA	Cleavage
tae-miR9662a-3p	Traes_6BS_843505CB3.7	PF02536				GO:GO:0006355,GO:GO:0005739,GO:GO:0003690	AT1G21150.1	Mitochondrial transcription termination factor family protein	LOC_Os06g12100.1	mTERF family protein, expressed	2,05	-1.0	1	21	69	89	UUGAACAUCCCAGAGCCACCG	CCGUGGCUCUGGAAUGUUCAG	Cleavage
tae-miR9662b-3p	Traes_6BS_843505CB3.7	PF02536				GO:GO:0006355,GO:GO:0005739,GO:GO:0003690	AT1G21150.1	Mitochondrial transcription termination factor family protein	LOC_Os06g12100.1	mTERF family protein, expressed	2,05	-1.0	1	21	68	88	UGAACAUCCCAGAGCCACCGG	CCCGUGGCUCUGGAAUGUUCA	Cleavage
tae-miR1127b-3p	Traes_6BS_94CE86A01.2	PF00069	KOG0984	2.7.11.1		GO:GO:0006468,GO:GO:0005524,GO:GO:0004672	AT4G23270.1	cysteine-rich RLK (RECEPTOR-like protein kinase) 19	LOC_Os10g12620.1	protein kinase domain containing protein, expressed	2,05	-1.0	1	21	763	783	ACAAGUAUUUCUGGACGGAGG	AAUCCGUUCAGAAUUACUUGU	Cleavage
tae-miR1127a	Traes_6BS_94CE86A01.2	PF00069	KOG0984	2.7.11.1		GO:GO:0006468,GO:GO:0005524,GO:GO:0004672	AT4G23270.1	cysteine-rich RLK (RECEPTOR-like protein kinase) 19	LOC_Os10g12620.1	protein kinase domain containing protein, expressed	1,05	-1.0	1	19	842	860	UCCUUCCGUUCGGAAUUAC	GUAAUUCCGAACGGAGGGA	Cleavage
tae-miR9662a-3p	Traes_6BS_97770F04E.1	PF02536				GO:GO:0006355,GO:GO:0005739,GO:GO:0003690	AT5G07900.1	Mitochondrial transcription termination factor family protein	LOC_Os06g12100.1	mTERF family protein, expressed	2,05	-1.0	1	21	33	53	UUGAACAUCCCAGAGCCACCG	UCGUGGCUCUGGAAUGUUCAG	Cleavage
tae-miR9662b-3p	Traes_6BS_97770F04E.1	PF02536				GO:GO:0006355,GO:GO:0005739,GO:GO:0003690	AT5G07900.1	Mitochondrial transcription termination factor family protein	LOC_Os06g12100.1	mTERF family protein, expressed	2,05	-1.0	1	21	32	52	UGAACAUCCCAGAGCCACCGG	CUCGUGGCUCUGGAAUGUUCA	Cleavage
tae-miR9662a-3p	Traes_6BS_97770F04E.3	PF02536				GO:GO:0006355,GO:GO:0005739,GO:GO:0003690	AT5G07900.1	Mitochondrial transcription termination factor family protein	LOC_Os06g12100.1	mTERF family protein, expressed	2,05	-1.0	1	21	33	53	UUGAACAUCCCAGAGCCACCG	UCGUGGCUCUGGAAUGUUCAG	Cleavage
tae-miR9662b-3p	Traes_6BS_97770F04E.3	PF02536				GO:GO:0006355,GO:GO:0005739,GO:GO:0003690	AT5G07900.1	Mitochondrial transcription termination factor family protein	LOC_Os06g12100.1	mTERF family protein, expressed	2,05	-1.0	1	21	32	52	UGAACAUCCCAGAGCCACCGG	CUCGUGGCUCUGGAAUGUUCA	Cleavage
tae-miR9662a-3p	Traes_6BS_97770F04E.4	PF02536				GO:GO:0006355,GO:GO:0005739,GO:GO:0003690	AT5G07900.1	Mitochondrial transcription termination factor family protein	LOC_Os06g12100.1	mTERF family protein, expressed	2,05	-1.0	1	21	33	53	UUGAACAUCCCAGAGCCACCG	UCGUGGCUCUGGAAUGUUCAG	Cleavage
tae-miR9662b-3p	Traes_6BS_97770F04E.4	PF02536				GO:GO:0006355,GO:GO:0005739,GO:GO:0003690	AT5G07900.1	Mitochondrial transcription termination factor family protein	LOC_Os06g12100.1	mTERF family protein, expressed	2,05	-1.0	1	21	32	52	UGAACAUCCCAGAGCCACCGG	CUCGUGGCUCUGGAAUGUUCA	Cleavage
tae-miR9662a-3p	Traes_6BS_97770F04E.5	PF02536				GO:GO:0006355,GO:GO:0005739,GO:GO:0003690	AT5G07900.1	Mitochondrial transcription termination factor family protein	LOC_Os06g12100.1	mTERF family protein, expressed	2,05	-1.0	1	21	33	53	UUGAACAUCCCAGAGCCACCG	UCGUGGCUCUGGAAUGUUCAG	Cleavage
tae-miR9662b-3p	Traes_6BS_97770F04E.5	PF02536				GO:GO:0006355,GO:GO:0005739,GO:GO:0003690	AT5G07900.1	Mitochondrial transcription termination factor family protein	LOC_Os06g12100.1	mTERF family protein, expressed	2,05	-1.0	1	21	32	52	UGAACAUCCCAGAGCCACCGG	CUCGUGGCUCUGGAAUGUUCA	Cleavage
tae-miR9662a-3p	Traes_6BS_97770F04E.6	PF02536				GO:GO:0006355,GO:GO:0005739,GO:GO:0003690	AT5G07900.1	Mitochondrial transcription termination factor family protein	LOC_Os06g12100.1	mTERF family protein, expressed	2,05	-1.0	1	21	33	53	UUGAACAUCCCAGAGCCACCG	UCGUGGCUCUGGAAUGUUCAG	Cleavage
tae-miR9662b-3p	Traes_6BS_97770F04E.6	PF02536				GO:GO:0006355,GO:GO:0005739,GO:GO:0003690	AT5G07900.1	Mitochondrial transcription termination factor family protein	LOC_Os06g12100.1	mTERF family protein, expressed	2,05	-1.0	1	21	32	52	UGAACAUCCCAGAGCCACCGG	CUCGUGGCUCUGGAAUGUUCA	Cleavage
tae-miR1137a	Traes_6BS_A4F1E63CE.2	PF05971		2.1.1.181		GO:GO:0008168	AT2G21070.3	methyltransferases	LOC_Os02g02880.1	expressed protein	2,00	-1.0	1	20	884	903	UAGUACAAAGUUGAGUCAUC	AGUGUCUCAACUUUGUACUA	Cleavage
tae-miR1137a	Traes_6BS_A4F1E63CE.3	PF05971		2.1.1.181		GO:GO:0008168	AT2G21070.3	methyltransferases	LOC_Os02g02880.1	expressed protein	2,00	-1.0	1	20	871	890	UAGUACAAAGUUGAGUCAUC	AGUGUCUCAACUUUGUACUA	Cleavage
tae-miR1128	Traes_6BS_BF5C480A9.12										2,05	-1.0	1	21	60	80	UACUACUCCCUCCGUCCGAAA	UUUGGGACGGAGGGAGUAUUA	Cleavage
tae-miR9662a-3p	Traes_6BS_C3247654A.1	PF02536				GO:GO:0006355,GO:GO:0005739,GO:GO:0003690	AT5G07900.1	Mitochondrial transcription termination factor family protein	LOC_Os06g12100.1	mTERF family protein, expressed	2,05	-1.0	1	21	57	77	UUGAACAUCCCAGAGCCACCG	CUGUGGCUCUGCGAUGUUCAG	Translation
tae-miR9662b-3p	Traes_6BS_C3247654A.1	PF02536				GO:GO:0006355,GO:GO:0005739,GO:GO:0003690	AT5G07900.1	Mitochondrial transcription termination factor family protein	LOC_Os06g12100.1	mTERF family protein, expressed	2,05	-1.0	1	21	56	76	UGAACAUCCCAGAGCCACCGG	CCUGUGGCUCUGCGAUGUUCA	Cleavage
tae-miR9662a-3p	Traes_6BS_CBD931158.1	PF02536				GO:GO:0006355,GO:GO:0005739,GO:GO:0003690	AT1G21150.1	Mitochondrial transcription termination factor family protein	LOC_Os06g12100.1	mTERF family protein, expressed	2,00	-1.0	1	21	33	53	UUGAACAUCCCAGAGCCACCG	CCGUGCCUCUGGGAUGUUCAG	Cleavage
tae-miR9662b-3p	Traes_6BS_CBD931158.1	PF02536				GO:GO:0006355,GO:GO:0005739,GO:GO:0003690	AT1G21150.1	Mitochondrial transcription termination factor family protein	LOC_Os06g12100.1	mTERF family protein, expressed	2,00	-1.0	1	21	32	52	UGAACAUCCCAGAGCCACCGG	CCCGUGCCUCUGGGAUGUUCA	Cleavage
tae-miR9662a-3p	Traes_6BS_CBD931158.2	PF02536				GO:GO:0006355,GO:GO:0005739,GO:GO:0003690	AT1G21150.1	Mitochondrial transcription termination factor family protein	LOC_Os06g12100.1	mTERF family protein, expressed	2,00	-1.0	1	21	33	53	UUGAACAUCCCAGAGCCACCG	CCGUGCCUCUGGGAUGUUCAG	Cleavage
tae-miR9662b-3p	Traes_6BS_CBD931158.2	PF02536				GO:GO:0006355,GO:GO:0005739,GO:GO:0003690	AT1G21150.1	Mitochondrial transcription termination factor family protein	LOC_Os06g12100.1	mTERF family protein, expressed	2,00	-1.0	1	21	32	52	UGAACAUCCCAGAGCCACCGG	CCCGUGCCUCUGGGAUGUUCA	Cleavage
tae-miR9662a-3p	Traes_6BS_CBD931158.3	PF02536				GO:GO:0006355,GO:GO:0005739,GO:GO:0003690	AT1G21150.1	Mitochondrial transcription termination factor family protein	LOC_Os06g12100.1	mTERF family protein, expressed	2,00	-1.0	1	21	33	53	UUGAACAUCCCAGAGCCACCG	CCGUGCCUCUGGGAUGUUCAG	Cleavage
tae-miR9662b-3p	Traes_6BS_CBD931158.3	PF02536				GO:GO:0006355,GO:GO:0005739,GO:GO:0003690	AT1G21150.1	Mitochondrial transcription termination factor family protein	LOC_Os06g12100.1	mTERF family protein, expressed	2,00	-1.0	1	21	32	52	UGAACAUCCCAGAGCCACCGG	CCCGUGCCUCUGGGAUGUUCA	Cleavage
tae-miR9662a-3p	Traes_6BS_CBD931158.4	PF02536				GO:GO:0006355,GO:GO:0005739,GO:GO:0003690	AT1G21150.1	Mitochondrial transcription termination factor family protein	LOC_Os06g12100.1	mTERF family protein, expressed	2,00	-1.0	1	21	33	53	UUGAACAUCCCAGAGCCACCG	CCGUGCCUCUGGGAUGUUCAG	Cleavage
tae-miR9662b-3p	Traes_6BS_CBD931158.4	PF02536				GO:GO:0006355,GO:GO:0005739,GO:GO:0003690	AT1G21150.1	Mitochondrial transcription termination factor family protein	LOC_Os06g12100.1	mTERF family protein, expressed	2,00	-1.0	1	21	32	52	UGAACAUCCCAGAGCCACCGG	CCCGUGCCUCUGGGAUGUUCA	Cleavage
tae-miR9662a-3p	Traes_6BS_CBD931158.5	PF02536				GO:GO:0006355,GO:GO:0005739,GO:GO:0003690	AT1G21150.1	Mitochondrial transcription termination factor family protein	LOC_Os06g12100.1	mTERF family protein, expressed	2,00	-1.0	1	21	33	53	UUGAACAUCCCAGAGCCACCG	CCGUGCCUCUGGGAUGUUCAG	Cleavage
tae-miR9662b-3p	Traes_6BS_CBD931158.5	PF02536				GO:GO:0006355,GO:GO:0005739,GO:GO:0003690	AT1G21150.1	Mitochondrial transcription termination factor family protein	LOC_Os06g12100.1	mTERF family protein, expressed	2,00	-1.0	1	21	32	52	UGAACAUCCCAGAGCCACCGG	CCCGUGCCUCUGGGAUGUUCA	Cleavage
tae-miR9662a-3p	Traes_6BS_CBD931158.6	PF02536				GO:GO:0006355,GO:GO:0005739,GO:GO:0003690	AT1G21150.1	Mitochondrial transcription termination factor family protein	LOC_Os06g12100.1	mTERF family protein, expressed	2,00	-1.0	1	21	33	53	UUGAACAUCCCAGAGCCACCG	CCGUGCCUCUGGGAUGUUCAG	Cleavage
tae-miR9662b-3p	Traes_6BS_CBD931158.6	PF02536				GO:GO:0006355,GO:GO:0005739,GO:GO:0003690	AT1G21150.1	Mitochondrial transcription termination factor family protein	LOC_Os06g12100.1	mTERF family protein, expressed	2,00	-1.0	1	21	32	52	UGAACAUCCCAGAGCCACCGG	CCCGUGCCUCUGGGAUGUUCA	Cleavage
tae-miR156	Traes_6BS_F50663DF7.1	PF03110				GO:GO:0005634,GO:GO:0003677	AT5G43270.1	squamosa promoter binding protein-like 2	LOC_Os02g04680.2	OsSPL3 - SBP-box gene family member, expressed	1,00	-1.0	1	21	1387	1407	UGACAGAAGAGAGUGAGCACA	CAUGCUCUCUCUCUUCUGUCA	Cleavage
tae-miR9662a-3p	Traes_6BS_F8176B2AA.1	PF02536				GO:GO:0006355,GO:GO:0005739,GO:GO:0003690	AT5G64950.1	Mitochondrial transcription termination factor family protein	LOC_Os06g12100.1	mTERF family protein, expressed	2,05	-1.0	1	21	204	224	UUGAACAUCCCAGAGCCACCG	CCGUGGCUCUGCGAUGUUCAG	Translation
tae-miR9662b-3p	Traes_6BS_F8176B2AA.1	PF02536				GO:GO:0006355,GO:GO:0005739,GO:GO:0003690	AT5G64950.1	Mitochondrial transcription termination factor family protein	LOC_Os06g12100.1	mTERF family protein, expressed	2,05	-1.0	1	21	203	223	UGAACAUCCCAGAGCCACCGG	CCCGUGGCUCUGCGAUGUUCA	Cleavage
tae-miR9662a-3p	Traes_6BS_F8176B2AA.2	PF02536				GO:GO:0006355,GO:GO:0005739,GO:GO:0003690	AT5G64950.1	Mitochondrial transcription termination factor family protein	LOC_Os06g12100.1	mTERF family protein, expressed	2,05	-1.0	1	21	204	224	UUGAACAUCCCAGAGCCACCG	CCGUGGCUCUGCGAUGUUCAG	Translation
tae-miR9662b-3p	Traes_6BS_F8176B2AA.2	PF02536				GO:GO:0006355,GO:GO:0005739,GO:GO:0003690	AT5G64950.1	Mitochondrial transcription termination factor family protein	LOC_Os06g12100.1	mTERF family protein, expressed	2,05	-1.0	1	21	203	223	UGAACAUCCCAGAGCCACCGG	CCCGUGGCUCUGCGAUGUUCA	Cleavage
tae-miR9662a-3p	Traes_6BS_F8176B2AA.3	PF02536				GO:GO:0006355,GO:GO:0005739,GO:GO:0003690	AT5G64950.1	Mitochondrial transcription termination factor family protein	LOC_Os06g12100.1	mTERF family protein, expressed	2,05	-1.0	1	21	198	218	UUGAACAUCCCAGAGCCACCG	CCGUGGCUCUGCGAUGUUCAG	Translation
tae-miR9662b-3p	Traes_6BS_F8176B2AA.3	PF02536				GO:GO:0006355,GO:GO:0005739,GO:GO:0003690	AT5G64950.1	Mitochondrial transcription termination factor family protein	LOC_Os06g12100.1	mTERF family protein, expressed	2,05	-1.0	1	21	197	217	UGAACAUCCCAGAGCCACCGG	CCCGUGGCUCUGCGAUGUUCA	Cleavage
tae-miR5049-3p	Traes_6DL_1E6A51BD3.10	PF00063		3.6.4.1		GO:GO:0016459,GO:GO:0005524,GO:GO:0003774	AT1G04600.1	myosin XI A	LOC_Os03g64290.1	myosin, putative, expressed	1,05	-1.0	1	21	1321	1341	AAUAUGGAUCGGAGGGAGUAC	AUACUCCCUCCGAUCCAUAAU	Cleavage
tae-miR1122b-3p	Traes_6DL_244C908B5.14	PF12171					AT2G02070.1	indeterminate(ID)-domain 5	LOC_Os04g47860.4	ZOS4-11 - C2H2 zinc finger protein, expressed	1,05	-1.0	1	21	584	604	AGACUUAUAUGUAGGAACGGA	UCCGUUCCUAAAUAUAAGUCU	Translation
tae-miR5384-3p	Traes_6DL_5282E0007.1	PF14541	KOG1339	3.4.23.12		GO:GO:0006508,GO:GO:0004190	AT5G10770.1	Eukaryotic aspartyl protease family protein	LOC_Os02g48870.1	aspartic proteinase nepenthesin-2 precursor, putative, expressed	2,05	-1.0	1	21	1178	1198	UGAGCGCGCCGCCGUCGAAUG	UGUUCGACGGCGGCGCGGUCG	Cleavage
tae-miR5384-3p	Traes_6DL_5282E0007.2	PF14541,PF14543	KOG1339	3.4.23.12		GO:GO:0006508,GO:GO:0004190	AT5G10770.1	Eukaryotic aspartyl protease family protein	LOC_Os02g48870.1	aspartic proteinase nepenthesin-2 precursor, putative, expressed	2,05	-1.0	1	21	1016	1036	UGAGCGCGCCGCCGUCGAAUG	UGUUCGACGGCGGCGCGGUCG	Cleavage
tae-miR1122b-3p	Traes_6DL_84E14AD8D.4	PF00067				GO:GO:0055114,GO:GO:0020037,GO:GO:0016705,GO:GO:0005506	AT1G31800.1	cytochrome P450, family 97, subfamily A, polypeptide 3	LOC_Os02g57290.4	cytochrome P450, putative, expressed	1,05	-1.0	1	21	1030	1050	AGACUUAUAUGUAGGAACGGA	UCCGUUCCUAAAUAUAAGUCU	Translation
tae-miR1122a	Traes_6DL_84E14AD8D.4	PF00067				GO:GO:0055114,GO:GO:0020037,GO:GO:0016705,GO:GO:0005506	AT1G31800.1	cytochrome P450, family 97, subfamily A, polypeptide 3	LOC_Os02g57290.4	cytochrome P450, putative, expressed	2,05	-1.0	1	20	1071	1090	UAGAUACAUCCGUAUCUAGA	ACUACAUACGGAUGUAUAUA	Cleavage
tae-miR1118	Traes_6DL_C9216369C.2	PF00891				GO:GO:0008171	AT5G54160.1	O-methyltransferase 1	LOC_Os11g20090.1	O-methyltransferase, putative, expressed	2,05	-1.0	1	23	421	443	CACUACAUUAUGGAAUGGAGGGA	UUGCUCCGUUUCAUAAUGUAGUG	Cleavage
tae-miR444b	Traes_6DL_D1C1DBD34.1	PF00319,PF01486	KOG0014			GO:GO:0046983,GO:GO:0003677,GO:GO:0006355,GO:GO:0005634,GO:GO:0003700	AT3G57230.1	AGAMOUS-like 16	LOC_Os02g49840.1	OsMADS57 - MADS-box family gene with MIKCc type-box, expressed	0,00	-1.0	1	21	274	294	UUGCUGCCUCAAGCUUGCUGC	GCAGCAAGCUUGAGGCAGCAA	Cleavage
tae-miR444a	Traes_6DL_D1C1DBD34.1	PF00319,PF01486	KOG0014			GO:GO:0046983,GO:GO:0003677,GO:GO:0006355,GO:GO:0005634,GO:GO:0003700	AT3G57230.1	AGAMOUS-like 16	LOC_Os02g49840.1	OsMADS57 - MADS-box family gene with MIKCc type-box, expressed	0,00	-1.0	1	21	274	294	UUGCUGCCUCAAGCUUGCUGC	GCAGCAAGCUUGAGGCAGCAA	Cleavage
tae-miR1117	Traes_6DL_E940A7985.1	PF00854		3.6.3.43		GO:GO:0016020,GO:GO:0006810,GO:GO:0005215	AT1G62200.1	Major facilitator superfamily protein	LOC_Os04g50940.1	peptide transporter PTR2, putative, expressed	2,05	-1.0	1	24	155	178	UAGUACCGGUUCGUGGCACGAACC	GGUUCGUGGCACAAACCGGUACUA	Cleavage
tae-miR1131	Traes_6DL_E940A7985.1	PF00854		3.6.3.43		GO:GO:0016020,GO:GO:0006810,GO:GO:0005215	AT1G62200.1	Major facilitator superfamily protein	LOC_Os04g50940.1	peptide transporter PTR2, putative, expressed	1,00	-1.0	1	22	181	202	UAGUACCGGUUCGUGGCUAACC	GAUUCGCCACGAACCGGUACUA	Cleavage
tae-miR1122a	Traes_6DS_0D9BB546D.1	PF00300		3.1.3.3	K15634		AT3G50520.1	Phosphoglycerate mutase family protein	LOC_Os08g37140.1	phosphoglycerate mutase, putative, expressed	2,05	-1.0	1	20	111	130	UAGAUACAUCCGUAUCUAGA	ACUACAUACGGAUGUAUAUA	Cleavage
tae-miR9662a-3p	Traes_6DS_0E84AAF77.1	PF02536				GO:GO:0006355,GO:GO:0005739,GO:GO:0003690	AT5G07900.1	Mitochondrial transcription termination factor family protein	LOC_Os06g12100.1	mTERF family protein, expressed	2,05	-1.0	1	21	480	500	UUGAACAUCCCAGAGCCACCG	CCGUGGCUCUGGGAUGCUCAG	Cleavage
tae-miR9662b-3p	Traes_6DS_0E84AAF77.1	PF02536				GO:GO:0006355,GO:GO:0005739,GO:GO:0003690	AT5G07900.1	Mitochondrial transcription termination factor family protein	LOC_Os06g12100.1	mTERF family protein, expressed	2,05	-1.0	1	21	479	499	UGAACAUCCCAGAGCCACCGG	CCCGUGGCUCUGGGAUGCUCA	Cleavage
tae-miR9674b-5p	Traes_6DS_1BEF46735.1	PF13041					AT1G63130.1	Tetratricopeptide repeat (TPR)-like superfamily protein	LOC_Os10g35240.1	Rf1, mitochondrial precursor, putative, expressed	3,00	-1.0	1	21	537	557	AUAGCAUCAUCCAUCCUACCC	GGGCCGAAUGGAUGAUGCUAU	Cleavage
tae-miR1127b-3p	Traes_6DS_2207A3BC4.7	PF14438,PF06741	KOG2375				AT3G14010.4	CTC-interacting domain 4	LOC_Os02g27950.2	ataxin-2 related protein, putative, expressed	1,05	-1.0	1	21	1729	1749	ACAAGUAUUUCUGGACGGAGG	CCUCCGUCCGGAAAUACUUGU	Cleavage
tae-miR9662a-3p	Traes_6DS_2D4EFFF3E.1	PF02536				GO:GO:0006355,GO:GO:0005739,GO:GO:0003690	AT5G07900.1	Mitochondrial transcription termination factor family protein	LOC_Os06g12100.1	mTERF family protein, expressed	1,00	-1.0	1	21	369	389	UUGAACAUCCCAGAGCCACCG	CCGUGGCUCUGGGAUGUUCAG	Cleavage
tae-miR9662b-3p	Traes_6DS_2D4EFFF3E.1	PF02536				GO:GO:0006355,GO:GO:0005739,GO:GO:0003690	AT5G07900.1	Mitochondrial transcription termination factor family protein	LOC_Os06g12100.1	mTERF family protein, expressed	1,00	-1.0	1	21	368	388	UGAACAUCCCAGAGCCACCGG	CCCGUGGCUCUGGGAUGUUCA	Cleavage
tae-miR9662a-3p	Traes_6DS_3F194116E.1	PF02536				GO:GO:0006355,GO:GO:0005739,GO:GO:0003690	AT5G07900.1	Mitochondrial transcription termination factor family protein	LOC_Os06g12100.1	mTERF family protein, expressed	1,05	-1.0	1	21	525	545	UUGAACAUCCCAGAGCCACCG	CCGUGGCUCGGGGAUGUUCAA	Cleavage
tae-miR9662b-3p	Traes_6DS_3F194116E.1	PF02536				GO:GO:0006355,GO:GO:0005739,GO:GO:0003690	AT5G07900.1	Mitochondrial transcription termination factor family protein	LOC_Os06g12100.1	mTERF family protein, expressed	2,05	-1.0	1	21	524	544	UGAACAUCCCAGAGCCACCGG	CCCGUGGCUCGGGGAUGUUCA	Translation
tae-miR9662a-3p	Traes_6DS_3FC2ADA87.1	PF02536				GO:GO:0006355,GO:GO:0005739,GO:GO:0003690	AT5G07900.1	Mitochondrial transcription termination factor family protein	LOC_Os06g12100.1	mTERF family protein, expressed	3,00	-1.0	1	21	242	263	UUGAACAUCCCAGAGCCAC-CG	CGCGUGGCUCUGGGAUGUUCAG	Cleavage
tae-miR9662b-3p	Traes_6DS_3FC2ADA87.1	PF02536				GO:GO:0006355,GO:GO:0005739,GO:GO:0003690	AT5G07900.1	Mitochondrial transcription termination factor family protein	LOC_Os06g12100.1	mTERF family protein, expressed	2,00	-1.0	1	21	241	262	UGAACAUCCCAGAGCCAC-CGG	CCGCGUGGCUCUGGGAUGUUCA	Cleavage
tae-miR9662a-3p	Traes_6DS_3FC2ADA87.2	PF02536				GO:GO:0006355,GO:GO:0005739,GO:GO:0003690	AT1G21150.1	Mitochondrial transcription termination factor family protein	LOC_Os06g12100.1	mTERF family protein, expressed	3,00	-1.0	1	21	65	86	UUGAACAUCCCAGAGCCAC-CG	CGCGUGGCUCUGGGAUGUUCAG	Cleavage
tae-miR9662b-3p	Traes_6DS_3FC2ADA87.2	PF02536				GO:GO:0006355,GO:GO:0005739,GO:GO:0003690	AT1G21150.1	Mitochondrial transcription termination factor family protein	LOC_Os06g12100.1	mTERF family protein, expressed	2,00	-1.0	1	21	64	85	UGAACAUCCCAGAGCCAC-CGG	CCGCGUGGCUCUGGGAUGUUCA	Cleavage
tae-miR9662a-3p	Traes_6DS_422644406.1	PF02536				GO:GO:0006355,GO:GO:0005739,GO:GO:0003690	AT5G07900.1	Mitochondrial transcription termination factor family protein	LOC_Os06g12100.1	mTERF family protein, expressed	2,05	-1.0	1	21	468	488	UUGAACAUCCCAGAGCCACCG	CCGUGGCUCUGGAAUGUUCAG	Cleavage
tae-miR9662b-3p	Traes_6DS_422644406.1	PF02536				GO:GO:0006355,GO:GO:0005739,GO:GO:0003690	AT5G07900.1	Mitochondrial transcription termination factor family protein	LOC_Os06g12100.1	mTERF family protein, expressed	2,05	-1.0	1	21	467	487	UGAACAUCCCAGAGCCACCGG	CCCGUGGCUCUGGAAUGUUCA	Cleavage
tae-miR9662a-3p	Traes_6DS_422644406.2	PF02536				GO:GO:0006355,GO:GO:0005739,GO:GO:0003690	AT5G07900.1	Mitochondrial transcription termination factor family protein	LOC_Os06g12100.1	mTERF family protein, expressed	2,05	-1.0	1	21	66	86	UUGAACAUCCCAGAGCCACCG	CCGUGGCUCUGGAAUGUUCAG	Cleavage
tae-miR9662b-3p	Traes_6DS_422644406.2	PF02536				GO:GO:0006355,GO:GO:0005739,GO:GO:0003690	AT5G07900.1	Mitochondrial transcription termination factor family protein	LOC_Os06g12100.1	mTERF family protein, expressed	2,05	-1.0	1	21	65	85	UGAACAUCCCAGAGCCACCGG	CCCGUGGCUCUGGAAUGUUCA	Cleavage
tae-miR9662a-3p	Traes_6DS_422644406.3	PF02536				GO:GO:0006355,GO:GO:0005739,GO:GO:0003690	AT5G07900.1	Mitochondrial transcription termination factor family protein	LOC_Os06g12100.1	mTERF family protein, expressed	2,05	-1.0	1	21	66	86	UUGAACAUCCCAGAGCCACCG	CCGUGGCUCUGGAAUGUUCAG	Cleavage
tae-miR9662b-3p	Traes_6DS_422644406.3	PF02536				GO:GO:0006355,GO:GO:0005739,GO:GO:0003690	AT5G07900.1	Mitochondrial transcription termination factor family protein	LOC_Os06g12100.1	mTERF family protein, expressed	2,05	-1.0	1	21	65	85	UGAACAUCCCAGAGCCACCGG	CCCGUGGCUCUGGAAUGUUCA	Cleavage
tae-miR9662a-3p	Traes_6DS_422644406.4	PF02536				GO:GO:0006355,GO:GO:0005739,GO:GO:0003690	AT5G07900.1	Mitochondrial transcription termination factor family protein	LOC_Os06g12100.1	mTERF family protein, expressed	2,05	-1.0	1	21	66	86	UUGAACAUCCCAGAGCCACCG	CCGUGGCUCUGGAAUGUUCAG	Cleavage
tae-miR9662b-3p	Traes_6DS_422644406.4	PF02536				GO:GO:0006355,GO:GO:0005739,GO:GO:0003690	AT5G07900.1	Mitochondrial transcription termination factor family protein	LOC_Os06g12100.1	mTERF family protein, expressed	2,05	-1.0	1	21	65	85	UGAACAUCCCAGAGCCACCGG	CCCGUGGCUCUGGAAUGUUCA	Cleavage
tae-miR156	Traes_6DS_7B1596298.1	PF03110				GO:GO:0005634,GO:GO:0003677	AT5G43270.2	squamosa promoter binding protein-like 2	LOC_Os02g04680.2	OsSPL3 - SBP-box gene family member, expressed	1,00	-1.0	1	21	1150	1170	UGACAGAAGAGAGUGAGCACA	CAUGCUCUCUCUCUUCUGUCA	Cleavage
tae-miR156	Traes_6DS_7B1596298.2	PF03110				GO:GO:0005634,GO:GO:0003677	AT5G43270.1	squamosa promoter binding protein-like 2	LOC_Os02g04680.2	OsSPL3 - SBP-box gene family member, expressed	1,00	-1.0	1	21	1150	1170	UGACAGAAGAGAGUGAGCACA	CAUGCUCUCUCUCUUCUGUCA	Cleavage
tae-miR156	Traes_6DS_7B1596298.4								LOC_Os02g04680.1	OsSPL3 - SBP-box gene family member, expressed	1,00	-1.0	1	21	271	291	UGACAGAAGAGAGUGAGCACA	CAUGCUCUCUCUCUUCUGUCA	Cleavage
tae-miR9674b-5p	Traes_6DS_7C389C178.1	PF12854,PF13041					AT1G62670.1	rna processing factor 2	LOC_Os10g35240.2	Rf1, mitochondrial precursor, putative, expressed	3,00	-1.0	1	21	537	557	AUAGCAUCAUCCAUCCUACCC	GGGCCGAAUGGAUGAUGCUAU	Cleavage
tae-miR9674a-5p	Traes_6DS_7C389C178.1	PF12854,PF13041					AT1G62670.1	rna processing factor 2	LOC_Os10g35240.2	Rf1, mitochondrial precursor, putative, expressed	2,05	-1.0	1	21	954	974	GCAUCAUCCAUCCUACCAUUC	GAAUGGAAGGAUAGAUGAUGC	Cleavage
tae-miR9674a-5p	Traes_6DS_7C389C178.2	PF12854					AT1G62670.1	rna processing factor 2	LOC_Os10g35436.1	Rf1, mitochondrial precursor, putative, expressed	2,05	-1.0	1	21	51	71	GCAUCAUCCAUCCUACCAUUC	GAAUGGAAGGAUAGAUGAUGC	Cleavage
tae-miR9674b-5p	Traes_6DS_7C389C178.3	PF12854,PF13041					AT1G62670.1	rna processing factor 2	LOC_Os10g35240.1	Rf1, mitochondrial precursor, putative, expressed	3,00	-1.0	1	21	537	557	AUAGCAUCAUCCAUCCUACCC	GGGCCGAAUGGAUGAUGCUAU	Cleavage
tae-miR9674a-5p	Traes_6DS_7C389C178.3	PF12854,PF13041					AT1G62670.1	rna processing factor 2	LOC_Os10g35240.1	Rf1, mitochondrial precursor, putative, expressed	2,05	-1.0	1	21	954	974	GCAUCAUCCAUCCUACCAUUC	GAAUGGAAGGAUAGAUGAUGC	Cleavage
tae-miR9662a-3p	Traes_6DS_7D3BEF6A9.1	PF02536				GO:GO:0006355,GO:GO:0005739,GO:GO:0003690	AT1G21150.1	Mitochondrial transcription termination factor family protein	LOC_Os06g12100.1	mTERF family protein, expressed	2,05	-1.0	1	21	33	53	UUGAACAUCCCAGAGCCACCG	UCGUGGCUCGGGGAUGUUCAG	Cleavage
tae-miR9662b-3p	Traes_6DS_7D3BEF6A9.1	PF02536				GO:GO:0006355,GO:GO:0005739,GO:GO:0003690	AT1G21150.1	Mitochondrial transcription termination factor family protein	LOC_Os06g12100.1	mTERF family protein, expressed	2,05	-1.0	1	21	32	52	UGAACAUCCCAGAGCCACCGG	CUCGUGGCUCGGGGAUGUUCA	Translation
tae-miR9662a-3p	Traes_6DS_7D3BEF6A9.2	PF02536				GO:GO:0006355,GO:GO:0005739,GO:GO:0003690	AT1G21150.1	Mitochondrial transcription termination factor family protein	LOC_Os06g12100.1	mTERF family protein, expressed	2,05	-1.0	1	21	246	266	UUGAACAUCCCAGAGCCACCG	UCGUGGCUCGGGGAUGUUCAG	Cleavage
tae-miR9662b-3p	Traes_6DS_7D3BEF6A9.2	PF02536				GO:GO:0006355,GO:GO:0005739,GO:GO:0003690	AT1G21150.1	Mitochondrial transcription termination factor family protein	LOC_Os06g12100.1	mTERF family protein, expressed	2,05	-1.0	1	21	245	265	UGAACAUCCCAGAGCCACCGG	CUCGUGGCUCGGGGAUGUUCA	Translation
tae-miR9662a-3p	Traes_6DS_7D3BEF6A9.3	PF02536				GO:GO:0006355,GO:GO:0005739,GO:GO:0003690	AT1G21150.1	Mitochondrial transcription termination factor family protein	LOC_Os06g12100.1	mTERF family protein, expressed	2,05	-1.0	1	21	246	266	UUGAACAUCCCAGAGCCACCG	UCGUGGCUCGGGGAUGUUCAG	Cleavage
tae-miR9662b-3p	Traes_6DS_7D3BEF6A9.3	PF02536				GO:GO:0006355,GO:GO:0005739,GO:GO:0003690	AT1G21150.1	Mitochondrial transcription termination factor family protein	LOC_Os06g12100.1	mTERF family protein, expressed	2,05	-1.0	1	21	245	265	UGAACAUCCCAGAGCCACCGG	CUCGUGGCUCGGGGAUGUUCA	Translation
tae-miR9662a-3p	Traes_6DS_893F318A9.1	PF02536				GO:GO:0006355,GO:GO:0005739,GO:GO:0003690	AT5G07900.1	Mitochondrial transcription termination factor family protein	LOC_Os06g12100.1	mTERF family protein, expressed	1,05	-1.0	1	21	549	569	UUGAACAUCCCAGAGCCACCG	CCGUGGCUCGGGGAUGUUCAA	Cleavage
tae-miR9662b-3p	Traes_6DS_893F318A9.1	PF02536				GO:GO:0006355,GO:GO:0005739,GO:GO:0003690	AT5G07900.1	Mitochondrial transcription termination factor family protein	LOC_Os06g12100.1	mTERF family protein, expressed	2,05	-1.0	1	21	548	568	UGAACAUCCCAGAGCCACCGG	CCCGUGGCUCGGGGAUGUUCA	Translation
tae-miR9662a-3p	Traes_6DS_893F318A9.2	PF02536				GO:GO:0006355,GO:GO:0005739,GO:GO:0003690	AT5G07900.1	Mitochondrial transcription termination factor family protein	LOC_Os06g12100.1	mTERF family protein, expressed	1,05	-1.0	1	21	132	152	UUGAACAUCCCAGAGCCACCG	CCGUGGCUCGGGGAUGUUCAA	Cleavage
tae-miR9662b-3p	Traes_6DS_893F318A9.2	PF02536				GO:GO:0006355,GO:GO:0005739,GO:GO:0003690	AT5G07900.1	Mitochondrial transcription termination factor family protein	LOC_Os06g12100.1	mTERF family protein, expressed	2,05	-1.0	1	21	131	151	UGAACAUCCCAGAGCCACCGG	CCCGUGGCUCGGGGAUGUUCA	Translation
tae-miR9662a-3p	Traes_6DS_931C28AF6.1	PF02536				GO:GO:0006355,GO:GO:0005739,GO:GO:0003690	AT1G79220.1	Mitochondrial transcription termination factor family protein	LOC_Os06g12100.1	mTERF family protein, expressed	2,05	-1.0	1	21	288	308	UUGAACAUCCCAGAGCCACCG	AUGUGGCUCUCGGAUGUUCAG	Translation
tae-miR9662a-3p	Traes_6DS_931C28AF6.2	PF02536				GO:GO:0006355,GO:GO:0005739,GO:GO:0003690	AT1G79220.1	Mitochondrial transcription termination factor family protein	LOC_Os06g12100.1	mTERF family protein, expressed	2,05	-1.0	1	21	276	296	UUGAACAUCCCAGAGCCACCG	AUGUGGCUCUCGGAUGUUCAG	Translation
tae-miR9662a-3p	Traes_6DS_931C28AF6.3	PF02536				GO:GO:0006355,GO:GO:0005739,GO:GO:0003690	AT1G79220.1	Mitochondrial transcription termination factor family protein	LOC_Os06g12100.1	mTERF family protein, expressed	2,05	-1.0	1	21	273	293	UUGAACAUCCCAGAGCCACCG	AUGUGGCUCUCGGAUGUUCAG	Translation
tae-miR9662a-3p	Traes_6DS_931C28AF6.4	PF02536				GO:GO:0006355,GO:GO:0005739,GO:GO:0003690	AT1G79220.1	Mitochondrial transcription termination factor family protein	LOC_Os06g12100.1	mTERF family protein, expressed	2,05	-1.0	1	21	273	293	UUGAACAUCCCAGAGCCACCG	AUGUGGCUCUCGGAUGUUCAG	Translation
tae-miR9662a-3p	Traes_6DS_931C28AF6.5	PF02536				GO:GO:0006355,GO:GO:0005739,GO:GO:0003690	AT1G79220.1	Mitochondrial transcription termination factor family protein	LOC_Os06g12100.1	mTERF family protein, expressed	2,05	-1.0	1	21	276	296	UUGAACAUCCCAGAGCCACCG	AUGUGGCUCUCGGAUGUUCAG	Translation
tae-miR9662b-3p	Traes_6DS_93C7DA226.1	PF02536				GO:GO:0006355,GO:GO:0005739,GO:GO:0003690	AT5G07900.1	Mitochondrial transcription termination factor family protein	LOC_Os06g12100.1	mTERF family protein, expressed	3,00	-1.0	1	21	503	523	UGAACAUCCCAGAGCCACCGG	CCCGUGCUUCUGGGAUGUUCA	Cleavage
tae-miR9662b-3p	Traes_6DS_93C7DA2261.1	PF02536				GO:GO:0006355,GO:GO:0005739,GO:GO:0003690	AT5G07900.1	Mitochondrial transcription termination factor family protein	LOC_Os06g12100.1	mTERF family protein, expressed	3,00	-1.0	1	21	503	523	UGAACAUCCCAGAGCCACCGG	CCCGUGCUUCUGGGAUGUUCA	Cleavage
tae-miR9662a-3p	Traes_6DS_A490DF33E.1	PF02536				GO:GO:0006355,GO:GO:0005739,GO:GO:0003690	AT5G07900.1	Mitochondrial transcription termination factor family protein	LOC_Os06g12100.1	mTERF family protein, expressed	2,05	-1.0	1	21	471	491	UUGAACAUCCCAGAGCCACCG	CCGUGGCUCUGGAAUGUUCAG	Cleavage
tae-miR9662b-3p	Traes_6DS_A490DF33E.1	PF02536				GO:GO:0006355,GO:GO:0005739,GO:GO:0003690	AT5G07900.1	Mitochondrial transcription termination factor family protein	LOC_Os06g12100.1	mTERF family protein, expressed	2,05	-1.0	1	21	470	490	UGAACAUCCCAGAGCCACCGG	CCCGUGGCUCUGGAAUGUUCA	Cleavage
tae-miR9662a-3p	Traes_6DS_A490DF33E.2	PF02536				GO:GO:0006355,GO:GO:0005739,GO:GO:0003690	AT1G21150.1	Mitochondrial transcription termination factor family protein	LOC_Os06g12100.1	mTERF family protein, expressed	2,05	-1.0	1	21	66	86	UUGAACAUCCCAGAGCCACCG	CCGUGGCUCUGGAAUGUUCAG	Cleavage
tae-miR9662b-3p	Traes_6DS_A490DF33E.2	PF02536				GO:GO:0006355,GO:GO:0005739,GO:GO:0003690	AT1G21150.1	Mitochondrial transcription termination factor family protein	LOC_Os06g12100.1	mTERF family protein, expressed	2,05	-1.0	1	21	65	85	UGAACAUCCCAGAGCCACCGG	CCCGUGGCUCUGGAAUGUUCA	Cleavage
tae-miR1122b-3p	Traes_6DS_C1B7F14A41.2	PF08263,PF07714,PF13855	KOG1187	2.7.11.1		GO:GO:0006468,GO:GO:0004672,GO:GO:0005515	AT1G72300.1	Leucine-rich receptor-like protein kinase family protein	LOC_Os02g05930.1	phytosulfokine receptor precursor, putative, expressed	1,05	-1.0	1	21	3044	3064	AGACUUAUAUGUAGGAACGGA	UCCGUUCCUAAAUAUAAGUCU	Translation
tae-miR156	Traes_6DS_C4D93239C.1	PF03110				GO:GO:0005634,GO:GO:0003677	AT5G43270.1	squamosa promoter binding protein-like 2	LOC_Os06g45310.1	OsSPL11 - SBP-box gene family member, expressed	1,00	-1.0	1	21	577	597	UGACAGAAGAGAGUGAGCACA	CAUGCUCUCUCUCUUCUGUCA	Cleavage
tae-miR156	Traes_6DS_C4D93239C.2								LOC_Os06g45310.1	OsSPL11 - SBP-box gene family member, expressed	1,00	-1.0	1	21	88	108	UGACAGAAGAGAGUGAGCACA	CAUGCUCUCUCUCUUCUGUCA	Cleavage
tae-miR156	Traes_6DS_C4D93239C.3								LOC_Os06g45310.1	OsSPL11 - SBP-box gene family member, expressed	1,00	-1.0	1	21	64	84	UGACAGAAGAGAGUGAGCACA	CAUGCUCUCUCUCUUCUGUCA	Cleavage
tae-miR9662a-3p	Traes_6DS_CF627A1BC.1								LOC_Os06g12100.1	mTERF family protein, expressed	2,05	-1.0	1	21	75	95	UUGAACAUCCCAGAGCCACCG	CCGUGGCUCGGGGAUGUUCAG	Cleavage
tae-miR9662b-3p	Traes_6DS_CF627A1BC.1								LOC_Os06g12100.1	mTERF family protein, expressed	2,05	-1.0	1	21	74	94	UGAACAUCCCAGAGCCACCGG	CCCGUGGCUCGGGGAUGUUCA	Translation
tae-miR9662a-3p	Traes_6DS_DAD8BEF41.1	PF02536				GO:GO:0006355,GO:GO:0005739,GO:GO:0003690	AT5G07900.1	Mitochondrial transcription termination factor family protein	LOC_Os06g12100.1	mTERF family protein, expressed	2,05	-1.0	1	21	477	497	UUGAACAUCCCAGAGCCACCG	CCGUGGCUCUGGAAUGUUCAG	Cleavage
tae-miR9662b-3p	Traes_6DS_DAD8BEF41.1	PF02536				GO:GO:0006355,GO:GO:0005739,GO:GO:0003690	AT5G07900.1	Mitochondrial transcription termination factor family protein	LOC_Os06g12100.1	mTERF family protein, expressed	2,05	-1.0	1	21	476	496	UGAACAUCCCAGAGCCACCGG	CCCGUGGCUCUGGAAUGUUCA	Cleavage
tae-miR9662a-3p	Traes_6DS_E352D66A4.1	PF02536				GO:GO:0006355,GO:GO:0005739,GO:GO:0003690	AT5G64950.1	Mitochondrial transcription termination factor family protein	LOC_Os06g12100.1	mTERF family protein, expressed	2,05	-1.0	1	21	450	470	UUGAACAUCCCAGAGCCACCG	CCGUGGCUCUGGGAUGUUUAG	Cleavage
tae-miR9662b-3p	Traes_6DS_E352D66A4.1	PF02536				GO:GO:0006355,GO:GO:0005739,GO:GO:0003690	AT5G64950.1	Mitochondrial transcription termination factor family protein	LOC_Os06g12100.1	mTERF family protein, expressed	2,05	-1.0	1	21	449	469	UGAACAUCCCAGAGCCACCGG	CCCGUGGCUCUGGGAUGUUUA	Cleavage
tae-miR9662a-3p	Traes_6DS_E95930B7D.1	PF02536				GO:GO:0006355,GO:GO:0005739,GO:GO:0003690	AT5G64950.1	Mitochondrial transcription termination factor family protein	LOC_Os06g12100.1	mTERF family protein, expressed	2,05	-1.0	1	21	450	470	UUGAACAUCCCAGAGCCACCG	CCGUGGCUCUGGGAUGUUUAG	Cleavage
tae-miR9662b-3p	Traes_6DS_E95930B7D.1	PF02536				GO:GO:0006355,GO:GO:0005739,GO:GO:0003690	AT5G64950.1	Mitochondrial transcription termination factor family protein	LOC_Os06g12100.1	mTERF family protein, expressed	2,05	-1.0	1	21	449	469	UGAACAUCCCAGAGCCACCGG	CCCGUGGCUCUGGGAUGUUUA	Cleavage
tae-miR9662a-3p	Traes_6DS_EF75D5C67.1	PF02536				GO:GO:0006355,GO:GO:0005739,GO:GO:0003690	AT5G07900.1	Mitochondrial transcription termination factor family protein	LOC_Os06g12100.1	mTERF family protein, expressed	2,00	-1.0	1	21	66	86	UUGAACAUCCCAGAGCCACCG	UCGUGGCGCUGGGAUGUUCAG	Cleavage
tae-miR9662b-3p	Traes_6DS_EF75D5C67.1	PF02536				GO:GO:0006355,GO:GO:0005739,GO:GO:0003690	AT5G07900.1	Mitochondrial transcription termination factor family protein	LOC_Os06g12100.1	mTERF family protein, expressed	2,05	-1.0	1	21	65	85	UGAACAUCCCAGAGCCACCGG	CUCGUGGCGCUGGGAUGUUCA	Cleavage
tae-miR9662a-3p	Traes_6DS_F6C7E588F.1	PF02536	KOG1267			GO:GO:0006355,GO:GO:0005739,GO:GO:0003690	AT5G07900.1	Mitochondrial transcription termination factor family protein	LOC_Os06g12100.1	mTERF family protein, expressed	2,00	-1.0	1	21	657	677	UUGAACAUCCCAGAGCCACCG	CCGUGGUUCUGGGAUGUUCAG	Cleavage
tae-miR9662b-3p	Traes_6DS_F6C7E588F.1	PF02536	KOG1267			GO:GO:0006355,GO:GO:0005739,GO:GO:0003690	AT5G07900.1	Mitochondrial transcription termination factor family protein	LOC_Os06g12100.1	mTERF family protein, expressed	2,00	-1.0	1	21	656	676	UGAACAUCCCAGAGCCACCGG	CCCGUGGUUCUGGGAUGUUCA	Cleavage
tae-miR9662a-3p	Traes_6DS_FB434BB3C.1	PF02536				GO:GO:0006355,GO:GO:0005739,GO:GO:0003690	AT5G64950.1	Mitochondrial transcription termination factor family protein	LOC_Os06g12100.1	mTERF family protein, expressed	2,05	-1.0	1	21	246	266	UUGAACAUCCCAGAGCCACCG	CCGUGGCUCUCGGAUGUUCAG	Translation
tae-miR9662b-3p	Traes_6DS_FB434BB3C.1	PF02536				GO:GO:0006355,GO:GO:0005739,GO:GO:0003690	AT5G64950.1	Mitochondrial transcription termination factor family protein	LOC_Os06g12100.1	mTERF family protein, expressed	2,05	-1.0	1	21	245	265	UGAACAUCCCAGAGCCACCGG	CCCGUGGCUCUCGGAUGUUCA	Translation
tae-miR9662a-3p	Traes_6DS_FB434BB3C.2	PF02536				GO:GO:0006355,GO:GO:0005739,GO:GO:0003690	AT5G64950.1	Mitochondrial transcription termination factor family protein	LOC_Os06g12100.1	mTERF family protein, expressed	2,05	-1.0	1	21	246	266	UUGAACAUCCCAGAGCCACCG	CCGUGGCUCUCGGAUGUUCAG	Translation
tae-miR9662b-3p	Traes_6DS_FB434BB3C.2	PF02536				GO:GO:0006355,GO:GO:0005739,GO:GO:0003690	AT5G64950.1	Mitochondrial transcription termination factor family protein	LOC_Os06g12100.1	mTERF family protein, expressed	2,05	-1.0	1	21	245	265	UGAACAUCCCAGAGCCACCGG	CCCGUGGCUCUCGGAUGUUCA	Translation
tae-miR1122a	Traes_7AL_135129885.3	PF01138	KOG1612				AT3G07750.1	3\'-5\'-exoribonuclease family protein	LOC_Os12g21500.1	exosome complex exonuclease, putative, expressed	2,05	-1.0	1	20	721	740	UAGAUACAUCCGUAUCUAGA	ACUACAUACGGAUGUAUAUA	Cleavage
tae-miR164	Traes_7AL_628A69311.1	PF02365				GO:GO:0006355,GO:GO:0003677	AT1G56010.2	NAC domain containing protein 1	LOC_Os06g46270.1	no apical meristem protein, putative, expressed	1,00	-1.0	1	21	650	670	UGGAGAAGCAGGGCACGUGCA	AGCAAGUGCCCUGCUUCUCCA	Cleavage
tae-miR1130b-3p	Traes_7AL_955F54182.1	PF02705				GO:GO:0071805,GO:GO:0016020,GO:GO:0015079	AT2G30070.1	potassium transporter 1	LOC_Os06g45940.2	potassium transporter, putative, expressed	0,00	-1.0	1	21	1141	1161	UCUUAUAUUAUGGGACGGAGG	CCUCCGUCCCAUAAUAUAAGA	Cleavage
tae-miR1122c-3p	Traes_7AL_955F54182.1	PF02705				GO:GO:0071805,GO:GO:0016020,GO:GO:0015079	AT2G30070.1	potassium transporter 1	LOC_Os06g45940.2	potassium transporter, putative, expressed	1,05	-1.0	1	21	1141	1161	UCUAAUAUUAUGGGACGGAGG	CCUCCGUCCCAUAAUAUAAGA	Cleavage
tae-miR1133	Traes_7AL_ADBB1224B.1	PF08315					AT3G05070.1		LOC_Os06g51420.1	coiled-coil domain-containing protein 12, putative, expressed	2,05	-1.0	1	22	52	73	CAUAUACUCCCUCCGUCCGAAA	UUCCGGACGGAGGGAGUAUAAU	Cleavage
tae-miR164	Traes_7AL_BE3253C8F.1	PF02365				GO:GO:0006355,GO:GO:0003677	AT1G56010.2	NAC domain containing protein 1	LOC_Os06g46270.1	no apical meristem protein, putative, expressed	1,00	-1.0	1	21	653	673	UGGAGAAGCAGGGCACGUGCA	AGCAAGUGCCCUGCUUCUCCA	Cleavage
tae-miR164	Traes_7AL_BE3253C8F.2						AT1G56010.2	NAC domain containing protein 1	LOC_Os06g46270.1	no apical meristem protein, putative, expressed	1,00	-1.0	1	21	215	235	UGGAGAAGCAGGGCACGUGCA	AGCAAGUGCCCUGCUUCUCCA	Cleavage
tae-miR160	Traes_7AL_E3ADC8C38.1	PF06507				GO:GO:0009725,GO:GO:0006355,GO:GO:0005634,GO:GO:0003677	AT2G28350.1	auxin response factor 10	LOC_Os06g47150.2	auxin response factor 18, putative, expressed	0,00	-1.0	1	21	399	419	UGCCUGGCUCCCUGUAUGCCA	AGGCAUACAGGGAGCCAGGCA	Cleavage
tae-miR9666a-3p	Traes_7AL_EE3CD1CF3.10	PF05001		2.7.7.6,2.7.7.48		GO:GO:0006366,GO:GO:0005665,GO:GO:0003677	AT4G35800.1	RNA polymerase II large subunit	LOC_Os05g05860.1	retrotransposon protein, putative, unclassified, expressed	2,00	-1.0	1	22	229	250	CGGUAGGGCUGUAUGAUGGCGA	UCCCCGUCAUACAGCCCUACCU	Cleavage
tae-miR9666a-3p	Traes_7AL_EE3CD1CF3.2	PF05001,PF05000,PF04983,PF04990,PF04992,PF00623,PF04997,PF04998		2.7.7.6	K03006	GO:GO:0006366,GO:GO:0005665,GO:GO:0003677,GO:GO:0006351,GO:GO:0003899	AT4G35800.1	RNA polymerase II large subunit	LOC_Os05g05860.1	retrotransposon protein, putative, unclassified, expressed	2,00	-1.0	1	22	5032	5053	CGGUAGGGCUGUAUGAUGGCGA	UCCCCGUCAUACAGCCCUACCU	Cleavage
tae-miR9666a-3p	Traes_7AL_EE3CD1CF3.3	PF05001,PF05000,PF04983,PF04990,PF04992,PF00623,PF04997,PF04998		2.7.7.6	K03006	GO:GO:0006366,GO:GO:0005665,GO:GO:0003677,GO:GO:0006351,GO:GO:0003899	AT4G35800.1	RNA polymerase II large subunit	LOC_Os05g05860.1	retrotransposon protein, putative, unclassified, expressed	2,00	-1.0	1	22	4948	4969	CGGUAGGGCUGUAUGAUGGCGA	UCCCCGUCAUACAGCCCUACCU	Cleavage
tae-miR9666a-3p	Traes_7AL_EE3CD1CF3.4	PF05001,PF05000,PF04983,PF04990,PF04997,PF04992,PF00623,PF04998		2.7.7.6	K03006	GO:GO:0006366,GO:GO:0005665,GO:GO:0003677,GO:GO:0006351,GO:GO:0003899	AT4G35800.1	RNA polymerase II large subunit	LOC_Os05g05860.1	retrotransposon protein, putative, unclassified, expressed	2,00	-1.0	1	22	4801	4822	CGGUAGGGCUGUAUGAUGGCGA	UCCCCGUCAUACAGCCCUACCU	Cleavage
tae-miR9666a-3p	Traes_7AL_EE3CD1CF3.5	PF05001,PF05000,PF04997,PF04983,PF04990,PF04992,PF00623,PF04998		2.7.7.6		GO:GO:0006366,GO:GO:0005665,GO:GO:0003677,GO:GO:0006351,GO:GO:0003899	AT4G35800.1	RNA polymerase II large subunit	LOC_Os05g05860.1	retrotransposon protein, putative, unclassified, expressed	2,00	-1.0	1	22	4393	4414	CGGUAGGGCUGUAUGAUGGCGA	UCCCCGUCAUACAGCCCUACCU	Cleavage
tae-miR9666a-3p	Traes_7AL_EE3CD1CF3.6	PF05001,PF05000,PF04983,PF04990,PF04992,PF04998		2.7.7.6		GO:GO:0006366,GO:GO:0005665,GO:GO:0003677,GO:GO:0006351,GO:GO:0003899	AT4G35800.1	RNA polymerase II large subunit	LOC_Os05g05860.1	retrotransposon protein, putative, unclassified, expressed	2,00	-1.0	1	22	3409	3430	CGGUAGGGCUGUAUGAUGGCGA	UCCCCGUCAUACAGCCCUACCU	Cleavage
tae-miR9666a-3p	Traes_7AL_EE3CD1CF3.8	PF05001,PF05000,PF04990,PF04992,PF04998		2.7.7.6		GO:GO:0006366,GO:GO:0005665,GO:GO:0003677,GO:GO:0006351,GO:GO:0003899	AT4G35800.1	RNA polymerase II large subunit	LOC_Os05g05860.1	retrotransposon protein, putative, unclassified, expressed	2,00	-1.0	1	22	2941	2962	CGGUAGGGCUGUAUGAUGGCGA	UCCCCGUCAUACAGCCCUACCU	Cleavage
tae-miR1137b-5p	Traes_7AS_0F0291904.1	PF00657		3.1.1.1		GO:GO:0016788	AT5G45910.1	GDSL-like Lipase/Acylhydrolase superfamily protein	LOC_Os01g12320.1	GDSL-like lipase/acylhydrolase, putative, expressed	2,05	-1.0	1	21	844	864	UCCGUUCCAGAAUAGAUGACC	ACUCAUCUAUUCUGGGACGGU	Cleavage
tae-miR1137b-5p	Traes_7AS_0F0291904.2	PF00657		3.1.1.1		GO:GO:0016788	AT2G27360.1	GDSL-like Lipase/Acylhydrolase superfamily protein	LOC_Os01g12320.2	GDSL-like lipase/acylhydrolase, putative, expressed	2,05	-1.0	1	21	451	471	UCCGUUCCAGAAUAGAUGACC	ACUCAUCUAUUCUGGGACGGU	Cleavage
tae-miR1137b-5p	Traes_7AS_23DFA827E.1	PF00400	KOG1188			GO:GO:0005515	AT2G47790.1	Transducin/WD40 repeat-like superfamily protein	LOC_Os12g19590.3	WD domain, G-beta repeat domain containing protein, expressed	1,05	-1.0	1	21	11	31	UCCGUUCCAGAAUAGAUGACC	AGUCAUCUAUUUUGGAACGGA	Translation
tae-miR1122a	Traes_7AS_9674F6981.1	PF01031		3.6.5.5		GO:GO:0005525	AT1G10290.1	dynamin-like protein 6	LOC_Os06g13820.1	dynamin, putative, expressed	1,05	-1.0	1	20	1467	1486	UAGAUACAUCCGUAUCUAGA	UCUAGAUACAGAUGUAUCUA	Translation
tae-miR1122a	Traes_7AS_9674F6981.2	PF01031		3.6.5.5		GO:GO:0005525	AT1G10290.1	dynamin-like protein 6	LOC_Os06g13820.1	dynamin, putative, expressed	1,05	-1.0	1	20	1473	1492	UAGAUACAUCCGUAUCUAGA	UCUAGAUACAGAUGUAUCUA	Translation
tae-miR1122a	Traes_7AS_9674F6981.3	PF01031		3.6.5.5		GO:GO:0005525	AT1G10290.1	dynamin-like protein 6	LOC_Os06g13820.1	dynamin, putative, expressed	1,05	-1.0	1	20	1467	1486	UAGAUACAUCCGUAUCUAGA	UCUAGAUACAGAUGUAUCUA	Translation
tae-miR1130b-3p	Traes_7AS_9819F7C48.1	PF01154,PF08540		2.3.3.10		GO:GO:0008299,GO:GO:0004421	AT4G11820.2	hydroxymethylglutaryl-CoA synthase / HMG-CoA synthase / 3-hydroxy-3-methylglutaryl coenzyme A synthase	LOC_Os08g43170.1	hydroxymethylglutaryl-CoA synthase, putative, expressed	0,00	-1.0	1	21	1268	1288	UCUUAUAUUAUGGGACGGAGG	AAUCCGUCCCAUAAUAUAAGA	Cleavage
tae-miR1122c-3p	Traes_7AS_9819F7C48.1	PF01154,PF08540		2.3.3.10		GO:GO:0008299,GO:GO:0004421	AT4G11820.2	hydroxymethylglutaryl-CoA synthase / HMG-CoA synthase / 3-hydroxy-3-methylglutaryl coenzyme A synthase	LOC_Os08g43170.1	hydroxymethylglutaryl-CoA synthase, putative, expressed	1,05	-1.0	1	21	1268	1288	UCUAAUAUUAUGGGACGGAGG	AAUCCGUCCCAUAAUAUAAGA	Cleavage
tae-miR1120c-5p	Traes_7AS_9819F7C48.1	PF01154,PF08540		2.3.3.10		GO:GO:0008299,GO:GO:0004421	AT4G11820.2	hydroxymethylglutaryl-CoA synthase / HMG-CoA synthase / 3-hydroxy-3-methylglutaryl coenzyme A synthase	LOC_Os08g43170.1	hydroxymethylglutaryl-CoA synthase, putative, expressed	1,05	-1.0	1	21	1311	1331	UAAUAUAAGAACGUUUUUGAC	GUCAAAAACGCUCUUAUAUUA	Translation
tae-miR5086	Traes_7AS_9E1AC3FE6.1										3,00	-1.0	1	21	324	344	ACAUUGGUGGAAGGCGUGGUA	CGCCAUGGCUUCCACCAAUGG	Cleavage
tae-miR7757-5p	Traes_7AS_E43D64A80.1	PF00931	KOG4658			GO:GO:0043531	AT3G46530.1	NB-ARC domain-containing disease resistance protein	LOC_Os07g26430.1	resistance protein, putative, expressed	2,05	-1.0	1	22	377	398	AUAAAACCUUCAGCUAUCCAUC	AAUGGAUAACUGAAGAUUUUAU	Cleavage
tae-miR7757-5p	Traes_7AS_E43D64A80.2	PF00931	KOG4658			GO:GO:0043531	AT3G46530.1	NB-ARC domain-containing disease resistance protein	LOC_Os07g26430.1	resistance protein, putative, expressed	2,05	-1.0	1	22	1424	1445	AUAAAACCUUCAGCUAUCCAUC	AAUGGAUAACUGAAGAUUUUAU	Cleavage
tae-miR156	Traes_7AS_FE67F9E86.1								LOC_Os08g41940.1	OsSPL16 - SBP-box gene family member, expressed	1,00	-1.0	1	21	358	378	UGACAGAAGAGAGUGAGCACA	UGUGCUCUCUCUCUUCUGUCA	Cleavage
tae-miR156	Traes_7AS_FE67F9E86.2								LOC_Os08g41940.1	OsSPL16 - SBP-box gene family member, expressed	1,00	-1.0	1	21	358	378	UGACAGAAGAGAGUGAGCACA	UGUGCUCUCUCUCUUCUGUCA	Cleavage
tae-miR156	Traes_7AS_FE67F9E86.3								LOC_Os08g41940.1	OsSPL16 - SBP-box gene family member, expressed	1,00	-1.0	1	21	331	351	UGACAGAAGAGAGUGAGCACA	UGUGCUCUCUCUCUUCUGUCA	Cleavage
tae-miR9654a-3p	Traes_7BL_03C3797A2.1	PF00635,PF00400,PF00069	KOG1187			GO:GO:0005515,GO:GO:0006468,GO:GO:0005524,GO:GO:0004672	AT1G79990.3	structural molecules	LOC_Os11g10640.1	protein kinase domain containing protein, expressed	2,00	-1.0	1	22	1813	1834	UUCUGAAAGGCUUGAAGCGAAU	GAGGGCCUCAAGCCUUUCAGAA	Cleavage

tae-miR9654a-3p	Traes_7BL_03C3797A2.2	PF00635,PF00400,PF00069	KOG1187			GO:GO:0005515,GO:GO:0006468,GO:GO:0005524,GO:GO:0004672	AT1G79990.3	structural molecules	LOC_Os11g10640.1	protein kinase domain containing protein, expressed	2,00	-1.0	1	22	1813	1834	UUCUGAAAGGCUUGAAGCGAAU	GAGGGCCUCAAGCCUUUCAGAA	Cleavage
tae-miR9654a-3p	Traes_7BL_03C3797A2.3	PF00635,PF00400,PF00069	KOG1187			GO:GO:0005515,GO:GO:0006468,GO:GO:0005524,GO:GO:0004672	AT1G79990.3	structural molecules	LOC_Os11g10640.1	protein kinase domain containing protein, expressed	2,00	-1.0	1	22	1513	1534	UUCUGAAAGGCUUGAAGCGAAU	GAGGGCCUCAAGCCUUUCAGAA	Cleavage
tae-miR9654a-3p	Traes_7BL_03C3797A2.4	PF00635,PF00400,PF00069	KOG1187			GO:GO:0005515,GO:GO:0006468,GO:GO:0005524,GO:GO:0004672	AT1G79990.3	structural molecules	LOC_Os11g10640.1	protein kinase domain containing protein, expressed	2,00	-1.0	1	22	1513	1534	UUCUGAAAGGCUUGAAGCGAAU	GAGGGCCUCAAGCCUUUCAGAA	Cleavage
tae-miR9654a-3p	Traes_7BL_03C3797A2.5	PF00635,PF00400,PF00069	KOG0294			GO:GO:0005515,GO:GO:0006468,GO:GO:0005524,GO:GO:0004672	AT1G79990.3	structural molecules	LOC_Os11g10640.1	protein kinase domain containing protein, expressed	2,00	-1.0	1	22	1399	1420	UUCUGAAAGGCUUGAAGCGAAU	GAGGGCCUCAAGCCUUUCAGAA	Cleavage
tae-miR9654a-3p	Traes_7BL_03C3797A2.6	PF00635,PF00400	KOG0294			GO:GO:0005515	AT1G79990.3	structural molecules	LOC_Os02g11830.2	coatomer subunit beta, putative, expressed	2,00	-1.0	1	22	973	994	UUCUGAAAGGCUUGAAGCGAAU	GAGGGCCUCAAGCCUUUCAGAA	Cleavage
tae-miR9654a-3p	Traes_7BL_03C3797A2.7	PF00635,PF00069	KOG1187	2.7.11.1		GO:GO:0006468,GO:GO:0005524,GO:GO:0004672	AT4G21410.1	cysteine-rich RLK (RECEPTOR-like protein kinase) 29	LOC_Os04g30030.1	cysteine-rich receptor-like protein kinase 12 precursor, putative, expressed	2,00	-1.0	1	22	1813	1834	UUCUGAAAGGCUUGAAGCGAAU	GAGGGCCUCAAGCCUUUCAGAA	Cleavage
tae-miR1130b-3p	Traes_7BL_0665E8707.6										1,00	-1.0	1	21	46	66	UCUUAUAUUAUGGGACGGAGG	CCUCCGUCCCAUAAUAUAAGU	Cleavage
tae-miR1122c-3p	Traes_7BL_0665E8707.6										2,05	-1.0	1	21	46	66	UCUAAUAUUAUGGGACGGAGG	CCUCCGUCCCAUAAUAUAAGU	Cleavage
tae-miR1133	Traes_7BL_07C4E8081.43										0,00	-1.0	1	22	46	67	CAUAUACUCCCUCCGUCCGAAA	UUUCGGACGGAGGGAGUAUAUG	Cleavage
tae-miR1133	Traes_7BL_07C4E8081.44										0,00	-1.0	1	22	46	67	CAUAUACUCCCUCCGUCCGAAA	UUUCGGACGGAGGGAGUAUAUG	Cleavage
tae-miR1133	Traes_7BL_07C4E8081.45										0,00	-1.0	1	22	46	67	CAUAUACUCCCUCCGUCCGAAA	UUUCGGACGGAGGGAGUAUAUG	Cleavage
tae-miR160	Traes_7BL_18D335F08.1						AT4G30080.1	auxin response factor 16	LOC_Os06g47150.2	auxin response factor 18, putative, expressed	0,00	-1.0	1	21	171	191	UGCCUGGCUCCCUGUAUGCCA	AGGCAUACAGGGAGCCAGGCA	Cleavage
tae-miR160	Traes_7BL_18D335F08.2						AT4G30080.1	auxin response factor 16	LOC_Os06g47150.1	auxin response factor 18, putative, expressed	0,00	-1.0	1	21	171	191	UGCCUGGCUCCCUGUAUGCCA	AGGCAUACAGGGAGCCAGGCA	Cleavage
tae-miR1137a	Traes_7BL_C184D0525.2	PF00249					AT5G61620.1	myb-like transcription factor family protein	LOC_Os08g05510.1	MYB family transcription factor, putative, expressed	0,00	-1.0	1	20	913	932	UAGUACAAAGUUGAGUCAUC	GAUGACUCAACUUUGUACUA	Cleavage
tae-miR395b	Traes_7BL_DC0D8773C.1										2,05	-1.0	1	20	1385	1404	UGAAGUGUUUGGGGGAACUC	CAGUUCACCCAAAAACUUCA	Cleavage
tae-miR395b	Traes_7BL_DC0D8773C.10										2,05	-1.0	1	20	1148	1167	UGAAGUGUUUGGGGGAACUC	CAGUUCACCCAAAAACUUCA	Cleavage
tae-miR395b	Traes_7BL_DC0D8773C.11										2,05	-1.0	1	20	848	867	UGAAGUGUUUGGGGGAACUC	CAGUUCACCCAAAAACUUCA	Cleavage
tae-miR395b	Traes_7BL_DC0D8773C.12										2,05	-1.0	1	20	848	867	UGAAGUGUUUGGGGGAACUC	CAGUUCACCCAAAAACUUCA	Cleavage
tae-miR395b	Traes_7BL_DC0D8773C.13										2,05	-1.0	1	20	848	867	UGAAGUGUUUGGGGGAACUC	CAGUUCACCCAAAAACUUCA	Cleavage
tae-miR395b	Traes_7BL_DC0D8773C.14										2,05	-1.0	1	20	848	867	UGAAGUGUUUGGGGGAACUC	CAGUUCACCCAAAAACUUCA	Cleavage
tae-miR395b	Traes_7BL_DC0D8773C.2										2,05	-1.0	1	20	1385	1404	UGAAGUGUUUGGGGGAACUC	CAGUUCACCCAAAAACUUCA	Cleavage
tae-miR395b	Traes_7BL_DC0D8773C.3										2,05	-1.0	1	20	1385	1404	UGAAGUGUUUGGGGGAACUC	CAGUUCACCCAAAAACUUCA	Cleavage
tae-miR395b	Traes_7BL_DC0D8773C.4										2,05	-1.0	1	20	1385	1404	UGAAGUGUUUGGGGGAACUC	CAGUUCACCCAAAAACUUCA	Cleavage
tae-miR395b	Traes_7BL_DC0D8773C.5										2,05	-1.0	1	20	1385	1404	UGAAGUGUUUGGGGGAACUC	CAGUUCACCCAAAAACUUCA	Cleavage
tae-miR395b	Traes_7BL_DC0D8773C.6										2,05	-1.0	1	20	1148	1167	UGAAGUGUUUGGGGGAACUC	CAGUUCACCCAAAAACUUCA	Cleavage
tae-miR395b	Traes_7BL_DC0D8773C.7										2,05	-1.0	1	20	1148	1167	UGAAGUGUUUGGGGGAACUC	CAGUUCACCCAAAAACUUCA	Cleavage
tae-miR395b	Traes_7BL_DC0D8773C.8										2,05	-1.0	1	20	1148	1167	UGAAGUGUUUGGGGGAACUC	CAGUUCACCCAAAAACUUCA	Cleavage
tae-miR395b	Traes_7BL_DC0D8773C.9										2,05	-1.0	1	20	1148	1167	UGAAGUGUUUGGGGGAACUC	CAGUUCACCCAAAAACUUCA	Cleavage
tae-miR164	Traes_7BL_F1A0270C6.1								LOC_Os06g46270.1	no apical meristem protein, putative, expressed	1,00	-1.0	1	21	32	52	UGGAGAAGCAGGGCACGUGCA	AGCAAGUGCCCUGCUUCUCCA	Cleavage
tae-miR9666a-3p	Traes_7BL_F8D5E60B1.1	PF05001,PF05000,PF04983,PF04990,PF04992,PF00623,PF04997,PF04998		2.7.7.6	K03006	GO:GO:0006366,GO:GO:0005665,GO:GO:0003677,GO:GO:0006351,GO:GO:0003899	AT4G35800.1	RNA polymerase II large subunit	LOC_Os05g05860.1	retrotransposon protein, putative, unclassified, expressed	2,00	-1.0	1	22	5050	5071	CGGUAGGGCUGUAUGAUGGCGA	UCCCCCUCAUACAGCCCUACCU	Cleavage
tae-miR9666a-3p	Traes_7BL_F8D5E60B1.1	PF05001,PF05000,PF04983,PF04990,PF04992,PF00623,PF04997,PF04998		2.7.7.6	K03006	GO:GO:0006366,GO:GO:0005665,GO:GO:0003677,GO:GO:0006351,GO:GO:0003899	AT4G35800.1	RNA polymerase II large subunit	LOC_Os05g05860.1	retrotransposon protein, putative, unclassified, expressed	2,05	-1.0	1	22	4966	4987	CGGUAGGGCUGUAUGAUGGCGA	UCGCCAUCAUACAGCCCUACUU	Cleavage
tae-miR9666a-3p	Traes_7BL_F8D5E60B1.2	PF05001,PF05000,PF04983,PF04990,PF04992,PF00623,PF04997,PF04998		2.7.7.6	K03006	GO:GO:0006366,GO:GO:0005665,GO:GO:0003677,GO:GO:0006351,GO:GO:0003899	AT4G35800.1	RNA polymerase II large subunit	LOC_Os05g05860.1	retrotransposon protein, putative, unclassified, expressed	2,00	-1.0	1	22	5011	5032	CGGUAGGGCUGUAUGAUGGCGA	UCCCCCUCAUACAGCCCUACCU	Cleavage
tae-miR9666a-3p	Traes_7BL_F8D5E60B1.2	PF05001,PF05000,PF04983,PF04990,PF04992,PF00623,PF04997,PF04998		2.7.7.6	K03006	GO:GO:0006366,GO:GO:0005665,GO:GO:0003677,GO:GO:0006351,GO:GO:0003899	AT4G35800.1	RNA polymerase II large subunit	LOC_Os05g05860.1	retrotransposon protein, putative, unclassified, expressed	2,05	-1.0	1	22	4927	4948	CGGUAGGGCUGUAUGAUGGCGA	UCGCCAUCAUACAGCCCUACUU	Cleavage
tae-miR9666a-3p	Traes_7BL_F8D5E60B1.3	PF05001,PF05000,PF04983,PF04990,PF04997,PF04992,PF00623,PF04998		2.7.7.6		GO:GO:0006366,GO:GO:0005665,GO:GO:0003677,GO:GO:0006351,GO:GO:0003899	AT4G35800.1	RNA polymerase II large subunit	LOC_Os05g05860.1	retrotransposon protein, putative, unclassified, expressed	2,00	-1.0	1	22	4780	4801	CGGUAGGGCUGUAUGAUGGCGA	UCCCCCUCAUACAGCCCUACCU	Cleavage
tae-miR9666a-3p	Traes_7BL_F8D5E60B1.3	PF05001,PF05000,PF04983,PF04990,PF04997,PF04992,PF00623,PF04998		2.7.7.6		GO:GO:0006366,GO:GO:0005665,GO:GO:0003677,GO:GO:0006351,GO:GO:0003899	AT4G35800.1	RNA polymerase II large subunit	LOC_Os05g05860.1	retrotransposon protein, putative, unclassified, expressed	2,05	-1.0	1	22	4696	4717	CGGUAGGGCUGUAUGAUGGCGA	UCGCCAUCAUACAGCCCUACUU	Cleavage
tae-miR9666a-3p	Traes_7BL_F8D5E60B1.4	PF05001,PF05000,PF04983,PF04990,PF04992,PF04998		2.7.7.6		GO:GO:0006366,GO:GO:0005665,GO:GO:0003677,GO:GO:0006351,GO:GO:0003899	AT4G35800.1	RNA polymerase II large subunit	LOC_Os05g05860.1	retrotransposon protein, putative, unclassified, expressed	2,00	-1.0	1	22	3409	3430	CGGUAGGGCUGUAUGAUGGCGA	UCCCCCUCAUACAGCCCUACCU	Cleavage
tae-miR9666a-3p	Traes_7BL_F8D5E60B1.4	PF05001,PF05000,PF04983,PF04990,PF04992,PF04998		2.7.7.6		GO:GO:0006366,GO:GO:0005665,GO:GO:0003677,GO:GO:0006351,GO:GO:0003899	AT4G35800.1	RNA polymerase II large subunit	LOC_Os05g05860.1	retrotransposon protein, putative, unclassified, expressed	2,05	-1.0	1	22	3325	3346	CGGUAGGGCUGUAUGAUGGCGA	UCGCCAUCAUACAGCCCUACUU	Cleavage
tae-miR1137a	Traes_7BS_2A6C29F49.3	PF00566					AT3G49350.1	Ypt/Rab-GAP domain of gyp1p superfamily protein	LOC_Os09g35960.1	TBC domain containing protein, expressed	2,00	-1.0	1	20	75	94	UAGUACAAAGUUGAGUCAUC	AGUGUCUCAACUUUGUACUA	Cleavage
tae-miR1137a	Traes_7BS_2A6C29F49.4	PF00566					AT3G49350.1	Ypt/Rab-GAP domain of gyp1p superfamily protein	LOC_Os09g35960.1	TBC domain containing protein, expressed	2,00	-1.0	1	20	81	100	UAGUACAAAGUUGAGUCAUC	AGUGUCUCAACUUUGUACUA	Cleavage
tae-miR1130b-3p	Traes_7BS_47B8FC9CB.2	PF01154,PF08540		2.3.3.10		GO:GO:0008299,GO:GO:0004421	AT4G11820.2	hydroxymethylglutaryl-CoA synthase / HMG-CoA synthase / 3-hydroxy-3-methylglutaryl coenzyme A synthase	LOC_Os08g43170.1	hydroxymethylglutaryl-CoA synthase, putative, expressed	0,00	-1.0	1	21	1268	1288	UCUUAUAUUAUGGGACGGAGG	CCUCCGUCCCAUAAUAUAAGA	Cleavage
tae-miR1122c-3p	Traes_7BS_47B8FC9CB.2	PF01154,PF08540		2.3.3.10		GO:GO:0008299,GO:GO:0004421	AT4G11820.2	hydroxymethylglutaryl-CoA synthase / HMG-CoA synthase / 3-hydroxy-3-methylglutaryl coenzyme A synthase	LOC_Os08g43170.1	hydroxymethylglutaryl-CoA synthase, putative, expressed	1,05	-1.0	1	21	1268	1288	UCUAAUAUUAUGGGACGGAGG	CCUCCGUCCCAUAAUAUAAGA	Cleavage
tae-miR1120c-5p	Traes_7BS_47B8FC9CB.2	PF01154,PF08540		2.3.3.10		GO:GO:0008299,GO:GO:0004421	AT4G11820.2	hydroxymethylglutaryl-CoA synthase / HMG-CoA synthase / 3-hydroxy-3-methylglutaryl coenzyme A synthase	LOC_Os08g43170.1	hydroxymethylglutaryl-CoA synthase, putative, expressed	1,05	-1.0	1	21	1311	1331	UAAUAUAAGAACGUUUUUGAC	GUCAAAAACGCUCUUAUAUUA	Translation
tae-miR1130b-3p	Traes_7BS_47B8FC9CB.3	PF01154,PF08540		2.3.3.10	K01641	GO:GO:0008299,GO:GO:0004421	AT4G11820.2	hydroxymethylglutaryl-CoA synthase / HMG-CoA synthase / 3-hydroxy-3-methylglutaryl coenzyme A synthase	LOC_Os08g43170.1	hydroxymethylglutaryl-CoA synthase, putative, expressed	0,00	-1.0	1	21	1265	1285	UCUUAUAUUAUGGGACGGAGG	CCUCCGUCCCAUAAUAUAAGA	Cleavage
tae-miR1122c-3p	Traes_7BS_47B8FC9CB.3	PF01154,PF08540		2.3.3.10	K01641	GO:GO:0008299,GO:GO:0004421	AT4G11820.2	hydroxymethylglutaryl-CoA synthase / HMG-CoA synthase / 3-hydroxy-3-methylglutaryl coenzyme A synthase	LOC_Os08g43170.1	hydroxymethylglutaryl-CoA synthase, putative, expressed	1,05	-1.0	1	21	1265	1285	UCUAAUAUUAUGGGACGGAGG	CCUCCGUCCCAUAAUAUAAGA	Cleavage
tae-miR1120c-5p	Traes_7BS_47B8FC9CB.3	PF01154,PF08540		2.3.3.10	K01641	GO:GO:0008299,GO:GO:0004421	AT4G11820.2	hydroxymethylglutaryl-CoA synthase / HMG-CoA synthase / 3-hydroxy-3-methylglutaryl coenzyme A synthase	LOC_Os08g43170.1	hydroxymethylglutaryl-CoA synthase, putative, expressed	1,05	-1.0	1	21	1308	1328	UAAUAUAAGAACGUUUUUGAC	GUCAAAAACGCUCUUAUAUUA	Translation
tae-miR9662a-3p	Traes_7BS_598310C32.1	PF02536				GO:GO:0006355,GO:GO:0005739,GO:GO:0003690	AT1G21150.1	Mitochondrial transcription termination factor family protein	LOC_Os06g12100.1	mTERF family protein, expressed	2,05	-1.0	1	21	33	53	UUGAACAUCCCAGAGCCACCG	CCGUGGCUCUGCGAUGUUCAG	Translation
tae-miR9662b-3p	Traes_7BS_598310C32.1	PF02536				GO:GO:0006355,GO:GO:0005739,GO:GO:0003690	AT1G21150.1	Mitochondrial transcription termination factor family protein	LOC_Os06g12100.1	mTERF family protein, expressed	2,05	-1.0	1	21	32	52	UGAACAUCCCAGAGCCACCGG	CCCGUGGCUCUGCGAUGUUCA	Cleavage
tae-miR1121	Traes_7BS_5A0E77FB8.2	PF00664,PF00005		3.6.3.44		GO:GO:0055085,GO:GO:0042626,GO:GO:0016021,GO:GO:0006810,GO:GO:0005524,GO:GO:0016887	AT3G59140.1	multidrug resistance-associated protein 14	LOC_Os06g06440.1	ABC transporter, ATP-binding protein, putative, expressed	1,00	-1.0	1	22	3478	3499	AGUAGUGAUCUAAACGCUCUUA	UAAGAGUGUUUAGAUCACUACU	Cleavage
tae-miR1137a	Traes_7BS_6716D1D07.2	PF01105				GO:GO:0016021,GO:GO:0006810	AT1G09580.1	emp24/gp25L/p24 family/GOLD family protein	LOC_Os06g10760.1	emp24/gp25L/p24 family protein, putative, expressed	0,00	-1.0	1	20	347	366	UAGUACAAAGUUGAGUCAUC	GAUGACUCAACUUUGUACUA	Cleavage
tae-miR9781	Traes_7BS_A42F893FC.1	PF10150		3.1.26.12		GO:GO:0003723	AT2G04270.2	RNAse E/G-like	LOC_Os08g23430.1	starch binding domain containing protein, putative, expressed	1,05	-1.0	1	21	117	137	UUUUGUCACAUAUAAUACAUA	UAUGUAUUUUAUGUGACAAAA	Cleavage
tae-miR9664-3p	Traes_7DL_101A9F756.1	PF00931	KOG4658			GO:GO:0043531	AT1G58807.1	Disease resistance protein (CC-NBS-LRR class) family	LOC_Os11g11550.1	NBS-LRR disease resistance protein, putative, expressed	2,05	-1.0	1	21	102	122	UUGCAGUCCUCGAUGUCGUAG	GCACGACAUCGAGGAUUGCAU	Cleavage
tae-miR9664-3p	Traes_7DL_101A9F756.2	PF00931	KOG4658			GO:GO:0043531	AT1G58807.1	Disease resistance protein (CC-NBS-LRR class) family	LOC_Os11g11550.2	NBS-LRR disease resistance protein, putative, expressed	2,05	-1.0	1	21	102	122	UUGCAGUCCUCGAUGUCGUAG	GCACGACAUCGAGGAUUGCAU	Cleavage
tae-miR9664-3p	Traes_7DL_101A9F756.3	PF00931	KOG4658			GO:GO:0043531	AT1G58807.1	Disease resistance protein (CC-NBS-LRR class) family	LOC_Os11g11550.1	NBS-LRR disease resistance protein, putative, expressed	2,05	-1.0	1	21	231	251	UUGCAGUCCUCGAUGUCGUAG	GCACGACAUCGAGGAUUGCAU	Cleavage
tae-miR9666a-3p	Traes_7DL_18F2A8567.1	PF05001,PF05000,PF04983,PF04990,PF04992,PF00623,PF04997,PF04998		2.7.7.6	K03006	GO:GO:0006366,GO:GO:0005665,GO:GO:0003677,GO:GO:0006351,GO:GO:0003899	AT4G35800.1	RNA polymerase II large subunit	LOC_Os05g05860.1	retrotransposon protein, putative, unclassified, expressed	2,05	-1.0	1	22	4927	4948	CGGUAGGGCUGUAUGAUGGCGA	UCGCCAUCAUACAGCCCUACUU	Cleavage
tae-miR9666a-3p	Traes_7DL_18F2A8567.11	PF05001		2.7.7.6,2.7.7.48		GO:GO:0006366,GO:GO:0005665,GO:GO:0003677					2,00	-1.0	1	22	244	265	CGGUAGGGCUGUAUGAUGGCGA	UCGCCCUCAUACAGCCCUACCU	Cleavage
tae-miR9666a-3p	Traes_7DL_18F2A8567.11	PF05001		2.7.7.6,2.7.7.48		GO:GO:0006366,GO:GO:0005665,GO:GO:0003677					2,05	-1.0	1	22	139	160	CGGUAGGGCUGUAUGAUGGCGA	UCGCCAUCAUACAGCCCUACUU	Cleavage
tae-miR9666a-3p	Traes_7DL_18F2A8567.12	PF05001		2.7.7.6,2.7.7.48		GO:GO:0006366,GO:GO:0005665,GO:GO:0003677					2,00	-1.0	1	22	250	271	CGGUAGGGCUGUAUGAUGGCGA	UCGCCCUCAUACAGCCCUACCU	Cleavage
tae-miR9666a-3p	Traes_7DL_18F2A8567.12	PF05001		2.7.7.6,2.7.7.48		GO:GO:0006366,GO:GO:0005665,GO:GO:0003677					2,05	-1.0	1	22	145	166	CGGUAGGGCUGUAUGAUGGCGA	UCGCCAUCAUACAGCCCUACUU	Cleavage
tae-miR9666a-3p	Traes_7DL_18F2A8567.14										2,00	-1.0	1	22	201	222	CGGUAGGGCUGUAUGAUGGCGA	UCGCCCUCAUACAGCCCUACCU	Cleavage
tae-miR9666a-3p	Traes_7DL_18F2A8567.14										2,05	-1.0	1	22	96	117	CGGUAGGGCUGUAUGAUGGCGA	UCGCCAUCAUACAGCCCUACUU	Cleavage
tae-miR9666a-3p	Traes_7DL_18F2A8567.2	PF05001,PF05000,PF04983,PF04990,PF04992,PF00623,PF04997,PF04998		2.7.7.6	K03006	GO:GO:0006366,GO:GO:0005665,GO:GO:0003677,GO:GO:0006351,GO:GO:0003899	AT4G35800.1	RNA polymerase II large subunit	LOC_Os05g05860.1	retrotransposon protein, putative, unclassified, expressed	2,00	-1.0	1	22	5032	5053	CGGUAGGGCUGUAUGAUGGCGA	UCGCCCUCAUACAGCCCUACCU	Cleavage
tae-miR9666a-3p	Traes_7DL_18F2A8567.2	PF05001,PF05000,PF04983,PF04990,PF04992,PF00623,PF04997,PF04998		2.7.7.6	K03006	GO:GO:0006366,GO:GO:0005665,GO:GO:0003677,GO:GO:0006351,GO:GO:0003899	AT4G35800.1	RNA polymerase II large subunit	LOC_Os05g05860.1	retrotransposon protein, putative, unclassified, expressed	2,05	-1.0	1	22	4927	4948	CGGUAGGGCUGUAUGAUGGCGA	UCGCCAUCAUACAGCCCUACUU	Cleavage
tae-miR9666a-3p	Traes_7DL_18F2A8567.3	PF05001,PF05000,PF04983,PF04990,PF04992,PF00623,PF04997,PF04998		2.7.7.6	K03006	GO:GO:0006366,GO:GO:0005665,GO:GO:0003677,GO:GO:0006351,GO:GO:0003899	AT4G35800.1	RNA polymerase II large subunit	LOC_Os05g05860.1	retrotransposon protein, putative, unclassified, expressed	2,00	-1.0	1	22	4969	4990	CGGUAGGGCUGUAUGAUGGCGA	UCGCCCUCAUACAGCCCUACCU	Cleavage
tae-miR9666a-3p	Traes_7DL_18F2A8567.3	PF05001,PF05000,PF04983,PF04990,PF04992,PF00623,PF04997,PF04998		2.7.7.6	K03006	GO:GO:0006366,GO:GO:0005665,GO:GO:0003677,GO:GO:0006351,GO:GO:0003899	AT4G35800.1	RNA polymerase II large subunit	LOC_Os05g05860.1	retrotransposon protein, putative, unclassified, expressed	2,05	-1.0	1	22	4927	4948	CGGUAGGGCUGUAUGAUGGCGA	UCGCCAUCAUACAGCCCUACUU	Cleavage
tae-miR9666a-3p	Traes_7DL_18F2A8567.4	PF05001,PF05000,PF04983,PF04990,PF04992,PF00623,PF04997,PF04998		2.7.7.6	K03006	GO:GO:0006366,GO:GO:0005665,GO:GO:0003677,GO:GO:0006351,GO:GO:0003899	AT4G35800.1	RNA polymerase II large subunit	LOC_Os05g05860.1	retrotransposon protein, putative, unclassified, expressed	2,05	-1.0	1	22	4927	4948	CGGUAGGGCUGUAUGAUGGCGA	UCGCCAUCAUACAGCCCUACUU	Cleavage
tae-miR9666a-3p	Traes_7DL_18F2A8567.5	PF05001,PF05000,PF04983,PF04990,PF04992,PF00623,PF04997,PF04998		2.7.7.6	K03006	GO:GO:0006366,GO:GO:0005665,GO:GO:0003677,GO:GO:0006351,GO:GO:0003899	AT4G35800.1	RNA polymerase II large subunit	LOC_Os05g05860.1	retrotransposon protein, putative, unclassified, expressed	2,05	-1.0	1	22	4927	4948	CGGUAGGGCUGUAUGAUGGCGA	UCGCCAUCAUACAGCCCUACUU	Cleavage
tae-miR9666a-3p	Traes_7DL_18F2A8567.6	PF05001,PF05000,PF04983,PF04990,PF04992,PF00623,PF04997,PF04998		2.7.7.6	K03006	GO:GO:0006366,GO:GO:0005665,GO:GO:0003677,GO:GO:0006351,GO:GO:0003899	AT4G35800.1	RNA polymerase II large subunit	LOC_Os05g05860.1	retrotransposon protein, putative, unclassified, expressed	2,00	-1.0	1	22	5032	5053	CGGUAGGGCUGUAUGAUGGCGA	UCGCCCUCAUACAGCCCUACCU	Cleavage
tae-miR9666a-3p	Traes_7DL_18F2A8567.6	PF05001,PF05000,PF04983,PF04990,PF04992,PF00623,PF04997,PF04998		2.7.7.6	K03006	GO:GO:0006366,GO:GO:0005665,GO:GO:0003677,GO:GO:0006351,GO:GO:0003899	AT4G35800.1	RNA polymerase II large subunit	LOC_Os05g05860.1	retrotransposon protein, putative, unclassified, expressed	2,05	-1.0	1	22	4927	4948	CGGUAGGGCUGUAUGAUGGCGA	UCGCCAUCAUACAGCCCUACUU	Cleavage
tae-miR9666a-3p	Traes_7DL_18F2A8567.7	PF05001,PF05000,PF04983,PF04990,PF04997,PF04992,PF00623,PF04998		2.7.7.6	K03006	GO:GO:0006366,GO:GO:0005665,GO:GO:0003677,GO:GO:0006351,GO:GO:0003899	AT4G35800.1	RNA polymerase II large subunit	LOC_Os05g05860.1	retrotransposon protein, putative, unclassified, expressed	2,00	-1.0	1	22	4801	4822	CGGUAGGGCUGUAUGAUGGCGA	UCGCCCUCAUACAGCCCUACCU	Cleavage
tae-miR9666a-3p	Traes_7DL_18F2A8567.7	PF05001,PF05000,PF04983,PF04990,PF04997,PF04992,PF00623,PF04998		2.7.7.6	K03006	GO:GO:0006366,GO:GO:0005665,GO:GO:0003677,GO:GO:0006351,GO:GO:0003899	AT4G35800.1	RNA polymerase II large subunit	LOC_Os05g05860.1	retrotransposon protein, putative, unclassified, expressed	2,05	-1.0	1	22	4696	4717	CGGUAGGGCUGUAUGAUGGCGA	UCGCCAUCAUACAGCCCUACUU	Cleavage
tae-miR9666a-3p	Traes_7DL_18F2A8567.8	PF05001,PF05000,PF04983,PF04990,PF04992,PF04998		2.7.7.6		GO:GO:0006366,GO:GO:0005665,GO:GO:0003677,GO:GO:0006351,GO:GO:0003899	AT4G35800.1	RNA polymerase II large subunit	LOC_Os05g05860.1	retrotransposon protein, putative, unclassified, expressed	2,00	-1.0	1	22	3409	3430	CGGUAGGGCUGUAUGAUGGCGA	UCGCCCUCAUACAGCCCUACCU	Cleavage
tae-miR9666a-3p	Traes_7DL_18F2A8567.8	PF05001,PF05000,PF04983,PF04990,PF04992,PF04998		2.7.7.6		GO:GO:0006366,GO:GO:0005665,GO:GO:0003677,GO:GO:0006351,GO:GO:0003899	AT4G35800.1	RNA polymerase II large subunit	LOC_Os05g05860.1	retrotransposon protein, putative, unclassified, expressed	2,05	-1.0	1	22	3304	3325	CGGUAGGGCUGUAUGAUGGCGA	UCGCCAUCAUACAGCCCUACUU	Cleavage
tae-miR9666a-3p	Traes_7DL_18F2A8567.9	PF05001,PF05000,PF04990,PF04992,PF04998		2.7.7.6		GO:GO:0006366,GO:GO:0005665,GO:GO:0003677,GO:GO:0006351,GO:GO:0003899	AT4G35800.1	RNA polymerase II large subunit	LOC_Os05g05860.1	retrotransposon protein, putative, unclassified, expressed	2,00	-1.0	1	22	2941	2962	CGGUAGGGCUGUAUGAUGGCGA	UCGCCCUCAUACAGCCCUACCU	Cleavage
tae-miR9666a-3p	Traes_7DL_18F2A8567.9	PF05001,PF05000,PF04990,PF04992,PF04998		2.7.7.6		GO:GO:0006366,GO:GO:0005665,GO:GO:0003677,GO:GO:0006351,GO:GO:0003899	AT4G35800.1	RNA polymerase II large subunit	LOC_Os05g05860.1	retrotransposon protein, putative, unclassified, expressed	2,05	-1.0	1	22	2836	2857	CGGUAGGGCUGUAUGAUGGCGA	UCGCCAUCAUACAGCCCUACUU	Cleavage
tae-miR164	Traes_7DL_1E343B6E6.1						AT1G56010.2	NAC domain containing protein 1	LOC_Os06g46270.1	no apical meristem protein, putative, expressed	1,00	-1.0	1	21	236	256	UGGAGAAGCAGGGCACGUGCA	AGCAAGUGCCCUGCUUCUCCA	Cleavage
tae-miR164	Traes_7DL_1E343B6E6.2								LOC_Os06g46270.1	no apical meristem protein, putative, expressed	1,00	-1.0	1	21	32	52	UGGAGAAGCAGGGCACGUGCA	AGCAAGUGCCCUGCUUCUCCA	Cleavage
tae-miR1135	Traes_7DL_20BFD8A19.4	PF00145		2.1.1.37			AT4G19020.1	chromomethylase 2	LOC_Os10g01570.1	C-5 cytosine-specific DNA methylase, putative, expressed	1,05	-1.0	1	24	954	977	CUGCGACAAGUAAUUCCGAACGGA	UCUGUUCGGAAUUACUUGUCGUAG	Cleavage
tae-miR1127a	Traes_7DL_20BFD8A19.4	PF00145		2.1.1.37			AT4G19020.1	chromomethylase 2	LOC_Os10g01570.1	C-5 cytosine-specific DNA methylase, putative, expressed	1,05	-1.0	1	19	1032	1050	UCCUUCCGUUCGGAAUUAC	GUAAUUCCGAACGGAGGGA	Cleavage
tae-miR9778	Traes_7DL_31A23053A.1								LOC_Os11g39600.1	resistance protein, putative, expressed	1,00	-1.0	1	21	132	152	UGCAUCAUCUCGAACUCGUCG	CCUCGAGUUCGAGAUGAUGCA	Cleavage
tae-miR1122a	Traes_7DL_37135CE6E.2	PF00076	KOG0128			GO:GO:0003676	AT4G24270.2	EMBRYO DEFECTIVE 140	LOC_Os08g02130.2	RNA recognition motif containing protein, putative, expressed	2,05	-1.0	1	20	2105	2124	UAGAUACAUCCGUAUCUAGA	UCUAAAUACGGAUGUAUCAA	Cleavage
tae-miR1122a	Traes_7DL_37135CE6E.3	PF00076	KOG0128			GO:GO:0003676	AT4G24270.2	EMBRYO DEFECTIVE 140	LOC_Os08g02130.2	RNA recognition motif containing protein, putative, expressed	2,05	-1.0	1	20	2090	2109	UAGAUACAUCCGUAUCUAGA	UCUAAAUACGGAUGUAUCAA	Cleavage
tae-miR1122a	Traes_7DL_37135CE6E.6	PF00076	KOG0130			GO:GO:0003676	AT4G24270.2	EMBRYO DEFECTIVE 140	LOC_Os08g02130.1	RNA recognition motif containing protein, putative, expressed	2,05	-1.0	1	20	1241	1260	UAGAUACAUCCGUAUCUAGA	UCUAAAUACGGAUGUAUCAA	Cleavage
tae-miR160	Traes_7DL_55ADB3528.1	PF06507				GO:GO:0009725,GO:GO:0006355,GO:GO:0005634,GO:GO:0003677	AT4G30080.1	auxin response factor 16	LOC_Os06g47150.1	auxin response factor 18, putative, expressed	0,00	-1.0	1	21	714	734	UGCCUGGCUCCCUGUAUGCCA	AGGCAUACAGGGAGCCAGGCA	Cleavage
tae-miR160	Traes_7DL_55ADB3528.2	PF06507				GO:GO:0009725,GO:GO:0006355,GO:GO:0005634,GO:GO:0003677	AT4G30080.1	auxin response factor 16	LOC_Os06g47150.3	auxin response factor 18, putative, expressed	0,00	-1.0	1	21	714	734	UGCCUGGCUCCCUGUAUGCCA	AGGCAUACAGGGAGCCAGGCA	Cleavage
tae-miR1134	Traes_7DL_5E2559796.1										2,00	-1.0	1	24	2562	2585	CAACAACAACAAGAAGAAGAAGAU	UUGUUGUUGUUCUUGUUGUUGUUG	Cleavage
tae-miR1134	Traes_7DL_5E2559796.2										2,00	-1.0	1	24	2556	2579	CAACAACAACAAGAAGAAGAAGAU	UUGUUGUUGUUCUUGUUGUUGUUG	Cleavage
tae-miR1122b-3p	Traes_7DL_613007B24.2	PF00400	KOG0279,KOG1332			GO:GO:0005515	AT1G15750.3	Transducin family protein / WD-40 repeat family protein	LOC_Os08g06480.1	lissencephaly type-1-like homology motif, putative, expressed	1,05	-1.0	1	21	1492	1512	AGACUUAUAUGUAGGAACGGA	UCCGUUCCUAAAUAUAAGUCU	Translation
tae-miR164	Traes_7DL_91CFE828F.1	PF02365				GO:GO:0006355,GO:GO:0003677	AT1G56010.2	NAC domain containing protein 1	LOC_Os06g46270.1	no apical meristem protein, putative, expressed	1,00	-1.0	1	21	497	517	UGGAGAAGCAGGGCACGUGCA	AGCAAGUGCCCUGCUUCUCCA	Cleavage
tae-miR164	Traes_7DL_91CFE828F.2						AT1G56010.2	NAC domain containing protein 1	LOC_Os06g46270.1	no apical meristem protein, putative, expressed	1,00	-1.0	1	21	296	316	UGGAGAAGCAGGGCACGUGCA	AGCAAGUGCCCUGCUUCUCCA	Cleavage
tae-miR9778	Traes_7DL_9F0AD190A.2	PF00931	KOG4658			GO:GO:0043531	AT1G58400.1	Disease resistance protein (CC-NBS-LRR class) family	LOC_Os08g14850.1	resistance protein, putative, expressed	2,05	-1.0	1	21	126	146	UGCAUCAUCUCGAACUCGUCG	AGGCGAGUUUGAGAUGAUGCA	Cleavage
tae-miR9778	Traes_7DL_9F0AD190A.3	PF00931	KOG4658			GO:GO:0043531	AT1G58400.1	Disease resistance protein (CC-NBS-LRR class) family	LOC_Os08g14850.1	resistance protein, putative, expressed	2,05	-1.0	1	21	126	146	UGCAUCAUCUCGAACUCGUCG	AGGCGAGUUUGAGAUGAUGCA	Cleavage
tae-miR5048-5p	Traes_7DL_AB7C59D49.1	PF00069		2.7.11.1		GO:GO:0006468,GO:GO:0005524,GO:GO:0004672	AT4G21230.1	cysteine-rich RLK (RECEPTOR-like protein kinase) 27	LOC_Os11g12270.2	expressed protein	0,00	-1.0	1	22	53	74	UUUGCAGGUUUUAGGUCUAAGU	ACUUAGACCUAAAACCUGCAAA	Cleavage
tae-miR5048-5p	Traes_7DL_AB7C59D49.2	PF00069		2.7.11.1		GO:GO:0006468,GO:GO:0005524,GO:GO:0004672	AT1G11300.1	protein serine/threonine kinases;protein kinases;ATP binding;sugar binding;kinases;carbohydrate binding	LOC_Os11g17380.1	protein kinase domain containing protein, expressed	0,00	-1.0	1	22	53	74	UUUGCAGGUUUUAGGUCUAAGU	ACUUAGACCUAAAACCUGCAAA	Cleavage
tae-miR5048-5p	Traes_7DL_AB7C59D49.3	PF00069		2.7.11.1		GO:GO:0006468,GO:GO:0005524,GO:GO:0004672	AT1G11300.1	protein serine/threonine kinases;protein kinases;ATP binding;sugar binding;kinases;carbohydrate binding	LOC_Os11g17380.1	protein kinase domain containing protein, expressed	0,00	-1.0	1	22	53	74	UUUGCAGGUUUUAGGUCUAAGU	ACUUAGACCUAAAACCUGCAAA	Cleavage
tae-miR5048-5p	Traes_7DL_AB7C59D49.4	PF00069		2.7.11.1		GO:GO:0006468,GO:GO:0005524,GO:GO:0004672	AT1G11300.1	protein serine/threonine kinases;protein kinases;ATP binding;sugar binding;kinases;carbohydrate binding	LOC_Os11g17380.1	protein kinase domain containing protein, expressed	0,00	-1.0	1	22	53	74	UUUGCAGGUUUUAGGUCUAAGU	ACUUAGACCUAAAACCUGCAAA	Cleavage
tae-miR5048-5p	Traes_7DL_AB7C59D49.5	PF00069		2.7.11.1		GO:GO:0006468,GO:GO:0005524,GO:GO:0004672	AT4G21230.1	cysteine-rich RLK (RECEPTOR-like protein kinase) 27	LOC_Os11g12270.2	expressed protein	0,00	-1.0	1	22	53	74	UUUGCAGGUUUUAGGUCUAAGU	ACUUAGACCUAAAACCUGCAAA	Cleavage
tae-miR5048-5p	Traes_7DL_AB7C59D49.6	PF00069		2.7.11.1		GO:GO:0006468,GO:GO:0005524,GO:GO:0004672	AT4G21230.1	cysteine-rich RLK (RECEPTOR-like protein kinase) 27	LOC_Os11g12270.2	expressed protein	0,00	-1.0	1	22	53	74	UUUGCAGGUUUUAGGUCUAAGU	ACUUAGACCUAAAACCUGCAAA	Cleavage
tae-miR1122a	Traes_7DL_C074DC1F6.3	PF00141		1.11.1.7		GO:GO:0055114,GO:GO:0020037,GO:GO:0006979,GO:GO:0004601	AT5G64120.1	Peroxidase superfamily protein	LOC_Os07g44550.1	peroxidase precursor, putative, expressed	2,05	-1.0	1	20	109	128	UAGAUACAUCCGUAUCUAGA	ACUACAUACGGAUGUAUAUA	Cleavage
tae-miR395b	Traes_7DL_CB073ADC6.1										2,05	-1.0	1	20	1598	1617	UGAAGUGUUUGGGGGAACUC	CAGUUCACCCAAAAACUUCA	Cleavage
tae-miR395b	Traes_7DL_CB073ADC6.10										2,05	-1.0	1	20	851	870	UGAAGUGUUUGGGGGAACUC	CAGUUCACCCAAAAACUUCA	Cleavage
tae-miR395b	Traes_7DL_CB073ADC6.11	PF04926,PF04928		2.7.7.19		GO:GO:0043631,GO:GO:0003723,GO:GO:0004652,GO:GO:0005634	AT4G32850.5	nuclear poly(a) polymerase	LOC_Os06g36360.2	expressed protein	2,05	-1.0	1	20	851	870	UGAAGUGUUUGGGGGAACUC	CAGUUCACCCAAAAACUUCA	Cleavage
tae-miR395b	Traes_7DL_CB073ADC6.12	PF04926,PF04928		2.7.7.19		GO:GO:0043631,GO:GO:0003723,GO:GO:0004652,GO:GO:0005634	AT4G32850.9	nuclear poly(a) polymerase	LOC_Os06g36360.2	expressed protein	2,05	-1.0	1	20	851	870	UGAAGUGUUUGGGGGAACUC	CAGUUCACCCAAAAACUUCA	Cleavage
tae-miR395b	Traes_7DL_CB073ADC6.2	PF01909,PF04926,PF04928		2.7.7.19	K14376	GO:GO:0016779,GO:GO:0043631,GO:GO:0003723,GO:GO:0004652,GO:GO:0005634	AT4G32850.5	nuclear poly(a) polymerase	LOC_Os06g36360.2	expressed protein	2,05	-1.0	1	20	1598	1617	UGAAGUGUUUGGGGGAACUC	CAGUUCACCCAAAAACUUCA	Cleavage
tae-miR395b	Traes_7DL_CB073ADC6.3	PF01909,PF04926,PF04928		2.7.7.19	K14376	GO:GO:0016779,GO:GO:0043631,GO:GO:0003723,GO:GO:0004652,GO:GO:0005634	AT4G32850.9	nuclear poly(a) polymerase	LOC_Os06g36360.1	expressed protein	2,05	-1.0	1	20	1598	1617	UGAAGUGUUUGGGGGAACUC	CAGUUCACCCAAAAACUUCA	Cleavage
tae-miR395b	Traes_7DL_CB073ADC6.4	PF04926,PF04928		2.7.7.19		GO:GO:0043631,GO:GO:0003723,GO:GO:0004652,GO:GO:0005634	AT4G32850.10	nuclear poly(a) polymerase	LOC_Os06g36360.3	expressed protein	2,05	-1.0	1	20	1157	1176	UGAAGUGUUUGGGGGAACUC	CAGUUCACCCAAAAACUUCA	Cleavage
tae-miR395b	Traes_7DL_CB073ADC6.5	PF04926,PF04928		2.7.7.19		GO:GO:0043631,GO:GO:0003723,GO:GO:0004652,GO:GO:0005634	AT4G32850.10	nuclear poly(a) polymerase	LOC_Os06g36360.1	expressed protein	2,05	-1.0	1	20	1157	1176	UGAAGUGUUUGGGGGAACUC	CAGUUCACCCAAAAACUUCA	Cleavage
tae-miR395b	Traes_7DL_CB073ADC6.6	PF04926,PF04928		2.7.7.19		GO:GO:0043631,GO:GO:0003723,GO:GO:0004652,GO:GO:0005634	AT4G32850.10	nuclear poly(a) polymerase	LOC_Os06g36360.3	expressed protein	2,05	-1.0	1	20	1157	1176	UGAAGUGUUUGGGGGAACUC	CAGUUCACCCAAAAACUUCA	Cleavage
tae-miR395b	Traes_7DL_CB073ADC6.7	PF04926,PF04928		2.7.7.19		GO:GO:0043631,GO:GO:0003723,GO:GO:0004652,GO:GO:0005634	AT4G32850.10	nuclear poly(a) polymerase	LOC_Os06g36360.2	expressed protein	2,05	-1.0	1	20	1157	1176	UGAAGUGUUUGGGGGAACUC	CAGUUCACCCAAAAACUUCA	Cleavage
tae-miR395b	Traes_7DL_CB073ADC6.8	PF04926,PF04928		2.7.7.19		GO:GO:0043631,GO:GO:0003723,GO:GO:0004652,GO:GO:0005634	AT4G32850.10	nuclear poly(a) polymerase	LOC_Os06g36360.2	expressed protein	2,05	-1.0	1	20	1079	1098	UGAAGUGUUUGGGGGAACUC	CAGUUCACCCAAAAACUUCA	Cleavage
tae-miR395b	Traes_7DL_CB073ADC6.9	PF04926,PF04928		2.7.7.19		GO:GO:0043631,GO:GO:0003723,GO:GO:0004652,GO:GO:0005634	AT4G32850.9	nuclear poly(a) polymerase	LOC_Os06g36360.4	expressed protein	2,05	-1.0	1	20	851	870	UGAAGUGUUUGGGGGAACUC	CAGUUCACCCAAAAACUUCA	Cleavage
tae-miR1130b-3p	Traes_7DL_CF6AABA1F.2						AT4G31430.3		LOC_Os06g40460.1	expressed protein	0,00	-1.0	1	21	1271	1291	UCUUAUAUUAUGGGACGGAGG	CCUCCGUCCCAUAAUAUAAGA	Cleavage
tae-miR1122c-3p	Traes_7DL_CF6AABA1F.2						AT4G31430.3		LOC_Os06g40460.1	expressed protein	1,05	-1.0	1	21	1271	1291	UCUAAUAUUAUGGGACGGAGG	CCUCCGUCCCAUAAUAUAAGA	Cleavage
tae-miR1128	Traes_7DL_D5FCF9F33.4								LOC_Os06g46480.1	expressed protein	2,05	-1.0	1	21	96	116	UACUACUCCCUCCGUCCGAAA	UUUGGGACGGAGGGAGUAUUA	Cleavage
tae-miR1122a	Traes_7DL_D5FCF9F33.4								LOC_Os06g46480.1	expressed protein	0,00	-1.0	1	20	23	42	UAGAUACAUCCGUAUCUAGA	UCUAGAUACGGAUGUAUCUA	Cleavage
tae-miR5049-3p	Traes_7DS_06CD5BBFB.4						AT3G21350.2	RNA polymerase transcriptional regulation mediator-related	LOC_Os06g11370.1	mediator of RNA polymerase II transcription subunit 6, putative, expressed	0,00	-1.0	1	21	99	119	AAUAUGGAUCGGAGGGAGUAC	UUACUCCCUCCGAUCCAUAUU	Cleavage
tae-miR1134	Traes_7DS_205267E87.1										2,05	-1.0	1	24	92	115	CAACAACAACAAGAAGAAGAAGAU	CUGUUCUUCUUCUUGUUGUUGUCU	Cleavage
tae-miR1130b-3p	Traes_7DS_25B02BA46.5	PF00005		3.6.3.44		GO:GO:0016887,GO:GO:0005524	AT3G53480.1	pleiotropic drug resistance 9	LOC_Os02g32690.1	pleiotropic drug resistance protein 15, putative, expressed	1,05	-1.0	1	21	730	750	UCUUAUAUUAUGGGACGGAGG	CCUCCGUCCCAUAAUGUAAGA	Cleavage
tae-miR1133	Traes_7DS_25F082B1E.8										3,00	-1.0	1	22	19	41	CAUAUACUCCCUCCGUCC-GAAA	UUUCCGGACGGAGGGAGUAUAUA	Cleavage
tae-miR9662a-3p	Traes_7DS_2B1FE04A6.1	PF02536				GO:GO:0006355,GO:GO:0005739,GO:GO:0003690	AT5G07900.1	Mitochondrial transcription termination factor family protein	LOC_Os06g12100.1	mTERF family protein, expressed	2,05	-1.0	1	21	561	581	UUGAACAUCCCAGAGCCACCG	CCGUGGCUCUGCGAUGUUCAG	Translation
tae-miR9662b-3p	Traes_7DS_2B1FE04A6.1	PF02536				GO:GO:0006355,GO:GO:0005739,GO:GO:0003690	AT5G07900.1	Mitochondrial transcription termination factor family protein	LOC_Os06g12100.1	mTERF family protein, expressed	2,05	-1.0	1	21	560	580	UGAACAUCCCAGAGCCACCGG	CCCGUGGCUCUGCGAUGUUCA	Cleavage
tae-miR1130b-3p	Traes_7DS_3C83C8EF9.1						AT4G31340.1	myosin heavy chain-related	LOC_Os08g40790.1	DNA repair ATPase-related, putative, expressed	1,00	-1.0	1	21	37	57	UCUUAUAUUAUGGGACGGAGG	CCUCCGUUCCAUAAUAUAAGA	Cleavage
tae-miR1122c-3p	Traes_7DS_3C83C8EF9.1						AT4G31340.1	myosin heavy chain-related	LOC_Os08g40790.1	DNA repair ATPase-related, putative, expressed	2,05	-1.0	1	21	37	57	UCUAAUAUUAUGGGACGGAGG	CCUCCGUUCCAUAAUAUAAGA	Cleavage
tae-miR1120c-5p	Traes_7DS_3C83C8EF9.1						AT4G31340.1	myosin heavy chain-related	LOC_Os08g40790.1	DNA repair ATPase-related, putative, expressed	1,05	-1.0	1	21	80	100	UAAUAUAAGAACGUUUUUGAC	GUCAAAAACGCUCUUAUAUUA	Translation
tae-miR1130b-3p	Traes_7DS_3C83C8EF9.3						AT4G31340.1	myosin heavy chain-related	LOC_Os08g40790.3	DNA repair ATPase-related, putative, expressed	1,00	-1.0	1	21	37	57	UCUUAUAUUAUGGGACGGAGG	CCUCCGUUCCAUAAUAUAAGA	Cleavage
tae-miR1122c-3p	Traes_7DS_3C83C8EF9.3						AT4G31340.1	myosin heavy chain-related	LOC_Os08g40790.3	DNA repair ATPase-related, putative, expressed	2,05	-1.0	1	21	37	57	UCUAAUAUUAUGGGACGGAGG	CCUCCGUUCCAUAAUAUAAGA	Cleavage
tae-miR1120c-5p	Traes_7DS_3C83C8EF9.3						AT4G31340.1	myosin heavy chain-related	LOC_Os08g40790.3	DNA repair ATPase-related, putative, expressed	1,05	-1.0	1	21	80	100	UAAUAUAAGAACGUUUUUGAC	GUCAAAAACGCUCUUAUAUUA	Translation
tae-miR9662a-3p	Traes_7DS_5B1210681.1	PF02536			K15032	GO:GO:0006355,GO:GO:0005739,GO:GO:0003690	AT1G21150.1	Mitochondrial transcription termination factor family protein	LOC_Os06g12100.1	mTERF family protein, expressed	2,05	-1.0	1	21	285	305	UUGAACAUCCCAGAGCCACCG	CCGUGGCUCUGCGAUGUUCAG	Translation
tae-miR9662b-3p	Traes_7DS_5B1210681.1	PF02536			K15032	GO:GO:0006355,GO:GO:0005739,GO:GO:0003690	AT1G21150.1	Mitochondrial transcription termination factor family protein	LOC_Os06g12100.1	mTERF family protein, expressed	2,05	-1.0	1	21	284	304	UGAACAUCCCAGAGCCACCGG	CCCGUGGCUCUGCGAUGUUCA	Cleavage
tae-miR9778	Traes_7DS_74534FCD3.1	PF00931				GO:GO:0043531	AT1G50180.1	NB-ARC domain-containing disease resistance protein	LOC_Os10g03570.1	RGH1A, putative, expressed	2,05	-1.0	1	21	111	131	UGCAUCAUCUCGAACUCGUCG	AGACGAGCUGGAGAUGAUGCA	Cleavage
tae-miR9778	Traes_7DS_74534FCD3.2	PF00931				GO:GO:0043531	AT3G46530.1	NB-ARC domain-containing disease resistance protein	LOC_Os10g03570.1	RGH1A, putative, expressed	2,05	-1.0	1	21	111	131	UGCAUCAUCUCGAACUCGUCG	AGACGAGCUGGAGAUGAUGCA	Cleavage
tae-miR9778	Traes_7DS_74534FCD3.3	PF00931				GO:GO:0043531	AT3G46530.1	NB-ARC domain-containing disease resistance protein	LOC_Os10g03570.1	RGH1A, putative, expressed	2,05	-1.0	1	21	111	131	UGCAUCAUCUCGAACUCGUCG	AGACGAGCUGGAGAUGAUGCA	Cleavage
tae-miR9778	Traes_7DS_74534FCD3.4	PF00931				GO:GO:0043531	AT3G46530.1	NB-ARC domain-containing disease resistance protein	LOC_Os10g03570.1	RGH1A, putative, expressed	2,05	-1.0	1	21	111	131	UGCAUCAUCUCGAACUCGUCG	AGACGAGCUGGAGAUGAUGCA	Cleavage
tae-miR9778	Traes_7DS_74534FCD3.5	PF00931				GO:GO:0043531	AT3G46530.1	NB-ARC domain-containing disease resistance protein	LOC_Os10g03570.1	RGH1A, putative, expressed	2,05	-1.0	1	21	111	131	UGCAUCAUCUCGAACUCGUCG	AGACGAGCUGGAGAUGAUGCA	Cleavage
tae-miR9778	Traes_7DS_74534FCD3.6	PF00931				GO:GO:0043531	AT3G46530.1	NB-ARC domain-containing disease resistance protein	LOC_Os10g03570.1	RGH1A, putative, expressed	2,05	-1.0	1	21	111	131	UGCAUCAUCUCGAACUCGUCG	AGACGAGCUGGAGAUGAUGCA	Cleavage
tae-miR1133	Traes_7DS_98F9E4E55.2	PF16211,PF00125	KOG1757			GO:GO:0003677	AT3G54560.1	histone H2A 11	LOC_Os03g53190.2	Core histone H2A/H2B/H3/H4 domain containing protein, putative, expressed	2,00	-1.0	1	22	2	24	CAUAUACUCCCUCCGUCCGA-AA	UUAUGGGACGGAGGGAGUAUAUA	Cleavage
tae-miR1122a	Traes_7DS_CA61ECF35.1						AT1G05060.1		LOC_Os06g13220.1	expressed protein	0,00	-1.0	1	20	33	52	UAGAUACAUCCGUAUCUAGA	UCUAGAUACGGAUGUAUCUA	Cleavage
tae-miR9775	Traes_7DS_D4DFDF981.1										2,00	-1.0	1	22	1609	1630	UGUGCGCAAUAAGAUUUUGCUA	GUGCAUGAUCUUAUUGCGCACA	Cleavage
tae-miR9775	Traes_7DS_D4DFDF981.2										2,00	-1.0	1	22	1702	1723	UGUGCGCAAUAAGAUUUUGCUA	GUGCAUGAUCUUAUUGCGCACA	Cleavage
tae-miR156	Traes_7DS_DBB29C7CD.1								LOC_Os08g41940.1	OsSPL16 - SBP-box gene family member, expressed	1,00	-1.0	1	21	359	379	UGACAGAAGAGAGUGAGCACA	UGUGCUCUCUCUCUUCUGUCA	Cleavage
tae-miR5049-3p	Traes_7DS_EFB144315.5	PF00076,PF04059	KOG4660			GO:GO:0003676	AT1G29400.2	MEI2-like protein 5	LOC_Os02g48790.5	AML1, putative, expressed	1,05	-1.0	1	21	2298	2318	AAUAUGGAUCGGAGGGAGUAC	UUACUCCCUCUGAUCCAUAUU	Translation

Triticum turgidum Stress Root

miRNA_Acc.	Target_Acc.	Pfam	KOG	KEGG/ec	KO	GO	Best-hit-arabi-name	arabi-defline	Best-hit-rice-name	rice-defline	Expectation	UPE$	miRNA_start	miRNA_end	Target_start	Target_end	miRNA_aligned_fragment	Target_aligned_fragment	Inhibition
tae-miR159a	Traes_1AL_041EBB4A6.1						AT2G32460.1	myb domain protein 101	LOC_Os01g59660.2	MYB family transcription factor, putative, expressed	2,05	-1.0	1	21	488	508	UUUGGAUUGAAGGGAGCUCUG	UGGAGCUCCCUUCAUUCCAAU	Cleavage
tae-miR159b	Traes_1AL_041EBB4A6.1						AT2G32460.1	myb domain protein 101	LOC_Os01g59660.2	MYB family transcription factor, putative, expressed	2,05	-1.0	1	21	488	508	UUUGGAUUGAAGGGAGCUCUG	UGGAGCUCCCUUCAUUCCAAU	Cleavage
tae-miR159a	Traes_1AL_041EBB4A6.2						AT2G32460.1	myb domain protein 101	LOC_Os01g59660.2	MYB family transcription factor, putative, expressed	2,05	-1.0	1	21	512	532	UUUGGAUUGAAGGGAGCUCUG	UGGAGCUCCCUUCAUUCCAAU	Cleavage
tae-miR159b	Traes_1AL_041EBB4A6.2						AT2G32460.1	myb domain protein 101	LOC_Os01g59660.2	MYB family transcription factor, putative, expressed	2,05	-1.0	1	21	512	532	UUUGGAUUGAAGGGAGCUCUG	UGGAGCUCCCUUCAUUCCAAU	Cleavage
tae-miR159a	Traes_1AL_041EBB4A6.3						AT2G32460.1	myb domain protein 101	LOC_Os01g59660.2	MYB family transcription factor, putative, expressed	2,05	-1.0	1	21	488	508	UUUGGAUUGAAGGGAGCUCUG	UGGAGCUCCCUUCAUUCCAAU	Cleavage
tae-miR159b	Traes_1AL_041EBB4A6.3						AT2G32460.1	myb domain protein 101	LOC_Os01g59660.2	MYB family transcription factor, putative, expressed	2,05	-1.0	1	21	488	508	UUUGGAUUGAAGGGAGCUCUG	UGGAGCUCCCUUCAUUCCAAU	Cleavage
tae-miR159a	Traes_1AL_041EBB4A6.4								LOC_Os01g59660.2	MYB family transcription factor, putative, expressed	2,05	-1.0	1	21	410	430	UUUGGAUUGAAGGGAGCUCUG	UGGAGCUCCCUUCAUUCCAAU	Cleavage
tae-miR159b	Traes_1AL_041EBB4A6.4								LOC_Os01g59660.2	MYB family transcription factor, putative, expressed	2,05	-1.0	1	21	410	430	UUUGGAUUGAAGGGAGCUCUG	UGGAGCUCCCUUCAUUCCAAU	Cleavage
tae-miR160	Traes_1AL_147CF243C.1	PF06507				GO:GO:0009725,GO:GO:0006355,GO:GO:0005634,GO:GO:0003677	AT4G30080.1	auxin response factor 16	LOC_Os10g33940.1	auxin response factor 18, putative, expressed	0,00	-1.0	1	21	642	662	UGCCUGGCUCCCUGUAUGCCA	AGGCAUACAGGGAGCCAGGCA	Cleavage
tae-miR160	Traes_1AL_147CF243C.2						AT4G30080.1	auxin response factor 16	LOC_Os10g33940.1	auxin response factor 18, putative, expressed	0,00	-1.0	1	21	267	287	UGCCUGGCUCCCUGUAUGCCA	AGGCAUACAGGGAGCCAGGCA	Cleavage
tae-miR1127b-3p	Traes_1AL_4A458DF14.2	PF13812,PF01535,PF13041					AT3G62470.1	Pentatricopeptide repeat (PPR) superfamily protein	LOC_Os05g28720.1	PPR repeat containing protein, expressed	1,05	-1.0	1	21	1738	1758	ACAAGUAUUUCUGGACGGAGG	CCUCCGUCCGGAAAUACUUGU	Cleavage
tae-miR1130b-3p	Traes_1AL_51B1D5DBC.6	PF07714		2.7.11.1		GO:GO:0006468,GO:GO:0004672	AT5G14640.1	shaggy-like kinase 13	LOC_Os10g37740.1	CGMC_GSK.9 - CGMC includes CDA, MAPK, GSK3, and CLKC kinases, expressed	0,00	-1.0	1	21	914	934	UCUUAUAUUAUGGGACGGAGG	CCUCCGUCCCAUAAUAUAAGA	Cleavage
tae-miR1122c-3p	Traes_1AL_51B1D5DBC.6	PF07714		2.7.11.1		GO:GO:0006468,GO:GO:0004672	AT5G14640.1	shaggy-like kinase 13	LOC_Os10g37740.1	CGMC_GSK.9 - CGMC includes CDA, MAPK, GSK3, and CLKC kinases, expressed	1,05	-1.0	1	21	914	934	UCUAAUAUUAUGGGACGGAGG	CCUCCGUCCCAUAAUAUAAGA	Cleavage
tae-miR5049-3p	Traes_1AL_7D6DC73FC.3	PF14531	KOG0583	2.7.11.1			AT3G01090.2	SNF1 kinase homolog 10	LOC_Os05g45420.2	CAMK_KIN1/SNF1/Nim1_like_AMPKh.3 - CAMK includes calcium/calmodulin depedent protein kinases, expressed	2,00	-1.0	1	21	1256	1276	AAUAUGGAUCGGAGGGAGUAC	UUUCUCCCUCCGAUCCAUAUA	Cleavage
tae-miR1128	Traes_1AL_968B97E50.2	PF06480,PF00004,PF01434			K08956	GO:GO:0016021,GO:GO:0008270,GO:GO:0005524,GO:GO:0004222,GO:GO:0006508	AT1G07510.1	FTSH protease 10	LOC_Os05g38400.3	OsFtsH8 FtsH protease, homologue of AtFtsH3/10, expressed	3,00	-1.0	1	21	100	121	UACUACUCCCUCCGUCC-GAAA	UUUCCGGACGGAGGGAGUAGUU	Cleavage
tae-miR7757-5p	Traes_1AL_B6931117F.1	PF00931				GO:GO:0043531	AT3G46530.1	NB-ARC domain-containing disease resistance protein	LOC_Os08g10440.1	NBS-LRR disease resistance protein, putative, expressed	1,05	-1.0	1	22	1301	1322	AUAAAACCUUCAGCUAUCCAUC	AAUGGAUAGCUGAAGGCUUUAU	Cleavage
tae-miR7757-5p	Traes_1AL_B6931117F.2	PF00931				GO:GO:0043531	AT3G46530.1	NB-ARC domain-containing disease resistance protein	LOC_Os08g10440.1	NBS-LRR disease resistance protein, putative, expressed	1,05	-1.0	1	22	1343	1364	AUAAAACCUUCAGCUAUCCAUC	AAUGGAUAGCUGAAGGCUUUAU	Cleavage
tae-miR7757-5p	Traes_1AL_B6931117F.3	PF00931				GO:GO:0043531	AT3G46530.1	NB-ARC domain-containing disease resistance protein	LOC_Os08g10440.1	NBS-LRR disease resistance protein, putative, expressed	1,05	-1.0	1	22	1301	1322	AUAAAACCUUCAGCUAUCCAUC	AAUGGAUAGCUGAAGGCUUUAU	Cleavage
tae-miR7757-5p	Traes_1AL_B6931117F.4	PF00931				GO:GO:0043531	AT3G46530.1	NB-ARC domain-containing disease resistance protein	LOC_Os08g10440.1	NBS-LRR disease resistance protein, putative, expressed	1,05	-1.0	1	22	1301	1322	AUAAAACCUUCAGCUAUCCAUC	AAUGGAUAGCUGAAGGCUUUAU	Cleavage
tae-miR9778	Traes_1AS_17306388F.1	PF00931				GO:GO:0043531	AT3G07040.1	NB-ARC domain-containing disease resistance protein	LOC_Os08g42670.2	resistance protein, putative, expressed	2,00	-1.0	1	21	126	146	UGCAUCAUCUCGAACUCGUCG	CAAUGAGCUCGAGAUGAUGCA	Cleavage
tae-miR9778	Traes_1AS_17306388F.3	PF00931				GO:GO:0043531	AT3G46530.1	NB-ARC domain-containing disease resistance protein	LOC_Os12g37290.1	resistance protein T10rga2-1A, putative, expressed	2,00	-1.0	1	21	126	146	UGCAUCAUCUCGAACUCGUCG	CAAUGAGCUCGAGAUGAUGCA	Cleavage
tae-miR1135	Traes_1AS_4A925522B.4	PF13920					AT5G01960.1	RING/U-box superfamily protein	LOC_Os01g07390.1	zinc finger, C3HC4 type family protein, expressed	0,00	-1.0	1	24	511	534	CUGCGACAAGUAAUUCCGAACGGA	UCCGUUCGGAAUUACUUGUCGCAG	Cleavage
tae-miR6197-5p	Traes_1AS_5DCFDC258.1	PF04578,PF13968					AT5G45540.1	Protein of unknown function (DUF594)	LOC_Os07g08270.1	expressed protein	3,00	-1.0	1	21	1353	1376	UCUGUAAACAAAUGUA---GGACG	ACUCCAACUACAUUUGUUUACAGA	Cleavage
tae-miR1137a	Traes_1AS_8912BD052.27										0,00	-1.0	1	20	136	155	UAGUACAAAGUUGAGUCAUC	GAUGACUCAACUUUGUACUA	Cleavage
tae-miR9674a-5p	Traes_1AS_92A698B19.1	PF01535,PF13041			K17964		AT1G63130.1	Tetratricopeptide repeat (TPR)-like superfamily protein	LOC_Os04g28300.1	Rf1, mitochondrial precursor, putative, expressed	2,05	-1.0	1	21	1761	1781	GCAUCAUCCAUCCUACCAUUC	AAAUGGAAGGAUCGAUGAUGC	Cleavage
tae-miR9674a-5p	Traes_1AS_92A698B19.10	PF01535,PF13041					AT1G63130.1	Tetratricopeptide repeat (TPR)-like superfamily protein	LOC_Os04g28300.1	Rf1, mitochondrial precursor, putative, expressed	2,05	-1.0	1	21	1761	1781	GCAUCAUCCAUCCUACCAUUC	AAAUGGAAGGAUCGAUGAUGC	Cleavage
tae-miR9674a-5p	Traes_1AS_92A698B19.12	PF13041					AT1G12300.1	Tetratricopeptide repeat (TPR)-like superfamily protein	LOC_Os04g28300.1	Rf1, mitochondrial precursor, putative, expressed	2,05	-1.0	1	21	96	116	GCAUCAUCCAUCCUACCAUUC	AAAUGGAAGGAUCGAUGAUGC	Cleavage
tae-miR9674a-5p	Traes_1AS_92A698B19.2	PF01535,PF13041			K17964		AT1G63130.1	Tetratricopeptide repeat (TPR)-like superfamily protein	LOC_Os04g28300.1	Rf1, mitochondrial precursor, putative, expressed	2,05	-1.0	1	21	1761	1781	GCAUCAUCCAUCCUACCAUUC	AAAUGGAAGGAUCGAUGAUGC	Cleavage
tae-miR9674a-5p	Traes_1AS_92A698B19.3	PF01535,PF13041			K17964		AT1G63130.1	Tetratricopeptide repeat (TPR)-like superfamily protein	LOC_Os04g28300.1	Rf1, mitochondrial precursor, putative, expressed	2,05	-1.0	1	21	1761	1781	GCAUCAUCCAUCCUACCAUUC	AAAUGGAAGGAUCGAUGAUGC	Cleavage
tae-miR9674a-5p	Traes_1AS_92A698B19.4	PF01535,PF13041			K17964		AT1G63130.1	Tetratricopeptide repeat (TPR)-like superfamily protein	LOC_Os04g28300.1	Rf1, mitochondrial precursor, putative, expressed	2,05	-1.0	1	21	1761	1781	GCAUCAUCCAUCCUACCAUUC	AAAUGGAAGGAUCGAUGAUGC	Cleavage
tae-miR9674a-5p	Traes_1AS_92A698B19.5	PF01535,PF13041			K17964		AT1G63130.1	Tetratricopeptide repeat (TPR)-like superfamily protein	LOC_Os04g28300.1	Rf1, mitochondrial precursor, putative, expressed	2,05	-1.0	1	21	1761	1781	GCAUCAUCCAUCCUACCAUUC	AAAUGGAAGGAUCGAUGAUGC	Cleavage
tae-miR9674a-5p	Traes_1AS_92A698B19.6	PF01535,PF13041			K17964		AT1G63130.1	Tetratricopeptide repeat (TPR)-like superfamily protein	LOC_Os04g28300.1	Rf1, mitochondrial precursor, putative, expressed	2,05	-1.0	1	21	1761	1781	GCAUCAUCCAUCCUACCAUUC	AAAUGGAAGGAUCGAUGAUGC	Cleavage
tae-miR9674a-5p	Traes_1AS_92A698B19.7	PF01535,PF13041			K17964		AT1G63130.1	Tetratricopeptide repeat (TPR)-like superfamily protein	LOC_Os04g28300.1	Rf1, mitochondrial precursor, putative, expressed	2,05	-1.0	1	21	1761	1781	GCAUCAUCCAUCCUACCAUUC	AAAUGGAAGGAUCGAUGAUGC	Cleavage
tae-miR9674a-5p	Traes_1AS_92A698B19.8	PF01535,PF13041			K17964		AT1G63130.1	Tetratricopeptide repeat (TPR)-like superfamily protein	LOC_Os04g28300.1	Rf1, mitochondrial precursor, putative, expressed	2,05	-1.0	1	21	1761	1781	GCAUCAUCCAUCCUACCAUUC	AAAUGGAAGGAUCGAUGAUGC	Cleavage
tae-miR9674a-5p	Traes_1AS_92A698B19.9	PF01535,PF13041			K17964		AT1G63130.1	Tetratricopeptide repeat (TPR)-like superfamily protein	LOC_Os04g28300.1	Rf1, mitochondrial precursor, putative, expressed	2,05	-1.0	1	21	1761	1781	GCAUCAUCCAUCCUACCAUUC	AAAUGGAAGGAUCGAUGAUGC	Cleavage
tae-miR5049-3p	Traes_1AS_A482D4FF9.1	PF04997		2.7.7.6		GO:GO:0006351,GO:GO:0003899,GO:GO:0003677	AT1G63020.1	nuclear RNA polymerase D1A	LOC_Os02g05880.1	RNA polymerase Rpb1, domain 2 family protein, expressed	0,00	-1.0	1	21	557	577	AAUAUGGAUCGGAGGGAGUAC	GUACUCCCUCCGAUCCAUAUU	Cleavage
tae-miR9778	Traes_1AS_C7A8188D1.1	PF13191,PF00931	KOG4658			GO:GO:0043531	AT1G58390.1	Disease resistance protein (CC-NBS-LRR class) family	LOC_Os08g42670.2	resistance protein, putative, expressed	1,00	-1.0	1	21	126	146	UGCAUCAUCUCGAACUCGUCG	CAACGAGCUCGAGAUGAUGCA	Cleavage
tae-miR9778	Traes_1AS_C7A8188D1.3	PF00931				GO:GO:0043531	AT3G46530.1	NB-ARC domain-containing disease resistance protein	LOC_Os02g57310.1	pib, putative, expressed	1,00	-1.0	1	21	126	146	UGCAUCAUCUCGAACUCGUCG	CAACGAGCUCGAGAUGAUGCA	Cleavage
tae-miR1128	Traes_1BL_087CA0963.1	PF16876,PF08235	KOG2116	3.1.3.4			AT3G09560.1	Lipin family protein	LOC_Os05g38710.1	lipin, N-terminal conserved region family protein, expressed	3,00	-1.0	1	21	2048	2069	UACUACUCCCUCCG-UCCGAAA	UUUAGGAACGGAGGGAGUAGUA	Cleavage
tae-miR1128	Traes_1BL_0FDC38712.1	PF13639				GO:GO:0008270,GO:GO:0005515	AT1G17970.1	RING/U-box superfamily protein	LOC_Os05g47670.1	zinc finger, C3HC4 type domain containing protein, expressed	1,05	-1.0	1	21	225	245	UACUACUCCCUCCGUCCGAAA	UUUCGGACGGAGGGAGUACUA	Cleavage
tae-miR1127b-3p	Traes_1BL_0FDC38712.1	PF13639				GO:GO:0008270,GO:GO:0005515	AT1G17970.1	RING/U-box superfamily protein	LOC_Os05g47670.1	zinc finger, C3HC4 type domain containing protein, expressed	1,05	-1.0	1	21	121	141	ACAAGUAUUUCUGGACGGAGG	CCUCCGUCCGGAAAUACUUGU	Cleavage
tae-miR5062-5p	Traes_1BL_219B2BB6D.16						AT2G46060.1	transmembrane protein-related	LOC_Os10g30910.2	transmembrane protein-related, putative, expressed	2,05	-1.0	1	23	836	858	UGAACCUUAGGGAACAGCCGCAU	UUUUGGAUGUUCCCCAAGGUUCA	Cleavage
tae-miR5062-5p	Traes_1BL_219B2BB6D.17						AT2G46060.1	transmembrane protein-related	LOC_Os10g30910.2	transmembrane protein-related, putative, expressed	2,05	-1.0	1	23	836	858	UGAACCUUAGGGAACAGCCGCAU	UUUUGGAUGUUCCCCAAGGUUCA	Cleavage
tae-miR5062-5p	Traes_1BL_219B2BB6D.18						AT2G46060.1	transmembrane protein-related	LOC_Os10g30910.2	transmembrane protein-related, putative, expressed	2,05	-1.0	1	23	836	858	UGAACCUUAGGGAACAGCCGCAU	UUUUGGAUGUUCCCCAAGGUUCA	Cleavage
tae-miR160	Traes_1BL_54CD82AC3.1	PF06507				GO:GO:0009725,GO:GO:0006355,GO:GO:0005634,GO:GO:0003677	AT4G30080.1	auxin response factor 16	LOC_Os10g33940.1	auxin response factor 18, putative, expressed	0,00	-1.0	1	21	642	662	UGCCUGGCUCCCUGUAUGCCA	AGGCAUACAGGGAGCCAGGCA	Cleavage
tae-miR160	Traes_1BL_54CD82AC3.2						AT4G30080.1	auxin response factor 16	LOC_Os10g33940.1	auxin response factor 18, putative, expressed	0,00	-1.0	1	21	369	389	UGCCUGGCUCCCUGUAUGCCA	AGGCAUACAGGGAGCCAGGCA	Cleavage
tae-miR160	Traes_1BL_54CD82AC3.3						AT4G30080.1	auxin response factor 16	LOC_Os10g33940.1	auxin response factor 18, putative, expressed	0,00	-1.0	1	21	267	287	UGCCUGGCUCCCUGUAUGCCA	AGGCAUACAGGGAGCCAGGCA	Cleavage
tae-miR1130b-3p	Traes_1BL_89E785AAA.2	PF00436	KOG1653			GO:GO:0003697,GO:GO:0006260	AT4G11060.1	mitochondrially targeted single-stranded DNA binding protein	LOC_Os05g43440.2	single-stranded DNA-binding protein, putative, expressed	2,00	-1.0	1	21	591	611	UCUUAUAUUAUGGGACGGAGG	CCUCUGUCCCAUAAUAUAAGG	Cleavage
tae-miR159a	Traes_1BL_CF98E922B.1	PF00249					AT5G06100.2	myb domain protein 33	LOC_Os01g59660.2	MYB family transcription factor, putative, expressed	2,05	-1.0	1	21	839	859	UUUGGAUUGAAGGGAGCUCUG	UGGAGCUCCCUUCACUCCAAU	Cleavage
tae-miR159b	Traes_1BL_CF98E922B.1	PF00249					AT5G06100.2	myb domain protein 33	LOC_Os01g59660.2	MYB family transcription factor, putative, expressed	2,05	-1.0	1	21	839	859	UUUGGAUUGAAGGGAGCUCUG	UGGAGCUCCCUUCACUCCAAU	Cleavage
tae-miR159a	Traes_1BL_CF98E922B.2	PF00249					AT5G06100.3	myb domain protein 33	LOC_Os01g59660.1	MYB family transcription factor, putative, expressed	2,05	-1.0	1	21	839	859	UUUGGAUUGAAGGGAGCUCUG	UGGAGCUCCCUUCACUCCAAU	Cleavage
tae-miR159b	Traes_1BL_CF98E922B.2	PF00249					AT5G06100.3	myb domain protein 33	LOC_Os01g59660.1	MYB family transcription factor, putative, expressed	2,05	-1.0	1	21	839	859	UUUGGAUUGAAGGGAGCUCUG	UGGAGCUCCCUUCACUCCAAU	Cleavage
tae-miR159a	Traes_1BL_CF98E922B.3						AT5G06100.3	myb domain protein 33	LOC_Os01g59660.2	MYB family transcription factor, putative, expressed	2,05	-1.0	1	21	512	532	UUUGGAUUGAAGGGAGCUCUG	UGGAGCUCCCUUCACUCCAAU	Cleavage
tae-miR159b	Traes_1BL_CF98E922B.3						AT5G06100.3	myb domain protein 33	LOC_Os01g59660.2	MYB family transcription factor, putative, expressed	2,05	-1.0	1	21	512	532	UUUGGAUUGAAGGGAGCUCUG	UGGAGCUCCCUUCACUCCAAU	Cleavage
tae-miR159a	Traes_1BL_CF98E922B.4						AT5G06100.1	myb domain protein 33	LOC_Os01g59660.1	MYB family transcription factor, putative, expressed	2,05	-1.0	1	21	512	532	UUUGGAUUGAAGGGAGCUCUG	UGGAGCUCCCUUCACUCCAAU	Cleavage
tae-miR159b	Traes_1BL_CF98E922B.4						AT5G06100.1	myb domain protein 33	LOC_Os01g59660.1	MYB family transcription factor, putative, expressed	2,05	-1.0	1	21	512	532	UUUGGAUUGAAGGGAGCUCUG	UGGAGCUCCCUUCACUCCAAU	Cleavage
tae-miR1117	Traes_1BL_F01A87F44.1	PF13359					AT2G13770.1		LOC_Os01g33990.1	transposon protein, putative, CACTA, En/Spm sub-class, expressed	2,05	-1.0	1	24	37	60	UAGUACCGGUUCGUGGCACGAACC	GGUUCGUGGCACGAACCGGUGCUA	Cleavage
tae-miR1131	Traes_1BL_F01A87F44.1	PF13359					AT2G13770.1		LOC_Os01g33990.1	transposon protein, putative, CACTA, En/Spm sub-class, expressed	2,05	-1.0	1	22	63	84	UAGUACCGGUUCGUGGCUAACC	GGUUAGCCACGAACCGGUAAUG	Cleavage
tae-miR1122b-3p	Traes_1BS_34C60A54E.1	PF00651				GO:GO:0005515	AT5G19000.1	BTB-POZ and MATH domain 1	LOC_Os08g41120.1	MBTB29 - Bric-a-Brac, Tramtrack, Broad Complex BTB domain with Meprin and TRAF Homology MATH domain, expressed	1,05	-1.0	1	21	47	67	AGACUUAUAUGUAGGAACGGA	UCCGUUCCUAAAUAUAAGUCU	Translation
tae-miR9662a-3p	Traes_1BS_566CEE7F6.1	PF02536				GO:GO:0006355,GO:GO:0005739,GO:GO:0003690	AT1G21150.1	Mitochondrial transcription termination factor family protein	LOC_Os06g12100.1	mTERF family protein, expressed	2,00	-1.0	1	21	441	461	UUGAACAUCCCAGAGCCACCG	UCGUGGCGCUGGGAUGUUCAG	Cleavage
tae-miR9662b-3p	Traes_1BS_566CEE7F6.1	PF02536				GO:GO:0006355,GO:GO:0005739,GO:GO:0003690	AT1G21150.1	Mitochondrial transcription termination factor family protein	LOC_Os06g12100.1	mTERF family protein, expressed	2,05	-1.0	1	21	440	460	UGAACAUCCCAGAGCCACCGG	CUCGUGGCGCUGGGAUGUUCA	Cleavage
tae-miR9662a-3p	Traes_1BS_566CEE7F6.2	PF02536				GO:GO:0006355,GO:GO:0005739,GO:GO:0003690	AT1G21150.1	Mitochondrial transcription termination factor family protein	LOC_Os06g12100.1	mTERF family protein, expressed	2,00	-1.0	1	21	66	86	UUGAACAUCCCAGAGCCACCG	UCGUGGCGCUGGGAUGUUCAG	Cleavage
tae-miR9662b-3p	Traes_1BS_566CEE7F6.2	PF02536				GO:GO:0006355,GO:GO:0005739,GO:GO:0003690	AT1G21150.1	Mitochondrial transcription termination factor family protein	LOC_Os06g12100.1	mTERF family protein, expressed	2,05	-1.0	1	21	65	85	UGAACAUCCCAGAGCCACCGG	CUCGUGGCGCUGGGAUGUUCA	Cleavage
tae-miR9778	Traes_1BS_70B733D34.1	PF13191,PF00931				GO:GO:0043531	AT3G07040.1	NB-ARC domain-containing disease resistance protein	LOC_Os08g42670.1	resistance protein, putative, expressed	2,00	-1.0	1	21	126	146	UGCAUCAUCUCGAACUCGUCG	CAAUGAGCUCGAGAUGAUGCA	Cleavage
tae-miR9778	Traes_1BS_70B733D34.2	PF13191,PF00931				GO:GO:0043531	AT3G07040.1	NB-ARC domain-containing disease resistance protein	LOC_Os08g42670.2	resistance protein, putative, expressed	2,00	-1.0	1	21	126	146	UGCAUCAUCUCGAACUCGUCG	CAAUGAGCUCGAGAUGAUGCA	Cleavage
tae-miR5049-3p	Traes_1BS_7EC2BDE95.1	PF16760		2.4.1.21			AT1G11720.2	starch synthase 3	LOC_Os08g09230.1	starch synthase III, putative, expressed	1,05	-1.0	1	21	3430	3450	AAUAUGGAUCGGAGGGAGUAC	CUACUCCCUCCAAUCCAUAUU	Translation
tae-miR5049-3p	Traes_1BS_B0BB95B97.6	PF04997		2.7.7.6		GO:GO:0006351,GO:GO:0003899,GO:GO:0003677	AT1G63020.2	nuclear RNA polymerase D1A	LOC_Os02g05880.1	RNA polymerase Rpb1, domain 2 family protein, expressed	0,00	-1.0	1	21	623	643	AAUAUGGAUCGGAGGGAGUAC	GUACUCCCUCCGAUCCAUAUU	Cleavage
tae-miR7757-5p	Traes_1BS_CF1C4F54E.2	PF00931				GO:GO:0043531	AT1G09920.1	TRAF-type zinc finger-related	LOC_Os11g45750.1	WRKY125, expressed	2,05	-1.0	1	22	119	140	AUAAAACCUUCAGCUAUCCAUC	AAUGGUUAGCUGAAGGUUUCAU	Cleavage
tae-miR159a	Traes_1DL_25540DE53.1						AT2G32460.1	myb domain protein 101	LOC_Os01g59660.2	MYB family transcription factor, putative, expressed	2,05	-1.0	1	21	512	532	UUUGGAUUGAAGGGAGCUCUG	UGGAGCUCCCUUCAUUCCAAU	Cleavage
tae-miR159b	Traes_1DL_25540DE53.1						AT2G32460.1	myb domain protein 101	LOC_Os01g59660.2	MYB family transcription factor, putative, expressed	2,05	-1.0	1	21	512	532	UUUGGAUUGAAGGGAGCUCUG	UGGAGCUCCCUUCAUUCCAAU	Cleavage
tae-miR159a	Traes_1DL_25540DE53.2						AT2G32460.1	myb domain protein 101	LOC_Os01g59660.1	MYB family transcription factor, putative, expressed	2,05	-1.0	1	21	512	532	UUUGGAUUGAAGGGAGCUCUG	UGGAGCUCCCUUCAUUCCAAU	Cleavage
tae-miR159b	Traes_1DL_25540DE53.2						AT2G32460.1	myb domain protein 101	LOC_Os01g59660.1	MYB family transcription factor, putative, expressed	2,05	-1.0	1	21	512	532	UUUGGAUUGAAGGGAGCUCUG	UGGAGCUCCCUUCAUUCCAAU	Cleavage
tae-miR159a	Traes_1DL_25540DE53.3						AT5G06100.3	myb domain protein 33	LOC_Os01g59660.1	MYB family transcription factor, putative, expressed	2,05	-1.0	1	21	470	490	UUUGGAUUGAAGGGAGCUCUG	UGGAGCUCCCUUCAUUCCAAU	Cleavage
tae-miR159b	Traes_1DL_25540DE53.3						AT5G06100.3	myb domain protein 33	LOC_Os01g59660.1	MYB family transcription factor, putative, expressed	2,05	-1.0	1	21	470	490	UUUGGAUUGAAGGGAGCUCUG	UGGAGCUCCCUUCAUUCCAAU	Cleavage
tae-miR159a	Traes_1DL_25540DE53.4						AT5G06100.3	myb domain protein 33	LOC_Os01g59660.2	MYB family transcription factor, putative, expressed	2,05	-1.0	1	21	470	490	UUUGGAUUGAAGGGAGCUCUG	UGGAGCUCCCUUCAUUCCAAU	Cleavage
tae-miR159b	Traes_1DL_25540DE53.4						AT5G06100.3	myb domain protein 33	LOC_Os01g59660.2	MYB family transcription factor, putative, expressed	2,05	-1.0	1	21	470	490	UUUGGAUUGAAGGGAGCUCUG	UGGAGCUCCCUUCAUUCCAAU	Cleavage
tae-miR159a	Traes_1DL_25540DE53.5						AT5G06100.2	myb domain protein 33	LOC_Os01g59660.2	MYB family transcription factor, putative, expressed	2,05	-1.0	1	21	470	490	UUUGGAUUGAAGGGAGCUCUG	UGGAGCUCCCUUCAUUCCAAU	Cleavage
tae-miR159b	Traes_1DL_25540DE53.5						AT5G06100.2	myb domain protein 33	LOC_Os01g59660.2	MYB family transcription factor, putative, expressed	2,05	-1.0	1	21	470	490	UUUGGAUUGAAGGGAGCUCUG	UGGAGCUCCCUUCAUUCCAAU	Cleavage
tae-miR159a	Traes_1DL_25540DE53.6						AT5G06100.2	myb domain protein 33	LOC_Os01g59660.1	MYB family transcription factor, putative, expressed	2,05	-1.0	1	21	470	490	UUUGGAUUGAAGGGAGCUCUG	UGGAGCUCCCUUCAUUCCAAU	Cleavage
tae-miR159b	Traes_1DL_25540DE53.6						AT5G06100.2	myb domain protein 33	LOC_Os01g59660.1	MYB family transcription factor, putative, expressed	2,05	-1.0	1	21	470	490	UUUGGAUUGAAGGGAGCUCUG	UGGAGCUCCCUUCAUUCCAAU	Cleavage
tae-miR1137a	Traes_1DL_34A359364.2	PF09439	KOG0070		K07937		AT5G14670.1	ADP-ribosylation factor A1B	LOC_Os03g59740.1	ADP-ribosylation factor, putative, expressed	2,05	-1.0	1	20	516	535	UAGUACAAAGUUGAGUCAUC	GAUGACCCAAUUUUGUACUA	Translation
tae-miR5062-5p	Traes_1DL_38415F71A.19						AT2G46060.2	transmembrane protein-related	LOC_Os10g30910.2	transmembrane protein-related, putative, expressed	2,05	-1.0	1	23	836	858	UGAACCUUAGGGAACAGCCGCAU	UUUUGGAUGUUCCCCAAGGUUCA	Cleavage
tae-miR5062-5p	Traes_1DL_38415F71A.20						AT2G46060.2	transmembrane protein-related	LOC_Os10g30910.2	transmembrane protein-related, putative, expressed	2,05	-1.0	1	23	836	858	UGAACCUUAGGGAACAGCCGCAU	UUUUGGAUGUUCCCCAAGGUUCA	Cleavage
tae-miR5062-5p	Traes_1DL_38415F71A.22						AT2G46060.1	transmembrane protein-related	LOC_Os10g30910.2	transmembrane protein-related, putative, expressed	2,05	-1.0	1	23	836	858	UGAACCUUAGGGAACAGCCGCAU	UUUUGGAUGUUCCCCAAGGUUCA	Cleavage
tae-miR1122a	Traes_1DL_38B88249C.2	PF00069	KOG0580	2.7.11.1		GO:GO:0006468,GO:GO:0005524,GO:GO:0004672	AT2G42960.1	Protein kinase superfamily protein	LOC_Os10g35450.1	serine/threonine-protein kinase, putative, expressed	0,00	-1.0	1	20	976	995	UAGAUACAUCCGUAUCUAGA	UCUAGAUACGGAUGUAUCUA	Cleavage
tae-miR1120a	Traes_1DL_6A91BC5A5.1	PF08652			K14845		AT4G17620.1	glycine-rich protein	LOC_Os05g32580.1	glycine-rich protein, putative, expressed	2,05	-1.0	1	24	25	48	ACAUUCUUAUAUUAUGAGACGGAG	CUCCGUCCCAUAAUAUAAGAACGU	Cleavage
tae-miR1130b-3p	Traes_1DL_6A91BC5A5.1	PF08652			K14845		AT4G17620.1	glycine-rich protein	LOC_Os05g32580.1	glycine-rich protein, putative, expressed	0,00	-1.0	1	21	24	44	UCUUAUAUUAUGGGACGGAGG	CCUCCGUCCCAUAAUAUAAGA	Cleavage
tae-miR1120b-3p	Traes_1DL_6A91BC5A5.1	PF08652			K14845		AT4G17620.1	glycine-rich protein	LOC_Os05g32580.1	glycine-rich protein, putative, expressed	2,05	-1.0	1	21	25	45	UUCUUAUAUUGUGGGACAGAG	CUCCGUCCCAUAAUAUAAGAA	Translation
tae-miR1122c-3p	Traes_1DL_6A91BC5A5.1	PF08652			K14845		AT4G17620.1	glycine-rich protein	LOC_Os05g32580.1	glycine-rich protein, putative, expressed	1,05	-1.0	1	21	24	44	UCUAAUAUUAUGGGACGGAGG	CCUCCGUCCCAUAAUAUAAGA	Cleavage
tae-miR1120c-5p	Traes_1DL_6A91BC5A5.1	PF08652			K14845		AT4G17620.1	glycine-rich protein	LOC_Os05g32580.1	glycine-rich protein, putative, expressed	2,05	-1.0	1	21	67	87	UAAUAUAAGAACGUUUUUGAC	GUCAAACACGCUCUUAUAUUA	Translation
tae-miR160	Traes_1DL_AEC2F3FB3.1	PF06507				GO:GO:0009725,GO:GO:0006355,GO:GO:0005634,GO:GO:0003677	AT4G30080.1	auxin response factor 16	LOC_Os10g33940.1	auxin response factor 18, putative, expressed	0,00	-1.0	1	21	642	662	UGCCUGGCUCCCUGUAUGCCA	AGGCAUACAGGGAGCCAGGCA	Cleavage
tae-miR1122b-3p	Traes_1DL_B5E6E273B.1										1,05	-1.0	1	21	35	55	AGACUUAUAUGUAGGAACGGA	UCCGUUCCUAAAUAUAAGUCU	Translation
tae-miR5175-5p	Traes_1DL_C1BE1742F.2	PF03641					AT3G53450.1	Putative lysine decarboxylase family protein	LOC_Os10g33900.1	possible lysine decarboxylase domain containing protein, expressed	2,00	-1.0	1	21	109	130	UUCCAAAUUACUCGUCG-UGGU	UUCAGCGACGAGUAAUUUGGAA	Cleavage
tae-miR1133	Traes_1DL_D391AE3DC.1	PF16212		3.6.3.1		GO:GO:0016021,GO:GO:0015914,GO:GO:0005524,GO:GO:0004012,GO:GO:0000287	AT1G59820.1	aminophospholipid ATPase 3	LOC_Os10g27220.1	phospholipid-transporting ATPase 3, putative, expressed	2,00	-1.0	1	22	858	879	CAUAUACUCCCUCCGUCCGAAA	AAAUGAACGGAGGGAGUAUAUG	Cleavage
tae-miR1133	Traes_1DL_D391AE3DC.2	PF16212		3.6.3.1		GO:GO:0016021,GO:GO:0015914,GO:GO:0005524,GO:GO:0004012,GO:GO:0000287	AT1G59820.1	aminophospholipid ATPase 3	LOC_Os10g27220.1	phospholipid-transporting ATPase 3, putative, expressed	2,00	-1.0	1	22	858	879	CAUAUACUCCCUCCGUCCGAAA	AAAUGAACGGAGGGAGUAUAUG	Cleavage
tae-miR1133	Traes_1DL_D391AE3DC.3	PF16212		3.6.3.1		GO:GO:0016021,GO:GO:0015914,GO:GO:0005524,GO:GO:0004012,GO:GO:0000287	AT1G59820.1	aminophospholipid ATPase 3	LOC_Os10g27220.1	phospholipid-transporting ATPase 3, putative, expressed	2,00	-1.0	1	22	822	843	CAUAUACUCCCUCCGUCCGAAA	AAAUGAACGGAGGGAGUAUAUG	Cleavage
tae-miR1128	Traes_1DL_E30609261.7						AT1G04300.3	TRAF-like superfamily protein	LOC_Os05g43280.1	MATH domain containing protein, expressed	3,00	-1.0	1	21	76	96	UACUACUCCCUCCGUCCGAAA	UUCCAGACGGAGGGAGUAGUU	Cleavage
tae-miR1122a	Traes_1DS_06B8348E0.1	PF03283				GO:GO:0016787	AT3G09410.1	Pectinacetylesterase family protein	LOC_Os05g02120.1	pectinacetylesterase domain containing protein, expressed	0,00	-1.0	1	20	794	813	UAGAUACAUCCGUAUCUAGA	UCUAGAUACGGAUGUAUCUA	Cleavage
tae-miR1122a	Traes_1DS_06B8348E0.2	PF03283				GO:GO:0016787	AT3G09410.1	Pectinacetylesterase family protein	LOC_Os05g02120.1	pectinacetylesterase domain containing protein, expressed	0,00	-1.0	1	20	620	639	UAGAUACAUCCGUAUCUAGA	UCUAGAUACGGAUGUAUCUA	Cleavage
tae-miR5048-5p	Traes_1DS_26F1546F6.1	PF00168,PF07714,PF00069	KOG1187	2.7.11.1		GO:GO:0005515,GO:GO:0006468,GO:GO:0004672,GO:GO:0005524	AT1G63430.1	Leucine-rich repeat protein kinase family protein	LOC_Os04g58700.1	expressed protein	2,05	-1.0	1	22	917	938	UUUGCAGGUUUUAGGUCUAAGU	ACUUGGACCUAAAACCUGCCAA	Cleavage
tae-miR5048-5p	Traes_1DS_26F1546F6.2	PF00168,PF07714,PF00069	KOG1187	2.7.11.1		GO:GO:0005515,GO:GO:0006468,GO:GO:0004672,GO:GO:0005524	AT1G63430.1	Leucine-rich repeat protein kinase family protein	LOC_Os04g58700.1	expressed protein	2,05	-1.0	1	22	917	938	UUUGCAGGUUUUAGGUCUAAGU	ACUUGGACCUAAAACCUGCCAA	Cleavage
tae-miR7757-5p	Traes_1DS_35EF41CCB.2	PF00931				GO:GO:0043531	AT3G50950.1	HOPZ-ACTIVATED RESISTANCE 1	LOC_Os05g40150.1	RGH2B, putative, expressed	1,05	-1.0	1	22	587	608	AUAAAACCUUCAGCUAUCCAUC	AGUGGAUAGCUGAAGGUUUCAU	Cleavage
tae-miR9674a-5p	Traes_1DS_F3DE3946F.1	PF13041					AT1G62930.1	Tetratricopeptide repeat (TPR)-like superfamily protein	LOC_Os04g28300.1	Rf1, mitochondrial precursor, putative, expressed	2,05	-1.0	1	21	522	542	GCAUCAUCCAUCCUACCAUUC	AAAUGGAAGGAUCGAUGAUGC	Cleavage
tae-miR5049-3p	Traes_1DS_FA7FC5C3C.1	PF04997		2.7.7.6		GO:GO:0006351,GO:GO:0003899,GO:GO:0003677	AT1G63020.1	nuclear RNA polymerase D1A	LOC_Os02g05880.1	RNA polymerase Rpb1, domain 2 family protein, expressed	0,00	-1.0	1	21	623	643	AAUAUGGAUCGGAGGGAGUAC	GUACUCCCUCCGAUCCAUAUU	Cleavage
tae-miR1128	Traes_2AL_24A1BF00C.1						AT5G06210.1	RNA binding (RRM/RBD/RNP motifs) family protein	LOC_Os04g33810.1	RNA recognition motif containing protein, putative, expressed	0,00	-1.0	1	21	37	57	UACUACUCCCUCCGUCCGAAA	UUUCGGACGGAGGGAGUAGUA	Cleavage
tae-miR5049-3p	Traes_2AL_28B13CC56.1					GO:GO:0005681,GO:GO:0000398	AT1G80070.1	Pre-mRNA-processing-splicing factor	LOC_Os06g07080.1	pre-mRNA-processing-splicing factor 8, putative, expressed	1,05	-1.0	1	21	346	366	AAUAUGGAUCGGAGGGAGUAC	CUACUCCCUCCGUUCCAUAUU	Cleavage
tae-miR1127a	Traes_2AL_4197D219B.1						AT3G16200.1		LOC_Os01g31220.1	expressed protein	2,05	-1.0	1	19	991	1009	UCCUUCCGUUCGGAAUUAC	GUAAUUCCGAACGGAGGGU	Cleavage
tae-miR164	Traes_2AL_57BF37A86.1	PF02365				GO:GO:0006355,GO:GO:0003677	AT5G61430.1	NAC domain containing protein 100	LOC_Os04g38720.1	no apical meristem protein, putative, expressed	1,05	-1.0	1	21	680	700	UGGAGAAGCAGGGCACGUGCA	CGCACGUGACCUGCUUCUCCA	Cleavage
tae-miR164	Traes_2AL_57BF37A86.2	PF02365				GO:GO:0006355,GO:GO:0003677	AT5G61430.1	NAC domain containing protein 100	LOC_Os04g38720.1	no apical meristem protein, putative, expressed	1,05	-1.0	1	21	606	626	UGGAGAAGCAGGGCACGUGCA	CGCACGUGACCUGCUUCUCCA	Cleavage
tae-miR1122a	Traes_2AL_64B73E3ED.1			2.8.1.8		GO:GO:0051539,GO:GO:0016992,GO:GO:0009107	AT2G20860.1	lipoic acid synthase 1	LOC_Os04g38330.1	radical SAM enzyme, putative, expressed	1,00	-1.0	1	20	121	140	UAGAUACAUCCGUAUCUAGA	UCUAAAUACGGAUGUAUCUA	Cleavage
tae-miR1120b-3p	Traes_2AL_673CA8FB2.1	PF04603	KOG3329				AT1G69680.1	Mog1/PsbP/DUF1795-like photosystem II reaction center PsbP family protein	LOC_Os04g38610.2	RAN guanine nucleotide release factor, putative, expressed	2,05	-1.0	1	21	493	513	UUCUUAUAUUGUGGGACAGAG	CUCUGUCCCACAAUGUAAGAC	Cleavage
tae-miR1133	Traes_2AL_673CA8FB2.1	PF04603	KOG3329				AT1G69680.1	Mog1/PsbP/DUF1795-like photosystem II reaction center PsbP family protein	LOC_Os04g38610.2	RAN guanine nucleotide release factor, putative, expressed	2,05	-1.0	1	22	553	575	CAUAUACUCCCUCCGUCCGA-AA	UUGUGGGACGGAGGGAGUAUUUG	Cleavage
tae-miR1120a	Traes_2AL_979E96822.1	PF00443		3.4.19.12		GO:GO:0036459,GO:GO:0016579	AT4G39370.3	ubiquitin-specific protease 27	LOC_Os04g34984.1	ubiquitin carboxyl-terminal hydrolase domain containing protein, expressed	2,05	-1.0	1	24	753	776	ACAUUCUUAUAUUAUGAGACGGAG	CUCCAUCCCAUAAUAUAAGAGUGU	Cleavage
tae-miR1130b-3p	Traes_2AL_979E96822.1	PF00443		3.4.19.12		GO:GO:0036459,GO:GO:0016579	AT4G39370.3	ubiquitin-specific protease 27	LOC_Os04g34984.1	ubiquitin carboxyl-terminal hydrolase domain containing protein, expressed	1,00	-1.0	1	21	752	772	UCUUAUAUUAUGGGACGGAGG	CCUCCAUCCCAUAAUAUAAGA	Cleavage
tae-miR1122c-3p	Traes_2AL_979E96822.1	PF00443		3.4.19.12		GO:GO:0036459,GO:GO:0016579	AT4G39370.3	ubiquitin-specific protease 27	LOC_Os04g34984.1	ubiquitin carboxyl-terminal hydrolase domain containing protein, expressed	2,05	-1.0	1	21	752	772	UCUAAUAUUAUGGGACGGAGG	CCUCCAUCCCAUAAUAUAAGA	Cleavage
tae-miR1120c-5p	Traes_2AL_979E96822.1	PF00443		3.4.19.12		GO:GO:0036459,GO:GO:0016579	AT4G39370.3	ubiquitin-specific protease 27	LOC_Os04g34984.1	ubiquitin carboxyl-terminal hydrolase domain containing protein, expressed	2,05	-1.0	1	21	795	815	UAAUAUAAGAACGUUUUUGAC	GUAAAAAACGCUCUUAUAUUA	Translation
tae-miR160	Traes_2AL_C70217249.1	PF06507,PF02362				GO:GO:0009725,GO:GO:0006355,GO:GO:0005634,GO:GO:0003677	AT1G77850.1	auxin response factor 17	LOC_Os04g59430.1	auxin response factor, putative, expressed	1,00	-1.0	1	21	1164	1184	UGCCUGGCUCCCUGUAUGCCA	UGGCAUGCAGGGAGCCAGGCA	Cleavage
tae-miR1134	Traes_2AL_D2CC35E59.1										3,00	-1.0	1	24	512	535	CAACAACAACAAGAAGAAGAAGAU	AUCUUCAUGGUCUUGUUGUUGUUG	Cleavage
tae-miR1134	Traes_2AL_D2CC35E59.2										3,00	-1.0	1	24	620	643	CAACAACAACAAGAAGAAGAAGAU	AUCUUCAUGGUCUUGUUGUUGUUG	Cleavage
tae-miR1134	Traes_2AL_D2CC35E59.3										3,00	-1.0	1	24	509	532	CAACAACAACAAGAAGAAGAAGAU	AUCUUCAUGGUCUUGUUGUUGUUG	Cleavage
tae-miR1134	Traes_2AL_D2CC35E59.4										3,00	-1.0	1	24	611	634	CAACAACAACAAGAAGAAGAAGAU	AUCUUCAUGGUCUUGUUGUUGUUG	Cleavage
tae-miR1134	Traes_2AL_D2CC35E59.5										3,00	-1.0	1	24	509	532	CAACAACAACAAGAAGAAGAAGAU	AUCUUCAUGGUCUUGUUGUUGUUG	Cleavage
tae-miR1134	Traes_2AL_D2CC35E59.6										3,00	-1.0	1	24	509	532	CAACAACAACAAGAAGAAGAAGAU	AUCUUCAUGGUCUUGUUGUUGUUG	Cleavage
tae-miR5384-3p	Traes_2AL_D79C9535C.1	PF00067	KOG0157	1.14.13.109		GO:GO:0055114,GO:GO:0020037,GO:GO:0016705,GO:GO:0005506	AT5G36110.1	cytochrome P450, family 716, subfamily A, polypeptide 1	LOC_Os09g21260.1	cytochrome P450 domain containing protein, expressed	2,05	-1.0	1	21	1250	1270	UGAGCGCGCCGCCGUCGAAUG	CGUUCGGCGGCGGCGCGCGCA	Cleavage
tae-miR9778	Traes_2AS_530CF4042.10	PF00931				GO:GO:0043531	AT3G50950.1	HOPZ-ACTIVATED RESISTANCE 1	LOC_Os04g12830.1	expressed protein	2,05	-1.0	1	21	126	146	UGCAUCAUCUCGAACUCGUCG	CGGCGAGUUCCAGAUGAUGCA	Translation
tae-miR9778	Traes_2AS_530CF4042.2	PF00931				GO:GO:0043531	AT3G50950.1	HOPZ-ACTIVATED RESISTANCE 1	LOC_Os04g12830.1	expressed protein	2,05	-1.0	1	21	126	146	UGCAUCAUCUCGAACUCGUCG	CGGCGAGUUCCAGAUGAUGCA	Translation
tae-miR9778	Traes_2AS_530CF4042.3	PF00931				GO:GO:0043531	AT3G50950.1	HOPZ-ACTIVATED RESISTANCE 1	LOC_Os04g12830.1	expressed protein	2,05	-1.0	1	21	126	146	UGCAUCAUCUCGAACUCGUCG	CGGCGAGUUCCAGAUGAUGCA	Translation
tae-miR1130b-3p	Traes_2AS_6DCA4AADD.1					GO:GO:0016020,GO:GO:0008236,GO:GO:0006508	AT2G31140.1	Peptidase S24/S26A/S26B/S26C family protein	LOC_Os04g01240.1	serine-type peptidase, putative, expressed	1,05	-1.0	1	21	288	308	UCUUAUAUUAUGGGACGGAGG	CCUCCGUCCCAUAAUAUAGGA	Cleavage
tae-miR9664-3p	Traes_2AS_86633353C.1	PF00931	KOG4658		K13457	GO:GO:0043531	AT3G07040.1	NB-ARC domain-containing disease resistance protein	LOC_Os11g46210.1	NB-ARC domain containing protein, expressed	2,05	-1.0	1	21	240	260	UUGCAGUCCUCGAUGUCGUAG	CUACGACAUCGAGGAUUGCAU	Cleavage
tae-miR1130b-3p	Traes_2BL_171777733.1	PF00443		3.4.19.12		GO:GO:0036459,GO:GO:0016579	AT4G39370.3	ubiquitin-specific protease 27	LOC_Os04g34984.1	ubiquitin carboxyl-terminal hydrolase domain containing protein, expressed	0,00	-1.0	1	21	752	772	UCUUAUAUUAUGGGACGGAGG	CCUCCGUCCCAUAAUAUAAGA	Cleavage
tae-miR1122c-3p	Traes_2BL_171777733.1	PF00443		3.4.19.12		GO:GO:0036459,GO:GO:0016579	AT4G39370.3	ubiquitin-specific protease 27	LOC_Os04g34984.1	ubiquitin carboxyl-terminal hydrolase domain containing protein, expressed	1,05	-1.0	1	21	752	772	UCUAAUAUUAUGGGACGGAGG	CCUCCGUCCCAUAAUAUAAGA	Cleavage
tae-miR1120c-5p	Traes_2BL_171777733.1	PF00443		3.4.19.12		GO:GO:0036459,GO:GO:0016579	AT4G39370.3	ubiquitin-specific protease 27	LOC_Os04g34984.1	ubiquitin carboxyl-terminal hydrolase domain containing protein, expressed	2,05	-1.0	1	21	795	815	UAAUAUAAGAACGUUUUUGAC	GUAAAAAACGCUCUUAUAUUA	Translation
tae-miR5384-3p	Traes_2BL_1BE5C9C71.1	PF00067		1.14.13.109		GO:GO:0055114,GO:GO:0020037,GO:GO:0016705,GO:GO:0005506	AT2G42850.1	cytochrome P450, family 718	LOC_Os09g21260.1	cytochrome P450 domain containing protein, expressed	2,05	-1.0	1	21	455	475	UGAGCGCGCCGCCGUCGAAUG	CGUUCGGCGGCGGCGCGCGCA	Cleavage
tae-miR5384-3p	Traes_2BL_1BE5C9C71.2	PF00067				GO:GO:0055114,GO:GO:0020037,GO:GO:0016705,GO:GO:0005506	AT5G36110.1	cytochrome P450, family 716, subfamily A, polypeptide 1	LOC_Os09g21260.1	cytochrome P450 domain containing protein, expressed	2,05	-1.0	1	21	455	475	UGAGCGCGCCGCCGUCGAAUG	CGUUCGGCGGCGGCGCGCGCA	Cleavage
tae-miR1122a	Traes_2BL_40442CE82.2	PF14392,PF00098				GO:GO:0008270,GO:GO:0003676	AT5G52380.1	VASCULAR-RELATED NAC-DOMAIN 6	LOC_Os04g46920.1	zinc knuckle domain containing protein, expressed	1,05	-1.0	1	20	703	722	UAGAUACAUCCGUAUCUAGA	UCUAGAUACAGAUGUAUCUA	Translation
tae-miR164	Traes_2BL_6AEE8AC28.1	PF02365				GO:GO:0006355,GO:GO:0003677	AT5G61430.1	NAC domain containing protein 100	LOC_Os04g38720.1	no apical meristem protein, putative, expressed	1,05	-1.0	1	21	680	700	UGGAGAAGCAGGGCACGUGCA	CGCACGUGACCUGCUUCUCCA	Cleavage
tae-miR164	Traes_2BL_6AEE8AC28.2	PF02365				GO:GO:0006355,GO:GO:0003677	AT5G61430.1	NAC domain containing protein 100	LOC_Os04g38720.1	no apical meristem protein, putative, expressed	1,05	-1.0	1	21	672	692	UGGAGAAGCAGGGCACGUGCA	CGCACGUGACCUGCUUCUCCA	Cleavage
tae-miR164	Traes_2BL_6AEE8AC28.3	PF02365				GO:GO:0006355,GO:GO:0003677	AT5G61430.1	NAC domain containing protein 100	LOC_Os04g38720.1	no apical meristem protein, putative, expressed	1,05	-1.0	1	21	633	653	UGGAGAAGCAGGGCACGUGCA	CGCACGUGACCUGCUUCUCCA	Cleavage
tae-miR1122b-3p	Traes_2BL_7141904F2.2	PF03097					AT1G73390.1	Endosomal targeting BRO1-like domain-containing protein	LOC_Os04g44530.3	expressed protein	2,05	-1.0	1	21	1058	1078	AGACUUAUAUGUAGGAACGGA	UCCGUUCUUAAAUAUAAGUCU	Translation
tae-miR1122a	Traes_2BL_7141904F2.2	PF03097					AT1G73390.1	Endosomal targeting BRO1-like domain-containing protein	LOC_Os04g44530.3	expressed protein	2,05	-1.0	1	20	1098	1117	UAGAUACAUCCGUAUCUAGA	ACUACAUACGGAUGUAUAUA	Cleavage
tae-miR1134	Traes_2BL_8339D35AC.1										3,00	-1.0	1	24	509	532	CAACAACAACAAGAAGAAGAAGAU	AUCUUCAUGGUCUUGUUGUUGUUG	Cleavage
tae-miR1134	Traes_2BL_8339D35AC.2										3,00	-1.0	1	24	512	535	CAACAACAACAAGAAGAAGAAGAU	AUCUUCAUGGUCUUGUUGUUGUUG	Cleavage
tae-miR1134	Traes_2BL_8339D35AC.3										3,00	-1.0	1	24	509	532	CAACAACAACAAGAAGAAGAAGAU	AUCUUCAUGGUCUUGUUGUUGUUG	Cleavage
tae-miR1134	Traes_2BL_8339D35AC.4										3,00	-1.0	1	24	302	325	CAACAACAACAAGAAGAAGAAGAU	AUCUUCAUGGUCUUGUUGUUGUUG	Cleavage
tae-miR1134	Traes_2BL_8339D35AC.5										3,00	-1.0	1	24	509	532	CAACAACAACAAGAAGAAGAAGAU	AUCUUCAUGGUCUUGUUGUUGUUG	Cleavage
tae-miR1135	Traes_2BL_A4AE931B0.1						AT2G02090.1	SNF2 domain-containing protein / helicase domain-containing protein	LOC_Os04g47830.1	SNF2 family N-terminal domain containing protein, expressed	2,00	-1.0	1	24	1154	1177	CUGCGACAAGUAAUUCCGAACGGA	UCUGUCCGGAAUUACUUGUCGCAA	Cleavage
tae-miR1122b-3p	Traes_2BL_FF8002798.2	PF13414					AT1G56090.1	Tetratricopeptide repeat (TPR)-like superfamily protein	LOC_Os04g52580.1	tetratricopeptide repeat domain containing protein, expressed	2,05	-1.0	1	21	889	909	AGACUUAUAUGUAGGAACGGA	UCGGUUCCUAAAUAUAAGUCU	Translation
tae-miR9778	Traes_2BS_0EFFAAE28.1	PF00931				GO:GO:0043531	AT3G07040.1	NB-ARC domain-containing disease resistance protein	LOC_Os08g14850.1	resistance protein, putative, expressed	2,05	-1.0	1	21	126	146	UGCAUCAUCUCGAACUCGUCG	CGGCGAGUUCCAGAUGAUGCA	Translation
tae-miR9778	Traes_2BS_0EFFAAE28.2	PF00931				GO:GO:0043531	AT3G07040.1	NB-ARC domain-containing disease resistance protein	LOC_Os08g14850.1	resistance protein, putative, expressed	2,05	-1.0	1	21	126	146	UGCAUCAUCUCGAACUCGUCG	CGGCGAGUUCCAGAUGAUGCA	Translation
tae-miR9662a-3p	Traes_2BS_257C5B099.1	PF02536				GO:GO:0006355,GO:GO:0005739,GO:GO:0003690	AT5G07900.1	Mitochondrial transcription termination factor family protein	LOC_Os06g12100.1	mTERF family protein, expressed	2,00	-1.0	1	21	66	86	UUGAACAUCCCAGAGCCACCG	CCGUGGUUCUGGGAUGUUCAG	Cleavage
tae-miR9662b-3p	Traes_2BS_257C5B099.1	PF02536				GO:GO:0006355,GO:GO:0005739,GO:GO:0003690	AT5G07900.1	Mitochondrial transcription termination factor family protein	LOC_Os06g12100.1	mTERF family protein, expressed	2,00	-1.0	1	21	65	85	UGAACAUCCCAGAGCCACCGG	CCCGUGGUUCUGGGAUGUUCA	Cleavage
tae-miR9662a-3p	Traes_2BS_257C5B0991.1	PF02536				GO:GO:0006355,GO:GO:0005739,GO:GO:0003690	AT5G07900.1	Mitochondrial transcription termination factor family protein	LOC_Os06g12100.1	mTERF family protein, expressed	2,00	-1.0	1	21	66	86	UUGAACAUCCCAGAGCCACCG	CCGUGGUUCUGGGAUGUUCAG	Cleavage
tae-miR9662b-3p	Traes_2BS_257C5B0991.1	PF02536				GO:GO:0006355,GO:GO:0005739,GO:GO:0003690	AT5G07900.1	Mitochondrial transcription termination factor family protein	LOC_Os06g12100.1	mTERF family protein, expressed	2,00	-1.0	1	21	65	85	UGAACAUCCCAGAGCCACCGG	CCCGUGGUUCUGGGAUGUUCA	Cleavage
tae-miR5048-5p	Traes_2BS_47E2D6444.1	PF07714		2.7.11.1		GO:GO:0006468,GO:GO:0004672	AT4G04570.2	cysteine-rich RLK (RECEPTOR-like protein kinase) 40	LOC_Os04g30030.1	cysteine-rich receptor-like protein kinase 12 precursor, putative, expressed	2,05	-1.0	1	22	221	242	UUUGCAGGUUUUAGGUCUAAGU	ACCUGGACCUAAAACCUGCGAA	Cleavage
tae-miR5048-5p	Traes_2BS_47E2D6444.2	PF00069	KOG1187	2.7.11.1		GO:GO:0006468,GO:GO:0005524,GO:GO:0004672	AT4G38830.1	cysteine-rich RLK (RECEPTOR-like protein kinase) 26	LOC_Os04g30030.1	cysteine-rich receptor-like protein kinase 12 precursor, putative, expressed	2,05	-1.0	1	22	959	980	UUUGCAGGUUUUAGGUCUAAGU	ACCUGGACCUAAAACCUGCGAA	Cleavage
tae-miR5048-5p	Traes_2BS_47E2D6444.3	PF00069	KOG1187	2.7.11.1		GO:GO:0006468,GO:GO:0005524,GO:GO:0004672	AT4G38830.1	cysteine-rich RLK (RECEPTOR-like protein kinase) 26	LOC_Os04g30030.1	cysteine-rich receptor-like protein kinase 12 precursor, putative, expressed	2,05	-1.0	1	22	947	968	UUUGCAGGUUUUAGGUCUAAGU	ACCUGGACCUAAAACCUGCGAA	Cleavage
tae-miR5048-5p	Traes_2BS_47E2D6444.4	PF07714		2.7.11.1		GO:GO:0006468,GO:GO:0004672	AT4G23160.1	cysteine-rich RLK (RECEPTOR-like protein kinase) 8	LOC_Os04g30030.1	cysteine-rich receptor-like protein kinase 12 precursor, putative, expressed	2,05	-1.0	1	22	221	242	UUUGCAGGUUUUAGGUCUAAGU	ACCUGGACCUAAAACCUGCGAA	Cleavage
tae-miR9664-3p	Traes_2BS_5045C640C.1	PF00931	KOG4658		K13457	GO:GO:0043531	AT3G07040.1	NB-ARC domain-containing disease resistance protein	LOC_Os11g46210.1	NB-ARC domain containing protein, expressed	2,05	-1.0	1	21	240	260	UUGCAGUCCUCGAUGUCGUAG	CUACGACAUCGAGGAUUGCAU	Cleavage
tae-miR9664-3p	Traes_2BS_5045C640C.2	PF00931	KOG4658		K13457	GO:GO:0043531	AT3G07040.1	NB-ARC domain-containing disease resistance protein	LOC_Os11g46210.1	NB-ARC domain containing protein, expressed	2,05	-1.0	1	21	240	260	UUGCAGUCCUCGAUGUCGUAG	CUACGACAUCGAGGAUUGCAU	Cleavage
tae-miR9664-3p	Traes_2BS_5045C640C.5	PF00931	KOG4658		K13457	GO:GO:0043531	AT3G07040.1	NB-ARC domain-containing disease resistance protein	LOC_Os11g46210.1	NB-ARC domain containing protein, expressed	2,05	-1.0	1	21	240	260	UUGCAGUCCUCGAUGUCGUAG	CUACGACAUCGAGGAUUGCAU	Cleavage
tae-miR1120c-5p	Traes_2BS_5C64FC44A.1	PF07714	KOG1187	2.7.11.1		GO:GO:0006468,GO:GO:0004672	AT4G23140.1	cysteine-rich RLK (RECEPTOR-like protein kinase) 6	LOC_Os07g35580.1	TKL_IRAK_DUF26-lc.20 - DUF26 kinases have homology to DUF26 containing loci, expressed	0,00	-1.0	1	21	21	41	UAAUAUAAGAACGUUUUUGAC	GUCAAAAACGUUCUUAUAUUA	Cleavage
tae-miR1120c-5p	Traes_2BS_5C64FC44A.2	PF07714		2.7.11.1		GO:GO:0006468,GO:GO:0004672	AT4G23180.1	cysteine-rich RLK (RECEPTOR-like protein kinase) 10	LOC_Os07g35580.1	TKL_IRAK_DUF26-lc.20 - DUF26 kinases have homology to DUF26 containing loci, expressed	0,00	-1.0	1	21	2	22	UAAUAUAAGAACGUUUUUGAC	GUCAAAAACGUUCUUAUAUUA	Cleavage
tae-miR1130b-3p	Traes_2BS_5CDCD5152.1	PF04178	KOG1743			GO:GO:0016192	AT5G01430.1	Got1/Sft2-like vescicle transport protein family	LOC_Os03g11100.1	vesicle transport protein GOT1B, putative, expressed	2,00	-1.0	1	20	1	21	UCUUAUAUUAUGGGA-CGGAG	GUCCGAUCCCAUAAUAUAAGA	Cleavage
tae-miR1121	Traes_2BS_6249E432F.1	PF01426				GO:GO:0003682	AT4G22140.1	PHD finger family protein / bromo-adjacent homology (BAH) domain-containing protein	LOC_Os07g08880.1	ES43 protein, putative, expressed	0,00	-1.0	1	22	538	559	AGUAGUGAUCUAAACGCUCUUA	UAAGAGCGUUUAGAUCACUACU	Cleavage
tae-miR1121	Traes_2BS_6249E432F.2	PF01426				GO:GO:0003682	AT4G22140.1	PHD finger family protein / bromo-adjacent homology (BAH) domain-containing protein	LOC_Os07g08880.1	ES43 protein, putative, expressed	0,00	-1.0	1	22	538	559	AGUAGUGAUCUAAACGCUCUUA	UAAGAGCGUUUAGAUCACUACU	Cleavage
tae-miR1121	Traes_2BS_6249E432F.3	PF01426				GO:GO:0003682	AT4G39100.1	PHD finger family protein / bromo-adjacent homology (BAH) domain-containing protein	LOC_Os07g08880.1	ES43 protein, putative, expressed	0,00	-1.0	1	22	448	469	AGUAGUGAUCUAAACGCUCUUA	UAAGAGCGUUUAGAUCACUACU	Cleavage

tae-miR1133	Traes_2BS_67D862F4A.1	PF01428	KOG3183			GO:GO:0008270	AT2G41835.1	zinc finger (C2H2 type, AN1-like) family protein	LOC_Os07g38240.1	ZOS7-05 - C2H2 zinc finger protein, expressed	2,05	-1.0	1	22	30	51	CAUAUACUCCCUCCGUCCGAAA	UUUGGGACGGAGGGAGUAUAAG	Cleavage
tae-miR9662a-3p	Traes_2BS_74269A4C5.1	PF02536				GO:GO:0006355,GO:GO:0005739,GO:GO:0003690	AT5G07900.1	Mitochondrial transcription termination factor family protein	LOC_Os06g12100.1	mTERF family protein, expressed	2,00	-1.0	1	21	189	209	UUGAACAUCCCAGAGCCACCG	CCGUGGUUCUGGGAUGUUCAG	Cleavage
tae-miR9662b-3p	Traes_2BS_74269A4C5.1	PF02536				GO:GO:0006355,GO:GO:0005739,GO:GO:0003690	AT5G07900.1	Mitochondrial transcription termination factor family protein	LOC_Os06g12100.1	mTERF family protein, expressed	2,00	-1.0	1	21	188	208	UGAACAUCCCAGAGCCACCGG	CCCGUGGUUCUGGGAUGUUCA	Cleavage
tae-miR1128	Traes_2BS_7AEB138EB.1	PF01397,PF03936		4.2.3.104		GO:GO:0016829,GO:GO:0010333,GO:GO:0008152,GO:GO:0000287	AT5G23960.2	terpene synthase 21	LOC_Os07g11790.1	terpene synthase, putative, expressed	1,05	-1.0	1	21	84	104	UACUACUCCCUCCGUCCGAAA	UUUCGGACGGAGGGAGUACUA	Cleavage
tae-miR1122b-3p	Traes_2BS_A20CCBEC3.1	PF05368		1.23.1.1,1.23.1.3			AT1G32100.1	pinoresinol reductase 1	LOC_Os12g16220.2	nmrA-like family domain containing protein, expressed	2,05	-1.0	1	21	389	409	AGACUUAUAUGUAGGAACGGA	UCUGUUCCUAAAUAUAAGUCU	Translation
tae-miR1128	Traes_2BS_B3A88F254.2	PF00479,PF02781		1.1.1.49	K00036	GO:GO:0055114,GO:GO:0050661,GO:GO:0006006,GO:GO:0004345	AT5G13110.1	glucose-6-phosphate dehydrogenase 2	LOC_Os07g22350.1	glucose-6-phosphate 1-dehydrogenase, chloroplast precursor, putative, expressed	2,00	-1.0	1	21	88	109	UACUACUCCCUCCGUCC-GAAA	UUUCCGGACGGAGGGAGUAGUA	Cleavage
tae-miR1128	Traes_2BS_B3A88F254.3	PF00479,PF02781		1.1.1.49	K00036	GO:GO:0055114,GO:GO:0050661,GO:GO:0006006,GO:GO:0004345	AT5G13110.1	glucose-6-phosphate dehydrogenase 2	LOC_Os07g22350.1	glucose-6-phosphate 1-dehydrogenase, chloroplast precursor, putative, expressed	2,00	-1.0	1	21	88	109	UACUACUCCCUCCGUCC-GAAA	UUUCCGGACGGAGGGAGUAGUA	Cleavage
tae-miR1127a	Traes_2BS_C00FC92D4.2						AT2G18910.1	hydroxyproline-rich glycoprotein family protein	LOC_Os02g26370.1	wiskott-Aldrich syndrome protein family member 2, putative, expressed	1,05	-1.0	1	19	497	515	UCCUUCCGUUCGGAAUUAC	GUAAUUCCGAACGGAGGGA	Cleavage
tae-miR1117	Traes_2BS_C5AF5419E.1			3.4.23.47,3.1.26.13,3.1.13.2,2.7.7.49,2.7.7.7					LOC_Os02g49670.1	zinc knuckle family protein, expressed	0,00	-1.0	1	24	926	949	UAGUACCGGUUCGUGGCACGAACC	GGUUCGUGCCACGAACCGGUACUA	Cleavage
tae-miR9662a-3p	Traes_2BS_DDF26C784.1	PF02536				GO:GO:0006355,GO:GO:0005739,GO:GO:0003690	AT5G07900.1	Mitochondrial transcription termination factor family protein	LOC_Os06g12100.1	mTERF family protein, expressed	2,00	-1.0	1	21	459	479	UUGAACAUCCCAGAGCCACCG	CCGUGGUUCUGGGAUGUUCAG	Cleavage
tae-miR9662b-3p	Traes_2BS_DDF26C784.1	PF02536				GO:GO:0006355,GO:GO:0005739,GO:GO:0003690	AT5G07900.1	Mitochondrial transcription termination factor family protein	LOC_Os06g12100.1	mTERF family protein, expressed	2,00	-1.0	1	21	458	478	UGAACAUCCCAGAGCCACCGG	CCCGUGGUUCUGGGAUGUUCA	Cleavage
tae-miR5384-3p	Traes_2BS_EBCD10293.1	PF02225,PF00082		3.4.21.25		GO:GO:0006508,GO:GO:0004252	AT1G04110.1	Subtilase family protein	LOC_Os04g10360.1	OsSub40 - Putative Subtilisin homologue, expressed	2,05	-1.0	1	21	116	136	UGAGCGCGCCGCCGUCGAAUG	UGUUCGCCGACGGCGCGCUCA	Cleavage
tae-miR5384-3p	Traes_2BS_EBCD10293.2	PF02225,PF00082		3.4.21.25		GO:GO:0006508,GO:GO:0004252	AT1G04110.1	Subtilase family protein	LOC_Os04g10360.1	OsSub40 - Putative Subtilisin homologue, expressed	2,05	-1.0	1	21	116	136	UGAGCGCGCCGCCGUCGAAUG	UGUUCGCCGACGGCGCGCUCA	Cleavage
tae-miR1130b-3p	Traes_2DL_10B2965EF.8	PF00443		3.4.19.12		GO:GO:0036459,GO:GO:0016579	AT4G39370.2	ubiquitin-specific protease 27	LOC_Os04g34984.3	ubiquitin carboxyl-terminal hydrolase domain containing protein, expressed	0,00	-1.0	1	21	752	772	UCUUAUAUUAUGGGACGGAGG	CCUCCGUCCCAUAAUAUAAGA	Cleavage
tae-miR1122c-3p	Traes_2DL_10B2965EF.8	PF00443		3.4.19.12		GO:GO:0036459,GO:GO:0016579	AT4G39370.2	ubiquitin-specific protease 27	LOC_Os04g34984.3	ubiquitin carboxyl-terminal hydrolase domain containing protein, expressed	1,05	-1.0	1	21	752	772	UCUAAUAUUAUGGGACGGAGG	CCUCCGUCCCAUAAUAUAAGA	Cleavage
tae-miR1120c-5p	Traes_2DL_10B2965EF.8	PF00443		3.4.19.12		GO:GO:0036459,GO:GO:0016579	AT4G39370.2	ubiquitin-specific protease 27	LOC_Os04g34984.3	ubiquitin carboxyl-terminal hydrolase domain containing protein, expressed	1,05	-1.0	1	21	795	815	UAAUAUAAGAACGUUUUUGAC	GUCAAAAACGCUCUUAUAUUA	Translation
tae-miR1130b-3p	Traes_2DL_3C9129432.1	PF00656				GO:GO:0006508,GO:GO:0004197	AT1G79340.1	metacaspase 4	LOC_Os01g58580.1	ICE-like protease p20 domain containing protein, putative, expressed	1,05	-1.0	1	21	164	184	UCUUAUAUUAUGGGACGGAGG	CUUCCGUCCCAUAAUGUAAGA	Cleavage
tae-miR1133	Traes_2DL_4481C0380.4										2,05	-1.0	1	22	1	22	CAUAUACUCCCUCCGUCCGAAA	UUUGGGACGGAGGGAGUACAUG	Cleavage
tae-miR1137a	Traes_2DL_4F8C87C42.5	PF04499	KOG2073				AT1G30470.1	SIT4 phosphatase-associated family protein	LOC_Os04g52940.1	SIT4 phosphatase-associated protein domain containing protein, expressed	1,00	-1.0	1	20	1791	1810	UAGUACAAAGUUGAGUCAUC	GAUGACCCAACUUUGUACUA	Cleavage
tae-miR1127b-3p	Traes_2DL_4F8C87C42.5	PF04499	KOG2073				AT1G30470.1	SIT4 phosphatase-associated family protein	LOC_Os04g52940.1	SIT4 phosphatase-associated protein domain containing protein, expressed	1,05	-1.0	1	21	1822	1842	ACAAGUAUUUCUGGACGGAGG	CCUCCGUCCGGAAAUACUUGU	Cleavage
tae-miR1137b-5p	Traes_2DL_4F8C87C42.5	PF04499	KOG2073				AT1G30470.1	SIT4 phosphatase-associated family protein	LOC_Os04g52940.1	SIT4 phosphatase-associated protein domain containing protein, expressed	1,05	-1.0	1	21	1944	1964	UCCGUUCCAGAAUAGAUGACC	GGUCAUCUAUUUUGGAACGGA	Translation
tae-miR1122a	Traes_2DL_4F8C87C42.6	PF04499					AT1G30470.1	SIT4 phosphatase-associated family protein	LOC_Os04g52940.1	SIT4 phosphatase-associated protein domain containing protein, expressed	2,05	-1.0	1	20	1750	1769	UAGAUACAUCCGUAUCUAGA	ACUACAUACGGAUGUAUAUA	Cleavage
tae-miR1121	Traes_2DL_74E241841.2	PF01426				GO:GO:0003682	AT4G39100.1	PHD finger family protein / bromo-adjacent homology (BAH) domain-containing protein	LOC_Os07g08880.1	ES43 protein, putative, expressed	2,05	-1.0	1	22	448	469	AGUAGUGAUCUAAACGCUCUUA	UAAGAGUGUUUGGAUCACUACU	Translation
tae-miR1130b-3p	Traes_2DL_8F04980F0.5	PF03405		1.14.19.2		GO:GO:0055114,GO:GO:0045300,GO:GO:0006631	AT3G02630.1	Plant stearoyl-acyl-carrier-protein desaturase family protein	LOC_Os04g31070.1	acyl-desaturase, chloroplast precursor, putative, expressed	2,05	-1.0	1	21	468	488	UCUUAUAUUAUGGGACGGAGG	CCUCCGUCCCCUAAUAUAAGG	Translation
tae-miR1128	Traes_2DL_9AD532712.8	PF15613,PF15614					AT5G08630.1	DDT domain-containing protein	LOC_Os04g35864.1	DDT domain-containing protein, putative, expressed	2,00	-1.0	1	21	305	325	UACUACUCCCUCCGUCCGAAA	GUCCGGACGGAGGGAGUAGUU	Cleavage
tae-miR1122a	Traes_2DL_B3CD23EBC.1	PF05496,PF00004				GO:GO:0009378,GO:GO:0006310,GO:GO:0006281,GO:GO:0005524	AT1G03000.1	peroxin 6	LOC_Os04g52690.1	AAA family ATPase, putative, expressed	2,05	-1.0	1	20	9	28	UAGAUACAUCCGUAUCUAGA	ACUACAUACGGAUGUAUAUA	Cleavage
tae-miR9662a-3p	Traes_2DL_BE754F0C1.1	PF02536	KOG1267		K15032	GO:GO:0006355,GO:GO:0005739,GO:GO:0003690	AT5G07900.1	Mitochondrial transcription termination factor family protein	LOC_Os06g12100.1	mTERF family protein, expressed	1,00	-1.0	1	21	687	707	UUGAACAUCCCAGAGCCACCG	CCGUGGCUCUGGGAUGUUCAG	Cleavage
tae-miR9662b-3p	Traes_2DL_BE754F0C1.1	PF02536	KOG1267		K15032	GO:GO:0006355,GO:GO:0005739,GO:GO:0003690	AT5G07900.1	Mitochondrial transcription termination factor family protein	LOC_Os06g12100.1	mTERF family protein, expressed	1,00	-1.0	1	21	686	706	UGAACAUCCCAGAGCCACCGG	CCCGUGGCUCUGGGAUGUUCA	Cleavage
tae-miR1134	Traes_2DL_E8A7ABB5D.1										3,00	-1.0	1	24	509	532	CAACAACAACAAGAAGAAGAAGAU	AUCUUCAUGGUCUUGUUGUUGUUG	Cleavage
tae-miR1134	Traes_2DL_E8A7ABB5D.2										3,00	-1.0	1	24	629	652	CAACAACAACAAGAAGAAGAAGAU	AUCUUCAUGGUCUUGUUGUUGUUG	Cleavage
tae-miR1134	Traes_2DL_E8A7ABB5D.3										3,00	-1.0	1	24	614	637	CAACAACAACAAGAAGAAGAAGAU	AUCUUCAUGGUCUUGUUGUUGUUG	Cleavage
tae-miR1134	Traes_2DL_E8A7ABB5D.4										3,00	-1.0	1	24	512	535	CAACAACAACAAGAAGAAGAAGAU	AUCUUCAUGGUCUUGUUGUUGUUG	Cleavage
tae-miR1134	Traes_2DL_E8A7ABB5D.5										3,00	-1.0	1	24	509	532	CAACAACAACAAGAAGAAGAAGAU	AUCUUCAUGGUCUUGUUGUUGUUG	Cleavage
tae-miR1134	Traes_2DL_E8A7ABB5D.6										3,00	-1.0	1	24	509	532	CAACAACAACAAGAAGAAGAAGAU	AUCUUCAUGGUCUUGUUGUUGUUG	Cleavage
tae-miR1134	Traes_2DL_E8A7ABB5D.7										3,00	-1.0	1	24	91	114	CAACAACAACAAGAAGAAGAAGAU	AUCUUCAUGGUCUUGUUGUUGUUG	Cleavage
tae-miR1134	Traes_2DL_E8A7ABB5D.8										3,00	-1.0	1	24	77	100	CAACAACAACAAGAAGAAGAAGAU	AUCUUCAUGGUCUUGUUGUUGUUG	Cleavage
tae-miR1134	Traes_2DL_E8A7ABB5D.9										3,00	-1.0	1	24	28	51	CAACAACAACAAGAAGAAGAAGAU	AUCUUCAUGGUCUUGUUGUUGUUG	Cleavage
tae-miR160	Traes_2DL_EF12C0B89.2						AT1G77850.1	auxin response factor 17	LOC_Os04g59430.1	auxin response factor, putative, expressed	1,00	-1.0	1	21	225	245	UGCCUGGCUCCCUGUAUGCCA	UGGCAUGCAGGGAGCCAGGCA	Cleavage
tae-miR9664-3p	Traes_2DS_480CFDFE4.1	PF01637	KOG4658		K13457	GO:GO:0005524	AT3G07040.1	NB-ARC domain-containing disease resistance protein	LOC_Os11g46210.1	NB-ARC domain containing protein, expressed	2,05	-1.0	1	21	243	263	UUGCAGUCCUCGAUGUCGUAG	CUACGACAUCGAGGAUUGCAU	Cleavage
tae-miR9664-3p	Traes_2DS_480CFDFE4.2	PF01637				GO:GO:0005524	AT3G07040.1	NB-ARC domain-containing disease resistance protein	LOC_Os11g46210.1	NB-ARC domain containing protein, expressed	2,05	-1.0	1	21	259	279	UUGCAGUCCUCGAUGUCGUAG	CUACGACAUCGAGGAUUGCAU	Cleavage
tae-miR5049-3p	Traes_2DS_851973269.7										1,05	-1.0	1	21	178	198	AAUAUGGAUCGGAGGGAGUAC	GUACUCCCUCCGAUUCAUAUU	Cleavage
tae-miR1122b-3p	Traes_2DS_9B301D4F8.2	PF00069	KOG0580,KOG2345	2.7.11.1		GO:GO:0006468,GO:GO:0005524,GO:GO:0004672	AT5G18500.2	Protein kinase superfamily protein	LOC_Os11g39450.1	cysteine-rich receptor-like protein kinase 7 precursor, putative, expressed	1,05	-1.0	1	21	843	863	AGACUUAUAUGUAGGAACGGA	UCCGUUCCUAAAUAUAAGUCU	Translation
tae-miR1123	Traes_2DS_C866A54F1.2						AT4G36730.1	G-box binding factor 1	LOC_Os07g10890.1	transcription factor, putative, expressed	1,00	-1.0	1	23	792	814	UCCGUGAGACCUGGUCUCAUAGA	UCUAUGAGACCAGGUCUCACGGG	Cleavage
tae-miR1123	Traes_2DS_C866A54F1.4						AT4G36730.1	G-box binding factor 1	LOC_Os07g10890.1	transcription factor, putative, expressed	1,00	-1.0	1	23	786	808	UCCGUGAGACCUGGUCUCAUAGA	UCUAUGAGACCAGGUCUCACGGG	Cleavage
tae-miR9778	Traes_2DS_C9635AFF7.1	PF00931				GO:GO:0043531	AT3G07040.1	NB-ARC domain-containing disease resistance protein	LOC_Os08g14850.1	resistance protein, putative, expressed	2,05	-1.0	1	21	126	146	UGCAUCAUCUCGAACUCGUCG	CGGCGAGUUCCAGAUGAUGCA	Translation
tae-miR9778	Traes_2DS_C9635AFF7.2	PF00931				GO:GO:0043531	AT3G07040.1	NB-ARC domain-containing disease resistance protein	LOC_Os08g14850.1	resistance protein, putative, expressed	2,05	-1.0	1	21	105	125	UGCAUCAUCUCGAACUCGUCG	CGGCGAGUUCCAGAUGAUGCA	Translation
tae-miR1117	Traes_3AL_018D4C27C.1	PF07714,PF12819		2.7.11.1		GO:GO:0006468,GO:GO:0004672	AT3G51550.1	Malectin/receptor-like protein kinase family protein	LOC_Os05g25430.2	receptor-like protein kinase At3g46290 precursor, putative, expressed	1,05	-1.0	1	24	1609	1632	UAGUACCGGUUCGUGGCACGAACC	GGUUCGUGCCACGAACCGGUGCUA	Cleavage
tae-miR1133	Traes_3AL_30294D0A5.1			1.1.1.8		GO:GO:0055114,GO:GO:0009331,GO:GO:0006072,GO:GO:0004367	AT2G41540.3	6-phosphogluconate dehydrogenase family protein	LOC_Os01g71280.1	glycerol-3-phosphate dehydrogenase, putative, expressed	2,05	-1.0	1	22	392	413	CAUAUACUCCCUCCGUCCGAAA	UUUAGGACGGAGGGAGUAUUUG	Cleavage
tae-miR1133	Traes_3AL_386795528.2	PF00249					AT5G02320.2	myb domain protein 3r-5	LOC_Os01g62410.1	MYB family transcription factor, putative, expressed	0,00	-1.0	1	22	39	60	CAUAUACUCCCUCCGUCCGAAA	UUUCGGACGGAGGGAGUAUAUG	Cleavage
tae-miR1122a	Traes_3AL_5E5C8D264.2								LOC_Os05g46470.1	expressed protein	1,00	-1.0	1	20	18	37	UAGAUACAUCCGUAUCUAGA	UCUAGACACGGAUGUAUCUA	Cleavage
tae-miR1137b-5p	Traes_3AL_7A4D34425.2						AT1G29820.2	Magnesium transporter CorA-like family protein	LOC_Os01g47460.1	expressed protein	2,05	-1.0	1	21	1006	1026	UCCGUUCCAGAAUAGAUGACC	UAUCAUUUAUUCAGGAACGGA	Cleavage
tae-miR1122a	Traes_3AL_B9EDE61D1.1	PF06017				GO:GO:0016459,GO:GO:0003774	AT5G53310.1	myosin heavy chain-related	LOC_Os01g43360.1	myosin-Ie, putative, expressed	2,05	-1.0	1	20	4	23	UAGAUACAUCCGUAUCUAGA	ACUACAUACGGAUGUAUAUA	Cleavage
tae-miR1122a	Traes_3AL_B9EDE61D1.2	PF06017				GO:GO:0016459,GO:GO:0003774	AT5G53310.1	myosin heavy chain-related	LOC_Os01g43360.1	myosin-Ie, putative, expressed	2,05	-1.0	1	20	4	23	UAGAUACAUCCGUAUCUAGA	ACUACAUACGGAUGUAUAUA	Cleavage
tae-miR5049-3p	Traes_3AL_CF1A4ACDE.1	PF03759				GO:GO:0005089	AT1G52240.1	RHO guanyl-nucleotide exchange factor 11	LOC_Os01g55520.1	ATROPGEF7/ROPGEF7, putative, expressed	1,00	-1.0	1	21	917	937	AAUAUGGAUCGGAGGGAGUAC	UCCCUCCCUCCGAUCCAUAUU	Cleavage
tae-miR159a	Traes_3AL_F09C7CA19.1	PF00249					AT5G06100.1	myb domain protein 33	LOC_Os01g59660.2	MYB family transcription factor, putative, expressed	2,05	-1.0	1	21	620	640	UUUGGAUUGAAGGGAGCUCUG	UGGAGCUCCCUUCACUCCAAG	Cleavage
tae-miR159b	Traes_3AL_F09C7CA19.1	PF00249					AT5G06100.1	myb domain protein 33	LOC_Os01g59660.2	MYB family transcription factor, putative, expressed	2,05	-1.0	1	21	620	640	UUUGGAUUGAAGGGAGCUCUG	UGGAGCUCCCUUCACUCCAAG	Cleavage
tae-miR1121	Traes_3AL_F2EDB3661.7										2,00	-1.0	1	22	224	245	AGUAGUGAUCUAAACGCUCUUA	CCUCCGCGUUUAGAUCACUACU	Cleavage
tae-miR1133	Traes_3AL_FA6F160DF.7										3,00	-1.0	1	22	142	164	CAUAUACUCCCUCCG-UCCGAAA	UUUCGGAACGGAGGGAGUAUAUU	Cleavage
tae-miR5200	Traes_3AS_2A0765E10.1	PF14531		2.7.11.1			AT1G62400.1	Protein kinase superfamily protein	LOC_Os01g01740.1	protein kinase domain containing protein, expressed	2,05	-1.0	1	21	177	197	UGUAGAUACUCCCUAAGGCUU	UUCCCUUAGGAAGUAUCUACA	Translation
tae-miR5200	Traes_3AS_2A0765E10.2	PF14531		2.7.11.1			AT1G62400.1	Protein kinase superfamily protein	LOC_Os01g01740.1	protein kinase domain containing protein, expressed	2,05	-1.0	1	21	66	86	UGUAGAUACUCCCUAAGGCUU	UUCCCUUAGGAAGUAUCUACA	Translation
tae-miR5200	Traes_3AS_2A0765E10.3	PF14531		2.7.11.1			AT1G62400.1	Protein kinase superfamily protein	LOC_Os01g01740.1	protein kinase domain containing protein, expressed	2,05	-1.0	1	21	12	32	UGUAGAUACUCCCUAAGGCUU	UUCCCUUAGGAAGUAUCUACA	Translation
tae-miR9674b-5p	Traes_3AS_6CB1DBD28.1	PF01535,PF13041					AT3G09040.1	Pentatricopeptide repeat (PPR) superfamily protein	LOC_Os04g43430.3	pentatricopeptide, putative, expressed	2,00	-1.0	1	21	429	449	AUAGCAUCAUCCAUCCUACCC	GGGUAGGCUGGAUGAUGCUAG	Cleavage
tae-miR1121	Traes_3AS_A466B9ECC.1	PF13424					AT3G54360.1	zinc ion binding	LOC_Os01g01420.2	expressed protein	0,00	-1.0	1	22	725	746	AGUAGUGAUCUAAACGCUCUUA	UAAGAGCGUUUAGAUCACUACU	Cleavage
tae-miR6197-5p	Traes_3AS_A466B9ECC.1	PF13424					AT3G54360.1	zinc ion binding	LOC_Os01g01420.2	expressed protein	2,05	-1.0	1	21	763	783	UCUGUAAACAAAUGUAGGACG	GCUCUUACAUUUCUUUACAGA	Cleavage
tae-miR1121	Traes_3AS_A466B9ECC.2	PF13424					AT3G54360.1	zinc ion binding	LOC_Os01g01420.2	expressed protein	0,00	-1.0	1	22	725	746	AGUAGUGAUCUAAACGCUCUUA	UAAGAGCGUUUAGAUCACUACU	Cleavage
tae-miR6197-5p	Traes_3AS_A466B9ECC.2	PF13424					AT3G54360.1	zinc ion binding	LOC_Os01g01420.2	expressed protein	2,05	-1.0	1	21	763	783	UCUGUAAACAAAUGUAGGACG	GCUCUUACAUUUCUUUACAGA	Cleavage
tae-miR1133	Traes_3AS_A6D3DD27E.2						AT4G32920.2	glycine-rich protein	LOC_Os01g09246.1	glycine-rich protein, putative, expressed	2,05	-1.0	1	22	16	37	CAUAUACUCCCUCCGUCCGAAA	UUUGGGACGGAGGGAGUAUGUG	Cleavage
tae-miR1133	Traes_3AS_A6D3DD27E.4						AT4G32920.1	glycine-rich protein	LOC_Os01g09246.1	glycine-rich protein, putative, expressed	2,05	-1.0	1	22	9	30	CAUAUACUCCCUCCGUCCGAAA	UUUGGGACGGAGGGAGUAUGUG	Cleavage
tae-miR1130b-3p	Traes_3AS_AA474B823.1	PF00725,PF16113,PF02737	KOG2304	5.1.2.3,4.2.1.17,1.1.1.35,5.3.3.8		GO:GO:0055114,GO:GO:0016491,GO:GO:0006631,GO:GO:0003857	AT4G29010.1	Enoyl-CoA hydratase/isomerase family	LOC_Os02g17390.1	3-hydroxyacyl-CoA dehydrogenase, putative, expressed	1,05	-1.0	1	21	1652	1672	UCUUAUAUUAUGGGACGGAGG	CCUCCGUCCCAUAAUAUGAGA	Cleavage
tae-miR1122c-3p	Traes_3AS_AA474B823.1	PF00725,PF16113,PF02737	KOG2304	5.1.2.3,4.2.1.17,1.1.1.35,5.3.3.8		GO:GO:0055114,GO:GO:0016491,GO:GO:0006631,GO:GO:0003857	AT4G29010.1	Enoyl-CoA hydratase/isomerase family	LOC_Os02g17390.1	3-hydroxyacyl-CoA dehydrogenase, putative, expressed	1,05	-1.0	1	21	1652	1672	UCUAAUAUUAUGGGACGGAGG	CCUCCGUCCCAUAAUAUGAGA	Cleavage
tae-miR1130b-3p	Traes_3AS_AA474B823.3	PF00725,PF00378,PF02737	KOG2304	5.1.2.3,4.2.1.17,1.1.1.35,5.3.3.8		GO:GO:0055114,GO:GO:0016491,GO:GO:0006631,GO:GO:0003857,GO:GO:0008152,GO:GO:0003824	AT4G29010.1	Enoyl-CoA hydratase/isomerase family	LOC_Os02g17390.1	3-hydroxyacyl-CoA dehydrogenase, putative, expressed	1,05	-1.0	1	21	1397	1417	UCUUAUAUUAUGGGACGGAGG	CCUCCGUCCCAUAAUAUGAGA	Cleavage
tae-miR1122c-3p	Traes_3AS_AA474B823.3	PF00725,PF00378,PF02737	KOG2304	5.1.2.3,4.2.1.17,1.1.1.35,5.3.3.8		GO:GO:0055114,GO:GO:0016491,GO:GO:0006631,GO:GO:0003857,GO:GO:0008152,GO:GO:0003824	AT4G29010.1	Enoyl-CoA hydratase/isomerase family	LOC_Os02g17390.1	3-hydroxyacyl-CoA dehydrogenase, putative, expressed	1,05	-1.0	1	21	1397	1417	UCUAAUAUUAUGGGACGGAGG	CCUCCGUCCCAUAAUAUGAGA	Cleavage
tae-miR159a	Traes_3AS_D1D1A1994.1								LOC_Os01g11430.1	expressed protein	2,05	-1.0	1	21	101	121	UUUGGAUUGAAGGGAGCUCUG	CAGAGCCCCCUUCAAACCAAA	Cleavage
tae-miR159b	Traes_3AS_D1D1A1994.1								LOC_Os01g11430.1	expressed protein	2,05	-1.0	1	21	101	121	UUUGGAUUGAAGGGAGCUCUG	CAGAGCCCCCUUCAAACCAAA	Cleavage
tae-miR1122b-3p	Traes_3AS_D7F2F36BB.2						AT3G49720.2		LOC_Os01g05070.1	expressed protein	1,05	-1.0	1	21	564	584	AGACUUAUAUGUAGGAACGGA	UCCGUUCCUAAAUAUAAGUCU	Translation
tae-miR1122b-3p	Traes_3AS_D7F2F36BB.3						AT3G49720.1		LOC_Os01g05070.1	expressed protein	1,05	-1.0	1	21	540	560	AGACUUAUAUGUAGGAACGGA	UCCGUUCCUAAAUAUAAGUCU	Translation
tae-miR5200	Traes_3B_0E2173F88.1	PF14531	KOG0580	2.7.11.1			AT1G62400.1	Protein kinase superfamily protein	LOC_Os01g01740.1	protein kinase domain containing protein, expressed	2,05	-1.0	1	21	171	191	UGUAGAUACUCCCUAAGGCUU	UUCCCUUAGGAAGUAUCUACA	Translation
tae-miR5200	Traes_3B_0E2173F88.2	PF14531	KOG1164	2.7.11.1			AT1G62400.1	Protein kinase superfamily protein	LOC_Os01g01740.1	protein kinase domain containing protein, expressed	2,05	-1.0	1	21	177	197	UGUAGAUACUCCCUAAGGCUU	UUCCCUUAGGAAGUAUCUACA	Translation
tae-miR5200	Traes_3B_0E2173F88.3	PF14531		2.7.11.1			AT1G62400.1	Protein kinase superfamily protein	LOC_Os01g01740.1	protein kinase domain containing protein, expressed	2,05	-1.0	1	21	171	191	UGUAGAUACUCCCUAAGGCUU	UUCCCUUAGGAAGUAUCUACA	Translation
tae-miR9652-5p	Traes_3B_119F6A405.1	PF16487,PF16488,PF08699,PF02170				GO:GO:0005515	AT1G48410.1	Stabilizer of iron transporter SufD / Polynucleotidyl transferase	LOC_Os03g58600.1	PAZ domain containing protein, putative, expressed	2,05	-1.0	1	22	589	610	CCUGUUUGUCAUUAAGUUUCUU	AAGAAACUGAAUGACAAGCAGG	Cleavage
tae-miR9662a-3p	Traes_3B_224A6B7E5.1	PF02536				GO:GO:0006355,GO:GO:0005739,GO:GO:0003690	AT5G07900.1	Mitochondrial transcription termination factor family protein	LOC_Os06g12100.1	mTERF family protein, expressed	2,00	-1.0	1	21	189	209	UUGAACAUCCCAGAGCCACCG	CCGUGGUUCUGGGAUGUUCAG	Cleavage
tae-miR9662b-3p	Traes_3B_224A6B7E5.1	PF02536				GO:GO:0006355,GO:GO:0005739,GO:GO:0003690	AT5G07900.1	Mitochondrial transcription termination factor family protein	LOC_Os06g12100.1	mTERF family protein, expressed	2,00	-1.0	1	21	188	208	UGAACAUCCCAGAGCCACCGG	CCCGUGGUUCUGGGAUGUUCA	Cleavage
tae-miR159a	Traes_3B_25320ED72.1	PF00249	KOG0048		K09422		AT5G06100.1	myb domain protein 33	LOC_Os01g59660.2	MYB family transcription factor, putative, expressed	2,05	-1.0	1	21	956	976	UUUGGAUUGAAGGGAGCUCUG	UGGAGCUCCCUUCACUCCAAG	Cleavage
tae-miR159b	Traes_3B_25320ED72.1	PF00249	KOG0048		K09422		AT5G06100.1	myb domain protein 33	LOC_Os01g59660.2	MYB family transcription factor, putative, expressed	2,05	-1.0	1	21	956	976	UUUGGAUUGAAGGGAGCUCUG	UGGAGCUCCCUUCACUCCAAG	Cleavage
tae-miR159a	Traes_3B_25320ED72.2	PF00249	KOG0048		K09422		AT5G06100.1	myb domain protein 33	LOC_Os01g59660.1	MYB family transcription factor, putative, expressed	2,05	-1.0	1	21	956	976	UUUGGAUUGAAGGGAGCUCUG	UGGAGCUCCCUUCACUCCAAG	Cleavage
tae-miR159b	Traes_3B_25320ED72.2	PF00249	KOG0048		K09422		AT5G06100.1	myb domain protein 33	LOC_Os01g59660.1	MYB family transcription factor, putative, expressed	2,05	-1.0	1	21	956	976	UUUGGAUUGAAGGGAGCUCUG	UGGAGCUCCCUUCACUCCAAG	Cleavage
tae-miR1130b-3p	Traes_3B_467787390.8	PF00917,PF13423,PF14533,PF12436		3.4.19.12		GO:GO:0005515	AT3G11910.1	ubiquitin-specific protease 13	LOC_Os01g56490.1	ubiquitin carboxyl-terminal hydrolase, putative, expressed	1,05	-1.0	1	21	3080	3100	UCUUAUAUUAUGGGACGGAGG	CCUCCGUCCCAAAAUAUAAGA	Translation
tae-miR9776	Traes_3B_470061369.1	PF00270		3.6.4.13		GO:GO:0005524,GO:GO:0003676	AT5G51280.1	DEAD-box protein abstrakt, putative	LOC_Os02g05660.1	DEAD-box ATP-dependent RNA helicase 35A, putative, expressed	2,05	-1.0	1	21	34	54	UUGGACGAGGAUGUGCAACUG	AAGUGGCACAUCCUCGUCGAA	Cleavage
tae-miR1122a	Traes_3B_472072804.1	PF04874,PF01778	KOG3064		K14831		AT1G23280.1	MAK16 protein-related	LOC_Os07g16950.1	Mak16 protein domain containing protein, expressed	1,05	-1.0	1	20	789	808	UAGAUACAUCCGUAUCUAGA	UCUAGAUACGGAGGUAUCUA	Cleavage
tae-miR9778	Traes_3B_4C839468C.10						AT3G46530.1	NB-ARC domain-containing disease resistance protein	LOC_Os12g37290.1	resistance protein T10rga2-1A, putative, expressed	1,00	-1.0	1	21	126	146	UGCAUCAUCUCGAACUCGUCG	GGACGAGCUCGAGAUGAUGCA	Cleavage
tae-miR9778	Traes_3B_4C839468C.11						AT3G46530.1	NB-ARC domain-containing disease resistance protein	LOC_Os12g37290.1	resistance protein T10rga2-1A, putative, expressed	1,00	-1.0	1	21	126	146	UGCAUCAUCUCGAACUCGUCG	GGACGAGCUCGAGAUGAUGCA	Cleavage
tae-miR9778	Traes_3B_4C839468C.12						AT3G46530.1	NB-ARC domain-containing disease resistance protein	LOC_Os12g37290.1	resistance protein T10rga2-1A, putative, expressed	1,00	-1.0	1	21	126	146	UGCAUCAUCUCGAACUCGUCG	GGACGAGCUCGAGAUGAUGCA	Cleavage
tae-miR9778	Traes_3B_4C839468C.13						AT1G50180.1	NB-ARC domain-containing disease resistance protein	LOC_Os02g57310.1	pib, putative, expressed	1,00	-1.0	1	21	102	122	UGCAUCAUCUCGAACUCGUCG	GGACGAGCUCGAGAUGAUGCA	Cleavage
tae-miR9778	Traes_3B_4C839468C.2						AT3G46530.1	NB-ARC domain-containing disease resistance protein	LOC_Os12g37290.1	resistance protein T10rga2-1A, putative, expressed	1,00	-1.0	1	21	126	146	UGCAUCAUCUCGAACUCGUCG	GGACGAGCUCGAGAUGAUGCA	Cleavage
tae-miR9778	Traes_3B_4C839468C.3						AT3G46530.1	NB-ARC domain-containing disease resistance protein	LOC_Os12g37290.1	resistance protein T10rga2-1A, putative, expressed	1,00	-1.0	1	21	126	146	UGCAUCAUCUCGAACUCGUCG	GGACGAGCUCGAGAUGAUGCA	Cleavage
tae-miR9778	Traes_3B_4C839468C.4						AT3G46530.1	NB-ARC domain-containing disease resistance protein	LOC_Os12g37290.1	resistance protein T10rga2-1A, putative, expressed	1,00	-1.0	1	21	126	146	UGCAUCAUCUCGAACUCGUCG	GGACGAGCUCGAGAUGAUGCA	Cleavage
tae-miR9778	Traes_3B_4C839468C.5						AT3G46530.1	NB-ARC domain-containing disease resistance protein	LOC_Os12g37290.1	resistance protein T10rga2-1A, putative, expressed	1,00	-1.0	1	21	126	146	UGCAUCAUCUCGAACUCGUCG	GGACGAGCUCGAGAUGAUGCA	Cleavage
tae-miR9778	Traes_3B_4C839468C.6						AT3G46530.1	NB-ARC domain-containing disease resistance protein	LOC_Os12g37290.1	resistance protein T10rga2-1A, putative, expressed	1,00	-1.0	1	21	126	146	UGCAUCAUCUCGAACUCGUCG	GGACGAGCUCGAGAUGAUGCA	Cleavage
tae-miR9778	Traes_3B_4C839468C.7						AT3G46530.1	NB-ARC domain-containing disease resistance protein	LOC_Os12g37290.1	resistance protein T10rga2-1A, putative, expressed	1,00	-1.0	1	21	126	146	UGCAUCAUCUCGAACUCGUCG	GGACGAGCUCGAGAUGAUGCA	Cleavage
tae-miR9778	Traes_3B_4C839468C.8						AT3G46530.1	NB-ARC domain-containing disease resistance protein	LOC_Os12g37290.1	resistance protein T10rga2-1A, putative, expressed	1,00	-1.0	1	21	126	146	UGCAUCAUCUCGAACUCGUCG	GGACGAGCUCGAGAUGAUGCA	Cleavage
tae-miR9778	Traes_3B_4C839468C.9						AT3G46530.1	NB-ARC domain-containing disease resistance protein	LOC_Os12g37290.1	resistance protein T10rga2-1A, putative, expressed	1,00	-1.0	1	21	126	146	UGCAUCAUCUCGAACUCGUCG	GGACGAGCUCGAGAUGAUGCA	Cleavage
tae-miR1137a	Traes_3B_59B827C5C.2	PF01912	KOG3185			GO:GO:0043022,GO:GO:0042256	AT3G55620.1	Translation initiation factor IF6	LOC_Os07g44620.2	eukaryotic translation initiation factor 6, putative, expressed	0,00	-1.0	1	20	748	767	UAGUACAAAGUUGAGUCAUC	GAUGACUCAACUUUGUACUA	Cleavage
tae-miR1122b-3p	Traes_3B_5DD9C1BE5.1								LOC_Os01g40830.1	transposon protein, putative, Mutator sub-class, expressed	2,05	-1.0	1	21	71	91	AGACUUAUAUGUAGGAACGGA	UCCGUUCUUAAAUAUAAGUCU	Translation
tae-miR7757-5p	Traes_3B_5EFB9A61F.1								LOC_Os12g17490.1	stripe rust resistance protein Yr10, putative, expressed	1,05	-1.0	1	22	113	134	AUAAAACCUUCAGCUAUCCAUC	GAUGGAUAGCUGAAGGUUUUGU	Cleavage
tae-miR7757-5p	Traes_3B_5EFB9A61F.3								LOC_Os12g17490.1	stripe rust resistance protein Yr10, putative, expressed	1,05	-1.0	1	22	89	110	AUAAAACCUUCAGCUAUCCAUC	GAUGGAUAGCUGAAGGUUUUGU	Cleavage
tae-miR9676-5p	Traes_3B_5F0E1F6A4.1						AT1G52700.1	alpha/beta-Hydrolases superfamily protein	LOC_Os05g51050.1	acyl-protein thioesterase, putative, expressed	2,05	-1.0	1	22	38	59	UGGAUGUCAUCGUGGCCGUACA	UGUACGGACAUGAUGACAUCCA	Cleavage
tae-miR1122b-3p	Traes_3B_6D2DCE280.3	PF00025	KOG0084			GO:GO:0005525	AT4G17530.1	RAB GTPase homolog 1C	LOC_Os01g37800.1	ras-related protein, putative, expressed	2,05	-1.0	1	21	576	596	AGACUUAUAUGUAGGAACGGA	UCUGUUCCUAAAUAUAAGUCU	Translation
tae-miR7757-5p	Traes_3B_7FFFF7BCF.1								LOC_Os02g18510.1	stripe rust resistance protein Yr10, putative, expressed	0,00	-1.0	1	22	89	110	AUAAAACCUUCAGCUAUCCAUC	GAUGGAUAGCUGAAGGUUUUAU	Cleavage
tae-miR531	Traes_3B_879F704A0.1						AT2G20585.3	nuclear fusion defective 6	LOC_Os01g13720.3	expressed protein	2,05	-1.0	1	21	155	175	CGCUCGCCGGAGCAGCGUGCA	UCCGCGCUGCUCCGGCGAGGG	Cleavage
tae-miR531	Traes_3B_879F704A0.2						AT2G20585.1	nuclear fusion defective 6	LOC_Os01g13720.2	expressed protein	2,05	-1.0	1	21	155	175	CGCUCGCCGGAGCAGCGUGCA	UCCGCGCUGCUCCGGCGAGGG	Cleavage
tae-miR531	Traes_3B_879F704A0.3						AT2G20585.1	nuclear fusion defective 6	LOC_Os01g13720.3	expressed protein	2,05	-1.0	1	21	50	70	CGCUCGCCGGAGCAGCGUGCA	UCCGCGCUGCUCCGGCGAGGG	Cleavage
tae-miR531	Traes_3B_879F704A0.4						AT2G20585.3	nuclear fusion defective 6	LOC_Os01g13720.3	expressed protein	2,05	-1.0	1	21	50	70	CGCUCGCCGGAGCAGCGUGCA	UCCGCGCUGCUCCGGCGAGGG	Cleavage
tae-miR5049-3p	Traes_3B_9D776A8C4.1	PF00804				GO:GO:0016020	AT3G24350.2	syntaxin of plants 32	LOC_Os01g08400.1	syntaxin, putative, expressed	0,00	-1.0	1	21	575	595	AAUAUGGAUCGGAGGGAGUAC	GUACUCCCUCCGAUCCAUAUU	Cleavage
tae-miR1121	Traes_3B_9F321609E.8										1,05	-1.0	1	22	6	28	AGUAGUGAUCUAAACGCUCU-UA	UAUAGAGCGUUUAGAUCACUAUU	Cleavage
tae-miR9778	Traes_3B_F07C33521.1						AT3G46710.1	NB-ARC domain-containing disease resistance protein	LOC_Os02g57310.1	pib, putative, expressed	1,00	-1.0	1	21	126	146	UGCAUCAUCUCGAACUCGUCG	GGACGAGCUCGAGAUGAUGCA	Cleavage
tae-miR9778	Traes_3B_F07C33521.10						AT3G46710.1	NB-ARC domain-containing disease resistance protein	LOC_Os02g57310.1	pib, putative, expressed	1,00	-1.0	1	21	126	146	UGCAUCAUCUCGAACUCGUCG	GGACGAGCUCGAGAUGAUGCA	Cleavage
tae-miR9778	Traes_3B_F07C33521.2	PF00931				GO:GO:0043531	AT3G46530.1	NB-ARC domain-containing disease resistance protein	LOC_Os08g42670.2	resistance protein, putative, expressed	1,00	-1.0	1	21	126	146	UGCAUCAUCUCGAACUCGUCG	GGACGAGCUCGAGAUGAUGCA	Cleavage
tae-miR9778	Traes_3B_F07C33521.3	PF00931				GO:GO:0043531	AT3G46530.1	NB-ARC domain-containing disease resistance protein	LOC_Os08g42670.2	resistance protein, putative, expressed	1,00	-1.0	1	21	126	146	UGCAUCAUCUCGAACUCGUCG	GGACGAGCUCGAGAUGAUGCA	Cleavage
tae-miR9778	Traes_3B_F07C33521.4	PF00931				GO:GO:0043531	AT3G46530.1	NB-ARC domain-containing disease resistance protein	LOC_Os08g42670.2	resistance protein, putative, expressed	1,00	-1.0	1	21	126	146	UGCAUCAUCUCGAACUCGUCG	GGACGAGCUCGAGAUGAUGCA	Cleavage
tae-miR9778	Traes_3B_F07C33521.5	PF00931				GO:GO:0043531	AT3G46530.1	NB-ARC domain-containing disease resistance protein	LOC_Os08g42670.1	resistance protein, putative, expressed	1,00	-1.0	1	21	126	146	UGCAUCAUCUCGAACUCGUCG	GGACGAGCUCGAGAUGAUGCA	Cleavage
tae-miR9778	Traes_3B_F07C33521.6	PF00931				GO:GO:0043531	AT3G46530.1	NB-ARC domain-containing disease resistance protein	LOC_Os08g42670.1	resistance protein, putative, expressed	1,00	-1.0	1	21	126	146	UGCAUCAUCUCGAACUCGUCG	GGACGAGCUCGAGAUGAUGCA	Cleavage
tae-miR9778	Traes_3B_F07C33521.7	PF00931				GO:GO:0043531	AT3G46530.1	NB-ARC domain-containing disease resistance protein	LOC_Os08g42670.2	resistance protein, putative, expressed	1,00	-1.0	1	21	126	146	UGCAUCAUCUCGAACUCGUCG	GGACGAGCUCGAGAUGAUGCA	Cleavage
tae-miR9778	Traes_3B_F07C33521.8						AT3G46710.1	NB-ARC domain-containing disease resistance protein	LOC_Os02g57310.1	pib, putative, expressed	1,00	-1.0	1	21	126	146	UGCAUCAUCUCGAACUCGUCG	GGACGAGCUCGAGAUGAUGCA	Cleavage
tae-miR9778	Traes_3B_F07C33521.9						AT3G46710.1	NB-ARC domain-containing disease resistance protein	LOC_Os02g57310.1	pib, putative, expressed	1,00	-1.0	1	21	126	146	UGCAUCAUCUCGAACUCGUCG	GGACGAGCUCGAGAUGAUGCA	Cleavage
tae-miR1128	Traes_3B_F61EDC1AE.3	PF02212,PF00350,PF01031				GO:GO:0005525,GO:GO:0003924	AT4G33650.1	dynamin-related protein 3A	LOC_Os01g69130.1	dynamin family protein, putative, expressed	2,00	-1.0	1	21	1581	1601	UACUACUCCCUCCGUCCGAAA	UUCCGAACGGAGGGAGUAGUA	Cleavage
tae-miR1127a	Traes_3B_F61EDC1AE.3	PF02212,PF00350,PF01031				GO:GO:0005525,GO:GO:0003924	AT4G33650.1	dynamin-related protein 3A	LOC_Os01g69130.1	dynamin family protein, putative, expressed	1,05	-1.0	1	19	1577	1595	UCCUUCCGUUCGGAAUUAC	GUAAUUCCGAACGGAGGGA	Cleavage
tae-miR1130b-3p	Traes_3B_FB51F185F.2						AT1G53560.1	Ribosomal protein L18ae family	LOC_Os01g14070.1	60S ribosomal protein L18a-1, putative, expressed	1,05	-1.0	1	21	43	63	UCUUAUAUUAUGGGACGGAGG	CCUCCGUCCCAUAAUGUAAGA	Cleavage
tae-miR164	Traes_3B_FD8ACC50C.1			6.3.2.19		GO:GO:0007275,GO:GO:0006511,GO:GO:0005634	AT1G66650.1	Protein with RING/U-box and TRAF-like domains	LOC_Os01g03190.1	expressed protein	2,05	-1.0	1	21	1544	1564	UGGAGAAGCAGGGCACGUGCA	CGCACGGGCCCUGCUUCUGCA	Cleavage
tae-miR164	Traes_3B_FD8ACC50C.2			6.3.2.19		GO:GO:0007275,GO:GO:0006511,GO:GO:0005634	AT1G66650.1	Protein with RING/U-box and TRAF-like domains	LOC_Os01g03190.1	expressed protein	2,05	-1.0	1	21	1499	1519	UGGAGAAGCAGGGCACGUGCA	CGCACGGGCCCUGCUUCUGCA	Cleavage
tae-miR164	Traes_3B_FD8ACC50C.3			6.3.2.19		GO:GO:0007275,GO:GO:0006511,GO:GO:0005634	AT1G66650.1	Protein with RING/U-box and TRAF-like domains	LOC_Os01g03190.1	expressed protein	2,05	-1.0	1	21	1433	1453	UGGAGAAGCAGGGCACGUGCA	CGCACGGGCCCUGCUUCUGCA	Cleavage
tae-miR164	Traes_3B_FD8ACC50C.4			6.3.2.19		GO:GO:0007275,GO:GO:0006511,GO:GO:0005634	AT1G66650.1	Protein with RING/U-box and TRAF-like domains	LOC_Os01g03190.1	expressed protein	2,05	-1.0	1	21	764	784	UGGAGAAGCAGGGCACGUGCA	CGCACGGGCCCUGCUUCUGCA	Cleavage
tae-miR164	Traes_3B_FD8ACC50C.5			6.3.2.19		GO:GO:0007275,GO:GO:0006511,GO:GO:0005634	AT1G66650.1	Protein with RING/U-box and TRAF-like domains	LOC_Os01g03190.1	expressed protein	2,05	-1.0	1	21	752	772	UGGAGAAGCAGGGCACGUGCA	CGCACGGGCCCUGCUUCUGCA	Cleavage
tae-miR164	Traes_3B_FD8ACC50C.6			6.3.2.19		GO:GO:0007275,GO:GO:0006511,GO:GO:0005634	AT1G66650.1	Protein with RING/U-box and TRAF-like domains	LOC_Os01g03190.1	expressed protein	2,05	-1.0	1	21	731	751	UGGAGAAGCAGGGCACGUGCA	CGCACGGGCCCUGCUUCUGCA	Cleavage
tae-miR164	Traes_3B_FD8ACC50C.7			6.3.2.19		GO:GO:0007275,GO:GO:0006511,GO:GO:0005634	AT1G66650.1	Protein with RING/U-box and TRAF-like domains	LOC_Os01g03190.1	expressed protein	2,05	-1.0	1	21	710	730	UGGAGAAGCAGGGCACGUGCA	CGCACGGGCCCUGCUUCUGCA	Cleavage
tae-miR164	Traes_3B_FD8ACC50C.8			6.3.2.19		GO:GO:0007275,GO:GO:0006511,GO:GO:0005634	AT1G66650.1	Protein with RING/U-box and TRAF-like domains	LOC_Os01g03190.1	expressed protein	2,05	-1.0	1	21	653	673	UGGAGAAGCAGGGCACGUGCA	CGCACGGGCCCUGCUUCUGCA	Cleavage
tae-miR164	Traes_3B_FD8ACC50C.9			6.3.2.19		GO:GO:0007275,GO:GO:0006511,GO:GO:0005634	AT1G66650.1	Protein with RING/U-box and TRAF-like domains	LOC_Os01g03190.1	expressed protein	2,05	-1.0	1	21	356	376	UGGAGAAGCAGGGCACGUGCA	CGCACGGGCCCUGCUUCUGCA	Cleavage
tae-miR5049-3p	Traes_3DL_2B3694DC3.2	PF01217					AT3G09800.1	SNARE-like superfamily protein	LOC_Os05g38610.1	clathrin adaptor complex small chain domain containing protein, expressed	1,05	-1.0	1	21	228	248	AAUAUGGAUCGGAGGGAGUAC	CUACUCCCUCCGAUCCAAAUU	Cleavage
tae-miR1130b-3p	Traes_3DL_426EF6A68.2										2,05	-1.0	1	21	214	234	UCUUAUAUUAUGGGACGGAGG	CCUCCAUCCCGUAAUAUAAGA	Translation
tae-miR7757-5p	Traes_3DL_5C0BC5611.4								LOC_Os12g17490.1	stripe rust resistance protein Yr10, putative, expressed	1,05	-1.0	1	22	89	110	AUAAAACCUUCAGCUAUCCAUC	GAUGGAUAGCUGAAGGUUUUGU	Cleavage
tae-miR1137a	Traes_3DL_96847DCCF.1	PF00069	KOG1187	2.7.11.1		GO:GO:0006468,GO:GO:0005524,GO:GO:0004672	AT1G34300.1	lectin protein kinase family protein	LOC_Os01g47840.1	S-locus-like receptor protein kinase, putative, expressed	1,00	-1.0	1	20	88	107	UAGUACAAAGUUGAGUCAUC	GAUGACCCAACUUUGUACUA	Cleavage
tae-miR5049-3p	Traes_3DL_D1CF4784D.2	PF13307,PF06733		3.6.4.12		GO:GO:0016818,GO:GO:0008026,GO:GO:0006139,GO:GO:0005524,GO:GO:0003676,GO:GO:0004003,GO:GO:0003677	AT1G79950.1	RAD3-like DNA-binding helicase protein	LOC_Os01g40980.1	helicase, putative, expressed	2,05	-1.0	1	21	1646	1666	AAUAUGGAUCGGAGGGAGUAC	AUAAUCCCUCCGUUCCAUAUU	Cleavage
tae-miR159a	Traes_3DL_D9A29948B.1	PF00249					AT5G06100.1	myb domain protein 33	LOC_Os01g59660.1	MYB family transcription factor, putative, expressed	2,05	-1.0	1	21	620	640	UUUGGAUUGAAGGGAGCUCUG	UGGAGCUCCCUUCACUCCAAG	Cleavage
tae-miR159b	Traes_3DL_D9A29948B.1	PF00249					AT5G06100.1	myb domain protein 33	LOC_Os01g59660.1	MYB family transcription factor, putative, expressed	2,05	-1.0	1	21	620	640	UUUGGAUUGAAGGGAGCUCUG	UGGAGCUCCCUUCACUCCAAG	Cleavage
tae-miR159a	Traes_3DL_D9A29948B.2	PF00249					AT5G06100.1	myb domain protein 33	LOC_Os01g59660.1	MYB family transcription factor, putative, expressed	2,05	-1.0	1	21	620	640	UUUGGAUUGAAGGGAGCUCUG	UGGAGCUCCCUUCACUCCAAG	Cleavage
tae-miR159b	Traes_3DL_D9A29948B.2	PF00249					AT5G06100.1	myb domain protein 33	LOC_Os01g59660.1	MYB family transcription factor, putative, expressed	2,05	-1.0	1	21	620	640	UUUGGAUUGAAGGGAGCUCUG	UGGAGCUCCCUUCACUCCAAG	Cleavage
tae-miR1128	Traes_3DS_1DE20CFF3.2	PF01121	KOG3220	2.7.1.24	K00859	GO:GO:0015937,GO:GO:0005524,GO:GO:0004140	AT2G27490.1	dephospho-CoA kinase family	LOC_Os01g25880.1	dephospho-CoA kinase, putative, expressed	2,05	-1.0	1	21	71	91	UACUACUCCCUCCGUCCGAAA	UUUGGGACGGAGGGAGUACUA	Cleavage
tae-miR1122a	Traes_3DS_1DE20CFF3.2	PF01121	KOG3220	2.7.1.24	K00859	GO:GO:0015937,GO:GO:0005524,GO:GO:0004140	AT2G27490.1	dephospho-CoA kinase family	LOC_Os01g25880.1	dephospho-CoA kinase, putative, expressed	1,05	-1.0	1	20	2	21	UAGAUACAUCCGUAUCUAGA	UCUAGAUACAGAUGUAUCUA	Translation
tae-miR1128	Traes_3DS_1DE20CFF3.3	PF01121	KOG3220	2.7.1.24	K00859	GO:GO:0015937,GO:GO:0005524,GO:GO:0004140	AT2G27490.1	dephospho-CoA kinase family	LOC_Os01g25880.1	dephospho-CoA kinase, putative, expressed	2,05	-1.0	1	21	71	91	UACUACUCCCUCCGUCCGAAA	UUUGGGACGGAGGGAGUACUA	Cleavage
tae-miR1122a	Traes_3DS_1DE20CFF3.3	PF01121	KOG3220	2.7.1.24	K00859	GO:GO:0015937,GO:GO:0005524,GO:GO:0004140	AT2G27490.1	dephospho-CoA kinase family	LOC_Os01g25880.1	dephospho-CoA kinase, putative, expressed	1,05	-1.0	1	20	2	21	UAGAUACAUCCGUAUCUAGA	UCUAGAUACAGAUGUAUCUA	Translation
tae-miR1122b-3p	Traes_3DS_D42FCD586.1	PF00274		4.1.2.13		GO:GO:0006096,GO:GO:0004332	AT2G01140.1	Aldolase superfamily protein	LOC_Os01g02880.1	fructose-bisphospate aldolase isozyme, putative, expressed	1,05	-1.0	1	21	786	806	AGACUUAUAUGUAGGAACGGA	UCCGUUCCUAAAUAUAAGUCU	Translation
tae-miR1122b-3p	Traes_3DS_D42FCD586.2	PF00274		4.1.2.13		GO:GO:0006096,GO:GO:0004332	AT2G01140.1	Aldolase superfamily protein	LOC_Os01g02880.1	fructose-bisphospate aldolase isozyme, putative, expressed	1,05	-1.0	1	21	795	815	AGACUUAUAUGUAGGAACGGA	UCCGUUCCUAAAUAUAAGUCU	Translation
tae-miR7757-5p	Traes_4AL_13A3A40D3.1	PF13401	KOG4658				AT3G50950.2	HOPZ-ACTIVATED RESISTANCE 1	LOC_Os11g11810.1	NBS-LRR disease resistance protein, putative, expressed	1,05	-1.0	1	22	1337	1358	AUAAAACCUUCAGCUAUCCAUC	AGUGGAUAGCUGAAGGUUUCAU	Cleavage
tae-miR1135	Traes_4AL_158B970F4.1						AT1G50020.1		LOC_Os11g34870.1	expressed protein	2,05	-1.0	1	24	464	487	CUGCGACAAGUAAUUCCGAACGGA	UCCGUUCCGAAUUACUCGUCGCAG	Cleavage
tae-miR5175-5p	Traes_4AL_158B970F4.1						AT1G50020.1		LOC_Os11g34870.1	expressed protein	3,00	-1.0	1	21	532	552	UUCCAAAUUACUCGUCGUGGU	UCUGAGACGAGUAAUUUGGAA	Cleavage
tae-miR1117	Traes_4AL_2475C298D.1	PF07993		1.2.1.84		GO:GO:0080019	AT3G44540.1	fatty acid reductase 4	LOC_Os04g28600.1	male sterility protein, putative, expressed	2,05	-1.0	1	24	684	707	UAGUACCGGUUCGUGGCACGAACC	GGUUCAUGCCAUGAACCGGUACUA	Cleavage
tae-miR9778	Traes_4AL_2FC39B934.1	PF13191,PF00931				GO:GO:0043531	AT1G53350.1	Disease resistance protein (CC-NBS-LRR class) family	LOC_Os08g14850.1	resistance protein, putative, expressed	2,05	-1.0	1	21	126	146	UGCAUCAUCUCGAACUCGUCG	UGGCGAGUUCCAGAUGAUGCA	Translation
tae-miR408	Traes_4AL_364F56EDB.1										0,00	-1.0	1	21	8	28	CUGCACUGCCUCUUCCCUGGC	CCCAGGGAAGAGGCAGUGCAG	Cleavage
tae-miR9662a-3p	Traes_4AL_3A009739E.1	PF02536				GO:GO:0006355,GO:GO:0005739,GO:GO:0003690	AT1G21150.1	Mitochondrial transcription termination factor family protein	LOC_Os06g12100.1	mTERF family protein, expressed	2,05	-1.0	1	21	66	86	UUGAACAUCCCAGAGCCACCG	CCGUGGCUCUGGAAUGUUCAG	Cleavage
tae-miR9662b-3p	Traes_4AL_3A009739E.1	PF02536				GO:GO:0006355,GO:GO:0005739,GO:GO:0003690	AT1G21150.1	Mitochondrial transcription termination factor family protein	LOC_Os06g12100.1	mTERF family protein, expressed	2,05	-1.0	1	21	65	85	UGAACAUCCCAGAGCCACCGG	CCCGUGGCUCUGGAAUGUUCA	Cleavage
tae-miR1121	Traes_4AL_400EB3C17.10	PF14259,PF00076	KOG0120			GO:GO:0003676	AT4G36690.4	U2 snRNP auxilliary factor, large subunit, splicing factor	LOC_Os11g41820.2	RNA recognition motif containing protein, expressed	2,05	-1.0	1	22	10	31	AGUAGUGAUCUAAACGCUCUUA	UAAGAGCGUUUAGAUCACUAAA	Cleavage
tae-miR1121	Traes_4AL_400EB3C17.3	PF14259,PF00076	KOG0120			GO:GO:0003676	AT4G36690.1	U2 snRNP auxilliary factor, large subunit, splicing factor	LOC_Os11g41820.1	RNA recognition motif containing protein, expressed	2,05	-1.0	1	22	37	58	AGUAGUGAUCUAAACGCUCUUA	UAAGAGCGUUUAGAUCACUAAA	Cleavage
tae-miR1121	Traes_4AL_400EB3C17.7	PF14259,PF00076	KOG0120			GO:GO:0003676	AT4G36690.4	U2 snRNP auxilliary factor, large subunit, splicing factor	LOC_Os11g41820.1	RNA recognition motif containing protein, expressed	2,05	-1.0	1	22	10	31	AGUAGUGAUCUAAACGCUCUUA	UAAGAGCGUUUAGAUCACUAAA	Cleavage
tae-miR1137a	Traes_4AL_40790E994.6	PF05185		2.1.1.125,2.1.1.126		GO:GO:0008168,GO:GO:0006479	AT4G16570.1	protein arginine methyltransferase 7	LOC_Os06g01640.1	arginine N-methyltransferase 7, putative, expressed	1,00	-1.0	1	20	1324	1343	UAGUACAAAGUUGAGUCAUC	GAUGACCCAACUUUGUACUA	Cleavage
tae-miR1137a	Traes_4AL_40790E994.7			2.1.1.125,2.1.1.126		GO:GO:0008168,GO:GO:0006479	AT4G16570.1	protein arginine methyltransferase 7	LOC_Os06g01640.1	arginine N-methyltransferase 7, putative, expressed	1,00	-1.0	1	20	835	854	UAGUACAAAGUUGAGUCAUC	GAUGACCCAACUUUGUACUA	Cleavage
tae-miR1127b-3p	Traes_4AL_44E99BA01.2	PF07714		2.7.11.1		GO:GO:0006468,GO:GO:0004672	AT4G33950.1	Protein kinase superfamily protein	LOC_Os03g41460.1	CAMK_CAMK_like.20 - CAMK includes calcium/calmodulin depedent protein kinases, expressed	1,05	-1.0	1	21	394	414	ACAAGUAUUUCUGGACGGAGG	CCUCCGUCCGGAAAUACUUGU	Cleavage
tae-miR1121	Traes_4AL_541C86EB9.2	PF00076				GO:GO:0003676,GO:GO:0017069,GO:GO:0000398	AT1G21320.1	nucleotide binding;nucleic acid binding	LOC_Os06g03690.2	RNA recognition motif containing protein, putative, expressed	1,00	-1.0	1	22	558	579	AGUAGUGAUCUAAACGCUCUUA	UGAGAUCGUUUAGAUCACUACU	Cleavage
tae-miR1121	Traes_4AL_541C86EB9.3	PF00076				GO:GO:0003676,GO:GO:0017069,GO:GO:0000398	AT1G21320.1	nucleotide binding;nucleic acid binding	LOC_Os06g03690.2	RNA recognition motif containing protein, putative, expressed	1,00	-1.0	1	22	558	579	AGUAGUGAUCUAAACGCUCUUA	UGAGAUCGUUUAGAUCACUACU	Cleavage
tae-miR1122b-3p	Traes_4AL_6252B0BA0.1	PF05562					AT1G29395.1	COLD REGULATED 314 INNER MEMBRANE 1	LOC_Os05g49170.1	cold acclimation protein WCOR413, putative, expressed	1,05	-1.0	1	21	46	66	AGACUUAUAUGUAGGAACGGA	UCCGUUCCUAAAUAUAAGUCU	Translation

tae-miR1137b-5p	Traes_4AL_748BB4675.1	PF00657		3.1.1.1		GO:GO:0016788	AT5G45910.1	GDSL-like Lipase/Acylhydrolase superfamily protein	LOC_Os01g12320.1	GDSL-like lipase/acylhydrolase, putative, expressed	2,05	-1.0	1	21	844	864	UCCGUUCCAGAAUAGAUGACC	ACUCAUCUAUUCUGGGACGGU	Cleavage
tae-miR9778	Traes_4AL_79B47AF61.1	PF13191,PF00931				GO:GO:0043531	AT3G07040.1	NB-ARC domain-containing disease resistance protein	LOC_Os08g42670.1	resistance protein, putative, expressed	2,05	-1.0	1	21	126	146	UGCAUCAUCUCGAACUCGUCG	GGACGAGCUCGACAUGAUGCA	Cleavage
tae-miR9778	Traes_4AL_79B47AF61.3	PF00931				GO:GO:0043531	AT3G46530.1	NB-ARC domain-containing disease resistance protein	LOC_Os08g42670.2	resistance protein, putative, expressed	2,05	-1.0	1	21	126	146	UGCAUCAUCUCGAACUCGUCG	GGACGAGCUCGACAUGAUGCA	Cleavage
tae-miR9778	Traes_4AL_79B47AF61.4	PF00931				GO:GO:0043531	AT3G46530.1	NB-ARC domain-containing disease resistance protein	LOC_Os08g42670.2	resistance protein, putative, expressed	2,05	-1.0	1	21	126	146	UGCAUCAUCUCGAACUCGUCG	GGACGAGCUCGACAUGAUGCA	Cleavage
tae-miR5048-5p	Traes_4AL_85A7C60F9.1	PF00069		2.7.11.1		GO:GO:0006468,GO:GO:0005524,GO:GO:0004672	AT4G21230.1	cysteine-rich RLK (RECEPTOR-like protein kinase) 27	LOC_Os11g10640.1	protein kinase domain containing protein, expressed	2,05	-1.0	1	22	170	191	UUUGCAGGUUUUAGGUCUAAGU	ACUUGGACCUAAAACCUGCGAA	Cleavage
tae-miR5048-5p	Traes_4AL_85A7C60F9.7	PF00069		2.7.11.1		GO:GO:0006468,GO:GO:0005524,GO:GO:0004672	AT4G21230.1	cysteine-rich RLK (RECEPTOR-like protein kinase) 27	LOC_Os11g10640.1	protein kinase domain containing protein, expressed	2,05	-1.0	1	22	170	191	UUUGCAGGUUUUAGGUCUAAGU	ACUUGGACCUAAAACCUGCGAA	Cleavage
tae-miR5048-5p	Traes_4AL_85A7C60F9.8	PF00069		2.7.11.1		GO:GO:0006468,GO:GO:0005524,GO:GO:0004672	AT4G21230.1	cysteine-rich RLK (RECEPTOR-like protein kinase) 27	LOC_Os11g10640.1	protein kinase domain containing protein, expressed	2,05	-1.0	1	22	170	191	UUUGCAGGUUUUAGGUCUAAGU	ACUUGGACCUAAAACCUGCGAA	Cleavage
tae-miR1122b-3p	Traes_4AL_954AE4933.1	PF12171					AT2G02080.1	indeterminate(ID)-domain 4	LOC_Os04g47860.4	ZOS4-11 - C2H2 zinc finger protein, expressed	1,05	-1.0	1	21	583	603	AGACUUAUAUGUAGGAACGGA	UCCGUUCCUAAAUAUAAGUCU	Translation
tae-miR1122a	Traes_4AL_9726307FF.1	PF01926,PF06421,PF00679		3.6.5.3		GO:GO:0005525	AT5G39900.1	Small GTP-binding protein	LOC_Os06g05250.2	GTP-binding protein GUF1, putative, expressed	0,00	-1.0	1	20	1903	1922	UAGAUACAUCCGUAUCUAGA	UCUAGAUACGGAUGUAUCUA	Cleavage
tae-miR1128	Traes_4AL_99942CBEA.1	PF02365				GO:GO:0006355,GO:GO:0003677	AT2G46770.1	NAC (No Apical Meristem) domain transcriptional regulator superfamily protein	LOC_Os06g04090.1	no apical meristem protein, putative, expressed	3,00	-1.0	1	21	693	714	UACUACUCCCUCCG-UCCGAAA	UUUAGGAACGGAGGGAGUAGUA	Cleavage
tae-miR1122b-3p	Traes_4AL_99942CBEA.1	PF02365				GO:GO:0006355,GO:GO:0003677	AT2G46770.1	NAC (No Apical Meristem) domain transcriptional regulator superfamily protein	LOC_Os06g04090.1	no apical meristem protein, putative, expressed	2,05	-1.0	1	21	557	577	AGACUUAUAUGUAGGAACGGA	UCCGUUCUUAAAUAUAAGUCU	Translation
tae-miR9662a-3p	Traes_4AL_A4B73CC6A1.1	PF02536	KOG1267		K15032	GO:GO:0006355,GO:GO:0005739,GO:GO:0003690	AT5G07900.1	Mitochondrial transcription termination factor family protein	LOC_Os06g12100.1	mTERF family protein, expressed	2,05	-1.0	1	21	693	713	UUGAACAUCCCAGAGCCACCG	CCGUGGCUCUGGAAUGUUCAG	Cleavage
tae-miR9662b-3p	Traes_4AL_A4B73CC6A1.1	PF02536	KOG1267		K15032	GO:GO:0006355,GO:GO:0005739,GO:GO:0003690	AT5G07900.1	Mitochondrial transcription termination factor family protein	LOC_Os06g12100.1	mTERF family protein, expressed	2,05	-1.0	1	21	692	712	UGAACAUCCCAGAGCCACCGG	CCCGUGGCUCUGGAAUGUUCA	Cleavage
tae-miR9662a-3p	Traes_4AL_A4B73CC6A1.2	PF02536	KOG1267		K15032	GO:GO:0006355,GO:GO:0005739,GO:GO:0003690	AT5G07900.1	Mitochondrial transcription termination factor family protein	LOC_Os06g12100.1	mTERF family protein, expressed	2,05	-1.0	1	21	693	713	UUGAACAUCCCAGAGCCACCG	CCGUGGCUCUGGAAUGUUCAG	Cleavage
tae-miR9662b-3p	Traes_4AL_A4B73CC6A1.2	PF02536	KOG1267		K15032	GO:GO:0006355,GO:GO:0005739,GO:GO:0003690	AT5G07900.1	Mitochondrial transcription termination factor family protein	LOC_Os06g12100.1	mTERF family protein, expressed	2,05	-1.0	1	21	692	712	UGAACAUCCCAGAGCCACCGG	CCCGUGGCUCUGGAAUGUUCA	Cleavage
tae-miR1130b-3p	Traes_4AL_C55D89913.1	PF03219				GO:GO:0016021,GO:GO:0006810,GO:GO:0005524,GO:GO:0005471	AT5G05310.2	TLC ATP/ADP transporter	LOC_Os11g02159.1	expressed protein	0,00	-1.0	1	21	10	30	UCUUAUAUUAUGGGACGGAGG	CCUCCGUCCCAUAAUAUAAGA	Cleavage
tae-miR1122c-3p	Traes_4AL_C55D89913.1	PF03219				GO:GO:0016021,GO:GO:0006810,GO:GO:0005524,GO:GO:0005471	AT5G05310.2	TLC ATP/ADP transporter	LOC_Os11g02159.1	expressed protein	1,05	-1.0	1	21	10	30	UCUAAUAUUAUGGGACGGAGG	CCUCCGUCCCAUAAUAUAAGA	Cleavage
tae-miR1130b-3p	Traes_4AL_C55D89913.5	PF03219				GO:GO:0016021,GO:GO:0006810,GO:GO:0005524,GO:GO:0005471	AT5G05310.2	TLC ATP/ADP transporter	LOC_Os11g02159.2	expressed protein	0,00	-1.0	1	21	10	30	UCUUAUAUUAUGGGACGGAGG	CCUCCGUCCCAUAAUAUAAGA	Cleavage
tae-miR1122c-3p	Traes_4AL_C55D89913.5	PF03219				GO:GO:0016021,GO:GO:0006810,GO:GO:0005524,GO:GO:0005471	AT5G05310.2	TLC ATP/ADP transporter	LOC_Os11g02159.2	expressed protein	1,05	-1.0	1	21	10	30	UCUAAUAUUAUGGGACGGAGG	CCUCCGUCCCAUAAUAUAAGA	Cleavage
tae-miR1130b-3p	Traes_4AL_C55D89913.6	PF03219				GO:GO:0016021,GO:GO:0006810,GO:GO:0005524,GO:GO:0005471	AT5G05310.2	TLC ATP/ADP transporter	LOC_Os11g02159.1	expressed protein	0,00	-1.0	1	21	10	30	UCUUAUAUUAUGGGACGGAGG	CCUCCGUCCCAUAAUAUAAGA	Cleavage
tae-miR1122c-3p	Traes_4AL_C55D89913.6	PF03219				GO:GO:0016021,GO:GO:0006810,GO:GO:0005524,GO:GO:0005471	AT5G05310.2	TLC ATP/ADP transporter	LOC_Os11g02159.1	expressed protein	1,05	-1.0	1	21	10	30	UCUAAUAUUAUGGGACGGAGG	CCUCCGUCCCAUAAUAUAAGA	Cleavage
tae-miR1130b-3p	Traes_4AL_C55D89913.7	PF03219				GO:GO:0016021,GO:GO:0006810,GO:GO:0005524,GO:GO:0005471	AT5G05310.2	TLC ATP/ADP transporter	LOC_Os11g02159.2	expressed protein	0,00	-1.0	1	21	10	30	UCUUAUAUUAUGGGACGGAGG	CCUCCGUCCCAUAAUAUAAGA	Cleavage
tae-miR1122c-3p	Traes_4AL_C55D89913.7	PF03219				GO:GO:0016021,GO:GO:0006810,GO:GO:0005524,GO:GO:0005471	AT5G05310.2	TLC ATP/ADP transporter	LOC_Os11g02159.2	expressed protein	1,05	-1.0	1	21	10	30	UCUAAUAUUAUGGGACGGAGG	CCUCCGUCCCAUAAUAUAAGA	Cleavage
tae-miR1130b-3p	Traes_4AL_C55D89913.8					GO:GO:0016021,GO:GO:0006810,GO:GO:0005524,GO:GO:0005471	AT5G05310.2	TLC ATP/ADP transporter	LOC_Os11g02159.2	expressed protein	0,00	-1.0	1	21	10	30	UCUUAUAUUAUGGGACGGAGG	CCUCCGUCCCAUAAUAUAAGA	Cleavage
tae-miR1122c-3p	Traes_4AL_C55D89913.8					GO:GO:0016021,GO:GO:0006810,GO:GO:0005524,GO:GO:0005471	AT5G05310.2	TLC ATP/ADP transporter	LOC_Os11g02159.2	expressed protein	1,05	-1.0	1	21	10	30	UCUAAUAUUAUGGGACGGAGG	CCUCCGUCCCAUAAUAUAAGA	Cleavage
tae-miR5049-3p	Traes_4AL_C9E08C423.5										0,00	-1.0	1	21	69	89	AAUAUGGAUCGGAGGGAGUAC	CUACUCCCUCCGAUCCAUAUU	Cleavage
tae-miR9782	Traes_4AL_D576D98B4.1	PF00657		3.1.1.3		GO:GO:0016788	AT5G03810.1	GDSL-like Lipase/Acylhydrolase family protein	LOC_Os03g47940.1	GDSL-like lipase/acylhydrolase, putative, expressed	1,00	-1.0	1	24	2	25	GUAUUAGGUUGGUCAAAUUGACGA	UCGUCAAUUUGACCAACCUAAUAU	Cleavage
tae-miR9782	Traes_4AL_D576D98B4.2			3.1.1.1			AT5G03810.1	GDSL-like Lipase/Acylhydrolase family protein	LOC_Os03g47940.1	GDSL-like lipase/acylhydrolase, putative, expressed	1,00	-1.0	1	24	2	25	GUAUUAGGUUGGUCAAAUUGACGA	UCGUCAAUUUGACCAACCUAAUAU	Cleavage
tae-miR5384-3p	Traes_4AL_E60E5B7BA.1	PF02861	KOG1051		K03695	GO:GO:0019538	AT3G52490.1	Double Clp-N motif-containing P-loop nucleoside triphosphate hydrolases superfamily protein	LOC_Os11g05820.1	transposon protein, putative, Mutator sub-class, expressed	2,05	-1.0	1	21	269	289	UGAGCGCGCCGCCGUCGAAUG	UGUCCCACGGCGGCGCGCACA	Cleavage
tae-miR5384-3p	Traes_4AL_E60E5B7BA.2		KOG1051				AT3G52490.1	Double Clp-N motif-containing P-loop nucleoside triphosphate hydrolases superfamily protein	LOC_Os11g05820.1	transposon protein, putative, Mutator sub-class, expressed	2,05	-1.0	1	21	140	160	UGAGCGCGCCGCCGUCGAAUG	UGUCCCACGGCGGCGCGCACA	Cleavage
tae-miR1130b-3p	Traes_4AS_0E2735576.2			3.4.21.108					LOC_Os05g05330.1	expressed protein	1,05	-1.0	1	21	977	997	UCUUAUAUUAUGGGACGGAGG	CCUCCGUCUCAUAAUAUAAGA	Cleavage
tae-miR1120c-5p	Traes_4AS_0E2735576.2	PF11955					AT5G21970.1	Ubiquitin carboxyl-terminal hydrolase family protein	LOC_Os11g34160.1	ubiquitin carboxyl-terminal hydrolase, family 1, putative, expressed	0,00	-1.0	1	21	1020	1040	UAAUAUAAGAACGUUUUUGAC	GACAAAAACGUUCUUAUAUUA	Cleavage
tae-miR9674a-5p	Traes_4AS_AB9088E9B.2	PF11955					AT5G21970.1	Ubiquitin carboxyl-terminal hydrolase family protein	LOC_Os11g34160.1	ubiquitin carboxyl-terminal hydrolase, family 1, putative, expressed	2,05	-1.0	1	21	1383	1403	GCAUCAUCCAUCCUACCAUUC	UGAUGGUCAGAUGGAUGAUGC	Cleavage
tae-miR9674a-5p	Traes_4AS_AB9088E9B.3	PF03703					AT1G14345.1	NAD(P)-linked oxidoreductase superfamily protein	LOC_Os03g07440.1	putativ eribonuclease P, putative, expressed	2,05	-1.0	1	21	1383	1403	GCAUCAUCCAUCCUACCAUUC	UGAUGGUCAGAUGGAUGAUGC	Cleavage
tae-miR5384-3p	Traes_4AS_C7CBD4A47.1	PF01485	KOG1815	6.3.2.19		GO:GO:0016567,GO:GO:0004842	AT2G16090.1	RING/U-box superfamily protein	LOC_Os03g13100.1	ariadne-1, putative, expressed	2,05	-1.0	1	21	194	214	UGAGCGCGCCGCCGUCGAAUG	UCUUCGACGGCGGCGCGCACU	Cleavage
tae-miR1135	Traes_4AS_D533B306B.3										2,00	-1.0	1	24	1698	1721	CUGCGACAAGUAAUUCCGAACGGA	CGAACCAGGAAUUACUUGUCGCAG	Cleavage
tae-miR1131	Traes_4BL_178D6FFF1.1										1,05	-1.0	1	22	167	188	UAGUACCGGUUCGUGGCUAACC	GGUUAGCCACGAACCGAUACUA	Cleavage
tae-miR1131	Traes_4BL_178D6FFF1.2										1,05	-1.0	1	22	167	188	UAGUACCGGUUCGUGGCUAACC	GGUUAGCCACGAACCGAUACUA	Cleavage
tae-miR9662a-3p	Traes_4BL_29D8171E0.1								LOC_Os06g12100.1	mTERF family protein, expressed	2,00	-1.0	1	21	132	152	UUGAACAUCCCAGAGCCACCG	UCGUGGCGCUGGGAUGUUCAG	Cleavage
tae-miR9662b-3p	Traes_4BL_29D8171E0.1								LOC_Os06g12100.1	mTERF family protein, expressed	2,05	-1.0	1	21	131	151	UGAACAUCCCAGAGCCACCGG	CUCGUGGCGCUGGGAUGUUCA	Cleavage
tae-miR1122b-3p	Traes_4BL_2D34597CF.2	PF04068,PF04034					AT5G10070.2	RNase L inhibitor protein-related	LOC_Os03g09920.1	RNase L inhibitor protein-related, putative, expressed	2,05	-1.0	1	21	454	474	AGACUUAUAUGUAGGAACGGA	UCUGUUCCUAAAUAUAAGUCU	Translation
tae-miR1122b-3p	Traes_4BL_2D34597CF.3	PF04068,PF04034					AT5G10070.2	RNase L inhibitor protein-related	LOC_Os03g09920.1	RNase L inhibitor protein-related, putative, expressed	2,05	-1.0	1	21	454	474	AGACUUAUAUGUAGGAACGGA	UCUGUUCCUAAAUAUAAGUCU	Translation
tae-miR1128	Traes_4BL_37FDD905B.1	PF10250					AT1G04910.1	O-fucosyltransferase family protein	LOC_Os11g29120.2	growth regulator related protein, putative, expressed	2,05	-1.0	1	21	125	145	UACUACUCCCUCCGUCCGAAA	UUUGGGACGGAGGGAGUAGGA	Cleavage
tae-miR5384-3p	Traes_4BL_3B7A98FD6.1	PF03703					AT1G14345.1	NAD(P)-linked oxidoreductase superfamily protein	LOC_Os03g07440.1	putativ eribonuclease P, putative, expressed	2,05	-1.0	1	21	191	211	UGAGCGCGCCGCCGUCGAAUG	UCUUCGACGGCGGCGCGCACU	Cleavage
tae-miR9666a-3p	Traes_4BL_44010E331.1										3,00	-1.0	1	22	176	197	CGGUAGGGCUGUAUGAUGGCGA	UCGGCAUGAUACAGCCCUACCU	Cleavage
tae-miR1122b-3p	Traes_4BL_62355968B.1	PF00013				GO:GO:0003723	AT5G15270.2	RNA-binding KH domain-containing protein	LOC_Os03g25970.1	KH domain-containing protein, putative, expressed	2,05	-1.0	1	21	706	726	AGACUUAUAUGUAGGAACGGA	UCUGUUCCUAAAUAUAAGUCU	Translation
tae-miR1122a	Traes_4BL_62355968B.1	PF00013				GO:GO:0003723	AT5G15270.2	RNA-binding KH domain-containing protein	LOC_Os03g25970.1	KH domain-containing protein, putative, expressed	2,05	-1.0	1	20	747	766	UAGAUACAUCCGUAUCUAGA	ACUACAUACGGAUGUAUAUA	Cleavage
tae-miR1137a	Traes_4BL_D08D5FFB5.2	PF08282	KOG3189	5.4.2.8		GO:GO:0009298,GO:GO:0005737,GO:GO:0004615	AT2G45790.1	phosphomannomutase	LOC_Os04g58580.1	phosphomannomutase, putative, expressed	2,00	-1.0	1	20	722	741	UAGUACAAAGUUGAGUCAUC	AGUGUCUCAACUUUGUACUA	Cleavage
tae-miR1137a	Traes_4BL_F3D5D7034.2						AT3G16990.1	Haem oxygenase-like, multi-helical	LOC_Os03g19390.1	TENA/THI-4 family protein, putative, expressed	1,00	-1.0	1	19	1	19	UAGUACAAAGUUGAGUCAU	AUGACCCAACUUUGUACUA	Cleavage
tae-miR1137b-5p	Traes_4BL_F3D5D7034.2						AT3G16990.1	Haem oxygenase-like, multi-helical	LOC_Os03g19390.1	TENA/THI-4 family protein, putative, expressed	1,05	-1.0	1	21	37	57	UCCGUUCCAGAAUAGAUGACC	GGUCAUCUAUUUUGGAACGGA	Translation
tae-miR9662a-3p	Traes_4BS_257C5B099.1	PF02536				GO:GO:0006355,GO:GO:0005739,GO:GO:0003690	AT5G07900.1	Mitochondrial transcription termination factor family protein	LOC_Os06g12100.1	mTERF family protein, expressed	2,00	-1.0	1	21	66	86	UUGAACAUCCCAGAGCCACCG	CCGUGGUUCUGGGAUGUUCAG	Cleavage
tae-miR9662b-3p	Traes_4BS_257C5B099.1	PF02536				GO:GO:0006355,GO:GO:0005739,GO:GO:0003690	AT5G07900.1	Mitochondrial transcription termination factor family protein	LOC_Os06g12100.1	mTERF family protein, expressed	2,00	-1.0	1	21	65	85	UGAACAUCCCAGAGCCACCGG	CCCGUGGUUCUGGGAUGUUCA	Cleavage
tae-miR156	Traes_4BS_375EF1D15.1								LOC_Os01g69830.1	OsSPL2 - SBP-box gene family member, expressed	2,05	-1.0	1	21	235	255	UGACAGAAGAGAGUGAGCACA	UGUGCUCUCUCUUUUCUGUCA	Cleavage
tae-miR1118	Traes_4BS_4BC02509F.1						AT3G43790.3	zinc induced facilitator-like 2	LOC_Os12g03830.1	major facilitator superfamily antiporter, putative, expressed	2,05	-1.0	1	23	525	547	CACUACAUUAUGGAAUGGAGGGA	UCCCUCCGUUCCAUAAUGUAAUG	Cleavage
tae-miR9666a-3p	Traes_4BS_5419B1DE7.1										3,00	-1.0	1	22	176	197	CGGUAGGGCUGUAUGAUGGCGA	UCGGCAUGAUACAGCCCUACCU	Cleavage
tae-miR1122b-3p	Traes_4BS_6252B0BA0.1	PF05562					AT1G29395.1	COLD REGULATED 314 INNER MEMBRANE 1	LOC_Os05g49170.1	cold acclimation protein WCOR413, putative, expressed	1,05	-1.0	1	21	46	66	AGACUUAUAUGUAGGAACGGA	UCCGUUCCUAAAUAUAAGUCU	Translation
tae-miR7757-5p	Traes_4BS_B45729F4D.4	PF00931				GO:GO:0043531	AT3G07040.1	NB-ARC domain-containing disease resistance protein	LOC_Os11g11810.1	NBS-LRR disease resistance protein, putative, expressed	2,05	-1.0	1	22	653	674	AUAAAACCUUCAGCUAUCCAUC	AAUGGAUAUCUGAAGGUUUCAU	Cleavage
tae-miR5384-3p	Traes_4BS_F1D7488B8.1	PF02861	KOG1051		K03695	GO:GO:0019538	AT3G52490.1	Double Clp-N motif-containing P-loop nucleoside triphosphate hydrolases superfamily protein	LOC_Os11g05820.1	transposon protein, putative, Mutator sub-class, expressed	2,05	-1.0	1	21	269	289	UGAGCGCGCCGCCGUCGAAUG	UGUCCCACGGCGGCGCGCACA	Cleavage
tae-miR1122a	Traes_4BS_F395A220A.2	PF01595	KOG2118				AT2G14520.1	CBS domain-containing protein with a domain of unknown function (DUF21)	LOC_Os03g47120.1	CBS domain containing protein, expressed	0,00	-1.0	1	20	1336	1355	UAGAUACAUCCGUAUCUAGA	UCUAGAUACGGAUGUAUCUA	Cleavage
tae-miR7757-5p	Traes_4DL_113F6DD2B.1	PF00931				GO:GO:0043531	AT1G50180.1	NB-ARC domain-containing disease resistance protein	LOC_Os12g13550.1	NBS-LRR disease resistance protein, putative, expressed	2,05	-1.0	1	22	215	236	AUAAAACCUUCAGCUAUCCAUC	AAUGGAUGGCUGAAGGCUUUAU	Cleavage
tae-miR7757-5p	Traes_4DL_113F6DD2B.2	PF00931				GO:GO:0043531	AT1G50180.1	NB-ARC domain-containing disease resistance protein	LOC_Os10g22484.2	RGH2B, putative, expressed	2,05	-1.0	1	22	215	236	AUAAAACCUUCAGCUAUCCAUC	AAUGGAUGGCUGAAGGCUUUAU	Cleavage
tae-miR5085	Traes_4DL_341830EFF.1										2,05	-1.0	1	21	583	603	AAGGACAUUUUUUGUGGCCUG	GAGGACACAAAAAAUGUCGUU	Cleavage
tae-miR1133	Traes_4DL_529524815.5						AT1G67250.1	Proteasome maturation factor UMP1	LOC_Os02g55640.1	proteasome maturation factor UMP1 family protein, expressed	2,00	-1.0	1	22	103	127	CAUAUACUCCCUCCG-UCCGA--AA	UUAGUCGGAUCGGAGGGAGUAUAUG	Cleavage
tae-miR1130b-3p	Traes_4DL_84745919A.2			2.7.7.19			AT3G51620.2	PAP/OAS1 substrate-binding domain superfamily	LOC_Os03g21850.1	nucleotidyltransferase, putative, expressed	2,05	-1.0	1	21	2964	2984	UCUUAUAUUAUGGGACGGAGG	CCUCUGUCCCAUAAUAUAGGA	Cleavage
tae-miR1130a	Traes_4DL_84745919A.2			2.7.7.19			AT3G51620.2	PAP/OAS1 substrate-binding domain superfamily	LOC_Os03g21850.1	nucleotidyltransferase, putative, expressed	1,05	-1.0	1	23	3016	3038	CCUCCGUCUCGUAAUGUAAGACG	UGUCUUACAUUAUGAGACGGAGG	Translation
tae-miR1128	Traes_4DL_93D4DED90.3	PF00009		3.6.5.3	K03231		AT5G60390.3	GTP binding Elongation factor Tu family protein	LOC_Os03g08010.1	elongation factor Tu, putative, expressed	3,00	-1.0	1	21	565	586	UACUACUCCCUCCG-UCCGAAA	UUUUGGAACGGAGGGAGUAGUA	Cleavage
tae-miR1137a	Traes_4DL_93D4DED90.3	PF00009		3.6.5.3	K03231		AT5G60390.3	GTP binding Elongation factor Tu family protein	LOC_Os03g08010.1	elongation factor Tu, putative, expressed	2,05	-1.0	1	20	508	527	UAGUACAAAGUUGAGUCAUC	GAUGACCCAACUUUGUAUUA	Cleavage
tae-miR1137b-5p	Traes_4DL_93D4DED90.3	PF00009		3.6.5.3	K03231		AT5G60390.3	GTP binding Elongation factor Tu family protein	LOC_Os03g08010.1	elongation factor Tu, putative, expressed	1,05	-1.0	1	21	556	576	UCCGUUCCAGAAUAGAUGACC	GGUCAUCUAUUUUGGAACGGA	Translation
tae-miR1137b-5p	Traes_4DS_28E6C4F21.2										1,05	-1.0	1	21	61	81	UCCGUUCCAGAAUAGAUGACC	AGUCAUCUAUUUUGGAACGGA	Translation
tae-miR1137b-5p	Traes_4DS_28E6C4F21.3										1,05	-1.0	1	21	61	81	UCCGUUCCAGAAUAGAUGACC	AGUCAUCUAUUUUGGAACGGA	Translation
tae-miR1137b-5p	Traes_4DS_28E6C4F21.4										1,05	-1.0	1	21	61	81	UCCGUUCCAGAAUAGAUGACC	AGUCAUCUAUUUUGGAACGGA	Translation
tae-miR7757-5p	Traes_4DS_5B29EE3DD.5	PF00931				GO:GO:0043531	AT3G50950.1	HOPZ-ACTIVATED RESISTANCE 1	LOC_Os11g11810.1	NBS-LRR disease resistance protein, putative, expressed	2,05	-1.0	1	22	1379	1400	AUAAAACCUUCAGCUAUCCAUC	AAUGGAUAUCUGAAGGUUUCAU	Cleavage
tae-miR7757-5p	Traes_4DS_5B29EE3DD.6	PF00931				GO:GO:0043531	AT3G50950.1	HOPZ-ACTIVATED RESISTANCE 1	LOC_Os11g11810.1	NBS-LRR disease resistance protein, putative, expressed	2,05	-1.0	1	22	1379	1400	AUAAAACCUUCAGCUAUCCAUC	AAUGGAUAUCUGAAGGUUUCAU	Cleavage
tae-miR7757-5p	Traes_4DS_5B29EE3DD.7	PF00931				GO:GO:0043531	AT1G58390.1	Disease resistance protein (CC-NBS-LRR class) family	LOC_Os11g11810.1	NBS-LRR disease resistance protein, putative, expressed	2,05	-1.0	1	22	824	845	AUAAAACCUUCAGCUAUCCAUC	AAUGGAUAUCUGAAGGUUUCAU	Cleavage
tae-miR1130b-3p	Traes_4DS_9179FF158.4	PF06964		3.2.1.55		GO:GO:0046556,GO:GO:0046373	AT3G10740.1	alpha-L-arabinofuranosidase 1	LOC_Os12g03470.1	alpha-N-arabinofuranosidase A, putative, expressed	1,05	-1.0	1	21	41	61	UCUUAUAUUAUGGGACGGAGG	CCUCCGUCCCAUAAUGUAAGA	Cleavage
tae-miR9778	Traes_4DS_B08D83A2F.1	PF00931				GO:GO:0043531	AT3G46530.1	NB-ARC domain-containing disease resistance protein	LOC_Os11g39550.1	Leucine Rich Repeat family protein, expressed	2,05	-1.0	1	21	126	146	UGCAUCAUCUCGAACUCGUCG	UGGCGAGUUCCAGAUGAUGCA	Translation
tae-miR164	Traes_4DS_C08BF9DB2.1			2.4.1.14			AT1G04920.1	sucrose phosphate synthase 3F	LOC_Os11g12810.1	sucrose-phosphate synthase, putative, expressed	2,05	-1.0	1	21	387	407	UGGAGAAGCAGGGCACGUGCA	UUCACGUGCCCUGCUUUUCCU	Cleavage
tae-miR1128	Traes_4DS_C22A101FE.2						AT1G07910.1	RNAligase	LOC_Os07g09400.1	RNA ligase isoform 1, putative, expressed	2,05	-1.0	1	21	670	690	UACUACUCCCUCCGUCCGAAA	UUUGGGACGAAGGGAGUAGUA	Cleavage
tae-miR1128	Traes_4DS_C22A101FE.9						AT1G07910.1	RNAligase	LOC_Os07g09400.1	RNA ligase isoform 1, putative, expressed	2,05	-1.0	1	21	358	378	UACUACUCCCUCCGUCCGAAA	UUUGGGACGAAGGGAGUAGUA	Cleavage
tae-miR1122a	Traes_4DS_E8239194E.2	PF01394					AT3G08530.1	Clathrin, heavy chain	LOC_Os12g01390.1	clathrin heavy chain, putative, expressed	2,05	-1.0	1	20	692	711	UAGAUACAUCCGUAUCUAGA	UCUAAAUACGGAUGUAUCAA	Cleavage
tae-miR5384-3p	Traes_4DS_ECFDD82BA.1	PF02861	KOG1051		K03695	GO:GO:0019538	AT3G52490.1	Double Clp-N motif-containing P-loop nucleoside triphosphate hydrolases superfamily protein	LOC_Os11g05820.1	transposon protein, putative, Mutator sub-class, expressed	2,05	-1.0	1	21	269	289	UGAGCGCGCCGCCGUCGAAUG	UGUCCCACGGCGGCGCGCACA	Cleavage
tae-miR1137b-5p	Traes_4DS_EFE0451D7.3	PF00270		3.6.4.12		GO:GO:0005524,GO:GO:0003676	AT1G31360.1	RECQ helicase L2	LOC_Os11g48090.5	helicase conserved C-terminal domain containing protein, expressed	1,05	-1.0	1	21	950	970	UCCGUUCCAGAAUAGAUGACC	AGUCAUCUAUUUUGGAACGGA	Translation
tae-miR1128	Traes_5AL_3AB81DF9B.1	PF00056,PF02866	KOG1496	1.1.1.82		GO:GO:0055114,GO:GO:0016491,GO:GO:0016616,GO:GO:0016615,GO:GO:0006108	AT5G58330.1	lactate/malate dehydrogenase family protein	LOC_Os08g44810.1	lactate/malate dehydrogenase, putative, expressed	3,00	-1.0	1	21	151	172	UACUACUCCCUCCG-UCCGAAA	UUUAGGAACGGAGGGAGUAGUA	Cleavage
tae-miR9652-5p	Traes_5AL_481F73E59.1	PF16487,PF16486,PF16488,PF08699,PF02170,PF02171	KOG1041			GO:GO:0005515,GO:GO:0003676	AT5G43810.1	Stabilizer of iron transporter SufD / Polynucleotidyl transferase	LOC_Os03g58600.1	PAZ domain containing protein, putative, expressed	2,05	-1.0	1	22	883	904	CCUGUUUGUCAUUAAGUUUCUU	AAGAAACUGAAUGACAAGCAGG	Cleavage
tae-miR9652-5p	Traes_5AL_481F73E59.2	PF00240,PF16487,PF16486,PF16488,PF08699,PF02170,PF02171	KOG1041			GO:GO:0005515,GO:GO:0003676	AT5G43810.1	Stabilizer of iron transporter SufD / Polynucleotidyl transferase	LOC_Os03g58600.1	PAZ domain containing protein, putative, expressed	2,05	-1.0	1	22	883	904	CCUGUUUGUCAUUAAGUUUCUU	AAGAAACUGAAUGACAAGCAGG	Cleavage
tae-miR9652-5p	Traes_5AL_481F73E59.3	PF00240,PF16487,PF16486,PF16488,PF08699,PF02170,PF02171	KOG1041			GO:GO:0005515,GO:GO:0003676	AT5G43810.2	Stabilizer of iron transporter SufD / Polynucleotidyl transferase	LOC_Os03g58600.1	PAZ domain containing protein, putative, expressed	2,05	-1.0	1	22	883	904	CCUGUUUGUCAUUAAGUUUCUU	AAGAAACUGAAUGACAAGCAGG	Cleavage
tae-miR9652-5p	Traes_5AL_481F73E59.4	PF00240,PF16486,PF16488,PF08699,PF02170,PF02171	KOG1041			GO:GO:0005515,GO:GO:0003676	AT1G48410.1	Stabilizer of iron transporter SufD / Polynucleotidyl transferase	LOC_Os03g58600.1	PAZ domain containing protein, putative, expressed	2,05	-1.0	1	22	883	904	CCUGUUUGUCAUUAAGUUUCUU	AAGAAACUGAAUGACAAGCAGG	Cleavage
tae-miR9652-5p	Traes_5AL_481F73E59.5	PF00240,PF16488,PF08699,PF02170,PF02171				GO:GO:0005515,GO:GO:0003676	AT1G48410.1	Stabilizer of iron transporter SufD / Polynucleotidyl transferase	LOC_Os03g58600.1	PAZ domain containing protein, putative, expressed	2,05	-1.0	1	22	583	604	CCUGUUUGUCAUUAAGUUUCUU	AAGAAACUGAAUGACAAGCAGG	Cleavage
tae-miR9652-5p	Traes_5AL_481F73E59.6	PF02171,PF16486,PF16488,PF08699,PF02170				GO:GO:0003676,GO:GO:0005515	AT1G48410.1	Stabilizer of iron transporter SufD / Polynucleotidyl transferase	LOC_Os03g58600.1	PAZ domain containing protein, putative, expressed	2,05	-1.0	1	22	883	904	CCUGUUUGUCAUUAAGUUUCUU	AAGAAACUGAAUGACAAGCAGG	Cleavage
tae-miR5062-5p	Traes_5AL_500FF0B72.1	PF02171				GO:GO:0003676	AT2G27880.1	Argonaute family protein	LOC_Os03g58600.1	PAZ domain containing protein, putative, expressed	2,05	-1.0	1	23	43	65	UGAACCUUAGGGAACAGCCGCAU	ACCAGGCUAUUCCCUGAGGUUCA	Cleavage
tae-miR5062-5p	Traes_5AL_60D577E34.1	PF02171				GO:GO:0003676	AT2G27880.1	Argonaute family protein	LOC_Os03g58600.1	PAZ domain containing protein, putative, expressed	1,05	-1.0	1	23	43	65	UGAACCUUAGGGAACAGCCGCAU	ACCAGGCUGUUCCCUGAGGUUCA	Cleavage
tae-miR1127b-3p	Traes_5AL_7256A9F19.2	PF00400	KOG0279			GO:GO:0005515	AT3G18860.2	transducin family protein / WD-40 repeat family protein	LOC_Os07g03160.1	WD domain, G-beta repeat domain containing protein, expressed	1,05	-1.0	1	21	991	1011	ACAAGUAUUUCUGGACGGAGG	CCUCCGUCCGGAAAUACUUGU	Cleavage
tae-miR1122b-3p	Traes_5AL_7FF780810.1						AT5G41940.1	Ypt/Rab-GAP domain of gyp1p superfamily protein	LOC_Os09g35960.1	TBC domain containing protein, expressed	1,05	-1.0	1	21	22	42	AGACUUAUAUGUAGGAACGGA	UCCGUUCCUAAAUAUAAGUCU	Translation
tae-miR5086	Traes_5AL_8B87707C4.1										3,00	-1.0	1	21	350	370	ACAUUGGUGGAAGGCGUGGUA	AGUCAUGCCUUCCACCAAUGA	Cleavage
tae-miR5086	Traes_5AL_8B87707C4.2										3,00	-1.0	1	21	350	370	ACAUUGGUGGAAGGCGUGGUA	AGUCAUGCCUUCCACCAAUGA	Cleavage
tae-miR5086	Traes_5AL_8B87707C4.3										3,00	-1.0	1	21	326	346	ACAUUGGUGGAAGGCGUGGUA	AGUCAUGCCUUCCACCAAUGA	Cleavage
tae-miR7757-5p	Traes_5AL_B4E8A3115.1	PF00931				GO:GO:0043531	AT3G46710.1	NB-ARC domain-containing disease resistance protein	LOC_Os05g40150.1	RGH2B, putative, expressed	1,05	-1.0	1	22	368	389	AUAAAACCUUCAGCUAUCCAUC	AAUGGAUAGCUGAAGGUCUUAU	Cleavage
tae-miR7757-5p	Traes_5AL_B4E8A3115.2	PF03106,PF12796,PF00931	KOG4658			GO:GO:0043565,GO:GO:0006355,GO:GO:0003700,GO:GO:0043531	AT2G31820.1	Ankyrin repeat family protein	LOC_Os11g45750.2	WRKY125, expressed	1,05	-1.0	1	22	2324	2345	AUAAAACCUUCAGCUAUCCAUC	AAUGGAUAGCUGAAGGUCUUAU	Cleavage
tae-miR7757-5p	Traes_5AL_B4E8A3115.3	PF03106,PF12796,PF00931	KOG4658			GO:GO:0043565,GO:GO:0006355,GO:GO:0003700,GO:GO:0043531	AT2G31820.1	Ankyrin repeat family protein	LOC_Os11g45750.2	WRKY125, expressed	1,05	-1.0	1	22	2324	2345	AUAAAACCUUCAGCUAUCCAUC	AAUGGAUAGCUGAAGGUCUUAU	Cleavage
tae-miR7757-5p	Traes_5AL_B4E8A3115.4	PF03106,PF12796,PF00931	KOG4658			GO:GO:0043565,GO:GO:0006355,GO:GO:0003700,GO:GO:0043531	AT2G31820.1	Ankyrin repeat family protein	LOC_Os11g45750.2	WRKY125, expressed	1,05	-1.0	1	22	2324	2345	AUAAAACCUUCAGCUAUCCAUC	AAUGGAUAGCUGAAGGUCUUAU	Cleavage
tae-miR7757-5p	Traes_5AL_B4E8A3115.5	PF03106,PF12796,PF00931	KOG4658			GO:GO:0043565,GO:GO:0006355,GO:GO:0003700,GO:GO:0043531	AT2G31820.1	Ankyrin repeat family protein	LOC_Os11g45750.1	WRKY125, expressed	1,05	-1.0	1	22	2324	2345	AUAAAACCUUCAGCUAUCCAUC	AAUGGAUAGCUGAAGGUCUUAU	Cleavage
tae-miR7757-5p	Traes_5AL_B4E8A3115.6	PF00931,PF03106				GO:GO:0043531,GO:GO:0043565,GO:GO:0006355,GO:GO:0003700	AT3G46710.1	NB-ARC domain-containing disease resistance protein	LOC_Os05g40150.1	RGH2B, putative, expressed	1,05	-1.0	1	22	368	389	AUAAAACCUUCAGCUAUCCAUC	AAUGGAUAGCUGAAGGUCUUAU	Cleavage
tae-miR9652-5p	Traes_5AL_C9236F111.1	PF16487,PF16488,PF08699,PF02170				GO:GO:0005515	AT1G48410.2	Stabilizer of iron transporter SufD / Polynucleotidyl transferase	LOC_Os03g58600.1	PAZ domain containing protein, putative, expressed	2,05	-1.0	1	22	778	799	CCUGUUUGUCAUUAAGUUUCUU	AAGAAACUGAAUGACAAGCAGG	Cleavage
tae-miR5384-3p	Traes_5AS_A928BA201.1	PF04535					AT5G44550.1	Uncharacterised protein family (UPF0497)	LOC_Os12g41690.1	membrane associated DUF588 domain containing protein, putative, expressed	2,05	-1.0	1	21	506	526	UGAGCGCGCCGCCGUCGAAUG	CAUUCGUCGGCGUCGCGCUCA	Cleavage
tae-miR7757-5p	Traes_5AS_BDFB08E23.1	PF00931	KOG4658			GO:GO:0043531	AT3G07040.1	NB-ARC domain-containing disease resistance protein	LOC_Os12g37740.1	resistance protein LR10, putative, expressed	2,05	-1.0	1	22	1349	1370	AUAAAACCUUCAGCUAUCCAUC	GAUGGAUUGCUGAAGGUUUCAU	Cleavage
tae-miR7757-5p	Traes_5AS_BDFB08E23.2	PF00931	KOG4658			GO:GO:0043531	AT3G07040.1	NB-ARC domain-containing disease resistance protein	LOC_Os12g37740.1	resistance protein LR10, putative, expressed	2,05	-1.0	1	22	1349	1370	AUAAAACCUUCAGCUAUCCAUC	GAUGGAUUGCUGAAGGUUUCAU	Cleavage
tae-miR7757-5p	Traes_5AS_BDFB08E23.3	PF00931				GO:GO:0043531	AT3G07040.1	NB-ARC domain-containing disease resistance protein	LOC_Os12g37740.1	resistance protein LR10, putative, expressed	2,05	-1.0	1	22	524	545	AUAAAACCUUCAGCUAUCCAUC	GAUGGAUUGCUGAAGGUUUCAU	Cleavage
tae-miR7757-5p	Traes_5AS_BDFB08E23.4	PF00931				GO:GO:0043531	AT3G46530.1	NB-ARC domain-containing disease resistance protein	LOC_Os12g37740.1	resistance protein LR10, putative, expressed	2,05	-1.0	1	22	1349	1370	AUAAAACCUUCAGCUAUCCAUC	GAUGGAUUGCUGAAGGUUUCAU	Cleavage
tae-miR7757-5p	Traes_5AS_BDFB08E23.5	PF00931				GO:GO:0043531	AT3G46530.1	NB-ARC domain-containing disease resistance protein	LOC_Os12g37740.1	resistance protein LR10, putative, expressed	2,05	-1.0	1	22	1349	1370	AUAAAACCUUCAGCUAUCCAUC	GAUGGAUUGCUGAAGGUUUCAU	Cleavage
tae-miR1128	Traes_5AS_E1F692526.2										3,00	-1.0	1	21	111	132	UACUACUCCCUCCGUCCGA-AA	UUAUGGGACGGAGGGAGUAGUA	Cleavage
tae-miR1130b-3p	Traes_5AS_E1F692526.2										3,00	-1.0	1	21	40	61	UCUU-AUAUUAUGGGACGGAGG	CCUCCGUCCCAUAAUAUUAAGA	Cleavage
tae-miR1122c-3p	Traes_5AS_E1F692526.2										3,00	-1.0	1	21	40	61	UC-UAAUAUUAUGGGACGGAGG	CCUCCGUCCCAUAAUAUUAAGA	Cleavage
tae-miR164	Traes_5AS_E5FD82E36.1	PF02365				GO:GO:0006355,GO:GO:0003677	AT1G56010.2	NAC domain containing protein 1	LOC_Os12g41680.1	No apical meristem protein, putative, expressed	1,00	-1.0	1	21	653	673	UGGAGAAGCAGGGCACGUGCA	AGCAAGUGCCCUGCUUCUCCA	Cleavage
tae-miR164	Traes_5AS_E5FD82E36.2	PF02365				GO:GO:0006355,GO:GO:0003677	AT1G56010.2	NAC domain containing protein 1	LOC_Os12g41680.1	No apical meristem protein, putative, expressed	1,00	-1.0	1	21	656	676	UGGAGAAGCAGGGCACGUGCA	AGCAAGUGCCCUGCUUCUCCA	Cleavage
tae-miR1118	Traes_5AS_EC41EF7CA.4	PF00013	KOG2190			GO:GO:0003723	AT3G04610.1	RNA-binding KH domain-containing protein	LOC_Os12g40560.1	KH domain containing protein, putative, expressed	1,05	-1.0	1	23	1333	1355	CACUACAUUAUGGAAUGGAGGGA	UCCCUCCAUUCCACAAUGUAGUG	Translation
tae-miR1125	Traes_5AS_EC41EF7CA.4	PF00013	KOG2190			GO:GO:0003723	AT3G04610.1	RNA-binding KH domain-containing protein	LOC_Os12g40560.1	KH domain containing protein, putative, expressed	1,00	-1.0	1	24	1450	1473	AACCAACGAGACCAACUGCGGCGG	CCGCCGCAGUCGGUCUCGUUGGUU	Cleavage
tae-miR408	Traes_5BL_0212ACBD9.1										2,05	-1.0	1	21	2512	2532	CUGCACUGCCUCUUCCCUGGC	GCCAAGGAAGAGGCUGUGCAG	Cleavage
tae-miR1137a	Traes_5BL_0EC4788EF.2	PF08244,PF00251	KOG0228	3.2.1.26			AT1G55120.1	beta-fructofuranosidase 5	LOC_Os01g73580.1	glycosyl hydrolases, putative, expressed	1,00	-1.0	1	20	1543	1562	UAGUACAAAGUUGAGUCAUC	GAUGACCCAACUUUGUACUA	Cleavage
tae-miR9652-5p	Traes_5BL_0F64FDA44.1	PF08699,PF02170				GO:GO:0005515	AT1G48410.1	Stabilizer of iron transporter SufD / Polynucleotidyl transferase	LOC_Os03g58600.1	PAZ domain containing protein, putative, expressed	2,05	-1.0	1	22	583	604	CCUGUUUGUCAUUAAGUUUCUU	AAGAAACUGAAUGACAAGCAGG	Cleavage
tae-miR1122a	Traes_5BL_1B9186ED8.1	PF07762							LOC_Os11g07360.1	expressed protein	2,05	-1.0	1	20	986	1005	UAGAUACAUCCGUAUCUAGA	UCGAGAUACAGAUGUAUCUA	Translation
tae-miR7757-5p	Traes_5BL_1E751EF1F.1	PF00931,PF03514	KOG4658			GO:GO:0043531	AT1G50600.1	scarecrow-like 5	LOC_Os10g22484.1	RGH2B, putative, expressed	2,00	-1.0	1	22	1799	1820	AUAAAACCUUCAGCUAUCCAUC	AAUGGGUUGCUGAAGGUUUUAU	Cleavage
tae-miR7757-5p	Traes_5BL_1E751EF1F.2	PF00931				GO:GO:0043531	AT3G46730.1	NB-ARC domain-containing disease resistance protein	LOC_Os10g22484.1	RGH2B, putative, expressed	2,00	-1.0	1	22	1799	1820	AUAAAACCUUCAGCUAUCCAUC	AAUGGGUUGCUGAAGGUUUUAU	Cleavage
tae-miR7757-5p	Traes_5BL_1E751EF1F.5	PF00931,PF03514				GO:GO:0043531	AT1G50600.1	scarecrow-like 5	LOC_Os10g22484.1	RGH2B, putative, expressed	2,00	-1.0	1	22	512	533	AUAAAACCUUCAGCUAUCCAUC	AAUGGGUUGCUGAAGGUUUUAU	Cleavage
tae-miR5049-3p	Traes_5BL_4BFE76791.5								LOC_Os08g04590.1	csAtPR5, putative, expressed	2,05	-1.0	1	21	416	436	AAUAUGGAUCGGAGGGAGUAC	AUGCUCCCUCUGAUCCAUAUU	Translation
tae-miR1127b-3p	Traes_5BL_5E94C243F.1						AT3G54630.1		LOC_Os08g36490.1	kinetochore protein, putative, expressed	2,05	-1.0	1	21	914	934	ACAAGUAUUUCUGGACGGAGG	CCUCUGUCCUGAAAUACUUGU	Cleavage
tae-miR1127b-3p	Traes_5BL_5E94C243F.2						AT3G54630.1		LOC_Os08g36490.1	kinetochore protein, putative, expressed	2,05	-1.0	1	21	898	918	ACAAGUAUUUCUGGACGGAGG	CCUCUGUCCUGAAAUACUUGU	Cleavage
tae-miR9674b-5p	Traes_5BL_6216A6108.1	PF13041					AT3G22470.1	Pentatricopeptide repeat (PPR) superfamily protein	LOC_Os10g35240.2	Rf1, mitochondrial precursor, putative, expressed	3,00	-1.0	1	21	411	431	AUAGCAUCAUCCAUCCUACCC	GGGCCGAAUGGAUGAUGCUAU	Cleavage
tae-miR5384-3p	Traes_5BL_6240DE6BA.1	PF00067		1.14.13.89		GO:GO:0055114,GO:GO:0020037,GO:GO:0016705,GO:GO:0005506	AT5G67310.1	cytochrome P450, family 81, subfamily G, polypeptide 1	LOC_Os03g55240.1	cytochrome P450, putative, expressed	2,05	-1.0	1	21	338	358	UGAGCGCGCCGCCGUCGAAUG	CCUUCGACGGCGCCGCGCUCG	Cleavage
tae-miR5384-3p	Traes_5BL_6240DE6BA.2	PF00067		1.14.13.89		GO:GO:0055114,GO:GO:0020037,GO:GO:0016705,GO:GO:0005506	AT5G67310.1	cytochrome P450, family 81, subfamily G, polypeptide 1	LOC_Os03g55240.1	cytochrome P450, putative, expressed	2,05	-1.0	1	21	338	358	UGAGCGCGCCGCCGUCGAAUG	CCUUCGACGGCGCCGCGCUCG	Cleavage
tae-miR5384-3p	Traes_5BL_6240DE6BA.3	PF00067		1.14.13.89		GO:GO:0055114,GO:GO:0020037,GO:GO:0016705,GO:GO:0005506	AT5G67310.1	cytochrome P450, family 81, subfamily G, polypeptide 1	LOC_Os03g55240.1	cytochrome P450, putative, expressed	2,05	-1.0	1	21	338	358	UGAGCGCGCCGCCGUCGAAUG	CCUUCGACGGCGCCGCGCUCG	Cleavage
tae-miR5384-3p	Traes_5BL_6240DE6BA.4	PF00067		1.14.13.89		GO:GO:0055114,GO:GO:0020037,GO:GO:0016705,GO:GO:0005506	AT5G67310.1	cytochrome P450, family 81, subfamily G, polypeptide 1	LOC_Os03g55240.1	cytochrome P450, putative, expressed	2,05	-1.0	1	21	338	358	UGAGCGCGCCGCCGUCGAAUG	CCUUCGACGGCGCCGCGCUCG	Cleavage
tae-miR1122a	Traes_5BL_637A3C970.8	PF01501		2.4.1.43		GO:GO:0016757,GO:GO:0047262	AT5G47780.1	galacturonosyltransferase 4	LOC_Os09g30280.1	glycosyl transferase 8 domain containing protein, putative, expressed	1,05	-1.0	1	20	49	68	UAGAUACAUCCGUAUCUAGA	UCUAGAUACGAAUGUAUCUA	Translation
tae-miR1121	Traes_5BL_72EFF0AF0.1	PF07690				GO:GO:0055085,GO:GO:0016021	AT5G59250.1	Major facilitator superfamily protein	LOC_Os10g42830.1	transporter family protein, putative, expressed	0,00	-1.0	1	22	1237	1258	AGUAGUGAUCUAAACGCUCUUA	UAAGAGCGUUUAGAUCACUACU	Cleavage
tae-miR408	Traes_5BL_7486582EB.1										2,05	-1.0	1	21	8	28	CUGCACUGCCUCUUCCCUGGC	CGCAGGGAAGAGGCAGUGCCA	Cleavage
tae-miR1122a	Traes_5BL_7821274AB.1	PF07762							LOC_Os11g07360.1	expressed protein	2,05	-1.0	1	20	986	1005	UAGAUACAUCCGUAUCUAGA	UCGAGAUACAGAUGUAUCUA	Translation
tae-miR7757-5p	Traes_5BL_7F0FD1538.2	PF00847,PF00931	KOG4658			GO:GO:0006355,GO:GO:0003700,GO:GO:0043531	AT3G46710.1	NB-ARC domain-containing disease resistance protein	LOC_Os05g40150.1	RGH2B, putative, expressed	1,05	-1.0	1	22	2096	2117	AUAAAACCUUCAGCUAUCCAUC	AAUGGAUAGCUGAAAGUUUUAU	Cleavage
tae-miR7757-5p	Traes_5BL_7F0FD1538.3	PF00847,PF00931	KOG4658			GO:GO:0006355,GO:GO:0003700,GO:GO:0043531	AT3G46710.1	NB-ARC domain-containing disease resistance protein	LOC_Os05g40150.1	RGH2B, putative, expressed	1,05	-1.0	1	22	1379	1400	AUAAAACCUUCAGCUAUCCAUC	AAUGGAUAGCUGAAAGUUUUAU	Cleavage
tae-miR7757-5p	Traes_5BL_7F0FD1538.4	PF00931	KOG4658			GO:GO:0043531	AT3G46710.1	NB-ARC domain-containing disease resistance protein	LOC_Os05g40150.1	RGH2B, putative, expressed	1,05	-1.0	1	22	1379	1400	AUAAAACCUUCAGCUAUCCAUC	AAUGGAUAGCUGAAAGUUUUAU	Cleavage
tae-miR5086	Traes_5BL_83D9CB7C3.1										3,00	-1.0	1	21	497	517	ACAUUGGUGGAAGGCGUGGUA	AGUCAUGCCUUCCACCAAUGA	Cleavage
tae-miR5086	Traes_5BL_83D9CB7C3.2										3,00	-1.0	1	21	608	628	ACAUUGGUGGAAGGCGUGGUA	AGUCAUGCCUUCCACCAAUGA	Cleavage
tae-miR5086	Traes_5BL_83D9CB7C3.3										3,00	-1.0	1	21	473	493	ACAUUGGUGGAAGGCGUGGUA	AGUCAUGCCUUCCACCAAUGA	Cleavage
tae-miR5086	Traes_5BL_83D9CB7C3.4										3,00	-1.0	1	21	608	628	ACAUUGGUGGAAGGCGUGGUA	AGUCAUGCCUUCCACCAAUGA	Cleavage
tae-miR5086	Traes_5BL_83D9CB7C3.5										3,00	-1.0	1	21	497	517	ACAUUGGUGGAAGGCGUGGUA	AGUCAUGCCUUCCACCAAUGA	Cleavage
tae-miR156	Traes_5BL_96D5EA4EE.1	PF03110				GO:GO:0005634,GO:GO:0003677	AT2G42200.1	squamosa promoter binding protein-like 9	LOC_Os09g31438.1	OsSPL17 - SBP-box gene family member, expressed	1,00	-1.0	1	21	805	825	UGACAGAAGAGAGUGAGCACA	UGUGCUCUCUCUCUUCUGUCA	Cleavage
tae-miR156	Traes_5BL_96D5EA4EE.2	PF03110				GO:GO:0005634,GO:GO:0003677	AT2G42200.1	squamosa promoter binding protein-like 9	LOC_Os09g31438.1	OsSPL17 - SBP-box gene family member, expressed	1,00	-1.0	1	21	802	822	UGACAGAAGAGAGUGAGCACA	UGUGCUCUCUCUCUUCUGUCA	Cleavage
tae-miR156	Traes_5BL_96D5EA4EE.3								LOC_Os09g31438.1	OsSPL17 - SBP-box gene family member, expressed	1,00	-1.0	1	21	67	87	UGACAGAAGAGAGUGAGCACA	UGUGCUCUCUCUCUUCUGUCA	Cleavage
tae-miR156	Traes_5BL_96FDFC152.1	PF03110				GO:GO:0005634,GO:GO:0003677	AT1G76580.1	Squamosa promoter-binding protein-like (SBP domain) transcription factor family protein	LOC_Os09g32944.1	OsSPL18 - SBP-box gene family member, expressed	1,00	-1.0	1	21	508	528	UGACAGAAGAGAGUGAGCACA	UGUGCUCUCUCUCUUCUGUCA	Cleavage
tae-miR156	Traes_5BL_96FDFC152.2	PF03110				GO:GO:0005634,GO:GO:0003677	AT5G50570.1	Squamosa promoter-binding protein-like (SBP domain) transcription factor family protein	LOC_Os09g32944.1	OsSPL18 - SBP-box gene family member, expressed	1,00	-1.0	1	21	490	510	UGACAGAAGAGAGUGAGCACA	UGUGCUCUCUCUCUUCUGUCA	Cleavage
tae-miR5384-3p	Traes_5BL_AD734CCEF.1	PF12695	KOG1515	3.1.1.1			AT3G48690.1	alpha/beta-Hydrolases superfamily protein	LOC_Os09g28730.1	gibberellin receptor GID1L2, putative, expressed	2,05	-1.0	1	21	242	262	UGAGCGCGCCGCCGUCGAAUG	UCUUCCACGGCGGCGCGUUCA	Cleavage
tae-miR9664-3p	Traes_5BL_D27214492.1	PF00931				GO:GO:0043531	AT3G46530.1	NB-ARC domain-containing disease resistance protein	LOC_Os11g11550.1	NBS-LRR disease resistance protein, putative, expressed	2,05	-1.0	1	21	237	257	UUGCAGUCCUCGAUGUCGUAG	GCACGACAUCGAGGAUUGCAU	Cleavage
tae-miR9664-3p	Traes_5BL_D27214492.2	PF00931				GO:GO:0043531	AT3G46530.1	NB-ARC domain-containing disease resistance protein	LOC_Os11g11550.2	NBS-LRR disease resistance protein, putative, expressed	2,05	-1.0	1	21	237	257	UUGCAGUCCUCGAUGUCGUAG	GCACGACAUCGAGGAUUGCAU	Cleavage
tae-miR9664-3p	Traes_5BL_D27214492.3	PF00931				GO:GO:0043531	AT3G46530.1	NB-ARC domain-containing disease resistance protein	LOC_Os11g11550.2	NBS-LRR disease resistance protein, putative, expressed	2,05	-1.0	1	21	237	257	UUGCAGUCCUCGAUGUCGUAG	GCACGACAUCGAGGAUUGCAU	Cleavage
tae-miR9664-3p	Traes_5BL_D27214492.4	PF00931				GO:GO:0043531	AT3G46530.1	NB-ARC domain-containing disease resistance protein	LOC_Os11g11550.2	NBS-LRR disease resistance protein, putative, expressed	2,05	-1.0	1	21	237	257	UUGCAGUCCUCGAUGUCGUAG	GCACGACAUCGAGGAUUGCAU	Cleavage
tae-miR9664-3p	Traes_5BL_D27214492.5	PF00931				GO:GO:0043531	AT3G46530.1	NB-ARC domain-containing disease resistance protein	LOC_Os11g11550.1	NBS-LRR disease resistance protein, putative, expressed	2,05	-1.0	1	21	237	257	UUGCAGUCCUCGAUGUCGUAG	GCACGACAUCGAGGAUUGCAU	Cleavage
tae-miR7757-5p	Traes_5BL_DD4C9F446.1	PF00069	KOG1187	2.7.11.1		GO:GO:0006468,GO:GO:0005524,GO:GO:0004672	AT3G55950.1	CRINKLY4 related 3	LOC_Os05g40150.1	RGH2B, putative, expressed	1,05	-1.0	1	22	62	83	AUAAAACCUUCAGCUAUCCAUC	AAUGGAUAGCGGAAGGUUUUAU	Cleavage
tae-miR7757-5p	Traes_5BL_DD4C9F446.2	PF00069	KOG1187	2.7.11.1		GO:GO:0006468,GO:GO:0005524,GO:GO:0004672	AT3G55950.1	CRINKLY4 related 3	LOC_Os05g40150.1	RGH2B, putative, expressed	1,05	-1.0	1	22	62	83	AUAAAACCUUCAGCUAUCCAUC	AAUGGAUAGCGGAAGGUUUUAU	Cleavage
tae-miR7757-5p	Traes_5BL_DD4C9F446.4						AT3G46710.1	NB-ARC domain-containing disease resistance protein	LOC_Os05g40150.1	RGH2B, putative, expressed	1,05	-1.0	1	22	62	83	AUAAAACCUUCAGCUAUCCAUC	AAUGGAUAGCGGAAGGUUUUAU	Cleavage
tae-miR7757-5p	Traes_5BL_DD4C9F446.5						AT3G46710.1	NB-ARC domain-containing disease resistance protein	LOC_Os05g40150.1	RGH2B, putative, expressed	1,05	-1.0	1	22	62	83	AUAAAACCUUCAGCUAUCCAUC	AAUGGAUAGCGGAAGGUUUUAU	Cleavage
tae-miR7757-5p	Traes_5BL_DD4C9F446.6						AT3G46710.1	NB-ARC domain-containing disease resistance protein	LOC_Os05g40150.1	RGH2B, putative, expressed	1,05	-1.0	1	22	62	83	AUAAAACCUUCAGCUAUCCAUC	AAUGGAUAGCGGAAGGUUUUAU	Cleavage
tae-miR9652-5p	Traes_5BL_FC199BC2D.1	PF16488,PF02170				GO:GO:0005515	AT1G48410.2	Stabilizer of iron transporter SufD / Polynucleotidyl transferase	LOC_Os03g58600.1	PAZ domain containing protein, putative, expressed	2,05	-1.0	1	22	412	433	CCUGUUUGUCAUUAAGUUUCUU	AAGAAACUGAAUGACAAGCAGG	Cleavage
tae-miR1122b-3p	Traes_5BS_321AF7B69.2	PF14416,PF13839					AT5G20680.3	TRICHOME BIREFRINGENCE-LIKE 16	LOC_Os12g33194.1	expressed protein	2,05	-1.0	1	21	23	43	AGACUUAUAUGUAGGAACGGA	UCCAUUCCUAAAUAUAAGUCU	Translation
tae-miR1122a	Traes_5BS_321AF7B69.2	PF14416,PF13839					AT5G20680.3	TRICHOME BIREFRINGENCE-LIKE 16	LOC_Os12g33194.1	expressed protein	2,05	-1.0	1	20	63	82	UAGAUACAUCCGUAUCUAGA	ACUACAUACGGAUGUAUAUA	Cleavage
tae-miR7757-5p	Traes_5BS_3FFCC89AD.1	PF00931	KOG4658			GO:GO:0043531	AT3G07040.1	NB-ARC domain-containing disease resistance protein	LOC_Os12g37770.1	RGH1A, putative, expressed	2,05	-1.0	1	22	1337	1358	AUAAAACCUUCAGCUAUCCAUC	GGUGGAUUGCUGAAGGAUUUAU	Cleavage
tae-miR7757-5p	Traes_5BS_3FFCC89AD.2	PF00931	KOG4658			GO:GO:0043531	AT3G46730.1	NB-ARC domain-containing disease resistance protein	LOC_Os12g37770.1	RGH1A, putative, expressed	2,05	-1.0	1	22	1298	1319	AUAAAACCUUCAGCUAUCCAUC	GGUGGAUUGCUGAAGGAUUUAU	Cleavage
tae-miR1118	Traes_5BS_550BDEE86.2	PF00013	KOG2190			GO:GO:0003723	AT3G04610.1	RNA-binding KH domain-containing protein	LOC_Os12g40560.1	KH domain containing protein, putative, expressed	1,05	-1.0	1	23	1135	1157	CACUACAUUAUGGAAUGGAGGGA	UCCCUCCAUUCCACAAUGUAGUG	Translation
tae-miR5384-3p	Traes_5BS_90796C6CA.1	PF07762							LOC_Os01g56900.1	expressed protein	2,00	-1.0	1	21	461	481	UGAGCGCGCCGCCGUCGAAUG	UGUACGAUGGCGGCGCGCUCA	Cleavage
tae-miR5384-3p	Traes_5BS_90796C6CA.2	PF07762							LOC_Os01g56900.1	expressed protein	2,00	-1.0	1	21	34	54	UGAGCGCGCCGCCGUCGAAUG	UGUACGAUGGCGGCGCGCUCA	Cleavage
tae-miR5384-3p	Traes_5BS_90796C6CA.3	PF07762							LOC_Os01g56900.1	expressed protein	2,00	-1.0	1	21	392	412	UGAGCGCGCCGCCGUCGAAUG	UGUACGAUGGCGGCGCGCUCA	Cleavage

tae-miR164	Traes_5BS_9F043AE17.1	PF02365				GO:GO:0006355,GO:GO:0003677	AT1G56010.2	NAC domain containing protein 1	LOC_Os12g41680.1	No apical meristem protein, putative, expressed	1,00	-1.0	1	21	656	676	UGGAGAAGCAGGGCACGUGCA	AGCAAGUGCCCUGCUUCUCCA	Cleavage
tae-miR5384-3p	Traes_5BS_BA0400E10.1	PF14570,PF03552		2.4.2.24	K00770	GO:GO:0030244,GO:GO:0016760,GO:GO:0016020	AT1G02730.1	cellulose synthase-like D5	LOC_Os12g36890.1	CSLD4 - cellulose synthase-like family D, expressed	3,00	-1.0	1	21	1358	1378	UGAGCGCGCCGCCGUCGAAUG	CCGACGACGGCGGCGCGCUCC	Cleavage
tae-miR7757-5p	Traes_5BS_BCEA0112C.1	PF00931	KOG4658			GO:GO:0043531	AT3G07040.1	NB-ARC domain-containing disease resistance protein	LOC_Os12g37740.1	resistance protein LR10, putative, expressed	2,05	-1.0	1	22	1340	1361	AUAAAACCUUCAGCUAUCCAUC	GAUGGAUCGCGGAAGGUUUUAU	Cleavage
tae-miR7757-5p	Traes_5BS_BCEA0112C.2	PF00931	KOG4658			GO:GO:0043531	AT3G07040.1	NB-ARC domain-containing disease resistance protein	LOC_Os12g37740.1	resistance protein LR10, putative, expressed	2,05	-1.0	1	22	1340	1361	AUAAAACCUUCAGCUAUCCAUC	GAUGGAUCGCGGAAGGUUUUAU	Cleavage
tae-miR7757-5p	Traes_5BS_BCEA0112C.3	PF00931	KOG4658			GO:GO:0043531	AT3G07040.1	NB-ARC domain-containing disease resistance protein	LOC_Os12g37740.1	resistance protein LR10, putative, expressed	2,05	-1.0	1	22	1307	1328	AUAAAACCUUCAGCUAUCCAUC	GAUGGAUCGCGGAAGGUUUUAU	Cleavage
tae-miR7757-5p	Traes_5BS_BCEA0112C.4	PF00931				GO:GO:0043531	AT3G07040.1	NB-ARC domain-containing disease resistance protein	LOC_Os12g37740.1	resistance protein LR10, putative, expressed	2,05	-1.0	1	22	128	149	AUAAAACCUUCAGCUAUCCAUC	GAUGGAUCGCGGAAGGUUUUAU	Cleavage
tae-miR408	Traes_5BS_E10756355.1										2,05	-1.0	1	21	2512	2532	CUGCACUGCCUCUUCCCUGGC	GCCAAGGAAGAGGCUGUGCAG	Cleavage
tae-miR7757-5p	Traes_5BS_F5AFB24CD.2	PF00931				GO:GO:0043531	AT3G46530.1	NB-ARC domain-containing disease resistance protein	LOC_Os10g03570.1	RGH1A, putative, expressed	1,05	-1.0	1	22	779	800	AUAAAACCUUCAGCUAUCCAUC	GGUGGAUAGCUGAAGGUCUUAU	Cleavage
tae-miR7757-5p	Traes_5BS_F5AFB24CD.3	PF00931				GO:GO:0043531	AT3G46530.1	NB-ARC domain-containing disease resistance protein	LOC_Os10g03570.1	RGH1A, putative, expressed	1,05	-1.0	1	22	779	800	AUAAAACCUUCAGCUAUCCAUC	GGUGGAUAGCUGAAGGUCUUAU	Cleavage
tae-miR7757-5p	Traes_5BS_F5AFB24CD.4	PF00931				GO:GO:0043531	AT5G43470.2	Disease resistance protein (CC-NBS-LRR class) family	LOC_Os10g03570.1	RGH1A, putative, expressed	1,05	-1.0	1	22	371	392	AUAAAACCUUCAGCUAUCCAUC	GGUGGAUAGCUGAAGGUCUUAU	Cleavage
tae-miR156	Traes_5DL_1272B4BAC.1	PF03110				GO:GO:0005634,GO:GO:0003677	AT5G50670.1	Squamosa promoter-binding protein-like (SBP domain) transcription factor family protein	LOC_Os09g32944.1	OsSPL18 - SBP-box gene family member, expressed	1,00	-1.0	1	21	505	525	UGACAGAAGAGAGUGAGCACA	UGUGCUCUCUCUCUUCUGUCA	Cleavage
tae-miR156	Traes_5DL_1272B4BAC.2	PF03110				GO:GO:0005634,GO:GO:0003677	AT5G50670.1	Squamosa promoter-binding protein-like (SBP domain) transcription factor family protein	LOC_Os09g32944.1	OsSPL18 - SBP-box gene family member, expressed	1,00	-1.0	1	21	487	507	UGACAGAAGAGAGUGAGCACA	UGUGCUCUCUCUCUUCUGUCA	Cleavage
tae-miR156	Traes_5DL_1272B4BAC.3								LOC_Os09g32944.1	OsSPL18 - SBP-box gene family member, expressed	1,00	-1.0	1	21	382	402	UGACAGAAGAGAGUGAGCACA	UGUGCUCUCUCUCUUCUGUCA	Cleavage
tae-miR7757-5p	Traes_5DL_1733FB4DA.1	PF00931,PF03106				GO:GO:0043531,GO:GO:0043565,GO:GO:0006355,GO:GO:0003700	AT3G46530.1	NB-ARC domain-containing disease resistance protein	LOC_Os05g40150.1	RGH2B, putative, expressed	1,05	-1.0	1	22	617	638	AUAAAACCUUCAGCUAUCCAUC	AAUGGAUAGCUGAAGGAUUUAU	Cleavage
tae-miR7757-5p	Traes_5DL_1733FB4DA.2	PF03106,PF00931				GO:GO:0043565,GO:GO:0006355,GO:GO:0003700,GO:GO:0043531	AT3G46530.1	NB-ARC domain-containing disease resistance protein	LOC_Os05g40150.1	RGH2B, putative, expressed	1,05	-1.0	1	22	617	638	AUAAAACCUUCAGCUAUCCAUC	AAUGGAUAGCUGAAGGAUUUAU	Cleavage
tae-miR7757-5p	Traes_5DL_1733FB4DA.3	PF03106,PF00931				GO:GO:0043565,GO:GO:0006355,GO:GO:0003700,GO:GO:0043531	AT3G46530.1	NB-ARC domain-containing disease resistance protein	LOC_Os05g40150.1	RGH2B, putative, expressed	1,05	-1.0	1	22	617	638	AUAAAACCUUCAGCUAUCCAUC	AAUGGAUAGCUGAAGGAUUUAU	Cleavage
tae-miR7757-5p	Traes_5DL_1733FB4DA.4	PF00931				GO:GO:0043531	AT3G46530.1	NB-ARC domain-containing disease resistance protein	LOC_Os05g40150.1	RGH2B, putative, expressed	1,05	-1.0	1	22	617	638	AUAAAACCUUCAGCUAUCCAUC	AAUGGAUAGCUGAAGGAUUUAU	Cleavage
tae-miR7757-5p	Traes_5DL_1733FB4DA.5	PF00931				GO:GO:0043531	AT3G46530.1	NB-ARC domain-containing disease resistance protein	LOC_Os05g40150.1	RGH2B, putative, expressed	1,05	-1.0	1	22	617	638	AUAAAACCUUCAGCUAUCCAUC	AAUGGAUAGCUGAAGGAUUUAU	Cleavage
tae-miR7757-5p	Traes_5DL_1733FB4DA.6	PF00931				GO:GO:0043531	AT3G46530.1	NB-ARC domain-containing disease resistance protein	LOC_Os05g40150.1	RGH2B, putative, expressed	1,05	-1.0	1	22	617	638	AUAAAACCUUCAGCUAUCCAUC	AAUGGAUAGCUGAAGGAUUUAU	Cleavage
tae-miR7757-5p	Traes_5DL_1733FB4DA.7	PF00931				GO:GO:0043531	AT3G46530.1	NB-ARC domain-containing disease resistance protein	LOC_Os11g45750.1	WRKY125, expressed	1,05	-1.0	1	22	617	638	AUAAAACCUUCAGCUAUCCAUC	AAUGGAUAGCUGAAGGAUUUAU	Cleavage
tae-miR7757-5p	Traes_5DL_1733FB4DA.8	PF00931				GO:GO:0043531	AT3G46530.1	NB-ARC domain-containing disease resistance protein	LOC_Os11g45750.2	WRKY125, expressed	1,05	-1.0	1	22	617	638	AUAAAACCUUCAGCUAUCCAUC	AAUGGAUAGCUGAAGGAUUUAU	Cleavage
tae-miR5062-5p	Traes_5DL_26B51AAF8.1	PF02171				GO:GO:0003676	AT1G48410.2	Stabilizer of iron transporter SufD / Polynucleotidyl transferase	LOC_Os03g58600.1	PAZ domain containing protein, putative, expressed	1,05	-1.0	1	23	43	65	UGAACCUUAGGGAACAGCCGCAU	ACCAGGCUGUUCCCUGAGGUUCA	Cleavage
tae-miR1127b-3p	Traes_5DL_4E67E0243.9	PF03828		2.7.7.19			AT3G51620.2	PAP/OAS1 substrate-binding domain superfamily	LOC_Os12g02260.1	nucleotidyltransferase, putative, expressed	1,05	-1.0	1	21	41	61	ACAAGUAUUUCUGGACGGAGG	CCUCCGUCCGGAAAUACUUGU	Cleavage
tae-miR9652-5p	Traes_5DL_714CCB5BC.1	PF16488,PF02170				GO:GO:0005515	AT1G48410.3	Stabilizer of iron transporter SufD / Polynucleotidyl transferase	LOC_Os03g58600.1	PAZ domain containing protein, putative, expressed	2,05	-1.0	1	22	412	433	CCUGUUUGUCAUUAAGUUUCUU	AAGAAACUGAAUGACAAGCAGG	Cleavage
tae-miR9662a-3p	Traes_5DL_7643B2ADB.1	PF02536				GO:GO:0006355,GO:GO:0005739,GO:GO:0003690	AT5G64950.1	Mitochondrial transcription termination factor family protein	LOC_Os06g12100.1	mTERF family protein, expressed	2,05	-1.0	1	21	411	431	UUGAACAUCCCAGAGCCACCG	CCGUGGCUCUGUGAUGUUCAG	Translation
tae-miR9662b-3p	Traes_5DL_7643B2ADB.1	PF02536				GO:GO:0006355,GO:GO:0005739,GO:GO:0003690	AT5G64950.1	Mitochondrial transcription termination factor family protein	LOC_Os06g12100.1	mTERF family protein, expressed	2,05	-1.0	1	21	410	430	UGAACAUCCCAGAGCCACCGG	CCCGUGGCUCUGUGAUGUUCA	Cleavage
tae-miR5384-3p	Traes_5DL_78767F75C.1	PF00135	KOG1515	3.1.1.1			AT3G48690.1	alpha/beta-Hydrolases superfamily protein	LOC_Os09g28730.1	gibberellin receptor GID1L2, putative, expressed	2,05	-1.0	1	21	242	262	UGAGCGCGCCGCCGUCGAAUG	UCUUCCACGGCGGCGCGUUCA	Cleavage
tae-miR5049-3p	Traes_5DL_799647EA7.4										1,05	-1.0	1	21	65	85	AAUAUGGAUCGGAGGGAGUAC	GUACUCCCUCCGUUCCAUAUU	Cleavage
tae-miR9652-5p	Traes_5DL_8DE6C67FA.1	PF02171,PF16487,PF16488,PF02170				GO:GO:0003676,GO:GO:0005515	AT1G48410.2	Stabilizer of iron transporter SufD / Polynucleotidyl transferase	LOC_Os03g58600.1	PAZ domain containing protein, putative, expressed	2,05	-1.0	1	22	412	433	CCUGUUUGUCAUUAAGUUUCUU	AAGAAACUGAAUGACAAGCAGG	Cleavage
tae-miR9652-5p	Traes_5DL_8DE6C67FA.2	PF16487,PF16488,PF02170				GO:GO:0005515	AT1G48410.3	Stabilizer of iron transporter SufD / Polynucleotidyl transferase	LOC_Os03g58600.1	PAZ domain containing protein, putative, expressed	2,05	-1.0	1	22	412	433	CCUGUUUGUCAUUAAGUUUCUU	AAGAAACUGAAUGACAAGCAGG	Cleavage
tae-miR9652-5p	Traes_5DL_8DE6C67FA.3	PF16487,PF16488,PF02170				GO:GO:0005515	AT1G48410.3	Stabilizer of iron transporter SufD / Polynucleotidyl transferase	LOC_Os03g58600.1	PAZ domain containing protein, putative, expressed	2,05	-1.0	1	22	415	436	CCUGUUUGUCAUUAAGUUUCUU	AAGAAACUGAAUGACAAGCAGG	Cleavage
tae-miR9652-5p	Traes_5DL_8DE6C67FA.4	PF02170				GO:GO:0005515	AT1G48410.3	Stabilizer of iron transporter SufD / Polynucleotidyl transferase	LOC_Os03g58600.1	PAZ domain containing protein, putative, expressed	2,05	-1.0	1	22	400	421	CCUGUUUGUCAUUAAGUUUCUU	AAGAAACUGAAUGACAAGCAGG	Cleavage
tae-miR9652-5p	Traes_5DL_8DE6C67FA.5	PF02170				GO:GO:0005515	AT1G48410.2	Stabilizer of iron transporter SufD / Polynucleotidyl transferase	LOC_Os03g58600.1	PAZ domain containing protein, putative, expressed	2,05	-1.0	1	22	400	421	CCUGUUUGUCAUUAAGUUUCUU	AAGAAACUGAAUGACAAGCAGG	Cleavage
tae-miR408	Traes_5DL_90BD9F306.1										0,00	-1.0	1	21	8	28	CUGCACUGCCUCUUCCCUGGC	CCCAGGGAAGAGGCAGUGCAG	Cleavage
tae-miR9666b-3p	Traes_5DL_9101D5AEC.1	PF05001		2.7.7.6		GO:GO:0006366,GO:GO:0005665,GO:GO:0003677	AT4G35800.1	RNA polymerase II large subunit	LOC_Os05g05860.1	retrotransposon protein, putative, unclassified, expressed	2,05	-1.0	1	22	1183	1204	CGGUUGGGCUGUAUGAUGGCGA	AGUCCAUCAUACAGCCCAACAA	Cleavage
tae-miR1130b-3p	Traes_5DL_A6EECBC04.1								LOC_Os03g32160.1	calmodulin binding protein, putative, expressed	1,05	-1.0	1	21	376	396	UCUUAUAUUAUGGGACGGAGG	AUUCCGUCCCAUAAUGUAAGA	Cleavage
tae-miR9652-5p	Traes_5DL_B3713A4D1.1	PF16488,PF08699,PF16486,PF02170				GO:GO:0005515	AT1G48410.1	Stabilizer of iron transporter SufD / Polynucleotidyl transferase	LOC_Os03g58600.1	PAZ domain containing protein, putative, expressed	2,05	-1.0	1	22	940	961	CCUGUUUGUCAUUAAGUUUCUU	AAGAAACUGAAUGACAAGCAGG	Cleavage
tae-miR5086	Traes_5DL_CECECF832.1										3,00	-1.0	1	21	461	481	ACAUUGGUGGAAGGCGUGGUA	AGUCAUGCCUUCCACCAAUGA	Cleavage
tae-miR5086	Traes_5DL_CECECF832.2										3,00	-1.0	1	21	485	505	ACAUUGGUGGAAGGCGUGGUA	AGUCAUGCCUUCCACCAAUGA	Cleavage
tae-miR5086	Traes_5DL_CECECF832.3										3,00	-1.0	1	21	101	121	ACAUUGGUGGAAGGCGUGGUA	AGUCAUGCCUUCCACCAAUGA	Cleavage
tae-miR5086	Traes_5DL_CECECF832.4										3,00	-1.0	1	21	485	505	ACAUUGGUGGAAGGCGUGGUA	AGUCAUGCCUUCCACCAAUGA	Cleavage
tae-miR5086	Traes_5DL_CECECF832.5										3,00	-1.0	1	21	101	121	ACAUUGGUGGAAGGCGUGGUA	AGUCAUGCCUUCCACCAAUGA	Cleavage
tae-miR5062-5p	Traes_5DL_F5D3E4763.1	PF02171				GO:GO:0003676	AT1G48410.3	Stabilizer of iron transporter SufD / Polynucleotidyl transferase	LOC_Os03g58600.1	PAZ domain containing protein, putative, expressed	2,05	-1.0	1	23	43	65	UGAACCUUAGGGAACAGCCGCAU	ACCAGGUUGUUCCCUGAGGUUCA	Cleavage
tae-miR7757-5p	Traes_5DS_0479C6E0F.1	PF00931				GO:GO:0043531	AT3G07040.1	NB-ARC domain-containing disease resistance protein	LOC_Os12g06920.1	NBS-LRR disease resistance protein, putative, expressed	2,05	-1.0	1	22	383	404	AUAAAACCUUCAGCUAUCCAUC	GAUGGAUCGCUGAAGGUUUGAU	Cleavage
tae-miR9662a-3p	Traes_5DS_094A03C13.1	PF02536				GO:GO:0006355,GO:GO:0005739,GO:GO:0003690	AT5G07900.1	Mitochondrial transcription termination factor family protein	LOC_Os06g12100.1	mTERF family protein, expressed	1,00	-1.0	1	21	549	569	UUGAACAUCCCAGAGCCACCG	CCGUGGCUCUGGGAUGUUCAG	Cleavage
tae-miR9662b-3p	Traes_5DS_094A03C13.1	PF02536				GO:GO:0006355,GO:GO:0005739,GO:GO:0003690	AT5G07900.1	Mitochondrial transcription termination factor family protein	LOC_Os06g12100.1	mTERF family protein, expressed	1,00	-1.0	1	21	548	568	UGAACAUCCCAGAGCCACCGG	CCCGUGGCUCUGGGAUGUUCA	Cleavage
tae-miR9662a-3p	Traes_5DS_094A03C13.2	PF02536				GO:GO:0006355,GO:GO:0005739,GO:GO:0003690	AT5G07900.1	Mitochondrial transcription termination factor family protein	LOC_Os06g12100.1	mTERF family protein, expressed	1,00	-1.0	1	21	243	263	UUGAACAUCCCAGAGCCACCG	CCGUGGCUCUGGGAUGUUCAG	Cleavage
tae-miR9662b-3p	Traes_5DS_094A03C13.2	PF02536				GO:GO:0006355,GO:GO:0005739,GO:GO:0003690	AT5G07900.1	Mitochondrial transcription termination factor family protein	LOC_Os06g12100.1	mTERF family protein, expressed	1,00	-1.0	1	21	242	262	UGAACAUCCCAGAGCCACCGG	CCCGUGGCUCUGGGAUGUUCA	Cleavage
tae-miR5384-3p	Traes_5DS_149714893.1	PF04535					AT5G44550.1	Uncharacterised protein family (UPF0497)	LOC_Os12g41690.1	membrane associated DUF588 domain containing protein, putative, expressed	2,05	-1.0	1	21	158	178	UGAGCGCGCCGCCGUCGAAUG	CGUUCGUCGGCGUCGCGCUCA	Cleavage
tae-miR5384-3p	Traes_5DS_149714893.2	PF04535					AT5G44550.1	Uncharacterised protein family (UPF0497)	LOC_Os12g41690.1	membrane associated DUF588 domain containing protein, putative, expressed	2,05	-1.0	1	21	152	172	UGAGCGCGCCGCCGUCGAAUG	CGUUCGUCGGCGUCGCGCUCA	Cleavage
tae-miR164	Traes_5DS_511F35A45.1						AT1G56010.2	NAC domain containing protein 1	LOC_Os12g41680.1	No apical meristem protein, putative, expressed	1,00	-1.0	1	21	209	229	UGGAGAAGCAGGGCACGUGCA	AGCAAGUGCCCUGCUUCUCCA	Cleavage
tae-miR7757-5p	Traes_5DS_8DB03B7DA.1	PF00931	KOG4658			GO:GO:0043531	AT3G07040.1	NB-ARC domain-containing disease resistance protein	LOC_Os12g37740.1	resistance protein LR10, putative, expressed	2,05	-1.0	1	22	1349	1370	AUAAAACCUUCAGCUAUCCAUC	GAUGGAUCGCGGAAGGUUUUAU	Cleavage
tae-miR7757-5p	Traes_5DS_8DB03B7DA.2	PF00931				GO:GO:0043531	AT3G07040.1	NB-ARC domain-containing disease resistance protein	LOC_Os12g37740.1	resistance protein LR10, putative, expressed	2,05	-1.0	1	22	128	149	AUAAAACCUUCAGCUAUCCAUC	GAUGGAUCGCGGAAGGUUUUAU	Cleavage
tae-miR7757-5p	Traes_5DS_9DFE4D217.1	PF00931				GO:GO:0043531	AT1G53350.1	Disease resistance protein (CC-NBS-LRR class) family	LOC_Os10g22484.1	RGH2B, putative, expressed	2,05	-1.0	1	22	185	206	AUAAAACCUUCAGCUAUCCAUC	AAUGGGUAGCUGAAGGUUGUAU	Cleavage
tae-miR7757-5p	Traes_5DS_9DFE4D217.2	PF00931				GO:GO:0043531	AT1G53350.1	Disease resistance protein (CC-NBS-LRR class) family	LOC_Os10g22484.1	RGH2B, putative, expressed	2,05	-1.0	1	22	185	206	AUAAAACCUUCAGCUAUCCAUC	AAUGGGUAGCUGAAGGUUGUAU	Cleavage
tae-miR7757-5p	Traes_5DS_9DFE4D217.3	PF00931				GO:GO:0043531	AT1G53350.1	Disease resistance protein (CC-NBS-LRR class) family	LOC_Os10g22484.1	RGH2B, putative, expressed	2,05	-1.0	1	22	185	206	AUAAAACCUUCAGCUAUCCAUC	AAUGGGUAGCUGAAGGUUGUAU	Cleavage
tae-miR9662a-3p	Traes_5DS_9F4A5AE9F.1	PF02536				GO:GO:0006355,GO:GO:0005739,GO:GO:0003690	AT5G07900.1	Mitochondrial transcription termination factor family protein	LOC_Os06g12100.1	mTERF family protein, expressed	2,00	-1.0	1	21	441	461	UUGAACAUCCCAGAGCCACCG	UCGUGGCGCUGGGAUGUUCAG	Cleavage
tae-miR9662b-3p	Traes_5DS_9F4A5AE9F.1	PF02536				GO:GO:0006355,GO:GO:0005739,GO:GO:0003690	AT5G07900.1	Mitochondrial transcription termination factor family protein	LOC_Os06g12100.1	mTERF family protein, expressed	2,05	-1.0	1	21	440	460	UGAACAUCCCAGAGCCACCGG	CUCGUGGCGCUGGGAUGUUCA	Cleavage
tae-miR9662a-3p	Traes_5DS_9F4A5AE9F.2	PF02536				GO:GO:0006355,GO:GO:0005739,GO:GO:0003690	AT5G07900.1	Mitochondrial transcription termination factor family protein	LOC_Os06g12100.1	mTERF family protein, expressed	2,00	-1.0	1	21	66	86	UUGAACAUCCCAGAGCCACCG	UCGUGGCGCUGGGAUGUUCAG	Cleavage
tae-miR9662b-3p	Traes_5DS_9F4A5AE9F.2	PF02536				GO:GO:0006355,GO:GO:0005739,GO:GO:0003690	AT5G07900.1	Mitochondrial transcription termination factor family protein	LOC_Os06g12100.1	mTERF family protein, expressed	2,05	-1.0	1	21	65	85	UGAACAUCCCAGAGCCACCGG	CUCGUGGCGCUGGGAUGUUCA	Cleavage
tae-miR1122b-3p	Traes_5DS_9F8FFD461.1	PF00792,PF00613		2.7.1.137		GO:GO:0048015,GO:GO:0046854	AT1G60490.1	vacuolar protein sorting 34	LOC_Os05g08810.2	phosphatidylinositol 3-kinase, root isoform, putative, expressed	1,05	-1.0	1	21	1777	1797	AGACUUAUAUGUAGGAACGGA	UCCGUUCCUAAAUAUAAGUCU	Translation
tae-miR1122b-3p	Traes_5DS_9F8FFD461.2	PF00792,PF00613		2.7.1.137		GO:GO:0048015,GO:GO:0046854	AT1G60490.1	vacuolar protein sorting 34	LOC_Os05g08810.2	phosphatidylinositol 3-kinase, root isoform, putative, expressed	1,05	-1.0	1	21	1882	1902	AGACUUAUAUGUAGGAACGGA	UCCGUUCCUAAAUAUAAGUCU	Translation
tae-miR1122b-3p	Traes_5DS_9F8FFD461.3	PF00792,PF00613		2.7.1.137		GO:GO:0048015,GO:GO:0046854	AT1G60490.1	vacuolar protein sorting 34	LOC_Os05g08810.2	phosphatidylinositol 3-kinase, root isoform, putative, expressed	1,05	-1.0	1	21	1801	1821	AGACUUAUAUGUAGGAACGGA	UCCGUUCCUAAAUAUAAGUCU	Translation
tae-miR1122b-3p	Traes_5DS_A6AE32DCD.1	PF03483	KOG2472	6.1.1.20		GO:GO:0004826,GO:GO:0003723	AT1G72550.1	tRNA synthetase beta subunit family protein	LOC_Os05g48510.1	phenylalanyl-tRNA synthetase beta chain, putative, expressed	1,05	-1.0	1	21	1065	1085	AGACUUAUAUGUAGGAACGGA	UCCGUUCCUAAAUAUAAGUCU	Translation
tae-miR1122b-3p	Traes_5DS_A6AE32DCD.2	PF03484,PF03483		6.1.1.20		GO:GO:0006432,GO:GO:0005524,GO:GO:0003723,GO:GO:0000287,GO:GO:0004826	AT1G72550.2	tRNA synthetase beta subunit family protein	LOC_Os05g48510.1	phenylalanyl-tRNA synthetase beta chain, putative, expressed	1,05	-1.0	1	21	1146	1166	AGACUUAUAUGUAGGAACGGA	UCCGUUCCUAAAUAUAAGUCU	Translation
tae-miR1122b-3p	Traes_5DS_A6AE32DCD.3	PF03484,PF03483		6.1.1.20		GO:GO:0006432,GO:GO:0005524,GO:GO:0003723,GO:GO:0000287,GO:GO:0004826	AT1G72550.2	tRNA synthetase beta subunit family protein	LOC_Os05g48510.1	phenylalanyl-tRNA synthetase beta chain, putative, expressed	1,05	-1.0	1	21	1146	1166	AGACUUAUAUGUAGGAACGGA	UCCGUUCCUAAAUAUAAGUCU	Translation
tae-miR9662a-3p	Traes_5DS_C87840EBA.2						AT1G79220.1	Mitochondrial transcription termination factor family protein	LOC_Os06g12100.1	mTERF family protein, expressed	2,00	-1.0	1	21	429	449	UUGAACAUCCCAGAGCCACCG	CCGUGGUUCUGGGAUGUUCAG	Cleavage
tae-miR9662b-3p	Traes_5DS_C87840EBA.2						AT1G79220.1	Mitochondrial transcription termination factor family protein	LOC_Os06g12100.1	mTERF family protein, expressed	2,00	-1.0	1	21	428	448	UGAACAUCCCAGAGCCACCGG	CCCGUGGUUCUGGGAUGUUCA	Cleavage
tae-miR1128	Traes_5DS_D165422B3.1	PF00503	KOG0082			GO:GO:0031683,GO:GO:0019001,GO:GO:0007186,GO:GO:0004871,GO:GO:0003924	AT2G23460.1	extra-large G-protein 1	LOC_Os12g40190.1	G-protein alpha subunit, putative, expressed	3,00	-1.0	1	21	59	80	UACUACUCCCUCCG-UCCGAAA	UUUAGGAACGGAGGGAGUAGUA	Cleavage
tae-miR1118	Traes_5DS_F8D323D4D.2	PF00013	KOG2190			GO:GO:0003723	AT3G04610.1	RNA-binding KH domain-containing protein	LOC_Os12g40560.1	KH domain containing protein, putative, expressed	1,05	-1.0	1	23	1329	1351	CACUACAUUAUGGAAUGGAGGGA	CUCCUCCAUUCCACAAUGUAGUG	Translation
tae-miR160	Traes_6AL_232AF0B76.1						AT4G30080.1	auxin response factor 16	LOC_Os02g41800.1	auxin response factor, putative, expressed	0,00	-1.0	1	21	195	215	UGCCUGGCUCCCUGUAUGCCA	AGGCAUACAGGGAGCCAGGCA	Cleavage
tae-miR1121	Traes_6AL_23B31796F.2	PF00230	KOG0223			GO:GO:0016020,GO:GO:0006810,GO:GO:0005215	AT3G53420.1	plasma membrane intrinsic protein 2A	LOC_Os02g41860.3	aquaporin protein, putative, expressed	2,00	-1.0	1	22	846	867	AGUAGUGAUCUAAACGCUCUUA	UAAAAGUGUUUAGAUCACUACU	Cleavage
tae-miR1121	Traes_6AL_23B31796F.3	PF00230	KOG0223			GO:GO:0016020,GO:GO:0006810,GO:GO:0005215	AT3G53420.1	plasma membrane intrinsic protein 2A	LOC_Os02g41860.3	aquaporin protein, putative, expressed	2,00	-1.0	1	22	760	781	AGUAGUGAUCUAAACGCUCUUA	UAAAAGUGUUUAGAUCACUACU	Cleavage
tae-miR1121	Traes_6AL_23B31796F.5	PF00230	KOG0223			GO:GO:0016020,GO:GO:0006810,GO:GO:0005215	AT3G53420.1	plasma membrane intrinsic protein 2A	LOC_Os02g41860.3	aquaporin protein, putative, expressed	2,00	-1.0	1	22	742	763	AGUAGUGAUCUAAACGCUCUUA	UAAAAGUGUUUAGAUCACUACU	Cleavage
tae-miR5049-3p	Traes_6AL_36AE1B4CF.4	PF03031		3.1.3.16			AT4G21670.1	C-terminal domain phosphatase-like 1	LOC_Os02g42600.1	double-stranded RNA binding motif containing protein, expressed	1,05	-1.0	1	21	1787	1807	AAUAUGGAUCGGAGGGAGUAC	AUACUCCCUCCGACCCAUAUU	Cleavage
tae-miR5049-3p	Traes_6AL_36AE1B4CF1.4	PF03031		3.1.3.16			AT4G21670.1	C-terminal domain phosphatase-like 1	LOC_Os02g42600.1	double-stranded RNA binding motif containing protein, expressed	1,05	-1.0	1	21	1787	1807	AAUAUGGAUCGGAGGGAGUAC	AUACUCCCUCCGACCCAUAUU	Cleavage
tae-miR5049-3p	Traes_6AL_6AD463C67.3			3.6.4.12			AT2G01440.1	DEAD/DEAH box RNA helicase family protein			2,05	-1.0	1	21	15	35	AAUAUGGAUCGGAGGGAGUAC	GUACUCCUUCCGAUCCAUAAU	Cleavage
tae-miR1121	Traes_6AL_7E70268F1.3	PF00719		3.6.1.1		GO:GO:0006796,GO:GO:0005737,GO:GO:0004427,GO:GO:0000287	AT2G46860.1	pyrophosphorylase 3	LOC_Os02g47600.1	soluble inorganic pyrophosphatase, putative, expressed	0,00	-1.0	1	22	497	518	AGUAGUGAUCUAAACGCUCUUA	UAAGAGCGUUUAGAUCACUACU	Cleavage
tae-miR1122b-3p	Traes_6AL_94CA6F0D8.5	PF00067				GO:GO:0055114,GO:GO:0020037,GO:GO:0016705,GO:GO:0005506	AT1G31800.1	cytochrome P450, family 97, subfamily A, polypeptide 3	LOC_Os02g57290.2	cytochrome P450, putative, expressed	2,05	-1.0	1	21	1030	1050	AGACUUAUAUGUAGGAACGGA	UCCAUUCCUAAAUAUAAGUCU	Translation
tae-miR1122a	Traes_6AL_94CA6F0D8.5	PF00067				GO:GO:0055114,GO:GO:0020037,GO:GO:0016705,GO:GO:0005506	AT1G31800.1	cytochrome P450, family 97, subfamily A, polypeptide 3	LOC_Os02g57290.2	cytochrome P450, putative, expressed	2,05	-1.0	1	20	1071	1090	UAGAUACAUCCGUAUCUAGA	ACUACAUACGGAUGUAUAUA	Cleavage
tae-miR9662a-3p	Traes_6AS_0A204AEC9.1	PF02536	KOG1267		K15032	GO:GO:0006355,GO:GO:0005739,GO:GO:0003690	AT5G07900.1	Mitochondrial transcription termination factor family protein	LOC_Os06g12100.1	mTERF family protein, expressed	2,00	-1.0	1	21	681	701	UUGAACAUCCCAGAGCCACCG	CCGUGCCUCUGGGAUGUUCAG	Cleavage
tae-miR9662b-3p	Traes_6AS_0A204AEC9.1	PF02536	KOG1267		K15032	GO:GO:0006355,GO:GO:0005739,GO:GO:0003690	AT5G07900.1	Mitochondrial transcription termination factor family protein	LOC_Os06g12100.1	mTERF family protein, expressed	2,00	-1.0	1	21	680	700	UGAACAUCCCAGAGCCACCGG	CCCGUGCCUCUGGGAUGUUCA	Cleavage
tae-miR9662a-3p	Traes_6AS_0A204AEC9.2	PF02536	KOG1267		K15032	GO:GO:0006355,GO:GO:0005739,GO:GO:0003690	AT5G07900.1	Mitochondrial transcription termination factor family protein	LOC_Os06g12100.1	mTERF family protein, expressed	2,00	-1.0	1	21	681	701	UUGAACAUCCCAGAGCCACCG	CCGUGCCUCUGGGAUGUUCAG	Cleavage
tae-miR9662b-3p	Traes_6AS_0A204AEC9.2	PF02536	KOG1267		K15032	GO:GO:0006355,GO:GO:0005739,GO:GO:0003690	AT5G07900.1	Mitochondrial transcription termination factor family protein	LOC_Os06g12100.1	mTERF family protein, expressed	2,00	-1.0	1	21	680	700	UGAACAUCCCAGAGCCACCGG	CCCGUGCCUCUGGGAUGUUCA	Cleavage
tae-miR9662a-3p	Traes_6AS_1496A7825.1	PF02536				GO:GO:0006355,GO:GO:0005739,GO:GO:0003690	AT5G07900.1	Mitochondrial transcription termination factor family protein	LOC_Os06g12100.1	mTERF family protein, expressed	1,05	-1.0	1	21	549	569	UUGAACAUCCCAGAGCCACCG	CCGUGGCUCGGGGAUGUUCAA	Cleavage
tae-miR9662b-3p	Traes_6AS_1496A7825.1	PF02536				GO:GO:0006355,GO:GO:0005739,GO:GO:0003690	AT5G07900.1	Mitochondrial transcription termination factor family protein	LOC_Os06g12100.1	mTERF family protein, expressed	2,05	-1.0	1	21	548	568	UGAACAUCCCAGAGCCACCGG	CCCGUGGCUCGGGGAUGUUCA	Translation
tae-miR9662a-3p	Traes_6AS_1496A7825.2	PF02536				GO:GO:0006355,GO:GO:0005739,GO:GO:0003690	AT5G07900.1	Mitochondrial transcription termination factor family protein	LOC_Os06g12100.1	mTERF family protein, expressed	1,05	-1.0	1	21	132	152	UUGAACAUCCCAGAGCCACCG	CCGUGGCUCGGGGAUGUUCAA	Cleavage
tae-miR9662b-3p	Traes_6AS_1496A7825.2	PF02536				GO:GO:0006355,GO:GO:0005739,GO:GO:0003690	AT5G07900.1	Mitochondrial transcription termination factor family protein	LOC_Os06g12100.1	mTERF family protein, expressed	2,05	-1.0	1	21	131	151	UGAACAUCCCAGAGCCACCGG	CCCGUGGCUCGGGGAUGUUCA	Translation
tae-miR9662a-3p	Traes_6AS_15C1E6F18.1	PF02536				GO:GO:0006355,GO:GO:0005739,GO:GO:0003690	AT1G21150.1	Mitochondrial transcription termination factor family protein	LOC_Os06g12100.1	mTERF family protein, expressed	2,05	-1.0	1	21	33	53	UUGAACAUCCCAGAGCCACCG	CUGUGGCUCUGGAAUGUUCAG	Cleavage
tae-miR9662b-3p	Traes_6AS_15C1E6F18.1	PF02536				GO:GO:0006355,GO:GO:0005739,GO:GO:0003690	AT1G21150.1	Mitochondrial transcription termination factor family protein	LOC_Os06g12100.1	mTERF family protein, expressed	2,05	-1.0	1	21	32	52	UGAACAUCCCAGAGCCACCGG	CCUGUGGCUCUGGAAUGUUCA	Cleavage
tae-miR9662a-3p	Traes_6AS_15C1E6F18.2	PF02536				GO:GO:0006355,GO:GO:0005739,GO:GO:0003690	AT1G21150.1	Mitochondrial transcription termination factor family protein	LOC_Os06g12100.1	mTERF family protein, expressed	2,05	-1.0	1	21	33	53	UUGAACAUCCCAGAGCCACCG	CUGUGGCUCUGGAAUGUUCAG	Cleavage
tae-miR9662b-3p	Traes_6AS_15C1E6F18.2	PF02536				GO:GO:0006355,GO:GO:0005739,GO:GO:0003690	AT1G21150.1	Mitochondrial transcription termination factor family protein	LOC_Os06g12100.1	mTERF family protein, expressed	2,05	-1.0	1	21	32	52	UGAACAUCCCAGAGCCACCGG	CCUGUGGCUCUGGAAUGUUCA	Cleavage
tae-miR9662a-3p	Traes_6AS_2BDC1663A.1	PF02536	KOG1267		K15032	GO:GO:0006355,GO:GO:0005739,GO:GO:0003690	AT5G07900.1	Mitochondrial transcription termination factor family protein	LOC_Os06g12100.1	mTERF family protein, expressed	1,00	-1.0	1	21	681	701	UUGAACAUCCCAGAGCCACCG	CCGUGGCUCUGGGAUGUUCAG	Cleavage
tae-miR9662b-3p	Traes_6AS_2BDC1663A.1	PF02536	KOG1267		K15032	GO:GO:0006355,GO:GO:0005739,GO:GO:0003690	AT5G07900.1	Mitochondrial transcription termination factor family protein	LOC_Os06g12100.1	mTERF family protein, expressed	1,00	-1.0	1	21	680	700	UGAACAUCCCAGAGCCACCGG	CCCGUGGCUCUGGGAUGUUCA	Cleavage
tae-miR156	Traes_6AS_32A35158A.1	PF03110				GO:GO:0005634,GO:GO:0003677	AT5G43270.2	squamosa promoter binding protein-like 2	LOC_Os06g45310.1	OsSPL11 - SBP-box gene family member, expressed	1,00	-1.0	1	21	736	756	UGACAGAAGAGAGUGAGCACA	CGUGCUCUCUCUCUUCUGUCA	Cleavage
tae-miR156	Traes_6AS_32A35158A.2								LOC_Os06g45310.1	OsSPL11 - SBP-box gene family member, expressed	1,00	-1.0	1	21	292	312	UGACAGAAGAGAGUGAGCACA	CGUGCUCUCUCUCUUCUGUCA	Cleavage
tae-miR156	Traes_6AS_32A35158A.3								LOC_Os06g45310.1	OsSPL11 - SBP-box gene family member, expressed	1,00	-1.0	1	21	64	84	UGACAGAAGAGAGUGAGCACA	CGUGCUCUCUCUCUUCUGUCA	Cleavage
tae-miR9662a-3p	Traes_6AS_3641B9C871.1	PF02536	KOG1267		K15032	GO:GO:0006355,GO:GO:0005739,GO:GO:0003690	AT5G07900.1	Mitochondrial transcription termination factor family protein	LOC_Os06g12100.1	mTERF family protein, expressed	1,00	-1.0	1	21	687	707	UUGAACAUCCCAGAGCCACCG	CCGUGGCUCUGGGAUGUUCAG	Cleavage
tae-miR9662b-3p	Traes_6AS_3641B9C871.1	PF02536	KOG1267		K15032	GO:GO:0006355,GO:GO:0005739,GO:GO:0003690	AT5G07900.1	Mitochondrial transcription termination factor family protein	LOC_Os06g12100.1	mTERF family protein, expressed	1,00	-1.0	1	21	686	706	UGAACAUCCCAGAGCCACCGG	CCCGUGGCUCUGGGAUGUUCA	Cleavage
tae-miR9662a-3p	Traes_6AS_3641B9C871.2	PF02536	KOG1267		K15032	GO:GO:0006355,GO:GO:0005739,GO:GO:0003690	AT5G07900.1	Mitochondrial transcription termination factor family protein	LOC_Os06g12100.1	mTERF family protein, expressed	1,00	-1.0	1	21	687	707	UUGAACAUCCCAGAGCCACCG	CCGUGGCUCUGGGAUGUUCAG	Cleavage
tae-miR9662b-3p	Traes_6AS_3641B9C871.2	PF02536	KOG1267		K15032	GO:GO:0006355,GO:GO:0005739,GO:GO:0003690	AT5G07900.1	Mitochondrial transcription termination factor family protein	LOC_Os06g12100.1	mTERF family protein, expressed	1,00	-1.0	1	21	686	706	UGAACAUCCCAGAGCCACCGG	CCCGUGGCUCUGGGAUGUUCA	Cleavage
tae-miR9662a-3p	Traes_6AS_36EAEADDD.1	PF02536				GO:GO:0006355,GO:GO:0005739,GO:GO:0003690	AT1G21150.1	Mitochondrial transcription termination factor family protein	LOC_Os06g12100.1	mTERF family protein, expressed	2,00	-1.0	1	21	33	53	UUGAACAUCCCAGAGCCACCG	CUGUGGUUCUGGGAUGUUCAG	Cleavage
tae-miR9662b-3p	Traes_6AS_36EAEADDD.1	PF02536				GO:GO:0006355,GO:GO:0005739,GO:GO:0003690	AT1G21150.1	Mitochondrial transcription termination factor family protein	LOC_Os06g12100.1	mTERF family protein, expressed	2,00	-1.0	1	21	32	52	UGAACAUCCCAGAGCCACCGG	CCUGUGGUUCUGGGAUGUUCA	Cleavage
tae-miR9662a-3p	Traes_6AS_36EAEADDD.2								LOC_Os06g12100.1	mTERF family protein, expressed	2,00	-1.0	1	21	33	53	UUGAACAUCCCAGAGCCACCG	CUGUGGUUCUGGGAUGUUCAG	Cleavage
tae-miR9662b-3p	Traes_6AS_36EAEADDD.2								LOC_Os06g12100.1	mTERF family protein, expressed	2,00	-1.0	1	21	32	52	UGAACAUCCCAGAGCCACCGG	CCUGUGGUUCUGGGAUGUUCA	Cleavage
tae-miR9662a-3p	Traes_6AS_36EAEADDD.3	PF02536				GO:GO:0006355,GO:GO:0005739,GO:GO:0003690	AT1G21150.1	Mitochondrial transcription termination factor family protein	LOC_Os06g12100.1	mTERF family protein, expressed	2,00	-1.0	1	21	33	53	UUGAACAUCCCAGAGCCACCG	CUGUGGUUCUGGGAUGUUCAG	Cleavage
tae-miR9662b-3p	Traes_6AS_36EAEADDD.3	PF02536				GO:GO:0006355,GO:GO:0005739,GO:GO:0003690	AT1G21150.1	Mitochondrial transcription termination factor family protein	LOC_Os06g12100.1	mTERF family protein, expressed	2,00	-1.0	1	21	32	52	UGAACAUCCCAGAGCCACCGG	CCUGUGGUUCUGGGAUGUUCA	Cleavage
tae-miR9662a-3p	Traes_6AS_48B701BB41.1	PF02536				GO:GO:0006355,GO:GO:0005739,GO:GO:0003690	AT1G21150.1	Mitochondrial transcription termination factor family protein	LOC_Os06g12100.1	mTERF family protein, expressed	1,00	-1.0	1	21	33	53	UUGAACAUCCCAGAGCCACCG	ACGUGGCUCUGGGAUGUUCAG	Cleavage
tae-miR9662b-3p	Traes_6AS_48B701BB41.1	PF02536				GO:GO:0006355,GO:GO:0005739,GO:GO:0003690	AT1G21150.1	Mitochondrial transcription termination factor family protein	LOC_Os06g12100.1	mTERF family protein, expressed	1,00	-1.0	1	21	32	52	UGAACAUCCCAGAGCCACCGG	CACGUGGCUCUGGGAUGUUCA	Cleavage
tae-miR9662a-3p	Traes_6AS_48B701BB41.2	PF02536				GO:GO:0006355,GO:GO:0005739,GO:GO:0003690	AT1G21150.1	Mitochondrial transcription termination factor family protein	LOC_Os06g12100.1	mTERF family protein, expressed	1,00	-1.0	1	21	33	53	UUGAACAUCCCAGAGCCACCG	ACGUGGCUCUGGGAUGUUCAG	Cleavage
tae-miR9662b-3p	Traes_6AS_48B701BB41.2	PF02536				GO:GO:0006355,GO:GO:0005739,GO:GO:0003690	AT1G21150.1	Mitochondrial transcription termination factor family protein	LOC_Os06g12100.1	mTERF family protein, expressed	1,00	-1.0	1	21	32	52	UGAACAUCCCAGAGCCACCGG	CACGUGGCUCUGGGAUGUUCA	Cleavage
tae-miR9662a-3p	Traes_6AS_48B701BB41.3	PF02536				GO:GO:0006355,GO:GO:0005739,GO:GO:0003690	AT1G21150.1	Mitochondrial transcription termination factor family protein	LOC_Os06g12100.1	mTERF family protein, expressed	1,00	-1.0	1	21	33	53	UUGAACAUCCCAGAGCCACCG	ACGUGGCUCUGGGAUGUUCAG	Cleavage
tae-miR9662b-3p	Traes_6AS_48B701BB41.3	PF02536				GO:GO:0006355,GO:GO:0005739,GO:GO:0003690	AT1G21150.1	Mitochondrial transcription termination factor family protein	LOC_Os06g12100.1	mTERF family protein, expressed	1,00	-1.0	1	21	32	52	UGAACAUCCCAGAGCCACCGG	CACGUGGCUCUGGGAUGUUCA	Cleavage
tae-miR9662a-3p	Traes_6AS_48B701BB41.4	PF02536				GO:GO:0006355,GO:GO:0005739,GO:GO:0003690	AT1G21150.1	Mitochondrial transcription termination factor family protein	LOC_Os06g12100.1	mTERF family protein, expressed	1,00	-1.0	1	21	33	53	UUGAACAUCCCAGAGCCACCG	ACGUGGCUCUGGGAUGUUCAG	Cleavage
tae-miR9662b-3p	Traes_6AS_48B701BB41.4	PF02536				GO:GO:0006355,GO:GO:0005739,GO:GO:0003690	AT1G21150.1	Mitochondrial transcription termination factor family protein	LOC_Os06g12100.1	mTERF family protein, expressed	1,00	-1.0	1	21	32	52	UGAACAUCCCAGAGCCACCGG	CACGUGGCUCUGGGAUGUUCA	Cleavage
tae-miR9662a-3p	Traes_6AS_48B701BB41.5	PF02536				GO:GO:0006355,GO:GO:0005739,GO:GO:0003690	AT1G21150.1	Mitochondrial transcription termination factor family protein	LOC_Os06g12100.1	mTERF family protein, expressed	1,00	-1.0	1	21	33	53	UUGAACAUCCCAGAGCCACCG	ACGUGGCUCUGGGAUGUUCAG	Cleavage
tae-miR9662b-3p	Traes_6AS_48B701BB41.5	PF02536				GO:GO:0006355,GO:GO:0005739,GO:GO:0003690	AT1G21150.1	Mitochondrial transcription termination factor family protein	LOC_Os06g12100.1	mTERF family protein, expressed	1,00	-1.0	1	21	32	52	UGAACAUCCCAGAGCCACCGG	CACGUGGCUCUGGGAUGUUCA	Cleavage
tae-miR1138	Traes_6AS_5F8725CDA.1	PF02705			K03549	GO:GO:0071805,GO:GO:0016020,GO:GO:0015079	AT4G13420.1	high affinity K+ transporter 5	LOC_Os02g31910.2	potassium transporter, putative, expressed	3,00	-1.0	1	23	2223	2245	GCUUAGAUGUGACAUCCUUAAAA	AGUUAGUUAUGUCACAUCUAAGC	Cleavage
tae-miR1138	Traes_6AS_5F8725CDA.2	PF02705				GO:GO:0071805,GO:GO:0016020,GO:GO:0015079	AT4G13420.1	high affinity K+ transporter 5	LOC_Os02g31910.1	potassium transporter, putative, expressed	3,00	-1.0	1	23	1969	1991	GCUUAGAUGUGACAUCCUUAAAA	AGUUAGUUAUGUCACAUCUAAGC	Cleavage
tae-miR9662a-3p	Traes_6AS_6612DF8D7.1	PF02536				GO:GO:0006355,GO:GO:0005739,GO:GO:0003690	AT5G07900.1	Mitochondrial transcription termination factor family protein	LOC_Os06g12100.1	mTERF family protein, expressed	1,00	-1.0	1	21	81	101	UUGAACAUCCCAGAGCCACCG	CCGUGGCUCUGGGAUGUUCAG	Cleavage
tae-miR9662b-3p	Traes_6AS_6612DF8D7.1	PF02536				GO:GO:0006355,GO:GO:0005739,GO:GO:0003690	AT5G07900.1	Mitochondrial transcription termination factor family protein	LOC_Os06g12100.1	mTERF family protein, expressed	1,00	-1.0	1	21	80	100	UGAACAUCCCAGAGCCACCGG	CCCGUGGCUCUGGGAUGUUCA	Cleavage
tae-miR9662a-3p	Traes_6AS_6612DF8D7.2	PF02536				GO:GO:0006355,GO:GO:0005739,GO:GO:0003690	AT5G07900.1	Mitochondrial transcription termination factor family protein	LOC_Os06g12100.1	mTERF family protein, expressed	1,00	-1.0	1	21	81	101	UUGAACAUCCCAGAGCCACCG	CCGUGGCUCUGGGAUGUUCAG	Cleavage
tae-miR9662b-3p	Traes_6AS_6612DF8D7.2	PF02536				GO:GO:0006355,GO:GO:0005739,GO:GO:0003690	AT5G07900.1	Mitochondrial transcription termination factor family protein	LOC_Os06g12100.1	mTERF family protein, expressed	1,00	-1.0	1	21	80	100	UGAACAUCCCAGAGCCACCGG	CCCGUGGCUCUGGGAUGUUCA	Cleavage
tae-miR9662a-3p	Traes_6AS_6612DF8D7.3	PF02536				GO:GO:0006355,GO:GO:0005739,GO:GO:0003690	AT5G07900.1	Mitochondrial transcription termination factor family protein	LOC_Os06g12100.1	mTERF family protein, expressed	1,00	-1.0	1	21	81	101	UUGAACAUCCCAGAGCCACCG	CCGUGGCUCUGGGAUGUUCAG	Cleavage
tae-miR9662b-3p	Traes_6AS_6612DF8D7.3	PF02536				GO:GO:0006355,GO:GO:0005739,GO:GO:0003690	AT5G07900.1	Mitochondrial transcription termination factor family protein	LOC_Os06g12100.1	mTERF family protein, expressed	1,00	-1.0	1	21	80	100	UGAACAUCCCAGAGCCACCGG	CCCGUGGCUCUGGGAUGUUCA	Cleavage
tae-miR9662a-3p	Traes_6AS_8D2111A83.1	PF02536				GO:GO:0006355,GO:GO:0005739,GO:GO:0003690	AT1G21150.1	Mitochondrial transcription termination factor family protein	LOC_Os06g12100.1	mTERF family protein, expressed	2,05	-1.0	1	21	33	53	UUGAACAUCCCAGAGCCACCG	UCGUGGCUCGGGGAUGUUCAG	Cleavage
tae-miR9662b-3p	Traes_6AS_8D2111A83.1	PF02536				GO:GO:0006355,GO:GO:0005739,GO:GO:0003690	AT1G21150.1	Mitochondrial transcription termination factor family protein	LOC_Os06g12100.1	mTERF family protein, expressed	2,05	-1.0	1	21	32	52	UGAACAUCCCAGAGCCACCGG	CUCGUGGCUCGGGGAUGUUCA	Translation
tae-miR9662a-3p	Traes_6AS_8D2111A83.2	PF02536				GO:GO:0006355,GO:GO:0005739,GO:GO:0003690	AT1G21150.1	Mitochondrial transcription termination factor family protein	LOC_Os06g12100.1	mTERF family protein, expressed	2,05	-1.0	1	21	246	266	UUGAACAUCCCAGAGCCACCG	UCGUGGCUCGGGGAUGUUCAG	Cleavage
tae-miR9662b-3p	Traes_6AS_8D2111A83.2	PF02536				GO:GO:0006355,GO:GO:0005739,GO:GO:0003690	AT1G21150.1	Mitochondrial transcription termination factor family protein	LOC_Os06g12100.1	mTERF family protein, expressed	2,05	-1.0	1	21	245	265	UGAACAUCCCAGAGCCACCGG	CUCGUGGCUCGGGGAUGUUCA	Translation
tae-miR9662a-3p	Traes_6AS_8D2111A83.3	PF02536				GO:GO:0006355,GO:GO:0005739,GO:GO:0003690	AT1G21150.1	Mitochondrial transcription termination factor family protein	LOC_Os06g12100.1	mTERF family protein, expressed	2,05	-1.0	1	21	246	266	UUGAACAUCCCAGAGCCACCG	UCGUGGCUCGGGGAUGUUCAG	Cleavage
tae-miR9662b-3p	Traes_6AS_8D2111A83.3	PF02536				GO:GO:0006355,GO:GO:0005739,GO:GO:0003690	AT1G21150.1	Mitochondrial transcription termination factor family protein	LOC_Os06g12100.1	mTERF family protein, expressed	2,05	-1.0	1	21	245	265	UGAACAUCCCAGAGCCACCGG	CUCGUGGCUCGGGGAUGUUCA	Translation
tae-miR9662a-3p	Traes_6AS_8D2111A83.4	PF02536				GO:GO:0006355,GO:GO:0005739,GO:GO:0003690	AT1G21150.1	Mitochondrial transcription termination factor family protein	LOC_Os06g12100.1	mTERF family protein, expressed	2,05	-1.0	1	21	246	266	UUGAACAUCCCAGAGCCACCG	UCGUGGCUCGGGGAUGUUCAG	Cleavage
tae-miR9662b-3p	Traes_6AS_8D2111A83.4	PF02536				GO:GO:0006355,GO:GO:0005739,GO:GO:0003690	AT1G21150.1	Mitochondrial transcription termination factor family protein	LOC_Os06g12100.1	mTERF family protein, expressed	2,05	-1.0	1	21	245	265	UGAACAUCCCAGAGCCACCGG	CUCGUGGCUCGGGGAUGUUCA	Translation
tae-miR9662a-3p	Traes_6AS_8D2111A83.5	PF02536				GO:GO:0006355,GO:GO:0005739,GO:GO:0003690	AT1G21150.1	Mitochondrial transcription termination factor family protein	LOC_Os06g12100.1	mTERF family protein, expressed	2,05	-1.0	1	21	246	266	UUGAACAUCCCAGAGCCACCG	UCGUGGCUCGGGGAUGUUCAG	Cleavage
tae-miR9662b-3p	Traes_6AS_8D2111A83.5	PF02536				GO:GO:0006355,GO:GO:0005739,GO:GO:0003690	AT1G21150.1	Mitochondrial transcription termination factor family protein	LOC_Os06g12100.1	mTERF family protein, expressed	2,05	-1.0	1	21	245	265	UGAACAUCCCAGAGCCACCGG	CUCGUGGCUCGGGGAUGUUCA	Translation
tae-miR9662a-3p	Traes_6AS_8D2111A83.6						AT1G21150.1	Mitochondrial transcription termination factor family protein	LOC_Os06g12100.1	mTERF family protein, expressed	2,05	-1.0	1	21	246	266	UUGAACAUCCCAGAGCCACCG	UCGUGGCUCGGGGAUGUUCAG	Cleavage
tae-miR9662b-3p	Traes_6AS_8D2111A83.6						AT1G21150.1	Mitochondrial transcription termination factor family protein	LOC_Os06g12100.1	mTERF family protein, expressed	2,05	-1.0	1	21	245	265	UGAACAUCCCAGAGCCACCGG	CUCGUGGCUCGGGGAUGUUCA	Translation
tae-miR9662a-3p	Traes_6AS_8D2111A83.7	PF02536				GO:GO:0006355,GO:GO:0005739,GO:GO:0003690	AT1G21150.1	Mitochondrial transcription termination factor family protein	LOC_Os06g12100.1	mTERF family protein, expressed	2,05	-1.0	1	21	33	53	UUGAACAUCCCAGAGCCACCG	UCGUGGCUCGGGGAUGUUCAG	Cleavage
tae-miR9662b-3p	Traes_6AS_8D2111A83.7	PF02536				GO:GO:0006355,GO:GO:0005739,GO:GO:0003690	AT1G21150.1	Mitochondrial transcription termination factor family protein	LOC_Os06g12100.1	mTERF family protein, expressed	2,05	-1.0	1	21	32	52	UGAACAUCCCAGAGCCACCGG	CUCGUGGCUCGGGGAUGUUCA	Translation
tae-miR1122b-3p	Traes_6AS_8E6E52967.2	PF08263					AT1G17250.1	receptor like protein 3	LOC_Os02g06120.1	Leucine Rich Repeat family protein, expressed	1,05	-1.0	1	21	120	140	AGACUUAUAUGUAGGAACGGA	UCCGUUCCUAAAUAUAAGUCU	Translation
tae-miR156	Traes_6AS_99C6A3B0B.1	PF03110				GO:GO:0005634,GO:GO:0003677	AT5G43270.1	squamosa promoter binding protein-like 2	LOC_Os02g04680.2	OsSPL3 - SBP-box gene family member, expressed	1,00	-1.0	1	21	1477	1497	UGACAGAAGAGAGUGAGCACA	CAUGCUCUCUCUCUUCUGUCA	Cleavage
tae-miR156	Traes_6AS_99C6A3B0B.2	PF03110				GO:GO:0005634,GO:GO:0003677	AT5G43270.3	squamosa promoter binding protein-like 2	LOC_Os02g04680.2	OsSPL3 - SBP-box gene family member, expressed	1,00	-1.0	1	21	1477	1497	UGACAGAAGAGAGUGAGCACA	CAUGCUCUCUCUCUUCUGUCA	Cleavage
tae-miR156	Traes_6AS_99C6A3B0B.3	PF03110				GO:GO:0005634,GO:GO:0003677	AT5G43270.2	squamosa promoter binding protein-like 2	LOC_Os02g04680.2	OsSPL3 - SBP-box gene family member, expressed	1,00	-1.0	1	21	1477	1497	UGACAGAAGAGAGUGAGCACA	CAUGCUCUCUCUCUUCUGUCA	Cleavage
tae-miR156	Traes_6AS_99C6A3B0B.4	PF03110				GO:GO:0005634,GO:GO:0003677	AT5G43270.2	squamosa promoter binding protein-like 2	LOC_Os02g04680.2	OsSPL3 - SBP-box gene family member, expressed	1,00	-1.0	1	21	1156	1176	UGACAGAAGAGAGUGAGCACA	CAUGCUCUCUCUCUUCUGUCA	Cleavage
tae-miR156	Traes_6AS_99C6A3B0B.5	PF03110				GO:GO:0005634,GO:GO:0003677	AT5G43270.3	squamosa promoter binding protein-like 2	LOC_Os02g04680.2	OsSPL3 - SBP-box gene family member, expressed	1,00	-1.0	1	21	1156	1176	UGACAGAAGAGAGUGAGCACA	CAUGCUCUCUCUCUUCUGUCA	Cleavage
tae-miR156	Traes_6AS_99C6A3B0B.6	PF03110				GO:GO:0005634,GO:GO:0003677	AT5G43270.1	squamosa promoter binding protein-like 2	LOC_Os02g04680.2	OsSPL3 - SBP-box gene family member, expressed	1,00	-1.0	1	21	1156	1176	UGACAGAAGAGAGUGAGCACA	CAUGCUCUCUCUCUUCUGUCA	Cleavage
tae-miR156	Traes_6AS_99C6A3B0B.7	PF03110				GO:GO:0005634,GO:GO:0003677	AT5G43270.3	squamosa promoter binding protein-like 2	LOC_Os02g04680.2	OsSPL3 - SBP-box gene family member, expressed	1,00	-1.0	1	21	1156	1176	UGACAGAAGAGAGUGAGCACA	CAUGCUCUCUCUCUUCUGUCA	Cleavage
tae-miR156	Traes_6AS_99C6A3B0B.8	PF03110				GO:GO:0005634,GO:GO:0003677	AT5G43270.1	squamosa promoter binding protein-like 2	LOC_Os02g04680.2	OsSPL3 - SBP-box gene family member, expressed	1,00	-1.0	1	21	1156	1176	UGACAGAAGAGAGUGAGCACA	CAUGCUCUCUCUCUUCUGUCA	Cleavage
tae-miR9662a-3p	Traes_6AS_A2EAE01C1.1	PF02536				GO:GO:0006355,GO:GO:0005739,GO:GO:0003690	AT5G07900.1	Mitochondrial transcription termination factor family protein	LOC_Os06g12100.1	mTERF family protein, expressed	2,05	-1.0	1	21	465	485	UUGAACAUCCCAGAGCCACCG	CCGUGGCUCUGGAAUGUUCAG	Cleavage
tae-miR9662b-3p	Traes_6AS_A2EAE01C1.1	PF02536				GO:GO:0006355,GO:GO:0005739,GO:GO:0003690	AT5G07900.1	Mitochondrial transcription termination factor family protein	LOC_Os06g12100.1	mTERF family protein, expressed	2,05	-1.0	1	21	464	484	UGAACAUCCCAGAGCCACCGG	CCCGUGGCUCUGGAAUGUUCA	Cleavage
tae-miR9662a-3p	Traes_6AS_A2EAE01C1.2	PF02536				GO:GO:0006355,GO:GO:0005739,GO:GO:0003690	AT5G07900.1	Mitochondrial transcription termination factor family protein	LOC_Os06g12100.1	mTERF family protein, expressed	2,05	-1.0	1	21	66	86	UUGAACAUCCCAGAGCCACCG	CCGUGGCUCUGGAAUGUUCAG	Cleavage
tae-miR9662b-3p	Traes_6AS_A2EAE01C1.2	PF02536				GO:GO:0006355,GO:GO:0005739,GO:GO:0003690	AT5G07900.1	Mitochondrial transcription termination factor family protein	LOC_Os06g12100.1	mTERF family protein, expressed	2,05	-1.0	1	21	65	85	UGAACAUCCCAGAGCCACCGG	CCCGUGGCUCUGGAAUGUUCA	Cleavage
tae-miR9662a-3p	Traes_6AS_B24138E62.1	PF02536				GO:GO:0006355,GO:GO:0005739,GO:GO:0003690	AT5G07900.1	Mitochondrial transcription termination factor family protein	LOC_Os06g12100.1	mTERF family protein, expressed	2,05	-1.0	1	21	375	395	UUGAACAUCCCAGAGCCACCG	CCGUGGCUCUGGAAUGUUCAG	Cleavage
tae-miR9662b-3p	Traes_6AS_B24138E62.1	PF02536				GO:GO:0006355,GO:GO:0005739,GO:GO:0003690	AT5G07900.1	Mitochondrial transcription termination factor family protein	LOC_Os06g12100.1	mTERF family protein, expressed	2,05	-1.0	1	21	374	394	UGAACAUCCCAGAGCCACCGG	CCCGUGGCUCUGGAAUGUUCA	Cleavage
tae-miR9662a-3p	Traes_6AS_EBADF37C7.1	PF02536				GO:GO:0006355,GO:GO:0005739,GO:GO:0003690	AT5G07900.1	Mitochondrial transcription termination factor family protein	LOC_Os06g12100.1	mTERF family protein, expressed	2,05	-1.0	1	21	681	701	UUGAACAUCCCAGAGCCACCG	CCGUGGCUCUGGGAUGCUCAG	Cleavage
tae-miR9662b-3p	Traes_6AS_EBADF37C7.1	PF02536				GO:GO:0006355,GO:GO:0005739,GO:GO:0003690	AT5G07900.1	Mitochondrial transcription termination factor family protein	LOC_Os06g12100.1	mTERF family protein, expressed	2,05	-1.0	1	21	680	700	UGAACAUCCCAGAGCCACCGG	CCCGUGGCUCUGGGAUGCUCA	Cleavage
tae-miR9662a-3p	Traes_6AS_EBADF37C7.2	PF02536				GO:GO:0006355,GO:GO:0005739,GO:GO:0003690	AT5G07900.1	Mitochondrial transcription termination factor family protein	LOC_Os06g12100.1	mTERF family protein, expressed	2,05	-1.0	1	21	378	398	UUGAACAUCCCAGAGCCACCG	CCGUGGCUCUGGGAUGCUCAG	Cleavage
tae-miR9662b-3p	Traes_6AS_EBADF37C7.2	PF02536				GO:GO:0006355,GO:GO:0005739,GO:GO:0003690	AT5G07900.1	Mitochondrial transcription termination factor family protein	LOC_Os06g12100.1	mTERF family protein, expressed	2,05	-1.0	1	21	377	397	UGAACAUCCCAGAGCCACCGG	CCCGUGGCUCUGGGAUGCUCA	Cleavage
tae-miR9662a-3p	Traes_6AS_EE73C76541.2	PF02536				GO:GO:0006355,GO:GO:0005739,GO:GO:0003690	AT5G07900.1	Mitochondrial transcription termination factor family protein	LOC_Os06g12100.1	mTERF family protein, expressed	1,00	-1.0	1	21	420	440	UUGAACAUCCCAGAGCCACCG	CCGUGGCUCUGGGAUGUUCAG	Cleavage
tae-miR9662b-3p	Traes_6AS_EE73C76541.2	PF02536				GO:GO:0006355,GO:GO:0005739,GO:GO:0003690	AT5G07900.1	Mitochondrial transcription termination factor family protein	LOC_Os06g12100.1	mTERF family protein, expressed	1,00	-1.0	1	21	419	439	UGAACAUCCCAGAGCCACCGG	CCCGUGGCUCUGGGAUGUUCA	Cleavage
tae-miR9662a-3p	Traes_6AS_EE73C76541.6	PF02536				GO:GO:0006355,GO:GO:0005739,GO:GO:0003690	AT5G07900.1	Mitochondrial transcription termination factor family protein	LOC_Os06g12100.1	mTERF family protein, expressed	1,00	-1.0	1	21	66	86	UUGAACAUCCCAGAGCCACCG	CCGUGGCUCUGGGAUGUUCAG	Cleavage
tae-miR9662b-3p	Traes_6AS_EE73C76541.6	PF02536				GO:GO:0006355,GO:GO:0005739,GO:GO:0003690	AT5G07900.1	Mitochondrial transcription termination factor family protein	LOC_Os06g12100.1	mTERF family protein, expressed	1,00	-1.0	1	21	65	85	UGAACAUCCCAGAGCCACCGG	CCCGUGGCUCUGGGAUGUUCA	Cleavage
tae-miR9662a-3p	Traes_6AS_EE8BA0A57.1	PF02536				GO:GO:0006355,GO:GO:0005739,GO:GO:0003690	AT1G21150.1	Mitochondrial transcription termination factor family protein	LOC_Os06g12100.1	mTERF family protein, expressed	2,00	-1.0	1	21	462	482	UUGAACAUCCCAGAGCCACCG	CCGUGCCUCUGGGAUGUUCAG	Cleavage
tae-miR9662b-3p	Traes_6AS_EE8BA0A57.1	PF02536				GO:GO:0006355,GO:GO:0005739,GO:GO:0003690	AT1G21150.1	Mitochondrial transcription termination factor family protein	LOC_Os06g12100.1	mTERF family protein, expressed	2,00	-1.0	1	21	461	481	UGAACAUCCCAGAGCCACCGG	CCCGUGCCUCUGGGAUGUUCA	Cleavage
tae-miR9662a-3p	Traes_6AS_EE8BA0A57.2	PF02536				GO:GO:0006355,GO:GO:0005739,GO:GO:0003690	AT5G07900.1	Mitochondrial transcription termination factor family protein	LOC_Os06g12100.1	mTERF family protein, expressed	2,00	-1.0	1	21	510	530	UUGAACAUCCCAGAGCCACCG	CCGUGCCUCUGGGAUGUUCAG	Cleavage
tae-miR9662b-3p	Traes_6AS_EE8BA0A57.2	PF02536				GO:GO:0006355,GO:GO:0005739,GO:GO:0003690	AT5G07900.1	Mitochondrial transcription termination factor family protein	LOC_Os06g12100.1	mTERF family protein, expressed	2,00	-1.0	1	21	509	529	UGAACAUCCCAGAGCCACCGG	CCCGUGCCUCUGGGAUGUUCA	Cleavage
tae-miR5049-3p	Traes_6AS_F5DBB6DBD.3	PF13855	KOG0472			GO:GO:0005515	AT1G72300.1	Leucine-rich receptor-like protein kinase family protein	LOC_Os02g05970.1	phytosulfokine receptor precursor, putative, expressed	1,05	-1.0	1	21	1927	1947	AAUAUGGAUCGGAGGGAGUAC	UUACUCCCUCCAAUCCAUAUU	Translation
tae-miR9662a-3p	Traes_6AS_FBBCFBCB0.1	PF02536	KOG1267			GO:GO:0006355,GO:GO:0005739,GO:GO:0003690	AT5G07900.1	Mitochondrial transcription termination factor family protein	LOC_Os06g12100.1	mTERF family protein, expressed	2,05	-1.0	1	21	681	701	UUGAACAUCCCAGAGCCACCG	CCGUGGCUCUGGGAUGUUUAG	Cleavage

tae-miR9662b-3p	Traes_6AS_FBBCFBCB0.1	PF02536	KOG1267			GO:GO:0006355,GO:GO:0005739,GO:GO:0003690	AT5G07900.1	Mitochondrial transcription termination factor family protein	LOC_Os06g12100.1	mTERF family protein, expressed	2,05	-1.0	1	21	680	700	UGAACAUCCCAGAGCCACCGG	CCCGUGGCUCUGGGAUGUUUA	Cleavage
tae-miR1127b-3p	Traes_6BL_028068EFA.2		KOG1877				AT5G21080.1	Uncharacterized protein	LOC_Os02g53990.1	cyclin-related protein, putative, expressed	1,05	-1.0	1	21	2801	2821	ACAAGUAUUUCUGGACGGAGG	CCUCCGUCCGGAAAUACUUGU	Cleavage
tae-miR6197-5p	Traes_6BL_67725F06B.2	PF02358		3.1.3.12		GO:GO:0005992,GO:GO:0003824	AT5G65140.2	Haloacid dehalogenase-like hydrolase (HAD) superfamily protein	LOC_Os02g51680.1	uncharacterized glycosyl hydrolase Rv2006/MT2062, putative, expressed	3,00	-1.0	1	21	278	298	UCUGUAAACAAAUGUAGGACG	GAACCUCCAUUUGUUUACAGG	Cleavage
tae-miR1127b-3p	Traes_6BL_731ED1C4C.1	PF00646				GO:GO:0005515	AT5G56820.1	F-box/RNI-like/FBD-like domains-containing protein	LOC_Os02g57910.1	OsFBX73 - F-box domain containing protein, expressed	1,05	-1.0	1	21	1020	1040	ACAAGUAUUUCUGGACGGAGG	UCUCCGUCCAAAAAUACUUGU	Translation
tae-miR1130b-3p	Traes_6BL_7BD68BAC2.3	PF00366				GO:GO:0006412,GO:GO:0005840,GO:GO:0005622,GO:GO:0003735	AT5G23740.1	ribosomal protein S11-beta	LOC_Os04g52361.1	ribosomal protein S17, putative, expressed	1,05	-1.0	1	21	215	235	UCUUAUAUUAUGGGACGGAGG	CCUCCGUCCCAUAAUGUAAGA	Cleavage
tae-miR1122a	Traes_6BL_7E9C131F4.3	PF00249	KOG0724				AT5G52660.1	Homeodomain-like superfamily protein	LOC_Os02g45670.1	myb-like DNA-binding domain containing protein, expressed	0,00	-1.0	1	20	761	780	UAGAUACAUCCGUAUCUAGA	UCUAGAUACGGAUGUAUCUA	Cleavage
tae-miR1130b-3p	Traes_6BL_887005D55.2										1,05	-1.0	1	21	79	99	UCUUAUAUUAUGGGACGGAGG	CCUCCGUCCCAUAAUAUAAUA	Cleavage
tae-miR1118	Traes_6BL_E14604962.2	PF00076				GO:GO:0003676	AT5G51120.2	polyadenylate-binding protein 1	LOC_Os02g52140.2	RNA recognition motif containing protein, putative, expressed	2,00	-1.0	1	23	35	57	CACUACAUUAUGGAAUGGAGGGA	UCCCUCUGUUCCAUAAUGUAGUG	Cleavage
tae-miR1118	Traes_6BL_E14604962.3	PF00076				GO:GO:0003676	AT5G51120.2	polyadenylate-binding protein 1	LOC_Os02g52140.2	RNA recognition motif containing protein, putative, expressed	2,00	-1.0	1	23	35	57	CACUACAUUAUGGAAUGGAGGGA	UCCCUCUGUUCCAUAAUGUAGUG	Cleavage
tae-miR1127b-3p	Traes_6BS_1AFDF35E5.3										2,05	-1.0	1	21	171	191	ACAAGUAUUUCUGGACGGAGG	CUUUCGUCCGGAAAUACUUGU	Cleavage
tae-miR9662a-3p	Traes_6BS_28F0D2955.1	PF02536				GO:GO:0006355,GO:GO:0005739,GO:GO:0003690	AT5G64950.1	Mitochondrial transcription termination factor family protein	LOC_Os06g12100.1	mTERF family protein, expressed	2,05	-1.0	1	21	396	416	UUGAACAUCCCAGAGCCACCG	CUGUGGCUCUCGGAUGUUCAG	Translation
tae-miR9662b-3p	Traes_6BS_28F0D2955.1	PF02536				GO:GO:0006355,GO:GO:0005739,GO:GO:0003690	AT5G64950.1	Mitochondrial transcription termination factor family protein	LOC_Os06g12100.1	mTERF family protein, expressed	2,05	-1.0	1	21	395	415	UGAACAUCCCAGAGCCACCGG	CCUGUGGCUCUCGGAUGUUCA	Translation
tae-miR9662a-3p	Traes_6BS_2AC6A2287.1	PF02536				GO:GO:0006355,GO:GO:0005739,GO:GO:0003690	AT5G07900.1	Mitochondrial transcription termination factor family protein	LOC_Os06g12100.1	mTERF family protein, expressed	2,05	-1.0	1	21	66	86	UUGAACAUCCCAGAGCCACCG	CCGUGGCUCUGGGAUGCUCAG	Cleavage
tae-miR9662b-3p	Traes_6BS_2AC6A2287.1	PF02536				GO:GO:0006355,GO:GO:0005739,GO:GO:0003690	AT5G07900.1	Mitochondrial transcription termination factor family protein	LOC_Os06g12100.1	mTERF family protein, expressed	2,05	-1.0	1	21	65	85	UGAACAUCCCAGAGCCACCGG	CCCGUGGCUCUGGGAUGCUCA	Cleavage
tae-miR9662a-3p	Traes_6BS_2F108C5BB.1	PF02536				GO:GO:0006355,GO:GO:0005739,GO:GO:0003690	AT5G07900.1	Mitochondrial transcription termination factor family protein	LOC_Os06g12100.1	mTERF family protein, expressed	2,00	-1.0	1	21	435	455	UUGAACAUCCCAGAGCCACCG	UCGUGGCGCUGGGAUGUUCAG	Cleavage
tae-miR9662b-3p	Traes_6BS_2F108C5BB.1	PF02536				GO:GO:0006355,GO:GO:0005739,GO:GO:0003690	AT5G07900.1	Mitochondrial transcription termination factor family protein	LOC_Os06g12100.1	mTERF family protein, expressed	2,05	-1.0	1	21	434	454	UGAACAUCCCAGAGCCACCGG	CUCGUGGCGCUGGGAUGUUCA	Cleavage
tae-miR408	Traes_6BS_348308070.13										2,05	-1.0	1	21	2	22	CUGCACUGCCUCUUCCCUGGC	CCCAGGGCCGAGGCAGUGCAG	Cleavage
tae-miR408	Traes_6BS_348308070.15										2,05	-1.0	1	21	11	31	CUGCACUGCCUCUUCCCUGGC	CCCAGGGCCGAGGCAGUGCAG	Cleavage
tae-miR408	Traes_6BS_348308070.17										2,05	-1.0	1	21	14	34	CUGCACUGCCUCUUCCCUGGC	CCCAGGGCCGAGGCAGUGCAG	Cleavage
tae-miR408	Traes_6BS_348308070.19										2,05	-1.0	1	21	11	31	CUGCACUGCCUCUUCCCUGGC	CCCAGGGCCGAGGCAGUGCAG	Cleavage
tae-miR408	Traes_6BS_348308070.2										2,05	-1.0	1	21	14	34	CUGCACUGCCUCUUCCCUGGC	CCCAGGGCCGAGGCAGUGCAG	Cleavage
tae-miR408	Traes_6BS_348308070.3										2,05	-1.0	1	21	17	37	CUGCACUGCCUCUUCCCUGGC	CCCAGGGCCGAGGCAGUGCAG	Cleavage
tae-miR408	Traes_6BS_348308070.5										2,05	-1.0	1	21	17	37	CUGCACUGCCUCUUCCCUGGC	CCCAGGGCCGAGGCAGUGCAG	Cleavage
tae-miR408	Traes_6BS_348308070.6										2,05	-1.0	1	21	14	34	CUGCACUGCCUCUUCCCUGGC	CCCAGGGCCGAGGCAGUGCAG	Cleavage
tae-miR408	Traes_6BS_348308070.8										2,05	-1.0	1	21	17	37	CUGCACUGCCUCUUCCCUGGC	CCCAGGGCCGAGGCAGUGCAG	Cleavage
tae-miR408	Traes_6BS_348308070.9										2,05	-1.0	1	21	17	37	CUGCACUGCCUCUUCCCUGGC	CCCAGGGCCGAGGCAGUGCAG	Cleavage
tae-miR9662a-3p	Traes_6BS_3A00751B5.1	PF02536				GO:GO:0006355,GO:GO:0005739,GO:GO:0003690	AT5G07900.1	Mitochondrial transcription termination factor family protein	LOC_Os06g12100.1	mTERF family protein, expressed	2,05	-1.0	1	21	69	89	UUGAACAUCCCAGAGCCACCG	CCGUGGCUCUGGGAUGUUUAG	Cleavage
tae-miR9662b-3p	Traes_6BS_3A00751B5.1	PF02536				GO:GO:0006355,GO:GO:0005739,GO:GO:0003690	AT5G07900.1	Mitochondrial transcription termination factor family protein	LOC_Os06g12100.1	mTERF family protein, expressed	2,05	-1.0	1	21	68	88	UGAACAUCCCAGAGCCACCGG	CCCGUGGCUCUGGGAUGUUUA	Cleavage
tae-miR9662a-3p	Traes_6BS_3A00751B5.2	PF02536				GO:GO:0006355,GO:GO:0005739,GO:GO:0003690	AT5G07900.1	Mitochondrial transcription termination factor family protein	LOC_Os06g12100.1	mTERF family protein, expressed	2,05	-1.0	1	21	69	89	UUGAACAUCCCAGAGCCACCG	CCGUGGCUCUGGGAUGUUUAG	Cleavage
tae-miR9662b-3p	Traes_6BS_3A00751B5.2	PF02536				GO:GO:0006355,GO:GO:0005739,GO:GO:0003690	AT5G07900.1	Mitochondrial transcription termination factor family protein	LOC_Os06g12100.1	mTERF family protein, expressed	2,05	-1.0	1	21	68	88	UGAACAUCCCAGAGCCACCGG	CCCGUGGCUCUGGGAUGUUUA	Cleavage
tae-miR9662a-3p	Traes_6BS_46641470F.1	PF02536				GO:GO:0006355,GO:GO:0005739,GO:GO:0003690	AT1G21150.1	Mitochondrial transcription termination factor family protein	LOC_Os06g12100.1	mTERF family protein, expressed	2,05	-1.0	1	21	33	53	UUGAACAUCCCAGAGCCACCG	CCGUGGCUCUGGGAUGUUUAG	Cleavage
tae-miR9662b-3p	Traes_6BS_46641470F.1	PF02536				GO:GO:0006355,GO:GO:0005739,GO:GO:0003690	AT1G21150.1	Mitochondrial transcription termination factor family protein	LOC_Os06g12100.1	mTERF family protein, expressed	2,05	-1.0	1	21	32	52	UGAACAUCCCAGAGCCACCGG	CCCGUGGCUCUGGGAUGUUUA	Cleavage
tae-miR9662a-3p	Traes_6BS_4E6E57FEB.1	PF02536				GO:GO:0006355,GO:GO:0005739,GO:GO:0003690	AT5G07900.1	Mitochondrial transcription termination factor family protein	LOC_Os06g12100.1	mTERF family protein, expressed	3,00	-1.0	1	21	65	86	UUGAACAUCCCAGAGCCAC-CG	CGCGUGGCUCUGGGAUGUUCAG	Cleavage
tae-miR9662b-3p	Traes_6BS_4E6E57FEB.1	PF02536				GO:GO:0006355,GO:GO:0005739,GO:GO:0003690	AT5G07900.1	Mitochondrial transcription termination factor family protein	LOC_Os06g12100.1	mTERF family protein, expressed	2,00	-1.0	1	21	64	85	UGAACAUCCCAGAGCCAC-CGG	CCGCGUGGCUCUGGGAUGUUCA	Cleavage
tae-miR9662a-3p	Traes_6BS_4E6E57FEB.2	PF02536				GO:GO:0006355,GO:GO:0005739,GO:GO:0003690	AT5G07900.1	Mitochondrial transcription termination factor family protein	LOC_Os06g12100.1	mTERF family protein, expressed	3,00	-1.0	1	21	65	86	UUGAACAUCCCAGAGCCAC-CG	CGCGUGGCUCUGGGAUGUUCAG	Cleavage
tae-miR9662b-3p	Traes_6BS_4E6E57FEB.2	PF02536				GO:GO:0006355,GO:GO:0005739,GO:GO:0003690	AT5G07900.1	Mitochondrial transcription termination factor family protein	LOC_Os06g12100.1	mTERF family protein, expressed	2,00	-1.0	1	21	64	85	UGAACAUCCCAGAGCCAC-CGG	CCGCGUGGCUCUGGGAUGUUCA	Cleavage
tae-miR9662a-3p	Traes_6BS_4E6E57FEB.3	PF02536				GO:GO:0006355,GO:GO:0005739,GO:GO:0003690	AT5G07900.1	Mitochondrial transcription termination factor family protein	LOC_Os06g12100.1	mTERF family protein, expressed	3,00	-1.0	1	21	65	86	UUGAACAUCCCAGAGCCAC-CG	CGCGUGGCUCUGGGAUGUUCAG	Cleavage
tae-miR9662b-3p	Traes_6BS_4E6E57FEB.3	PF02536				GO:GO:0006355,GO:GO:0005739,GO:GO:0003690	AT5G07900.1	Mitochondrial transcription termination factor family protein	LOC_Os06g12100.1	mTERF family protein, expressed	2,00	-1.0	1	21	64	85	UGAACAUCCCAGAGCCAC-CGG	CCGCGUGGCUCUGGGAUGUUCA	Cleavage
tae-miR9662b-3p	Traes_6BS_508C2BEA2.1	PF02536				GO:GO:0006355,GO:GO:0005739,GO:GO:0003690	AT1G21150.1	Mitochondrial transcription termination factor family protein	LOC_Os06g12100.1	mTERF family protein, expressed	3,00	-1.0	1	21	32	52	UGAACAUCCCAGAGCCACCGG	CCUGUGGUUCUGGGAUGUUCG	Cleavage
tae-miR156	Traes_6BS_542961EA4.1								LOC_Os06g45310.1	OsSPL11 - SBP-box gene family member, expressed	1,00	-1.0	1	21	283	303	UGACAGAAGAGAGUGAGCACA	CGUGCUCUCUCUCUUCUGUCA	Cleavage
tae-miR156	Traes_6BS_542961EA4.2								LOC_Os06g45310.1	OsSPL11 - SBP-box gene family member, expressed	1,00	-1.0	1	21	349	369	UGACAGAAGAGAGUGAGCACA	CGUGCUCUCUCUCUUCUGUCA	Cleavage
tae-miR1122b-3p	Traes_6BS_7965FEB75.1	PF00300	KOG0235	3.1.3.3	K15634		AT3G50520.1	Phosphoglycerate mutase family protein	LOC_Os08g37140.1	phosphoglycerate mutase, putative, expressed	2,05	-1.0	1	21	87	107	AGACUUAUAUGUAGGAACGGA	UCCGUUCGUAUAUAUAAGUCU	Translation
tae-miR9662a-3p	Traes_6BS_7F1846171.1	PF02536				GO:GO:0006355,GO:GO:0005739,GO:GO:0003690	AT5G07900.1	Mitochondrial transcription termination factor family protein	LOC_Os06g12100.1	mTERF family protein, expressed	2,05	-1.0	1	21	33	53	UUGAACAUCCCAGAGCCACCG	CUGUGGCUCUCGGAUGUUCAG	Translation
tae-miR9662b-3p	Traes_6BS_7F1846171.1	PF02536				GO:GO:0006355,GO:GO:0005739,GO:GO:0003690	AT5G07900.1	Mitochondrial transcription termination factor family protein	LOC_Os06g12100.1	mTERF family protein, expressed	2,05	-1.0	1	21	32	52	UGAACAUCCCAGAGCCACCGG	CCUGUGGCUCUCGGAUGUUCA	Translation
tae-miR9662a-3p	Traes_6BS_82F5BF11F.1	PF02536				GO:GO:0006355,GO:GO:0005739,GO:GO:0003690	AT1G79220.1	Mitochondrial transcription termination factor family protein	LOC_Os06g12100.1	mTERF family protein, expressed	2,00	-1.0	1	21	267	287	UUGAACAUCCCAGAGCCACCG	CCGUGCCUCUGGGAUGUUCAG	Cleavage
tae-miR9662b-3p	Traes_6BS_82F5BF11F.1	PF02536				GO:GO:0006355,GO:GO:0005739,GO:GO:0003690	AT1G79220.1	Mitochondrial transcription termination factor family protein	LOC_Os06g12100.1	mTERF family protein, expressed	2,00	-1.0	1	21	266	286	UGAACAUCCCAGAGCCACCGG	CCCGUGCCUCUGGGAUGUUCA	Cleavage
tae-miR9662a-3p	Traes_6BS_843505CB3.1	PF02536				GO:GO:0006355,GO:GO:0005739,GO:GO:0003690	AT1G21150.1	Mitochondrial transcription termination factor family protein	LOC_Os06g12100.1	mTERF family protein, expressed	2,05	-1.0	1	21	69	89	UUGAACAUCCCAGAGCCACCG	CCGUGGCUCUGGAAUGUUCAG	Cleavage
tae-miR9662b-3p	Traes_6BS_843505CB3.1	PF02536				GO:GO:0006355,GO:GO:0005739,GO:GO:0003690	AT1G21150.1	Mitochondrial transcription termination factor family protein	LOC_Os06g12100.1	mTERF family protein, expressed	2,05	-1.0	1	21	68	88	UGAACAUCCCAGAGCCACCGG	CCCGUGGCUCUGGAAUGUUCA	Cleavage
tae-miR9662a-3p	Traes_6BS_843505CB3.2	PF02536				GO:GO:0006355,GO:GO:0005739,GO:GO:0003690	AT1G21150.1	Mitochondrial transcription termination factor family protein	LOC_Os06g12100.1	mTERF family protein, expressed	2,05	-1.0	1	21	69	89	UUGAACAUCCCAGAGCCACCG	CCGUGGCUCUGGAAUGUUCAG	Cleavage
tae-miR9662b-3p	Traes_6BS_843505CB3.2	PF02536				GO:GO:0006355,GO:GO:0005739,GO:GO:0003690	AT1G21150.1	Mitochondrial transcription termination factor family protein	LOC_Os06g12100.1	mTERF family protein, expressed	2,05	-1.0	1	21	68	88	UGAACAUCCCAGAGCCACCGG	CCCGUGGCUCUGGAAUGUUCA	Cleavage
tae-miR9662a-3p	Traes_6BS_843505CB3.3	PF02536				GO:GO:0006355,GO:GO:0005739,GO:GO:0003690	AT1G21150.1	Mitochondrial transcription termination factor family protein	LOC_Os06g12100.1	mTERF family protein, expressed	2,05	-1.0	1	21	69	89	UUGAACAUCCCAGAGCCACCG	CCGUGGCUCUGGAAUGUUCAG	Cleavage
tae-miR9662b-3p	Traes_6BS_843505CB3.3	PF02536				GO:GO:0006355,GO:GO:0005739,GO:GO:0003690	AT1G21150.1	Mitochondrial transcription termination factor family protein	LOC_Os06g12100.1	mTERF family protein, expressed	2,05	-1.0	1	21	68	88	UGAACAUCCCAGAGCCACCGG	CCCGUGGCUCUGGAAUGUUCA	Cleavage
tae-miR9662a-3p	Traes_6BS_843505CB3.4	PF02536				GO:GO:0006355,GO:GO:0005739,GO:GO:0003690	AT1G21150.1	Mitochondrial transcription termination factor family protein	LOC_Os06g12100.1	mTERF family protein, expressed	2,05	-1.0	1	21	69	89	UUGAACAUCCCAGAGCCACCG	CCGUGGCUCUGGAAUGUUCAG	Cleavage
tae-miR9662b-3p	Traes_6BS_843505CB3.4	PF02536				GO:GO:0006355,GO:GO:0005739,GO:GO:0003690	AT1G21150.1	Mitochondrial transcription termination factor family protein	LOC_Os06g12100.1	mTERF family protein, expressed	2,05	-1.0	1	21	68	88	UGAACAUCCCAGAGCCACCGG	CCCGUGGCUCUGGAAUGUUCA	Cleavage
tae-miR9662a-3p	Traes_6BS_843505CB3.5	PF02536				GO:GO:0006355,GO:GO:0005739,GO:GO:0003690	AT1G21150.1	Mitochondrial transcription termination factor family protein	LOC_Os06g12100.1	mTERF family protein, expressed	2,05	-1.0	1	21	69	89	UUGAACAUCCCAGAGCCACCG	CCGUGGCUCUGGAAUGUUCAG	Cleavage
tae-miR9662b-3p	Traes_6BS_843505CB3.5	PF02536				GO:GO:0006355,GO:GO:0005739,GO:GO:0003690	AT1G21150.1	Mitochondrial transcription termination factor family protein	LOC_Os06g12100.1	mTERF family protein, expressed	2,05	-1.0	1	21	68	88	UGAACAUCCCAGAGCCACCGG	CCCGUGGCUCUGGAAUGUUCA	Cleavage
tae-miR9662a-3p	Traes_6BS_843505CB3.6	PF02536				GO:GO:0006355,GO:GO:0005739,GO:GO:0003690	AT1G21150.1	Mitochondrial transcription termination factor family protein	LOC_Os06g12100.1	mTERF family protein, expressed	2,05	-1.0	1	21	69	89	UUGAACAUCCCAGAGCCACCG	CCGUGGCUCUGGAAUGUUCAG	Cleavage
tae-miR9662b-3p	Traes_6BS_843505CB3.6	PF02536				GO:GO:0006355,GO:GO:0005739,GO:GO:0003690	AT1G21150.1	Mitochondrial transcription termination factor family protein	LOC_Os06g12100.1	mTERF family protein, expressed	2,05	-1.0	1	21	68	88	UGAACAUCCCAGAGCCACCGG	CCCGUGGCUCUGGAAUGUUCA	Cleavage
tae-miR9662a-3p	Traes_6BS_843505CB3.7	PF02536				GO:GO:0006355,GO:GO:0005739,GO:GO:0003690	AT1G21150.1	Mitochondrial transcription termination factor family protein	LOC_Os06g12100.1	mTERF family protein, expressed	2,05	-1.0	1	21	69	89	UUGAACAUCCCAGAGCCACCG	CCGUGGCUCUGGAAUGUUCAG	Cleavage
tae-miR9662b-3p	Traes_6BS_843505CB3.7	PF02536				GO:GO:0006355,GO:GO:0005739,GO:GO:0003690	AT1G21150.1	Mitochondrial transcription termination factor family protein	LOC_Os06g12100.1	mTERF family protein, expressed	2,05	-1.0	1	21	68	88	UGAACAUCCCAGAGCCACCGG	CCCGUGGCUCUGGAAUGUUCA	Cleavage
tae-miR1127b-3p	Traes_6BS_94CE86A01.2	PF00069	KOG0984	2.7.11.1		GO:GO:0006468,GO:GO:0005524,GO:GO:0004672	AT4G23270.1	cysteine-rich RLK (RECEPTOR-like protein kinase) 19	LOC_Os10g12620.1	protein kinase domain containing protein, expressed	2,05	-1.0	1	21	763	783	ACAAGUAUUUCUGGACGGAGG	AAUCCGUUCAGAAUUACUUGU	Cleavage
tae-miR1127a	Traes_6BS_94CE86A01.2	PF00069	KOG0984	2.7.11.1		GO:GO:0006468,GO:GO:0005524,GO:GO:0004672	AT4G23270.1	cysteine-rich RLK (RECEPTOR-like protein kinase) 19	LOC_Os10g12620.1	protein kinase domain containing protein, expressed	1,05	-1.0	1	19	842	860	UCCUUCCGUUCGGAAUUAC	GUAAUUCCGAACGGAGGGA	Cleavage
tae-miR9662a-3p	Traes_6BS_97770F04E.1	PF02536				GO:GO:0006355,GO:GO:0005739,GO:GO:0003690	AT5G07900.1	Mitochondrial transcription termination factor family protein	LOC_Os06g12100.1	mTERF family protein, expressed	2,05	-1.0	1	21	33	53	UUGAACAUCCCAGAGCCACCG	UCGUGGCUCUGGAAUGUUCAG	Cleavage
tae-miR9662b-3p	Traes_6BS_97770F04E.1	PF02536				GO:GO:0006355,GO:GO:0005739,GO:GO:0003690	AT5G07900.1	Mitochondrial transcription termination factor family protein	LOC_Os06g12100.1	mTERF family protein, expressed	2,05	-1.0	1	21	32	52	UGAACAUCCCAGAGCCACCGG	CUCGUGGCUCUGGAAUGUUCA	Cleavage
tae-miR9662a-3p	Traes_6BS_97770F04E.3	PF02536				GO:GO:0006355,GO:GO:0005739,GO:GO:0003690	AT5G07900.1	Mitochondrial transcription termination factor family protein	LOC_Os06g12100.1	mTERF family protein, expressed	2,05	-1.0	1	21	33	53	UUGAACAUCCCAGAGCCACCG	UCGUGGCUCUGGAAUGUUCAG	Cleavage
tae-miR9662b-3p	Traes_6BS_97770F04E.3	PF02536				GO:GO:0006355,GO:GO:0005739,GO:GO:0003690	AT5G07900.1	Mitochondrial transcription termination factor family protein	LOC_Os06g12100.1	mTERF family protein, expressed	2,05	-1.0	1	21	32	52	UGAACAUCCCAGAGCCACCGG	CUCGUGGCUCUGGAAUGUUCA	Cleavage
tae-miR9662a-3p	Traes_6BS_97770F04E.4	PF02536				GO:GO:0006355,GO:GO:0005739,GO:GO:0003690	AT5G07900.1	Mitochondrial transcription termination factor family protein	LOC_Os06g12100.1	mTERF family protein, expressed	2,05	-1.0	1	21	33	53	UUGAACAUCCCAGAGCCACCG	UCGUGGCUCUGGAAUGUUCAG	Cleavage
tae-miR9662b-3p	Traes_6BS_97770F04E.4	PF02536				GO:GO:0006355,GO:GO:0005739,GO:GO:0003690	AT5G07900.1	Mitochondrial transcription termination factor family protein	LOC_Os06g12100.1	mTERF family protein, expressed	2,05	-1.0	1	21	32	52	UGAACAUCCCAGAGCCACCGG	CUCGUGGCUCUGGAAUGUUCA	Cleavage
tae-miR9662a-3p	Traes_6BS_97770F04E.5	PF02536				GO:GO:0006355,GO:GO:0005739,GO:GO:0003690	AT5G07900.1	Mitochondrial transcription termination factor family protein	LOC_Os06g12100.1	mTERF family protein, expressed	2,05	-1.0	1	21	33	53	UUGAACAUCCCAGAGCCACCG	UCGUGGCUCUGGAAUGUUCAG	Cleavage
tae-miR9662b-3p	Traes_6BS_97770F04E.5	PF02536				GO:GO:0006355,GO:GO:0005739,GO:GO:0003690	AT5G07900.1	Mitochondrial transcription termination factor family protein	LOC_Os06g12100.1	mTERF family protein, expressed	2,05	-1.0	1	21	32	52	UGAACAUCCCAGAGCCACCGG	CUCGUGGCUCUGGAAUGUUCA	Cleavage
tae-miR9662a-3p	Traes_6BS_97770F04E.6	PF02536				GO:GO:0006355,GO:GO:0005739,GO:GO:0003690	AT5G07900.1	Mitochondrial transcription termination factor family protein	LOC_Os06g12100.1	mTERF family protein, expressed	2,05	-1.0	1	21	33	53	UUGAACAUCCCAGAGCCACCG	UCGUGGCUCUGGAAUGUUCAG	Cleavage
tae-miR9662b-3p	Traes_6BS_97770F04E.6	PF02536				GO:GO:0006355,GO:GO:0005739,GO:GO:0003690	AT5G07900.1	Mitochondrial transcription termination factor family protein	LOC_Os06g12100.1	mTERF family protein, expressed	2,05	-1.0	1	21	32	52	UGAACAUCCCAGAGCCACCGG	CUCGUGGCUCUGGAAUGUUCA	Cleavage
tae-miR1137a	Traes_6BS_A4F1E63CE.2	PF05971		2.1.1.181		GO:GO:0008168	AT2G21070.3	methyltransferases	LOC_Os02g02880.1	expressed protein	2,00	-1.0	1	20	884	903	UAGUACAAAGUUGAGUCAUC	AGUGUCUCAACUUUGUACUA	Cleavage
tae-miR1137a	Traes_6BS_A4F1E63CE.3	PF05971		2.1.1.181		GO:GO:0008168	AT2G21070.3	methyltransferases	LOC_Os02g02880.1	expressed protein	2,00	-1.0	1	20	871	890	UAGUACAAAGUUGAGUCAUC	AGUGUCUCAACUUUGUACUA	Cleavage
tae-miR1128	Traes_6BS_BF5C480A9.12										2,05	-1.0	1	21	60	80	UACUACUCCCUCCGUCCGAAA	UUUGGGACGGAGGGAGUAUUA	Cleavage
tae-miR9662a-3p	Traes_6BS_C3247654A.1	PF02536				GO:GO:0006355,GO:GO:0005739,GO:GO:0003690	AT5G07900.1	Mitochondrial transcription termination factor family protein	LOC_Os06g12100.1	mTERF family protein, expressed	2,05	-1.0	1	21	57	77	UUGAACAUCCCAGAGCCACCG	CUGUGGCUCUGCGAUGUUCAG	Translation
tae-miR9662b-3p	Traes_6BS_C3247654A.1	PF02536				GO:GO:0006355,GO:GO:0005739,GO:GO:0003690	AT5G07900.1	Mitochondrial transcription termination factor family protein	LOC_Os06g12100.1	mTERF family protein, expressed	2,05	-1.0	1	21	56	76	UGAACAUCCCAGAGCCACCGG	CCUGUGGCUCUGCGAUGUUCA	Cleavage
tae-miR9662a-3p	Traes_6BS_CBD931158.1	PF02536				GO:GO:0006355,GO:GO:0005739,GO:GO:0003690	AT1G21150.1	Mitochondrial transcription termination factor family protein	LOC_Os06g12100.1	mTERF family protein, expressed	2,00	-1.0	1	21	33	53	UUGAACAUCCCAGAGCCACCG	CCGUGCCUCUGGGAUGUUCAG	Cleavage
tae-miR9662b-3p	Traes_6BS_CBD931158.1	PF02536				GO:GO:0006355,GO:GO:0005739,GO:GO:0003690	AT1G21150.1	Mitochondrial transcription termination factor family protein	LOC_Os06g12100.1	mTERF family protein, expressed	2,00	-1.0	1	21	32	52	UGAACAUCCCAGAGCCACCGG	CCCGUGCCUCUGGGAUGUUCA	Cleavage
tae-miR9662a-3p	Traes_6BS_CBD931158.2	PF02536				GO:GO:0006355,GO:GO:0005739,GO:GO:0003690	AT1G21150.1	Mitochondrial transcription termination factor family protein	LOC_Os06g12100.1	mTERF family protein, expressed	2,00	-1.0	1	21	33	53	UUGAACAUCCCAGAGCCACCG	CCGUGCCUCUGGGAUGUUCAG	Cleavage
tae-miR9662b-3p	Traes_6BS_CBD931158.2	PF02536				GO:GO:0006355,GO:GO:0005739,GO:GO:0003690	AT1G21150.1	Mitochondrial transcription termination factor family protein	LOC_Os06g12100.1	mTERF family protein, expressed	2,00	-1.0	1	21	32	52	UGAACAUCCCAGAGCCACCGG	CCCGUGCCUCUGGGAUGUUCA	Cleavage
tae-miR9662a-3p	Traes_6BS_CBD931158.3	PF02536				GO:GO:0006355,GO:GO:0005739,GO:GO:0003690	AT1G21150.1	Mitochondrial transcription termination factor family protein	LOC_Os06g12100.1	mTERF family protein, expressed	2,00	-1.0	1	21	33	53	UUGAACAUCCCAGAGCCACCG	CCGUGCCUCUGGGAUGUUCAG	Cleavage
tae-miR9662b-3p	Traes_6BS_CBD931158.3	PF02536				GO:GO:0006355,GO:GO:0005739,GO:GO:0003690	AT1G21150.1	Mitochondrial transcription termination factor family protein	LOC_Os06g12100.1	mTERF family protein, expressed	2,00	-1.0	1	21	32	52	UGAACAUCCCAGAGCCACCGG	CCCGUGCCUCUGGGAUGUUCA	Cleavage
tae-miR9662a-3p	Traes_6BS_CBD931158.4	PF02536				GO:GO:0006355,GO:GO:0005739,GO:GO:0003690	AT1G21150.1	Mitochondrial transcription termination factor family protein	LOC_Os06g12100.1	mTERF family protein, expressed	2,00	-1.0	1	21	33	53	UUGAACAUCCCAGAGCCACCG	CCGUGCCUCUGGGAUGUUCAG	Cleavage
tae-miR9662b-3p	Traes_6BS_CBD931158.4	PF02536				GO:GO:0006355,GO:GO:0005739,GO:GO:0003690	AT1G21150.1	Mitochondrial transcription termination factor family protein	LOC_Os06g12100.1	mTERF family protein, expressed	2,00	-1.0	1	21	32	52	UGAACAUCCCAGAGCCACCGG	CCCGUGCCUCUGGGAUGUUCA	Cleavage
tae-miR9662a-3p	Traes_6BS_CBD931158.5	PF02536				GO:GO:0006355,GO:GO:0005739,GO:GO:0003690	AT1G21150.1	Mitochondrial transcription termination factor family protein	LOC_Os06g12100.1	mTERF family protein, expressed	2,00	-1.0	1	21	33	53	UUGAACAUCCCAGAGCCACCG	CCGUGCCUCUGGGAUGUUCAG	Cleavage
tae-miR9662b-3p	Traes_6BS_CBD931158.5	PF02536				GO:GO:0006355,GO:GO:0005739,GO:GO:0003690	AT1G21150.1	Mitochondrial transcription termination factor family protein	LOC_Os06g12100.1	mTERF family protein, expressed	2,00	-1.0	1	21	32	52	UGAACAUCCCAGAGCCACCGG	CCCGUGCCUCUGGGAUGUUCA	Cleavage
tae-miR9662a-3p	Traes_6BS_CBD931158.6	PF02536				GO:GO:0006355,GO:GO:0005739,GO:GO:0003690	AT1G21150.1	Mitochondrial transcription termination factor family protein	LOC_Os06g12100.1	mTERF family protein, expressed	2,00	-1.0	1	21	33	53	UUGAACAUCCCAGAGCCACCG	CCGUGCCUCUGGGAUGUUCAG	Cleavage
tae-miR9662b-3p	Traes_6BS_CBD931158.6	PF02536				GO:GO:0006355,GO:GO:0005739,GO:GO:0003690	AT1G21150.1	Mitochondrial transcription termination factor family protein	LOC_Os06g12100.1	mTERF family protein, expressed	2,00	-1.0	1	21	32	52	UGAACAUCCCAGAGCCACCGG	CCCGUGCCUCUGGGAUGUUCA	Cleavage
tae-miR156	Traes_6BS_F50663DF7.1	PF03110				GO:GO:0005634,GO:GO:0003677	AT5G43270.1	squamosa promoter binding protein-like 2	LOC_Os02g04680.2	OsSPL3 - SBP-box gene family member, expressed	1,00	-1.0	1	21	1387	1407	UGACAGAAGAGAGUGAGCACA	CAUGCUCUCUCUCUUCUGUCA	Cleavage
tae-miR9662a-3p	Traes_6BS_F8176B2AA.1	PF02536				GO:GO:0006355,GO:GO:0005739,GO:GO:0003690	AT5G64950.1	Mitochondrial transcription termination factor family protein	LOC_Os06g12100.1	mTERF family protein, expressed	2,05	-1.0	1	21	204	224	UUGAACAUCCCAGAGCCACCG	CCGUGGCUCUGCGAUGUUCAG	Translation
tae-miR9662b-3p	Traes_6BS_F8176B2AA.1	PF02536				GO:GO:0006355,GO:GO:0005739,GO:GO:0003690	AT5G64950.1	Mitochondrial transcription termination factor family protein	LOC_Os06g12100.1	mTERF family protein, expressed	2,05	-1.0	1	21	203	223	UGAACAUCCCAGAGCCACCGG	CCCGUGGCUCUGCGAUGUUCA	Cleavage
tae-miR9662a-3p	Traes_6BS_F8176B2AA.2	PF02536				GO:GO:0006355,GO:GO:0005739,GO:GO:0003690	AT5G64950.1	Mitochondrial transcription termination factor family protein	LOC_Os06g12100.1	mTERF family protein, expressed	2,05	-1.0	1	21	204	224	UUGAACAUCCCAGAGCCACCG	CCGUGGCUCUGCGAUGUUCAG	Translation
tae-miR9662b-3p	Traes_6BS_F8176B2AA.2	PF02536				GO:GO:0006355,GO:GO:0005739,GO:GO:0003690	AT5G64950.1	Mitochondrial transcription termination factor family protein	LOC_Os06g12100.1	mTERF family protein, expressed	2,05	-1.0	1	21	203	223	UGAACAUCCCAGAGCCACCGG	CCCGUGGCUCUGCGAUGUUCA	Cleavage
tae-miR9662a-3p	Traes_6BS_F8176B2AA.3	PF02536				GO:GO:0006355,GO:GO:0005739,GO:GO:0003690	AT5G64950.1	Mitochondrial transcription termination factor family protein	LOC_Os06g12100.1	mTERF family protein, expressed	2,05	-1.0	1	21	198	218	UUGAACAUCCCAGAGCCACCG	CCGUGGCUCUGCGAUGUUCAG	Translation
tae-miR9662b-3p	Traes_6BS_F8176B2AA.3	PF02536				GO:GO:0006355,GO:GO:0005739,GO:GO:0003690	AT5G64950.1	Mitochondrial transcription termination factor family protein	LOC_Os06g12100.1	mTERF family protein, expressed	2,05	-1.0	1	21	197	217	UGAACAUCCCAGAGCCACCGG	CCCGUGGCUCUGCGAUGUUCA	Cleavage
tae-miR5049-3p	Traes_6DL_1E6A51BD3.10	PF00063		3.6.4.1		GO:GO:0016459,GO:GO:0005524,GO:GO:0003774	AT1G04600.1	myosin XI A	LOC_Os03g64290.1	myosin, putative, expressed	1,05	-1.0	1	21	1321	1341	AAUAUGGAUCGGAGGGAGUAC	AUACUCCCUCCGAUCCAUAAU	Cleavage
tae-miR1122b-3p	Traes_6DL_244C908B5.14	PF12171					AT2G02070.1	indeterminate(ID)-domain 5	LOC_Os04g47860.4	ZOS4-11 - C2H2 zinc finger protein, expressed	1,05	-1.0	1	21	584	604	AGACUUAUAUGUAGGAACGGA	UCCGUUCCUAAAUAUAAGUCU	Translation
tae-miR5384-3p	Traes_6DL_5282E0007.1	PF14541	KOG1339	3.4.23.12		GO:GO:0006508,GO:GO:0004190	AT5G10770.1	Eukaryotic aspartyl protease family protein	LOC_Os02g48870.1	aspartic proteinase nepenthesin-2 precursor, putative, expressed	2,05	-1.0	1	21	1178	1198	UGAGCGCGCCGCCGUCGAAUG	UGUUCGACGGCGGCGCGGUCG	Cleavage
tae-miR5384-3p	Traes_6DL_5282E0007.2	PF14541,PF14543	KOG1339	3.4.23.12		GO:GO:0006508,GO:GO:0004190	AT5G10770.1	Eukaryotic aspartyl protease family protein	LOC_Os02g48870.1	aspartic proteinase nepenthesin-2 precursor, putative, expressed	2,05	-1.0	1	21	1016	1036	UGAGCGCGCCGCCGUCGAAUG	UGUUCGACGGCGGCGCGGUCG	Cleavage
tae-miR1122b-3p	Traes_6DL_84E14AD8D.4	PF00067				GO:GO:0055114,GO:GO:0020037,GO:GO:0016705,GO:GO:0005506	AT1G31800.1	cytochrome P450, family 97, subfamily A, polypeptide 3	LOC_Os02g57290.4	cytochrome P450, putative, expressed	1,05	-1.0	1	21	1030	1050	AGACUUAUAUGUAGGAACGGA	UCCGUUCCUAAAUAUAAGUCU	Translation
tae-miR1122a	Traes_6DL_84E14AD8D.4	PF00067				GO:GO:0055114,GO:GO:0020037,GO:GO:0016705,GO:GO:0005506	AT1G31800.1	cytochrome P450, family 97, subfamily A, polypeptide 3	LOC_Os02g57290.4	cytochrome P450, putative, expressed	2,05	-1.0	1	20	1071	1090	UAGAUACAUCCGUAUCUAGA	ACUACAUACGGAUGUAUAUA	Cleavage
tae-miR1118	Traes_6DL_C9216369C.2	PF00891				GO:GO:0008171	AT5G54160.1	O-methyltransferase 1	LOC_Os11g20090.1	O-methyltransferase, putative, expressed	2,05	-1.0	1	23	421	443	CACUACAUUAUGGAAUGGAGGGA	UUGCUCCGUUUCAUAAUGUAGUG	Cleavage
tae-miR444a	Traes_6DL_D1C1DBD34.1	PF00319,PF01486	KOG0014			GO:GO:0046983,GO:GO:0003677,GO:GO:0006355,GO:GO:0005634,GO:GO:0003700	AT3G57230.1	AGAMOUS-like 16	LOC_Os02g49840.1	OsMADS57 - MADS-box family gene with MIKCc type-box, expressed	0,00	-1.0	1	21	274	294	UUGCUGCCUCAAGCUUGCUGC	GCAGCAAGCUUGAGGCAGCAA	Cleavage
tae-miR444b	Traes_6DL_D1C1DBD34.1	PF00319,PF01486	KOG0014			GO:GO:0046983,GO:GO:0003677,GO:GO:0006355,GO:GO:0005634,GO:GO:0003700	AT3G57230.1	AGAMOUS-like 16	LOC_Os02g49840.1	OsMADS57 - MADS-box family gene with MIKCc type-box, expressed	0,00	-1.0	1	21	274	294	UUGCUGCCUCAAGCUUGCUGC	GCAGCAAGCUUGAGGCAGCAA	Cleavage
tae-miR1117	Traes_6DL_E940A7985.1	PF00854		3.6.3.43		GO:GO:0016020,GO:GO:0006810,GO:GO:0005215	AT1G62200.1	Major facilitator superfamily protein	LOC_Os04g50940.1	peptide transporter PTR2, putative, expressed	2,05	-1.0	1	24	155	178	UAGUACCGGUUCGUGGCACGAACC	GGUUCGUGGCACAAACCGGUACUA	Cleavage
tae-miR1131	Traes_6DL_E940A7985.1	PF00854		3.6.3.43		GO:GO:0016020,GO:GO:0006810,GO:GO:0005215	AT1G62200.1	Major facilitator superfamily protein	LOC_Os04g50940.1	peptide transporter PTR2, putative, expressed	1,00	-1.0	1	22	181	202	UAGUACCGGUUCGUGGCUAACC	GAUUCGCCACGAACCGGUACUA	Cleavage
tae-miR1122a	Traes_6DS_0D9BB546D.1	PF00300		3.1.3.3	K15634		AT3G50520.1	Phosphoglycerate mutase family protein	LOC_Os08g37140.1	phosphoglycerate mutase, putative, expressed	2,05	-1.0	1	20	111	130	UAGAUACAUCCGUAUCUAGA	ACUACAUACGGAUGUAUAUA	Cleavage
tae-miR9662a-3p	Traes_6DS_0E84AAF77.1	PF02536				GO:GO:0006355,GO:GO:0005739,GO:GO:0003690	AT5G07900.1	Mitochondrial transcription termination factor family protein	LOC_Os06g12100.1	mTERF family protein, expressed	2,05	-1.0	1	21	480	500	UUGAACAUCCCAGAGCCACCG	CCGUGGCUCUGGGAUGCUCAG	Cleavage
tae-miR9662b-3p	Traes_6DS_0E84AAF77.1	PF02536				GO:GO:0006355,GO:GO:0005739,GO:GO:0003690	AT5G07900.1	Mitochondrial transcription termination factor family protein	LOC_Os06g12100.1	mTERF family protein, expressed	2,05	-1.0	1	21	479	499	UGAACAUCCCAGAGCCACCGG	CCCGUGGCUCUGGGAUGCUCA	Cleavage
tae-miR9674b-5p	Traes_6DS_1BEF46735.1	PF13041					AT1G63130.1	Tetratricopeptide repeat (TPR)-like superfamily protein	LOC_Os10g35240.1	Rf1, mitochondrial precursor, putative, expressed	3,00	-1.0	1	21	537	557	AUAGCAUCAUCCAUCCUACCC	GGGCCGAAUGGAUGAUGCUAU	Cleavage
tae-miR1127b-3p	Traes_6DS_2207A3BC4.7	PF14438,PF06741	KOG2375				AT3G14010.4	CTC-interacting domain 4	LOC_Os02g27950.2	ataxin-2 related protein, putative, expressed	1,05	-1.0	1	21	1729	1749	ACAAGUAUUUCUGGACGGAGG	CCUCCGUCCGGAAAUACUUGU	Cleavage
tae-miR9662a-3p	Traes_6DS_2D4EFFF3E.1	PF02536				GO:GO:0006355,GO:GO:0005739,GO:GO:0003690	AT5G07900.1	Mitochondrial transcription termination factor family protein	LOC_Os06g12100.1	mTERF family protein, expressed	1,00	-1.0	1	21	369	389	UUGAACAUCCCAGAGCCACCG	CCGUGGCUCUGGGAUGUUCAG	Cleavage
tae-miR9662b-3p	Traes_6DS_2D4EFFF3E.1	PF02536				GO:GO:0006355,GO:GO:0005739,GO:GO:0003690	AT5G07900.1	Mitochondrial transcription termination factor family protein	LOC_Os06g12100.1	mTERF family protein, expressed	1,00	-1.0	1	21	368	388	UGAACAUCCCAGAGCCACCGG	CCCGUGGCUCUGGGAUGUUCA	Cleavage
tae-miR9662a-3p	Traes_6DS_3F194116E.1	PF02536				GO:GO:0006355,GO:GO:0005739,GO:GO:0003690	AT5G07900.1	Mitochondrial transcription termination factor family protein	LOC_Os06g12100.1	mTERF family protein, expressed	1,05	-1.0	1	21	525	545	UUGAACAUCCCAGAGCCACCG	CCGUGGCUCGGGGAUGUUCAA	Cleavage
tae-miR9662b-3p	Traes_6DS_3F194116E.1	PF02536				GO:GO:0006355,GO:GO:0005739,GO:GO:0003690	AT5G07900.1	Mitochondrial transcription termination factor family protein	LOC_Os06g12100.1	mTERF family protein, expressed	2,05	-1.0	1	21	524	544	UGAACAUCCCAGAGCCACCGG	CCCGUGGCUCGGGGAUGUUCA	Translation
tae-miR9662a-3p	Traes_6DS_3FC2ADA87.1	PF02536				GO:GO:0006355,GO:GO:0005739,GO:GO:0003690	AT5G07900.1	Mitochondrial transcription termination factor family protein	LOC_Os06g12100.1	mTERF family protein, expressed	3,00	-1.0	1	21	242	263	UUGAACAUCCCAGAGCCAC-CG	CGCGUGGCUCUGGGAUGUUCAG	Cleavage
tae-miR9662b-3p	Traes_6DS_3FC2ADA87.1	PF02536				GO:GO:0006355,GO:GO:0005739,GO:GO:0003690	AT5G07900.1	Mitochondrial transcription termination factor family protein	LOC_Os06g12100.1	mTERF family protein, expressed	2,00	-1.0	1	21	241	262	UGAACAUCCCAGAGCCAC-CGG	CCGCGUGGCUCUGGGAUGUUCA	Cleavage
tae-miR9662a-3p	Traes_6DS_3FC2ADA87.2	PF02536				GO:GO:0006355,GO:GO:0005739,GO:GO:0003690	AT1G21150.1	Mitochondrial transcription termination factor family protein	LOC_Os06g12100.1	mTERF family protein, expressed	3,00	-1.0	1	21	65	86	UUGAACAUCCCAGAGCCAC-CG	CGCGUGGCUCUGGGAUGUUCAG	Cleavage
tae-miR9662b-3p	Traes_6DS_3FC2ADA87.2	PF02536				GO:GO:0006355,GO:GO:0005739,GO:GO:0003690	AT1G21150.1	Mitochondrial transcription termination factor family protein	LOC_Os06g12100.1	mTERF family protein, expressed	2,00	-1.0	1	21	64	85	UGAACAUCCCAGAGCCAC-CGG	CCGCGUGGCUCUGGGAUGUUCA	Cleavage
tae-miR9662a-3p	Traes_6DS_422644406.1	PF02536				GO:GO:0006355,GO:GO:0005739,GO:GO:0003690	AT5G07900.1	Mitochondrial transcription termination factor family protein	LOC_Os06g12100.1	mTERF family protein, expressed	2,05	-1.0	1	21	468	488	UUGAACAUCCCAGAGCCACCG	CCGUGGCUCUGGAAUGUUCAG	Cleavage
tae-miR9662b-3p	Traes_6DS_422644406.1	PF02536				GO:GO:0006355,GO:GO:0005739,GO:GO:0003690	AT5G07900.1	Mitochondrial transcription termination factor family protein	LOC_Os06g12100.1	mTERF family protein, expressed	2,05	-1.0	1	21	467	487	UGAACAUCCCAGAGCCACCGG	CCCGUGGCUCUGGAAUGUUCA	Cleavage
tae-miR9662a-3p	Traes_6DS_422644406.2	PF02536				GO:GO:0006355,GO:GO:0005739,GO:GO:0003690	AT5G07900.1	Mitochondrial transcription termination factor family protein	LOC_Os06g12100.1	mTERF family protein, expressed	2,05	-1.0	1	21	66	86	UUGAACAUCCCAGAGCCACCG	CCGUGGCUCUGGAAUGUUCAG	Cleavage
tae-miR9662b-3p	Traes_6DS_422644406.2	PF02536				GO:GO:0006355,GO:GO:0005739,GO:GO:0003690	AT5G07900.1	Mitochondrial transcription termination factor family protein	LOC_Os06g12100.1	mTERF family protein, expressed	2,05	-1.0	1	21	65	85	UGAACAUCCCAGAGCCACCGG	CCCGUGGCUCUGGAAUGUUCA	Cleavage
tae-miR9662a-3p	Traes_6DS_422644406.3	PF02536				GO:GO:0006355,GO:GO:0005739,GO:GO:0003690	AT5G07900.1	Mitochondrial transcription termination factor family protein	LOC_Os06g12100.1	mTERF family protein, expressed	2,05	-1.0	1	21	66	86	UUGAACAUCCCAGAGCCACCG	CCGUGGCUCUGGAAUGUUCAG	Cleavage
tae-miR9662b-3p	Traes_6DS_422644406.3	PF02536				GO:GO:0006355,GO:GO:0005739,GO:GO:0003690	AT5G07900.1	Mitochondrial transcription termination factor family protein	LOC_Os06g12100.1	mTERF family protein, expressed	2,05	-1.0	1	21	65	85	UGAACAUCCCAGAGCCACCGG	CCCGUGGCUCUGGAAUGUUCA	Cleavage
tae-miR9662a-3p	Traes_6DS_422644406.4	PF02536				GO:GO:0006355,GO:GO:0005739,GO:GO:0003690	AT5G07900.1	Mitochondrial transcription termination factor family protein	LOC_Os06g12100.1	mTERF family protein, expressed	2,05	-1.0	1	21	66	86	UUGAACAUCCCAGAGCCACCG	CCGUGGCUCUGGAAUGUUCAG	Cleavage
tae-miR9662b-3p	Traes_6DS_422644406.4	PF02536				GO:GO:0006355,GO:GO:0005739,GO:GO:0003690	AT5G07900.1	Mitochondrial transcription termination factor family protein	LOC_Os06g12100.1	mTERF family protein, expressed	2,05	-1.0	1	21	65	85	UGAACAUCCCAGAGCCACCGG	CCCGUGGCUCUGGAAUGUUCA	Cleavage
tae-miR156	Traes_6DS_7B1596298.1	PF03110				GO:GO:0005634,GO:GO:0003677	AT5G43270.2	squamosa promoter binding protein-like 2	LOC_Os02g04680.2	OsSPL3 - SBP-box gene family member, expressed	1,00	-1.0	1	21	1150	1170	UGACAGAAGAGAGUGAGCACA	CAUGCUCUCUCUCUUCUGUCA	Cleavage
tae-miR156	Traes_6DS_7B1596298.2	PF03110				GO:GO:0005634,GO:GO:0003677	AT5G43270.1	squamosa promoter binding protein-like 2	LOC_Os02g04680.2	OsSPL3 - SBP-box gene family member, expressed	1,00	-1.0	1	21	1150	1170	UGACAGAAGAGAGUGAGCACA	CAUGCUCUCUCUCUUCUGUCA	Cleavage
tae-miR156	Traes_6DS_7B1596298.4								LOC_Os02g04680.1	OsSPL3 - SBP-box gene family member, expressed	1,00	-1.0	1	21	271	291	UGACAGAAGAGAGUGAGCACA	CAUGCUCUCUCUCUUCUGUCA	Cleavage
tae-miR9674b-5p	Traes_6DS_7C389C178.1	PF12854,PF13041					AT1G62670.1	rna processing factor 2	LOC_Os10g35240.2	Rf1, mitochondrial precursor, putative, expressed	3,00	-1.0	1	21	537	557	AUAGCAUCAUCCAUCCUACCC	GGGCCGAAUGGAUGAUGCUAU	Cleavage
tae-miR9674a-5p	Traes_6DS_7C389C178.1	PF12854,PF13041					AT1G62670.1	rna processing factor 2	LOC_Os10g35240.2	Rf1, mitochondrial precursor, putative, expressed	2,05	-1.0	1	21	954	974	GCAUCAUCCAUCCUACCAUUC	GAAUGGAAGGAUAGAUGAUGC	Cleavage
tae-miR9674a-5p	Traes_6DS_7C389C178.2	PF12854					AT1G62670.1	rna processing factor 2	LOC_Os10g35436.1	Rf1, mitochondrial precursor, putative, expressed	2,05	-1.0	1	21	51	71	GCAUCAUCCAUCCUACCAUUC	GAAUGGAAGGAUAGAUGAUGC	Cleavage
tae-miR9674b-5p	Traes_6DS_7C389C178.3	PF12854,PF13041					AT1G62670.1	rna processing factor 2	LOC_Os10g35240.1	Rf1, mitochondrial precursor, putative, expressed	3,00	-1.0	1	21	537	557	AUAGCAUCAUCCAUCCUACCC	GGGCCGAAUGGAUGAUGCUAU	Cleavage
tae-miR9674a-5p	Traes_6DS_7C389C178.3	PF12854,PF13041					AT1G62670.1	rna processing factor 2	LOC_Os10g35240.1	Rf1, mitochondrial precursor, putative, expressed	2,05	-1.0	1	21	954	974	GCAUCAUCCAUCCUACCAUUC	GAAUGGAAGGAUAGAUGAUGC	Cleavage
tae-miR9662a-3p	Traes_6DS_7D3BEF6A9.1	PF02536				GO:GO:0006355,GO:GO:0005739,GO:GO:0003690	AT1G21150.1	Mitochondrial transcription termination factor family protein	LOC_Os06g12100.1	mTERF family protein, expressed	2,05	-1.0	1	21	33	53	UUGAACAUCCCAGAGCCACCG	UCGUGGCUCGGGAUGUUCAG	Cleavage
tae-miR9662b-3p	Traes_6DS_7D3BEF6A9.1	PF02536				GO:GO:0006355,GO:GO:0005739,GO:GO:0003690	AT1G21150.1	Mitochondrial transcription termination factor family protein	LOC_Os06g12100.1	mTERF family protein, expressed	2,05	-1.0	1	21	32	52	UGAACAUCCCAGAGCCACCGG	CUCGUGGCUCGGGGAUGUUCA	Translation
tae-miR9662a-3p	Traes_6DS_7D3BEF6A9.2	PF02536				GO:GO:0006355,GO:GO:0005739,GO:GO:0003690	AT1G21150.1	Mitochondrial transcription termination factor family protein	LOC_Os06g12100.1	mTERF family protein, expressed	2,05	-1.0	1	21	246	266	UUGAACAUCCCAGAGCCACCG	UCGUGGCUCGGGGAUGUUCAG	Cleavage
tae-miR9662b-3p	Traes_6DS_7D3BEF6A9.2	PF02536				GO:GO:0006355,GO:GO:0005739,GO:GO:0003690	AT1G21150.1	Mitochondrial transcription termination factor family protein	LOC_Os06g12100.1	mTERF family protein, expressed	2,05	-1.0	1	21	245	265	UGAACAUCCCAGAGCCACCGG	CUCGUGGCUCGGGGAUGUUCA	Translation
tae-miR9662a-3p	Traes_6DS_7D3BEF6A9.3	PF02536				GO:GO:0006355,GO:GO:0005739,GO:GO:0003690	AT1G21150.1	Mitochondrial transcription termination factor family protein	LOC_Os06g12100.1	mTERF family protein, expressed	2,05	-1.0	1	21	246	266	UUGAACAUCCCAGAGCCACCG	UCGUGGCUCGGGGAUGUUCAG	Cleavage
tae-miR9662b-3p	Traes_6DS_7D3BEF6A9.3	PF02536				GO:GO:0006355,GO:GO:0005739,GO:GO:0003690	AT1G21150.1	Mitochondrial transcription termination factor family protein	LOC_Os06g12100.1	mTERF family protein, expressed	2,05	-1.0	1	21	245	265	UGAACAUCCCAGAGCCACCGG	CUCGUGGCUCGGGGAUGUUCA	Translation
tae-miR9662a-3p	Traes_6DS_893F318A9.1	PF02536				GO:GO:0006355,GO:GO:0005739,GO:GO:0003690	AT5G07900.1	Mitochondrial transcription termination factor family protein	LOC_Os06g12100.1	mTERF family protein, expressed	1,05	-1.0	1	21	549	569	UUGAACAUCCCAGAGCCACCG	CCGUGGCUCGGGGAUGUUCAA	Cleavage
tae-miR9662b-3p	Traes_6DS_893F318A9.1	PF02536				GO:GO:0006355,GO:GO:0005739,GO:GO:0003690	AT5G07900.1	Mitochondrial transcription termination factor family protein	LOC_Os06g12100.1	mTERF family protein, expressed	2,05	-1.0	1	21	548	568	UGAACAUCCCAGAGCCACCGG	CCCGUGGCUCGGGGAUGUUCA	Translation
tae-miR9662a-3p	Traes_6DS_893F318A9.2	PF02536				GO:GO:0006355,GO:GO:0005739,GO:GO:0003690	AT5G07900.1	Mitochondrial transcription termination factor family protein	LOC_Os06g12100.1	mTERF family protein, expressed	1,05	-1.0	1	21	132	152	UUGAACAUCCCAGAGCCACCG	CCGUGGCUCGGGGAUGUUCAA	Cleavage
tae-miR9662b-3p	Traes_6DS_893F318A9.2	PF02536				GO:GO:0006355,GO:GO:0005739,GO:GO:0003690	AT5G07900.1	Mitochondrial transcription termination factor family protein	LOC_Os06g12100.1	mTERF family protein, expressed	2,05	-1.0	1	21	131	151	UGAACAUCCCAGAGCCACCGG	CCCGUGGCUCGGGGAUGUUCA	Translation
tae-miR9662a-3p	Traes_6DS_931C28AF6.1	PF02536				GO:GO:0006355,GO:GO:0005739,GO:GO:0003690	AT1G79220.1	Mitochondrial transcription termination factor family protein	LOC_Os06g12100.1	mTERF family protein, expressed	2,05	-1.0	1	21	288	308	UUGAACAUCCCAGAGCCACCG	AUGUGGCUCUCGGAUGUUCAG	Translation
tae-miR9662a-3p	Traes_6DS_931C28AF6.2	PF02536				GO:GO:0006355,GO:GO:0005739,GO:GO:0003690	AT1G79220.1	Mitochondrial transcription termination factor family protein	LOC_Os06g12100.1	mTERF family protein, expressed	2,05	-1.0	1	21	276	296	UUGAACAUCCCAGAGCCACCG	AUGUGGCUCUCGGAUGUUCAG	Translation
tae-miR9662a-3p	Traes_6DS_931C28AF6.3	PF02536				GO:GO:0006355,GO:GO:0005739,GO:GO:0003690	AT1G79220.1	Mitochondrial transcription termination factor family protein	LOC_Os06g12100.1	mTERF family protein, expressed	2,05	-1.0	1	21	273	293	UUGAACAUCCCAGAGCCACCG	AUGUGGCUCUCGGAUGUUCAG	Translation
tae-miR9662a-3p	Traes_6DS_931C28AF6.4	PF02536				GO:GO:0006355,GO:GO:0005739,GO:GO:0003690	AT1G79220.1	Mitochondrial transcription termination factor family protein	LOC_Os06g12100.1	mTERF family protein, expressed	2,05	-1.0	1	21	273	293	UUGAACAUCCCAGAGCCACCG	AUGUGGCUCUCGGAUGUUCAG	Translation
tae-miR9662a-3p	Traes_6DS_931C28AF6.5	PF02536				GO:GO:0006355,GO:GO:0005739,GO:GO:0003690	AT1G79220.1	Mitochondrial transcription termination factor family protein	LOC_Os06g12100.1	mTERF family protein, expressed	2,05	-1.0	1	21	276	296	UUGAACAUCCCAGAGCCACCG	AUGUGGCUCUCGGAUGUUCAG	Translation
tae-miR9662b-3p	Traes_6DS_93C7DA226.1	PF02536				GO:GO:0006355,GO:GO:0005739,GO:GO:0003690	AT5G07900.1	Mitochondrial transcription termination factor family protein	LOC_Os06g12100.1	mTERF family protein, expressed	3,00	-1.0	1	21	503	523	UGAACAUCCCAGAGCCACCGG	CCCGUGCUUCUGGGAUGUUCA	Cleavage
tae-miR9662b-3p	Traes_6DS_93C7DA2261.1	PF02536				GO:GO:0006355,GO:GO:0005739,GO:GO:0003690	AT5G07900.1	Mitochondrial transcription termination factor family protein	LOC_Os06g12100.1	mTERF family protein, expressed	3,00	-1.0	1	21	503	523	UGAACAUCCCAGAGCCACCGG	CCCGUGCUUCUGGGAUGUUCA	Cleavage
tae-miR9662a-3p	Traes_6DS_A490DF33E.1	PF02536				GO:GO:0006355,GO:GO:0005739,GO:GO:0003690	AT5G07900.1	Mitochondrial transcription termination factor family protein	LOC_Os06g12100.1	mTERF family protein, expressed	2,05	-1.0	1	21	471	491	UUGAACAUCCCAGAGCCACCG	CCGUGGCUCUGGAAUGUUCAG	Cleavage
tae-miR9662b-3p	Traes_6DS_A490DF33E.1	PF02536				GO:GO:0006355,GO:GO:0005739,GO:GO:0003690	AT5G07900.1	Mitochondrial transcription termination factor family protein	LOC_Os06g12100.1	mTERF family protein, expressed	2,05	-1.0	1	21	470	490	UGAACAUCCCAGAGCCACCGG	CCCGUGGCUCUGGAAUGUUCA	Cleavage
tae-miR9662a-3p	Traes_6DS_A490DF33E.2	PF02536				GO:GO:0006355,GO:GO:0005739,GO:GO:0003690	AT1G21150.1	Mitochondrial transcription termination factor family protein	LOC_Os06g12100.1	mTERF family protein, expressed	2,05	-1.0	1	21	66	86	UUGAACAUCCCAGAGCCACCG	CCGUGGCUCUGGAAUGUUCAG	Cleavage
tae-miR9662b-3p	Traes_6DS_A490DF33E.2	PF02536				GO:GO:0006355,GO:GO:0005739,GO:GO:0003690	AT1G21150.1	Mitochondrial transcription termination factor family protein	LOC_Os06g12100.1	mTERF family protein, expressed	2,05	-1.0	1	21	65	85	UGAACAUCCCAGAGCCACCGG	CCCGUGGCUCUGGAAUGUUCA	Cleavage
tae-miR1122b-3p	Traes_6DS_C1B7F14A41.2	PF08263,PF07714,PF13855	KOG1187	2.7.11.1		GO:GO:0006468,GO:GO:0004672,GO:GO:0005515	AT1G72300.1	Leucine-rich receptor-like protein kinase family protein	LOC_Os02g05930.1	phytosulfokine receptor precursor, putative, expressed	1,05	-1.0	1	21	3044	3064	AGACUUAUAUGUAGGAACGGA	UCCGUUCCUAAAUAUAAGUCU	Translation
tae-miR156	Traes_6DS_C4D93239C.1	PF03110				GO:GO:0005634,GO:GO:0003677	AT5G43270.1	squamosa promoter binding protein-like 2	LOC_Os06g45310.1	OsSPL11 - SBP-box gene family member, expressed	1,00	-1.0	1	21	577	597	UGACAGAAGAGAGUGAGCACA	CAUGCUCUCUCUCUUCUGUCA	Cleavage
tae-miR156	Traes_6DS_C4D93239C.2								LOC_Os06g45310.1	OsSPL11 - SBP-box gene family member, expressed	1,00	-1.0	1	21	88	108	UGACAGAAGAGAGUGAGCACA	CAUGCUCUCUCUCUUCUGUCA	Cleavage
tae-miR156	Traes_6DS_C4D93239C.3								LOC_Os06g45310.1	OsSPL11 - SBP-box gene family member, expressed	1,00	-1.0	1	21	64	84	UGACAGAAGAGAGUGAGCACA	CAUGCUCUCUCUCUUCUGUCA	Cleavage
tae-miR9662a-3p	Traes_6DS_CF627A1BC.1								LOC_Os06g12100.1	mTERF family protein, expressed	2,05	-1.0	1	21	75	95	UUGAACAUCCCAGAGCCACCG	CCGUGGCUCGGGGAUGUUCAG	Cleavage
tae-miR9662b-3p	Traes_6DS_CF627A1BC.1								LOC_Os06g12100.1	mTERF family protein, expressed	2,05	-1.0	1	21	74	94	UGAACAUCCCAGAGCCACCGG	CCCGUGGCUCGGGGAUGUUCA	Translation
tae-miR9662a-3p	Traes_6DS_DAD8BEF41.1	PF02536				GO:GO:0006355,GO:GO:0005739,GO:GO:0003690	AT5G07900.1	Mitochondrial transcription termination factor family protein	LOC_Os06g12100.1	mTERF family protein, expressed	2,05	-1.0	1	21	477	497	UUGAACAUCCCAGAGCCACCG	CCGUGGCUCUGGAAUGUUCAG	Cleavage
tae-miR9662b-3p	Traes_6DS_DAD8BEF41.1	PF02536				GO:GO:0006355,GO:GO:0005739,GO:GO:0003690	AT5G07900.1	Mitochondrial transcription termination factor family protein	LOC_Os06g12100.1	mTERF family protein, expressed	2,05	-1.0	1	21	476	496	UGAACAUCCCAGAGCCACCGG	CCCGUGGCUCUGGAAUGUUCA	Cleavage
tae-miR9662a-3p	Traes_6DS_E352D66A4.1	PF02536				GO:GO:0006355,GO:GO:0005739,GO:GO:0003690	AT5G64950.1	Mitochondrial transcription termination factor family protein	LOC_Os06g12100.1	mTERF family protein, expressed	2,05	-1.0	1	21	450	470	UUGAACAUCCCAGAGCCACCG	CCGUGGCUCUGGGAUGUUUAG	Cleavage
tae-miR9662b-3p	Traes_6DS_E352D66A4.1	PF02536				GO:GO:0006355,GO:GO:0005739,GO:GO:0003690	AT5G64950.1	Mitochondrial transcription termination factor family protein	LOC_Os06g12100.1	mTERF family protein, expressed	2,05	-1.0	1	21	449	469	UGAACAUCCCAGAGCCACCGG	CCCGUGGCUCUGGGAUGUUUA	Cleavage
tae-miR9662a-3p	Traes_6DS_E95930B7D.1	PF02536				GO:GO:0006355,GO:GO:0005739,GO:GO:0003690	AT5G64950.1	Mitochondrial transcription termination factor family protein	LOC_Os06g12100.1	mTERF family protein, expressed	2,05	-1.0	1	21	450	470	UUGAACAUCCCAGAGCCACCG	CCGUGGCUCUGGGAUGUUUAG	Cleavage

tae-miR9662b-3p	Traes_6DS_E95930B7D.1	PF02536				GO:GO:0006355,GO:GO:0005739,GO:GO:0003690	AT5G64950.1	Mitochondrial transcription termination factor family protein	LOC_Os06g12100.1	mTERF family protein, expressed	2,05	-1.0	1	21	449	469	UGAACAUCCCAGAGCCACCGG	CCCGUGGCUCUGGGAUGUUUA	Cleavage
tae-miR9662a-3p	Traes_6DS_EF75D5C67.1	PF02536				GO:GO:0006355,GO:GO:0005739,GO:GO:0003690	AT5G07900.1	Mitochondrial transcription termination factor family protein	LOC_Os06g12100.1	mTERF family protein, expressed	2,00	-1.0	1	21	66	86	UUGAACAUCCCAGAGCCACCG	UCGUGGCGCUGGGAUGUUCAG	Cleavage
tae-miR9662b-3p	Traes_6DS_EF75D5C67.1	PF02536				GO:GO:0006355,GO:GO:0005739,GO:GO:0003690	AT5G07900.1	Mitochondrial transcription termination factor family protein	LOC_Os06g12100.1	mTERF family protein, expressed	2,05	-1.0	1	21	65	85	UGAACAUCCCAGAGCCACCGG	CUCGUGGCGCUGGGAUGUUCA	Cleavage
tae-miR9662a-3p	Traes_6DS_F6C7E588F.1	PF02536	KOG1267			GO:GO:0006355,GO:GO:0005739,GO:GO:0003690	AT5G07900.1	Mitochondrial transcription termination factor family protein	LOC_Os06g12100.1	mTERF family protein, expressed	2,00	-1.0	1	21	657	677	UUGAACAUCCCAGAGCCACCG	CCGUGGUUCUGGGAUGUUCAG	Cleavage
tae-miR9662b-3p	Traes_6DS_F6C7E588F.1	PF02536	KOG1267			GO:GO:0006355,GO:GO:0005739,GO:GO:0003690	AT5G07900.1	Mitochondrial transcription termination factor family protein	LOC_Os06g12100.1	mTERF family protein, expressed	2,00	-1.0	1	21	656	676	UGAACAUCCCAGAGCCACCGG	CCCGUGGUUCUGGGAUGUUCA	Cleavage
tae-miR9662a-3p	Traes_6DS_FB434BB3C.1	PF02536				GO:GO:0006355,GO:GO:0005739,GO:GO:0003690	AT5G64950.1	Mitochondrial transcription termination factor family protein	LOC_Os06g12100.1	mTERF family protein, expressed	2,05	-1.0	1	21	246	266	UUGAACAUCCCAGAGCCACCG	CCGUGGCUCUCGGAUGUUCAG	Translation
tae-miR9662b-3p	Traes_6DS_FB434BB3C.1	PF02536				GO:GO:0006355,GO:GO:0005739,GO:GO:0003690	AT5G64950.1	Mitochondrial transcription termination factor family protein	LOC_Os06g12100.1	mTERF family protein, expressed	2,05	-1.0	1	21	245	265	UGAACAUCCCAGAGCCACCGG	CCCGUGGCUCUCGGAUGUUCA	Translation
tae-miR9662a-3p	Traes_6DS_FB434BB3C.2	PF02536				GO:GO:0006355,GO:GO:0005739,GO:GO:0003690	AT5G64950.1	Mitochondrial transcription termination factor family protein	LOC_Os06g12100.1	mTERF family protein, expressed	2,05	-1.0	1	21	246	266	UUGAACAUCCCAGAGCCACCG	CCGUGGCUCUCGGAUGUUCAG	Translation
tae-miR9662b-3p	Traes_6DS_FB434BB3C.2	PF02536				GO:GO:0006355,GO:GO:0005739,GO:GO:0003690	AT5G64950.1	Mitochondrial transcription termination factor family protein	LOC_Os06g12100.1	mTERF family protein, expressed	2,05	-1.0	1	21	245	265	UGAACAUCCCAGAGCCACCGG	CCCGUGGCUCUCGGAUGUUCA	Translation
tae-miR1122a	Traes_7AL_135129885.3	PF01138	KOG1612				AT3G07750.1	3\'-5\'-exoribonuclease family protein	LOC_Os12g21500.1	exosome complex exonuclease, putative, expressed	2,05	-1.0	1	20	721	740	UAGAUACAUCCGUAUCUAGA	ACUACAUACGGAUGUAUAUA	Cleavage
tae-miR164	Traes_7AL_628A69311.1	PF02365				GO:GO:0006355,GO:GO:0003677	AT1G56010.2	NAC domain containing protein 1	LOC_Os06g46270.1	no apical meristem protein, putative, expressed	1,00	-1.0	1	21	650	670	UGGAGAAGCAGGGCACGUGCA	AGCAAGUGCCCUGCUUCUCCA	Cleavage
tae-miR1130b-3p	Traes_7AL_955F54182.1	PF02705				GO:GO:0071805,GO:GO:0016020,GO:GO:0015079	AT2G30070.1	potassium transporter 1	LOC_Os06g45940.2	potassium transporter, putative, expressed	0,00	-1.0	1	21	1141	1161	UCUUAUAUUAUGGGACGGAGG	CCUCCGUCCCAUAAUAUAAGA	Cleavage
tae-miR1122c-3p	Traes_7AL_955F54182.1	PF02705				GO:GO:0071805,GO:GO:0016020,GO:GO:0015079	AT2G30070.1	potassium transporter 1	LOC_Os06g45940.2	potassium transporter, putative, expressed	1,05	-1.0	1	21	1141	1161	UCUAAUAUUAUGGGACGGAGG	CCUCCGUCCCAUAAUAUAAGA	Cleavage
tae-miR1133	Traes_7AL_ADBB1224B.1	PF08315					AT3G05070.1		LOC_Os06g51420.1	coiled-coil domain-containing protein 12, putative, expressed	2,05	-1.0	1	22	52	73	CAUAUACUCCCUCCGUCCGAAA	UUCGGACGGAGGGAGUAUAAU	Cleavage
tae-miR164	Traes_7AL_BE3253C8F.1	PF02365				GO:GO:0006355,GO:GO:0003677	AT1G56010.2	NAC domain containing protein 1	LOC_Os06g46270.1	no apical meristem protein, putative, expressed	1,00	-1.0	1	21	653	673	UGGAGAAGCAGGGCACGUGCA	AGCAAGUGCCCUGCUUCUCCA	Cleavage
tae-miR164	Traes_7AL_BE3253C8F.2						AT1G56010.2	NAC domain containing protein 1	LOC_Os06g46270.1	no apical meristem protein, putative, expressed	1,00	-1.0	1	21	215	235	UGGAGAAGCAGGGCACGUGCA	AGCAAGUGCCCUGCUUCUCCA	Cleavage
tae-miR160	Traes_7AL_E3ADC8C38.1	PF06507				GO:GO:0009725,GO:GO:0006355,GO:GO:0005634,GO:GO:0003677	AT2G28350.1	auxin response factor 10	LOC_Os06g47150.2	auxin response factor 18, putative, expressed	0,00	-1.0	1	21	399	419	UGCCUGGCUCCCUGUAUGCCA	AGGCAUACAGGGAGCCAGGCA	Cleavage
tae-miR9666a-3p	Traes_7AL_EE3CD1CF3.10										2,00	-1.0	1	22	229	250	CGGUAGGGCUGUAUGAUGGCGA	UCCCCGUCAUACAGCCCUACCU	Cleavage
tae-miR9666a-3p	Traes_7AL_EE3CD1CF3.2										2,00	-1.0	1	22	5032	5053	CGGUAGGGCUGUAUGAUGGCGA	UCCCCGUCAUACAGCCCUACCU	Cleavage
tae-miR9666a-3p	Traes_7AL_EE3CD1CF3.3										2,00	-1.0	1	22	4948	4969	CGGUAGGGCUGUAUGAUGGCGA	UCCCCGUCAUACAGCCCUACCU	Cleavage
tae-miR9666a-3p	Traes_7AL_EE3CD1CF3.4										2,00	-1.0	1	22	4801	4822	CGGUAGGGCUGUAUGAUGGCGA	UCCCCGUCAUACAGCCCUACCU	Cleavage
tae-miR9666a-3p	Traes_7AL_EE3CD1CF3.5										2,00	-1.0	1	22	4393	4414	CGGUAGGGCUGUAUGAUGGCGA	UCCCCGUCAUACAGCCCUACCU	Cleavage
tae-miR9666a-3p	Traes_7AL_EE3CD1CF3.6										2,00	-1.0	1	22	3409	3430	CGGUAGGGCUGUAUGAUGGCGA	UCCCCGUCAUACAGCCCUACCU	Cleavage
tae-miR9666a-3p	Traes_7AL_EE3CD1CF3.8										2,00	-1.0	1	22	2941	2962	CGGUAGGGCUGUAUGAUGGCGA	UCCCCGUCAUACAGCCCUACCU	Cleavage
tae-miR1137b-5p	Traes_7AS_0F0291904.1	PF00657		3.1.1.1		GO:GO:0016788	AT5G45910.1	GDSL-like Lipase/Acylhydrolase superfamily protein	LOC_Os01g12320.1	GDSL-like lipase/acylhydrolase, putative, expressed	2,05	-1.0	1	21	844	864	UCCGUUCCAGAAUAGAUGACC	ACUCAUCUAUUCUGGGACGGU	Cleavage
tae-miR1137b-5p	Traes_7AS_0F0291904.2	PF00657		3.1.1.1		GO:GO:0016788	AT2G27360.1	GDSL-like Lipase/Acylhydrolase superfamily protein	LOC_Os01g12320.2	GDSL-like lipase/acylhydrolase, putative, expressed	2,05	-1.0	1	21	451	471	UCCGUUCCAGAAUAGAUGACC	ACUCAUCUAUUCUGGGACGGU	Cleavage
tae-miR1137b-5p	Traes_7AS_23DFA827E.1	PF00400	KOG1188			GO:GO:0005515	AT2G47790.1	Transducin/WD40 repeat-like superfamily protein	LOC_Os12g19590.3	WD domain, G-beta repeat domain containing protein, expressed	1,05	-1.0	1	21	11	31	UCCGUUCCAGAAUAGAUGACC	AGUCAUCUAUUUUGGAACGGA	Translation
tae-miR1122a	Traes_7AS_9674F6981.1	PF01031		3.6.5.5		GO:GO:0005525	AT1G10290.1	dynamin-like protein 6	LOC_Os06g13820.1	dynamin, putative, expressed	1,05	-1.0	1	20	1467	1486	UAGAUACAUCCGUAUCUAGA	UCUAGAUACAGAGUGUAUCUA	Translation
tae-miR1122a	Traes_7AS_9674F6981.2	PF01031		3.6.5.5		GO:GO:0005525	AT1G10290.1	dynamin-like protein 6	LOC_Os06g13820.1	dynamin, putative, expressed	1,05	-1.0	1	20	1473	1492	UAGAUACAUCCGUAUCUAGA	UCUAGAUACAGAGUGUAUCUA	Translation
tae-miR1122a	Traes_7AS_9674F6981.3	PF01031		3.6.5.5		GO:GO:0005525	AT1G10290.1	dynamin-like protein 6	LOC_Os06g13820.1	dynamin, putative, expressed	1,05	-1.0	1	20	1467	1486	UAGAUACAUCCGUAUCUAGA	UCUAGAUACAGAGUGUAUCUA	Translation
tae-miR1130b-3p	Traes_7AS_9819F7C48.1	PF01154,PF08540		2.3.3.10		GO:GO:0008299,GO:GO:0004421	AT4G11820.2	hydroxymethylglutaryl-CoA synthase / HMG-CoA synthase / 3-hydroxy-3-methylglutaryl coenzyme A synthase	LOC_Os08g43170.1	hydroxymethylglutaryl-CoA synthase, putative, expressed	0,00	-1.0	1	21	1268	1288	UCUUAUAUUAUGGGACGGAGG	AAUCCGUCCCAUAAUAUAAGA	Cleavage
tae-miR1122c-3p	Traes_7AS_9819F7C48.1	PF01154,PF08540		2.3.3.10		GO:GO:0008299,GO:GO:0004421	AT4G11820.2	hydroxymethylglutaryl-CoA synthase / HMG-CoA synthase / 3-hydroxy-3-methylglutaryl coenzyme A synthase	LOC_Os08g43170.1	hydroxymethylglutaryl-CoA synthase, putative, expressed	1,05	-1.0	1	21	1268	1288	UCUAAUAUUAUGGGACGGAGG	AAUCCGUCCCAUAAUAUAAGA	Cleavage
tae-miR1120c-5p	Traes_7AS_9819F7C48.1	PF01154,PF08540		2.3.3.10		GO:GO:0008299,GO:GO:0004421	AT4G11820.2	hydroxymethylglutaryl-CoA synthase / HMG-CoA synthase / 3-hydroxy-3-methylglutaryl coenzyme A synthase	LOC_Os08g43170.1	hydroxymethylglutaryl-CoA synthase, putative, expressed	1,05	-1.0	1	21	1311	1331	UAAUAUAAGAACGUUUUUGAC	GUCAAAAACGCUCUUAUAUUA	Translation
tae-miR5086	Traes_7AS_9E1AC3FE6.1										3,00	-1.0	1	21	324	344	ACAUUGGUGGAAGGCGUGGUA	CGCCAUGGCUUCCACCAAUGG	Cleavage
tae-miR7757-5p	Traes_7AS_E43D64A80.1	PF00931	KOG4658			GO:GO:0043531	AT3G46530.1	NB-ARC domain-containing disease resistance protein	LOC_Os07g26430.1	resistance protein, putative, expressed	2,05	-1.0	1	22	377	398	AUAAAACCUUCAGCUAUCCAUC	AAUGGAUAACUGAAGAUUUUAU	Cleavage
tae-miR7757-5p	Traes_7AS_E43D64A80.2	PF00931	KOG4658			GO:GO:0043531	AT3G46530.1	NB-ARC domain-containing disease resistance protein	LOC_Os07g26430.1	resistance protein, putative, expressed	2,05	-1.0	1	22	1424	1445	AUAAAACCUUCAGCUAUCCAUC	AAUGGAUAACUGAAGAUUUUAU	Cleavage
tae-miR156	Traes_7AS_FE67F9E86.1								LOC_Os08g41940.1	OsSPL16 - SBP-box gene family member, expressed	1,00	-1.0	1	21	358	378	UGACAGAAGAGAGUGAGCACA	UGUGCUCUCUCUCUUCUGUCA	Cleavage
tae-miR156	Traes_7AS_FE67F9E86.2								LOC_Os08g41940.1	OsSPL16 - SBP-box gene family member, expressed	1,00	-1.0	1	21	358	378	UGACAGAAGAGAGUGAGCACA	UGUGCUCUCUCUCUUCUGUCA	Cleavage
tae-miR156	Traes_7AS_FE67F9E86.3								LOC_Os08g41940.1	OsSPL16 - SBP-box gene family member, expressed	1,00	-1.0	1	21	331	351	UGACAGAAGAGAGUGAGCACA	UGUGCUCUCUCUCUUCUGUCA	Cleavage
tae-miR9654a-3p	Traes_7BL_03C3797A2.1	PF00635,PF00400,PF00069	KOG1187			GO:GO:0005515,GO:GO:0006468,GO:GO:0005524,GO:GO:0004672	AT1G79990.3	structural molecules	LOC_Os11g10640.1	protein kinase domain containing protein, expressed	2,00	-1.0	1	22	1813	1834	UUCUGAAAGGCUUGAAGCGAAU	GAGGGCCUCAAGCCUUUCAGAA	Cleavage
tae-miR9654a-3p	Traes_7BL_03C3797A2.2	PF00635,PF00400,PF00069	KOG1187			GO:GO:0005515,GO:GO:0006468,GO:GO:0005524,GO:GO:0004672	AT1G79990.3	structural molecules	LOC_Os11g10640.1	protein kinase domain containing protein, expressed	2,00	-1.0	1	22	1813	1834	UUCUGAAAGGCUUGAAGCGAAU	GAGGGCCUCAAGCCUUUCAGAA	Cleavage
tae-miR9654a-3p	Traes_7BL_03C3797A2.3	PF00635,PF00400,PF00069	KOG1187			GO:GO:0005515,GO:GO:0006468,GO:GO:0005524,GO:GO:0004672	AT1G79990.3	structural molecules	LOC_Os11g10640.1	protein kinase domain containing protein, expressed	2,00	-1.0	1	22	1513	1534	UUCUGAAAGGCUUGAAGCGAAU	GAGGGCCUCAAGCCUUUCAGAA	Cleavage
tae-miR9654a-3p	Traes_7BL_03C3797A2.4	PF00635,PF00400,PF00069	KOG1187			GO:GO:0005515,GO:GO:0006468,GO:GO:0005524,GO:GO:0004672	AT1G79990.3	structural molecules	LOC_Os11g10640.1	protein kinase domain containing protein, expressed	2,00	-1.0	1	22	1513	1534	UUCUGAAAGGCUUGAAGCGAAU	GAGGGCCUCAAGCCUUUCAGAA	Cleavage
tae-miR9654a-3p	Traes_7BL_03C3797A2.5	PF00635,PF00400,PF00069	KOG0294			GO:GO:0005515,GO:GO:0006468,GO:GO:0005524,GO:GO:0004672	AT1G79990.3	structural molecules	LOC_Os11g10640.1	protein kinase domain containing protein, expressed	2,00	-1.0	1	22	1399	1420	UUCUGAAAGGCUUGAAGCGAAU	GAGGGCCUCAAGCCUUUCAGAA	Cleavage
tae-miR9654a-3p	Traes_7BL_03C3797A2.6	PF00635,PF00400	KOG0294			GO:GO:0005515	AT1G79990.3	structural molecules	LOC_Os02g11830.2	coatomer subunit beta, putative, expressed	2,00	-1.0	1	22	973	994	UUCUGAAAGGCUUGAAGCGAAU	GAGGGCCUCAAGCCUUUCAGAA	Cleavage
tae-miR9654a-3p	Traes_7BL_03C3797A2.7	PF00635,PF00069	KOG1187	2.7.11.1		GO:GO:0006468,GO:GO:0005524,GO:GO:0004672	AT4G21410.1	cysteine-rich RLK (RECEPTOR-like protein kinase) 29	LOC_Os04g30030.1	cysteine-rich receptor-like protein kinase 12 precursor, putative, expressed	2,00	-1.0	1	22	1813	1834	UUCUGAAAGGCUUGAAGCGAAU	GAGGGCCUCAAGCCUUUCAGAA	Cleavage
tae-miR1130b-3p	Traes_7BL_0665E8707.6										1,00	-1.0	1	21	46	66	UCUUAUAUUAUGGGACGGAGG	CCUCCGUCCCAUAAUAUAAGU	Cleavage
tae-miR1122c-3p	Traes_7BL_0665E8707.6										2,05	-1.0	1	21	46	66	UCUAAUAUUAUGGGACGGAGG	CCUCCGUCCCAUAAUAUAAGU	Cleavage
tae-miR1133	Traes_7BL_07C4E8081.43										0,00	-1.0	1	22	46	67	CAUAUACUCCCUCCGUCCGAAA	UUUCGGACGGAGGGAGUAUAUG	Cleavage
tae-miR1133	Traes_7BL_07C4E8081.44										0,00	-1.0	1	22	46	67	CAUAUACUCCCUCCGUCCGAAA	UUUCGGACGGAGGGAGUAUAUG	Cleavage
tae-miR1133	Traes_7BL_07C4E8081.45										0,00	-1.0	1	22	46	67	CAUAUACUCCCUCCGUCCGAAA	UUUCGGACGGAGGGAGUAUAUG	Cleavage
tae-miR160	Traes_7BL_18D335F08.1						AT4G30080.1	auxin response factor 16	LOC_Os06g47150.2	auxin response factor 18, putative, expressed	0,00	-1.0	1	21	171	191	UGCCUGGCUCCCUGUAUGCCA	AGGCAUACAGGGAGCCAGGCA	Cleavage
tae-miR160	Traes_7BL_18D335F08.2						AT4G30080.1	auxin response factor 16	LOC_Os06g47150.1	auxin response factor 18, putative, expressed	0,00	-1.0	1	21	171	191	UGCCUGGCUCCCUGUAUGCCA	AGGCAUACAGGGAGCCAGGCA	Cleavage
tae-miR1137a	Traes_7BL_C184D0525.2	PF00249					AT5G61620.1	myb-like transcription factor family protein	LOC_Os08g05510.1	MYB family transcription factor, putative, expressed	0,00	-1.0	1	20	913	932	UAGUACAAAGUUGAGUCAUC	GAUGACUCAACUUUGUACUA	Cleavage
tae-miR395b	Traes_7BL_DC0D8773C.1	PF01909,PF04926,PF04928		2.7.7.19		GO:GO:0016779,GO:GO:0043631,GO:GO:0003723,GO:GO:0004652,GO:GO:0005634	AT4G32850.5	nuclear poly(a) polymerase	LOC_Os06g36360.4	expressed protein	2,05	-1.0	1	20	1385	1404	UGAAGUGUUUGGGGGAACUC	CAGUUCACCCAAAAACUUCA	Cleavage
tae-miR395b	Traes_7BL_DC0D8773C.10	PF04926,PF04928		2.7.7.19		GO:GO:0043631,GO:GO:0003723,GO:GO:0004652,GO:GO:0005634	AT4G32850.10	nuclear poly(a) polymerase	LOC_Os06g36360.1	expressed protein	2,05	-1.0	1	20	1148	1167	UGAAGUGUUUGGGGGAACUC	CAGUUCACCCAAAAACUUCA	Cleavage
tae-miR395b	Traes_7BL_DC0D8773C.11	PF04926,PF04928		2.7.7.19		GO:GO:0043631,GO:GO:0003723,GO:GO:0004652,GO:GO:0005634	AT4G32850.5	nuclear poly(a) polymerase	LOC_Os06g36360.3	expressed protein	2,05	-1.0	1	20	848	867	UGAAGUGUUUGGGGGAACUC	CAGUUCACCCAAAAACUUCA	Cleavage
tae-miR395b	Traes_7BL_DC0D8773C.12	PF04926,PF04928		2.7.7.19		GO:GO:0043631,GO:GO:0003723,GO:GO:0004652,GO:GO:0005634	AT4G32850.5	nuclear poly(a) polymerase	LOC_Os06g36360.1	expressed protein	2,05	-1.0	1	20	848	867	UGAAGUGUUUGGGGGAACUC	CAGUUCACCCAAAAACUUCA	Cleavage
tae-miR395b	Traes_7BL_DC0D8773C.13	PF04926,PF04928		2.7.7.19		GO:GO:0043631,GO:GO:0003723,GO:GO:0004652,GO:GO:0005634	AT4G32850.9	nuclear poly(a) polymerase	LOC_Os06g36360.1	expressed protein	2,05	-1.0	1	20	848	867	UGAAGUGUUUGGGGGAACUC	CAGUUCACCCAAAAACUUCA	Cleavage
tae-miR395b	Traes_7BL_DC0D8773C.14	PF04926,PF04928		2.7.7.19		GO:GO:0043631,GO:GO:0003723,GO:GO:0004652,GO:GO:0005634	AT4G32850.5	nuclear poly(a) polymerase	LOC_Os06g36360.3	expressed protein	2,05	-1.0	1	20	848	867	UGAAGUGUUUGGGGGAACUC	CAGUUCACCCAAAAACUUCA	Cleavage
tae-miR395b	Traes_7BL_DC0D8773C.2	PF01909,PF04926,PF04928		2.7.7.19		GO:GO:0016779,GO:GO:0043631,GO:GO:0003723,GO:GO:0004652,GO:GO:0005634	AT4G32850.5	nuclear poly(a) polymerase	LOC_Os06g36360.4	expressed protein	2,05	-1.0	1	20	1385	1404	UGAAGUGUUUGGGGGAACUC	CAGUUCACCCAAAAACUUCA	Cleavage
tae-miR395b	Traes_7BL_DC0D8773C.3	PF01909,PF04926,PF04928		2.7.7.19		GO:GO:0016779,GO:GO:0043631,GO:GO:0003723,GO:GO:0004652,GO:GO:0005634	AT4G32850.9	nuclear poly(a) polymerase	LOC_Os06g36360.1	expressed protein	2,05	-1.0	1	20	1385	1404	UGAAGUGUUUGGGGGAACUC	CAGUUCACCCAAAAACUUCA	Cleavage
tae-miR395b	Traes_7BL_DC0D8773C.4	PF01909,PF04926,PF04928		2.7.7.19		GO:GO:0016779,GO:GO:0043631,GO:GO:0003723,GO:GO:0004652,GO:GO:0005634	AT4G32850.5	nuclear poly(a) polymerase	LOC_Os06g36360.2	expressed protein	2,05	-1.0	1	20	1385	1404	UGAAGUGUUUGGGGGAACUC	CAGUUCACCCAAAAACUUCA	Cleavage
tae-miR395b	Traes_7BL_DC0D8773C.5	PF01909,PF04926,PF04928		2.7.7.19		GO:GO:0016779,GO:GO:0043631,GO:GO:0003723,GO:GO:0004652,GO:GO:0005634	AT4G32850.5	nuclear poly(a) polymerase	LOC_Os06g36360.2	expressed protein	2,05	-1.0	1	20	1385	1404	UGAAGUGUUUGGGGGAACUC	CAGUUCACCCAAAAACUUCA	Cleavage
tae-miR395b	Traes_7BL_DC0D8773C.6	PF04926,PF04928		2.7.7.19		GO:GO:0043631,GO:GO:0003723,GO:GO:0004652,GO:GO:0005634	AT4G32850.10	nuclear poly(a) polymerase	LOC_Os06g36360.1	expressed protein	2,05	-1.0	1	20	1148	1167	UGAAGUGUUUGGGGGAACUC	CAGUUCACCCAAAAACUUCA	Cleavage
tae-miR395b	Traes_7BL_DC0D8773C.7	PF04926,PF04928		2.7.7.19		GO:GO:0043631,GO:GO:0003723,GO:GO:0004652,GO:GO:0005634	AT4G32850.10	nuclear poly(a) polymerase	LOC_Os06g36360.2	expressed protein	2,05	-1.0	1	20	1148	1167	UGAAGUGUUUGGGGGAACUC	CAGUUCACCCAAAAACUUCA	Cleavage
tae-miR395b	Traes_7BL_DC0D8773C.8	PF04926,PF04928		2.7.7.19		GO:GO:0043631,GO:GO:0003723,GO:GO:0004652,GO:GO:0005634	AT4G32850.10	nuclear poly(a) polymerase	LOC_Os06g36360.1	expressed protein	2,05	-1.0	1	20	1148	1167	UGAAGUGUUUGGGGGAACUC	CAGUUCACCCAAAAACUUCA	Cleavage
tae-miR395b	Traes_7BL_DC0D8773C.9	PF04926,PF04928		2.7.7.19		GO:GO:0043631,GO:GO:0003723,GO:GO:0004652,GO:GO:0005634	AT4G32850.10	nuclear poly(a) polymerase	LOC_Os06g36360.1	expressed protein	2,05	-1.0	1	20	1148	1167	UGAAGUGUUUGGGGGAACUC	CAGUUCACCCAAAAACUUCA	Cleavage
tae-miR164	Traes_7BL_F1A0270C6.1								LOC_Os06g46270.1	no apical meristem protein, putative, expressed	1,00	-1.0	1	21	32	52	UGGAGAAGCAGGGCACGUGCA	AGCAAGUGCCCUGCUUCUCCA	Cleavage
tae-miR9666a-3p	Traes_7BL_F8D5E60B1.1										2,00	-1.0	1	22	5050	5071	CGGUAGGGCUGUAUGAUGGCGA	UCCCCCUCAUACAGCCCUACCU	Cleavage
tae-miR9666a-3p	Traes_7BL_F8D5E60B1.1										2,05	-1.0	1	22	4966	4987	CGGUAGGGCUGUAUGAUGGCGA	UCGCCAUCAUACAGCCCUACUU	Cleavage
tae-miR9666a-3p	Traes_7BL_F8D5E60B1.2										2,00	-1.0	1	22	5011	5032	CGGUAGGGCUGUAUGAUGGCGA	UCCCCCUCAUACAGCCCUACCU	Cleavage
tae-miR9666a-3p	Traes_7BL_F8D5E60B1.2										2,05	-1.0	1	22	4927	4948	CGGUAGGGCUGUAUGAUGGCGA	UCGCCAUCAUACAGCCCUACUU	Cleavage
tae-miR9666a-3p	Traes_7BL_F8D5E60B1.3										2,00	-1.0	1	22	4780	4801	CGGUAGGGCUGUAUGAUGGCGA	UCCCCCUCAUACAGCCCUACCU	Cleavage
tae-miR9666a-3p	Traes_7BL_F8D5E60B1.3										2,05	-1.0	1	22	4696	4717	CGGUAGGGCUGUAUGAUGGCGA	UCGCCAUCAUACAGCCCUACUU	Cleavage
tae-miR9666a-3p	Traes_7BL_F8D5E60B1.4										2,00	-1.0	1	22	3409	3430	CGGUAGGGCUGUAUGAUGGCGA	UCCCCCUCAUACAGCCCUACCU	Cleavage
tae-miR9666a-3p	Traes_7BL_F8D5E60B1.4										2,05	-1.0	1	22	3325	3346	CGGUAGGGCUGUAUGAUGGCGA	UCGCCAUCAUACAGCCCUACUU	Cleavage
tae-miR1137a	Traes_7BS_2A6C29F49.3	PF00566					AT3G49350.1	Ypt/Rab-GAP domain of gyp1p superfamily protein	LOC_Os09g35960.1	TBC domain containing protein, expressed	2,00	-1.0	1	20	75	94	UAGUACAAAGUUGAGUCAUC	AGUGUCUCAACUUUGUACUA	Cleavage
tae-miR1137a	Traes_7BS_2A6C29F49.4	PF00566					AT3G49350.1	Ypt/Rab-GAP domain of gyp1p superfamily protein	LOC_Os09g35960.1	TBC domain containing protein, expressed	2,00	-1.0	1	20	81	100	UAGUACAAAGUUGAGUCAUC	AGUGUCUCAACUUUGUACUA	Cleavage
tae-miR1130b-3p	Traes_7BS_47B8FC9CB.2	PF01154,PF08540		2.3.3.10		GO:GO:0008299,GO:GO:0004421	AT4G11820.2	hydroxymethylglutaryl-CoA synthase / HMG-CoA synthase / 3-hydroxy-3-methylglutaryl coenzyme A synthase	LOC_Os08g43170.1	hydroxymethylglutaryl-CoA synthase, putative, expressed	0,00	-1.0	1	21	1268	1288	UCUUAUAUUAUGGGACGGAGG	CCUCCGUCCCAUAAUAUAAGA	Cleavage
tae-miR1122c-3p	Traes_7BS_47B8FC9CB.2	PF01154,PF08540		2.3.3.10		GO:GO:0008299,GO:GO:0004421	AT4G11820.2	hydroxymethylglutaryl-CoA synthase / HMG-CoA synthase / 3-hydroxy-3-methylglutaryl coenzyme A synthase	LOC_Os08g43170.1	hydroxymethylglutaryl-CoA synthase, putative, expressed	1,05	-1.0	1	21	1268	1288	UCUAAUAUUAUGGGACGGAGG	CCUCCGUCCCAUAAUAUAAGA	Cleavage
tae-miR1120c-5p	Traes_7BS_47B8FC9CB.2	PF01154,PF08540		2.3.3.10		GO:GO:0008299,GO:GO:0004421	AT4G11820.2	hydroxymethylglutaryl-CoA synthase / HMG-CoA synthase / 3-hydroxy-3-methylglutaryl coenzyme A synthase	LOC_Os08g43170.1	hydroxymethylglutaryl-CoA synthase, putative, expressed	1,05	-1.0	1	21	1311	1331	UAAUAUAAGAACGUUUUUGAC	GUCAAAAACGCUCUUAUAUUA	Translation
tae-miR1130b-3p	Traes_7BS_47B8FC9CB.3	PF01154,PF08540		2.3.3.10	K01641	GO:GO:0008299,GO:GO:0004421	AT4G11820.2	hydroxymethylglutaryl-CoA synthase / HMG-CoA synthase / 3-hydroxy-3-methylglutaryl coenzyme A synthase	LOC_Os08g43170.1	hydroxymethylglutaryl-CoA synthase, putative, expressed	0,00	-1.0	1	21	1265	1285	UCUUAUAUUAUGGGACGGAGG	CCUCCGUCCCAUAAUAUAAGA	Cleavage
tae-miR1122c-3p	Traes_7BS_47B8FC9CB.3	PF01154,PF08540		2.3.3.10	K01641	GO:GO:0008299,GO:GO:0004421	AT4G11820.2	hydroxymethylglutaryl-CoA synthase / HMG-CoA synthase / 3-hydroxy-3-methylglutaryl coenzyme A synthase	LOC_Os08g43170.1	hydroxymethylglutaryl-CoA synthase, putative, expressed	1,05	-1.0	1	21	1265	1285	UCUAAUAUUAUGGGACGGAGG	CCUCCGUCCCAUAAUAUAAGA	Cleavage
tae-miR1120c-5p	Traes_7BS_47B8FC9CB.3	PF01154,PF08540		2.3.3.10	K01641	GO:GO:0008299,GO:GO:0004421	AT4G11820.2	hydroxymethylglutaryl-CoA synthase / HMG-CoA synthase / 3-hydroxy-3-methylglutaryl coenzyme A synthase	LOC_Os08g43170.1	hydroxymethylglutaryl-CoA synthase, putative, expressed	1,05	-1.0	1	21	1308	1328	UAAUAUAAGAACGUUUUUGAC	GUCAAAAACGCUCUUAUAUUA	Translation
tae-miR9662a-3p	Traes_7BS_598310C32.1	PF02536				GO:GO:0006355,GO:GO:0005739,GO:GO:0003690	AT1G21150.1	Mitochondrial transcription termination factor family protein	LOC_Os06g12100.1	mTERF family protein, expressed	2,05	-1.0	1	21	33	53	UUGAACAUCCCAGAGCCACCG	CCGUGGCUCUGCGAUGUUCAG	Translation
tae-miR9662b-3p	Traes_7BS_598310C32.1	PF02536				GO:GO:0006355,GO:GO:0005739,GO:GO:0003690	AT1G21150.1	Mitochondrial transcription termination factor family protein	LOC_Os06g12100.1	mTERF family protein, expressed	2,05	-1.0	1	21	32	52	UGAACAUCCCAGAGCCACCGG	CCCGUGGCUCUGCGAUGUUCA	Cleavage
tae-miR1121	Traes_7BS_5A0E77FB8.2	PF00664,PF00005		3.6.3.44		GO:GO:0055085,GO:GO:0042626,GO:GO:0016021,GO:GO:0006810,GO:GO:0005524,GO:GO:0016887	AT3G59140.1	multidrug resistance-associated protein 14	LOC_Os06g06440.1	ABC transporter, ATP-binding protein, putative, expressed	1,00	-1.0	1	22	3478	3499	AGUAGUGAUCUAAACGCUCUUA	UAAGAGUGUUUAGAUCACUACU	Cleavage
tae-miR1137a	Traes_7BS_6716D1D07.2	PF01105				GO:GO:0016021,GO:GO:0006810	AT1G09580.1	emp24/gp25L/p24 family/GOLD family protein	LOC_Os06g10760.1	emp24/gp25L/p24 family protein, putative, expressed	0,00	-1.0	1	20	347	366	UAGUACAAAGUUGAGUCAUC	GAUGACUCAACUUUGUACUA	Cleavage
tae-miR9781	Traes_7BS_A42F893FC.1	PF10150		3.1.26.12		GO:GO:0003723	AT2G04270.2	RNAse E/G-like	LOC_Os08g23430.1	starch binding domain containing protein, putative, expressed	1,05	-1.0	1	21	117	137	UUUUGUCACAUAUAAUACAUA	UAUGUAUUUUAUGUGACAAAA	Cleavage
tae-miR9664-3p	Traes_7DL_101A9F756.1	PF00931	KOG4658			GO:GO:0043531	AT1G58807.1	Disease resistance protein (CC-NBS-LRR class) family	LOC_Os11g11550.1	NBS-LRR disease resistance protein, putative, expressed	2,05	-1.0	1	21	102	122	UUGCAGUCCUCGAUGUCGUAG	GCACGACAUCGAGGAUUGCAU	Cleavage
tae-miR9664-3p	Traes_7DL_101A9F756.2	PF00931	KOG4658			GO:GO:0043531	AT1G58807.1	Disease resistance protein (CC-NBS-LRR class) family	LOC_Os11g11550.2	NBS-LRR disease resistance protein, putative, expressed	2,05	-1.0	1	21	102	122	UUGCAGUCCUCGAUGUCGUAG	GCACGACAUCGAGGAUUGCAU	Cleavage
tae-miR9664-3p	Traes_7DL_101A9F756.3	PF00931	KOG4658			GO:GO:0043531	AT1G58807.1	Disease resistance protein (CC-NBS-LRR class) family	LOC_Os11g11550.1	NBS-LRR disease resistance protein, putative, expressed	2,05	-1.0	1	21	231	251	UUGCAGUCCUCGAUGUCGUAG	GCACGACAUCGAGGAUUGCAU	Cleavage
tae-miR9666a-3p	Traes_7DL_18F2A8567.1										2,05	-1.0	1	22	4927	4948	CGGUAGGGCUGUAUGAUGGCGA	UCGCCAUCAUACAGCCCUACUU	Cleavage
tae-miR9666a-3p	Traes_7DL_18F2A8567.11										2,00	-1.0	1	22	244	265	CGGUAGGGCUGUAUGAUGGCGA	UCGCCCUCAUACAGCCCUACCU	Cleavage
tae-miR9666a-3p	Traes_7DL_18F2A8567.11										2,05	-1.0	1	22	139	160	CGGUAGGGCUGUAUGAUGGCGA	UCGCCAUCAUACAGCCCUACUU	Cleavage
tae-miR9666a-3p	Traes_7DL_18F2A8567.12										2,00	-1.0	1	22	250	271	CGGUAGGGCUGUAUGAUGGCGA	UCGCCCUCAUACAGCCCUACCU	Cleavage
tae-miR9666a-3p	Traes_7DL_18F2A8567.12										2,05	-1.0	1	22	145	166	CGGUAGGGCUGUAUGAUGGCGA	UCGCCAUCAUACAGCCCUACUU	Cleavage
tae-miR9666a-3p	Traes_7DL_18F2A8567.14										2,00	-1.0	1	22	201	222	CGGUAGGGCUGUAUGAUGGCGA	UCGCCCUCAUACAGCCCUACCU	Cleavage
tae-miR9666a-3p	Traes_7DL_18F2A8567.14										2,05	-1.0	1	22	96	117	CGGUAGGGCUGUAUGAUGGCGA	UCGCCAUCAUACAGCCCUACUU	Cleavage
tae-miR9666a-3p	Traes_7DL_18F2A8567.2										2,00	-1.0	1	22	5032	5053	CGGUAGGGCUGUAUGAUGGCGA	UCGCCCUCAUACAGCCCUACCU	Cleavage
tae-miR9666a-3p	Traes_7DL_18F2A8567.2										2,05	-1.0	1	22	4927	4948	CGGUAGGGCUGUAUGAUGGCGA	UCGCCAUCAUACAGCCCUACUU	Cleavage
tae-miR9666a-3p	Traes_7DL_18F2A8567.3										2,00	-1.0	1	22	4969	4990	CGGUAGGGCUGUAUGAUGGCGA	UCGCCCUCAUACAGCCCUACCU	Cleavage
tae-miR9666a-3p	Traes_7DL_18F2A8567.3										2,05	-1.0	1	22	4927	4948	CGGUAGGGCUGUAUGAUGGCGA	UCGCCAUCAUACAGCCCUACUU	Cleavage
tae-miR9666a-3p	Traes_7DL_18F2A8567.4										2,05	-1.0	1	22	4927	4948	CGGUAGGGCUGUAUGAUGGCGA	UCGCCAUCAUACAGCCCUACUU	Cleavage
tae-miR9666a-3p	Traes_7DL_18F2A8567.5										2,05	-1.0	1	22	4927	4948	CGGUAGGGCUGUAUGAUGGCGA	UCGCCAUCAUACAGCCCUACUU	Cleavage
tae-miR9666a-3p	Traes_7DL_18F2A8567.6										2,00	-1.0	1	22	5032	5053	CGGUAGGGCUGUAUGAUGGCGA	UCGCCCUCAUACAGCCCUACCU	Cleavage
tae-miR9666a-3p	Traes_7DL_18F2A8567.6										2,05	-1.0	1	22	4927	4948	CGGUAGGGCUGUAUGAUGGCGA	UCGCCAUCAUACAGCCCUACUU	Cleavage
tae-miR9666a-3p	Traes_7DL_18F2A8567.7										2,00	-1.0	1	22	4801	4822	CGGUAGGGCUGUAUGAUGGCGA	UCGCCCUCAUACAGCCCUACCU	Cleavage
tae-miR9666a-3p	Traes_7DL_18F2A8567.7										2,05	-1.0	1	22	4696	4717	CGGUAGGGCUGUAUGAUGGCGA	UCGCCAUCAUACAGCCCUACUU	Cleavage
tae-miR9666a-3p	Traes_7DL_18F2A8567.8										2,00	-1.0	1	22	3409	3430	CGGUAGGGCUGUAUGAUGGCGA	UCGCCCUCAUACAGCCCUACCU	Cleavage
tae-miR9666a-3p	Traes_7DL_18F2A8567.8										2,05	-1.0	1	22	3304	3325	CGGUAGGGCUGUAUGAUGGCGA	UCGCCAUCAUACAGCCCUACUU	Cleavage
tae-miR9666a-3p	Traes_7DL_18F2A8567.9										2,00	-1.0	1	22	2941	2962	CGGUAGGGCUGUAUGAUGGCGA	UCGCCCUCAUACAGCCCUACCU	Cleavage
tae-miR9666a-3p	Traes_7DL_18F2A8567.9										2,05	-1.0	1	22	2836	2857	CGGUAGGGCUGUAUGAUGGCGA	UCGCCAUCAUACAGCCCUACUU	Cleavage
tae-miR164	Traes_7DL_1E343B6E6.1						AT1G56010.2	NAC domain containing protein 1	LOC_Os06g46270.1	no apical meristem protein, putative, expressed	1,00	-1.0	1	21	236	256	UGGAGAAGCAGGGCACGUGCA	AGCAAGUGCCCUGCUUCUCCA	Cleavage
tae-miR164	Traes_7DL_1E343B6E6.2								LOC_Os06g46270.1	no apical meristem protein, putative, expressed	1,00	-1.0	1	21	32	52	UGGAGAAGCAGGGCACGUGCA	AGCAAGUGCCCUGCUUCUCCA	Cleavage
tae-miR1135	Traes_7DL_20BFD8A19.4	PF00145		2.1.1.37			AT4G19020.1	chromomethylase 2	LOC_Os10g01570.1	C-5 cytosine-specific DNA methylase, putative, expressed	1,05	-1.0	1	24	954	977	CUGCGACAAGUAAUUCCGAACGGA	UCUGUUCGGAAUUACUUGUCGUAG	Cleavage
tae-miR1127a	Traes_7DL_20BFD8A19.4	PF00145		2.1.1.37			AT4G19020.1	chromomethylase 2	LOC_Os10g01570.1	C-5 cytosine-specific DNA methylase, putative, expressed	1,05	-1.0	1	19	1032	1050	UCCUUCCGUUCGGAAUUAC	GUAAUUCCGAACGGAGGGA	Cleavage
tae-miR9778	Traes_7DL_31A23053A.1								LOC_Os11g39600.1	resistance protein, putative, expressed	1,00	-1.0	1	21	132	152	UGCAUCAUCUCGAACUCGUCG	CCUCGAGUUCGAGAUGAUGCA	Cleavage
tae-miR1122a	Traes_7DL_37135CE6E.2	PF00076	KOG0128			GO:GO:0003676	AT4G24270.2	EMBRYO DEFECTIVE 140	LOC_Os08g02130.2	RNA recognition motif containing protein, putative, expressed	2,05	-1.0	1	20	2105	2124	UAGAUACAUCCGUAUCUAGA	UCUAAAUACGGAUGUAUCAA	Cleavage
tae-miR1122a	Traes_7DL_37135CE6E.3	PF00076	KOG0128			GO:GO:0003676	AT4G24270.2	EMBRYO DEFECTIVE 140	LOC_Os08g02130.2	RNA recognition motif containing protein, putative, expressed	2,05	-1.0	1	20	2090	2109	UAGAUACAUCCGUAUCUAGA	UCUAAAUACGGAUGUAUCAA	Cleavage
tae-miR1122a	Traes_7DL_37135CE6E.6	PF00076	KOG0130			GO:GO:0003676	AT4G24270.2	EMBRYO DEFECTIVE 140	LOC_Os08g02130.1	RNA recognition motif containing protein, putative, expressed	2,05	-1.0	1	20	1241	1260	UAGAUACAUCCGUAUCUAGA	UCUAAAUACGGAUGUAUCAA	Cleavage
tae-miR160	Traes_7DL_55ADB3528.1	PF06507				GO:GO:0009725,GO:GO:0006355,GO:GO:0005634,GO:GO:0003677	AT4G30080.1	auxin response factor 16	LOC_Os06g47150.1	auxin response factor 18, putative, expressed	0,00	-1.0	1	21	714	734	UGCCUGGCUCCCUGUAUGCCA	AGGCAUACAGGGAGCCAGGCA	Cleavage
tae-miR160	Traes_7DL_55ADB3528.2	PF06507				GO:GO:0009725,GO:GO:0006355,GO:GO:0005634,GO:GO:0003677	AT4G30080.1	auxin response factor 16	LOC_Os06g47150.3	auxin response factor 18, putative, expressed	0,00	-1.0	1	21	714	734	UGCCUGGCUCCCUGUAUGCCA	AGGCAUACAGGGAGCCAGGCA	Cleavage
tae-miR1134	Traes_7DL_5E2559796.1										2,00	-1.0	1	24	2562	2585	CAACAACAACAAGAAGAAGAAGAU	UUGUUGUUGUUCUUGUUGUUGUUG	Cleavage
tae-miR1134	Traes_7DL_5E2559796.2										2,00	-1.0	1	24	2556	2579	CAACAACAACAAGAAGAAGAAGAU	UUGUUGUUGUUCUUGUUGUUGUUG	Cleavage
tae-miR1122b-3p	Traes_7DL_613007B24.2	PF00400	KOG0279,KOG1332			GO:GO:0005515	AT1G15750.3	Transducin family protein / WD-40 repeat family protein	LOC_Os08g06480.1	lissencephaly type-1-like homology motif, putative, expressed	1,05	-1.0	1	21	1492	1512	AGACUUAUAUGUAGGAACGGA	UCCGUUCCUAAAUAUAAGUCU	Translation
tae-miR164	Traes_7DL_91CFE828F.1	PF02365				GO:GO:0006355,GO:GO:0003677	AT1G56010.2	NAC domain containing protein 1	LOC_Os06g46270.1	no apical meristem protein, putative, expressed	1,00	-1.0	1	21	497	517	UGGAGAAGCAGGGCACGUGCA	AGCAAGUGCCCUGCUUCUCCA	Cleavage
tae-miR164	Traes_7DL_91CFE828F.2						AT1G56010.2	NAC domain containing protein 1	LOC_Os06g46270.1	no apical meristem protein, putative, expressed	1,00	-1.0	1	21	296	316	UGGAGAAGCAGGGCACGUGCA	AGCAAGUGCCCUGCUUCUCCA	Cleavage
tae-miR9778	Traes_7DL_9F0AD190A.2	PF00931	KOG4658			GO:GO:0043531	AT1G58400.1	Disease resistance protein (CC-NBS-LRR class) family	LOC_Os08g14850.1	resistance protein, putative, expressed	2,05	-1.0	1	21	126	146	UGCAUCAUCUCGAACUCGUCG	AGGCGAGUUUGAGAUGAUGCA	Cleavage
tae-miR9778	Traes_7DL_9F0AD190A.3	PF00931	KOG4658			GO:GO:0043531	AT1G58400.1	Disease resistance protein (CC-NBS-LRR class) family	LOC_Os08g14850.1	resistance protein, putative, expressed	2,05	-1.0	1	21	126	146	UGCAUCAUCUCGAACUCGUCG	AGGCGAGUUUGAGAUGAUGCA	Cleavage
tae-miR5048-5p	Traes_7DL_AB7C59D49.1	PF00069		2.7.11.1		GO:GO:0006468,GO:GO:0005524,GO:GO:0004672	AT4G21230.1	cysteine-rich RLK (RECEPTOR-like protein kinase) 27	LOC_Os11g12270.2	expressed protein	0,00	-1.0	1	22	53	74	UUUGCAGGUUUUAGGUCUAAGU	ACUUAGACCUAAAACCUGCAAA	Cleavage
tae-miR5048-5p	Traes_7DL_AB7C59D49.2	PF00069		2.7.11.1		GO:GO:0006468,GO:GO:0005524,GO:GO:0004672	AT1G11300.1	protein serine/threonine kinases;protein kinases;ATP binding;sugar binding;kinases;carbohydrate binding	LOC_Os11g17380.1	protein kinase domain containing protein, expressed	0,00	-1.0	1	22	53	74	UUUGCAGGUUUUAGGUCUAAGU	ACUUAGACCUAAAACCUGCAAA	Cleavage
tae-miR5048-5p	Traes_7DL_AB7C59D49.3	PF00069		2.7.11.1		GO:GO:0006468,GO:GO:0005524,GO:GO:0004672	AT1G11300.1	protein serine/threonine kinases;protein kinases;ATP binding;sugar binding;kinases;carbohydrate binding	LOC_Os11g17380.1	protein kinase domain containing protein, expressed	0,00	-1.0	1	22	53	74	UUUGCAGGUUUUAGGUCUAAGU	ACUUAGACCUAAAACCUGCAAA	Cleavage
tae-miR5048-5p	Traes_7DL_AB7C59D49.4	PF00069		2.7.11.1		GO:GO:0006468,GO:GO:0005524,GO:GO:0004672	AT1G11300.1	protein serine/threonine kinases;protein kinases;ATP binding;sugar binding;kinases;carbohydrate binding	LOC_Os11g17380.1	protein kinase domain containing protein, expressed	0,00	-1.0	1	22	53	74	UUUGCAGGUUUUAGGUCUAAGU	ACUUAGACCUAAAACCUGCAAA	Cleavage
tae-miR5048-5p	Traes_7DL_AB7C59D49.5	PF00069		2.7.11.1		GO:GO:0006468,GO:GO:0005524,GO:GO:0004672	AT4G21230.1	cysteine-rich RLK (RECEPTOR-like protein kinase) 27	LOC_Os11g12270.2	expressed protein	0,00	-1.0	1	22	53	74	UUUGCAGGUUUUAGGUCUAAGU	ACUUAGACCUAAAACCUGCAAA	Cleavage
tae-miR5048-5p	Traes_7DL_AB7C59D49.6	PF00069		2.7.11.1		GO:GO:0006468,GO:GO:0005524,GO:GO:0004672	AT4G21230.1	cysteine-rich RLK (RECEPTOR-like protein kinase) 27	LOC_Os11g12270.2	expressed protein	0,00	-1.0	1	22	53	74	UUUGCAGGUUUUAGGUCUAAGU	ACUUAGACCUAAAACCUGCAAA	Cleavage
tae-miR1122a	Traes_7DL_C074DC1F6.3	PF00141		1.11.1.7		GO:GO:0055114,GO:GO:0020037,GO:GO:0006979,GO:GO:0004601	AT5G64120.1	Peroxidase superfamily protein	LOC_Os07g44550.1	peroxidase precursor, putative, expressed	2,05	-1.0	1	20	109	128	UAGAUACAUCCGUAUCUAGA	ACUACAUACGGAUGUAUAUA	Cleavage
tae-miR395b	Traes_7DL_CB073ADC6.1	PF01909,PF04926,PF04928		2.7.7.19	K14376	GO:GO:0016779,GO:GO:0043631,GO:GO:0003723,GO:GO:0004652,GO:GO:0005634	AT4G32850.5	nuclear poly(a) polymerase	LOC_Os06g36360.3	expressed protein	2,05	-1.0	1	20	1598	1617	UGAAGUGUUUGGGGGAACUC	CAGUUCACCCAAAAACUUCA	Cleavage
tae-miR395b	Traes_7DL_CB073ADC6.10	PF04926,PF04928		2.7.7.19		GO:GO:0043631,GO:GO:0003723,GO:GO:0004652,GO:GO:0005634	AT4G32850.9	nuclear poly(a) polymerase	LOC_Os06g36360.1	expressed protein	2,05	-1.0	1	20	851	870	UGAAGUGUUUGGGGGAACUC	CAGUUCACCCAAAAACUUCA	Cleavage
tae-miR395b	Traes_7DL_CB073ADC6.11	PF04926,PF04928		2.7.7.19		GO:GO:0043631,GO:GO:0003723,GO:GO:0004652,GO:GO:0005634	AT4G32850.5	nuclear poly(a) polymerase	LOC_Os06g36360.2	expressed protein	2,05	-1.0	1	20	851	870	UGAAGUGUUUGGGGGAACUC	CAGUUCACCCAAAAACUUCA	Cleavage
tae-miR395b	Traes_7DL_CB073ADC6.12	PF04926,PF04928		2.7.7.19		GO:GO:0043631,GO:GO:0003723,GO:GO:0004652,GO:GO:0005634	AT4G32850.9	nuclear poly(a) polymerase	LOC_Os06g36360.2	expressed protein	2,05	-1.0	1	20	851	870	UGAAGUGUUUGGGGGAACUC	CAGUUCACCCAAAAACUUCA	Cleavage
tae-miR395b	Traes_7DL_CB073ADC6.2	PF01909,PF04926,PF04928		2.7.7.19	K14376	GO:GO:0016779,GO:GO:0043631,GO:GO:0003723,GO:GO:0004652,GO:GO:0005634	AT4G32850.5	nuclear poly(a) polymerase	LOC_Os06g36360.2	expressed protein	2,05	-1.0	1	20	1598	1617	UGAAGUGUUUGGGGGAACUC	CAGUUCACCCAAAAACUUCA	Cleavage
tae-miR395b	Traes_7DL_CB073ADC6.3	PF01909,PF04926,PF04928		2.7.7.19	K14376	GO:GO:0016779,GO:GO:0043631,GO:GO:0003723,GO:GO:0004652,GO:GO:0005634	AT4G32850.9	nuclear poly(a) polymerase	LOC_Os06g36360.1	expressed protein	2,05	-1.0	1	20	1598	1617	UGAAGUGUUUGGGGGAACUC	CAGUUCACCCAAAAACUUCA	Cleavage
tae-miR395b	Traes_7DL_CB073ADC6.4	PF04926,PF04928		2.7.7.19		GO:GO:0043631,GO:GO:0003723,GO:GO:0004652,GO:GO:0005634	AT4G32850.10	nuclear poly(a) polymerase	LOC_Os06g36360.3	expressed protein	2,05	-1.0	1	20	1157	1176	UGAAGUGUUUGGGGGAACUC	CAGUUCACCCAAAAACUUCA	Cleavage
tae-miR395b	Traes_7DL_CB073ADC6.5	PF04926,PF04928		2.7.7.19		GO:GO:0043631,GO:GO:0003723,GO:GO:0004652,GO:GO:0005634	AT4G32850.10	nuclear poly(a) polymerase	LOC_Os06g36360.1	expressed protein	2,05	-1.0	1	20	1157	1176	UGAAGUGUUUGGGGGAACUC	CAGUUCACCCAAAAACUUCA	Cleavage
tae-miR395b	Traes_7DL_CB073ADC6.6	PF04926,PF04928		2.7.7.19		GO:GO:0043631,GO:GO:0003723,GO:GO:0004652,GO:GO:0005634	AT4G32850.10	nuclear poly(a) polymerase	LOC_Os06g36360.3	expressed protein	2,05	-1.0	1	20	1157	1176	UGAAGUGUUUGGGGGAACUC	CAGUUCACCCAAAAACUUCA	Cleavage
tae-miR395b	Traes_7DL_CB073ADC6.7	PF04926,PF04928		2.7.7.19		GO:GO:0043631,GO:GO:0003723,GO:GO:0004652,GO:GO:0005634	AT4G32850.10	nuclear poly(a) polymerase	LOC_Os06g36360.2	expressed protein	2,05	-1.0	1	20	1157	1176	UGAAGUGUUUGGGGGAACUC	CAGUUCACCCAAAAACUUCA	Cleavage
tae-miR395b	Traes_7DL_CB073ADC6.8	PF04926,PF04928		2.7.7.19		GO:GO:0043631,GO:GO:0003723,GO:GO:0004652,GO:GO:0005634	AT4G32850.10	nuclear poly(a) polymerase	LOC_Os06g36360.2	expressed protein	2,05	-1.0	1	20	1079	1098	UGAAGUGUUUGGGGGAACUC	CAGUUCACCCAAAAACUUCA	Cleavage
tae-miR395b	Traes_7DL_CB073ADC6.9	PF04926,PF04928		2.7.7.19		GO:GO:0043631,GO:GO:0003723,GO:GO:0004652,GO:GO:0005634	AT4G32850.9	nuclear poly(a) polymerase	LOC_Os06g36360.4	expressed protein	2,05	-1.0	1	20	851	870	UGAAGUGUUUGGGGGAACUC	CAGUUCACCCAAAAACUUCA	Cleavage
tae-miR1130b-3p	Traes_7DL_CF6AABA1F.2						AT4G31430.3		LOC_Os06g40460.1	expressed protein	0,00	-1.0	1	21	1271	1291	UCUUAUAUUAUGGGACGGAGG	CCUCCGUCCCAUAAUAUAAGA	Cleavage
tae-miR1122c-3p	Traes_7DL_CF6AABA1F.2						AT4G31430.3		LOC_Os06g40460.1	expressed protein	1,05	-1.0	1	21	1271	1291	UCUAAUAUUAUGGGACGGAGG	CCUCCGUCCCAUAAUAUAAGA	Cleavage
tae-miR1128	Traes_7DL_D5FCF9F33.4								LOC_Os06g46480.1	expressed protein	2,05	-1.0	1	21	96	116	UACUACUCCCUCCGUCCGAAA	UUUGGGACGGAGGGAGUAUUA	Cleavage
tae-miR1122a	Traes_7DL_D5FCF9F33.4								LOC_Os06g46480.1	expressed protein	0,00	-1.0	1	20	23	42	UAGAUACAUCCGUAUCUAGA	UCUAGAUACGGAUGUAUCUA	Cleavage
tae-miR5049-3p	Traes_7DS_06CD5BBFB.4						AT3G21350.2	RNA polymerase transcriptional regulation mediator-related	LOC_Os06g11370.1	mediator of RNA polymerase II transcription subunit 6, putative, expressed	0,00	-1.0	1	21	99	119	AAUAUGGAUCGGAGGGAGUAC	UUACUCCCUCCGAUCCAUAUU	Cleavage
tae-miR1134	Traes_7DS_205267E87.1										2,05	-1.0	1	24	92	115	CAACAACAACAAGAAGAAGAAGAU	CUGUUCUUCUUCUUGUUGUUGUCU	Cleavage
tae-miR1130b-3p	Traes_7DS_25B02BA46.5	PF00005		3.6.3.44		GO:GO:0016887,GO:GO:0005524	AT3G53480.1	pleiotropic drug resistance 9	LOC_Os02g32690.1	pleiotropic drug resistance protein 15, putative, expressed	1,05	-1.0	1	21	730	750	UCUUAUAUUAUGGGACGGAGG	CCUCCGUCCCAUAAUGUAAGA	Cleavage
tae-miR1133	Traes_7DS_25F082B1E.8										3,00	-1.0	1	22	19	41	CAUAUACUCCCUCCGUCC-GAAA	UUUCCGGACGGAGGGAGUAUAUA	Cleavage
tae-miR9662a-3p	Traes_7DS_2B1FE04A6.1	PF02536				GO:GO:0006355,GO:GO:0005739,GO:GO:0003690	AT5G07900.1	Mitochondrial transcription termination factor family protein	LOC_Os06g12100.1	mTERF family protein, expressed	2,05	-1.0	1	21	561	581	UUGAACAUCCCAGAGCCACCG	CCGUGGCUCUGCGAUGUUCAG	Translation
tae-miR9662b-3p	Traes_7DS_2B1FE04A6.1	PF02536				GO:GO:0006355,GO:GO:0005739,GO:GO:0003690	AT5G07900.1	Mitochondrial transcription termination factor family protein	LOC_Os06g12100.1	mTERF family protein, expressed	2,05	-1.0	1	21	560	580	UGAACAUCCCAGAGCCACCGG	CCCGUGGCUCUGCGAUGUUCA	Cleavage
tae-miR1130b-3p	Traes_7DS_3C83C8EF9.1						AT4G31340.1	myosin heavy chain-related	LOC_Os08g40790.1	DNA repair ATPase-related, putative, expressed	1,00	-1.0	1	21	37	57	UCUUAUAUUAUGGGACGGAGG	CCUCCGUUCCAUAAUAUAAGA	Cleavage
tae-miR1122c-3p	Traes_7DS_3C83C8EF9.1						AT4G31340.1	myosin heavy chain-related	LOC_Os08g40790.1	DNA repair ATPase-related, putative, expressed	2,05	-1.0	1	21	37	57	UCUAAUAUUAUGGGACGGAGG	CCUCCGUUCCAUAAUAUAAGA	Cleavage
tae-miR1120c-5p	Traes_7DS_3C83C8EF9.1						AT4G31340.1	myosin heavy chain-related	LOC_Os08g40790.1	DNA repair ATPase-related, putative, expressed	1,05	-1.0	1	21	80	100	UAAUAUAAGAACGUUUUUGAC	GUCAAAAACGCUCUUAUAUUA	Translation
tae-miR1130b-3p	Traes_7DS_3C83C8EF9.3						AT4G31340.1	myosin heavy chain-related	LOC_Os08g40790.3	DNA repair ATPase-related, putative, expressed	1,00	-1.0	1	21	37	57	UCUUAUAUUAUGGGACGGAGG	CCUCCGUUCCAUAAUAUAAGA	Cleavage
tae-miR1122c-3p	Traes_7DS_3C83C8EF9.3						AT4G31340.1	myosin heavy chain-related	LOC_Os08g40790.3	DNA repair ATPase-related, putative, expressed	2,05	-1.0	1	21	37	57	UCUAAUAUUAUGGGACGGAGG	CCUCCGUUCCAUAAUAUAAGA	Cleavage
tae-miR1120c-5p	Traes_7DS_3C83C8EF9.3						AT4G31340.1	myosin heavy chain-related	LOC_Os08g40790.3	DNA repair ATPase-related, putative, expressed	1,05	-1.0	1	21	80	100	UAAUAUAAGAACGUUUUUGAC	GUCAAAAACGCUCUUAUAUUA	Translation
tae-miR9662a-3p	Traes_7DS_5B1210681.1	PF02536			K15032	GO:GO:0006355,GO:GO:0005739,GO:GO:0003690	AT1G21150.1	Mitochondrial transcription termination factor family protein	LOC_Os06g12100.1	mTERF family protein, expressed	2,05	-1.0	1	21	285	305	UUGAACAUCCCAGAGCCACCG	CCGUGGCUCUGCGAUGUUCAG	Translation
tae-miR9662b-3p	Traes_7DS_5B1210681.1	PF02536			K15032	GO:GO:0006355,GO:GO:0005739,GO:GO:0003690	AT1G21150.1	Mitochondrial transcription termination factor family protein	LOC_Os06g12100.1	mTERF family protein, expressed	2,05	-1.0	1	21	284	304	UGAACAUCCCAGAGCCACCGG	CCCGUGGCUCUGCGAUGUUCA	Cleavage

tae-miR9778	Traes_7DS_74534FCD3.1	PF00931				GO:GO:0043531	AT1G50180.1	NB-ARC domain-containing disease resistance protein	LOC_Os10g03570.1	RGH1A, putative, expressed	2,05	-1.0	1	21	111	131	UGCAUCAUCUCGAACUCGUCG	AGACGAGCUGGAGAUGAUGCA	Cleavage
tae-miR9778	Traes_7DS_74534FCD3.2	PF00931				GO:GO:0043531	AT3G46530.1	NB-ARC domain-containing disease resistance protein	LOC_Os10g03570.1	RGH1A, putative, expressed	2,05	-1.0	1	21	111	131	UGCAUCAUCUCGAACUCGUCG	AGACGAGCUGGAGAUGAUGCA	Cleavage
tae-miR9778	Traes_7DS_74534FCD3.3	PF00931				GO:GO:0043531	AT3G46530.1	NB-ARC domain-containing disease resistance protein	LOC_Os10g03570.1	RGH1A, putative, expressed	2,05	-1.0	1	21	111	131	UGCAUCAUCUCGAACUCGUCG	AGACGAGCUGGAGAUGAUGCA	Cleavage
tae-miR9778	Traes_7DS_74534FCD3.4	PF00931				GO:GO:0043531	AT3G46530.1	NB-ARC domain-containing disease resistance protein	LOC_Os10g03570.1	RGH1A, putative, expressed	2,05	-1.0	1	21	111	131	UGCAUCAUCUCGAACUCGUCG	AGACGAGCUGGAGAUGAUGCA	Cleavage
tae-miR9778	Traes_7DS_74534FCD3.5	PF00931				GO:GO:0043531	AT3G46530.1	NB-ARC domain-containing disease resistance protein	LOC_Os10g03570.1	RGH1A, putative, expressed	2,05	-1.0	1	21	111	131	UGCAUCAUCUCGAACUCGUCG	AGACGAGCUGGAGAUGAUGCA	Cleavage
tae-miR9778	Traes_7DS_74534FCD3.6	PF00931				GO:GO:0043531	AT3G46530.1	NB-ARC domain-containing disease resistance protein	LOC_Os10g03570.1	RGH1A, putative, expressed	2,05	-1.0	1	21	111	131	UGCAUCAUCUCGAACUCGUCG	AGACGAGCUGGAGAUGAUGCA	Cleavage
tae-miR1133	Traes_7DS_98F9E4E55.2	PF16211,PF00125	KOG1757			GO:GO:0003677	AT3G54560.1	histone H2A 11	LOC_Os03g53190.2	Core histone H2A/H2B/H3/H4 domain containing protein, putative, expressed	2,00	-1.0	1	22	2	24	CAUAUACUCCCUCCGUCCGA-AA	UUAUGGGACGGAGGGAGUAUAUA	Cleavage
tae-miR1122a	Traes_7DS_CA61ECF35.1						AT1G05060.1		LOC_Os06g13220.1	expressed protein	0,00	-1.0	1	20	33	52	UAGAUACAUCCGUAUCUAGA	UCUAGAUACGGAUGUAUCUA	Cleavage
tae-miR9775	Traes_7DS_D4DFDF981.1										2,00	-1.0	1	22	1609	1630	UGUGCGCAAUAAGAUUUUGCUA	GUGCAUGAUCUUAUUGCGCACA	Cleavage
tae-miR9775	Traes_7DS_D4DFDF981.2										2,00	-1.0	1	22	1702	1723	UGUGCGCAAUAAGAUUUUGCUA	GUGCAUGAUCUUAUUGCGCACA	Cleavage
tae-miR156	Traes_7DS_DBB29C7CD.1								LOC_Os08g41940.1	OsSPL16 - SBP-box gene family member, expressed	1,00	-1.0	1	21	359	379	UGACAGAAGAGAGUGAGCACA	UGUGCUCUCUCUCUUCUGUCA	Cleavage
tae-miR5049-3p	Traes_7DS_EFB144315.5	PF00076,PF04059	KOG4660			GO:GO:0003676	AT1G29400.2	MEI2-like protein 5	LOC_Os02g48790.5	AML1, putative, expressed	1,05	-1.0	1	21	2298	2318	AAUAUGGAUCGGAGGGAGUAC	UUACUCCCUCUGAUCCAUAUU	Translation

Triticum monococcum_Control_Root

miRNA_Acc.	Target_Acc.	Pfam	KOG	KEGG/ec	KO	GO	Best-hit-arabi-name	arabi-defline	Best-hit-rice-name	rice-defline	Expectation	UPE$	miRNA_start	miRNA_end	Target_start	Target_end	miRNA_aligned_fragment	Target_aligned_fragment	Inhibition
tae-miR159a	Traes_1AL_041EBB4A6.1						AT2G32460.1	myb domain protein 101	LOC_Os01g59660.2	MYB family transcription factor, putative, expressed	2,05	-1.0	1	21	488	508	UUUGGAUUGAAGGGAGCUCUG	UGGAGCUCCCUUCAUUCCAAU	Cleavage
tae-miR159b	Traes_1AL_041EBB4A6.1						AT2G32460.1	myb domain protein 101	LOC_Os01g59660.2	MYB family transcription factor, putative, expressed	2,05	-1.0	1	21	488	508	UUUGGAUUGAAGGGAGCUCUG	UGGAGCUCCCUUCAUUCCAAU	Cleavage
tae-miR159a	Traes_1AL_041EBB4A6.2						AT2G32460.1	myb domain protein 101	LOC_Os01g59660.2	MYB family transcription factor, putative, expressed	2,05	-1.0	1	21	512	532	UUUGGAUUGAAGGGAGCUCUG	UGGAGCUCCCUUCAUUCCAAU	Cleavage
tae-miR159b	Traes_1AL_041EBB4A6.2						AT2G32460.1	myb domain protein 101	LOC_Os01g59660.2	MYB family transcription factor, putative, expressed	2,05	-1.0	1	21	512	532	UUUGGAUUGAAGGGAGCUCUG	UGGAGCUCCCUUCAUUCCAAU	Cleavage
tae-miR159a	Traes_1AL_041EBB4A6.3						AT2G32460.1	myb domain protein 101	LOC_Os01g59660.2	MYB family transcription factor, putative, expressed	2,05	-1.0	1	21	488	508	UUUGGAUUGAAGGGAGCUCUG	UGGAGCUCCCUUCAUUCCAAU	Cleavage
tae-miR159b	Traes_1AL_041EBB4A6.3						AT2G32460.1	myb domain protein 101	LOC_Os01g59660.2	MYB family transcription factor, putative, expressed	2,05	-1.0	1	21	488	508	UUUGGAUUGAAGGGAGCUCUG	UGGAGCUCCCUUCAUUCCAAU	Cleavage
tae-miR159a	Traes_1AL_041EBB4A6.4								LOC_Os01g59660.2	MYB family transcription factor, putative, expressed	2,05	-1.0	1	21	410	430	UUUGGAUUGAAGGGAGCUCUG	UGGAGCUCCCUUCAUUCCAAU	Cleavage
tae-miR159b	Traes_1AL_041EBB4A6.4								LOC_Os01g59660.2	MYB family transcription factor, putative, expressed	2,05	-1.0	1	21	410	430	UUUGGAUUGAAGGGAGCUCUG	UGGAGCUCCCUUCAUUCCAAU	Cleavage
tae-miR160	Traes_1AL_147CF243C.1										0,00	-1.0	1	21	642	662	UGCCUGGCUCCCUGUAUGCCA	AGGCAUACAGGGAGCCAGGCA	Cleavage
tae-miR160	Traes_1AL_147CF243C.2										0,00	-1.0	1	21	267	287	UGCCUGGCUCCCUGUAUGCCA	AGGCAUACAGGGAGCCAGGCA	Cleavage
tae-miR1127b-3p	Traes_1AL_4A458DF14.2										1,05	-1.0	1	21	1738	1758	ACAAGUAUUUCUGGACGGAGG	CCUCCGUCCGGAAAUACUUGU	Cleavage
tae-miR1130b-3p	Traes_1AL_51B1D5DBC.6										0,00	-1.0	1	21	914	934	UCUUAUAUUAUGGGACGGAGG	CCUCCGUCCCAUAAUAUAAGA	Cleavage
tae-miR1122c-3p	Traes_1AL_51B1D5DBC.6										1,05	-1.0	1	21	914	934	UCUAAUAUUAUGGGACGGAGG	CCUCCGUCCCAUAAUAUAAGA	Cleavage
tae-miR5049-3p	Traes_1AL_7D6DC73FC.3										2,00	-1.0	1	21	1256	1276	AAUAUGGAUCGGAGGGAGUAC	UUUCUCCCUCCGAUCCAUAUA	Cleavage
tae-miR1128	Traes_1AL_968B97E50.2										3,00	-1.0	1	21	100	121	UACUACUCCCUCCGUCC-GAAA	UUUCCGGACGGAGGGAGUAGUU	Cleavage
tae-miR1134	Traes_1AL_AF222553D.2										2,05	-1.0	1	24	568	591	CAACAACAACAAGAAGAAGAAGAU	CUCUCCUUGUUGUUGUUGUUGUUG	Cleavage
tae-miR7757-5p	Traes_1AL_B6931117F.1	PF00931				GO:GO:0043531	AT3G46530.1	NB-ARC domain-containing disease resistance protein	LOC_Os08g10440.1	NBS-LRR disease resistance protein, putative, expressed	1,05	-1.0	1	22	1301	1322	AUAAAACCUUCAGCUAUCCAUC	AAUGGAUAGCUGAAGGCUUUAU	Cleavage
tae-miR7757-5p	Traes_1AL_B6931117F.2	PF00931				GO:GO:0043531	AT3G46530.1	NB-ARC domain-containing disease resistance protein	LOC_Os08g10440.1	NBS-LRR disease resistance protein, putative, expressed	1,05	-1.0	1	22	1343	1364	AUAAAACCUUCAGCUAUCCAUC	AAUGGAUAGCUGAAGGCUUUAU	Cleavage
tae-miR7757-5p	Traes_1AL_B6931117F.3	PF00931				GO:GO:0043531	AT3G46530.1	NB-ARC domain-containing disease resistance protein	LOC_Os08g10440.1	NBS-LRR disease resistance protein, putative, expressed	1,05	-1.0	1	22	1301	1322	AUAAAACCUUCAGCUAUCCAUC	AAUGGAUAGCUGAAGGCUUUAU	Cleavage
tae-miR7757-5p	Traes_1AL_B6931117F.4	PF00931				GO:GO:0043531	AT3G46530.1	NB-ARC domain-containing disease resistance protein	LOC_Os08g10440.1	NBS-LRR disease resistance protein, putative, expressed	1,05	-1.0	1	22	1301	1322	AUAAAACCUUCAGCUAUCCAUC	AAUGGAUAGCUGAAGGCUUUAU	Cleavage
tae-miR9778	Traes_1AS_17306388F.1										2,00	-1.0	1	21	126	146	UGCAUCAUCUCGAACUCGUCG	CAAUGAGCUCGAGAUGAUGCA	Cleavage
tae-miR9778	Traes_1AS_17306388F.3										2,00	-1.0	1	21	126	146	UGCAUCAUCUCGAACUCGUCG	CAAUGAGCUCGAGAUGAUGCA	Cleavage
tae-miR1135	Traes_1AS_4A925522B.4	PF13920					AT5G01960.1	RING/U-box superfamily protein	LOC_Os01g07390.1	zinc finger, C3HC4 type family protein, expressed	0,00	-1.0	1	24	511	534	CUGCGACAAGUAAUUCCGAACGGA	UCCGUUCGGAAUUACUUGUCGCAG	Cleavage
tae-miR6197-5p	Traes_1AS_5DCFDC258.1										3,00	-1.0	1	21	1353	1376	UCUGUAAACAAAUGUA---GGACG	ACUCCAACUACAUUUGUUUACAGA	Cleavage
tae-miR1137a	Traes_1AS_8912BD052.27										0,00	-1.0	1	20	136	155	UAGUACAAAGUUGAGUCAUC	GAUGACUCAACUUUGUACUA	Cleavage
tae-miR9674a-5p	Traes_1AS_92A698B19.1										2,05	-1.0	1	21	1761	1781	GCAUCAUCCAUCCUACCAUUC	AAAUGGAAGGAUCGAUGAUGC	Cleavage
tae-miR9674a-5p	Traes_1AS_92A698B19.10										2,05	-1.0	1	21	1761	1781	GCAUCAUCCAUCCUACCAUUC	AAAUGGAAGGAUCGAUGAUGC	Cleavage
tae-miR9674a-5p	Traes_1AS_92A698B19.12										2,05	-1.0	1	21	96	116	GCAUCAUCCAUCCUACCAUUC	AAAUGGAAGGAUCGAUGAUGC	Cleavage
tae-miR9674a-5p	Traes_1AS_92A698B19.2										2,05	-1.0	1	21	1761	1781	GCAUCAUCCAUCCUACCAUUC	AAAUGGAAGGAUCGAUGAUGC	Cleavage
tae-miR9674a-5p	Traes_1AS_92A698B19.3										2,05	-1.0	1	21	1761	1781	GCAUCAUCCAUCCUACCAUUC	AAAUGGAAGGAUCGAUGAUGC	Cleavage
tae-miR9674a-5p	Traes_1AS_92A698B19.4										2,05	-1.0	1	21	1761	1781	GCAUCAUCCAUCCUACCAUUC	AAAUGGAAGGAUCGAUGAUGC	Cleavage
tae-miR9674a-5p	Traes_1AS_92A698B19.5										2,05	-1.0	1	21	1761	1781	GCAUCAUCCAUCCUACCAUUC	AAAUGGAAGGAUCGAUGAUGC	Cleavage
tae-miR9674a-5p	Traes_1AS_92A698B19.6										2,05	-1.0	1	21	1761	1781	GCAUCAUCCAUCCUACCAUUC	AAAUGGAAGGAUCGAUGAUGC	Cleavage
tae-miR9674a-5p	Traes_1AS_92A698B19.7										2,05	-1.0	1	21	1761	1781	GCAUCAUCCAUCCUACCAUUC	AAAUGGAAGGAUCGAUGAUGC	Cleavage
tae-miR9674a-5p	Traes_1AS_92A698B19.8										2,05	-1.0	1	21	1761	1781	GCAUCAUCCAUCCUACCAUUC	AAAUGGAAGGAUCGAUGAUGC	Cleavage
tae-miR9674a-5p	Traes_1AS_92A698B19.9										2,05	-1.0	1	21	1761	1781	GCAUCAUCCAUCCUACCAUUC	AAAUGGAAGGAUCGAUGAUGC	Cleavage
tae-miR5049-3p	Traes_1AS_A482D4FF9.1										0,00	-1.0	1	21	557	577	AAUAUGGAUCGGAGGGAGUAC	GUACUCCCUCCGAUCCAUAUU	Cleavage
tae-miR9778	Traes_1AS_C7A8188D1.1										1,00	-1.0	1	21	126	146	UGCAUCAUCUCGAACUCGUCG	CAACGAGCUCGAGAUGAUGCA	Cleavage
tae-miR9778	Traes_1AS_C7A8188D1.3										1,00	-1.0	1	21	126	146	UGCAUCAUCUCGAACUCGUCG	CAACGAGCUCGAGAUGAUGCA	Cleavage
tae-miR9661-5p	Traes_1AS_D8E37233E.1										2,00	-1.0	1	21	101	121	UGAAGUAGAGCAGGGACCUCA	CCAAGUCCCUGCUCUACUUCG	Cleavage
tae-miR1128	Traes_1BL_087CA0963.1										3,00	-1.0	1	21	2048	2069	UACUACUCCCUCCG-UCCGAAA	UUUAGGAACGGAGGGAGUAGUA	Cleavage
tae-miR1128	Traes_1BL_0FDC38712.1										1,05	-1.0	1	21	225	245	UACUACUCCCUCCGUCCGAAA	UUUCGGACGGAGGGAGUACUA	Cleavage
tae-miR1127b-3p	Traes_1BL_0FDC38712.1										1,05	-1.0	1	21	121	141	ACAAGUAUUUCUGGACGGAGG	CCUCCGUCCGGAAAUACUUGU	Cleavage
tae-miR160	Traes_1BL_54CD82AC3.1										0,00	-1.0	1	21	642	662	UGCCUGGCUCCCUGUAUGCCA	AGGCAUACAGGGAGCCAGGCA	Cleavage
tae-miR160	Traes_1BL_54CD82AC3.2										0,00	-1.0	1	21	369	389	UGCCUGGCUCCCUGUAUGCCA	AGGCAUACAGGGAGCCAGGCA	Cleavage
tae-miR160	Traes_1BL_54CD82AC3.3										0,00	-1.0	1	21	267	287	UGCCUGGCUCCCUGUAUGCCA	AGGCAUACAGGGAGCCAGGCA	Cleavage
tae-miR1130b-3p	Traes_1BL_89E785AAA.2										2,00	-1.0	1	21	591	611	UCUUAUAUUAUGGGACGGAGG	CCUCUGUCCCAUAAUAUAAGG	Cleavage
tae-miR159a	Traes_1BL_CF98E922B.1	PF00249					AT5G06100.2	myb domain protein 33	LOC_Os01g59660.2	MYB family transcription factor, putative, expressed	2,05	-1.0	1	21	839	859	UUUGGAUUGAAGGGAGCUCUG	UGGAGCUCCCUUCACUCCAAU	Cleavage
tae-miR159b	Traes_1BL_CF98E922B.1	PF00249					AT5G06100.2	myb domain protein 33	LOC_Os01g59660.2	MYB family transcription factor, putative, expressed	2,05	-1.0	1	21	839	859	UUUGGAUUGAAGGGAGCUCUG	UGGAGCUCCCUUCACUCCAAU	Cleavage
tae-miR159a	Traes_1BL_CF98E922B.2	PF00249					AT5G06100.3	myb domain protein 33	LOC_Os01g59660.1	MYB family transcription factor, putative, expressed	2,05	-1.0	1	21	839	859	UUUGGAUUGAAGGGAGCUCUG	UGGAGCUCCCUUCACUCCAAU	Cleavage
tae-miR159b	Traes_1BL_CF98E922B.2	PF00249					AT5G06100.3	myb domain protein 33	LOC_Os01g59660.1	MYB family transcription factor, putative, expressed	2,05	-1.0	1	21	839	859	UUUGGAUUGAAGGGAGCUCUG	UGGAGCUCCCUUCACUCCAAU	Cleavage
tae-miR159a	Traes_1BL_CF98E922B.3						AT5G06100.3	myb domain protein 33	LOC_Os01g59660.2	MYB family transcription factor, putative, expressed	2,05	-1.0	1	21	512	532	UUUGGAUUGAAGGGAGCUCUG	UGGAGCUCCCUUCACUCCAAU	Cleavage
tae-miR159b	Traes_1BL_CF98E922B.3						AT5G06100.3	myb domain protein 33	LOC_Os01g59660.2	MYB family transcription factor, putative, expressed	2,05	-1.0	1	21	512	532	UUUGGAUUGAAGGGAGCUCUG	UGGAGCUCCCUUCACUCCAAU	Cleavage
tae-miR159a	Traes_1BL_CF98E922B.4						AT5G06100.1	myb domain protein 33	LOC_Os01g59660.1	MYB family transcription factor, putative, expressed	2,05	-1.0	1	21	512	532	UUUGGAUUGAAGGGAGCUCUG	UGGAGCUCCCUUCACUCCAAU	Cleavage
tae-miR159b	Traes_1BL_CF98E922B.4						AT5G06100.1	myb domain protein 33	LOC_Os01g59660.1	MYB family transcription factor, putative, expressed	2,05	-1.0	1	21	512	532	UUUGGAUUGAAGGGAGCUCUG	UGGAGCUCCCUUCACUCCAAU	Cleavage
tae-miR1117	Traes_1BL_F01A87F44.1										2,05	-1.0	1	24	37	60	UAGUACCGGUUCGUGGCACGAACC	GGUUCGUGGCACGAACCGGUGCUA	Cleavage
tae-miR1131	Traes_1BL_F01A87F44.1										2,05	-1.0	1	22	63	84	UAGUACCGGUUCGUGGCUAACC	GGUUAGCCACGAACCGGUAAUG	Cleavage
tae-miR1122b-3p	Traes_1BS_34C60A54E.1										1,05	-1.0	1	21	47	67	AGACUUAUAUGUAGGAACGGA	UCCGUUCCUAAAUAUAAGUCU	Translation
tae-miR9662a-3p	Traes_1BS_566CEE7F6.1	PF02536				GO:GO:0006355,GO:GO:0005739,GO:GO:0003690	AT1G21150.1	Mitochondrial transcription termination factor family protein	LOC_Os06g12100.1	mTERF family protein, expressed	2,00	-1.0	1	21	441	461	UUGAACAUCCCAGAGCCACCG	UCGUGGCGCUGGGAUGUUCAG	Cleavage
tae-miR9662b-3p	Traes_1BS_566CEE7F6.1	PF02536				GO:GO:0006355,GO:GO:0005739,GO:GO:0003690	AT1G21150.1	Mitochondrial transcription termination factor family protein	LOC_Os06g12100.1	mTERF family protein, expressed	2,05	-1.0	1	21	440	460	UGAACAUCCCAGAGCCACCGG	CUCGUGGCGCUGGGAUGUUCA	Cleavage
tae-miR9662a-3p	Traes_1BS_566CEE7F6.2	PF02536				GO:GO:0006355,GO:GO:0005739,GO:GO:0003690	AT1G21150.1	Mitochondrial transcription termination factor family protein	LOC_Os06g12100.1	mTERF family protein, expressed	2,00	-1.0	1	21	66	86	UUGAACAUCCCAGAGCCACCG	UCGUGGCGCUGGGAUGUUCAG	Cleavage
tae-miR9662b-3p	Traes_1BS_566CEE7F6.2	PF02536				GO:GO:0006355,GO:GO:0005739,GO:GO:0003690	AT1G21150.1	Mitochondrial transcription termination factor family protein	LOC_Os06g12100.1	mTERF family protein, expressed	2,05	-1.0	1	21	65	85	UGAACAUCCCAGAGCCACCGG	CUCGUGGCGCUGGGAUGUUCA	Cleavage
tae-miR9778	Traes_1BS_70B733D34.1										2,00	-1.0	1	21	126	146	UGCAUCAUCUCGAACUCGUCG	CAAUGAGCUCGAGAUGAUGCA	Cleavage
tae-miR9778	Traes_1BS_70B733D34.2										2,00	-1.0	1	21	126	146	UGCAUCAUCUCGAACUCGUCG	CAAUGAGCUCGAGAUGAUGCA	Cleavage
tae-miR5049-3p	Traes_1BS_7EC2BDE95.1										1,05	-1.0	1	21	3430	3450	AAUAUGGAUCGGAGGGAGUAC	CUACUCCCUCCAAUCCAUAUU	Translation
tae-miR5049-3p	Traes_1BS_B0BB95B97.6										0,00	-1.0	1	21	623	643	AAUAUGGAUCGGAGGGAGUAC	GUACUCCCUCCGAUCCAUAUU	Cleavage
tae-miR7757-5p	Traes_1BS_CF1C4F54E.2	PF00931				GO:GO:0043531	AT1G09920.1	TRAF-type zinc finger-related	LOC_Os11g45750.1	WRKY125, expressed	2,05	-1.0	1	22	119	140	AUAAAACCUUCAGCUAUCCAUC	AAUGGUAGCUGAAGGUUUCAU	Cleavage
tae-miR159a	Traes_1DL_25540DE53.1						AT2G32460.1	myb domain protein 101	LOC_Os01g59660.2	MYB family transcription factor, putative, expressed	2,05	-1.0	1	21	512	532	UUUGGAUUGAAGGGAGCUCUG	UGGAGCUCCCUUCAUUCCAAU	Cleavage
tae-miR159b	Traes_1DL_25540DE53.1						AT2G32460.1	myb domain protein 101	LOC_Os01g59660.2	MYB family transcription factor, putative, expressed	2,05	-1.0	1	21	512	532	UUUGGAUUGAAGGGAGCUCUG	UGGAGCUCCCUUCAUUCCAAU	Cleavage
tae-miR159a	Traes_1DL_25540DE53.2						AT2G32460.1	myb domain protein 101	LOC_Os01g59660.1	MYB family transcription factor, putative, expressed	2,05	-1.0	1	21	512	532	UUUGGAUUGAAGGGAGCUCUG	UGGAGCUCCCUUCAUUCCAAU	Cleavage
tae-miR159b	Traes_1DL_25540DE53.2						AT2G32460.1	myb domain protein 101	LOC_Os01g59660.1	MYB family transcription factor, putative, expressed	2,05	-1.0	1	21	512	532	UUUGGAUUGAAGGGAGCUCUG	UGGAGCUCCCUUCAUUCCAAU	Cleavage
tae-miR159a	Traes_1DL_25540DE53.3						AT5G06100.3	myb domain protein 33	LOC_Os01g59660.1	MYB family transcription factor, putative, expressed	2,05	-1.0	1	21	470	490	UUUGGAUUGAAGGGAGCUCUG	UGGAGCUCCCUUCAUUCCAAU	Cleavage
tae-miR159b	Traes_1DL_25540DE53.3						AT5G06100.3	myb domain protein 33	LOC_Os01g59660.1	MYB family transcription factor, putative, expressed	2,05	-1.0	1	21	470	490	UUUGGAUUGAAGGGAGCUCUG	UGGAGCUCCCUUCAUUCCAAU	Cleavage
tae-miR159a	Traes_1DL_25540DE53.4						AT5G06100.3	myb domain protein 33	LOC_Os01g59660.2	MYB family transcription factor, putative, expressed	2,05	-1.0	1	21	470	490	UUUGGAUUGAAGGGAGCUCUG	UGGAGCUCCCUUCAUUCCAAU	Cleavage
tae-miR159b	Traes_1DL_25540DE53.4						AT5G06100.3	myb domain protein 33	LOC_Os01g59660.2	MYB family transcription factor, putative, expressed	2,05	-1.0	1	21	470	490	UUUGGAUUGAAGGGAGCUCUG	UGGAGCUCCCUUCAUUCCAAU	Cleavage
tae-miR159a	Traes_1DL_25540DE53.5						AT5G06100.2	myb domain protein 33	LOC_Os01g59660.2	MYB family transcription factor, putative, expressed	2,05	-1.0	1	21	470	490	UUUGGAUUGAAGGGAGCUCUG	UGGAGCUCCCUUCAUUCCAAU	Cleavage
tae-miR159b	Traes_1DL_25540DE53.5						AT5G06100.2	myb domain protein 33	LOC_Os01g59660.2	MYB family transcription factor, putative, expressed	2,05	-1.0	1	21	470	490	UUUGGAUUGAAGGGAGCUCUG	UGGAGCUCCCUUCAUUCCAAU	Cleavage
tae-miR159a	Traes_1DL_25540DE53.6						AT5G06100.2	myb domain protein 33	LOC_Os01g59660.1	MYB family transcription factor, putative, expressed	2,05	-1.0	1	21	470	490	UUUGGAUUGAAGGGAGCUCUG	UGGAGCUCCCUUCAUUCCAAU	Cleavage
tae-miR159b	Traes_1DL_25540DE53.6						AT5G06100.2	myb domain protein 33	LOC_Os01g59660.1	MYB family transcription factor, putative, expressed	2,05	-1.0	1	21	470	490	UUUGGAUUGAAGGGAGCUCUG	UGGAGCUCCCUUCAUUCCAAU	Cleavage
tae-miR1137a	Traes_1DL_34A359364.2										2,05	-1.0	1	20	516	535	UAGUACAAAGUUGAGUCAUC	GAUGACCCAAUUUUGUACUA	Translation
tae-miR1122a	Traes_1DL_38B8B249C.2										0,00	-1.0	1	20	976	995	UAGAUACAUCCGUAUCUAGA	UCUAGAUACGGAUGUAUCUA	Cleavage
tae-miR1120a	Traes_1DL_6A91BC5A5.1										2,05	-1.0	1	24	25	48	ACAUUCUUAUAUUAUGAGACGGAG	CUCCGUCCCAUAAUAUAAGAACGU	Cleavage
tae-miR1120b-3p	Traes_1DL_6A91BC5A5.1										2,05	-1.0	1	21	25	45	UUCUUAUAUUGUGGGACAGAG	CUCCGUCCCAUAAUAUAAGAA	Translation
tae-miR1130b-3p	Traes_1DL_6A91BC5A5.1										0,00	-1.0	1	21	24	44	UCUUAUAUUAUGGGACGGAGG	CCUCCGUCCCAUAAUAUAAGA	Cleavage
tae-miR1122c-3p	Traes_1DL_6A91BC5A5.1										1,05	-1.0	1	21	24	44	UCUAAUAUUAUGGGACGGAGG	CCUCCGUCCCAUAAUAUAAGA	Cleavage
tae-miR1120c-5p	Traes_1DL_6A91BC5A5.1										2,05	-1.0	1	21	67	87	UAAUAUAAGAACGUUUUUGAC	GUCAAACACGCUCUUAUAUUA	Translation
tae-miR160	Traes_1DL_AEC2F3FB3.1										0,00	-1.0	1	21	642	662	UGCCUGGCUCCCUGUAUGCCA	AGGCAUACAGGGAGCCAGGCA	Cleavage
tae-miR1122b-3p	Traes_1DL_B5E6E2738.1										1,05	-1.0	1	21	35	55	AGACUUAUAUGUAGGAACGGA	UCCGUUCCUAAAUAUAAGUCU	Translation
tae-miR5175-5p	Traes_1DL_C1BE1742F.2										2,00	-1.0	1	21	109	130	UUCCAAAUUACUCGUCG-UGGU	UUCAGCGACGAGUAAUUUGGAA	Cleavage
tae-miR1133	Traes_1DL_D391AE3DC.1										2,00	-1.0	1	22	858	879	CAUAUACUCCCUCCGUCCGAAA	AAAUGAACGGAGGGAGUAUAUG	Cleavage
tae-miR1133	Traes_1DL_D391AE3DC.2										2,00	-1.0	1	22	858	879	CAUAUACUCCCUCCGUCCGAAA	AAAUGAACGGAGGGAGUAUAUG	Cleavage
tae-miR1133	Traes_1DL_D391AE3DC.3										2,00	-1.0	1	22	822	843	CAUAUACUCCCUCCGUCCGAAA	AAAUGAACGGAGGGAGUAUAUG	Cleavage
tae-miR1128	Traes_1DL_E30609261.7										3,00	-1.0	1	21	76	96	UACUACUCCCUCCGUCCGAAA	UUCCAGACGGAGGGAGUAGUU	Cleavage
tae-miR1122a	Traes_1DS_06B8348E0.1										0,00	-1.0	1	20	794	813	UAGAUACAUCCGUAUCUAGA	UCUAGAUACGGAUGUAUCUA	Cleavage
tae-miR1122a	Traes_1DS_06B8348E0.2										0,00	-1.0	1	20	620	639	UAGAUACAUCCGUAUCUAGA	UCUAGAUACGGAUGUAUCUA	Cleavage
tae-miR5048-5p	Traes_1DS_26F1546F6.1	PF00168,PF07714,PF00069	KOG1187	2.7.11.1		GO:GO:0005515,GO:GO:0006468,GO:GO:0004672,GO:GO:0005524	AT1G63430.1	Leucine-rich repeat protein kinase family protein	LOC_Os04g58700.1	expressed protein	2,05	-1.0	1	22	917	938	UUUGCAGGUUUUAGGUCUAAGU	ACUUGGACCUAAAACCUGCCAA	Cleavage
tae-miR5048-5p	Traes_1DS_26F1546F6.2	PF00168,PF07714,PF00069	KOG1187	2.7.11.1		GO:GO:0005515,GO:GO:0006468,GO:GO:0004672,GO:GO:0005524	AT1G63430.1	Leucine-rich repeat protein kinase family protein	LOC_Os04g58700.1	expressed protein	2,05	-1.0	1	22	917	938	UUUGCAGGUUUUAGGUCUAAGU	ACUUGGACCUAAAACCUGCCAA	Cleavage
tae-miR7757-5p	Traes_1DS_35EF41CCB.2	PF00931				GO:GO:0043531	AT3G50950.1	HOPZ-ACTIVATED RESISTANCE 1	LOC_Os05g40150.1	RGH2B, putative, expressed	1,05	-1.0	1	22	587	608	AUAAAACCUUCAGCUAUCCAUC	AGUGGAUAGCUGAAGGUUUCAU	Cleavage
tae-miR9674a-5p	Traes_1DS_F3DE3946F.1										2,05	-1.0	1	21	522	542	GCAUCAUCCAUCCUACCAUUC	AAAUGGAAGGAUCGAUGAUGC	Cleavage
tae-miR5049-3p	Traes_1DS_FA7FC5C3C.1										0,00	-1.0	1	21	623	643	AAUAUGGAUCGGAGGGAGUAC	GUACUCCCUCCGAUCCAUAUU	Cleavage
tae-miR1128	Traes_2AL_24A1BF00C.1										0,00	-1.0	1	21	37	57	UACUACUCCCUCCGUCCGAAA	UUUCGGACGGAGGGAGUAGUA	Cleavage
tae-miR5049-3p	Traes_2AL_28B13CC56.1										1,05	-1.0	1	21	346	366	AAUAUGGAUCGGAGGGAGUAC	CUACUCCCUCCGUUCCAUAUU	Cleavage
tae-miR1127a	Traes_2AL_4197D219B.1										2,05	-1.0	1	19	991	1009	UCCUUCCGUUCGGAAUUAC	GUAAUUCCGAACGGAGGGU	Cleavage
tae-miR164	Traes_2AL_578F37A86.1										1,05	-1.0	1	21	680	700	UGGAGAAGCAGGGCACGUGCA	CGCACGUGACCUGCUUCUCCA	Cleavage
tae-miR164	Traes_2AL_578F37A86.2										1,05	-1.0	1	21	606	626	UGGAGAAGCAGGGCACGUGCA	CGCACGUGACCUGCUUCUCCA	Cleavage
tae-miR1122a	Traes_2AL_64B73E3ED.1										1,00	-1.0	1	20	121	140	UAGAUACAUCCGUAUCUAGA	UCUAAAUACGGAUGUAUCUA	Cleavage
tae-miR1120b-3p	Traes_2AL_673CA8FB2.1										2,05	-1.0	1	21	493	513	UUCUUAUAUUGUGGGACAGAG	CUCUGUCCCACAAUGUAAGAC	Cleavage
tae-miR1133	Traes_2AL_673CA8FB2.1										2,05	-1.0	1	22	553	575	CAUAUACUCCCUCCGUCCGA-AA	UUGUGGGACGGAGGGAGUAUUUG	Cleavage
tae-miR1120a	Traes_2AL_979E96822.1										2,05	-1.0	1	24	753	776	ACAUUCUUAUAUUAUGAGACGGAG	CUCCAUCCCAUAAUAUAAGAGUGU	Cleavage
tae-miR1130b-3p	Traes_2AL_979E96822.1										1,00	-1.0	1	21	752	772	UCUUAUAUUAUGGGACGGAGG	CCUCCAUCCCAUAAUAUAAGA	Cleavage
tae-miR1122c-3p	Traes_2AL_979E96822.1										1,00	-1.0	1	21	752	772	UCUAAUAUUAUGGGACGGAGG	CCUCCAUCCCAUAAUAUAAGA	Cleavage
tae-miR1120c-5p	Traes_2AL_979E96822.1										2,05	-1.0	1	21	795	815	UAAUAUAAGAACGUUUUUGAC	GUAAAAAACGCUCUUAUAUUA	Translation
tae-miR160	Traes_2AL_C70217249.1										1,00	-1.0	1	21	1164	1184	UGCCUGGCUCCCUGUAUGCCA	UGGCAUGCAGGGAGCCAGGCA	Cleavage
tae-miR1134	Traes_2AL_D2CC35E59.1										3,00	-1.0	1	24	512	535	CAACAACAACAAGAAGAAGAAGAU	AUCUUCAUGGUCUUGUUGUUGUUG	Cleavage
tae-miR1134	Traes_2AL_D2CC35E59.2										3,00	-1.0	1	24	620	643	CAACAACAACAAGAAGAAGAAGAU	AUCUUCAUGGUCUUGUUGUUGUUG	Cleavage
tae-miR1134	Traes_2AL_D2CC35E59.3										3,00	-1.0	1	24	509	532	CAACAACAACAAGAAGAAGAAGAU	AUCUUCAUGGUCUUGUUGUUGUUG	Cleavage
tae-miR1134	Traes_2AL_D2CC35E59.4										3,00	-1.0	1	24	611	634	CAACAACAACAAGAAGAAGAAGAU	AUCUUCAUGGUCUUGUUGUUGUUG	Cleavage
tae-miR1134	Traes_2AL_D2CC35E59.5										3,00	-1.0	1	24	509	532	CAACAACAACAAGAAGAAGAAGAU	AUCUUCAUGGUCUUGUUGUUGUUG	Cleavage
tae-miR1134	Traes_2AL_D2CC35E59.6										3,00	-1.0	1	24	509	532	CAACAACAACAAGAAGAAGAAGAU	AUCUUCAUGGUCUUGUUGUUGUUG	Cleavage
tae-miR5384-3p	Traes_2AL_D79C9535C.1										2,05	-1.0	1	21	1250	1270	UGAGCGCGCCGCCGUCGAAUG	CGUUCGGCGGCGGCGCGCGCA	Cleavage
tae-miR9778	Traes_2AS_530CF4042.10										2,05	-1.0	1	21	126	146	UGCAUCAUCUCGAACUCGUCG	CGGCGAGUUCCAGAUGAUGCA	Translation
tae-miR9778	Traes_2AS_530CF4042.2										2,05	-1.0	1	21	126	146	UGCAUCAUCUCGAACUCGUCG	CGGCGAGUUCCAGAUGAUGCA	Translation
tae-miR9778	Traes_2AS_530CF4042.3										2,05	-1.0	1	21	126	146	UGCAUCAUCUCGAACUCGUCG	CGGCGAGUUCCAGAUGAUGCA	Translation
tae-miR1130b-3p	Traes_2AS_6DCA4AADD.1										1,05	-1.0	1	21	288	308	UCUUAUAUUAUGGGACGGAGG	CCUCCGUCCCAUAAUAUAGGA	Cleavage
tae-miR9664-3p	Traes_2AS_86633353C.1	PF00931	KOG4658		K13457	GO:GO:0043531	AT3G07040.1	NB-ARC domain-containing disease resistance protein	LOC_Os11g46210.1	NB-ARC domain containing protein, expressed	2,05	-1.0	1	21	240	260	UUGCAGUCCUCGAUGUCGUAG	CUACGACAUCGAGGAUUGCAU	Cleavage
tae-miR1130b-3p	Traes_2BL_171777733.1										0,00	-1.0	1	21	752	772	UCUUAUAUUAUGGGACGGAGG	CCUCCGUCCCAUAAUAUAAGA	Cleavage
tae-miR1122c-3p	Traes_2BL_171777733.1										1,05	-1.0	1	21	752	772	UCUAAUAUUAUGGGACGGAGG	CCUCCGUCCCAUAAUAUAAGA	Cleavage
tae-miR1120c-5p	Traes_2BL_171777733.1										2,05	-1.0	1	21	795	815	UAAUAUAAGAACGUUUUUGAC	GUAAAAAACGCUCUUAUAUUA	Translation
tae-miR5384-3p	Traes_2BL_1BE5C9C71.1										2,05	-1.0	1	21	455	475	UGAGCGCGCCGCCGUCGAAUG	CGUUCGGCGGCGGCGCGCGCA	Cleavage
tae-miR5384-3p	Traes_2BL_1BE5C9C71.2										2,05	-1.0	1	21	455	475	UGAGCGCGCCGCCGUCGAAUG	CGUUCGGCGGCGGCGCGCGCA	Cleavage
tae-miR1122a	Traes_2BL_40442CE82.2										1,05	-1.0	1	20	703	722	UAGAUACAUCCGUAUCUAGA	UCUAGAUACAGAUGUAUCUA	Translation
tae-miR164	Traes_2BL_6AEE8AC28.1										1,05	-1.0	1	21	680	700	UGGAGAAGCAGGGCACGUGCA	CGCACGUGACCUGCUUCUCCA	Cleavage
tae-miR164	Traes_2BL_6AEE8AC28.2										1,05	-1.0	1	21	672	692	UGGAGAAGCAGGGCACGUGCA	CGCACGUGACCUGCUUCUCCA	Cleavage
tae-miR164	Traes_2BL_6AEE8AC28.3										1,05	-1.0	1	21	633	653	UGGAGAAGCAGGGCACGUGCA	CGCACGUGACCUGCUUCUCCA	Cleavage
tae-miR1122b-3p	Traes_2BL_7141904F2.2										2,05	-1.0	1	21	1058	1078	AGACUUAUAUGUAGGAACGGA	UCCGUUCCUAAAUAUAAGUCU	Translation
tae-miR1122a	Traes_2BL_7141904F2.2										2,05	-1.0	1	20	1098	1117	UAGAUACAUCCGUAUCUAGA	ACUAGAUACGGAUGUAUAUA	Cleavage
tae-miR1134	Traes_2BL_8339D35AC.1										3,00	-1.0	1	24	509	532	CAACAACAACAAGAAGAAGAAGAU	AUCUUCAUGGUCUUGUUGUUGUUG	Cleavage
tae-miR1134	Traes_2BL_8339D35AC.2										3,00	-1.0	1	24	512	535	CAACAACAACAAGAAGAAGAAGAU	AUCUUCAUGGUCUUGUUGUUGUUG	Cleavage
tae-miR1134	Traes_2BL_8339D35AC.3										3,00	-1.0	1	24	509	532	CAACAACAACAAGAAGAAGAAGAU	AUCUUCAUGGUCUUGUUGUUGUUG	Cleavage
tae-miR1134	Traes_2BL_8339D35AC.4										3,00	-1.0	1	24	302	325	CAACAACAACAAGAAGAAGAAGAU	AUCUUCAUGGUCUUGUUGUUGUUG	Cleavage
tae-miR1134	Traes_2BL_8339D35AC.5										3,00	-1.0	1	24	509	532	CAACAACAACAAGAAGAAGAAGAU	AUCUUCAUGGUCUUGUUGUUGUUG	Cleavage
tae-miR1135	Traes_2BL_A4AE931B0.1						AT2G02090.1	SNF2 domain-containing protein / helicase domain-containing protein	LOC_Os04g47830.1	SNF2 family N-terminal domain containing protein, expressed	2,00	-1.0	1	24	1154	1177	CUGCGACAAGUAAUUCCGAACGGA	UCUGUCCGGAAUUACUUGUCGCAA	Cleavage
tae-miR1122b-3p	Traes_2BL_FF8002798.2										2,05	-1.0	1	21	889	909	AGACUUAUAUGUAGGAACGGA	UCGGUUCCUAAAUAUAAGUCU	Translation
tae-miR9778	Traes_2BS_0EFFAAE28.1										2,05	-1.0	1	21	126	146	UGCAUCAUCUCGAACUCGUCG	CGGCGAGUUCCAGAUGAUGCA	Translation
tae-miR9778	Traes_2BS_0EFFAAE28.2										2,05	-1.0	1	21	126	146	UGCAUCAUCUCGAACUCGUCG	CGGCGAGUUCCAGAUGAUGCA	Translation
tae-miR9662a-3p	Traes_2BS_257C5B099.1	PF02536				GO:GO:0006355,GO:GO:0005739,GO:GO:0003690	AT5G07900.1	Mitochondrial transcription termination factor family protein	LOC_Os06g12100.1	mTERF family protein, expressed	2,00	-1.0	1	21	66	86	UUGAACAUCCCAGAGCCACCG	CCGUGGUUCUGGGAUGUUCAG	Cleavage
tae-miR9662b-3p	Traes_2BS_257C5B099.1	PF02536				GO:GO:0006355,GO:GO:0005739,GO:GO:0003690	AT5G07900.1	Mitochondrial transcription termination factor family protein	LOC_Os06g12100.1	mTERF family protein, expressed	2,00	-1.0	1	21	65	85	UGAACAUCCCAGAGCCACCGG	CCCGUGGUUCUGGGAUGUUCA	Cleavage
tae-miR9662a-3p	Traes_2BS_257C5B0991.1	PF02536				GO:GO:0006355,GO:GO:0005739,GO:GO:0003690	AT5G07900.1	Mitochondrial transcription termination factor family protein	LOC_Os06g12100.1	mTERF family protein, expressed	2,00	-1.0	1	21	66	86	UUGAACAUCCCAGAGCCACCG	CCGUGGUUCUGGGAUGUUCAG	Cleavage

tae-miR9662b-3p	Traes_2BS_257C5B0991.1	PF02536				GO:GO:0006355,GO:GO:0005739,GO:GO:0003690	AT5G07900.1	Mitochondrial transcription termination factor family protein	LOC_Os06g12100.1	mTERF family protein, expressed	2,00	-1.0	1	21	65	85	UGAACAUCCCAGAGCCACCGG	CCCGUGGUUCUGGGAUGUUCA	Cleavage
tae-miR5048-5p	Traes_2BS_47E2D6444.1	PF07714		2.7.11.1		GO:GO:0006468,GO:GO:0004672	AT4G04570.2	cysteine-rich RLK (RECEPTOR-like protein kinase) 40	LOC_Os04g30030.1	cysteine-rich receptor-like protein kinase 12 precursor, putative, expressed	2,05	-1.0	1	22	221	242	UUUGCAGGUUUUAGGUCUAAGU	ACCUGGACCUAAAACCUGCGAA	Cleavage
tae-miR5048-5p	Traes_2BS_47E2D6444.2	PF00069	KOG1187	2.7.11.1		GO:GO:0006468,GO:GO:0005524,GO:GO:0004672	AT4G38830.1	cysteine-rich RLK (RECEPTOR-like protein kinase) 26	LOC_Os04g30030.1	cysteine-rich receptor-like protein kinase 12 precursor, putative, expressed	2,05	-1.0	1	22	959	980	UUUGCAGGUUUUAGGUCUAAGU	ACCUGGACCUAAAACCUGCGAA	Cleavage
tae-miR5048-5p	Traes_2BS_47E2D6444.3	PF00069	KOG1187	2.7.11.1		GO:GO:0006468,GO:GO:0005524,GO:GO:0004672	AT4G38830.1	cysteine-rich RLK (RECEPTOR-like protein kinase) 26	LOC_Os04g30030.1	cysteine-rich receptor-like protein kinase 12 precursor, putative, expressed	2,05	-1.0	1	22	947	968	UUUGCAGGUUUUAGGUCUAAGU	ACCUGGACCUAAAACCUGCGAA	Cleavage
tae-miR5048-5p	Traes_2BS_47E2D6444.4	PF07714		2.7.11.1		GO:GO:0006468,GO:GO:0004672	AT4G23160.1	cysteine-rich RLK (RECEPTOR-like protein kinase) 8	LOC_Os04g30030.1	cysteine-rich receptor-like protein kinase 12 precursor, putative, expressed	2,05	-1.0	1	22	221	242	UUUGCAGGUUUUAGGUCUAAGU	ACCUGGACCUAAAACCUGCGAA	Cleavage
tae-miR9664-3p	Traes_2BS_5045C640C.1	PF00931	KOG4658		K13457	GO:GO:0043531	AT3G07040.1	NB-ARC domain-containing disease resistance protein	LOC_Os11g46210.1	NB-ARC domain containing protein, expressed	2,05	-1.0	1	21	240	260	UUGCAGUCCUCGAUGUCGUAG	CUACGACAUCGAGGAUUGCAU	Cleavage
tae-miR9664-3p	Traes_2BS_5045C640C.2	PF00931	KOG4658		K13457	GO:GO:0043531	AT3G07040.1	NB-ARC domain-containing disease resistance protein	LOC_Os11g46210.1	NB-ARC domain containing protein, expressed	2,05	-1.0	1	21	240	260	UUGCAGUCCUCGAUGUCGUAG	CUACGACAUCGAGGAUUGCAU	Cleavage
tae-miR9664-3p	Traes_2BS_5045C640C.5	PF00931	KOG4658		K13457	GO:GO:0043531	AT3G07040.1	NB-ARC domain-containing disease resistance protein	LOC_Os11g46210.1	NB-ARC domain containing protein, expressed	2,05	-1.0	1	21	240	260	UUGCAGUCCUCGAUGUCGUAG	CUACGACAUCGAGGAUUGCAU	Cleavage
tae-miR1120c-5p	Traes_2BS_5C64FC44A.1										0,00	-1.0	1	21	21	41	UAAUAUAAGAACGUUUUUGAC	GUCAAAAACGUUCUUAUAUUA	Cleavage
tae-miR1120c-5p	Traes_2BS_5C64FC44A.2										0,00	-1.0	1	21	2	22	UAAUAUAAGAACGUUUUUGAC	GUCAAAAACGUUCUUAUAUUA	Cleavage
tae-miR1130b-3p	Traes_2BS_5CDCD5152.1										2,00	-1.0	1	20	1	21	UCUUAUAUUAUGGGA-CGGAG	GUCCGAUCCCAUAAUAUAAGA	Cleavage
tae-miR1121	Traes_2BS_6249E432F.1										0,00	-1.0	1	22	538	559	AGUAGUGAUCUAAACGCUCUUA	UAAGAGCGUUUAGAUCACUACU	Cleavage
tae-miR1121	Traes_2BS_6249E432F.2										0,00	-1.0	1	22	538	559	AGUAGUGAUCUAAACGCUCUUA	UAAGAGCGUUUAGAUCACUACU	Cleavage
tae-miR1121	Traes_2BS_6249E432F.3										0,00	-1.0	1	22	448	469	AGUAGUGAUCUAAACGCUCUUA	UAAGAGCGUUUAGAUCACUACU	Cleavage
tae-miR1133	Traes_2BS_67D862F4A.1										2,05	-1.0	1	22	30	51	CAUAUACUCCCUCCGUCCGAAA	UUUGGGACGGAGGGAGUAUAAG	Cleavage
tae-miR9662a-3p	Traes_2BS_74269A4C5.1	PF02536				GO:GO:0006355,GO:GO:0005739,GO:GO:0003690	AT5G07900.1	Mitochondrial transcription termination factor family protein	LOC_Os06g12100.1	mTERF family protein, expressed	2,00	-1.0	1	21	189	209	UUGAACAUCCCAGAGCCACCG	CCGUGGUUCUGGGAUGUUCAG	Cleavage
tae-miR9662b-3p	Traes_2BS_74269A4C5.1	PF02536				GO:GO:0006355,GO:GO:0005739,GO:GO:0003690	AT5G07900.1	Mitochondrial transcription termination factor family protein	LOC_Os06g12100.1	mTERF family protein, expressed	2,00	-1.0	1	21	188	208	UGAACAUCCCAGAGCCACCGG	CCCGUGGUUCUGGGAUGUUCA	Cleavage
tae-miR1128	Traes_2BS_7AEB138EB.1										1,05	-1.0	1	21	84	104	UACUACUCCCUCCGUCCGAAA	UUUCGGACGGAGGGAGUACUA	Cleavage
tae-miR9661-5p	Traes_2BS_8281050F1.1										3,00	-1.0	1	21	845	865	UGAAGUAGAGCAGGGACCUCA	GACCGUUCCUGCUCUACUUCA	Cleavage
tae-miR9661-5p	Traes_2BS_8281050F11.1										3,00	-1.0	1	21	845	865	UGAAGUAGAGCAGGGACCUCA	GACCGUUCCUGCUCUACUUCA	Cleavage
tae-miR1122b-3p	Traes_2BS_A20CCBEC3.1										2,05	-1.0	1	21	389	409	AGACUUAUAUGUAGGAACGGA	UCUGUUCCUAAAUAUAAGUCU	Translation
tae-miR1128	Traes_2BS_B3A88F254.2										2,00	-1.0	1	21	88	109	UACUACUCCCUCCGUCC-GAAA	UUUCCGGACGGAGGGAGUAGUA	Cleavage
tae-miR1128	Traes_2BS_B3A88F254.3										2,00	-1.0	1	21	88	109	UACUACUCCCUCCGUCC-GAAA	UUUCCGGACGGAGGGAGUAGUA	Cleavage
tae-miR1127a	Traes_2BS_C00FC92D4.2										1,05	-1.0	1	19	497	515	UCCUUCCGUUCGGAAUUAC	GUAAUUCCGAACGGAGGGA	Cleavage
tae-miR1117	Traes_2BS_C5AF5419E.1										0,00	-1.0	1	24	926	949	UAGUACCGGUUCGUGGCACGAACC	GGUUCGUGCCACGAACCGGUACUA	Cleavage
tae-miR9662a-3p	Traes_2BS_DDF26C784.1	PF02536				GO:GO:0006355,GO:GO:0005739,GO:GO:0003690	AT5G07900.1	Mitochondrial transcription termination factor family protein	LOC_Os06g12100.1	mTERF family protein, expressed	2,00	-1.0	1	21	459	479	UUGAACAUCCCAGAGCCACCG	CCGUGGUUCUGGGAUGUUCAG	Cleavage
tae-miR9662b-3p	Traes_2BS_DDF26C784.1	PF02536				GO:GO:0006355,GO:GO:0005739,GO:GO:0003690	AT5G07900.1	Mitochondrial transcription termination factor family protein	LOC_Os06g12100.1	mTERF family protein, expressed	2,00	-1.0	1	21	458	478	UGAACAUCCCAGAGCCACCGG	CCCGUGGUUCUGGGAUGUUCA	Cleavage
tae-miR5384-3p	Traes_2BS_EBCD10293.1										2,05	-1.0	1	21	116	136	UGAGCGCGCCGCCGUCGAAUG	UGUUCGCCGACGGCGCGCUCA	Cleavage
tae-miR5384-3p	Traes_2BS_EBCD10293.2										2,05	-1.0	1	21	116	136	UGAGCGCGCCGCCGUCGAAUG	UGUUCGCCGACGGCGCGCUCA	Cleavage
tae-miR1130b-3p	Traes_2DL_10B2965EF.8										0,00	-1.0	1	21	752	772	UCUUAUAUUAUGGGACGGAGG	CCUCCGUCCCAUAAUAUAAGA	Cleavage
tae-miR1122c-3p	Traes_2DL_10B2965EF.8										1,05	-1.0	1	21	752	772	UCUAAUAUUAUGGGACGGAGG	CCUCCGUCCCAUAAUAUAAGA	Cleavage
tae-miR1120c-5p	Traes_2DL_10B2965EF.8										1,05	-1.0	1	21	795	815	UAAUAUAAGAACGUUUUUGAC	GUCAAAAACGCUCUUAUAUUA	Translation
tae-miR1130b-3p	Traes_2DL_3C9129432.1										1,05	-1.0	1	21	164	184	UCUUAUAUUAUGGGACGGAGG	CUUCCGUCCCAUAAUGUAAGA	Cleavage
tae-miR1133	Traes_2DL_4481C0380.4										2,05	-1.0	1	22	1	22	CAUAUACUCCCUCCGUCCGAAA	UUUGGGACGGAGGGAGUACAUG	Cleavage
tae-miR1127b-3p	Traes_2DL_4F8C87C42.5										1,05	-1.0	1	21	1822	1842	ACAAGUAUUUCUGGACGGAGG	CCUCCGUCCGGAAAUACUUGU	Cleavage
tae-miR1137a	Traes_2DL_4F8C87C42.5										1,00	-1.0	1	20	1791	1810	UAGUACAAAGUUGAGUCAUC	GAUGACCCAACUUUGUACUA	Cleavage
tae-miR1137b-5p	Traes_2DL_4F8C87C42.5										1,05	-1.0	1	21	1944	1964	UCCGUUCCAGAAUAGAUGACC	GGUCAUCUAUUUUGGAACGGA	Translation
tae-miR1122a	Traes_2DL_4F8C87C42.6										2,05	-1.0	1	20	1750	1769	UAGAUACAUCCGUAUCUAGA	ACUACAUACGGAUGUAUAUA	Cleavage
tae-miR1121	Traes_2DL_74E241841.2										2,05	-1.0	1	22	448	469	AGUAGUGAUCUAAACGCUCUUA	UAAGAGUGUUUGGAUCACUACU	Translation
tae-miR1130b-3p	Traes_2DL_8F04980F0.5										2,05	-1.0	1	21	468	488	UCUUAUAUUAUGGGACGGAGG	CCUCCGUCCCUAAUAUAAGG	Translation
tae-miR1128	Traes_2DL_9AD532712.8										2,00	-1.0	1	21	305	325	UACUACUCCCUCCGUCCGAAA	GUCCGGACGGAGGGAGUAGUU	Cleavage
tae-miR1122a	Traes_2DL_B3CD23EBC.1										2,05	-1.0	1	20	9	28	UAGAUACAUCCGUAUCUAGA	ACUACAUACGGAUGUAUAUA	Cleavage
tae-miR9662a-3p	Traes_2DL_BE754F0C1.1	PF02536	KOG1267		K15032	GO:GO:0006355,GO:GO:0005739,GO:GO:0003690	AT5G07900.1	Mitochondrial transcription termination factor family protein	LOC_Os06g12100.1	mTERF family protein, expressed	1,00	-1.0	1	21	687	707	UUGAACAUCCCAGAGCCACCG	CCGUGGCUCUGGGAUGUUCAG	Cleavage
tae-miR9662b-3p	Traes_2DL_BE754F0C1.1	PF02536	KOG1267		K15032	GO:GO:0006355,GO:GO:0005739,GO:GO:0003690	AT5G07900.1	Mitochondrial transcription termination factor family protein	LOC_Os06g12100.1	mTERF family protein, expressed	1,00	-1.0	1	21	686	706	UGAACAUCCCAGAGCCACCGG	CCCGUGGCUCUGGGAUGUUCA	Cleavage
tae-miR1134	Traes_2DL_E8A7ABB5D.1										3,00	-1.0	1	24	509	532	CAACAACAACAAGAAGAAGAAGAU	AUCUUCAUGGUCUUGUUGUUGUUG	Cleavage
tae-miR1134	Traes_2DL_E8A7ABB5D.2										3,00	-1.0	1	24	629	652	CAACAACAACAAGAAGAAGAAGAU	AUCUUCAUGGUCUUGUUGUUGUUG	Cleavage
tae-miR1134	Traes_2DL_E8A7ABB5D.3										3,00	-1.0	1	24	614	637	CAACAACAACAAGAAGAAGAAGAU	AUCUUCAUGGUCUUGUUGUUGUUG	Cleavage
tae-miR1134	Traes_2DL_E8A7ABB5D.4										3,00	-1.0	1	24	512	535	CAACAACAACAAGAAGAAGAAGAU	AUCUUCAUGGUCUUGUUGUUGUUG	Cleavage
tae-miR1134	Traes_2DL_E8A7ABB5D.5										3,00	-1.0	1	24	509	532	CAACAACAACAAGAAGAAGAAGAU	AUCUUCAUGGUCUUGUUGUUGUUG	Cleavage
tae-miR1134	Traes_2DL_E8A7ABB5D.6										3,00	-1.0	1	24	509	532	CAACAACAACAAGAAGAAGAAGAU	AUCUUCAUGGUCUUGUUGUUGUUG	Cleavage
tae-miR1134	Traes_2DL_E8A7ABB5D.7										3,00	-1.0	1	24	91	114	CAACAACAACAAGAAGAAGAAGAU	AUCUUCAUGGUCUUGUUGUUGUUG	Cleavage
tae-miR1134	Traes_2DL_E8A7ABB5D.8										3,00	-1.0	1	24	77	100	CAACAACAACAAGAAGAAGAAGAU	AUCUUCAUGGUCUUGUUGUUGUUG	Cleavage
tae-miR1134	Traes_2DL_E8A7ABB5D.9										3,00	-1.0	1	24	28	51	CAACAACAACAAGAAGAAGAAGAU	AUCUUCAUGGUCUUGUUGUUGUUG	Cleavage
tae-miR160	Traes_2DL_EF12C0B89.2										1,00	-1.0	1	21	225	245	UGCCUGGCUCCCUGUAUGCCA	UGGCAUGCAGGGAGCCAGGCA	Cleavage
tae-miR9664-3p	Traes_2DS_480CFDFE4.1	PF01637	KOG4658		K13457	GO:GO:0005524	AT3G07040.1	NB-ARC domain-containing disease resistance protein	LOC_Os11g46210.1	NB-ARC domain containing protein, expressed	2,05	-1.0	1	21	243	263	UUGCAGUCCUCGAUGUCGUAG	CUACGACAUCGAGGAUUGCAU	Cleavage
tae-miR9664-3p	Traes_2DS_480CFDFE4.2	PF01637				GO:GO:0005524	AT3G07040.1	NB-ARC domain-containing disease resistance protein	LOC_Os11g46210.1	NB-ARC domain containing protein, expressed	2,05	-1.0	1	21	259	279	UUGCAGUCCUCGAUGUCGUAG	CUACGACAUCGAGGAUUGCAU	Cleavage
tae-miR5049-3p	Traes_2DS_851973269.7										1,05	-1.0	1	21	178	198	AAUAUGGAUCGGAGGGAGUAC	GUACUCCCUCCGAUUCAUAUU	Cleavage
tae-miR1122b-3p	Traes_2DS_9B301D4F8.2										1,05	-1.0	1	21	843	863	AGACUUAUAUGUAGGAACGGA	UCCGUUCCUAAAUAUAAGUCU	Translation
tae-miR1123	Traes_2DS_C866A54F1.2										1,00	-1.0	1	23	792	814	UCCGUGAGACCUGGUCUCAUAGA	UCUAUGAGACCAGGUCUCACGGG	Cleavage
tae-miR1123	Traes_2DS_C866A54F1.4										1,00	-1.0	1	23	786	808	UCCGUGAGACCUGGUCUCAUAGA	UCUAUGAGACCAGGUCUCACGGG	Cleavage
tae-miR9778	Traes_2DS_C9635AFF7.1										2,05	-1.0	1	21	126	146	UGCAUCAUCUCGAACUCGUCG	CGGCGAGUUCCAGAUGAUGCA	Translation
tae-miR9778	Traes_2DS_C9635AFF7.2										2,05	-1.0	1	21	105	125	UGCAUCAUCUCGAACUCGUCG	CGGCGAGUUCCAGAUGAUGCA	Translation
tae-miR1117	Traes_3AL_018D4C27C.1										1,05	-1.0	1	24	1609	1632	UAGUACCGGUUCGUGGCACGAACC	GGUUCGUGCCACGAACCGGUGCUA	Cleavage
tae-miR1133	Traes_3AL_30294D0A5.1										2,05	-1.0	1	22	392	413	CAUAUACUCCCUCCGUCCGAAA	UUUAGGACGGAGGGAGUAUUUG	Cleavage
tae-miR1133	Traes_3AL_386795528.2										0,00	-1.0	1	22	39	60	CAUAUACUCCCUCCGUCCGAAA	UUUCGGACGGAGGGAGUAUAUG	Cleavage
tae-miR1122a	Traes_3AL_5E5C8D264.2										1,00	-1.0	1	20	18	37	UAGAUACAUCCGUAUCUAGA	UCUAGACACGGAUGUAUCUA	Cleavage
tae-miR1137b-5p	Traes_3AL_7A4D34425.2										2,05	-1.0	1	21	1006	1026	UCCGUUCCAGAAUAGAUGACC	UAUCAUUUAUUCAGGAACGGA	Cleavage
tae-miR1122a	Traes_3AL_B9EDE61D1.1										2,05	-1.0	1	20	4	23	UAGAUACAUCCGUAUCUAGA	ACUACAUACGGAUGUAUAUA	Cleavage
tae-miR1122a	Traes_3AL_B9EDE61D1.2										2,05	-1.0	1	20	4	23	UAGAUACAUCCGUAUCUAGA	ACUACAUACGGAUGUAUAUA	Cleavage
tae-miR5049-3p	Traes_3AL_CF1A4ACDE.1										1,00	-1.0	1	21	917	937	AAUAUGGAUCGGAGGGAGUAC	UCCCUCCCUCCGAUCCAUAUU	Cleavage
tae-miR159a	Traes_3AL_F09C7CA19.1	PF00249					AT5G06100.1	myb domain protein 33	LOC_Os01g59660.2	MYB family transcription factor, putative, expressed	2,05	-1.0	1	21	620	640	UUUGGAUUGAAGGGAGCUCUG	UGGAGCUCCCUUCACUCCAAG	Cleavage
tae-miR159b	Traes_3AL_F09C7CA19.1	PF00249					AT5G06100.1	myb domain protein 33	LOC_Os01g59660.2	MYB family transcription factor, putative, expressed	2,05	-1.0	1	21	620	640	UUUGGAUUGAAGGGAGCUCUG	UGGAGCUCCCUUCACUCCAAG	Cleavage
tae-miR1121	Traes_3AL_F2EDB3661.7										2,00	-1.0	1	22	224	245	AGUAGUGAUCUAAACGCUCUUA	CCUCCGCGUUUAGAUCACUACU	Cleavage
tae-miR1133	Traes_3AL_FA6F160DF.7										3,00	-1.0	1	22	142	164	CAUAUACUCCCUCCG-UCCGAAA	UUUCGGAACGGAGGGAGUAUAUU	Cleavage
tae-miR5200	Traes_3AS_2A0765E10.1										2,05	-1.0	1	21	177	197	UGUAGAUACUCCCUAAGGCUU	UUCCCUUAGGAAGUAUCUACA	Translation
tae-miR5200	Traes_3AS_2A0765E10.2										2,05	-1.0	1	21	66	86	UGUAGAUACUCCCUAAGGCUU	UUCCCUUAGGAAGUAUCUACA	Translation
tae-miR5200	Traes_3AS_2A0765E10.3										2,05	-1.0	1	21	12	32	UGUAGAUACUCCCUAAGGCUU	UUCCCUUAGGAAGUAUCUACA	Translation
tae-miR9674b-5p	Traes_3AS_6CB1DBD28.1										2,00	-1.0	1	21	429	449	AUAGCAUCAUCCAUCCUACCC	GGGUAGGCUGGAUGAUGCUAG	Cleavage
tae-miR9674b-5p	Traes_3AS_6CB1DBD28.1										2,00	-1.0	1	21	429	449	AUAGCAUCAUCCAUCCUACCC	GGGUAGGCUGGAUGAUGCUAG	Cleavage
tae-miR1121	Traes_3AS_A466B9ECC.1										0,00	-1.0	1	22	725	746	AGUAGUGAUCUAAACGCUCUUA	UAAGAGCGUUUAGAUCACUACU	Cleavage
tae-miR6197-5p	Traes_3AS_A466B9ECC.1										2,05	-1.0	1	21	763	783	UCUGUAAACAAAUGUAGGACG	GCUCUUACAUUUCUUUACAGA	Cleavage
tae-miR1121	Traes_3AS_A466B9ECC.2										0,00	-1.0	1	22	725	746	AGUAGUGAUCUAAACGCUCUUA	UAAGAGCGUUUAGAUCACUACU	Cleavage
tae-miR6197-5p	Traes_3AS_A466B9ECC.2										2,05	-1.0	1	21	763	783	UCUGUAAACAAAUGUAGGACG	GCUCUUACAUUUCUUUACAGA	Cleavage
tae-miR1133	Traes_3AS_A6D3DD27E.2										2,05	-1.0	1	22	16	37	CAUAUACUCCCUCCGUCCGAAA	UUUGGGACGGAGGGAGUAUGUG	Cleavage
tae-miR1133	Traes_3AS_A6D3DD27E.4										2,05	-1.0	1	22	9	30	CAUAUACUCCCUCCGUCCGAAA	UUUGGGACGGAGGGAGUAUGUG	Cleavage
tae-miR1130b-3p	Traes_3AS_AA474B823.1										1,05	-1.0	1	21	1652	1672	UCUUAUAUUAUGGGACGGAGG	CCUCCGUCCCAUAAUAUGAGA	Cleavage
tae-miR1122c-3p	Traes_3AS_AA474B823.1										1,05	-1.0	1	21	1652	1672	UCUAAUAUUAUGGGACGGAGG	CCUCCGUCCCAUAAUAUGAGA	Cleavage
tae-miR1130b-3p	Traes_3AS_AA474B823.3										1,05	-1.0	1	21	1397	1417	UCUUAUAUUAUGGGACGGAGG	CCUCCGUCCCAUAAUAUGAGA	Cleavage
tae-miR1122c-3p	Traes_3AS_AA474B823.3										1,05	-1.0	1	21	1397	1417	UCUAAUAUUAUGGGACGGAGG	CCUCCGUCCCAUAAUAUGAGA	Cleavage
tae-miR159a	Traes_3AS_D1D1A1994.1								LOC_Os01g11430.1	expressed protein	2,05	-1.0	1	21	101	121	UUUGGAUUGAAGGGAGCUCUG	CAGAGCCCCCUUCAAACCAAA	Cleavage
tae-miR159b	Traes_3AS_D1D1A1994.1								LOC_Os01g11430.1	expressed protein	2,05	-1.0	1	21	101	121	UUUGGAUUGAAGGGAGCUCUG	CAGAGCCCCCUUCAAACCAAA	Cleavage
tae-miR1122b-3p	Traes_3AS_D7F2F36BB.2										1,05	-1.0	1	21	564	584	AGACUUAUAUGUAGGAACGGA	UCCGUUCCUAAAUAUAAGUCU	Translation
tae-miR1122b-3p	Traes_3AS_D7F2F36BB.3										1,05	-1.0	1	21	540	560	AGACUUAUAUGUAGGAACGGA	UCCGUUCCUAAAUAUAAGUCU	Translation
tae-miR5200	Traes_3B_0E2173F88.1										2,05	-1.0	1	21	171	191	UGUAGAUACUCCCUAAGGCUU	UUCCCUUAGGAAGUAUCUACA	Translation
tae-miR5200	Traes_3B_0E2173F88.2										2,05	-1.0	1	21	177	197	UGUAGAUACUCCCUAAGGCUU	UUCCCUUAGGAAGUAUCUACA	Translation
tae-miR5200	Traes_3B_0E2173F88.3										2,05	-1.0	1	21	171	191	UGUAGAUACUCCCUAAGGCUU	UUCCCUUAGGAAGUAUCUACA	Translation
tae-miR9652-5p	Traes_3B_119F6A405.1										2,05	-1.0	1	22	589	610	CCUGUUUGUCAUUAAGUUUCUU	AAGAAACUGAAUGACAAGCAGG	Cleavage
tae-miR9662a-3p	Traes_3B_224A6B7E5.1	PF02536				GO:GO:0006355,GO:GO:0005739,GO:GO:0003690	AT5G07900.1	Mitochondrial transcription termination factor family protein	LOC_Os06g12100.1	mTERF family protein, expressed	2,00	-1.0	1	21	189	209	UUGAACAUCCCAGAGCCACCG	CCGUGGUUCUGGGAUGUUCAG	Cleavage
tae-miR9662b-3p	Traes_3B_224A6B7E5.1	PF02536				GO:GO:0006355,GO:GO:0005739,GO:GO:0003690	AT5G07900.1	Mitochondrial transcription termination factor family protein	LOC_Os06g12100.1	mTERF family protein, expressed	2,00	-1.0	1	21	188	208	UGAACAUCCCAGAGCCACCGG	CCCGUGGUUCUGGGAUGUUCA	Cleavage
tae-miR159a	Traes_3B_25320ED72.1	PF00249	KOG0048		K09422		AT5G06100.1	myb domain protein 33	LOC_Os01g59660.2	MYB family transcription factor, putative, expressed	2,05	-1.0	1	21	956	976	UUUGGAUUGAAGGGAGCUCUG	UGGAGCUCCCUUCACUCCAAG	Cleavage
tae-miR159b	Traes_3B_25320ED72.1	PF00249	KOG0048		K09422		AT5G06100.1	myb domain protein 33	LOC_Os01g59660.2	MYB family transcription factor, putative, expressed	2,05	-1.0	1	21	956	976	UUUGGAUUGAAGGGAGCUCUG	UGGAGCUCCCUUCACUCCAAG	Cleavage
tae-miR159a	Traes_3B_25320ED72.2	PF00249	KOG0048		K09422		AT5G06100.1	myb domain protein 33	LOC_Os01g59660.1	MYB family transcription factor, putative, expressed	2,05	-1.0	1	21	956	976	UUUGGAUUGAAGGGAGCUCUG	UGGAGCUCCCUUCACUCCAAG	Cleavage
tae-miR159b	Traes_3B_25320ED72.2	PF00249	KOG0048		K09422		AT5G06100.1	myb domain protein 33	LOC_Os01g59660.1	MYB family transcription factor, putative, expressed	2,05	-1.0	1	21	956	976	UUUGGAUUGAAGGGAGCUCUG	UGGAGCUCCCUUCACUCCAAG	Cleavage
tae-miR1130b-3p	Traes_3B_467787390.8										1,05	-1.0	1	21	3080	3100	UCUUAUAUUAUGGGACGGAGG	CCUCCGUCCCAAAAUAUAAGA	Translation
tae-miR9776	Traes_3B_470061369.1	PF00270		3.6.4.13		GO:GO:0005524,GO:GO:0003676	AT5G51280.1	DEAD-box protein abstrakt, putative	LOC_Os02g05660.1	DEAD-box ATP-dependent RNA helicase 35A, putative, expressed	2,05	-1.0	1	21	34	54	UUGGACGAGGAUGUGCAACUG	AAGUGGCACAUCCUCGUCGAA	Cleavage
tae-miR1122a	Traes_3B_472072804.1										1,05	-1.0	1	20	789	808	UAGAUACAUCCGUAUCUAGA	UCUAGAUACGGAGGUAUCUA	Cleavage
tae-miR9778	Traes_3B_4C839468C.10										1,00	-1.0	1	21	126	146	UGCAUCAUCUCGAACUCGUCG	GGACGAGCUCGAGAUGAUGCA	Cleavage
tae-miR9778	Traes_3B_4C839468C.11										1,00	-1.0	1	21	126	146	UGCAUCAUCUCGAACUCGUCG	GGACGAGCUCGAGAUGAUGCA	Cleavage
tae-miR9778	Traes_3B_4C839468C.12										1,00	-1.0	1	21	126	146	UGCAUCAUCUCGAACUCGUCG	GGACGAGCUCGAGAUGAUGCA	Cleavage
tae-miR9778	Traes_3B_4C839468C.13										1,00	-1.0	1	21	102	122	UGCAUCAUCUCGAACUCGUCG	GGACGAGCUCGAGAUGAUGCA	Cleavage
tae-miR9778	Traes_3B_4C839468C.2										1,00	-1.0	1	21	126	146	UGCAUCAUCUCGAACUCGUCG	GGACGAGCUCGAGAUGAUGCA	Cleavage
tae-miR9778	Traes_3B_4C839468C.3										1,00	-1.0	1	21	126	146	UGCAUCAUCUCGAACUCGUCG	GGACGAGCUCGAGAUGAUGCA	Cleavage
tae-miR9778	Traes_3B_4C839468C.4										1,00	-1.0	1	21	126	146	UGCAUCAUCUCGAACUCGUCG	GGACGAGCUCGAGAUGAUGCA	Cleavage
tae-miR9778	Traes_3B_4C839468C.5										1,00	-1.0	1	21	126	146	UGCAUCAUCUCGAACUCGUCG	GGACGAGCUCGAGAUGAUGCA	Cleavage
tae-miR9778	Traes_3B_4C839468C.6										1,00	-1.0	1	21	126	146	UGCAUCAUCUCGAACUCGUCG	GGACGAGCUCGAGAUGAUGCA	Cleavage
tae-miR9778	Traes_3B_4C839468C.7										1,00	-1.0	1	21	126	146	UGCAUCAUCUCGAACUCGUCG	GGACGAGCUCGAGAUGAUGCA	Cleavage
tae-miR9778	Traes_3B_4C839468C.8										1,00	-1.0	1	21	126	146	UGCAUCAUCUCGAACUCGUCG	GGACGAGCUCGAGAUGAUGCA	Cleavage
tae-miR9778	Traes_3B_4C839468C.9										1,00	-1.0	1	21	126	146	UGCAUCAUCUCGAACUCGUCG	GGACGAGCUCGAGAUGAUGCA	Cleavage
tae-miR1137a	Traes_3B_59B827C5C.2										0,00	-1.0	1	20	748	767	UAGUACAAAGUUGAGUCAUC	GAUGACUCAACUUUGUACUA	Cleavage
tae-miR1122b-3p	Traes_3B_5DD9C1BE5.1										2,05	-1.0	1	21	71	91	AGACUUAUAUGUAGGAACGGA	UCCGUUCUUAAAUAUAAGUCU	Translation
tae-miR7757-5p	Traes_3B_5EFB9A61F.1								LOC_Os12g17490.1	stripe rust resistance protein Yr10, putative, expressed	1,05	-1.0	1	22	113	134	AUAAAACCUUCAGCUAUCCAUC	GAUGGAUAGCUGAAGGUUUUGU	Cleavage
tae-miR7757-5p	Traes_3B_5EFB9A61F.3								LOC_Os12g17490.1	stripe rust resistance protein Yr10, putative, expressed	1,05	-1.0	1	22	89	110	AUAAAACCUUCAGCUAUCCAUC	GAUGGAUAGCUGAAGGUUUUGU	Cleavage
tae-miR1122b-3p	Traes_3B_6D2DCE280.3										2,05	-1.0	1	21	576	596	AGACUUAUAUGUAGGAACGGA	UCUGUUCCUAAAUAUAAGUCU	Translation
tae-miR7757-5p	Traes_3B_7FFFF7BCF.1								LOC_Os02g18510.1	stripe rust resistance protein Yr10, putative, expressed	0,00	-1.0	1	22	89	110	AUAAAACCUUCAGCUAUCCAUC	GAUGGAUAGCUGAAGGUUUUAU	Cleavage
tae-miR5049-3p	Traes_3B_9D776A8C4.1										0,00	-1.0	1	21	575	595	AAUAUGGAUCGGAGGGAGUAC	GUACUCCCUCCGAUCCAUAUU	Cleavage
tae-miR1121	Traes_3B_9F321609E.8										1,05	-1.0	1	22	6	28	AGUAGUGAUCUAAACGCUCU-UA	UAUAGAGCGUUUAGAUCACUAUU	Cleavage
tae-miR9778	Traes_3B_F07C33521.1										1,00	-1.0	1	21	126	146	UGCAUCAUCUCGAACUCGUCG	GGACGAGCUCGAGAUGAUGCA	Cleavage
tae-miR9778	Traes_3B_F07C33521.10										1,00	-1.0	1	21	126	146	UGCAUCAUCUCGAACUCGUCG	GGACGAGCUCGAGAUGAUGCA	Cleavage
tae-miR9778	Traes_3B_F07C33521.2										1,00	-1.0	1	21	126	146	UGCAUCAUCUCGAACUCGUCG	GGACGAGCUCGAGAUGAUGCA	Cleavage
tae-miR9778	Traes_3B_F07C33521.3										1,00	-1.0	1	21	126	146	UGCAUCAUCUCGAACUCGUCG	GGACGAGCUCGAGAUGAUGCA	Cleavage
tae-miR9778	Traes_3B_F07C33521.4										1,00	-1.0	1	21	126	146	UGCAUCAUCUCGAACUCGUCG	GGACGAGCUCGAGAUGAUGCA	Cleavage
tae-miR9778	Traes_3B_F07C33521.5										1,00	-1.0	1	21	126	146	UGCAUCAUCUCGAACUCGUCG	GGACGAGCUCGAGAUGAUGCA	Cleavage
tae-miR9778	Traes_3B_F07C33521.6										1,00	-1.0	1	21	126	146	UGCAUCAUCUCGAACUCGUCG	GGACGAGCUCGAGAUGAUGCA	Cleavage
tae-miR9778	Traes_3B_F07C33521.7										1,00	-1.0	1	21	126	146	UGCAUCAUCUCGAACUCGUCG	GGACGAGCUCGAGAUGAUGCA	Cleavage
tae-miR9778	Traes_3B_F07C33521.8										1,00	-1.0	1	21	126	146	UGCAUCAUCUCGAACUCGUCG	GGACGAGCUCGAGAUGAUGCA	Cleavage
tae-miR9778	Traes_3B_F07C33521.9										1,00	-1.0	1	21	126	146	UGCAUCAUCUCGAACUCGUCG	GGACGAGCUCGAGAUGAUGCA	Cleavage
tae-miR1128	Traes_3B_F61EDC1AE.3										2,00	-1.0	1	21	1581	1601	UACUACUCCCUCCGUCCGAAA	UUCCGAACGGAGGGAGUAGUA	Cleavage
tae-miR1127a	Traes_3B_F61EDC1AE.3										1,05	-1.0	1	19	1577	1595	UCCUUCCGUUCGGAAUUAC	GUAAUUCCGAACGGAGGGA	Cleavage
tae-miR1130b-3p	Traes_3B_FB51F185F.2										1,05	-1.0	1	21	43	63	UCUUAUAUUAUGGGACGGAGG	CCUCCGUCCCAUAAUGUAAGA	Cleavage
tae-miR164	Traes_3B_FD8ACC50C.1										2,05	-1.0	1	21	1544	1564	UGGAGAAGCAGGGCACGUGCA	CGCACGGGCCCUGCUUCUGCA	Cleavage
tae-miR164	Traes_3B_FD8ACC50C.2										2,05	-1.0	1	21	1499	1519	UGGAGAAGCAGGGCACGUGCA	CGCACGGGCCCUGCUUCUGCA	Cleavage
tae-miR164	Traes_3B_FD8ACC50C.3										2,05	-1.0	1	21	1433	1453	UGGAGAAGCAGGGCACGUGCA	CGCACGGGCCCUGCUUCUGCA	Cleavage
tae-miR164	Traes_3B_FD8ACC50C.4										2,05	-1.0	1	21	764	784	UGGAGAAGCAGGGCACGUGCA	CGCACGGGCCCUGCUUCUGCA	Cleavage
tae-miR164	Traes_3B_FD8ACC50C.5										2,05	-1.0	1	21	752	772	UGGAGAAGCAGGGCACGUGCA	CGCACGGGCCCUGCUUCUGCA	Cleavage
tae-miR164	Traes_3B_FD8ACC50C.6										2,05	-1.0	1	21	731	751	UGGAGAAGCAGGGCACGUGCA	CGCACGGGCCCUGCUUCUGCA	Cleavage
tae-miR164	Traes_3B_FD8ACC50C.7										2,05	-1.0	1	21	710	730	UGGAGAAGCAGGGCACGUGCA	CGCACGGGCCCUGCUUCUGCA	Cleavage
tae-miR164	Traes_3B_FD8ACC50C.8										2,05	-1.0	1	21	653	673	UGGAGAAGCAGGGCACGUGCA	CGCACGGGCCCUGCUUCUGCA	Cleavage
tae-miR164	Traes_3B_FD8ACC50C.9										2,05	-1.0	1	21	356	376	UGGAGAAGCAGGGCACGUGCA	CGCACGGGCCCUGCUUCUGCA	Cleavage
tae-miR5049-3p	Traes_3DL_2B3694DC3.2										1,05	-1.0	1	21	228	248	AAUAUGGAUCGGAGGGAGUAC	CUACUCCCUCCGAUCCAAAUU	Cleavage
tae-miR1130b-3p	Traes_3DL_426EF6A68.2										2,05	-1.0	1	21	214	234	UCUUAUAUUAUGGGACGGAGG	CCUCCAUCCCGUAAUAUAAGA	Translation

tae-miR7757-5p	Traes_3DL_5C0BC5611.4								LOC_Os12g17490.1	stripe rust resistance protein Yr10, putative, expressed	1,05	-1.0	1	22	89	110	AUAAAACCUUCAGCUAUCCAUC	GAUGGAUAGCUGAAGGUUUUGU	Cleavage
tae-miR1137a	Traes_3DL_96847DCCF.1										1,00	-1.0	1	20	88	107	UAGUACAAAGUUGAGUCAUC	GAUGACCCAACUUUGUACUA	Cleavage
tae-miR5049-3p	Traes_3DL_D1CF4784D.2										2,05	-1.0	1	21	1646	1666	AAUAUGGAUCGGAGGGAGUAC	AUAAUCCCUCCGUUCCAUAUU	Cleavage
tae-miR159a	Traes_3DL_D9A29948B.1	PF00249					AT5G06100.1	myb domain protein 33	LOC_Os01g59660.1	MYB family transcription factor, putative, expressed	2,05	-1.0	1	21	620	640	UUUGGAUUGAAGGGAGCUCUG	UGGAGCUCCCUUCACUCCAAG	Cleavage
tae-miR159b	Traes_3DL_D9A29948B.1	PF00249					AT5G06100.1	myb domain protein 33	LOC_Os01g59660.1	MYB family transcription factor, putative, expressed	2,05	-1.0	1	21	620	640	UUUGGAUUGAAGGGAGCUCUG	UGGAGCUCCCUUCACUCCAAG	Cleavage
tae-miR159a	Traes_3DL_D9A29948B.2	PF00249					AT5G06100.1	myb domain protein 33	LOC_Os01g59660.1	MYB family transcription factor, putative, expressed	2,05	-1.0	1	21	620	640	UUUGGAUUGAAGGGAGCUCUG	UGGAGCUCCCUUCACUCCAAG	Cleavage
tae-miR159b	Traes_3DL_D9A29948B.2	PF00249					AT5G06100.1	myb domain protein 33	LOC_Os01g59660.1	MYB family transcription factor, putative, expressed	2,05	-1.0	1	21	620	640	UUUGGAUUGAAGGGAGCUCUG	UGGAGCUCCCUUCACUCCAAG	Cleavage
tae-miR1128	Traes_3DS_1DE20CFF3.2										2,05	-1.0	1	21	71	91	UACUACUCCCUCCGUCCGAAA	UUUGGGACGGAGGGAGUACUA	Cleavage
tae-miR1122a	Traes_3DS_1DE20CFF3.2										1,05	-1.0	1	20	2	21	UAGAUACAUCCGUAUCUAGA	UCUAGAUACAGAUGUAUCUA	Translation
tae-miR1128	Traes_3DS_1DE20CFF3.3										2,05	-1.0	1	21	71	91	UACUACUCCCUCCGUCCGAAA	UUUGGGACGGAGGGAGUACUA	Cleavage
tae-miR1122a	Traes_3DS_1DE20CFF3.3										1,05	-1.0	1	20	2	21	UAGAUACAUCCGUAUCUAGA	UCUAGAUACAGAUGUAUCUA	Translation
tae-miR1122b-3p	Traes_3DS_D42FCD586.1										1,05	-1.0	1	21	786	806	AGACUUAUAUGUAGGAACGGA	UCCGUUCCUAAAUAUAAGUCU	Translation
tae-miR1122b-3p	Traes_3DS_D42FCD586.2										1,05	-1.0	1	21	795	815	AGACUUAUAUGUAGGAACGGA	UCCGUUCCUAAAUAUAAGUCU	Translation
tae-miR7757-5p	Traes_4AL_13A3A40D3.1	PF13401	KOG4658				AT3G50950.2	HOPZ-ACTIVATED RESISTANCE 1	LOC_Os11g11810.1	NBS-LRR disease resistance protein, putative, expressed	1,05	-1.0	1	22	1337	1358	AUAAAACCUUCAGCUAUCCAUC	AGUGGAUAGCUGAAGGUUUCAU	Cleavage
tae-miR1135	Traes_4AL_158B970F4.1						AT1G50020.1		LOC_Os11g34870.1	expressed protein	2,05	-1.0	1	24	464	487	CUGCGACAAGUAAUUCCGAACGGA	UCCGUUCCGAAUUACUCGUCGCAG	Cleavage
tae-miR5175-5p	Traes_4AL_158B970F4.1						AT1G50020.1		LOC_Os11g34870.1	expressed protein	3,00	-1.0	1	21	532	552	UUCCAAAUUACUCGUCGUGGU	UCUGAGACGAGUAAUUUGGAA	Cleavage
tae-miR1117	Traes_4AL_2475C298D.1										2,05	-1.0	1	24	684	707	UAGUACCGGUUCGUGGCACGAACC	GGUUCAUGCCAUGAACCGGUACUA	Cleavage
tae-miR9778	Traes_4AL_2FC39B934.1										2,05	-1.0	1	21	126	146	UGCAUCAUCUCGAACUCGUCG	UGGCGAGUUCCAGAUGAUGCA	Translation
tae-miR408	Traes_4AL_364F56EDB.1										0,00	-1.0	1	21	8	28	CUGCACUGCCUCUUCCCUGGC	CCCAGGGAAGAGGCAGUGCAG	Cleavage
tae-miR9662a-3p	Traes_4AL_3A009739E.1	PF02536				GO:GO:0006355,GO:GO:0005739,GO:GO:0003690	AT1G21150.1	Mitochondrial transcription termination factor family protein	LOC_Os06g12100.1	mTERF family protein, expressed	2,05	-1.0	1	21	66	86	UUGAACAUCCCAGAGCCACCG	CCGUGGCUCUGGAAUGUUCAG	Cleavage
tae-miR9662b-3p	Traes_4AL_3A009739E.1	PF02536				GO:GO:0006355,GO:GO:0005739,GO:GO:0003690	AT1G21150.1	Mitochondrial transcription termination factor family protein	LOC_Os06g12100.1	mTERF family protein, expressed	2,05	-1.0	1	21	65	85	UGAACAUCCCAGAGCCACCGG	CCCGUGGCUCUGGAAUGUUCA	Cleavage
tae-miR1121	Traes_4AL_400EB3C17.10										2,05	-1.0	1	22	10	31	AGUAGUGAUCUAAACGCUCUUA	UAAGAGCGUUUAGAUCACUAAA	Cleavage
tae-miR1121	Traes_4AL_400EB3C17.3										2,05	-1.0	1	22	37	58	AGUAGUGAUCUAAACGCUCUUA	UAAGAGCGUUUAGAUCACUAAA	Cleavage
tae-miR1121	Traes_4AL_400EB3C17.7										2,05	-1.0	1	22	10	31	AGUAGUGAUCUAAACGCUCUUA	UAAGAGCGUUUAGAUCACUAAA	Cleavage
tae-miR1137a	Traes_4AL_40790E994.6										1,00	-1.0	1	20	1324	1343	UAGUACAAAGUUGAGUCAUC	GAUGACCCAACUUUGUACUA	Cleavage
tae-miR1137a	Traes_4AL_40790E994.7										1,00	-1.0	1	20	835	854	UAGUACAAAGUUGAGUCAUC	GAUGACCCAACUUUGUACUA	Cleavage
tae-miR1127b-3p	Traes_4AL_44E99BA01.2										1,05	-1.0	1	21	394	414	ACAAGUAUUUCUGGACGGAGG	CCUCCGUCCGGAAAUACUUGU	Cleavage
tae-miR1121	Traes_4AL_541C86EB9.2										1,00	-1.0	1	22	558	579	AGUAGUGAUCUAAACGCUCUUA	UGAGAUCGUUUAGAUCACUACU	Cleavage
tae-miR1121	Traes_4AL_541C86EB9.3										1,00	-1.0	1	22	558	579	AGUAGUGAUCUAAACGCUCUUA	UGAGAUCGUUUAGAUCACUACU	Cleavage
tae-miR1122b-3p	Traes_4AL_6252B0BA0.1										1,05	-1.0	1	21	46	66	AGACUUAUAUGUAGGAACGGA	UCCGUUCCUAAAUAUAAGUCU	Translation
tae-miR1137b-5p	Traes_4AL_748B84675.1										2,05	-1.0	1	21	844	864	UCCGUUCCAGAAUAGAUGACC	ACUCAUCUAUUCUGGGACGGU	Cleavage
tae-miR9778	Traes_4AL_79B47AF61.1										2,05	-1.0	1	21	126	146	UGCAUCAUCUCGAACUCGUCG	GGACGAGCUCGACAUGAUGCA	Cleavage
tae-miR9778	Traes_4AL_79B47AF61.3										2,05	-1.0	1	21	126	146	UGCAUCAUCUCGAACUCGUCG	GGACGAGCUCGACAUGAUGCA	Cleavage
tae-miR9778	Traes_4AL_79B47AF61.4										2,05	-1.0	1	21	126	146	UGCAUCAUCUCGAACUCGUCG	GGACGAGCUCGACAUGAUGCA	Cleavage
tae-miR5048-5p	Traes_4AL_85A7C60F9.1	PF00069		2.7.11.1		GO:GO:0006468,GO:GO:0005524,GO:GO:0004672	AT4G21230.1	cysteine-rich RLK (RECEPTOR-like protein kinase) 27	LOC_Os11g10640.1	protein kinase domain containing protein, expressed	2,05	-1.0	1	22	170	191	UUUGCAGGUUUUAGGUCUAAGU	ACUUGGACCUAAAACCUGCGAA	Cleavage
tae-miR5048-5p	Traes_4AL_85A7C60F9.7	PF00069		2.7.11.1		GO:GO:0006468,GO:GO:0005524,GO:GO:0004672	AT4G21230.1	cysteine-rich RLK (RECEPTOR-like protein kinase) 27	LOC_Os11g10640.1	protein kinase domain containing protein, expressed	2,05	-1.0	1	22	170	191	UUUGCAGGUUUUAGGUCUAAGU	ACUUGGACCUAAAACCUGCGAA	Cleavage
tae-miR5048-5p	Traes_4AL_85A7C60F9.8	PF00069		2.7.11.1		GO:GO:0006468,GO:GO:0005524,GO:GO:0004672	AT4G21230.1	cysteine-rich RLK (RECEPTOR-like protein kinase) 27	LOC_Os11g10640.1	protein kinase domain containing protein, expressed	2,05	-1.0	1	22	170	191	UUUGCAGGUUUUAGGUCUAAGU	ACUUGGACCUAAAACCUGCGAA	Cleavage
tae-miR1122b-3p	Traes_4AL_954AE4933.1										1,05	-1.0	1	21	583	603	AGACUUAUAUGUAGGAACGGA	UCCGUUCCUAAAUAUAAGUCU	Translation
tae-miR1122a	Traes_4AL_9726307FF.1										0,00	-1.0	1	20	1903	1922	UAGAUACAUCCGUAUCUAGA	UCUAGAUACGGAUGUAUCUA	Cleavage
tae-miR1128	Traes_4AL_99942CBEA.1										3,00	-1.0	1	21	693	714	UACUACUCCCUCCG-UCCGAAA	UUUAGGAACGGAGGGAGUAGUA	Cleavage
tae-miR1122b-3p	Traes_4AL_99942CBEA.1										2,05	-1.0	1	21	557	577	AGACUUAUAUGUAGGAACGGA	UCCGUUCUUAAAUAUAAGUCU	Translation
tae-miR9662a-3p	Traes_4AL_A4B73CC6A1.1	PF02536	KOG1267		K15032	GO:GO:0006355,GO:GO:0005739,GO:GO:0003690	AT5G07900.1	Mitochondrial transcription termination factor family protein	LOC_Os06g12100.1	mTERF family protein, expressed	2,05	-1.0	1	21	693	713	UUGAACAUCCCAGAGCCACCG	CCGUGGCUCUGGAAUGUUCAG	Cleavage
tae-miR9662b-3p	Traes_4AL_A4B73CC6A1.1	PF02536	KOG1267		K15032	GO:GO:0006355,GO:GO:0005739,GO:GO:0003690	AT5G07900.1	Mitochondrial transcription termination factor family protein	LOC_Os06g12100.1	mTERF family protein, expressed	2,05	-1.0	1	21	692	712	UGAACAUCCCAGAGCCACCGG	CCCGUGGCUCUGGAAUGUUCA	Cleavage
tae-miR9662a-3p	Traes_4AL_A4B73CC6A1.2	PF02536	KOG1267		K15032	GO:GO:0006355,GO:GO:0005739,GO:GO:0003690	AT5G07900.1	Mitochondrial transcription termination factor family protein	LOC_Os06g12100.1	mTERF family protein, expressed	2,05	-1.0	1	21	693	713	UUGAACAUCCCAGAGCCACCG	CCGUGGCUCUGGAAUGUUCAG	Cleavage
tae-miR9662b-3p	Traes_4AL_A4B73CC6A1.2	PF02536	KOG1267		K15032	GO:GO:0006355,GO:GO:0005739,GO:GO:0003690	AT5G07900.1	Mitochondrial transcription termination factor family protein	LOC_Os06g12100.1	mTERF family protein, expressed	2,05	-1.0	1	21	692	712	UGAACAUCCCAGAGCCACCGG	CCCGUGGCUCUGGAAUGUUCA	Cleavage
tae-miR1130b-3p	Traes_4AL_C55D89913.1										0,00	-1.0	1	21	10	30	UCUUAUAUUAUGGGACGGAGG	CCUCCGUCCCAUAAUAUAAGA	Cleavage
tae-miR1122c-3p	Traes_4AL_C55D89913.1										1,05	-1.0	1	21	10	30	UCUAAUAUUAUGGGACGGAGG	CCUCCGUCCCAUAAUAUAAGA	Cleavage
tae-miR1130b-3p	Traes_4AL_C55D89913.5										0,00	-1.0	1	21	10	30	UCUUAUAUUAUGGGACGGAGG	CCUCCGUCCCAUAAUAUAAGA	Cleavage
tae-miR1122c-3p	Traes_4AL_C55D89913.5										1,05	-1.0	1	21	10	30	UCUAAUAUUAUGGGACGGAGG	CCUCCGUCCCAUAAUAUAAGA	Cleavage
tae-miR1130b-3p	Traes_4AL_C55D89913.6										0,00	-1.0	1	21	10	30	UCUUAUAUUAUGGGACGGAGG	CCUCCGUCCCAUAAUAUAAGA	Cleavage
tae-miR1122c-3p	Traes_4AL_C55D89913.6										1,05	-1.0	1	21	10	30	UCUAAUAUUAUGGGACGGAGG	CCUCCGUCCCAUAAUAUAAGA	Cleavage
tae-miR1130b-3p	Traes_4AL_C55D89913.7										0,00	-1.0	1	21	10	30	UCUUAUAUUAUGGGACGGAGG	CCUCCGUCCCAUAAUAUAAGA	Cleavage
tae-miR1122c-3p	Traes_4AL_C55D89913.7										1,05	-1.0	1	21	10	30	UCUAAUAUUAUGGGACGGAGG	CCUCCGUCCCAUAAUAUAAGA	Cleavage
tae-miR1130b-3p	Traes_4AL_C55D89913.8										0,00	-1.0	1	21	10	30	UCUUAUAUUAUGGGACGGAGG	CCUCCGUCCCAUAAUAUAAGA	Cleavage
tae-miR1122c-3p	Traes_4AL_C55D89913.8										1,05	-1.0	1	21	10	30	UCUAAUAUUAUGGGACGGAGG	CCUCCGUCCCAUAAUAUAAGA	Cleavage
tae-miR5049-3p	Traes_4AL_C9E08C423.5										0,00	-1.0	1	21	69	89	AAUAUGGAUCGGAGGGAGUAC	CUACUCCCUCCGAUCCAUAUU	Cleavage
tae-miR9782	Traes_4AL_D576D98B4.1										1,00	-1.0	1	24	2	25	GUAUUAGGUUGGUCAAAUUGACGA	UCGUCAAUUUGACCAACCUAAUAU	Cleavage
tae-miR9782	Traes_4AL_D576D98B4.2										1,00	-1.0	1	24	2	25	GUAUUAGGUUGGUCAAAUUGACGA	UCGUCAAUUUGACCAACCUAAUAU	Cleavage
tae-miR9661-5p	Traes_4AL_D8CCE0D2E.1										2,00	-1.0	1	21	647	667	UGAAGUAGAGCAGGGACCUCA	GGAGGUCUCUGCUCUACUUCC	Cleavage
tae-miR9661-5p	Traes_4AL_D8CCE0D2E.2										2,00	-1.0	1	21	644	664	UGAAGUAGAGCAGGGACCUCA	GGAGGUCUCUGCUCUACUUCC	Cleavage
tae-miR9661-5p	Traes_4AL_D8CCE0D2E.3										2,00	-1.0	1	21	620	640	UGAAGUAGAGCAGGGACCUCA	GGAGGUCUCUGCUCUACUUCC	Cleavage
tae-miR9661-5p	Traes_4AL_D8CCE0D2E.4										2,00	-1.0	1	21	587	607	UGAAGUAGAGCAGGGACCUCA	GGAGGUCUCUGCUCUACUUCC	Cleavage
tae-miR5384-3p	Traes_4AL_E60E5B7BA.1										2,05	-1.0	1	21	269	289	UGAGCGCGCCGCCGUCGAAUG	UGUUCCACGGCGGCGCGCACA	Cleavage
tae-miR5384-3p	Traes_4AL_E60E5B7BA.2										2,05	-1.0	1	21	140	160	UGAGCGCGCCGCCGUCGAAUG	UGUUCCACGGCGGCGCGCACA	Cleavage
tae-miR1130b-3p	Traes_4AS_0E2735576.2										1,05	-1.0	1	21	977	997	UCUUAUAUUAUGGGACGGAGG	CCUCCGUCUCAUAAUAUAAGA	Cleavage
tae-miR1120c-5p	Traes_4AS_0E2735576.2										0,00	-1.0	1	21	1020	1040	UAAUAUAAGAACGUUUUUGAC	GACAAAAACGUUCUUAUAUUA	Cleavage
tae-miR9674a-5p	Traes_4AS_AB9088E9B.2										2,05	-1.0	1	21	1383	1403	GCAUCAUCCAUCCUACCUAUC	UGAUGGUCAGAUGGAUGAUGC	Cleavage
tae-miR9674a-5p	Traes_4AS_AB9088E9B.3										2,05	-1.0	1	21	1383	1403	GCAUCAUCCAUCCUACCUAUC	UGAUGGUCAGAUGGAUGAUGC	Cleavage
tae-miR5384-3p	Traes_4AS_C7CBD4A47.1										2,05	-1.0	1	21	194	214	UGAGCGCGCCGCCGUCGAAUG	UCUUCGACGGCGGCGCGCACU	Cleavage
tae-miR1135	Traes_4AS_D533B306B.3	PF01485	KOG1815	6.3.2.19		GO:GO:0016567,GO:GO:0004842	AT2G16090.1	RING/U-box superfamily protein	LOC_Os03g13100.1	ariadne-1, putative, expressed	2,00	-1.0	1	24	1698	1721	CUGCGACAAGUAAUUCCGAACGGA	CGAACCAGGAAUUACUUGUCGCAG	Cleavage
tae-miR1131	Traes_4BL_178D6FFF1.1										1,05	-1.0	1	22	167	188	UAGUACCGGUUCGUGGCUAACC	GGUUAGCCACGAACGAUACUA	Cleavage
tae-miR1131	Traes_4BL_178D6FFF1.2										1,05	-1.0	1	22	167	188	UAGUACCGGUUCGUGGCUAACC	GGUUAGCCACGAACCGAUACUA	Cleavage
tae-miR9662a-3p	Traes_4BL_29D8171E0.1								LOC_Os06g12100.1	mTERF family protein, expressed	2,00	-1.0	1	21	132	152	UUGAACAUCCCAGAGCCACCG	UCGUGGCGCUGGGAUGUUCAG	Cleavage
tae-miR9662b-3p	Traes_4BL_29D8171E0.1								LOC_Os06g12100.1	mTERF family protein, expressed	2,05	-1.0	1	21	131	151	UGAACAUCCCAGAGCCACCGG	CUCGUGGCGCUGGGAUGUUCA	Cleavage
tae-miR1122b-3p	Traes_4BL_2D34597CF.2										2,05	-1.0	1	21	454	474	AGACUUAUAUGUAGGAACGGA	UCUGUUCCUAAAUAUAAGUCU	Translation
tae-miR1122b-3p	Traes_4BL_2D34597CF.3										2,05	-1.0	1	21	454	474	AGACUUAUAUGUAGGAACGGA	UCUGUUCCUAAAUAUAAGUCU	Translation
tae-miR1128	Traes_4BL_37FDD905B.1										2,05	-1.0	1	21	125	145	UACUACUCCCUCCGUCCGAAA	UUUGGGACGGAGGGAGUAGGA	Cleavage
tae-miR5384-3p	Traes_4BL_3B7A98FD6.1										2,05	-1.0	1	21	191	211	UGAGCGCGCCGCCGUCGAAUG	UCUUCGACGGCGGCGCGCACU	Cleavage
tae-miR9666a-3p	Traes_4BL_44010E331.1										3,00	-1.0	1	22	176	197	CGGUAGGGCUGUAUGAUGGCGA	UCGGCAUGAUACAGCCCUACCU	Cleavage
tae-miR1122b-3p	Traes_4BL_62355968B.1										2,05	-1.0	1	21	706	726	AGACUUAUAUGUAGGAACGGA	UCUGUUCCUAAAUAUAAGUCU	Translation
tae-miR1122a	Traes_4BL_62355968B.1										2,05	-1.0	1	20	747	766	UAGAUACAUCCGUAUCUAGA	ACUACAUACGGAUGUAUAUA	Cleavage
tae-miR1137a	Traes_4BL_D08D5FF85.2										2,00	-1.0	1	20	722	741	UAGUACAAAGUUGAGUCAUC	AGUGUCUCAACUUUGUACUA	Cleavage
tae-miR1137a	Traes_4BL_F3D5D7034.2										1,00	-1.0	1	19	1	19	UAGUACAAAGUUGAGUCAU	AUGACCCAACUUUGUACUA	Cleavage
tae-miR1137b-5p	Traes_4BL_F3D5D7034.2										1,05	-1.0	1	21	37	57	UCCGUUCCAGAAUAGAUGACC	GGUCAUCUAUUUUGGAACGGA	Translation
tae-miR9662a-3p	Traes_4BS_257C5B099.1	PF02536				GO:GO:0006355,GO:GO:0005739,GO:GO:0003690	AT5G07900.1	Mitochondrial transcription termination factor family protein	LOC_Os06g12100.1	mTERF family protein, expressed	2,00	-1.0	1	21	66	86	UUGAACAUCCCAGAGCCACCG	CCGUGGUUCUGGGAUGUUCAG	Cleavage
tae-miR9662b-3p	Traes_4BS_257C5B099.1	PF02536				GO:GO:0006355,GO:GO:0005739,GO:GO:0003690	AT5G07900.1	Mitochondrial transcription termination factor family protein	LOC_Os06g12100.1	mTERF family protein, expressed	2,00	-1.0	1	21	65	85	UGAACAUCCCAGAGCCACCGG	CCCGUGGUUCUGGGAUGUUCA	Cleavage
tae-miR156	Traes_4BS_375EF1D15.1								LOC_Os01g69830.1	OsSPL2 - SBP-box gene family member, expressed	2,05	-1.0	1	21	235	255	UGACAGAAGAGAGUGAGCACA	UGUGCUCUCUCUUUUCUGUCA	Cleavage
tae-miR1118	Traes_4BS_4BC02509F.1										2,05	-1.0	1	23	525	547	CACUACAUUAUGGAAUGGAGGGA	UCCCUCCGUUCCAUAAUGUAAUG	Cleavage
tae-miR9666a-3p	Traes_4BS_5419B1DE7.1										3,00	-1.0	1	22	176	197	CGGUAGGGCUGUAUGAUGGCGA	UCGGCAUGAUACAGCCCUACCU	Cleavage
tae-miR9661-5p	Traes_4BS_5EC01BC81.1										2,00	-1.0	1	21	653	673	UGAAGUAGAGCAGGGACCUCA	GGAGGUCUCUGCUCUACUUCC	Cleavage
tae-miR9661-5p	Traes_4BS_5EC01BC81.2										2,00	-1.0	1	21	653	673	UGAAGUAGAGCAGGGACCUCA	GGAGGUCUCUGCUCUACUUCC	Cleavage
tae-miR9661-5p	Traes_4BS_5EC01BC81.3										2,00	-1.0	1	21	653	673	UGAAGUAGAGCAGGGACCUCA	GGAGGUCUCUGCUCUACUUCC	Cleavage
tae-miR1122b-3p	Traes_4BS_6252B0BA0.1										1,05	-1.0	1	21	46	66	AGACUUAUAUGUAGGAACGGA	UCCGUUCCUAAAUAUAAGUCU	Translation
tae-miR7757-5p	Traes_4BS_B45729F4D.4	PF00931				GO:GO:0043531	AT3G07040.1	NB-ARC domain-containing disease resistance protein	LOC_Os11g11810.1	NBS-LRR disease resistance protein, putative, expressed	2,05	-1.0	1	22	653	674	AUAAAACCUUCAGCUAUCCAUC	AAUGGAUAUCUGAAGGUUUCAU	Cleavage
tae-miR5384-3p	Traes_4BS_F1D748B88.1										2,05	-1.0	1	21	269	289	UGAGCGCGCCGCCGUCGAAUG	UGUUCCACGGCGGCGCGCACA	Cleavage
tae-miR1122a	Traes_4BS_F395A220A.2										0,00	-1.0	1	20	1336	1355	UAGAUACAUCCGUAUCUAGA	UCUAGAUACGGAUGUAUCUA	Cleavage
tae-miR7757-5p	Traes_4DL_113F6DD2B.1	PF00931				GO:GO:0043531	AT1G50180.1	NB-ARC domain-containing disease resistance protein	LOC_Os12g13550.1	NBS-LRR disease resistance protein, putative, expressed	2,05	-1.0	1	22	215	236	AUAAAACCUUCAGCUAUCCAUC	AAUGGAUGGCUGAAGGCUUUAU	Cleavage
tae-miR7757-5p	Traes_4DL_113F6DD2B.2	PF00931				GO:GO:0043531	AT1G50180.1	NB-ARC domain-containing disease resistance protein	LOC_Os10g22484.2	RGH2B, putative, expressed	2,05	-1.0	1	22	215	236	AUAAAACCUUCAGCUAUCCAUC	AAUGGAUGGCUGAAGGCUUUAU	Cleavage
tae-miR5085	Traes_4DL_341830EFF.1										2,05	-1.0	1	21	583	603	AAGGACAUUUUUUGUGGCCUG	GAGGACACAAAAAAUGUCGUU	Cleavage
tae-miR1133	Traes_4DL_529524815.5										2,00	-1.0	1	22	103	127	CAUAUACUCCCUCCG-UCCGA--AA	UUAGUCGGAUCGGAGGGAGUAUAUG	Cleavage
tae-miR1130b-3p	Traes_4DL_84745919A.2										2,05	-1.0	1	21	2964	2984	UCUUAUAUUAUGGGACGGAGG	CCUCUGUCCCAUAAUAUAGGA	Cleavage
tae-miR1130a	Traes_4DL_84745919A.2										1,05	-1.0	1	23	3016	3038	CCUCCGUCUCGUAAUGUAAGACG	UGUCUUACAUUAUGAGACGGAGG	Translation
tae-miR1128	Traes_4DL_93D4DED90.3										3,00	-1.0	1	21	565	586	UACUACUCCCUCCG-UCCGAAA	UUUUGGAACGGAGGGAGUAGUA	Cleavage
tae-miR1137a	Traes_4DL_93D4DED90.3										2,05	-1.0	1	20	508	527	UAGUACAAAGUUGAGUCAUC	GAUGACCCAACUUUGUAUUA	Cleavage
tae-miR1137b-5p	Traes_4DL_93D4DED90.3										1,05	-1.0	1	21	556	576	UCCGUUCCAGAAUAGAUGACC	GGUCAUCUAUUUUGGAACGGA	Translation
tae-miR1137b-5p	Traes_4DS_28E6C4F21.2										1,05	-1.0	1	21	61	81	UCCGUUCCAGAAUAGAUGACC	AGUCAUCUAUUUUGGAACGGA	Translation
tae-miR1137b-5p	Traes_4DS_28E6C4F21.3										1,05	-1.0	1	21	61	81	UCCGUUCCAGAAUAGAUGACC	AGUCAUCUAUUUUGGAACGGA	Translation
tae-miR1137b-5p	Traes_4DS_28E6C4F21.4										1,05	-1.0	1	21	61	81	UCCGUUCCAGAAUAGAUGACC	AGUCAUCUAUUUUGGAACGGA	Translation
tae-miR7757-5p	Traes_4DS_5B29EE3DD.5	PF00931				GO:GO:0043531	AT3G50950.1	HOPZ-ACTIVATED RESISTANCE 1	LOC_Os11g11810.1	NBS-LRR disease resistance protein, putative, expressed	2,05	-1.0	1	22	1379	1400	AUAAAACCUUCAGCUAUCCAUC	AAUGGAUAUCUGAAGGUUUCAU	Cleavage
tae-miR7757-5p	Traes_4DS_5B29EE3DD.6	PF00931				GO:GO:0043531	AT3G50950.1	HOPZ-ACTIVATED RESISTANCE 1	LOC_Os11g11810.1	NBS-LRR disease resistance protein, putative, expressed	2,05	-1.0	1	22	1379	1400	AUAAAACCUUCAGCUAUCCAUC	AAUGGAUAUCUGAAGGUUUCAU	Cleavage
tae-miR7757-5p	Traes_4DS_5B29EE3DD.7	PF00931				GO:GO:0043531	AT1G58390.1	Disease resistance protein (CC-NBS-LRR class) family	LOC_Os11g11810.1	NBS-LRR disease resistance protein, putative, expressed	2,05	-1.0	1	22	824	845	AUAAAACCUUCAGCUAUCCAUC	AAUGGAUAUCUGAAGGUUUCAU	Cleavage
tae-miR1130b-3p	Traes_4DS_9179FF158.4										1,05	-1.0	1	21	41	61	UCUUAUAUUAUGGGACGGAGG	CCUCCGUCCCAUAAUGUAAGA	Cleavage
tae-miR9778	Traes_4DS_B08D83A2F.1										2,05	-1.0	1	21	126	146	UGCAUCAUCUCGAACUCGUCG	UGGCGAGUUCCAGAUGAUGCA	Translation
tae-miR164	Traes_4DS_C08BF9DB2.1										2,05	-1.0	1	21	387	407	UGGAGAAGCAGGGCACGUGCA	UUCACGUGCCCUGCUUUUCCU	Cleavage
tae-miR1128	Traes_4DS_C22A101FE.2										2,05	-1.0	1	21	670	690	UACUACUCCCUCCGUCCGAAA	UUUGGGACGAAGGGAGUAGUA	Cleavage
tae-miR1128	Traes_4DS_C22A101FE.9										2,05	-1.0	1	21	358	378	UACUACUCCCUCCGUCCGAAA	UUUGGGACGAAGGGAGUAGUA	Cleavage
tae-miR1122a	Traes_4DS_E8239194E.2										2,05	-1.0	1	20	692	711	UAGAUACAUCCGUAUCUAGA	UCUAAAUACGGAUGUAUCAA	Cleavage
tae-miR5384-3p	Traes_4DS_ECFDD82BA.1										2,05	-1.0	1	21	269	289	UGAGCGCGCCGCCGUCGAAUG	UGUUCCACGGCGGCGCGCACA	Cleavage
tae-miR1137b-5p	Traes_4DS_EFE0451D7.3										1,05	-1.0	1	21	950	970	UCCGUUCCAGAAUAGAUGACC	AGUCAUCUAUUUUGGAACGGA	Translation
tae-miR1128	Traes_5AL_3AB81DF9B.1										3,00	-1.0	1	21	151	172	UACUACUCCCUCCG-UCCGAAA	UUUAGGAACGGAGGGAGUAGUA	Cleavage
tae-miR9652-5p	Traes_5AL_481F73E59.1										2,05	-1.0	1	22	883	904	CCUGUUUGUCAUUAAGUUUCUU	AAGAAACUGAAUGACAAGCAGG	Cleavage
tae-miR9652-5p	Traes_5AL_481F73E59.2										2,05	-1.0	1	22	883	904	CCUGUUUGUCAUUAAGUUUCUU	AAGAAACUGAAUGACAAGCAGG	Cleavage
tae-miR9652-5p	Traes_5AL_481F73E59.3										2,05	-1.0	1	22	883	904	CCUGUUUGUCAUUAAGUUUCUU	AAGAAACUGAAUGACAAGCAGG	Cleavage
tae-miR9652-5p	Traes_5AL_481F73E59.4										2,05	-1.0	1	22	883	904	CCUGUUUGUCAUUAAGUUUCUU	AAGAAACUGAAUGACAAGCAGG	Cleavage
tae-miR9652-5p	Traes_5AL_481F73E59.5										2,05	-1.0	1	22	583	604	CCUGUUUGUCAUUAAGUUUCUU	AAGAAACUGAAUGACAAGCAGG	Cleavage
tae-miR9652-5p	Traes_5AL_481F73E59.6										2,05	-1.0	1	22	883	904	CCUGUUUGUCAUUAAGUUUCUU	AAGAAACUGAAUGACAAGCAGG	Cleavage
tae-miR1127b-3p	Traes_5AL_7256A9F19.2										1,05	-1.0	1	21	991	1011	ACAAGUAUUUCUGGACGGAGG	CCUCCGUCCGGAAAUACUUGU	Cleavage
tae-miR1122b-3p	Traes_5AL_7FF780810.1										1,05	-1.0	1	21	22	42	AGACUUAUAUGUAGGAACGGA	UCCGUUCCUAAAUAUAAGUCU	Translation
tae-miR7757-5p	Traes_5AL_B4E8A3115.1	PF00931				GO:GO:0043531	AT3G46710.1	NB-ARC domain-containing disease resistance protein	LOC_Os05g40150.1	RGH2B, putative, expressed	1,05	-1.0	1	22	368	389	AUAAAACCUUCAGCUAUCCAUC	AAUGGAUAGCUGAAGGUCUUAU	Cleavage
tae-miR7757-5p	Traes_5AL_B4E8A3115.2	PF03106,PF12796,PF00931	KOG4658			GO:GO:0043565,GO:GO:0006355,GO:GO:0003700,GO:GO:0043531	AT2G31820.1	Ankyrin repeat family protein	LOC_Os11g45750.2	WRKY125, expressed	1,05	-1.0	1	22	2324	2345	AUAAAACCUUCAGCUAUCCAUC	AAUGGAUAGCUGAAGGUCUUAU	Cleavage
tae-miR7757-5p	Traes_5AL_B4E8A3115.3	PF03106,PF12796,PF00931	KOG4658			GO:GO:0043565,GO:GO:0006355,GO:GO:0003700,GO:GO:0043531	AT2G31820.1	Ankyrin repeat family protein	LOC_Os11g45750.2	WRKY125, expressed	1,05	-1.0	1	22	2324	2345	AUAAAACCUUCAGCUAUCCAUC	AAUGGAUAGCUGAAGGUCUUAU	Cleavage
tae-miR7757-5p	Traes_5AL_B4E8A3115.4	PF03106,PF12796,PF00931	KOG4658			GO:GO:0043565,GO:GO:0006355,GO:GO:0003700,GO:GO:0043531	AT2G31820.1	Ankyrin repeat family protein	LOC_Os11g45750.2	WRKY125, expressed	1,05	-1.0	1	22	2324	2345	AUAAAACCUUCAGCUAUCCAUC	AAUGGAUAGCUGAAGGUCUUAU	Cleavage
tae-miR7757-5p	Traes_5AL_B4E8A3115.5	PF03106,PF12796,PF00931	KOG4658			GO:GO:0043565,GO:GO:0006355,GO:GO:0003700,GO:GO:0043531	AT2G31820.1	Ankyrin repeat family protein	LOC_Os11g45750.1	WRKY125, expressed	1,05	-1.0	1	22	2324	2345	AUAAAACCUUCAGCUAUCCAUC	AAUGGAUAGCUGAAGGUCUUAU	Cleavage
tae-miR7757-5p	Traes_5AL_B4E8A3115.6	PF00931,PF03106				GO:GO:0043531,GO:GO:0043565,GO:GO:0006355,GO:GO:0003700	AT3G46710.1	NB-ARC domain-containing disease resistance protein	LOC_Os05g40150.1	RGH2B, putative, expressed	1,05	-1.0	1	22	368	389	AUAAAACCUUCAGCUAUCCAUC	AAUGGAUAGCUGAAGGUCUUAU	Cleavage
tae-miR9652-5p	Traes_5AL_C9236F111.1										2,05	-1.0	1	22	778	799	CCUGUUUGUCAUUAAGUUUCUU	AAGAAACUGAAUGACAAGCAGG	Cleavage
tae-miR5384-3p	Traes_5AS_A928BA201.1										2,05	-1.0	1	21	506	526	UGAGCGCGCCGCCGUCGAAUG	CAUUCGUCGGCGUCGCGCUCA	Cleavage
tae-miR7757-5p	Traes_5AS_BDFB08E23.1	PF00931	KOG4658			GO:GO:0043531	AT3G07040.1	NB-ARC domain-containing disease resistance protein	LOC_Os12g37740.1	resistance protein LR10, putative, expressed	2,05	-1.0	1	22	1349	1370	AUAAAACCUUCAGCUAUCCAUC	GAUGGAUUGCUGAAGGUUUCAU	Cleavage
tae-miR7757-5p	Traes_5AS_BDFB08E23.2	PF00931	KOG4658			GO:GO:0043531	AT3G07040.1	NB-ARC domain-containing disease resistance protein	LOC_Os12g37740.1	resistance protein LR10, putative, expressed	2,05	-1.0	1	22	1349	1370	AUAAAACCUUCAGCUAUCCAUC	GAUGGAUUGCUGAAGGUUUCAU	Cleavage
tae-miR7757-5p	Traes_5AS_BDFB08E23.3	PF00931				GO:GO:0043531	AT3G07040.1	NB-ARC domain-containing disease resistance protein	LOC_Os12g37740.1	resistance protein LR10, putative, expressed	2,05	-1.0	1	22	524	545	AUAAAACCUUCAGCUAUCCAUC	GAUGGAUUGCUGAAGGUUUCAU	Cleavage
tae-miR7757-5p	Traes_5AS_BDFB08E23.4	PF00931				GO:GO:0043531	AT3G46530.1	NB-ARC domain-containing disease resistance protein	LOC_Os12g37740.1	resistance protein LR10, putative, expressed	2,05	-1.0	1	22	1349	1370	AUAAAACCUUCAGCUAUCCAUC	GAUGGAUUGCUGAAGGUUUCAU	Cleavage
tae-miR7757-5p	Traes_5AS_BDFB08E23.5	PF00931				GO:GO:0043531	AT3G46530.1	NB-ARC domain-containing disease resistance protein	LOC_Os12g37740.1	resistance protein LR10, putative, expressed	2,05	-1.0	1	22	1349	1370	AUAAAACCUUCAGCUAUCCAUC	GAUGGAUUGCUGAAGGUUUCAU	Cleavage
tae-miR1128	Traes_5AS_E1F692526.2										3,00	-1.0	1	21	111	132	UACUACUCCCUCCGUCCG-AA	UUAUGGGACGGAGGGAGUAGUA	Cleavage
tae-miR1130b-3p	Traes_5AS_E1F692526.2										3,00	-1.0	1	21	40	61	UCUU-AUAUUAUGGGACGGAGG	CCUCCGUCCCAUAAUAUUAAGA	Cleavage
tae-miR1122c-3p	Traes_5AS_E1F692526.2										3,00	-1.0	1	21	40	61	UC-UAAUAUUAUGGGACGGAGG	CCUCCGUCCCAUAAUAUUAAGA	Cleavage
tae-miR164	Traes_5AS_E5FD82E36.1										1,00	-1.0	1	21	653	673	UGGAGAAGCAGGGCACGUGCA	AGCAAGUGCCCUGCUUCUCCA	Cleavage
tae-miR164	Traes_5AS_E5FD82E36.2										1,00	-1.0	1	21	656	676	UGGAGAAGCAGGGCACGUGCA	AGCAAGUGCCCUGCUUCUCCA	Cleavage
tae-miR1118	Traes_5AS_EC41EF7CA.4										1,05	-1.0	1	23	1333	1355	CACUACAUUAUGGAAUGGAGGGA	UCCCUCCAUUCCACAAUGUAGUG	Translation
tae-miR1125	Traes_5AS_EC41EF7CA.4										1,00	-1.0	1	24	1450	1473	AACCAACGAGACCAACUGCGGCGG	CCGCCGCAGUCGGUCUCGUUGGUU	Cleavage
tae-miR408	Traes_5BL_0212ACBD9.1										2,05	-1.0	1	21	2512	2532	CUGCACUGCCUCUUCCCUGGC	GCCAAGGAAGAGGCUGUGCAG	Cleavage
tae-miR1137a	Traes_5BL_0EC4788EF.2										1,00	-1.0	1	20	1543	1562	UAGUACAAAGUUGAGUCAUC	GAUGACCCAACUUUGUACUA	Cleavage

tae-miR9652-5p	Traes_5BL_0F64FDA44.1										2,05	-1.0	1	22	583	604	CCUGUUUGUCAUUAAGUUUCUU	AAGAAACUGAAUGACAAGCAGG	Cleavage
tae-miR1122a	Traes_5BL_1B9186ED8.1										2,05	-1.0	1	20	986	1005	UAGAUACAUCCGUAUCUAGA	UCGAGAUACAGAUGUAUCUA	Translation
tae-miR7757-5p	Traes_5BL_1E751EF1F.1	PF00931,PF03514	KOG4658			GO:GO:0043531	AT1G50600.1	scarecrow-like 5	LOC_Os10g22484.1	RGH2B, putative, expressed	2,00	-1.0	1	22	1799	1820	AUAAAACCUUCAGCUAUCCAUC	AAUGGGUUGCUGAAGGUUUUAU	Cleavage
tae-miR7757-5p	Traes_5BL_1E751EF1F.2	PF00931				GO:GO:0043531	AT3G46730.1	NB-ARC domain-containing disease resistance protein	LOC_Os10g22484.1	RGH2B, putative, expressed	2,00	-1.0	1	22	1799	1820	AUAAAACCUUCAGCUAUCCAUC	AAUGGGUUGCUGAAGGUUUUAU	Cleavage
tae-miR7757-5p	Traes_5BL_1E751EF1F.5	PF00931,PF03514				GO:GO:0043531	AT1G50600.1	scarecrow-like 5	LOC_Os10g22484.1	RGH2B, putative, expressed	2,00	-1.0	1	22	512	533	AUAAAACCUUCAGCUAUCCAUC	AAUGGGUUGCUGAAGGUUUUAU	Cleavage
tae-miR5049-3p	Traes_5BL_4BFE76791.5										2,05	-1.0	1	21	416	436	AAUAUGGAUCGGAGGGAGUAC	AUGCUCCCUCUGAUCCAUAUU	Translation
tae-miR1127b-3p	Traes_5BL_5E94C243F.1										2,05	-1.0	1	21	914	934	ACAAGUAUUUCUGGACGGAGG	CCUCUGUCCUGAAAUACUUGU	Cleavage
tae-miR1127b-3p	Traes_5BL_5E94C243F.2										2,05	-1.0	1	21	898	918	ACAAGUAUUUCUGGACGGAGG	CCUCUGUCCUGAAAUACUUGU	Cleavage
tae-miR9674b-5p	Traes_5BL_6216A6108.1										3,00	-1.0	1	21	411	431	AUAGCAUCAUCCAUCCUACCC	GGGCCGAAUGGAUGAUGCUAU	Cleavage
tae-miR9674b-5p	Traes_5BL_6216A6108.1										3,00	-1.0	1	21	411	431	AUAGCAUCAUCCAUCCUACCC	GGGCCGAAUGGAUGAUGCUAU	Cleavage
tae-miR5384-3p	Traes_5BL_6240DE6BA.1										2,05	-1.0	1	21	338	358	UGAGCGCGCCGCCGUCGAAUG	CCUUCGACGGCGCCGCGCUCG	Cleavage
tae-miR5384-3p	Traes_5BL_6240DE6BA.2										2,05	-1.0	1	21	338	358	UGAGCGCGCCGCCGUCGAAUG	CCUUCGACGGCGCCGCGCUCG	Cleavage
tae-miR5384-3p	Traes_5BL_6240DE6BA.3										2,05	-1.0	1	21	338	358	UGAGCGCGCCGCCGUCGAAUG	CCUUCGACGGCGCCGCGCUCG	Cleavage
tae-miR5384-3p	Traes_5BL_6240DE6BA.4										2,05	-1.0	1	21	338	358	UGAGCGCGCCGCCGUCGAAUG	CCUUCGACGGCGCCGCGCUCG	Cleavage
tae-miR1122a	Traes_5BL_637A3C970.8										1,05	-1.0	1	20	49	68	UAGAUACAUCCGUAUCUAGA	UCUAGAUACGAAUGUAUCUA	Translation
tae-miR1121	Traes_5BL_72EFF0AF0.1										0,00	-1.0	1	22	1237	1258	AGUAGUGAUCUAAACGCUCUUA	UAAGAGCGUUUAGAUCACUACU	Cleavage
tae-miR408	Traes_5BL_7486582EB.1										2,05	-1.0	1	21	8	28	CUGCACUGCCUCUUCCCUGGC	CGCAGGGAAGAGGCAGUGCCA	Cleavage
tae-miR1122a	Traes_5BL_7821274AB.1										2,05	-1.0	1	20	986	1005	UAGAUACAUCCGUAUCUAGA	UCGAGAUACAGAUGUAUCUA	Translation
tae-miR7757-5p	Traes_5BL_7F0FD1538.2	PF00847,PF00931	KOG4658			GO:GO:0006355,GO:GO:0003700,GO:GO:0043531	AT3G46710.1	NB-ARC domain-containing disease resistance protein	LOC_Os05g40150.1	RGH2B, putative, expressed	1,05	-1.0	1	22	2096	2117	AUAAAACCUUCAGCUAUCCAUC	AAUGGAUAGCUGAAAGUUUUAU	Cleavage
tae-miR7757-5p	Traes_5BL_7F0FD1538.3	PF00847,PF00931	KOG4658			GO:GO:0006355,GO:GO:0003700,GO:GO:0043531	AT3G46710.1	NB-ARC domain-containing disease resistance protein	LOC_Os05g40150.1	RGH2B, putative, expressed	1,05	-1.0	1	22	1379	1400	AUAAAACCUUCAGCUAUCCAUC	AAUGGAUAGCUGAAAGUUUUAU	Cleavage
tae-miR7757-5p	Traes_5BL_7F0FD1538.4	PF00931	KOG4658			GO:GO:0043531	AT3G46710.1	NB-ARC domain-containing disease resistance protein	LOC_Os05g40150.1	RGH2B, putative, expressed	1,05	-1.0	1	22	1379	1400	AUAAAACCUUCAGCUAUCCAUC	AAUGGAUAGCUGAAAGUUUUAU	Cleavage
tae-miR156	Traes_5BL_96D5EA4EE.1	PF03110				GO:GO:0005634,GO:GO:0003677	AT2G42200.1	squamosa promoter binding protein-like 9	LOC_Os09g31438.1	OsSPL17 - SBP-box gene family member, expressed	1,00	-1.0	1	21	805	825	UGACAGAAGAGAGUGAGCACA	UGUGCUCUCUCUCUUCUGUCA	Cleavage
tae-miR156	Traes_5BL_96D5EA4EE.2	PF03110				GO:GO:0005634,GO:GO:0003677	AT2G42200.1	squamosa promoter binding protein-like 9	LOC_Os09g31438.1	OsSPL17 - SBP-box gene family member, expressed	1,00	-1.0	1	21	802	822	UGACAGAAGAGAGUGAGCACA	UGUGCUCUCUCUCUUCUGUCA	Cleavage
tae-miR156	Traes_5BL_96D5EA4EE.3								LOC_Os09g31438.1	OsSPL17 - SBP-box gene family member, expressed	1,00	-1.0	1	21	67	87	UGACAGAAGAGAGUGAGCACA	UGUGCUCUCUCUCUUCUGUCA	Cleavage
tae-miR156	Traes_5BL_96FDFC152.1	PF03110				GO:GO:0005634,GO:GO:0003677	AT1G76580.1	Squamosa promoter-binding protein-like (SBP domain) transcription factor family protein	LOC_Os09g32944.1	OsSPL18 - SBP-box gene family member, expressed	1,00	-1.0	1	21	508	528	UGACAGAAGAGAGUGAGCACA	UGUGCUCUCUCUCUUCUGUCA	Cleavage
tae-miR156	Traes_5BL_96FDFC152.2	PF03110				GO:GO:0005634,GO:GO:0003677	AT5G50570.1	Squamosa promoter-binding protein-like (SBP domain) transcription factor family protein	LOC_Os09g32944.1	OsSPL18 - SBP-box gene family member, expressed	1,00	-1.0	1	21	490	510	UGACAGAAGAGAGUGAGCACA	UGUGCUCUCUCUCUUCUGUCA	Cleavage
tae-miR5384-3p	Traes_5BL_AD734CCEF.1										2,05	-1.0	1	21	242	262	UGAGCGCGCCGCCGUCGAAUG	UCUUCCACGGCGGCGCGUUCA	Cleavage
tae-miR9664-3p	Traes_5BL_D27214492.1	PF00931				GO:GO:0043531	AT3G46530.1	NB-ARC domain-containing disease resistance protein	LOC_Os11g11550.1	NBS-LRR disease resistance protein, putative, expressed	2,05	-1.0	1	21	237	257	UUGCAGUCCUCGAUGUCGUAG	GCACGACAUCGAGGAUUGCAU	Cleavage
tae-miR9664-3p	Traes_5BL_D27214492.2	PF00931				GO:GO:0043531	AT3G46530.1	NB-ARC domain-containing disease resistance protein	LOC_Os11g11550.2	NBS-LRR disease resistance protein, putative, expressed	2,05	-1.0	1	21	237	257	UUGCAGUCCUCGAUGUCGUAG	GCACGACAUCGAGGAUUGCAU	Cleavage
tae-miR9664-3p	Traes_5BL_D27214492.3	PF00931				GO:GO:0043531	AT3G46530.1	NB-ARC domain-containing disease resistance protein	LOC_Os11g11550.2	NBS-LRR disease resistance protein, putative, expressed	2,05	-1.0	1	21	237	257	UUGCAGUCCUCGAUGUCGUAG	GCACGACAUCGAGGAUUGCAU	Cleavage
tae-miR9664-3p	Traes_5BL_D27214492.4	PF00931				GO:GO:0043531	AT3G46530.1	NB-ARC domain-containing disease resistance protein	LOC_Os11g11550.2	NBS-LRR disease resistance protein, putative, expressed	2,05	-1.0	1	21	237	257	UUGCAGUCCUCGAUGUCGUAG	GCACGACAUCGAGGAUUGCAU	Cleavage
tae-miR9664-3p	Traes_5BL_D27214492.5	PF00931				GO:GO:0043531	AT3G46530.1	NB-ARC domain-containing disease resistance protein	LOC_Os11g11550.1	NBS-LRR disease resistance protein, putative, expressed	2,05	-1.0	1	21	237	257	UUGCAGUCCUCGAUGUCGUAG	GCACGACAUCGAGGAUUGCAU	Cleavage
tae-miR7757-5p	Traes_5BL_DD4C9F446.1	PF00069	KOG1187	2.7.11.1		GO:GO:0006468,GO:GO:0005524,GO:GO:0004672	AT3G55950.1	CRINKLY4 related 3	LOC_Os05g40150.1	RGH2B, putative, expressed	1,05	-1.0	1	22	62	83	AUAAAACCUUCAGCUAUCCAUC	AAUGGAUAGCGGAAGGUUUUAU	Cleavage
tae-miR7757-5p	Traes_5BL_DD4C9F446.2	PF00069	KOG1187	2.7.11.1		GO:GO:0006468,GO:GO:0005524,GO:GO:0004672	AT3G55950.1	CRINKLY4 related 3	LOC_Os05g40150.1	RGH2B, putative, expressed	1,05	-1.0	1	22	62	83	AUAAAACCUUCAGCUAUCCAUC	AAUGGAUAGCGGAAGGUUUUAU	Cleavage
tae-miR7757-5p	Traes_5BL_DD4C9F446.4						AT3G46710.1	NB-ARC domain-containing disease resistance protein	LOC_Os05g40150.1	RGH2B, putative, expressed	1,05	-1.0	1	22	62	83	AUAAAACCUUCAGCUAUCCAUC	AAUGGAUAGCGGAAGGUUUUAU	Cleavage
tae-miR7757-5p	Traes_5BL_DD4C9F446.5						AT3G46710.1	NB-ARC domain-containing disease resistance protein	LOC_Os05g40150.1	RGH2B, putative, expressed	1,05	-1.0	1	22	62	83	AUAAAACCUUCAGCUAUCCAUC	AAUGGAUAGCGGAAGGUUUUAU	Cleavage
tae-miR7757-5p	Traes_5BL_DD4C9F446.6						AT3G46710.1	NB-ARC domain-containing disease resistance protein	LOC_Os05g40150.1	RGH2B, putative, expressed	1,05	-1.0	1	22	62	83	AUAAAACCUUCAGCUAUCCAUC	AAUGGAUAGCGGAAGGUUUUAU	Cleavage
tae-miR9661-5p	Traes_5BL_E0C2C7D72.1										3,00	-1.0	1	21	98	118	UGAAGUAGAGCAGGGACCUCA	UCGUCUCCCUGCUCUACUUCA	Cleavage
tae-miR9661-5p	Traes_5BL_E0C2C7D72.2										3,00	-1.0	1	21	98	118	UGAAGUAGAGCAGGGACCUCA	UCGUCUCCCUGCUCUACUUCA	Cleavage
tae-miR9661-5p	Traes_5BL_E0C2C7D72.3										3,00	-1.0	1	21	95	115	UGAAGUAGAGCAGGGACCUCA	UCGUCUCCCUGCUCUACUUCA	Cleavage
tae-miR9652-5p	Traes_5BL_FC199BC2D.1										2,05	-1.0	1	22	412	433	CCUGUUUGUCAUUAAGUUUCUU	AAGAAACUGAAUGACAAGCAGG	Cleavage
tae-miR1122b-3p	Traes_5BS_321AF7B69.2										2,05	-1.0	1	21	23	43	AGACUUAUAUGUAGGAACGGA	UCCAUUCCUAAAUAUAAGUCU	Translation
tae-miR1122a	Traes_5BS_321AF7B69.2										2,05	-1.0	1	20	63	82	UAGAUACAUCCGUAUCUAGA	ACUACAUACGGAUGUAUAUA	Cleavage
tae-miR7757-5p	Traes_5BS_3FFCC89AD.1	PF00931	KOG4658			GO:GO:0043531	AT3G07040.1	NB-ARC domain-containing disease resistance protein	LOC_Os12g37770.1	RGH1A, putative, expressed	2,05	-1.0	1	22	1337	1358	AUAAAACCUUCAGCUAUCCAUC	GGUGGAUUGCUGAAGGAUUUAU	Cleavage
tae-miR7757-5p	Traes_5BS_3FFCC89AD.2	PF00931	KOG4658			GO:GO:0043531	AT3G46730.1	NB-ARC domain-containing disease resistance protein	LOC_Os12g37770.1	RGH1A, putative, expressed	2,05	-1.0	1	22	1298	1319	AUAAAACCUUCAGCUAUCCAUC	GGUGGAUUGCUGAAGGAUUUAU	Cleavage
tae-miR1118	Traes_5BS_550BDEE86.2										1,05	-1.0	1	23	1135	1157	CACUACAUUAUGGAAUGGAGGGA	UCCCUCCAUUCCACAAUGUAGUG	Translation
tae-miR5384-3p	Traes_5BS_90796C6CA.1										2,00	-1.0	1	21	461	481	UGAGCGCGCCGCCGUCGAAUG	UGUACGAUGGCGGCGCGCUCA	Cleavage
tae-miR5384-3p	Traes_5BS_90796C6CA.2										2,00	-1.0	1	21	34	54	UGAGCGCGCCGCCGUCGAAUG	UGUACGAUGGCGGCGCGCUCA	Cleavage
tae-miR5384-3p	Traes_5BS_90796C6CA.3										2,00	-1.0	1	21	392	412	UGAGCGCGCCGCCGUCGAAUG	UGUACGAUGGCGGCGCGCUCA	Cleavage
tae-miR164	Traes_5BS_9F043AE17.1										1,00	-1.0	1	21	656	676	UGGAGAAGCAGGGCACGUGCA	AGCAAGUGCCCUGCUUCUCCA	Cleavage
tae-miR5384-3p	Traes_5BS_BA040DE10.1										3,00	-1.0	1	21	1358	1378	UGAGCGCGCCGCCGUCGAAUG	CCGACGACGGCGGCGCGCUCC	Cleavage
tae-miR7757-5p	Traes_5BS_BCEA0112C.1	PF00931	KOG4658			GO:GO:0043531	AT3G07040.1	NB-ARC domain-containing disease resistance protein	LOC_Os12g37740.1	resistance protein LR10, putative, expressed	2,05	-1.0	1	22	1340	1361	AUAAAACCUUCAGCUAUCCAUC	GAUGGAUCGCGGAAGGUUUUAU	Cleavage
tae-miR7757-5p	Traes_5BS_BCEA0112C.2	PF00931	KOG4658			GO:GO:0043531	AT3G07040.1	NB-ARC domain-containing disease resistance protein	LOC_Os12g37740.1	resistance protein LR10, putative, expressed	2,05	-1.0	1	22	1340	1361	AUAAAACCUUCAGCUAUCCAUC	GAUGGAUCGCGGAAGGUUUUAU	Cleavage
tae-miR7757-5p	Traes_5BS_BCEA0112C.3	PF00931	KOG4658			GO:GO:0043531	AT3G07040.1	NB-ARC domain-containing disease resistance protein	LOC_Os12g37740.1	resistance protein LR10, putative, expressed	2,05	-1.0	1	22	1307	1328	AUAAAACCUUCAGCUAUCCAUC	GAUGGAUCGCGGAAGGUUUUAU	Cleavage
tae-miR7757-5p	Traes_5BS_BCEA0112C.4	PF00931				GO:GO:0043531	AT3G07040.1	NB-ARC domain-containing disease resistance protein	LOC_Os12g37740.1	resistance protein LR10, putative, expressed	2,05	-1.0	1	22	128	149	AUAAAACCUUCAGCUAUCCAUC	GAUGGAUCGCGGAAGGUUUUAU	Cleavage
tae-miR408	Traes_5BS_E10756355.1										2,05	-1.0	1	21	2512	2532	CUGCACUGCCUCUUCCCUGGC	GCCAAGGAAGAGGCUGUGCAG	Cleavage
tae-miR7757-5p	Traes_5BS_F5AFB24CD.2	PF00931				GO:GO:0043531	AT3G46530.1	NB-ARC domain-containing disease resistance protein	LOC_Os10g03570.1	RGH1A, putative, expressed	1,05	-1.0	1	22	779	800	AUAAAACCUUCAGCUAUCCAUC	GGUGGAUAGCUGAAGGUCUUAU	Cleavage
tae-miR7757-5p	Traes_5BS_F5AFB24CD.3	PF00931				GO:GO:0043531	AT3G46530.1	NB-ARC domain-containing disease resistance protein	LOC_Os10g03570.1	RGH1A, putative, expressed	1,05	-1.0	1	22	779	800	AUAAAACCUUCAGCUAUCCAUC	GGUGGAUAGCUGAAGGUCUUAU	Cleavage
tae-miR7757-5p	Traes_5BS_F5AFB24CD.4	PF00931				GO:GO:0043531	AT5G43470.2	Disease resistance protein (CC-NBS-LRR class) family	LOC_Os10g03570.1	RGH1A, putative, expressed	1,05	-1.0	1	22	371	392	AUAAAACCUUCAGCUAUCCAUC	GGUGGAUAGCUGAAGGUCUUAU	Cleavage
tae-miR156	Traes_5DL_1272B4BAC.1	PF03110				GO:GO:0005634,GO:GO:0003677	AT5G50670.1	Squamosa promoter-binding protein-like (SBP domain) transcription factor family protein	LOC_Os09g32944.1	OsSPL18 - SBP-box gene family member, expressed	1,00	-1.0	1	21	505	525	UGACAGAAGAGAGUGAGCACA	UGUGCUCUCUCUCUUCUGUCA	Cleavage
tae-miR156	Traes_5DL_1272B4BAC.2	PF03110				GO:GO:0005634,GO:GO:0003677	AT5G50670.1	Squamosa promoter-binding protein-like (SBP domain) transcription factor family protein	LOC_Os09g32944.1	OsSPL18 - SBP-box gene family member, expressed	1,00	-1.0	1	21	487	507	UGACAGAAGAGAGUGAGCACA	UGUGCUCUCUCUCUUCUGUCA	Cleavage
tae-miR156	Traes_5DL_1272B4BAC.3								LOC_Os09g32944.1	OsSPL18 - SBP-box gene family member, expressed	1,00	-1.0	1	21	382	402	UGACAGAAGAGAGUGAGCACA	UGUGCUCUCUCUCUUCUGUCA	Cleavage
tae-miR7757-5p	Traes_5DL_1733FB4DA.1	PF00931,PF03106				GO:GO:0043531,GO:GO:0043565,GO:GO:0006355,GO:GO:0003700	AT3G46530.1	NB-ARC domain-containing disease resistance protein	LOC_Os05g40150.1	RGH2B, putative, expressed	1,05	-1.0	1	22	617	638	AUAAAACCUUCAGCUAUCCAUC	AAUGGAUAGCUGAAGGAUUUAU	Cleavage
tae-miR7757-5p	Traes_5DL_1733FB4DA.2	PF03106,PF00931				GO:GO:0043565,GO:GO:0006355,GO:GO:0003700,GO:GO:0043531	AT3G46530.1	NB-ARC domain-containing disease resistance protein	LOC_Os05g40150.1	RGH2B, putative, expressed	1,05	-1.0	1	22	617	638	AUAAAACCUUCAGCUAUCCAUC	AAUGGAUAGCUGAAGGAUUUAU	Cleavage
tae-miR7757-5p	Traes_5DL_1733FB4DA.3	PF03106,PF00931				GO:GO:0043565,GO:GO:0006355,GO:GO:0003700,GO:GO:0043531	AT3G46530.1	NB-ARC domain-containing disease resistance protein	LOC_Os05g40150.1	RGH2B, putative, expressed	1,05	-1.0	1	22	617	638	AUAAAACCUUCAGCUAUCCAUC	AAUGGAUAGCUGAAGGAUUUAU	Cleavage
tae-miR7757-5p	Traes_5DL_1733FB4DA.4	PF00931				GO:GO:0043531	AT3G46530.1	NB-ARC domain-containing disease resistance protein	LOC_Os05g40150.1	RGH2B, putative, expressed	1,05	-1.0	1	22	617	638	AUAAAACCUUCAGCUAUCCAUC	AAUGGAUAGCUGAAGGAUUUAU	Cleavage
tae-miR7757-5p	Traes_5DL_1733FB4DA.5	PF00931				GO:GO:0043531	AT3G46530.1	NB-ARC domain-containing disease resistance protein	LOC_Os05g40150.1	RGH2B, putative, expressed	1,05	-1.0	1	22	617	638	AUAAAACCUUCAGCUAUCCAUC	AAUGGAUAGCUGAAGGAUUUAU	Cleavage
tae-miR7757-5p	Traes_5DL_1733FB4DA.6	PF00931				GO:GO:0043531	AT3G46530.1	NB-ARC domain-containing disease resistance protein	LOC_Os05g40150.1	RGH2B, putative, expressed	1,05	-1.0	1	22	617	638	AUAAAACCUUCAGCUAUCCAUC	AAUGGAUAGCUGAAGGAUUUAU	Cleavage
tae-miR7757-5p	Traes_5DL_1733FB4DA.7	PF00931				GO:GO:0043531	AT3G46530.1	NB-ARC domain-containing disease resistance protein	LOC_Os11g45750.1	WRKY125, expressed	1,05	-1.0	1	22	617	638	AUAAAACCUUCAGCUAUCCAUC	AAUGGAUAGCUGAAGGAUUUAU	Cleavage
tae-miR7757-5p	Traes_5DL_1733FB4DA.8	PF00931				GO:GO:0043531	AT3G46530.1	NB-ARC domain-containing disease resistance protein	LOC_Os11g45750.2	WRKY125, expressed	1,05	-1.0	1	22	617	638	AUAAAACCUUCAGCUAUCCAUC	AAUGGAUAGCUGAAGGAUUUAU	Cleavage
tae-miR1127b-3p	Traes_5DL_4E67E0243.9										1,05	-1.0	1	21	41	61	ACAAGUAUUUCUGGACGGAGG	CCUCCGUCCGGAAAUACUUGU	Cleavage
tae-miR9652-5p	Traes_5DL_714CCB5BC.1										2,05	-1.0	1	22	412	433	CCUGUUUGUCAUUAAGUUUCUU	AAGAAACUGAAUGACAAGCAGG	Cleavage
tae-miR9662a-3p	Traes_5DL_7643B2ADB.1	PF02536				GO:GO:0006355,GO:GO:0005739,GO:GO:0003690	AT5G64950.1	Mitochondrial transcription termination factor family protein	LOC_Os06g12100.1	mTERF family protein, expressed	2,05	-1.0	1	21	411	431	UUGAACAUCCCAGAGCCACCG	CCGUGGCUCUGUGAUGUUCAG	Translation
tae-miR9662b-3p	Traes_5DL_7643B2ADB.1	PF02536				GO:GO:0006355,GO:GO:0005739,GO:GO:0003690	AT5G64950.1	Mitochondrial transcription termination factor family protein	LOC_Os06g12100.1	mTERF family protein, expressed	2,05	-1.0	1	21	410	430	UGAACAUCCCAGAGCCACCGG	CCCGUGGCUCUGUGAUGUUCA	Cleavage
tae-miR5384-3p	Traes_5DL_78767F75C.1										2,05	-1.0	1	21	242	262	UGAGCGCGCCGCCGUCGAAUG	UCUUCCACGGCGGCGCGUUCA	Cleavage
tae-miR5049-3p	Traes_5DL_799647EA7.4										1,05	-1.0	1	21	65	85	AAUAUGGAUCGGAGGGAGUAC	GUACUCCCUCCGUUCCAUAUU	Cleavage
tae-miR9652-5p	Traes_5DL_8DE6C67FA.1										2,05	-1.0	1	22	412	433	CCUGUUUGUCAUUAAGUUUCUU	AAGAAACUGAAUGACAAGCAGG	Cleavage
tae-miR9652-5p	Traes_5DL_8DE6C67FA.2										2,05	-1.0	1	22	412	433	CCUGUUUGUCAUUAAGUUUCUU	AAGAAACUGAAUGACAAGCAGG	Cleavage
tae-miR9652-5p	Traes_5DL_8DE6C67FA.3										2,05	-1.0	1	22	415	436	CCUGUUUGUCAUUAAGUUUCUU	AAGAAACUGAAUGACAAGCAGG	Cleavage
tae-miR9652-5p	Traes_5DL_8DE6C67FA.4										2,05	-1.0	1	22	400	421	CCUGUUUGUCAUUAAGUUUCUU	AAGAAACUGAAUGACAAGCAGG	Cleavage
tae-miR9652-5p	Traes_5DL_8DE6C67FA.5										2,05	-1.0	1	22	400	421	CCUGUUUGUCAUUAAGUUUCUU	AAGAAACUGAAUGACAAGCAGG	Cleavage
tae-miR408	Traes_5DL_90BD9F306.1										0,00	-1.0	1	21	8	28	CUGCACUGCCUCUUCCCUGGC	CCCAGGGAAGAGGCAGUGCAG	Cleavage
tae-miR9666b-3p	Traes_5DL_9101D5AEC.1										2,05	-1.0	1	22	1183	1204	CGGUUGGGCUGUAUGAUGGCGA	AGUCCAUCAUACAGCCCAACAA	Cleavage
tae-miR1130b-3p	Traes_5DL_A6EECBC04.1										1,05	-1.0	1	21	376	396	UCUUAUAUUAUUGGACGGAGG	AUUCCGUCCCAUAAUGUAAGA	Cleavage
tae-miR9652-5p	Traes_5DL_B3713A4D1.1										2,05	-1.0	1	22	940	961	CCUGUUUGUCAUUAAGUUUCUU	AAGAAACUGAAUGACAAGCAGG	Cleavage
tae-miR7757-5p	Traes_5DS_0479C6E0F.1	PF00931				GO:GO:0043531	AT3G07040.1	NB-ARC domain-containing disease resistance protein	LOC_Os12g06920.1	NBS-LRR disease resistance protein, putative, expressed	2,05	-1.0	1	22	383	404	AUAAAACCUUCAGCUAUCCAUC	GAUGGAUCGCUGAAGGUUUGAU	Cleavage
tae-miR9662a-3p	Traes_5DS_094A03C13.1	PF02536				GO:GO:0006355,GO:GO:0005739,GO:GO:0003690	AT5G07900.1	Mitochondrial transcription termination factor family protein	LOC_Os06g12100.1	mTERF family protein, expressed	1,00	-1.0	1	21	549	569	UUGAACAUCCCAGAGCCACCG	CCGUGGCUCUGGGAUGUUCAG	Cleavage
tae-miR9662b-3p	Traes_5DS_094A03C13.1	PF02536				GO:GO:0006355,GO:GO:0005739,GO:GO:0003690	AT5G07900.1	Mitochondrial transcription termination factor family protein	LOC_Os06g12100.1	mTERF family protein, expressed	1,00	-1.0	1	21	548	568	UGAACAUCCCAGAGCCACCGG	CCCGUGGCUCUGGGAUGUUCA	Cleavage
tae-miR9662a-3p	Traes_5DS_094A03C13.2	PF02536				GO:GO:0006355,GO:GO:0005739,GO:GO:0003690	AT5G07900.1	Mitochondrial transcription termination factor family protein	LOC_Os06g12100.1	mTERF family protein, expressed	1,00	-1.0	1	21	243	263	UUGAACAUCCCAGAGCCACCG	CCGUGGCUCUGGGAUGUUCAG	Cleavage
tae-miR9662b-3p	Traes_5DS_094A03C13.2	PF02536				GO:GO:0006355,GO:GO:0005739,GO:GO:0003690	AT5G07900.1	Mitochondrial transcription termination factor family protein	LOC_Os06g12100.1	mTERF family protein, expressed	1,00	-1.0	1	21	242	262	UGAACAUCCCAGAGCCACCGG	CCCGUGGCUCUGGGAUGUUCA	Cleavage
tae-miR5384-3p	Traes_5DS_149714893.1										2,05	-1.0	1	21	158	178	UGAGCGCGCCGCCGUCGAAUG	CGUUCGUCGGCGUCGCGCUCA	Cleavage
tae-miR5384-3p	Traes_5DS_149714893.2										2,05	-1.0	1	21	152	172	UGAGCGCGCCGCCGUCGAAUG	CGUUCGUCGGCGUCGCGCUCA	Cleavage
tae-miR164	Traes_5DS_511F35A45.1										1,00	-1.0	1	21	209	229	UGGAGAAGCAGGGCACGUGCA	AGCAAGUGCCCUGCUUCUCCA	Cleavage
tae-miR7757-5p	Traes_5DS_8DB03B7DA.1	PF00931	KOG4658			GO:GO:0043531	AT3G07040.1	NB-ARC domain-containing disease resistance protein	LOC_Os12g37740.1	resistance protein LR10, putative, expressed	2,05	-1.0	1	22	1349	1370	AUAAAACCUUCAGCUAUCCAUC	GAUGGAUCGCGGAAGGUUUUAU	Cleavage
tae-miR7757-5p	Traes_5DS_8DB03B7DA.2	PF00931				GO:GO:0043531	AT3G07040.1	NB-ARC domain-containing disease resistance protein	LOC_Os12g37740.1	resistance protein LR10, putative, expressed	2,05	-1.0	1	22	128	149	AUAAAACCUUCAGCUAUCCAUC	GAUGGAUCGCGGAAGGUUUUAU	Cleavage
tae-miR7757-5p	Traes_5DS_9DFE4D217.1	PF00931				GO:GO:0043531	AT1G53350.1	Disease resistance protein (CC-NBS-LRR class) family	LOC_Os10g22484.1	RGH2B, putative, expressed	2,05	-1.0	1	22	185	206	AUAAAACCUUCAGCUAUCCAUC	AAUGGGUAGCUGAAGGUUGUAU	Cleavage
tae-miR7757-5p	Traes_5DS_9DFE4D217.2	PF00931				GO:GO:0043531	AT1G53350.1	Disease resistance protein (CC-NBS-LRR class) family	LOC_Os10g22484.1	RGH2B, putative, expressed	2,05	-1.0	1	22	185	206	AUAAAACCUUCAGCUAUCCAUC	AAUGGGUAGCUGAAGGUUGUAU	Cleavage
tae-miR7757-5p	Traes_5DS_9DFE4D217.3	PF00931				GO:GO:0043531	AT1G53350.1	Disease resistance protein (CC-NBS-LRR class) family	LOC_Os10g22484.1	RGH2B, putative, expressed	2,05	-1.0	1	22	185	206	AUAAAACCUUCAGCUAUCCAUC	AAUGGGUAGCUGAAGGUUGUAU	Cleavage
tae-miR9662a-3p	Traes_5DS_9F4A5AE9F.1	PF02536				GO:GO:0006355,GO:GO:0005739,GO:GO:0003690	AT5G07900.1	Mitochondrial transcription termination factor family protein	LOC_Os06g12100.1	mTERF family protein, expressed	2,00	-1.0	1	21	441	461	UUGAACAUCCCAGAGCCACCG	UCGUGGCGCUGGGAUGUUCAG	Cleavage
tae-miR9662b-3p	Traes_5DS_9F4A5AE9F.1	PF02536				GO:GO:0006355,GO:GO:0005739,GO:GO:0003690	AT5G07900.1	Mitochondrial transcription termination factor family protein	LOC_Os06g12100.1	mTERF family protein, expressed	2,05	-1.0	1	21	440	460	UGAACAUCCCAGAGCCACCGG	CUCGUGGCGCUGGGAUGUUCA	Cleavage
tae-miR9662a-3p	Traes_5DS_9F4A5AE9F.2	PF02536				GO:GO:0006355,GO:GO:0005739,GO:GO:0003690	AT5G07900.1	Mitochondrial transcription termination factor family protein	LOC_Os06g12100.1	mTERF family protein, expressed	2,00	-1.0	1	21	66	86	UUGAACAUCCCAGAGCCACCG	UCGUGGCGCUGGGAUGUUCAG	Cleavage
tae-miR9662b-3p	Traes_5DS_9F4A5AE9F.2	PF02536				GO:GO:0006355,GO:GO:0005739,GO:GO:0003690	AT5G07900.1	Mitochondrial transcription termination factor family protein	LOC_Os06g12100.1	mTERF family protein, expressed	2,05	-1.0	1	21	65	85	UGAACAUCCCAGAGCCACCGG	CUCGUGGCGCUGGGAUGUUCA	Cleavage
tae-miR1122b-3p	Traes_5DS_9F8FFD461.1										1,05	-1.0	1	21	1777	1797	AGACUUAUAUGUAGGAACGGA	UCCGUUCCUAAAUAUAAGUCU	Translation
tae-miR1122b-3p	Traes_5DS_9F8FFD461.2										1,05	-1.0	1	21	1882	1902	AGACUUAUAUGUAGGAACGGA	UCCGUUCCUAAAUAUAAGUCU	Translation
tae-miR1122b-3p	Traes_5DS_9F8FFD461.3										1,05	-1.0	1	21	1801	1821	AGACUUAUAUGUAGGAACGGA	UCCGUUCCUAAAUAUAAGUCU	Translation
tae-miR1122b-3p	Traes_5DS_A6AE32DCD.1										1,05	-1.0	1	21	1065	1085	AGACUUAUAUGUAGGAACGGA	UCCGUUCCUAAAUAUAAGUCU	Translation
tae-miR1122b-3p	Traes_5DS_A6AE32DCD.2										1,05	-1.0	1	21	1146	1166	AGACUUAUAUGUAGGAACGGA	UCCGUUCCUAAAUAUAAGUCU	Translation
tae-miR1122b-3p	Traes_5DS_A6AE32DCD.3										1,05	-1.0	1	21	1146	1166	AGACUUAUAUGUAGGAACGGA	UCCGUUCCUAAAUAUAAGUCU	Translation
tae-miR9662a-3p	Traes_5DS_C87840EBA.2						AT1G79220.1	Mitochondrial transcription termination factor family protein	LOC_Os06g12100.1	mTERF family protein, expressed	2,00	-1.0	1	21	429	449	UUGAACAUCCCAGAGCCACCG	CCGUGGUUCUGGGAUGUUCAG	Cleavage
tae-miR9662b-3p	Traes_5DS_C87840EBA.2						AT1G79220.1	Mitochondrial transcription termination factor family protein	LOC_Os06g12100.1	mTERF family protein, expressed	2,00	-1.0	1	21	428	448	UGAACAUCCCAGAGCCACCGG	CCCGUGGUUCUGGGAUGUUCA	Cleavage
tae-miR1128	Traes_5DS_D165422B3.1										3,00	-1.0	1	21	59	80	UACUACUCCCUCCG-UCCGAAA	UUUAGGAACGGAGGGAGUAGUA	Cleavage
tae-miR1118	Traes_5DS_F8D323D4D.2										1,05	-1.0	1	23	1329	1351	CACUACAUUAUGGAAUGGAGGGA	CUCCUCCAUUCCACAAUGUAGUG	Translation
tae-miR160	Traes_6AL_232AF0B76.1										0,00	-1.0	1	21	195	215	UGCCUGGCUCCCUGUAUGCCA	AGGCAUACAGGGAGCCAGGCA	Cleavage
tae-miR1121	Traes_6AL_23B31796F.2										2,00	-1.0	1	22	846	867	AGUAGUGAUCUAAACGCUCUUA	UAAAAGUGUUUAGAUCACUACU	Cleavage
tae-miR1121	Traes_6AL_23B31796F.3										2,00	-1.0	1	22	760	781	AGUAGUGAUCUAAACGCUCUUA	UAAAAGUGUUUAGAUCACUACU	Cleavage
tae-miR1121	Traes_6AL_23B31796F.5										2,00	-1.0	1	22	742	763	AGUAGUGAUCUAAACGCUCUUA	UAAAAGUGUUUAGAUCACUACU	Cleavage
tae-miR5049-3p	Traes_6AL_36AE1B4CF.4										1,05	-1.0	1	21	1787	1807	AAUAUGGAUCGGAGGGAGUAC	AUACUCCCUCCGACCCAUAUU	Cleavage
tae-miR5049-3p	Traes_6AL_36AE1B4CF1.4										1,05	-1.0	1	21	1787	1807	AAUAUGGAUCGGAGGGAGUAC	AUACUCCCUCCGACCCAUAUU	Cleavage
tae-miR5049-3p	Traes_6AL_6AD463C67.3										2,05	-1.0	1	21	15	35	AAUAUGGAUCGGAGGGAGUAC	GUACUCCCUUCCGAUCCAUAU	Cleavage
tae-miR1121	Traes_6AL_7E70268F1.3										0,00	-1.0	1	22	497	518	AGUAGUGAUCUAAACGCUCUUA	UAAGAGCGUUUAGAUCACUACU	Cleavage
tae-miR1122b-3p	Traes_6AL_94CA6F0D8.5										2,05	-1.0	1	21	1030	1050	AGACUUAUAUGUAGGAACGGA	UCCAUUCCUAAAUAUAAGUCU	Translation
tae-miR1122a	Traes_6AL_94CA6F0D8.5										2,05	-1.0	1	20	1071	1090	UAGAUACAUCCGUAUCUAGA	ACUACAUACGGAUGUAUAUA	Cleavage
tae-miR9662a-3p	Traes_6AS_0A204AEC9.1	PF02536	KOG1267		K15032	GO:GO:0006355,GO:GO:0005739,GO:GO:0003690	AT5G07900.1	Mitochondrial transcription termination factor family protein	LOC_Os06g12100.1	mTERF family protein, expressed	2,00	-1.0	1	21	681	701	UUGAACAUCCCAGAGCCACCG	CCGUGCCUCUGGGAUGUUCAG	Cleavage
tae-miR9662b-3p	Traes_6AS_0A204AEC9.1	PF02536	KOG1267		K15032	GO:GO:0006355,GO:GO:0005739,GO:GO:0003690	AT5G07900.1	Mitochondrial transcription termination factor family protein	LOC_Os06g12100.1	mTERF family protein, expressed	2,00	-1.0	1	21	680	700	UGAACAUCCCAGAGCCACCGG	CCCGUGCCUCUGGGAUGUUCA	Cleavage
tae-miR9662a-3p	Traes_6AS_0A204AEC9.2	PF02536	KOG1267		K15032	GO:GO:0006355,GO:GO:0005739,GO:GO:0003690	AT5G07900.1	Mitochondrial transcription termination factor family protein	LOC_Os06g12100.1	mTERF family protein, expressed	2,00	-1.0	1	21	681	701	UUGAACAUCCCAGAGCCACCG	CCGUGCCUCUGGGAUGUUCAG	Cleavage
tae-miR9662b-3p	Traes_6AS_0A204AEC9.2	PF02536	KOG1267		K15032	GO:GO:0006355,GO:GO:0005739,GO:GO:0003690	AT5G07900.1	Mitochondrial transcription termination factor family protein	LOC_Os06g12100.1	mTERF family protein, expressed	2,00	-1.0	1	21	680	700	UGAACAUCCCAGAGCCACCGG	CCCGUGCCUCUGGGAUGUUCA	Cleavage
tae-miR9662a-3p	Traes_6AS_1496A7825.1	PF02536				GO:GO:0006355,GO:GO:0005739,GO:GO:0003690	AT5G07900.1	Mitochondrial transcription termination factor family protein	LOC_Os06g12100.1	mTERF family protein, expressed	1,05	-1.0	1	21	549	569	UUGAACAUCCCAGAGCCACCG	CCGUGGCUCGGGAUGUUCAA	Cleavage
tae-miR9662b-3p	Traes_6AS_1496A7825.1	PF02536				GO:GO:0006355,GO:GO:0005739,GO:GO:0003690	AT5G07900.1	Mitochondrial transcription termination factor family protein	LOC_Os06g12100.1	mTERF family protein, expressed	2,05	-1.0	1	21	548	568	UGAACAUCCCAGAGCCACCGG	CCCGUGGCUCGGGGAUGUUCA	Translation
tae-miR9662a-3p	Traes_6AS_1496A7825.2	PF02536				GO:GO:0006355,GO:GO:0005739,GO:GO:0003690	AT5G07900.1	Mitochondrial transcription termination factor family protein	LOC_Os06g12100.1	mTERF family protein, expressed	1,05	-1.0	1	21	132	152	UUGAACAUCCCAGAGCCACCG	CCGUGGCUCGGGAUGUUCAA	Cleavage
tae-miR9662b-3p	Traes_6AS_1496A7825.2	PF02536				GO:GO:0006355,GO:GO:0005739,GO:GO:0003690	AT5G07900.1	Mitochondrial transcription termination factor family protein	LOC_Os06g12100.1	mTERF family protein, expressed	2,05	-1.0	1	21	131	151	UGAACAUCCCAGAGCCACCGG	CCCGUGGCUCGGGGAUGUUCA	Translation
tae-miR9662a-3p	Traes_6AS_15C1E6F18.1	PF02536				GO:GO:0006355,GO:GO:0005739,GO:GO:0003690	AT1G21150.1	Mitochondrial transcription termination factor family protein	LOC_Os06g12100.1	mTERF family protein, expressed	2,05	-1.0	1	21	33	53	UUGAACAUCCCAGAGCCACCG	CUGUGGCUCUGGAAUGUUCAG	Cleavage
tae-miR9662b-3p	Traes_6AS_15C1E6F18.1	PF02536				GO:GO:0006355,GO:GO:0005739,GO:GO:0003690	AT1G21150.1	Mitochondrial transcription termination factor family protein	LOC_Os06g12100.1	mTERF family protein, expressed	2,05	-1.0	1	21	32	52	UGAACAUCCCAGAGCCACCGG	CCUGUGGCUCUGGAAUGUUCA	Cleavage
tae-miR9662a-3p	Traes_6AS_15C1E6F18.2	PF02536				GO:GO:0006355,GO:GO:0005739,GO:GO:0003690	AT1G21150.1	Mitochondrial transcription termination factor family protein	LOC_Os06g12100.1	mTERF family protein, expressed	2,05	-1.0	1	21	33	53	UUGAACAUCCCAGAGCCACCG	CUGUGGCUCUGGAAUGUUCAG	Cleavage
tae-miR9662b-3p	Traes_6AS_15C1E6F18.2	PF02536				GO:GO:0006355,GO:GO:0005739,GO:GO:0003690	AT1G21150.1	Mitochondrial transcription termination factor family protein	LOC_Os06g12100.1	mTERF family protein, expressed	2,05	-1.0	1	21	32	52	UGAACAUCCCAGAGCCACCGG	CCUGUGGCUCUGGAAUGUUCA	Cleavage
tae-miR9662a-3p	Traes_6AS_2BDC1663A.1	PF02536	KOG1267		K15032	GO:GO:0006355,GO:GO:0005739,GO:GO:0003690	AT5G07900.1	Mitochondrial transcription termination factor family protein	LOC_Os06g12100.1	mTERF family protein, expressed	1,00	-1.0	1	21	681	701	UUGAACAUCCCAGAGCCACCG	CCGUGGCUCUGGGAUGUUCAG	Cleavage
tae-miR9662b-3p	Traes_6AS_2BDC1663A.1	PF02536	KOG1267		K15032	GO:GO:0006355,GO:GO:0005739,GO:GO:0003690	AT5G07900.1	Mitochondrial transcription termination factor family protein	LOC_Os06g12100.1	mTERF family protein, expressed	1,00	-1.0	1	21	680	700	UGAACAUCCCAGAGCCACCGG	CCCGUGGCUCUGGGAUGUUCA	Cleavage
tae-miR156	Traes_6AS_32A35158A.1	PF03110				GO:GO:0005634,GO:GO:0003677	AT5G43270.2	squamosa promoter binding protein-like 2	LOC_Os06g45310.1	OsSPL11 - SBP-box gene family member, expressed	1,00	-1.0	1	21	736	756	UGACAGAAGAGAGUGAGCACA	CGUGCUCUCUCUCUUCUGUCA	Cleavage
tae-miR156	Traes_6AS_32A35158A.2								LOC_Os06g45310.1	OsSPL11 - SBP-box gene family member, expressed	1,00	-1.0	1	21	292	312	UGACAGAAGAGAGUGAGCACA	CGUGCUCUCUCUCUUCUGUCA	Cleavage
tae-miR156	Traes_6AS_32A35158A.3								LOC_Os06g45310.1	OsSPL11 - SBP-box gene family member, expressed	1,00	-1.0	1	21	64	84	UGACAGAAGAGAGUGAGCACA	CGUGCUCUCUCUCUUCUGUCA	Cleavage
tae-miR9662a-3p	Traes_6AS_3641B9C871.1	PF02536	KOG1267		K15032	GO:GO:0006355,GO:GO:0005739,GO:GO:0003690	AT5G07900.1	Mitochondrial transcription termination factor family protein	LOC_Os06g12100.1	mTERF family protein, expressed	1,00	-1.0	1	21	687	707	UUGAACAUCCCAGAGCCACCG	CCGUGGCUCUGGGAUGUUCAG	Cleavage
tae-miR9662b-3p	Traes_6AS_3641B9C871.1	PF02536	KOG1267		K15032	GO:GO:0006355,GO:GO:0005739,GO:GO:0003690	AT5G07900.1	Mitochondrial transcription termination factor family protein	LOC_Os06g12100.1	mTERF family protein, expressed	1,00	-1.0	1	21	686	706	UGAACAUCCCAGAGCCACCGG	CCCGUGGCUCUGGGAUGUUCA	Cleavage
tae-miR9662a-3p	Traes_6AS_3641B9C871.2	PF02536	KOG1267		K15032	GO:GO:0006355,GO:GO:0005739,GO:GO:0003690	AT5G07900.1	Mitochondrial transcription termination factor family protein	LOC_Os06g12100.1	mTERF family protein, expressed	1,00	-1.0	1	21	687	707	UUGAACAUCCCAGAGCCACCG	CCGUGGCUCUGGGAUGUUCAG	Cleavage
tae-miR9662b-3p	Traes_6AS_3641B9C871.2	PF02536	KOG1267		K15032	GO:GO:0006355,GO:GO:0005739,GO:GO:0003690	AT5G07900.1	Mitochondrial transcription termination factor family protein	LOC_Os06g12100.1	mTERF family protein, expressed	1,00	-1.0	1	21	686	706	UGAACAUCCCAGAGCCACCGG	CCCGUGGCUCUGGGAUGUUCA	Cleavage
tae-miR9662a-3p	Traes_6AS_36EAEADDD.1	PF02536				GO:GO:0006355,GO:GO:0005739,GO:GO:0003690	AT1G21150.1	Mitochondrial transcription termination factor family protein	LOC_Os06g12100.1	mTERF family protein, expressed	2,00	-1.0	1	21	33	53	UUGAACAUCCCAGAGCCACCG	CUGUGGUUCUGGGAUGUUCAG	Cleavage
tae-miR9662b-3p	Traes_6AS_36EAEADDD.1	PF02536				GO:GO:0006355,GO:GO:0005739,GO:GO:0003690	AT1G21150.1	Mitochondrial transcription termination factor family protein	LOC_Os06g12100.1	mTERF family protein, expressed	2,00	-1.0	1	21	32	52	UGAACAUCCCAGAGCCACCGG	CCUGUGGUUCUGGGAUGUUCA	Cleavage
tae-miR9662a-3p	Traes_6AS_36EAEADDD.2								LOC_Os06g12100.1	mTERF family protein, expressed	2,00	-1.0	1	21	33	53	UUGAACAUCCCAGAGCCACCG	CUGUGGUUCUGGGAUGUUCAG	Cleavage
tae-miR9662b-3p	Traes_6AS_36EAEADDD.2								LOC_Os06g12100.1	mTERF family protein, expressed	2,00	-1.0	1	21	32	52	UGAACAUCCCAGAGCCACCGG	CCUGUGGUUCUGGGAUGUUCA	Cleavage
tae-miR9662a-3p	Traes_6AS_36EAEADDD.3	PF02536				GO:GO:0006355,GO:GO:0005739,GO:GO:0003690	AT1G21150.1	Mitochondrial transcription termination factor family protein	LOC_Os06g12100.1	mTERF family protein, expressed	2,00	-1.0	1	21	33	53	UUGAACAUCCCAGAGCCACCG	CUGUGGUUCUGGGAUGUUCAG	Cleavage
tae-miR9662b-3p	Traes_6AS_36EAEADDD.3	PF02536				GO:GO:0006355,GO:GO:0005739,GO:GO:0003690	AT1G21150.1	Mitochondrial transcription termination factor family protein	LOC_Os06g12100.1	mTERF family protein, expressed	2,00	-1.0	1	21	32	52	UGAACAUCCCAGAGCCACCGG	CCUGUGGUUCUGGGAUGUUCA	Cleavage
tae-miR9662a-3p	Traes_6AS_48B701BB41.1	PF02536				GO:GO:0006355,GO:GO:0005739,GO:GO:0003690	AT1G21150.1	Mitochondrial transcription termination factor family protein	LOC_Os06g12100.1	mTERF family protein, expressed	1,00	-1.0	1	21	33	53	UUGAACAUCCCAGAGCCACCG	ACGUGGCUCUGGGAUGUUCAG	Cleavage

tae-miR9662b-3p	Traes_6AS_48B701BB41.1	PF02536				GO:GO:0006355,GO:GO:0005739,GO:GO:0003690	AT1G21150.1	Mitochondrial transcription termination factor family protein	LOC_Os06g12100.1	mTERF family protein, expressed	1,00	-1.0	1	21	32	52	UGAACAUCCCAGAGCCACCGG	CACGUGGCUCUGGGAUGUUCA	Cleavage
tae-miR9662a-3p	Traes_6AS_48B701BB41.2	PF02536				GO:GO:0006355,GO:GO:0005739,GO:GO:0003690	AT1G21150.1	Mitochondrial transcription termination factor family protein	LOC_Os06g12100.1	mTERF family protein, expressed	1,00	-1.0	1	21	33	53	UUGAACAUCCCAGAGCCACCG	ACGUGGCUCUGGGAUGUUCAG	Cleavage
tae-miR9662b-3p	Traes_6AS_48B701BB41.2	PF02536				GO:GO:0006355,GO:GO:0005739,GO:GO:0003690	AT1G21150.1	Mitochondrial transcription termination factor family protein	LOC_Os06g12100.1	mTERF family protein, expressed	1,00	-1.0	1	21	32	52	UGAACAUCCCAGAGCCACCGG	CACGUGGCUCUGGGAUGUUCA	Cleavage
tae-miR9662a-3p	Traes_6AS_48B701BB41.3	PF02536				GO:GO:0006355,GO:GO:0005739,GO:GO:0003690	AT1G21150.1	Mitochondrial transcription termination factor family protein	LOC_Os06g12100.1	mTERF family protein, expressed	1,00	-1.0	1	21	33	53	UUGAACAUCCCAGAGCCACCG	ACGUGGCUCUGGGAUGUUCAG	Cleavage
tae-miR9662b-3p	Traes_6AS_48B701BB41.3	PF02536				GO:GO:0006355,GO:GO:0005739,GO:GO:0003690	AT1G21150.1	Mitochondrial transcription termination factor family protein	LOC_Os06g12100.1	mTERF family protein, expressed	1,00	-1.0	1	21	32	52	UGAACAUCCCAGAGCCACCGG	CACGUGGCUCUGGGAUGUUCA	Cleavage
tae-miR9662a-3p	Traes_6AS_48B701BB41.4	PF02536				GO:GO:0006355,GO:GO:0005739,GO:GO:0003690	AT1G21150.1	Mitochondrial transcription termination factor family protein	LOC_Os06g12100.1	mTERF family protein, expressed	1,00	-1.0	1	21	33	53	UUGAACAUCCCAGAGCCACCG	ACGUGGCUCUGGGAUGUUCAG	Cleavage
tae-miR9662b-3p	Traes_6AS_48B701BB41.4	PF02536				GO:GO:0006355,GO:GO:0005739,GO:GO:0003690	AT1G21150.1	Mitochondrial transcription termination factor family protein	LOC_Os06g12100.1	mTERF family protein, expressed	1,00	-1.0	1	21	32	52	UGAACAUCCCAGAGCCACCGG	CACGUGGCUCUGGGAUGUUCA	Cleavage
tae-miR9662a-3p	Traes_6AS_48B701BB41.5	PF02536				GO:GO:0006355,GO:GO:0005739,GO:GO:0003690	AT1G21150.1	Mitochondrial transcription termination factor family protein	LOC_Os06g12100.1	mTERF family protein, expressed	1,00	-1.0	1	21	33	53	UUGAACAUCCCAGAGCCACCG	ACGUGGCUCUGGGAUGUUCAG	Cleavage
tae-miR9662b-3p	Traes_6AS_48B701BB41.5	PF02536				GO:GO:0006355,GO:GO:0005739,GO:GO:0003690	AT1G21150.1	Mitochondrial transcription termination factor family protein	LOC_Os06g12100.1	mTERF family protein, expressed	1,00	-1.0	1	21	32	52	UGAACAUCCCAGAGCCACCGG	CACGUGGCUCUGGGAUGUUCA	Cleavage
tae-miR1138	Traes_6AS_5F8725CDA.1										3,00	-1.0	1	23	2223	2245	GCUUAGAUGUGACAUCCUUAAAA	AGUUAGUUAUGUCACAUCUAAGC	Cleavage
tae-miR1138	Traes_6AS_5F8725CDA.2										3,00	-1.0	1	23	1969	1991	GCUUAGAUGUGACAUCCUUAAAA	AGUUAGUUAUGUCACAUCUAAGC	Cleavage
tae-miR9662a-3p	Traes_6AS_6612DF8D7.1	PF02536				GO:GO:0006355,GO:GO:0005739,GO:GO:0003690	AT5G07900.1	Mitochondrial transcription termination factor family protein	LOC_Os06g12100.1	mTERF family protein, expressed	1,00	-1.0	1	21	81	101	UUGAACAUCCCAGAGCCACCG	CCGUGGCUCUGGGAUGUUCAG	Cleavage
tae-miR9662b-3p	Traes_6AS_6612DF8D7.1	PF02536				GO:GO:0006355,GO:GO:0005739,GO:GO:0003690	AT5G07900.1	Mitochondrial transcription termination factor family protein	LOC_Os06g12100.1	mTERF family protein, expressed	1,00	-1.0	1	21	80	100	UGAACAUCCCAGAGCCACCGG	CCCGUGGCUCUGGGAUGUUCA	Cleavage
tae-miR9662a-3p	Traes_6AS_6612DF8D7.2	PF02536				GO:GO:0006355,GO:GO:0005739,GO:GO:0003690	AT5G07900.1	Mitochondrial transcription termination factor family protein	LOC_Os06g12100.1	mTERF family protein, expressed	1,00	-1.0	1	21	81	101	UUGAACAUCCCAGAGCCACCG	CCGUGGCUCUGGGAUGUUCAG	Cleavage
tae-miR9662b-3p	Traes_6AS_6612DF8D7.2	PF02536				GO:GO:0006355,GO:GO:0005739,GO:GO:0003690	AT5G07900.1	Mitochondrial transcription termination factor family protein	LOC_Os06g12100.1	mTERF family protein, expressed	1,00	-1.0	1	21	80	100	UGAACAUCCCAGAGCCACCGG	CCCGUGGCUCUGGGAUGUUCA	Cleavage
tae-miR9662a-3p	Traes_6AS_6612DF8D7.3	PF02536				GO:GO:0006355,GO:GO:0005739,GO:GO:0003690	AT5G07900.1	Mitochondrial transcription termination factor family protein	LOC_Os06g12100.1	mTERF family protein, expressed	1,00	-1.0	1	21	81	101	UUGAACAUCCCAGAGCCACCG	CCGUGGCUCUGGGAUGUUCAG	Cleavage
tae-miR9662b-3p	Traes_6AS_6612DF8D7.3	PF02536				GO:GO:0006355,GO:GO:0005739,GO:GO:0003690	AT5G07900.1	Mitochondrial transcription termination factor family protein	LOC_Os06g12100.1	mTERF family protein, expressed	1,00	-1.0	1	21	80	100	UGAACAUCCCAGAGCCACCGG	CCCGUGGCUCUGGGAUGUUCA	Cleavage
tae-miR9662a-3p	Traes_6AS_8D2111A83.1	PF02536				GO:GO:0006355,GO:GO:0005739,GO:GO:0003690	AT1G21150.1	Mitochondrial transcription termination factor family protein	LOC_Os06g12100.1	mTERF family protein, expressed	2,05	-1.0	1	21	33	53	UUGAACAUCCCAGAGCCACCG	UCGUGGCUCGGGGAUGUUCAG	Cleavage
tae-miR9662b-3p	Traes_6AS_8D2111A83.1	PF02536				GO:GO:0006355,GO:GO:0005739,GO:GO:0003690	AT1G21150.1	Mitochondrial transcription termination factor family protein	LOC_Os06g12100.1	mTERF family protein, expressed	2,05	-1.0	1	21	32	52	UGAACAUCCCAGAGCCACCGG	CUCGUGGCUCGGGGAUGUUCA	Translation
tae-miR9662a-3p	Traes_6AS_8D2111A83.2	PF02536				GO:GO:0006355,GO:GO:0005739,GO:GO:0003690	AT1G21150.1	Mitochondrial transcription termination factor family protein	LOC_Os06g12100.1	mTERF family protein, expressed	2,05	-1.0	1	21	246	266	UUGAACAUCCCAGAGCCACCG	UCGUGGCUCGGGGAUGUUCAG	Cleavage
tae-miR9662b-3p	Traes_6AS_8D2111A83.2	PF02536				GO:GO:0006355,GO:GO:0005739,GO:GO:0003690	AT1G21150.1	Mitochondrial transcription termination factor family protein	LOC_Os06g12100.1	mTERF family protein, expressed	2,05	-1.0	1	21	245	265	UGAACAUCCCAGAGCCACCGG	CUCGUGGCUCGGGGAUGUUCA	Translation
tae-miR9662a-3p	Traes_6AS_8D2111A83.3	PF02536				GO:GO:0006355,GO:GO:0005739,GO:GO:0003690	AT1G21150.1	Mitochondrial transcription termination factor family protein	LOC_Os06g12100.1	mTERF family protein, expressed	2,05	-1.0	1	21	246	266	UUGAACAUCCCAGAGCCACCG	UCGUGGCUCGGGGAUGUUCAG	Cleavage
tae-miR9662b-3p	Traes_6AS_8D2111A83.3	PF02536				GO:GO:0006355,GO:GO:0005739,GO:GO:0003690	AT1G21150.1	Mitochondrial transcription termination factor family protein	LOC_Os06g12100.1	mTERF family protein, expressed	2,05	-1.0	1	21	245	265	UGAACAUCCCAGAGCCACCGG	CUCGUGGCUCGGGGAUGUUCA	Translation
tae-miR9662a-3p	Traes_6AS_8D2111A83.4	PF02536				GO:GO:0006355,GO:GO:0005739,GO:GO:0003690	AT1G21150.1	Mitochondrial transcription termination factor family protein	LOC_Os06g12100.1	mTERF family protein, expressed	2,05	-1.0	1	21	246	266	UUGAACAUCCCAGAGCCACCG	UCGUGGCUCGGGGAUGUUCAG	Cleavage
tae-miR9662b-3p	Traes_6AS_8D2111A83.4	PF02536				GO:GO:0006355,GO:GO:0005739,GO:GO:0003690	AT1G21150.1	Mitochondrial transcription termination factor family protein	LOC_Os06g12100.1	mTERF family protein, expressed	2,05	-1.0	1	21	245	265	UGAACAUCCCAGAGCCACCGG	CUCGUGGCUCGGGGAUGUUCA	Translation
tae-miR9662a-3p	Traes_6AS_8D2111A83.5	PF02536				GO:GO:0006355,GO:GO:0005739,GO:GO:0003690	AT1G21150.1	Mitochondrial transcription termination factor family protein	LOC_Os06g12100.1	mTERF family protein, expressed	2,05	-1.0	1	21	246	266	UUGAACAUCCCAGAGCCACCG	UCGUGGCUCGGGGAUGUUCAG	Cleavage
tae-miR9662b-3p	Traes_6AS_8D2111A83.5	PF02536				GO:GO:0006355,GO:GO:0005739,GO:GO:0003690	AT1G21150.1	Mitochondrial transcription termination factor family protein	LOC_Os06g12100.1	mTERF family protein, expressed	2,05	-1.0	1	21	245	265	UGAACAUCCCAGAGCCACCGG	CUCGUGGCUCGGGGAUGUUCA	Translation
tae-miR9662a-3p	Traes_6AS_8D2111A83.6						AT1G21150.1	Mitochondrial transcription termination factor family protein	LOC_Os06g12100.1	mTERF family protein, expressed	2,05	-1.0	1	21	246	266	UUGAACAUCCCAGAGCCACCG	UCGUGGCUCGGGGAUGUUCAG	Cleavage
tae-miR9662b-3p	Traes_6AS_8D2111A83.6						AT1G21150.1	Mitochondrial transcription termination factor family protein	LOC_Os06g12100.1	mTERF family protein, expressed	2,05	-1.0	1	21	245	265	UGAACAUCCCAGAGCCACCGG	CUCGUGGCUCGGGGAUGUUCA	Translation
tae-miR9662a-3p	Traes_6AS_8D2111A83.7	PF02536				GO:GO:0006355,GO:GO:0005739,GO:GO:0003690	AT1G21150.1	Mitochondrial transcription termination factor family protein	LOC_Os06g12100.1	mTERF family protein, expressed	2,05	-1.0	1	21	33	53	UUGAACAUCCCAGAGCCACCG	UCGUGGCUCGGGGAUGUUCAG	Cleavage
tae-miR9662b-3p	Traes_6AS_8D2111A83.7	PF02536				GO:GO:0006355,GO:GO:0005739,GO:GO:0003690	AT1G21150.1	Mitochondrial transcription termination factor family protein	LOC_Os06g12100.1	mTERF family protein, expressed	2,05	-1.0	1	21	32	52	UGAACAUCCCAGAGCCACCGG	CUCGUGGCUCGGGGAUGUUCA	Translation
tae-miR1122b-3p	Traes_6AS_8E6E52967.2										1,05	-1.0	1	21	120	140	AGACUUAUAUGUAGGAACGGA	UCCGUUCCUAAAUAUAAGUCU	Translation
tae-miR156	Traes_6AS_99C6A3B0B.1	PF03110				GO:GO:0005634,GO:GO:0003677	AT5G43270.1	squamosa promoter binding protein-like 2	LOC_Os02g04680.2	OsSPL3 - SBP-box gene family member, expressed	1,00	-1.0	1	21	1477	1497	UGACAGAAGAGAGUGAGCACA	CAUGCUCUCUCUCUUCUGUCA	Cleavage
tae-miR156	Traes_6AS_99C6A3B0B.2	PF03110				GO:GO:0005634,GO:GO:0003677	AT5G43270.3	squamosa promoter binding protein-like 2	LOC_Os02g04680.2	OsSPL3 - SBP-box gene family member, expressed	1,00	-1.0	1	21	1477	1497	UGACAGAAGAGAGUGAGCACA	CAUGCUCUCUCUCUUCUGUCA	Cleavage
tae-miR156	Traes_6AS_99C6A3B0B.3	PF03110				GO:GO:0005634,GO:GO:0003677	AT5G43270.2	squamosa promoter binding protein-like 2	LOC_Os02g04680.2	OsSPL3 - SBP-box gene family member, expressed	1,00	-1.0	1	21	1477	1497	UGACAGAAGAGAGUGAGCACA	CAUGCUCUCUCUCUUCUGUCA	Cleavage
tae-miR156	Traes_6AS_99C6A3B0B.4	PF03110				GO:GO:0005634,GO:GO:0003677	AT5G43270.2	squamosa promoter binding protein-like 2	LOC_Os02g04680.2	OsSPL3 - SBP-box gene family member, expressed	1,00	-1.0	1	21	1156	1176	UGACAGAAGAGAGUGAGCACA	CAUGCUCUCUCUCUUCUGUCA	Cleavage
tae-miR156	Traes_6AS_99C6A3B0B.5	PF03110				GO:GO:0005634,GO:GO:0003677	AT5G43270.3	squamosa promoter binding protein-like 2	LOC_Os02g04680.2	OsSPL3 - SBP-box gene family member, expressed	1,00	-1.0	1	21	1156	1176	UGACAGAAGAGAGUGAGCACA	CAUGCUCUCUCUCUUCUGUCA	Cleavage
tae-miR156	Traes_6AS_99C6A3B0B.6	PF03110				GO:GO:0005634,GO:GO:0003677	AT5G43270.1	squamosa promoter binding protein-like 2	LOC_Os02g04680.2	OsSPL3 - SBP-box gene family member, expressed	1,00	-1.0	1	21	1156	1176	UGACAGAAGAGAGUGAGCACA	CAUGCUCUCUCUCUUCUGUCA	Cleavage
tae-miR156	Traes_6AS_99C6A3B0B.7	PF03110				GO:GO:0005634,GO:GO:0003677	AT5G43270.3	squamosa promoter binding protein-like 2	LOC_Os02g04680.2	OsSPL3 - SBP-box gene family member, expressed	1,00	-1.0	1	21	1156	1176	UGACAGAAGAGAGUGAGCACA	CAUGCUCUCUCUCUUCUGUCA	Cleavage
tae-miR156	Traes_6AS_99C6A3B0B.8	PF03110				GO:GO:0005634,GO:GO:0003677	AT5G43270.1	squamosa promoter binding protein-like 2	LOC_Os02g04680.2	OsSPL3 - SBP-box gene family member, expressed	1,00	-1.0	1	21	1156	1176	UGACAGAAGAGAGUGAGCACA	CAUGCUCUCUCUCUUCUGUCA	Cleavage
tae-miR9662a-3p	Traes_6AS_A2EAE01C1.1	PF02536				GO:GO:0006355,GO:GO:0005739,GO:GO:0003690	AT5G07900.1	Mitochondrial transcription termination factor family protein	LOC_Os06g12100.1	mTERF family protein, expressed	2,05	-1.0	1	21	465	485	UUGAACAUCCCAGAGCCACCG	CCGUGGCUCUGGAAUGUUCAG	Cleavage
tae-miR9662b-3p	Traes_6AS_A2EAE01C1.1	PF02536				GO:GO:0006355,GO:GO:0005739,GO:GO:0003690	AT5G07900.1	Mitochondrial transcription termination factor family protein	LOC_Os06g12100.1	mTERF family protein, expressed	2,05	-1.0	1	21	464	484	UGAACAUCCCAGAGCCACCGG	CCCGUGGCUCUGGAAUGUUCA	Cleavage
tae-miR9662a-3p	Traes_6AS_A2EAE01C1.2	PF02536				GO:GO:0006355,GO:GO:0005739,GO:GO:0003690	AT5G07900.1	Mitochondrial transcription termination factor family protein	LOC_Os06g12100.1	mTERF family protein, expressed	2,05	-1.0	1	21	66	86	UUGAACAUCCCAGAGCCACCG	CCGUGGCUCUGGAAUGUUCAG	Cleavage
tae-miR9662b-3p	Traes_6AS_A2EAE01C1.2	PF02536				GO:GO:0006355,GO:GO:0005739,GO:GO:0003690	AT5G07900.1	Mitochondrial transcription termination factor family protein	LOC_Os06g12100.1	mTERF family protein, expressed	2,05	-1.0	1	21	65	85	UGAACAUCCCAGAGCCACCGG	CCCGUGGCUCUGGAAUGUUCA	Cleavage
tae-miR9662a-3p	Traes_6AS_B24138E62.1	PF02536				GO:GO:0006355,GO:GO:0005739,GO:GO:0003690	AT5G07900.1	Mitochondrial transcription termination factor family protein	LOC_Os06g12100.1	mTERF family protein, expressed	2,05	-1.0	1	21	375	395	UUGAACAUCCCAGAGCCACCG	CCGUGGCUCUGGAAUGUUCAG	Cleavage
tae-miR9662b-3p	Traes_6AS_B24138E62.1	PF02536				GO:GO:0006355,GO:GO:0005739,GO:GO:0003690	AT5G07900.1	Mitochondrial transcription termination factor family protein	LOC_Os06g12100.1	mTERF family protein, expressed	2,05	-1.0	1	21	374	394	UGAACAUCCCAGAGCCACCGG	CCCGUGGCUCUGGAAUGUUCA	Cleavage
tae-miR9662a-3p	Traes_6AS_EBADF37C7.1	PF02536				GO:GO:0006355,GO:GO:0005739,GO:GO:0003690	AT5G07900.1	Mitochondrial transcription termination factor family protein	LOC_Os06g12100.1	mTERF family protein, expressed	2,05	-1.0	1	21	681	701	UUGAACAUCCCAGAGCCACCG	CCGUGGCUCUGGGAUGCUCAG	Cleavage
tae-miR9662b-3p	Traes_6AS_EBADF37C7.1	PF02536				GO:GO:0006355,GO:GO:0005739,GO:GO:0003690	AT5G07900.1	Mitochondrial transcription termination factor family protein	LOC_Os06g12100.1	mTERF family protein, expressed	2,05	-1.0	1	21	680	700	UGAACAUCCCAGAGCCACCGG	CCCGUGGCUCUGGGAUGCUCA	Cleavage
tae-miR9662a-3p	Traes_6AS_EBADF37C7.2	PF02536				GO:GO:0006355,GO:GO:0005739,GO:GO:0003690	AT5G07900.1	Mitochondrial transcription termination factor family protein	LOC_Os06g12100.1	mTERF family protein, expressed	2,05	-1.0	1	21	378	398	UUGAACAUCCCAGAGCCACCG	CCGUGGCUCUGGGAUGCUCAG	Cleavage
tae-miR9662b-3p	Traes_6AS_EBADF37C7.2	PF02536				GO:GO:0006355,GO:GO:0005739,GO:GO:0003690	AT5G07900.1	Mitochondrial transcription termination factor family protein	LOC_Os06g12100.1	mTERF family protein, expressed	2,05	-1.0	1	21	377	397	UGAACAUCCCAGAGCCACCGG	CCCGUGGCUCUGGGAUGCUCA	Cleavage
tae-miR9662a-3p	Traes_6AS_EE73C76541.2	PF02536				GO:GO:0006355,GO:GO:0005739,GO:GO:0003690	AT5G07900.1	Mitochondrial transcription termination factor family protein	LOC_Os06g12100.1	mTERF family protein, expressed	1,00	-1.0	1	21	420	440	UUGAACAUCCCAGAGCCACCG	CCGUGGCUCUGGGAUGUUCAG	Cleavage
tae-miR9662b-3p	Traes_6AS_EE73C76541.2	PF02536				GO:GO:0006355,GO:GO:0005739,GO:GO:0003690	AT5G07900.1	Mitochondrial transcription termination factor family protein	LOC_Os06g12100.1	mTERF family protein, expressed	1,00	-1.0	1	21	419	439	UGAACAUCCCAGAGCCACCGG	CCCGUGGCUCUGGGAUGUUCA	Cleavage
tae-miR9662a-3p	Traes_6AS_EE73C76541.6	PF02536				GO:GO:0006355,GO:GO:0005739,GO:GO:0003690	AT5G07900.1	Mitochondrial transcription termination factor family protein	LOC_Os06g12100.1	mTERF family protein, expressed	1,00	-1.0	1	21	66	86	UUGAACAUCCCAGAGCCACCG	CCGUGGCUCUGGGAUGUUCAG	Cleavage
tae-miR9662b-3p	Traes_6AS_EE73C76541.6	PF02536				GO:GO:0006355,GO:GO:0005739,GO:GO:0003690	AT5G07900.1	Mitochondrial transcription termination factor family protein	LOC_Os06g12100.1	mTERF family protein, expressed	1,00	-1.0	1	21	65	85	UGAACAUCCCAGAGCCACCGG	CCCGUGGCUCUGGGAUGUUCA	Cleavage
tae-miR9662a-3p	Traes_6AS_EE8BA0A57.1	PF02536				GO:GO:0006355,GO:GO:0005739,GO:GO:0003690	AT1G21150.1	Mitochondrial transcription termination factor family protein	LOC_Os06g12100.1	mTERF family protein, expressed	2,00	-1.0	1	21	462	482	UUGAACAUCCCAGAGCCACCG	CCGUGCCUCUGGGAUGUUCAG	Cleavage
tae-miR9662b-3p	Traes_6AS_EE8BA0A57.1	PF02536				GO:GO:0006355,GO:GO:0005739,GO:GO:0003690	AT1G21150.1	Mitochondrial transcription termination factor family protein	LOC_Os06g12100.1	mTERF family protein, expressed	2,00	-1.0	1	21	461	481	UGAACAUCCCAGAGCCACCGG	CCCGUGCCUCUGGGAUGUUCA	Cleavage
tae-miR9662a-3p	Traes_6AS_EE8BA0A57.2	PF02536				GO:GO:0006355,GO:GO:0005739,GO:GO:0003690	AT5G07900.1	Mitochondrial transcription termination factor family protein	LOC_Os06g12100.1	mTERF family protein, expressed	2,00	-1.0	1	21	510	530	UUGAACAUCCCAGAGCCACCG	CCGUGCCUCUGGGAUGUUCAG	Cleavage
tae-miR9662b-3p	Traes_6AS_EE8BA0A57.2	PF02536				GO:GO:0006355,GO:GO:0005739,GO:GO:0003690	AT5G07900.1	Mitochondrial transcription termination factor family protein	LOC_Os06g12100.1	mTERF family protein, expressed	2,00	-1.0	1	21	509	529	UGAACAUCCCAGAGCCACCGG	CCCGUGCCUCUGGGAUGUUCA	Cleavage
tae-miR5049-3p	Traes_6AS_F5DBB6DBD.3										1,05	-1.0	1	21	1927	1947	AAUAUGGAUCGGAGGGAGUAC	UUACUCCCUCCAAUCCAUAUU	Translation
tae-miR9662a-3p	Traes_6AS_FBBCFBCB0.1	PF02536		KOG1267		GO:GO:0006355,GO:GO:0005739,GO:GO:0003690	AT5G07900.1	Mitochondrial transcription termination factor family protein	LOC_Os06g12100.1	mTERF family protein, expressed	2,05	-1.0	1	21	681	701	UUGAACAUCCCAGAGCCACCG	CCGUGGCUCUGGGAUGUUUAG	Cleavage
tae-miR9662b-3p	Traes_6AS_FBBCFBCB0.1	PF02536		KOG1267		GO:GO:0006355,GO:GO:0005739,GO:GO:0003690	AT5G07900.1	Mitochondrial transcription termination factor family protein	LOC_Os06g12100.1	mTERF family protein, expressed	2,05	-1.0	1	21	680	700	UGAACAUCCCAGAGCCACCGG	CCCGUGGCUCUGGGAUGUUUA	Cleavage
tae-miR1127b-3p	Traes_6BL_028068EFA.2										1,05	-1.0	1	21	2801	2821	ACAAGUAUUUCUGGACGGAGG	CCUCCGUCCGGAAAUACUUGU	Cleavage
tae-miR6197-5p	Traes_6BL_67725F06B.2										3,00	-1.0	1	21	278	298	UCUGUAAACAAAUGUAGGACG	GAACCUCCAUUUGUUUACAGG	Cleavage
tae-miR1127b-3p	Traes_6BL_731ED1C4C.1										1,05	-1.0	1	21	1020	1040	ACAAGUAUUUCUGGACGGAGG	UCUCCGUCCAAAAAUACUUGU	Translation
tae-miR1130b-3p	Traes_6BL_7BD68BAC2.3										1,05	-1.0	1	21	215	235	UCUUAUAUUAUGGGACGGAGG	CCUCCGUCCCAUAAUGUAAGA	Cleavage
tae-miR1122a	Traes_6BL_7E9C131F4.3										0,00	-1.0	1	20	761	780	UAGAUACAUCCGUAUCUAGA	UCUAGAUACGGAUGUAUCUA	Cleavage
tae-miR1130b-3p	Traes_6BL_887005D55.2										1,05	-1.0	1	21	79	99	UCUUAUAUUAUGGGACGGAGG	CCUCCGUCCCAUAAUAUAAUA	Cleavage
tae-miR1118	Traes_6BL_E14604962.2										2,00	-1.0	1	23	35	57	CACUACAUUAUGGAAUGGAGGGA	UCCCUCUGUUCCAUAAUGUAGUG	Cleavage
tae-miR1118	Traes_6BL_E14604962.3										2,00	-1.0	1	23	35	57	CACUACAUUAUGGAAUGGAGGGA	UCCCUCUGUUCCAUAAUGUAGUG	Cleavage
tae-miR1127b-3p	Traes_6BS_1AFDF35E5.3										2,05	-1.0	1	21	171	191	ACAAGUAUUUCUGGACGGAGG	CUUUCGUCCGGAAAUACUUGU	Cleavage
tae-miR9662a-3p	Traes_6BS_28F0D2955.1	PF02536				GO:GO:0006355,GO:GO:0005739,GO:GO:0003690	AT5G64950.1	Mitochondrial transcription termination factor family protein	LOC_Os06g12100.1	mTERF family protein, expressed	2,05	-1.0	1	21	396	416	UUGAACAUCCCAGAGCCACCG	CUGUGGCUCUCGGAUGUUCAG	Translation
tae-miR9662b-3p	Traes_6BS_28F0D2955.1	PF02536				GO:GO:0006355,GO:GO:0005739,GO:GO:0003690	AT5G64950.1	Mitochondrial transcription termination factor family protein	LOC_Os06g12100.1	mTERF family protein, expressed	2,05	-1.0	1	21	395	415	UGAACAUCCCAGAGCCACCGG	CCUGUGGCUCUCGGAUGUUCA	Translation
tae-miR9662a-3p	Traes_6BS_2AC6A2287.1	PF02536				GO:GO:0006355,GO:GO:0005739,GO:GO:0003690	AT5G07900.1	Mitochondrial transcription termination factor family protein	LOC_Os06g12100.1	mTERF family protein, expressed	2,05	-1.0	1	21	66	86	UUGAACAUCCCAGAGCCACCG	CCGUGGCUCUGGGAUGCUCAG	Cleavage
tae-miR9662b-3p	Traes_6BS_2AC6A2287.1	PF02536				GO:GO:0006355,GO:GO:0005739,GO:GO:0003690	AT5G07900.1	Mitochondrial transcription termination factor family protein	LOC_Os06g12100.1	mTERF family protein, expressed	2,05	-1.0	1	21	65	85	UGAACAUCCCAGAGCCACCGG	CCCGUGGCUCUGGGAUGCUCA	Cleavage
tae-miR9662a-3p	Traes_6BS_2F108C5BB.1	PF02536				GO:GO:0006355,GO:GO:0005739,GO:GO:0003690	AT5G07900.1	Mitochondrial transcription termination factor family protein	LOC_Os06g12100.1	mTERF family protein, expressed	2,00	-1.0	1	21	435	455	UUGAACAUCCCAGAGCCACCG	UCGUGGCGCUGGGAUGUUCAG	Cleavage
tae-miR9662b-3p	Traes_6BS_2F108C5BB.1	PF02536				GO:GO:0006355,GO:GO:0005739,GO:GO:0003690	AT5G07900.1	Mitochondrial transcription termination factor family protein	LOC_Os06g12100.1	mTERF family protein, expressed	2,05	-1.0	1	21	434	454	UGAACAUCCCAGAGCCACCGG	CUCGUGGCGCUGGGAUGUUCA	Cleavage
tae-miR408	Traes_6BS_348308070.13										2,05	-1.0	1	21	2	22	CUGCACUGCCUCUUCCCUGGC	CCCAGGGCCGAGGCAGUGCAG	Cleavage
tae-miR408	Traes_6BS_348308070.15										2,05	-1.0	1	21	11	31	CUGCACUGCCUCUUCCCUGGC	CCCAGGGCCGAGGCAGUGCAG	Cleavage
tae-miR408	Traes_6BS_348308070.17										2,05	-1.0	1	21	14	34	CUGCACUGCCUCUUCCCUGGC	CCCAGGGCCGAGGCAGUGCAG	Cleavage
tae-miR408	Traes_6BS_348308070.19										2,05	-1.0	1	21	11	31	CUGCACUGCCUCUUCCCUGGC	CCCAGGGCCGAGGCAGUGCAG	Cleavage
tae-miR408	Traes_6BS_348308070.2										2,05	-1.0	1	21	14	34	CUGCACUGCCUCUUCCCUGGC	CCCAGGGCCGAGGCAGUGCAG	Cleavage
tae-miR408	Traes_6BS_348308070.3										2,05	-1.0	1	21	17	37	CUGCACUGCCUCUUCCCUGGC	CCCAGGGCCGAGGCAGUGCAG	Cleavage
tae-miR408	Traes_6BS_348308070.5										2,05	-1.0	1	21	17	37	CUGCACUGCCUCUUCCCUGGC	CCCAGGGCCGAGGCAGUGCAG	Cleavage
tae-miR408	Traes_6BS_348308070.6										2,05	-1.0	1	21	14	34	CUGCACUGCCUCUUCCCUGGC	CCCAGGGCCGAGGCAGUGCAG	Cleavage
tae-miR408	Traes_6BS_348308070.8										2,05	-1.0	1	21	17	37	CUGCACUGCCUCUUCCCUGGC	CCCAGGGCCGAGGCAGUGCAG	Cleavage
tae-miR408	Traes_6BS_348308070.9										2,05	-1.0	1	21	17	37	CUGCACUGCCUCUUCCCUGGC	CCCAGGGCCGAGGCAGUGCAG	Cleavage
tae-miR9662a-3p	Traes_6BS_3A00751B5.1	PF02536				GO:GO:0006355,GO:GO:0005739,GO:GO:0003690	AT5G07900.1	Mitochondrial transcription termination factor family protein	LOC_Os06g12100.1	mTERF family protein, expressed	2,05	-1.0	1	21	69	89	UUGAACAUCCCAGAGCCACCG	CCGUGGCUCUGGGAUGUUUAG	Cleavage
tae-miR9662b-3p	Traes_6BS_3A00751B5.1	PF02536				GO:GO:0006355,GO:GO:0005739,GO:GO:0003690	AT5G07900.1	Mitochondrial transcription termination factor family protein	LOC_Os06g12100.1	mTERF family protein, expressed	2,05	-1.0	1	21	68	88	UGAACAUCCCAGAGCCACCGG	CCCGUGGCUCUGGGAUGUUUA	Cleavage
tae-miR9662a-3p	Traes_6BS_3A00751B5.2	PF02536				GO:GO:0006355,GO:GO:0005739,GO:GO:0003690	AT5G07900.1	Mitochondrial transcription termination factor family protein	LOC_Os06g12100.1	mTERF family protein, expressed	2,05	-1.0	1	21	69	89	UUGAACAUCCCAGAGCCACCG	CCGUGGCUCUGGGAUGUUUAG	Cleavage
tae-miR9662b-3p	Traes_6BS_3A00751B5.2	PF02536				GO:GO:0006355,GO:GO:0005739,GO:GO:0003690	AT5G07900.1	Mitochondrial transcription termination factor family protein	LOC_Os06g12100.1	mTERF family protein, expressed	2,05	-1.0	1	21	68	88	UGAACAUCCCAGAGCCACCGG	CCCGUGGCUCUGGGAUGUUUA	Cleavage
tae-miR9662a-3p	Traes_6BS_46641470F.1	PF02536				GO:GO:0006355,GO:GO:0005739,GO:GO:0003690	AT1G21150.1	Mitochondrial transcription termination factor family protein	LOC_Os06g12100.1	mTERF family protein, expressed	2,05	-1.0	1	21	33	53	UUGAACAUCCCAGAGCCACCG	CCGUGGCUCUGGGAUGUUUAG	Cleavage
tae-miR9662b-3p	Traes_6BS_46641470F.1	PF02536				GO:GO:0006355,GO:GO:0005739,GO:GO:0003690	AT1G21150.1	Mitochondrial transcription termination factor family protein	LOC_Os06g12100.1	mTERF family protein, expressed	2,05	-1.0	1	21	32	52	UGAACAUCCCAGAGCCACCGG	CCCGUGGCUCUGGGAUGUUUA	Cleavage
tae-miR9662a-3p	Traes_6BS_4E6E57FEB.1	PF02536				GO:GO:0006355,GO:GO:0005739,GO:GO:0003690	AT5G07900.1	Mitochondrial transcription termination factor family protein	LOC_Os06g12100.1	mTERF family protein, expressed	3,00	-1.0	1	21	65	86	UUGAACAUCCCAGAGCCAC-CG	CGCGUGGCUCUGGGAUGUUCAG	Cleavage
tae-miR9662b-3p	Traes_6BS_4E6E57FEB.1	PF02536				GO:GO:0006355,GO:GO:0005739,GO:GO:0003690	AT5G07900.1	Mitochondrial transcription termination factor family protein	LOC_Os06g12100.1	mTERF family protein, expressed	2,00	-1.0	1	21	64	85	UGAACAUCCCAGAGCCAC-CGG	CCGCGUGGCUCUGGGAUGUUCA	Cleavage
tae-miR9662a-3p	Traes_6BS_4E6E57FEB.2	PF02536				GO:GO:0006355,GO:GO:0005739,GO:GO:0003690	AT5G07900.1	Mitochondrial transcription termination factor family protein	LOC_Os06g12100.1	mTERF family protein, expressed	3,00	-1.0	1	21	65	86	UUGAACAUCCCAGAGCCAC-CG	CGCGUGGCUCUGGGAUGUUCAG	Cleavage
tae-miR9662b-3p	Traes_6BS_4E6E57FEB.2	PF02536				GO:GO:0006355,GO:GO:0005739,GO:GO:0003690	AT5G07900.1	Mitochondrial transcription termination factor family protein	LOC_Os06g12100.1	mTERF family protein, expressed	2,00	-1.0	1	21	64	85	UGAACAUCCCAGAGCCAC-CGG	CCGCGUGGCUCUGGGAUGUUCA	Cleavage
tae-miR9662a-3p	Traes_6BS_4E6E57FEB.3	PF02536				GO:GO:0006355,GO:GO:0005739,GO:GO:0003690	AT5G07900.1	Mitochondrial transcription termination factor family protein	LOC_Os06g12100.1	mTERF family protein, expressed	3,00	-1.0	1	21	65	86	UUGAACAUCCCAGAGCCAC-CG	CGCGUGGCUCUGGGAUGUUCAG	Cleavage
tae-miR9662b-3p	Traes_6BS_4E6E57FEB.3	PF02536				GO:GO:0006355,GO:GO:0005739,GO:GO:0003690	AT5G07900.1	Mitochondrial transcription termination factor family protein	LOC_Os06g12100.1	mTERF family protein, expressed	2,00	-1.0	1	21	64	85	UGAACAUCCCAGAGCCAC-CGG	CCGCGUGGCUCUGGGAUGUUCA	Cleavage
tae-miR9662b-3p	Traes_6BS_508C2BEA2.1	PF02536				GO:GO:0006355,GO:GO:0005739,GO:GO:0003690	AT1G21150.1	Mitochondrial transcription termination factor family protein	LOC_Os06g12100.1	mTERF family protein, expressed	3,00	-1.0	1	21	32	52	UGAACAUCCCAGAGCCACCGG	CCUGUGGUUCUGGGAUGUUCG	Cleavage
tae-miR156	Traes_6BS_542961EA4.1								LOC_Os06g45310.1	OsSPL11 - SBP-box gene family member, expressed	1,00	-1.0	1	21	283	303	UGACAGAAGAGAGUGAGCACA	CGUGCUCUCUCUCUUCUGUCA	Cleavage
tae-miR156	Traes_6BS_542961EA4.2								LOC_Os06g45310.1	OsSPL11 - SBP-box gene family member, expressed	1,00	-1.0	1	21	349	369	UGACAGAAGAGAGUGAGCACA	CGUGCUCUCUCUCUUCUGUCA	Cleavage
tae-miR1122b-3p	Traes_6BS_7965FEB75.1										2,05	-1.0	1	21	87	107	AGACUUAUAUGUAGGAACGGA	UCCGUUCGUAUAUAUAAGUCU	Translation
tae-miR9662a-3p	Traes_6BS_7F1846171.1	PF02536				GO:GO:0006355,GO:GO:0005739,GO:GO:0003690	AT5G07900.1	Mitochondrial transcription termination factor family protein	LOC_Os06g12100.1	mTERF family protein, expressed	2,05	-1.0	1	21	33	53	UUGAACAUCCCAGAGCCACCG	CUGUGGCUCUCGGAUGUUCAG	Translation
tae-miR9662b-3p	Traes_6BS_7F1846171.1	PF02536				GO:GO:0006355,GO:GO:0005739,GO:GO:0003690	AT5G07900.1	Mitochondrial transcription termination factor family protein	LOC_Os06g12100.1	mTERF family protein, expressed	2,05	-1.0	1	21	32	52	UGAACAUCCCAGAGCCACCGG	CCUGUGGCUCUCGGAUGUUCA	Translation
tae-miR9662a-3p	Traes_6BS_82F5BF11F.1	PF02536				GO:GO:0006355,GO:GO:0005739,GO:GO:0003690	AT1G79220.1	Mitochondrial transcription termination factor family protein	LOC_Os06g12100.1	mTERF family protein, expressed	2,00	-1.0	1	21	267	287	UUGAACAUCCCAGAGCCACCG	CCGUGCCUCUGGGAUGUUCAG	Cleavage
tae-miR9662b-3p	Traes_6BS_82F5BF11F.1	PF02536				GO:GO:0006355,GO:GO:0005739,GO:GO:0003690	AT1G79220.1	Mitochondrial transcription termination factor family protein	LOC_Os06g12100.1	mTERF family protein, expressed	2,00	-1.0	1	21	266	286	UGAACAUCCCAGAGCCACCGG	CCCGUGCCUCUGGGAUGUUCA	Cleavage
tae-miR9662a-3p	Traes_6BS_843505CB3.1	PF02536				GO:GO:0006355,GO:GO:0005739,GO:GO:0003690	AT1G21150.1	Mitochondrial transcription termination factor family protein	LOC_Os06g12100.1	mTERF family protein, expressed	2,05	-1.0	1	21	69	89	UUGAACAUCCCAGAGCCACCG	CCGUGGCUCUGGAAUGUUCAG	Cleavage
tae-miR9662b-3p	Traes_6BS_843505CB3.1	PF02536				GO:GO:0006355,GO:GO:0005739,GO:GO:0003690	AT1G21150.1	Mitochondrial transcription termination factor family protein	LOC_Os06g12100.1	mTERF family protein, expressed	2,05	-1.0	1	21	68	88	UGAACAUCCCAGAGCCACCGG	CCCGUGGCUCUGGAAUGUUCA	Cleavage
tae-miR9662a-3p	Traes_6BS_843505CB3.2	PF02536				GO:GO:0006355,GO:GO:0005739,GO:GO:0003690	AT1G21150.1	Mitochondrial transcription termination factor family protein	LOC_Os06g12100.1	mTERF family protein, expressed	2,05	-1.0	1	21	69	89	UUGAACAUCCCAGAGCCACCG	CCGUGGCUCUGGAAUGUUCAG	Cleavage
tae-miR9662b-3p	Traes_6BS_843505CB3.2	PF02536				GO:GO:0006355,GO:GO:0005739,GO:GO:0003690	AT1G21150.1	Mitochondrial transcription termination factor family protein	LOC_Os06g12100.1	mTERF family protein, expressed	2,05	-1.0	1	21	68	88	UGAACAUCCCAGAGCCACCGG	CCCGUGGCUCUGGAAUGUUCA	Cleavage
tae-miR9662a-3p	Traes_6BS_843505CB3.3	PF02536				GO:GO:0006355,GO:GO:0005739,GO:GO:0003690	AT1G21150.1	Mitochondrial transcription termination factor family protein	LOC_Os06g12100.1	mTERF family protein, expressed	2,05	-1.0	1	21	69	89	UUGAACAUCCCAGAGCCACCG	CCGUGGCUCUGGAAUGUUCAG	Cleavage
tae-miR9662b-3p	Traes_6BS_843505CB3.3	PF02536				GO:GO:0006355,GO:GO:0005739,GO:GO:0003690	AT1G21150.1	Mitochondrial transcription termination factor family protein	LOC_Os06g12100.1	mTERF family protein, expressed	2,05	-1.0	1	21	68	88	UGAACAUCCCAGAGCCACCGG	CCCGUGGCUCUGGAAUGUUCA	Cleavage
tae-miR9662a-3p	Traes_6BS_843505CB3.4	PF02536				GO:GO:0006355,GO:GO:0005739,GO:GO:0003690	AT1G21150.1	Mitochondrial transcription termination factor family protein	LOC_Os06g12100.1	mTERF family protein, expressed	2,05	-1.0	1	21	69	89	UUGAACAUCCCAGAGCCACCG	CCGUGGCUCUGGAAUGUUCAG	Cleavage
tae-miR9662b-3p	Traes_6BS_843505CB3.4	PF02536				GO:GO:0006355,GO:GO:0005739,GO:GO:0003690	AT1G21150.1	Mitochondrial transcription termination factor family protein	LOC_Os06g12100.1	mTERF family protein, expressed	2,05	-1.0	1	21	68	88	UGAACAUCCCAGAGCCACCGG	CCCGUGGCUCUGGAAUGUUCA	Cleavage
tae-miR9662a-3p	Traes_6BS_843505CB3.5	PF02536				GO:GO:0006355,GO:GO:0005739,GO:GO:0003690	AT1G21150.1	Mitochondrial transcription termination factor family protein	LOC_Os06g12100.1	mTERF family protein, expressed	2,05	-1.0	1	21	69	89	UUGAACAUCCCAGAGCCACCG	CCGUGGCUCUGGAAUGUUCAG	Cleavage
tae-miR9662b-3p	Traes_6BS_843505CB3.5	PF02536				GO:GO:0006355,GO:GO:0005739,GO:GO:0003690	AT1G21150.1	Mitochondrial transcription termination factor family protein	LOC_Os06g12100.1	mTERF family protein, expressed	2,05	-1.0	1	21	68	88	UGAACAUCCCAGAGCCACCGG	CCCGUGGCUCUGGAAUGUUCA	Cleavage
tae-miR9662a-3p	Traes_6BS_843505CB3.6	PF02536				GO:GO:0006355,GO:GO:0005739,GO:GO:0003690	AT1G21150.1	Mitochondrial transcription termination factor family protein	LOC_Os06g12100.1	mTERF family protein, expressed	2,05	-1.0	1	21	69	89	UUGAACAUCCCAGAGCCACCG	CCGUGGCUCUGGAAUGUUCAG	Cleavage
tae-miR9662b-3p	Traes_6BS_843505CB3.6	PF02536				GO:GO:0006355,GO:GO:0005739,GO:GO:0003690	AT1G21150.1	Mitochondrial transcription termination factor family protein	LOC_Os06g12100.1	mTERF family protein, expressed	2,05	-1.0	1	21	68	88	UGAACAUCCCAGAGCCACCGG	CCCGUGGCUCUGGAAUGUUCA	Cleavage
tae-miR9662a-3p	Traes_6BS_843505CB3.7	PF02536				GO:GO:0006355,GO:GO:0005739,GO:GO:0003690	AT1G21150.1	Mitochondrial transcription termination factor family protein	LOC_Os06g12100.1	mTERF family protein, expressed	2,05	-1.0	1	21	69	89	UUGAACAUCCCAGAGCCACCG	CCGUGGCUCUGGAAUGUUCAG	Cleavage
tae-miR9662b-3p	Traes_6BS_843505CB3.7	PF02536				GO:GO:0006355,GO:GO:0005739,GO:GO:0003690	AT1G21150.1	Mitochondrial transcription termination factor family protein	LOC_Os06g12100.1	mTERF family protein, expressed	2,05	-1.0	1	21	68	88	UGAACAUCCCAGAGCCACCGG	CCCGUGGCUCUGGAAUGUUCA	Cleavage
tae-miR1127b-3p	Traes_6BS_94CE86A01.2										2,05	-1.0	1	21	763	783	ACAAGUAUUUCUGGACGGAGG	AAUCCGUUCAGAAUUACUUGU	Cleavage
tae-miR1127a	Traes_6BS_94CE86A01.2										1,05	-1.0	1	19	842	860	UCCUUCCGUUCGGAAUUAC	GUAAUUCCGAACGGAGGGA	Cleavage
tae-miR9662a-3p	Traes_6BS_97770F04E.1	PF02536				GO:GO:0006355,GO:GO:0005739,GO:GO:0003690	AT5G07900.1	Mitochondrial transcription termination factor family protein	LOC_Os06g12100.1	mTERF family protein, expressed	2,05	-1.0	1	21	33	53	UUGAACAUCCCAGAGCCACCG	UCGUGGCUCUGGAAUGUUCAG	Cleavage
tae-miR9662b-3p	Traes_6BS_97770F04E.1	PF02536				GO:GO:0006355,GO:GO:0005739,GO:GO:0003690	AT5G07900.1	Mitochondrial transcription termination factor family protein	LOC_Os06g12100.1	mTERF family protein, expressed	2,05	-1.0	1	21	32	52	UGAACAUCCCAGAGCCACCGG	CUCGUGGCUCUGGAAUGUUCA	Cleavage
tae-miR9662a-3p	Traes_6BS_97770F04E.3	PF02536				GO:GO:0006355,GO:GO:0005739,GO:GO:0003690	AT5G07900.1	Mitochondrial transcription termination factor family protein	LOC_Os06g12100.1	mTERF family protein, expressed	2,05	-1.0	1	21	33	53	UUGAACAUCCCAGAGCCACCG	UCGUGGCUCUGGAAUGUUCAG	Cleavage
tae-miR9662b-3p	Traes_6BS_97770F04E.3	PF02536				GO:GO:0006355,GO:GO:0005739,GO:GO:0003690	AT5G07900.1	Mitochondrial transcription termination factor family protein	LOC_Os06g12100.1	mTERF family protein, expressed	2,05	-1.0	1	21	32	52	UGAACAUCCCAGAGCCACCGG	CUCGUGGCUCUGGAAUGUUCA	Cleavage
tae-miR9662a-3p	Traes_6BS_97770F04E.4	PF02536				GO:GO:0006355,GO:GO:0005739,GO:GO:0003690	AT5G07900.1	Mitochondrial transcription termination factor family protein	LOC_Os06g12100.1	mTERF family protein, expressed	2,05	-1.0	1	21	33	53	UUGAACAUCCCAGAGCCACCG	UCGUGGCUCUGGAAUGUUCAG	Cleavage
tae-miR9662b-3p	Traes_6BS_97770F04E.4	PF02536				GO:GO:0006355,GO:GO:0005739,GO:GO:0003690	AT5G07900.1	Mitochondrial transcription termination factor family protein	LOC_Os06g12100.1	mTERF family protein, expressed	2,05	-1.0	1	21	32	52	UGAACAUCCCAGAGCCACCGG	CUCGUGGCUCUGGAAUGUUCA	Cleavage
tae-miR9662a-3p	Traes_6BS_97770F04E.5	PF02536				GO:GO:0006355,GO:GO:0005739,GO:GO:0003690	AT5G07900.1	Mitochondrial transcription termination factor family protein	LOC_Os06g12100.1	mTERF family protein, expressed	2,05	-1.0	1	21	33	53	UUGAACAUCCCAGAGCCACCG	UCGUGGCUCUGGAAUGUUCAG	Cleavage
tae-miR9662b-3p	Traes_6BS_97770F04E.5	PF02536				GO:GO:0006355,GO:GO:0005739,GO:GO:0003690	AT5G07900.1	Mitochondrial transcription termination factor family protein	LOC_Os06g12100.1	mTERF family protein, expressed	2,05	-1.0	1	21	32	52	UGAACAUCCCAGAGCCACCGG	CUCGUGGCUCUGGAAUGUUCA	Cleavage
tae-miR9662a-3p	Traes_6BS_97770F04E.6	PF02536				GO:GO:0006355,GO:GO:0005739,GO:GO:0003690	AT5G07900.1	Mitochondrial transcription termination factor family protein	LOC_Os06g12100.1	mTERF family protein, expressed	2,05	-1.0	1	21	33	53	UUGAACAUCCCAGAGCCACCG	UCGUGGCUCUGGAAUGUUCAG	Cleavage
tae-miR9662b-3p	Traes_6BS_97770F04E.6	PF02536				GO:GO:0006355,GO:GO:0005739,GO:GO:0003690	AT5G07900.1	Mitochondrial transcription termination factor family protein	LOC_Os06g12100.1	mTERF family protein, expressed	2,05	-1.0	1	21	32	52	UGAACAUCCCAGAGCCACCGG	CUCGUGGCUCUGGAAUGUUCA	Cleavage
tae-miR1137a	Traes_6BS_A4F1E63CE.2										2,00	-1.0	1	20	884	903	UAGUACAAAGUUGAGUCAUC	AGUGUCUCAACUUUGUACUA	Cleavage
tae-miR1137a	Traes_6BS_A4F1E63CE.3										2,00	-1.0	1	20	871	890	UAGUACAAAGUUGAGUCAUC	AGUGUCUCAACUUUGUACUA	Cleavage
tae-miR1128	Traes_6BS_BF5C480A9.12										2,05	-1.0	1	21	60	80	UACUACUCCCUCCGUCCGAAA	UUUGGGACGGAGGGAGUAUUA	Cleavage
tae-miR9662a-3p	Traes_6BS_C3247654A.1	PF02536				GO:GO:0006355,GO:GO:0005739,GO:GO:0003690	AT5G07900.1	Mitochondrial transcription termination factor family protein	LOC_Os06g12100.1	mTERF family protein, expressed	2,05	-1.0	1	21	57	77	UUGAACAUCCCAGAGCCACCG	CUGUGGCUCUGCGAUGUUCAG	Translation
tae-miR9662b-3p	Traes_6BS_C3247654A.1	PF02536				GO:GO:0006355,GO:GO:0005739,GO:GO:0003690	AT5G07900.1	Mitochondrial transcription termination factor family protein	LOC_Os06g12100.1	mTERF family protein, expressed	2,05	-1.0	1	21	56	76	UGAACAUCCCAGAGCCACCGG	CCUGUGGCUCUGCGAUGUUCA	Cleavage
tae-miR9662a-3p	Traes_6BS_CBD931158.1	PF02536				GO:GO:0006355,GO:GO:0005739,GO:GO:0003690	AT1G21150.1	Mitochondrial transcription termination factor family protein	LOC_Os06g12100.1	mTERF family protein, expressed	2,00	-1.0	1	21	33	53	UUGAACAUCCCAGAGCCACCG	CCGUGCCUCUGGGAUGUUCAG	Cleavage
tae-miR9662b-3p	Traes_6BS_CBD931158.1	PF02536				GO:GO:0006355,GO:GO:0005739,GO:GO:0003690	AT1G21150.1	Mitochondrial transcription termination factor family protein	LOC_Os06g12100.1	mTERF family protein, expressed	2,00	-1.0	1	21	32	52	UGAACAUCCCAGAGCCACCGG	CCCGUGCCUCUGGGAUGUUCA	Cleavage
tae-miR9662a-3p	Traes_6BS_CBD931158.2	PF02536				GO:GO:0006355,GO:GO:0005739,GO:GO:0003690	AT1G21150.1	Mitochondrial transcription termination factor family protein	LOC_Os06g12100.1	mTERF family protein, expressed	2,00	-1.0	1	21	33	53	UUGAACAUCCCAGAGCCACCG	CCGUGCCUCUGGGAUGUUCAG	Cleavage
tae-miR9662b-3p	Traes_6BS_CBD931158.2	PF02536				GO:GO:0006355,GO:GO:0005739,GO:GO:0003690	AT1G21150.1	Mitochondrial transcription termination factor family protein	LOC_Os06g12100.1	mTERF family protein, expressed	2,00	-1.0	1	21	32	52	UGAACAUCCCAGAGCCACCGG	CCCGUGCCUCUGGGAUGUUCA	Cleavage
tae-miR9662a-3p	Traes_6BS_CBD931158.3	PF02536				GO:GO:0006355,GO:GO:0005739,GO:GO:0003690	AT1G21150.1	Mitochondrial transcription termination factor family protein	LOC_Os06g12100.1	mTERF family protein, expressed	2,00	-1.0	1	21	33	53	UUGAACAUCCCAGAGCCACCG	CCGUGCCUCUGGGAUGUUCAG	Cleavage
tae-miR9662b-3p	Traes_6BS_CBD931158.3	PF02536				GO:GO:0006355,GO:GO:0005739,GO:GO:0003690	AT1G21150.1	Mitochondrial transcription termination factor family protein	LOC_Os06g12100.1	mTERF family protein, expressed	2,00	-1.0	1	21	32	52	UGAACAUCCCAGAGCCACCGG	CCCGUGCCUCUGGGAUGUUCA	Cleavage
tae-miR9662a-3p	Traes_6BS_CBD931158.4	PF02536				GO:GO:0006355,GO:GO:0005739,GO:GO:0003690	AT1G21150.1	Mitochondrial transcription termination factor family protein	LOC_Os06g12100.1	mTERF family protein, expressed	2,00	-1.0	1	21	33	53	UUGAACAUCCCAGAGCCACCG	CCGUGCCUCUGGGAUGUUCAG	Cleavage
tae-miR9662b-3p	Traes_6BS_CBD931158.4	PF02536				GO:GO:0006355,GO:GO:0005739,GO:GO:0003690	AT1G21150.1	Mitochondrial transcription termination factor family protein	LOC_Os06g12100.1	mTERF family protein, expressed	2,00	-1.0	1	21	32	52	UGAACAUCCCAGAGCCACCGG	CCCGUGCCUCUGGGAUGUUCA	Cleavage
tae-miR9662a-3p	Traes_6BS_CBD931158.5	PF02536				GO:GO:0006355,GO:GO:0005739,GO:GO:0003690	AT1G21150.1	Mitochondrial transcription termination factor family protein	LOC_Os06g12100.1	mTERF family protein, expressed	2,00	-1.0	1	21	33	53	UUGAACAUCCCAGAGCCACCG	CCGUGCCUCUGGGAUGUUCAG	Cleavage
tae-miR9662b-3p	Traes_6BS_CBD931158.5	PF02536				GO:GO:0006355,GO:GO:0005739,GO:GO:0003690	AT1G21150.1	Mitochondrial transcription termination factor family protein	LOC_Os06g12100.1	mTERF family protein, expressed	2,00	-1.0	1	21	32	52	UGAACAUCCCAGAGCCACCGG	CCCGUGCCUCUGGGAUGUUCA	Cleavage
tae-miR9662a-3p	Traes_6BS_CBD931158.6	PF02536				GO:GO:0006355,GO:GO:0005739,GO:GO:0003690	AT1G21150.1	Mitochondrial transcription termination factor family protein	LOC_Os06g12100.1	mTERF family protein, expressed	2,00	-1.0	1	21	33	53	UUGAACAUCCCAGAGCCACCG	CCGUGCCUCUGGGAUGUUCAG	Cleavage
tae-miR9662b-3p	Traes_6BS_CBD931158.6	PF02536				GO:GO:0006355,GO:GO:0005739,GO:GO:0003690	AT1G21150.1	Mitochondrial transcription termination factor family protein	LOC_Os06g12100.1	mTERF family protein, expressed	2,00	-1.0	1	21	32	52	UGAACAUCCCAGAGCCACCGG	CCCGUGCCUCUGGGAUGUUCA	Cleavage
tae-miR156	Traes_6BS_F50663DF7.1	PF03110				GO:GO:0005634,GO:GO:0003677	AT5G43270.1	squamosa promoter binding protein-like 2	LOC_Os02g04680.2	OsSPL3 - SBP-box gene family member, expressed	1,00	-1.0	1	21	1387	1407	UGACAGAAGAGAGUGAGCACA	CAUGCUCUCUCUCUUCUGUCA	Cleavage

tae-miR9662a-3p	Traes_6BS_F8176B2AA.1	PF02536				GO:GO:0006355,GO:GO:0005739,GO:GO:0003690	AT5G64950.1	Mitochondrial transcription termination factor family protein	LOC_Os06g12100.1	mTERF family protein, expressed	2,05	-1.0	1	21	204	224	UUGAACAUCCCAGAGCCACCG	CCGUGGCUCUGCGAUGUUCAG	Translation
tae-miR9662b-3p	Traes_6BS_F8176B2AA.1	PF02536				GO:GO:0006355,GO:GO:0005739,GO:GO:0003690	AT5G64950.1	Mitochondrial transcription termination factor family protein	LOC_Os06g12100.1	mTERF family protein, expressed	2,05	-1.0	1	21	203	223	UGAACAUCCCAGAGCCACCGG	CCCGUGGCUCUGCGAUGUUCA	Cleavage
tae-miR9662a-3p	Traes_6BS_F8176B2AA.2	PF02536				GO:GO:0006355,GO:GO:0005739,GO:GO:0003690	AT5G64950.1	Mitochondrial transcription termination factor family protein	LOC_Os06g12100.1	mTERF family protein, expressed	2,05	-1.0	1	21	204	224	UUGAACAUCCCAGAGCCACCG	CCGUGGCUCUGCGAUGUUCAG	Translation
tae-miR9662b-3p	Traes_6BS_F8176B2AA.2	PF02536				GO:GO:0006355,GO:GO:0005739,GO:GO:0003690	AT5G64950.1	Mitochondrial transcription termination factor family protein	LOC_Os06g12100.1	mTERF family protein, expressed	2,05	-1.0	1	21	203	223	UGAACAUCCCAGAGCCACCGG	CCCGUGGCUCUGCGAUGUUCA	Cleavage
tae-miR9662a-3p	Traes_6BS_F8176B2AA.3	PF02536				GO:GO:0006355,GO:GO:0005739,GO:GO:0003690	AT5G64950.1	Mitochondrial transcription termination factor family protein	LOC_Os06g12100.1	mTERF family protein, expressed	2,05	-1.0	1	21	198	218	UUGAACAUCCCAGAGCCACCG	CCGUGGCUCUGCGAUGUUCAG	Translation
tae-miR9662b-3p	Traes_6BS_F8176B2AA.3	PF02536				GO:GO:0006355,GO:GO:0005739,GO:GO:0003690	AT5G64950.1	Mitochondrial transcription termination factor family protein	LOC_Os06g12100.1	mTERF family protein, expressed	2,05	-1.0	1	21	197	217	UGAACAUCCCAGAGCCACCGG	CCCGUGGCUCUGCGAUGUUCA	Cleavage
tae-miR5049-3p	Traes_6DL_1E6A51BD3.10										1,05	-1.0	1	21	1321	1341	AAUAUGGAUCGGAGGGAGUAC	AUACUCCCUCCGAUCCAUAAU	Cleavage
tae-miR1122b-3p	Traes_6DL_244C908B5.14										1,05	-1.0	1	21	584	604	AGACUUAUAUGUAGGAACGGA	UCCGUUCCUAAAUAUAAGUCU	Translation
tae-miR5384-3p	Traes_6DL_5282E0007.1										2,05	-1.0	1	21	1178	1198	UGAGCGCGCCGCCGUCGAAUG	UGUUCGACGGCGGCGCGGUCG	Cleavage
tae-miR5384-3p	Traes_6DL_5282E0007.2										2,05	-1.0	1	21	1016	1036	UGAGCGCGCCGCCGUCGAAUG	UGUUCGACGGCGGCGCGGUCG	Cleavage
tae-miR1122b-3p	Traes_6DL_84E14AD8D.4										1,05	-1.0	1	21	1030	1050	AGACUUAUAUGUAGGAACGGA	UCCGUUCCUAAAUAUAAGUCU	Translation
tae-miR1122a	Traes_6DL_84E14AD8D.4										2,05	-1.0	1	20	1071	1090	UAGAUACAUCCGUAUCUAGA	ACUACAUACGGAUGUAUAUA	Cleavage
tae-miR1118	Traes_6DL_C9216369C.2										2,05	-1.0	1	23	421	443	CACUACAUUAUGGAAUGGAGGGA	UUGCUCCGUUUCAUAAUGUAGUG	Cleavage
tae-miR444b	Traes_6DL_D1C1DBD34.1										0,00	-1.0	1	21	274	294	UUGCUGCCUCAAGCUUGCUGC	GCAGCAAGCUUGAGGCAGCAA	Cleavage
tae-miR444a	Traes_6DL_D1C1DBD34.1										0,00	-1.0	1	21	274	294	UUGCUGCCUCAAGCUUGCUGC	GCAGCAAGCUUGAGGCAGCAA	Cleavage
tae-miR1117	Traes_6DL_E940A7985.1										2,05	-1.0	1	24	155	178	UAGUACCGGUUCGUGGCACGAACC	GGUUCGUGGCACAAACCGGUACUA	Cleavage
tae-miR1131	Traes_6DL_E940A7985.1										1,00	-1.0	1	22	181	202	UAGUACCGGUUCGUGGCUAACC	GAUUCGCCACGAACCGGUACUA	Cleavage
tae-miR1122a	Traes_6DS_0D9BB546D.1										2,05	-1.0	1	20	111	130	UAGAUACAUCCGUAUCUAGA	ACUACAUACGGAUGUAUAUA	Cleavage
tae-miR9662a-3p	Traes_6DS_0E84AAF77.1	PF02536				GO:GO:0006355,GO:GO:0005739,GO:GO:0003690	AT5G07900.1	Mitochondrial transcription termination factor family protein	LOC_Os06g12100.1	mTERF family protein, expressed	2,05	-1.0	1	21	480	500	UUGAACAUCCCAGAGCCACCG	CCGUGGCUCUGGGAUGCUCAG	Cleavage
tae-miR9662b-3p	Traes_6DS_0E84AAF77.1	PF02536				GO:GO:0006355,GO:GO:0005739,GO:GO:0003690	AT5G07900.1	Mitochondrial transcription termination factor family protein	LOC_Os06g12100.1	mTERF family protein, expressed	2,05	-1.0	1	21	479	499	UGAACAUCCCAGAGCCACCGG	CCCGUGGCUCUGGGAUGCUCA	Cleavage
tae-miR9674b-5p	Traes_6DS_1BEF46735.1										3,00	-1.0	1	21	537	557	AUAGCAUCAUCCAUCCUACCC	GGGCCGAAUGGAUGAUGCUAU	Cleavage
tae-miR9674b-5p	Traes_6DS_1BEF46735.1										3,00	-1.0	1	21	537	557	AUAGCAUCAUCCAUCCUACCC	GGGCCGAAUGGAUGAUGCUAU	Cleavage
tae-miR1127b-3p	Traes_6DS_2207A3BC4.7										1,05	-1.0	1	21	1729	1749	ACAAGUAUUUCUGGACGGAGG	CCUCCGUCCGGAAAUACUUGU	Cleavage
tae-miR9662a-3p	Traes_6DS_2D4EFFF3E.1	PF02536				GO:GO:0006355,GO:GO:0005739,GO:GO:0003690	AT5G07900.1	Mitochondrial transcription termination factor family protein	LOC_Os06g12100.1	mTERF family protein, expressed	1,00	-1.0	1	21	369	389	UUGAACAUCCCAGAGCCACCG	CCGUGGCUCUGGGAUGUUCAG	Cleavage
tae-miR9662b-3p	Traes_6DS_2D4EFFF3E.1	PF02536				GO:GO:0006355,GO:GO:0005739,GO:GO:0003690	AT5G07900.1	Mitochondrial transcription termination factor family protein	LOC_Os06g12100.1	mTERF family protein, expressed	1,00	-1.0	1	21	368	388	UGAACAUCCCAGAGCCACCGG	CCCGUGGCUCUGGGAUGUUCA	Cleavage
tae-miR9662a-3p	Traes_6DS_3F194116E.1	PF02536				GO:GO:0006355,GO:GO:0005739,GO:GO:0003690	AT5G07900.1	Mitochondrial transcription termination factor family protein	LOC_Os06g12100.1	mTERF family protein, expressed	1,05	-1.0	1	21	525	545	UUGAACAUCCCAGAGCCACCG	CCGUGGCUCUGGGGAUGUUCAA	Cleavage
tae-miR9662b-3p	Traes_6DS_3F194116E.1	PF02536				GO:GO:0006355,GO:GO:0005739,GO:GO:0003690	AT5G07900.1	Mitochondrial transcription termination factor family protein	LOC_Os06g12100.1	mTERF family protein, expressed	2,05	-1.0	1	21	524	544	UGAACAUCCCAGAGCCACCGG	CCCGUGGCUCUGGGGAUGUUCA	Translation
tae-miR9662a-3p	Traes_6DS_3FC2ADA87.1	PF02536				GO:GO:0006355,GO:GO:0005739,GO:GO:0003690	AT5G07900.1	Mitochondrial transcription termination factor family protein	LOC_Os06g12100.1	mTERF family protein, expressed	3,00	-1.0	1	21	242	263	UUGAACAUCCCAGAGCCAC-CG	CGCGUGGCUCUGGGAUGUUCAG	Cleavage
tae-miR9662b-3p	Traes_6DS_3FC2ADA87.1	PF02536				GO:GO:0006355,GO:GO:0005739,GO:GO:0003690	AT5G07900.1	Mitochondrial transcription termination factor family protein	LOC_Os06g12100.1	mTERF family protein, expressed	2,00	-1.0	1	21	241	262	UGAACAUCCCAGAGCCAC-CGG	CCGCGUGGCUCUGGGAUGUUCA	Cleavage
tae-miR9662a-3p	Traes_6DS_3FC2ADA87.2	PF02536				GO:GO:0006355,GO:GO:0005739,GO:GO:0003690	AT1G21150.1	Mitochondrial transcription termination factor family protein	LOC_Os06g12100.1	mTERF family protein, expressed	3,00	-1.0	1	21	65	86	UUGAACAUCCCAGAGCCAC-CG	CGCGUGGCUCUGGGAUGUUCAG	Cleavage
tae-miR9662b-3p	Traes_6DS_3FC2ADA87.2	PF02536				GO:GO:0006355,GO:GO:0005739,GO:GO:0003690	AT1G21150.1	Mitochondrial transcription termination factor family protein	LOC_Os06g12100.1	mTERF family protein, expressed	2,00	-1.0	1	21	64	85	UGAACAUCCCAGAGCCAC-CGG	CCGCGUGGCUCUGGGAUGUUCA	Cleavage
tae-miR9662a-3p	Traes_6DS_422644406.1	PF02536				GO:GO:0006355,GO:GO:0005739,GO:GO:0003690	AT5G07900.1	Mitochondrial transcription termination factor family protein	LOC_Os06g12100.1	mTERF family protein, expressed	2,05	-1.0	1	21	468	488	UUGAACAUCCCAGAGCCACCG	CCGUGGCUCUGGAAUGUUCAG	Cleavage
tae-miR9662b-3p	Traes_6DS_422644406.1	PF02536				GO:GO:0006355,GO:GO:0005739,GO:GO:0003690	AT5G07900.1	Mitochondrial transcription termination factor family protein	LOC_Os06g12100.1	mTERF family protein, expressed	2,05	-1.0	1	21	467	487	UGAACAUCCCAGAGCCACCGG	CCCGUGGCUCUGGAAUGUUCA	Cleavage
tae-miR9662a-3p	Traes_6DS_422644406.2	PF02536				GO:GO:0006355,GO:GO:0005739,GO:GO:0003690	AT5G07900.1	Mitochondrial transcription termination factor family protein	LOC_Os06g12100.1	mTERF family protein, expressed	2,05	-1.0	1	21	66	86	UUGAACAUCCCAGAGCCACCG	CCGUGGCUCUGGAAUGUUCAG	Cleavage
tae-miR9662b-3p	Traes_6DS_422644406.2	PF02536				GO:GO:0006355,GO:GO:0005739,GO:GO:0003690	AT5G07900.1	Mitochondrial transcription termination factor family protein	LOC_Os06g12100.1	mTERF family protein, expressed	2,05	-1.0	1	21	65	85	UGAACAUCCCAGAGCCACCGG	CCCGUGGCUCUGGAAUGUUCA	Cleavage
tae-miR9662a-3p	Traes_6DS_422644406.3	PF02536				GO:GO:0006355,GO:GO:0005739,GO:GO:0003690	AT5G07900.1	Mitochondrial transcription termination factor family protein	LOC_Os06g12100.1	mTERF family protein, expressed	2,05	-1.0	1	21	66	86	UUGAACAUCCCAGAGCCACCG	CCGUGGCUCUGGAAUGUUCAG	Cleavage
tae-miR9662b-3p	Traes_6DS_422644406.3	PF02536				GO:GO:0006355,GO:GO:0005739,GO:GO:0003690	AT5G07900.1	Mitochondrial transcription termination factor family protein	LOC_Os06g12100.1	mTERF family protein, expressed	2,05	-1.0	1	21	65	85	UGAACAUCCCAGAGCCACCGG	CCCGUGGCUCUGGAAUGUUCA	Cleavage
tae-miR9662a-3p	Traes_6DS_422644406.4	PF02536				GO:GO:0006355,GO:GO:0005739,GO:GO:0003690	AT5G07900.1	Mitochondrial transcription termination factor family protein	LOC_Os06g12100.1	mTERF family protein, expressed	2,05	-1.0	1	21	66	86	UUGAACAUCCCAGAGCCACCG	CCGUGGCUCUGGAAUGUUCAG	Cleavage
tae-miR9662b-3p	Traes_6DS_422644406.4	PF02536				GO:GO:0006355,GO:GO:0005739,GO:GO:0003690	AT5G07900.1	Mitochondrial transcription termination factor family protein	LOC_Os06g12100.1	mTERF family protein, expressed	2,05	-1.0	1	21	65	85	UGAACAUCCCAGAGCCACCGG	CCCGUGGCUCUGGAAUGUUCA	Cleavage
tae-miR156	Traes_6DS_7B1596298.1	PF03110				GO:GO:0005634,GO:GO:0003677	AT5G43270.2	squamosa promoter binding protein-like 2	LOC_Os02g04680.2	OsSPL3 - SBP-box gene family member, expressed	1,00	-1.0	1	21	1150	1170	UGACAGAAGAGAGUGAGCACA	CAUGCUCUCUCUCUUCUGUCA	Cleavage
tae-miR156	Traes_6DS_7B1596298.2	PF03110				GO:GO:0005634,GO:GO:0003677	AT5G43270.1	squamosa promoter binding protein-like 2	LOC_Os02g04680.2	OsSPL3 - SBP-box gene family member, expressed	1,00	-1.0	1	21	1150	1170	UGACAGAAGAGAGUGAGCACA	CAUGCUCUCUCUCUUCUGUCA	Cleavage
tae-miR156	Traes_6DS_7B1596298.4								LOC_Os02g04680.1	OsSPL3 - SBP-box gene family member, expressed	1,00	-1.0	1	21	271	291	UGACAGAAGAGAGUGAGCACA	CAUGCUCUCUCUCUUCUGUCA	Cleavage
tae-miR9674b-5p	Traes_6DS_7C389C178.1										3,00	-1.0	1	21	537	557	AUAGCAUCAUCCAUCCUACCC	GGGCCGAAUGGAUGAUGCUAU	Cleavage
tae-miR9674a-5p	Traes_6DS_7C389C178.1										2,05	-1.0	1	21	954	974	GCAUCAUCCAUCCUACCAUUC	GAAUGGAAGGAUAGAUGAUGC	Cleavage
tae-miR9674b-5p	Traes_6DS_7C389C178.1										3,00	-1.0	1	21	537	557	AUAGCAUCAUCCAUCCUACCC	GGGCCGAAUGGAUGAUGCUAU	Cleavage
tae-miR9674a-5p	Traes_6DS_7C389C178.2										2,05	-1.0	1	21	51	71	GCAUCAUCCAUCCUACCAUUC	GAAUGGAAGGAUAGAUGAUGC	Cleavage
tae-miR9674b-5p	Traes_6DS_7C389C178.3										3,00	-1.0	1	21	537	557	AUAGCAUCAUCCAUCCUACCC	GGGCCGAAUGGAUGAUGCUAU	Cleavage
tae-miR9674a-5p	Traes_6DS_7C389C178.3										2,05	-1.0	1	21	954	974	GCAUCAUCCAUCCUACCAUUC	GAAUGGAAGGAUAGAUGAUGC	Cleavage
tae-miR9674b-5p	Traes_6DS_7C389C178.3										3,00	-1.0	1	21	537	557	AUAGCAUCAUCCAUCCUACCC	GGGCCGAAUGGAUGAUGCUAU	Cleavage
tae-miR9662a-3p	Traes_6DS_7D3BEF6A9.1	PF02536				GO:GO:0006355,GO:GO:0005739,GO:GO:0003690	AT1G21150.1	Mitochondrial transcription termination factor family protein	LOC_Os06g12100.1	mTERF family protein, expressed	2,05	-1.0	1	21	33	53	UUGAACAUCCCAGAGCCACCG	UCGUGGCUCGGGGAUGUUCAG	Cleavage
tae-miR9662b-3p	Traes_6DS_7D3BEF6A9.1	PF02536				GO:GO:0006355,GO:GO:0005739,GO:GO:0003690	AT1G21150.1	Mitochondrial transcription termination factor family protein	LOC_Os06g12100.1	mTERF family protein, expressed	2,05	-1.0	1	21	32	52	UGAACAUCCCAGAGCCACCGG	CUCGUGGCUCGGGGAUGUUCA	Translation
tae-miR9662a-3p	Traes_6DS_7D3BEF6A9.2	PF02536				GO:GO:0006355,GO:GO:0005739,GO:GO:0003690	AT1G21150.1	Mitochondrial transcription termination factor family protein	LOC_Os06g12100.1	mTERF family protein, expressed	2,05	-1.0	1	21	246	266	UUGAACAUCCCAGAGCCACCG	UCGUGGCUCGGGGAUGUUCAG	Cleavage
tae-miR9662b-3p	Traes_6DS_7D3BEF6A9.2	PF02536				GO:GO:0006355,GO:GO:0005739,GO:GO:0003690	AT1G21150.1	Mitochondrial transcription termination factor family protein	LOC_Os06g12100.1	mTERF family protein, expressed	2,05	-1.0	1	21	245	265	UGAACAUCCCAGAGCCACCGG	CUCGUGGCUCGGGGAUGUUCA	Translation
tae-miR9662a-3p	Traes_6DS_7D3BEF6A9.3	PF02536				GO:GO:0006355,GO:GO:0005739,GO:GO:0003690	AT1G21150.1	Mitochondrial transcription termination factor family protein	LOC_Os06g12100.1	mTERF family protein, expressed	2,05	-1.0	1	21	246	266	UUGAACAUCCCAGAGCCACCG	UCGUGGCUCGGGGAUGUUCAG	Cleavage
tae-miR9662b-3p	Traes_6DS_7D3BEF6A9.3	PF02536				GO:GO:0006355,GO:GO:0005739,GO:GO:0003690	AT1G21150.1	Mitochondrial transcription termination factor family protein	LOC_Os06g12100.1	mTERF family protein, expressed	2,05	-1.0	1	21	245	265	UGAACAUCCCAGAGCCACCGG	CUCGUGGCUCGGGGAUGUUCA	Translation
tae-miR9662a-3p	Traes_6DS_893F318A9.1	PF02536				GO:GO:0006355,GO:GO:0005739,GO:GO:0003690	AT5G07900.1	Mitochondrial transcription termination factor family protein	LOC_Os06g12100.1	mTERF family protein, expressed	1,05	-1.0	1	21	549	569	UUGAACAUCCCAGAGCCACCG	CCGUGGCUCGGGGAUGUUCAA	Cleavage
tae-miR9662b-3p	Traes_6DS_893F318A9.1	PF02536				GO:GO:0006355,GO:GO:0005739,GO:GO:0003690	AT5G07900.1	Mitochondrial transcription termination factor family protein	LOC_Os06g12100.1	mTERF family protein, expressed	2,05	-1.0	1	21	548	568	UGAACAUCCCAGAGCCACCGG	CCCGUGGCUCGGGGAUGUUCA	Translation
tae-miR9662a-3p	Traes_6DS_893F318A9.2	PF02536				GO:GO:0006355,GO:GO:0005739,GO:GO:0003690	AT5G07900.1	Mitochondrial transcription termination factor family protein	LOC_Os06g12100.1	mTERF family protein, expressed	1,05	-1.0	1	21	132	152	UUGAACAUCCCAGAGCCACCG	CCGUGGCUCGGGGAUGUUCAA	Cleavage
tae-miR9662b-3p	Traes_6DS_893F318A9.2	PF02536				GO:GO:0006355,GO:GO:0005739,GO:GO:0003690	AT5G07900.1	Mitochondrial transcription termination factor family protein	LOC_Os06g12100.1	mTERF family protein, expressed	2,05	-1.0	1	21	131	151	UGAACAUCCCAGAGCCACCGG	CCCGUGGCUCGGGGAUGUUCA	Translation
tae-miR9662a-3p	Traes_6DS_931C28AF6.1	PF02536				GO:GO:0006355,GO:GO:0005739,GO:GO:0003690	AT1G79220.1	Mitochondrial transcription termination factor family protein	LOC_Os06g12100.1	mTERF family protein, expressed	2,05	-1.0	1	21	288	308	UUGAACAUCCCAGAGCCACCG	AUGUGGCUCUCGGAUGUUCAG	Translation
tae-miR9662a-3p	Traes_6DS_931C28AF6.2	PF02536				GO:GO:0006355,GO:GO:0005739,GO:GO:0003690	AT1G79220.1	Mitochondrial transcription termination factor family protein	LOC_Os06g12100.1	mTERF family protein, expressed	2,05	-1.0	1	21	276	296	UUGAACAUCCCAGAGCCACCG	AUGUGGCUCUCGGAUGUUCAG	Translation
tae-miR9662a-3p	Traes_6DS_931C28AF6.3	PF02536				GO:GO:0006355,GO:GO:0005739,GO:GO:0003690	AT1G79220.1	Mitochondrial transcription termination factor family protein	LOC_Os06g12100.1	mTERF family protein, expressed	2,05	-1.0	1	21	273	293	UUGAACAUCCCAGAGCCACCG	AUGUGGCUCUCGGAUGUUCAG	Translation
tae-miR9662a-3p	Traes_6DS_931C28AF6.4	PF02536				GO:GO:0006355,GO:GO:0005739,GO:GO:0003690	AT1G79220.1	Mitochondrial transcription termination factor family protein	LOC_Os06g12100.1	mTERF family protein, expressed	2,05	-1.0	1	21	273	293	UUGAACAUCCCAGAGCCACCG	AUGUGGCUCUCGGAUGUUCAG	Translation
tae-miR9662a-3p	Traes_6DS_931C28AF6.5	PF02536				GO:GO:0006355,GO:GO:0005739,GO:GO:0003690	AT1G79220.1	Mitochondrial transcription termination factor family protein	LOC_Os06g12100.1	mTERF family protein, expressed	2,05	-1.0	1	21	276	296	UUGAACAUCCCAGAGCCACCG	AUGUGGCUCUCGGAUGUUCAG	Translation
tae-miR9662b-3p	Traes_6DS_93C7DA226.1	PF02536				GO:GO:0006355,GO:GO:0005739,GO:GO:0003690	AT5G07900.1	Mitochondrial transcription termination factor family protein	LOC_Os06g12100.1	mTERF family protein, expressed	3,00	-1.0	1	21	503	523	UGAACAUCCCAGAGCCACCGG	CCCGUGGCUUCUGGGAUGUUCA	Cleavage
tae-miR9662b-3p	Traes_6DS_93C7DA2261.1	PF02536				GO:GO:0006355,GO:GO:0005739,GO:GO:0003690	AT5G07900.1	Mitochondrial transcription termination factor family protein	LOC_Os06g12100.1	mTERF family protein, expressed	3,00	-1.0	1	21	503	523	UGAACAUCCCAGAGCCACCGG	CCCGUGGCUUCUGGGAUGUUCA	Cleavage
tae-miR9662a-3p	Traes_6DS_A490DF33E.1	PF02536				GO:GO:0006355,GO:GO:0005739,GO:GO:0003690	AT5G07900.1	Mitochondrial transcription termination factor family protein	LOC_Os06g12100.1	mTERF family protein, expressed	2,05	-1.0	1	21	471	491	UUGAACAUCCCAGAGCCACCG	CCGUGGCUCUGGAAUGUUCAG	Cleavage
tae-miR9662b-3p	Traes_6DS_A490DF33E.1	PF02536				GO:GO:0006355,GO:GO:0005739,GO:GO:0003690	AT5G07900.1	Mitochondrial transcription termination factor family protein	LOC_Os06g12100.1	mTERF family protein, expressed	2,05	-1.0	1	21	470	490	UGAACAUCCCAGAGCCACCGG	CCCGUGGCUCUGGAAUGUUCA	Cleavage
tae-miR9662a-3p	Traes_6DS_A490DF33E.2	PF02536				GO:GO:0006355,GO:GO:0005739,GO:GO:0003690	AT1G21150.1	Mitochondrial transcription termination factor family protein	LOC_Os06g12100.1	mTERF family protein, expressed	2,05	-1.0	1	21	66	86	UUGAACAUCCCAGAGCCACCG	CCGUGGCUCUGGAAUGUUCAG	Cleavage
tae-miR9662b-3p	Traes_6DS_A490DF33E.2	PF02536				GO:GO:0006355,GO:GO:0005739,GO:GO:0003690	AT1G21150.1	Mitochondrial transcription termination factor family protein	LOC_Os06g12100.1	mTERF family protein, expressed	2,05	-1.0	1	21	65	85	UGAACAUCCCAGAGCCACCGG	CCCGUGGCUCUGGAAUGUUCA	Cleavage
tae-miR1122b-3p	Traes_6DS_C1B7F14A41.2										1,05	-1.0	1	21	3044	3064	AGACUUAUAUGUAGGAACGGA	UCCGUUCCUAAAUAUAAGUCU	Translation
tae-miR156	Traes_6DS_C4D93239C.1	PF03110				GO:GO:0005634,GO:GO:0003677	AT5G43270.1	squamosa promoter binding protein-like 2	LOC_Os06g45310.1	OsSPL11 - SBP-box gene family member, expressed	1,00	-1.0	1	21	577	597	UGACAGAAGAGAGUGAGCACA	CAUGCUCUCUCUCUUCUGUCA	Cleavage
tae-miR156	Traes_6DS_C4D93239C.2								LOC_Os06g45310.1	OsSPL11 - SBP-box gene family member, expressed	1,00	-1.0	1	21	88	108	UGACAGAAGAGAGUGAGCACA	CAUGCUCUCUCUCUUCUGUCA	Cleavage
tae-miR156	Traes_6DS_C4D93239C.3								LOC_Os06g45310.1	OsSPL11 - SBP-box gene family member, expressed	1,00	-1.0	1	21	64	84	UGACAGAAGAGAGUGAGCACA	CAUGCUCUCUCUCUUCUGUCA	Cleavage
tae-miR9662a-3p	Traes_6DS_CF627A1BC.1								LOC_Os06g12100.1	mTERF family protein, expressed	2,05	-1.0	1	21	75	95	UUGAACAUCCCAGAGCCACCG	CCGUGGCUCGGGGAUGUUCAG	Cleavage
tae-miR9662b-3p	Traes_6DS_CF627A1BC.1								LOC_Os06g12100.1	mTERF family protein, expressed	2,05	-1.0	1	21	74	94	UGAACAUCCCAGAGCCACCGG	CCCGUGGCUCGGGGAUGUUCA	Translation
tae-miR9662a-3p	Traes_6DS_DAD8BEF41.1	PF02536				GO:GO:0006355,GO:GO:0005739,GO:GO:0003690	AT5G07900.1	Mitochondrial transcription termination factor family protein	LOC_Os06g12100.1	mTERF family protein, expressed	2,05	-1.0	1	21	477	497	UUGAACAUCCCAGAGCCACCG	CCGUGGCUCUGGAAUGUUCAG	Cleavage
tae-miR9662b-3p	Traes_6DS_DAD8BEF41.1	PF02536				GO:GO:0006355,GO:GO:0005739,GO:GO:0003690	AT5G07900.1	Mitochondrial transcription termination factor family protein	LOC_Os06g12100.1	mTERF family protein, expressed	2,05	-1.0	1	21	476	496	UGAACAUCCCAGAGCCACCGG	CCCGUGGCUCUGGAAUGUUCA	Cleavage
tae-miR9662a-3p	Traes_6DS_E352D66A4.1	PF02536				GO:GO:0006355,GO:GO:0005739,GO:GO:0003690	AT5G64950.1	Mitochondrial transcription termination factor family protein	LOC_Os06g12100.1	mTERF family protein, expressed	2,05	-1.0	1	21	450	470	UUGAACAUCCCAGAGCCACCG	CCGUGGCUCUGGGAUGUUUAG	Cleavage
tae-miR9662b-3p	Traes_6DS_E352D66A4.1	PF02536				GO:GO:0006355,GO:GO:0005739,GO:GO:0003690	AT5G64950.1	Mitochondrial transcription termination factor family protein	LOC_Os06g12100.1	mTERF family protein, expressed	2,05	-1.0	1	21	449	469	UGAACAUCCCAGAGCCACCGG	CCCGUGGCUCUGGGAUGUUUA	Cleavage
tae-miR9662a-3p	Traes_6DS_E95930B7D.1	PF02536				GO:GO:0006355,GO:GO:0005739,GO:GO:0003690	AT5G64950.1	Mitochondrial transcription termination factor family protein	LOC_Os06g12100.1	mTERF family protein, expressed	2,05	-1.0	1	21	450	470	UUGAACAUCCCAGAGCCACCG	CCGUGGCUCUGGGAUGUUUAG	Cleavage
tae-miR9662b-3p	Traes_6DS_E95930B7D.1	PF02536				GO:GO:0006355,GO:GO:0005739,GO:GO:0003690	AT5G64950.1	Mitochondrial transcription termination factor family protein	LOC_Os06g12100.1	mTERF family protein, expressed	2,05	-1.0	1	21	449	469	UGAACAUCCCAGAGCCACCGG	CCCGUGGCUCUGGGAUGUUUA	Cleavage
tae-miR9662a-3p	Traes_6DS_EF75D5C67.1	PF02536				GO:GO:0006355,GO:GO:0005739,GO:GO:0003690	AT5G07900.1	Mitochondrial transcription termination factor family protein	LOC_Os06g12100.1	mTERF family protein, expressed	2,00	-1.0	1	21	66	86	UUGAACAUCCCAGAGCCACCG	UCGUGGCUCUGGGAUGUUCAG	Cleavage
tae-miR9662b-3p	Traes_6DS_EF75D5C67.1	PF02536				GO:GO:0006355,GO:GO:0005739,GO:GO:0003690	AT5G07900.1	Mitochondrial transcription termination factor family protein	LOC_Os06g12100.1	mTERF family protein, expressed	2,05	-1.0	1	21	65	85	UGAACAUCCCAGAGCCACCGG	CUCGUGGCGCUGGGAUGUUCA	Cleavage
tae-miR9662a-3p	Traes_6DS_F6C7E588F.1	PF02536	KOG1267			GO:GO:0006355,GO:GO:0005739,GO:GO:0003690	AT5G07900.1	Mitochondrial transcription termination factor family protein	LOC_Os06g12100.1	mTERF family protein, expressed	2,00	-1.0	1	21	657	677	UUGAACAUCCCAGAGCCACCG	CCGUGGUUCUGGGAUGUUCAG	Cleavage
tae-miR9662b-3p	Traes_6DS_F6C7E588F.1	PF02536	KOG1267			GO:GO:0006355,GO:GO:0005739,GO:GO:0003690	AT5G07900.1	Mitochondrial transcription termination factor family protein	LOC_Os06g12100.1	mTERF family protein, expressed	2,00	-1.0	1	21	656	676	UGAACAUCCCAGAGCCACCGG	CCCGUGGUUCUGGGAUGUUCA	Cleavage
tae-miR9662a-3p	Traes_6DS_FB434BB3C.1	PF02536				GO:GO:0006355,GO:GO:0005739,GO:GO:0003690	AT5G64950.1	Mitochondrial transcription termination factor family protein	LOC_Os06g12100.1	mTERF family protein, expressed	2,05	-1.0	1	21	246	266	UUGAACAUCCCAGAGCCACCG	CCGUGGCUCUCGGAUGUUCAG	Translation
tae-miR9662b-3p	Traes_6DS_FB434BB3C.1	PF02536				GO:GO:0006355,GO:GO:0005739,GO:GO:0003690	AT5G64950.1	Mitochondrial transcription termination factor family protein	LOC_Os06g12100.1	mTERF family protein, expressed	2,05	-1.0	1	21	245	265	UGAACAUCCCAGAGCCACCGG	CCCGUGGCUCUCGGAUGUUCA	Translation
tae-miR9662a-3p	Traes_6DS_FB434BB3C.2	PF02536				GO:GO:0006355,GO:GO:0005739,GO:GO:0003690	AT5G64950.1	Mitochondrial transcription termination factor family protein	LOC_Os06g12100.1	mTERF family protein, expressed	2,05	-1.0	1	21	246	266	UUGAACAUCCCAGAGCCACCG	CCGUGGCUCUCGGAUGUUCAG	Translation
tae-miR9662b-3p	Traes_6DS_FB434BB3C.2	PF02536				GO:GO:0006355,GO:GO:0005739,GO:GO:0003690	AT5G64950.1	Mitochondrial transcription termination factor family protein	LOC_Os06g12100.1	mTERF family protein, expressed	2,05	-1.0	1	21	245	265	UGAACAUCCCAGAGCCACCGG	CCCGUGGCUCUCGGAUGUUCA	Translation
tae-miR1122a	Traes_7AL_135129885.3										2,05	-1.0	1	20	721	740	UAGAUACAUCCGUAUCUAGA	ACUACAUACGGAUGUAUAUA	Cleavage
tae-miR164	Traes_7AL_628A69311.1										1,00	-1.0	1	21	650	670	UGGAGAAGCAGGGCACGUGCA	AGCAAGUGCCCUGCUUCUCCA	Cleavage
tae-miR1130b-3p	Traes_7AL_955F54182.1										0,00	-1.0	1	21	1141	1161	UCUUAUAUUAUGGGACGGAGG	CCUCCGUCCCAUAAUAUAAGA	Cleavage
tae-miR1122c-3p	Traes_7AL_955F54182.1										1,05	-1.0	1	21	1141	1161	UCUAAUAUUAUGGGACGGAGG	CCUCCGUCCCAUAAUAUAAGA	Cleavage
tae-miR1133	Traes_7AL_ADBB1224B.1										2,05	-1.0	1	22	52	73	CAUAUACUCCCUCCGUCCGAAA	UUCCGGACGGAGGGAGUAUAAU	Cleavage
tae-miR164	Traes_7AL_BE3253C8F.1										1,00	-1.0	1	21	653	673	UGGAGAAGCAGGGCACGUGCA	AGCAAGUGCCCUGCUUCUCCA	Cleavage
tae-miR164	Traes_7AL_BE3253C8F.2										1,00	-1.0	1	21	215	235	UGGAGAAGCAGGGCACGUGCA	AGCAAGUGCCCUGCUUCUCCA	Cleavage
tae-miR160	Traes_7AL_E3ADC8C38.1										0,00	-1.0	1	21	399	419	UGCCUGGCUCCCUGUAUGCCA	AGGCAUACAGGGAGCCAGGCA	Cleavage
tae-miR9666a-3p	Traes_7AL_EE3CD1CF3.10										2,00	-1.0	1	22	229	250	CGGUAGGGCUGUAUGAUGGCGA	UCCCCGUCAUACAGCCCUACCU	Cleavage
tae-miR9666a-3p	Traes_7AL_EE3CD1CF3.2										2,00	-1.0	1	22	5032	5053	CGGUAGGGCUGUAUGAUGGCGA	UCCCCGUCAUACAGCCCUACCU	Cleavage
tae-miR9666a-3p	Traes_7AL_EE3CD1CF3.3										2,00	-1.0	1	22	4948	4969	CGGUAGGGCUGUAUGAUGGCGA	UCCCCGUCAUACAGCCCUACCU	Cleavage
tae-miR9666a-3p	Traes_7AL_EE3CD1CF3.4										2,00	-1.0	1	22	4801	4822	CGGUAGGGCUGUAUGAUGGCGA	UCCCCGUCAUACAGCCCUACCU	Cleavage
tae-miR9666a-3p	Traes_7AL_EE3CD1CF3.5										2,00	-1.0	1	22	4393	4414	CGGUAGGGCUGUAUGAUGGCGA	UCCCCGUCAUACAGCCCUACCU	Cleavage
tae-miR9666a-3p	Traes_7AL_EE3CD1CF3.6										2,00	-1.0	1	22	3409	3430	CGGUAGGGCUGUAUGAUGGCGA	UCCCCGUCAUACAGCCCUACCU	Cleavage
tae-miR9666a-3p	Traes_7AL_EE3CD1CF3.8										2,00	-1.0	1	22	2941	2962	CGGUAGGGCUGUAUGAUGGCGA	UCCCCGUCAUACAGCCCUACCU	Cleavage
tae-miR1137b-5p	Traes_7AS_0F0291904.1										2,05	-1.0	1	21	844	864	UCCGUUCCAGAAUAGAUGACC	ACUCAUCUAUUCUGGGACGGU	Cleavage
tae-miR1137b-5p	Traes_7AS_0F0291904.2										2,05	-1.0	1	21	451	471	UCCGUUCCAGAAUAGAUGACC	ACUCAUCUAUUCUGGGACGGU	Cleavage
tae-miR1137b-5p	Traes_7AS_23DFA827E.1										1,05	-1.0	1	21	11	31	UCCGUUCCAGAAUAGAUGACC	AGUCAUCUAUUUUGGAACGGA	Translation
tae-miR1122a	Traes_7AS_9674F6981.1										1,05	-1.0	1	20	1467	1486	UAGAUACAUCCGUAUCUAGA	UCUAGAUACAGAUGUAUCUA	Translation
tae-miR1122a	Traes_7AS_9674F6981.2										1,05	-1.0	1	20	1473	1492	UAGAUACAUCCGUAUCUAGA	UCUAGAUACAGAUGUAUCUA	Translation
tae-miR1122a	Traes_7AS_9674F6981.3										1,05	-1.0	1	20	1467	1486	UAGAUACAUCCGUAUCUAGA	UCUAGAUACAGAUGUAUCUA	Translation
tae-miR1130b-3p	Traes_7AS_9819F7C48.1										0,00	-1.0	1	21	1268	1288	UCUUAUAUUAUGGGACGGAGG	AAUCCGUCCCAUAAUAUAAGA	Cleavage
tae-miR1122c-3p	Traes_7AS_9819F7C48.1										1,05	-1.0	1	21	1268	1288	UCUAAUAUUAUGGGACGGAGG	AAUCCGUCCCAUAAUAUAAGA	Cleavage
tae-miR1120c-5p	Traes_7AS_9819F7C48.1										1,05	-1.0	1	21	1311	1331	UAAUAUAAGAACGUUUUUGAC	GUCAAAAACGCUCUUAUAUUA	Translation
tae-miR7757-5p	Traes_7AS_E43D64A80.1	PF00931	KOG4658			GO:GO:0043531	AT3G46530.1	NB-ARC domain-containing disease resistance protein	LOC_Os07g26430.1	resistance protein, putative, expressed	2,05	-1.0	1	22	377	398	AUAAAACCUUCAGCUAUCCAUC	AAUGGAUAACUGAAGAUUUUAU	Cleavage
tae-miR7757-5p	Traes_7AS_E43D64A80.2	PF00931	KOG4658			GO:GO:0043531	AT3G46530.1	NB-ARC domain-containing disease resistance protein	LOC_Os07g26430.1	resistance protein, putative, expressed	2,05	-1.0	1	22	1424	1445	AUAAAACCUUCAGCUAUCCAUC	AAUGGAUAACUGAAGAUUUUAU	Cleavage
tae-miR156	Traes_7AS_FE67F9E86.1								LOC_Os08g41940.1	OsSPL16 - SBP-box gene family member, expressed	1,00	-1.0	1	21	358	378	UGACAGAAGAGAGUGAGCACA	UGUGCUCUCUCUCUUCUGUCA	Cleavage
tae-miR156	Traes_7AS_FE67F9E86.2								LOC_Os08g41940.1	OsSPL16 - SBP-box gene family member, expressed	1,00	-1.0	1	21	358	378	UGACAGAAGAGAGUGAGCACA	UGUGCUCUCUCUCUUCUGUCA	Cleavage
tae-miR156	Traes_7AS_FE67F9E86.3								LOC_Os08g41940.1	OsSPL16 - SBP-box gene family member, expressed	1,00	-1.0	1	21	331	351	UGACAGAAGAGAGUGAGCACA	UGUGCUCUCUCUCUUCUGUCA	Cleavage
tae-miR9654a-3p	Traes_7BL_03C3797A2.1										2,00	-1.0	1	22	1813	1834	UUCUGAAAGGCUUGAAGCGAAU	GAGGGCCUCAAGCCUUUCAGAA	Cleavage
tae-miR9654a-3p	Traes_7BL_03C3797A2.2										2,00	-1.0	1	22	1813	1834	UUCUGAAAGGCUUGAAGCGAAU	GAGGGCCUCAAGCCUUUCAGAA	Cleavage
tae-miR9654a-3p	Traes_7BL_03C3797A2.3										2,00	-1.0	1	22	1513	1534	UUCUGAAAGGCUUGAAGCGAAU	GAGGGCCUCAAGCCUUUCAGAA	Cleavage
tae-miR9654a-3p	Traes_7BL_03C3797A2.4										2,00	-1.0	1	22	1513	1534	UUCUGAAAGGCUUGAAGCGAAU	GAGGGCCUCAAGCCUUUCAGAA	Cleavage
tae-miR9654a-3p	Traes_7BL_03C3797A2.5										2,00	-1.0	1	22	1399	1420	UUCUGAAAGGCUUGAAGCGAAU	GAGGGCCUCAAGCCUUUCAGAA	Cleavage
tae-miR9654a-3p	Traes_7BL_03C3797A2.6										2,00	-1.0	1	22	973	994	UUCUGAAAGGCUUGAAGCGAAU	GAGGGCCUCAAGCCUUUCAGAA	Cleavage
tae-miR9654a-3p	Traes_7BL_03C3797A2.7										2,00	-1.0	1	22	1813	1834	UUCUGAAAGGCUUGAAGCGAAU	GAGGGCCUCAAGCCUUUCAGAA	Cleavage
tae-miR1130b-3p	Traes_7BL_0665E8707.6										1,00	-1.0	1	21	46	66	UCUUAUAUUAUGGGACGGAGG	CCUCCGUCCCAUAAUAUAAGU	Cleavage
tae-miR1122c-3p	Traes_7BL_0665E8707.6										2,05	-1.0	1	21	46	66	UCUAAUAUUAUGGGACGGAGG	CCUCCGUCCCAUAAUAUAAGU	Cleavage
tae-miR1133	Traes_7BL_07C4E8081.43										0,00	-1.0	1	22	46	67	CAUAUACUCCCUCCGUCCGAAA	UUUCGGACGGAGGGAGUAUAUG	Cleavage
tae-miR1133	Traes_7BL_07C4E8081.44										0,00	-1.0	1	22	46	67	CAUAUACUCCCUCCGUCCGAAA	UUUCGGACGGAGGGAGUAUAUG	Cleavage
tae-miR1133	Traes_7BL_07C4E8081.45										0,00	-1.0	1	22	46	67	CAUAUACUCCCUCCGUCCGAAA	UUUCGGACGGAGGGAGUAUAUG	Cleavage
tae-miR160	Traes_7BL_18D335F08.1										0,00	-1.0	1	21	171	191	UGCCUGGCUCCCUGUAUGCCA	AGGCAUACAGGGAGCCAGGCA	Cleavage
tae-miR160	Traes_7BL_18D335F08.2										0,00	-1.0	1	21	171	191	UGCCUGGCUCCCUGUAUGCCA	AGGCAUACAGGGAGCCAGGCA	Cleavage
tae-miR1137a	Traes_7BL_C184D0525.2										0,00	-1.0	1	20	913	932	UAGUACAAAGUUGAGUCAUC	GAUGACUCAACUUUGUACUA	Cleavage
tae-miR395b	Traes_7BL_DC0D8773C.1										2,05	-1.0	1	20	1385	1404	UGAAGUGUUUGGGGGAACUC	CAGUUCACCCAAAAACUUCA	Cleavage
tae-miR395b	Traes_7BL_DC0D8773C.10										2,05	-1.0	1	20	1148	1167	UGAAGUGUUUGGGGGAACUC	CAGUUCACCCAAAAACUUCA	Cleavage
tae-miR395b	Traes_7BL_DC0D8773C.11										2,05	-1.0	1	20	848	867	UGAAGUGUUUGGGGGAACUC	CAGUUCACCCAAAAACUUCA	Cleavage
tae-miR395b	Traes_7BL_DC0D8773C.12										2,05	-1.0	1	20	848	867	UGAAGUGUUUGGGGGAACUC	CAGUUCACCCAAAAACUUCA	Cleavage
tae-miR395b	Traes_7BL_DC0D8773C.13										2,05	-1.0	1	20	848	867	UGAAGUGUUUGGGGGAACUC	CAGUUCACCCAAAAACUUCA	Cleavage
tae-miR395b	Traes_7BL_DC0D8773C.14										2,05	-1.0	1	20	848	867	UGAAGUGUUUGGGGGAACUC	CAGUUCACCCAAAAACUUCA	Cleavage
tae-miR395b	Traes_7BL_DC0D8773C.2										2,05	-1.0	1	20	1385	1404	UGAAGUGUUUGGGGGAACUC	CAGUUCACCCAAAAACUUCA	Cleavage
tae-miR395b	Traes_7BL_DC0D8773C.3										2,05	-1.0	1	20	1385	1404	UGAAGUGUUUGGGGGAACUC	CAGUUCACCCAAAAACUUCA	Cleavage
tae-miR395b	Traes_7BL_DC0D8773C.4										2,05	-1.0	1	20	1385	1404	UGAAGUGUUUGGGGGAACUC	CAGUUCACCCAAAAACUUCA	Cleavage
tae-miR395b	Traes_7BL_DC0D8773C.5										2,05	-1.0	1	20	1385	1404	UGAAGUGUUUGGGGGAACUC	CAGUUCACCCAAAAACUUCA	Cleavage
tae-miR395b	Traes_7BL_DC0D8773C.6										2,05	-1.0	1	20	1148	1167	UGAAGUGUUUGGGGGAACUC	CAGUUCACCCAAAAACUUCA	Cleavage
tae-miR395b	Traes_7BL_DC0D8773C.7										2,05	-1.0	1	20	1148	1167	UGAAGUGUUUGGGGGAACUC	CAGUUCACCCAAAAACUUCA	Cleavage
tae-miR395b	Traes_7BL_DC0D8773C.8										2,05	-1.0	1	20	1148	1167	UGAAGUGUUUGGGGGAACUC	CAGUUCACCCAAAAACUUCA	Cleavage
tae-miR395b	Traes_7BL_DC0D8773C.9										2,05	-1.0	1	20	1148	1167	UGAAGUGUUUGGGGGAACUC	CAGUUCACCCAAAAACUUCA	Cleavage
tae-miR164	Traes_7BL_F1A0270C6.1										1,00	-1.0	1	21	32	52	UGGAGAAGCAGGGCACGUGCA	AGCAAGUGCCCUGCUUCUCCA	Cleavage
tae-miR9666a-3p	Traes_7BL_F8D5E60B1.1										2,00	-1.0	1	22	5050	5071	CGGUAGGGCUGUAUGAUGGCGA	UCCCCCUCAUACAGCCCUACCU	Cleavage

tae-miR9666a-3p	Traes_7BL_F8D5E60B1.1										2,05	-1.0	1	22	4966	4987	CGGUAGGGCUGUAUGAUGGCGA	UCGCCAUCAUACAGCCCUACUU	Cleavage
tae-miR9666a-3p	Traes_7BL_F8D5E60B1.2										2,00	-1.0	1	22	5011	5032	CGGUAGGGCUGUAUGAUGGCGA	UCCCCCUCAUACAGCCCUACCU	Cleavage
tae-miR9666a-3p	Traes_7BL_F8D5E60B1.2										2,05	-1.0	1	22	4927	4948	CGGUAGGGCUGUAUGAUGGCGA	UCGCCAUCAUACAGCCCUACUU	Cleavage
tae-miR9666a-3p	Traes_7BL_F8D5E60B1.3										2,00	-1.0	1	22	4780	4801	CGGUAGGGCUGUAUGAUGGCGA	UCCCCCUCAUACAGCCCUACCU	Cleavage
tae-miR9666a-3p	Traes_7BL_F8D5E60B1.3										2,05	-1.0	1	22	4696	4717	CGGUAGGGCUGUAUGAUGGCGA	UCGCCAUCAUACAGCCCUACUU	Cleavage
tae-miR9666a-3p	Traes_7BL_F8D5E60B1.4										2,00	-1.0	1	22	3409	3430	CGGUAGGGCUGUAUGAUGGCGA	UCCCCCUCAUACAGCCCUACCU	Cleavage
tae-miR9666a-3p	Traes_7BL_F8D5E60B1.4										2,05	-1.0	1	22	3325	3346	CGGUAGGGCUGUAUGAUGGCGA	UCGCCAUCAUACAGCCCUACUU	Cleavage
tae-miR1137a	Traes_7BS_2A6C29F49.3										2,00	-1.0	1	20	75	94	UAGUACAAAGUUGAGUCAUC	AGUGUCUCAACUUUGUACUA	Cleavage
tae-miR1137a	Traes_7BS_2A6C29F49.4										2,00	-1.0	1	20	81	100	UAGUACAAAGUUGAGUCAUC	AGUGUCUCAACUUUGUACUA	Cleavage
tae-miR1130b-3p	Traes_7BS_47B8FC9CB.2										0,00	-1.0	1	21	1268	1288	UCUUAUAUUAUGGGACGGAGG	CCUCCGUCCCAUAAUAUAAGA	Cleavage
tae-miR1122c-3p	Traes_7BS_47B8FC9CB.2										1,05	-1.0	1	21	1268	1288	UCUAAUAUUAUGGGACGGAGG	CCUCCGUCCCAUAAUAUAAGA	Cleavage
tae-miR1120c-5p	Traes_7BS_47B8FC9CB.2										1,05	-1.0	1	21	1311	1331	UAAUAUAAGAACGUUUUUGAC	GUCAAAAACGCUCUUAUAUUA	Translation
tae-miR1130b-3p	Traes_7BS_47B8FC9CB.3										0,00	-1.0	1	21	1265	1285	UCUUAUAUUAUGGGACGGAGG	CCUCCGUCCCAUAAUAUAAGA	Cleavage
tae-miR1122c-3p	Traes_7BS_47B8FC9CB.3										1,05	-1.0	1	21	1265	1285	UCUAAUAUUAUGGGACGGAGG	CCUCCGUCCCAUAAUAUAAGA	Cleavage
tae-miR1120c-5p	Traes_7BS_47B8FC9CB.3										1,05	-1.0	1	21	1308	1328	UAAUAUAAGAACGUUUUUGAC	GUCAAAAACGCUCUUAUAUUA	Translation
tae-miR9662a-3p	Traes_7BS_598310C32.1	PF02536				GO:GO:0006355,GO:GO:0005739,GO:GO:0003690	AT1G21150.1	Mitochondrial transcription termination factor family protein	LOC_Os06g12100.1	mTERF family protein, expressed	2,05	-1.0	1	21	33	53	UUGAACAUCCCAGAGCCACCG	CCGUGGCUCUGCGAUGUUCAG	Translation
tae-miR9662b-3p	Traes_7BS_598310C32.1	PF02536				GO:GO:0006355,GO:GO:0005739,GO:GO:0003690	AT1G21150.1	Mitochondrial transcription termination factor family protein	LOC_Os06g12100.1	mTERF family protein, expressed	2,05	-1.0	1	21	32	52	UGAACAUCCCAGAGCCACCGG	CCCGUGGCUCUGCGAUGUUCA	Cleavage
tae-miR1121	Traes_7BS_5A0E77FB8.2										1,00	-1.0	1	22	3478	3499	AGUAGUGAUCUAAACGCUCUUA	UAAGAGUGUUUAGAUCACUACU	Cleavage
tae-miR1137a	Traes_7BS_6716D1D07.2										0,00	-1.0	1	20	347	366	UAGUACAAAGUUGAGUCAUC	GAUGACUCAACUUUGUACUA	Cleavage
tae-miR9781	Traes_7BS_A42F893FC.1										1,05	-1.0	1	21	117	137	UUUUGUCACAUAUAAUACAUA	UAUGUAUUUUAUGUGACAAAA	Cleavage
tae-miR9664-3p	Traes_7DL_101A9F756.1	PF00931	KOG4658			GO:GO:0043531	AT1G58807.1	Disease resistance protein (CC-NBS-LRR class) family	LOC_Os11g11550.1	NBS-LRR disease resistance protein, putative, expressed	2,05	-1.0	1	21	102	122	UUGCAGUCCUCGAUGUCGUAG	GCACGACAUCGAGGAUUGCAU	Cleavage
tae-miR9664-3p	Traes_7DL_101A9F756.2	PF00931	KOG4658			GO:GO:0043531	AT1G58807.1	Disease resistance protein (CC-NBS-LRR class) family	LOC_Os11g11550.2	NBS-LRR disease resistance protein, putative, expressed	2,05	-1.0	1	21	102	122	UUGCAGUCCUCGAUGUCGUAG	GCACGACAUCGAGGAUUGCAU	Cleavage
tae-miR9664-3p	Traes_7DL_101A9F756.3	PF00931	KOG4658			GO:GO:0043531	AT1G58807.1	Disease resistance protein (CC-NBS-LRR class) family	LOC_Os11g11550.1	NBS-LRR disease resistance protein, putative, expressed	2,05	-1.0	1	21	231	251	UUGCAGUCCUCGAUGUCGUAG	GCACGACAUCGAGGAUUGCAU	Cleavage
tae-miR9666a-3p	Traes_7DL_18F2A8567.1										2,05	-1.0	1	22	4927	4948	CGGUAGGGCUGUAUGAUGGCGA	UCGCCAUCAUACAGCCCUACUU	Cleavage
tae-miR9666a-3p	Traes_7DL_18F2A8567.11										2,00	-1.0	1	22	244	265	CGGUAGGGCUGUAUGAUGGCGA	UCGCCCUCAUACAGCCCUACCU	Cleavage
tae-miR9666a-3p	Traes_7DL_18F2A8567.11										2,05	-1.0	1	22	139	160	CGGUAGGGCUGUAUGAUGGCGA	UCGCCAUCAUACAGCCCUACUU	Cleavage
tae-miR9666a-3p	Traes_7DL_18F2A8567.12										2,00	-1.0	1	22	250	271	CGGUAGGGCUGUAUGAUGGCGA	UCGCCCUCAUACAGCCCUACCU	Cleavage
tae-miR9666a-3p	Traes_7DL_18F2A8567.12										2,05	-1.0	1	22	145	166	CGGUAGGGCUGUAUGAUGGCGA	UCGCCAUCAUACAGCCCUACUU	Cleavage
tae-miR9666a-3p	Traes_7DL_18F2A8567.14										2,00	-1.0	1	22	201	222	CGGUAGGGCUGUAUGAUGGCGA	UCGCCCUCAUACAGCCCUACCU	Cleavage
tae-miR9666a-3p	Traes_7DL_18F2A8567.14										2,05	-1.0	1	22	96	117	CGGUAGGGCUGUAUGAUGGCGA	UCGCCAUCAUACAGCCCUACUU	Cleavage
tae-miR9666a-3p	Traes_7DL_18F2A8567.2										2,00	-1.0	1	22	5032	5053	CGGUAGGGCUGUAUGAUGGCGA	UCGCCCUCAUACAGCCCUACCU	Cleavage
tae-miR9666a-3p	Traes_7DL_18F2A8567.2										2,05	-1.0	1	22	4927	4948	CGGUAGGGCUGUAUGAUGGCGA	UCGCCAUCAUACAGCCCUACUU	Cleavage
tae-miR9666a-3p	Traes_7DL_18F2A8567.3										2,00	-1.0	1	22	4969	4990	CGGUAGGGCUGUAUGAUGGCGA	UCGCCCUCAUACAGCCCUACCU	Cleavage
tae-miR9666a-3p	Traes_7DL_18F2A8567.3										2,05	-1.0	1	22	4927	4948	CGGUAGGGCUGUAUGAUGGCGA	UCGCCAUCAUACAGCCCUACUU	Cleavage
tae-miR9666a-3p	Traes_7DL_18F2A8567.4										2,05	-1.0	1	22	4927	4948	CGGUAGGGCUGUAUGAUGGCGA	UCGCCAUCAUACAGCCCUACUU	Cleavage
tae-miR9666a-3p	Traes_7DL_18F2A8567.5										2,05	-1.0	1	22	4927	4948	CGGUAGGGCUGUAUGAUGGCGA	UCGCCAUCAUACAGCCCUACUU	Cleavage
tae-miR9666a-3p	Traes_7DL_18F2A8567.6										2,00	-1.0	1	22	5032	5053	CGGUAGGGCUGUAUGAUGGCGA	UCGCCCUCAUACAGCCCUACCU	Cleavage
tae-miR9666a-3p	Traes_7DL_18F2A8567.6										2,05	-1.0	1	22	4927	4948	CGGUAGGGCUGUAUGAUGGCGA	UCGCCAUCAUACAGCCCUACUU	Cleavage
tae-miR9666a-3p	Traes_7DL_18F2A8567.7										2,00	-1.0	1	22	4801	4822	CGGUAGGGCUGUAUGAUGGCGA	UCGCCCUCAUACAGCCCUACCU	Cleavage
tae-miR9666a-3p	Traes_7DL_18F2A8567.7										2,05	-1.0	1	22	4696	4717	CGGUAGGGCUGUAUGAUGGCGA	UCGCCAUCAUACAGCCCUACUU	Cleavage
tae-miR9666a-3p	Traes_7DL_18F2A8567.8										2,00	-1.0	1	22	3409	3430	CGGUAGGGCUGUAUGAUGGCGA	UCGCCCUCAUACAGCCCUACCU	Cleavage
tae-miR9666a-3p	Traes_7DL_18F2A8567.8										2,05	-1.0	1	22	3304	3325	CGGUAGGGCUGUAUGAUGGCGA	UCGCCAUCAUACAGCCCUACUU	Cleavage
tae-miR9666a-3p	Traes_7DL_18F2A8567.9										2,00	-1.0	1	22	2941	2962	CGGUAGGGCUGUAUGAUGGCGA	UCGCCCUCAUACAGCCCUACCU	Cleavage
tae-miR9666a-3p	Traes_7DL_18F2A8567.9										2,05	-1.0	1	22	2836	2857	CGGUAGGGCUGUAUGAUGGCGA	UCGCCAUCAUACAGCCCUACUU	Cleavage
tae-miR164	Traes_7DL_1E343B6E6.1										1,00	-1.0	1	21	236	256	UGGAGAAGCAGGGCACGUGCA	AGCAAGUGCCCUGCUUCUCCA	Cleavage
tae-miR164	Traes_7DL_1E343B6E6.2										1,00	-1.0	1	21	32	52	UGGAGAAGCAGGGCACGUGCA	AGCAAGUGCCCUGCUUCUCCA	Cleavage
tae-miR1135	Traes_7DL_20BFD8A19.4	PF00145		2.1.1.37			AT4G19020.1	chromomethylase 2	LOC_Os10g01570.1	C-5 cytosine-specific DNA methylase, putative, expressed	1,05	-1.0	1	24	954	977	CUGCGACAAGUAAUUCCGAACGGA	UCUGUUCGGAAUUACUUGUCGUAG	Cleavage
tae-miR1127a	Traes_7DL_20BFD8A19.4	PF00145		2.1.1.37			AT4G19020.1	chromomethylase 2	LOC_Os10g01570.1	C-5 cytosine-specific DNA methylase, putative, expressed	1,05	-1.0	1	19	1032	1050	UCCUUCCGUUCGGAAUUAC	GUAAUUCCGAACGGAGGGA	Cleavage
tae-miR9778	Traes_7DL_31A23053A.1										1,00	-1.0	1	21	132	152	UGCAUCAUCUCGAACUCGUCG	CCUCGAGUUCGAGAUGAUGCA	Cleavage
tae-miR1122a	Traes_7DL_37135CE6E.2										2,05	-1.0	1	20	2105	2124	UAGAUACAUCCGUAUCUAGA	UCUAAAUACGGAUGUAUCAA	Cleavage
tae-miR1122a	Traes_7DL_37135CE6E.3										2,05	-1.0	1	20	2090	2109	UAGAUACAUCCGUAUCUAGA	UCUAAAUACGGAUGUAUCAA	Cleavage
tae-miR1122a	Traes_7DL_37135CE6E.6										2,05	-1.0	1	20	1241	1260	UAGAUACAUCCGUAUCUAGA	UCUAAAUACGGAUGUAUCAA	Cleavage
tae-miR9661-5p	Traes_7DL_55AC0BE09.1										2,05	-1.0	1	21	143	163	UGAAGUAGAGCAGGGACCUCA	UCAGGUCCCUGCGCUACUUCG	Cleavage
tae-miR9661-5p	Traes_7DL_55AC0BE09.2										2,05	-1.0	1	21	143	163	UGAAGUAGAGCAGGGACCUCA	UCAGGUCCCUGCGCUACUUCG	Cleavage
tae-miR9661-5p	Traes_7DL_55AC0BE09.3										2,05	-1.0	1	21	116	136	UGAAGUAGAGCAGGGACCUCA	UCAGGUCCCUGCGCUACUUCG	Cleavage
tae-miR9661-5p	Traes_7DL_55AC0BE09.4										2,05	-1.0	1	21	116	136	UGAAGUAGAGCAGGGACCUCA	UCAGGUCCCUGCGCUACUUCG	Cleavage
tae-miR9661-5p	Traes_7DL_55AC0BE09.5										2,05	-1.0	1	21	116	136	UGAAGUAGAGCAGGGACCUCA	UCAGGUCCCUGCGCUACUUCG	Cleavage
tae-miR160	Traes_7DL_55ADB3528.1										0,00	-1.0	1	21	714	734	UGCCUGGCUCCCUGUAUGCCA	AGGCAUACAGGGAGCCAGGCA	Cleavage
tae-miR160	Traes_7DL_55ADB3528.2										0,00	-1.0	1	21	714	734	UGCCUGGCUCCCUGUAUGCCA	AGGCAUACAGGGAGCCAGGCA	Cleavage
tae-miR1134	Traes_7DL_5E2559796.1										2,00	-1.0	1	24	2562	2585	CAACAACAACAAGAAGAAGAAGAU	UUGUUGUUGUUCUUGUUGUUGUUG	Cleavage
tae-miR1134	Traes_7DL_5E2559796.2										2,00	-1.0	1	24	2556	2579	CAACAACAACAAGAAGAAGAAGAU	UUGUUGUUGUUCUUGUUGUUGUUG	Cleavage
tae-miR1122b-3p	Traes_7DL_613007B24.2										1,05	-1.0	1	21	1492	1512	AGACUUAUAUGUAGGAACGGA	UCCGUUCCUAAAUAUAAGUCU	Translation
tae-miR164	Traes_7DL_91CFE828F.1										1,00	-1.0	1	21	497	517	UGGAGAAGCAGGGCACGUGCA	AGCAAGUGCCCUGCUUCUCCA	Cleavage
tae-miR164	Traes_7DL_91CFE828F.2										1,00	-1.0	1	21	296	316	UGGAGAAGCAGGGCACGUGCA	AGCAAGUGCCCUGCUUCUCCA	Cleavage
tae-miR9778	Traes_7DL_9F0AD190A.2										2,05	-1.0	1	21	126	146	UGCAUCAUCUCGAACUCGUCG	AGGCGAGUUUGAGAUGAUGCA	Cleavage
tae-miR9778	Traes_7DL_9F0AD190A.3										2,05	-1.0	1	21	126	146	UGCAUCAUCUCGAACUCGUCG	AGGCGAGUUUGAGAUGAUGCA	Cleavage
tae-miR5048-5p	Traes_7DL_AB7C59D49.1	PF00069		2.7.11.1		GO:GO:0006468,GO:GO:0005524,GO:GO:0004672	AT4G21230.1	cysteine-rich RLK (RECEPTOR-like protein kinase) 27	LOC_Os11g12270.2	expressed protein	0,00	-1.0	1	22	53	74	UUUGCAGGUUUUAGGUCUAAGU	ACUUAGACCUAAAACCUGCAAA	Cleavage
tae-miR5048-5p	Traes_7DL_AB7C59D49.2	PF00069		2.7.11.1		GO:GO:0006468,GO:GO:0005524,GO:GO:0004672	AT1G11300.1	protein serine/threonine kinases;protein kinases;ATP binding;sugar binding;kinases;carbohydrate binding	LOC_Os11g17380.1	protein kinase domain containing protein, expressed	0,00	-1.0	1	22	53	74	UUUGCAGGUUUUAGGUCUAAGU	ACUUAGACCUAAAACCUGCAAA	Cleavage
tae-miR5048-5p	Traes_7DL_AB7C59D49.3	PF00069		2.7.11.1		GO:GO:0006468,GO:GO:0005524,GO:GO:0004672	AT1G11300.1	protein serine/threonine kinases;protein kinases;ATP binding;sugar binding;kinases;carbohydrate binding	LOC_Os11g17380.1	protein kinase domain containing protein, expressed	0,00	-1.0	1	22	53	74	UUUGCAGGUUUUAGGUCUAAGU	ACUUAGACCUAAAACCUGCAAA	Cleavage
tae-miR5048-5p	Traes_7DL_AB7C59D49.4	PF00069		2.7.11.1		GO:GO:0006468,GO:GO:0005524,GO:GO:0004672	AT1G11300.1	protein serine/threonine kinases;protein kinases;ATP binding;sugar binding;kinases;carbohydrate binding	LOC_Os11g17380.1	protein kinase domain containing protein, expressed	0,00	-1.0	1	22	53	74	UUUGCAGGUUUUAGGUCUAAGU	ACUUAGACCUAAAACCUGCAAA	Cleavage
tae-miR5048-5p	Traes_7DL_AB7C59D49.5	PF00069		2.7.11.1		GO:GO:0006468,GO:GO:0005524,GO:GO:0004672	AT4G21230.1	cysteine-rich RLK (RECEPTOR-like protein kinase) 27	LOC_Os11g12270.2	expressed protein	0,00	-1.0	1	22	53	74	UUUGCAGGUUUUAGGUCUAAGU	ACUUAGACCUAAAACCUGCAAA	Cleavage
tae-miR5048-5p	Traes_7DL_AB7C59D49.6	PF00069		2.7.11.1		GO:GO:0006468,GO:GO:0005524,GO:GO:0004672	AT4G21230.1	cysteine-rich RLK (RECEPTOR-like protein kinase) 27	LOC_Os11g12270.2	expressed protein	0,00	-1.0	1	22	53	74	UUUGCAGGUUUUAGGUCUAAGU	ACUUAGACCUAAAACCUGCAAA	Cleavage
tae-miR1122a	Traes_7DL_C074DC1F6.3										2,05	-1.0	1	20	109	128	UAGAUACAUCCGUAUCUAGA	ACUACAUACGGAUGUAUAUA	Cleavage
tae-miR395b	Traes_7DL_CB073ADC6.1										2,05	-1.0	1	20	1598	1617	UGAAGUGUUUGGGGGAACUC	CAGUUCACCCAAAAACUUCA	Cleavage
tae-miR395b	Traes_7DL_CB073ADC6.10										2,05	-1.0	1	20	851	870	UGAAGUGUUUGGGGGAACUC	CAGUUCACCCAAAAACUUCA	Cleavage
tae-miR395b	Traes_7DL_CB073ADC6.11										2,05	-1.0	1	20	851	870	UGAAGUGUUUGGGGGAACUC	CAGUUCACCCAAAAACUUCA	Cleavage
tae-miR395b	Traes_7DL_CB073ADC6.12										2,05	-1.0	1	20	851	870	UGAAGUGUUUGGGGGAACUC	CAGUUCACCCAAAAACUUCA	Cleavage
tae-miR395b	Traes_7DL_CB073ADC6.2										2,05	-1.0	1	20	1598	1617	UGAAGUGUUUGGGGGAACUC	CAGUUCACCCAAAAACUUCA	Cleavage
tae-miR395b	Traes_7DL_CB073ADC6.3										2,05	-1.0	1	20	1598	1617	UGAAGUGUUUGGGGGAACUC	CAGUUCACCCAAAAACUUCA	Cleavage
tae-miR395b	Traes_7DL_CB073ADC6.4										2,05	-1.0	1	20	1157	1176	UGAAGUGUUUGGGGGAACUC	CAGUUCACCCAAAAACUUCA	Cleavage
tae-miR395b	Traes_7DL_CB073ADC6.5										2,05	-1.0	1	20	1157	1176	UGAAGUGUUUGGGGGAACUC	CAGUUCACCCAAAAACUUCA	Cleavage
tae-miR395b	Traes_7DL_CB073ADC6.6										2,05	-1.0	1	20	1157	1176	UGAAGUGUUUGGGGGAACUC	CAGUUCACCCAAAAACUUCA	Cleavage
tae-miR395b	Traes_7DL_CB073ADC6.7										2,05	-1.0	1	20	1157	1176	UGAAGUGUUUGGGGGAACUC	CAGUUCACCCAAAAACUUCA	Cleavage
tae-miR395b	Traes_7DL_CB073ADC6.8										2,05	-1.0	1	20	1079	1098	UGAAGUGUUUGGGGGAACUC	CAGUUCACCCAAAAACUUCA	Cleavage
tae-miR395b	Traes_7DL_CB073ADC6.9										2,05	-1.0	1	20	851	870	UGAAGUGUUUGGGGGAACUC	CAGUUCACCCAAAAACUUCA	Cleavage
tae-miR1130b-3p	Traes_7DL_CF6AABA1F.2										0,00	-1.0	1	21	1271	1291	UCUUAUAUUAUGGGACGGAGG	CCUCCGUCCCAUAAUAUAAGA	Cleavage
tae-miR1122c-3p	Traes_7DL_CF6AABA1F.2										1,05	-1.0	1	21	1271	1291	UCUAAUAUUAUGGGACGGAGG	CCUCCGUCCCAUAAUAUAAGA	Cleavage
tae-miR1128	Traes_7DL_D5FCF9F33.4										2,05	-1.0	1	21	96	116	UACUACUCCCUCCGUCCGAAA	UUUGGGACGGAGGGAGUAUUA	Cleavage
tae-miR1122a	Traes_7DL_D5FCF9F33.4										0,00	-1.0	1	20	23	42	UAGAUACAUCCGUAUCUAGA	UCUAGAUACGGAUGUAUCUA	Cleavage
tae-miR5049-3p	Traes_7DS_06CD5BBFB.4										0,00	-1.0	1	21	99	119	AAUAUGGAUCGGAGGGAGUAC	UUACUCCCUCCGAUCCAUAUU	Cleavage
tae-miR1134	Traes_7DS_205267E87.1										2,05	-1.0	1	24	92	115	CAACAACAACAAGAAGAAGAAGAU	CUGUUCUUCUUCUUGUUGUUGUCU	Cleavage
tae-miR1130b-3p	Traes_7DS_25B02BA46.5										1,05	-1.0	1	21	730	750	UCUUAUAUUAUGGGACGGAGG	CCUCCGUCCCAUAAUGUAAGA	Cleavage
tae-miR1133	Traes_7DS_25F082B1E.8										3,00	-1.0	1	22	19	41	CAUAUACUCCCUCCGUCC-GAAA	UUUCCGGACGGAGGGAGUAUAUA	Cleavage
tae-miR9662a-3p	Traes_7DS_2B1FE04A6.1	PF02536				GO:GO:0006355,GO:GO:0005739,GO:GO:0003690	AT5G07900.1	Mitochondrial transcription termination factor family protein	LOC_Os06g12100.1	mTERF family protein, expressed	2,05	-1.0	1	21	561	581	UUGAACAUCCCAGAGCCACCG	CCGUGGCUCUGCGAUGUUCAG	Translation
tae-miR9662b-3p	Traes_7DS_2B1FE04A6.1	PF02536				GO:GO:0006355,GO:GO:0005739,GO:GO:0003690	AT5G07900.1	Mitochondrial transcription termination factor family protein	LOC_Os06g12100.1	mTERF family protein, expressed	2,05	-1.0	1	21	560	580	UGAACAUCCCAGAGCCACCGG	CCCGUGGCUCUGCGAUGUUCA	Cleavage
tae-miR1130b-3p	Traes_7DS_3C83C8EF9.1										1,00	-1.0	1	21	37	57	UCUUAUAUUAUGGGACGGAGG	CCUCCGUUCCAUAAUAUAAGA	Cleavage
tae-miR1122c-3p	Traes_7DS_3C83C8EF9.1										2,05	-1.0	1	21	37	57	UCUAAUAUUAUGGGACGGAGG	CCUCCGUUCCAUAAUAUAAGA	Cleavage
tae-miR1120c-5p	Traes_7DS_3C83C8EF9.1										1,05	-1.0	1	21	80	100	UAAUAUAAGAACGUUUUUGAC	GUCAAAAACGCUCUUAUAUUA	Translation
tae-miR1130b-3p	Traes_7DS_3C83C8EF9.3										1,00	-1.0	1	21	37	57	UCUUAUAUUAUGGGACGGAGG	CCUCCGUUCCAUAAUAUAAGA	Cleavage
tae-miR1122c-3p	Traes_7DS_3C83C8EF9.3										2,05	-1.0	1	21	37	57	UCUAAUAUUAUGGGACGGAGG	CCUCCGUUCCAUAAUAUAAGA	Cleavage
tae-miR1120c-5p	Traes_7DS_3C83C8EF9.3										1,05	-1.0	1	21	80	100	UAAUAUAAGAACGUUUUUGAC	GUCAAAAACGCUCUUAUAUUA	Translation
tae-miR9662a-3p	Traes_7DS_5B1210681.1	PF02536			K15032	GO:GO:0006355,GO:GO:0005739,GO:GO:0003690	AT1G21150.1	Mitochondrial transcription termination factor family protein	LOC_Os06g12100.1	mTERF family protein, expressed	2,05	-1.0	1	21	285	305	UUGAACAUCCCAGAGCCACCG	CCGUGGCUCUGCGAUGUUCAG	Translation
tae-miR9662b-3p	Traes_7DS_5B1210681.1	PF02536			K15032	GO:GO:0006355,GO:GO:0005739,GO:GO:0003690	AT1G21150.1	Mitochondrial transcription termination factor family protein	LOC_Os06g12100.1	mTERF family protein, expressed	2,05	-1.0	1	21	284	304	UGAACAUCCCAGAGCCACCGG	CCCGUGGCUCUGCGAUGUUCA	Cleavage
tae-miR9778	Traes_7DS_74534FCD3.1										2,05	-1.0	1	21	111	131	UGCAUCAUCUCGAACUCGUCG	AGACGAGCUGGAGAUGAUGCA	Cleavage
tae-miR9778	Traes_7DS_74534FCD3.2										2,05	-1.0	1	21	111	131	UGCAUCAUCUCGAACUCGUCG	AGACGAGCUGGAGAUGAUGCA	Cleavage
tae-miR9778	Traes_7DS_74534FCD3.3										2,05	-1.0	1	21	111	131	UGCAUCAUCUCGAACUCGUCG	AGACGAGCUGGAGAUGAUGCA	Cleavage
tae-miR9778	Traes_7DS_74534FCD3.4										2,05	-1.0	1	21	111	131	UGCAUCAUCUCGAACUCGUCG	AGACGAGCUGGAGAUGAUGCA	Cleavage
tae-miR9778	Traes_7DS_74534FCD3.5										2,05	-1.0	1	21	111	131	UGCAUCAUCUCGAACUCGUCG	AGACGAGCUGGAGAUGAUGCA	Cleavage
tae-miR9778	Traes_7DS_74534FCD3.6										2,05	-1.0	1	21	111	131	UGCAUCAUCUCGAACUCGUCG	AGACGAGCUGGAGAUGAUGCA	Cleavage
tae-miR1133	Traes_7DS_98F9E4E55.2										2,00	-1.0	1	22	2	24	CAUAUACUCCCUCCGUCCGA-AA	UUAUGGGACGGAGGGAGUAUAUA	Cleavage
tae-miR5050	Traes_7DS_A9CCF5A83.5										2,00	-1.0	1	21	297	317	UUGAACGACCUCACCAUGUCG	UGCCAUGAUGAGGUCGUUCAA	Cleavage
tae-miR1122a	Traes_7DS_CA61ECF35.1										0,00	-1.0	1	20	33	52	UAGAUACAUCCGUAUCUAGA	UCUAGAUACGGAUGUAUCUA	Cleavage
tae-miR9775	Traes_7DS_D4DFDF981.1										2,00	-1.0	1	22	1609	1630	UGUGCGCAAUAAGAUUUUGCUA	GUGCAUGAUCUUAUUGCGCACA	Cleavage
tae-miR9775	Traes_7DS_D4DFDF981.2										2,00	-1.0	1	22	1702	1723	UGUGCGCAAUAAGAUUUUGCUA	GUGCAUGAUCUUAUUGCGCACA	Cleavage
tae-miR156	Traes_7DS_DBB29C7CD.1									LOC_Os08g41940.1	OsSPL16 - SBP-box gene family member, expressed	1,00	-1.0	1	21	359	379	UGACAGAAGAGAGUGAGCACA	UGUGCUCUCUCUCUUCUGUCA	Cleavage
tae-miR5049-3p	Traes_7DS_EFB144315.5										1,05	-1.0	1	21	2298	2318	AAUAUGGAUCGGAGGGAGUAC	UUACUCCCUCUGAUCCAUAUU	Translation

*Triticum monococcum*_Stress_Root

miRNA_Acc.	Target_Acc.	Pfam	KOG	KEGG/ec	KO	GO	Best-hit-arabi-name	arabi-defline	Best-hit-rice-name	rice-defline	Expectation	UPE$	miRNA_start	miRNA_end	Target_start	Target_end	miRNA_aligned_fragment	Target_aligned_fragment	Inhibition
tae-miR159a	Traes_1AL_041EBB4A6.1						AT2G32460.1	myb domain protein 101	LOC_Os01g59660.2	MYB family transcription factor, putative, expressed	2,05	-1.0	1	21	488	508	UUUGGAUUGAAGGGAGCUCUG	UGGAGCUCCCUUCAUUCCAAU	Cleavage
tae-miR159b	Traes_1AL_041EBB4A6.1						AT2G32460.1	myb domain protein 101	LOC_Os01g59660.2	MYB family transcription factor, putative, expressed	2,05	-1.0	1	21	488	508	UUUGGAUUGAAGGGAGCUCUG	UGGAGCUCCCUUCAUUCCAAU	Cleavage
tae-miR159a	Traes_1AL_041EBB4A6.2						AT2G32460.1	myb domain protein 101	LOC_Os01g59660.2	MYB family transcription factor, putative, expressed	2,05	-1.0	1	21	512	532	UUUGGAUUGAAGGGAGCUCUG	UGGAGCUCCCUUCAUUCCAAU	Cleavage
tae-miR159b	Traes_1AL_041EBB4A6.2						AT2G32460.1	myb domain protein 101	LOC_Os01g59660.2	MYB family transcription factor, putative, expressed	2,05	-1.0	1	21	512	532	UUUGGAUUGAAGGGAGCUCUG	UGGAGCUCCCUUCAUUCCAAU	Cleavage
tae-miR159a	Traes_1AL_041EBB4A6.3						AT2G32460.1	myb domain protein 101	LOC_Os01g59660.2	MYB family transcription factor, putative, expressed	2,05	-1.0	1	21	488	508	UUUGGAUUGAAGGGAGCUCUG	UGGAGCUCCCUUCAUUCCAAU	Cleavage
tae-miR159b	Traes_1AL_041EBB4A6.3						AT2G32460.1	myb domain protein 101	LOC_Os01g59660.2	MYB family transcription factor, putative, expressed	2,05	-1.0	1	21	488	508	UUUGGAUUGAAGGGAGCUCUG	UGGAGCUCCCUUCAUUCCAAU	Cleavage
tae-miR159a	Traes_1AL_041EBB4A6.4								LOC_Os01g59660.2	MYB family transcription factor, putative, expressed	2,05	-1.0	1	21	410	430	UUUGGAUUGAAGGGAGCUCUG	UGGAGCUCCCUUCAUUCCAAU	Cleavage
tae-miR159b	Traes_1AL_041EBB4A6.4								LOC_Os01g59660.2	MYB family transcription factor, putative, expressed	2,05	-1.0	1	21	410	430	UUUGGAUUGAAGGGAGCUCUG	UGGAGCUCCCUUCAUUCCAAU	Cleavage
tae-miR160	Traes_1AL_147CF243C.1										0,00	-1.0	1	21	642	662	UGCCUGGCUCCCUGUAUGCCA	AGGCAUACAGGGAGCCAGGCA	Cleavage
tae-miR160	Traes_1AL_147CF243C.2										0,00	-1.0	1	21	267	287	UGCCUGGCUCCCUGUAUGCCA	AGGCAUACAGGGAGCCAGGCA	Cleavage
tae-miR1127b-3p	Traes_1AL_4A458DF14.2										1,05	-1.0	1	21	1738	1758	ACAAGUAUUUCUGGACGGAGG	CCUCCGUCCGGAAAUACUUGU	Cleavage
tae-miR1130b-3p	Traes_1AL_51B1D5DBC.6										0,00	-1.0	1	21	914	934	UCUUAUAUUAUGGGACGGAGG	CCUCCGUCCCAUAAUAUAAGA	Cleavage
tae-miR1122c-3p	Traes_1AL_51B1D5DBC.6										1,05	-1.0	1	21	914	934	UCUAAUAUUAUGGGACGGAGG	CCUCCGUCCCAUAAUAUAAGA	Cleavage
tae-miR5049-3p	Traes_1AL_7D6DC73FC.3										2,00	-1.0	1	21	1256	1276	AAUAUGGAUCGGAGGGAGUAC	UUUCUCCCUCCGAUCCAUAUA	Cleavage
tae-miR1128	Traes_1AL_968B97E50.2										3,00	-1.0	1	21	100	121	UACUACUCCCUCCGUCC-GAAA	UUUCCGGACGGAGGGAGUAGUU	Cleavage
tae-miR1134	Traes_1AL_AF222553D.2										2,05	-1.0	1	24	568	591	CAACAACAACAAGAAGAAGAAGAU	CUCUCCUUGUUGUUGUUGUUGUUG	Cleavage
tae-miR7757-5p	Traes_1AL_B6931117F.1	PF00931				GO:GO:0043531	AT3G46530.1	NB-ARC domain-containing disease resistance protein	LOC_Os08g10440.1	NBS-LRR disease resistance protein, putative, expressed	1,05	-1.0	1	22	1301	1322	AUAAAACCUUCAGCUAUCCAUC	AAUGGAUAGCUGAAGGCUUUAU	Cleavage
tae-miR7757-5p	Traes_1AL_B6931117F.2	PF00931				GO:GO:0043531	AT3G46530.1	NB-ARC domain-containing disease resistance protein	LOC_Os08g10440.1	NBS-LRR disease resistance protein, putative, expressed	1,05	-1.0	1	22	1343	1364	AUAAAACCUUCAGCUAUCCAUC	AAUGGAUAGCUGAAGGCUUUAU	Cleavage
tae-miR7757-5p	Traes_1AL_B6931117F.3	PF00931				GO:GO:0043531	AT3G46530.1	NB-ARC domain-containing disease resistance protein	LOC_Os08g10440.1	NBS-LRR disease resistance protein, putative, expressed	1,05	-1.0	1	22	1301	1322	AUAAAACCUUCAGCUAUCCAUC	AAUGGAUAGCUGAAGGCUUUAU	Cleavage
tae-miR7757-5p	Traes_1AL_B6931117F.4	PF00931				GO:GO:0043531	AT3G46530.1	NB-ARC domain-containing disease resistance protein	LOC_Os08g10440.1	NBS-LRR disease resistance protein, putative, expressed	1,05	-1.0	1	22	1301	1322	AUAAAACCUUCAGCUAUCCAUC	AAUGGAUAGCUGAAGGCUUUAU	Cleavage
tae-miR9778	Traes_1AS_17306388F.1	PF00931				GO:GO:0043531	AT3G07040.1	NB-ARC domain-containing disease resistance protein	LOC_Os08g42670.2	resistance protein, putative, expressed	2,00	-1.0	1	21	126	146	UGCAUCAUCUCGAACUCGUCG	CAAUGAGCUCGAGAUGAUGCA	Cleavage
tae-miR9778	Traes_1AS_17306388F.3	PF00931				GO:GO:0043531	AT3G46530.1	NB-ARC domain-containing disease resistance protein	LOC_Os12g37290.1	resistance protein T10rga2-1A, putative, expressed	2,00	-1.0	1	21	126	146	UGCAUCAUCUCGAACUCGUCG	CAAUGAGCUCGAGAUGAUGCA	Cleavage
tae-miR1135	Traes_1AS_4A925522B.4	PF13920					AT5G01960.1	RING/U-box superfamily protein	LOC_Os01g07390.1	zinc finger, C3HC4 type family protein, expressed	0,00	-1.0	1	24	511	534	CUGCGACAAGUAAUUCCGAACGGA	UCCGUUCGGAAUUACUUGUCGCAG	Cleavage
tae-miR6197-5p	Traes_1AS_5DCFDC258.1										3,00	-1.0	1	21	1353	1376	UCUGUAAACAAAUGUA---GGACG	ACUCCAACUACAUUUGUUUACAGA	Cleavage
tae-miR1137a	Traes_1AS_8912BD052.27										0,00	-1.0	1	20	136	155	UAGUACAAAGUUGAGUCAUC	GAUGACUCAACUUUGUACUA	Cleavage
tae-miR9674a-5p	Traes_1AS_92A698B19.1										2,05	-1.0	1	21	1761	1781	GCAUCAUCCAUCCUACCAUUC	AAAUGGAAGGAUCGAUGAUGC	Cleavage
tae-miR9674a-5p	Traes_1AS_92A698B19.10										2,05	-1.0	1	21	1761	1781	GCAUCAUCCAUCCUACCAUUC	AAAUGGAAGGAUCGAUGAUGC	Cleavage
tae-miR9674a-5p	Traes_1AS_92A698B19.12										2,05	-1.0	1	21	96	116	GCAUCAUCCAUCCUACCAUUC	AAAUGGAAGGAUCGAUGAUGC	Cleavage
tae-miR9674a-5p	Traes_1AS_92A698B19.2										2,05	-1.0	1	21	1761	1781	GCAUCAUCCAUCCUACCAUUC	AAAUGGAAGGAUCGAUGAUGC	Cleavage
tae-miR9674a-5p	Traes_1AS_92A698B19.3										2,05	-1.0	1	21	1761	1781	GCAUCAUCCAUCCUACCAUUC	AAAUGGAAGGAUCGAUGAUGC	Cleavage
tae-miR9674a-5p	Traes_1AS_92A698B19.4										2,05	-1.0	1	21	1761	1781	GCAUCAUCCAUCCUACCAUUC	AAAUGGAAGGAUCGAUGAUGC	Cleavage
tae-miR9674a-5p	Traes_1AS_92A698B19.5										2,05	-1.0	1	21	1761	1781	GCAUCAUCCAUCCUACCAUUC	AAAUGGAAGGAUCGAUGAUGC	Cleavage
tae-miR9674a-5p	Traes_1AS_92A698B19.6										2,05	-1.0	1	21	1761	1781	GCAUCAUCCAUCCUACCAUUC	AAAUGGAAGGAUCGAUGAUGC	Cleavage
tae-miR9674a-5p	Traes_1AS_92A698B19.7										2,05	-1.0	1	21	1761	1781	GCAUCAUCCAUCCUACCAUUC	AAAUGGAAGGAUCGAUGAUGC	Cleavage
tae-miR9674a-5p	Traes_1AS_92A698B19.8										2,05	-1.0	1	21	1761	1781	GCAUCAUCCAUCCUACCAUUC	AAAUGGAAGGAUCGAUGAUGC	Cleavage
tae-miR9674a-5p	Traes_1AS_92A698B19.9										2,05	-1.0	1	21	1761	1781	GCAUCAUCCAUCCUACCAUUC	AAAUGGAAGGAUCGAUGAUGC	Cleavage
tae-miR5049-3p	Traes_1AS_A482D4FF9.1										0,00	-1.0	1	21	557	577	AAUAUGGAUCGGAGGGAGUAC	GUACUCCCUCCGAUCCAUAUU	Cleavage
tae-miR9778	Traes_1AS_C7A8188D1.1	PF13191,PF00931	KOG4658			GO:GO:0043531	AT1G58390.1	Disease resistance protein (CC-NBS-LRR class) family	LOC_Os08g42670.2	resistance protein, putative, expressed	1,00	-1.0	1	21	126	146	UGCAUCAUCUCGAACUCGUCG	CAACGAGCUCGAGAUGAUGCA	Cleavage
tae-miR9778	Traes_1AS_C7A8188D1.3	PF00931				GO:GO:0043531	AT3G46530.1	NB-ARC domain-containing disease resistance protein	LOC_Os02g57310.1	pib, putative, expressed	1,00	-1.0	1	21	126	146	UGCAUCAUCUCGAACUCGUCG	CAACGAGCUCGAGAUGAUGCA	Cleavage
tae-miR9661-5p	Traes_1AS_D8E37233E.1										2,00	-1.0	1	21	101	121	UGAAGUAGAGCAGGGACCUCA	CCAAGUCCCUGCUCUACUUCG	Cleavage
tae-miR1128	Traes_1BL_087CA0963.1										3,00	-1.0	1	21	2048	2069	UACUACUCCCUCCG-UCCGAAA	UUUAGGAACGGAGGGAGUAGUA	Cleavage
tae-miR1128	Traes_1BL_0FDC38712.1										1,05	-1.0	1	21	225	245	UACUACUCCCUCCGUCCGAAA	UUUCGGACGGAGGGAGUACUA	Cleavage
tae-miR1127b-3p	Traes_1BL_0FDC38712.1										1,05	-1.0	1	21	121	141	ACAAGUAUUUCUGGACGGAGG	CCUCCGUCCGGAAAUACUUGU	Cleavage
tae-miR160	Traes_1BL_54CD82AC3.1										0,00	-1.0	1	21	642	662	UGCCUGGCUCCCUGUAUGCCA	AGGCAUACAGGGAGCCAGGCA	Cleavage
tae-miR160	Traes_1BL_54CD82AC3.2										0,00	-1.0	1	21	369	389	UGCCUGGCUCCCUGUAUGCCA	AGGCAUACAGGGAGCCAGGCA	Cleavage
tae-miR160	Traes_1BL_54CD82AC3.3										0,00	-1.0	1	21	267	287	UGCCUGGCUCCCUGUAUGCCA	AGGCAUACAGGGAGCCAGGCA	Cleavage
tae-miR1130b-3p	Traes_1BL_89E785AAA.2										2,00	-1.0	1	21	591	611	UCUUAUAUUAUGGGACGGAGG	CCUCUGUCCCAUAAUAUAAGG	Cleavage
tae-miR159a	Traes_1BL_CF98E922B.1	PF00249					AT5G06100.2	myb domain protein 33	LOC_Os01g59660.2	MYB family transcription factor, putative, expressed	2,05	-1.0	1	21	839	859	UUUGGAUUGAAGGGAGCUCUG	UGGAGCUCCCUUCACUCCAAU	Cleavage
tae-miR159b	Traes_1BL_CF98E922B.1	PF00249					AT5G06100.2	myb domain protein 33	LOC_Os01g59660.2	MYB family transcription factor, putative, expressed	2,05	-1.0	1	21	839	859	UUUGGAUUGAAGGGAGCUCUG	UGGAGCUCCCUUCACUCCAAU	Cleavage
tae-miR159a	Traes_1BL_CF98E922B.2	PF00249					AT5G06100.3	myb domain protein 33	LOC_Os01g59660.1	MYB family transcription factor, putative, expressed	2,05	-1.0	1	21	839	859	UUUGGAUUGAAGGGAGCUCUG	UGGAGCUCCCUUCACUCCAAU	Cleavage
tae-miR159b	Traes_1BL_CF98E922B.2	PF00249					AT5G06100.3	myb domain protein 33	LOC_Os01g59660.1	MYB family transcription factor, putative, expressed	2,05	-1.0	1	21	839	859	UUUGGAUUGAAGGGAGCUCUG	UGGAGCUCCCUUCACUCCAAU	Cleavage
tae-miR159a	Traes_1BL_CF98E922B.3						AT5G06100.3	myb domain protein 33	LOC_Os01g59660.2	MYB family transcription factor, putative, expressed	2,05	-1.0	1	21	512	532	UUUGGAUUGAAGGGAGCUCUG	UGGAGCUCCCUUCACUCCAAU	Cleavage
tae-miR159b	Traes_1BL_CF98E922B.3						AT5G06100.3	myb domain protein 33	LOC_Os01g59660.2	MYB family transcription factor, putative, expressed	2,05	-1.0	1	21	512	532	UUUGGAUUGAAGGGAGCUCUG	UGGAGCUCCCUUCACUCCAAU	Cleavage
tae-miR159a	Traes_1BL_CF98E922B.4						AT5G06100.1	myb domain protein 33	LOC_Os01g59660.1	MYB family transcription factor, putative, expressed	2,05	-1.0	1	21	512	532	UUUGGAUUGAAGGGAGCUCUG	UGGAGCUCCCUUCACUCCAAU	Cleavage
tae-miR159b	Traes_1BL_CF98E922B.4						AT5G06100.1	myb domain protein 33	LOC_Os01g59660.1	MYB family transcription factor, putative, expressed	2,05	-1.0	1	21	512	532	UUUGGAUUGAAGGGAGCUCUG	UGGAGCUCCCUUCACUCCAAU	Cleavage
tae-miR1117	Traes_1BL_F01A87F44.1										2,05	-1.0	1	24	37	60	UAGUACCGGUUCGUGGCACGAACC	GGUUCGUGGCACGAACCGGUGCUA	Cleavage
tae-miR1131	Traes_1BL_F01A87F44.1										2,05	-1.0	1	22	63	84	UAGUACCGGUUCGUGGCUAACC	GGUUAGCCACGAACCGGUAAUG	Cleavage
tae-miR1122b-3p	Traes_1BS_34C60A54E.1										1,05	-1.0	1	21	47	67	AGACUUAUAUGUAGGAACGGA	UCCGUUCCUAAAUAUAAGUCU	Translation
tae-miR9662a-3p	Traes_1BS_566CEE7F6.1	PF02536				GO:GO:0006355,GO:GO:0005739,GO:GO:0003690	AT1G21150.1	Mitochondrial transcription termination factor family protein	LOC_Os06g12100.1	mTERF family protein, expressed	2,00	-1.0	1	21	441	461	UUGAACAUCCCAGAGCCACCG	UCGUGGCGCUGGGAUGUUCAG	Cleavage
tae-miR9662b-3p	Traes_1BS_566CEE7F6.1	PF02536				GO:GO:0006355,GO:GO:0005739,GO:GO:0003690	AT1G21150.1	Mitochondrial transcription termination factor family protein	LOC_Os06g12100.1	mTERF family protein, expressed	2,05	-1.0	1	21	440	460	UGAACAUCCCAGAGCCACCGG	CUCGUGGCGCUGGGAUGUUCA	Cleavage
tae-miR9662a-3p	Traes_1BS_566CEE7F6.2	PF02536				GO:GO:0006355,GO:GO:0005739,GO:GO:0003690	AT1G21150.1	Mitochondrial transcription termination factor family protein	LOC_Os06g12100.1	mTERF family protein, expressed	2,00	-1.0	1	21	66	86	UUGAACAUCCCAGAGCCACCG	UCGUGGCGCUGGGAUGUUCAG	Cleavage
tae-miR9662b-3p	Traes_1BS_566CEE7F6.2	PF02536				GO:GO:0006355,GO:GO:0005739,GO:GO:0003690	AT1G21150.1	Mitochondrial transcription termination factor family protein	LOC_Os06g12100.1	mTERF family protein, expressed	2,05	-1.0	1	21	65	85	UGAACAUCCCAGAGCCACCGG	CUCGUGGCGCUGGGAUGUUCA	Cleavage
tae-miR9778	Traes_1BS_70B733D34.1	PF13191,PF00931				GO:GO:0043531	AT3G07040.1	NB-ARC domain-containing disease resistance protein	LOC_Os08g42670.1	resistance protein, putative, expressed	2,00	-1.0	1	21	126	146	UGCAUCAUCUCGAACUCGUCG	CAAUGAGCUCGAGAUGAUGCA	Cleavage
tae-miR9778	Traes_1BS_70B733D34.2	PF13191,PF00931				GO:GO:0043531	AT3G07040.1	NB-ARC domain-containing disease resistance protein	LOC_Os08g42670.2	resistance protein, putative, expressed	2,00	-1.0	1	21	126	146	UGCAUCAUCUCGAACUCGUCG	CAAUGAGCUCGAGAUGAUGCA	Cleavage
tae-miR5049-3p	Traes_1BS_7EC2BDE95.1										1,05	-1.0	1	21	3430	3450	AAUAUGGAUCGGAGGGAGUAC	CUACUCCCUCCAAUCCAUAUU	Translation
tae-miR5049-3p	Traes_1BS_B0BB95B97.6										0,00	-1.0	1	21	623	643	AAUAUGGAUCGGAGGGAGUAC	GUACUCCCUCCGAUCCAUAUU	Cleavage
tae-miR7757-5p	Traes_1BS_CF1C4F54E.2	PF00931				GO:GO:0043531	AT1G09920.1	TRAF-type zinc finger-related	LOC_Os11g45750.1	WRKY125, expressed	2,05	-1.0	1	22	119	140	AUAAAACCUUCAGCUAUCCAUC	AAUGGUUAGCUGAAGGUUUCAU	Cleavage
tae-miR159a	Traes_1DL_25540DE53.1						AT2G32460.1	myb domain protein 101	LOC_Os01g59660.2	MYB family transcription factor, putative, expressed	2,05	-1.0	1	21	512	532	UUUGGAUUGAAGGGAGCUCUG	UGGAGCUCCCUUCAUUCCAAU	Cleavage
tae-miR159b	Traes_1DL_25540DE53.1						AT2G32460.1	myb domain protein 101	LOC_Os01g59660.2	MYB family transcription factor, putative, expressed	2,05	-1.0	1	21	512	532	UUUGGAUUGAAGGGAGCUCUG	UGGAGCUCCCUUCAUUCCAAU	Cleavage
tae-miR159a	Traes_1DL_25540DE53.2						AT2G32460.1	myb domain protein 101	LOC_Os01g59660.1	MYB family transcription factor, putative, expressed	2,05	-1.0	1	21	512	532	UUUGGAUUGAAGGGAGCUCUG	UGGAGCUCCCUUCAUUCCAAU	Cleavage
tae-miR159b	Traes_1DL_25540DE53.2						AT2G32460.1	myb domain protein 101	LOC_Os01g59660.1	MYB family transcription factor, putative, expressed	2,05	-1.0	1	21	512	532	UUUGGAUUGAAGGGAGCUCUG	UGGAGCUCCCUUCAUUCCAAU	Cleavage
tae-miR159a	Traes_1DL_25540DE53.3						AT5G06100.3	myb domain protein 33	LOC_Os01g59660.1	MYB family transcription factor, putative, expressed	2,05	-1.0	1	21	470	490	UUUGGAUUGAAGGGAGCUCUG	UGGAGCUCCCUUCAUUCCAAU	Cleavage
tae-miR159b	Traes_1DL_25540DE53.3						AT5G06100.3	myb domain protein 33	LOC_Os01g59660.1	MYB family transcription factor, putative, expressed	2,05	-1.0	1	21	470	490	UUUGGAUUGAAGGGAGCUCUG	UGGAGCUCCCUUCAUUCCAAU	Cleavage
tae-miR159a	Traes_1DL_25540DE53.4						AT5G06100.3	myb domain protein 33	LOC_Os01g59660.2	MYB family transcription factor, putative, expressed	2,05	-1.0	1	21	470	490	UUUGGAUUGAAGGGAGCUCUG	UGGAGCUCCCUUCAUUCCAAU	Cleavage
tae-miR159b	Traes_1DL_25540DE53.4						AT5G06100.3	myb domain protein 33	LOC_Os01g59660.2	MYB family transcription factor, putative, expressed	2,05	-1.0	1	21	470	490	UUUGGAUUGAAGGGAGCUCUG	UGGAGCUCCCUUCAUUCCAAU	Cleavage
tae-miR159a	Traes_1DL_25540DE53.5						AT5G06100.2	myb domain protein 33	LOC_Os01g59660.2	MYB family transcription factor, putative, expressed	2,05	-1.0	1	21	470	490	UUUGGAUUGAAGGGAGCUCUG	UGGAGCUCCCUUCAUUCCAAU	Cleavage
tae-miR159b	Traes_1DL_25540DE53.5						AT5G06100.2	myb domain protein 33	LOC_Os01g59660.2	MYB family transcription factor, putative, expressed	2,05	-1.0	1	21	470	490	UUUGGAUUGAAGGGAGCUCUG	UGGAGCUCCCUUCAUUCCAAU	Cleavage
tae-miR159a	Traes_1DL_25540DE53.6						AT5G06100.2	myb domain protein 33	LOC_Os01g59660.1	MYB family transcription factor, putative, expressed	2,05	-1.0	1	21	470	490	UUUGGAUUGAAGGGAGCUCUG	UGGAGCUCCCUUCAUUCCAAU	Cleavage
tae-miR159b	Traes_1DL_25540DE53.6						AT5G06100.2	myb domain protein 33	LOC_Os01g59660.1	MYB family transcription factor, putative, expressed	2,05	-1.0	1	21	470	490	UUUGGAUUGAAGGGAGCUCUG	UGGAGCUCCCUUCAUUCCAAU	Cleavage
tae-miR1137a	Traes_1DL_34A359364.2										2,05	-1.0	1	20	516	535	UAGUACAAAGUUGAGUCAUC	GAUGACCCAAUUUUGUACUA	Translation
tae-miR1122a	Traes_1DL_38B88249C.2										0,00	-1.0	1	20	976	995	UAGAUACAUCCGUAUCUAGA	UCUAGAUACGGAUGUAUCUA	Cleavage
tae-miR1120a	Traes_1DL_6A91BC5A5.1										2,05	-1.0	1	24	25	48	ACAUUCUUAUAUUAUGAGACGGAG	CUCCGUCCCAUAAUAUAAGAACGU	Cleavage
tae-miR1120b-3p	Traes_1DL_6A91BC5A5.1										2,05	-1.0	1	21	25	45	UUCUUAUAUUGUGGGACAGAG	CUCCGUCCCAUAAUAUAAGAA	Translation
tae-miR1130b-3p	Traes_1DL_6A91BC5A5.1										0,00	-1.0	1	21	24	44	UCUUAUAUUAUGGGACGGAGG	CCUCCGUCCCAUAAUAUAAGA	Cleavage
tae-miR1122c-3p	Traes_1DL_6A91BC5A5.1										1,05	-1.0	1	21	24	44	UCUAAUAUUAUGGGACGGAGG	CCUCCGUCCCAUAAUAUAAGA	Cleavage
tae-miR1120c-5p	Traes_1DL_6A91BC5A5.1										2,05	-1.0	1	21	67	87	UAAUAUAAGAACGUUUUUGAC	GUCAAACACGCUCUUAUAUUA	Translation
tae-miR160	Traes_1DL_AEC2F3FB3.1										0,00	-1.0	1	21	642	662	UGCCUGGCUCCCUGUAUGCCA	AGGCAUACAGGGAGCCAGGCA	Cleavage
tae-miR1122b-3p	Traes_1DL_B5E6E273B.1										1,05	-1.0	1	21	35	55	AGACUUAUAUGUAGGAACGGA	UCCGUUCCUAAAUAUAAGUCU	Translation
tae-miR5175-5p	Traes_1DL_C1BE1742F.2										2,00	-1.0	1	21	109	130	UUCCAAAUUACUCGUCG-UGGU	UUCAGCGACGAGUAAUUUGGAA	Cleavage
tae-miR1133	Traes_1DL_D391AE3DC.1										2,00	-1.0	1	22	858	879	CAUAUACUCCCUCCGUCCGAAA	AAAUGAACGGAGGGAGUAUAUG	Cleavage
tae-miR1133	Traes_1DL_D391AE3DC.2										2,00	-1.0	1	22	858	879	CAUAUACUCCCUCCGUCCGAAA	AAAUGAACGGAGGGAGUAUAUG	Cleavage
tae-miR1133	Traes_1DL_D391AE3DC.3										2,00	-1.0	1	22	822	843	CAUAUACUCCCUCCGUCCGAAA	AAAUGAACGGAGGGAGUAUAUG	Cleavage
tae-miR1128	Traes_1DL_E30609261.7										3,00	-1.0	1	21	76	96	UACUACUCCCUCCGUCCGAAA	UUCCAGACGGAGGGAGUAGUU	Cleavage
tae-miR1122a	Traes_1DS_06B8348E0.1										0,00	-1.0	1	20	794	813	UAGAUACAUCCGUAUCUAGA	UCUAGAUACGGAUGUAUCUA	Cleavage
tae-miR1122a	Traes_1DS_06B8348E0.2										0,00	-1.0	1	20	620	639	UAGAUACAUCCGUAUCUAGA	UCUAGAUACGGAUGUAUCUA	Cleavage
tae-miR5048-5p	Traes_1DS_26F1546F6.1	PF00168,PF07714,PF00069	KOG1187	2.7.11.1		GO:GO:0005515,GO:GO:0006468,GO:GO:0004672,GO:GO:0005524	AT1G63430.1	Leucine-rich repeat protein kinase family protein	LOC_Os04g58700.1	expressed protein	2,05	-1.0	1	22	917	938	UUUGCAGGUUUUAGGUCUAAGU	ACUUGGACCUAAAACCUGCCAA	Cleavage
tae-miR5048-5p	Traes_1DS_26F1546F6.2	PF00168,PF07714,PF00069	KOG1187	2.7.11.1		GO:GO:0005515,GO:GO:0006468,GO:GO:0004672,GO:GO:0005524	AT1G63430.1	Leucine-rich repeat protein kinase family protein	LOC_Os04g58700.1	expressed protein	2,05	-1.0	1	22	917	938	UUUGCAGGUUUUAGGUCUAAGU	ACUUGGACCUAAAACCUGCCAA	Cleavage
tae-miR7757-5p	Traes_1DS_35EF41CCB.2	PF00931				GO:GO:0043531	AT3G50950.1	HOPZ-ACTIVATED RESISTANCE 1	LOC_Os05g40150.1	RGH2B, putative, expressed	1,05	-1.0	1	22	587	608	AUAAAACCUUCAGCUAUCCAUC	AGUGGAUAGCUGAAGGUUUCAU	Cleavage
tae-miR9674a-5p	Traes_1DS_F3DE3946F.1										2,05	-1.0	1	21	522	542	GCAUCAUCCAUCCUACCAUUC	AAAUGGAAGGAUCGAUGAUGC	Cleavage
tae-miR5049-3p	Traes_1DS_FA7FC5C3C.1										0,00	-1.0	1	21	623	643	AAUAUGGAUCGGAGGGAGUAC	GUACUCCCUCCGAUCCAUAUU	Cleavage
tae-miR1128	Traes_2AL_24A1BF00C.1										0,00	-1.0	1	21	37	57	UACUACUCCCUCCGUCCGAAA	UUUCGGACGGAGGGAGUAGUA	Cleavage
tae-miR5049-3p	Traes_2AL_28B13CC56.1										1,05	-1.0	1	21	346	366	AAUAUGGAUCGGAGGGAGUAC	CUACUCCCUCCGUUCCAUAUU	Cleavage
tae-miR1127a	Traes_2AL_4197D219B.1										2,05	-1.0	1	19	991	1009	UCCUUCCGUUCGGAAUUAC	GUAAUUCCGAACGGAGGGU	Cleavage
tae-miR164	Traes_2AL_57BF37A86.1										1,05	-1.0	1	21	680	700	UGGAGAAGCAGGGCACGUGCA	CGCACGUGACCUGCUUCUCCA	Cleavage
tae-miR164	Traes_2AL_57BF37A86.2										1,05	-1.0	1	21	606	626	UGGAGAAGCAGGGCACGUGCA	CGCACGUGACCUGCUUCUCCA	Cleavage
tae-miR1122a	Traes_2AL_64B73E3ED.1										1,00	-1.0	1	20	121	140	UAGAUACAUCCGUAUCUAGA	UCUAAAUACGGAUGUAUCUA	Cleavage
tae-miR1120b-3p	Traes_2AL_673CA8FB2.1										2,05	-1.0	1	21	493	513	UUCUUAUAUUGUGGGACAGAG	CUCUGUCCCACAAUGUAAGAC	Cleavage
tae-miR1133	Traes_2AL_673CA8FB2.1										2,05	-1.0	1	22	553	575	CAUAUACUCCCUCCGUCCGA-AA	UUGUGGGACGGAGGGAGUAUUUG	Cleavage
tae-miR1120a	Traes_2AL_979E96822.1										2,05	-1.0	1	24	753	776	ACAUUCUUAUAUUAUGAGACGGAG	CUCCAUCCCAUAAUAUAAGAGUGU	Cleavage
tae-miR1130b-3p	Traes_2AL_979E96822.1										1,00	-1.0	1	21	752	772	UCUUAUAUUAUGGGACGGAGG	CCUCCAUCCCAUAAUAUAAGA	Cleavage
tae-miR1122c-3p	Traes_2AL_979E96822.1										2,05	-1.0	1	21	752	772	UCUAAUAUUAUGGGACGGAGG	CCUCCAUCCCAUAAUAUAAGA	Cleavage
tae-miR1120c-5p	Traes_2AL_979E96822.1										2,05	-1.0	1	21	795	815	UAAUAUAAGAACGUUUUUGAC	GUAAAAAACGCUCUUAUAUUA	Translation
tae-miR160	Traes_2AL_C70217249.1										1,00	-1.0	1	21	1164	1184	UGCCUGGCUCCCUGUAUGCCA	UGGCAUGCAGGGAGCCAGGCA	Cleavage
tae-miR1134	Traes_2AL_D2CC35E59.1										3,00	-1.0	1	24	512	535	CAACAACAACAAGAAGAAGAAGAU	AUCUUCAUGGUCUUGUUGUUGUUG	Cleavage
tae-miR1134	Traes_2AL_D2CC35E59.2										3,00	-1.0	1	24	620	643	CAACAACAACAAGAAGAAGAAGAU	AUCUUCAUGGUCUUGUUGUUGUUG	Cleavage
tae-miR1134	Traes_2AL_D2CC35E59.3										3,00	-1.0	1	24	509	532	CAACAACAACAAGAAGAAGAAGAU	AUCUUCAUGGUCUUGUUGUUGUUG	Cleavage
tae-miR1134	Traes_2AL_D2CC35E59.4										3,00	-1.0	1	24	611	634	CAACAACAACAAGAAGAAGAAGAU	AUCUUCAUGGUCUUGUUGUUGUUG	Cleavage
tae-miR1134	Traes_2AL_D2CC35E59.5										3,00	-1.0	1	24	509	532	CAACAACAACAAGAAGAAGAAGAU	AUCUUCAUGGUCUUGUUGUUGUUG	Cleavage
tae-miR1134	Traes_2AL_D2CC35E59.6										3,00	-1.0	1	24	509	532	CAACAACAACAAGAAGAAGAAGAU	AUCUUCAUGGUCUUGUUGUUGUUG	Cleavage
tae-miR5384-3p	Traes_2AL_D79C9535C.1										2,05	-1.0	1	21	1250	1270	UGAGCGCGCCGCCGUCGAAUG	CGUUCGGCGGCGGCGCGCGCA	Cleavage
tae-miR9778	Traes_2AS_530CF4042.10	PF00931				GO:GO:0043531	AT3G50950.1	HOPZ-ACTIVATED RESISTANCE 1	LOC_Os04g12830.1	expressed protein	2,05	-1.0	1	21	126	146	UGCAUCAUCUCGAACUCGUCG	CGGCGAGUUCCAGAUGAUGCA	Translation
tae-miR9778	Traes_2AS_530CF4042.2	PF00931				GO:GO:0043531	AT3G50950.1	HOPZ-ACTIVATED RESISTANCE 1	LOC_Os04g12830.1	expressed protein	2,05	-1.0	1	21	126	146	UGCAUCAUCUCGAACUCGUCG	CGGCGAGUUCCAGAUGAUGCA	Translation
tae-miR9778	Traes_2AS_530CF4042.3	PF00931				GO:GO:0043531	AT3G50950.1	HOPZ-ACTIVATED RESISTANCE 1	LOC_Os04g12830.1	expressed protein	2,05	-1.0	1	21	126	146	UGCAUCAUCUCGAACUCGUCG	CGGCGAGUUCCAGAUGAUGCA	Translation
tae-miR1130b-3p	Traes_2AS_6DCA4AADD.1										1,05	-1.0	1	21	288	308	UCUUAUAUUAUGGGACGGAGG	CCUCCGUCCCAUAAUAUAGGA	Cleavage
tae-miR9664-3p	Traes_2AS_86633353C.1	PF00931	KOG4658		K13457	GO:GO:0043531	AT3G07040.1	NB-ARC domain-containing disease resistance protein	LOC_Os11g46210.1	NB-ARC domain containing protein, expressed	2,05	-1.0	1	21	240	260	UUGCAGUCCUCGAUGUCGUAG	CUACGACAUCGAGGAUUGCAU	Cleavage
tae-miR1130b-3p	Traes_2BL_171777733.1										0,00	-1.0	1	21	752	772	UCUUAUAUUAUGGGACGGAGG	CCUCCGUCCCAUAAUAUAAGA	Cleavage
tae-miR1122c-3p	Traes_2BL_171777733.1										1,05	-1.0	1	21	752	772	UCUAAUAUUAUGGGACGGAGG	CCUCCGUCCCAUAAUAUAAGA	Cleavage
tae-miR1120c-5p	Traes_2BL_171777733.1										2,05	-1.0	1	21	795	815	UAAUAUAAGAACGUUUUUGAC	GUAAAAAACGCUCUUAUAUUA	Translation
tae-miR5384-3p	Traes_2BL_1BE5C9C71.1										2,05	-1.0	1	21	455	475	UGAGCGCGCCGCCGUCGAAUG	CGUUCGGCGGCGGCGCGCGCA	Cleavage
tae-miR5384-3p	Traes_2BL_1BE5C9C71.2										2,05	-1.0	1	21	455	475	UGAGCGCGCCGCCGUCGAAUG	CGUUCGGCGGCGGCGCGCGCA	Cleavage
tae-miR1122a	Traes_2BL_40442CE82.2										1,05	-1.0	1	20	703	722	UAGAUACAUCCGUAUCUAGA	UCUAGAUACAGAUGUAUCUA	Translation
tae-miR164	Traes_2BL_6AEE8AC28.1										1,05	-1.0	1	21	680	700	UGGAGAAGCAGGGCACGUGCA	CGCACGUGACCUGCUUCUCCA	Cleavage
tae-miR164	Traes_2BL_6AEE8AC28.2										1,05	-1.0	1	21	672	692	UGGAGAAGCAGGGCACGUGCA	CGCACGUGACCUGCUUCUCCA	Cleavage
tae-miR164	Traes_2BL_6AEE8AC28.3										1,05	-1.0	1	21	633	653	UGGAGAAGCAGGGCACGUGCA	CGCACGUGACCUGCUUCUCCA	Cleavage
tae-miR1122b-3p	Traes_2BL_7141904F2.2										2,05	-1.0	1	21	1058	1078	AGACUUAUAUGUAGGAACGGA	UCCGUUCUUAAAUAUAAGUCU	Translation
tae-miR1122a	Traes_2BL_7141904F2.2										2,05	-1.0	1	20	1098	1117	UAGAUACAUCCGUAUCUAGA	ACUACAUACGGAUGUAUAUA	Cleavage
tae-miR1134	Traes_2BL_8339D35AC.1										3,00	-1.0	1	24	509	532	CAACAACAACAAGAAGAAGAAGAU	AUCUUCAUGGUCUUGUUGUUGUUG	Cleavage
tae-miR1134	Traes_2BL_8339D35AC.2										3,00	-1.0	1	24	512	535	CAACAACAACAAGAAGAAGAAGAU	AUCUUCAUGGUCUUGUUGUUGUUG	Cleavage
tae-miR1134	Traes_2BL_8339D35AC.3										3,00	-1.0	1	24	509	532	CAACAACAACAAGAAGAAGAAGAU	AUCUUCAUGGUCUUGUUGUUGUUG	Cleavage
tae-miR1134	Traes_2BL_8339D35AC.4										3,00	-1.0	1	24	302	325	CAACAACAACAAGAAGAAGAAGAU	AUCUUCAUGGUCUUGUUGUUGUUG	Cleavage
tae-miR1134	Traes_2BL_8339D35AC.5										3,00	-1.0	1	24	509	532	CAACAACAACAAGAAGAAGAAGAU	AUCUUCAUGGUCUUGUUGUUGUUG	Cleavage
tae-miR1135	Traes_2BL_A4AE931B0.1						AT2G02090.1	SNF2 domain-containing protein / helicase domain-containing protein	LOC_Os04g47830.1	SNF2 family N-terminal domain containing protein, expressed	2,00	-1.0	1	24	1154	1177	CUGCGACAAGUAAUUCCGAACGGA	UCUGUCCGGAAUUACUUGUCGCAA	Cleavage
tae-miR1122b-3p	Traes_2BL_FF8002798.2										2,05	-1.0	1	21	889	909	AGACUUAUAUGUAGGAACGGA	UCGGUUCCUAAAUAUAAGUCU	Translation
tae-miR9778	Traes_2BS_0EFFAAE28.1	PF00931				GO:GO:0043531	AT3G07040.1	NB-ARC domain-containing disease resistance protein	LOC_Os08g14850.1	resistance protein, putative, expressed	2,05	-1.0	1	21	126	146	UGCAUCAUCUCGAACUCGUCG	CGGCGAGUUCCAGAUGAUGCA	Translation
tae-miR9778	Traes_2BS_0EFFAAE28.2	PF00931				GO:GO:0043531	AT3G07040.1	NB-ARC domain-containing disease resistance protein	LOC_Os08g14850.1	resistance protein, putative, expressed	2,05	-1.0	1	21	126	146	UGCAUCAUCUCGAACUCGUCG	CGGCGAGUUCCAGAUGAUGCA	Translation
tae-miR9662a-3p	Traes_2BS_257C5B099.1	PF02536				GO:GO:0006355,GO:GO:0005739,GO:GO:0003690	AT5G07900.1	Mitochondrial transcription termination factor family protein	LOC_Os06g12100.1	mTERF family protein, expressed	2,00	-1.0	1	21	66	86	UUGAACAUCCCAGAGCCACCG	CCGUGGUUCUGGGAUGUUCAG	Cleavage
tae-miR9662b-3p	Traes_2BS_257C5B099.1	PF02536				GO:GO:0006355,GO:GO:0005739,GO:GO:0003690	AT5G07900.1	Mitochondrial transcription termination factor family protein	LOC_Os06g12100.1	mTERF family protein, expressed	2,00	-1.0	1	21	65	85	UGAACAUCCCAGAGCCACCGG	CCCGUGGUUCUGGGAUGUUCA	Cleavage
tae-miR9662a-3p	Traes_2BS_257C5B0991.1	PF02536				GO:GO:0006355,GO:GO:0005739,GO:GO:0003690	AT5G07900.1	Mitochondrial transcription termination factor family protein	LOC_Os06g12100.1	mTERF family protein, expressed	2,00	-1.0	1	21	66	86	UUGAACAUCCCAGAGCCACCG	CCGUGGUUCUGGGAUGUUCAG	Cleavage

tae-miR9662b-3p	Traes_2BS_257C580991.1	PF02536				GO:GO:0006355,GO:GO:0005739,GO:GO:0003690	AT5G07900.1	Mitochondrial transcription termination factor family protein	LOC_Os06g12100.1	mTERF family protein, expressed	2,00	-1.0	1	21	65	85	UGAACAUCCCAGAGCCACCGG	CCCGUGGUUCUGGGAUGUUCA	Cleavage
tae-miR5048-5p	Traes_2BS_47E2D6444.1	PF07714		2.7.11.1		GO:GO:0006468,GO:GO:0004672	AT4G04570.2	cysteine-rich RLK (RECEPTOR-like protein kinase) 40	LOC_Os04g30030.1	cysteine-rich receptor-like protein kinase 12 precursor, putative, expressed	2,05	-1.0	1	22	221	242	UUUGCAGGUUUUAGGUCUAAGU	ACCUGGACCUAAAACCUGCGAA	Cleavage
tae-miR5048-5p	Traes_2BS_47E2D6444.2	PF00069	KOG1187	2.7.11.1		GO:GO:0006468,GO:GO:0005524,GO:GO:0004672	AT4G38830.1	cysteine-rich RLK (RECEPTOR-like protein kinase) 26	LOC_Os04g30030.1	cysteine-rich receptor-like protein kinase 12 precursor, putative, expressed	2,05	-1.0	1	22	959	980	UUUGCAGGUUUUAGGUCUAAGU	ACCUGGACCUAAAACCUGCGAA	Cleavage
tae-miR5048-5p	Traes_2BS_47E2D6444.3	PF00069	KOG1187	2.7.11.1		GO:GO:0006468,GO:GO:0005524,GO:GO:0004672	AT4G38830.1	cysteine-rich RLK (RECEPTOR-like protein kinase) 26	LOC_Os04g30030.1	cysteine-rich receptor-like protein kinase 12 precursor, putative, expressed	2,05	-1.0	1	22	947	968	UUUGCAGGUUUUAGGUCUAAGU	ACCUGGACCUAAAACCUGCGAA	Cleavage
tae-miR5048-5p	Traes_2BS_47E2D6444.4	PF07714		2.7.11.1		GO:GO:0006468,GO:GO:0004672	AT4G23160.1	cysteine-rich RLK (RECEPTOR-like protein kinase) 8	LOC_Os04g30030.1	cysteine-rich receptor-like protein kinase 12 precursor, putative, expressed	2,05	-1.0	1	22	221	242	UUUGCAGGUUUUAGGUCUAAGU	ACCUGGACCUAAAACCUGCGAA	Cleavage
tae-miR9664-3p	Traes_2BS_5045C640C.1	PF00931	KOG4658		K13457	GO:GO:0043531	AT3G07040.1	NB-ARC domain-containing disease resistance protein	LOC_Os11g46210.1	NB-ARC domain containing protein, expressed	2,05	-1.0	1	21	240	260	UUGCAGUCCUCGAUGUCGUAG	CUACGACAUCGAGGAUUGCAU	Cleavage
tae-miR9664-3p	Traes_2BS_5045C640C.2	PF00931	KOG4658		K13457	GO:GO:0043531	AT3G07040.1	NB-ARC domain-containing disease resistance protein	LOC_Os11g46210.1	NB-ARC domain containing protein, expressed	2,05	-1.0	1	21	240	260	UUGCAGUCCUCGAUGUCGUAG	CUACGACAUCGAGGAUUGCAU	Cleavage
tae-miR9664-3p	Traes_2BS_5045C640C.5	PF00931	KOG4658		K13457	GO:GO:0043531	AT3G07040.1	NB-ARC domain-containing disease resistance protein	LOC_Os11g46210.1	NB-ARC domain containing protein, expressed	2,05	-1.0	1	21	240	260	UUGCAGUCCUCGAUGUCGUAG	CUACGACAUCGAGGAUUGCAU	Cleavage
tae-miR1120c-5p	Traes_2BS_5C64FC44A.1										0,00	-1.0	1	21	21	41	UAAUAUAAGAACGUUUUUGAC	GUCAAAAACGUUCUUAUAUUA	Cleavage
tae-miR1120c-5p	Traes_2BS_5C64FC44A.2										0,00	-1.0	1	21	2	22	UAAUAUAAGAACGUUUUUGAC	GUCAAAAACGUUCUUAUAUUA	Cleavage
tae-miR1130b-3p	Traes_2BS_5CDCD5152.1										2,00	-1.0	1	20	1	21	UCUUAUAUUAUGGGA-CGGAG	GUCCGAUCCCAUAAUAUAAGA	Cleavage
tae-miR1121	Traes_2BS_6249E432F.1										0,00	-1.0	1	22	538	559	AGUAGUGAUCUAAACGCUCUUA	UAAGAGCGUUUAGAUCACUACU	Cleavage
tae-miR1121	Traes_2BS_6249E432F.2										0,00	-1.0	1	22	538	559	AGUAGUGAUCUAAACGCUCUUA	UAAGAGCGUUUAGAUCACUACU	Cleavage
tae-miR1121	Traes_2BS_6249E432F.3										0,00	-1.0	1	22	448	469	AGUAGUGAUCUAAACGCUCUUA	UAAGAGCGUUUAGAUCACUACU	Cleavage
tae-miR1133	Traes_2BS_67D862F4A.1										2,05	-1.0	1	22	30	51	CAUAUACUCCCUCCGUCCGAAA	UUUGGGACGGAGGGAGUAUAAG	Cleavage
tae-miR9662a-3p	Traes_2BS_74269A4C5.1	PF02536				GO:GO:0006355,GO:GO:0005739,GO:GO:0003690	AT5G07900.1	Mitochondrial transcription termination factor family protein	LOC_Os06g12100.1	mTERF family protein, expressed	2,00	-1.0	1	21	189	209	UUGAACAUCCCAGAGCCACCG	CCGUGGUUCUGGGAUGUUCAG	Cleavage
tae-miR9662b-3p	Traes_2BS_74269A4C5.1	PF02536				GO:GO:0006355,GO:GO:0005739,GO:GO:0003690	AT5G07900.1	Mitochondrial transcription termination factor family protein	LOC_Os06g12100.1	mTERF family protein, expressed	2,00	-1.0	1	21	188	208	UGAACAUCCCAGAGCCACCGG	CCCGUGGUUCUGGGAUGUUCA	Cleavage
tae-miR1128	Traes_2BS_7AEB138E8.1										1,05	-1.0	1	21	84	104	UACUACUCCCUCCGUCCGAAA	UUUCGGACGGAGGGAGUACUA	Cleavage
tae-miR9661-5p	Traes_2BS_8281050F1.1										3,00	-1.0	1	21	845	865	UGAAGUAGAGCAGGGACCUCA	GACCGUUCCUGCUCUACUUCA	Cleavage
tae-miR9661-5p	Traes_2BS_8281050F11.1										3,00	-1.0	1	21	845	865	UGAAGUAGAGCAGGGACCUCA	GACCGUUCCUGCUCUACUUCA	Cleavage
tae-miR1122b-3p	Traes_2BS_A20CCBEC3.1										2,05	-1.0	1	21	389	409	AGACUUAUAUGUAGGAACGGA	UCUGUUCCUAAAUAUAAGUCU	Translation
tae-miR1128	Traes_2BS_B3A88F254.2										2,00	-1.0	1	21	88	109	UACUACUCCCUCCGUCC-GAAA	UUUCGGACGGAGGGAGUAGUA	Cleavage
tae-miR1128	Traes_2BS_B3A88F254.3										2,00	-1.0	1	21	88	109	UACUACUCCCUCCGUCC-GAAA	UUUCGGACGGAGGGAGUAGUA	Cleavage
tae-miR1127a	Traes_2BS_C00FC92D4.2										1,05	-1.0	1	19	497	515	UCCUUCCGUUCGGAAUUAC	GUAAUUCCGAACGGAGGGA	Cleavage
tae-miR1117	Traes_2BS_C5AF5419E.1										0,00	-1.0	1	24	926	949	UAGUACCGGUUCGUGGCACGAACC	GGUUCGUGCCACGAACCGGUACUA	Cleavage
tae-miR9662a-3p	Traes_2BS_DDF26C784.1	PF02536				GO:GO:0006355,GO:GO:0005739,GO:GO:0003690	AT5G07900.1	Mitochondrial transcription termination factor family protein	LOC_Os06g12100.1	mTERF family protein, expressed	2,00	-1.0	1	21	459	479	UUGAACAUCCCAGAGCCACCG	CCGUGGUUCUGGGAUGUUCAG	Cleavage
tae-miR9662b-3p	Traes_2BS_DDF26C784.1	PF02536				GO:GO:0006355,GO:GO:0005739,GO:GO:0003690	AT5G07900.1	Mitochondrial transcription termination factor family protein	LOC_Os06g12100.1	mTERF family protein, expressed	2,00	-1.0	1	21	458	478	UGAACAUCCCAGAGCCACCGG	CCCGUGGUUCUGGGAUGUUCA	Cleavage
tae-miR5384-3p	Traes_2BS_EBCD10293.1										2,05	-1.0	1	21	116	136	UGAGCGCGCCGCCGUCGAAUG	UGUUCGCCGACGGCGCGCUCA	Cleavage
tae-miR5384-3p	Traes_2BS_EBCD10293.2										2,05	-1.0	1	21	116	136	UGAGCGCGCCGCCGUCGAAUG	UGUUCGCCGACGGCGCGCUCA	Cleavage
tae-miR1130b-3p	Traes_2DL_10B2965EF.8										0,00	-1.0	1	21	752	772	UCUUAUAUUAUGGGACGGAGG	CCUCCGUCCCAUAAUAUAAGA	Cleavage
tae-miR1122c-3p	Traes_2DL_10B2965EF.8										1,05	-1.0	1	21	752	772	UCUAAUAUUAUGGGACGGAGG	CCUCCGUCCCAUAAUAUAAGA	Cleavage
tae-miR1120c-5p	Traes_2DL_10B2965EF.8										1,05	-1.0	1	21	795	815	UAAUAUAAGAACGUUUUUGAC	GUCAAAAACGUCUUUAUAUUA	Translation
tae-miR1130b-3p	Traes_2DL_3C9129432.1										1,05	-1.0	1	21	164	184	UCUUAUAUUAUGGGACGGAGG	CUUCCGUCCCAUAAUGUAAGA	Cleavage
tae-miR1133	Traes_2DL_4481C0380.4										2,05	-1.0	1	22	1	22	CAUAUACUCCCUCCGUCCGAAA	UUUGGGACGGAGGGAGUACAUG	Cleavage
tae-miR1137a	Traes_2DL_4F8C87C42.5										1,00	-1.0	1	20	1791	1810	UAGUACAAAGUUGAGUCAUC	GAUGACCCAACUUUGUACUA	Cleavage
tae-miR1127b-3p	Traes_2DL_4F8C87C42.5										1,05	-1.0	1	21	1822	1842	ACAAGUAUUUCUGGACGGAGG	CCUCCGUCCGGAAAUACUUGU	Cleavage
tae-miR1137b-5p	Traes_2DL_4F8C87C42.5										1,05	-1.0	1	21	1944	1964	UCCGUUCCAGAAUAGAUGACC	GGUCAUCUAUUUUGGAACGGA	Translation
tae-miR1122a	Traes_2DL_4F8C87C42.6										2,05	-1.0	1	20	1750	1769	UAGAUACAUCCGUAUCUAGA	ACUACAUACGGAUGUAUAUA	Cleavage
tae-miR1121	Traes_2DL_74E241841.2										2,05	-1.0	1	22	448	469	AGUAGUGAUCUAAACGCUCUUA	UAAGAGUGUUUGGAUCACUACU	Translation
tae-miR1130b-3p	Traes_2DL_8F04980F0.5										2,05	-1.0	1	21	468	488	UCUUAUAUUAUGGGACGGAGG	CCUCCGUCCCUAAUAUAAGG	Translation
tae-miR1128	Traes_2DL_9AD532712.8										2,00	-1.0	1	21	305	325	UACUACUCCCUCCGUCCGAAA	GUCCGGACGGAGGGAGUAGUU	Cleavage
tae-miR1122a	Traes_2DL_B3CD23EBC.1										2,05	-1.0	1	20	9	28	UAGAUACAUCCGUAUCUAGA	ACUACAUACGGAUGUAUAUA	Cleavage
tae-miR9662a-3p	Traes_2DL_BE754F0C1.1	PF02536	KOG1267		K15032	GO:GO:0006355,GO:GO:0005739,GO:GO:0003690	AT5G07900.1	Mitochondrial transcription termination factor family protein	LOC_Os06g12100.1	mTERF family protein, expressed	1,00	-1.0	1	21	687	707	UUGAACAUCCCAGAGCCACCG	CCGUGGCUCUGGGAUGUUCAG	Cleavage
tae-miR9662b-3p	Traes_2DL_BE754F0C1.1	PF02536	KOG1267		K15032	GO:GO:0006355,GO:GO:0005739,GO:GO:0003690	AT5G07900.1	Mitochondrial transcription termination factor family protein	LOC_Os06g12100.1	mTERF family protein, expressed	1,00	-1.0	1	21	686	706	UGAACAUCCCAGAGCCACCGG	CCCGUGGCUCUGGGAUGUUCA	Cleavage
tae-miR1134	Traes_2DL_E8A7ABB5D.1										3,00	-1.0	1	24	509	532	CAACAACAACAAGAAGAAGAAGAU	AUCUUCAUGGUCUUGUUGUUGUUG	Cleavage
tae-miR1134	Traes_2DL_E8A7ABB5D.2										3,00	-1.0	1	24	629	652	CAACAACAACAAGAAGAAGAAGAU	AUCUUCAUGGUCUUGUUGUUGUUG	Cleavage
tae-miR1134	Traes_2DL_E8A7ABB5D.3										3,00	-1.0	1	24	614	637	CAACAACAACAAGAAGAAGAAGAU	AUCUUCAUGGUCUUGUUGUUGUUG	Cleavage
tae-miR1134	Traes_2DL_E8A7ABB5D.4										3,00	-1.0	1	24	512	535	CAACAACAACAAGAAGAAGAAGAU	AUCUUCAUGGUCUUGUUGUUGUUG	Cleavage
tae-miR1134	Traes_2DL_E8A7ABB5D.5										3,00	-1.0	1	24	509	532	CAACAACAACAAGAAGAAGAAGAU	AUCUUCAUGGUCUUGUUGUUGUUG	Cleavage
tae-miR1134	Traes_2DL_E8A7ABB5D.6										3,00	-1.0	1	24	509	532	CAACAACAACAAGAAGAAGAAGAU	AUCUUCAUGGUCUUGUUGUUGUUG	Cleavage
tae-miR1134	Traes_2DL_E8A7ABB5D.7										3,00	-1.0	1	24	91	114	CAACAACAACAAGAAGAAGAAGAU	AUCUUCAUGGUCUUGUUGUUGUUG	Cleavage
tae-miR1134	Traes_2DL_E8A7ABB5D.8										3,00	-1.0	1	24	77	100	CAACAACAACAAGAAGAAGAAGAU	AUCUUCAUGGUCUUGUUGUUGUUG	Cleavage
tae-miR1134	Traes_2DL_E8A7ABB5D.9										3,00	-1.0	1	24	28	51	CAACAACAACAAGAAGAAGAAGAU	AUCUUCAUGGUCUUGUUGUUGUUG	Cleavage
tae-miR160	Traes_2DL_EF12C0B89.2										1,00	-1.0	1	21	225	245	UGCCUGGCUCCCUGUAUGCCA	UGGCAUGCAGGGAGCCAGGCA	Cleavage
tae-miR9664-3p	Traes_2DS_480CFDFE4.1	PF01637	KOG4658		K13457	GO:GO:0005524	AT3G07040.1	NB-ARC domain-containing disease resistance protein	LOC_Os11g46210.1	NB-ARC domain containing protein, expressed	2,05	-1.0	1	21	243	263	UUGCAGUCCUCGAUGUCGUAG	CUACGACAUCGAGGAUUGCAU	Cleavage
tae-miR9664-3p	Traes_2DS_480CFDFE4.2	PF01637				GO:GO:0005524	AT3G07040.1	NB-ARC domain-containing disease resistance protein	LOC_Os11g46210.1	NB-ARC domain containing protein, expressed	2,05	-1.0	1	21	259	279	UUGCAGUCCUCGAUGUCGUAG	CUACGACAUCGAGGAUUGCAU	Cleavage
tae-miR5049-3p	Traes_2DS_851973269.7										1,05	-1.0	1	21	178	198	AAUAUGGAUCGGAGGGAGUAC	GUACUCCCUCCGAUCCAUAUU	Cleavage
tae-miR1122b-3p	Traes_2DS_9B301D4F8.2										1,05	-1.0	1	21	843	863	AGACUUAUAUGUAGGAACGGA	UCCGUUCCUAAAUAUAAGUCU	Translation
tae-miR1123	Traes_2DS_C866A54F1.2										1,00	-1.0	1	23	792	814	UCCGUGAGACCUGGUCUCAUAGA	UCUAUGAGACCAGGUCUCACGGG	Cleavage
tae-miR1123	Traes_2DS_C866A54F1.4										1,00	-1.0	1	23	786	808	UCCGUGAGACCUGGUCUCAUAGA	UCUAUGAGACCAGGUCUCACGGG	Cleavage
tae-miR9778	Traes_2DS_C9635AFF7.1	PF00931				GO:GO:0043531	AT3G07040.1	NB-ARC domain-containing disease resistance protein	LOC_Os08g14850.1	resistance protein, putative, expressed	2,05	-1.0	1	21	126	146	UGCAUCAUCUCGAACUCGUCG	CGGCGAGUUCCAGAUGAUGCA	Translation
tae-miR9778	Traes_2DS_C9635AFF7.2	PF00931				GO:GO:0043531	AT3G07040.1	NB-ARC domain-containing disease resistance protein	LOC_Os08g14850.1	resistance protein, putative, expressed	2,05	-1.0	1	21	105	125	UGCAUCAUCUCGAACUCGUCG	CGGCGAGUUCCAGAUGAUGCA	Translation
tae-miR1117	Traes_3AL_018D4C27C.1										1,05	-1.0	1	24	1609	1632	UAGUACCGGUUCGUGGCACGAACC	GGUUCGUGCCACGAACCGGUGCUA	Cleavage
tae-miR1133	Traes_3AL_30294D0A5.1										2,05	-1.0	1	22	392	413	CAUAUACUCCCUCCGUCCGAAA	UUUAGGACGGAGGGAGUAUUUG	Cleavage
tae-miR1133	Traes_3AL_386795528.2										0,00	-1.0	1	22	39	60	CAUAUACUCCCUCCGUCCGAAA	UUUCGGACGGAGGGAGUAUAUG	Cleavage
tae-miR1122a	Traes_3AL_5E5C8D264.2										1,00	-1.0	1	20	18	37	UAGAUACAUCCGUAUCUAGA	UCUAGACACGGAUGUAUCUA	Cleavage
tae-miR1137b-5p	Traes_3AL_7A4D34425.2										2,05	-1.0	1	21	1006	1026	UCCGUUCCAGAAUAGAUGACC	UAUCAUUUAUUCAGGAACGGA	Cleavage
tae-miR1122a	Traes_3AL_B9EDE61D1.1										2,05	-1.0	1	20	4	23	UAGAUACAUCCGUAUCUAGA	ACUACAUACGGAUGUAUAUA	Cleavage
tae-miR1122a	Traes_3AL_B9EDE61D1.2										2,05	-1.0	1	20	4	23	UAGAUACAUCCGUAUCUAGA	ACUACAUACGGAUGUAUAUA	Cleavage
tae-miR5049-3p	Traes_3AL_CF1A4ACDE.1										1,00	-1.0	1	21	917	937	AAUAUGGAUCGGAGGGAGUAC	UCCCUCCCUCCGAUCCAUAUU	Cleavage
tae-miR159a	Traes_3AL_F09C7CA19.1	PF00249					AT5G06100.1	myb domain protein 33	LOC_Os01g59660.2	MYB family transcription factor, putative, expressed	2,05	-1.0	1	21	620	640	UUUGGAUUGAAGGGAGCUCUG	UGGAGCUCCCUUCACUCCAAG	Cleavage
tae-miR159b	Traes_3AL_F09C7CA19.1	PF00249					AT5G06100.1	myb domain protein 33	LOC_Os01g59660.2	MYB family transcription factor, putative, expressed	2,05	-1.0	1	21	620	640	UUUGGAUUGAAGGGAGCUCUG	UGGAGCUCCCUUCACUCCAAG	Cleavage
tae-miR1121	Traes_3AL_F2EDB3661.7										2,00	-1.0	1	22	224	245	AGUAGUGAUCUAAACGCUCUUA	CCUCCGCGUUUAGAUCACUACU	Cleavage
tae-miR1133	Traes_3AL_FA6F160DF.7										3,00	-1.0	1	22	142	164	CAUAUACUCCCUCCG-UCCGAAA	UUUCGGACGGAGGGAGUAUAUU	Cleavage
tae-miR5200	Traes_3AS_2A0765E10.1										2,05	-1.0	1	21	177	197	UGUAGAUACUCCCUAAGGCUU	UUCCCUUAGGAAGUAUCUACA	Translation
tae-miR5200	Traes_3AS_2A0765E10.2										2,05	-1.0	1	21	66	86	UGUAGAUACUCCCUAAGGCUU	UUCCCUUAGGAAGUAUCUACA	Translation
tae-miR5200	Traes_3AS_2A0765E10.3										2,05	-1.0	1	21	12	32	UGUAGAUACUCCCUAAGGCUU	UUCCCUUAGGAAGUAUCUACA	Translation
tae-miR9674b-5p	Traes_3AS_6CB1DBD28.1										2,00	-1.0	1	21	429	449	AUAGCAUCAUCCAUCCUACCC	GGGUAGGCUGGAUGAUGCUAG	Cleavage
tae-miR9674b-5p	Traes_3AS_6CB1DBD28.1										2,00	-1.0	1	21	429	449	AUAGCAUCAUCCAUCCUACCC	GGGUAGGCUGGAUGAUGCUAG	Cleavage
tae-miR1121	Traes_3AS_A466B9ECC.1										0,00	-1.0	1	22	725	746	AGUAGUGAUCUAAACGCUCUUA	UAAGAGCGUUUAGAUCACUACU	Cleavage
tae-miR6197-5p	Traes_3AS_A466B9ECC.1										2,05	-1.0	1	21	763	783	UCUGUAAACAAAUGUAGGACG	GCUCUUACAUUUCUUUACAGA	Cleavage
tae-miR1121	Traes_3AS_A466B9ECC.2										0,00	-1.0	1	22	725	746	AGUAGUGAUCUAAACGCUCUUA	UAAGAGCGUUUAGAUCACUACU	Cleavage
tae-miR6197-5p	Traes_3AS_A466B9ECC.2										2,05	-1.0	1	21	763	783	UCUGUAAACAAAUGUAGGACG	GCUCUUACAUUUCUUUACAGA	Cleavage
tae-miR1133	Traes_3AS_A6D3DD27E.2										2,05	-1.0	1	22	16	37	CAUAUACUCCCUCCGUCCGAAA	UUUGGGACGGAGGGAGUAUGUG	Cleavage
tae-miR1133	Traes_3AS_A6D3DD27E.4										2,05	-1.0	1	22	9	30	CAUAUACUCCCUCCGUCCGAAA	UUUGGGACGGAGGGAGUAUGUG	Cleavage
tae-miR1130b-3p	Traes_3AS_AA474B823.1										1,05	-1.0	1	21	1652	1672	UCUUAUAUUAUGGGACGGAGG	CCUCCGUCCCAUAAUAUGAGA	Cleavage
tae-miR1122c-3p	Traes_3AS_AA474B823.1										1,05	-1.0	1	21	1652	1672	UCUAAUAUUAUGGGACGGAGG	CCUCCGUCCCAUAAUAUGAGA	Cleavage
tae-miR1130b-3p	Traes_3AS_AA474B823.3										1,05	-1.0	1	21	1397	1417	UCUUAUAUUAUGGGACGGAGG	CCUCCGUCCCAUAAUAUGAGA	Cleavage
tae-miR1122c-3p	Traes_3AS_AA474B823.3										1,05	-1.0	1	21	1397	1417	UCUAAUAUUAUGGGACGGAGG	CCUCCGUCCCAUAAUAUGAGA	Cleavage
tae-miR159a	Traes_3AS_D1D1A1994.1								LOC_Os01g11430.1	expressed protein	2,05	-1.0	1	21	101	121	UUUGGAUUGAAGGGAGCUCUG	CAGAGCCCCCUUCAAACCAAA	Cleavage
tae-miR159b	Traes_3AS_D1D1A1994.1								LOC_Os01g11430.1	expressed protein	2,05	-1.0	1	21	101	121	UUUGGAUUGAAGGGAGCUCUG	CAGAGCCCCCUUCAAACCAAA	Cleavage
tae-miR1122b-3p	Traes_3AS_D7F2F36BB.2										1,05	-1.0	1	21	564	584	AGACUUAUAUGUAGGAACGGA	UCCGUUCCUAAAUAUAAGUCU	Translation
tae-miR1122b-3p	Traes_3AS_D7F2F36BB.3										1,05	-1.0	1	21	540	560	AGACUUAUAUGUAGGAACGGA	UCCGUUCCUAAAUAUAAGUCU	Translation
tae-miR5200	Traes_3B_0E2173F88.1										2,05	-1.0	1	21	171	191	UGUAGAUACUCCCUAAGGCUU	UUCCCUUAGGAAGUAUCUACA	Translation
tae-miR5200	Traes_3B_0E2173F88.2										2,05	-1.0	1	21	177	197	UGUAGAUACUCCCUAAGGCUU	UUCCCUUAGGAAGUAUCUACA	Translation
tae-miR5200	Traes_3B_0E2173F88.3										2,05	-1.0	1	21	171	191	UGUAGAUACUCCCUAAGGCUU	UUCCCUUAGGAAGUAUCUACA	Translation
tae-miR9652-5p	Traes_3B_119F6A405.1										2,05	-1.0	1	22	589	610	CCUGUUUGUCAUUAAGUUUCUU	AAGAAACUGAAUGACAAGCAGG	Cleavage
tae-miR9662a-3p	Traes_3B_224A6B7E5.1	PF02536				GO:GO:0006355,GO:GO:0005739,GO:GO:0003690	AT5G07900.1	Mitochondrial transcription termination factor family protein	LOC_Os06g12100.1	mTERF family protein, expressed	2,00	-1.0	1	21	189	209	UUGAACAUCCCAGAGCCACCG	CCGUGGUUCUGGGAUGUUCAG	Cleavage
tae-miR9662b-3p	Traes_3B_224A6B7E5.1	PF02536				GO:GO:0006355,GO:GO:0005739,GO:GO:0003690	AT5G07900.1	Mitochondrial transcription termination factor family protein	LOC_Os06g12100.1	mTERF family protein, expressed	2,00	-1.0	1	21	188	208	UGAACAUCCCAGAGCCACCGG	CCCGUGGUUCUGGGAUGUUCA	Cleavage
tae-miR159a	Traes_3B_25320ED72.1	PF00249	KOG0048		K09422		AT5G06100.1	myb domain protein 33	LOC_Os01g59660.2	MYB family transcription factor, putative, expressed	2,05	-1.0	1	21	956	976	UUUGGAUUGAAGGGAGCUCUG	UGGAGCUCCCUUCACUCCAAG	Cleavage
tae-miR159b	Traes_3B_25320ED72.1	PF00249	KOG0048		K09422		AT5G06100.1	myb domain protein 33	LOC_Os01g59660.2	MYB family transcription factor, putative, expressed	2,05	-1.0	1	21	956	976	UUUGGAUUGAAGGGAGCUCUG	UGGAGCUCCCUUCACUCCAAG	Cleavage
tae-miR159a	Traes_3B_25320ED72.2	PF00249	KOG0048		K09422		AT5G06100.1	myb domain protein 33	LOC_Os01g59660.1	MYB family transcription factor, putative, expressed	2,05	-1.0	1	21	956	976	UUUGGAUUGAAGGGAGCUCUG	UGGAGCUCCCUUCACUCCAAG	Cleavage
tae-miR159b	Traes_3B_25320ED72.2	PF00249	KOG0048		K09422		AT5G06100.1	myb domain protein 33	LOC_Os01g59660.1	MYB family transcription factor, putative, expressed	2,05	-1.0	1	21	956	976	UUUGGAUUGAAGGGAGCUCUG	UGGAGCUCCCUUCACUCCAAG	Cleavage
tae-miR1130b-3p	Traes_3B_467787390.8										1,05	-1.0	1	21	3080	3100	UCUUAUAUUAUGGGACGGAGG	CCUCCGUCCCAAAAUAUAAGA	Translation
tae-miR9776	Traes_3B_470061369.1	PF00270		3.6.4.13		GO:GO:0005524,GO:GO:0003676	AT5G51280.1	DEAD-box protein abstrakt, putative	LOC_Os02g05660.1	DEAD-box ATP-dependent RNA helicase 35A, putative, expressed	2,05	-1.0	1	21	34	54	UUGGACGAGGAUGUGCAACUG	AAGUGGCACAUCCUCGUCGAA	Cleavage
tae-miR1122a	Traes_3B_472072804.1										1,05	-1.0	1	20	789	808	UAGAUACAUCCGUAUCUAGA	UCUAGAUACGGAGGUAUCUA	Cleavage
tae-miR9778	Traes_3B_4C839468C.10						AT3G46530.1	NB-ARC domain-containing disease resistance protein	LOC_Os12g37290.1	resistance protein T10rga2-1A, putative, expressed	1,00	-1.0	1	21	126	146	UGCAUCAUCUCGAACUCGUCG	GGACGAGCUCGAGAUGAUGCA	Cleavage
tae-miR9778	Traes_3B_4C839468C.11						AT3G46530.1	NB-ARC domain-containing disease resistance protein	LOC_Os12g37290.1	resistance protein T10rga2-1A, putative, expressed	1,00	-1.0	1	21	126	146	UGCAUCAUCUCGAACUCGUCG	GGACGAGCUCGAGAUGAUGCA	Cleavage
tae-miR9778	Traes_3B_4C839468C.12						AT3G46530.1	NB-ARC domain-containing disease resistance protein	LOC_Os12g37290.1	resistance protein T10rga2-1A, putative, expressed	1,00	-1.0	1	21	126	146	UGCAUCAUCUCGAACUCGUCG	GGACGAGCUCGAGAUGAUGCA	Cleavage
tae-miR9778	Traes_3B_4C839468C.13						AT1G50180.1	NB-ARC domain-containing disease resistance protein	LOC_Os02g57310.1	pib, putative, expressed	1,00	-1.0	1	21	102	122	UGCAUCAUCUCGAACUCGUCG	GGACGAGCUCGAGAUGAUGCA	Cleavage
tae-miR9778	Traes_3B_4C839468C.2						AT3G46530.1	NB-ARC domain-containing disease resistance protein	LOC_Os12g37290.1	resistance protein T10rga2-1A, putative, expressed	1,00	-1.0	1	21	126	146	UGCAUCAUCUCGAACUCGUCG	GGACGAGCUCGAGAUGAUGCA	Cleavage
tae-miR9778	Traes_3B_4C839468C.3						AT3G46530.1	NB-ARC domain-containing disease resistance protein	LOC_Os12g37290.1	resistance protein T10rga2-1A, putative, expressed	1,00	-1.0	1	21	126	146	UGCAUCAUCUCGAACUCGUCG	GGACGAGCUCGAGAUGAUGCA	Cleavage
tae-miR9778	Traes_3B_4C839468C.4						AT3G46530.1	NB-ARC domain-containing disease resistance protein	LOC_Os12g37290.1	resistance protein T10rga2-1A, putative, expressed	1,00	-1.0	1	21	126	146	UGCAUCAUCUCGAACUCGUCG	GGACGAGCUCGAGAUGAUGCA	Cleavage
tae-miR9778	Traes_3B_4C839468C.5						AT3G46530.1	NB-ARC domain-containing disease resistance protein	LOC_Os12g37290.1	resistance protein T10rga2-1A, putative, expressed	1,00	-1.0	1	21	126	146	UGCAUCAUCUCGAACUCGUCG	GGACGAGCUCGAGAUGAUGCA	Cleavage
tae-miR9778	Traes_3B_4C839468C.6						AT3G46530.1	NB-ARC domain-containing disease resistance protein	LOC_Os12g37290.1	resistance protein T10rga2-1A, putative, expressed	1,00	-1.0	1	21	126	146	UGCAUCAUCUCGAACUCGUCG	GGACGAGCUCGAGAUGAUGCA	Cleavage
tae-miR9778	Traes_3B_4C839468C.7						AT3G46530.1	NB-ARC domain-containing disease resistance protein	LOC_Os12g37290.1	resistance protein T10rga2-1A, putative, expressed	1,00	-1.0	1	21	126	146	UGCAUCAUCUCGAACUCGUCG	GGACGAGCUCGAGAUGAUGCA	Cleavage
tae-miR9778	Traes_3B_4C839468C.8						AT3G46530.1	NB-ARC domain-containing disease resistance protein	LOC_Os12g37290.1	resistance protein T10rga2-1A, putative, expressed	1,00	-1.0	1	21	126	146	UGCAUCAUCUCGAACUCGUCG	GGACGAGCUCGAGAUGAUGCA	Cleavage
tae-miR9778	Traes_3B_4C839468C.9						AT3G46530.1	NB-ARC domain-containing disease resistance protein	LOC_Os12g37290.1	resistance protein T10rga2-1A, putative, expressed	1,00	-1.0	1	21	126	146	UGCAUCAUCUCGAACUCGUCG	GGACGAGCUCGAGAUGAUGCA	Cleavage
tae-miR1137a	Traes_3B_59B827C5C.2										0,00	-1.0	1	20	748	767	UAGUACAAAGUUGAGUCAUC	GAUGACUCAACUUUGUACUA	Cleavage
tae-miR1122b-3p	Traes_3B_5DD9C1BE5.1										2,05	-1.0	1	21	71	91	AGACUUAUAUGUAGGAACGGA	UCCGUUCUUAAAUAUAAGUCU	Translation
tae-miR7757-5p	Traes_3B_5EFB9A61F.1								LOC_Os12g17490.1	stripe rust resistance protein Yr10, putative, expressed	1,05	-1.0	1	22	113	134	AUAAAACCUUCAGCUAUCCAUC	GAUGGAUAGCUGAAGGUUUUGU	Cleavage
tae-miR7757-5p	Traes_3B_5EFB9A61F.3								LOC_Os12g17490.1	stripe rust resistance protein Yr10, putative, expressed	1,05	-1.0	1	22	89	110	AUAAAACCUUCAGCUAUCCAUC	GAUGGAUAGCUGAAGGUUUUGU	Cleavage
tae-miR1122b-3p	Traes_3B_6D2DCE280.3										2,05	-1.0	1	21	576	596	AGACUUAUAUGUAGGAACGGA	UCUGUUCCUAAAUAUAAGUCU	Translation
tae-miR7757-5p	Traes_3B_7FFFF7BCF.1								LOC_Os02g18510.1	stripe rust resistance protein Yr10, putative, expressed	0,00	-1.0	1	22	89	110	AUAAAACCUUCAGCUAUCCAUC	GAUGGAUAGCUGAAGGUUUUAU	Cleavage
tae-miR5049-3p	Traes_3B_9D776A8C4.1										0,00	-1.0	1	21	575	595	AAUAUGGAUCGGAGGGAGUAC	GUACUCCCUCCGAUCCAUAUU	Cleavage
tae-miR1121	Traes_3B_9F321609E.8										1,05	-1.0	1	22	6	28	AGUAGUGAUCUAAACGCUCU-UA	UAUAGAGCGUUUAGAUCACUAUU	Cleavage
tae-miR9778	Traes_3B_F07C33521.1						AT3G46710.1	NB-ARC domain-containing disease resistance protein	LOC_Os02g57310.1	pib, putative, expressed	1,00	-1.0	1	21	126	146	UGCAUCAUCUCGAACUCGUCG	GGACGAGCUCGAGAUGAUGCA	Cleavage
tae-miR9778	Traes_3B_F07C33521.10						AT3G46710.1	NB-ARC domain-containing disease resistance protein	LOC_Os02g57310.1	pib, putative, expressed	1,00	-1.0	1	21	126	146	UGCAUCAUCUCGAACUCGUCG	GGACGAGCUCGAGAUGAUGCA	Cleavage
tae-miR9778	Traes_3B_F07C33521.2	PF00931				GO:GO:0043531	AT3G46530.1	NB-ARC domain-containing disease resistance protein	LOC_Os08g42670.2	resistance protein, putative, expressed	1,00	-1.0	1	21	126	146	UGCAUCAUCUCGAACUCGUCG	GGACGAGCUCGAGAUGAUGCA	Cleavage
tae-miR9778	Traes_3B_F07C33521.3	PF00931				GO:GO:0043531	AT3G46530.1	NB-ARC domain-containing disease resistance protein	LOC_Os08g42670.2	resistance protein, putative, expressed	1,00	-1.0	1	21	126	146	UGCAUCAUCUCGAACUCGUCG	GGACGAGCUCGAGAUGAUGCA	Cleavage
tae-miR9778	Traes_3B_F07C33521.4	PF00931				GO:GO:0043531	AT3G46530.1	NB-ARC domain-containing disease resistance protein	LOC_Os08g42670.2	resistance protein, putative, expressed	1,00	-1.0	1	21	126	146	UGCAUCAUCUCGAACUCGUCG	GGACGAGCUCGAGAUGAUGCA	Cleavage
tae-miR9778	Traes_3B_F07C33521.5	PF00931				GO:GO:0043531	AT3G46530.1	NB-ARC domain-containing disease resistance protein	LOC_Os08g42670.1	resistance protein, putative, expressed	1,00	-1.0	1	21	126	146	UGCAUCAUCUCGAACUCGUCG	GGACGAGCUCGAGAUGAUGCA	Cleavage
tae-miR9778	Traes_3B_F07C33521.6	PF00931				GO:GO:0043531	AT3G46530.1	NB-ARC domain-containing disease resistance protein	LOC_Os08g42670.1	resistance protein, putative, expressed	1,00	-1.0	1	21	126	146	UGCAUCAUCUCGAACUCGUCG	GGACGAGCUCGAGAUGAUGCA	Cleavage
tae-miR9778	Traes_3B_F07C33521.7	PF00931				GO:GO:0043531	AT3G46530.1	NB-ARC domain-containing disease resistance protein	LOC_Os08g42670.2	resistance protein, putative, expressed	1,00	-1.0	1	21	126	146	UGCAUCAUCUCGAACUCGUCG	GGACGAGCUCGAGAUGAUGCA	Cleavage
tae-miR9778	Traes_3B_F07C33521.8						AT3G46710.1	NB-ARC domain-containing disease resistance protein	LOC_Os02g57310.1	pib, putative, expressed	1,00	-1.0	1	21	126	146	UGCAUCAUCUCGAACUCGUCG	GGACGAGCUCGAGAUGAUGCA	Cleavage
tae-miR9778	Traes_3B_F07C33521.9						AT3G46710.1	NB-ARC domain-containing disease resistance protein	LOC_Os02g57310.1	pib, putative, expressed	1,00	-1.0	1	21	126	146	UGCAUCAUCUCGAACUCGUCG	GGACGAGCUCGAGAUGAUGCA	Cleavage
tae-miR1128	Traes_3B_F61EDC1AE.3										2,00	-1.0	1	21	1581	1601	UACUACUCCCUCCGUCCGAAA	UUCCGAACGGAGGGAGUAGUA	Cleavage
tae-miR1127a	Traes_3B_F61EDC1AE.3										1,05	-1.0	1	19	1577	1595	UCCUUCCGUUCGGAAUUAC	GUAAUUCCGAACGGAGGGA	Cleavage
tae-miR1130b-3p	Traes_3B_FB51F185F.2										1,05	-1.0	1	21	43	63	UCUUAUAUUAUGGGACGGAGG	CCUCCGUCCCAUAAUGUAAGA	Cleavage
tae-miR164	Traes_3B_FD8ACC50C.1										2,05	-1.0	1	21	1544	1564	UGGAGAAGCAGGGCACGUGCA	CGCACGGGCCCUGCUUCUGCA	Cleavage
tae-miR164	Traes_3B_FD8ACC50C.2										2,05	-1.0	1	21	1499	1519	UGGAGAAGCAGGGCACGUGCA	CGCACGGGCCCUGCUUCUGCA	Cleavage
tae-miR164	Traes_3B_FD8ACC50C.3										2,05	-1.0	1	21	1433	1453	UGGAGAAGCAGGGCACGUGCA	CGCACGGGCCCUGCUUCUGCA	Cleavage
tae-miR164	Traes_3B_FD8ACC50C.4										2,05	-1.0	1	21	764	784	UGGAGAAGCAGGGCACGUGCA	CGCACGGGCCCUGCUUCUGCA	Cleavage
tae-miR164	Traes_3B_FD8ACC50C.5										2,05	-1.0	1	21	752	772	UGGAGAAGCAGGGCACGUGCA	CGCACGGGCCCUGCUUCUGCA	Cleavage
tae-miR164	Traes_3B_FD8ACC50C.6										2,05	-1.0	1	21	731	751	UGGAGAAGCAGGGCACGUGCA	CGCACGGGCCCUGCUUCUGCA	Cleavage
tae-miR164	Traes_3B_FD8ACC50C.7										2,05	-1.0	1	21	710	730	UGGAGAAGCAGGGCACGUGCA	CGCACGGGCCCUGCUUCUGCA	Cleavage
tae-miR164	Traes_3B_FD8ACC50C.8										2,05	-1.0	1	21	653	673	UGGAGAAGCAGGGCACGUGCA	CGCACGGGCCCUGCUUCUGCA	Cleavage
tae-miR164	Traes_3B_FD8ACC50C.9										2,05	-1.0	1	21	356	376	UGGAGAAGCAGGGCACGUGCA	CGCACGGGCCCUGCUUCUGCA	Cleavage
tae-miR5049-3p	Traes_3DL_2B3694DC3.2										1,05	-1.0	1	21	228	248	AAUAUGGAUCGGAGGGAGUAC	CUACUCCCUCCGAUCCAAAUU	Cleavage
tae-miR1130b-3p	Traes_3DL_426EF6A68.2										2,05	-1.0	1	21	214	234	UCUUAUAUUAUGGGACGGAGG	CCUCCAUCCCGUAAUAUAAGA	Translation

tae-miR7757-5p	Traes_3DL_5C0BC5611.4								LOC_Os12g17490.1	stripe rust resistance protein Yr10, putative, expressed	1,05	-1.0	1	22	89	110	AUAAAACCUUCAGCUAUCCAUC	GAUGGAUAGCUGAAGGUUUUGU	Cleavage
tae-miR1137a	Traes_3DL_96847DCCF.1										1,00	-1.0	1	20	88	107	UAGUACAAAGUUGAGUCAUC	GAUGACCCAACUUUGUACUA	Cleavage
tae-miR5049-3p	Traes_3DL_D1CF4784D.2										2,05	-1.0	1	21	1646	1666	AAUAUGGAUCGGAGGGAGUAC	AUAAUCCCUCCGUUCCAUAUU	Cleavage
tae-miR159a	Traes_3DL_D9A29948B.1	PF00249					AT5G06100.1	myb domain protein 33	LOC_Os01g59660.1	MYB family transcription factor, putative, expressed	2,05	-1.0	1	21	620	640	UUUGGAUUGAAGGGAGCUCUG	UGGAGCUCCCUUCACUCCAAG	Cleavage
tae-miR159b	Traes_3DL_D9A29948B.1	PF00249					AT5G06100.1	myb domain protein 33	LOC_Os01g59660.1	MYB family transcription factor, putative, expressed	2,05	-1.0	1	21	620	640	UUUGGAUUGAAGGGAGCUCUG	UGGAGCUCCCUUCACUCCAAG	Cleavage
tae-miR159a	Traes_3DL_D9A29948B.2	PF00249					AT5G06100.1	myb domain protein 33	LOC_Os01g59660.1	MYB family transcription factor, putative, expressed	2,05	-1.0	1	21	620	640	UUUGGAUUGAAGGGAGCUCUG	UGGAGCUCCCUUCACUCCAAG	Cleavage
tae-miR159b	Traes_3DL_D9A29948B.2	PF00249					AT5G06100.1	myb domain protein 33	LOC_Os01g59660.1	MYB family transcription factor, putative, expressed	2,05	-1.0	1	21	620	640	UUUGGAUUGAAGGGAGCUCUG	UGGAGCUCCCUUCACUCCAAG	Cleavage
tae-miR1128	Traes_3DS_1DE20CFF3.2										2,05	-1.0	1	21	71	91	UACUACUCCCUCCGUCCGAAA	UUUGGGACGGAGGGAGUACUA	Cleavage
tae-miR1122a	Traes_3DS_1DE20CFF3.2										1,05	-1.0	1	20	2	21	UAGAUACAUCCGUAUCUAGA	UCUAGAUACAGAUGUAUCUA	Translation
tae-miR1128	Traes_3DS_1DE20CFF3.3										2,05	-1.0	1	21	71	91	UACUACUCCCUCCGUCCGAAA	UUUGGGACGGAGGGAGUACUA	Cleavage
tae-miR1122a	Traes_3DS_1DE20CFF3.3										1,05	-1.0	1	20	2	21	UAGAUACAUCCGUAUCUAGA	UCUAGAUACAGAUGUAUCUA	Translation
tae-miR1122b-3p	Traes_3DS_D42FCD586.1										1,05	-1.0	1	21	786	806	AGACUUAUAUGUAGGAACGGA	UCCGUUCCUAAAUAUAAGUCU	Translation
tae-miR1122b-3p	Traes_3DS_D42FCD586.2										1,05	-1.0	1	21	795	815	AGACUUAUAUGUAGGAACGGA	UCCGUUCCUAAAUAUAAGUCU	Translation
tae-miR7757-5p	Traes_4AL_13A3A40D3.1	PF13401	KOG4658				AT3G50950.2	HOPZ-ACTIVATED RESISTANCE 1	LOC_Os11g11810.1	NBS-LRR disease resistance protein, putative, expressed	1,05	-1.0	1	22	1337	1358	AUAAAACCUUCAGCUAUCCAUC	AGUGGAUAGCUGAAGGUUUCAU	Cleavage
tae-miR1135	Traes_4AL_158B970F4.1						AT1G50020.1		LOC_Os11g34870.1	expressed protein	2,05	-1.0	1	24	464	487	CUGCGACAAGUAAUUCCGAACGGA	UCCGUUCCGAAUUACUCGUCGCAG	Cleavage
tae-miR5175-5p	Traes_4AL_158B970F4.1										3,00	-1.0	1	21	532	552	UUCCAAAUUACUCGUCGUGGU	UCUGAGACGAGUAAUUUGGAA	Cleavage
tae-miR1117	Traes_4AL_2475C298D.1										2,05	-1.0	1	24	684	707	UAGUACCGGUUCGUGGCACGAACC	GGUUCAUGCCAUGAACCGGUACUA	Cleavage
tae-miR9778	Traes_4AL_2FC39B934.1	PF13191,PF00931				GO:GO:0043531	AT1G53350.1	Disease resistance protein (CC-NBS-LRR class) family	LOC_Os08g14850.1	resistance protein, putative, expressed	2,05	-1.0	1	21	126	146	UGCAUCAUCUCGAACUCGUCG	UGGCGAGUUCCAGAUGAUGCA	Translation
tae-miR408	Traes_4AL_364F56EDB.1										0,00	-1.0	1	21	8	28	CUGCACUGCCUCUUCCCUGGC	CCCAGGGAAGAGGCAGUGCAG	Cleavage
tae-miR9662a-3p	Traes_4AL_3A009739E.1	PF02536				GO:GO:0006355,GO:GO:0005739,GO:GO:0003690	AT1G21150.1	Mitochondrial transcription termination factor family protein	LOC_Os06g12100.1	mTERF family protein, expressed	2,05	-1.0	1	21	66	86	UUGAACAUCCCAGAGCCACCG	CCGUGGCUCUGGAAUGUUCAG	Cleavage
tae-miR9662b-3p	Traes_4AL_3A009739E.1	PF02536				GO:GO:0006355,GO:GO:0005739,GO:GO:0003690	AT1G21150.1	Mitochondrial transcription termination factor family protein	LOC_Os06g12100.1	mTERF family protein, expressed	2,05	-1.0	1	21	65	85	UGAACAUCCCAGAGCCACCGG	CCCGUGGCUCUGGAAUGUUCA	Cleavage
tae-miR1121	Traes_4AL_400EB3C17.10										2,05	-1.0	1	22	10	31	AGUAGUGAUCUAAACGCUCUUA	UAAGAGCGUUUAGAUCACUAAA	Cleavage
tae-miR1121	Traes_4AL_400EB3C17.3										2,05	-1.0	1	22	37	58	AGUAGUGAUCUAAACGCUCUUA	UAAGAGCGUUUAGAUCACUAAA	Cleavage
tae-miR1121	Traes_4AL_400EB3C17.7										2,05	-1.0	1	22	10	31	AGUAGUGAUCUAAACGCUCUUA	UAAGAGCGUUUAGAUCACUAAA	Cleavage
tae-miR1137a	Traes_4AL_40790E994.6										1,00	-1.0	1	20	1324	1343	UAGUACAAAGUUGAGUCAUC	GAUGACCCAACUUUGUACUA	Cleavage
tae-miR1137a	Traes_4AL_40790E994.7										1,00	-1.0	1	20	835	854	UAGUACAAAGUUGAGUCAUC	GAUGACCCAACUUUGUACUA	Cleavage
tae-miR1127b-3p	Traes_4AL_44E99BA01.2										1,05	-1.0	1	21	394	414	ACAAGUAUUUCUGGACGGAGG	CCUCCGUCCGGAAAUACUUGU	Cleavage
tae-miR1121	Traes_4AL_541C86EB9.2										1,00	-1.0	1	22	558	579	AGUAGUGAUCUAAACGCUCUUA	UGAGAUCGUUUAGAUCACUACU	Cleavage
tae-miR1121	Traes_4AL_541C86EB9.3										1,00	-1.0	1	22	558	579	AGUAGUGAUCUAAACGCUCUUA	UGAGAUCGUUUAGAUCACUACU	Cleavage
tae-miR1122b-3p	Traes_4AL_6252B0BA0.1										1,05	-1.0	1	21	46	66	AGACUUAUAUGUAGGAACGGA	UCCGUUCCUAAAUAUAAGUCU	Translation
tae-miR1137b-5p	Traes_4AL_748BB4675.1										2,05	-1.0	1	21	844	864	UCCGUUCCAGAAUAGAUGACC	ACUCAUCUAUUCUGGGACGGU	Cleavage
tae-miR9778	Traes_4AL_79B47AF61.1										2,05	-1.0	1	21	126	146	UGCAUCAUCUCGAACUCGUCG	GGACGAGCUCGACAUGAUGCA	Cleavage
tae-miR9778	Traes_4AL_79B47AF61.3	PF00931				GO:GO:0043531	AT3G46530.1	NB-ARC domain-containing disease resistance protein	LOC_Os08g42670.2	resistance protein, putative, expressed	2,05	-1.0	1	21	126	146	UGCAUCAUCUCGAACUCGUCG	GGACGAGCUCGACAUGAUGCA	Cleavage
tae-miR9778	Traes_4AL_79B47AF61.4	PF00931				GO:GO:0043531	AT3G46530.1	NB-ARC domain-containing disease resistance protein	LOC_Os08g42670.2	resistance protein, putative, expressed	2,05	-1.0	1	21	126	146	UGCAUCAUCUCGAACUCGUCG	GGACGAGCUCGACAUGAUGCA	Cleavage
tae-miR5048-5p	Traes_4AL_85A7C60F9.1	PF00069		2.7.11.1		GO:GO:0006468,GO:GO:0005524,GO:GO:0004672	AT4G21230.1	cysteine-rich RLK (RECEPTOR-like protein kinase) 27	LOC_Os11g10640.1	protein kinase domain containing protein, expressed	2,05	-1.0	1	22	170	191	UUUGCAGGUUUUAGGUCUAAGU	ACUUGGACCUAAAACCUGCGAA	Cleavage
tae-miR5048-5p	Traes_4AL_85A7C60F9.7	PF00069		2.7.11.1		GO:GO:0006468,GO:GO:0005524,GO:GO:0004672	AT4G21230.1	cysteine-rich RLK (RECEPTOR-like protein kinase) 27	LOC_Os11g10640.1	protein kinase domain containing protein, expressed	2,05	-1.0	1	22	170	191	UUUGCAGGUUUUAGGUCUAAGU	ACUUGGACCUAAAACCUGCGAA	Cleavage
tae-miR5048-5p	Traes_4AL_85A7C60F9.8	PF00069		2.7.11.1		GO:GO:0006468,GO:GO:0005524,GO:GO:0004672	AT4G21230.1	cysteine-rich RLK (RECEPTOR-like protein kinase) 27	LOC_Os11g10640.1	protein kinase domain containing protein, expressed	2,05	-1.0	1	22	170	191	UUUGCAGGUUUUAGGUCUAAGU	ACUUGGACCUAAAACCUGCGAA	Cleavage
tae-miR1122b-3p	Traes_4AL_954AE4933.1										1,05	-1.0	1	21	583	603	AGACUUAUAUGUAGGAACGGA	UCCGUUCCUAAAUAUAAGUCU	Translation
tae-miR1122a	Traes_4AL_9726307FF.1										0,00	-1.0	1	20	1903	1922	UAGAUACAUCCGUAUCUAGA	UCUAGAUACGGAUGUAUCUA	Cleavage
tae-miR1128	Traes_4AL_99942CBEA.1										3,00	-1.0	1	21	693	714	UACUACUCCCUCCG-UCCGAAA	UUUAGGAACGGAGGGAGUAGUA	Cleavage
tae-miR1122b-3p	Traes_4AL_99942CBEA.1										2,05	-1.0	1	21	557	577	AGACUUAUAUGUAGGAACGGA	UCCGUUCUUAAAUAUAAGUCU	Translation
tae-miR9662a-3p	Traes_4AL_A4B73CC6A1.1	PF02536	KOG1267		K15032	GO:GO:0006355,GO:GO:0005739,GO:GO:0003690	AT5G07900.1	Mitochondrial transcription termination factor family protein	LOC_Os06g12100.1	mTERF family protein, expressed	2,05	-1.0	1	21	693	713	UUGAACAUCCCAGAGCCACCG	CCGUGGCUCUGGAAUGUUCAG	Cleavage
tae-miR9662b-3p	Traes_4AL_A4B73CC6A1.1	PF02536	KOG1267		K15032	GO:GO:0006355,GO:GO:0005739,GO:GO:0003690	AT5G07900.1	Mitochondrial transcription termination factor family protein	LOC_Os06g12100.1	mTERF family protein, expressed	2,05	-1.0	1	21	692	712	UGAACAUCCCAGAGCCACCGG	CCCGUGGCUCUGGAAUGUUCA	Cleavage
tae-miR9662a-3p	Traes_4AL_A4B73CC6A1.2	PF02536	KOG1267		K15032	GO:GO:0006355,GO:GO:0005739,GO:GO:0003690	AT5G07900.1	Mitochondrial transcription termination factor family protein	LOC_Os06g12100.1	mTERF family protein, expressed	2,05	-1.0	1	21	693	713	UUGAACAUCCCAGAGCCACCG	CCGUGGCUCUGGAAUGUUCAG	Cleavage
tae-miR9662b-3p	Traes_4AL_A4B73CC6A1.2	PF02536	KOG1267		K15032	GO:GO:0006355,GO:GO:0005739,GO:GO:0003690	AT5G07900.1	Mitochondrial transcription termination factor family protein	LOC_Os06g12100.1	mTERF family protein, expressed	2,05	-1.0	1	21	692	712	UGAACAUCCCAGAGCCACCGG	CCCGUGGCUCUGGAAUGUUCA	Cleavage
tae-miR1130b-3p	Traes_4AL_C55D89913.1										0,00	-1.0	1	21	10	30	UCUAAUAUUAUGGGACGGAGG	CCUCCGUCCCAUAAUAUAAGA	Cleavage
tae-miR1122c-3p	Traes_4AL_C55D89913.1										1,05	-1.0	1	21	10	30	UCUAAUAUUAUGGGACGGAGG	CCUCCGUCCCAUAAUAUAAGA	Cleavage
tae-miR1130b-3p	Traes_4AL_C55D89913.5										0,00	-1.0	1	21	10	30	UCUAAUAUUAUGGGACGGAGG	CCUCCGUCCCAUAAUAUAAGA	Cleavage
tae-miR1122c-3p	Traes_4AL_C55D89913.5										1,05	-1.0	1	21	10	30	UCUAAUAUUAUGGGACGGAGG	CCUCCGUCCCAUAAUAUAAGA	Cleavage
tae-miR1130b-3p	Traes_4AL_C55D89913.6										0,00	-1.0	1	21	10	30	UCUAAUAUUAUGGGACGGAGG	CCUCCGUCCCAUAAUAUAAGA	Cleavage
tae-miR1122c-3p	Traes_4AL_C55D89913.6										1,05	-1.0	1	21	10	30	UCUAAUAUUAUGGGACGGAGG	CCUCCGUCCCAUAAUAUAAGA	Cleavage
tae-miR1130b-3p	Traes_4AL_C55D89913.7										0,00	-1.0	1	21	10	30	UCUAAUAUUAUGGGACGGAGG	CCUCCGUCCCAUAAUAUAAGA	Cleavage
tae-miR1122c-3p	Traes_4AL_C55D89913.7										1,05	-1.0	1	21	10	30	UCUAAUAUUAUGGGACGGAGG	CCUCCGUCCCAUAAUAUAAGA	Cleavage
tae-miR1130b-3p	Traes_4AL_C55D89913.8										0,00	-1.0	1	21	10	30	UCUAAUAUUAUGGGACGGAGG	CCUCCGUCCCAUAAUAUAAGA	Cleavage
tae-miR1122c-3p	Traes_4AL_C55D89913.8										1,05	-1.0	1	21	10	30	UCUAAUAUUAUGGGACGGAGG	CCUCCGUCCCAUAAUAUAAGA	Cleavage
tae-miR5049-3p	Traes_4AL_C9E08C423.5										0,00	-1.0	1	21	69	89	AAUAUGGAUCGGAGGGAGUAC	CUACUCCCUCCGAUCCAUAUU	Cleavage
tae-miR9782	Traes_4AL_D576D98B4.1										1,00	-1.0	1	24	2	25	GUAUUAGGUUGGUCAAAUUGACGA	UCGUCAAUUUGACCAACCUAAUAU	Cleavage
tae-miR9782	Traes_4AL_D576D98B4.2										1,00	-1.0	1	24	2	25	GUAUUAGGUUGGUCAAAUUGACGA	UCGUCAAUUUGACCAACCUAAUAU	Cleavage
tae-miR9661-5p	Traes_4AL_D8CCE0D2E.1										2,00	-1.0	1	21	647	667	UGAAGUAGAGCAGGGACCUCA	GGAGGUCUCUGCUCUACUUCC	Cleavage
tae-miR9661-5p	Traes_4AL_D8CCE0D2E.2										2,00	-1.0	1	21	644	664	UGAAGUAGAGCAGGGACCUCA	GGAGGUCUCUGCUCUACUUCC	Cleavage
tae-miR9661-5p	Traes_4AL_D8CCE0D2E.3										2,00	-1.0	1	21	620	640	UGAAGUAGAGCAGGGACCUCA	GGAGGUCUCUGCUCUACUUCC	Cleavage
tae-miR9661-5p	Traes_4AL_D8CCE0D2E.4										2,00	-1.0	1	21	587	607	UGAAGUAGAGCAGGGACCUCA	GGAGGUCUCUGCUCUACUUCC	Cleavage
tae-miR5384-3p	Traes_4AL_E60E5B7BA.1										2,05	-1.0	1	21	269	289	UGAGCGCGCCGCCGUCGAAUG	UGUUCCACGGCGGCGCGCACA	Cleavage
tae-miR5384-3p	Traes_4AL_E60E5B7BA.2										2,05	-1.0	1	21	140	160	UGAGCGCGCCGCCGUCGAAUG	UGUUCCACGGCGGCGCGCACA	Cleavage
tae-miR1130b-3p	Traes_4AS_0E2735576.2										1,05	-1.0	1	21	977	997	UCUUAUAUUAUGGGACGGAGG	CCUCCGUCUCAUAAUAUAAGA	Cleavage
tae-miR1120c-5p	Traes_4AS_0E2735576.2										0,00	-1.0	1	21	1020	1040	UAAUAUAAGAACGUUUUUGAC	GACAAAAACGUUCUUAUAUUA	Cleavage
tae-miR9674a-5p	Traes_4AS_AB9088E9B.2										2,05	-1.0	1	21	1383	1403	GCAUCAUCCAUCCUACCAUUC	UGAUGGUCAGAUGGAUGAUGC	Cleavage
tae-miR9674a-5p	Traes_4AS_AB9088E9B.3										2,05	-1.0	1	21	1383	1403	GCAUCAUCCAUCCUACCAUUC	UGAUGGUCAGAUGGAUGAUGC	Cleavage
tae-miR5384-3p	Traes_4AS_C7CBD4A47.1										2,05	-1.0	1	21	194	214	UGAGCGCGCCGCCGUCGAAUG	UCUUCGACGGCGGCGCGCACU	Cleavage
tae-miR1135	Traes_4AS_D533B306B.3	PF01485	KOG1815	6.3.2.19		GO:GO:0016567,GO:GO:0004842	AT2G16090.1	RING/U-box superfamily protein	LOC_Os03g13100.1	ariadne-1, putative, expressed	2,00	-1.0	1	24	1698	1721	CUGCGACAAGUAAUUCCGAACGGA	CGAACCAGGAAUUACUUGUCGCAG	Cleavage
tae-miR1131	Traes_4BL_178D6FFF1.1										1,05	-1.0	1	22	167	188	UAGUACCGGUUCGUGGCUAACC	GGUUAGCCACGAACCGAUACUA	Cleavage
tae-miR1131	Traes_4BL_178D6FFF1.2										1,05	-1.0	1	22	167	188	UAGUACCGGUUCGUGGCUAACC	GGUUAGCCACGAACCGAUACUA	Cleavage
tae-miR9662a-3p	Traes_4BL_29D8171E0.1								LOC_Os06g12100.1	mTERF family protein, expressed	2,00	-1.0	1	21	132	152	UUGAACAUCCCAGAGCCACCG	UCGUGGCGCUGGGAUGUUCAG	Cleavage
tae-miR9662b-3p	Traes_4BL_29D8171E0.1								LOC_Os06g12100.1	mTERF family protein, expressed	2,05	-1.0	1	21	131	151	UGAACAUCCCAGAGCCACCGG	CUCGUGGCGCUGGGAUGUUCA	Cleavage
tae-miR1122b-3p	Traes_4BL_2D34597CF.2										2,05	-1.0	1	21	454	474	AGACUUAUAUGUAGGAACGGA	UCUGUUCCUAAAUAUAAGUCU	Translation
tae-miR1122b-3p	Traes_4BL_2D34597CF.3										2,05	-1.0	1	21	454	474	AGACUUAUAUGUAGGAACGGA	UCUGUUCCUAAAUAUAAGUCU	Translation
tae-miR1128	Traes_4BL_37FDD905B.1										2,05	-1.0	1	21	125	145	UACUACUCCCUCCGUCCGAAA	UUUGGGACGGAGGGAGUAGGA	Cleavage
tae-miR5384-3p	Traes_4BL_3B7A98FD6.1										2,05	-1.0	1	21	191	211	UGAGCGCGCCGCCGUCGAAUG	UCUUCGACGGCGGCGCGCACU	Cleavage
tae-miR9666a-3p	Traes_4BL_44010E331.1										3,00	-1.0	1	22	176	197	CGGUAGGGCUGUAUGAUGGCGA	UCGGCAUGAUACAGCCCUACCU	Cleavage
tae-miR1122b-3p	Traes_4BL_62355968B.1										2,05	-1.0	1	21	706	726	AGACUUAUAUGUAGGAACGGA	UCUGUUCCUAAAUAUAAGUCU	Translation
tae-miR1122a	Traes_4BL_62355968B.1										2,05	-1.0	1	20	747	766	UAGAUACAUCCGUAUCUAGA	ACUACAUACGGAUGUAUAUA	Cleavage
tae-miR1137a	Traes_4BL_D08D5FFB5.2										2,00	-1.0	1	20	722	741	UAGUACAAAGUUGAGUCAUC	AGUGUCUCAACUUUGUACUA	Cleavage
tae-miR1137a	Traes_4BL_F3D5D7034.2										1,00	-1.0	1	19	1	19	UAGUACAAAGUUGAGUCAU	AUGACCCAACUUUGUACUA	Cleavage
tae-miR1137b-5p	Traes_4BL_F3D5D7034.2										1,05	-1.0	1	21	37	57	UCCGUUCCAGAAUAGAUGACC	GGUCAUCUAUUUUGGAACGGA	Translation
tae-miR9662a-3p	Traes_4BS_257C5B099.1	PF02536				GO:GO:0006355,GO:GO:0005739,GO:GO:0003690	AT5G07900.1	Mitochondrial transcription termination factor family protein	LOC_Os06g12100.1	mTERF family protein, expressed	2,00	-1.0	1	21	66	86	UUGAACAUCCCAGAGCCACCG	CCGUGGUUCUGGGAUGUUCAG	Cleavage
tae-miR9662b-3p	Traes_4BS_257C5B099.1	PF02536				GO:GO:0006355,GO:GO:0005739,GO:GO:0003690	AT5G07900.1	Mitochondrial transcription termination factor family protein	LOC_Os06g12100.1	mTERF family protein, expressed	2,00	-1.0	1	21	65	85	UGAACAUCCCAGAGCCACCGG	CCCGUGGUUCUGGGAUGUUCA	Cleavage
tae-miR156	Traes_4BS_375EF1D15.1								LOC_Os01g69830.1	OsSPL2 - SBP-box gene family member, expressed	2,05	-1.0	1	21	235	255	UGACAGAAGAGAGUGAGCACA	UGUGCUCUCUCUUUUCUGUCA	Cleavage
tae-miR1118	Traes_4BS_4BC02509F.1										2,05	-1.0	1	23	525	547	CACUACAUUAUGGAAUGGAGGGA	UCCCUCCGUUCCAUAAUGUAAUG	Cleavage
tae-miR9666a-3p	Traes_4BS_5419B1DE7.1										3,00	-1.0	1	22	176	197	CGGUAGGGCUGUAUGAUGGCGA	UCGGCAUGAUACAGCCCUACCU	Cleavage
tae-miR9661-5p	Traes_4BS_5EC01BC81.1										2,00	-1.0	1	21	653	673	UGAAGUAGAGCAGGGACCUCA	GGAGGUCUCUGCUCUACUUCC	Cleavage
tae-miR9661-5p	Traes_4BS_5EC01BC81.2										2,00	-1.0	1	21	653	673	UGAAGUAGAGCAGGGACCUCA	GGAGGUCUCUGCUCUACUUCC	Cleavage
tae-miR9661-5p	Traes_4BS_5EC01BC81.3										2,00	-1.0	1	21	653	673	UGAAGUAGAGCAGGGACCUCA	GGAGGUCUCUGCUCUACUUCC	Cleavage
tae-miR1122b-3p	Traes_4BS_6252B0BA0.1										1,05	-1.0	1	21	46	66	AGACUUAUAUGUAGGAACGGA	UCCGUUCCUAAAUAUAAGUCU	Translation
tae-miR7757-5p	Traes_4BS_B45729F4D.4	PF00931				GO:GO:0043531	AT3G07040.1	NB-ARC domain-containing disease resistance protein	LOC_Os11g11810.1	NBS-LRR disease resistance protein, putative, expressed	2,05	-1.0	1	22	653	674	AUAAAACCUUCAGCUAUCCAUC	AAUGGAUAUCUGAAGGUUUCAU	Cleavage
tae-miR5384-3p	Traes_4BS_F1D7488B8.1										2,05	-1.0	1	21	269	289	UGAGCGCGCCGCCGUCGAAUG	UGUUCCACGGCGGCGCGCACA	Cleavage
tae-miR1122a	Traes_4BS_F395A220A.2										0,00	-1.0	1	20	1336	1355	UAGAUACAUCCGUAUCUAGA	UCUAGAUACGGAUGUAUCUA	Cleavage
tae-miR7757-5p	Traes_4DL_113F6DD2B.1	PF00931				GO:GO:0043531	AT1G50180.1	NB-ARC domain-containing disease resistance protein	LOC_Os12g13550.1	NBS-LRR disease resistance protein, putative, expressed	2,05	-1.0	1	22	215	236	AUAAAACCUUCAGCUAUCCAUC	AAUGGAUGGCUGAAGGCUUUAU	Cleavage
tae-miR7757-5p	Traes_4DL_113F6DD2B.2	PF00931				GO:GO:0043531	AT1G50180.1	NB-ARC domain-containing disease resistance protein	LOC_Os10g22484.2	RGH2B, putative, expressed	2,05	-1.0	1	22	215	236	AUAAAACCUUCAGCUAUCCAUC	AAUGGAUGGCUGAAGGCUUUAU	Cleavage
tae-miR5085	Traes_4DL_341830EFF.1										2,05	-1.0	1	21	583	603	AAGGACAUUUUUUGUGGCCUG	GAGGACACAAAAAAUGUCGUU	Cleavage
tae-miR1133	Traes_4DL_529524815.5										2,00	-1.0	1	22	103	127	CAUAUACUCCCUCCG-UCCGA--AA	UUAGUCGGAUCGGAGGGAGUAUAUG	Cleavage
tae-miR1130b-3p	Traes_4DL_84745919A.2										2,05	-1.0	1	21	2964	2984	UCUUAUAUUAUGGGACGGAGG	CCUCUGUCCCAUAAUAUAGGA	Cleavage
tae-miR1130a	Traes_4DL_84745919A.2										1,05	-1.0	1	23	3016	3038	CCUCCGUCUCGUAAUGUAAGACG	UGUCUUACAUUAUGAGACGGAGG	Translation
tae-miR1128	Traes_4DL_93D4DED90.3										3,00	-1.0	1	21	565	586	UACUACUCCCUCCG-UCCGAAA	UUUUGGAACGGAGGGAGUAGUA	Cleavage
tae-miR1137a	Traes_4DL_93D4DED90.3										2,05	-1.0	1	20	508	527	UAGUACAAAGUUGAGUCAUC	GAUGACCCAACUUUGUAUUA	Cleavage
tae-miR1137b-5p	Traes_4DL_93D4DED90.3										1,05	-1.0	1	21	556	576	UCCGUUCCAGAAUAGAUGACC	GGUCAUCUAUUUUGGAACGGA	Translation
tae-miR1137b-5p	Traes_4DS_28E6C4F21.2										1,05	-1.0	1	21	61	81	UCCGUUCCAGAAUAGAUGACC	AGUCAUCUAUUUUGGAACGGA	Translation
tae-miR1137b-5p	Traes_4DS_28E6C4F21.3										1,05	-1.0	1	21	61	81	UCCGUUCCAGAAUAGAUGACC	AGUCAUCUAUUUUGGAACGGA	Translation
tae-miR1137b-5p	Traes_4DS_28E6C4F21.4										1,05	-1.0	1	21	61	81	UCCGUUCCAGAAUAGAUGACC	AGUCAUCUAUUUUGGAACGGA	Translation
tae-miR7757-5p	Traes_4DS_5B29EE3DD.5	PF00931				GO:GO:0043531	AT3G50950.1	HOPZ-ACTIVATED RESISTANCE 1	LOC_Os11g11810.1	NBS-LRR disease resistance protein, putative, expressed	2,05	-1.0	1	22	1379	1400	AUAAAACCUUCAGCUAUCCAUC	AAUGGAUAUCUGAAGGUUUCAU	Cleavage
tae-miR7757-5p	Traes_4DS_5B29EE3DD.6	PF00931				GO:GO:0043531	AT3G50950.1	HOPZ-ACTIVATED RESISTANCE 1	LOC_Os11g11810.1	NBS-LRR disease resistance protein, putative, expressed	2,05	-1.0	1	22	1379	1400	AUAAAACCUUCAGCUAUCCAUC	AAUGGAUAUCUGAAGGUUUCAU	Cleavage
tae-miR7757-5p	Traes_4DS_5B29EE3DD.7	PF00931				GO:GO:0043531	AT1G58390.1	Disease resistance protein (CC-NBS-LRR class) family	LOC_Os11g11810.1	NBS-LRR disease resistance protein, putative, expressed	2,05	-1.0	1	22	824	845	AUAAAACCUUCAGCUAUCCAUC	AAUGGAUAUCUGAAGGUUUCAU	Cleavage
tae-miR1130b-3p	Traes_4DS_9179FF158.4										1,05	-1.0	1	21	41	61	UCUUAUAUUAUGGGACGGAGG	CCUCCGUCCCAUAAUGUAAGA	Cleavage
tae-miR9778	Traes_4DS_B08D83A2F.1	PF00931				GO:GO:0043531	AT3G46530.1	NB-ARC domain-containing disease resistance protein	LOC_Os11g39550.1	Leucine Rich Repeat family protein, expressed	2,05	-1.0	1	21	126	146	UGCAUCAUCUCGAACUCGUCG	UGGCGAGUUCCAGAUGAUGCA	Translation
tae-miR164	Traes_4DS_C08BF9DB2.1										2,05	-1.0	1	21	387	407	UGGAGAAGCAGGGCACGUGCA	UUCACGUGCCCUGCUUUUCCU	Cleavage
tae-miR1128	Traes_4DS_C22A101FE.2										2,05	-1.0	1	21	670	690	UACUACUCCCUCCGUCCGAAA	UUUGGGACGAAGGGAGUAGUA	Cleavage
tae-miR1128	Traes_4DS_C22A101FE.9										2,05	-1.0	1	21	358	378	UACUACUCCCUCCGUCCGAAA	UUUGGGACGAAGGGAGUAGUA	Cleavage
tae-miR1122a	Traes_4DS_E8239194E.2										2,05	-1.0	1	20	692	711	UAGAUACAUCCGUAUCUAGA	UCUAAAUACGGAUGUAUCAA	Cleavage
tae-miR5384-3p	Traes_4DS_ECFDD82BA.1										2,05	-1.0	1	21	269	289	UGAGCGCGCCGCCGUCGAAUG	UGUUCCACGGCGGCGCGCACA	Cleavage
tae-miR1137b-5p	Traes_4DS_EFE0451D7.3										1,05	-1.0	1	21	950	970	UCCGUUCCAGAAUAGAUGACC	AGUCAUCUAUUUUGGAACGGA	Translation
tae-miR1128	Traes_5AL_3AB81DF9B.1										3,00	-1.0	1	21	151	172	UACUACUCCCUCCG-UCCGAAA	UUUAGGAACGGAGGGAGUAGUA	Cleavage
tae-miR9652-5p	Traes_5AL_481F73E59.1	PF00931				GO:GO:0043531	AT3G46710.1	NB-ARC domain-containing disease resistance protein	LOC_Os05g40150.1	RGH2B, putative, expressed	2,05	-1.0	1	22	883	904	CCUGUUUGUCAUUAAGUUUCUU	AAGAAACUGAAUGACAAGCAGG	Cleavage
tae-miR9652-5p	Traes_5AL_481F73E59.2	PF03106,PF12796,PF00931	KOG4658			GO:GO:0043565,GO:GO:0006355,GO:GO:0003700,GO:GO:0043531	AT2G31820.1	Ankyrin repeat family protein	LOC_Os11g45750.2	WRKY125, expressed	2,05	-1.0	1	22	883	904	CCUGUUUGUCAUUAAGUUUCUU	AAGAAACUGAAUGACAAGCAGG	Cleavage
tae-miR9652-5p	Traes_5AL_481F73E59.3	PF03106,PF12796,PF00931	KOG4658			GO:GO:0043565,GO:GO:0006355,GO:GO:0003700,GO:GO:0043531	AT2G31820.1	Ankyrin repeat family protein	LOC_Os11g45750.2	WRKY125, expressed	2,05	-1.0	1	22	883	904	CCUGUUUGUCAUUAAGUUUCUU	AAGAAACUGAAUGACAAGCAGG	Cleavage
tae-miR9652-5p	Traes_5AL_481F73E59.4	PF03106,PF12796,PF00931	KOG4658			GO:GO:0043565,GO:GO:0006355,GO:GO:0003700,GO:GO:0043531	AT2G31820.1	Ankyrin repeat family protein	LOC_Os11g45750.2	WRKY125, expressed	2,05	-1.0	1	22	883	904	CCUGUUUGUCAUUAAGUUUCUU	AAGAAACUGAAUGACAAGCAGG	Cleavage
tae-miR9652-5p	Traes_5AL_481F73E59.5	PF03106,PF12796,PF00931	KOG4658			GO:GO:0043565,GO:GO:0006355,GO:GO:0003700,GO:GO:0043531	AT2G31820.1	Ankyrin repeat family protein	LOC_Os11g45750.1	WRKY125, expressed	2,05	-1.0	1	22	583	604	CCUGUUUGUCAUUAAGUUUCUU	AAGAAACUGAAUGACAAGCAGG	Cleavage
tae-miR9652-5p	Traes_5AL_481F73E59.6	PF00931,PF03106				GO:GO:0043531,GO:GO:0043565,GO:GO:0006355,GO:GO:0003700	AT3G46710.1	NB-ARC domain-containing disease resistance protein	LOC_Os05g40150.1	RGH2B, putative, expressed	2,05	-1.0	1	22	883	904	CCUGUUUGUCAUUAAGUUUCUU	AAGAAACUGAAUGACAAGCAGG	Cleavage
tae-miR1127b-3p	Traes_5AL_7256A9F19.2										1,05	-1.0	1	21	991	1011	ACAAGUAUUUCUGGACGGAGG	CCUCCGUCCGGAAAUACUUGU	Cleavage
tae-miR1122b-3p	Traes_5AL_7FF780810.1										1,05	-1.0	1	21	22	42	AGACUUAUAUGUAGGAACGGA	UCCGUUCCUAAAUAUAAGUCU	Translation
tae-miR7757-5p	Traes_5AL_B4E8A3115.1										1,05	-1.0	1	22	368	389	AUAAAACCUUCAGCUAUCCAUC	AAUGGAUAGCUGAAGGUCUUAU	Cleavage
tae-miR7757-5p	Traes_5AL_B4E8A3115.2										1,05	-1.0	1	22	2324	2345	AUAAAACCUUCAGCUAUCCAUC	AAUGGAUAGCUGAAGGUCUUAU	Cleavage
tae-miR7757-5p	Traes_5AL_B4E8A3115.3										1,05	-1.0	1	22	2324	2345	AUAAAACCUUCAGCUAUCCAUC	AAUGGAUAGCUGAAGGUCUUAU	Cleavage
tae-miR7757-5p	Traes_5AL_B4E8A3115.4										1,05	-1.0	1	22	2324	2345	AUAAAACCUUCAGCUAUCCAUC	AAUGGAUAGCUGAAGGUCUUAU	Cleavage
tae-miR7757-5p	Traes_5AL_B4E8A3115.5										1,05	-1.0	1	22	2324	2345	AUAAAACCUUCAGCUAUCCAUC	AAUGGAUAGCUGAAGGUCUUAU	Cleavage
tae-miR7757-5p	Traes_5AL_B4E8A3115.6										1,05	-1.0	1	22	368	389	AUAAAACCUUCAGCUAUCCAUC	AAUGGAUAGCUGAAGGUCUUAU	Cleavage
tae-miR9652-5p	Traes_5AL_C9236F111.1										2,05	-1.0	1	22	778	799	CCUGUUUGUCAUUAAGUUUCUU	AAGAAACUGAAUGACAAGCAGG	Cleavage
tae-miR5384-3p	Traes_5AS_A928BA201.1										2,05	-1.0	1	21	506	526	UGAGCGCGCCGCCGUCGAAUG	CAUUCGUCGGCGUCGCGCUCA	Cleavage
tae-miR7757-5p	Traes_5AS_BDFB08E23.1	PF00931	KOG4658			GO:GO:0043531	AT3G07040.1	NB-ARC domain-containing disease resistance protein	LOC_Os12g37740.1	resistance protein LR10, putative, expressed	2,05	-1.0	1	22	1349	1370	AUAAAACCUUCAGCUAUCCAUC	GAUGGAUUGCUGAAGGUUUCAU	Cleavage
tae-miR7757-5p	Traes_5AS_BDFB08E23.2	PF00931	KOG4658			GO:GO:0043531	AT3G07040.1	NB-ARC domain-containing disease resistance protein	LOC_Os12g37740.1	resistance protein LR10, putative, expressed	2,05	-1.0	1	22	1349	1370	AUAAAACCUUCAGCUAUCCAUC	GAUGGAUUGCUGAAGGUUUCAU	Cleavage
tae-miR7757-5p	Traes_5AS_BDFB08E23.3	PF00931				GO:GO:0043531	AT3G07040.1	NB-ARC domain-containing disease resistance protein	LOC_Os12g37740.1	resistance protein LR10, putative, expressed	2,05	-1.0	1	22	524	545	AUAAAACCUUCAGCUAUCCAUC	GAUGGAUUGCUGAAGGUUUCAU	Cleavage
tae-miR7757-5p	Traes_5AS_BDFB08E23.4	PF00931				GO:GO:0043531	AT3G46530.1	NB-ARC domain-containing disease resistance protein	LOC_Os12g37740.1	resistance protein LR10, putative, expressed	2,05	-1.0	1	22	1349	1370	AUAAAACCUUCAGCUAUCCAUC	GAUGGAUUGCUGAAGGUUUCAU	Cleavage
tae-miR7757-5p	Traes_5AS_BDFB08E23.5	PF00931				GO:GO:0043531	AT3G46530.1	NB-ARC domain-containing disease resistance protein	LOC_Os12g37740.1	resistance protein LR10, putative, expressed	2,05	-1.0	1	22	1349	1370	AUAAAACCUUCAGCUAUCCAUC	GAUGGAUUGCUGAAGGUUUCAU	Cleavage
tae-miR1128	Traes_5AS_E1F692526.2										3,00	-1.0	1	21	111	132	UACUACUCCCUCCGUCCGA-AA	UUAUGGGACGGAGGGAGUAGUA	Cleavage
tae-miR1130b-3p	Traes_5AS_E1F692526.2										3,00	-1.0	1	21	40	61	UCUU-AUAUUAUGGGACGGAGG	CCUCCGUCCCAUAAUAUUAAGA	Cleavage
tae-miR1122c-3p	Traes_5AS_E1F692526.2										3,00	-1.0	1	21	40	61	UC-UAAUAUUAUGGGACGGAGG	CCUCCGUCCCAUAAUAUUAAGA	Cleavage
tae-miR164	Traes_5AS_E5FD82E36.1										1,00	-1.0	1	21	653	673	UGGAGAAGCAGGGCACGUGCA	AGCAAGUGCCCUGCUUCUCCA	Cleavage
tae-miR164	Traes_5AS_E5FD82E36.2										1,00	-1.0	1	21	656	676	UGGAGAAGCAGGGCACGUGCA	AGCAAGUGCCCUGCUUCUCCA	Cleavage
tae-miR1118	Traes_5AS_EC41EF7CA.4										1,05	-1.0	1	23	1333	1355	CACUACAUUAUGGAAUGGAGGGA	UCCCUCCAUUCCACAAUGUAGUG	Translation
tae-miR1125	Traes_5AS_EC41EF7CA.4										1,00	-1.0	1	24	1450	1473	AACCAACGAGACCAACUGCGGCGG	CCGCCGCAGUCGGUCUCGUUGGUU	Cleavage
tae-miR408	Traes_5BL_0212ACBD9.1										2,05	-1.0	1	21	2512	2532	CUGCACUGCCUCUUCCCUGGC	GCCAAGGAAGAGGCUGUGCAG	Cleavage
tae-miR1137a	Traes_5BL_0EC4788EF.2										1,00	-1.0	1	20	1543	1562	UAGUACAAAGUUGAGUCAUC	GAUGACCCAACUUUGUACUA	Cleavage

tae-miR9652-5p	Traes_5BL_0F64FDA44.1										2,05	-1.0	1	22	583	604	CCUGUUUGUCAUUAAGUUUCUU	AAGAAACUGAAUGACAAGCAGG	Cleavage
tae-miR1122a	Traes_5BL_1B9186ED8.1										2,05	-1.0	1	20	986	1005	UAGAUACAUCCGUAUCUAGA	UCGAGAUACAGAUGUAUCUA	Translation
tae-miR7757-5p	Traes_5BL_1E751EF1F.1	PF00931,PF03514	KOG4658			GO:GO:0043531	AT1G50600.1	scarecrow-like 5	LOC_Os10g22484.1	RGH2B, putative, expressed	2,00	-1.0	1	22	1799	1820	AUAAAACCUUCAGCUAUCCAUC	AAUGGGUUGCUGAAGGUUUUAU	Cleavage
tae-miR7757-5p	Traes_5BL_1E751EF1F.2	PF00931				GO:GO:0043531	AT3G46730.1	NB-ARC domain-containing disease resistance protein	LOC_Os10g22484.1	RGH2B, putative, expressed	2,00	-1.0	1	22	1799	1820	AUAAAACCUUCAGCUAUCCAUC	AAUGGGUUGCUGAAGGUUUUAU	Cleavage
tae-miR7757-5p	Traes_5BL_1E751EF1F.5	PF00931,PF03514				GO:GO:0043531	AT1G50600.1	scarecrow-like 5	LOC_Os10g22484.1	RGH2B, putative, expressed	2,00	-1.0	1	22	512	533	AUAAAACCUUCAGCUAUCCAUC	AAUGGGUUGCUGAAGGUUUUAU	Cleavage
tae-miR5049-3p	Traes_5BL_4BFE76791.5										2,05	-1.0	1	21	416	436	AAUAUGGAUCGGAGGGAGUAC	AUGCUCCCUCUGAUCCAUAUU	Translation
tae-miR1127b-3p	Traes_5BL_5E94C243F.1										2,05	-1.0	1	21	914	934	ACAAGUAUUUCUGGACGGAGG	CCUCUGUCCUGAAAUACUUGU	Cleavage
tae-miR1127b-3p	Traes_5BL_5E94C243F.2										2,05	-1.0	1	21	898	918	ACAAGUAUUUCUGGACGGAGG	CCUCUGUCCUGAAAUACUUGU	Cleavage
tae-miR9674b-5p	Traes_5BL_6216A6108.1										3,00	-1.0	1	21	411	431	AUAGCAUCAUCCAUCCUACCC	GGGCCGAAUGGAUGAUGCUAU	Cleavage
tae-miR9674b-5p	Traes_5BL_6216A6108.1										3,00	-1.0	1	21	411	431	AUAGCAUCAUCCAUCCUACCC	GGGCCGAAUGGAUGAUGCUAU	Cleavage
tae-miR5384-3p	Traes_5BL_6240DE6BA.1										2,05	-1.0	1	21	338	358	UGAGCGCGCCGCCGUCGAAUG	CCUUCGACGGCGCCGCGCUCG	Cleavage
tae-miR5384-3p	Traes_5BL_6240DE6BA.2										2,05	-1.0	1	21	338	358	UGAGCGCGCCGCCGUCGAAUG	CCUUCGACGGCGCCGCGCUCG	Cleavage
tae-miR5384-3p	Traes_5BL_6240DE6BA.3										2,05	-1.0	1	21	338	358	UGAGCGCGCCGCCGUCGAAUG	CCUUCGACGGCGCCGCGCUCG	Cleavage
tae-miR5384-3p	Traes_5BL_6240DE6BA.4										2,05	-1.0	1	21	338	358	UGAGCGCGCCGCCGUCGAAUG	CCUUCGACGGCGCCGCGCUCG	Cleavage
tae-miR1122a	Traes_5BL_637A3C970.8										1,05	-1.0	1	20	49	68	UAGAUACAUCCGUAUCUAGA	UCUAGAUACGAAUGUAUCUA	Translation
tae-miR1121	Traes_5BL_72EFF0AF0.1										0,00	-1.0	1	22	1237	1258	AGUAGUGAUCUAAACGCUCUUA	UAAGAGCGUUUAGAUCACUACU	Cleavage
tae-miR408	Traes_5BL_7486582EB.1										2,05	-1.0	1	21	8	28	CUGCACUGCCUCUUCCCUGGC	CGCAGGGAAGAGGCAGUGCCA	Cleavage
tae-miR1122a	Traes_5BL_7821274AB.1										2,05	-1.0	1	20	986	1005	UAGAUACAUCCGUAUCUAGA	UCGAGAUACAGAUGUAUCUA	Translation
tae-miR7757-5p	Traes_5BL_7F0FD1538.2	PF00847,PF00931	KOG4658			GO:GO:0006355,GO:GO:0003700,GO:GO:0043531	AT3G46710.1	NB-ARC domain-containing disease resistance protein	LOC_Os05g40150.1	RGH2B, putative, expressed	1,05	-1.0	1	22	2096	2117	AUAAAACCUUCAGCUAUCCAUC	AAUGGAUAGCUGAAAGUUUUAU	Cleavage
tae-miR7757-5p	Traes_5BL_7F0FD1538.3	PF00847,PF00931	KOG4658			GO:GO:0006355,GO:GO:0003700,GO:GO:0043531	AT3G46710.1	NB-ARC domain-containing disease resistance protein	LOC_Os05g40150.1	RGH2B, putative, expressed	1,05	-1.0	1	22	1379	1400	AUAAAACCUUCAGCUAUCCAUC	AAUGGAUAGCUGAAAGUUUUAU	Cleavage
tae-miR7757-5p	Traes_5BL_7F0FD1538.4	PF00931	KOG4658			GO:GO:0043531	AT3G46710.1	NB-ARC domain-containing disease resistance protein	LOC_Os05g40150.1	RGH2B, putative, expressed	1,05	-1.0	1	22	1379	1400	AUAAAACCUUCAGCUAUCCAUC	AAUGGAUAGCUGAAAGUUUUAU	Cleavage
tae-miR156	Traes_5BL_96D5EA4EE.1	PF03110				GO:GO:0005634,GO:GO:0003677	AT2G42200.1	squamosa promoter binding protein-like 9	LOC_Os09g31438.1	OsSPL17 - SBP-box gene family member, expressed	1,00	-1.0	1	21	805	825	UGACAGAAGAGAGUGAGCACA	UGUGCUCUCUCUCUUCUGUCA	Cleavage
tae-miR156	Traes_5BL_96D5EA4EE.2	PF03110				GO:GO:0005634,GO:GO:0003677	AT2G42200.1	squamosa promoter binding protein-like 9	LOC_Os09g31438.1	OsSPL17 - SBP-box gene family member, expressed	1,00	-1.0	1	21	802	822	UGACAGAAGAGAGUGAGCACA	UGUGCUCUCUCUCUUCUGUCA	Cleavage
tae-miR156	Traes_5BL_96D5EA4EE.3								LOC_Os09g31438.1	OsSPL17 - SBP-box gene family member, expressed	1,00	-1.0	1	21	67	87	UGACAGAAGAGAGUGAGCACA	UGUGCUCUCUCUCUUCUGUCA	Cleavage
tae-miR156	Traes_5BL_96FDFC152.1	PF03110				GO:GO:0005634,GO:GO:0003677	AT1G76580.1	Squamosa promoter-binding protein-like (SBP domain) transcription factor family protein	LOC_Os09g32944.1	OsSPL18 - SBP-box gene family member, expressed	1,00	-1.0	1	21	508	528	UGACAGAAGAGAGUGAGCACA	UGUGCUCUCUCUCUUCUGUCA	Cleavage
tae-miR156	Traes_5BL_96FDFC152.2	PF03110				GO:GO:0005634,GO:GO:0003677	AT5G50570.1	Squamosa promoter-binding protein-like (SBP domain) transcription factor family protein	LOC_Os09g32944.1	OsSPL18 - SBP-box gene family member, expressed	1,00	-1.0	1	21	490	510	UGACAGAAGAGAGUGAGCACA	UGUGCUCUCUCUCUUCUGUCA	Cleavage
tae-miR5384-3p	Traes_5BL_AD734CCEF.1										2,05	-1.0	1	21	242	262	UGAGCGCGCCGCCGUCGAAUG	UCUUCCACGGCGGCGCGUUCA	Cleavage
tae-miR9664-3p	Traes_5BL_D27214492.1	PF00931				GO:GO:0043531	AT3G46530.1	NB-ARC domain-containing disease resistance protein	LOC_Os11g11550.1	NBS-LRR disease resistance protein, putative, expressed	2,05	-1.0	1	21	237	257	UUGCAGUCCUCGAUGUCGUAG	GCACGACAUCGAGGAUUGCAU	Cleavage
tae-miR9664-3p	Traes_5BL_D27214492.2	PF00931				GO:GO:0043531	AT3G46530.1	NB-ARC domain-containing disease resistance protein	LOC_Os11g11550.2	NBS-LRR disease resistance protein, putative, expressed	2,05	-1.0	1	21	237	257	UUGCAGUCCUCGAUGUCGUAG	GCACGACAUCGAGGAUUGCAU	Cleavage
tae-miR9664-3p	Traes_5BL_D27214492.3	PF00931				GO:GO:0043531	AT3G46530.1	NB-ARC domain-containing disease resistance protein	LOC_Os11g11550.2	NBS-LRR disease resistance protein, putative, expressed	2,05	-1.0	1	21	237	257	UUGCAGUCCUCGAUGUCGUAG	GCACGACAUCGAGGAUUGCAU	Cleavage
tae-miR9664-3p	Traes_5BL_D27214492.4	PF00931				GO:GO:0043531	AT3G46530.1	NB-ARC domain-containing disease resistance protein	LOC_Os11g11550.2	NBS-LRR disease resistance protein, putative, expressed	2,05	-1.0	1	21	237	257	UUGCAGUCCUCGAUGUCGUAG	GCACGACAUCGAGGAUUGCAU	Cleavage
tae-miR9664-3p	Traes_5BL_D27214492.5	PF00931				GO:GO:0043531	AT3G46530.1	NB-ARC domain-containing disease resistance protein	LOC_Os11g11550.1	NBS-LRR disease resistance protein, putative, expressed	2,05	-1.0	1	21	237	257	UUGCAGUCCUCGAUGUCGUAG	GCACGACAUCGAGGAUUGCAU	Cleavage
tae-miR7757-5p	Traes_5BL_DD4C9F446.1	PF00069	KOG1187	2.7.11.1		GO:GO:0006468,GO:GO:0005524,GO:GO:0004672	AT3G55950.1	CRINKLY4 related 3	LOC_Os05g40150.1	RGH2B, putative, expressed	1,05	-1.0	1	22	62	83	AUAAAACCUUCAGCUAUCCAUC	AAUGGAUAGCGGAAGGUUUUAU	Cleavage
tae-miR7757-5p	Traes_5BL_DD4C9F446.2	PF00069	KOG1187	2.7.11.1		GO:GO:0006468,GO:GO:0005524,GO:GO:0004672	AT3G55950.1	CRINKLY4 related 3	LOC_Os05g40150.1	RGH2B, putative, expressed	1,05	-1.0	1	22	62	83	AUAAAACCUUCAGCUAUCCAUC	AAUGGAUAGCGGAAGGUUUUAU	Cleavage
tae-miR7757-5p	Traes_5BL_DD4C9F446.4						AT3G46710.1	NB-ARC domain-containing disease resistance protein	LOC_Os05g40150.1	RGH2B, putative, expressed	1,05	-1.0	1	22	62	83	AUAAAACCUUCAGCUAUCCAUC	AAUGGAUAGCGGAAGGUUUUAU	Cleavage
tae-miR7757-5p	Traes_5BL_DD4C9F446.5						AT3G46710.1	NB-ARC domain-containing disease resistance protein	LOC_Os05g40150.1	RGH2B, putative, expressed	1,05	-1.0	1	22	62	83	AUAAAACCUUCAGCUAUCCAUC	AAUGGAUAGCGGAAGGUUUUAU	Cleavage
tae-miR7757-5p	Traes_5BL_DD4C9F446.6						AT3G46710.1	NB-ARC domain-containing disease resistance protein	LOC_Os05g40150.1	RGH2B, putative, expressed	1,05	-1.0	1	22	62	83	AUAAAACCUUCAGCUAUCCAUC	AAUGGAUAGCGGAAGGUUUUAU	Cleavage
tae-miR9661-5p	Traes_5BL_E0C2C7D72.1										3,00	-1.0	1	21	98	118	UGAAGUAGAGCAGGGACCUCA	UCGUCUCCCUGCUCUACUUCA	Cleavage
tae-miR9661-5p	Traes_5BL_E0C2C7D72.2										3,00	-1.0	1	21	98	118	UGAAGUAGAGCAGGGACCUCA	UCGUCUCCCUGCUCUACUUCA	Cleavage
tae-miR9661-5p	Traes_5BL_E0C2C7D72.3										3,00	-1.0	1	21	95	115	UGAAGUAGAGCAGGGACCUCA	UCGUCUCCCUGCUCUACUUCA	Cleavage
tae-miR9652-5p	Traes_5BL_FC199BC2D.1										2,05	-1.0	1	22	412	433	CCUGUUUGUCAUUAAGUUUCUU	AAGAAACUGAAUGACAAGCAGG	Cleavage
tae-miR1122b-3p	Traes_5BS_321AF7B69.2										2,05	-1.0	1	21	23	43	AGACUUAUAUGUAGGAACGGA	UCCAUUCCUAAAUAUAAGUCU	Translation
tae-miR1122a	Traes_5BS_321AF7B69.2										2,05	-1.0	1	20	63	82	UAGAUACAUCCGUAUCUAGA	ACUACAUACGGAUGUAUAUA	Cleavage
tae-miR7757-5p	Traes_5BS_3FFCC89AD.1	PF00931	KOG4658			GO:GO:0043531	AT3G07040.1	NB-ARC domain-containing disease resistance protein	LOC_Os12g37770.1	RGH1A, putative, expressed	2,05	-1.0	1	22	1337	1358	AUAAAACCUUCAGCUAUCCAUC	GGUGGAUUGCUGAAGGAUUUAU	Cleavage
tae-miR7757-5p	Traes_5BS_3FFCC89AD.2	PF00931	KOG4658			GO:GO:0043531	AT3G46730.1	NB-ARC domain-containing disease resistance protein	LOC_Os12g37770.1	RGH1A, putative, expressed	2,05	-1.0	1	22	1298	1319	AUAAAACCUUCAGCUAUCCAUC	GGUGGAUUGCUGAAGGAUUUAU	Cleavage
tae-miR1118	Traes_5BS_550BDEE86.2										1,05	-1.0	1	23	1135	1157	CACUACAUUAUGGAAUGGAGGGA	UCCCUCCAUUCCACAAUGUAGUG	Translation
tae-miR5384-3p	Traes_5BS_90796C6CA.1										2,00	-1.0	1	21	461	481	UGAGCGCGCCGCCGUCGAAUG	UGUACGAUGGCGGCGCGCUCA	Cleavage
tae-miR5384-3p	Traes_5BS_90796C6CA.2										2,00	-1.0	1	21	34	54	UGAGCGCGCCGCCGUCGAAUG	UGUACGAUGGCGGCGCGCUCA	Cleavage
tae-miR5384-3p	Traes_5BS_90796C6CA.3										2,00	-1.0	1	21	392	412	UGAGCGCGCCGCCGUCGAAUG	UGUACGAUGGCGGCGCGCUCA	Cleavage
tae-miR164	Traes_5BS_9F043AE17.1										1,00	-1.0	1	21	656	676	UGGAGAAGCAGGGCACGUGCA	AGCAAGUGCCCUGCUUCUCCA	Cleavage
tae-miR5384-3p	Traes_5BS_BA0400E10.1										3,00	-1.0	1	21	1358	1378	UGAGCGCGCCGCCGUCGAAUG	CCGACGACGGCGGCGCGCUCC	Cleavage
tae-miR7757-5p	Traes_5BS_BCEA0112C.1	PF00931	KOG4658			GO:GO:0043531	AT3G07040.1	NB-ARC domain-containing disease resistance protein	LOC_Os12g37740.1	resistance protein LR10, putative, expressed	2,05	-1.0	1	22	1340	1361	AUAAAACCUUCAGCUAUCCAUC	GAUGGAUCGCGGAAGGUUUUAU	Cleavage
tae-miR7757-5p	Traes_5BS_BCEA0112C.2	PF00931	KOG4658			GO:GO:0043531	AT3G07040.1	NB-ARC domain-containing disease resistance protein	LOC_Os12g37740.1	resistance protein LR10, putative, expressed	2,05	-1.0	1	22	1340	1361	AUAAAACCUUCAGCUAUCCAUC	GAUGGAUCGCGGAAGGUUUUAU	Cleavage
tae-miR7757-5p	Traes_5BS_BCEA0112C.3	PF00931	KOG4658			GO:GO:0043531	AT3G07040.1	NB-ARC domain-containing disease resistance protein	LOC_Os12g37740.1	resistance protein LR10, putative, expressed	2,05	-1.0	1	22	1307	1328	AUAAAACCUUCAGCUAUCCAUC	GAUGGAUCGCGGAAGGUUUUAU	Cleavage
tae-miR7757-5p	Traes_5BS_BCEA0112C.4	PF00931				GO:GO:0043531	AT3G07040.1	NB-ARC domain-containing disease resistance protein	LOC_Os12g37740.1	resistance protein LR10, putative, expressed	2,05	-1.0	1	22	128	149	AUAAAACCUUCAGCUAUCCAUC	GAUGGAUCGCGGAAGGUUUUAU	Cleavage
tae-miR408	Traes_5BS_E10756355.1										2,05	-1.0	1	21	2512	2532	CUGCACUGCCUCUUCCCUGGC	GCCAAGGAAGAGGCUGUGCAG	Cleavage
tae-miR7757-5p	Traes_5BS_F5AFB24CD.2	PF00931				GO:GO:0043531	AT3G46530.1	NB-ARC domain-containing disease resistance protein	LOC_Os10g03570.1	RGH1A, putative, expressed	1,05	-1.0	1	22	779	800	AUAAAACCUUCAGCUAUCCAUC	GGUGGAUAGCUGAAGGUCUUAU	Cleavage
tae-miR7757-5p	Traes_5BS_F5AFB24CD.3	PF00931				GO:GO:0043531	AT3G46530.1	NB-ARC domain-containing disease resistance protein	LOC_Os10g03570.1	RGH1A, putative, expressed	1,05	-1.0	1	22	779	800	AUAAAACCUUCAGCUAUCCAUC	GGUGGAUAGCUGAAGGUCUUAU	Cleavage
tae-miR7757-5p	Traes_5BS_F5AFB24CD.4	PF00931				GO:GO:0043531	AT5G43470.2	Disease resistance protein (CC-NBS-LRR class) family	LOC_Os10g03570.1	RGH1A, putative, expressed	1,05	-1.0	1	22	371	392	AUAAAACCUUCAGCUAUCCAUC	GGUGGAUAGCUGAAGGUCUUAU	Cleavage
tae-miR156	Traes_5DL_1272B4BAC.1	PF03110				GO:GO:0005634,GO:GO:0003677	AT5G50670.1	Squamosa promoter-binding protein-like (SBP domain) transcription factor family protein	LOC_Os09g32944.1	OsSPL18 - SBP-box gene family member, expressed	1,00	-1.0	1	21	505	525	UGACAGAAGAGAGUGAGCACA	UGUGCUCUCUCUCUUCUGUCA	Cleavage
tae-miR156	Traes_5DL_1272B4BAC.2	PF03110				GO:GO:0005634,GO:GO:0003677	AT5G50670.1	Squamosa promoter-binding protein-like (SBP domain) transcription factor family protein	LOC_Os09g32944.1	OsSPL18 - SBP-box gene family member, expressed	1,00	-1.0	1	21	487	507	UGACAGAAGAGAGUGAGCACA	UGUGCUCUCUCUCUUCUGUCA	Cleavage
tae-miR156	Traes_5DL_1272B4BAC.3								LOC_Os09g32944.1	OsSPL18 - SBP-box gene family member, expressed	1,00	-1.0	1	21	382	402	UGACAGAAGAGAGUGAGCACA	UGUGCUCUCUCUCUUCUGUCA	Cleavage
tae-miR7757-5p	Traes_5DL_1733FB4DA.1	PF00931,PF03106				GO:GO:0043531,GO:GO:0043565,GO:GO:0006355,GO:GO:0003700	AT3G46530.1	NB-ARC domain-containing disease resistance protein	LOC_Os05g40150.1	RGH2B, putative, expressed	1,05	-1.0	1	22	617	638	AUAAAACCUUCAGCUAUCCAUC	AAUGGAUAGCUGAAGGAUUUAU	Cleavage
tae-miR7757-5p	Traes_5DL_1733FB4DA.2	PF03106,PF00931				GO:GO:0043565,GO:GO:0006355,GO:GO:0003700,GO:GO:0043531	AT3G46530.1	NB-ARC domain-containing disease resistance protein	LOC_Os05g40150.1	RGH2B, putative, expressed	1,05	-1.0	1	22	617	638	AUAAAACCUUCAGCUAUCCAUC	AAUGGAUAGCUGAAGGAUUUAU	Cleavage
tae-miR7757-5p	Traes_5DL_1733FB4DA.3	PF03106,PF00931				GO:GO:0043565,GO:GO:0006355,GO:GO:0003700,GO:GO:0043531	AT3G46530.1	NB-ARC domain-containing disease resistance protein	LOC_Os05g40150.1	RGH2B, putative, expressed	1,05	-1.0	1	22	617	638	AUAAAACCUUCAGCUAUCCAUC	AAUGGAUAGCUGAAGGAUUUAU	Cleavage
tae-miR7757-5p	Traes_5DL_1733FB4DA.4	PF00931				GO:GO:0043531	AT3G46530.1	NB-ARC domain-containing disease resistance protein	LOC_Os05g40150.1	RGH2B, putative, expressed	1,05	-1.0	1	22	617	638	AUAAAACCUUCAGCUAUCCAUC	AAUGGAUAGCUGAAGGAUUUAU	Cleavage
tae-miR7757-5p	Traes_5DL_1733FB4DA.5	PF00931				GO:GO:0043531	AT3G46530.1	NB-ARC domain-containing disease resistance protein	LOC_Os05g40150.1	RGH2B, putative, expressed	1,05	-1.0	1	22	617	638	AUAAAACCUUCAGCUAUCCAUC	AAUGGAUAGCUGAAGGAUUUAU	Cleavage
tae-miR7757-5p	Traes_5DL_1733FB4DA.6	PF00931				GO:GO:0043531	AT3G46530.1	NB-ARC domain-containing disease resistance protein	LOC_Os05g40150.1	RGH2B, putative, expressed	1,05	-1.0	1	22	617	638	AUAAAACCUUCAGCUAUCCAUC	AAUGGAUAGCUGAAGGAUUUAU	Cleavage
tae-miR7757-5p	Traes_5DL_1733FB4DA.7	PF00931				GO:GO:0043531	AT3G46530.1	NB-ARC domain-containing disease resistance protein	LOC_Os11g45750.1	WRKY125, expressed	1,05	-1.0	1	22	617	638	AUAAAACCUUCAGCUAUCCAUC	AAUGGAUAGCUGAAGGAUUUAU	Cleavage
tae-miR7757-5p	Traes_5DL_1733FB4DA.8	PF00931				GO:GO:0043531	AT3G46530.1	NB-ARC domain-containing disease resistance protein	LOC_Os11g45750.2	WRKY125, expressed	1,05	-1.0	1	22	617	638	AUAAAACCUUCAGCUAUCCAUC	AAUGGAUAGCUGAAGGAUUUAU	Cleavage
tae-miR1127b-3p	Traes_5DL_4E67E0243.9										1,05	-1.0	1	21	41	61	ACAAGUAUUUCUGGACGGAGG	CCUCCGUCCGGAAAUACUUGU	Cleavage
tae-miR9652-5p	Traes_5DL_714CCB5BC.1										2,05	-1.0	1	22	412	433	CCUGUUUGUCAUUAAGUUUCUU	AAGAAACUGAAUGACAAGCAGG	Cleavage
tae-miR9662a-3p	Traes_5DL_7643B2ADB.1	PF02536				GO:GO:0006355,GO:GO:0005739,GO:GO:0003690	AT5G64950.1	Mitochondrial transcription termination factor family protein	LOC_Os06g12100.1	mTERF family protein, expressed	2,05	-1.0	1	21	411	431	UUGAACAUCCCAGAGCCACCG	CCGUGGCUCUGUGAUGUUCAG	Translation
tae-miR9662b-3p	Traes_5DL_7643B2ADB.1	PF02536				GO:GO:0006355,GO:GO:0005739,GO:GO:0003690	AT5G64950.1	Mitochondrial transcription termination factor family protein	LOC_Os06g12100.1	mTERF family protein, expressed	2,05	-1.0	1	21	410	430	UGAACAUCCCAGAGCCACCGG	CCCGUGGCUCUGUGAUGUUCA	Cleavage
tae-miR5384-3p	Traes_5DL_78767F75C.1										2,05	-1.0	1	21	242	262	UGAGCGCGCCGCCGUCGAAUG	UCUUCCACGGCGGCGCGUUCA	Cleavage
tae-miR5049-3p	Traes_5DL_799647EA7.4										1,05	-1.0	1	21	65	85	AAUAUGGAUCGGAGGGAGUAC	GUACUCCCUCCGUUCCAUAUU	Cleavage
tae-miR9652-5p	Traes_5DL_8DE6C67FA.1										2,05	-1.0	1	22	412	433	CCUGUUUGUCAUUAAGUUUCUU	AAGAAACUGAAUGACAAGCAGG	Cleavage
tae-miR9652-5p	Traes_5DL_8DE6C67FA.2										2,05	-1.0	1	22	412	433	CCUGUUUGUCAUUAAGUUUCUU	AAGAAACUGAAUGACAAGCAGG	Cleavage
tae-miR9652-5p	Traes_5DL_8DE6C67FA.3										2,05	-1.0	1	22	415	436	CCUGUUUGUCAUUAAGUUUCUU	AAGAAACUGAAUGACAAGCAGG	Cleavage
tae-miR9652-5p	Traes_5DL_8DE6C67FA.4										2,05	-1.0	1	22	400	421	CCUGUUUGUCAUUAAGUUUCUU	AAGAAACUGAAUGACAAGCAGG	Cleavage
tae-miR9652-5p	Traes_5DL_8DE6C67FA.5										2,05	-1.0	1	22	400	421	CCUGUUUGUCAUUAAGUUUCUU	AAGAAACUGAAUGACAAGCAGG	Cleavage
tae-miR408	Traes_5DL_90BD9F306.1										0,00	-1.0	1	21	8	28	CUGCACUGCCUCUUCCCUGGC	CCCAGGGAAGAGGCAGUGCAG	Cleavage
tae-miR9666b-3p	Traes_5DL_9101D5AEC.1										2,05	-1.0	1	22	1183	1204	CGGUUGGGCUGUAUGAUGGCGA	AGUCCAUCAUACAGCCCAACAA	Cleavage
tae-miR1130b-3p	Traes_5DL_A6EECBC04.1										1,05	-1.0	1	21	376	396	UCUUAUAUUAUGGGACGGAGG	AUUCCGUCCCAUAAUGUAAGA	Cleavage
tae-miR9652-5p	Traes_5DL_B3713A4D1.1										2,05	-1.0	1	22	940	961	CCUGUUUGUCAUUAAGUUUCUU	AAGAAACUGAAUGACAAGCAGG	Cleavage
tae-miR7757-5p	Traes_5DS_0479C6E0F.1	PF00931				GO:GO:0043531	AT3G07040.1	NB-ARC domain-containing disease resistance protein	LOC_Os12g06920.1	NBS-LRR disease resistance protein, putative, expressed	2,05	-1.0	1	22	383	404	AUAAAACCUUCAGCUAUCCAUC	GAUGGAUCGCUGAAGGUUUGAU	Cleavage
tae-miR9662a-3p	Traes_5DS_094A03C13.1	PF02536				GO:GO:0006355,GO:GO:0005739,GO:GO:0003690	AT5G07900.1	Mitochondrial transcription termination factor family protein	LOC_Os06g12100.1	mTERF family protein, expressed	1,00	-1.0	1	21	549	569	UUGAACAUCCCAGAGCCACCG	CCGUGGCUCUGGGAUGUUCAG	Cleavage
tae-miR9662b-3p	Traes_5DS_094A03C13.1	PF02536				GO:GO:0006355,GO:GO:0005739,GO:GO:0003690	AT5G07900.1	Mitochondrial transcription termination factor family protein	LOC_Os06g12100.1	mTERF family protein, expressed	1,00	-1.0	1	21	548	568	UGAACAUCCCAGAGCCACCGG	CCCGUGGCUCUGGGAUGUUCA	Cleavage
tae-miR9662a-3p	Traes_5DS_094A03C13.2	PF02536				GO:GO:0006355,GO:GO:0005739,GO:GO:0003690	AT5G07900.1	Mitochondrial transcription termination factor family protein	LOC_Os06g12100.1	mTERF family protein, expressed	1,00	-1.0	1	21	243	263	UUGAACAUCCCAGAGCCACCG	CCGUGGCUCUGGGAUGUUCAG	Cleavage
tae-miR9662b-3p	Traes_5DS_094A03C13.2	PF02536				GO:GO:0006355,GO:GO:0005739,GO:GO:0003690	AT5G07900.1	Mitochondrial transcription termination factor family protein	LOC_Os06g12100.1	mTERF family protein, expressed	1,00	-1.0	1	21	242	262	UGAACAUCCCAGAGCCACCGG	CCCGUGGCUCUGGGAUGUUCA	Cleavage
tae-miR5384-3p	Traes_5DS_149714893.1										2,05	-1.0	1	21	158	178	UGAGCGCGCCGCCGUCGAAUG	CGUUCGUCGGCGUCGCGCUCA	Cleavage
tae-miR5384-3p	Traes_5DS_149714893.2										2,05	-1.0	1	21	152	172	UGAGCGCGCCGCCGUCGAAUG	CGUUCGUCGGCGUCGCGCUCA	Cleavage
tae-miR164	Traes_5DS_511F35A45.1										1,00	-1.0	1	21	209	229	UGGAGAAGCAGGGCACGUGCA	AGCAAGUGCCCUGCUUCUCCA	Cleavage
tae-miR7757-5p	Traes_5DS_8DB03B7DA.1	PF00931	KOG4658			GO:GO:0043531	AT3G07040.1	NB-ARC domain-containing disease resistance protein	LOC_Os12g37740.1	resistance protein LR10, putative, expressed	2,05	-1.0	1	22	1349	1370	AUAAAACCUUCAGCUAUCCAUC	GAUGGAUCGCGGAAGGUUUUAU	Cleavage
tae-miR7757-5p	Traes_5DS_8DB03B7DA.2	PF00931				GO:GO:0043531	AT3G07040.1	NB-ARC domain-containing disease resistance protein	LOC_Os12g37740.1	resistance protein LR10, putative, expressed	2,05	-1.0	1	22	128	149	AUAAAACCUUCAGCUAUCCAUC	GAUGGAUCGCGGAAGGUUUUAU	Cleavage
tae-miR7757-5p	Traes_5DS_9DFE4D217.1	PF00931				GO:GO:0043531	AT1G53350.1	Disease resistance protein (CC-NBS-LRR class) family	LOC_Os10g22484.1	RGH2B, putative, expressed	2,05	-1.0	1	22	185	206	AUAAAACCUUCAGCUAUCCAUC	AAUGGGUAGCUGAAGGUUGUAU	Cleavage
tae-miR7757-5p	Traes_5DS_9DFE4D217.2	PF00931				GO:GO:0043531	AT1G53350.1	Disease resistance protein (CC-NBS-LRR class) family	LOC_Os10g22484.1	RGH2B, putative, expressed	2,05	-1.0	1	22	185	206	AUAAAACCUUCAGCUAUCCAUC	AAUGGGUAGCUGAAGGUUGUAU	Cleavage
tae-miR7757-5p	Traes_5DS_9DFE4D217.3	PF00931				GO:GO:0043531	AT1G53350.1	Disease resistance protein (CC-NBS-LRR class) family	LOC_Os10g22484.1	RGH2B, putative, expressed	2,05	-1.0	1	22	185	206	AUAAAACCUUCAGCUAUCCAUC	AAUGGGUAGCUGAAGGUUGUAU	Cleavage
tae-miR9662a-3p	Traes_5DS_9F4A5AE9F.1	PF02536				GO:GO:0006355,GO:GO:0005739,GO:GO:0003690	AT5G07900.1	Mitochondrial transcription termination factor family protein	LOC_Os06g12100.1	mTERF family protein, expressed	2,00	-1.0	1	21	441	461	UUGAACAUCCCAGAGCCACCG	UCGUGGCGCUGGGAUGUUCAG	Cleavage
tae-miR9662b-3p	Traes_5DS_9F4A5AE9F.1	PF02536				GO:GO:0006355,GO:GO:0005739,GO:GO:0003690	AT5G07900.1	Mitochondrial transcription termination factor family protein	LOC_Os06g12100.1	mTERF family protein, expressed	2,05	-1.0	1	21	440	460	UGAACAUCCCAGAGCCACCGG	CUCGUGGCGCUGGGAUGUUCA	Cleavage
tae-miR9662a-3p	Traes_5DS_9F4A5AE9F.2	PF02536				GO:GO:0006355,GO:GO:0005739,GO:GO:0003690	AT5G07900.1	Mitochondrial transcription termination factor family protein	LOC_Os06g12100.1	mTERF family protein, expressed	2,00	-1.0	1	21	66	86	UUGAACAUCCCAGAGCCACCG	UCGUGGCGCUGGGAUGUUCAG	Cleavage
tae-miR9662b-3p	Traes_5DS_9F4A5AE9F.2	PF02536				GO:GO:0006355,GO:GO:0005739,GO:GO:0003690	AT5G07900.1	Mitochondrial transcription termination factor family protein	LOC_Os06g12100.1	mTERF family protein, expressed	2,05	-1.0	1	21	65	85	UGAACAUCCCAGAGCCACCGG	CUCGUGGCGCUGGGAUGUUCA	Cleavage
tae-miR1122b-3p	Traes_5DS_9F8FFD461.1										1,05	-1.0	1	21	1777	1797	AGACUUAUAUGUAGGAACGGA	UCCGUUCCUAAAUAUAAGUCU	Translation
tae-miR1122b-3p	Traes_5DS_9F8FFD461.2										1,05	-1.0	1	21	1882	1902	AGACUUAUAUGUAGGAACGGA	UCCGUUCCUAAAUAUAAGUCU	Translation
tae-miR1122b-3p	Traes_5DS_9F8FFD461.3										1,05	-1.0	1	21	1801	1821	AGACUUAUAUGUAGGAACGGA	UCCGUUCCUAAAUAUAAGUCU	Translation
tae-miR1122b-3p	Traes_5DS_A6AE32DCD.1										1,05	-1.0	1	21	1065	1085	AGACUUAUAUGUAGGAACGGA	UCCGUUCCUAAAUAUAAGUCU	Translation
tae-miR1122b-3p	Traes_5DS_A6AE32DCD.2										1,05	-1.0	1	21	1146	1166	AGACUUAUAUGUAGGAACGGA	UCCGUUCCUAAAUAUAAGUCU	Translation
tae-miR1122b-3p	Traes_5DS_A6AE32DCD.3										1,05	-1.0	1	21	1146	1166	AGACUUAUAUGUAGGAACGGA	UCCGUUCCUAAAUAUAAGUCU	Translation
tae-miR9662a-3p	Traes_5DS_C87840EBA.2						AT1G79220.1	Mitochondrial transcription termination factor family protein	LOC_Os06g12100.1	mTERF family protein, expressed	2,00	-1.0	1	21	429	449	UUGAACAUCCCAGAGCCACCG	CCGUGGUUCUGGGAUGUUCAG	Cleavage
tae-miR9662b-3p	Traes_5DS_C87840EBA.2						AT1G79220.1	Mitochondrial transcription termination factor family protein	LOC_Os06g12100.1	mTERF family protein, expressed	2,00	-1.0	1	21	428	448	UGAACAUCCCAGAGCCACCGG	CCCGUGGUUCUGGGAUGUUCA	Cleavage
tae-miR1128	Traes_5DS_D165422B3.1										3,00	-1.0	1	21	59	80	UACUACUCCCUCCG-UCCGAAA	UUUAGGAACGGAGGGAGUAGUA	Cleavage
tae-miR1118	Traes_5DS_F8D323D4D.2										1,05	-1.0	1	23	1329	1351	CACUACAUUAUGGAAUGGAGGGA	CUCCUCCAUUCCACAAUGUAGUG	Translation
tae-miR160	Traes_6AL_232AF0B76.1										0,00	-1.0	1	21	195	215	UGCCUGGCUCCCUGUAUGCCA	AGGCAUACAGGGAGCCAGGCA	Cleavage
tae-miR1121	Traes_6AL_23B31796F.2										2,00	-1.0	1	22	846	867	AGUAGUGAUCUAAACGCUCUUA	UAAAAGUGUUUAGAUCACUACU	Cleavage
tae-miR1121	Traes_6AL_23B31796F.3										2,00	-1.0	1	22	760	781	AGUAGUGAUCUAAACGCUCUUA	UAAAAGUGUUUAGAUCACUACU	Cleavage
tae-miR1121	Traes_6AL_23B31796F.5										2,00	-1.0	1	22	742	763	AGUAGUGAUCUAAACGCUCUUA	UAAAAGUGUUUAGAUCACUACU	Cleavage
tae-miR5049-3p	Traes_6AL_36AE1B4CF.4										1,05	-1.0	1	21	1787	1807	AAUAUGGAUCGGAGGGAGUAC	AUACUCCCUCCGACCCAUAUU	Cleavage
tae-miR5049-3p	Traes_6AL_36AE1B4CF1.4										1,05	-1.0	1	21	1787	1807	AAUAUGGAUCGGAGGGAGUAC	AUACUCCCUCCGACCCAUAUU	Cleavage
tae-miR5049-3p	Traes_6AL_6AD463C67.3										2,05	-1.0	1	21	15	35	AAUAUGGAUCGGAGGGAGUAC	GUACUCCUUCCGAUCCAUAAU	Cleavage
tae-miR1121	Traes_6AL_7E70268F1.3										0,00	-1.0	1	22	497	518	AGUAGUGAUCUAAACGCUCUUA	UAAGAGCGUUUAGAUCACUACU	Cleavage
tae-miR1122b-3p	Traes_6AL_94CA6F0D8.5										2,05	-1.0	1	21	1030	1050	AGACUUAUAUGUAGGAACGGA	UCCAUUCCUAAAUAUAAGUCU	Translation
tae-miR1122a	Traes_6AL_94CA6F0D8.5										2,05	-1.0	1	20	1071	1090	UAGAUACAUCCGUAUCUAGA	ACUACAUACGGAUGUAUAUA	Cleavage
tae-miR9662a-3p	Traes_6AS_0A204AEC9.1	PF02536	KOG1267		K15032	GO:GO:0006355,GO:GO:0005739,GO:GO:0003690	AT5G07900.1	Mitochondrial transcription termination factor family protein	LOC_Os06g12100.1	mTERF family protein, expressed	2,00	-1.0	1	21	681	701	UUGAACAUCCCAGAGCCACCG	CCGUGCCUCUGGGAUGUUCAG	Cleavage
tae-miR9662b-3p	Traes_6AS_0A204AEC9.1	PF02536	KOG1267		K15032	GO:GO:0006355,GO:GO:0005739,GO:GO:0003690	AT5G07900.1	Mitochondrial transcription termination factor family protein	LOC_Os06g12100.1	mTERF family protein, expressed	2,00	-1.0	1	21	680	700	UGAACAUCCCAGAGCCACCGG	CCCGUGCCUCUGGGAUGUUCA	Cleavage
tae-miR9662a-3p	Traes_6AS_0A204AEC9.2	PF02536	KOG1267		K15032	GO:GO:0006355,GO:GO:0005739,GO:GO:0003690	AT5G07900.1	Mitochondrial transcription termination factor family protein	LOC_Os06g12100.1	mTERF family protein, expressed	2,00	-1.0	1	21	681	701	UUGAACAUCCCAGAGCCACCG	CCGUGCCUCUGGGAUGUUCAG	Cleavage
tae-miR9662b-3p	Traes_6AS_0A204AEC9.2	PF02536	KOG1267		K15032	GO:GO:0006355,GO:GO:0005739,GO:GO:0003690	AT5G07900.1	Mitochondrial transcription termination factor family protein	LOC_Os06g12100.1	mTERF family protein, expressed	2,00	-1.0	1	21	680	700	UGAACAUCCCAGAGCCACCGG	CCCGUGCCUCUGGGAUGUUCA	Cleavage
tae-miR9662a-3p	Traes_6AS_1496A7825.1	PF02536				GO:GO:0006355,GO:GO:0005739,GO:GO:0003690	AT5G07900.1	Mitochondrial transcription termination factor family protein	LOC_Os06g12100.1	mTERF family protein, expressed	1,05	-1.0	1	21	549	569	UUGAACAUCCCAGAGCCACCG	CCGUGGCUCGGGGAUGUUCAA	Cleavage
tae-miR9662b-3p	Traes_6AS_1496A7825.1	PF02536				GO:GO:0006355,GO:GO:0005739,GO:GO:0003690	AT5G07900.1	Mitochondrial transcription termination factor family protein	LOC_Os06g12100.1	mTERF family protein, expressed	2,05	-1.0	1	21	548	568	UGAACAUCCCAGAGCCACCGG	CCCGUGGCUCGGGGAUGUUCA	Translation
tae-miR9662a-3p	Traes_6AS_1496A7825.2	PF02536				GO:GO:0006355,GO:GO:0005739,GO:GO:0003690	AT5G07900.1	Mitochondrial transcription termination factor family protein	LOC_Os06g12100.1	mTERF family protein, expressed	1,05	-1.0	1	21	132	152	UUGAACAUCCCAGAGCCACCG	CCGUGGCUCGGGGAUGUUCAA	Cleavage
tae-miR9662b-3p	Traes_6AS_1496A7825.2	PF02536				GO:GO:0006355,GO:GO:0005739,GO:GO:0003690	AT5G07900.1	Mitochondrial transcription termination factor family protein	LOC_Os06g12100.1	mTERF family protein, expressed	2,05	-1.0	1	21	131	151	UGAACAUCCCAGAGCCACCGG	CCCGUGGCUCGGGGAUGUUCA	Translation
tae-miR9662a-3p	Traes_6AS_15C1E6F18.1	PF02536				GO:GO:0006355,GO:GO:0005739,GO:GO:0003690	AT1G21150.1	Mitochondrial transcription termination factor family protein	LOC_Os06g12100.1	mTERF family protein, expressed	2,05	-1.0	1	21	33	53	UUGAACAUCCCAGAGCCACCG	CUGUGGCUCUGGAAUGUUCAG	Cleavage
tae-miR9662b-3p	Traes_6AS_15C1E6F18.1	PF02536				GO:GO:0006355,GO:GO:0005739,GO:GO:0003690	AT1G21150.1	Mitochondrial transcription termination factor family protein	LOC_Os06g12100.1	mTERF family protein, expressed	2,05	-1.0	1	21	32	52	UGAACAUCCCAGAGCCACCGG	CCUGUGGCUCUGGAAUGUUCA	Cleavage
tae-miR9662a-3p	Traes_6AS_15C1E6F18.2	PF02536				GO:GO:0006355,GO:GO:0005739,GO:GO:0003690	AT1G21150.1	Mitochondrial transcription termination factor family protein	LOC_Os06g12100.1	mTERF family protein, expressed	2,05	-1.0	1	21	33	53	UUGAACAUCCCAGAGCCACCG	CUGUGGCUCUGGAAUGUUCAG	Cleavage
tae-miR9662b-3p	Traes_6AS_15C1E6F18.2	PF02536				GO:GO:0006355,GO:GO:0005739,GO:GO:0003690	AT1G21150.1	Mitochondrial transcription termination factor family protein	LOC_Os06g12100.1	mTERF family protein, expressed	2,05	-1.0	1	21	32	52	UGAACAUCCCAGAGCCACCGG	CCUGUGGCUCUGGAAUGUUCA	Cleavage
tae-miR9662a-3p	Traes_6AS_2BDC1663A.1	PF02536	KOG1267		K15032	GO:GO:0006355,GO:GO:0005739,GO:GO:0003690	AT5G07900.1	Mitochondrial transcription termination factor family protein	LOC_Os06g12100.1	mTERF family protein, expressed	1,00	-1.0	1	21	681	701	UUGAACAUCCCAGAGCCACCG	CCGUGGCUCUGGGAUGUUCAG	Cleavage
tae-miR9662b-3p	Traes_6AS_2BDC1663A.1	PF02536	KOG1267		K15032	GO:GO:0006355,GO:GO:0005739,GO:GO:0003690	AT5G07900.1	Mitochondrial transcription termination factor family protein	LOC_Os06g12100.1	mTERF family protein, expressed	1,00	-1.0	1	21	680	700	UGAACAUCCCAGAGCCACCGG	CCCGUGGCUCUGGGAUGUUCA	Cleavage
tae-miR156	Traes_6AS_32A35158A.1	PF03110				GO:GO:0005634,GO:GO:0003677	AT5G43270.2	squamosa promoter binding protein-like 2	LOC_Os06g45310.1	OsSPL11 - SBP-box gene family member, expressed	1,00	-1.0	1	21	736	756	UGACAGAAGAGAGUGAGCACA	CGUGCUCUCUCUCUUCUGUCA	Cleavage
tae-miR156	Traes_6AS_32A35158A.2								LOC_Os06g45310.1	OsSPL11 - SBP-box gene family member, expressed	1,00	-1.0	1	21	292	312	UGACAGAAGAGAGUGAGCACA	CGUGCUCUCUCUCUUCUGUCA	Cleavage
tae-miR156	Traes_6AS_32A35158A.3								LOC_Os06g45310.1	OsSPL11 - SBP-box gene family member, expressed	1,00	-1.0	1	21	64	84	UGACAGAAGAGAGUGAGCACA	CGUGCUCUCUCUCUUCUGUCA	Cleavage
tae-miR9662a-3p	Traes_6AS_3641B9C871.1	PF02536	KOG1267		K15032	GO:GO:0006355,GO:GO:0005739,GO:GO:0003690	AT5G07900.1	Mitochondrial transcription termination factor family protein	LOC_Os06g12100.1	mTERF family protein, expressed	1,00	-1.0	1	21	687	707	UUGAACAUCCCAGAGCCACCG	CCGUGGCUCUGGGAUGUUCAG	Cleavage
tae-miR9662b-3p	Traes_6AS_3641B9C871.1	PF02536	KOG1267		K15032	GO:GO:0006355,GO:GO:0005739,GO:GO:0003690	AT5G07900.1	Mitochondrial transcription termination factor family protein	LOC_Os06g12100.1	mTERF family protein, expressed	1,00	-1.0	1	21	686	706	UGAACAUCCCAGAGCCACCGG	CCCGUGGCUCUGGGAUGUUCA	Cleavage
tae-miR9662a-3p	Traes_6AS_3641B9C871.2	PF02536	KOG1267		K15032	GO:GO:0006355,GO:GO:0005739,GO:GO:0003690	AT5G07900.1	Mitochondrial transcription termination factor family protein	LOC_Os06g12100.1	mTERF family protein, expressed	1,00	-1.0	1	21	687	707	UUGAACAUCCCAGAGCCACCG	CCGUGGCUCUGGGAUGUUCAG	Cleavage
tae-miR9662b-3p	Traes_6AS_3641B9C871.2	PF02536	KOG1267		K15032	GO:GO:0006355,GO:GO:0005739,GO:GO:0003690	AT5G07900.1	Mitochondrial transcription termination factor family protein	LOC_Os06g12100.1	mTERF family protein, expressed	1,00	-1.0	1	21	686	706	UGAACAUCCCAGAGCCACCGG	CCCGUGGCUCUGGGAUGUUCA	Cleavage
tae-miR9662a-3p	Traes_6AS_36EAEADDD.1	PF02536				GO:GO:0006355,GO:GO:0005739,GO:GO:0003690	AT1G21150.1	Mitochondrial transcription termination factor family protein	LOC_Os06g12100.1	mTERF family protein, expressed	2,00	-1.0	1	21	33	53	UUGAACAUCCCAGAGCCACCG	CUGUGGUUCUGGGAUGUUCAG	Cleavage
tae-miR9662b-3p	Traes_6AS_36EAEADDD.1	PF02536				GO:GO:0006355,GO:GO:0005739,GO:GO:0003690	AT1G21150.1	Mitochondrial transcription termination factor family protein	LOC_Os06g12100.1	mTERF family protein, expressed	2,00	-1.0	1	21	32	52	UGAACAUCCCAGAGCCACCGG	CCUGUGGUUCUGGGAUGUUCA	Cleavage
tae-miR9662a-3p	Traes_6AS_36EAEADDD.2								LOC_Os06g12100.1	mTERF family protein, expressed	2,00	-1.0	1	21	33	53	UUGAACAUCCCAGAGCCACCG	CUGUGGUUCUGGGAUGUUCAG	Cleavage
tae-miR9662b-3p	Traes_6AS_36EAEADDD.2								LOC_Os06g12100.1	mTERF family protein, expressed	2,00	-1.0	1	21	32	52	UGAACAUCCCAGAGCCACCGG	CCUGUGGUUCUGGGAUGUUCA	Cleavage
tae-miR9662a-3p	Traes_6AS_36EAEADDD.3	PF02536				GO:GO:0006355,GO:GO:0005739,GO:GO:0003690	AT1G21150.1	Mitochondrial transcription termination factor family protein	LOC_Os06g12100.1	mTERF family protein, expressed	2,00	-1.0	1	21	33	53	UUGAACAUCCCAGAGCCACCG	CUGUGGUUCUGGGAUGUUCAG	Cleavage
tae-miR9662b-3p	Traes_6AS_36EAEADDD.3	PF02536				GO:GO:0006355,GO:GO:0005739,GO:GO:0003690	AT1G21150.1	Mitochondrial transcription termination factor family protein	LOC_Os06g12100.1	mTERF family protein, expressed	2,00	-1.0	1	21	32	52	UGAACAUCCCAGAGCCACCGG	CCUGUGGUUCUGGGAUGUUCA	Cleavage
tae-miR9662a-3p	Traes_6AS_48B701BB41.1	PF02536				GO:GO:0006355,GO:GO:0005739,GO:GO:0003690	AT1G21150.1	Mitochondrial transcription termination factor family protein	LOC_Os06g12100.1	mTERF family protein, expressed	1,00	-1.0	1	21	33	53	UUGAACAUCCCAGAGCCACCG	ACGUGGCUCUGGGAUGUUCAG	Cleavage

tae-miR9662b-3p	Traes_6AS_48B701BB41.1	PF02536				GO:GO:0006355,GO:GO:0005739,GO:GO:0003690	AT1G21150.1	Mitochondrial transcription termination factor family protein	LOC_Os06g12100.1	mTERF family protein, expressed	1,00	-1.0	1	21	32	52	UGAACAUCCCAGAGCCACCGG	CACGUGGCUCUGGGAUGUUCA	Cleavage
tae-miR9662a-3p	Traes_6AS_48B701BB41.2	PF02536				GO:GO:0006355,GO:GO:0005739,GO:GO:0003690	AT1G21150.1	Mitochondrial transcription termination factor family protein	LOC_Os06g12100.1	mTERF family protein, expressed	1,00	-1.0	1	21	33	53	UUGAACAUCCCAGAGCCACCG	ACGUGGCUCUGGGAUGUUCAG	Cleavage
tae-miR9662b-3p	Traes_6AS_48B701BB41.2	PF02536				GO:GO:0006355,GO:GO:0005739,GO:GO:0003690	AT1G21150.1	Mitochondrial transcription termination factor family protein	LOC_Os06g12100.1	mTERF family protein, expressed	1,00	-1.0	1	21	32	52	UGAACAUCCCAGAGCCACCGG	CACGUGGCUCUGGGAUGUUCA	Cleavage
tae-miR9662a-3p	Traes_6AS_48B701BB41.3	PF02536				GO:GO:0006355,GO:GO:0005739,GO:GO:0003690	AT1G21150.1	Mitochondrial transcription termination factor family protein	LOC_Os06g12100.1	mTERF family protein, expressed	1,00	-1.0	1	21	33	53	UUGAACAUCCCAGAGCCACCG	ACGUGGCUCUGGGAUGUUCAG	Cleavage
tae-miR9662b-3p	Traes_6AS_48B701BB41.3	PF02536				GO:GO:0006355,GO:GO:0005739,GO:GO:0003690	AT1G21150.1	Mitochondrial transcription termination factor family protein	LOC_Os06g12100.1	mTERF family protein, expressed	1,00	-1.0	1	21	32	52	UGAACAUCCCAGAGCCACCGG	CACGUGGCUCUGGGAUGUUCA	Cleavage
tae-miR9662a-3p	Traes_6AS_48B701BB41.4	PF02536				GO:GO:0006355,GO:GO:0005739,GO:GO:0003690	AT1G21150.1	Mitochondrial transcription termination factor family protein	LOC_Os06g12100.1	mTERF family protein, expressed	1,00	-1.0	1	21	33	53	UUGAACAUCCCAGAGCCACCG	ACGUGGCUCUGGGAUGUUCAG	Cleavage
tae-miR9662b-3p	Traes_6AS_48B701BB41.4	PF02536				GO:GO:0006355,GO:GO:0005739,GO:GO:0003690	AT1G21150.1	Mitochondrial transcription termination factor family protein	LOC_Os06g12100.1	mTERF family protein, expressed	1,00	-1.0	1	21	32	52	UGAACAUCCCAGAGCCACCGG	CACGUGGCUCUGGGAUGUUCA	Cleavage
tae-miR9662a-3p	Traes_6AS_48B701BB41.5	PF02536				GO:GO:0006355,GO:GO:0005739,GO:GO:0003690	AT1G21150.1	Mitochondrial transcription termination factor family protein	LOC_Os06g12100.1	mTERF family protein, expressed	1,00	-1.0	1	21	33	53	UUGAACAUCCCAGAGCCACCG	ACGUGGCUCUGGGAUGUUCAG	Cleavage
tae-miR9662b-3p	Traes_6AS_48B701BB41.5	PF02536				GO:GO:0006355,GO:GO:0005739,GO:GO:0003690	AT1G21150.1	Mitochondrial transcription termination factor family protein	LOC_Os06g12100.1	mTERF family protein, expressed	1,00	-1.0	1	21	32	52	UGAACAUCCCAGAGCCACCGG	CACGUGGCUCUGGGAUGUUCA	Cleavage
tae-miR1138	Traes_6AS_5F8725CDA.1										3,00	-1.0	1	23	2223	2245	GCUUAGAUGUGACAUCCUUAAAA	AGUUAGUUAUGUCACAUCUAAGC	Cleavage
tae-miR1138	Traes_6AS_5F8725CDA.2										3,00	-1.0	1	23	1969	1991	GCUUAGAUGUGACAUCCUUAAAA	AGUUAGUUAUGUCACAUCUAAGC	Cleavage
tae-miR9662a-3p	Traes_6AS_6612DF8D7.1	PF02536				GO:GO:0006355,GO:GO:0005739,GO:GO:0003690	AT5G07900.1	Mitochondrial transcription termination factor family protein	LOC_Os06g12100.1	mTERF family protein, expressed	1,00	-1.0	1	21	81	101	UUGAACAUCCCAGAGCCACCG	CCGUGGCUCUGGGAUGUUCAG	Cleavage
tae-miR9662b-3p	Traes_6AS_6612DF8D7.1	PF02536				GO:GO:0006355,GO:GO:0005739,GO:GO:0003690	AT5G07900.1	Mitochondrial transcription termination factor family protein	LOC_Os06g12100.1	mTERF family protein, expressed	1,00	-1.0	1	21	80	100	UGAACAUCCCAGAGCCACCGG	CCCGUGGCUCUGGGAUGUUCA	Cleavage
tae-miR9662a-3p	Traes_6AS_6612DF8D7.2	PF02536				GO:GO:0006355,GO:GO:0005739,GO:GO:0003690	AT5G07900.1	Mitochondrial transcription termination factor family protein	LOC_Os06g12100.1	mTERF family protein, expressed	1,00	-1.0	1	21	81	101	UUGAACAUCCCAGAGCCACCG	CCGUGGCUCUGGGAUGUUCAG	Cleavage
tae-miR9662b-3p	Traes_6AS_6612DF8D7.2	PF02536				GO:GO:0006355,GO:GO:0005739,GO:GO:0003690	AT5G07900.1	Mitochondrial transcription termination factor family protein	LOC_Os06g12100.1	mTERF family protein, expressed	1,00	-1.0	1	21	80	100	UGAACAUCCCAGAGCCACCGG	CCCGUGGCUCUGGGAUGUUCA	Cleavage
tae-miR9662a-3p	Traes_6AS_6612DF8D7.3	PF02536				GO:GO:0006355,GO:GO:0005739,GO:GO:0003690	AT5G07900.1	Mitochondrial transcription termination factor family protein	LOC_Os06g12100.1	mTERF family protein, expressed	1,00	-1.0	1	21	81	101	UUGAACAUCCCAGAGCCACCG	CCGUGGCUCUGGGAUGUUCAG	Cleavage
tae-miR9662b-3p	Traes_6AS_6612DF8D7.3	PF02536				GO:GO:0006355,GO:GO:0005739,GO:GO:0003690	AT5G07900.1	Mitochondrial transcription termination factor family protein	LOC_Os06g12100.1	mTERF family protein, expressed	1,00	-1.0	1	21	80	100	UGAACAUCCCAGAGCCACCGG	CCCGUGGCUCUGGGAUGUUCA	Cleavage
tae-miR9662a-3p	Traes_6AS_8D2111A83.1	PF02536				GO:GO:0006355,GO:GO:0005739,GO:GO:0003690	AT1G21150.1	Mitochondrial transcription termination factor family protein	LOC_Os06g12100.1	mTERF family protein, expressed	2,05	-1.0	1	21	33	53	UUGAACAUCCCAGAGCCACCG	UCGUGGCUCGGGGAUGUUCAG	Cleavage
tae-miR9662b-3p	Traes_6AS_8D2111A83.1	PF02536				GO:GO:0006355,GO:GO:0005739,GO:GO:0003690	AT1G21150.1	Mitochondrial transcription termination factor family protein	LOC_Os06g12100.1	mTERF family protein, expressed	2,05	-1.0	1	21	32	52	UGAACAUCCCAGAGCCACCGG	CUCGUGGCUCGGGGAUGUUCA	Translation
tae-miR9662a-3p	Traes_6AS_8D2111A83.2	PF02536				GO:GO:0006355,GO:GO:0005739,GO:GO:0003690	AT1G21150.1	Mitochondrial transcription termination factor family protein	LOC_Os06g12100.1	mTERF family protein, expressed	2,05	-1.0	1	21	246	266	UUGAACAUCCCAGAGCCACCG	UCGUGGCUCGGGGAUGUUCAG	Cleavage
tae-miR9662b-3p	Traes_6AS_8D2111A83.2	PF02536				GO:GO:0006355,GO:GO:0005739,GO:GO:0003690	AT1G21150.1	Mitochondrial transcription termination factor family protein	LOC_Os06g12100.1	mTERF family protein, expressed	2,05	-1.0	1	21	245	265	UGAACAUCCCAGAGCCACCGG	CUCGUGGCUCGGGGAUGUUCA	Translation
tae-miR9662a-3p	Traes_6AS_8D2111A83.3	PF02536				GO:GO:0006355,GO:GO:0005739,GO:GO:0003690	AT1G21150.1	Mitochondrial transcription termination factor family protein	LOC_Os06g12100.1	mTERF family protein, expressed	2,05	-1.0	1	21	246	266	UUGAACAUCCCAGAGCCACCG	UCGUGGCUCGGGGAUGUUCAG	Cleavage
tae-miR9662b-3p	Traes_6AS_8D2111A83.3	PF02536				GO:GO:0006355,GO:GO:0005739,GO:GO:0003690	AT1G21150.1	Mitochondrial transcription termination factor family protein	LOC_Os06g12100.1	mTERF family protein, expressed	2,05	-1.0	1	21	245	265	UGAACAUCCCAGAGCCACCGG	CUCGUGGCUCGGGGAUGUUCA	Translation
tae-miR9662a-3p	Traes_6AS_8D2111A83.4	PF02536				GO:GO:0006355,GO:GO:0005739,GO:GO:0003690	AT1G21150.1	Mitochondrial transcription termination factor family protein	LOC_Os06g12100.1	mTERF family protein, expressed	2,05	-1.0	1	21	246	266	UUGAACAUCCCAGAGCCACCG	UCGUGGCUCGGGGAUGUUCAG	Cleavage
tae-miR9662b-3p	Traes_6AS_8D2111A83.4	PF02536				GO:GO:0006355,GO:GO:0005739,GO:GO:0003690	AT1G21150.1	Mitochondrial transcription termination factor family protein	LOC_Os06g12100.1	mTERF family protein, expressed	2,05	-1.0	1	21	245	265	UGAACAUCCCAGAGCCACCGG	CUCGUGGCUCGGGGAUGUUCA	Translation
tae-miR9662a-3p	Traes_6AS_8D2111A83.5	PF02536				GO:GO:0006355,GO:GO:0005739,GO:GO:0003690	AT1G21150.1	Mitochondrial transcription termination factor family protein	LOC_Os06g12100.1	mTERF family protein, expressed	2,05	-1.0	1	21	246	266	UUGAACAUCCCAGAGCCACCG	UCGUGGCUCGGGGAUGUUCAG	Cleavage
tae-miR9662b-3p	Traes_6AS_8D2111A83.5	PF02536				GO:GO:0006355,GO:GO:0005739,GO:GO:0003690	AT1G21150.1	Mitochondrial transcription termination factor family protein	LOC_Os06g12100.1	mTERF family protein, expressed	2,05	-1.0	1	21	245	265	UGAACAUCCCAGAGCCACCGG	CUCGUGGCUCGGGGAUGUUCA	Translation
tae-miR9662a-3p	Traes_6AS_8D2111A83.6						AT1G21150.1	Mitochondrial transcription termination factor family protein	LOC_Os06g12100.1	mTERF family protein, expressed	2,05	-1.0	1	21	246	266	UUGAACAUCCCAGAGCCACCG	UCGUGGCUCGGGGAUGUUCAG	Cleavage
tae-miR9662b-3p	Traes_6AS_8D2111A83.6						AT1G21150.1	Mitochondrial transcription termination factor family protein	LOC_Os06g12100.1	mTERF family protein, expressed	2,05	-1.0	1	21	245	265	UGAACAUCCCAGAGCCACCGG	CUCGUGGCUCGGGGAUGUUCA	Translation
tae-miR9662a-3p	Traes_6AS_8D2111A83.7	PF02536				GO:GO:0006355,GO:GO:0005739,GO:GO:0003690	AT1G21150.1	Mitochondrial transcription termination factor family protein	LOC_Os06g12100.1	mTERF family protein, expressed	2,05	-1.0	1	21	33	53	UUGAACAUCCCAGAGCCACCG	UCGUGGCUCGGGGAUGUUCAG	Cleavage
tae-miR9662b-3p	Traes_6AS_8D2111A83.7	PF02536				GO:GO:0006355,GO:GO:0005739,GO:GO:0003690	AT1G21150.1	Mitochondrial transcription termination factor family protein	LOC_Os06g12100.1	mTERF family protein, expressed	2,05	-1.0	1	21	32	52	UGAACAUCCCAGAGCCACCGG	CUCGUGGCUCGGGGAUGUUCA	Translation
tae-miR1122b-3p	Traes_6AS_8E6E52967.2										1,05	-1.0	1	21	120	140	AGACUUAUAUGUAGGAACGGA	UCCGUUCCUAAAUAUAAGUCU	Translation
tae-miR156	Traes_6AS_99C6A3B0B.1	PF03110				GO:GO:0005634,GO:GO:0003677	AT5G43270.1	squamosa promoter binding protein-like 2	LOC_Os02g04680.2	OsSPL3 - SBP-box gene family member, expressed	1,00	-1.0	1	21	1477	1497	UGACAGAAGAGAGUGAGCACA	CAUGCUCUCUCUCUUCUGUCA	Cleavage
tae-miR156	Traes_6AS_99C6A3B0B.2	PF03110				GO:GO:0005634,GO:GO:0003677	AT5G43270.3	squamosa promoter binding protein-like 2	LOC_Os02g04680.2	OsSPL3 - SBP-box gene family member, expressed	1,00	-1.0	1	21	1477	1497	UGACAGAAGAGAGUGAGCACA	CAUGCUCUCUCUCUUCUGUCA	Cleavage
tae-miR156	Traes_6AS_99C6A3B0B.3	PF03110				GO:GO:0005634,GO:GO:0003677	AT5G43270.2	squamosa promoter binding protein-like 2	LOC_Os02g04680.2	OsSPL3 - SBP-box gene family member, expressed	1,00	-1.0	1	21	1477	1497	UGACAGAAGAGAGUGAGCACA	CAUGCUCUCUCUCUUCUGUCA	Cleavage
tae-miR156	Traes_6AS_99C6A3B0B.4	PF03110				GO:GO:0005634,GO:GO:0003677	AT5G43270.2	squamosa promoter binding protein-like 2	LOC_Os02g04680.2	OsSPL3 - SBP-box gene family member, expressed	1,00	-1.0	1	21	1156	1176	UGACAGAAGAGAGUGAGCACA	CAUGCUCUCUCUCUUCUGUCA	Cleavage
tae-miR156	Traes_6AS_99C6A3B0B.5	PF03110				GO:GO:0005634,GO:GO:0003677	AT5G43270.3	squamosa promoter binding protein-like 2	LOC_Os02g04680.2	OsSPL3 - SBP-box gene family member, expressed	1,00	-1.0	1	21	1156	1176	UGACAGAAGAGAGUGAGCACA	CAUGCUCUCUCUCUUCUGUCA	Cleavage
tae-miR156	Traes_6AS_99C6A3B0B.6	PF03110				GO:GO:0005634,GO:GO:0003677	AT5G43270.1	squamosa promoter binding protein-like 2	LOC_Os02g04680.2	OsSPL3 - SBP-box gene family member, expressed	1,00	-1.0	1	21	1156	1176	UGACAGAAGAGAGUGAGCACA	CAUGCUCUCUCUCUUCUGUCA	Cleavage
tae-miR156	Traes_6AS_99C6A3B0B.7	PF03110				GO:GO:0005634,GO:GO:0003677	AT5G43270.3	squamosa promoter binding protein-like 2	LOC_Os02g04680.2	OsSPL3 - SBP-box gene family member, expressed	1,00	-1.0	1	21	1156	1176	UGACAGAAGAGAGUGAGCACA	CAUGCUCUCUCUCUUCUGUCA	Cleavage
tae-miR156	Traes_6AS_99C6A3B0B.8	PF03110				GO:GO:0005634,GO:GO:0003677	AT5G43270.1	squamosa promoter binding protein-like 2	LOC_Os02g04680.2	OsSPL3 - SBP-box gene family member, expressed	1,00	-1.0	1	21	1156	1176	UGACAGAAGAGAGUGAGCACA	CAUGCUCUCUCUCUUCUGUCA	Cleavage
tae-miR9662a-3p	Traes_6AS_A2EAE01C1.1	PF02536				GO:GO:0006355,GO:GO:0005739,GO:GO:0003690	AT5G07900.1	Mitochondrial transcription termination factor family protein	LOC_Os06g12100.1	mTERF family protein, expressed	2,05	-1.0	1	21	465	485	UUGAACAUCCCAGAGCCACCG	CCGUGGCUCUGGAAUGUUCAG	Cleavage
tae-miR9662b-3p	Traes_6AS_A2EAE01C1.1	PF02536				GO:GO:0006355,GO:GO:0005739,GO:GO:0003690	AT5G07900.1	Mitochondrial transcription termination factor family protein	LOC_Os06g12100.1	mTERF family protein, expressed	2,05	-1.0	1	21	464	484	UGAACAUCCCAGAGCCACCGG	CCCGUGGCUCUGGAAUGUUCA	Cleavage
tae-miR9662a-3p	Traes_6AS_A2EAE01C1.2	PF02536				GO:GO:0006355,GO:GO:0005739,GO:GO:0003690	AT5G07900.1	Mitochondrial transcription termination factor family protein	LOC_Os06g12100.1	mTERF family protein, expressed	2,05	-1.0	1	21	66	86	UUGAACAUCCCAGAGCCACCG	CCGUGGCUCUGGAAUGUUCAG	Cleavage
tae-miR9662b-3p	Traes_6AS_A2EAE01C1.2	PF02536				GO:GO:0006355,GO:GO:0005739,GO:GO:0003690	AT5G07900.1	Mitochondrial transcription termination factor family protein	LOC_Os06g12100.1	mTERF family protein, expressed	2,05	-1.0	1	21	65	85	UGAACAUCCCAGAGCCACCGG	CCCGUGGCUCUGGAAUGUUCA	Cleavage
tae-miR9662a-3p	Traes_6AS_B24138E62.1	PF02536				GO:GO:0006355,GO:GO:0005739,GO:GO:0003690	AT5G07900.1	Mitochondrial transcription termination factor family protein	LOC_Os06g12100.1	mTERF family protein, expressed	2,05	-1.0	1	21	375	395	UUGAACAUCCCAGAGCCACCG	CCGUGGCUCUGGAAUGUUCAG	Cleavage
tae-miR9662b-3p	Traes_6AS_B24138E62.1	PF02536				GO:GO:0006355,GO:GO:0005739,GO:GO:0003690	AT5G07900.1	Mitochondrial transcription termination factor family protein	LOC_Os06g12100.1	mTERF family protein, expressed	2,05	-1.0	1	21	374	394	UGAACAUCCCAGAGCCACCGG	CCCGUGGCUCUGGAAUGUUCA	Cleavage
tae-miR9662a-3p	Traes_6AS_EBADF37C7.1	PF02536				GO:GO:0006355,GO:GO:0005739,GO:GO:0003690	AT5G07900.1	Mitochondrial transcription termination factor family protein	LOC_Os06g12100.1	mTERF family protein, expressed	2,05	-1.0	1	21	681	701	UUGAACAUCCCAGAGCCACCG	CCGUGGCUCUGGGAUGCUCAG	Cleavage
tae-miR9662b-3p	Traes_6AS_EBADF37C7.1	PF02536				GO:GO:0006355,GO:GO:0005739,GO:GO:0003690	AT5G07900.1	Mitochondrial transcription termination factor family protein	LOC_Os06g12100.1	mTERF family protein, expressed	2,05	-1.0	1	21	680	700	UGAACAUCCCAGAGCCACCGG	CCCGUGGCUCUGGGAUGCUCA	Cleavage
tae-miR9662a-3p	Traes_6AS_EBADF37C7.2	PF02536				GO:GO:0006355,GO:GO:0005739,GO:GO:0003690	AT5G07900.1	Mitochondrial transcription termination factor family protein	LOC_Os06g12100.1	mTERF family protein, expressed	2,05	-1.0	1	21	378	398	UUGAACAUCCCAGAGCCACCG	CCGUGGCUCUGGGAUGCUCAG	Cleavage
tae-miR9662b-3p	Traes_6AS_EBADF37C7.2	PF02536				GO:GO:0006355,GO:GO:0005739,GO:GO:0003690	AT5G07900.1	Mitochondrial transcription termination factor family protein	LOC_Os06g12100.1	mTERF family protein, expressed	2,05	-1.0	1	21	377	397	UGAACAUCCCAGAGCCACCGG	CCCGUGGCUCUGGGAUGCUCA	Cleavage
tae-miR9662a-3p	Traes_6AS_EE73C76541.2	PF02536				GO:GO:0006355,GO:GO:0005739,GO:GO:0003690	AT5G07900.1	Mitochondrial transcription termination factor family protein	LOC_Os06g12100.1	mTERF family protein, expressed	1,00	-1.0	1	21	420	440	UUGAACAUCCCAGAGCCACCG	CCGUGGCUCUGGGAUGUUCAG	Cleavage
tae-miR9662b-3p	Traes_6AS_EE73C76541.2	PF02536				GO:GO:0006355,GO:GO:0005739,GO:GO:0003690	AT5G07900.1	Mitochondrial transcription termination factor family protein	LOC_Os06g12100.1	mTERF family protein, expressed	1,00	-1.0	1	21	419	439	UGAACAUCCCAGAGCCACCGG	CCCGUGGCUCUGGGAUGUUCA	Cleavage
tae-miR9662a-3p	Traes_6AS_EE73C76541.6	PF02536				GO:GO:0006355,GO:GO:0005739,GO:GO:0003690	AT5G07900.1	Mitochondrial transcription termination factor family protein	LOC_Os06g12100.1	mTERF family protein, expressed	1,00	-1.0	1	21	66	86	UUGAACAUCCCAGAGCCACCG	CCGUGGCUCUGGGAUGUUCAG	Cleavage
tae-miR9662b-3p	Traes_6AS_EE73C76541.6	PF02536				GO:GO:0006355,GO:GO:0005739,GO:GO:0003690	AT5G07900.1	Mitochondrial transcription termination factor family protein	LOC_Os06g12100.1	mTERF family protein, expressed	1,00	-1.0	1	21	65	85	UGAACAUCCCAGAGCCACCGG	CCCGUGGCUCUGGGAUGUUCA	Cleavage
tae-miR9662a-3p	Traes_6AS_EE8BA0A57.1	PF02536				GO:GO:0006355,GO:GO:0005739,GO:GO:0003690	AT1G21150.1	Mitochondrial transcription termination factor family protein	LOC_Os06g12100.1	mTERF family protein, expressed	2,00	-1.0	1	21	462	482	UUGAACAUCCCAGAGCCACCG	CCGUGCCUCUGGGAUGUUCAG	Cleavage
tae-miR9662b-3p	Traes_6AS_EE8BA0A57.1	PF02536				GO:GO:0006355,GO:GO:0005739,GO:GO:0003690	AT1G21150.1	Mitochondrial transcription termination factor family protein	LOC_Os06g12100.1	mTERF family protein, expressed	2,00	-1.0	1	21	461	481	UGAACAUCCCAGAGCCACCGG	CCCGUGCCUCUGGGAUGUUCA	Cleavage
tae-miR9662a-3p	Traes_6AS_EE8BA0A57.2	PF02536				GO:GO:0006355,GO:GO:0005739,GO:GO:0003690	AT5G07900.1	Mitochondrial transcription termination factor family protein	LOC_Os06g12100.1	mTERF family protein, expressed	2,00	-1.0	1	21	510	530	UUGAACAUCCCAGAGCCACCG	CCGUGCCUCUGGGAUGUUCAG	Cleavage
tae-miR9662b-3p	Traes_6AS_EE8BA0A57.2	PF02536				GO:GO:0006355,GO:GO:0005739,GO:GO:0003690	AT5G07900.1	Mitochondrial transcription termination factor family protein	LOC_Os06g12100.1	mTERF family protein, expressed	2,00	-1.0	1	21	509	529	UGAACAUCCCAGAGCCACCGG	CCCGUGCCUCUGGGAUGUUCA	Cleavage
tae-miR5049-3p	Traes_6AS_F5DBB6DBD.3										1,05	-1.0	1	21	1927	1947	AAUAUGGAUCGGAGGGAGUAC	UUACUCCCUCCAAUCCAUAUU	Translation
tae-miR9662a-3p	Traes_6AS_FBBCFBCB0.1	PF02536	KOG1267			GO:GO:0006355,GO:GO:0005739,GO:GO:0003690	AT5G07900.1	Mitochondrial transcription termination factor family protein	LOC_Os06g12100.1	mTERF family protein, expressed	2,05	-1.0	1	21	681	701	UUGAACAUCCCAGAGCCACCG	CCGUGGCUCUGGGAUGUUUAG	Cleavage
tae-miR9662b-3p	Traes_6AS_FBBCFBCB0.1	PF02536	KOG1267			GO:GO:0006355,GO:GO:0005739,GO:GO:0003690	AT5G07900.1	Mitochondrial transcription termination factor family protein	LOC_Os06g12100.1	mTERF family protein, expressed	2,05	-1.0	1	21	680	700	UGAACAUCCCAGAGCCACCGG	CCCGUGGCUCUGGGAUGUUUA	Cleavage
tae-miR1127b-3p	Traes_6BL_028068EFA.2										1,05	-1.0	1	21	2801	2821	ACAAGUAUUUCUGGACGGAGG	CCUCCGUCCGGAAAUACUUGU	Cleavage
tae-miR6197-5p	Traes_6BL_67725F06B.2										3,00	-1.0	1	21	278	298	UCUGUAAACAAAUGUAGGACG	GAACCUCCAUUUGUUUACAGG	Cleavage
tae-miR1127b-3p	Traes_6BL_731ED1C4C.1										1,05	-1.0	1	21	1020	1040	ACAAGUAUUUCUGGACGGAGG	UCUCCGUCCAAAAAUACUUGU	Translation
tae-miR1130b-3p	Traes_6BL_7BD68BAC2.3										1,05	-1.0	1	21	215	235	UCUUAUAUUAUGGGACGGAGG	CCUCCGUCCCAUAAUGUAAGA	Cleavage
tae-miR1122a	Traes_6BL_7E9C131F4.3										0,00	-1.0	1	20	761	780	UAGAUACAUCCGUAUCUAGA	UCUAGAUACGGAUGUAUCUA	Cleavage
tae-miR1130b-3p	Traes_6BL_887005D55.2										1,05	-1.0	1	21	79	99	UCUUAUAUUAUGGGACGGAGG	CCUCCGUCCCAUAAUAUAAUA	Cleavage
tae-miR1118	Traes_6BL_E14604962.2										2,00	-1.0	1	23	35	57	CACUACAUUAUGGAAUGGAGGGA	UCCCUCUGUUCCAUAAUGUAGUG	Cleavage
tae-miR1118	Traes_6BL_E14604962.3										2,00	-1.0	1	23	35	57	CACUACAUUAUGGAAUGGAGGGA	UCCCUCUGUUCCAUAAUGUAGUG	Cleavage
tae-miR1127b-3p	Traes_6BS_1AFDF35E5.3										2,05	-1.0	1	21	171	191	ACAAGUAUUUCUGGACGGAGG	CUUUCGUCCGGAAAUACUUGU	Cleavage
tae-miR9662a-3p	Traes_6BS_28F0D2955.1	PF02536				GO:GO:0006355,GO:GO:0005739,GO:GO:0003690	AT5G64950.1	Mitochondrial transcription termination factor family protein	LOC_Os06g12100.1	mTERF family protein, expressed	2,05	-1.0	1	21	396	416	UUGAACAUCCCAGAGCCACCG	CUGUGGCUCUCGGAUGUUCAG	Translation
tae-miR9662b-3p	Traes_6BS_28F0D2955.1	PF02536				GO:GO:0006355,GO:GO:0005739,GO:GO:0003690	AT5G64950.1	Mitochondrial transcription termination factor family protein	LOC_Os06g12100.1	mTERF family protein, expressed	2,05	-1.0	1	21	395	415	UGAACAUCCCAGAGCCACCGG	CCUGUGGCUCUCGGAUGUUCA	Translation
tae-miR9662a-3p	Traes_6BS_2AC6A2287.1	PF02536				GO:GO:0006355,GO:GO:0005739,GO:GO:0003690	AT5G07900.1	Mitochondrial transcription termination factor family protein	LOC_Os06g12100.1	mTERF family protein, expressed	2,05	-1.0	1	21	66	86	UUGAACAUCCCAGAGCCACCG	CCGUGGCUCUGGGAUGCUCAG	Cleavage
tae-miR9662b-3p	Traes_6BS_2AC6A2287.1	PF02536				GO:GO:0006355,GO:GO:0005739,GO:GO:0003690	AT5G07900.1	Mitochondrial transcription termination factor family protein	LOC_Os06g12100.1	mTERF family protein, expressed	2,05	-1.0	1	21	65	85	UGAACAUCCCAGAGCCACCGG	CCCGUGGCUCUGGGAUGCUCA	Cleavage
tae-miR9662a-3p	Traes_6BS_2F108C5BB.1	PF02536				GO:GO:0006355,GO:GO:0005739,GO:GO:0003690	AT5G07900.1	Mitochondrial transcription termination factor family protein	LOC_Os06g12100.1	mTERF family protein, expressed	2,00	-1.0	1	21	435	455	UUGAACAUCCCAGAGCCACCG	UCGUGGCGCUGGGAUGUUCAG	Cleavage
tae-miR9662b-3p	Traes_6BS_2F108C5BB.1	PF02536				GO:GO:0006355,GO:GO:0005739,GO:GO:0003690	AT5G07900.1	Mitochondrial transcription termination factor family protein	LOC_Os06g12100.1	mTERF family protein, expressed	2,05	-1.0	1	21	434	454	UGAACAUCCCAGAGCCACCGG	CUCGUGGCGCUGGGAUGUUCA	Cleavage
tae-miR408	Traes_6BS_348308070.13										2,05	-1.0	1	21	2	22	CUGCACUGCCUCUUCCCUGGC	CCCAGGGCCGAGGCAGUGCAG	Cleavage
tae-miR408	Traes_6BS_348308070.15										2,05	-1.0	1	21	11	31	CUGCACUGCCUCUUCCCUGGC	CCCAGGGCCGAGGCAGUGCAG	Cleavage
tae-miR408	Traes_6BS_348308070.17										2,05	-1.0	1	21	14	34	CUGCACUGCCUCUUCCCUGGC	CCCAGGGCCGAGGCAGUGCAG	Cleavage
tae-miR408	Traes_6BS_348308070.19										2,05	-1.0	1	21	11	31	CUGCACUGCCUCUUCCCUGGC	CCCAGGGCCGAGGCAGUGCAG	Cleavage
tae-miR408	Traes_6BS_348308070.2										2,05	-1.0	1	21	14	34	CUGCACUGCCUCUUCCCUGGC	CCCAGGGCCGAGGCAGUGCAG	Cleavage
tae-miR408	Traes_6BS_348308070.3										2,05	-1.0	1	21	17	37	CUGCACUGCCUCUUCCCUGGC	CCCAGGGCCGAGGCAGUGCAG	Cleavage
tae-miR408	Traes_6BS_348308070.5										2,05	-1.0	1	21	17	37	CUGCACUGCCUCUUCCCUGGC	CCCAGGGCCGAGGCAGUGCAG	Cleavage
tae-miR408	Traes_6BS_348308070.6										2,05	-1.0	1	21	14	34	CUGCACUGCCUCUUCCCUGGC	CCCAGGGCCGAGGCAGUGCAG	Cleavage
tae-miR408	Traes_6BS_348308070.8										2,05	-1.0	1	21	17	37	CUGCACUGCCUCUUCCCUGGC	CCCAGGGCCGAGGCAGUGCAG	Cleavage
tae-miR408	Traes_6BS_348308070.9										2,05	-1.0	1	21	17	37	CUGCACUGCCUCUUCCCUGGC	CCCAGGGCCGAGGCAGUGCAG	Cleavage
tae-miR9662a-3p	Traes_6BS_3A00751B5.1	PF02536				GO:GO:0006355,GO:GO:0005739,GO:GO:0003690	AT5G07900.1	Mitochondrial transcription termination factor family protein	LOC_Os06g12100.1	mTERF family protein, expressed	2,05	-1.0	1	21	69	89	UUGAACAUCCCAGAGCCACCG	CCGUGGCUCUGGGAUGUUUAG	Cleavage
tae-miR9662b-3p	Traes_6BS_3A00751B5.1	PF02536				GO:GO:0006355,GO:GO:0005739,GO:GO:0003690	AT5G07900.1	Mitochondrial transcription termination factor family protein	LOC_Os06g12100.1	mTERF family protein, expressed	2,05	-1.0	1	21	68	88	UGAACAUCCCAGAGCCACCGG	CCCGUGGCUCUGGGAUGUUUA	Cleavage
tae-miR9662a-3p	Traes_6BS_3A00751B5.2	PF02536				GO:GO:0006355,GO:GO:0005739,GO:GO:0003690	AT5G07900.1	Mitochondrial transcription termination factor family protein	LOC_Os06g12100.1	mTERF family protein, expressed	2,05	-1.0	1	21	69	89	UUGAACAUCCCAGAGCCACCG	CCGUGGCUCUGGGAUGUUUAG	Cleavage
tae-miR9662b-3p	Traes_6BS_3A00751B5.2	PF02536				GO:GO:0006355,GO:GO:0005739,GO:GO:0003690	AT5G07900.1	Mitochondrial transcription termination factor family protein	LOC_Os06g12100.1	mTERF family protein, expressed	2,05	-1.0	1	21	68	88	UGAACAUCCCAGAGCCACCGG	CCCGUGGCUCUGGGAUGUUUA	Cleavage
tae-miR9662a-3p	Traes_6BS_46641470F.1	PF02536				GO:GO:0006355,GO:GO:0005739,GO:GO:0003690	AT1G21150.1	Mitochondrial transcription termination factor family protein	LOC_Os06g12100.1	mTERF family protein, expressed	2,05	-1.0	1	21	33	53	UUGAACAUCCCAGAGCCACCG	CCGUGGCUCUGGGAUGUUUAG	Cleavage
tae-miR9662b-3p	Traes_6BS_46641470F.1	PF02536				GO:GO:0006355,GO:GO:0005739,GO:GO:0003690	AT1G21150.1	Mitochondrial transcription termination factor family protein	LOC_Os06g12100.1	mTERF family protein, expressed	2,05	-1.0	1	21	32	52	UGAACAUCCCAGAGCCACCGG	CCCGUGGCUCUGGGAUGUUUA	Cleavage
tae-miR9662a-3p	Traes_6BS_4E6E57FEB.1	PF02536				GO:GO:0006355,GO:GO:0005739,GO:GO:0003690	AT5G07900.1	Mitochondrial transcription termination factor family protein	LOC_Os06g12100.1	mTERF family protein, expressed	3,00	-1.0	1	21	65	86	UUGAACAUCCCAGAGCCAC-CG	CGCGUGGCUCUGGGAUGUUCAG	Cleavage
tae-miR9662b-3p	Traes_6BS_4E6E57FEB.1	PF02536				GO:GO:0006355,GO:GO:0005739,GO:GO:0003690	AT5G07900.1	Mitochondrial transcription termination factor family protein	LOC_Os06g12100.1	mTERF family protein, expressed	2,00	-1.0	1	21	64	85	UGAACAUCCCAGAGCCAC-CGG	CCGCGUGGCUCUGGGAUGUUCA	Cleavage
tae-miR9662a-3p	Traes_6BS_4E6E57FEB.2	PF02536				GO:GO:0006355,GO:GO:0005739,GO:GO:0003690	AT5G07900.1	Mitochondrial transcription termination factor family protein	LOC_Os06g12100.1	mTERF family protein, expressed	3,00	-1.0	1	21	65	86	UUGAACAUCCCAGAGCCAC-CG	CGCGUGGCUCUGGGAUGUUCAG	Cleavage
tae-miR9662b-3p	Traes_6BS_4E6E57FEB.2	PF02536				GO:GO:0006355,GO:GO:0005739,GO:GO:0003690	AT5G07900.1	Mitochondrial transcription termination factor family protein	LOC_Os06g12100.1	mTERF family protein, expressed	2,00	-1.0	1	21	64	85	UGAACAUCCCAGAGCCAC-CGG	CCGCGUGGCUCUGGGAUGUUCA	Cleavage
tae-miR9662a-3p	Traes_6BS_4E6E57FEB.3	PF02536				GO:GO:0006355,GO:GO:0005739,GO:GO:0003690	AT5G07900.1	Mitochondrial transcription termination factor family protein	LOC_Os06g12100.1	mTERF family protein, expressed	3,00	-1.0	1	21	65	86	UUGAACAUCCCAGAGCCAC-CG	CGCGUGGCUCUGGGAUGUUCAG	Cleavage
tae-miR9662b-3p	Traes_6BS_4E6E57FEB.3	PF02536				GO:GO:0006355,GO:GO:0005739,GO:GO:0003690	AT5G07900.1	Mitochondrial transcription termination factor family protein	LOC_Os06g12100.1	mTERF family protein, expressed	2,00	-1.0	1	21	64	85	UGAACAUCCCAGAGCCAC-CGG	CCGCGUGGCUCUGGGAUGUUCA	Cleavage
tae-miR9662b-3p	Traes_6BS_508C2BEA2.1										3,00	-1.0	1	21	32	52	UGAACAUCCCAGAGCCACCGG	CCUGUGGUUCUGGGAUGUUCG	Cleavage
tae-miR156	Traes_6BS_542961EA4.1								LOC_Os06g45310.1	OsSPL11 - SBP-box gene family member, expressed	1,00	-1.0	1	21	283	303	UGACAGAAGAGAGUGAGCACA	CGUGCUCUCUCUCUUCUGUCA	Cleavage
tae-miR156	Traes_6BS_542961EA4.2								LOC_Os06g45310.1	OsSPL11 - SBP-box gene family member, expressed	1,00	-1.0	1	21	349	369	UGACAGAAGAGAGUGAGCACA	CGUGCUCUCUCUCUUCUGUCA	Cleavage
tae-miR1122b-3p	Traes_6BS_7965FEB75.1										2,05	-1.0	1	21	87	107	AGACUUAUAUGUAGGAACGGA	UCCGUUCGUAUAUAUAAGUCU	Translation
tae-miR9662a-3p	Traes_6BS_7F1846171.1	PF02536				GO:GO:0006355,GO:GO:0005739,GO:GO:0003690	AT5G07900.1	Mitochondrial transcription termination factor family protein	LOC_Os06g12100.1	mTERF family protein, expressed	2,05	-1.0	1	21	33	53	UUGAACAUCCCAGAGCCACCG	CUGUGGCUCUCGGAUGUUCAG	Translation
tae-miR9662b-3p	Traes_6BS_7F1846171.1	PF02536				GO:GO:0006355,GO:GO:0005739,GO:GO:0003690	AT5G07900.1	Mitochondrial transcription termination factor family protein	LOC_Os06g12100.1	mTERF family protein, expressed	2,05	-1.0	1	21	32	52	UGAACAUCCCAGAGCCACCGG	CCUGUGGCUCUCGGAUGUUCA	Translation
tae-miR9662a-3p	Traes_6BS_82F5BF11F.1	PF02536				GO:GO:0006355,GO:GO:0005739,GO:GO:0003690	AT1G79220.1	Mitochondrial transcription termination factor family protein	LOC_Os06g12100.1	mTERF family protein, expressed	2,00	-1.0	1	21	267	287	UUGAACAUCCCAGAGCCACCG	CCGUGCCUCUGGGAUGUUCAG	Cleavage
tae-miR9662b-3p	Traes_6BS_82F5BF11F.1	PF02536				GO:GO:0006355,GO:GO:0005739,GO:GO:0003690	AT1G79220.1	Mitochondrial transcription termination factor family protein	LOC_Os06g12100.1	mTERF family protein, expressed	2,00	-1.0	1	21	266	286	UGAACAUCCCAGAGCCACCGG	CCCGUGCCUCUGGGAUGUUCA	Cleavage
tae-miR9662a-3p	Traes_6BS_843505CB3.1	PF02536				GO:GO:0006355,GO:GO:0005739,GO:GO:0003690	AT1G21150.1	Mitochondrial transcription termination factor family protein	LOC_Os06g12100.1	mTERF family protein, expressed	2,05	-1.0	1	21	69	89	UUGAACAUCCCAGAGCCACCG	CCGUGGCUCUGGAAUGUUCAG	Cleavage
tae-miR9662b-3p	Traes_6BS_843505CB3.1	PF02536				GO:GO:0006355,GO:GO:0005739,GO:GO:0003690	AT1G21150.1	Mitochondrial transcription termination factor family protein	LOC_Os06g12100.1	mTERF family protein, expressed	2,05	-1.0	1	21	68	88	UGAACAUCCCAGAGCCACCGG	CCCGUGGCUCUGGAAUGUUCA	Cleavage
tae-miR9662a-3p	Traes_6BS_843505CB3.2	PF02536				GO:GO:0006355,GO:GO:0005739,GO:GO:0003690	AT1G21150.1	Mitochondrial transcription termination factor family protein	LOC_Os06g12100.1	mTERF family protein, expressed	2,05	-1.0	1	21	69	89	UUGAACAUCCCAGAGCCACCG	CCGUGGCUCUGGAAUGUUCAG	Cleavage
tae-miR9662b-3p	Traes_6BS_843505CB3.2	PF02536				GO:GO:0006355,GO:GO:0005739,GO:GO:0003690	AT1G21150.1	Mitochondrial transcription termination factor family protein	LOC_Os06g12100.1	mTERF family protein, expressed	2,05	-1.0	1	21	68	88	UGAACAUCCCAGAGCCACCGG	CCCGUGGCUCUGGAAUGUUCA	Cleavage
tae-miR9662a-3p	Traes_6BS_843505CB3.3	PF02536				GO:GO:0006355,GO:GO:0005739,GO:GO:0003690	AT1G21150.1	Mitochondrial transcription termination factor family protein	LOC_Os06g12100.1	mTERF family protein, expressed	2,05	-1.0	1	21	69	89	UUGAACAUCCCAGAGCCACCG	CCGUGGCUCUGGAAUGUUCAG	Cleavage
tae-miR9662b-3p	Traes_6BS_843505CB3.3	PF02536				GO:GO:0006355,GO:GO:0005739,GO:GO:0003690	AT1G21150.1	Mitochondrial transcription termination factor family protein	LOC_Os06g12100.1	mTERF family protein, expressed	2,05	-1.0	1	21	68	88	UGAACAUCCCAGAGCCACCGG	CCCGUGGCUCUGGAAUGUUCA	Cleavage
tae-miR9662a-3p	Traes_6BS_843505CB3.4	PF02536				GO:GO:0006355,GO:GO:0005739,GO:GO:0003690	AT1G21150.1	Mitochondrial transcription termination factor family protein	LOC_Os06g12100.1	mTERF family protein, expressed	2,05	-1.0	1	21	69	89	UUGAACAUCCCAGAGCCACCG	CCGUGGCUCUGGAAUGUUCAG	Cleavage
tae-miR9662b-3p	Traes_6BS_843505CB3.4	PF02536				GO:GO:0006355,GO:GO:0005739,GO:GO:0003690	AT1G21150.1	Mitochondrial transcription termination factor family protein	LOC_Os06g12100.1	mTERF family protein, expressed	2,05	-1.0	1	21	68	88	UGAACAUCCCAGAGCCACCGG	CCCGUGGCUCUGGAAUGUUCA	Cleavage
tae-miR9662a-3p	Traes_6BS_843505CB3.5	PF02536				GO:GO:0006355,GO:GO:0005739,GO:GO:0003690	AT1G21150.1	Mitochondrial transcription termination factor family protein	LOC_Os06g12100.1	mTERF family protein, expressed	2,05	-1.0	1	21	69	89	UUGAACAUCCCAGAGCCACCG	CCGUGGCUCUGGAAUGUUCAG	Cleavage
tae-miR9662b-3p	Traes_6BS_843505CB3.5	PF02536				GO:GO:0006355,GO:GO:0005739,GO:GO:0003690	AT1G21150.1	Mitochondrial transcription termination factor family protein	LOC_Os06g12100.1	mTERF family protein, expressed	2,05	-1.0	1	21	68	88	UGAACAUCCCAGAGCCACCGG	CCCGUGGCUCUGGAAUGUUCA	Cleavage
tae-miR9662a-3p	Traes_6BS_843505CB3.6	PF02536				GO:GO:0006355,GO:GO:0005739,GO:GO:0003690	AT1G21150.1	Mitochondrial transcription termination factor family protein	LOC_Os06g12100.1	mTERF family protein, expressed	2,05	-1.0	1	21	69	89	UUGAACAUCCCAGAGCCACCG	CCGUGGCUCUGGAAUGUUCAG	Cleavage
tae-miR9662b-3p	Traes_6BS_843505CB3.6	PF02536				GO:GO:0006355,GO:GO:0005739,GO:GO:0003690	AT1G21150.1	Mitochondrial transcription termination factor family protein	LOC_Os06g12100.1	mTERF family protein, expressed	2,05	-1.0	1	21	68	88	UGAACAUCCCAGAGCCACCGG	CCCGUGGCUCUGGAAUGUUCA	Cleavage
tae-miR9662a-3p	Traes_6BS_843505CB3.7	PF02536				GO:GO:0006355,GO:GO:0005739,GO:GO:0003690	AT1G21150.1	Mitochondrial transcription termination factor family protein	LOC_Os06g12100.1	mTERF family protein, expressed	2,05	-1.0	1	21	69	89	UUGAACAUCCCAGAGCCACCG	CCGUGGCUCUGGAAUGUUCAG	Cleavage
tae-miR9662b-3p	Traes_6BS_843505CB3.7	PF02536				GO:GO:0006355,GO:GO:0005739,GO:GO:0003690	AT1G21150.1	Mitochondrial transcription termination factor family protein	LOC_Os06g12100.1	mTERF family protein, expressed	2,05	-1.0	1	21	68	88	UGAACAUCCCAGAGCCACCGG	CCCGUGGCUCUGGAAUGUUCA	Cleavage
tae-miR1127b-3p	Traes_6BS_94CE86A01.2										2,05	-1.0	1	21	763	783	ACAAGUAUUUCUGGACGGAGG	AAUCCGUUCAGAAUUACUUGU	Cleavage
tae-miR1127a	Traes_6BS_94CE86A01.2										1,05	-1.0	1	19	842	860	UCCUUCCGUUCGGAAUUAC	GUAAUUCCGAACGGAGGGA	Cleavage
tae-miR9662a-3p	Traes_6BS_97770F04E.1	PF02536				GO:GO:0006355,GO:GO:0005739,GO:GO:0003690	AT5G07900.1	Mitochondrial transcription termination factor family protein	LOC_Os06g12100.1	mTERF family protein, expressed	2,05	-1.0	1	21	33	53	UUGAACAUCCCAGAGCCACCG	UCGUGGCUCUGGAAUGUUCAG	Cleavage
tae-miR9662b-3p	Traes_6BS_97770F04E.1	PF02536				GO:GO:0006355,GO:GO:0005739,GO:GO:0003690	AT5G07900.1	Mitochondrial transcription termination factor family protein	LOC_Os06g12100.1	mTERF family protein, expressed	2,05	-1.0	1	21	32	52	UGAACAUCCCAGAGCCACCGG	CUCGUGGCUCUGGAAUGUUCA	Cleavage
tae-miR9662a-3p	Traes_6BS_97770F04E.3	PF02536				GO:GO:0006355,GO:GO:0005739,GO:GO:0003690	AT5G07900.1	Mitochondrial transcription termination factor family protein	LOC_Os06g12100.1	mTERF family protein, expressed	2,05	-1.0	1	21	33	53	UUGAACAUCCCAGAGCCACCG	UCGUGGCUCUGGAAUGUUCAG	Cleavage
tae-miR9662b-3p	Traes_6BS_97770F04E.3	PF02536				GO:GO:0006355,GO:GO:0005739,GO:GO:0003690	AT5G07900.1	Mitochondrial transcription termination factor family protein	LOC_Os06g12100.1	mTERF family protein, expressed	2,05	-1.0	1	21	32	52	UGAACAUCCCAGAGCCACCGG	CUCGUGGCUCUGGAAUGUUCA	Cleavage
tae-miR9662a-3p	Traes_6BS_97770F04E.4	PF02536				GO:GO:0006355,GO:GO:0005739,GO:GO:0003690	AT5G07900.1	Mitochondrial transcription termination factor family protein	LOC_Os06g12100.1	mTERF family protein, expressed	2,05	-1.0	1	21	33	53	UUGAACAUCCCAGAGCCACCG	UCGUGGCUCUGGAAUGUUCAG	Cleavage
tae-miR9662b-3p	Traes_6BS_97770F04E.4	PF02536				GO:GO:0006355,GO:GO:0005739,GO:GO:0003690	AT5G07900.1	Mitochondrial transcription termination factor family protein	LOC_Os06g12100.1	mTERF family protein, expressed	2,05	-1.0	1	21	32	52	UGAACAUCCCAGAGCCACCGG	CUCGUGGCUCUGGAAUGUUCA	Cleavage
tae-miR9662a-3p	Traes_6BS_97770F04E.5	PF02536				GO:GO:0006355,GO:GO:0005739,GO:GO:0003690	AT5G07900.1	Mitochondrial transcription termination factor family protein	LOC_Os06g12100.1	mTERF family protein, expressed	2,05	-1.0	1	21	33	53	UUGAACAUCCCAGAGCCACCG	UCGUGGCUCUGGAAUGUUCAG	Cleavage
tae-miR9662b-3p	Traes_6BS_97770F04E.5	PF02536				GO:GO:0006355,GO:GO:0005739,GO:GO:0003690	AT5G07900.1	Mitochondrial transcription termination factor family protein	LOC_Os06g12100.1	mTERF family protein, expressed	2,05	-1.0	1	21	32	52	UGAACAUCCCAGAGCCACCGG	CUCGUGGCUCUGGAAUGUUCA	Cleavage
tae-miR9662a-3p	Traes_6BS_97770F04E.6	PF02536				GO:GO:0006355,GO:GO:0005739,GO:GO:0003690	AT5G07900.1	Mitochondrial transcription termination factor family protein	LOC_Os06g12100.1	mTERF family protein, expressed	2,05	-1.0	1	21	33	53	UUGAACAUCCCAGAGCCACCG	UCGUGGCUCUGGAAUGUUCAG	Cleavage
tae-miR9662b-3p	Traes_6BS_97770F04E.6	PF02536				GO:GO:0006355,GO:GO:0005739,GO:GO:0003690	AT5G07900.1	Mitochondrial transcription termination factor family protein	LOC_Os06g12100.1	mTERF family protein, expressed	2,05	-1.0	1	21	32	52	UGAACAUCCCAGAGCCACCGG	CUCGUGGCUCUGGAAUGUUCA	Cleavage
tae-miR1137a	Traes_6BS_A4F1E63CE.2										2,00	-1.0	1	20	884	903	UAGUACAAAGUUGAGUCAUC	AGUGUCUCAACUUUGUACUA	Cleavage
tae-miR1137a	Traes_6BS_A4F1E63CE.3										2,00	-1.0	1	20	871	890	UAGUACAAAGUUGAGUCAUC	AGUGUCUCAACUUUGUACUA	Cleavage
tae-miR1128	Traes_6BS_BF5C480A9.12										2,05	-1.0	1	21	60	80	UACUACUCCCUCCGUCCGAAA	UUUGGGACGGAGGGAGUAUUA	Cleavage
tae-miR9662a-3p	Traes_6BS_C3247654A.1	PF02536				GO:GO:0006355,GO:GO:0005739,GO:GO:0003690	AT5G07900.1	Mitochondrial transcription termination factor family protein	LOC_Os06g12100.1	mTERF family protein, expressed	2,05	-1.0	1	21	57	77	UUGAACAUCCCAGAGCCACCG	CUGUGGCUCUGCGAUGUUCAG	Translation
tae-miR9662b-3p	Traes_6BS_C3247654A.1	PF02536				GO:GO:0006355,GO:GO:0005739,GO:GO:0003690	AT5G07900.1	Mitochondrial transcription termination factor family protein	LOC_Os06g12100.1	mTERF family protein, expressed	2,05	-1.0	1	21	56	76	UGAACAUCCCAGAGCCACCGG	CCUGUGGCUCUGCGAUGUUCA	Cleavage
tae-miR9662a-3p	Traes_6BS_CBD931158.1	PF02536				GO:GO:0006355,GO:GO:0005739,GO:GO:0003690	AT1G21150.1	Mitochondrial transcription termination factor family protein	LOC_Os06g12100.1	mTERF family protein, expressed	2,00	-1.0	1	21	33	53	UUGAACAUCCCAGAGCCACCG	CCGUGCCUCUGGGAUGUUCAG	Cleavage
tae-miR9662b-3p	Traes_6BS_CBD931158.1	PF02536				GO:GO:0006355,GO:GO:0005739,GO:GO:0003690	AT1G21150.1	Mitochondrial transcription termination factor family protein	LOC_Os06g12100.1	mTERF family protein, expressed	2,00	-1.0	1	21	32	52	UGAACAUCCCAGAGCCACCGG	CCCGUGCCUCUGGGAUGUUCA	Cleavage
tae-miR9662a-3p	Traes_6BS_CBD931158.2	PF02536				GO:GO:0006355,GO:GO:0005739,GO:GO:0003690	AT1G21150.1	Mitochondrial transcription termination factor family protein	LOC_Os06g12100.1	mTERF family protein, expressed	2,00	-1.0	1	21	33	53	UUGAACAUCCCAGAGCCACCG	CCGUGCCUCUGGGAUGUUCAG	Cleavage
tae-miR9662b-3p	Traes_6BS_CBD931158.2	PF02536				GO:GO:0006355,GO:GO:0005739,GO:GO:0003690	AT1G21150.1	Mitochondrial transcription termination factor family protein	LOC_Os06g12100.1	mTERF family protein, expressed	2,00	-1.0	1	21	32	52	UGAACAUCCCAGAGCCACCGG	CCCGUGCCUCUGGGAUGUUCA	Cleavage
tae-miR9662a-3p	Traes_6BS_CBD931158.3	PF02536				GO:GO:0006355,GO:GO:0005739,GO:GO:0003690	AT1G21150.1	Mitochondrial transcription termination factor family protein	LOC_Os06g12100.1	mTERF family protein, expressed	2,00	-1.0	1	21	33	53	UUGAACAUCCCAGAGCCACCG	CCGUGCCUCUGGGAUGUUCAG	Cleavage
tae-miR9662b-3p	Traes_6BS_CBD931158.3	PF02536				GO:GO:0006355,GO:GO:0005739,GO:GO:0003690	AT1G21150.1	Mitochondrial transcription termination factor family protein	LOC_Os06g12100.1	mTERF family protein, expressed	2,00	-1.0	1	21	32	52	UGAACAUCCCAGAGCCACCGG	CCCGUGCCUCUGGGAUGUUCA	Cleavage
tae-miR9662a-3p	Traes_6BS_CBD931158.4	PF02536				GO:GO:0006355,GO:GO:0005739,GO:GO:0003690	AT1G21150.1	Mitochondrial transcription termination factor family protein	LOC_Os06g12100.1	mTERF family protein, expressed	2,00	-1.0	1	21	33	53	UUGAACAUCCCAGAGCCACCG	CCGUGCCUCUGGGAUGUUCAG	Cleavage
tae-miR9662b-3p	Traes_6BS_CBD931158.4	PF02536				GO:GO:0006355,GO:GO:0005739,GO:GO:0003690	AT1G21150.1	Mitochondrial transcription termination factor family protein	LOC_Os06g12100.1	mTERF family protein, expressed	2,00	-1.0	1	21	32	52	UGAACAUCCCAGAGCCACCGG	CCCGUGCCUCUGGGAUGUUCA	Cleavage
tae-miR9662a-3p	Traes_6BS_CBD931158.5	PF02536				GO:GO:0006355,GO:GO:0005739,GO:GO:0003690	AT1G21150.1	Mitochondrial transcription termination factor family protein	LOC_Os06g12100.1	mTERF family protein, expressed	2,00	-1.0	1	21	33	53	UUGAACAUCCCAGAGCCACCG	CCGUGCCUCUGGGAUGUUCAG	Cleavage
tae-miR9662b-3p	Traes_6BS_CBD931158.5	PF02536				GO:GO:0006355,GO:GO:0005739,GO:GO:0003690	AT1G21150.1	Mitochondrial transcription termination factor family protein	LOC_Os06g12100.1	mTERF family protein, expressed	2,00	-1.0	1	21	32	52	UGAACAUCCCAGAGCCACCGG	CCCGUGCCUCUGGGAUGUUCA	Cleavage
tae-miR9662a-3p	Traes_6BS_CBD931158.6	PF02536				GO:GO:0006355,GO:GO:0005739,GO:GO:0003690	AT1G21150.1	Mitochondrial transcription termination factor family protein	LOC_Os06g12100.1	mTERF family protein, expressed	2,00	-1.0	1	21	33	53	UUGAACAUCCCAGAGCCACCG	CCGUGCCUCUGGGAUGUUCAG	Cleavage
tae-miR9662b-3p	Traes_6BS_CBD931158.6	PF02536				GO:GO:0006355,GO:GO:0005739,GO:GO:0003690	AT1G21150.1	Mitochondrial transcription termination factor family protein	LOC_Os06g12100.1	mTERF family protein, expressed	2,00	-1.0	1	21	32	52	UGAACAUCCCAGAGCCACCGG	CCCGUGCCUCUGGGAUGUUCA	Cleavage
tae-miR156	Traes_6BS_F50663DF7.1	PF03110				GO:GO:0005634,GO:GO:0003677	AT5G43270.1	squamosa promoter binding protein-like 2	LOC_Os02g04680.2	OsSPL3 - SBP-box gene family member, expressed	1,00	-1.0	1	21	1387	1407	UGACAGAAGAGAGUGAGCACA	CAUGCUCUCUCUCUUCUGUCA	Cleavage

tae-miR9662a-3p	Traes_6BS_F8176B2AA.1	PF02536				GO:GO:0006355,GO:GO:0005739,GO:GO:0003690	AT5G64950.1	Mitochondrial transcription termination factor family protein	LOC_Os06g12100.1	mTERF family protein, expressed	2,05	-1.0	1	21	204	224	UUGAACAUCCCAGAGCCACCG	CCGUGGCUCUGCGAUGUUCAG	Translation
tae-miR9662b-3p	Traes_6BS_F8176B2AA.1	PF02536				GO:GO:0006355,GO:GO:0005739,GO:GO:0003690	AT5G64950.1	Mitochondrial transcription termination factor family protein	LOC_Os06g12100.1	mTERF family protein, expressed	2,05	-1.0	1	21	203	223	UGAACAUCCCAGAGCCACCGG	CCCGUGGCUCUGCGAUGUUCA	Cleavage
tae-miR9662a-3p	Traes_6BS_F8176B2AA.2	PF02536				GO:GO:0006355,GO:GO:0005739,GO:GO:0003690	AT5G64950.1	Mitochondrial transcription termination factor family protein	LOC_Os06g12100.1	mTERF family protein, expressed	2,05	-1.0	1	21	204	224	UUGAACAUCCCAGAGCCACCG	CCGUGGCUCUGCGAUGUUCAG	Translation
tae-miR9662b-3p	Traes_6BS_F8176B2AA.2	PF02536				GO:GO:0006355,GO:GO:0005739,GO:GO:0003690	AT5G64950.1	Mitochondrial transcription termination factor family protein	LOC_Os06g12100.1	mTERF family protein, expressed	2,05	-1.0	1	21	203	223	UGAACAUCCCAGAGCCACCGG	CCCGUGGCUCUGCGAUGUUCA	Cleavage
tae-miR9662a-3p	Traes_6BS_F8176B2AA.3	PF02536				GO:GO:0006355,GO:GO:0005739,GO:GO:0003690	AT5G64950.1	Mitochondrial transcription termination factor family protein	LOC_Os06g12100.1	mTERF family protein, expressed	2,05	-1.0	1	21	198	218	UUGAACAUCCCAGAGCCACCG	CCGUGGCUCUGCGAUGUUCAG	Translation
tae-miR9662b-3p	Traes_6BS_F8176B2AA.3	PF02536				GO:GO:0006355,GO:GO:0005739,GO:GO:0003690	AT5G64950.1	Mitochondrial transcription termination factor family protein	LOC_Os06g12100.1	mTERF family protein, expressed	2,05	-1.0	1	21	197	217	UGAACAUCCCAGAGCCACCGG	CCCGUGGCUCUGCGAUGUUCA	Cleavage
tae-miR5049-3p	Traes_6DL_1E6A51BD3.10										1,05	-1.0	1	21	1321	1341	AAUAUGGAUCGGAGGGAGUAC	AUACUCCCUCCGAUCCAUAAU	Cleavage
tae-miR1122b-3p	Traes_6DL_244C908B5.14										1,05	-1.0	1	21	584	604	AGACUUAUAUGUAGGAACGGA	UCCGUUCCUAAAUAUAAGUCU	Translation
tae-miR5384-3p	Traes_6DL_5282E0007.1										2,05	-1.0	1	21	1178	1198	UGAGCGCGCCGCCGUCGAAUG	UGUUCGACGGCGGCGCGGUCG	Cleavage
tae-miR5384-3p	Traes_6DL_5282E0007.2										2,05	-1.0	1	21	1016	1036	UGAGCGCGCCGCCGUCGAAUG	UGUUCGACGGCGGCGCGGUCG	Cleavage
tae-miR1122b-3p	Traes_6DL_84E14AD8D.4										1,05	-1.0	1	21	1030	1050	AGACUUAUAUGUAGGAACGGA	UCCGUUCCUAAAUAUAAGUCU	Translation
tae-miR1122a	Traes_6DL_84E14AD8D.4										2,05	-1.0	1	20	1071	1090	UAGAUACAUCCGUAUCUAGA	ACUACAUACGGAUGUAUAUA	Cleavage
tae-miR1118	Traes_6DL_C9216369C.2										2,05	-1.0	1	23	421	443	CACUACAUUAUGGAAUGGAGGGA	UUGCUCCGUUUCAUAAUGUAGUG	Cleavage
tae-miR444a	Traes_6DL_D1C1DBD34.1										0,00	-1.0	1	21	274	294	UUGCUGCCUCAAGCUUGCUGC	GCAGCAAGCUUGAGGCAGCAA	Cleavage
tae-miR444b	Traes_6DL_D1C1DBD34.1										0,00	-1.0	1	21	274	294	UUGCUGCCUCAAGCUUGCUGC	GCAGCAAGCUUGAGGCAGCAA	Cleavage
tae-miR1117	Traes_6DL_E940A7985.1										2,05	-1.0	1	24	155	178	UAGUACCGGUUCGUGGCACGAACC	GGUUCGUGGCACAAACCGGUACUA	Cleavage
tae-miR1131	Traes_6DL_E940A7985.1										1,00	-1.0	1	22	181	202	UAGUACCGGUUCGUGGCUAACC	GAUUCGCCACGAACCGGUACUA	Cleavage
tae-miR1122a	Traes_6DS_0D9BB546D.1										2,05	-1.0	1	20	111	130	UAGAUACAUCCGUAUCUAGA	ACUACAUACGGAUGUAUAUA	Cleavage
tae-miR9662a-3p	Traes_6DS_0E84AAF77.1	PF02536				GO:GO:0006355,GO:GO:0005739,GO:GO:0003690	AT5G07900.1	Mitochondrial transcription termination factor family protein	LOC_Os06g12100.1	mTERF family protein, expressed	2,05	-1.0	1	21	480	500	UUGAACAUCCCAGAGCCACCG	CCGUGGCUCUGGGAUGCUCAG	Cleavage
tae-miR9662b-3p	Traes_6DS_0E84AAF77.1	PF02536				GO:GO:0006355,GO:GO:0005739,GO:GO:0003690	AT5G07900.1	Mitochondrial transcription termination factor family protein	LOC_Os06g12100.1	mTERF family protein, expressed	2,05	-1.0	1	21	479	499	UGAACAUCCCAGAGCCACCGG	CCCGUGGCUCUGGGAUGCUCA	Cleavage
tae-miR9674b-5p	Traes_6DS_1BEF46735.1										3,00	-1.0	1	21	537	557	AUAGCAUCAUCCAUCCUACCC	GGGCCGAAUGGAUGAUGCUAU	Cleavage
tae-miR9674b-5p	Traes_6DS_1BEF46735.1										3,00	-1.0	1	21	537	557	AUAGCAUCAUCCAUCCUACCC	GGGCCGAAUGGAUGAUGCUAU	Cleavage
tae-miR1127b-3p	Traes_6DS_2207A3BC4.7										1,05	-1.0	1	21	1729	1749	ACAAGUAUUUCUGGACGGAGG	CCUCCGUCCGGAAAUACUUGU	Cleavage
tae-miR9662a-3p	Traes_6DS_2D4EFFF3E.1	PF02536				GO:GO:0006355,GO:GO:0005739,GO:GO:0003690	AT5G07900.1	Mitochondrial transcription termination factor family protein	LOC_Os06g12100.1	mTERF family protein, expressed	1,00	-1.0	1	21	369	389	UUGAACAUCCCAGAGCCACCG	CCGUGGCUCUGGGAUGUUCAG	Cleavage
tae-miR9662b-3p	Traes_6DS_2D4EFFF3E.1	PF02536				GO:GO:0006355,GO:GO:0005739,GO:GO:0003690	AT5G07900.1	Mitochondrial transcription termination factor family protein	LOC_Os06g12100.1	mTERF family protein, expressed	1,00	-1.0	1	21	368	388	UGAACAUCCCAGAGCCACCGG	CCCGUGGCUCUGGGAUGUUCA	Cleavage
tae-miR9662a-3p	Traes_6DS_3F194116E.1	PF02536				GO:GO:0006355,GO:GO:0005739,GO:GO:0003690	AT5G07900.1	Mitochondrial transcription termination factor family protein	LOC_Os06g12100.1	mTERF family protein, expressed	1,05	-1.0	1	21	525	545	UUGAACAUCCCAGAGCCACCG	CCGUGGCUCGGGGAUGUUCAA	Cleavage
tae-miR9662b-3p	Traes_6DS_3F194116E.1	PF02536				GO:GO:0006355,GO:GO:0005739,GO:GO:0003690	AT5G07900.1	Mitochondrial transcription termination factor family protein	LOC_Os06g12100.1	mTERF family protein, expressed	2,05	-1.0	1	21	524	544	UGAACAUCCCAGAGCCACCGG	CCCGUGGCUCGGGGAUGUUCA	Translation
tae-miR9662a-3p	Traes_6DS_3FC2ADA87.1	PF02536				GO:GO:0006355,GO:GO:0005739,GO:GO:0003690	AT5G07900.1	Mitochondrial transcription termination factor family protein	LOC_Os06g12100.1	mTERF family protein, expressed	3,00	-1.0	1	21	242	263	UUGAACAUCCCAGAGCCAC-CG	CGCGUGGCUCUGGGAUGUUCAG	Cleavage
tae-miR9662b-3p	Traes_6DS_3FC2ADA87.1	PF02536				GO:GO:0006355,GO:GO:0005739,GO:GO:0003690	AT5G07900.1	Mitochondrial transcription termination factor family protein	LOC_Os06g12100.1	mTERF family protein, expressed	2,00	-1.0	1	21	241	262	UGAACAUCCCAGAGCCAC-CGG	CCGCGUGGCUCUGGGAUGUUCA	Cleavage
tae-miR9662a-3p	Traes_6DS_3FC2ADA87.2	PF02536				GO:GO:0006355,GO:GO:0005739,GO:GO:0003690	AT1G21150.1	Mitochondrial transcription termination factor family protein	LOC_Os06g12100.1	mTERF family protein, expressed	3,00	-1.0	1	21	65	86	UUGAACAUCCCAGAGCCAC-CG	CGCGUGGCUCUGGGAUGUUCAG	Cleavage
tae-miR9662b-3p	Traes_6DS_3FC2ADA87.2	PF02536				GO:GO:0006355,GO:GO:0005739,GO:GO:0003690	AT1G21150.1	Mitochondrial transcription termination factor family protein	LOC_Os06g12100.1	mTERF family protein, expressed	2,00	-1.0	1	21	64	85	UGAACAUCCCAGAGCCAC-CGG	CCGCGUGGCUCUGGGAUGUUCA	Cleavage
tae-miR9662a-3p	Traes_6DS_422644406.1	PF02536				GO:GO:0006355,GO:GO:0005739,GO:GO:0003690	AT5G07900.1	Mitochondrial transcription termination factor family protein	LOC_Os06g12100.1	mTERF family protein, expressed	2,05	-1.0	1	21	468	488	UUGAACAUCCCAGAGCCACCG	CCGUGGCUCUGGAAUGUUCAG	Cleavage
tae-miR9662b-3p	Traes_6DS_422644406.1	PF02536				GO:GO:0006355,GO:GO:0005739,GO:GO:0003690	AT5G07900.1	Mitochondrial transcription termination factor family protein	LOC_Os06g12100.1	mTERF family protein, expressed	2,05	-1.0	1	21	467	487	UGAACAUCCCAGAGCCACCGG	CCCGUGGCUCUGGAAUGUUCA	Cleavage
tae-miR9662a-3p	Traes_6DS_422644406.2	PF02536				GO:GO:0006355,GO:GO:0005739,GO:GO:0003690	AT5G07900.1	Mitochondrial transcription termination factor family protein	LOC_Os06g12100.1	mTERF family protein, expressed	2,05	-1.0	1	21	66	86	UUGAACAUCCCAGAGCCACCG	CCGUGGCUCUGGAAUGUUCAG	Cleavage
tae-miR9662b-3p	Traes_6DS_422644406.2	PF02536				GO:GO:0006355,GO:GO:0005739,GO:GO:0003690	AT5G07900.1	Mitochondrial transcription termination factor family protein	LOC_Os06g12100.1	mTERF family protein, expressed	2,05	-1.0	1	21	65	85	UGAACAUCCCAGAGCCACCGG	CCCGUGGCUCUGGAAUGUUCA	Cleavage
tae-miR9662a-3p	Traes_6DS_422644406.3	PF02536				GO:GO:0006355,GO:GO:0005739,GO:GO:0003690	AT5G07900.1	Mitochondrial transcription termination factor family protein	LOC_Os06g12100.1	mTERF family protein, expressed	2,05	-1.0	1	21	66	86	UUGAACAUCCCAGAGCCACCG	CCGUGGCUCUGGAAUGUUCAG	Cleavage
tae-miR9662b-3p	Traes_6DS_422644406.3	PF02536				GO:GO:0006355,GO:GO:0005739,GO:GO:0003690	AT5G07900.1	Mitochondrial transcription termination factor family protein	LOC_Os06g12100.1	mTERF family protein, expressed	2,05	-1.0	1	21	65	85	UGAACAUCCCAGAGCCACCGG	CCCGUGGCUCUGGAAUGUUCA	Cleavage
tae-miR9662a-3p	Traes_6DS_422644406.4	PF02536				GO:GO:0006355,GO:GO:0005739,GO:GO:0003690	AT5G07900.1	Mitochondrial transcription termination factor family protein	LOC_Os06g12100.1	mTERF family protein, expressed	2,05	-1.0	1	21	66	86	UUGAACAUCCCAGAGCCACCG	CCGUGGCUCUGGAAUGUUCAG	Cleavage
tae-miR9662b-3p	Traes_6DS_422644406.4	PF02536				GO:GO:0006355,GO:GO:0005739,GO:GO:0003690	AT5G07900.1	Mitochondrial transcription termination factor family protein	LOC_Os06g12100.1	mTERF family protein, expressed	2,05	-1.0	1	21	65	85	UGAACAUCCCAGAGCCACCGG	CCCGUGGCUCUGGAAUGUUCA	Cleavage
tae-miR156	Traes_6DS_7B1596298.1	PF03110				GO:GO:0005634,GO:GO:0003677	AT5G43270.2	squamosa promoter binding protein-like 2	LOC_Os02g04680.2	OsSPL3 - SBP-box gene family member, expressed	1,00	-1.0	1	21	1150	1170	UGACAGAAGAGAGUGAGCACA	CAUGCUCUCUCUCUUCUGUCA	Cleavage
tae-miR156	Traes_6DS_7B1596298.2	PF03110				GO:GO:0005634,GO:GO:0003677	AT5G43270.1	squamosa promoter binding protein-like 2	LOC_Os02g04680.2	OsSPL3 - SBP-box gene family member, expressed	1,00	-1.0	1	21	1150	1170	UGACAGAAGAGAGUGAGCACA	CAUGCUCUCUCUCUUCUGUCA	Cleavage
tae-miR156	Traes_6DS_7B1596298.4								LOC_Os02g04680.1	OsSPL3 - SBP-box gene family member, expressed	1,00	-1.0	1	21	271	291	UGACAGAAGAGAGUGAGCACA	CAUGCUCUCUCUCUUCUGUCA	Cleavage
tae-miR9674b-5p	Traes_6DS_7C389C178.1										3,00	-1.0	1	21	537	557	AUAGCAUCAUCCAUCCUACCC	GGGCCGAAUGGAUGAUGCUAU	Cleavage
tae-miR9674a-5p	Traes_6DS_7C389C178.1										2,05	-1.0	1	21	954	974	GCAUCAUCCAUCCUACCAUUC	GAAUGGAAGGAUAGAUGAUGC	Cleavage
tae-miR9674b-5p	Traes_6DS_7C389C178.1										3,00	-1.0	1	21	537	557	AUAGCAUCAUCCAUCCUACCC	GGGCCGAAUGGAUGAUGCUAU	Cleavage
tae-miR9674a-5p	Traes_6DS_7C389C178.2										2,05	-1.0	1	21	51	71	GCAUCAUCCAUCCUACCAUUC	GAAUGGAAGGAUAGAUGAUGC	Cleavage
tae-miR9674b-5p	Traes_6DS_7C389C178.3										3,00	-1.0	1	21	537	557	AUAGCAUCAUCCAUCCUACCC	GGGCCGAAUGGAUGAUGCUAU	Cleavage
tae-miR9674a-5p	Traes_6DS_7C389C178.3										2,05	-1.0	1	21	954	974	GCAUCAUCCAUCCUACCAUUC	GAAUGGAAGGAUAGAUGAUGC	Cleavage
tae-miR9674b-5p	Traes_6DS_7C389C178.3										3,00	-1.0	1	21	537	557	AUAGCAUCAUCCAUCCUACCC	GGGCCGAAUGGAUGAUGCUAU	Cleavage
tae-miR9662a-3p	Traes_6DS_7D3BEF6A9.1	PF02536				GO:GO:0006355,GO:GO:0005739,GO:GO:0003690	AT1G21150.1	Mitochondrial transcription termination factor family protein	LOC_Os06g12100.1	mTERF family protein, expressed	2,05	-1.0	1	21	33	53	UUGAACAUCCCAGAGCCACCG	UCGUGGCUCGGGGAUGUUCAG	Cleavage
tae-miR9662b-3p	Traes_6DS_7D3BEF6A9.1	PF02536				GO:GO:0006355,GO:GO:0005739,GO:GO:0003690	AT1G21150.1	Mitochondrial transcription termination factor family protein	LOC_Os06g12100.1	mTERF family protein, expressed	2,05	-1.0	1	21	32	52	UGAACAUCCCAGAGCCACCGG	CUCGUGGCUCGGGGAUGUUCA	Translation
tae-miR9662a-3p	Traes_6DS_7D3BEF6A9.2	PF02536				GO:GO:0006355,GO:GO:0005739,GO:GO:0003690	AT1G21150.1	Mitochondrial transcription termination factor family protein	LOC_Os06g12100.1	mTERF family protein, expressed	2,05	-1.0	1	21	246	266	UUGAACAUCCCAGAGCCACCG	UCGUGGCUCGGGGAUGUUCAG	Cleavage
tae-miR9662b-3p	Traes_6DS_7D3BEF6A9.2	PF02536				GO:GO:0006355,GO:GO:0005739,GO:GO:0003690	AT1G21150.1	Mitochondrial transcription termination factor family protein	LOC_Os06g12100.1	mTERF family protein, expressed	2,05	-1.0	1	21	245	265	UGAACAUCCCAGAGCCACCGG	CUCGUGGCUCGGGGAUGUUCA	Translation
tae-miR9662a-3p	Traes_6DS_7D3BEF6A9.3	PF02536				GO:GO:0006355,GO:GO:0005739,GO:GO:0003690	AT1G21150.1	Mitochondrial transcription termination factor family protein	LOC_Os06g12100.1	mTERF family protein, expressed	2,05	-1.0	1	21	246	266	UUGAACAUCCCAGAGCCACCG	UCGUGGCUCGGGGAUGUUCAG	Cleavage
tae-miR9662b-3p	Traes_6DS_7D3BEF6A9.3	PF02536				GO:GO:0006355,GO:GO:0005739,GO:GO:0003690	AT1G21150.1	Mitochondrial transcription termination factor family protein	LOC_Os06g12100.1	mTERF family protein, expressed	2,05	-1.0	1	21	245	265	UGAACAUCCCAGAGCCACCGG	CUCGUGGCUCGGGGAUGUUCA	Translation
tae-miR9662a-3p	Traes_6DS_893F318A9.1	PF02536				GO:GO:0006355,GO:GO:0005739,GO:GO:0003690	AT5G07900.1	Mitochondrial transcription termination factor family protein	LOC_Os06g12100.1	mTERF family protein, expressed	1,05	-1.0	1	21	549	569	UUGAACAUCCCAGAGCCACCG	CCGUGGCUCGGGGAUGUUCAA	Cleavage
tae-miR9662b-3p	Traes_6DS_893F318A9.1	PF02536				GO:GO:0006355,GO:GO:0005739,GO:GO:0003690	AT5G07900.1	Mitochondrial transcription termination factor family protein	LOC_Os06g12100.1	mTERF family protein, expressed	2,05	-1.0	1	21	548	568	UGAACAUCCCAGAGCCACCGG	CCCGUGGCUCGGGGAUGUUCA	Translation
tae-miR9662a-3p	Traes_6DS_893F318A9.2	PF02536				GO:GO:0006355,GO:GO:0005739,GO:GO:0003690	AT5G07900.1	Mitochondrial transcription termination factor family protein	LOC_Os06g12100.1	mTERF family protein, expressed	1,05	-1.0	1	21	132	152	UUGAACAUCCCAGAGCCACCG	CCGUGGCUCGGGGAUGUUCAA	Cleavage
tae-miR9662b-3p	Traes_6DS_893F318A9.2	PF02536				GO:GO:0006355,GO:GO:0005739,GO:GO:0003690	AT5G07900.1	Mitochondrial transcription termination factor family protein	LOC_Os06g12100.1	mTERF family protein, expressed	2,05	-1.0	1	21	131	151	UGAACAUCCCAGAGCCACCGG	CCCGUGGCUCGGGGAUGUUCA	Translation
tae-miR9662a-3p	Traes_6DS_931C28AF6.1	PF02536				GO:GO:0006355,GO:GO:0005739,GO:GO:0003690	AT1G79220.1	Mitochondrial transcription termination factor family protein	LOC_Os06g12100.1	mTERF family protein, expressed	2,05	-1.0	1	21	288	308	UUGAACAUCCCAGAGCCACCG	AUGUGGCUCUCGGAUGUUCAG	Translation
tae-miR9662a-3p	Traes_6DS_931C28AF6.2	PF02536				GO:GO:0006355,GO:GO:0005739,GO:GO:0003690	AT1G79220.1	Mitochondrial transcription termination factor family protein	LOC_Os06g12100.1	mTERF family protein, expressed	2,05	-1.0	1	21	276	296	UUGAACAUCCCAGAGCCACCG	AUGUGGCUCUCGGAUGUUCAG	Translation
tae-miR9662a-3p	Traes_6DS_931C28AF6.3	PF02536				GO:GO:0006355,GO:GO:0005739,GO:GO:0003690	AT1G79220.1	Mitochondrial transcription termination factor family protein	LOC_Os06g12100.1	mTERF family protein, expressed	2,05	-1.0	1	21	273	293	UUGAACAUCCCAGAGCCACCG	AUGUGGCUCUCGGAUGUUCAG	Translation
tae-miR9662a-3p	Traes_6DS_931C28AF6.4	PF02536				GO:GO:0006355,GO:GO:0005739,GO:GO:0003690	AT1G79220.1	Mitochondrial transcription termination factor family protein	LOC_Os06g12100.1	mTERF family protein, expressed	2,05	-1.0	1	21	273	293	UUGAACAUCCCAGAGCCACCG	AUGUGGCUCUCGGAUGUUCAG	Translation
tae-miR9662a-3p	Traes_6DS_931C28AF6.5	PF02536				GO:GO:0006355,GO:GO:0005739,GO:GO:0003690	AT1G79220.1	Mitochondrial transcription termination factor family protein	LOC_Os06g12100.1	mTERF family protein, expressed	2,05	-1.0	1	21	276	296	UUGAACAUCCCAGAGCCACCG	AUGUGGCUCUCGGAUGUUCAG	Translation
tae-miR9662b-3p	Traes_6DS_93C7DA226.1										3,00	-1.0	1	21	503	523	UGAACAUCCCAGAGCCACCGG	CCCGUGCUUCUGGGAUGUUCA	Cleavage
tae-miR9662b-3p	Traes_6DS_93C7DA2261.1										3,00	-1.0	1	21	503	523	UGAACAUCCCAGAGCCACCGG	CCCGUGCUUCUGGGAUGUUCA	Cleavage
tae-miR9662a-3p	Traes_6DS_A490DF33E.1	PF02536				GO:GO:0006355,GO:GO:0005739,GO:GO:0003690	AT5G07900.1	Mitochondrial transcription termination factor family protein	LOC_Os06g12100.1	mTERF family protein, expressed	2,05	-1.0	1	21	471	491	UUGAACAUCCCAGAGCCACCG	CCGUGGCUCUGGAAUGUUCAG	Cleavage
tae-miR9662b-3p	Traes_6DS_A490DF33E.1	PF02536				GO:GO:0006355,GO:GO:0005739,GO:GO:0003690	AT5G07900.1	Mitochondrial transcription termination factor family protein	LOC_Os06g12100.1	mTERF family protein, expressed	2,05	-1.0	1	21	470	490	UGAACAUCCCAGAGCCACCGG	CCCGUGGCUCUGGAAUGUUCA	Cleavage
tae-miR9662a-3p	Traes_6DS_A490DF33E.2	PF02536				GO:GO:0006355,GO:GO:0005739,GO:GO:0003690	AT1G21150.1	Mitochondrial transcription termination factor family protein	LOC_Os06g12100.1	mTERF family protein, expressed	2,05	-1.0	1	21	66	86	UUGAACAUCCCAGAGCCACCG	CCGUGGCUCUGGAAUGUUCAG	Cleavage
tae-miR9662b-3p	Traes_6DS_A490DF33E.2	PF02536				GO:GO:0006355,GO:GO:0005739,GO:GO:0003690	AT1G21150.1	Mitochondrial transcription termination factor family protein	LOC_Os06g12100.1	mTERF family protein, expressed	2,05	-1.0	1	21	65	85	UGAACAUCCCAGAGCCACCGG	CCCGUGGCUCUGGAAUGUUCA	Cleavage
tae-miR1122b-3p	Traes_6DS_C1B7F14A41.2										1,05	-1.0	1	21	3044	3064	AGACUUAUAUGUAGGAACGGA	UCCGUUCCUAAAUAUAAGUCU	Translation
tae-miR156	Traes_6DS_C4D93239C.1	PF03110				GO:GO:0005634,GO:GO:0003677	AT5G43270.1	squamosa promoter binding protein-like 2	LOC_Os06g45310.1	OsSPL11 - SBP-box gene family member, expressed	1,00	-1.0	1	21	577	597	UGACAGAAGAGAGUGAGCACA	CAUGCUCUCUCUCUUCUGUCA	Cleavage
tae-miR156	Traes_6DS_C4D93239C.2								LOC_Os06g45310.1	OsSPL11 - SBP-box gene family member, expressed	1,00	-1.0	1	21	88	108	UGACAGAAGAGAGUGAGCACA	CAUGCUCUCUCUCUUCUGUCA	Cleavage
tae-miR156	Traes_6DS_C4D93239C.3								LOC_Os06g45310.1	OsSPL11 - SBP-box gene family member, expressed	1,00	-1.0	1	21	64	84	UGACAGAAGAGAGUGAGCACA	CAUGCUCUCUCUCUUCUGUCA	Cleavage
tae-miR9662a-3p	Traes_6DS_CF627A1BC.1								LOC_Os06g12100.1	mTERF family protein, expressed	2,05	-1.0	1	21	75	95	UUGAACAUCCCAGAGCCACCG	CCGUGGCUCGGGGAUGUUCAG	Cleavage
tae-miR9662b-3p	Traes_6DS_CF627A1BC.1								LOC_Os06g12100.1	mTERF family protein, expressed	2,05	-1.0	1	21	74	94	UGAACAUCCCAGAGCCACCGG	CCCGUGGCUCGGGGAUGUUCA	Translation
tae-miR9662a-3p	Traes_6DS_DAD8BEF41.1	PF02536				GO:GO:0006355,GO:GO:0005739,GO:GO:0003690	AT5G07900.1	Mitochondrial transcription termination factor family protein	LOC_Os06g12100.1	mTERF family protein, expressed	2,05	-1.0	1	21	477	497	UUGAACAUCCCAGAGCCACCG	CCGUGGCUCUGGAAUGUUCAG	Cleavage
tae-miR9662b-3p	Traes_6DS_DAD8BEF41.1	PF02536				GO:GO:0006355,GO:GO:0005739,GO:GO:0003690	AT5G07900.1	Mitochondrial transcription termination factor family protein	LOC_Os06g12100.1	mTERF family protein, expressed	2,05	-1.0	1	21	476	496	UGAACAUCCCAGAGCCACCGG	CCCGUGGCUCUGGAAUGUUCA	Cleavage
tae-miR9662a-3p	Traes_6DS_E352D66A4.1	PF02536				GO:GO:0006355,GO:GO:0005739,GO:GO:0003690	AT5G64950.1	Mitochondrial transcription termination factor family protein	LOC_Os06g12100.1	mTERF family protein, expressed	2,05	-1.0	1	21	450	470	UUGAACAUCCCAGAGCCACCG	CCGUGGCUCUGGGAUGUUUAG	Cleavage
tae-miR9662b-3p	Traes_6DS_E352D66A4.1	PF02536				GO:GO:0006355,GO:GO:0005739,GO:GO:0003690	AT5G64950.1	Mitochondrial transcription termination factor family protein	LOC_Os06g12100.1	mTERF family protein, expressed	2,05	-1.0	1	21	449	469	UGAACAUCCCAGAGCCACCGG	CCCGUGGCUCUGGGAUGUUUA	Cleavage
tae-miR9662a-3p	Traes_6DS_E95930B7D.1	PF02536				GO:GO:0006355,GO:GO:0005739,GO:GO:0003690	AT5G64950.1	Mitochondrial transcription termination factor family protein	LOC_Os06g12100.1	mTERF family protein, expressed	2,05	-1.0	1	21	450	470	UUGAACAUCCCAGAGCCACCG	CCGUGGCUCUGGGAUGUUUAG	Cleavage
tae-miR9662b-3p	Traes_6DS_E95930B7D.1	PF02536				GO:GO:0006355,GO:GO:0005739,GO:GO:0003690	AT5G64950.1	Mitochondrial transcription termination factor family protein	LOC_Os06g12100.1	mTERF family protein, expressed	2,05	-1.0	1	21	449	469	UGAACAUCCCAGAGCCACCGG	CCCGUGGCUCUGGGAUGUUUA	Cleavage
tae-miR9662a-3p	Traes_6DS_EF75D5C67.1	PF02536				GO:GO:0006355,GO:GO:0005739,GO:GO:0003690	AT5G07900.1	Mitochondrial transcription termination factor family protein	LOC_Os06g12100.1	mTERF family protein, expressed	2,00	-1.0	1	21	66	86	UUGAACAUCCCAGAGCCACCG	UCGUGGCGCUGGGAUGUUCAG	Cleavage
tae-miR9662b-3p	Traes_6DS_EF75D5C67.1	PF02536				GO:GO:0006355,GO:GO:0005739,GO:GO:0003690	AT5G07900.1	Mitochondrial transcription termination factor family protein	LOC_Os06g12100.1	mTERF family protein, expressed	2,05	-1.0	1	21	65	85	UGAACAUCCCAGAGCCACCGG	CUCGUGGCGCUGGGAUGUUCA	Cleavage
tae-miR9662a-3p	Traes_6DS_F6C7E588F.1	PF02536	KOG1267			GO:GO:0006355,GO:GO:0005739,GO:GO:0003690	AT5G07900.1	Mitochondrial transcription termination factor family protein	LOC_Os06g12100.1	mTERF family protein, expressed	2,00	-1.0	1	21	657	677	UUGAACAUCCCAGAGCCACCG	CCGUGGUUCUGGGAUGUUCAG	Cleavage
tae-miR9662b-3p	Traes_6DS_F6C7E588F.1	PF02536	KOG1267			GO:GO:0006355,GO:GO:0005739,GO:GO:0003690	AT5G07900.1	Mitochondrial transcription termination factor family protein	LOC_Os06g12100.1	mTERF family protein, expressed	2,00	-1.0	1	21	656	676	UGAACAUCCCAGAGCCACCGG	CCCGUGGUUCUGGGAUGUUCA	Cleavage
tae-miR9662a-3p	Traes_6DS_FB434BB3C.1	PF02536				GO:GO:0006355,GO:GO:0005739,GO:GO:0003690	AT5G64950.1	Mitochondrial transcription termination factor family protein	LOC_Os06g12100.1	mTERF family protein, expressed	2,05	-1.0	1	21	246	266	UUGAACAUCCCAGAGCCACCG	CCGUGGCUCUCGGAUGUUCAG	Translation
tae-miR9662b-3p	Traes_6DS_FB434BB3C.1	PF02536				GO:GO:0006355,GO:GO:0005739,GO:GO:0003690	AT5G64950.1	Mitochondrial transcription termination factor family protein	LOC_Os06g12100.1	mTERF family protein, expressed	2,05	-1.0	1	21	245	265	UGAACAUCCCAGAGCCACCGG	CCCGUGGCUCUCGGAUGUUCA	Translation
tae-miR9662a-3p	Traes_6DS_FB434BB3C.2	PF02536				GO:GO:0006355,GO:GO:0005739,GO:GO:0003690	AT5G64950.1	Mitochondrial transcription termination factor family protein	LOC_Os06g12100.1	mTERF family protein, expressed	2,05	-1.0	1	21	246	266	UUGAACAUCCCAGAGCCACCG	CCGUGGCUCUCGGAUGUUCAG	Translation
tae-miR9662b-3p	Traes_6DS_FB434BB3C.2	PF02536				GO:GO:0006355,GO:GO:0005739,GO:GO:0003690	AT5G64950.1	Mitochondrial transcription termination factor family protein	LOC_Os06g12100.1	mTERF family protein, expressed	2,05	-1.0	1	21	245	265	UGAACAUCCCAGAGCCACCGG	CCCGUGGCUCUCGGAUGUUCA	Translation
tae-miR1122a	Traes_7AL_135129885.3										2,05	-1.0	1	20	721	740	UAGAUACAUCCGUAUCUAGA	ACUACAUACGGAUGUAUAUA	Cleavage
tae-miR164	Traes_7AL_628A69311.1										1,00	-1.0	1	21	650	670	UGGAGAAGCAGGGCACGUGCA	AGCAAGUGCCCUGCUUCUCCA	Cleavage
tae-miR1130b-3p	Traes_7AL_955F54182.1										0,00	-1.0	1	21	1141	1161	UCUUAUAUUAUGGGACGGAGG	CCUCCGUCCCAUAAUAUAAGA	Cleavage
tae-miR1122c-3p	Traes_7AL_955F54182.1										1,05	-1.0	1	21	1141	1161	UCUAAUAUUAUGGGACGGAGG	CCUCCGUCCCAUAAUAUAAGA	Cleavage
tae-miR1133	Traes_7AL_ADBB1224B.1										2,05	-1.0	1	22	52	73	CAUAUACUCCCUCCGUCCGAAA	UUCCGGACGGAGGGAGUAUAAU	Cleavage
tae-miR164	Traes_7AL_BE3253C8F.1										1,00	-1.0	1	21	653	673	UGGAGAAGCAGGGCACGUGCA	AGCAAGUGCCCUGCUUCUCCA	Cleavage
tae-miR164	Traes_7AL_BE3253C8F.2										1,00	-1.0	1	21	215	235	UGGAGAAGCAGGGCACGUGCA	AGCAAGUGCCCUGCUUCUCCA	Cleavage
tae-miR160	Traes_7AL_E3ADC8C38.1										0,00	-1.0	1	21	399	419	UGCCUGGCUCCCUGUAUGCCA	AGGCAUACAGGGAGCCAGGCA	Cleavage
tae-miR9666a-3p	Traes_7AL_EE3CD1CF3.10										2,00	-1.0	1	22	229	250	CGGUAGGGCUGUAUGAUGGCGA	UCCCCGUCAUACAGCCCUACCU	Cleavage
tae-miR9666a-3p	Traes_7AL_EE3CD1CF3.2										2,00	-1.0	1	22	5032	5053	CGGUAGGGCUGUAUGAUGGCGA	UCCCCGUCAUACAGCCCUACCU	Cleavage
tae-miR9666a-3p	Traes_7AL_EE3CD1CF3.3										2,00	-1.0	1	22	4948	4969	CGGUAGGGCUGUAUGAUGGCGA	UCCCCGUCAUACAGCCCUACCU	Cleavage
tae-miR9666a-3p	Traes_7AL_EE3CD1CF3.4										2,00	-1.0	1	22	4801	4822	CGGUAGGGCUGUAUGAUGGCGA	UCCCCGUCAUACAGCCCUACCU	Cleavage
tae-miR9666a-3p	Traes_7AL_EE3CD1CF3.5										2,00	-1.0	1	22	4393	4414	CGGUAGGGCUGUAUGAUGGCGA	UCCCCGUCAUACAGCCCUACCU	Cleavage
tae-miR9666a-3p	Traes_7AL_EE3CD1CF3.6										2,00	-1.0	1	22	3409	3430	CGGUAGGGCUGUAUGAUGGCGA	UCCCCGUCAUACAGCCCUACCU	Cleavage
tae-miR9666a-3p	Traes_7AL_EE3CD1CF3.8										2,00	-1.0	1	22	2941	2962	CGGUAGGGCUGUAUGAUGGCGA	UCCCCGUCAUACAGCCCUACCU	Cleavage
tae-miR1137b-5p	Traes_7AS_0F0291904.1										2,05	-1.0	1	21	844	864	UCCGUUCCAGAAUAGAUGACC	ACUCAUCUAUUCUGGGACGGU	Cleavage
tae-miR1137b-5p	Traes_7AS_0F0291904.2										2,05	-1.0	1	21	451	471	UCCGUUCCAGAAUAGAUGACC	ACUCAUCUAUUCUGGGACGGU	Cleavage
tae-miR1137b-5p	Traes_7AS_23DFA827E.1										1,05	-1.0	1	21	11	31	UCCGUUCCAGAAUAGAUGACC	AGUCAUCUAUUUUGGAACGGA	Translation
tae-miR1122a	Traes_7AS_9674F6981.1										1,05	-1.0	1	20	1467	1486	UAGAUACAUCCGUAUCUAGA	UCUAGAUACAGAUGUAUCUA	Translation
tae-miR1122a	Traes_7AS_9674F6981.2										1,05	-1.0	1	20	1473	1492	UAGAUACAUCCGUAUCUAGA	UCUAGAUACAGAUGUAUCUA	Translation
tae-miR1122a	Traes_7AS_9674F6981.3										1,05	-1.0	1	20	1467	1486	UAGAUACAUCCGUAUCUAGA	UCUAGAUACAGAUGUAUCUA	Translation
tae-miR1130b-3p	Traes_7AS_9819F7C48.1										0,00	-1.0	1	21	1268	1288	UCUUAUAUUAUGGGACGGAGG	AAUCCGUCCCAUAAUAUAAGA	Cleavage
tae-miR1122c-3p	Traes_7AS_9819F7C48.1										1,05	-1.0	1	21	1268	1288	UCUAAUAUUAUGGGACGGAGG	AAUCCGUCCCAUAAUAUAAGA	Cleavage
tae-miR1120c-5p	Traes_7AS_9819F7C48.1										1,05	-1.0	1	21	1311	1331	UAAUAUAAGAACGUUUUUGAC	GUCAAAAACGCUCUUAUAUUA	Translation
tae-miR7757-5p	Traes_7AS_E43D64A80.1	PF00931	KOG4658			GO:GO:0043531	AT3G46530.1	NB-ARC domain-containing disease resistance protein	LOC_Os07g26430.1	resistance protein, putative, expressed	2,05	-1.0	1	22	377	398	AUAAAACCUUCAGCUAUCCAUC	AAUGGAUAACUGAAGAUUUUAU	Cleavage
tae-miR7757-5p	Traes_7AS_E43D64A80.2	PF00931	KOG4658			GO:GO:0043531	AT3G46530.1	NB-ARC domain-containing disease resistance protein	LOC_Os07g26430.1	resistance protein, putative, expressed	2,05	-1.0	1	22	1424	1445	AUAAAACCUUCAGCUAUCCAUC	AAUGGAUAACUGAAGAUUUUAU	Cleavage
tae-miR156	Traes_7AS_FE67F9E86.1								LOC_Os08g41940.1	OsSPL16 - SBP-box gene family member, expressed	1,00	-1.0	1	21	358	378	UGACAGAAGAGAGUGAGCACA	UGUGCUCUCUCUCUUCUGUCA	Cleavage
tae-miR156	Traes_7AS_FE67F9E86.2								LOC_Os08g41940.1	OsSPL16 - SBP-box gene family member, expressed	1,00	-1.0	1	21	358	378	UGACAGAAGAGAGUGAGCACA	UGUGCUCUCUCUCUUCUGUCA	Cleavage
tae-miR156	Traes_7AS_FE67F9E86.3								LOC_Os08g41940.1	OsSPL16 - SBP-box gene family member, expressed	1,00	-1.0	1	21	331	351	UGACAGAAGAGAGUGAGCACA	UGUGCUCUCUCUCUUCUGUCA	Cleavage
tae-miR9654a-3p	Traes_7BL_03C3797A2.1										2,00	-1.0	1	22	1813	1834	UUCUGAAAGGCUUGAAGCGAAU	GAGGGCCUCAAGCCUUUCAGAA	Cleavage
tae-miR9654a-3p	Traes_7BL_03C3797A2.2										2,00	-1.0	1	22	1813	1834	UUCUGAAAGGCUUGAAGCGAAU	GAGGGCCUCAAGCCUUUCAGAA	Cleavage
tae-miR9654a-3p	Traes_7BL_03C3797A2.3										2,00	-1.0	1	22	1513	1534	UUCUGAAAGGCUUGAAGCGAAU	GAGGGCCUCAAGCCUUUCAGAA	Cleavage
tae-miR9654a-3p	Traes_7BL_03C3797A2.4										2,00	-1.0	1	22	1513	1534	UUCUGAAAGGCUUGAAGCGAAU	GAGGGCCUCAAGCCUUUCAGAA	Cleavage
tae-miR9654a-3p	Traes_7BL_03C3797A2.5										2,00	-1.0	1	22	1399	1420	UUCUGAAAGGCUUGAAGCGAAU	GAGGGCCUCAAGCCUUUCAGAA	Cleavage
tae-miR9654a-3p	Traes_7BL_03C3797A2.6										2,00	-1.0	1	22	973	994	UUCUGAAAGGCUUGAAGCGAAU	GAGGGCCUCAAGCCUUUCAGAA	Cleavage
tae-miR9654a-3p	Traes_7BL_03C3797A2.7										2,00	-1.0	1	22	1813	1834	UUCUGAAAGGCUUGAAGCGAAU	GAGGGCCUCAAGCCUUUCAGAA	Cleavage
tae-miR1130b-3p	Traes_7BL_0665E8707.6										1,00	-1.0	1	21	46	66	UCUUAUAUUAUGGGACGGAGG	CCUCCGUCCCAUAAUAUAAGU	Cleavage
tae-miR1122c-3p	Traes_7BL_0665E8707.6										2,05	-1.0	1	21	46	66	UCUAAUAUUAUGGGACGGAGG	CCUCCGUCCCAUAAUAUAAGU	Cleavage
tae-miR1133	Traes_7BL_07C4E8081.43										0,00	-1.0	1	22	46	67	CAUAUACUCCCUCCGUCCGAAA	UUUCGGACGGAGGGAGUAUAUG	Cleavage
tae-miR1133	Traes_7BL_07C4E8081.44										0,00	-1.0	1	22	46	67	CAUAUACUCCCUCCGUCCGAAA	UUUCGGACGGAGGGAGUAUAUG	Cleavage
tae-miR1133	Traes_7BL_07C4E8081.45										0,00	-1.0	1	22	46	67	CAUAUACUCCCUCCGUCCGAAA	UUUCGGACGGAGGGAGUAUAUG	Cleavage
tae-miR160	Traes_7BL_18D335F08.1										0,00	-1.0	1	21	171	191	UGCCUGGCUCCCUGUAUGCCA	AGGCAUACAGGGAGCCAGGCA	Cleavage
tae-miR160	Traes_7BL_18D335F08.2										0,00	-1.0	1	21	171	191	UGCCUGGCUCCCUGUAUGCCA	AGGCAUACAGGGAGCCAGGCA	Cleavage
tae-miR1137a	Traes_7BL_C184D0525.2										0,00	-1.0	1	20	913	932	UAGUACAAAGUUGAGUCAUC	GAUGACUCAACUUUGUACUA	Cleavage
tae-miR395b	Traes_7BL_DC0D8773C.1										2,05	-1.0	1	20	1385	1404	UGAAGUGUUUGGGGGAACUC	CAGUUCACCCAAAAACUUCA	Cleavage
tae-miR395b	Traes_7BL_DC0D8773C.10										2,05	-1.0	1	20	1148	1167	UGAAGUGUUUGGGGGAACUC	CAGUUCACCCAAAAACUUCA	Cleavage
tae-miR395b	Traes_7BL_DC0D8773C.11										2,05	-1.0	1	20	848	867	UGAAGUGUUUGGGGGAACUC	CAGUUCACCCAAAAACUUCA	Cleavage
tae-miR395b	Traes_7BL_DC0D8773C.12										2,05	-1.0	1	20	848	867	UGAAGUGUUUGGGGGAACUC	CAGUUCACCCAAAAACUUCA	Cleavage
tae-miR395b	Traes_7BL_DC0D8773C.13										2,05	-1.0	1	20	848	867	UGAAGUGUUUGGGGGAACUC	CAGUUCACCCAAAAACUUCA	Cleavage
tae-miR395b	Traes_7BL_DC0D8773C.14										2,05	-1.0	1	20	848	867	UGAAGUGUUUGGGGGAACUC	CAGUUCACCCAAAAACUUCA	Cleavage
tae-miR395b	Traes_7BL_DC0D8773C.2										2,05	-1.0	1	20	1385	1404	UGAAGUGUUUGGGGGAACUC	CAGUUCACCCAAAAACUUCA	Cleavage
tae-miR395b	Traes_7BL_DC0D8773C.3										2,05	-1.0	1	20	1385	1404	UGAAGUGUUUGGGGGAACUC	CAGUUCACCCAAAAACUUCA	Cleavage
tae-miR395b	Traes_7BL_DC0D8773C.4										2,05	-1.0	1	20	1385	1404	UGAAGUGUUUGGGGGAACUC	CAGUUCACCCAAAAACUUCA	Cleavage
tae-miR395b	Traes_7BL_DC0D8773C.5										2,05	-1.0	1	20	1385	1404	UGAAGUGUUUGGGGGAACUC	CAGUUCACCCAAAAACUUCA	Cleavage
tae-miR395b	Traes_7BL_DC0D8773C.6										2,05	-1.0	1	20	1148	1167	UGAAGUGUUUGGGGGAACUC	CAGUUCACCCAAAAACUUCA	Cleavage
tae-miR395b	Traes_7BL_DC0D8773C.7										2,05	-1.0	1	20	1148	1167	UGAAGUGUUUGGGGGAACUC	CAGUUCACCCAAAAACUUCA	Cleavage
tae-miR395b	Traes_7BL_DC0D8773C.8										2,05	-1.0	1	20	1148	1167	UGAAGUGUUUGGGGGAACUC	CAGUUCACCCAAAAACUUCA	Cleavage
tae-miR395b	Traes_7BL_DC0D8773C.9										2,05	-1.0	1	20	1148	1167	UGAAGUGUUUGGGGGAACUC	CAGUUCACCCAAAAACUUCA	Cleavage
tae-miR164	Traes_7BL_F1A0270C6.1										1,00	-1.0	1	21	32	52	UGGAGAAGCAGGGCACGUGCA	AGCAAGUGCCCUGCUUCUCCA	Cleavage
tae-miR9666a-3p	Traes_7BL_F8D5E60B1.1										2,00	-1.0	1	22	5050	5071	CGGUAGGGCUGUAUGAUGGCGA	UCCCCCUCAUACAGCCCUACCU	Cleavage

tae-miR9666a-3p	Traes_7BL_F8D5E60B1.1										2,05	-1.0	1	22	4966	4987	CGGUAGGGCUGUAUGAUGGCGA	UCGCCAUCAUACAGCCCUACUU	Cleavage
tae-miR9666a-3p	Traes_7BL_F8D5E60B1.2										2,00	-1.0	1	22	5011	5032	CGGUAGGGCUGUAUGAUGGCGA	UCCCCCUCAUACAGCCCUACCU	Cleavage
tae-miR9666a-3p	Traes_7BL_F8D5E60B1.2										2,05	-1.0	1	22	4927	4948	CGGUAGGGCUGUAUGAUGGCGA	UCGCCAUCAUACAGCCCUACUU	Cleavage
tae-miR9666a-3p	Traes_7BL_F8D5E60B1.3										2,00	-1.0	1	22	4780	4801	CGGUAGGGCUGUAUGAUGGCGA	UCCCCCUCAUACAGCCCUACCU	Cleavage
tae-miR9666a-3p	Traes_7BL_F8D5E60B1.3										2,05	-1.0	1	22	4696	4717	CGGUAGGGCUGUAUGAUGGCGA	UCGCCAUCAUACAGCCCUACUU	Cleavage
tae-miR9666a-3p	Traes_7BL_F8D5E60B1.4										2,00	-1.0	1	22	3409	3430	CGGUAGGGCUGUAUGAUGGCGA	UCCCCCUCAUACAGCCCUACCU	Cleavage
tae-miR9666a-3p	Traes_7BL_F8D5E60B1.4										2,05	-1.0	1	22	3325	3346	CGGUAGGGCUGUAUGAUGGCGA	UCGCCAUCAUACAGCCCUACUU	Cleavage
tae-miR1137a	Traes_7BS_2A6C29F49.3										2,00	-1.0	1	20	75	94	UAGUACAAAGUUGAGUCAUC	AGUGUCUCAACUUUGUACUA	Cleavage
tae-miR1137a	Traes_7BS_2A6C29F49.4										2,00	-1.0	1	20	81	100	UAGUACAAAGUUGAGUCAUC	AGUGUCUCAACUUUGUACUA	Cleavage
tae-miR1130b-3p	Traes_7BS_47B8FC9C8.2										0,00	-1.0	1	21	1268	1288	UCUUAUAUUAUGGGACGGAGG	CCUCCGUCCCAUAAUAUAAGA	Cleavage
tae-miR1122c-3p	Traes_7BS_47B8FC9C8.2										1,05	-1.0	1	21	1268	1288	UCUAAUAUUAUGGGACGGAGG	CCUCCGUCCCAUAAUAUAAGA	Cleavage
tae-miR1120c-5p	Traes_7BS_47B8FC9C8.2										1,05	-1.0	1	21	1311	1331	UAAUAUAAGAACGUUUUUGAC	GUCAAAAACGCUCUUAUAUUA	Translation
tae-miR1130b-3p	Traes_7BS_47B8FC9C8.3										0,00	-1.0	1	21	1265	1285	UCUUAUAUUAUGGGACGGAGG	CCUCCGUCCCAUAAUAUAAGA	Cleavage
tae-miR1122c-3p	Traes_7BS_47B8FC9C8.3										1,05	-1.0	1	21	1265	1285	UCUAAUAUUAUGGGACGGAGG	CCUCCGUCCCAUAAUAUAAGA	Cleavage
tae-miR1120c-5p	Traes_7BS_47B8FC9C8.3										1,05	-1.0	1	21	1308	1328	UAAUAUAAGAACGUUUUUGAC	GUCAAAAACGCUCUUAUAUUA	Translation
tae-miR9662a-3p	Traes_7BS_598310C32.1	PF02536				GO:GO:0006355,GO:GO:0005739,GO:GO:0003690	AT1G21150.1	Mitochondrial transcription termination factor family protein	LOC_Os06g12100.1	mTERF family protein, expressed	2,05	-1.0	1	21	33	53	UUGAACAUCCCAGAGCCACCG	CCGGUGGCUCUGCGAUGUUCAG	Translation
tae-miR9662b-3p	Traes_7BS_598310C32.1	PF02536				GO:GO:0006355,GO:GO:0005739,GO:GO:0003690	AT1G21150.1	Mitochondrial transcription termination factor family protein	LOC_Os06g12100.1	mTERF family protein, expressed	2,05	-1.0	1	21	32	52	UGAACAUCCCAGAGCCACCGG	CCCGUGGCUCUGCGAUGUUCA	Cleavage
tae-miR1121	Traes_7BS_5A0E77FB8.2										1,00	-1.0	1	22	3478	3499	AGUAGUGAUCUAAACGCUCUUA	UAAGAGUGUUUAGAUCACUACU	Cleavage
tae-miR1137a	Traes_7BS_6716D1D07.2										0,00	-1.0	1	20	347	366	UAGUACAAAGUUGAGUCAUC	GAUGACUCAACUUUGUACUA	Cleavage
tae-miR9781	Traes_7BS_A42F893FC.1										1,05	-1.0	1	21	117	137	UUUUGUCACAUAUAAUACAUA	UAUGUAUUUUAUGUGACAAAA	Cleavage
tae-miR9664-3p	Traes_7DL_101A9F756.1	PF00931	KOG4658			GO:GO:0043531	AT1G58807.1	Disease resistance protein (CC-NBS-LRR class) family	LOC_Os11g11550.1	NBS-LRR disease resistance protein, putative, expressed	2,05	-1.0	1	21	102	122	UUGCAGUCCUCGAUGUCGUAG	GCACGACAUCGAGGAUUGCAU	Cleavage
tae-miR9664-3p	Traes_7DL_101A9F756.2	PF00931	KOG4658			GO:GO:0043531	AT1G58807.1	Disease resistance protein (CC-NBS-LRR class) family	LOC_Os11g11550.2	NBS-LRR disease resistance protein, putative, expressed	2,05	-1.0	1	21	102	122	UUGCAGUCCUCGAUGUCGUAG	GCACGACAUCGAGGAUUGCAU	Cleavage
tae-miR9664-3p	Traes_7DL_101A9F756.3	PF00931	KOG4658			GO:GO:0043531	AT1G58807.1	Disease resistance protein (CC-NBS-LRR class) family	LOC_Os11g11550.1	NBS-LRR disease resistance protein, putative, expressed	2,05	-1.0	1	21	231	251	UUGCAGUCCUCGAUGUCGUAG	GCACGACAUCGAGGAUUGCAU	Cleavage
tae-miR9666a-3p	Traes_7DL_18F2A8567.1										2,05	-1.0	1	22	4927	4948	CGGUAGGGCUGUAUGAUGGCGA	UCGCCAUCAUACAGCCCUACUU	Cleavage
tae-miR9666a-3p	Traes_7DL_18F2A8567.11										2,00	-1.0	1	22	244	265	CGGUAGGGCUGUAUGAUGGCGA	UCGCCCUCAUACAGCCCUACCU	Cleavage
tae-miR9666a-3p	Traes_7DL_18F2A8567.11										2,05	-1.0	1	22	139	160	CGGUAGGGCUGUAUGAUGGCGA	UCGCCAUCAUACAGCCCUACUU	Cleavage
tae-miR9666a-3p	Traes_7DL_18F2A8567.12										2,00	-1.0	1	22	250	271	CGGUAGGGCUGUAUGAUGGCGA	UCGCCCUCAUACAGCCCUACCU	Cleavage
tae-miR9666a-3p	Traes_7DL_18F2A8567.12										2,05	-1.0	1	22	145	166	CGGUAGGGCUGUAUGAUGGCGA	UCGCCAUCAUACAGCCCUACUU	Cleavage
tae-miR9666a-3p	Traes_7DL_18F2A8567.14										2,00	-1.0	1	22	201	222	CGGUAGGGCUGUAUGAUGGCGA	UCGCCCUCAUACAGCCCUACCU	Cleavage
tae-miR9666a-3p	Traes_7DL_18F2A8567.14										2,05	-1.0	1	22	96	117	CGGUAGGGCUGUAUGAUGGCGA	UCGCCAUCAUACAGCCCUACUU	Cleavage
tae-miR9666a-3p	Traes_7DL_18F2A8567.2										2,00	-1.0	1	22	5032	5053	CGGUAGGGCUGUAUGAUGGCGA	UCGCCCUCAUACAGCCCUACCU	Cleavage
tae-miR9666a-3p	Traes_7DL_18F2A8567.2										2,05	-1.0	1	22	4927	4948	CGGUAGGGCUGUAUGAUGGCGA	UCGCCAUCAUACAGCCCUACUU	Cleavage
tae-miR9666a-3p	Traes_7DL_18F2A8567.3										2,00	-1.0	1	22	4969	4990	CGGUAGGGCUGUAUGAUGGCGA	UCGCCCUCAUACAGCCCUACCU	Cleavage
tae-miR9666a-3p	Traes_7DL_18F2A8567.3										2,05	-1.0	1	22	4927	4948	CGGUAGGGCUGUAUGAUGGCGA	UCGCCAUCAUACAGCCCUACUU	Cleavage
tae-miR9666a-3p	Traes_7DL_18F2A8567.4										2,05	-1.0	1	22	4927	4948	CGGUAGGGCUGUAUGAUGGCGA	UCGCCAUCAUACAGCCCUACUU	Cleavage
tae-miR9666a-3p	Traes_7DL_18F2A8567.5										2,05	-1.0	1	22	4927	4948	CGGUAGGGCUGUAUGAUGGCGA	UCGCCAUCAUACAGCCCUACUU	Cleavage
tae-miR9666a-3p	Traes_7DL_18F2A8567.6										2,00	-1.0	1	22	5032	5053	CGGUAGGGCUGUAUGAUGGCGA	UCGCCCUCAUACAGCCCUACCU	Cleavage
tae-miR9666a-3p	Traes_7DL_18F2A8567.6										2,05	-1.0	1	22	4927	4948	CGGUAGGGCUGUAUGAUGGCGA	UCGCCAUCAUACAGCCCUACUU	Cleavage
tae-miR9666a-3p	Traes_7DL_18F2A8567.7										2,00	-1.0	1	22	4801	4822	CGGUAGGGCUGUAUGAUGGCGA	UCGCCCUCAUACAGCCCUACCU	Cleavage
tae-miR9666a-3p	Traes_7DL_18F2A8567.7										2,05	-1.0	1	22	4696	4717	CGGUAGGGCUGUAUGAUGGCGA	UCGCCAUCAUACAGCCCUACUU	Cleavage
tae-miR9666a-3p	Traes_7DL_18F2A8567.8										2,00	-1.0	1	22	3409	3430	CGGUAGGGCUGUAUGAUGGCGA	UCGCCCUCAUACAGCCCUACCU	Cleavage
tae-miR9666a-3p	Traes_7DL_18F2A8567.8										2,05	-1.0	1	22	3304	3325	CGGUAGGGCUGUAUGAUGGCGA	UCGCCAUCAUACAGCCCUACUU	Cleavage
tae-miR9666a-3p	Traes_7DL_18F2A8567.9										2,00	-1.0	1	22	2941	2962	CGGUAGGGCUGUAUGAUGGCGA	UCGCCCUCAUACAGCCCUACCU	Cleavage
tae-miR9666a-3p	Traes_7DL_18F2A8567.9										2,05	-1.0	1	22	2836	2857	CGGUAGGGCUGUAUGAUGGCGA	UCGCCAUCAUACAGCCCUACUU	Cleavage
tae-miR164	Traes_7DL_1E343B6E6.1										1,00	-1.0	1	21	236	256	UGGAGAAGCAGGGCACGUGCA	AGCAAGUGCCCUGCUUCUCCA	Cleavage
tae-miR164	Traes_7DL_1E343B6E6.2										1,00	-1.0	1	21	32	52	UGGAGAAGCAGGGCACGUGCA	AGCAAGUGCCCUGCUUCUCCA	Cleavage
tae-miR1135	Traes_7DL_20BFD8A19.4	PF00145		2.1.1.37			AT4G19020.1	chromomethylase 2	LOC_Os10g01570.1	C-5 cytosine-specific DNA methylase, putative, expressed	1,05	-1.0	1	24	954	977	CUGCGACAAGUAAUUCCGAACGGA	UCUGUUCGGAAUUACUUGUCGUAG	Cleavage
tae-miR1127a	Traes_7DL_20BFD8A19.4	PF00145		2.1.1.37			AT4G19020.1	chromomethylase 2	LOC_Os10g01570.1	C-5 cytosine-specific DNA methylase, putative, expressed	1,05	-1.0	1	19	1032	1050	UCCUUCCGUUCGGAAUUAC	GUAAUUCCGAACGGAGGGA	Cleavage
tae-miR9778	Traes_7DL_31A23053A.1								LOC_Os11g39600.1	resistance protein, putative, expressed	1,00	-1.0	1	21	132	152	UGCAUCAUCUCGAACUCGUCG	CCUCGAGUUCGAGAUGAUGCA	Cleavage
tae-miR1122a	Traes_7DL_37135CE6E.2										2,05	-1.0	1	20	2105	2124	UAGAUACAUCCGUAUCUAGA	UCUAAAUACGGAUGUAUCAA	Cleavage
tae-miR1122a	Traes_7DL_37135CE6E.3										2,05	-1.0	1	20	2090	2109	UAGAUACAUCCGUAUCUAGA	UCUAAAUACGGAUGUAUCAA	Cleavage
tae-miR1122a	Traes_7DL_37135CE6E.6										2,05	-1.0	1	20	1241	1260	UAGAUACAUCCGUAUCUAGA	UCUAAAUACGGAUGUAUCAA	Cleavage
tae-miR9661-5p	Traes_7DL_55AC0BE09.1										2,05	-1.0	1	21	143	163	UGAAGUAGAGCAGGGACCUCA	UCAGGUCCCUGCGCUACUUCG	Cleavage
tae-miR9661-5p	Traes_7DL_55AC0BE09.2										2,05	-1.0	1	21	143	163	UGAAGUAGAGCAGGGACCUCA	UCAGGUCCCUGCGCUACUUCG	Cleavage
tae-miR9661-5p	Traes_7DL_55AC0BE09.3										2,05	-1.0	1	21	116	136	UGAAGUAGAGCAGGGACCUCA	UCAGGUCCCUGCGCUACUUCG	Cleavage
tae-miR9661-5p	Traes_7DL_55AC0BE09.4										2,05	-1.0	1	21	116	136	UGAAGUAGAGCAGGGACCUCA	UCAGGUCCCUGCGCUACUUCG	Cleavage
tae-miR9661-5p	Traes_7DL_55AC0BE09.5										2,05	-1.0	1	21	116	136	UGAAGUAGAGCAGGGACCUCA	UCAGGUCCCUGCGCUACUUCG	Cleavage
tae-miR160	Traes_7DL_55ADB3528.1										0,00	-1.0	1	21	714	734	UGCCUGGCUCCCUGUAUGCCA	AGGCAUACAGGGAGCCAGGCA	Cleavage
tae-miR160	Traes_7DL_55ADB3528.2										0,00	-1.0	1	21	714	734	UGCCUGGCUCCCUGUAUGCCA	AGGCAUACAGGGAGCCAGGCA	Cleavage
tae-miR1134	Traes_7DL_5E2559796.1										2,00	-1.0	1	24	2562	2585	CAACAACAACAAGAAGAAGAAGAU	UUGUUGUUGUUCUUGUUGUUGUUG	Cleavage
tae-miR1134	Traes_7DL_5E2559796.2										2,00	-1.0	1	24	2556	2579	CAACAACAACAAGAAGAAGAAGAU	UUGUUGUUGUUCUUGUUGUUGUUG	Cleavage
tae-miR1122b-3p	Traes_7DL_613007B24.2										1,05	-1.0	1	21	1492	1512	AGACUUAUAUGUAGGAACGGA	UCCGUUCCUAAAUAUAAGUCU	Translation
tae-miR164	Traes_7DL_91CFE828F.1										1,00	-1.0	1	21	497	517	UGGAGAAGCAGGGCACGUGCA	AGCAAGUGCCCUGCUUCUCCA	Cleavage
tae-miR164	Traes_7DL_91CFE828F.2										1,00	-1.0	1	21	296	316	UGGAGAAGCAGGGCACGUGCA	AGCAAGUGCCCUGCUUCUCCA	Cleavage
tae-miR9778	Traes_7DL_9F0AD190A.2	PF00931	KOG4658			GO:GO:0043531	AT1G58400.1	Disease resistance protein (CC-NBS-LRR class) family	LOC_Os08g14850.1	resistance protein, putative, expressed	2,05	-1.0	1	21	126	146	UGCAUCAUCUCGAACUCGUCG	AGGCGAGUUUGAGAUGAUGCA	Cleavage
tae-miR9778	Traes_7DL_9F0AD190A.3	PF00931	KOG4658			GO:GO:0043531	AT1G58400.1	Disease resistance protein (CC-NBS-LRR class) family	LOC_Os08g14850.1	resistance protein, putative, expressed	2,05	-1.0	1	21	126	146	UGCAUCAUCUCGAACUCGUCG	AGGCGAGUUUGAGAUGAUGCA	Cleavage
tae-miR5048-5p	Traes_7DL_AB7C59D49.1	PF00069		2.7.11.1		GO:GO:0006468,GO:GO:0005524,GO:GO:0004672	AT4G21230.1	cysteine-rich RLK (RECEPTOR-like protein kinase) 27	LOC_Os11g12270.2	expressed protein	0,00	-1.0	1	22	53	74	UUUGCAGGUUUUAGGUCUAAGU	ACUUAGACCUAAAACCUGCAAA	Cleavage
tae-miR5048-5p	Traes_7DL_AB7C59D49.2	PF00069		2.7.11.1		GO:GO:0006468,GO:GO:0005524,GO:GO:0004672	AT1G11300.1	protein serine/threonine kinases;protein kinases;ATP binding;sugar binding;kinases;carbohydrate binding	LOC_Os11g17380.1	protein kinase domain containing protein, expressed	0,00	-1.0	1	22	53	74	UUUGCAGGUUUUAGGUCUAAGU	ACUUAGACCUAAAACCUGCAAA	Cleavage
tae-miR5048-5p	Traes_7DL_AB7C59D49.3	PF00069		2.7.11.1		GO:GO:0006468,GO:GO:0005524,GO:GO:0004672	AT1G11300.1	protein serine/threonine kinases;protein kinases;ATP binding;sugar binding;kinases;carbohydrate binding	LOC_Os11g17380.1	protein kinase domain containing protein, expressed	0,00	-1.0	1	22	53	74	UUUGCAGGUUUUAGGUCUAAGU	ACUUAGACCUAAAACCUGCAAA	Cleavage
tae-miR5048-5p	Traes_7DL_AB7C59D49.4	PF00069		2.7.11.1		GO:GO:0006468,GO:GO:0005524,GO:GO:0004672	AT1G11300.1	protein serine/threonine kinases;protein kinases;ATP binding;sugar binding;kinases;carbohydrate binding	LOC_Os11g17380.1	protein kinase domain containing protein, expressed	0,00	-1.0	1	22	53	74	UUUGCAGGUUUUAGGUCUAAGU	ACUUAGACCUAAAACCUGCAAA	Cleavage
tae-miR5048-5p	Traes_7DL_AB7C59D49.5	PF00069		2.7.11.1		GO:GO:0006468,GO:GO:0005524,GO:GO:0004672	AT4G21230.1	cysteine-rich RLK (RECEPTOR-like protein kinase) 27	LOC_Os11g12270.2	expressed protein	0,00	-1.0	1	22	53	74	UUUGCAGGUUUUAGGUCUAAGU	ACUUAGACCUAAAACCUGCAAA	Cleavage
tae-miR5048-5p	Traes_7DL_AB7C59D49.6	PF00069		2.7.11.1		GO:GO:0006468,GO:GO:0005524,GO:GO:0004672	AT4G21230.1	cysteine-rich RLK (RECEPTOR-like protein kinase) 27	LOC_Os11g12270.2	expressed protein	0,00	-1.0	1	22	53	74	UUUGCAGGUUUUAGGUCUAAGU	ACUUAGACCUAAAACCUGCAAA	Cleavage
tae-miR1122a	Traes_7DL_C074DC1F6.3										2,05	-1.0	1	20	109	128	UAGAUACAUCCGUAUCUAGA	ACUACAUACGGAUGUAUAUA	Cleavage
tae-miR395b	Traes_7DL_CB073ADC6.1										2,05	-1.0	1	20	1598	1617	UGAAGUGUUUGGGGGAACUC	CAGUUCACCCAAAAACUUCA	Cleavage
tae-miR395b	Traes_7DL_CB073ADC6.10										2,05	-1.0	1	20	851	870	UGAAGUGUUUGGGGGAACUC	CAGUUCACCCAAAAACUUCA	Cleavage
tae-miR395b	Traes_7DL_CB073ADC6.11										2,05	-1.0	1	20	851	870	UGAAGUGUUUGGGGGAACUC	CAGUUCACCCAAAAACUUCA	Cleavage
tae-miR395b	Traes_7DL_CB073ADC6.12										2,05	-1.0	1	20	851	870	UGAAGUGUUUGGGGGAACUC	CAGUUCACCCAAAAACUUCA	Cleavage
tae-miR395b	Traes_7DL_CB073ADC6.2										2,05	-1.0	1	20	1598	1617	UGAAGUGUUUGGGGGAACUC	CAGUUCACCCAAAAACUUCA	Cleavage
tae-miR395b	Traes_7DL_CB073ADC6.3										2,05	-1.0	1	20	1598	1617	UGAAGUGUUUGGGGGAACUC	CAGUUCACCCAAAAACUUCA	Cleavage
tae-miR395b	Traes_7DL_CB073ADC6.4										2,05	-1.0	1	20	1157	1176	UGAAGUGUUUGGGGGAACUC	CAGUUCACCCAAAAACUUCA	Cleavage
tae-miR395b	Traes_7DL_CB073ADC6.5										2,05	-1.0	1	20	1157	1176	UGAAGUGUUUGGGGGAACUC	CAGUUCACCCAAAAACUUCA	Cleavage
tae-miR395b	Traes_7DL_CB073ADC6.6										2,05	-1.0	1	20	1157	1176	UGAAGUGUUUGGGGGAACUC	CAGUUCACCCAAAAACUUCA	Cleavage
tae-miR395b	Traes_7DL_CB073ADC6.7										2,05	-1.0	1	20	1157	1176	UGAAGUGUUUGGGGGAACUC	CAGUUCACCCAAAAACUUCA	Cleavage
tae-miR395b	Traes_7DL_CB073ADC6.8										2,05	-1.0	1	20	1079	1098	UGAAGUGUUUGGGGGAACUC	CAGUUCACCCAAAAACUUCA	Cleavage
tae-miR395b	Traes_7DL_CB073ADC6.9										2,05	-1.0	1	20	851	870	UGAAGUGUUUGGGGGAACUC	CAGUUCACCCAAAAACUUCA	Cleavage
tae-miR1130b-3p	Traes_7DL_CF6AABA1F.2										0,00	-1.0	1	21	1271	1291	UCUUAUAUUAUGGGACGGAGG	CCUCCGUCCCAUAAUAUAAGA	Cleavage
tae-miR1122c-3p	Traes_7DL_CF6AABA1F.2										1,05	-1.0	1	21	1271	1291	UCUAAUAUUAUGGGACGGAGG	CCUCCGUCCCAUAAUAUAAGA	Cleavage
tae-miR1128	Traes_7DL_D5FCF9F33.4										2,05	-1.0	1	21	96	116	UACUACUCCCUCCGUCCGAAA	UUUGGGACGGAGGGAGUAUUA	Cleavage
tae-miR1122a	Traes_7DL_D5FCF9F33.4										0,00	-1.0	1	20	23	42	UAGAUACAUCCGUAUCUAGA	UCUAGAUACGGAUGUAUCUA	Cleavage
tae-miR5049-3p	Traes_7DS_06CD5BBFB.4										0,00	-1.0	1	21	99	119	AAUAUGGAUCGGAGGGAGUAC	UUACUCCCUCCGAUCCAUAUU	Cleavage
tae-miR1134	Traes_7DS_205267E87.1										2,05	-1.0	1	24	92	115	CAACAACAACAAGAAGAAGAAGAU	CUGUUCUUCUUCUUGUUGUUGUCU	Cleavage
tae-miR1130b-3p	Traes_7DS_25B02BA46.5										1,05	-1.0	1	21	730	750	UCUUAUAUUAUGGGACGGAGG	CCUCCGUCCCAUAAUGUAAGA	Cleavage
tae-miR1133	Traes_7DS_25F082B1E.8										3,00	-1.0	1	22	19	41	CAUAUACUCCCUCCGUCC-GAAA	UUUCCGGACGGAGGGAGUAUAUA	Cleavage
tae-miR9662a-3p	Traes_7DS_2B1FE04A6.1	PF02536				GO:GO:0006355,GO:GO:0005739,GO:GO:0003690	AT5G07900.1	Mitochondrial transcription termination factor family protein	LOC_Os06g12100.1	mTERF family protein, expressed	2,05	-1.0	1	21	561	581	UUGAACAUCCCAGAGCCACCG	CCGGUGGCUCUGCGAUGUUCAG	Translation
tae-miR9662b-3p	Traes_7DS_2B1FE04A6.1	PF02536				GO:GO:0006355,GO:GO:0005739,GO:GO:0003690	AT5G07900.1	Mitochondrial transcription termination factor family protein	LOC_Os06g12100.1	mTERF family protein, expressed	2,05	-1.0	1	21	560	580	UGAACAUCCCAGAGCCACCGG	CCCGUGGCUCUGCGAUGUUCA	Cleavage
tae-miR1130b-3p	Traes_7DS_3C83C8EF9.1										1,00	-1.0	1	21	37	57	UCUUAUAUUAUGGGACGGAGG	CCUCCGUUCCAUAAUAUAAGA	Cleavage
tae-miR1122c-3p	Traes_7DS_3C83C8EF9.1										2,05	-1.0	1	21	37	57	UCUAAUAUUAUGGGACGGAGG	CCUCCGUUCCAUAAUAUAAGA	Cleavage
tae-miR1120c-5p	Traes_7DS_3C83C8EF9.1										1,05	-1.0	1	21	80	100	UAAUAUAAGAACGUUUUUGAC	GUCAAAAACGCUCUUAUAUUA	Translation
tae-miR1130b-3p	Traes_7DS_3C83C8EF9.3										1,00	-1.0	1	21	37	57	UCUUAUAUUAUGGGACGGAGG	CCUCCGUUCCAUAAUAUAAGA	Cleavage
tae-miR1122c-3p	Traes_7DS_3C83C8EF9.3										2,05	-1.0	1	21	37	57	UCUAAUAUUAUGGGACGGAGG	CCUCCGUUCCAUAAUAUAAGA	Cleavage
tae-miR1120c-5p	Traes_7DS_3C83C8EF9.3										1,05	-1.0	1	21	80	100	UAAUAUAAGAACGUUUUUGAC	GUCAAAAACGCUCUUAUAUUA	Translation
tae-miR9662a-3p	Traes_7DS_5B1210681.1	PF02536			K15032	GO:GO:0006355,GO:GO:0005739,GO:GO:0003690	AT1G21150.1	Mitochondrial transcription termination factor family protein	LOC_Os06g12100.1	mTERF family protein, expressed	2,05	-1.0	1	21	285	305	UUGAACAUCCCAGAGCCACCG	CCGGUGGCUCUGCGAUGUUCAG	Translation
tae-miR9662b-3p	Traes_7DS_5B1210681.1	PF02536			K15032	GO:GO:0006355,GO:GO:0005739,GO:GO:0003690	AT1G21150.1	Mitochondrial transcription termination factor family protein	LOC_Os06g12100.1	mTERF family protein, expressed	2,05	-1.0	1	21	284	304	UGAACAUCCCAGAGCCACCGG	CCCGUGGCUCUGCGAUGUUCA	Cleavage
tae-miR9778	Traes_7DS_74534FCD3.1	PF00931				GO:GO:0043531	AT1G50180.1	NB-ARC domain-containing disease resistance protein	LOC_Os10g03570.1	RGH1A, putative, expressed	2,05	-1.0	1	21	111	131	UGCAUCAUCUCGAACUCGUCG	AGACGAGCUGGAGAUGAUGCA	Cleavage
tae-miR9778	Traes_7DS_74534FCD3.2	PF00931				GO:GO:0043531	AT3G46530.1	NB-ARC domain-containing disease resistance protein	LOC_Os10g03570.1	RGH1A, putative, expressed	2,05	-1.0	1	21	111	131	UGCAUCAUCUCGAACUCGUCG	AGACGAGCUGGAGAUGAUGCA	Cleavage
tae-miR9778	Traes_7DS_74534FCD3.3	PF00931				GO:GO:0043531	AT3G46530.1	NB-ARC domain-containing disease resistance protein	LOC_Os10g03570.1	RGH1A, putative, expressed	2,05	-1.0	1	21	111	131	UGCAUCAUCUCGAACUCGUCG	AGACGAGCUGGAGAUGAUGCA	Cleavage
tae-miR9778	Traes_7DS_74534FCD3.4	PF00931				GO:GO:0043531	AT3G46530.1	NB-ARC domain-containing disease resistance protein	LOC_Os10g03570.1	RGH1A, putative, expressed	2,05	-1.0	1	21	111	131	UGCAUCAUCUCGAACUCGUCG	AGACGAGCUGGAGAUGAUGCA	Cleavage
tae-miR9778	Traes_7DS_74534FCD3.5	PF00931				GO:GO:0043531	AT3G46530.1	NB-ARC domain-containing disease resistance protein	LOC_Os10g03570.1	RGH1A, putative, expressed	2,05	-1.0	1	21	111	131	UGCAUCAUCUCGAACUCGUCG	AGACGAGCUGGAGAUGAUGCA	Cleavage
tae-miR9778	Traes_7DS_74534FCD3.6	PF00931				GO:GO:0043531	AT3G46530.1	NB-ARC domain-containing disease resistance protein	LOC_Os10g03570.1	RGH1A, putative, expressed	2,05	-1.0	1	21	111	131	UGCAUCAUCUCGAACUCGUCG	AGACGAGCUGGAGAUGAUGCA	Cleavage
tae-miR1133	Traes_7DS_98F9E4E55.2										2,00	-1.0	1	22	2	24	CAUAUACUCCCUCCGUCCGA-AA	UUAUGGGACGGAGGGAGUAUAUA	Cleavage
tae-miR5050	Traes_7DS_A9CCF5A83.5										2,00	-1.0	1	21	297	317	UUGAACGACCUCACCAUGUCG	UGCCAUGAUGAGGUCGUUCAA	Cleavage
tae-miR1122a	Traes_7DS_CA61ECF35.1										0,00	-1.0	1	20	33	52	UAGAUACAUCCGUAUCUAGA	UCUAGAUACGGAUGUAUCUA	Cleavage
tae-miR9775	Traes_7DS_D4DFDF981.1										2,00	-1.0	1	22	1609	1630	UGUGCGCAAUAAGAUUUUGCUA	GUGCAUGAUCUUAUUGCGCACA	Cleavage
tae-miR9775	Traes_7DS_D4DFDF981.2										2,00	-1.0	1	22	1702	1723	UGUGCGCAAUAAGAUUUUGCUA	GUGCAUGAUCUUAUUGCGCACA	Cleavage
tae-miR156	Traes_7DS_DB829C7CD.1								LOC_Os08g41940.1	OsSPL16 - SBP-box gene family member, expressed	1,00	-1.0	1	21	359	379	UGACAGAAGAGAGUGAGCACA	UGUGCUCUCUCUCUUCUGUCA	Cleavage
tae-miR5049-3p	Traes_7DS_EFB144315.5										1,05	-1.0	1	21	2298	2318	AAUAUGGAUCGGAGGGAGUAC	UUACUCCCUCUGAUCCAUAUU	Translation
