# Supplementary figures and images for: Genome-wide analysis of miRNAs and their target genes in wheat cultivars with different ploidy levels under drought stress
Source: Planta. 2025 Jul 1;262(2):38. doi: 10.1007/s00425-025-04757-3 (PMC12213836; doi:10.1007/s00425-025-04757-3)

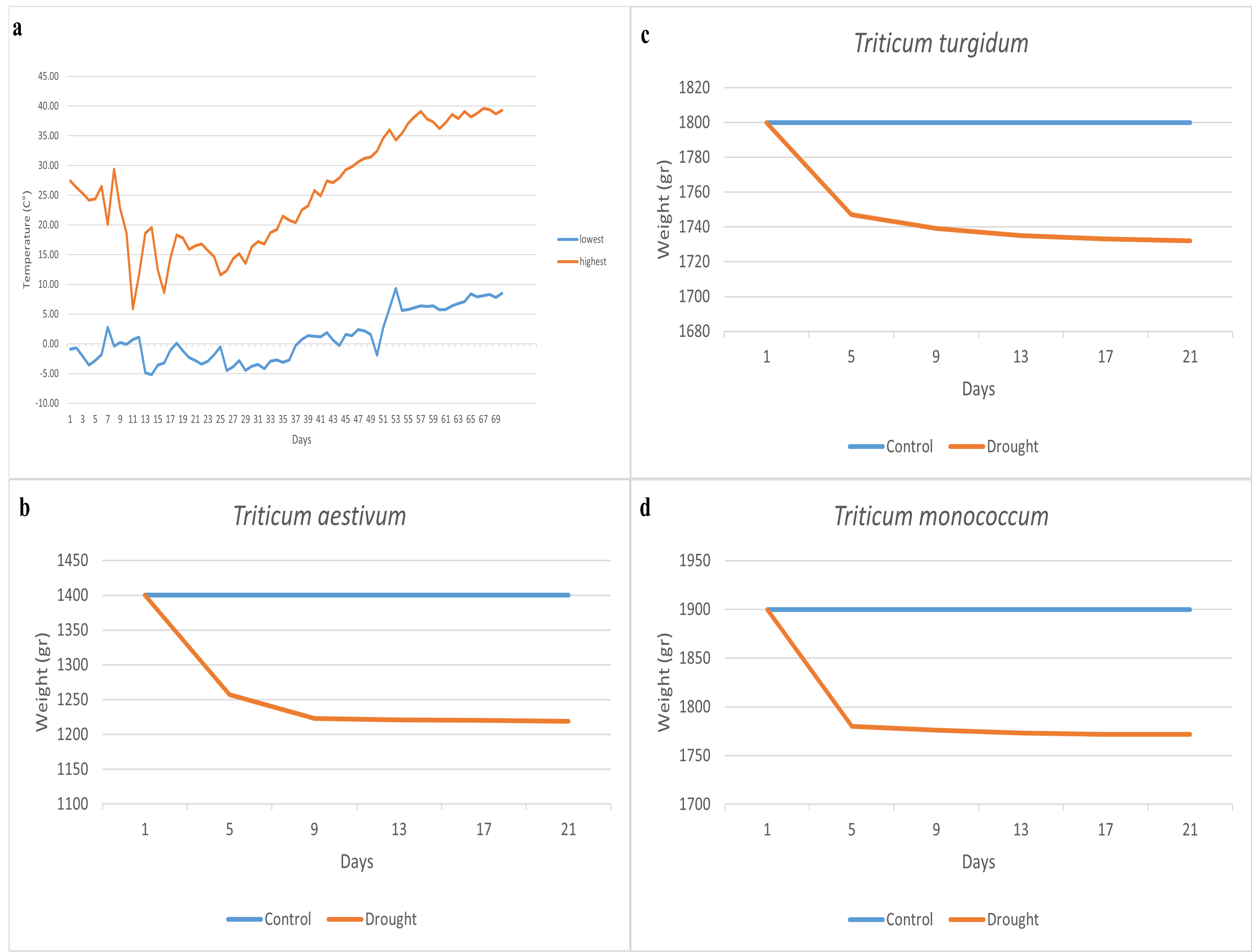

Supplement: Supplementary file 8 — Supplementary file8 (PNG 2772 KB) [file 425_2025_4757_MOESM8_ESM.png]

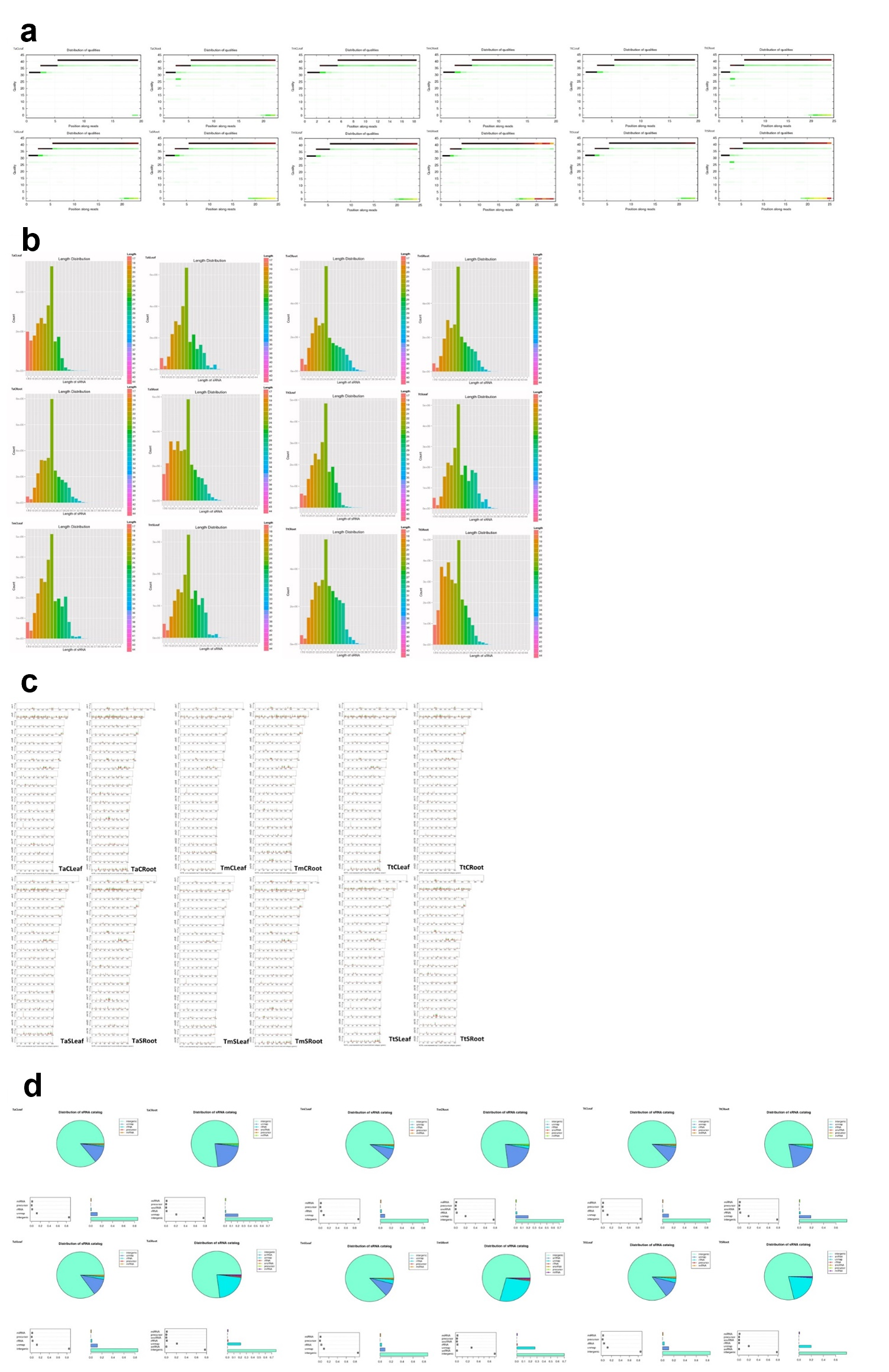

Supplement: Supplementary file 9 — Supplementary file9 (PNG 6046 KB) [file 425_2025_4757_MOESM9_ESM.png]

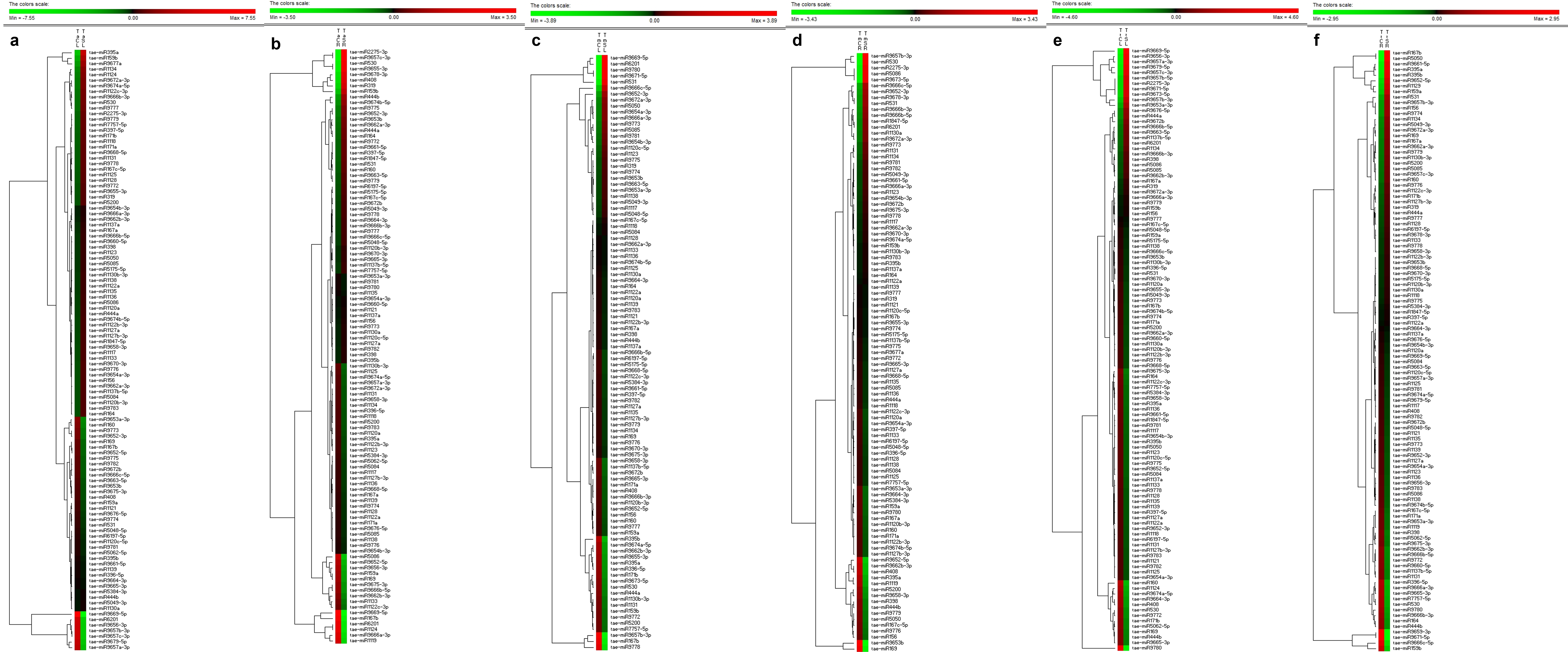

Supplement: Supplementary file 10 — Supplementary file10 (DOCX 17 KB) [file 425_2025_4757_MOESM10_ESM.png]

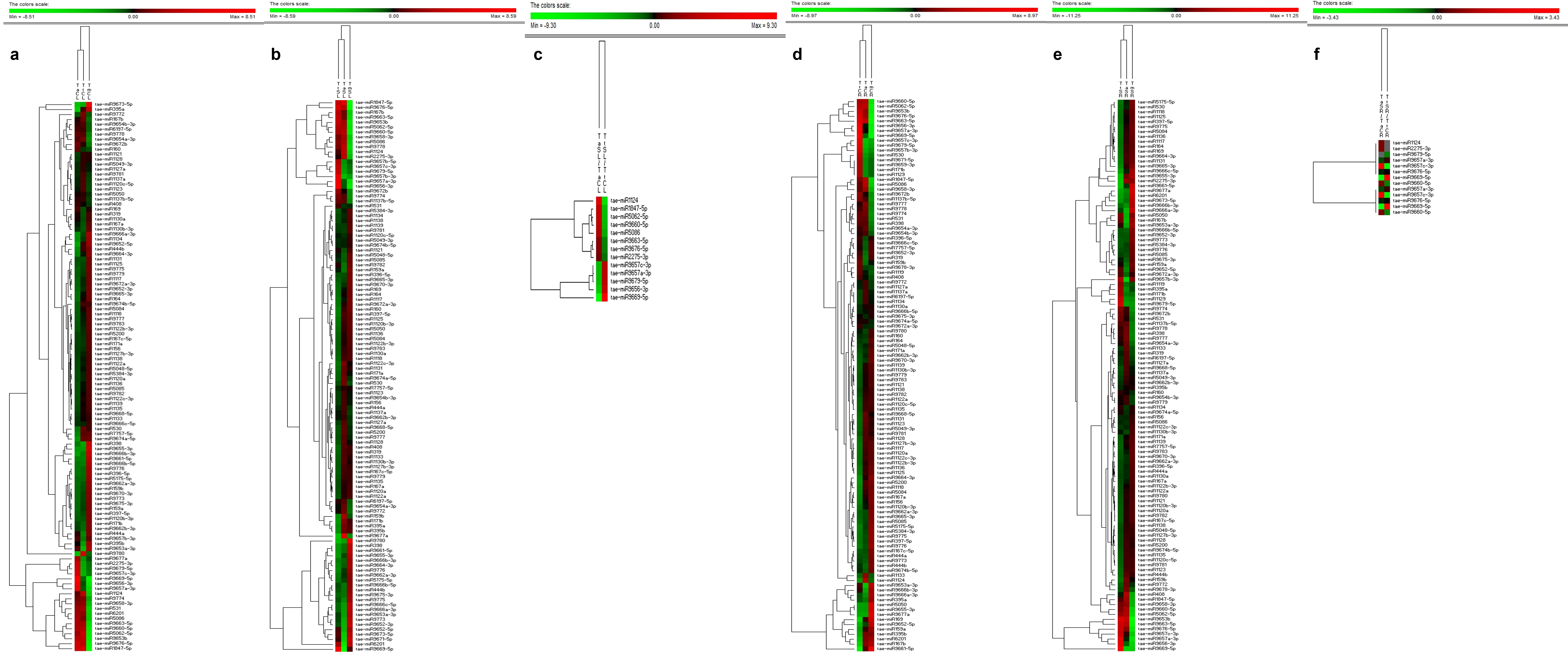

Supplement: Supplementary file 11 — Supplementary file11 (DOCX 16 KB) [file 425_2025_4757_MOESM11_ESM.png]

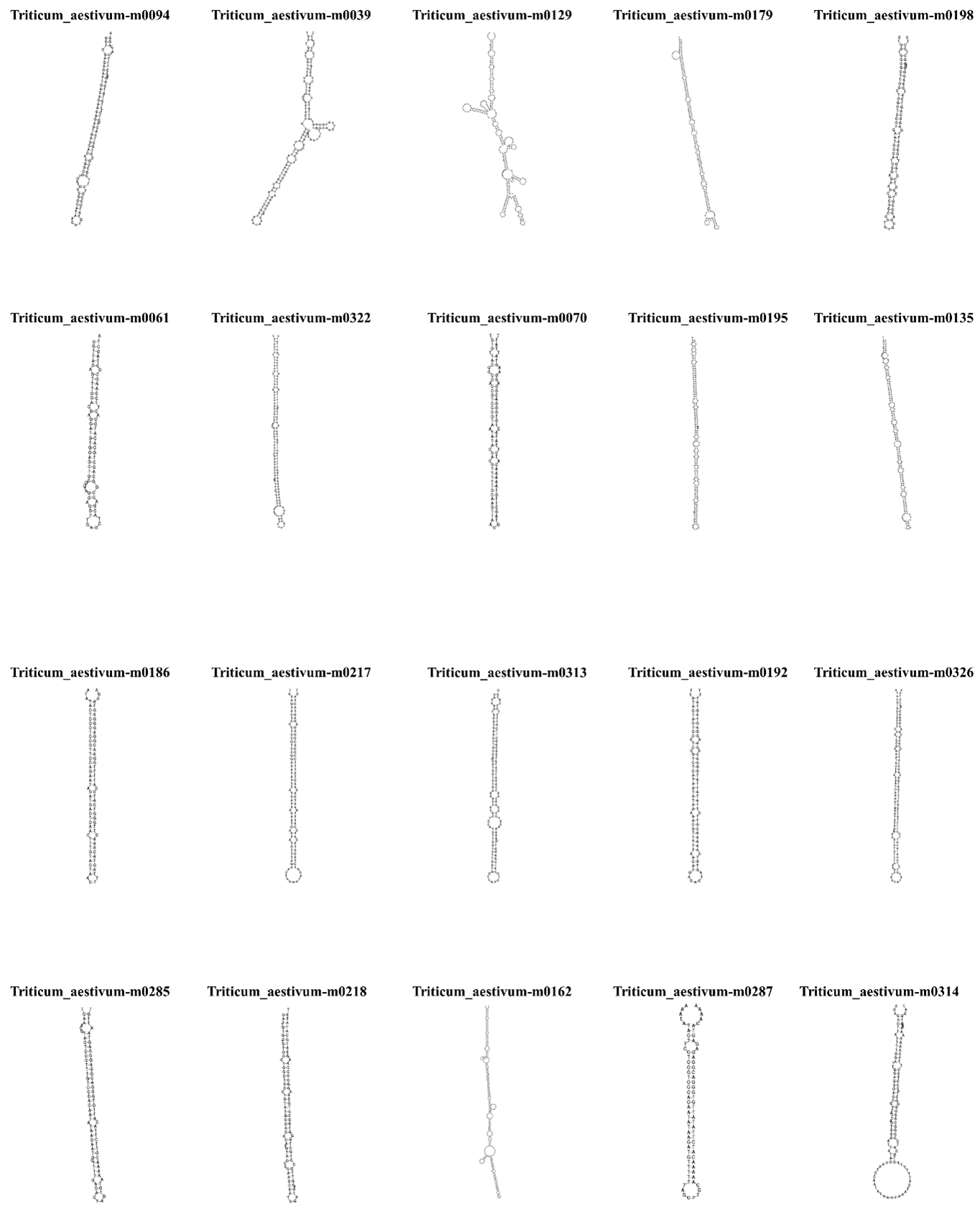

Supplement: Supplementary file 12 — Supplementary file12 (DOCX 16 KB) [file 425_2025_4757_MOESM12_ESM.png]

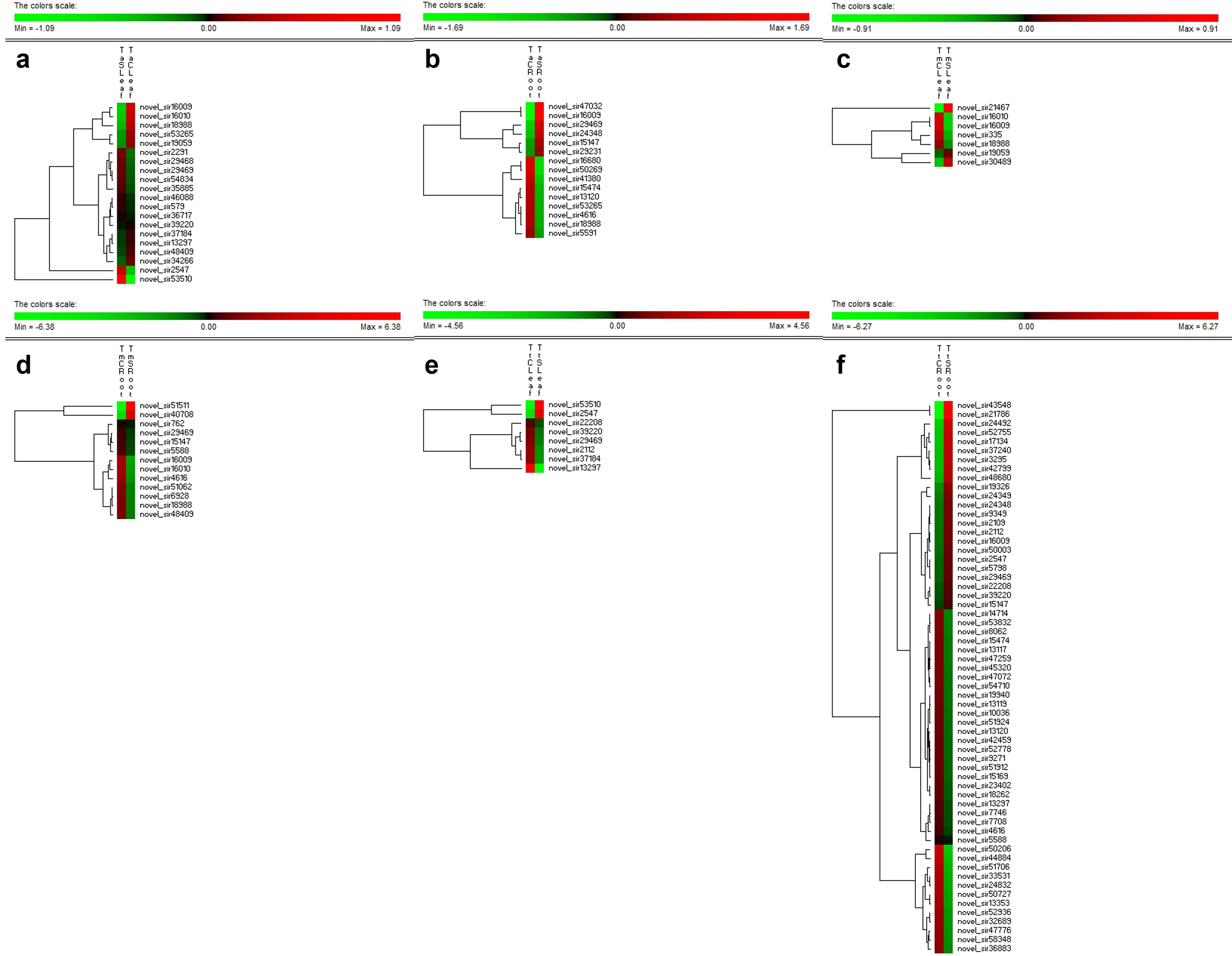

Supplement: Supplementary file 13 — Supplementary file13 (DOCX 15 KB) [file 425_2025_4757_MOESM13_ESM.png]

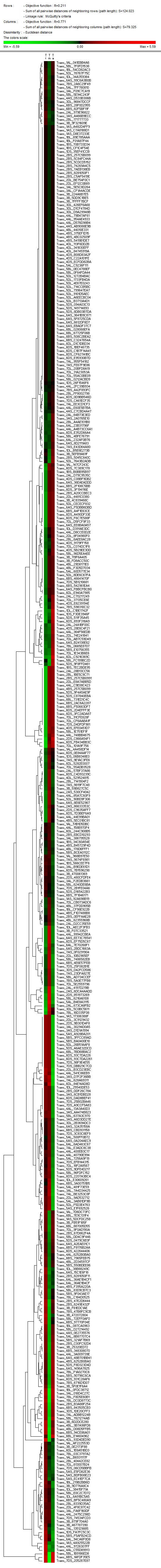

Supplement: Supplementary file 14 — Supplementary file14 (PDF 3230 KB) [file 425_2025_4757_MOESM14_ESM.jpg]

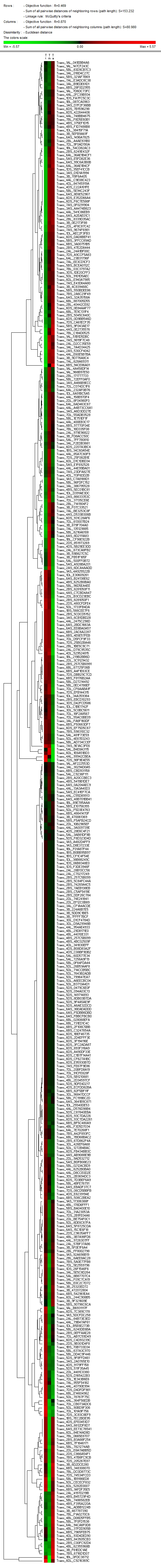

Supplement: Supplementary file 15 — Supplementary file15 (PDF 3178 KB) [file 425_2025_4757_MOESM15_ESM.jpg]

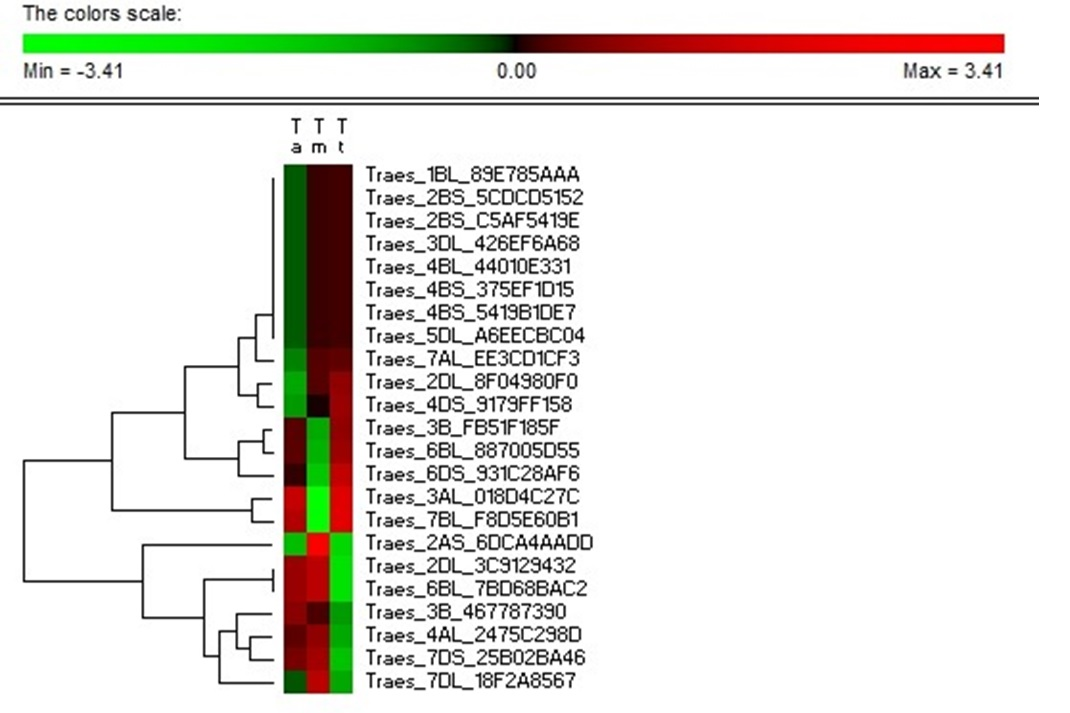

Supplement: Supplementary file 16 — Supplementary file16 (XLSX 223 KB) [file 425_2025_4757_MOESM16_ESM.png]

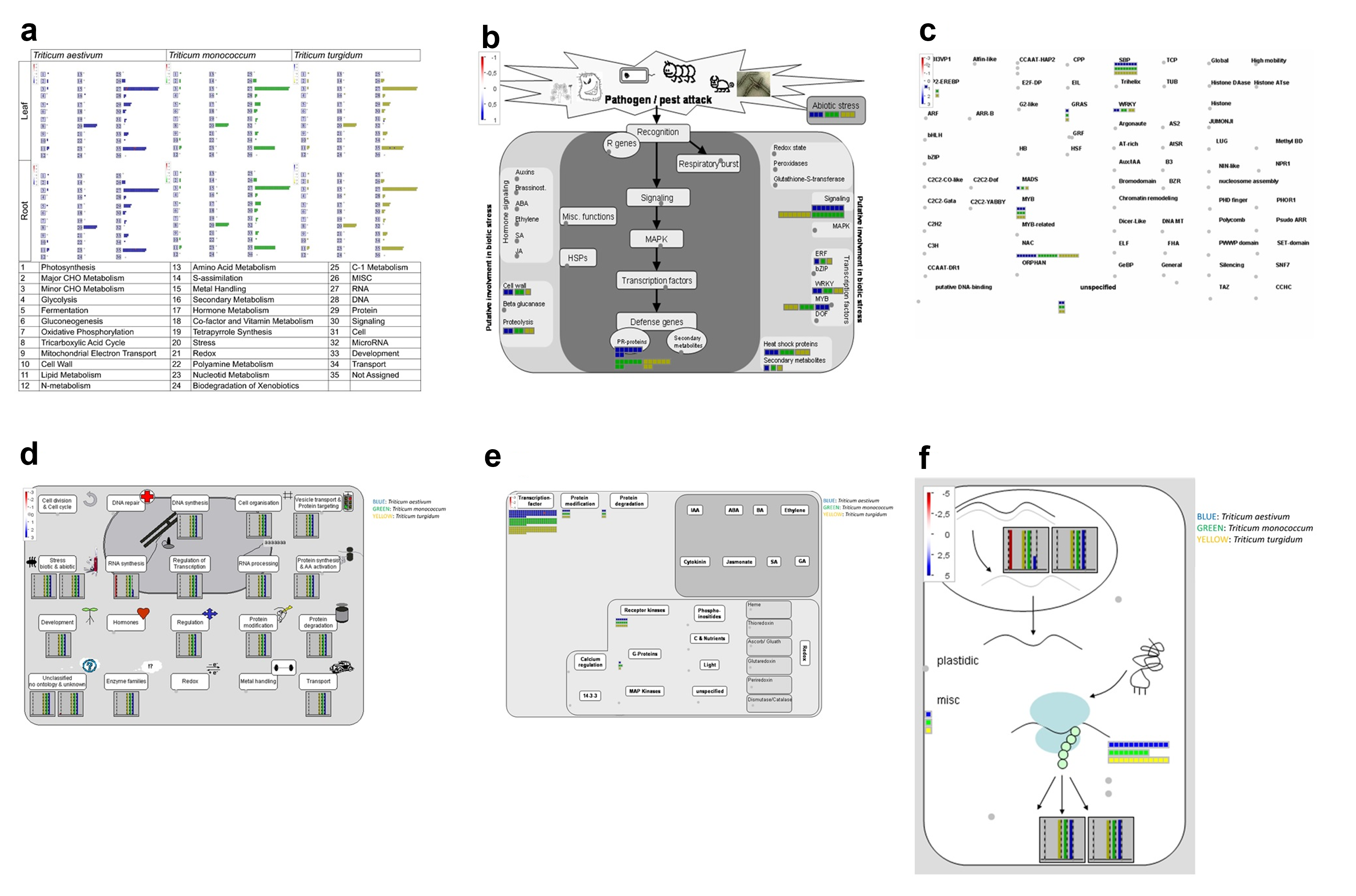

Supplement: Supplementary file 17 — Supplementary file17 (PNG 2759 KB) [file 425_2025_4757_MOESM17_ESM.png]

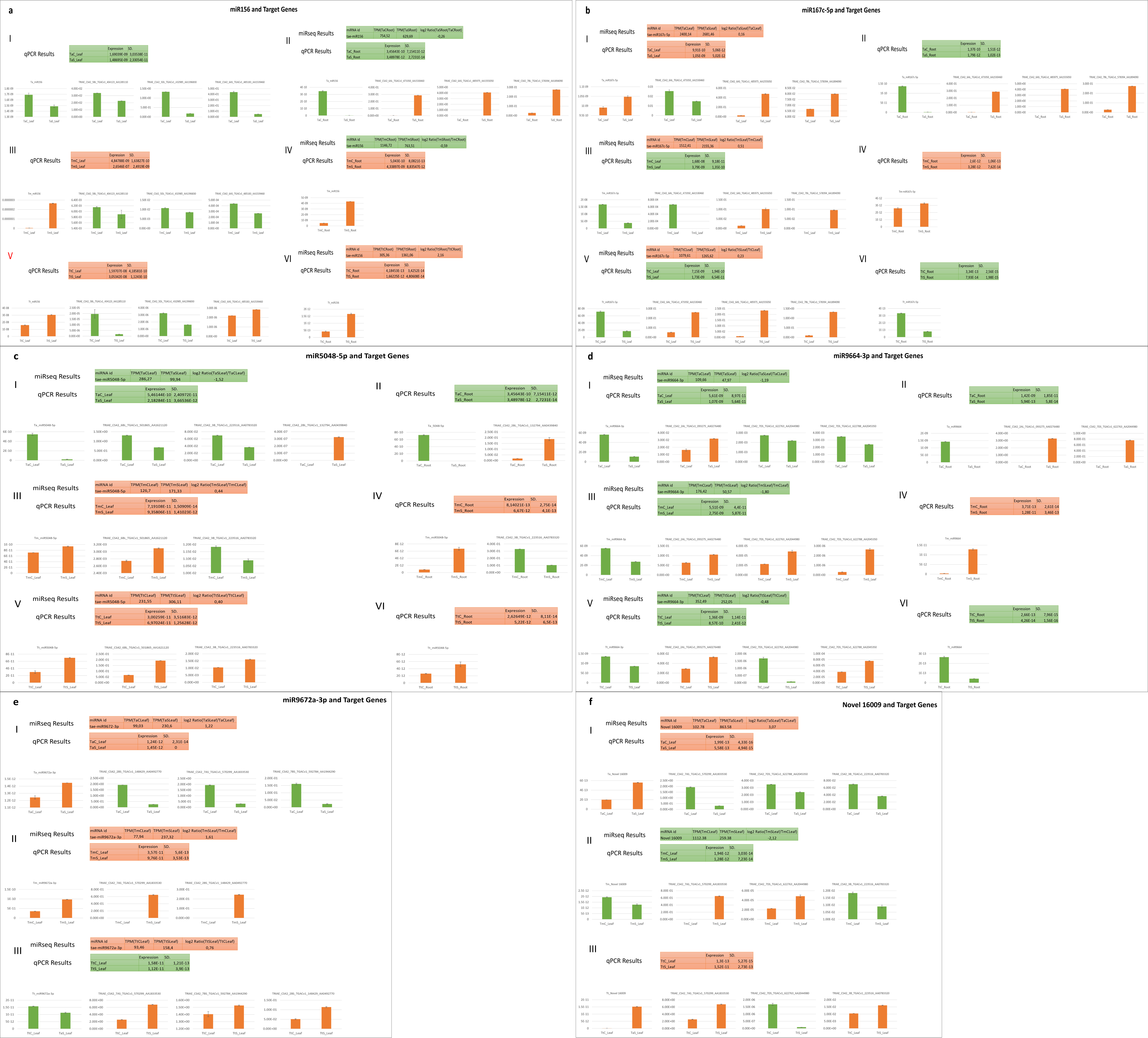

Supplement: Supplementary file 18 — Supplementary file18 (PNG 1916 KB) [file 425_2025_4757_MOESM18_ESM.png]
